# A peptide N-terminal protection strategy for comprehensive glycoproteome analysis using hydrazide chemistry based method

**DOI:** 10.1038/srep10164

**Published:** 2015-05-11

**Authors:** Junfeng Huang, Hongqiang Qin, Zhen Sun, Guang Huang, Jiawei Mao, Kai Cheng, Zhang Zhang, Hao Wan, Yating Yao, Jing Dong, Jun Zhu, Fangjun Wang, Mingliang Ye, Hanfa Zou

**Affiliations:** 1 CAS Key Laboratory of Separation Sciences for Analytical Chemistry, National Chromatographic R&A Center, Dalian Institute of Chemical Physics, Chinese Academy of Sciences, Dalian, China; 2 University of Chinese Academy of Sciences, Beijing, China; 3 Shanghai Key Laboratory of Functional Materials Chemistry, East China University of Science and Technology

## Abstract

Enrichment of glycopeptides by hydrazide chemistry (HC) is a popular method for glycoproteomics analysis. However, possible side reactions of peptide backbones during the glycan oxidation in this method have not been comprehensively studied. Here, we developed a proteomics approach to locate such side reactions and found several types of the side reactions that could seriously compromise the performance of glycoproteomics analysis. Particularly, the HC method failed to identify N-terminal Ser/Thr glycopeptides because the oxidation of vicinal amino alcohol on these peptides generates aldehyde groups and after they are covalently coupled to HC beads, these peptides cannot be released by PNGase F for identification. To overcome this drawback, we apply a peptide N-terminal protection strategy in which primary amine groups on peptides are chemically blocked via dimethyl labeling, thus the vicinal amino alcohols on peptide N-termini are eliminated. Our results showed that this strategy successfully prevented the oxidation of peptide N-termini and significantly improved the coverage of glycoproteome.

Protein glycosylation is one of the major post-translational modifications (PTMs) with significant effects on protein folding, stability and activity^1^,^2^. It plays structural, protective, stabilizing roles in living cells and is involved in multiple biological mechanisms^3^,^4^,^5^. It is well recognized that aberrant glycosylation is closely correlated with oncogenesis and tumor progression, and up to now, most of the clinically used cancer biomarkers are glycoproteins^6^,^7^,^8^. Therefore, comprehensive characterization of the glycoproteome in a biological sample is highly demanded but remains extremely challenging due to the vast dynamic range of protein concentration and the heterogeneity of protein glycosylation. To achieve in-depth glycoproteome analysis, glycoproteins/glycopeptides are often selectively enriched to reduce the sample complexity^9^,^10^,^11^. There are four glycoproteins/glycopeptides enrichment approaches that have been mostly applied so far including: 1) lectin affinity chromatography based method^12^,^13^; 2) titanium dioxide chromatography^14^,^15^; 3) hydrophilic interaction chromatography (HILIC)^16^,^17^,^18^ based method; and 4) chemical reaction based method using hydrazide chemistry (HC)^19^,^20^,^21^,^22^. Each method has its strength and weakness. The lectin chromatography is able to enrich glycopeptides with specific types of glycans, however it suffers from weak binding and low specificity^23^,^24^. Compared with the titanium dioxide chromatography which only enriches sialic acid containing glycopeptides^14^, the HILIC can enrich glycopeptides in non-glycan-specific fashion and is best fitted to study the site-specific glycan heterogeneity^24^. However it also has the disadvantage of poor specificity. To improve the enrichment specificity, lectin and HILIC enrichment methods were reported to be applied sequentially^25^,^26^. The HC method, on the other hand, has the advantage of capturing glycopeptides with high specificity. However unless with some specific modifications reported recently^27^,^28^,^29^, the general HC method cannot be applied to elucidate the structure of glycans due to the fact that glycans are oxidized and covalently linked to the hydrazide beads. It is also worth being notified that none of these approaches is able to capture the whole glycoproteome so far. Combining these highly complementary methods has been reported to be very effective in improving glycoproteome coverage^30^,^31^.

The HC method is particularly attractive because of its high enrichment specificity that benefits from the formation of covalent bonds between glycans of glycoproteins/glycopeptides and hydrazide groups on beads. Since the glycoproteins/glycopeptides are covalently bound to the HC beads, non-specific adsorption of proteins or peptides on the beads can be well removed by harsh washing procedures. The peptide moieties of the captured glycopeptides are specifically released by PNGase F, which cleaves the glycosidic bonds between the innermost GlcNAc and asparagine residues of N-linked glycopeptides. The released deglycosylated glycopeptides (de-glycopeptides) are then analyzed by LC-MS/MS for identifications. However, the strong oxidant periodate sodium (NaIO_4_) used to oxidize the cis-diol of glycans on glycopeptides/glycoproteins in this method, may oxidize other groups on the peptides as well. As a result, the peptide backbones can also be modified by oxidation that could restrict them from being identified through normal database search. Unfortunately, this important issue in the HC method has not been comprehensively investigated so far.

Such oxidation induced modifications could occur on any amino acid residue of peptides. However, there has been no high throughput way to locate the amino acid residues subjected to side reaction(s). In this study, we present a proteomics approach based on the fact that any peptides with unknown modifications will not be able to be identified by the normal database search. At least two types of side reactions on glycopeptide backbones were found by this approach including the oxidation on amino acids of the methionine (Met), carbamidomethylated cysteine (CamCys) and tryptophan (Trp) residues as well as aldehyde formation on the N-terminal serine (Ser) or threonine (Thr) residues. For the de-glycopeptides with oxidized Met, CamCys, and Trp residues, their identification could be easily achieved by setting variable modifications during database search. However, for the de-glycopeptides with N-terminal Ser/Thr, formation of the aldehyde groups on their N-termini resulted in covalent binding onto the hydrazide beads that cannot be released by PNGase F cleavage. Consequently these part of the de-glycopeptides with N-terminal Ser/Thr cannot be identified which account for about 15% of glycoproteome. To overcome this problem, we presented a peptide N-terminal protection (PNP) strategy to prevent the N-terminal Ser/Thr oxidation. This new strategy was demonstrated to be able to identify de-glycopeptides with N-terminal Ser/Thr efficiently and thereby significantly improved the glycoproteomics coverage.

## Results

### Discovery of the side reactions by an amino acid frequency check approach

**Figure 1:**
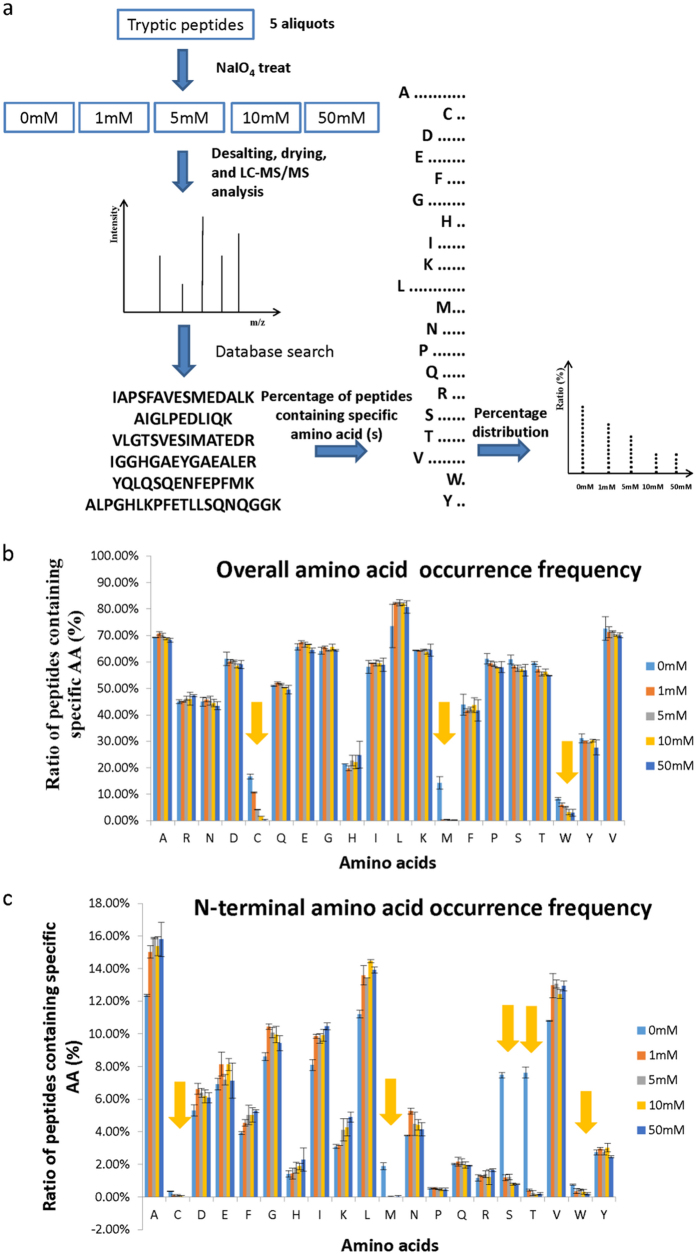
Proteome-wide amino acid frequency check approach to locate the residues modified with side reactions. (**a**) Scheme of the amino acid occurrence frequency check approach. (**b**) Comparison of overall amino acid occurrence frequencies and (**c**) Comparison of N-terminal amino acid occurrence frequencies for tryptic peptides treated with different NaIO_4_ concentrations. Above data are averaged from 4 replicate experiments (error bars represent the standard deviation).

To investigate the unexpected modifications occurred on peptide backbones during NaIO_4_ treatment in HC method, we proposed a proteome-wide amino acid occurrence frequency check approach (Fig. 1a). The proteins in mouse liver lysate were reduced by DTT followed with alkylation by iodoacetamide as in the standard proteomics sample preparation. After trypsin digestion, five aliquots of the resulted tryptic peptides were treated with 0, 1, 5, 10, and 50 mM NaIO_4_, respectively. The reaction was quenched by trifluoroacetic acid followed by direct LC-MS/MS analysis. The acquired spectra were searched by the MaxQuant^32^ without setting any variable modifications. By this way, only the peptides without any unexpected modifications were identified. The amino acid occurrence frequency of the identified peptides were compared in two aspects, i.e. the percentages of peptides containing each amino acid residue overall and the percentages of peptides containing each amino acid residue at the C-terminus or N-terminus. In this article we termed the former the overall and the latter the C-terminal or N-terminal amino acid occurrence frequency distributions, respectively. If there was any side reaction occurred on a residue, the peptides would be modified. Because these modified peptides cannot be identified by above searching scheme, the occurrence frequency for that type of peptides must decrease. This will give us a clue to determine which type of amino acid residue might be modified during the NaIO_4_ oxidation procedure.

Figure 1b gives the overall amino acid occurrence frequency distributions for five aliquots of the same sample treated with NaIO_4_ of different concentrations. It was found that the percentage of peptides containing any of the following 3 amino acid residues, Cys, Met, and Trp, decreased dramatically after oxidization. The frequency of Met-containing peptides had a sharp decrease in the sample treated with a minimum of 1 mM NaIO_4_, while the frequency of Cys/Trp-containing peptides gradually decrease with the increase of NaIO_4_ concentration (Table S1), indicating certain modifications occurred on these three amino acid residues upon oxidation.

Due to the presence of one additional group on peptide termini, that is primary amine at N-termini or carboxyl at C-termini, some additional side reactions may occur on these terminal residues. Therefore we further investigated if the termini residues of the peptides could be modified during the NaIO_4_ treatment. As the peptides were generated by trypsin digestion, mainly two types of residues, i.e. Lys and Arg, were presented on peptide C-termini. We found that the distribution of peptides with C-termini of Lys and Arg did not change following oxidization (Table S2), suggesting no modification occurred at the peptide C-termini. However, the situation for the peptide N-termini was significantly different as shown by the N-terminal amino acid occurrence frequency changes in Fig.1c. The percentages for peptides with N-termini of any of these 5 residues, Cys, Met, Ser, Thr and Trp, decreased significantly after oxidation, indicating modifications occurred at the N-termini of these peptides (Fig. 1c, Table S3). In comparison with the overall amino acid frequency distributions (Fig. 1b), specific modifications on Ser/Thr residues at the N-termini of these peptides were observed. We further examined the frequencies of the amino acid residues next to the N-terminal residues for all the identified peptides, it was found that no obvious changes occur on the Ser/Thr residues although the Cys, Met and Trp residues showed similar frequency changes as the overall amino acid frequency changes of these individual residues (Fig. S1). The above data suggested that the side reaction on Cys, Met and Trp could occur at any place of the peptides, while the side reaction on Ser/Thr could only occur at the N-termini of peptides.

**Figure 2:**
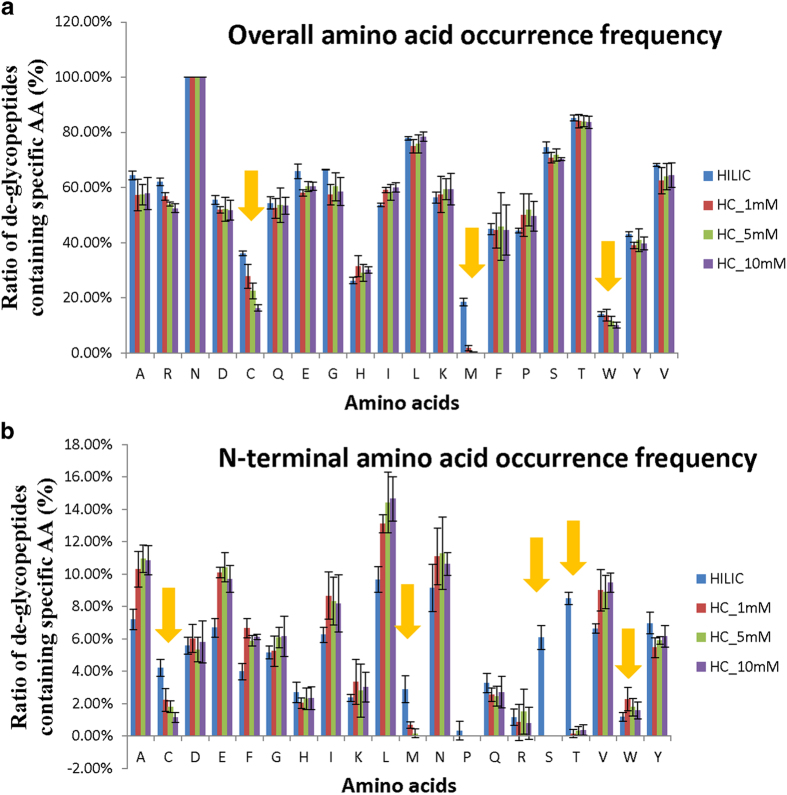
The influence of side reactions on identification of de-glycopeptides in HC method. (**a**) Comparison of overall amino acid frequency of de-glycopeptides identified by HILIC and HC methods (with 3 different NaIO_4_ concentrations: 1 mM, 5 mM and 10 mM) from mouse liver glycoproteome. (**b**) Comparison of N-terminal amino acid frequency of de-glycopeptides identified by HILIC and HC methods in mouse liver glycoproteome. Above data were averaged from 3 replicate experiments (error bars represent the standard deviation).

We further investigated how these side reactions affected the identification of glycopeptides in the HC method. The tryptic peptides from mouse liver were oxidized at 1, 5, 10 mM NaIO_4_, respectively. Then the oxidized peptide samples were incubated with hydrazide beads and the covalent bound glycopeptides were cleaved by PNGase F following the standard HC method protocol. Finally the resulted de-glycopeptides were submitted to LC-MS/MS for identification. The glycopeptides enriched by HILIC, which are not subjected to oxidation, were applied as a perfect control for the comparison. To facilitate the identification of the peptide backbones and glycosites, the glycans of the HILIC enriched glycopeptides were also removed by PNGase F. All the de-glycopeptides were analyzed by LC-MS/MS and searched by the MaxQuant^32^ under the same condition. The overall amino acid occurrence frequency distributions of the glycoproteomes determined by HILIC and conventional HC method are shown in Fig. 2a. Compared with those in HILIC method, the percentages of identified de-glycopeptides containing Cys, Met or Trp residues were obvious lower in the HC method. These data indicated that the conventional HC method is inefficient to identify these peptides. The N-terminal amino acid occurrence frequency distributions were also compared (Fig. 2b). It was found that the percentage of de-glycopeptides with N-terminal Ser/Thr was 14.6% for the glycoproteome datasets acquired by the HILIC methods, while almost no such peptides were identified by the HC enrichment method. These data indicated that the conventional HC method is unable to identify these peptides. Clearly the side reactions occurring on the peptides containing Cys, Met and Trp, as well as the N-terminal Ser/Thr severely impaired the glycoproteomics coverage of HC method.

**Figure 3:**
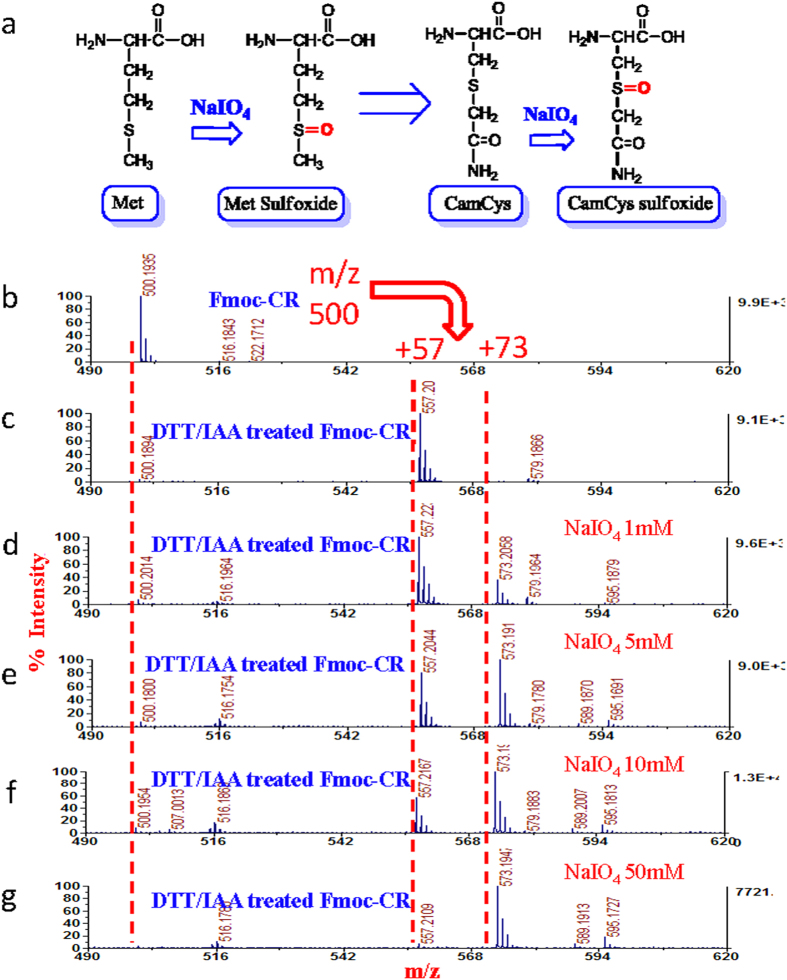
The side reaction occurred on CamCys. (**a**) Sulfurs on the Met and CamCys were oxidized to sulfoxides. (**b-c**) MALDI mass spectra of the Cys containing dipeptides Fmoc-CR treated without and with iodoacetamide. (**d-g**) MALDI mass spectra of carbamidomethylated Fmoc-CR oxidized at different NaIO_4_ concentrations.

**Figure 4:**
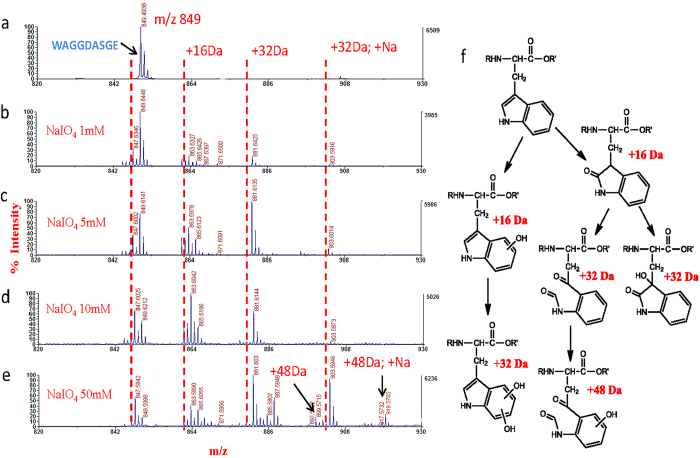
The side reactions occurred on Trp. (**a-e**) MALDI mass spectra of the Trp containing peptide WAGGDASGE (0.01 μg/μL, 0.5 μL) oxidized at 0, 1, 5, 10, 50 mM NaIO_4_. (**f**) Possible oxidized tryptophan derivates deduced according to literatures^33^.

To find ways to prevent the loss of identifications resulted from such side reactions, we further investigated what types of modifications could occur on these residues during the NaIO_4_ oxidation. It is well known that the oxidation of Met is often occurred in the living organism and during sample preparation for proteomics analysis, thus oxidation of Met is usually set as variable modification (+16 Da) in the database search^33^. To investigate the oxidation extent of Met in HC method, a peptide containing Met, YGGFM, was incubated with different concentrations of NaIO_4_, and the resulted products were analyzed by MALDI MS. As showed in Fig. S2, when the sample was treated with as low as 1 mM NaIO_4_, a + 16 Da mass shift was observed in the spectra. This mass shift is due to the Met residue in the peptide being oxidized to Met sulfoxide (MetO) by NaIO_4_ (Fig. 3a). Since the reduced and alkylated Cys has the similar chemical structure with Met (Fig. 3a), we hypothesized that it could also be oxidized by NaIO_4_ to form the CamCys sulfoxide. To confirm this hypothesis, we performed the following experiment. A dipeptide Cys-Arg (Fmoc-CR) was firstly reduced and alkylated as in standard proteomic sample preparation method. Then the dipeptide was incubated with different concentrations of NaIO_4_. The MALDI spectra in Fig. 3b–g show that the dipeptide was fully carbamidomethylated by iodoacetamide (+57 Da) and then it was gradually converted to its oxidized form with increasing the concentration of NaIO_4_ from 1 mM to 50 mM, which is shown by the appearance of a peak with mass shift of +16 Da. These data indicated that side reaction of Cys is similar to the oxidation of methionine residues, where sulfur of the CamCys was oxidized to its sulfoxide form. The side reaction on peptides containing Trp was also investigated. A standard peptide WAGGDASGE was treated with different concentration of NaIO_4_ in the same manner as treating the Cys containing peptide. As shown in Fig. 4a–e, the peptide containing Trp mainly had two oxidized derivate peaks (+16 Da and +32 Da), and when the NaIO_4_ concentration reach to 50 mM, more oxidized derivate peaks were observed. However the total conversion rate of Trp containing peptide to its oxidized derivate was not as high as that of the Cys containing peptide. According to the previous literature^34^,^35^, there are different oxidation pathways of Trp residues (Fig. 4f) and different oxidation types may add the same mass on peptides, therefore we cannot confirm the derivate peaks belong to which types of oxidation.

**Figure 5:**
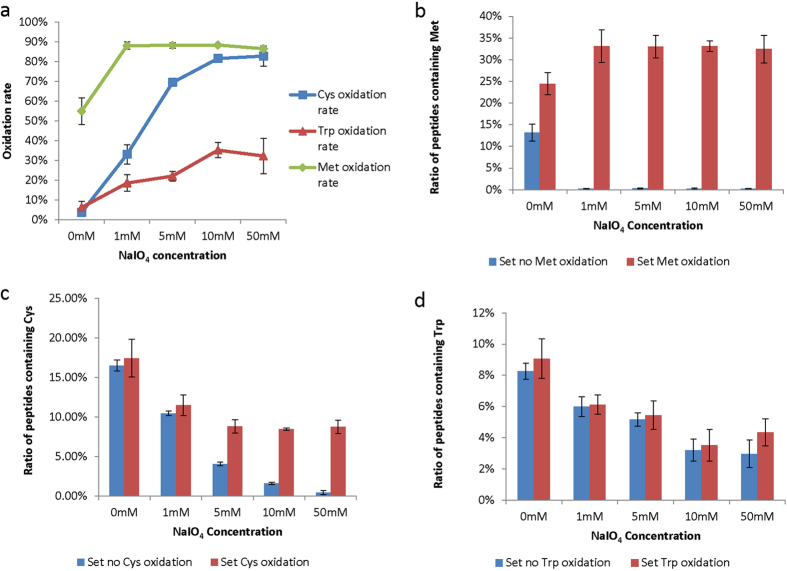
Comparison of the oxidation rate and the ratio of identified peptides containing Cys, Trp and Met at different NaIO4 concentrations. (**a**) Oxidation rates. (**b-d**) The percentages of the identified peptides containing Cys, Trp and Met with and without setting oxidation as a variable modification in database search. Above data were averaged from 4 replicate (error bars represent the standard deviation).

As the mass shift of the side reactions on Cys, Met and Trp had been determined, the best way to recover the loss of identification of these peptides is to set variable modification(s) during database search accordingly. To investigate the effectiveness of this approach, the acquired spectra for the NaIO_4_ treated samples were searched by setting the variable modification of the three amino acids, Met (+16 Da), Cys (+16 Da) and Trp (+16 and +32 Da) separately. Based on the search results, the oxidation rate, i.e. percentage of the oxidized residues among all residues in the identified peptides, could be determined (Fig. 5a). Obviously Met was more vulnerable to oxidation than the other two residues as shown by its oxidation rate of 88.0% while the rates for Cys and Trp were only 33.0% and 18.5% when the sample was treated with 1 mM NaIO_4_. Because the high oxidation rate of Met, the setting of variable modifications was very effective to recover the identifications of these peptides. The occurrence frequency of peptide containing Met in the NaIO_4_ treated sample had back to the normal level (Fig. 5b), while the Cys and Trp occurrence frequency was returned considerably when corresponding oxidation modification was set during database search (Fig. 5c–d). The above results demonstrated that setting the oxidation variable modification in database search was efficient for the identification of peptides containing oxidized Cys, Met and Trp residues.

**Figure 6:**
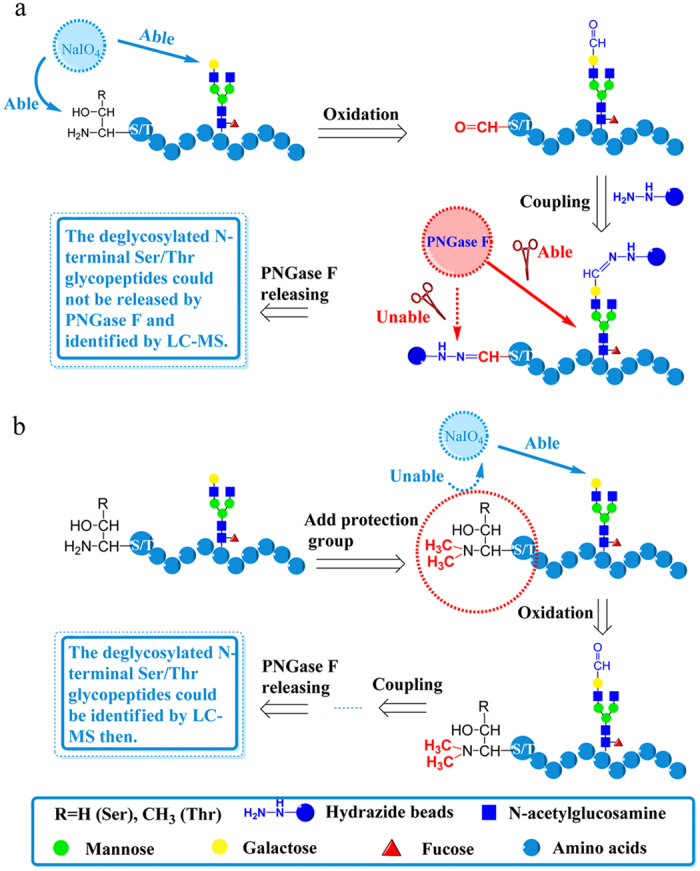
Schematics for the enrichment of glycopeptides by conventional HC method and the new PNP strategy. (**a**) The conventional HC method is failed to identify glycosites on peptides with N-terminal Ser/Thr. (**b**) PNP strategy enables the identification of glycosites on peptides with N-terminal Ser/Thr.

However, the peptides with N-terminal Ser/Thr oxidized remains insufficiently identified by simply setting variable modifications. It was reported that the vicinal amino alcohols of the N-terminal Ser/Thr residue of peptides could be oxidized to aldehyde groups by NaIO_4_^36^,^37^. If the aldehyde groups were formed at the N-termini of glycopeptides, they would be captured by hydrazide beads (Fig. 6a) as well but not be released by PNGase F from the beads any more. This is different to the glycopeptides captured on the hydrazide beads by reaction with aldehyde groups on glycan chain that could be easily released by PNGase F. As a result, the N-terminal Ser/Thr de-glycopeptides failed to be identified by conventional HC method. As shown in Fig. 2b, the peptides with N-terminal Ser/Thr account for 14.6% of all de-glycopeptides identified by HILIC. If these de-glycopeptides can be identified by HC method, the glycoproteome coverage will be improved.

### A peptide N-terminal protection strategy for the identification of de-glycopeptides with N-terminal Ser/Thr

To rescue identification of those de-glycopeptides with N-terminal Ser/Thr, we proposed a peptide N-terminal protection strategy. In this strategy, the primary amine on peptide is chemically blocked (Fig. 6b). Thus the N-terminal vicinal amino alcohol will not exist and the N-terminal Thr/Ser should not be oxidized by NaIO_4_ anymore. The selection of a reaction to block the primary amine is very important. Generally, the reaction should be quick and complete, moreover, it should not generate new side reactions on peptides. It has been reported that the reaction of formaldehyde with amino group through reductive amination reaction is very quick and enables nearly complete conversion of amino groups to the dimethyl derivatives without any detectable byproducts^38^. Moreover, this reaction does not change the ionic state of the peptides^39^. Because of these features, dimethyl labeling with different isotope reagents has also been widely used in quantitative proteomics^39^,^40^. Taking all these advantages, we adopted this reaction as the protection strategy to block the N-terminal primary amine on peptides. The scheme for the proposed peptide N-terminal protection (PNP) strategy was shown in Fig. 6b. Briefly, before oxidation, the primary amines on peptide N-termini and lysine side chains are blocked by dimethyl labeling, which is based on the reaction of peptide primary amines with formaldehyde to generate a Schiff base that is rapidly reduced by the occurrence of cyanoborohydride sodium in the reaction buffer^38^, then the subsequent procedures were just the same as the conventional HC method for enrichment of glycopeptides. To evaluate the labeling efficiency of this protection strategy, the labeled and enriched de-glycopeptides from 50 μg mouse liver proteins were subjected to 1D LC-MS/MS analysis, and it was found that 2987 (99.5%) of the 3001 identified peptides were labeled with dimethyl groups on their N-termini (The identification numbers here were for the total peptide identifications that did not remove the redundant identifications. The identification numbers otherwise were all for unique identifications.). Among them, 2399 were de-glycopeptides and all of them were N-terminal labeled, indicating the high reaction efficiency of dimethyl labeling for amine groups on the glycopeptides.

**Figure 7:**
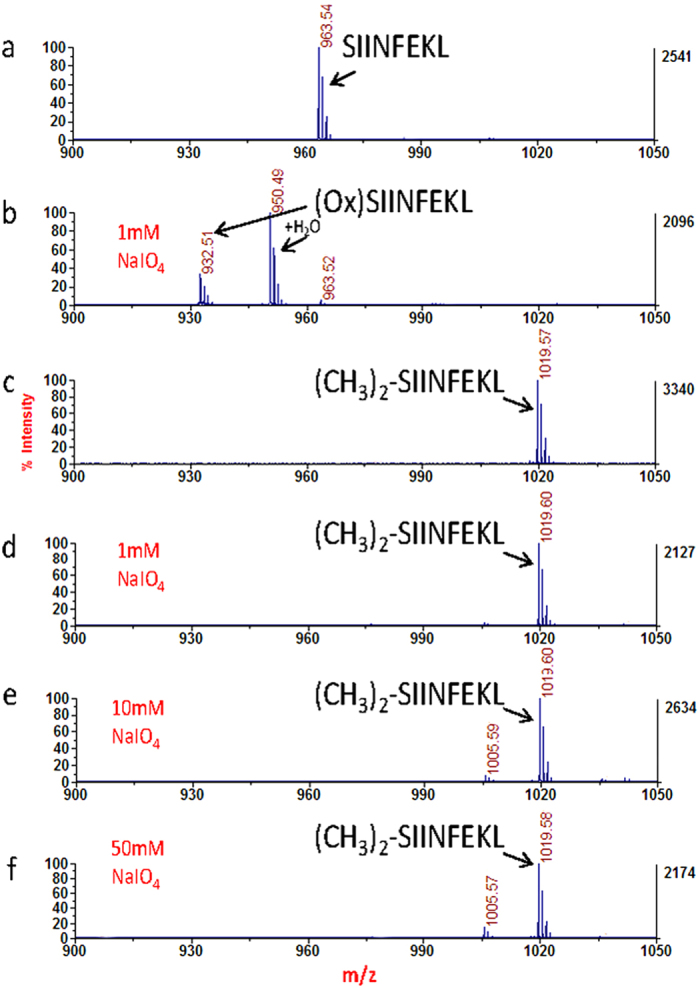
PNP strategy prevents the oxidation of peptides with N-terminal Ser. MALDI mass spectra of the standard peptide SIINFEKL with N-terminal serine: (**a**) Original peptide. (**b**) Peptide oxidized at 1 mM NaIO_4_. (**c**) Dimethyl labelled peptide. (**d**) (**e**) and (**f**) Dimethyl labelled peptide oxidized at 1 mM NaIO_4_, 10 mM NaIO_4_ and 50 mM NaIO_4_, respectively.

We then investigate if peptides with the N-terminal Ser/Thr residues could be oxidized after they were dimethyl labeled. The standard N-terminal Ser peptide SIINFEKL was used to evaluate the feasibility of this protection strategy. It was observed that this peptide was almost fully oxidized by incubating with 1 mM NaIO_4_, as a peptide N-terminal oxidized derivate (-31 Da) and its hydrates (+18 Da) were dominant in the MALDI mass spectra (Fig. 7a,b). When the dimethyl labeled peptide was subjected to the same oxidation as conventional HC method did, we could not find any noticeable peaks corresponding to the oxidation of the dimethyl labeled peptide, which indicated no oxidation product was generated. No oxidized derivative peaks were observed even though the NaIO_4_ concentration was increased to 50 mM (Fig. 7c–f). The similar result was also observed for the standard peptide with N-terminal Thr (Fig. S3). All these data indicated that the PNP strategy can effectively prevent the oxidation of peptides with N-terminal Ser/Thr residues. This is because the vicinal amino alcohols are no longer existed at the peptide N-termini after the labeling. Therefore, applying this PNP strategy to recover the identification of the lost de-glycopeptides with N-terminal Ser/Thr by HC method is theoretically feasible.

**Figure 8:**
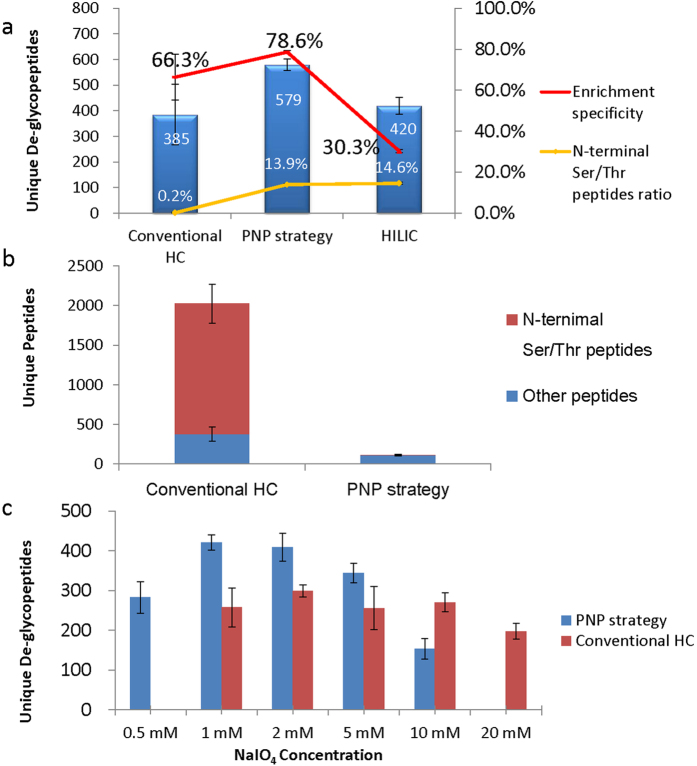
Performance of the PNP strategy. (**a**) Comparison of the number of de-glycopeptide identifications, ratio of N-terminal Ser/Thr de-glycopeptides and glycopeptide enrichment specificity for conventional HC method, PNP strategy and the HILIC method. (**b**) Comparison of hydroxylamine released peptides from hydrazide beads after enzymatic release of glycopeptides released by PNGase F. (**c**) The number of identified de-glycopeptides at different NaIO_4_ concentration for conventional HC method and PNP strategy. Tryptic peptides from mouse liver were used as the samples. Above data were averaged from 3 replicate experiments (error bars represent the standard deviation).

We then investigated the performance of PNP strategy for the glycoproteomics analysis. For comparison, three approaches, the new HC approach with PNP strategy (using 1 mM NaIO_4_ for oxidation), the conventional HC approach (using 2 mM NaIO_4_ for oxidation) and HILIC approach, were individually applied to analyze the mouse liver glycoproteome by loading the same amount of test sample (50 μg mouse liver proteins). It was observed that the ratio of N-terminal Ser/Thr de-glycopeptides increased from 0.2% of conventional HC approach to about 13.9% with the PNP approach, which was similar with HILIC method (14.6%). Based on the in silico digestion of all proteins in mouse proteome database, the peptides with N-terminal Ser/Thr accounts for 15.2% of all tryptic peptides (Fig. S4). Therefore the percentage of such peptides achieved with PNP approach reaches very close to the proteome level. Clearly the dimethyl labeling successfully prevented the NaIO_4_ oxidation of N-terminal Ser/Thr on peptides, resulting in efficient identification of N-terminal Ser/Thr de-glycopeptides using the PNP strategy (Fig. 8a). As to the enrichment specificity, the PNP strategy (78.6%) is also higher than that with the conventional HC method (66.3%) and HILIC method (30.3%). Because of the high specificity and the recovering of de-glycopeptides with N-terminal Ser/Thr, the new HC method yields much more de-glycopeptides identifications (Fig. 8a). Interestingly, the glycosites identified by these three methods were quite complementary, especially for the HC method and HILIC method (Fig. S6), which is consistent with previous literatures^41^,^42^.

In addition to improvement of de-glycopeptides identification, it’s also of interest to know whether many oxidized N-terminal Ser/Thr peptides are captured by the HC beads in the conventional and our improved HC methods. The enrichment of glycopeptides by HC beads was performed using the mouse liver sample as above. After PNGase F treatment and removing the de-glycopeptides, the hydrazide beads of both the conventional HC method and the PNP strategy were then subjected to a hydroxylamine treatment. As hydrazone bond is produced through the reaction of hydrazide with aldehyde on N-terminal Ser/Thr peptides, hydroxylamine treatment could effectively cleave the hydrazone bond between the N-terminal Ser/Thr peptides and the beads to generate N-terminal oxime peptides. Following this hydroxylamine treatment, the supernatants were collected and analyzed by 1D LC-MS/MS. As shown in Fig. 8b, about 2000 unique peptides could be identified from the supernatant from the conventional HC method beads, and about 80% of them were peptides of N-terminal Ser/Thr. While using the PNP strategy, only about 100 unique peptides were identified and less than 1% peptides were N-terminal Ser/Thr peptide. Clearly the hydroxylamine treatment experiment confirmed that peptides with N-terminal Ser/Thr were covalently captured by HC beads in the conventional method.

In conventional HC method, as enormous N-terminal Ser and Thr peptides coexist with glycopeptides, the total amount of the groups to be oxidized (cis-diols on glycans and vicinal amino alcohols on N-terminal Ser/Thr peptides) are much higher than those in the PNP strategy. Thus NaIO_4_ concentration used in conventional HC method may not be optimal in this PNP strategy. Therefore we performed a series of experiments to capture glycopeptides from aliquots of mouse liver protein digests oxidized with NaIO_4_ at 0.5, 1, 2, 5, and 10 mM for PNP strategy and 1, 2, 5, 10 and 20 mM for conventional HC method. As shown in Fig.8c 1 mM NaIO_4_ is sufficient for carbohydrate oxidation of glycopeptides in PNP strategy, while higher concentration of NaIO_4_ is needed in conventional HC strategy, which fits well with our anticipation. It should be noted that higher NaIO_4_ concentration could impair the identification of glycosites in both methods, partially due to the potential more severe side reactions occurring on peptides containing Cys and Trp.

### Comprehensive glycoproteomics analysis by the PNP strategy

**Figure 9:**
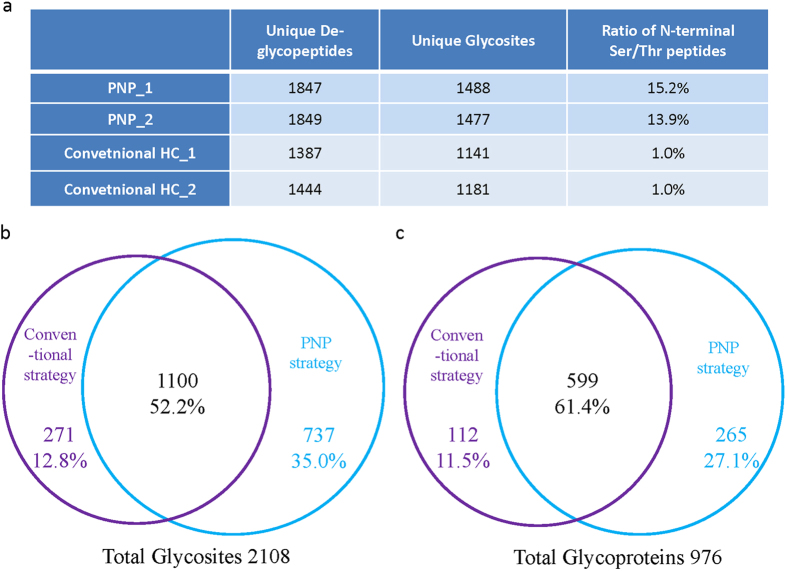
Comparison of the glycoproteome datasets obtained by the PNP strategy and the conventional HC approach. (**a**) Summary of the identification results. (**b**) Overlap of glycosites. (**c**) Overlap of glycoproteins.

Then we performed the comprehensive glycoproteome analysis of mouse liver tissues using the improved HC method with the PNP strategy. For comparison, conventional HC approach was also applied to analyze the same sample. Briefly each aliquot of 500 μg protein digest of mouse liver tissues was oxidized in 400 μL oxidation buffer containing 1 mM NaIO_4_ for PNP strategy and 2 mM for conventional HC strategy. Two replicate runs of SCX-RPLC MS/MS were then applied to analyze the enriched de-glycopeptides. The number of identified de-glycopeptides and glycosites were summarized in Fig. 9a. Compared with the conventional HC approach, the PNP strategy identified about 30% more de-glycopeptides. And the ratio of the identified N-terminal Ser/Thr de-glycopeptides reached to 13.9% which is close to its proteome level, while this ratio was only 1.0% when the conventional HC method was applied (Fig. 9a). By combining two replicate results, totally 1837 unique glycosites corresponding to 864 unique glycoproteins were identified by PNP strategy, and only 1371 unique glycosylation sites corresponding to 711 unique glycoproteins were identified by the conventional HC approach. And 34.0% more glycosites were identified in the PNP strategy clearly demonstrated that the new method surpasses the conventional method for comprehensive glycoproteomics analysis (Fig. 9b, Fig. S7).

Interestingly, the PNP strategy was found to be complementary in glycoproteomics analysis with the conventional HC approach. The overlaps of the two technical replicates in each method were about 60 ~ 70%, while the overlap between these two methods was only about 50% (Fig. 9b, Fig. S7). In addition to recovering the N-terminal Ser/Thr deglycosylated peptides, the change of fragmentation behavior due to the labeling may also contribute to the high complementary^39^. Thus the glycoproteome coverage could be improved by combining these two methods. Finally, 2108 unique glycosites corresponding to 976 unique glycoproteins were identified by combining the datasets of these two methods (Fig. 9b,c). Distribution of the number of glycosites on glycoproteins was investigated and it was found that approximately 50% glycoproteins carried a single N-linked sugar chain and 8.6% contained 5 or more N-glycosylation sites (Fig. S9). The average glycosites for each glycoprotein increase from 1.9 with conventional HC method to 2.1 with PNP strategy, which indicated that the identification of glycosites on peptides with N-terminal Ser/Thr could significantly improve the glycoproteome coverage. The HC methods were found to be highly complementary to HILIC method as mentioned in the above section (Fig. S6). Besides, HC methods and lectin methods were also highly complementary. As shown in Fig. S8, the overlap of large scale mouse liver glycoproteome obtained in this work with the dataset acquired from lectin affinity method was low, indicating high complementary of the identifications^43^. This is consistent with the results reported in previous literature^30^. Therefore, to achieve a more comprehensive glycoproteome analysis, combined using of all above methods is required.

## Discussion

Investigating side reactions and finding ways to prevent the side reactions in peptide/protein derivatization experiments are of broad interest to the protein science community. The side reactions could be investigated by performing derivatization experiments individually with a series of synthesized oligopeptides. However, this conventional approach is time consuming and labor intensive. In this study, we present a high throughput proteomics approach to achieve this goal. It is based on the fact that peptides with unknown modifications are in general unable to be identified by the normal database search. If there is any side reaction occurring on a residue, the peptides will be modified. And because such modified peptides fail to be identified, the occurrence frequency for the peptides with the modified amino acid residues must decrease. This gives us a clue to determine which type of amino acid residue might be modified. By using this approach, we successfully found that mainly two types of side reactions occurred on peptide backbones during the glycan oxidation step in HC method, namely the oxidation on the Met, CamCys and Trp residues, and the aldehyde formation reaction on N-terminal Ser/Thr residues. In principle, this approach is readily applicable to find the side reactions in other peptide derivatization experiments.

Then we presented approaches to prevent the loss of identifications resulted from such side reactions in HC method. For the de-glycopeptides with oxidized Met, CamCys and Trp residues, their identifications can be achieved by setting variable modifications. However, the identification of de-glycopeptides with N-terminal Ser/Thr residues cannot be achieved by simply setting variable modification during the database search. This is because the aldehyde groups on N-terminal of such glycopeptides react with hydrazide groups resulting in capture of these peptides on the HC beads. Therefore these peptides cannot be released by PNGase F cleavage, and thus fail to be identified by LC-MS/MS. To overcome this problem, we presented a PNP strategy by blocking the N-terminal amino groups with dimethyl groups, which effectively prevented the oxidation of N-terminal Ser/Thr on peptides. As a result, de-glycopeptides with N-terminal Ser/Thr could be efficiently identified with the developed PNP strategy.

Peptides with N-terminal Ser/Thr residues represent roughly 15.2% of proteome, however at least 30% more de-glycopeptides were identified by this new strategy in comparison with that using the conventional HC method. Clearly in addition to the recovery of de-glycopeptides with the N-terminal Ser/Thr, there are other reasons for the excellent improvement. We believe that the improved enrichment specificity could be one of the reasons. In addition to the oxidation of glycopeptides, many non-glycopeptides with N-terminal Ser/Thr could also be oxidized and coupled to the hydrazide beads in conventional HC method. As illustrated in Fig. S5, the covalent binding of these peptides makes the beads surface changing from hydrophilic to hydrophobic, from neutral to charged. Therefore, many non-glycosylated peptides may be adsorbed on the hydrazide beads through the hydrophobic interaction and/or charge-charge interaction which compromise the enrichment specificity. While in the newly developed PNP strategy, these peptides are no longer be oxidized and cannot be immobilized onto the beads, which ends up with much less non-specific adsorption of peptides. Above allegation was confirmed by the experiment of hydroxylamine treatment (Fig. 8b), which was performed after the PNGase F enzymatic release of glycopeptides. Among the 2044 unique peptides identified from the hydroxylamine treatment of HC beads of the conventional method, about 80% of them were N-terminal Ser/Thr peptide. While the corresponding number and percentage was 113 and less than 1% using the PNP strategy. Clearly a large amount of N-terminal Ser/Thr peptides were captured onto the HC beads in the conventional method as expected. And much more peptides with N-terminal amino acids other than Ser/Thr (380 in conventional method vs 112 in the new method) were identified in the conventional method. These peptides were retained on the hydrazide beads mainly through the non-specific interaction with the captured N-terminal Ser/Thr peptides on the beads. Clearly the PNP approach is able to reduce the non-specific binding. As a result, the PNP approach yields the higher specificity (78.6%) over the conventional HC method (66.3%) in glycoproteome analysis.

It should be noted that, higher NaIO_4_ concentration could impair the identification of de-glycopeptides in both approaches. This is because more severe side reactions could occur when higher NaIO_4_ concentration is used. As glycoproteomics analysis could be performed using only 1 mM NaIO_4_ for carbohydrate oxidation in the developed PNP strategy, the potential side reaction effect was reduced significantly. Though the peptides with oxidized Met, Trp and CamCys could be identified by setting variable modifications in the database searching, this approach significantly increases the search space and decreases the identification sensitivity. The setting of variable modification should be cautious. It should be adopted only when the extent of the side reaction is significant and the setting of modifications surely increases the number of peptide identification. Based on the data we obtained in this study, we believe that the setting of oxidation modification on Met in database searching is essential, while the setting of oxidation on CamCys and Trp is necessary only when high NaIO_4_ concentration is used.

## Methods

### Ethics statement

This study was approved by the Dalian Institute of Chemical Physics Ethics Committee. All experiments were performed in accordance with Ethics Committee’s guidelines and regulations.

### Reagents

Formic acid (FA) and sodium cyanoborohydride (NaBH_3_CN) were provided by Fluka (Buchs, Germany). Acetonitrile (ACN, HPLC grade) was purchased from Merck (Darmstadt, Germany). All the other chemicals and reagents were purchased from Sigma (St. Louis, MO). Sep-Pak C18 cartridges were provided by Waters (Milford, MA). Fused silica capillaries with 75 μm i.d. and 200 μm i.d. were obtained from Polymicro Technologies (Phoenix, AZ). All the water used in this experiment was prepared using a Mill-Q system (Millipore, Bedford, MA).

### Protein Sample Preparation

Adult female C57 mice were purchased from Dalian Medical University (Dalian, China). As described in our previous work^44^,^45^, the liver tissues were lysed in a homogenization buffer, consisting of 8 M urea, 1% Triton X-100 v/v, 65 mM DTT, 1 mM EDTA, 0.5 mM EGTA, 1 mM PMSF, 10 μL of protease inhibitor cocktail for 1 mL of homogenized buffer, phosphatase inhibitors (1 mM NaF, 1 mM Na_3_VO_4_, 1 mM C_3_H_7_Na_2_O_6_P, 10 mM Na4O_7_P_2_), and 40 mM Tris-HCl at pH 7.4. The protein concentration was determined by Bradford assay. The extracted proteins were precipitated by chloroform/methanol precipitation. After washing with methanol, the pellets were resuspended in denaturing buffer containing 100 mM Triethyl Ammonium Bicarbonate (TEAB, a versatile buffer compatible with the digestion and dimethyl labeling reaction) (pH 8.0) and 8 M urea. The protein concentration was determined again by Bradford assay.

### Protein Digestion and peptide dimethyl labelling

The proteins were reduced by DTT at 37 °C for 2 h and alkylated by iodoacetamide in the dark at room temperature for 40 min. Then, the solutions were diluted to 1 M urea with 100 mM TEAB (pH 8.0), and trypsin was added with the weight ratio of trypsin to protein at 1/25, and incubated at 37 °C overnight. All of the resulting peptide solution was stored at -80 °C. To the resulted tryptic digest (1 mg in 1 mL of 100 mM TEAB solution), 100 μL of CH_2_O (4%, v/v) was added followed with the addition of 100 μL of freshly prepared NaBH_3_CN (0.6 M). The resultant mixture was incubated for 1 h at room temperature. Then, 20 μL of ammonia (10%) added to consume the excess labeling reagents. After the labeled peptide mixture was acidified by addition of 10 μL of FA , it was desalted by the solid phase extraction (SPE) column and dried down in a Speed Vac.

**Glycopeptide Enrichment.** The glycopeptide enrichment by HC method was similar to that reported by Tian *et al* 21. Briefly, 1 mg of the dried tryptic peptides was reconstituted in oxidation buffer (100 mM NaAc, 150 mM NaCl, pH = 5.5) and NaIO_4_ was added to reach different final concentrations varied from 1 mM to 20 mM. The reaction was kept in dark for 1 h and quenched by adding sodium thiosulfate with final concentration two times of that of NaIO_4_. Then the oxidized peptides were coupled to 50 μL Affi-Gel Hz hydrazide beads (slurry volume) (Bio-Rad, USA) overnight at room temperature. The glycopeptides-coupled beads were washed extensively and sequentially with sodium chloride (1.5 M), ACN/H_2_O (80/20, v/v), and ammonium bicarbonate (100 mM, pH 8.3). The beads was resuspended in a minimum volume of ammonium bicarbonate solution (25 mM, pH 7.5) with 3 μL (500 U/μL) of PNGase F (New England Biolabs) and incubated overnight at 37 °C, which leaves a 0.9858 Da mass shift on the previously glycosylated site and will facilitate the identification of glycosylated sites by MS (it should be noted that chemical deamidation can also generate a 0.9858 Da mass shift on non-glycosylated asparagine and glutamine which may lead to false positive identifications^46^). Deglycosylated glycopeptides washed and dispersed in 80% ACN/2% TFA (v/v, 400 μL). Then 1 mg mouse liver glycoprotein digest were dissolved in 80% ACN/2% TFA (v/v, 100 μL) and the solution was added to the HILIC resin suspension in the centrifuge tube. After incubation under gentle agitation at room temperature for 10 min, the supernatant was discarding after centrifuge. The HILIC resins were washed three times with 80% ACN/0.1% TFA (v/v, 400 μL) to remove the non-glycopeptides. Then glycopeptides were eluted with 100 μL 0.1%TFA (v/v), 30%ACN/0.1%TFA (v/v), and 0.1%TFA (v/v), respectively. The eluted peptides were collected and combined. After lyophilization, 100 μL of 20 mM NH_4_HCO_3_ containing 500 U PNGase F was added and incubated at 37 °C for overnight for deglycosylation of the glycopeptides.

### Online RP-SCX-RP Multidimensional Separation and Mass Spectrometry Analysis

The lyophilized de-glycopeptides were resuspended in 0.1% FA. The automated sample injection and multidimensional separation using the RP-SCX-RP system were constructed as previously described^47^, and the RP segment of the RP-SCX biphasic column was used as the sample loading column to reduce the sample loss. The resuspended de-glycopeptides were loaded onto the biphasic column, and then, a 120 min RP gradient nanoflow LC-MS/MS (0 mM) was applied at first to transfer the peptides retained on the RP segment to the SCX monolithic of the biphasic column. Then a series stepwise elution with salt concentrations of 50, 100, 200, 300, 400, 500 and 1000 mM NH_4_AC (pH 2.7) was used to elute peptides from SCX monolithic column to an in-house packed 75 μm i.d. and 15 cm length C18 separation column (3 μm, 120 Å). Each salt step lasted 10 min followed by 15 min equilibrium with 0.1% FA in water.

RPLC-MS/MS analysis was performed using a quaternary surveyor MS pump (Thermo, San Jose, CA) and LTQ-Orbitrap Velos (Thermo, San Jose, CA). For the RPLC separation, 0.1% FA in water and in acetonitrile were used as mobile phases A and B, respectively, and the flow rate was adjusted to ~300 nL/min after splitting. The 200 min gradient elution was performed with a gradient of 0-3% B in 5 min, 3-25% B in 145 min, 25-35% B in 10 min, 35%-80% B in 3 min, 80% B in 7 min, 80-100% B in 3 min and 100% B lasted 27 min. Other gradients for 1 D LC-MS/MS analysis were just referred to the 200 min gradient and elongated or shorten the time of 3-25% B gradient as needed. A spray voltage of 2.2 kV was applied between the spray tip and the MS interface. The temperature of the ion transfer capillary was 250 °C. The LTQ-Orbitrap Velos mass spectrometer (Thermo, San Jose, CA) was operated in data-dependent MS/MS acquisition mode. Full mass scan performed in the Orbitrap analyzer was acquired from m/z 400 to 2000 (R = 60000 at m/z 400). The 20 most intense ions from the full scan were selected to fragmentation via collision induced dissociation (CID) in the LTQ. The dynamic exclusion function was set as follows: repeat count 2, repeat duration 30 s, and an exclusion duration of 60 s.

All MALDI-TOF mass data were obtained on AB Sciex 5800 MALDI-TOF/TOF mass spectrometer (AB Sciex, CA) equipped with a pulsed Nd/YAG laser at 355 nm. DHB (2,5-dihydroxybenzoic acid, 25 mg/mL in ACN/H_2_O/H_3_PO_4_ (70/29/1 v/v/v)) was used as the matrix for the analysis of peptides. Peptide sample solutions (0.5 μL) were deposited on the MALDI plate and dried at room temperature, and then DHB matrix solution (0.5 μL for peptide analysis) was deposited.

### Protein and Peptide Identification

All MS/MS spectra were searched using the MaxQuant version 1.3.0.05 against a composite International Protein Index (IPI) database (IPI mouse 3.87). Carbamidomethylation on cysteine (C, +57.0215 Da) was set as a fixed modification for all the searches. One or more following variable modifications were set: deamidation (N, +0.9858 Da), the oxidation on methionine (M, +15.9949 Da), cysteine (C, +15.9949 Da), and tryptophan (W, +15.9949, +31.9898 Da). For the identification of peptides released by the hydroxylamine treatment experiment, oximation on N-terminal serine (-16.0313 Da) and threonine (-30.0470 Da) was set as variable modification. To identify peptides from PNP strategy, single-plex label of dimethylation on lysine and peptide amino termini was set. Trypsin was set as the specific proteolytic enzyme with up to two missed cleavages allowed. The mass tolerance for the precursor ion was set to 10 ppm and 0.8 Da for the fragment ion. The peptide identifications with the false discovery rate ≤0.01 were accepted for protein identification. Only the identified deamidation sites which conformed to the N-glycosylation consensus sequence (N-!P-[S/T], N-X-C) were considered as glycosites^43^. The identified de-glycopeptides must include at least one glycosite defined above. The amino acid occurrence frequency distributions were determined based on the identified unique peptides. Specifically, the percentages of peptides containing each type of amino acid residue were determined by dividing the number of unique peptides containing the amino acid residue by the total number of identified unique peptides.

## Author Contributions

J.H., H.Q., M.Y. and H.Z. devised the strategy and experimental design. J.H. performed most of experiments, analyzed data and wrote the initial paper. Z.S., G.H., H.W., Y.Y., Z.Z. and J.Z. participated in the sample preparation. J.M., K.C. assisted with data analysis. H.W., H.Q., J.D., F.W., M.Y. and H.Z. contributed to data interpretation and the paper revision.

## Additional Information

**How to cite this article**: Huang, J. *et al*. A peptide N-terminal protection strategy for comprehensive glycoproteome analysis using hydrazide chemistry based method. *Sci. Rep.* **5**, 10164; doi: 10.1038/srep10164 (2015).

## Supplementary Material

Supplementary Information
