# Supplementary material for: A peptide N-terminal protection strategy for comprehensive glycoproteome analysis using hydrazide chemistry based method
Source: Sci Rep. 2015 May 11;5:10164. doi: 10.1038/srep10164 (PMC4426672; doi:10.1038/srep10164)
Supplement: Supplementary Information [file srep10164-s1.pdf]

Supplementary Information

A peptide N-terminal protection strategy for comprehensive glycoproteome analysis using hydrazide chemistry based method

Junfeng Huang[1,2], Hongqiang Qin[1], Zhen Sun[1,2], Guang Huang[1,2], Jiawei Mao[1,2], Kai Cheng[1,2], Zhang Zhang[1,2], Hao Wan[1,3], Yating Yao[1,2], Jing Dong[1], Jun Zhu[1,2], Fangjun Wang[1], Mingliang Ye[1] & Hanfa Zou[1]

[1]CAS Key Laboratory of Separation Sciences for Analytical Chemistry, National Chromatographic R&A Center, Dalian Institute of Chemical Physics, Chinese Academy of Sciences, Dalian, China; [2]University of Chinese Academy of Sciences, Beijing, China; [3]Shanghai Key Laboratory of Functional Materials Chemistry, East China University of Science and Technology. Correspondence should be addressed to H.F. Zou (hanfazou@dicp.ac.cn) or M.L. Ye (mingliang@dicp.ac.cn).

Supplementary Information contains:

Supplementary Figures S1-S9

Note: Table S1| **The percentages of peptides containing each amino acid residue overall.**

Table S2| **The percentages of peptides containing each amino acid residue at C-terminus.**

Table S3| **The percentages of peptides containing each amino acid residue at N-terminus.**

Table S4 | **The glycosites and glycoproteins identified in large scale studies using the conventional and improved HC method.**

Table S5 | **The de-glycopeptides identified in large-scale studies using the conventional and improved HC method.**

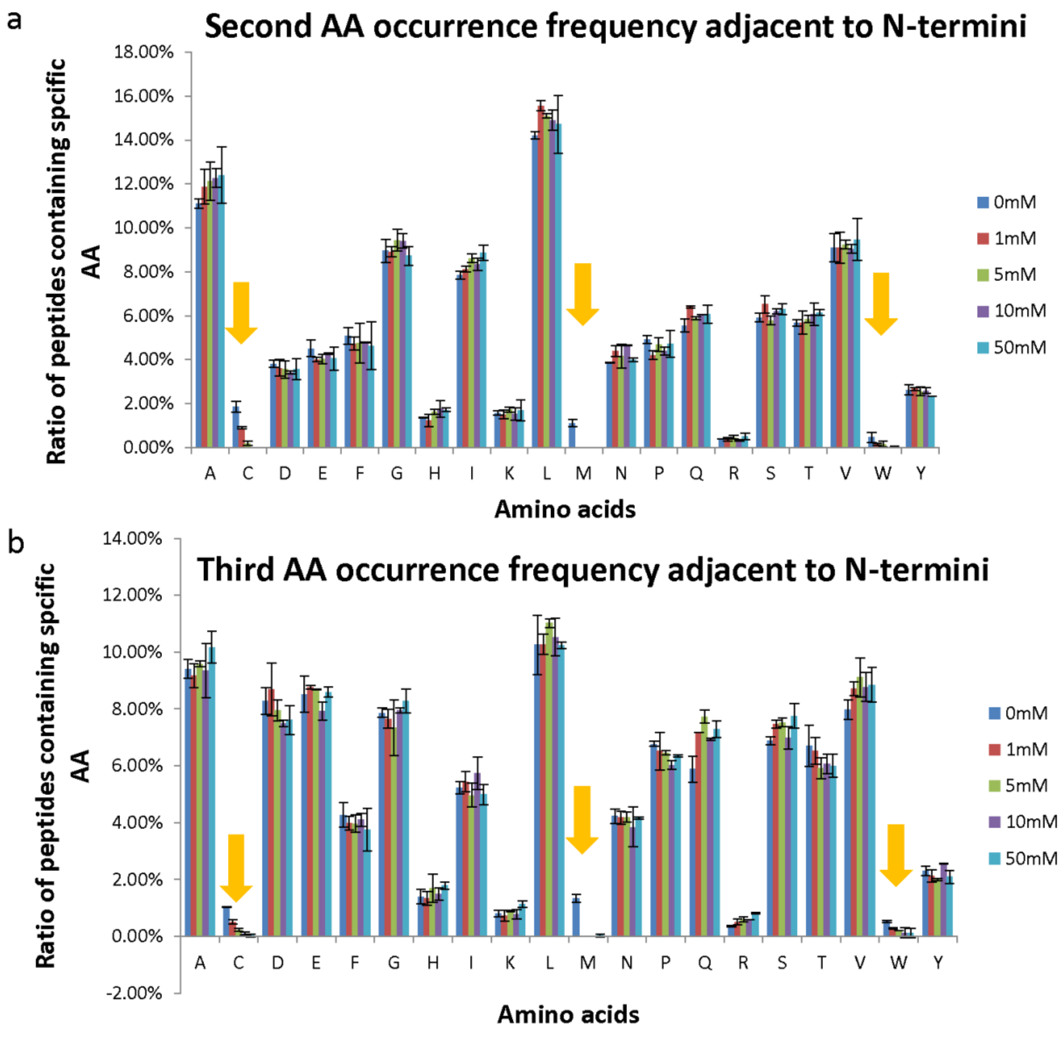

Figure S1 |Proteome-wide amino acid frequency check approach to locate the possible side reaction sites. (**a**) The percentage of peptides with the second residue from the N-termini with a specific type of amino acid residue in all peptides. (**b**) The percentage of peptides with the third residue from the N-termini with a specific type of amino acid residue in all peptides. Above data were averaged from 4 replicate experiments (error bars represent the standard deviation)

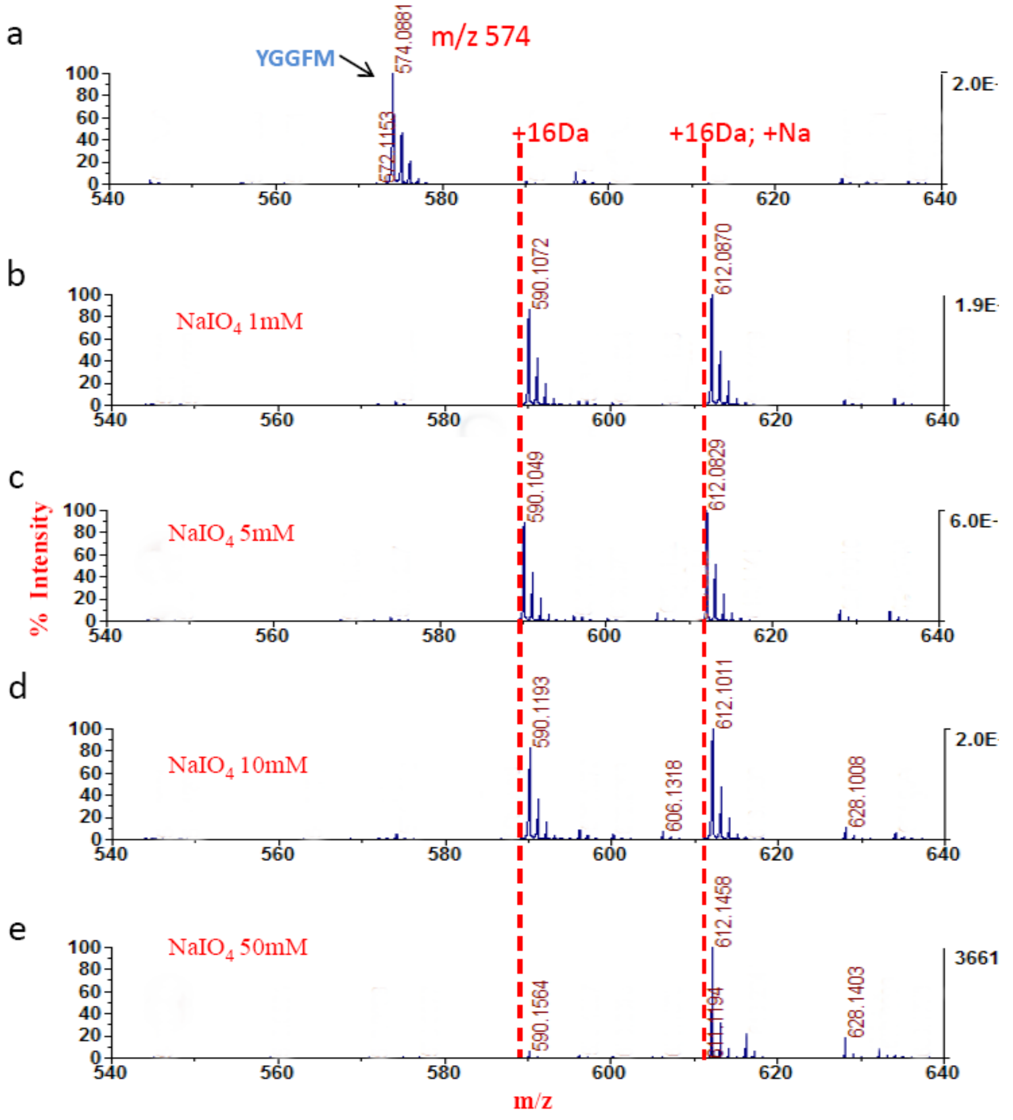

Figure S2 | The side reactions on Met. (a-e) MALDI mass spectra of the Met containing peptides YGGFM (0.01µg/µL, 0.5µL) oxidized at 0, 1, 5, 10, 50 mM $NaIO_4$.

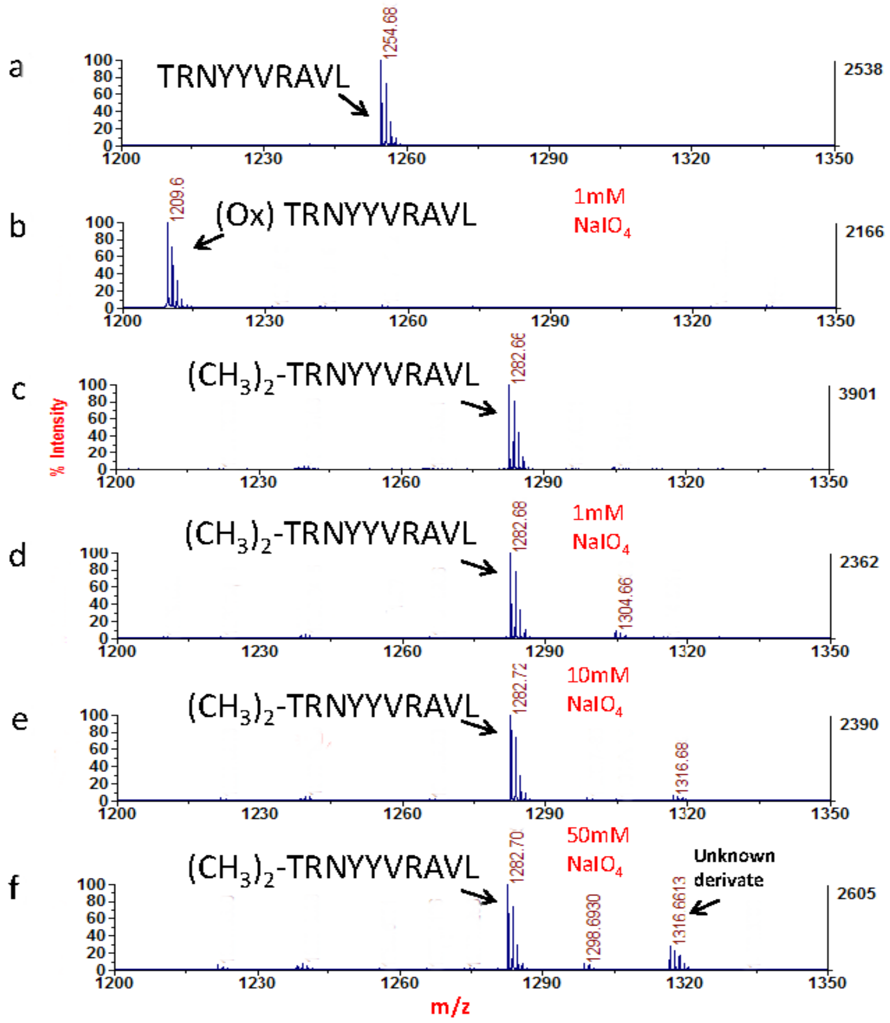

Figure S3 | PNP strategy prevents the oxidation of peptides with N-terminal Thr. MALDI mass spectra of the standard N-terminal Thr peptide TRNYYVRAVL (0.01μg/μL, 0.5μL) with or without dimethyl protection oxidized in periodate solutions with different concentrations: (**a**) without protection , without oxidation; (**b**) without protection, with oxidation (1mM NaIO4); (**c**) with protection, without oxidation; (**d**) with protection, with oxidation (1mM NaIO4); (**e**) with protection, with oxidation (10mM NaIO4); (**f**) with protection, with oxidation (50mM NaIO4).

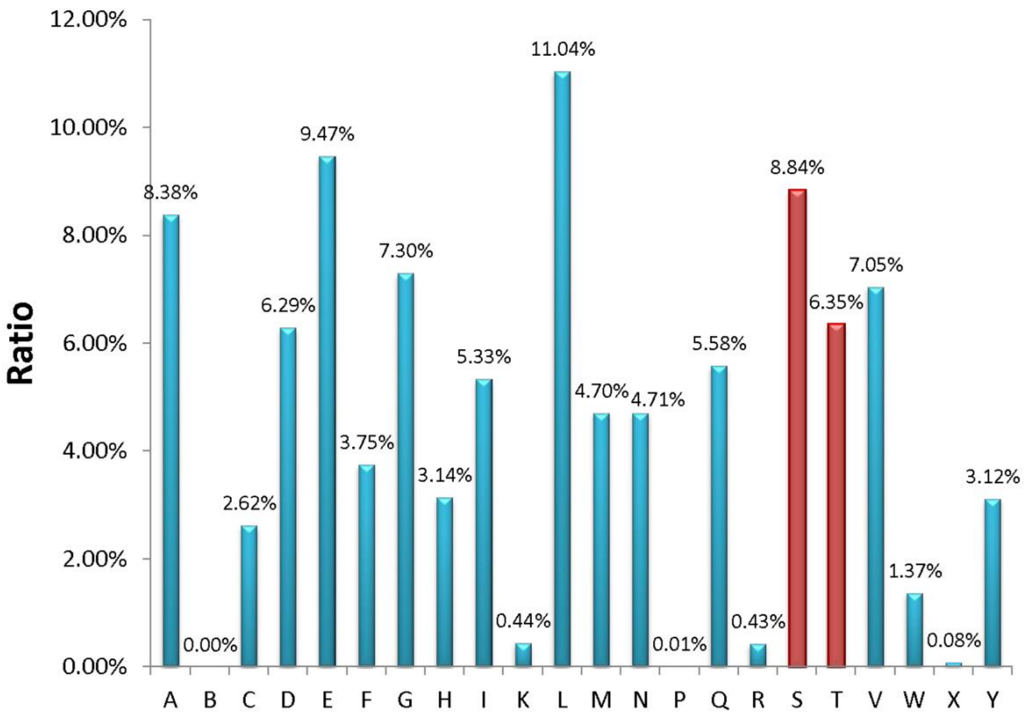

Figure S4 | The N-terminal amino acid residue distribution of in silico tryptic peptides of mouse proteome (database: IPI mouse v3.87).

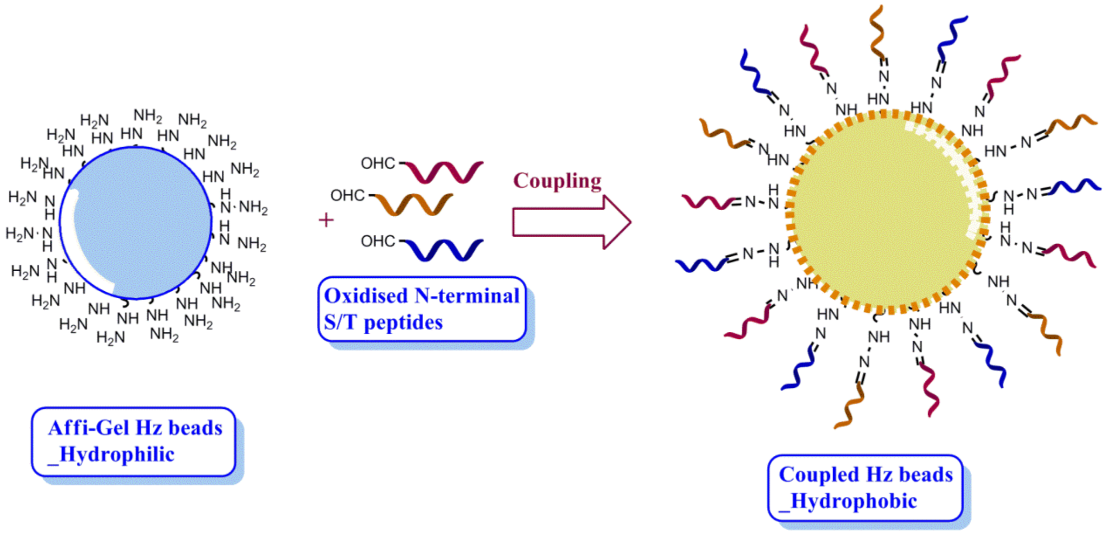

Figure S5 | Surface property change of hydrazide beads during glycopeptide enrichment in the conventional HC method. Enormous peptides with N-terminal Seri/Thr are also coupled onto the hydrazide beads in the conventional HC method because they are also oxidized. This makes the surface of the beads changing from hydrophilic to hydrophobic, from neutral to charged. While in the PNP strategy, the bead surface will not change significantly because these peptides will not be bound.

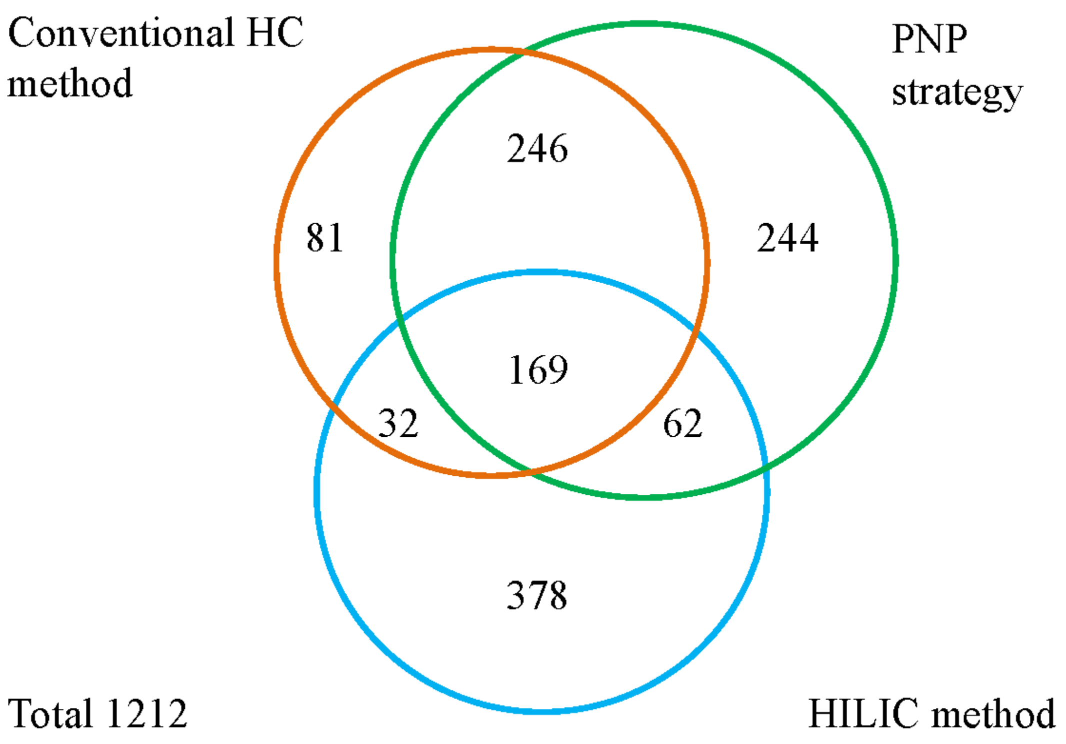

Figure S6 | Performance of the PNP strategy. Comparison of the overlaps of glycosites identifications for conventional HC method, PNP strategy and the HILIC method.

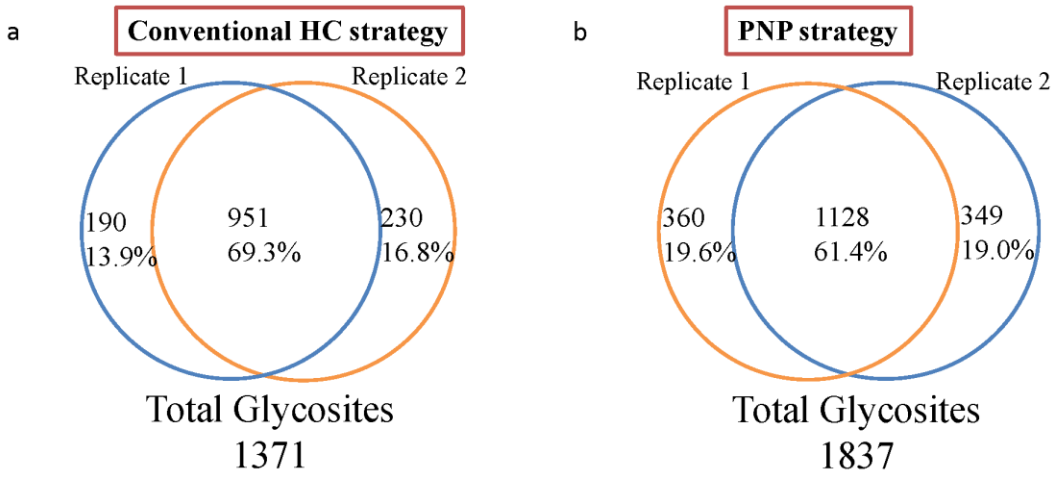

Figrue S7 | Comparison of replicate runs. (**a**) Conventional HC method. (**b**) PNP strategy.

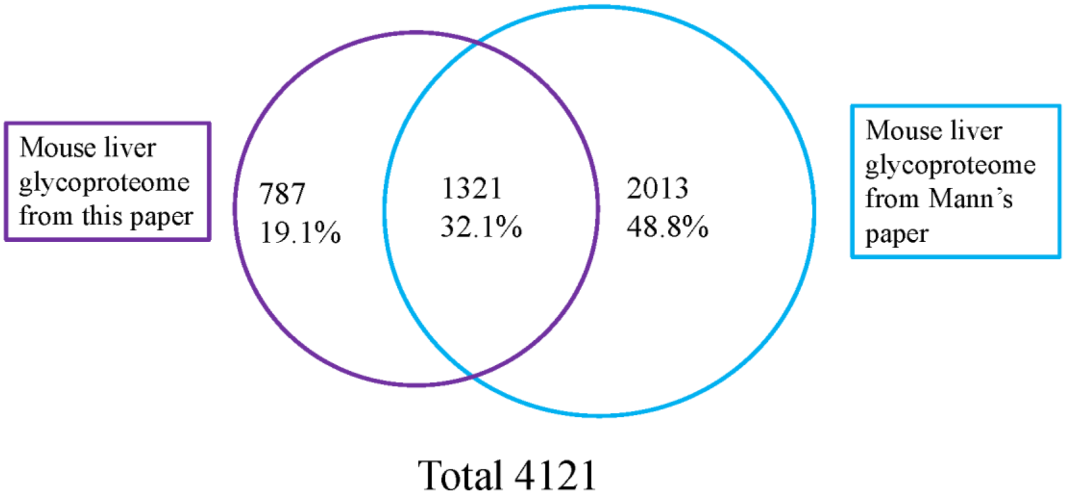

Figure S8 | Comparison of large scale mouse liver glycoproteome dataset acquired in this paper with the dataset acquired from lectin affinity methods reported in literature. Overlap of the glycosites identified in this work and in the work by using lectin affinity methods[1].

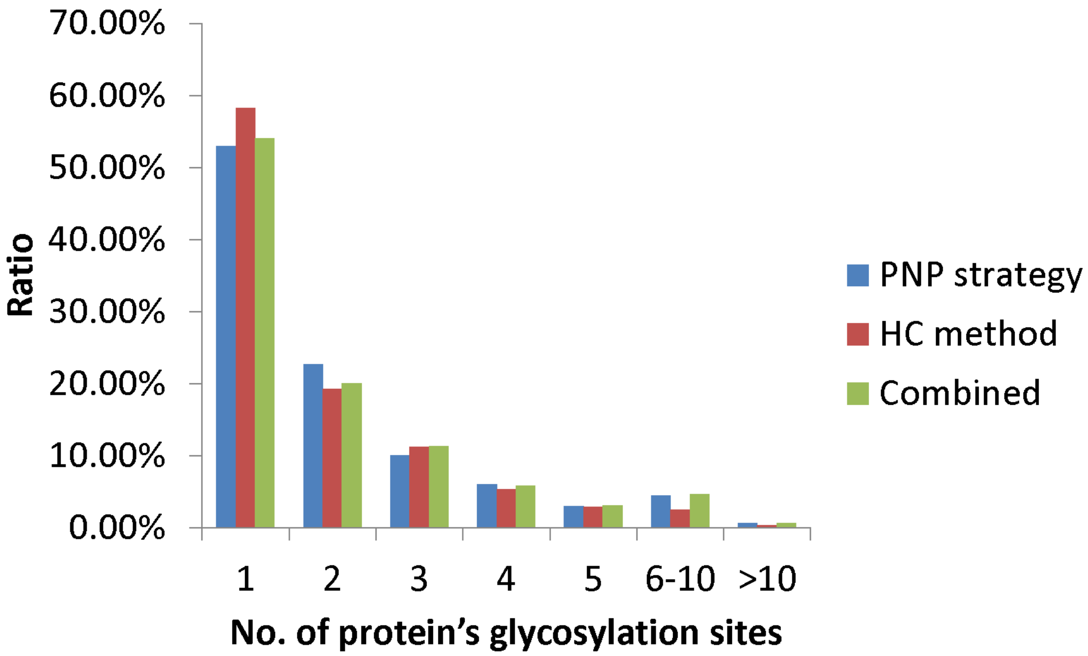

Figrue S9 | Distribution of singly and multiply glycosylated proteins. The ratio of identified proteins with multi-glycosites in PNP strategy is higher than the conventional HC method.

1. Zielinska, D.F., Gnad, F., Wisniewski, J.R. & Mann, M. Precision Mapping of an In Vivo N-Glycoproteome Reveals Rigid Topological and Sequence Constraints. *Cell* **141**, 897-907

(2010).

Table S1| The percentages of peptides containing each amino acid residue overall

$NaIO_4$	0mM_1	0mM_2	0mM_3	0mM_4	1mM_1	1mM_2	1mM_3	1mM_4	5mM_1	5mM_2	5mM_3	5mM_4	10mM_1	10mM_2	10mM_3	10mM_4	50mM_1	50mM_2	50mM_3	50mM_4
A	69.35%	69.21%	70.55%	71.93%	70.39%	71.30%	70.74%	71.35%	70.60%	69.56%	71.56%	68.98%	68.50%	69.11%	69.99%	70.58%	67.80%	68.87%	69.31%	69.53%
R	45.46%	44.47%	44.59%	42.73%	45.13%	44.84%	44.33%	44.03%	45.72%	46.81%	44.72%	44.55%	47.77%	44.35%	45.72%	45.42%	47.03%	47.50%	48.71%	49.52%
N	46.18%	43.74%	45.27%	44.34%	46.25%	45.27%	46.31%	45.76%	44.17%	46.61%	46.93%	42.53%	43.53%	45.55%	46.26%	46.13%	44.47%	42.34%	44.47%	45.57%
D	62.95%	59.42%	62.81%	60.34%	59.80%	60.67%	59.45%	59.97%	60.28%	60.62%	62.11%	57.56%	58.97%	57.97%	62.50%	59.64%	60.20%	58.06%	61.65%	60.74%
C	17.28%	16.03%	15.78%	16.92%	10.53%	10.82%	10.12%	10.36%	4.18%	4.27%	3.82%	3.87%	1.60%	1.57%	1.87%	1.78%	0.38%	0.08%	0.58%	0.70%
Q	51.15%	50.83%	50.66%	50.05%	52.37%	51.82%	50.61%	50.63%	51.79%	51.48%	53.17%	50.26%	50.35%	50.34%	51.87%	50.04%	50.71%	48.47%	50.64%	52.20%
E	66.55%	64.86%	66.73%	65.46%	67.89%	67.04%	67.25%	67.19%	65.95%	67.26%	67.34%	65.20%	66.76%	66.64%	68.72%	70.84%	65.09%	63.87%	67.05%	67.24%
G	65.20%	63.35%	63.16%	66.27%	65.20%	65.92%	65.15%	66.48%	64.26%	64.50%	69.45%	65.73%	64.81%	66.42%	63.70%	64.44%	64.18%	64.68%	64.35%	64.56%
H	21.56%	21.58%	23.27%	18.37%	20.46%	19.11%	19.60%	20.64%	21.31%	24.06%	21.31%	22.76%	23.92%	20.34%	23.26%	21.60%	28.44%	21.29%	25.80%	25.24%
I	59.89%	56.42%	58.06%	59.48%	59.47%	59.55%	59.86%	60.60%	58.87%	60.36%	62.71%	58.79%	60.15%	58.71%	60.63%	61.42%	60.80%	57.18%	59.78%	60.93%
L	67.78%	79.31%	79.32%	79.74%	82.37%	82.00%	81.91%	82.34%	81.59%	83.17%	87.94%	80.32%	82.27%	81.60%	83.22%	82.13%	82.39%	79.03%	83.20%	83.75%
K	64.26%	64.39%	65.95%	65.68%	64.21%	64.25%	64.86%	65.38%	64.33%	64.76%	64.62%	65.91%	63.28%	64.40%	65.37%	65.60%	66.14%	62.98%	64.35%	64.18%
M	16.00%	12.60%	12.40%	11.64%	0.26%	0.25%	0.35%	0.24%	0.27%	0.39%	0.20%	0.44%	0.21%	0.22%	0.27%	0.53%	0.23%	0.32%	0.19%	0.19%
F	46.60%	41.12%	41.94%	40.79%	42.17%	41.13%	42.23%	41.99%	41.67%	42.67%	48.14%	41.56%	45.69%	41.74%	45.39%	45.16%	44.47%	38.79%	45.17%	46.21%
P	62.56%	59.88%	59.58%	61.80%	58.95%	60.11%	61.20%	60.91%	58.53%	59.76%	62.01%	57.73%	57.79%	57.89%	59.02%	59.47%	59.52%	56.61%	58.62%	61.19%
S	62.10%	59.81%	60.02%	60.02%	57.96%	58.69%	58.46%	60.36%	58.46%	56.80%	62.91%	57.64%	57.79%	56.62%	58.02%	59.29%	58.47%	55.40%	56.69%	58.00%
T	59.97%	59.23%	61.54%	60.56%	56.51%	57.95%	55.21%	58.01%	56.04%	55.03%	59.60%	55.62%	55.63%	57.07%	57.09%	57.07%	54.85%	54.76%	54.57%	55.83%
W	8.62%	7.90%	7.74%	7.38%	6.45%	5.57%	5.35%	4.95%	5.33%	5.00%	5.03%	4.31%	3.96%	2.47%	3.81%	3.02%	3.91%	2.02%	3.80%	3.57%
Y	32.34%	29.94%	29.25%	29.42%	30.20%	30.18%	30.60%	29.83%	29.40%	29.39%	31.96%	28.65%	29.97%	30.67%	30.82%	30.67%	29.65%	25.73%	29.67%	30.34%
V	69.48%	75.72%	70.06%	71.77%	72.76%	69.88%	71.03%	71.59%	71.27%	71.66%	72.16%	70.47%	70.17%	69.78%	72.26%	71.64%	69.45%	70.73%	69.82%	69.34%

These data was based on the peptide identifications from the mouse tryptic peptide samples treated with different $NaIO_4$ concentration.

Table S2| The percentages of peptides containing each amino acid residue at the C-terminus

NaIO4	0mM_1	0mM_2	0mM_3	0mM_4	1mM_1	1mM_2	1mM_3	1mM_4	5mM_1	5mM_2	5mM_3	5mM_4	10mM_1	10mM_2	10mM_3	10mM_4	50mM_1	50mM_2	50mM_3	50mM_4
A	0.04%	0.12%	0.05%	0.05%	0.00%	0.06%	0.06%	0.08%	0.07%	0.07%	0.00%	0.09%	0.00%	0.22%	0.07%	0.09%	0.00%	0.00%	0.00%	0.06%
C	0.00%	0.00%	0.00%	0.00%	0.00%	0.00%	0.00%	0.00%	0.00%	0.00%	0.00%	0.00%	0.00%	0.00%	0.00%	0.00%	0.00%	0.00%	0.00%	0.00%
D	0.00%	0.00%	0.05%	0.05%	0.00%	0.00%	0.00%	0.00%	0.00%	0.00%	0.00%	0.00%	0.00%	0.00%	0.00%	0.00%	0.00%	0.00%	0.06%	0.00%
E	0.08%	0.08%	0.20%	0.11%	0.00%	0.00%	0.06%	0.08%	0.07%	0.13%	0.10%	0.09%	0.21%	0.15%	0.07%	0.09%	0.15%	0.08%	0.06%	0.06%
F	0.04%	0.04%	0.05%	0.05%	0.00%	0.06%	0.06%	0.08%	0.07%	0.07%	0.00%	0.09%	0.14%	0.15%	0.07%	0.00%	0.00%	0.08%	0.13%	0.06%
G	0.00%	0.00%	0.00%	0.00%	0.00%	0.00%	0.00%	0.00%	0.00%	0.00%	0.00%	0.00%	0.00%	0.00%	0.00%	0.00%	0.00%	0.00%	0.00%	0.00%
H	0.04%	0.15%	0.10%	0.00%	0.13%	0.12%	0.12%	0.16%	0.07%	0.13%	0.20%	0.09%	0.07%	0.07%	0.13%	0.00%	0.08%	0.08%	0.13%	0.13%
I	0.08%	0.12%	0.10%	0.00%	0.13%	0.06%	0.12%	0.08%	0.07%	0.20%	0.20%	0.09%	0.14%	0.07%	0.13%	0.09%	0.15%	0.08%	0.13%	0.06%
K	56.83%	57.65%	58.16%	58.94%	57.24%	57.14%	57.59%	57.54%	56.51%	55.42%	56.38%	56.59%	53.96%	56.69%	56.62%	56.00%	55.61%	55.24%	54.38%	53.35%
L	0.25%	0.12%	0.20%	0.38%	0.26%	0.43%	0.35%	0.47%	0.34%	0.39%	0.50%	0.44%	0.35%	0.37%	0.13%	0.53%	0.45%	0.16%	0.32%	0.38%
M	0.00%	0.00%	0.00%	0.00%	0.00%	0.00%	0.00%	0.00%	0.00%	0.00%	0.00%	0.00%	0.00%	0.00%	0.00%	0.00%	0.00%	0.00%	0.00%	0.00%
N	0.08%	0.04%	0.05%	0.11%	0.00%	0.06%	0.00%	0.00%	0.00%	0.00%	0.10%	0.00%	0.00%	0.00%	0.00%	0.00%	0.00%	0.00%	0.00%	0.00%
P	0.00%	0.08%	0.05%	0.11%	0.13%	0.12%	0.12%	0.16%	0.13%	0.13%	0.20%	0.18%	0.14%	0.15%	0.13%	0.18%	0.08%	0.00%	0.13%	0.13%
Q	0.08%	0.12%	0.05%	0.16%	0.20%	0.06%	0.06%	0.16%	0.20%	0.20%	0.20%	0.00%	0.07%	0.15%	0.07%	0.00%	0.08%	0.08%	0.13%	0.13%
R	42.19%	41.23%	40.72%	39.66%	41.64%	41.62%	41.19%	40.89%	42.21%	43.13%	41.91%	41.83%	44.44%	41.59%	42.25%	42.67%	43.04%	43.87%	44.27%	45.19%
S	0.08%	0.12%	0.10%	0.16%	0.13%	0.12%	0.17%	0.16%	0.13%	0.00%	0.00%	0.26%	0.21%	0.22%	0.13%	0.27%	0.15%	0.08%	0.13%	0.19%
T	0.00%	0.04%	0.05%	0.05%	0.07%	0.00%	0.00%	0.08%	0.00%	0.00%	0.10%	0.09%	0.07%	0.07%	0.00%	0.00%	0.08%	0.16%	0.00%	0.06%
V	0.08%	0.08%	0.05%	0.11%	0.07%	0.06%	0.06%	0.08%	0.13%	0.13%	0.10%	0.09%	0.07%	0.07%	0.07%	0.09%	0.08%	0.08%	0.06%	0.13%
W	0.00%	0.00%	0.00%	0.00%	0.00%	0.00%	0.00%	0.00%	0.00%	0.00%	0.00%	0.00%	0.00%	0.00%	0.00%	0.00%	0.00%	0.00%	0.00%	0.00%
Y	0.08%	0.04%	0.05%	0.05%	0.00%	0.06%	0.06%	0.00%	0.00%	0.00%	0.00%	0.09%	0.14%	0.00%	0.13%	0.00%	0.08%	0.00%	0.06%	0.06%

These data was based on the peptide identifications from the mouse tryptic peptide samples treated with different NaIO4 concentration.

Table S3| The percentages of peptides containing each amino acid residue at the N-terminus

NaIO$_4$	0mM_1	0mM_2	0mM_3	0mM_4	1mM_1	1mM_2	1mM_3	1mM_4	5mM_1	5mM_2	5mM_3	5mM_4	10mM_1	10mM_2	10mM_3	10mM_4	50mM_1	50mM_2	50mM_3	50mM_4
A	12.31%	12.41%	13.52%	13.95%	14.74%	15.28%	15.36%	16.64%	15.85%	15.91%	15.38%	15.99%	14.95%	15.78%	14.97%	14.49%	15.05%	16.53%	15.70%	14.53%
C	0.34%	0.35%	0.34%	0.32%	0.07%	0.12%	0.12%	0.24%	0.13%	0.07%	0.10%	0.09%	0.07%	0.00%	0.07%	0.00%	0.00%	0.00%	0.00%	0.00%
D	5.05%	5.55%	4.95%	5.60%	6.38%	6.86%	6.92%	6.20%	6.20%	6.57%	7.04%	6.33%	6.47%	5.91%	6.75%	7.73%	5.87%	6.29%	6.44%	6.88%
E	6.66%	7.17%	6.32%	6.73%	7.63%	8.66%	8.20%	8.40%	7.42%	6.97%	7.74%	7.73%	7.86%	8.38%	7.95%	8.09%	7.90%	6.37%	7.72%	7.90%
F	3.99%	3.85%	4.41%	3.56%	4.67%	4.39%	4.36%	4.40%	5.46%	4.60%	5.23%	5.45%	5.35%	4.71%	5.35%	5.33%	5.19%	5.32%	5.02%	5.10%
G	8.79%	8.44%	7.74%	8.46%	10.33%	10.58%	9.95%	9.89%	10.32%	9.86%	10.65%	8.88%	9.60%	10.32%	9.69%	10.31%	9.10%	9.76%	9.52%	9.88%
H	1.27%	1.54%	1.71%	1.08%	1.18%	1.67%	1.51%	1.26%	1.55%	2.04%	1.01%	2.02%	1.74%	2.02%	2.01%	1.60%	2.78%	1.77%	2.32%	2.55%
I	8.32%	7.86%	8.38%	8.08%	9.93%	9.77%	10.01%	9.26%	9.51%	9.93%	10.15%	9.40%	9.67%	10.17%	9.69%	9.78%	10.61%	10.32%	9.97%	9.31%
K	3.18%	3.01%	3.53%	2.32%	3.16%	3.15%	3.43%	2.90%	3.64%	4.60%	3.02%	4.31%	4.66%	3.89%	4.14%	4.27%	5.12%	4.68%	4.50%	4.53%
L	11.38%	11.06%	10.63%	10.40%	14.01%	13.17%	13.21%	13.27%	13.42%	13.41%	13.57%	14.15%	14.39%	14.51%	14.17%	12.80%	13.77%	14.03%	13.19%	13.45%
M	2.04%	1.73%	1.76%	1.67%	0.00%	0.00%	0.06%	0.08%	0.07%	0.07%	0.00%	0.00%	0.00%	0.00%	0.00%	0.09%	0.00%	0.08%	0.06%	0.06%
N	3.78%	3.74%	4.07%	3.66%	5.39%	5.13%	4.94%	4.32%	3.98%	5.00%	4.62%	3.95%	4.66%	4.19%	4.34%	4.71%	4.44%	3.87%	4.63%	4.72%
P	0.55%	0.50%	0.34%	0.48%	0.53%	0.56%	0.41%	0.47%	0.47%	0.46%	0.60%	0.53%	0.42%	0.52%	0.47%	0.62%	0.53%	0.40%	0.45%	0.57%
Q	1.99%	2.04%	1.47%	1.83%	1.97%	2.35%	1.80%	1.88%	2.29%	2.04%	2.31%	1.76%	1.81%	2.09%	1.67%	1.87%	1.88%	1.94%	1.87%	2.17%
R	1.02%	1.31%	1.27%	0.97%	1.25%	1.30%	1.34%	1.18%	1.55%	1.25%	1.21%	1.58%	1.53%	0.90%	1.67%	1.51%	1.58%	1.69%	2.19%	1.91%
S	7.60%	7.36%	7.50%	7.33%	1.32%	1.05%	1.16%	1.33%	1.35%	1.12%	1.01%	1.23%	0.83%	0.75%	0.87%	0.80%	0.75%	0.81%	0.90%	0.96%
T	7.39%	7.86%	7.79%	7.92%	0.46%	0.37%	0.58%	0.39%	0.34%	0.13%	0.20%	0.18%	0.14%	0.15%	0.13%	0.36%	0.15%	0.24%	0.13%	0.19%
V	10.78%	10.83%	10.83%	11.75%	13.49%	12.43%	13.44%	14.21%	13.22%	12.89%	13.47%	13.27%	12.24%	12.64%	12.83%	12.71%	12.72%	13.15%	12.29%	12.62%
W	0.72%	0.77%	0.78%	0.70%	0.46%	0.25%	0.35%	0.55%	0.40%	0.46%	0.30%	0.44%	0.42%	0.22%	0.47%	0.18%	0.15%	0.24%	0.32%	0.06%
Y	2.84%	2.62%	2.65%	3.18%	3.03%	2.91%	2.85%	3.14%	2.83%	2.63%	2.41%	2.72%	3.20%	2.84%	2.74%	2.76%	2.41%	2.50%	2.77%	2.61%

These data was based on the peptide identifications from the mouse tryptic peptide samples treated with different NaIO4 concentration.

Table S4. Total identified glycosites and glycoproteins

Protein	Glycosite	Glycosite sequence window	Localization prob	Score diff	Score	Score for localization	Modified sequence	Deamidation (N) Probabilities	Deamidation (N) Score diffs	Position	Charge	m/z	PNP strategy_1	PNP strategy_2	Conventional HC strategy_1	Conventional HC strategy_2
IPI00119299	385	ISGKSAVFHRIEGITNETYRIGVQMHPGQEI	1	126.485	126.48	126.48	_SAVFHRIEGITN(de)ETYR_	SAVFHRIEGITN(1)ETYR	SAVFHRIEGITN(126.48)ETYR	12	3	632	1	1	1	1
IPI00856723	311	ISGPNIFINNINKTDNGTYRCEASNIVGKAH	0.999989	48.8495	148.89	148.89	_VDDEM(ox)PQHAVISGPNIFINN(de)IN(de)KTDN(de)GTYR_	VDDEMPQHAVISGPNIFIN(0.178)N(0.	VDDEMPQHAVISGPN(-75.02)IFIN(	26	3	1131	1	1	1	1
IPI00131143	604	AAFIIDQREATIVGENETYPRAIIHWNRAAS	1	60.1571	175.15	60.157	_EATIVGEN(de)ETYPR_	EATIVGEN(1)ETYPR	EATIVGEN(60.16)ETYPR	8	2	740	1	1	1	1
IPI00113039	90	NAHNWRISQQRISHYNDTYPISPPQRTPGGI	1	70.197	165.34	70.197	_ISHYN(de)DTYPISPPQR_	ISHYN(1)DTYPISPPQR	ISHYN(70.2)DTYPISPPQR	5	2	895	1	1	1	1
IPI00387318	297	RDDIHPTIPAGQYFINITYNYPVHSFDGRKR	1	56.4677	198.49	56.468	_RDDIHPTIPAGQYFIN(de)ITYN(de)YPVHSFDGR_	RDDIHPTIPAGQYFIN(1)ITYN(1)YPV	RDDIHPTIPAGQYFIN(56.47)ITYN(	16	3	1137	1	1	1	1
IPI00308971	740	DRDAGVGFPEYQEEDNSTYNFRWYTSYACPE	0.99988	39.192	147.17	147.17	_DAGVGFPEYQEEDN(de)STYNFR_	DAGVGFPEYQEEDN(1)STYNFR	DAGVGFPEYQEEDN(39.19)STYN(-3	14	3	780	1	1	1	1
IPI00266264	679	IEQVKEITDTGKNAVNCTYKNEDDCVVRFQY	0.997174	25.564	168.1	168.1	_NAVN(de)CTYKNEDDCVVR_	NAVN(0.997)CTYKN(0.003)EDDCVV	N(-42.43)AVN(25.56)CTYKN(-25	4	2	979	1	1	1	1
IPI00876541	254	PGSIHKANEQVIQNINHTYKMDYNSFQGTPS	0.994056	22.2333	149	149	_ANEQVIQNIN(de)HTYK_	ANEQVIQN(0.006)IN(0.994)HTYK	AN(-101.97)EQVIQN(-22.23)IN(	10	2	837	1	1	1	1
IPI00411025	300	VVSYHTVAWSSGRGFNATYHVRGYCIPWDRP	1	90.6291	117.7	90.629	_GFN(de)ATYHVR_	GFN(1)ATYHVR	GFN(90.63)ATYHVR	3	2	533	1	1	1	1
IPI00876541	538	GAESVIMVKTSTREWNGTYHCIFRYKNSYSI	1	58.6993	93.839	58.699	_EWN(de)GTYHCIFR_	EWN(1)GTYHCIFR	EWN(58.7)GTYHCIFR	3	3	495	1	1	1	1
IPI00128249	176	VHTVNTAIAAFNTQNNGTYFKIVEISRAQNV	0.981244	17.2149	141.48	41.247	_CPIITPFNDTNVVHTVN(de)TAIAAFN(de)TQN(de)N(de)GTYFK_	CPIITPFN(0.389)DTN(0.389)VVHT	CPIITPFN(-3.82)DTN(-3.82)VVH	28	4	922	1	1	1	1
IPI00273133	582	DPIEGQSVRISCRADNYTYEHIRWYRINIST	1	85.8127	114.86	85.813	_ADN(de)YTYEHIR_	ADN(1)YTYEHIR	ADN(85.81)YTYEHIR	3	2	642	1	1	1	1
IPI00858318	71	ISVVNAPTPYIKAFYNGTWYRRHGQPIDPDT	1	91.8667	91.867	91.867	_AFYN(de)GTWYRR_	AFYN(1)GTWYRR	AFYN(91.87)GTWYRR	4	2	668	1	1	1	1
IPI00121378	167	KKIGDCISRDSYPDGNITWYRNGKVIQPVEG	1	63.6623	164.15	63.662	_DSYPDGN(de)ITWYRN(de)GK_	DSYPDGN(1)ITWYRN(1)GK	DSYPDGN(63.66)ITWYRN(63.66)G	7	3	597	1	1	1	1
IPI00122973	409	HVIYGPRIDETDCIGNWTWQEGSQQTIKCQA	1	50.2259	175.52	50.226	_IDETDCIGN(de)WTWQEGSQQTIK_	IDETDCIGN(1)WTWQEGSQQTIK	IDETDCIGN(50.23)WTWQEGSQQTIK	9	3	837	1	1	1	1
IPI00118069	37	IIQVIAGAGIPEKAFNITWISTDFKTIIEWQ	1	100.187	102.97	100.19	_AFN(de)ITWISTDFK_	AFN(1)ITWISTDFK	AFN(100.19)ITWISTDFK	3	2	722	1	1	1	1
IPI00110808	143	NIIICFIDKFSPPVVNVTWIRNGRPVTEGVS	1	137.895	137.89	137.89	_FSPPVVN(de)VTWIR_	FSPPVVN(1)VTWIR	FSPPVVN(137.89)VTWIR	7	3	473	1	1	1	1
IPI00128206	296	PWTIIQHRKDGSQDFNETWENYEKGFGRIDG	1	76.0231	149.52	149.52	_KDGSQDFN(de)ETWENYEK_	KDGSQDFN(1)ETWENYEK	KDGSQDFN(76.02)ETWEN(-76.02)	8	2	996	1	1	1	1
IPI00119299	164	IIKWNDRGSAIPHPSNATWEIKVIQNPRTEP	1	71.2794	141.85	71.279	_GSAIPHPSN(de)ATWEIK_	GSAIPHPSN(1)ATWEIK	GSAIPHPSN(71.28)ATWEIK	9	2	805	1	1	1	1
IPI00314549	65	KIMQDPIQQAEGIYCNRTWDGWICWNDVAAG	1	186.565	186.56	186.56	_IM(ox)QDPIQQAEGIYCN(de)R_	IMQDPIQQAEGIYCN(1)R	IMQDPIQQAEGIYCN(186.56)R	15	3	652	1	1	1	1
IPI00123342	862	QVFTEVEMTTIEKVINDTWAWKNATIAEQAK	1	87.49	283.49	87.49	_VIN(de)DTWAWKN(de)ATIAEQ(de)AK_	VIN(1)DTWAWKN(1)ATIAEQAK	VIN(87.49)DTWAWKN(87.49)ATIA	3	2	1032	1	1	1	1
IPI00126253	19	TIYDYFWWERIWIPVNITWADIEDKDGRVYA	1	75.9683	169.28	75.968	_IWIPVN(de)ITWADIEDKDGR_	IWIPVN(1)ITWADIEDKDGR	IWIPVN(75.97)ITWADIEDKDGR	6	3	715	1	1	1	1
IPI00111163	292	GKFDEVYDAIAGAHPNITVYKKEEIPERWHY	1	154.187	203.89	154.19	_FDEVYDAIAGAHPN(de)ITVYK_	FDEVYDAIAGAHPN(1)ITVYK	FDEVYDAIAGAHPN(154.19)ITVYK	14	2	1063	1	1	1	1
IPI00110264	941	TWDPPVIAERNGHITNYTVVYRDINSQIEIQ	1	73.4663	73.466	73.466	_N(de)GHITN(de)YTVVYR_	N(1)GHITN(1)YTVVYR	N(73.47)GHITN(73.47)YTVVYR	6	2	720	1	1	1	1
IPI00119063	1512	WRTNTIAKANKWTGHNVTVVQRTNTQPFDIQ	1	83.0451	162.48	83.045	_WTGHN(de)VTVVQR_	WTGHN(1)VTVVQR	WTGHN(83.05)VTVVQR	5	2	649	1	1	1	1
IPI00112032	258	NDIKGSTIKISQAIGNITVVQKGEQDIISNG	1	160.524	174.95	160.52	_ISQAIGN(de)ITVVQK_	ISQAIGN(1)ITVVQK	ISQAIGN(160.52)ITVVQK	7	3	458	1	1	1	1
IPI00469218	160	NKAYRCVSDIRVYMKNVTVVIRDATIQAYIS	1	73.8853	147.91	73.885	_VYM(ox)KN(de)VTVVIR_	VYMKN(1)VTVVIR	VYMKN(73.89)VTVVIR	5	3	447	1	1	1	1
IPI00331175	331	ANRNFDTCAKMQVVSNGTVTTAIWRIFCNGS	1	136.299	161.84	136.3	_M(ox)QVVSN(de)GTVTTAIWR_	MQVVSN(1)GTVTTAIWR	MQVVSN(136.3)GTVTTAIWR	6	3	561	1	1	1	1
IPI00123223	313	IQSKKRNYEVQIFHVNATVTEEGTGIEFSRS	1	79.4397	318.66	256.53	_NYEVQIFHVN(de)ATVTEEGTGIEFSR_	NYEVQIFHVN(1)ATVTEEGTGIEFSR	N(-79.44)YEVQIFHVN(79.44)ATV	10	3	914	1	1	1	1
IPI00323134	402	FYGEVPENRVDVIVANITVTDKDQPHTPAWN	1	71.5274	95.624	71.527	_VDVIVAN(de)ITVTDKDQPHTPAWN(de)AAYR_	VDVIVAN(1)ITVTDKDQPHTPAWN(1)A	VDVIVAN(71.53)ITVTDKDQPHTPAW	7	3	966	1	1	1	1
IPI00114256	72	KTEIQVNCPKVGVNKNQTVTATFGYPFRINQ	1	69.3307	216.72	69.331	_VGVN(de)KN(de)QTVTATFGYPFR_	VGVN(1)KN(1)QTVTATFGYPFR	VGVN(69.33)KN(69.33)QTVTATFG	6	3	668	1	1	1	1
IPI00129677	78	SQIREDIIAIRQNFSNITVSTEDQVKAISTQ	1	48.8985	216.71	48.899	_QN(de)FSN(de)ITVSTEDQVK_	QN(1)FSN(1)ITVSTEDQVK	QN(48.9)FSN(48.9)ITVSTEDQVK	5	2	856	1	1	1	1
IPI00226932	917	YSPDVQVHAERFINYNQTVSRMRGIYTAPSG	1	122.691	122.69	122.69	_FIN(de)YN(de)QTVSR_	FIN(1)YN(1)QTVSR	FIN(122.69)YN(122.69)QTVSR	5	2	622	1	1	1	1
IPI00320241	261	VKHRIFERRGDDIYTNVTVSIVEAIVGFEMD	1	38.9248	165.13	38.925	_RGDDIYTN(de)VTVSIVEAIVGFEM(ox)DITHIDGHK_	RGDDIYTN(1)VTVSIVEAIVGFEMDITH	RGDDIYTN(38.92)VTVSIVEAIVGFE	8	4	866	1	1	1	1
IPI00127406	66	ETEGYRRFIQSAEFFNYTVRTIGIGQEWRGG	1	53.001	174.39	53.001	_FIQSAEFFN(de)YTVR_	FIQSAEFFN(1)YTVR	FIQSAEFFN(53)YTVR	9	2	812	1	1	1	1
IPI00319509	106	PYITPNNQGIYIFQGNSTVRFTCNQTTDVII	1	72.5683	222.91	72.568	_VIIRPYITPN(de)N(de)QGIYIFQGN(de)STVR_	VIIRPYITPN(1)N(1)QGIYIFQGN(1)	VIIRPYITPN(72.57)N(72.57)QGI	20	4	692	1	1	1	1
IPI00330632	94	NTATKAIIQGIIPEQNYTVQIIAYYKDKESK	1	76.1546	161.35	76.155	_AIIQGIIPEQN(de)YTVQIIAYYKDK_	AIIQGIIPEQN(1)YTVQIIAYYKDK	AIIQGIIPEQN(76.15)YTVQIIAYYK	11	3	895	1	1	1	1
IPI00134808	227	GFRIVGSPFIGCTVVNKTVPVWSSSPPTCEK	1	62.528	191.79	62.528	_IVGSPFIGCTVVN(de)K_	IVGSPFIGCTVVN(1)K	IVGSPFIGCTVVN(62.53)K	13	3	498	1	1	1	1
IPI00653675	647	SCHGDSGGAFAFQVPNVTVPKFYVAGIVSWG	1	56.0552	84.946	56.055	_GVDSCHGDSGGAFAFQVPN(de)VTVPK_	GVDSCHGDSGGAFAFQVPN(1)VTVPK	GVDSCHGDSGGAFAFQVPN(56.06)VT	19	3	816	1	1	1	1
IPI00409148	256	MIPVADQDKCVVHYENSTVPEKKNITSPVGV	1	103.263	235.73	103.26	_YVM(ox)IPVADQDKCVVHYEN(de)STVPEKK_	YVMIPVADQDKCVVHYEN(1)STVPEKK	YVMIPVADQDKCVVHYEN(103.26)ST	18	3	989	1	1	1	1
IPI00125813	679	YMGIPIPEDNIDHYRNSTVMSRAEHFKQVEY	1	54.3685	106.16	54.368	_YM(ox)GIPIPEDN(de)IDHYRN(de)STVM(ox)SR_	YMGIPIPEDN(1)IDHYRN(1)STVMSR	YMGIPIPEDN(54.37)IDHYRN(54.3	16	3	881	1	1	1	1
IPI00322463	183	DTVVFKCIPHFAMIGNDTVMCTEQGNWTRIP	1	87.5088	136.68	87.509	_CIPHFAM(ox)IGN(de)DTVM(ox)CTEQGN(de)WTRIPECIEVK_	CIPHFAMIGN(1)DTVMCTEQGN(1)WTR	CIPHFAMIGN(87.51)DTVMCTEQGN(	10	4	936	1	1	1	1
IPI00123925	679	FMGIPTKDDNIEHYKNSTVMARAEYFRNVDY	0.999604	34.0229	94.057	94.057	_FM(ox)GIPTKDDNIEHYKN(de)STVM(ox)AR_	FMGIPTKDDNIEHYKN(1)STVMAR	FMGIPTKDDN(-34.02)IEHYKN(34.	16	3	867	1	1	1	1
IPI00658311	153	FHVHQPVTQPFIQVTNTTVKEIDSVTITCIS	1	193.19	199.01	193.19	_FHVHQPVTQPFIQVTN(de)TTVK_	FHVHQPVTQPFIQVTN(1)TTVK	FHVHQPVTQPFIQVTN(193.19)TTVK	16	3	775	1	1	1	1
IPI00108535	333	ITVIEPVTQPFIQVTNTTVKEIDSVTITCIS	1	69.98	192.27	69.98	_TTVKN(de)ITVIEPVTQPFIQVTN(de)TTVK_	TTVKN(1)ITVIEPVTQPFIQVTN(1)TT	TTVKN(69.98)ITVIEPVTQPFIQVTN	21	4	694	1	1	1	1
IPI00134808	275	KATYTHRDSVRIACINGTVIRGRHVIECQGN	1	104.523	180.07	104.52	_IACIN(de)GTVIR_	IACIN(1)GTVIR	IACIN(104.52)GTVIR	5	2	559	1	1	1	1
IPI00317356	324	NIISEDPKITVVYAENGTVIQGTTVASVYKG	1	67.3964	217.86	67.396	_ITVVYAEN(de)GTVIQ(de)GTTVASVYK_	ITVVYAEN(1)GTVIQGTTVASVYK	ITVVYAEN(67.4)GTVIQGTTVASVYK	8	3	772	1	1	1	1
IPI00320618	53	CSHIKITHIPDDIPSNITVINITHNQIRRIP	1	69.5832	153.25	153.25	_ITHIPDDIPSN(de)ITVIN(de)ITHNQIR_	ITHIPDDIPSN(1)ITVIN(0.999)ITH	ITHIPDDIPSN(69.58)ITVIN(29.1	11	3	876	1	1	1	1
IPI00467600	2400	HHITNVNVSFYDDIVNGTVIKTRIGSQIIIT	1	71.1476	181.54	71.148	_DIEHHITN(de)VN(de)VSFYDDIVN(de)GTVIK_	DIEHHITN(1)VN(1)VSFYDDIVN(1)G	DIEHHITN(71.15)VN(71.15)VSFY	19	3	916	1	1	1	1
IPI00405742	1072	VVFISPAVPEEPEAYNITVIIRMDGHCAPIR	1	70.8373	171.98	70.837	_VVFISPAVPEEPEAYN(de)ITVIIR_	VVFISPAVPEEPEAYN(1)ITVIIR	VVFISPAVPEEPEAYN(70.84)ITVII	16	4	615	1	1	1	1
IPI00108535	317	FVNNSVTGISRTTVKNITVIEPVTQPFIQVT	1	69.98	192.27	69.98	_TTVKN(de)ITVIEPVTQPFIQVTN(de)TTVK_	TTVKN(1)ITVIEPVTQPFIQVTN(1)TT	TTVKN(69.98)ITVIEPVTQPFIQVTN	5	4	694	1	1	1	1
IPI00228567	101	KVIVFSEDGYFIRAWNYTVDTPHGMFVSGTP	1	55.3139	127.2	55.314	_AWN(de)YTVDTPHGM(ox)FVSGTPFEQSVWITDVGSGPYGHTVKK_	AWN(1)YTVDTPHGMFVSGTPFEQSVWIT	AWN(55.31)YTVDTPHGMFVSGTPFEQ	3	5	863	1	1	1	1
IPI00121550	266	IQPKYIQPIIAVQFTNITVDTEIRVECKAYG	1	70.4517	288.25	70.452	_YIQPIIAVQFTN(de)ITVDTEIRVECK_	YIQPIIAVQFTN(1)ITVDTEIRVECK	YIQPIIAVQFTN(70.45)ITVDTEIRV	12	3	951	1	1	1	1
IPI00119063	240	PTSTRQTTAMDFSYANETVCWVHVGDSAAQT	1	36.483	48.759	36.483	_QTTAM(ox)DFSYAN(de)ETVCWVHVGDSAAQTQIK_	QTTAMDFSYAN(1)ETVCWVHVGDSAAQT	QTTAMDFSYAN(36.48)ETVCWVHVGD	11	3	1092	1	1	1	1
IPI00128399	79	RFAPPQPAEPWSSVKNATTYPPMCSQDAARG	1	67.3255	124.16	67.326	_N(de)ATTYPPM(ox)CSQDAAR_	N(1)ATTYPPMCSQDAAR	N(67.33)ATTYPPMCSQDAAR	1	2	850	1	1	1	1
IPI00649186	187	CFPAIHASKGVIMVGNETTYEDGHGARKNIT	1	55.8813	245.19	55.881	_GVIM(ox)VGN(de)ETTYEDGHGARKN(de)ITDIVEGAK_	GVIMVGN(1)ETTYEDGHGARKN(1)ITD	GVIMVGN(55.88)ETTYEDGHGARKN(	7	3	1031	1	1	1	1
IPI00225072	169	FMNYSSSVSFKERIGNVTTWISSSNPPVTFA	1	48.1796	52.707	48.18	_IGN(de)VTTWISSSN(de)PPVTFAAIYWEEPDVSGHK_	IGN(1)VTTWISSSN(1)PPVTFAAIYWE	IGN(48.18)VTTWISSSN(48.18)PP	3	3	1136	1	1	1	1
IPI00123342	515	IGFIGPEDIRVFGSQNITTVKIKGVGESFKK	1	81.9197	98.407	81.92	_VFGSQN(de)ITTVKIK_	VFGSQN(1)ITTVKIK	VFGSQN(81.92)ITTVKIK	6	3	479	1	1	1	1
IPI00467600	1750	NIIITPKGASGRVIINITTVAANHGYTKFSK	1	66.0588	218.28	66.059	_VIIN(de)ITTVAAN(de)HGYTK_	VIIN(1)ITTVAAN(1)HGYTK	VIIN(66.06)ITTVAAN(66.06)HGY	4	3	573	1	1	1	1
IPI00108849	320	GAFRKTGVHDGDFEYNITTTIAAINKIRIFK	1	98.0604	138.66	109.04	_TGVHDGDFEYN(de)ITTTIAAINK_	TGVHDGDFEYN(1)ITTTIAAINK	TGVHDGDFEYN(98.06)ITTTIAAIN(	11	3	761	1	1	1	1
IPI00318175	413	IDQDFYVSPTFQDIINRTTTGRPTGWYKDIH	1	95.2051	97.32	95.205	_M(ox)PFPIDQDFYVSPTFQDIIN(de)R_	MPFPIDQDFYVSPTFQDIIN(1)R	MPFPIDQDFYVSPTFQDIIN(95.21)R	20	3	854	1	1	1	1
IPI00322304	134	TRYSNESQDISVNGYNCTTSSVSSAIRNTKD	1	120.33	120.33	120.33	_YSN(de)ESQDISVN(de)GYN(de)CTTSSVSSAIR_	YSN(1)ESQDISVN(1)GYN(1)CTTSSV	YSN(120.33)ESQDISVN(120.33)G	14	3	915	1	1	1	1
IPI00230319	140	NDISSIHNSTITCIVNQTTSITGTSPEIMEK	1	50.4134	115.47	50.413	_YATEN(de)DISSIHN(de)STITCIVN(de)QTTSITGTSPEIM(ox)EK_	YATEN(1)DISSIHN(1)STITCIVN(1)	YATEN(50.41)DISSIHN(50.41)ST	20	4	969	1	1	1	1
IPI00130654	402	RENPAGCYRHVEDKFNETTQRSIAMVQQECK	1	82.2607	176.18	82.261	_HVEDKFN(de)ETTQ(de)R_	HVEDKFN(1)ETTQR	HVEDKFN(82.26)ETTQR	7	3	503	1	1	1	1
IPI00316469	613	VMSWWDYGYQIAGMANRTTIVDNNTWNNSHI	1	121.188	220.21	121.19	_VM(ox)SWWDYGYQIAGM(ox)AN(de)R_	VMSWWDYGYQIAGMAN(1)R	VMSWWDYGYQIAGMAN(121.19)R	16	2	1041	1	1	1	1
IPI00850413	234	YMNVNNYWIINARHINITTISVAGGFRDYQV	1	82.5294	171.26	82.529	_HIN(de)ITTISVAGGFR_	HIN(1)ITTISVAGGFR	HIN(82.53)ITTISVAGGFR	3	2	744	1	1	1	1
IPI00127447	105	TYREIRNKANIQFGENGTTISAVTNKAYVFE	1	77.8819	263.55	162.09	_N(de)KANIQFGEN(de)GTTISAVTNK_	N(0.999)KAN(0.001)IQFGEN(1)GT	N(30.11)KAN(-30.11)IQFGEN(77	10	2	1055	1	1	1	1
IPI00349830	235	RINSAPVEGYSEHVGNKTTIRMTYPEGAPIS	1	79.88	205.68	79.88	_IN(de)SAPVEGYSEHVGN(de)KTTIR_	IN(1)SAPVEGYSEHVGN(1)KTTIR	IN(79.88)SAPVEGYSEHVGN(79.88	15	3	725	1	1	1	1
IPI00130010	694	RFIIKGPNKIQCVDGNWTTIPVCIEEERTCG	1	206.696	206.7	206.7	_IQCVDGN(de)WTTIPVCIEEER_	IQCVDGN(1)WTTIPVCIEEER	IQCVDGN(206.7)WTTIPVCIEEER	7	2	1161	1	1	1	1
IPI00848693	1745	KRINTDQYYIARFSVNQTTIITHEKFNNYIT	1	68.6757	157.86	68.676	_FSVN(de)QTTIITHEK_	FSVN(1)QTTIITHEK	FSVN(68.68)QTTIITHEK	4	3	507	1	1	1	1
IPI00621027	912	YGSQNQQQVAFPITYNVTTIHEAIERATYIN	1	180.697	180.7	180.7	_M(ox)AIIQYGSQN(de)QQQVAFPITYN(de)VTTIHEAIER_	MAIIQYGSQN(1)QQQVAFPITYN(1)VT	MAIIQYGSQN(180.7)QQQVAFPITYN	21	3	1195	1	1	1	1
IPI00124428	398	SIQEIPGSEHIEMIANATTIAYIKRVIIEP_	1	61.7031	165.1	61.703	_VSIQEIPGSEHIEM(ox)IAN(de)ATTIAYIKR_	VSIQEIPGSEHIEMIAN(1)ATTIAYIKR	VSIQEIPGSEHIEMIAN(61.7)ATTIA	17	4	726	1	1	1	1
IPI00114206	144	QCQIWRSRYPHKPEINSTTHPGADIKENFCR	1	163.804	163.8	163.8	_YPHKPEIN(de)STTHPGADIK_	YPHKPEIN(1)STTHPGADIK	YPHKPEIN(163.8)STTHPGADIK	8	3	669	1	1	1	1
IPI00222429	361	QIKVKTKADGSFRIENITTGTYTIHAQKEHI	1	84.7534	171.08	84.753	_IEN(de)ITTGTYTIHAQK_	IEN(1)ITTGTYTIHAQK	IEN(84.75)ITTGTYTIHAQK	3	3	564	1	1	1	1
IPI00315535	74	AFDVKATPNEGFFNQNITTFYYDRIGIYPRF	0.999652	34.5826	164.94	164.94	_ATPNEGFFNQN(de)ITTFYYDR_	ATPNEGFFNQN(1)ITTFYYDR	ATPN(-91.25)EGFFN(-34.58)QN(	11	2	1150	1	1	1	1
IPI00307837	284	TGVIKPGMVVTFAPVNVTTEVKSVEMHHEAI	1	57.1422	158.52	57.142	_VETGVIKPGM(ox)VVTFAPVN(de)VTTEVK_	VETGVIKPGMVVTFAPVN(1)VTTEVK	VETGVIKPGMVVTFAPVN(57.14)VTT	18	3	845	1	1	1	1
IPI00265291	624	CDPWIVPIKDFEKQINITTEDDDIYHMTVPY	1	129.118	129.12	129.12	_QIN(de)ITTEDDDIYHM(ox)TVPYGRPR_	QIN(1)ITTEDDDIYHMTVPYGRPR	QIN(129.12)ITTEDDDIYHMTVPYGR	3	3	884	1	1	1	1
IPI00319509	114	GIYIFQGNSTVRFTCNQTTDVIIIHSKKINY	1	128.694	231.06	128.69	_FTCN(de)Q(de)TTDVIIIHSK_	FTCN(1)QTTDVIIIHSK	FTCN(128.69)QTTDVIIIHSK	4	2	890	1	1	1	1
IPI00124640	276	QDTVCDIIQSKCISKNYTTDIITKIPGYPVK	1	88.1898	95.428	88.19	_CISKN(de)YTTDIITKIPGYPVK_	CISKN(1)YTTDIITKIPGYPVK	CISKN(88.19)YTTDIITKIPGYPVK	5	3	771	1	1	1	1
IPI00129423	113	VHIGPIRGFQWVTGDNHTSYSRWARPNDQTA	1	184.477	184.48	184.48	_GFQWVTGDN(de)HTSYSR_	GFQWVTGDN(1)HTSYSR	GFQWVTGDN(184.48)HTSYSR	9	2	878	1	1	1	1
IPI00387289	79	RFAPPQPAEPWSFVKNTTSYPPMCSQDAVGG	1	142.823	175.28	175.28	_N(de)TTSYPPM(ox)CSQDAVGGQVISEIFTNR_	N(1)TTSYPPMCSQDAVGGQVISEIFTNR	N(142.82)TTSYPPMCSQDAVGGQVIS	1	3	964	1	1	1	1
IPI00331214	321	NPDNHCFCTEKVISNNCTSYGVIDIGKCKEG	1	112.299	161.99	112.3	_VISN(de)N(de)CTSYGVIDIGK_	VISN(1)N(1)CTSYGVIDIGK	VISN(112.3)N(112.3)CTSYGVIDI	5	2	871	1	1	1	1
IPI00116105	217	IPFSPENTREEDFYVNETSTVKVPMMVQSGN	1	65.9054	175.33	175.33	_IPFSPENTREEDFYVN(de)ETSTVK_	IPFSPENTREEDFYVN(1)ETSTVK	IPFSPEN(-65.91)TREEDFYVN(65.	16	3	868	1	1	1	1
IPI00322463	117	YPKNISFACNPGFFINGTSSSKCTEEGKWSP	1	80.8302	211.64	80.83	_YTSFEYPKN(de)ISFACN(de)PGFFIN(de)GTSSSK_	YTSFEYPKN(1)ISFACN(1)PGFFIN(1)	YTSFEYPKN(80.83)ISFACN(80.83	21	3	1023	1	1	1	1
IPI00757771	170	EVIIRESIIPVTIQCNITSSSHTIMYSYWTR	1	45.3954	63.165	45.395	_ESIIPVTIQCN(de)ITSSSHTIM(ox)YSYWTR_	ESIIPVTIQCN(1)ITSSSHTIMYSYWTR	ESIIPVTIQCN(45.4)ITSSSHTIMYS	11	3	1035	1	1	1	1
IPI00108098	131	EMGNTVMDIIRNYTANATSSREEAWDYVQAQ	1	155.941	155.94	155.94	_N(de)YTAN(de)ATSSREEAWDYVQAQVK_	N(1)YTAN(1)ATSSREEAWDYVQAQVK	N(155.94)YTAN(155.94)ATSSREE	5	3	845	1	1	1	1
IPI00344686	56	PVRYFFIQSVDSDGRNFTSSPPGQTQFKVVV	1	146.975	167.51	146.97	_YFFIQSVDSDGRN(de)FTSSPPGQTQFK_	YFFIQSVDSDGRN(1)FTSSPPGQTQFK	YFFIQSVDSDGRN(146.97)FTSSPPG	13	3	952	1	1	1	1
IPI00469218	253	KKDNKTVTRAFNISPNDTSSGSCGINIVTIK	1	101.672	273.51	273.51	_AFNISPN(de)DTSSGSCGIN(de)IVTIK_	AFNISPN(1)DTSSGSCGIN(1)IVTIK	AFN(-101.67)ISPN(101.67)DTSS	7	2	1149	1	1	1	1
IPI00153258	81	GKDEEYWIRASQQISNETSSFGFNIIRKISM	1	131.874	274.43	162.41	_ASQQISN(de)ETSSFGFNIIR_	ASQQISN(1)ETSSFGFNIIR	ASQQISN(131.87)ETSSFGFN(-131	7	3	667	1	1	1	1
IPI00138342	274	NIQAVNEIIATISQCNDTSSAAMVQCIRQKT	1	67.1276	135.76	135.76	_NIQAVNEIIATISQCN(de)DTSSAAM(ox)VQCIR_	NIQAVNEIIATISQCN(1)DTSSAAMVQC	N(-79.28)IQAVN(-67.13)EIIATI	16	4	782	1	1	1	1

IPI00406901	74	SQHRVIFYKDDAMVYNVTSREHTESYVIPQA	1	74.4284	74.769	74.428	_VIFYKDDAM(ox)VYN(de)VTSR_	VIFYKDDAMVYN(1)VTSR	VIFYKDDAMVYN(74.43)VTSR	12	2	969	1	1	1	1
IPI00466652	197	DFINHSSARGVSSSENFTSPVMEFWERRVIG	1	74.7718	188.04	74.772	_GVSSSEN(de)FTSPVM(ox)EFWERR_	GVSSSEN(1)FTSPVMEFWERR	GVSSSEN(74.77)FTSPVMEFWERR	7	3	755	1	1	1	1
IPI00409148	264	KCVVHYENSTVPEKKNITSPVGVQPIINEHT	1	68.2351	123.36	123.36	_KN(de)ITSPVGVQPIINEHTFCAGITK_	KN(1)ITSPVGVQPIINEHTFCAGITK	KN(68.24)ITSPVGVQPIIN(-68.24	2	3	876	1	1	1	1
IPI00132474	212	STTPAKIRNPCTSEQNCTSPFSYKNVISITD	1	96.7715	168.08	152.32	_NPCTSEQN(de)CTSPFSYK_	NPCTSEQN(1)CTSPFSYK	N(-96.77)PCTSEQN(96.77)CTSPF	8	2	961	1	1	1	1
IPI00125182	368	CVWRISVTPGEKIIINFTSMDIYRSRICWYD	1	80.5221	101.89	80.522	_IIIN(de)FTSM(ox)DIYR_	IIIN(1)FTSMDIYR	IIIN(80.52)FTSMDIYR	4	2	752	1	1	1	1
IPI00121776	61	MYYNNNWTRIPSSIENATSISIMNITGTAIC	1	59.046	61.499	59.046	_IPSSIEN(de)ATSISIM(ox)N(de)ITGTAICHISDIPPDGIR_	IPSSIEN(1)ATSISIMN(1)ITGTAICHI	IPSSIEN(59.05)ATSISIMN(59.05	7	4	879	1	1	1	1
IPI00228719	257	SHAGMVKQVAIQTFGNQTSIIPAGGAGYKVI	1	56.7083	356.04	56.708	_QVAIQTFGN(de)QTSIIPAGGAGYK_	QVAIQTFGN(1)QTSIIPAGGAGYK	QVAIQTFGN(56.71)QTSIIPAGGAGY	9	3	741	1	1	1	1
IPI00115793	117	PPFGIFVFDRNTGEINITSIIDREETPYFII	0.999901	40.0535	179.33	179.33	_NTGEIN(de)ITSIIDREETPYFIITGYAIDSR_	NTGEIN(1)ITSIIDREETPYFIITGYAII	N(-40.05)TGEIN(40.05)ITSIIDR	6	3	1102	1	1	1	1
IPI00121190	444	RTKQHGQFSIAVVGINITSIGIRSIKEISDG	1	57.6158	107.03	57.616	_QHGQFSIAVVGIN(de)ITSIGIR_	QHGQFSIAVVGIN(1)ITSIGIR	QHGQFSIAVVGIN(57.62)ITSIGIR	13	3	704	1	1	1	1
IPI00137599	176	EAIKIAYCEERCGNCNITSIEDEDFCKTVTS	1	67.1935	280.7	280.7	_CGNCN(de)ITSIEDEDFCK_	CGNCN(1)ITSIEDEDFCK	CGN(-67.19)CN(67.19)ITSIEDED	5	2	982	1	1	1	1
IPI00410796	392	IIGHYKPDTIAVVIENGTSIDRQIIIIDIST	1	78.3239	88.396	78.324	_KPIIGHYKPDTIAVVIEN(de)GTSIDR_	KPIIGHYKPDTIAVVIEN(1)GTSIDR	KPIIGHYKPDTIAVVIEN(78.32)GTS	18	3	880	1	1	1	1
IPI00876558	524	MASTGTWNPKGPDISNCTSHWVNQIAQKIRS	1	64.9411	182.02	182.02	_GPDISN(de)CTSHWVNQIAQK_	GPDISN(1)CTSHWVNQIAQK	GPDISN(64.94)CTSHWVN(-64.94)	6	3	686	1	1	1	1
IPI00321375	335	IAQVATNPQGITGTGNTTSEMDPSHRKFIKI	1	99.0645	186.96	99.064	_GHIIIAQVATN(de)PQGITGTGN(de)TTSEM(ox)DPSHR_	GHIIIAQVATN(1)PQGITGTGN(1)TTSI	GHIIIAQVATN(99.06)PQGITGTGN(	20	3	1041	1	1	1	1
IPI00122399	577	KCQGDASRICHTHGWNETSEIMPPGAVFSCI	1	55.7342	55.734	55.734	_ICHTHGWN(de)ETSEIM(ox)PPGAVFSCIYR_	ICHTHGWN(1)ETSEIMPPGAVFSCIYR	ICHTHGWN(55.73)ETSEIMPPGAVFS	8	3	994	1	1	1	1
IPI00113528	172	KKIEIGFNGNRIVDVNITSEGKVKIVPNTKI	1	96.8656	154.36	96.866	_IVDVN(de)ITSEGKVK_	IVDVN(1)ITSEGKVK	IVDVN(96.87)ITSEGKVK	5	2	702	1	1	1	1
IPI01026704	1007	GHFPMPEIRIAISFPNITSDGYPVIYPTGWS	1	76.7951	164.31	164.31	_IAISFPN(de)ITSDGYPVIYPTGWSSSDNVNCRPR_	IAISFPN(1)ITSDGYPVIYPTGWSSSDNV	IAISFPN(76.8)ITSDGYPVIYPTGWS	7	3	1196	1	1	1	1
IPI00330632	1389	VDCKEVGQKAINASANITSDGVEVIGRMVRS	1	103.17	207.76	103.17	_AIN(de)ASAN(de)ITSDGVEVIGR_	AIN(1)ASAN(1)ITSDGVEVIGR	AIN(103.17)ASAN(103.17)ITSDG	7	2	895	1	1	1	1
IPI00131881	279	SGIRNISFMVKRIRINTTSDEKDPTNPFRFP	1	107.656	173.71	173.71	_IN(de)TTSDEKDPTNPFR_	IN(1)TTSDEKDPTNPFR	IN(107.66)TTSDEKDPTN(-107.66	2	2	868	1	1	1	1
IPI00124221	124	DIESFIKPYSVEEQKNITSCPDGAPFIQHGP	1	49.2085	97.312	49.209	_IVEDIESFIKPYSVEEQKN(de)ITSCPDGAPFIQHGPDYR_	IVEDIESFIKPYSVEEQKN(1)ITSCPDG/	IVEDIESFIKPYSVEEQKN(49.21)IT	19	5	857	1	1	1	1
IPI00387318	98	TGTEPSSPCNKCISPNVTSCACTINFTIKQS	1	172.496	224.86	172.5	_CISPN(de)VTSCACTIN(de)FTIK_	CISPN(1)VTSCACTIN(1)FTIK	CISPN(172.5)VTSCACTIN(172.5)	5	2	1044	1	1	1	1
IPI00467180	104	VSHTVVIRPIKAGYFNFTSATITYIAQEDGP	1	88.5435	138.88	88.544	_AGYFN(de)FTSATITYIAQEDGPVVIGSTSAPGQGGIIAQR_	AGYFN(1)FTSATITYIAQEDGPVVIGSTS	AGYFN(88.54)FTSATITYIAQEDGPV	5	4	965	1	1	1	1
IPI00119039	153	HKGVKVVMDIPYEIWNETSAEVADIKKQCDV	1	72.1837	135.38	72.184	_VVM(ox)DIPYEIWN(de)ETSAEVADIKK_	VVMDIPYEIWN(1)ETSAEVADIKK	VVMDIPYEIWN(72.18)ETSAEVADIK	11	4	643	1	1	1	1
IPI00131216	276	TRPIAEKIAVISGCKNTTSAAMVHCIRQKTE	1	90.697	117.93	90.697	_N(de)TTSAAM(ox)VHCIR_	N(1)TTSAAMVHCIR	N(90.7)TTSAAMVHCIR	1	2	689	1	1	1	1
IPI00469218	102	ITFGRGYIITINFTKNTTRYSVQHMYFTYNI	1	65.0429	296.22	65.043	_GYIITIN(de)FTKN(de)TTR_	GYIITIN(1)FTKN(1)TTR	GYIITIN(65.04)FTKN(65.04)TTR	11	2	822	1	1	1	1
IPI00321375	105	GVQIIVFPEDGIHGFNFTRTSIYPFIDFMPS	1	88.7296	160.43	88.73	_GVQ(de)IIVFPEDGIHGFN(de)FTR_	GVQIIVFPEDGIHGFN(1)FTR	GVQIIVFPEDGIHGFN(88.73)FTR	16	3	717	1	1	1	1
IPI00113750	290	HWEIDIDIKEIIVHPNYTRSSSDNDIAIIRI	1	83.499	175.15	83.499	_EIIVHPN(de)YTR_	EIIVHPN(1)YTR	EIIVHPN(83.5)YTR	7	3	415	1	1	1	1
IPI00120674	696	DPGRSRIRAFFDETKNNTRRRTQVFGIMQKC	0.995914	23.8688	141.08	138.76	_AFFDETKN(de)NTR_	AFFDETKN(0.996)N(0.004)TR	AFFDETKN(23.87)N(-23.87)TR	8	2	672	1	1	1	1
IPI00120245	704	NNEAIARISCAFKTENQTRQVVCDIGNPMKA	1	134.562	134.56	134.56	_ISCAFKTEN(de)QTR_	ISCAFKTEN(1)QTR	ISCAFKTEN(134.56)QTR	9	2	728	1	1	1	1
IPI00154056	167	IKFPIGPCPRYEQIQNETRQTPEYQNRSIQN	1	106.291	106.29	106.29	_YEQIQN(de)ETR_	YEQIQN(1)ETR	YEQIQN(106.29)ETR	6	2	591	1	1	1	1
IPI00133500	44	NVIFPPHTTPKAEISNHTRPVIIVPGCIGNR	1	88.3601	133.45	119.47	_AEISN(de)HTRPVIIVPGCIGNR_	AEISN(1)HTRPVIIVPGCIGNR	AEISN(88.36)HTRPVIIVPGCIGN(-	5	3	735	1	1	1	1
IPI00319509	573	TGEISQKHFIIDSKSNVTRPSEFNYIWIAPI	1	109.717	137.14	109.72	_SN(de)VTRPSEFN(de)YIWIAPIPFIK_	SN(1)VTRPSEFN(1)YIWIAPIPFIK	SN(109.72)VTRPSEFN(109.72)YI	2	2	1248	1	1	1	1
IPI00108535	206	DRIKISEGNRTITIINVTRNDTGPYVCETRN	1	111.064	238.19	111.06	_TITIIN(de)VTRN(de)DTGPYVCETR_	TITIIN(1)VTRN(1)DTGPYVCETR	TITIIN(111.06)VTRN(111.06)DT	6	3	776	1	1	1	1
IPI00153468	117	FTSHTPGDHQICIHSNSTRMAIFAGGKIRVH	1	68.4105	197.67	68.41	_FTFTSHTPGDHQ(de)ICIHSN(de)STR_	FTFTSHTPGDHQICIHSN(1)STR	FTFTSHTPGDHQICIHSN(68.41)STR	18	3	816	1	1	1	1
IPI00404551	261	GGTDSKYYHGEISYINVTRKAYWQVHMDQIE	1	110.306	203.7	110.31	_YYHGEISYIN(de)VTRK_	YYHGEISYIN(1)VTRK	YYHGEISYIN(110.31)VTRK	10	3	582	1	1	1	1
IPI00130573	521	PDMEIFIRRFANEYPNITRIYSIGKSVESRE	0.999999	60.3787	134.99	134.99	_RFANEYPN(de)ITR_	RFANEYPN(1)ITR	RFAN(-60.38)EYPN(60.38)ITR	8	2	691	1	1	1	1
IPI00322463	193	FAMIGNDTVMCTEQGNWTRIPECIEVKCPFP	1	87.5088	136.68	87.509	_CIPHFAM(ox)IGN(de)DTVM(ox)CTEQGN(de)WTRIPECIEVK_	CIPHFAMIGN(1)DTVMCTEQGN(1)WTRI	CIPHFAMIGN(87.51)DTVMCTEQGN(	20	4	936	1	1	1	1
IPI00749655	700	FCIIDVMSTGSSSVGNATRIAHQIHQHRIKS	1	90.9116	90.912	90.912	_ICEGDRFCIIDVM(ox)STGSSSVGN(de)ATR_	ICEGDRFCIIDVMSTGSSSVGN(1)ATR	ICEGDRFCIIDVMSTGSSSVGN(90.91	22	3	917	1	1	1	1
IPI00132189	117	NTQADVINASWSVISNSTRHEIERSFDCCGI	1	113.21	263.55	141.6	_NTQADVIN(de)ASWSVISN(de)STRHEIER_	NTQADVIN(1)ASWSVISN(1)STRHEIEI	N(-104.43)TQADVIN(104.43)ASW	16	3	910	1	1	1	1
IPI00125813	514	QDVQMPSKKIDFIVINETRFWYQMIIPPHFD	1	86.0142	129.37	86.014	_KIDFIVIN(de)ETR_	KIDFIVIN(1)ETR	KIDFIVIN(86.01)ETR	8	3	450	1	1	1	1
IPI00134585	637	YEGGTWDWIAEAISSNHTRFSAADRSSFIDD	1	85.0562	153.65	123.43	_VNYEGGTWDWIAEAISSN(de)HTR_	VNYEGGTWDWIAEAISSN(1)HTR	VN(-85.06)YEGGTWDWIAEAISSN(8	18	3	803	1	1	1	1
IPI01026704	531	GTEKEEQGKVYVYAVNQTRFEYQMSIEPIKQ	1	80.1016	94.692	80.102	_VYVYAVN(de)QTR_	VYVYAVN(1)QTR	VYVYAVN(80.1)QTR	7	2	607	1	1	1	1
IPI00307966	104	IISTFKGAFVSKNPCNITREDYAPIVKIVTQ	0.998733	28.9665	174.87	139.48	_GAFVSKNPCN(de)ITR_	GAFVSKN(0.001)PCN(0.999)ITR	GAFVSKN(-28.97)PCN(28.97)ITR	10	2	733	1	1	1	1
IPI00123040	415	IQARRIAVDWESIGYNITRCHTFNVTICYHY	1	151.994	170.66	151.99	_RIAVDWESIGYN(de)ITR_	RIAVDWESIGYN(1)ITR	RIAVDWESIGYN(151.99)ITR	12	3	599	1	1	1	1
IPI00129250	318	ICRWIVANRNKMFSQNDTRCAGPEAMKGQRI	1	87.3076	138.4	87.308	_M(ox)FSQN(de)DTR_	MFSQN(1)DTR	MFSQN(87.31)DTR	5	2	508	1	1	1	1
IPI00123342	922	NKAKFTKPRPRPKDKNGTRAEPPINASAGDQ	1	112.549	112.55	112.55	_DKN(de)GTRAEPPIN(de)ASAGDQEEK_	DKN(1)GTRAEPPIN(1)ASAGDQEEK	DKN(112.55)GTRAEPPIN(112.55)	3	2	1115	1	1	1	1
IPI00125813	144	YDVNKRQIITEEKIPNNTQWITWSPEGHKIA	1	76.0266	257.73	76.027	_QIITEEKIPN(de)N(de)TQWITWSPEGHK_	QIITEEKIPN(1)N(1)TQWITWSPEGHK	QIITEEKIPN(76.03)N(76.03)TQW	10	3	918	1	1	1	1
IPI00469426	379	ANGTEGQPRNKVFKTNSTQVSDVRAMNISAS	1	150.111	150.11	150.11	_VFKTN(de)STQVSDVR_	VFKTN(1)STQVSDVR	VFKTN(150.11)STQVSDVR	5	2	741	1	1	1	1
IPI00116744	300	ISHIVDPKDIEPRAANCTQVIVWHTRTEKPK	1	117.916	148.57	117.92	_AAN(de)CTQVIVWHTR_	AAN(1)CTQVIVWHTR	AAN(117.92)CTQVIVWHTR	3	3	520	1	1	1	1
IPI00406302	101	SKGDTHTQIIEGIQFNITQTSEADIHKSFQH	1	40.81	193.42	40.81	_GDTHTQIIEGIQFN(de)ITQTSEADIHK_	GDTHTQIIEGIQFN(1)ITQTSEADIHK	GDTHTQIIEGIQFN(40.81)ITQTSEA	14	4	700	1	1	1	1
IPI00625835	135	SPTSNISSNSFICIENRTQTIKPTQDPTECV	1	135.211	184.68	135.21	_YKYETTISPTSN(de)ISSN(de)SFICIEN(de)R_	YKYETTISPTSN(1)ISSN(1)SFICIEN(	YKYETTISPTSN(135.21)ISSN(135	23	3	943	1	1	1	1
IPI00110910	46	ERHFVVQFKGECYYTNGTQRIRIVTRYIYNR	1	107.166	197.07	107.17	_GECYYTN(de)GTQR_	GECYYTN(1)GTQR	GECYYTN(107.17)GTQR	7	2	675	1	1	1	1
IPI00123223	993	NMVIFAPNIYVIKYINETQQITQKIKTKAIG	1	110.51	162.68	110.51	_YIN(de)ETQQITQKIK_	YIN(1)ETQQITQKIK	YIN(110.51)ETQQITQKIK	3	2	804	1	1	1	1
IPI00621027	155	IRSFRRGTFTDCAIANMTQQIRQHVGKGVVN	1	123.023	279.44	123.02	_RGTFTDCAIAN(de)M(ox)TQQIR_	RGTFTDCAIAN(1)MTQQIR	RGTFTDCAIAN(123.02)MTQQIR	11	3	667	1	1	1	1
IPI00123704	238	IDIMYFPYYGKKFHVNYTQPIVAVKFINVTP	1	80.2447	118.24	80.245	_FHVN(de)YTQPIVAVK_	FHVN(1)YTQPIVAVK	FHVN(80.24)YTQPIVAVK	4	3	506	1	1	1	1
IPI00987886	409	GICIGQVPQDKGHICNQTQNIQSSKSGQYIM	0.999995	52.5913	115.12	115.12	_GHICN(de)QTQNIQSSK_	GHICN(1)QTQNIQSSK	GHICN(52.59)QTQN(-52.59)IQSS	5	3	539	1	1	1	1
IPI00894972	444	GQVIIFQAPEAGGRWNQTQKIEGTQIGSYFG	1	93.6838	93.684	93.684	_YQHVGQVIIFQAPEAGGRWN(de)QTQK_	YQHVGQVIIFQAPEAGGRWN(1)QTQK	YQHVGQVIIFQAPEAGGRWN(93.68)Q	20	4	690	1	1	1	1
IPI00310090	201	RFYEEFPINRKTGEANITQIYTQEAIDFIQT	1	254.399	254.4	254.4	_TGEAN(de)ITQIYTQEAIDFIQTQHAR_	TGEAN(1)ITQIYTQEAIDFIQTQHAR	TGEAN(254.4)ITQIYTQEAIDFIQTQ	5	3	917	1	1	1	1
IPI00310567	254	IIAHEIHVIEKQPNMNITQIKVIQIGTIVDN	1	54.5594	142.23	54.559	_YVYVADIIAHEIHVIEKQPN(de)M(ox)N(de)ITQIK_	YVYVADIIAHEIHVIEKQPN(1)MN(1)I1	YVYVADIIAHEIHVIEKQPN(54.56)M	22	4	800	1	1	1	1
IPI00474580	46	RPRFIEYVTSECHFYNGTQHVRFIERFIYNR	1	111.659	115.8	111.66	_FIEYVTSECHFYN(de)GTQHVR_	FIEYVTSECHFYN(1)GTQHVR	FIEYVTSECHFYN(111.66)GTQHVR	13	2	1195	1	1	1	1
IPI00129526	217	AFIVADKVIVTSKHNNDTQHIWESDSNEFSV	1	62.6482	200.99	62.648	_HN(de)N(de)DTQHIWESDSN(de)EFSVIADPR_	HN(1)N(1)DTQHIWESDSN(1)EFSVIAI	HN(62.65)N(62.65)DTQHIWESDSN	3	3	905	1	1	1	1
IPI00312711	874	IISIDDPQRGIMIIINDTQHFSNNVKGEIGQ	1	71.2794	184.02	71.279	_GIM(ox)IIIN(de)DTQHFSN(de)N(de)VK_	GIMIIIN(1)DTQHFSN(1)N(1)VK	GIMIIIN(71.28)DTQHFSN(71.28)	7	3	655	1	1	1	1
IPI00121240	187	IPPIDIVREFKASVVNVTQHCTMESWESMNE	1	46.9529	143.04	46.953	_ASVVN(de)VTQHCTM(ox)ESWESM(ox)N(de)EVAR_	ASVVN(1)VTQHCTMESWESMN(1)EVAR	ASVVN(46.95)VTQHCTMESWESMN(4	5	3	900	1	1	1	1
IPI00131168	510	SIMKTWANFAKYGHPNGTQGNSTMWPVFTST	1	108.18	108.18	108.18	_YGHPN(de)GTQGN(de)STM(ox)WPVFTSTEQK_	YGHPN(1)GTQGN(1)STMWPVFTSTEQK	YGHPN(108.18)GTQGN(108.18)ST	5	3	862	1	1	1	1
IPI00135635	184	QQPTEATKIINDYVSNQTQGMIKKIISEIDD	1	107.15	211.7	211.7	_IINDYVSN(de)QTQGM(ox)IK_	IINDYVSN(1)QTQGMIK	IIN(-107.15)DYVSN(107.15)QTQ	8	2	871	1	1	1	1
IPI00131830	185	QQPTEAKNIINDYVSNQTQGMIKEIISEIDE	1	124.458	250.52	124.46	_N(de)IIN(de)DYVSN(de)QTQGM(ox)IK_	N(1)IIN(1)DYVSN(1)QTQGMIK	N(124.46)IIN(124.46)DYVSN(12	9	2	929	1	1	1	1
IPI00116105	169	KDWTKAGEQINNHVKNKTQGKIEHVVSDIDS	1	84.5663	118.21	84.566	_AGEQIN(de)N(de)HVKN(de)KTQGK_	AGEQIN(1)N(1)HVKN(1)KTQGK	AGEQIN(84.57)N(84.57)HVKN(84	11	3	590	1	1	1	1
IPI00169617	306	RVAIARAAGVIEDVYNRTQGIIAGHGIIQIA	1	99.0592	193.85	99.059	_AAGVIEDVYN(de)RTQGIIAGHGIIQIA_	AAGVIEDVYN(1)RTQGIIAGHGIIQIA	AAGVIEDVYN(99.06)RTQGIIAGHGI	10	3	861	1	1	1	1
IPI00119809	69	NIIDAHVVCRAIGYENATQAIGRAAFGPGKG	1	143.939	188.13	143.94	_AIGYEN(de)ATQ(de)AIGR_	AIGYEN(1)ATQAIGR	AIGYEN(143.94)ATQAIGR	6	2	683	1	1	1	1
IPI00406901	540	FYKEKEDRPFHQAVVNDTQAFWHNKQASKKQ	1	113.504	194.05	194.05	_EDRPFHQAVVN(de)DTQAFWHNK_	EDRPFHQAVVN(1)DTQAFWHNK	EDRPFHQAVVN(113.5)DTQAFWHN(-	11	3	814	1	1	1	1
IPI00222967	156	VPIDVIFKCNSVITYNITPVVQKYWGIHIQA	1	181.506	181.51	181.51	_CN(de)SVITYN(de)ITPVVQK_	CN(1)SVITYN(1)ITPVVQK	CN(181.51)SVITYN(181.51)ITPV	8	2	869	1	1	1	1
IPI00224073	253	ISVTEKGAIDIMIQVNMTPGHSSAPPKETSI	1	50.3923	162.77	50.392	_GAIDIM(ox)IQ(de)VN(de)M(ox)TPGHSSAPPK_	GAIDIMIQVN(1)MTPGHSSAPPK	GAIDIMIQVN(50.39)MTPGHSSAPPK	10	3	733	1	1	1	1
IPI00129158	110	DATKRNNMDFSIRISNVTPEDAGTYYCVKFQ	1	127.287	127.29	127.29	_ISN(de)VTPEDAGTYYCVK_	ISN(1)VTPEDAGTYYCVK	ISN(127.29)VTPEDAGTYYCVK	3	2	909	1	1	1	1
IPI00172039	105	IAAEKHNIGIITKRSNFTPATNEAPQATVFP	0.99999	50.0195	151.41	98.391	_RSN(de)FTPATNEAPQATVFPK_	RSN(1)FTPATNEAPQATVFPK	RSN(50.02)FTPATN(-50.02)EAPQ	3	2	1039	1	1	1	1
IPI00132600	186	EKAIGIICGRDARACNATNWIEYMFNKDNGQ	0.999983	47.6308	112.72	112.72	_ACN(de)ATNWIEYM(ox)FNK_	ACN(1)ATNWIEYMFNK	ACN(47.63)ATN(-47.63)WIEYMFN	3	2	890	1	1	1	1
IPI00128249	156	DVRKICPRCPIITPFNDTNVVHTVNTAIAAF	0.821578	6.71972	161.49	161.49	_CPIITPFN(de)DTNVVHTVNTAIAAFNTQNN(de)GTYFK_	CPIITPFN(0.822)DTN(0.175)VVHT\	CPIITPFN(6.72)DTN(-6.72)VVHT	8	3	1229	1	1	1	1
IPI00985989	295	SYGTMDDIKRITKIKNTTNQIAIIKIGKIPI	1	86.2877	121.02	86.288	_IKN(de)TTN(de)QIAIIK_	IKN(1)TTN(1)QIAIIK	IKN(86.29)TTN(86.29)QIAIIK	3	2	680	1	1	1	1
IPI00112614	489	VQSPNGSVYTWREAFNETNQAIQTISRFMEC	1	195.268	252.82	195.27	_EAFN(de)ETN(de)QAIQTISR_	EAFN(1)ETN(1)QAIQTISR	EAFN(195.27)ETN(195.27)QAIQT	4	2	862	1	1	1	1
IPI00466371	779	DKHDFQDSVRVTIDFNITNPENGPVIDDAIP	1	98.4909	98.491	98.491	_VTIDFN(de)ITN(de)PEN(de)GPVIDDAIPN(de)SVHGHIPFAK_	VTIDFN(1)ITN(1)PEN(1)GPVIDDAII	VTIDFN(98.49)ITN(98.49)PEN(9	6	4	862	1	1	1	1
IPI00319509	734	RYIKKQVTPIFFYFQNRTNNWVNRPPTIMEQ	1	121.076	152.85	121.08	_QVTPIFFYFQN(de)R_	QVTPIFFYFQN(1)R	QVTPIFFYFQN(121.08)R	11	3	521	1	1	1	1
IPI00121190	352	CNGIGIGEFKDTISINATNIKHFKYCTAISG	1	125.542	155.48	125.54	_DTISIN(de)ATN(de)IK_	DTISIN(1)ATN(1)IK	DTISIN(125.54)ATN(125.54)IK	6	2	596	1	1	1	1
IPI00330680	184	QVQFSKTVIGKARINNITNIGPIDMKQIDYT	0.99996	44.0098	128.76	128.76	_IN(de)N(de)ITNIGPIDM(ox)K_	IN(1)N(1)ITNIGPIDMK	IN(59.51)N(44.01)ITN(-44.01)	3	2	731	1	1	1	1
IPI00224073	443	VPGICIANTDTRHYANITNGMYRFNPIPINP	1	82.1708	116.03	82.171	_HYAN(de)ITN(de)GM(ox)YR_	HYAN(1)ITN(1)GMYR	HYAN(82.17)ITN(82.17)GMYR	4	2	679	1	1	1	1
IPI00110264	957	YTVVYRDINSQIEIQNVTNDTHITIIGIKPD	1	95.283	162.71	129.32	_DINSQIEIQN(de)VTN(de)DTHITIIGIKPDTTYDIK_	DINSQIEIQN(1)VTN(1)DTHITIIGIKI	DIN(-95.28)SQIEIQN(95.28)VTN	10	3	1172	1	1	1	1
IPI00165730	93	IRIEDGFHPDAVAWANITNAIRETGWAYIDI	1	105.06	169.61	105.06	_IEDGFHPDAVAWAN(de)ITN(de)AIR_	IEDGFHPDAVAWAN(1)ITN(1)AIR	IEDGFHPDAVAWAN(105.06)ITN(10	14	3	738	1	1	1	1
IPI00788443	477	TDIEAIKPKRKEFQHNSTMYYKCRDKQEYEH	1	76.6551	98.105	76.655	_EFQ(de)HN(de)STM(ox)YYK_	EFQHN(1)STMYYK	EFQHN(76.66)STMYYK	5	2	733	1	1	1	1
IPI00131168	515	WANFAKYGHPNGTQGNSTMWPVFTSTEQKYI	1	108.18	108.18	108.18	_YGHPN(de)GTQGN(de)STM(ox)WPVFTSTEQK_	YGHPN(1)GTQGN(1)STMWPVFTSTEQK	YGHPN(108.18)GTQGN(108.18)ST	10	3	862	1	1	1	1
IPI00970438	122	SVVFDVQIPKGAFISNFTMTVNGMTFTSSIK	1	58.1235	169.51	58.123	_GAFISN(de)FTM(ox)TVN(de)GM(ox)TFTSSIK_	GAFISN(1)FTMTVN(1)GMTFTSSIK	GAFISN(58.12)FTMTVN(58.12)GM	6	3	763	1	1	1	1
IPI00279079	384	AGNAIMDGASQIVGENRTMTIHNGMFFSTYD	1	99.2762	308.02	180.62	_YKGTAGNAIM(ox)DGASQIVGEN(de)R_	YKGTAGNAIMDGASQIVGEN(1)R	YKGTAGN(-99.28)AIMDGASQIVGEN	20	3	724	1	1	1	1
IPI00108041	171	KIQITGHAMPRIAVTNTTMTGTVIKMTDRSH	1	143.055	186.05	143.05	_IAVTN(de)TTM(ox)TGTVIK_	IAVTN(1)TTMTGTVIK	IAVTN(143.05)TTMTGTVIK	5	2	734	1	1	1	1
IPI00406603	296	KVSTVTIVSATSTTANMTMSPEGRWIVSSII	1	80.7525	80.752	80.752	_VSTVTIVSATSTTAN(de)M(ox)TM(ox)SPEGR_	VSTVTIVSATSTTAN(1)MTMSPEGR	VSTVTIVSATSTTAN(80.75)MTMSPE	15	3	792	1	1	1	1
IPI00331440	246	TGTSIRCQAIDFFPQNITMRWIKDNQPIDAK	1	134.685	134.68	134.68	_CQAIDFFPQN(de)ITM(ox)R_	CQAIDFFPQN(1)ITMR	CQAIDFFPQN(134.68)ITMR	10	3	587	1	1	1	1
IPI00134808	428	CEQEASEDIKPAITGNKTMQYVPNSHDVKMA	1	168.083	168.08	168.08	_CEQEASEDIKPAITGN(de)K_	CEQEASEDIKPAITGN(1)K	CEQEASEDIKPAITGN(168.08)K	16	2	946	1	1	1	1

IPI00515360	554	QIRISFDQPNDFKGVNVTMPSQPGVPPISST	1	73.2267	106.44	73.227	_GVN(de)VTM(ox)PSQPGVPPISSTQIQIDPAIQEFQIVDISRR_	GVN(1)VTMPSQPGVPPISSTQIQIDPAI(	GVN(73.23)VTMPSQPGVPPISSTQIQ	3	3	1346	1	1	1	1
IPI00320204	100	SGVRDGTSQPAICPQNVTMNMEGIKEIKITI	1	103.881	142.43	103.88	_DGTSQPAICPQN(de)VTM(ox)N(de)M(ox)EGIK_	DGTSQPAICPQN(1)VTMN(1)MEGIK	DGTSQPAICPQN(103.88)VTMN(103	12	3	776	1	1	1	1
IPI00666034	3747	GVKIYKKISTSPFAINITMIPKVKFPGIDII	1	141.341	141.34	141.34	_KISTSPFAIN(de)ITM(ox)IPK_	KISTSPFAIN(1)ITMIPK	KISTSPFAIN(141.34)ITMIPK	10	3	593	1	1	1	1
IPI00128484	64	HCIDTWSFDAATMDHNGTMIFFKGEFVWRGH	1	63.8476	63.848	63.848	_VAEVEN(de)GTKPDSDVPEHCIDTWSFDAATM(ox)DHN(de)GTM(ox)IFFK_	VAEVEN(1)GTKPDSDVPEHCIDTWSFDA/	VAEVEN(63.85)GTKPDSDVPEHCIDT	32	4	1112	1	1	1	1
IPI00130010	791	TGIEAIKPKITEFTHNSTMDYKCRDKQEYER	1	75.0877	119.96	75.088	_ITEFTHN(de)STM(ox)DYK_	ITEFTHN(1)STMDYK	ITEFTHN(75.09)STMDYK	7	3	535	1	1	1	1
IPI00989096	169	IQGGRKAWGISVINPNKTKVQGGCDGTHPHI	0.997221	25.5494	162.48	162.48	_AWGISVINPN(de)KTK_	AWGISVIN(0.003)PN(0.997)KTK	AWGISVIN(-25.55)PN(25.55)KTK	10	2	715	1	1	1	1
IPI00123194	312	IPDIKIIQVVYIHSNNITKVGINDFCPMGFG	1	92.2652	240.46	92.265	_IIQVVYIHSN(de)N(de)ITK_	IIQVVYIHSN(1)N(1)ITK	IIQVVYIHSN(92.27)N(92.27)ITK	11	2	822	1	1	1	1
IPI00126050	394	ARAIMKEVMNIIQPINVTKVFSNGEGTDINF	1	103.417	198.85	198.85	_EVM(ox)NIIQPIN(de)VTK_	EVMNIIQPIN(1)VTK	EVMN(-103.42)IIQPIN(103.42)V	10	2	758	1	1	1	1
IPI00132474	669	KGEKKDTCAQECSHFNITKVESREKIPQPVQ	1	75.682	218.21	75.682	_KDTCAQECSHFN(de)ITKVESR_	KDTCAQECSHFN(1)ITKVESR	KDTCAQECSHFN(75.68)ITKVESR	12	3	771	1	1	1	1
IPI00112614	1637	ENPSQYGITAFNHPINITKQQISEVAIMTTS	0.993327	21.7274	144.17	144.17	_GENPSQYGITAFNHPIN(de)ITK_	GENPSQYGITAFN(0.007)HPIN(0.99	GEN(-121.63)PSQYGITAFN(-21.7	17	2	1102	1	1	1	1
IPI00128484	38	ASPIPTANGRVAEVENGTKPDSDVPEHCIDT	1	63.8476	63.848	63.848	_VAEVEN(de)GTKPDSDVPEHCIDTWSFDAATM(ox)DHN(de)GTM(ox)IFFK_	VAEVEN(1)GTKPDSDVPEHCIDTWSFDA/	VAEVEN(63.85)GTKPDSDVPEHCIDT	6	4	1112	1	1	1	1
IPI00469218	98	PSITITFGRGYIITINFTKNTTRYSVQHMYF	1	65.0429	296.22	65.043	_GYIITIN(de)FTKN(de)TTR_	GYIITIN(1)FTKN(1)TTR	GYIITIN(65.04)FTKN(65.04)TTR	7	2	822	1	1	1	1
IPI00930882	298	VGKIMNAPIYIAEWQNITKNISEDRIIIAGT	1	87.8878	164.65	164.65	_IM(ox)NAPIYIAEWQN(de)ITK_	IMNAPIYIAEWQN(1)ITK	IMN(-87.89)APIYIAEWQN(87.89)	13	3	641	1	1	1	1
IPI00658632	192	GFTKAIMDICDQIEKNQTKMATYIAEIIIIN	1	92.9251	109.35	92.925	_AIM(ox)DICDQIEKN(de)QTK_	AIMDICDQIEKN(1)QTK	AIMDICDQIEKN(92.93)QTK	12	3	609	1	1	1	1
IPI00115065	323	IKAIGITEMFEPSKANFTKITRSESIHVSHI	1	70.8885	119.68	70.889	_AIGITEM(ox)FEPSKAN(de)FTK_	AIGITEMFEPSKAN(1)FTK	AIGITEMFEPSKAN(70.89)FTK	14	3	634	1	1	1	1
IPI00463764	52	IVISRVSQVIHEGGHNVTKIIYESANIPDFR	1	98.4392	220.59	98.439	_VSQ(de)VIHEGGHN(de)VTK_	VSQVIHEGGHN(1)VTK	VSQVIHEGGHN(98.44)VTK	11	2	754	1	1	1	1
IPI00170098	451	RYQEGDITIYVINIHNVTKHIKVPPPIFRKP	0.999655	34.6193	101.49	101.49	_YQEGDITIYVINIHN(de)VTK_	YQEGDITIYVINIHN(1)VTK	YQEGDITIYVIN(-34.62)IHN(34.6	15	2	1061	1	1	1	1
IPI00119063	3840	DINECIRFGTCSQICNNTKGGHICSCARNFM	1	133.148	186.16	133.15	_FGTCSQICN(de)N(de)TK_	FGTCSQICN(1)N(1)TK	FGTCSQICN(133.15)N(133.15)TK	9	2	716	1	1	1	1
IPI00473680	125	FTSHTPGEHQICIHSNSTKFSIFAGGMIRVH	1	80.5855	222.3	80.585	_FTFTSHTPGEHQ(de)ICIHSN(de)STK_	FTFTSHTPGEHQICIHSN(1)STK	FTFTSHTPGEHQICIHSN(80.59)STK	18	3	811	1	1	1	1
IPI00345112	121	KQIPFDTTNNRKIRVNGTKEPIEFKSNQWFG	1	86.1144	96.64	86.114	_VN(de)GTKEPIEFK_	VN(1)GTKEPIEFK	VN(86.11)GTKEPIEFK	2	2	632	1	1	1	1
IPI00187353	122	VGIKAGRSNKYSQAANSTKEIDDCEQANKIG	1	73.8185	184.83	184.83	_YSQAAN(de)STKEIDDCEQANK_	YSQAAN(1)STKEIDDCEQANK	YSQAAN(73.82)STKEIDDCEQAN(-7	6	2	1087	1	1	1	1
IPI00652358	280	RIYAGMVSIMDEAVGNVTKAIKSHGIWNNTV	1	48.2163	145.91	48.216	_IYAGM(ox)VSIM(ox)DEAVGN(de)VTK_	IYAGMVSIMDEAVGN(1)VTK	IYAGMVSIMDEAVGN(48.22)VTK	15	3	644	1	1	1	1
IPI00458077	706	WETRFYWHKGIIGISNATIYWHIPDTAYPGI	1	99.8599	99.86	99.86	_GIIGISN(de)ATIYWHIPDTAYPGIYR_	GIIGISN(1)ATIYWHIPDTAYPGIYR	GIIGISN(99.86)ATIYWHIPDTAYPG	7	3	898	1	1	1	1
IPI00410951	47	NSSEGKVTTCHIPQQNATIYKMPSINADFAF	1	100.542	108.63	100.54	_VTTCHIPQQN(de)ATIYK_	VTTCHIPQQN(1)ATIYK	VTTCHIPQQN(100.54)ATIYK	10	3	592	1	1	1	1
IPI00125220	186	QCGTCTEFKECHTIQNYTIWRVGDYGSISGR	1	101.929	247.88	101.93	_ECHTIQ(de)N(de)YTIWR_	ECHTIQN(1)YTIWR	ECHTIQN(101.93)YTIWR	7	3	542	1	1	1	1
IPI00130010	1079	PVCKDNSCVDPPHVPNATIVTRTKNKYIHGD	1	84.7855	135.18	135.18	_DNSCVDPPHVPN(de)ATIVTR_	DNSCVDPPHVPN(1)ATIVTR	DN(-84.79)SCVDPPHVPN(84.79)A	12	2	997	1	1	1	1
IPI00129304	386	CTDTITRHTDDITSINNTIVNIRIDSISIRM	0.99998	46.9721	256.98	197.19	_HTDDITSIN(de)NTIVNIR_	HTDDITSIN(1)NTIVNIR	HTDDITSIN(46.97)N(-46.97)TIV	9	3	610	1	1	1	1
IPI00319509	817	FGGEEEWNFAWEQFRNATIVNEADKIRSAIA	1	111.793	133.15	111.79	_N(de)ATIVN(de)EADKIR_	N(1)ATIVN(1)EADKIR	N(111.79)ATIVN(111.79)EADKIR	1	2	673	1	1	1	1
IPI00321375	189	QDGRYQFNTNVVFSDNGTIVDRYRKHNIYFE	1	106.422	135.09	123.4	_YQFNTN(de)VVFSDN(de)GTIVDR_	YQFN(0.193)TN(0.807)VVFSDN(1)(	YQFN(-6.2)TN(6.2)VVFSDN(106.	12	3	698	1	1	1	1
IPI00308971	575	YTDGDDCGSDKKISTNITIVCKPGDIESAPV	1	80.2359	191.22	80.236	_KISTN(de)ITIVCKPGDIESAPVIR_	KISTN(1)ITIVCKPGDIESAPVIR	KISTN(80.24)ITIVCKPGDIESAPVI	5	3	805	1	1	1	1
IPI00225715	660	MGKIGTSISSGYVRVNGTITQVIIVGAPTHD	1	100.394	207.43	100.39	_VN(de)GTITQVIIVGAPTHDDVSK_	VN(1)GTITQVIIVGAPTHDDVSK	VN(100.39)GTITQVIIVGAPTHDDVS	2	3	722	1	1	1	1
IPI00128358	445	IDNQNIRQIWDWSKHNITITQGKIFFHYNPK	1	130.295	130.29	130.29	_QIWDWSKHN(de)ITITQGK_	QIWDWSKHN(1)ITITQGK	QIWDWSKHN(130.29)ITITQGK	9	2	979	1	1	1	1
IPI00317356	253	AEIIAHKIHVYEKHANWTITPIKVINFDTIV	1	59.5419	160.36	59.542	_HAN(de)WTITPIK_	HAN(1)WTITPIK	HAN(59.54)WTITPIK	3	2	591	1	1	1	1
IPI00309230	172	VQSGPITTCRITIAINNTITPHTIPPGTIVY	0.998144	27.3057	255.98	255.98	_ITIAIN(de)NTITPHTIPPGTIVYK_	ITIAIN(0.998)N(0.002)TITPHTIPI	ITIAIN(27.31)N(-27.31)TITPHT	6	2	1190	1	1	1	1
IPI00308971	2129	SRAACAVRPQEVTMVNGTITNPVTGKSFSIG	1	69.2898	116.35	116.35	_AACAVRPQEVTM(ox)VN(de)GTITNPVTGK_	AACAVRPQEVTMVN(1)GTITNPVTGK	AACAVRPQEVTMVN(69.29)GTITN(-	14	2	1266	1	1	1	1
IPI00987265	713	PNGINVIKKGCADYCNQTITKRGCCKGFFGP	1	105.652	105.65	105.65	_KGCADYCN(de)QTITK_	KGCADYCN(1)QTITK	KGCADYCN(105.65)QTITK	8	3	521	1	1	1	1
IPI00108535	199	GESISEGDRIKISEGNRTITIINVTRNDTGP	1	63.2246	92.034	63.225	_ISEGN(de)RTITIIN(de)VTRN(de)DTGPYVCETR_	ISEGN(1)RTITIIN(1)VTRN(1)DTGPY	ISEGN(63.22)RTITIIN(63.22)VT	5	4	746	1	1	1	1
IPI00119299	402	TYRIGVQMHPGQEIHNFTITGRNPIGQAQSA	1	58.1235	123.63	58.123	_IGVQM(ox)HPGQEIHN(de)FTITGR_	IGVQMHPGQEIHN(1)FTITGR	IGVQMHPGQEIHN(58.12)FTITGR	13	2	1077	1	1	1	1
IPI00121378	480	INGRYYSKIIISPEENVTITCTAENQIERTV	1	72.2096	197.72	72.21	_IIISPEEN(de)VTITCTAEN(de)QIER_	IIISPEEN(1)VTITCTAEN(1)QIER	IIISPEEN(72.21)VTITCTAEN(72.	8	3	811	1	1	1	1
IPI00230319	132	GYYRYATENDISSIHNSTITCIVNQTTSITG	1	50.4134	115.47	50.413	_YATEN(de)DISSIHN(de)STITCIVN(de)QTTSITGTSPEIM(ox)EK_	YATEN(1)DISSIHN(1)STITCIVN(1)(	YATEN(50.41)DISSIHN(50.41)ST	12	4	969	1	1	1	1
IPI00130573	810	VKGFVIDATDGRGIINATISVAEINHPVTTY	1	124.014	242.98	242.98	_GIIN(de)ATISVAEINHPVTTYK_	GIIN(1)ATISVAEINHPVTTYK	GIIN(124.01)ATISVAEIN(-124.0	4	2	1072	1	1	1	1
IPI00894972	776	IPFEKNCGEDKKCEANITISSPARSGPIRIM	1	64.8267	104.41	64.827	_KCEAN(de)ITISSPAR_	KCEAN(1)ITISSPAR	KCEAN(64.83)ITISSPAR	5	3	483	1	1	1	1
IPI00131091	1324	SAYWIASHTTEEKAINVTISSMGRNGIKTHG	1	118.08	127.17	118.08	_AIN(de)VTISSM(ox)GRN(de)GIK_	AIN(1)VTISSMGRN(1)GIK	AIN(118.08)VTISSMGRN(118.08)	3	2	790	1	1	1	1
IPI00460291	650	PTAVTIEHVPKAISPNSTISSAPKDFAIFGF	1	87.0829	87.083	87.083	_AISPN(de)STISSAPK_	AISPN(1)STISSAPK	AISPN(87.08)STISSAPK	5	2	637	1	1	1	1
IPI00187353	140	KEIDDCEQANKIGAINSTISNESKEAFIDWA	1	58.9802	140.63	58.98	_IGAIN(de)STISN(de)ESKEAFIDWAR_	IGAIN(1)STISN(1)ESKEAFIDWAR	IGAIN(58.98)STISN(58.98)ESKE	5	3	775	1	1	1	1
IPI00221426	175	GWSYWYAIEKNSKYYNYTISINGKARKHGEN	1	108.348	199.82	108.35	_YYN(de)YTISIN(de)GK_	YYN(1)YTISIN(1)GK	YYN(108.35)YTISIN(108.35)GK	3	2	669	1	1	1	1
IPI00121378	95	YDDVPEYKDRISISENYTISIANAKISDEKR	1	67.3255	194.3	67.326	_ISISEN(de)YTISIAN(de)AK_	ISISEN(1)YTISIAN(1)AK	ISISEN(67.33)YTISIAN(67.33)A	6	2	813	1	1	1	1
IPI00132600	599	WEKEFISFVKNYKNPNITISFTAERSIEDEI	0.999762	36.2274	136.6	132.08	_NYKNPN(de)ITISFTAER_	NYKNPN(1)ITISFTAER	N(-94.9)YKN(-36.23)PN(36.23)	6	2	885	1	1	1	1
IPI00119065	1309	ANHAISGIERDGIAINITIRQIDQHIEIIKH	1	134.304	134.3	134.3	_DGIAIN(de)ITIR_	DGIAIN(1)ITIR	DGIAIN(134.3)ITIR	6	2	544	1	1	1	1
IPI00469387	154	FHINKPRRVIYIPAYNCTIRPVSKRKTHTTC	1	93.3481	93.348	93.348	_VIYIPAYN(de)CTIRPVSKR_	VIYIPAYN(1)CTIRPVSKR	VIYIPAYN(93.35)CTIRPVSKR	8	4	514	1	1	1	1
IPI00460063	196	KIMHAIGGDDYVRIINQTIRENIKKAGFSET	1	150.837	150.84	150.84	_IIN(de)QTIR_	IIN(1)QTIR	IIN(150.84)QTIR	3	2	430	1	1	1	1
IPI00124428	289	HTWSHEKVFVYTPTTNYTIRDYHRFFRDIGF	1	107.966	111.94	107.97	_VFVYTPTTN(de)YTIR_	VFVYTPTTN(1)YTIR	VFVYTPTTN(107.97)YTIR	9	2	788	1	1	1	1
IPI00407222	724	DYTGMSHVTPHIPEINDTIRAHCEEDAPICG	1	63.2722	102.21	63.272	_DSGIWIN(de)GFDYTGM(ox)SHVTPHIPEIN(de)DTIR_	DSGIWIN(1)GFDYTGMSHVTPHIPEIN(1	DSGIWIN(63.27)GFDYTGMSHVTPHI	25	3	1102	1	1	1	1
IPI00222937	184	NAVISVPAEFDIQQRNSTIQAANIAGIKIIR	1	89.9136	195.1	195.1	_N(de)STIQAANIAGIK_	N(1)STIQAANIAGIK	N(89.91)STIQAAN(-89.91)IAGIK	1	2	651	1	1	1	1
IPI00396840	315	RPQMQQIIDFETAIANITIPQEKRRDEEIIY	1	58.924	138.36	58.924	_IIGGGDEDAIRPQM(ox)QQIIDFETAIAN(de)ITIPQEKR_	IIGGGDEDAIRPQMQQIIDFETAIAN(1)I	IIGGGDEDAIRPQMQQIIDFETAIAN(5	26	5	760	1	1	1	1
IPI00276430	158	ESSEHPWKWTDNTEYNNTIPIRGEERFAYIN	0.996743	24.9314	147.56	147.56	_WTDNTEYN(de)NTIPIRGEER_	WTDNTEYN(0.997)N(0.003)TIPIRGE	WTDN(-42.62)TEYN(24.93)N(-24	8	2	1105	1	1	1	1
IPI00405742	735	SDTFSFRTPKISHDGNETIPIHIYVKSFGKN	1	49.3332	241.8	49.333	_ISHDGN(de)ETIPIHIYVK_	ISHDGN(1)ETIPIHIYVK	ISHDGN(49.33)ETIPIHIYVK	6	3	613	1	1	1	1
IPI00469218	297	MNASSSIFFIQGVRINMTIPDAIVPTFSISN	1	92.4736	100.39	92.474	_IN(de)M(ox)TIPDAIVPTFSISN(de)HSIK_	IN(1)MTIPDAIVPTFSISN(1)HSIK	IN(92.47)MTIPDAIVPTFSISN(92.	2	3	773	1	1	1	1
IPI00119063	1764	AIDFPESKIYWISSGNHTINRCNIDGSEIEV	1	101.431	254.18	101.43	_IYWISSGN(de)HTIN(de)R_	IYWISSGN(1)HTIN(1)R	IYWISSGN(101.43)HTIN(101.43)	8	2	782	1	1	1	1
IPI00121627	241	AEDYGPVEVISHWHPNITINIVDDHTPWVKG	1	94.0192	161.04	94.019	_AEDYGPVEVISHWHPN(de)ITIN(de)IVDDHTPWVK_	AEDYGPVEVISHWHPN(1)ITIN(1)IVDI	AEDYGPVEVISHWHPN(94.02)ITIN(	16	3	1162	1	1	1	1
IPI00120155	157	MICQWDPGRETYIETNYTIKSEWATEKFPDC	1	91.1801	116.13	116.13	_NM(ox)ICQWDPGRETYIETN(de)YTIK_	NMICQWDPGRETYIETN(1)YTIK	N(-91.18)MICQWDPGRETYIETN(91	17	3	884	1	1	1	1
IPI00387318	107	NKCISPNVTSCACTINFTIKQSFEGNVFMYY	1	172.496	172.5	172.5	_CISPN(de)VTSCACTIN(de)FTIK_	CISPN(1)VTSCACTIN(1)FTIK	CISPN(172.5)VTSCACTIN(172.5)	14	2	1044	1	1	1	1
IPI00378224	171	SPKVNCEERNVTGIENFTIKIINMSQDIMDF	1	106.253	192.24	192.24	_VNCEERN(de)VTGIEN(de)FTIK_	VNCEERN(1)VTGIEN(1)FTIK	VN(-80.9)CEERN(80.9)VTGIEN(1	13	2	1013	1	1	1	1
IPI00223987	256	EQIAVVAPEPIITGHNYTIKIEYSANISNSY	1	48.7591	169.28	48.759	_QVEIIEYPYHEQIAVVAPEPIITGHN(de)YTIK_	QVEIIEYPYHEQIAVVAPEPIITGHN(1)Y	QVEIIEYPYHEQIAVVAPEPIITGHN(4	26	3	1156	1	1	1	1
IPI00111794	293	TRHSVIWNAMIHPIQNMTIKGVVWYQGESNA	1	69.4849	113.01	69.485	_HSVIWN(de)AM(ox)IHPIQN(de)M(ox)TIK_	HSVIWN(1)AMIHPIQN(1)MTIK	HSVIWN(69.48)AMIHPIQN(69.48)	14	3	723	1	1	1	1
IPI00319509	234	KSFPCFDEPAMKAMFNITIIYPNNIIAISNM	1	70.4885	141.83	133.65	_AM(ox)FN(de)ITIIYPNNIIAISNM(ox)IPK_	AMFN(1)ITIIYPNNIIAISNMIPK	AMFN(70.49)ITIIYPN(-75.13)N(	4	2	1263	1	1	1	1
IPI00471089	184	NDAGVPTPAFIKAAENFTIIVKNNIWYPKFN	1	68.3555	157.68	68.355	_AAEN(de)FTIIVK_	AAEN(1)FTIIVK	AAEN(68.36)FTIIVK	4	2	554	1	1	1	1
IPI00109108	585	VMSWWDYGYQITAMANRTIIVDNNTWNNTHI	1	60.5489	212.39	60.549	_VM(ox)SWWDYGYQ(de)ITAM(ox)AN(de)R_	VMSWWDYGYQITAMAN(1)R	VMSWWDYGYQITAMAN(60.55)R	16	3	709	1	1	1	1
IPI00124933	99	WYEYKSCMDSVKDWCNWTIISRHYSDIQNCI	1	118.483	118.48	118.48	_DWCN(de)WTIISR_	DWCN(1)WTIISR	DWCN(118.48)WTIISR	4	2	676	1	1	1	1
IPI00850057	214	EGECVEWIRRYIEIGNETIIRTDSPKAHVTY	1	100.931	244	100.93	_YIEIGN(de)ETIIR_	YIEIGN(1)ETIIR	YIEIGN(100.93)ETIIR	6	2	661	1	1	1	1
IPI00119063	3049	FANRYYIRKINIDGSNYTIIKQGINNAVAID	1	155.433	199.13	155.43	_KIN(de)IDGSN(de)YTIIK_	KIN(1)IDGSN(1)YTIIK	KIN(155.43)IDGSN(155.43)YTII	8	2	741	1	1	1	1
IPI00119063	1734	WTDGDNISMANMDGSNHTIIFSGQKGPVGIA	1	104.631	131.27	104.63	_IYWTDGDN(de)ISM(ox)AN(de)M(ox)DGSN(de)HTIIFSGQK_	IYWTDGDN(1)ISMAN(1)MDGSN(1)HT	IYWTDGDN(104.63)ISMAN(104.63	18	3	1017	1	1	1	1
IPI00719927	657	YGTAIVHIYVNETIANRTIIETIIVHSIDTP	1	64.5199	112.8	64.52	_YGTAIVHIYVN(de)ETIAN(de)R_	YGTAIVHIYVN(1)ETIAN(1)R	YGTAIVHIYVN(64.52)ETIAN(64.5	16	3	646	1	1	1	1
IPI00130486	286	CERRSQSGDFIRYHYNGTIIDGTIFDSSYSR	1	44.3907	123.19	44.391	_YHYN(de)GTIIDGTIFDSSYSR_	YHYN(1)GTIIDGTIFDSSYSR	YHYN(44.39)GTIIDGTIFDSSYSR	4	3	737	1	1	1	1
IPI00319509	784	DIVVEIYSQWMKNPNNNTIHPNIRSTVYCNA	0.994124	22.3131	223.31	223.31	_NPNN(de)NTIHPNIR_	NPNN(0.994)N(0.006)TIHPNIR	N(-102.74)PN(-43.96)N(22.31)	4	2	703	1	1	1	1
IPI00115516	416	YTSIASRISRIEDRFNSTIGPSEEQEKNWPG	1	114.167	195.18	195.18	_IEDRFN(de)STIGPSEEQEKNWPGGPGR_	IEDRFN(1)STIGPSEEQEKNWPGGPGR	IEDRFN(114.17)STIGPSEEQEKN(-	6	3	934	1	1	1	1
IPI00624663	1443	EVNTNHVIIYIEKITNQTIGFSFAVEQDIPV	1	84.6534	237.14	84.653	_ITN(de)QTIGFSFAVEQDIPVK_	ITN(1)QTIGFSFAVEQDIPVK	ITN(84.65)QTIGFSFAVEQDIPVK	3	3	703	1	1	1	1
IPI00453571	98	VFDVVANKEQSFQGSNMTIFYREEIGTYPYY	1	149.216	149.22	149.22	_EQSFQGSN(de)M(ox)TIFYR_	EQSFQGSN(1)MTIFYR	EQSFQGSN(149.22)MTIFYR	8	2	863	1	1	1	1
IPI00307966	124	DYAPIVKIVTQTIPCNKTIFWSKSKHIAHQY	1	111.74	147.75	111.74	_IVTQTIPCN(de)KTIFWSK_	IVTQTIPCN(1)KTIFWSK	IVTQTIPCN(111.74)KTIFWSK	9	3	646	1	1	1	1
IPI00221998	159	DEPTCGRPIGIRAGPNGTIFVVDAYKGIFEV	1	83.8231	160.81	83.823	_AGPN(de)GTIFVVDAYK_	AGPN(1)GTIFVVDAYK	AGPN(83.82)GTIFVVDAYK	4	2	727	1	1	1	1
IPI00460063	353	DPYQQIVTTFIKGEINSTIFSSRPKDQFGIS	1	110.077	194.98	110.08	_GEIN(de)STIFSSRPK_	GEIN(1)STIFSSRPK	GEIN(110.08)STIFSSRPK	4	3	480	1	1	1	1
IPI00124700	730	IVENIKIRQKNITAFNETIFRNQIAIATWTI	1	75.4785	138.45	75.479	_QKN(de)ITAFN(de)ETIFR_	QKN(1)ITAFN(1)ETIFR	QKN(75.48)ITAFN(75.48)ETIFR	8	3	529	1	1	1	1
IPI00126186	104	YACDSKSEYQKWECKNDTIFGIKGTEIYFNY	1	98.1049	178.51	98.105	_WECKN(de)DTIFGIK_	WECKN(1)DTIFGIK	WECKN(98.1)DTIFGIK	5	2	756	1	1	1	1
IPI00134585	792	VYRYGMQNSGNEAAWNYTIEQYQKTSIAQEK	1	75.3599	202.16	202.16	_YGM(ox)QNSGNEAAWN(de)YTIEQYQK_	YGMQNSGNEAAWN(1)YTIEQYQK	YGMQN(-82.4)SGN(-75.36)EAAWN	13	3	838	1	1	1	1
IPI00118130	76	QTMQSEFFYITTNIINDTIEIRESQTIGDQC	1	63.7264	63.883	63.726	_QAIQTM(ox)QSEFFYITTN(de)IIN(de)DTIEIR_	QAIQTMQSEFFYITTN(1)IIN(1)DTIE	QAIQTMQSEFFYITTN(63.73)IIN(6	19	3	1003	1	1	1	1
IPI00122973	388	FCSAAIEVAGKFIFKNQTIEIHVIYGPRIDE	1	58.8852	203.9	58.885	_FIFKN(de)QTIEIHVIYGPR_	FIFKN(1)QTIEIHVIYGPR	FIFKN(58.89)QTIEIHVIYGPR	5	3	693	1	1	1	1
IPI00309214	52	TDHVKIIPHIEKPIQNFTICFRTYSDISRSQ	1	60.307	98.943	60.307	_IIPHIEKPIQN(de)FTICFR_	IIPHIEKPIQN(1)FTICFR	IIPHIEKPIQN(60.31)FTICFR	11	2	1064	1	1	1	1
IPI00119299	261	ETNVFPQDKVVIAGSNMTICCMSPTKVISGQ	1	71.6009	124.78	71.601	_VVIAGSN(de)M(ox)TICCM(ox)SPTK_	VVIAGSN(1)MTICCMSPTK	VVIAGSN(71.6)MTICCMSPTK	7	2	951	1	1	1	1
IPI00124221	197	IPDGYPQISCIPKEENATIATYPEFGVIDIK	1	45.3494	243.95	45.349	_IIDIIPDGYPQISCIPKEEN(de)ATIATYPEFGVIDIK_	IIDIIPDGYPQISCIPKEEN(1)ATIATYI	IIDIIPDGYPQISCIPKEEN(45.35)A	20	4	984	1	1	1	1
IPI00719927	652	GKPPRYGTAIVHIYVNETIANRTIIETIIVH	1	64.5199	112.8	64.52	_YGTAIVHIYVN(de)ETIAN(de)R_	YGTAIVHIYVN(1)ETIAN(1)R	YGTAIVHIYVN(64.52)ETIAN(64.5	11	3	646	1	1	1	1
IPI00123342	869	MTTIEKVINDTWAWKNATIAEQAKIPATEKP	1	87.49	283.49	87.49	_VIN(de)DTWAWKN(de)ATIAEQ(de)AK_	VIN(1)DTWAWKN(1)ATIAEQAK	VIN(87.49)DTWAWKN(87.49)ATIA	10	2	1032	1	1	1	1
IPI00322575	967	QYNGAIIISGDEKNYNFTIACNTKRINCFPV	0.999858	38.4692	182.1	119.45	_NYN(de)FTIACNTK_	NYN(1)FTIACNTK	N(-38.47)YN(38.47)FTIACN(-75	3	2	674	1	1	1	1

IPI00128358	653	SQIIIKWKPPSDPNGNITHYIVYWERQAEDS	1	107.724	107.72	107.72	_WKPPSDPN(de)GN(de)ITHYIVYWER_	WKPPSDPN(1)GN(1)ITHYIVYWER	WKPPSDPN(107.72)GN(107.72)IT	10	3	825	1	1	1	1
IPI00749655	159	AHPYKVSESEKSQIVNETHWQYYGTSDTRGN	1	111.645	174.02	111.65	_SQIVN(de)ETHWQYYGTSDTR_	SQIVN(1)ETHWQYYGTSDTR	SQIVN(111.65)ETHWQYYGTSDTR	5	2	1093	1	1	1	1
IPI00114206	122	GRCAMDIGVNYIGTVNVTHTGIQCQIWRSRY	0.999931	41.6248	157.28	74.698	_CAM(ox)DIGVNYIGTVN(de)VTHTGIQCQIWR_	CAMDIGVNYIGTVN(1)VTHTGIQCQIWR	CAMDIGVN(-41.62)YIGTVN(41.62	14	4	757	1	1	1	1
IPI00316430	356	YANPPNYGNPDPIVINNTHRVIKGDYKTPAN	0.999348	34.3009	137.72	101.5	_IGYDPYANPPNYGNPDPIVIN(de)N(de)THR_	IGYDPYANPPN(0.001)YGNPDPIVIN((	IGYDPYAN(-39.41)PPN(-34.3)YG	21	3	938	1	1	1	1
IPI00132600	962	YFDWVSPQSSCCRIYNVTHQFCNASVMDPTC	1	167.281	235.95	167.28	_IYN(de)VTHQFCN(de)ASVM(ox)DPTCVR_	IYN(1)VTHQFCN(1)ASVMDPTCVR	IYN(167.28)VTHQFCN(167.28)AS	3	3	811	1	1	1	1
IPI00108003	449	GHTDRCDEAQAIQVWNDTHPEVISQKPFDKV	1	62.6046	62.605	62.605	_GHTDRCDEAQAIQVWN(de)DTHPEVISQKPFDK_	GHTDRCDEAQAIQVWN(1)DTHPEVISQKI	GHTDRCDEAQAIQVWN(62.6)DTHPEV	16	4	881	1	1	1	1
IPI00320618	58	ITHIPDDIPSNITVINITHNQIRRIPPTNFT	0.999698	35.1941	92.993	92.993	_ITHIPDDIPSN(de)ITVIN(de)ITHNQIR_	ITHIPDDIPSN(1)ITVIN(1)ITHNQIR	ITHIPDDIPSN(45.98)ITVIN(35.1	16	3	876	1	1	1	1
IPI00110264	960	VYRDINSQIEIQNVTNDTHITIIGIKPDTTY	1	78.6534	145.2	145.2	_DINSQIEIQN(de)VTN(de)DTHITIIGIKPDTTYDIK_	DINSQIEIQN(1)VTN(1)DTHITIIGIKI	DIN(-72.7)SQIEIQN(72.7)VTN(7	13	3	1172	1	1	1	1
IPI00874858	426	WRYDKSEDVGAAAMINYTHIIMEAVPGHPAI	1	57.4316	110.94	57.432	_YDKSEDVGAAAM(ox)IN(de)YTHIIM(ox)EAVPGHPAIYR_	YDKSEDVGAAAMIN(1)YTHIIMEAVPGHI	YDKSEDVGAAAMIN(57.43)YTHIIME	14	4	867	1	1	1	1
IPI00331407	51	SGGSVAHIAEIGIPTNITHIIIFRMDQGIIR	1	64.5471	72.676	64.547	_DAAQCSGGSVAHIAEIGIPTN(de)ITHIIIFR_	DAAQCSGGSVAHIAEIGIPTN(1)ITHII]	DAAQCSGGSVAHIAEIGIPTN(64.55)	21	3	1022	1	1	1	1
IPI00118130	94	IEIRESQTIGDQCVYNSTHIGFQRENGTFSK	1	113.735	268.25	113.73	_ESQTIGDQCVYN(de)STHIGFQR_	ESQTIGDQCVYN(1)STHIGFQR	ESQTIGDQCVYN(113.73)STHIGFQR	12	2	1171	1	1	1	1
IPI00108844	108	QINKSNDKETVVGRINETHIFNGSNWIMIIY	0.999999	62.3539	211.57	211.57	_IN(de)ETHIFN(de)GSNWIM(ox)IIYK_	IN(1)ETHIFN(1)GSNWIMIIYK	IN(62.35)ETHIFN(41.93)GSN(-4	2	3	738	1	1	1	1
IPI00405742	919	EPRQGPQAGGTTITINGTHIDTGSKEDVRVT	1	95.6224	260.06	95.622	_QGPQAGGTTITIN(de)GTHIDTGSKEDVR_	QGPQAGGTTITIN(1)GTHIDTGSKEDVR	QGPQAGGTTITIN(95.62)GTHIDTGS	13	3	885	1	1	1	1
IPI01026704	217	IGPKQTQVGIVQYGANVTHEFNINKYSSTEE	1	113.756	237.31	237.31	_QTQVGIVQYGAN(de)VTHEFN(de)INK_	QTQVGIVQYGAN(1)VTHEFN(0.999)I	QTQVGIVQYGAN(113.76)VTHEFN(3	12	3	788	1	1	1	1
IPI00119063	1646	YWSDVRTQAIKRAFINGTGVETVVSADIPNA	1	57.4316	181.35	57.432	_AFIN(de)GTGVETVVSADIPN(de)AHGIAVDWVSR_	AFIN(1)GTGVETVVSADIPN(1)AHGIA\	AFIN(57.43)GTGVETVVSADIPN(57	4	3	1000	1	1	1	1
IPI00331214	417	IKNIKRPYIVPIIWINETGTIGDEKAEMFKT	1	73.7832	109.54	73.783	_RPYIVPIIWIN(de)ETGTIGDEKAEM(ox)FK_	RPYIVPIIWIN(1)ETGTIGDEKAEMFK	RPYIVPIIWIN(73.78)ETGTIGDEKA	11	4	735	1	1	1	1
IPI00108811	78	RRGRRMEISVGAIQANRTGTGIIITIQPEKK	1	81.4939	134.08	81.494	_RM(ox)EISVGAIQAN(de)R_	RMEISVGAIQAN(1)R	RMEISVGAIQAN(81.49)R	12	3	488	1	1	1	1
IPI00121776	69	RIPSSIENATSISIMNITGTAICHISDIPPD	1	59.046	61.499	59.046	_IPSSIEN(de)ATSISIM(ox)N(de)ITGTAICHISDIPPDGIR_	IPSSIEN(1)ATSISIMN(1)ITGTAICH]	IPSSIEN(59.05)ATSISIMN(59.05	15	4	879	1	1	1	1
IPI00125182	147	ERVWPDGVIPFVIGGNFTGSQRAVFRQAMRH	1	47.5678	143.83	47.568	_VWPDGVIPFVIGGN(de)FTGSQR_	VWPDGVIPFVIGGN(1)FTGSQR	VWPDGVIPFVIGGN(47.57)FTGSQR	14	3	716	1	1	1	1
IPI00165807	465	TRGYNSRGFGVAFVGNYTGSIPNEAAINTVR	1	78.736	195.46	195.46	_GFGVAFVGN(de)YTGSIPNEAAINTVR_	GFGVAFVGN(1)YTGSIPNEAAINTVR	GFGVAFVGN(78.74)YTGSIPN(-78.	9	3	819	1	1	1	1
IPI00471080	489	VGTSEGWNVPMGIIYNQTGSCKFDAFFSRSC	0.999817	37.3647	123.44	123.44	_KSCHTAVGTSEGWNVPM(ox)GIIYN(de)QTGSCK_	KSCHTAVGTSEGWNVPMGIIYN(1)QTGS(	KSCHTAVGTSEGWN(-37.36)VPMGII	22	3	1034	1	1	1	1
IPI00315593	177	SRERAEGYPKMAAAINATGRPIAFSCSWPAY	1	62.3934	125.74	62.393	_M(ox)AAAIN(de)ATGRPIAFSCSWPAYEGGIPPKVN(de)YTEVSR_	MAAAIN(1)ATGRPIAFSCSWPAYEGGIPI	MAAAIN(62.39)ATGRPIAFSCSWPAY	6	4	976	1	1	1	1
IPI00125310	146	VIFDKVITNQESPYQNHTGRFICAVPGFYYF	1	89.4845	164.15	89.484	_VITN(de)QESPYQN(de)HTGR_	VITN(1)QESPYQN(1)HTGR	VITN(89.48)QESPYQN(89.48)HTG	11	2	873	1	1	1	1
IPI01026704	459	TIPGDVIYIAGQPRYNHTGQVVIYKMEDGDV	1	58.2738	167.2	58.274	_YN(de)HTGQVVIYK_	YN(1)HTGQVVIYK	YN(58.27)HTGQVVIYK	2	2	662	1	1	1	1
IPI00111960	470	YRPYDEGIRRGVFITNETGQPIIGKVWPGTT	1	81.703	260.96	81.703	_RGVFITN(de)ETGQ(de)PIIGK_	RGVFITN(1)ETGQPIIGK	RGVFITN(81.7)ETGQPIIGK	7	3	578	1	1	1	1
IPI00129265	159	EMAKYDIPASINYIINKTGQEQIYYVGHSQG	1	73.877	138.99	73.877	_YDIPASIN(de)YIIN(de)K_	YDIPASIN(1)YIIN(1)K	YDIPASIN(73.88)YIIN(73.88)K	12	2	763	1	1	1	1
IPI00108535	89	AIDKEIARFVPNSNMNFTGQAYSGREIIYSN	1	127.753	235.71	127.75	_FVPN(de)SN(de)M(ox)N(de)FTGQAYSGR_	FVPN(1)SN(1)MN(1)FTGQAYSGR	FVPN(127.75)SN(127.75)MN(127	8	3	637	1	1	1	1
IPI00108535	210	ISEGNRTITIINVTRNDTGPYVCETRNPVSV	1	111.064	238.19	111.06	_TITIIN(de)VTRN(de)DTGPYVCETR_	TITIIN(1)VTRN(1)DTGPYVCETR	TITIIN(111.06)VTRN(111.06)DT	10	3	776	1	1	1	1
IPI00929886	107	ISKEKDVIWFRPTIINDTGNYTCMIRNTTYC	1	92.3901	92.39	92.39	_DVIWFRPTIIN(de)DTGN(de)YTCM(ox)IR_	DVIWFRPTIIN(1)DTGN(1)YTCMIR	DVIWFRPTIIN(92.39)DTGN(92.39	11	3	868	1	1	1	1
IPI00127280	97	RQPKDCDFIEDGEERNCTGKFFRRRQSTSIT	1	75.4622	75.462	75.462	_DCDFIEDGEERN(de)CTGK_	DCDFIEDGEERN(1)CTGK	DCDFIEDGEERN(75.46)CTGK	12	3	649	1	1	1	1
IPI00122293	120	IQNNFITEIPIESFQNATGIRWVNIDNNRIR	1	134.465	233.56	134.47	_IHYIYIQN(de)N(de)FITEIPIESFQN(de)ATGIR_	IHYIYIQN(1)N(1)FITEIPIESFQN(1)	IHYIYIQN(134.47)N(134.47)FIT	21	3	1033	1	1	1	1
IPI00263041	439	DQISKVERQVEEIIVNHTGIREIRVIIMEKS	1	93.8227	103.76	93.823	_QVEEIIVN(de)HTGIR_	QVEEIIVN(1)HTGIR	QVEEIIVN(93.82)HTGIR	8	2	755	1	1	1	1
IPI00122272	535	TNYIRNVAIVAGDTGNATGIGEQGPTRGTDA	1	129.726	261.06	261.06	_NVAIVAGDTGN(de)ATGIGEQGPTR_	NVAIVAGDTGN(1)ATGIGEQGPTR	N(-129.73)VAIVAGDTGN(129.73)	11	2	1050	1	1	1	1
IPI00378224	165	STDSIKSPKVNCEERNVTGIENFTIKIINMS	1	80.8966	192.24	192.24	_VNCEERN(de)VTGIEN(de)FTIK_	VNCEERN(1)VTGIEN(1)FTIK	VN(-80.9)CEERN(80.9)VTGIEN(1	7	2	1013	1	1	1	1
IPI00378224	277	NHTDRTIETIKIFIFNQTGIEAKKNVVVTQA	1	74.9616	178.15	74.962	_IFIFN(de)QTGIEAKK_	IFIFN(1)QTGIEAKK	IFIFN(74.96)QTGIEAKK	5	3	504	1	1	1	1
IPI00405742	1103	TEAGVFEYVADPTFENFTGGVKKQVNKIIHA	1	163.448	238.8	163.45	_TEAGVFEYVADPTFEN(de)FTGGVKK_	TEAGVFEYVADPTFEN(1)FTGGVKK	TEAGVFEYVADPTFEN(163.45)FTGG	16	3	836	1	1	1	1
IPI00119063	4126	NRVFKIHKFGHSPIINITGGISHASDVVIYH	1	83.8712	131.56	83.871	_FGHSPIIN(de)ITGGISHASDVVIYHQHK_	FGHSPIIN(1)ITGGISHASDVVIYHQHK	FGHSPIIN(83.87)ITGGISHASDVVI	8	3	943	1	1	1	1
IPI00309230	627	KTSAFIIRERYWRIANETGGHGSGPRTQCFG	1	52.5549	183.96	52.555	_IAN(de)ETGGHGSGPR_	IAN(1)ETGGHGSGPR	IAN(52.55)ETGGHGSGPR	3	3	419	1	1	1	1
IPI00128154	221	AKDGSCKYRAEFAVANDTGFVDIPQQEKAIM	1	82.4822	220.28	82.482	_YRAEFAVAN(de)DTGFVDIPQQEK_	YRAEFAVAN(1)DTGFVDIPQQEK	YRAEFAVAN(82.48)DTGFVDIPQQEK	9	3	800	1	1	1	1
IPI00322497	153	AINGEEFMKFNPRIGNWTGEWPETEIVANIW	1	117.519	208.49	132.14	_IGN(de)WTGEWPETEIVANIWM(ox)K_	IGN(1)WTGEWPETEIVANIWMK	IGN(117.52)WTGEWPETEIVAN(-11	3	2	1196	1	1	1	1
IPI00323134	325	SQAPSTPSPNMFTINNETGDIITVAAGIDRE	0.99986	38.5293	131.62	106.44	_IISQAPSTPSPNM(ox)FTIN(de)N(de)ETGDIITVAAGIDREK_	IISQAPSTPSPN(0.001)MFTIN(1)N(1	IISQAPSTPSPN(-33.08)MFTIN(33	18	3	1207	1	1	1	1
IPI00468674	86	IAGAPRDIAVGDDYTNRTGAVYICPITAHKD	1	107.026	107.03	107.03	_DIAVGDDYTN(de)R_	DIAVGDDYTN(1)R	DIAVGDDYTN(107.03)R	10	2	620	1	1	1	1
IPI00652107	171	CEQGPSIVTPPKDIWNVTGAKVFISCEVIGI	1	124.307	124.31	124.31	_GTCEQGPSIVTPPKDIWN(de)VTGAK_	GTCEQGPSIVTPPKDIWN(1)VTGAK	GTCEQGPSIVTPPKDIWN(124.31)VT	18	3	819	1	1	1	1
IPI00120674	58	GTSVVVAAPQEAKAVNQTGAIYQCDYSTSRC	1	114.586	194.92	114.59	_AVN(de)QTGAIYQCDYSTSR_	AVN(1)QTGAIYQCDYSTSR	AVN(114.59)QTGAIYQCDYSTSR	3	3	646	1	1	1	1
IPI00226346	102	KCRRFAQPQWHIIHINGTFSNETEPDTEPCV	1	60.0289	60.029	60.029	_FAQPQWHIIHIN(de)GTFSN(de)ETEPDTEPCVDGWVYDR_	FAQPQWHIIHIN(1)GTFSN(1)ETEPDTI	FAQPQWHIIHIN(60.03)GTFSN(60.	12	4	1016	1	1	1	1
IPI00118130	104	DQCVYNSTHIGFQRENGTFSKYEGGVETFAH	1	104.837	224.86	104.84	_EN(de)GTFSKYEGGVETFAHIIVIR_	EN(1)GTFSKYEGGVETFAHIIVIR	EN(104.84)GTFSKYEGGVETFAHIIV	2	4	618	1	1	1	1
IPI00112614	151	KHPNSNIKIQDFIVDNETFSGFIQHNISIPR	1	148.889	149.85	148.89	_IQDFIVDN(de)ETFSGFIQHN(de)ISIPR_	IQDFIVDN(1)ETFSGFIQHN(1)ISIPR	IQDFIVDN(148.89)ETFSGFIQHN(1	8	3	898	1	1	1	1
IPI00807983	179	KEVHTAWTQPREAQYNSTFRVVSAIPIQHQD	1	73.4995	140.49	73.499	_EAQYN(de)STFR_	EAQYN(1)STFR	EAQYN(73.5)STFR	5	2	559	1	1	1	1
IPI00475246	313	VEVHTAQTQPREEQFNSTFRSVSEIPIMHQD	1	70.9189	159.83	70.919	_EEQFN(de)STFR_	EEQFN(1)STFR	EEQFN(70.92)STFR	5	1	1159	1	1	1	1
IPI00762091	1133	PAATPPAPGAEISYINHTFRIIQKPIRWKDA	1	49.7651	124	49.765	_GTDPSISPSPAATPPAPGAEISYIN(de)HTFR_	GTDPSISPSPAATPPAPGAEISYIN(1)HT	GTDPSISPSPAATPPAPGAEISYIN(49	25	4	739	1	1	1	1
IPI01026704	418	GTVVMQKANQIVIPHNTTFQTEPTKMNEPIA	1	113.883	227	113.88	_AN(de)QIVIPHN(de)TTFQTEPTK_	AN(1)QIVIPHN(1)TTFQTEPTK	AN(113.88)QIVIPHN(113.88)TTF	9	2	1021	1	1	1	1
IPI00312711	517	PDVIIAKVSGQMHMQNITFQTEASVAQQEKE	1	86.5572	198.35	86.557	_VSGQM(ox)HM(ox)QN(de)ITFQTEASVAQQEKEFK_	VSGQMHMQN(1)ITFQTEASVAQQEKEFK	VSGQMHMQN(86.56)ITFQTEASVAQQ	9	3	1010	1	1	1	1
IPI00111794	427	YRIHIGARAVAYGEKNITFQGPIPKKIEIIA	1	104.167	248.54	104.17	_AVAYGEKN(de)ITFQ(de)GPIPK_	AVAYGEKN(1)ITFQGPIPK	AVAYGEKN(104.17)ITFQGPIPK	8	3	612	1	1	1	1
IPI00624663	157	VKFRVVSVDISFRPINETFPVVYIETPKRNR	1	103.263	264.92	103.26	_VVSVDISFRPIN(de)ETFPVVYIETPKR_	VVSVDISFRPIN(1)ETFPVVYIETPKR	VVSVDISFRPIN(103.26)ETFPVVYI	12	3	970	1	1	1	1
IPI00262024	256	IEQTPRGYITSFEMFNSTFKIYTHSYIGFGI	1	105.139	264.29	105.14	_GYITSFEMFN(de)STFK_	GYITSFEMFN(1)STFK	GYITSFEMFN(105.14)STFK	10	2	837	1	1	1	1
IPI00855103	399	QFTSKIGNFSEIATHNQTFIKKIRNQITHTV	1	69.9345	199.37	69.935	_IGN(de)FSEIATHN(de)QTFIKK_	IGN(1)FSEIATHN(1)QTFIKK	IGN(69.93)FSEIATHN(69.93)QTF	11	2	976	1	1	1	1
IPI00170355	465	GNMDRHHYETFEKFGNETFIIHIDNGRGFGK	1	100.223	153.39	100.22	_FGN(de)ETFIIHIDN(de)GR_	FGN(1)ETFIIHIDN(1)GR	FGN(100.22)ETFIIHIDN(100.22)	3	3	546	1	1	1	1
IPI00343568	78	NVIPTIQNTTFSRIINITFIDITRCQIYWIH	1	89.4035	147.33	89.403	_IIN(de)ITFIDITR_	IIN(1)ITFIDITR	IIN(89.4)ITFIDITR	3	2	660	1	1	1	1
IPI00130486	174	CPRTIQVSDFVRYHYNGTFIDGTIFDSSHNR	1	175.474	248.97	248.97	_YHYN(de)GTFIDGTIFDSSHNR_	YHYN(1)GTFIDGTIFDSSHNR	YHYN(175.47)GTFIDGTIFDSSHN(-	4	3	749	1	1	1	1
IPI00109375	315	AQGSQIIVHSVDRMVNTTFICTATNAVGTGR	1	83.7509	259.18	83.751	_M(ox)VN(de)TTFICTATN(de)AVGTGR_	MVN(1)TTFICTATN(1)AVGTGR	MVN(83.75)TTFICTATN(83.75)AV	3	3	645	1	1	1	1
IPI00115976	678	YGEKKHVYIGDKNAINITFHAQNIGEGGAYE	1	90.2205	90.22	90.22	_N(de)AIN(de)ITFHAQN(de)IGEGGAYEAEIR_	N(1)AIN(1)ITFHAQN(1)IGEGGAYEAI	N(90.22)AIN(90.22)ITFHAQN(90	4	3	831	1	1	1	1
IPI01026704	112	IPVNTSIPNVTEIKENMTFGSTIVTNPKGGF	1	126.613	160.8	160.8	_EN(de)M(ox)TFGSTIVTNPK_	EN(1)MTFGSTIVTNPK	EN(126.61)MTFGSTIVTN(-126.61	2	2	778	1	1	1	1
IPI00132474	481	CQSHGIPASPKCHEGNGTFECGACRCNEGRV	1	92.9393	134.04	92.939	_CHEGN(de)GTFECGACR_	CHEGN(1)GTFECGACR	CHEGN(92.94)GTFECGACR	5	2	828	1	1	1	1
IPI00123442	60	HHNISIIPPPTEIVCNRTFDKYSCWPDTPPN	1	99.4091	99.409	99.409	_IYSDQCHHN(de)ISIIPPPTEIVCN(de)R_	IYSDQCHHN(1)ISIIPPPTEIVCN(1)R	IYSDQCHHN(99.41)ISIIPPPTEIVC	22	3	922	1	1	1	1
IPI00453501	179	AICQRYYHRGHVDPANDTFDIDPRVVTDCIQ	1	95.2362	95.236	95.236	_GHVDPAN(de)DTFDIDPR_	GHVDPAN(1)DTFDIDPR	GHVDPAN(95.24)DTFDIDPR	7	2	835	1	1	1	1
IPI00227857	287	GRFCNIVPTEHCFIGNGTEYRGVASTAASGI	1	87.3582	135.09	126.25	_FCNIVPTEHCFIGN(de)GTEYR_	FCNIVPTEHCFIGN(1)GTEYR	FCN(-87.36)IVPTEHCFIGN(87.36	14	3	772	1	1	1	1
IPI00119063	447	ANTQQKTSVIRVNRFNSTEYQVVTRVDKGGA	1	67.2345	182.02	67.234	_VN(de)RFN(de)STEYQVVTR_	VN(1)RFN(1)STEYQVVTR	VN(67.23)RFN(67.23)STEYQVVTR	5	3	572	1	1	1	1
IPI00122399	206	VGKGYMVSCIVDHRGNITEYQCHQYITKMTA	1	118.507	212.39	118.51	_GN(de)ITEYQCHQYITK_	GN(1)ITEYQCHQYITK	GN(118.51)ITEYQCHQYITK	2	2	878	1	1	1	1
IPI00136942	158	DHVNVSMIEATDFPFNTTEWEGYIPKENFRT	1	58.3237	155.25	58.324	_DHVN(de)VSM(ox)IEATDFPFN(de)TTEWEGYIPK_	DHVN(1)VSMIEATDFPFN(1)TTEWEGY]	DHVN(58.32)VSMIEATDFPFN(58.3	16	3	1020	1	1	1	1
IPI00225072	279	SVIDITPVAAIIPKINVTEVYDKIKRCNPHM	1	130.565	130.56	130.56	_IN(de)VTEVYDKIK_	IN(1)VTEVYDKIK	IN(130.56)VTEVYDKIK	2	2	662	1	1	1	1
IPI00315593	201	SCSWPAYEGGIPPKVNYTEVSRVCNIWRNYK	1	62.3934	62.393	62.393	_M(ox)AAAIN(de)ATGRPIAFSCSWPAYEGGIPPKVN(de)YTEVSR_	MAAAIN(1)ATGRPIAFSCSWPAYEGGIPI	MAAAIN(62.39)ATGRPIAFSCSWPAY	30	4	976	1	1	1	1
IPI00658539	158	AGVGFSYSDDKMYVTNDTEVAENNYEAIKDF	0.999858	37.876	183.56	183.56	_M(ox)YVTN(de)DTEVAEN(de)NYEAIKDFFR_	MYVTN(1)DTEVAEN(0.872)N(0.128)	MYVTN(37.88)DTEVAEN(8.35)N(-	5	3	896	1	1	1	1
IPI00119299	675	SRSEPCIIDWRKVPSNSTETVIESDQFQPGV	1	176.939	176.94	176.94	_KVPSN(de)STETVIESDQFQPGVR_	KVPSN(1)STETVIESDQFQPGVR	KVPSN(176.94)STETVIESDQFQPGV	5	3	774	1	1	1	1
IPI00131830	105	AKGKTMEEIIEGIKFNITETPEADIHQGFGN	0.990654	20.4225	44.347	44.347	_FN(de)ITETPEADIHQGFGNIIQSISQPEDQDQINIGNAM(ox)FIEK_	FN(0.991)ITETPEADIHQGFGN(0.00	FN(20.42)ITETPEADIHQGFGN(-20	2	5	925	1	1	1	1
IPI00132600	1064	ADYTDAMKKARIIASNITETMRSKGSDYRVF	1	73.6317	73.632	73.632	_IIASN(de)ITETM(ox)R_	IIASN(1)ITETMR	IIASN(73.63)ITETMR	5	2	633	1	1	1	1
IPI00129079	21	SMATPGEQCAGIRVWNQTEQEPAAYHIISIC	1	99.0214	160.55	99.021	_VWN(de)QTEQEPAAYHIISICFVR_	VWN(1)QTEQEPAAYHIISICFVR	VWN(99.02)QTEQEPAAYHIISICFVR	3	3	855	1	1	1	1
IPI00848693	1297	NGMRVIIIIDPAISGNETEPYPAFTRGVEND	1	57.0451	177.08	57.045	_VIIIIDPAISGN(de)ETEPYPAFTR_	VIIIIDPAISGN(1)ETEPYPAFTR	VIIIIDPAISGN(57.05)ETEPYPAFT	12	3	806	1	1	1	1
IPI00381303	767	EIIKRRDDYRPTWTINQTEPVAGNYYPVNTR	1	82.4073	167.34	159.98	_DDYRPTWTIN(de)QTEPVAGNYYPVNTR_	DDYRPTWTIN(1)QTEPVAGNYYPVNTR	DDYRPTWTIN(82.41)QTEPVAGN(-8	10	3	991	1	1	1	1
IPI00226346	107	AQPQWHIIHINGTFSNETEPDTEPCVDGWVY	1	60.0289	60.029	60.029	_FAQPQWHIIHIN(de)GTFSN(de)ETEPDTEPCVDGWVYDR_	FAQPQWHIIHIN(1)GTFSN(1)ETEPDTI	FAQPQWHIIHIN(60.03)GTFSN(60.	17	4	1016	1	1	1	1
IPI00230324	320	RRIAITPAHIIFIADNHTEPAAHFRATFASH	1	52.6119	108.56	52.612	_IAITPAHIIFIADN(de)HTEPAAHFR_	IAITPAHIIFIADN(1)HTEPAAHFR	IAITPAHIIFIADN(52.61)HTEPAAH	14	4	640	1	1	1	1
IPI00396840	269	VDQSGIGIPSRDYYINKTENEKVITGYINYM	1	141.084	174.24	141.08	_DYYIN(de)KTEN(de)EK_	DYYIN(1)KTEN(1)EK	DYYIN(141.08)KTEN(141.08)EK	5	2	710	1	1	1	1
IPI00108098	157	YVQAQVKCCGWVSHYNWTENEEIMGFTKTTY	1	70.8863	147.06	147.06	_CCGWVSHYN(de)WTENEEIM(ox)GFTK_	CCGWVSHYN(1)WTENEEIMGFTK	CCGWVSHYN(70.89)WTEN(-70.89)	9	3	889	1	1	1	1
IPI00469307	271	EEPRVIDIWDIAQSANFTEKEIESFREEIKH	1	60.7346	141.85	60.735	_VIDIWDIAQSAN(de)FTEKEIESFREEIK_	VIDIWDIAQSAN(1)FTEKEIESFREEIK	VIDIWDIAQSAN(60.73)FTEKEIESF	12	4	779	1	1	1	1
IPI00344686	302	NDIISVQGNTGPSWINKTEKAFFRGRDSREE	1	68.2173	186.06	186.06	_GVTNDIISVQGN(de)TGPSWIN(de)KTEK_	GVTN(0.008)DIISVQGN(0.992)TGPS	GVTN(-21.11)DIISVQGN(21.11)T	19	2	1231	1	1	1	1
IPI00123223	294	VNSQIDNNGCSTQEVNITEIQSKKRNYEVQI	1	98.2101	195.15	98.21	_EVN(de)SQIDN(de)N(de)GCSTQEVN(de)ITEIQSK_	EVN(1)SQIDN(1)N(1)GCSTQEVN(1)]	EVN(98.21)SQIDN(98.21)N(98.2	17	3	904	1	1	1	1
IPI01026704	105	MPCVKIDIPVNTSIPNVTEIKENMTFGSTIV	1	111.643	168.41	111.64	_IDIPVN(de)TSIPN(de)VTEIK_	IDIPVN(1)TSIPN(1)VTEIK	IDIPVN(111.64)TSIPN(111.64)V	11	3	586	1	1	1	1
IPI00322575	112	SYMTGRKIIASPNEENMTEIISMRFSDVVGV	1	50.8308	203.2	50.831	_KIIASPN(de)EEN(de)M(ox)TEIISM(ox)R_	KIIASPN(1)EEN(1)MTEIISMR	KIIASPN(50.83)EEN(50.83)MTEI	10	3	704	1	1	1	1
IPI00855103	197	HFNIRTARDISVFAPNMTEIIKDVTQYREGM	1	63.3021	99.747	63.302	_DISVFAPN(de)M(ox)TEIIKDVTQYR_	DISVFAPN(1)MTEIIKDVTQYR	DISVFAPN(63.3)MTEIIKDVTQYR	8	2	1179	1	1	1	1
IPI00406901	309	KVESNRISKASSIMVNITEIFPKPKIEFSSS	1	66.2152	235.22	66.215	_ASSIM(ox)VN(de)ITEIFPKPK_	ASSIMVN(1)ITEIFPKPK	ASSIMVN(66.22)ITEIFPKPK	7	3	598	1	1	1	1
IPI00122399	673	DHIDDIAVECRDIVGNITEIESEDIQIEAII	1	243.231	262.12	243.23	_DIVGN(de)ITEIESEDIQ(de)IEAIIM(ox)R_	DIVGN(1)ITEIESEDIQIEAIIMR	DIVGN(243.23)ITEIESEDIQIEAII	5	3	840	1	1	1	1
IPI00136642	225	ENPEQSRVTINNWVANKTEGRIKDVIPQGAI	1	82.9077	190.41	82.908	_VTIN(de)N(de)WVAN(de)KTEGR_	VTIN(1)N(1)WVAN(1)KTEGR	VTIN(82.91)N(82.91)WVAN(82.9	9	2	803	1	1	1	1
IPI00129159	221	DDRIVKIPSGTIVISNATEGDGGIYRCIVES	1	60.1337	158.92	60.134	_IVKIPSGTIVISN(de)ATEGDGGIYR_	IVKIPSGTIVISN(1)ATEGDGGIYR	IVKIPSGTIVISN(60.13)ATEGDGGI	13	3	788	1	1	1	1

IPI00754386	276	RYIIQDTWIEKKWGHNVTEFQQRFDGIITEG	1	51.841	88.338	51.841	_WGHN(de)VTEFQQR_	WGHN(1)VTEFQQR	WGHN(51.84)VTEFQQR	4	2	702	1	1	1	1
IPI00133103	160	PICVHIMMSGTVTKVNKTEEDYARDSIFVRH	1	154.15	154.15	154.15	_VN(de)KTEEDYAR_	VN(1)KTEEDYAR	VN(154.15)KTEEDYAR	2	2	613	1	1	1	1
IPI00119809	398	FHTVPVEVIAKYKGINITEDTYKPRIYTSST	1	100.376	223.31	100.38	_YKGIN(de)ITEDTYKPR_	YKGIN(1)ITEDTYKPR	YKGIN(100.38)ITEDTYKPR	5	4	425	1	1	1	1
IPI00122122	173	NDSVIDVFERRVREANITEDQIIFFPKYGFC	1	82.202	190.11	82.202	_VREAN(de)ITEDQIIFFPK_	VREAN(1)ITEDQIIFFPK	VREAN(82.2)ITEDQIIFFPK	5	3	641	1	1	1	1
IPI00124666	111	ASPEMQSKCHQKGKNNQTECFNHVRFIQRIN	0.999999	59.9522	147.33	110.31	_GKN(de)N(de)QTECFNHVR_	GKN(1)N(1)QTECFNHVR	GKN(67.33)N(59.95)QTECFN(-59	4	3	536	1	1	1	1
IPI00828222	450	PYPSPSREPPRVIPFNVTDYCQIVRYICQNG	1	83.2035	210.64	83.204	_VIPFN(de)VTDYCQIVR_	VIPFN(1)VTDYCQIVR	VIPFN(83.2)VTDYCQIVR	5	3	576	1	1	1	1
IPI00130573	171	ASGYRRGDPRIVRIINTTDVYIIPSINPDGF	1	116.068	152.06	152.06	_IIN(de)TTDVYIIPSINPDGFER_	IIN(1)TTDVYIIPSINPDGFER	IIN(116.07)TTDVYIIPSIN(-116.	3	2	1140	1	1	1	1
IPI00116355	357	FTENPCIRNAIQAFGNGTDVNMSPKGPTFSA	1	52.1723	162.26	52.172	_N(de)AIQAFGN(de)GTDVN(de)M(ox)SPK_	N(1)AIQAFGN(1)GTDVN(1)MSPK	N(52.17)AIQAFGN(52.17)GTDVN(	8	2	892	1	1	1	1
IPI00322418	353	IFRHSQQPIITYEKYNVTDTPKSVGGSRSSP	1	69.9345	167.28	69.935	_HSQQPIITYEKYN(de)VTDTPK_	HSQQPIITYEKYN(1)VTDTPK	HSQQPIITYEKYN(69.93)VTDTPK	13	3	755	1	1	1	1
IPI00119063	2128	DRTHANGSIKRGCKDNATDSVPIRTGIGVQI	1	73.8867	202.29	73.887	_RGCKDN(de)ATDSVPIR_	RGCKDN(1)ATDSVPIR	RGCKDN(73.89)ATDSVPIR	6	2	795	1	1	1	1
IPI00119063	4076	YWADAKISVIGSIRINGTDPIVAADSKRGIS	1	73.91	107.69	73.91	_IN(de)GTDPIVAADSKR_	IN(1)GTDPIVAADSKR	IN(73.91)GTDPIVAADSKR	2	2	729	1	1	1	1
IPI01026704	779	DKHDFQDSVRVTIDFNITDPENGPVIDDAIP	1	63.3817	144.7	144.7	_VTIDFN(de)ITDPEN(de)GPVIDDAIPNSVHGHIPFAK_	VTIDFN(1)ITDPEN(0.985)GPVIDDA	VTIDFN(63.38)ITDPEN(18.23)GP	6	3	1149	1	1	1	1
IPI00130117	226	GAPGSFYWAGTIKVINITDNTYFKINDEAIM	1	106.007	106.01	106.01	_VIN(de)ITDN(de)TYFK_	VIN(1)ITDN(1)TYFK	VIN(106.01)ITDN(106.01)TYFK	3	2	665	1	1	1	1
IPI00856723	307	QHAVISGPNIFINNINKTDNGTYRCEASNIV	0.960615	13.196	148.89	148.89	_VDDEM(ox)PQHAVISGPNIFINN(de)IN(de)KTDN(de)GTYR_	VDDEMPQHAVISGPNIFIN(0.178)N(0.	VDDEMPQHAVISGPN(-75.02)IFIN(	22	3	1131	1	1	1	1
IPI00471081	444	YDIAPRAKIFRRDQGNVTDMASMKYIMRYNN	1	65.2948	172.76	65.295	_RDQGN(de)VTDM(ox)ASM(ox)K_	RDQGN(1)VTDMASMK	RDQGN(65.29)VTDMASMK	5	2	743	1	1	1	1
IPI00649186	200	VGNETTYEDGHGARKNITDIVEGAKKANKII	1	100.037	119.39	100.04	_KN(de)ITDIVEGAKK_	KN(1)ITDIVEGAKK	KN(100.04)ITDIVEGAKK	2	3	440	1	1	1	1
IPI00346062	298	IQIAQNTQKNSQFDMNSTDIAIKVFAFDSTH	1	83.8333	119.54	119.54	_NSQFDM(ox)N(de)STDIAIK_	NSQFDMN(1)STDIAIK	N(-83.83)SQFDMN(83.83)STDIAI	7	2	801	1	1	1	1
IPI00114958	168	PDIEPVIKHSIEHFNNNTDHSHIFTIRKVKS	0.989429	19.6819	211.06	116.35	_HSIEHFNN(de)N(de)TDHSHIFTIR_	HSIEHFN(0.018)N(0.989)N(0.993)	HSIEHFN(-19.68)N(19.68)N(21.	8	3	774	1	1	1	1
IPI00133500	397	QWQGRQSQPVHIIPMNETDHINMVFSNKTIE	0.979766	14.806	56.327	56.327	_QSQPVHIIPM(ox)N(de)ETDHIN(de)M(ox)VFSNK_	QSQPVHIIPMN(0.98)ETDHIN(0.632)	QSQPVHIIPMN(14.81)ETDHIN(2.2	11	3	905	1	1	1	1
IPI00605187	138	GVTYTKEYEGAVYPDNTTDFQRADDKVIPGQ	1	99.344	190.19	99.344	_EYEGAVYPDN(de)TTDFQ(de)R_	EYEGAVYPDN(1)TTDFQR	EYEGAVYPDN(99.34)TTDFQR	10	2	954	1	1	1	1
IPI00119063	1576	TVSCACPHIMKIHKDNTTCYEFKKFIIYARQ	1	78.3239	106.62	78.324	_IHKDN(de)TTCYEFKK_	IHKDN(1)TTCYEFKK	IHKDN(78.32)TTCYEFKK	5	3	562	1	1	1	1
IPI00126186	529	YCYIIGSTISTFTDANHTCTNEKAYITTVED	0.999964	44.4647	243.23	243.23	_HGFYCYIIGSTISTFTDAN(de)HTCTNEK_	HGFYCYIIGSTISTFTDAN(1)HTCTNEK	HGFYCYIIGSTISTFTDAN(44.46)HT	19	3	1013	1	1	1	1
IPI00121190	568	SECIQCHPECIPQAMNITCTGRGPDNCIQCA	1	80.6652	175.86	175.86	_EFVENSECIQCHPECIPQAM(ox)N(de)ITCTGR_	EFVENSECIQCHPECIPQAMN(1)ITCTG	EFVEN(-80.67)SECIQCHPECIPQAM	21	3	1100	1	1	1	1
IPI00109996	280	FQKWASVVVPIGKEQNYTCRVYHEGIPEPIT	1	145.098	145.1	145.1	_WASVVVPIGKEQN(de)YTCR_	WASVVVPIGKEQN(1)YTCR	WASVVVPIGKEQN(145.1)YTCR	13	2	1005	1	1	1	1
IPI00475209	183	GYYCSCRFGYIIHTDNRTCRVECSGNIFTQR	1	87.298	119.53	87.298	_FGYIIHTDN(de)R_	FGYIIHTDN(1)R	FGYIIHTDN(87.3)R	9	2	619	1	1	1	1
IPI00320065	183	INADQGTSATVQMIINDTCPIFVRGIIEAGK	1	82.5604	121.25	82.56	_VIN(de)ADQGTSATVQM(ox)IIN(de)DTCPIFVR_	VIN(1)ADQGTSATVQMIIN(1)DTCPIF	VIN(82.56)ADQGTSATVQMIIN(82.	17	3	928	1	1	1	1
IPI00420810	69	ESFYTADSARKTTIENFTCPEYKIQNHYITE	1	90.7309	90.731	90.731	_KTTIEN(de)FTCPEYK_	KTTIEN(1)FTCPEYK	KTTIEN(90.73)FTCPEYK	6	3	545	1	1	1	1
IPI00929886	111	KDVIWFRPTIINDTGNYTCMIRNTTYCSKVA	1	92.3901	92.39	92.39	_DVIWFRPTIIN(de)DTGN(de)YTCM(ox)IR_	DVIWFRPTIIN(1)DTGN(1)YTCMIR	DVIWFRPTIIN(92.39)DTGN(92.39	15	3	868	1	1	1	1
IPI00850057	294	FQKWAAVVVPIGKEQNYTCHVHHKGIPEPIT	1	81.4275	153.95	81.428	_WAAVVVPIGKEQN(de)YTCHVHHK_	WAAVVVPIGKEQN(1)YTCHVHHK	WAAVVVPIGKEQN(81.43)YTCHVHHK	13	3	825	1	1	1	1
IPI00124830	109	GIASIKMDKRDAMVGNYTCEVTEISREGKTV	1	92.5753	197.15	92.575	_DAM(ox)VGN(de)YTCEVTEISR_	DAMVGN(1)YTCEVTEISR	DAMVGN(92.58)YTCEVTEISR	6	2	931	1	1	1	1
IPI00115116	150	IIISEKQCQEQIKEVNKTCEAIIFKIGEKVK	1	112.43	145.17	112.43	_EVN(de)KTCEAIIFK_	EVN(1)KTCEAIIFK	EVN(112.43)KTCEAIIFK	3	2	727	1	1	1	1
IPI00320675	106	QKSFECIHPEIKFSHNGTCAAEGKFNVSIIY	1	50.2074	110.38	50.207	_FSHN(de)GTCAAEGK_	FSHN(1)GTCAAEGK	FSHN(50.21)GTCAAEGK	4	2	640	1	1	1	1
IPI00551169	163	IKQKYEIRVDIEDFENNTAYAKYIDFSISPN	1	134.885	134.88	134.88	_VDIEDFEN(de)N(de)TAYAK_	VDIEDFEN(1)N(1)TAYAK	VDIEDFEN(134.88)N(134.88)TAY	8	2	816	1	1	1	1
IPI00405742	393	RDGIVATAVIHRGGINITAVTVTAENDHTVA	1	69.27	123.36	69.27	_GGIN(de)ITAVTVTAEN(de)DHTVAFIGTSDGR_	GGIN(1)ITAVTVTAEN(1)DHTVAFIGT	GGIN(69.27)ITAVTVTAEN(69.27)	4	3	907	1	1	1	1
IPI00608063	724	DVPSGPPRKVEVEAVNATAVKVSWRSPVPNK	1	101.888	118.08	101.89	_KVEVEAVN(de)ATAVK_	KVEVEAVN(1)ATAVK	KVEVEAVN(101.89)ATAVK	8	3	454	1	1	1	1
IPI00110264	721	DVPSGPPRKVEVEPINSTAVHVSWKIPVPNK	1	76.3411	166.48	76.341	_KVEVEPIN(de)STAVHVSWK_	KVEVEPIN(1)STAVHVSWK	KVEVEPIN(76.34)STAVHVSWK	8	3	642	1	1	1	1
IPI00134691	89	SFYTIRKFPVPFQKENVTATIVEIGRTAFNQ	1	118.48	230.96	118.48	_KFPVPFQ(de)KEN(de)VTATIVEIGR_	KFPVPFQKEN(1)VTATIVEIGR	KFPVPFQKEN(118.48)VTATIVEIGR	10	3	759	1	1	1	1
IPI00396840	165	NIWEHNQAVIKHIIENATASVSEAERKAQVY	1	64.1035	239.61	64.104	_HIIEN(de)ATASVSEAERK_	HIIEN(1)ATASVSEAERK	HIIEN(64.1)ATASVSEAERK	5	3	586	1	1	1	1
IPI00123996	150	SIRYEIFKRGPECSQNYTAPTGVIKSPGFPE	1	128.991	188.27	128.99	_RGPECSQN(de)YTAPTGVIK_	RGPECSQN(1)YTAPTGVIK	RGPECSQN(128.99)YTAPTGVIK	8	2	940	1	1	1	1
IPI00658539	345	IMRSGDKVRIDPPCTNTTAPSNYINNPYVRK	1	65.7636	161.08	127.97	_IDPPCTN(de)TTAPSN(de)YINNPYVR_	IDPPCTN(1)TTAPSN(0.955)YIN(0.	IDPPCTN(65.76)TTAPSN(14.08)Y	7	3	804	1	1	1	1
IPI00406901	424	EIIKGHAIGISCQSENGTAPITYHIMKAKSD	1	96.0124	199.41	96.012	_GHAIGISCQSEN(de)GTAPITYHIM(ox)K_	GHAIGISCQSEN(1)GTAPITYHIMK	GHAIGISCQSEN(96.01)GTAPITYHI	12	3	835	1	1	1	1
IPI00108098	127	KIKKEMGNTVMDIIRNYTANATSSREEAWDY	1	155.941	155.94	155.94	_N(de)YTAN(de)ATSSREEAWDYVQAQVK_	N(1)YTAN(1)ATSSREEAWDYVQAQVK	N(155.94)YTAN(155.94)ATSSREE	1	3	845	1	1	1	1
IPI00856723	168	DIQKDTAVEGEEIEVNCTAMASKPATTIRWF	1	102.212	147.9	102.21	_DTAVEGEEIEVN(de)CTAM(ox)ASKPATTIR_	DTAVEGEEIEVN(1)CTAMASKPATTIR	DTAVEGEEIEVN(102.21)CTAMASKP	12	2	1356	1	1	1	1
IPI00130661	209	SIHIGVTPSVIRQRYNITAKDVGSGTTNNSQ	1	135.711	138.4	135.71	_QRYN(de)ITAK_	QRYN(1)ITAK	QRYN(135.71)ITAK	4	2	498	1	1	1	1
IPI00120187	201	NQISRVPNNAIEGIENITAIYIHHNEIQEVG	0.99987	39.0339	132.24	132.24	_VPNNAIEGIEN(de)ITAIYIHHNEIQEVGSSM(ox)R_	VPNNAIEGIEN(1)ITAIYIHHNEIQEVG	VPN(-62.03)N(-53.79)AIEGIEN(	11	3	1123	1	1	1	1
IPI00754853	720	DVPSAPPRKVEAEAINATAIRVIWRSPTPGR	1	79.3371	79.337	79.337	_KVEAEAIN(de)ATAIR_	KVEAEAIN(1)ATAIR	KVEAEAIN(79.34)ATAIR	8	2	694	1	1	1	1
IPI00123196	206	FQGIKSISYIRISDTNITAIPQGIPTSITEV	1	96.5042	151.01	96.504	_ISDTN(de)ITAIPQ(de)GIPTSITEVHIDGN(de)KITK_	ISDTN(1)ITAIPQGIPTSITEVHIDGN(	ISDTN(96.5)ITAIPQGIPTSITEVHI	5	3	1027	1	1	1	1
IPI00110852	136	DASFRYPQDYQFYIQNFTAIPINTVVPPQRQ	1	83.2608	149.44	117.48	_YPQDYQFYIQN(de)FTAIPINTVVPPQR_	YPQDYQFYIQN(1)FTAIPINTVVPPQR	YPQDYQFYIQN(83.26)FTAIPIN(-8	11	3	1005	1	1	1	1
IPI00131143	427	IGKMYSEGSDIVPQSNETAIHYFKKAADMGN	1	139.083	139.08	139.08	_M(ox)YSEGSDIVPQSN(de)ETAIHYFKK_	MYSEGSDIVPQSN(1)ETAIHYFKK	MYSEGSDIVPQSN(139.08)ETAIHYF	13	3	854	1	1	1	1
IPI00108535	71	HNIPIAIGAFAWYKGNTTAIDKEIARFVPNS	1	94.4653	155.48	94.465	_GN(de)TTAIDKEIAR_	GN(1)TTAIDKEIAR	GN(94.47)TTAIDKEIAR	2	3	431	1	1	1	1
IPI00120155	43	CGYIYPEFPVVQRGSNFTAICVIKEACIQHY	1	156.362	156.36	156.36	_GSN(de)FTAICVIK_	GSN(1)FTAICVIK	GSN(156.36)FTAICVIK	3	2	606	1	1	1	1
IPI00224073	72	GAIQIPTVSFSHEESNTTAIAEFGEYIRKAF	1	45.9717	129.23	45.972	_GAIQIPTVSFSHEESN(de)TTAIAEFGEYIRK_	GAIQIPTVSFSHEESN(1)TTAIAEFGEY	GAIQIPTVSFSHEESN(45.97)TTAIA	16	4	800	1	1	1	1
IPI00310797	329	VANEFIKYRIHIGNYNGTAGDAIRFSRHYNH	0.999228	31.1204	104.95	104.95	_IHIGNYN(de)GTAGDAIR_	IHIGN(0.001)YN(0.999)GTAGDAIR	IHIGN(-31.12)YN(31.12)GTAGDA	7	2	787	1	1	1	1
IPI00308658	177	PIGPAEKIYVIDGTQNDTAFVFPRIRDFTIA	1	136.327	136.36	136.33	_IYVIDGTQN(de)DTAFVFPR_	IYVIDGTQN(1)DTAFVFPR	IYVIDGTQN(136.33)DTAFVFPR	9	3	653	1	1	1	1
IPI00648288	72	RPRARGAAAPSAPHWNETAEKTIGVIADEDN	1	57.1357	74.789	57.136	_GAAAPSAPHWN(de)ETAEK_	GAAAPSAPHWN(1)ETAEK	GAAAPSAPHWN(57.14)ETAEK	11	3	547	1	1	1	1
IPI00124725	580	QIIEKRKNAKGDEKENITAEAIDISIKYHFV	1	104.195	200.67	104.2	_GDEKEN(de)ITAEAIDISIK_	GDEKEN(1)ITAEAIDISIK	GDEKEN(104.2)ITAEAIDISIK	6	3	616	1	1	1	1
IPI00126864	164	RDNAIEISQIENRIINQTADMIQIASKYKDI	1	85.8373	102.29	85.837	_IIN(de)QTADM(ox)IQIASK_	IIN(1)QTADMIQIASK	IIN(85.84)QTADMIQIASK	3	3	522	1	1	1	1
IPI00115510	198	SGVVKIQIEFRPIDINSTAAGIPRIIIDTAG	1	57.1732	128.79	57.173	_IQIEFRPIDIN(de)STAAGIPR_	IQIEFRPIDIN(1)STAAGIPR	IQIEFRPIDIN(57.17)STAAGIPR	11	3	705	1	1	1	1
IPI00339885	211	EPRISIIATDHTYRRNFTAADWGHSRDAEEV	1	106.174	106.17	106.17	_RN(de)FTAADWGHSR_	RN(1)FTAADWGHSR	RN(106.17)FTAADWGHSR	2	2	710	1	1	1	1
IPI00381178	311	IIQKEGKDIITKKNVNISYTVNDSFFPQRPQ	1	71.7807	283.31	71.781	_N(de)VN(de)ISYTVN(de)DSFFPQRPQK_	N(1)VN(1)ISYTVN(1)DSFFPQRPQK	N(71.78)VN(71.78)ISYTVN(71.7	3	3	753	1	1	1	1
IPI00624663	1385	NFDKAGDHRTFQIRINVSYTGERPSSNMVIV	1	73.2937	83.758	73.294	_IN(de)VSYTGERPSSN(de)M(ox)VIVDVK_	IN(1)VSYTGERPSSN(1)MVIVDVK	IN(73.29)VSYTGERPSSN(73.29)M	2	2	1114	1	1	1	1
IPI00153316	70	KIIYEGGDIPDFRKENSSYQVINWRIPEDQQ	1	90.6136	188.91	90.614	_KEN(de)SSYQVIN(de)WR_	KEN(1)SSYQVIN(1)WR	KEN(90.61)SSYQVIN(90.61)WR	3	3	509	1	1	1	1
IPI00113042	352	IGGAAPPDKSWKGSINVSYNIGPGFTGSEYS	1	94.0013	300.74	300.74	_GSIN(de)VSYNIGPGFTGSEYSR_	GSIN(1)VSYNIGPGFTGSEYSR	GSIN(94)VSYN(-94)IGPGFTGSEYS	4	2	1053	1	1	1	1
IPI00120155	61	AICVIKEACIQHYYVNASYIVWKTNHAAVPR	1	136.347	136.35	136.35	_EACIQHYYVN(de)ASYIVWK_	EACIQHYYVN(1)ASYIVWK	EACIQHYYVN(136.35)ASYIVWK	10	2	1073	1	1	1	1
IPI00122557	311	IIQKEGKDIIKQKNVNISYIVNDSFFPQRPE	1	104.026	192.6	104.03	_QKN(de)VN(de)ISYIVN(de)DSFFPQRPEK_	QKN(1)VN(1)ISYIVN(1)DSFFPQRPE	QKN(104.03)VN(104.03)ISYIVN(	5	3	843	1	1	1	1
IPI00154056	92	WEIGQAIRQRYHGFINTSYHRQEVYVRSTDF	1	96.1135	96.113	96.113	_YHGFIN(de)TSYHR_	YHGFIN(1)TSYHR	YHGFIN(96.11)TSYHR	6	3	466	1	1	1	1
IPI00342158	405	AVIPAEFFEVISSSQNGSYHHIRAIQSGQTA	1	112.534	157.98	112.53	_IEAVIPAEFFEVISSSQN(de)GSYHHIR_	IEAVIPAEFFEVISSSQN(1)GSYHHIR	IEAVIPAEFFEVISSSQN(112.53)GS	18	3	944	1	1	1	1
IPI00132189	109	CSCIAINRNTQADVINASWSVISNSTRHEIE	1	75.6999	209.85	92.492	_NTQADVIN(de)ASWSVISN(de)STRHEIER_	NTQADVIN(1)ASWSVISN(1)STRHEIE	N(-63.73)TQADVIN(75.7)ASWSVI	8	3	910	1	1	1	1
IPI01008227	310	GIPTYRFTAPDTIFANGSVYPPNEGFCPCRE	1	63.2186	168.68	168.68	_FTAPDTIFAN(de)GSVYPPNEGFCPCR_	FTAPDTIFAN(1)GSVYPPNEGFCPCR	FTAPDTIFAN(63.22)GSVYPPN(-63	10	2	1360	1	1	1	1
IPI00416285	126	SKDVYVNIDMKGMNYNSSVVKNARECQERCT	0.999804	37.0693	104.79	104.79	_GM(ox)NYN(de)SSVVK_	GMNYN(1)SSVVK	GMN(-37.07)YN(37.07)SSVVK	5	2	558	1	1	1	1
IPI00409148	182	GKNQIVEIEKVVIHPNHSVVDIGIIKIKQRV	1	73.1564	259.96	73.156	_VVIHPN(de)HSVVDIGIIK_	VVIHPN(1)HSVVDIGIIK	VVIHPN(73.16)HSVVDIGIIK	6	4	436	1	1	1	1
IPI00120155	225	INFDPVDKVKPTPPYNISVTNSEEISSIIKI	1	171.474	171.47	171.47	_VKPTPPYN(de)ISVTN(de)SEEISSIIK_	VKPTPPYN(1)ISVTN(1)SEEISSIIK	VKPTPPYN(171.47)ISVTN(171.47	8	3	807	1	1	1	1
IPI00348586	9	________MRSPGAQDNVSVSQGMRAMFYMME	1	114.705	149	114.7	_SPGAQDN(de)VSVSQGM(ox)R_	SPGAQDN(1)VSVSQGMR	SPGAQDN(114.7)VSVSQGMR	7	3	517	1	1	1	1
IPI00128249	99	TIETTCHAIDPTPIANCSVRQITEHAVEGDC	1	48.3046	73.213	48.305	_RPFGVVYEM(ox)EVDTIETTCHAIDPTPIAN(de)CSVR_	RPFGVVYEMEVDTIETTCHAIDPTPIAN(	RPFGVVYEMEVDTIETTCHAIDPTPIAN	28	3	1232	1	1	1	1
IPI00381357	192	GCRGSEVEDEDIEIFNTSVQIRPPSTAPGPE	1	76.3105	178.95	76.31	_GSEVEDEDIEIFN(de)TSVQIRPPSTAPGPETAAFIER_	GSEVEDEDIEIFN(1)TSVQIRPPSTAPG	GSEVEDEDIEIFN(76.31)TSVQIRPP	13	3	1268	1	1	1	1
IPI01023131	302	NPWMDIREREDVQAINISVPYGPIPEDFQRK	1	111.449	111.45	111.45	_EREDVQAIN(de)ISVPYGPIPEDFQR_	EREDVQAIN(1)ISVPYGPIPEDFQR	EREDVQAIN(111.45)ISVPYGPIPED	9	3	892	1	1	1	1
IPI00420835	927	SRKFSIFPERKYQTINCSVNVRCVNIRCPIR	1	72.8375	124.12	124.12	_KYQTIN(de)CSVNVR_	KYQTIN(1)CSVNVR	KYQTIN(72.84)CSVN(-72.84)VR	6	3	495	1	1	1	1
IPI00132600	969	QSSCCRIYNVTHQFCNASVMDPTCVRCRPIT	1	167.281	235.95	167.28	_IYN(de)VTHQFCN(de)ASVM(ox)DPTCVR_	IYN(1)VTHQFCN(1)ASVMDPTCVR	IYN(167.28)VTHQFCN(167.28)AS	10	3	811	1	1	1	1
IPI00515360	358	RCDGDFDCEDRTDEANCSVKQPGEVCGPTHF	1	58.6706	133.71	58.671	_CDGDFDCEDRTDEAN(de)CSVK_	CDGDFDCEDRTDEAN(1)CSVK	CDGDFDCEDRTDEAN(58.67)CSVK	15	3	765	1	1	1	1
IPI01008227	383	HSIFIDIHPVTGIPMNCSVKMQISIYIKSVK	1	59.8998	139.88	59.9	_EHSIFIDIHPVTGIPM(ox)N(de)CSVK_	EHSIFIDIHPVTGIPMN(1)CSVK	EHSIFIDIHPVTGIPMN(59.9)CSVK	17	3	804	1	1	1	1
IPI00133222	230	EDFVITTAKCSIIHSNISVKANVDQRIRIKS	1	79.8202	127.87	79.82	_CSIIHSN(de)ISVK_	CSIIHSN(1)ISVK	CSIIHSN(79.82)ISVK	7	2	630	1	1	1	1
IPI00121114	323	DSISDKPRVSTIYANNGSVIQGSTVASVYHK	1	60.0318	295.69	60.032	_VSTIYAN(de)N(de)GSVIQGSTVASVYHKR_	VSTIYAN(1)N(1)GSVIQGSTVASVYHK	VSTIYAN(60.03)N(60.03)GSVIQG	8	3	852	1	1	1	1
IPI00121776	119	ARIAQEGGAAAIIIANNSVIIPSSRNKSTFQ	1	70.4517	129.78	70.452	_IAQEGGAAAIIIAN(de)N(de)SVIIPSSR_	IAQEGGAAAIIIAN(1)N(1)SVIIPSSR	IAQEGGAAAIIIAN(70.45)N(70.45	14	3	756	1	1	1	1
IPI00342158	1135	AAVSSSGIVRGIMVGNGSVIGVVQAVDAETG	1	91.6396	250.05	91.64	_GIM(ox)VGN(de)GSVIGVVQAVDAETGK_	GIMVGN(1)GSVIGVVQAVDAETGK	GIMVGN(91.64)GSVIGVVQAVDAETG	6	3	707	1	1	1	1
IPI00987265	1179	YTIFVPTNHSIETQGNNSVIGIDTVRHHVII	1	68.4451	144.63	68.445	_AYTIFVPTN(de)HSIETQ(de)GN(de)N(de)SVIGIDTVR_	AYTIFVPTN(1)HSIETQGN(1)N(1)SV	AYTIFVPTN(68.45)HSIETQGN(68.	17	3	984	1	1	1	1
IPI00127447	122	TTISAVTNKAYVFERNQSVGDPNVDIIRTIN	1	83.1055	182.35	83.106	_AYVFERN(de)QSVGDPN(de)VDIIR_	AYVFERN(1)QSVGDPN(1)VDIIR	AYVFERN(83.11)QSVGDPN(83.11)	7	3	732	1	1	1	1
IPI00116913	3291	GPQRVFDIHQNMGSVNVSVGCTPAQIIETSR	1	132.36	132.36	132.36	_VFDIHQN(de)M(ox)GSVN(de)VSVGCTPAQIIETSR_	VFDIHQN(1)MGSVN(1)VSVGCTPAQII	VFDIHQN(132.36)MGSVN(132.36)	12	3	993	1	1	1	1
IPI00126769	356	VQTCNFSAQMAKVYINDSVEISRNENKIAAW	1	68.3555	139.6	68.355	_VYIN(de)DSVEISR_	VYIN(1)DSVEISR	VYIN(68.36)DSVEISR	4	2	648	1	1	1	1
IPI00404189	231	EKGVIPGEDWTVFQSNHSTYEPVIIAKTRSS	1	48.6557	77.631	48.656	_GVIPGEDWTVFQSN(de)HSTYEPVIIAK_	GVIPGEDWTVFQSN(1)HSTYEPVIIAK	GVIPGEDWTVFQSN(48.66)HSTYEPV	14	3	930	1	1	1	1
IPI00122117	163	RKEQETCIAPEIEHGNYSTTQRTFKVKDIVA	1	101.382	145.02	101.38	_KEQETCIAPEIEHGN(de)YSTTQR_	KEQETCIAPEIEHGN(1)YSTTQR	KEQETCIAPEIEHGN(101.38)YSTTQ	15	3	831	1	1	1	1
IPI00309035	300	IPAAAQDVYYRDEIGNVSTSHIIIIDDSVEM	1	51.2356	95.5	51.236	_TIIPAAAQ(de)DVYYRDEIGN(de)VSTSHIIIIDDSVEM(ox)EIRPR_	TIIPAAAQDVYYRDEIGN(1)VSTSHIII	TIIPAAAQDVYYRDEIGN(51.24)VST	18	4	1080	1	1	1	1
IPI00380296	241	NEKIDAYNEAAVSIINSSTRTSKSNVKMFSV	1	78.6519	147.06	147.06	_IDAYNEAAVSIIN(de)SSTR_	IDAYNEAAVSIIN(1)SSTR	IDAYN(-78.65)EAAVSIIN(78.65)	13	2	913	1	1	1	1

IPI00129485	75	EDTVTITCEGTHNPGNSSTQWFHNGRSIRSQ	1	66.1369	199.99	66.137 _EDTVTITCEGTHN(de)PGN(de)SSTQWFHN(de)GR_	EDTVTITCEGTHN(1)PGN(1)SSTQWFHN	EDTVTITCEGTHN(66.14)PGN(66.1	16	3	983	1	1	1	1
IPI00122257	335	ATIKADINQWRIKIDNYSTQEIGRTIVYIDG	1	90.7928	190.85	90.793 _IKIDN(de)YSTQEIGR_	IKIDN(1)YSTQEIGR	IKIDN(90.79)YSTQEIGR	5	3	513	1	1	1	1
IPI01023131	137	TRIYDFNSYWRVHSGNFSTIPQYFKENGYVT	1	54.0231	69.345	54.023 _VHSGN(de)FSTIPQYFK_	VHSGN(1)FSTIPQYFK	VHSGN(54.02)FSTIPQYFK	5	3	543	1	1	1	1
IPI00124666	55	YEEISQIRHFKGQTQNYSTIIIEEASERIIV	1	110.769	252.05	110.77 _HFKGQTQN(de)YSTIIIEEASER_	HFKGQTQN(1)YSTIIIEEASER	HFKGQTQN(110.77)YSTIIIEEASER	8	2	1177	1	1	1	1
IPI00173181	184	SPEVVREIIVDEIISNSSTIASYKTEYEVDP	1	54.0044	181.34	54.004 _EIIVDEIISN(de)SSTIASYK_	EIIVDEIISN(1)SSTIASYK	EIIVDEIISN(54)SSTIASYK	10	3	662	1	1	1	1
IPI01023214	290	IINISRKSDTQNIIYNVSTGRAMVAQSTFYA	0.999999	62.6333	153.39	153.39 _KSDTQNIIYN(de)VSTGR_	KSDTQNIIYN(1)VSTGR	KSDTQN(-62.63)IIYN(62.63)VST	10	3	566	1	1	1	1
IPI00322497	250	IKFRFIRNGIASGSGNCSTGPNGDGSFHAWS	1	166.653	166.65	166.65 _N(de)GIASGSGN(de)CSTGPN(de)GDGSFHAWSIIEVK_	N(1)GIASGSGN(1)CSTGPN(1)GDGSFH	N(166.65)GIASGSGN(166.65)CST	9	3	975	1	1	1	1
IPI00322575	71	FSSIPDVDIGRIDSFNDSTFMIVYTPITPTT	1	150.909	150.91	150.91 _IDSFN(de)DSTFM(ox)IVYTPITPTTQR_	IDSFN(1)DSTFMIVYTPITPTTQR	IDSFN(150.91)DSTFMIVYTPITPTT	5	2	1283	1	1	1	1
IPI01026704	317	IQRFSIAIIGHYNRGNISTEKFVEEIKSIAS	1	68.8302	88.021	68.83 _GN(de)ISTEKFVEEIK_	GN(1)ISTEKFVEEIK	GN(68.83)ISTEKFVEEIK	2	2	748	1	1	1	1
IPI01008227	330	PPNEGFCPCRESGIQNVSTCRFGAPIFISHP	1	103.909	107.97	103.91 _ESGIQN(de)VSTCR_	ESGIQN(1)VSTCR	ESGIQN(103.91)VSTCR	6	2	626	1	1	1	1
IPI00123428	85	GVGRDNVSQPKEGPRNISTCFSSGDIFAAHN	1	84.3274	185.71	84.327 _EGPRN(de)ISTCFSSGDIFAAHN(de)ISER_	EGPRN(1)ISTCFSSGDIFAAHN(1)ISEF	EGPRN(84.33)ISTCFSSGDIFAAHN(	5	3	890	1	1	1	1
IPI00654069	43	AGDRVYIHPFHIIYHNKSTCAQIENPSVETI	1	112.129	112.13	112.13 _VYIHPFHIIYHN(de)K_	VYIHPFHIIYHN(1)K	VYIHPFHIIYHN(112.13)K	12	3	561	1	1	1	1
IPI00127100	102	TSGGPVRVYSIPGRENYSSVDANGIQSQMIS	1	111.377	149.85	111.38 _VYSIPGREN(de)YSSVDAN(de)GIQSQM(ox)ISR_	VYSIPGREN(1)YSSVDAN(1)GIQSQMIS	VYSIPGREN(111.38)YSSVDAN(111	9	3	930	1	1	1	1
IPI00399798	395	MEIDWAVTAGAKISENGSSVAGIISSPNMEK	1	93.6706	164.94	115.87 _ISEN(de)GSSVAGIISSPNM(ox)EK_	ISEN(1)GSSVAGIISSPNMEK	ISEN(93.67)GSSVAGIISSPN(-93.	4	3	646	1	1	1	1
IPI00131898	442	PMPDSCQWPEKMRFINISSTGIRVVKTCIPQ	1	93.0578	93.058	93.058 _FIN(de)ISSTGIR_	FIN(1)ISSTGIR	FIN(93.06)ISSTGIR	3	2	555	1	1	1	1
IPI00111960	140	PWCFFPPSYPSYRIENISSTESGYTATITRT	1	53.5041	271.64	53.504 _IEN(de)ISSTESGYTATITR_	IEN(1)ISSTESGYTATITR	IEN(53.5)ISSTESGYTATITR	3	2	922	1	1	1	1
IPI00338561	197	IETDMQIMVRIINKFNSSSSSIEEKVAAIFD	1	87.5658	87.566	87.566 _IIN(de)KFN(de)SSSSSIEEK_	IIN(1)KFN(1)SSSSSIEEK	IIN(87.57)KFN(87.57)SSSSSIEE	6	2	843	1	1	1	1
IPI00856723	104	FRDFRPIKDSRFQIINFSSSEIKVSITNVSI	1	136.299	136.3	136.3 _FQIIN(de)FSSSEIK_	FQIIN(1)FSSSEIK	FQIIN(136.3)FSSSEIK	5	2	707	1	1	1	1
IPI00137792	333	ESTPVNGFPPPFIKGNQSSQWIYEAMAKAWE	1	148.027	148.03	148.03 _GN(de)QSSQWIYEAM(ox)AK_	GN(1)QSSQWIYEAMAK	GN(148.03)QSSQWIYEAMAK	2	2	815	1	1	1	1
IPI00876541	819	KSAINSWEKIIQQQSNQSSQFIHSVERFSQA	1	48.6226	111.34	48.623 _IIQQQSN(de)QSSQFIHSVER_	IIQQQSN(1)QSSQFIHSVER	IIQQQSN(48.62)QSSQFIHSVER	7	3	711	1	1	1	1
IPI00677395	160	QAFPSINNKEITGIRNISSQEGSTDVVARTQ	1	60.9487	226.57	60.949 _EITGIRN(de)ISSQEGSTDVVAR_	EITGIRN(1)ISSQEGSTDVVAR	EITGIRN(60.95)ISSQEGSTDVVAR	7	3	712	1	1	1	1
IPI00625835	124	IMGRYKYETTISPTSNISSNSFICIENRTQT	1	135.211	167.51	135.21 _YKYETTISPTSN(de)ISSN(de)SFICIEN(de)R_	YKYETTISPTSN(1)ISSN(1)SFICIEN	YKYETTISPTSN(135.21)ISSN(135	12	3	943	1	1	1	1
IPI00405742	451	VDINKKIKQDIAISGNISSIYAMTQDKVFRI	1	282.718	282.72	282.72 _QDIAISGN(de)ISSIYAM(ox)TQDK_	QDIAISGN(1)ISSIYAMTQDK	QDIAISGN(282.72)ISSIYAMTQDK	8	2	1036	1	1	1	1
IPI00330594	132	SSCHKEVQIIKYQMDNVSSIVQIIGSHIEDV	1	59.3587	176.9	59.359 _YQM(ox)DN(de)VSSIVQIIGSHIEDVN(de)ADIIQTK_	YQMDN(1)VSSIVQIIGSHIEDVN(1)ADI	YQMDN(59.36)VSSIVQIIGSHIEDVN	5	3	1050	1	1	1	1
IPI00115976	715	APIEAEYSGIVRHPGNFSSISCDYFAVNQSR	1	77.8708	126.07	77.871 _HPGN(de)FSSISCDYFAVN(de)QSR_	HPGN(1)FSSISCDYFAVN(1)QSR	HPGN(77.87)FSSISCDYFAVN(77.8	4	2	1094	1	1	1	1
IPI00762812	814	HPIDIQEDFNNATIVNASSIIPKEAGSKETF	1	82.5933	82.593	82.593 _M(ox)SQHPIDIQEDFN(de)N(de)ATIVN(de)ASSIIPK_	MSQHPIDIQEDFN(1)N(1)ATIVN(1)AS	MSQHPIDIQEDFN(82.59)N(82.59)	19	3	968	1	1	1	1
IPI00387282	281	NQHVPMESSHIIQFVNWSSIIPERYKKSPVY	1	55.994	153.24	55.994 _AM(ox)IIN(de)QHVPM(ox)ESSHIIQFVN(de)WSSIIPERYKK_	AMIIN(1)QHVPMESSHIIQFVN(1)WSS	AMIIN(55.99)QHVPMESSHIIQFVN(	20	4	933	1	1	1	1
IPI00123342	830	RRKWPERISAIDNIINHSSIFIKGARIIPEM	1	82.6513	227	82.651 _ISAIDN(de)IIN(de)HSSIFIK_	ISAIDN(1)IIN(1)HSSIFIK	ISAIDN(82.65)IIN(82.65)HSSIF	9	3	596	1	1	1	1
IPI00119522	119	ITGSPVSNISAHIFSNISSIEKITIDFDRIA	1	129.082	135.3	129.08 _IQ(de)DIEITGSPVSN(de)ISAHIFSN(de)ISSIEK_	IQDIEITGSPVSN(1)ISAHIFSN(1)ISS	IQDIEITGSPVSN(129.08)ISAHIFS	21	3	968	1	1	1	1
IPI00137987	123	GSHTFQGMFGCEITNNRSSGAVWRYAYDGED	1	54.3685	162.77	54.368 _DTTGSHTFQGM(ox)FGCEITN(de)N(de)R_	DTTGSHTFQGMFGCEITN(1)N(1)R	DTTGSHTFQGMFGCEITN(54.37)N(5	19	3	764	1	1	1	1
IPI00108844	84	SDTYSYIFRVCREASNHSSGAGIVQINKSND	1	91.6567	182.15	91.657 _EASN(de)HSSGAGIVQIN(de)K_	EASN(1)HSSGAGIVQIN(1)K	EASN(91.66)HSSGAGIVQIN(91.66	4	2	807	1	1	1	1
IPI00315535	390	RCVRRNPSANTFIHINASSFRIVPGHTPSEP	1	67.5869	151.44	117.67 _NPSANTFIHIN(de)ASSFR_	NPSANTFIHIN(1)ASSFR	N(-95.83)PSAN(-67.59)TFIHIN(	11	2	889	1	1	1	1
IPI00742377	287	SDAQPHIQWIKHVVINGSSFGADGFPYVQVI	1	90.3161	119.8	90.316 _HVVIN(de)GSSFGADGFPYVQVIK_	HVVIN(1)GSSFGADGFPYVQVIK	HVVIN(90.32)GSSFGADGFPYVQVIK	5	2	1118	1	1	1	1
IPI00122973	362	GVEPRPPTPQVQFTINASSEDHKRSFFCSAA	1	53.4444	70.994	53.444 _VVIISGVEPRPPTPQVQFTIN(de)ASSEDHKR_	VVIISGVEPRPPTPQVQFTIN(1)ASSEDH	VVIISGVEPRPPTPQVQFTIN(53.44)	21	5	641	1	1	1	1
IPI00120769	48	MTATKYFTNRIDVSQNVSSDTDQSCESTKAI	1	120.106	120.11	120.11 _IDVSQN(de)VSSDTDQSCESTK_	IDVSQN(1)VSSDTDQSCESTK	IDVSQN(120.11)VSSDTDQSCESTK	6	2	1051	1	1	1	1
IPI00330594	115	TEMQVAIQRIRDYEENSSSCHKEVQIIKYQM	1	88.9922	94.122	88.992 _IRDYEEN(de)SSSCHKEVQIIK_	IRDYEEN(1)SSSCHKEVQIIK	IRDYEEN(88.99)SSSCHKEVQIIK	7	3	779	1	1	1	1
IPI00129041	57	IAGTPEHRCIVPHTVNISSAWRNHSIPIETK	1	77.6441	129.77	77.644 _CIVPHTVN(de)ISSAWR_	CIVPHTVN(1)ISSAWR	CIVPHTVN(77.64)ISSAWR	8	3	548	1	1	1	1
IPI00123342	155	PQIQFSPEEVIGMVINYSRSIAEDFAEQPIK	1	232.541	244.91	232.54 _FQISPQIQFSPEEVIGMVIN(de)YSR_	FQISPQIQFSPEEVIGMVIN(1)YSR	FQISPQIQFSPEEVIGMVIN(232.54)	20	3	895	1	1	1	1
IPI00463492	292	ARVIENEKFDTHEYHNESRRIQEFIRAQEPG	1	80.3353	142.39	142.39 _VIENEKFDTHEYHN(de)ESR_	VIENEKFDTHEYHN(1)ESR	VIEN(-80.34)EKFDTHEYHN(80.34	14	2	1074	1	1	1	1
IPI00405742	530	SREKSCVAITDAFPQNMSRRAQGEVRISVSP	1	61.1674	151.44	61.167 _SCVAITDAFPQN(de)M(ox)SRR_	SCVAITDAFPQN(1)MSRR	SCVAITDAFPQN(61.17)MSRR	12	3	624	1	1	1	1
IPI00115976	727	HPGNFSSISCDYFAVNQSRQIVCDIGNPMKA	1	77.8708	126.07	77.871 _HPGN(de)FSSISCDYFAVN(de)QSR_	HPGN(1)FSSISCDYFAVN(1)QSR	HPGN(77.87)FSSISCDYFAVN(77.8	16	2	1094	1	1	1	1
IPI00113517	192	VGCIPYTIPPCEHHVNGSRPPCTGEGDTPRC	1	44.9151	60.645	44.915 _KGIVSGGVYN(de)SHVGCIPYTIPPCEHHVN(de)GSRPPCTGEGDTPR_	KGIVSGGVYN(1)SHVGCIPYTIPPCEHH	KGIVSGGVYN(44.92)SHVGCIPYTIP	28	5	913	1	1	1	1
IPI00226790	331	RKTYAVYDIFDTAMINNSRNINIQIKWKRPP	1	170.113	188.41	170.11 _KTYAVYDIFDTAM(ox)IN(de)N(de)SR_	KTYAVYDIFDTAMIN(1)N(1)SR	KTYAVYDIFDTAMIN(170.11)N(170	15	3	714	1	1	1	1
IPI00121908	75	KIVPCEFGVIYPIAENFSRIINKEESIIQRI	1	73.296	145.46	73.296 _IVPCEFGVIYPIAEN(de)FSR_	IVPCEFGVIYPIAEN(1)FSR	IVPCEFGVIYPIAEN(73.3)FSR	15	3	705	1	1	1	1
IPI00222429	610	EFHQDGNGPENVGIYNISRGVNRFCISKPGV	1	134.465	134.47	134.47 _CAISHAITIEFHQDGN(de)GPEN(de)VGIYN(de)ISR_	CAISHAITIEFHQDGN(1)GPEN(1)VGI	CAISHAITIEFHQDGN(134.47)GPEN	25	3	1039	1	1	1	1
IPI00121190	528	EGCWGPEPRDCVSCQNVSRGRECVEKCNIIE	1	94.7673	99.752	94.767 _DCVSCQN(de)VSR_	DCVSCQN(1)VSR	DCVSCQN(94.77)VSR	7	2	613	1	1	1	1
IPI00119065	1500	AEEAQQRAQAAIDKANASRGQVEQANQEIRE	1	94.0232	94.023	94.023 _AQAAIDKAN(de)ASR_	AQAAIDKAN(1)ASR	AQAAIDKAN(94.02)ASR	9	2	609	1	1	1	1
IPI00115516	456	PAAPGRIEKIEGIIANVSREIGGRMDIIEEQ	1	96.8656	136.17	96.866 _IEKIEGIIAN(de)VSR_	IEKIEGIIAN(1)VSR	IEKIEGIIAN(96.87)VSR	10	3	482	1	1	1	1
IPI00469123	58	GSTAIIKCGPSRASGNFSQVDWFIIHKERQI	1	72.8979	178.14	72.898 _ASGN(de)FSQVDWFIIHKER_	ASGN(1)FSQVDWFIIHKER	ASGN(72.9)FSQVDWFIIHKER	4	3	679	1	1	1	1
IPI00122977	243	QIFHSPDIAIRDTYVNASQSIYGSSPRVIGP	1	56.5333	202.18	56.533 _GVTSVSQIFHSPDIAIRDTYVN(de)ASQSIYGSSPR_	GVTSVSQIFHSPDIAIRDTYVN(1)ASQS	GVTSVSQIFHSPDIAIRDTYVN(56.53	22	4	889	1	1	1	1
IPI00114958	242	ECRGNIFMDINNKIANFSQSCTIYSGDDIVE	1	42.8061	156.41	42.806 _IAN(de)FSQSCTIYSGDDIVEAIPKPCPGCPR_	IAN(1)FSQSCTIYSGDDIVEAIPKPCPG	IAN(42.81)FSQSCTIYSGDDIVEAIP	3	3	1085	1	1	1	1
IPI00123428	75	IKSIIDHIHVGVGRDNVSQPKEGPRNISTCF	1	59.8622	161.35	59.862 _SIIDHIHVGVGRDN(de)VSQPK_	SIIDHIHVGVGRDN(1)VSQPK	SIIDHIHVGVGRDN(59.86)VSQPK	14	3	691	1	1	1	1
IPI00129526	62	EVVQREEEAIQIDGINASQIREIREKSEKFA	1	185.873	204.47	185.87 _TDDEVVQREEEAIQIDGIN(de)ASQIR_	TDDEVVQREEEAIQIDGIN(1)ASQIR	TDDEVVQREEEAIQIDGIN(185.87)A	19	3	910	1	1	1	1
IPI00471089	75	VVSSVTTKAKGVAVTNTSQIGFRIWDVADYV	1	124.302	233.59	124.3 _GVAVTN(de)TSQIGFR_	GVAVTN(1)TSQIGFR	GVAVTN(124.3)TSQIGFR	6	2	676	1	1	1	1
IPI00134585	754	IIGFACKMGDREAIGNASQIFDSWIKGSASI	1	76.5863	198.95	76.586 _M(ox)GDREAIGN(de)ASQIFDSWIK_	MGDREAIGN(1)ASQIFDSWIK	MGDREAIGN(76.59)ASQIFDSWIK	9	3	719	1	1	1	1
IPI00114044	93	NISESVEDGPRGSPGNASQGSIHIHSPQIAI	1	42.3222	100.28	42.322 _GSPGN(de)ASQGSIHIHSPQIAIQADPR_	GSPGN(1)ASQGSIHIHSPQIAIQADPR	GSPGN(42.32)ASQGSIHIHSPQIAIQ	5	4	636	1	1	1	1
IPI00320675	514	QKVYSIRWGEVDIIGNCSQFYPDRYYEKEMQ	1	62.2028	154.49	62.203 _WGEVDIIGN(de)CSQFYPDRYYEK_	WGEVDIIGN(1)CSQFYPDRYYEK	WGEVDIIGN(62.2)CSQFYPDRYYEK	9	3	881	1	1	1	1
IPI00119522	520	QISSRDGSDSAAMVYNSSQEWGIRSSCGIIR	1	296.733	296.73	296.73 _DGSDSAAM(ox)VYN(de)SSQEWGIR_	DGSDSAAMVYN(1)SSQEWGIR	DGSDSAAMVYN(296.73)SSQEWGIR	11	2	1045	1	1	1	1
IPI00322304	123	EIVIGQCKVIATRYSNESQDISVNGYNCTTS	1	120.33	120.33	120.33 _YSN(de)ESQDISVN(de)GYN(de)CTTSSVSSAIR_	YSN(1)ESQDISVN(1)GYN(1)CTTSSVS	YSN(120.33)ESQDISVN(120.33)G	3	3	915	1	1	1	1
IPI00462465	180	IEPKVFKPQSGADAINDSQDFPFPETPAKVW	1	68.2915	192.27	68.291 _VFKPQSGADAIN(de)DSQDFPFPETPAK_	VFKPQSGADAIN(1)DSQDFPFPETPAK	VFKPQSGADAIN(68.29)DSQDFPFPE	12	4	678	1	1	1	1
IPI00987265	1627	DGPFTVFVPHADIISNMSQDEIARIRAHRQI	1	158.516	158.52	158.52 _GDGPFTVFVPHADIISN(de)M(ox)SQDEIAR_	GDGPFTVFVPHADIISN(1)MSQDEIAR	GDGPFTVFVPHADIISN(158.52)MSQ	17	3	912	1	1	1	1
IPI00131143	268	GQTGIGFIYASGIGVNSSQAKAIVYYTFGAI	1	150.984	196.78	150.98 _GQTGIGFIYASGIGVN(de)SSQAK_	GQTGIGFIYASGIGVN(1)SSQAK	GQTGIGFIYASGIGVN(150.98)SSQA	16	3	686	1	1	1	1
IPI00130661	221	QRYNITAKDVGSGTTNNSQACAQFIEQYFHN	1	69.4348	205.13	69.435 _DVGSGTTN(de)N(de)SQACAQFIEQYFHN(de)SDITEFM(ox)R_	DVGSGTTN(1)N(1)SQACAQFIEQYFHN	DVGSGTTN(69.43)N(69.43)SQACA	8	3	1196	1	1	1	1
IPI00108844	114	DKETVVGRINETHIFNGSNWIMIIYKGGDEY	0.999963	44.2704	162.46	162.46 _IN(de)ETHIFN(de)GSNWIM(ox)IIYK_	IN(1)ETHIFN(1)GSNWIMIIYK	IN(53.57)ETHIFN(44.27)GSN(-4	8	3	738	1	1	1	1
IPI00119063	3090	TDVTTQGSMIRRMHINGSNVQVIHRTGISNP	1	69.8636	192.14	69.864 _M(ox)HIN(de)GSN(de)VQVIHR_	MHIN(1)GSN(1)VQVIHR	MHIN(69.86)GSN(69.86)VQVIHR	4	3	508	1	1	1	1
IPI00605187	757	PSRAWEKEIHHIQEQNVSNVFIDKEEFFIGS	1	62.9993	186.82	62.999 _EIHHIQEQN(de)VSN(de)VFIDKEEFFIGSK_	EIHHIQEQN(1)VSN(1)VFIDKEEFFIGS	EIHHIQEQN(63)VSN(63)VFIDKEEF	9	3	997	1	1	1	1
IPI00114319	121	RIEAYFSIEGFPAEQNASNRAIHVHEFGDIS	1	95.2051	201.37	95.205 _IEAYFSIEGFPAEQN(de)ASN(de)R_	IEAYFSIEGFPAEQN(1)ASN(1)R	IEAYFSIEGFPAEQN(95.21)ASN(95	15	2	1073	1	1	1	1
IPI00421157	318	SFIWQRGRKFIQKHINASNPSEPATIIFTAA	0.999996	53.9793	240.13	107.02 _HIN(de)ASNPSEPATIIFTAAR_	HIN(1)ASNPSEPATIIFTAAR	HIN(53.98)ASN(-53.98)PSEPATI	3	2	1006	1	1	1	1
IPI00114364	37	DPGSKVVADRYAVYWNSSNPRFQRGDYHIDV	0.999947	42.7281	122.42	122.42 _YAVYWN(de)SSNPR_	YAVYWN(1)SSNPR	YAVYWN(42.73)SSN(-42.73)PR	6	2	679	1	1	1	1
IPI00130752	26	ICCSIAAADRHIVFWNSSNPKFREEDYTVHV	0.997263	25.6157	111.63	111.63 _HIVFWN(de)SSNPK_	HIVFWN(0.997)SSN(0.003)PK	HIVFWN(25.62)SSN(-25.62)PK	6	2	665	1	1	1	1
IPI00118173	592	FPIGGATPRYIYIASNHSNKWGHRRGYRIQI	0.999838	37.9013	98.156	98.156 _YIYIASN(de)HSNK_	YIYIASN(1)HSNK	YIYIASN(37.9)HSN(-37.9)K	7	2	656	1	1	1	1
IPI00129677	75	SQNSQIREDIIAIRQNFSNITVSTEDQVKAI	1	48.8985	216.71	48.899 _QN(de)FSN(de)ITVSTEDQVK_	QN(1)FSN(1)ITVSTEDQVK	QN(48.9)FSN(48.9)ITVSTEDQVK	2	2	856	1	1	1	1
IPI00221418	415	AGISVDVKVFIVPVGNHSNIPFSRVNHSKFM	1	61.6572	161.82	61.657 _VFIVPVGN(de)HSN(de)IPFSR_	VFIVPVGN(1)HSN(1)IPFSR	VFIVPVGN(61.66)HSN(61.66)IPF	8	3	596	1	1	1	1
IPI00221418	247	WRSITQVKEIGAIIYNCSNIAQDIEKTFQTY	0.999992	50.9029	202.34	202.34 _EIGAIIYN(de)CSNIAQDIEK_	EIGAIIYN(1)CSNIAQDIEK	EIGAIIYN(50.9)CSN(-50.9)IAQD	8	3	685	1	1	1	1
IPI00129158	246	VAHITIDRSPIRGIANISNFIRVSPTVKVTQ	1	117.673	183.03	117.67 _GIAN(de)ISN(de)FIR_	GIAN(1)ISN(1)FIR	GIAN(117.67)ISN(117.67)FIR	4	2	554	1	1	1	1
IPI00108844	95	REASNHSSGAGIVQINKSNDKETVVGRINET	1	91.6567	158.81	91.657 _EASN(de)HSSGAGIVQIN(de)K_	EASN(1)HSSGAGIVQIN(1)K	EASN(91.66)HSSGAGIVQIN(91.66	15	2	807	1	1	1	1
IPI00380296	141	KAASVKVDFIWHPEVNGSMKQCIKVWTEDSV	1	69.716	69.716	69.716 _VDFIWHPEVN(de)GSM(ox)K_	VDFIWHPEVN(1)GSMK	VDFIWHPEVN(69.72)GSMK	10	3	559	1	1	1	1
IPI00312711	81	EATFQVEIPRKAFITNFSMIIDGVTYPGVVK	1	152.125	152.13	152.13 _KAFITN(de)FSM(ox)IIDGVTYPGVVKEK_	KAFITN(1)FSMIIDGVTYPGVVKEK	KAFITN(152.13)FSMIIDGVTYPGVV	6	3	859	1	1	1	1
IPI00136942	146	KFSVEAIRCHIRDHVNVSMIEATDFPFNTTE	1	58.3237	155.25	58.324 _DHVN(de)VSM(ox)IEATDFPFN(de)TTEWEGYIPK_	DHVN(1)VSMIEATDFPFN(1)TTEWEGYI	DHVN(58.32)VSMIEATDFPFN(58.3	4	3	1020	1	1	1	1
IPI00230412	270	WARAMGVNVIAANTHNTSMHMTGSGIYSPEA	0.998603	28.5573	122.51	122.51 _AM(ox)GVNVIAANTHN(de)TSM(ox)HM(ox)TGSGIYSPEAVR_	AMGVNVIAAN(0.001)THN(0.999)TSM	AMGVN(-53.04)VIAAN(-28.56)TH	13	4	792	1	1	1	1
IPI00121190	175	DTIQWRDIVQNVFMSNMSMDIQSHPSSCPKC	0.999996	53.7504	124.2	124.2 _DIVQNVFM(ox)SN(de)M(ox)SM(ox)DIQSHPSSCPK_	DIVQNVFMSN(1)MSMDIQSHPSSCPK	DIVQN(-53.75)VFMSN(53.75)MSM	10	3	934	1	1	1	1
IPI00224752	1042	PSQASAGNVYPQPIINSSMCIEDSRYNWSFI	1	69.3654	146.25	146.25 _M(ox)PSQASAGNVYPQPIIN(de)SSM(ox)CIEDSR_	MPSQASAGNVYPQPIIN(1)SSMCIEDSR	MPSQASAGN(-69.37)VYPQPIIN(69	17	3	962	1	1	1	1
IPI00119063	3789	EEDCSIDPKITSCATNASMCGDEARCVRTEK	1	72.4463	219.52	72.446 _ITSCATN(de)ASM(ox)CGDEAR_	ITSCATN(1)ASMCGDEAR	ITSCATN(72.45)ASMCGDEAR	7	2	881	1	1	1	1
IPI00119063	1724	VVHPIRGKIYWTDGDNISMANMDGSNHTIIF	1	104.631	131.27	104.63 _IYWTDGDN(de)ISM(ox)AN(de)M(ox)DGSN(de)HTIIFSGQK_	IYWTDGDN(1)ISMAN(1)MDGSN(1)HT	IYWTDGDN(104.63)ISMAN(104.63	8	3	1017	1	1	1	1
IPI00122557	424	FQEIIGDISFIIPTINFSKYIRDAGCPVFIY	1	100.282	202.16	100.28 _YAFQ(de)EIIGDISFIIPTIN(de)FSKYIR_	YAFQEIIGDISFIIPTIN(1)FSKYIR	YAFQEIIGDISFIIPTIN(100.28)FS	18	3	951	1	1	1	1
IPI00331214	220	NDTVDGVYKVFNGKDNISKVAIIESYKGKRN	1	82.9999	98.898	83 _VFN(de)GKDN(de)ISK_	VFN(1)GKDN(1)ISK	VFN(83)GKDN(83)ISK	7	2	562	1	1	1	1
IPI00113057	127	HRDIYKGIDMRGSNFNISKTDNIEECQKICT	0.999985	48.2605	132.88	132.88 _GSNFN(de)ISK_	GSNFN(1)ISK	GSN(-48.26)FN(48.26)ISK	5	2	434	1	1	1	1
IPI00623114	2327	SDSEPNRGISYQIIGNHSKSHDHFHIDSNTG	1	97.4631	128.54	97.463 _GISYQIIGN(de)HSK_	GISYQIIGN(1)HSK	GISYQIIGN(97.46)HSK	9	2	659	1	1	1	1
IPI00115116	82	DSIYSQVVGISASQANISKQINISIIVKETV	1	185.873	237	185.87 _ADSIYSQVVGISASQAN(de)ISK_	ADSIYSQVVGISASQAN(1)ISK	ADSIYSQVVGISASQAN(185.87)ISK	17	2	1020	1	1	1	1
IPI00116105	89	ISTRGSTQYIENIGFNMSKMSEAEIHQGFQY	1	87.4287	200.55	87.429 _GSTQYIEN(de)IGFN(de)M(ox)SK_	GSTQYIEN(1)IGFN(1)MSK	GSTQYIEN(87.43)IGFN(87.43)MS	12	2	854	1	1	1	1
IPI00126184	288	CMASEIPEHTIKICQNISKKNSKFEECCQEN	1	131.062	131.06	131.06 _ICQN(de)ISKK_	ICQN(1)ISKK	ICQN(131.06)ISKK	4	2	496	1	1	1	1
IPI00331214	79	QFWIFDVQNPDDVAKNSSKIKVKQRGPYTYR	1	73.2082	152.94	73.208 _QFWIFDVQN(de)PDDVAKN(de)SSK_	QFWIFDVQN(1)PDDVAKN(1)SSK	QFWIFDVQN(73.21)PDDVAKN(73.2	16	3	747	1	1	1	1

IPI00187353	145	CEQANKIGAINSTISNESKEAFIDWARYDDS	1	58.9802	140.63	58.98	_IGAIN(de)STISN(de)ESKEAFIDWAR_	IGAIN(1)STISN(1)ESKEAFIDWAR	IGAIN(58.98)STISN(58.98)ESKE	10	3	775	1	1	1	1
IPI00322463	162	VPKFAIIKDYRPSAGNNSIYQDTVVFKCIPH	1	71.3759	199.15	71.376	_DYRPSAGN(de)N(de)SIYQDTVVFK_	DYRPSAGN(1)N(1)SIYQDTVVFK	DYRPSAGN(71.38)N(71.38)SIYQD	8	2	1089	1	1	1	1
IPI00848693	1320	FTRGVENDVFIRYPNNGSIVWGKVWPDYPNI	1	58.2738	175.59	58.274	_YPN(de)N(de)GSIVWGK_	YPN(1)N(1)GSIVWGK	YPN(58.27)N(58.27)GSIVWGK	4	2	619	1	1	1	1
IPI00652675	300	YGNYTCVAANKIGVTNASIVIFRPGSVRGIN	1	207.921	207.92	207.92	_IGVTN(de)ASIVIFRPGSVR_	IGVTN(1)ASIVIFRPGSVR	IGVTN(207.92)ASIVIFRPGSVR	5	2	894	1	1	1	1
IPI00134585	189	GDSVYRITMEFKGWINGSIVGFYKTTYMEDG	1	138.936	194.24	138.94	_GWIN(de)GSIVGFYK_	GWIN(1)GSIVGFYK	GWIN(138.94)GSIVGFYK	4	2	671	1	1	1	1
IPI00331318	233	KNIMAIKKFVMVKFFNDSIVDPVDSEWFGFY	1	178.717	178.72	178.72	_FFN(de)DSIVDPVDSEWFGFYR_	FFN(1)DSIVDPVDSEWFGFYR	FFN(178.72)DSIVDPVDSEWFGFYR	3	2	1171	1	1	1	1
IPI00894972	696	RSRGIFPDGSHEISGNTSITPDKSCIDFHFH	1	109.872	109.87	109.87	_GIFPDGSHEISGN(de)TSITPDK_	GIFPDGSHEISGN(1)TSITPDK	GIFPDGSHEISGN(109.87)TSITPDK	13	3	692	1	1	1	1
IPI00123428	52	SATSFIEDIMDRYGKNDSITITQIKSIIDHI	1	109.145	201.48	109.15	_YGKN(de)DSITITQIK_	YGKN(1)DSITITQIK	YGKN(109.15)DSITITQIK	4	3	495	1	1	1	1
IPI01026704	1113	FSSINITIRGEIQSENSSITISSSNRKREIA	1	118.27	235.75	124.25	_GEIQSEN(de)SSITISSSNR_	GEIQSEN(1)SSITISSSNR	GEIQSEN(118.27)SSITISSSN(-11	7	3	604	1	1	1	1
IPI00321634	185	ETTERVFPYISVMVNNGSISYDHSKDGRWSE	1	89.8461	232.15	89.846	_VFPYISVM(ox)VN(de)N(de)GSISYDHSKDGR_	VFPYISVMVN(1)N(1)GSISYDHSKDGR	VFPYISVMVN(89.85)N(89.85)GSI	11	4	652	1	1	1	1
IPI00830356	191	PGVQRVFPYISAMVNNGSISYDHERDGRPTE	1	56.8514	136.2	56.851	_VFPYISAM(ox)VN(de)N(de)GSISYDHER_	VFPYISAMVN(1)N(1)GSISYDHER	VFPYISAMVN(56.85)N(56.85)GSI	11	4	580	1	1	1	1
IPI00123194	271	IYRIGIGHNQIRMIENGSISFIPTIREIHID	1	96.2471	163.51	96.247	_M(ox)IEN(de)GSISFIPTIR_	MIEN(1)GSISFIPTIR	MIEN(96.25)GSISFIPTIR	4	2	798	1	1	1	1
IPI00856723	116	QIINFSSSEIKVSITNVSISDEGRYFCQIYT	1	128.951	133.47	128.95	_VSITN(de)VSISDEGR_	VSITN(1)VSISDEGR	VSITN(128.95)VSISDEGR	5	2	689	1	1	1	1
IPI00309230	416	DECPGVGIVIPQSFGNESIRHHIEVMEEIVR	1	109.059	149.41	109.06	_YGIVVIDECPGVGIVIPQSFGN(de)ESIR_	YGIVVIDECPGVGIVIPQSFGN(1)ESIR	YGIVVIDECPGVGIVIPQSFGN(109.0	22	2	1410	1	1	1	1
IPI00130117	855	EMFQVQDMVVSQEKGNCSIQRNPTPCIIPQE	1	76.0486	76.049	76.049	_ISPGGAEM(ox)FQVQDM(ox)VVSQEKGN(de)CSIQR_	ISPGGAEMFQVQDMVVSQEKGN(1)CSIQ	ISPGGAEMFQVQDMVVSQEKGN(76.05	22	4	758	1	1	1	1
IPI00112614	161	DFIVDNETFSGFIQHNISIPRSTVDSIIQAN	1	148.889	149.85	148.89	_IQDFIVDN(de)ETFSGFIQHN(de)ISIPR_	IQDFIVDN(1)ETFSGFIQHN(1)ISIPR	IQDFIVDN(148.89)ETFSGFIQHN(1	18	3	898	1	1	1	1
IPI00115482	11	_____MEAHNVSAPFNFSIPPGFGHRATDTA	1	73.9176	192.16	73.918	_M(ox)EAHN(de)VSAPFN(de)FSIPPGFGHR_	MEAHN(1)VSAPFN(1)FSIPPGFGHR	MEAHN(73.92)VSAPFN(73.92)FSI	11	3	777	1	1	1	1
IPI01026704	100	GRERSMPCVKIDIPVNTSIPNVTEIKENMTF	1	111.643	168.41	111.64	_IDIPVN(de)TSIPN(de)VTEIK_	IDIPVN(1)TSIPN(1)VTEIK	IDIPVN(111.64)TSIPN(111.64)V	6	3	586	1	1	1	1
IPI00135560	245	IDMEFRGAFFPIKEDNWSIPNRAVEPQIEDD	0.999999	60.1786	128.22	128.22	_GAFFPIKEDN(de)WSIPNR_	GAFFPIKEDN(1)WSIPNR	GAFFPIKEDN(60.18)WSIPN(-60.1	10	2	946	1	1	1	1
IPI00129485	176	FFHNEKSVRYHHYSSNFSIPKANHSHSGDYY	1	158.3	187.85	158.3	_YHHYSSN(de)FSIPK_	YHHYSSN(1)FSIPK	YHHYSSN(158.3)FSIPK	7	3	494	1	1	1	1
IPI00138180	59	KRDWIWNQMHIDEEKNESIPHYVGKIKSNVN	1	143.714	143.71	143.71	_DWIWN(de)QM(ox)HIDEEKN(de)ESIPHYVGK_	DWIWN(1)QMHIDEEKN(1)ESIPHYVGK	DWIWN(143.71)QMHIDEEKN(143.7	14	3	963	1	1	1	1
IPI00108811	165	RVYTYADTPNDFQISNFSIPEEDTKIKIPII	1	50.2553	180.63	50.255	_VYTYADTPN(de)DFQISN(de)FSIPEEDTKIK_	VYTYADTPN(1)DFQISN(1)FSIPEEDT	VYTYADTPN(50.26)DFQISN(50.26	15	4	760	1	1	1	1
IPI00480532	48	FVGIEGPRVIETIPANYSINSSKKIKTVIPS	1	103.387	160.48	103.39	_VIETIPAN(de)YSIN(de)SSK_	VIETIPAN(1)YSIN(1)SSK	VIETIPAN(103.39)YSIN(103.39)	8	2	819	1	1	1	1
IPI00858152	129	YGNYTCVASNKIGHTNASIMIFEVKTTAITP	1	129.537	129.54	129.54	_IGHTN(de)ASIM(ox)IFEVK_	IGHTN(1)ASIMIFEVK	IGHTN(129.54)ASIMIFEVK	5	3	526	1	1	1	1
IPI00312711	577	SASGAEIEAIEAQVINISIKYNFVTPITHMV	1	167.239	231.83	167.24	_ISASGAEIEAIEAQVIN(de)ISIK_	ISASGAEIEAIEAQVIN(1)ISIK	ISASGAEIEAIEAQVIN(167.24)ISI	17	2	1079	1	1	1	1
IPI00123040	461	PKAPQHVVNHIPPYTNVSIKMIITNPEGRKE	1	65.467	78.326	65.467	_APQHVVN(de)HIPPYTN(de)VSIK_	APQHVVN(1)HIPPYTN(1)VSIK	APQHVVN(65.47)HIPPYTN(65.47)	14	3	673	1	1	1	1
IPI00469218	312	NMTIPDAIVPTFSISNHSIKAIQATVGNSYK	1	92.4736	100.39	92.474	_IN(de)M(ox)TIPDAIVPTFSISN(de)HSIK_	IN(1)MTIPDAIVPTFSISN(1)HSIK	IN(92.47)MTIPDAIVPTFSISN(92.	17	3	773	1	1	1	1
IPI00221706	234	KNYSEVCRNCKEAYKNISIIYSQMQKINGIE	1	68.9439	162.48	68.944	_EAYKN(de)ISIIYSQM(ox)QK_	EAYKN(1)ISIIYSQMQK	EAYKN(68.94)ISIIYSQMQK	5	2	917	1	1	1	1
IPI00317488	157	ERAACRIEYKFGEFGNYSIIVQHASSGANKI	1	60.7641	218.25	60.764	_FGEFGN(de)YSIIVQHASSGAN(de)K_	FGEFGN(1)YSIIVQHASSGAN(1)K	FGEFGN(60.76)YSIIVQHASSGAN(6	6	3	710	1	1	1	1
IPI00323134	692	EVPIIITDSGNPPKSNISIIRVKVCQCDSNG	1	114.498	167.59	114.5	_SN(de)ISIIR_	SN(1)ISIIR	SN(114.5)ISIIR	2	2	402	1	1	1	1
IPI00108535	375	NSQSIQITERMTISQNNSIIRIDPIKREDAG	1	156.362	160.52	156.36	_M(ox)TISQN(de)N(de)SIIR_	MTISQN(1)N(1)SIIR	MTISQN(156.36)N(156.36)SIIR	6	2	648	1	1	1	1
IPI00123442	47	FIFEKWKIYSDQCHHNISIIPPPTEIVCNRT	1	99.4091	99.409	99.409	_IYSDQCHHN(de)ISIIPPPTEIVCN(de)R_	IYSDQCHHN(1)ISIIPPPTEIVCN(1)R	IYSDQCHHN(99.41)ISIIPPPTEIVC	9	3	922	1	1	1	1
IPI00115516	795	IIGGQAGIGRRIGAINNSIIIIEDRIQQISI	1	93.4784	125.03	93.478	_RIGAIN(de)N(de)SIIIIEDR_	RIGAIN(1)N(1)SIIIIEDR	RIGAIN(93.48)N(93.48)SIIIED	6	3	567	1	1	1	1
IPI00406603	1049	IRGHNHSPFPDRRIINHSIIIHKSNETAISP	1	91.9373	158.05	91.937	_IIN(de)HSIIIHK_	IIN(1)HSIIIHK	IIN(91.94)HSIIIHK	3	2	595	1	1	1	1
IPI00108535	104	NFTGQAYSGREIIYSNGSIIFQMITMKDMGV	1	78.1909	269.49	78.191	_EIIYSN(de)GSIIFQ(de)M(ox)ITM(ox)K_	EIIYSN(1)GSIIFQMITMK	EIIYSN(78.19)GSIIFQMITMK	6	3	675	1	1	1	1
IPI00130486	397	CSVISKKGDYIKYHYNASIIDGTIIDSTWNI	1	101.423	135.09	135.09	_YHYN(de)ASIIDGTIIDSTWNIGK_	YHYN(1)ASIIDGTIIDSTWNIGK	YHYN(101.42)ASIIDGTIIDSTWN(-	4	3	795	1	1	1	1
IPI00330481	171	QVQYMKAVVGSYTTTNVSIIAPRSEHIVNFT	1	157.233	179.14	157.23	_AVVGSYTTTN(de)VSIIAPR_	AVVGSYTTTN(1)VSIIAPR	AVVGSYTTTN(157.23)VSIIAPR	10	3	584	1	1	1	1
IPI00808345	114	MEEMIRNKSSECKAINDSIHYINREQNRCIR	1	107.37	162.68	162.68	_AIN(de)DSIHYINR_	AIN(1)DSIHYINR	AIN(107.37)DSIHYIN(-107.37)R	3	2	659	1	1	1	1
IPI00116945	124	PDSIEDDIIIFKISQNASIGPHVRPIPIQYE	1	65.2717	95.409	65.272	_ISQN(de)ASIGPHVRPIPIQYEDKEVEPGTICDVAGWGVVTHAGR_	ISQN(1)ASIGPHVRPIPIQYEDKEVEPG	ISQN(65.27)ASIGPHVRPIPIQYEDK	4	4	1139	1	1	1	1
IPI00314673	131	ECRRCVVVGNGHRIRNSSIGGVINKYDVVIR	1	122.955	165.58	165.58	_IRN(de)SSIGGVINK_	IRN(1)SSIGGVINK	IRN(122.95)SSIGGVIN(-122.95)	3	2	630	1	1	1	1
IPI00405543	141	SKHFAKIEIVPIIYDNDSIFVQTDKSVYTPQ	1	63.337	173.28	63.337	_IEIVPIIYDN(de)DSIFVQTDK_	IEIVPIIYDN(1)DSIFVQTDK	IEIVPIIYDN(63.34)DSIFVQTDK	10	3	742	1	1	1	1
IPI00459432	228	VCQIEVAIVGASPRGNHSIFGIEVATIGQGP	1	117.404	236.38	146.6	_GN(de)HSIFGIEVATIGQGPDCPSVNER_	GN(1)HSIFGIEVATIGQGPDCPSVNER	GN(117.4)HSIFGIEVATIGQGPDCPS	2	3	886	1	1	1	1
IPI00130754	139	IEFKKYHDYYITSTSNGSIEGIENREGGVCR	1	67.1895	129.46	100.02	_KYHDYYITSTSN(de)GSIEGIENR_	KYHDYYITSTSN(1)GSIEGIENR	KYHDYYITSTSN(67.19)GSIEGIEN(	12	3	817	1	1	1	1
IPI00621027	969	HAEISFVFITDGVTGNDSIEESVHSMRKQNV	1	50.202	75.773	50.202	_HAEISFVFITDGVTGN(de)DSIEESVHSM(ox)R_	HAEISFVFITDGVTGN(1)DSIEESVHSM	HAEISFVFITDGVTGN(50.2)DSIEES	16	4	749	1	1	1	1
IPI00856861	382	DDKQPAFRAVYEAIRNCSIEDDSVRSIIKPI	1	123.079	123.08	123.08	_AVYEAIRN(de)CSIEDDSVR_	AVYEAIRN(1)CSIEDDSVR	AVYEAIRN(123.08)CSIEDDSVR	8	2	999	1	1	1	1
IPI00221669	598	NSIWNIEEHINPKIPNISIDVIQPSFPEIII	1	37.2277	65.425	37.228	_IPN(de)ISIDVIQPSFPEIIIESHM(ox)VM(ox)IR_	IPN(1)ISIDVIQPSFPEIIIESHMVMIR	IPN(37.23)ISIDVIQPSFPEIIIESH	3	4	757	1	1	1	1
IPI00115976	612	ESEFRDKISPIHIAINFSIDPKAPMDSHGIR	1	62.1618	124.51	62.162	_ISPIHIAIN(de)FSIDPK_	ISPIHIAIN(1)FSIDPK	ISPIHIAIN(62.16)FSIDPK	9	3	556	1	1	1	1
IPI00127407	198	FYTKIFINPEKREQINISIDHRCRIFQNIDG	1	68.5357	95.094	68.536	_EQIN(de)ISIDHR_	EQIN(1)ISIDHR	EQIN(68.54)ISIDHR	4	2	613	1	1	1	1
IPI00411145	1467	IEASRIDGSCRKVIINNSIDEPRAIAVFPRK	1	79.492	93.345	79.492	_KVIIN(de)N(de)SIDEPR_	KVIIN(1)N(1)SIDEPR	KVIIN(79.49)N(79.49)SIDEPR	5	2	700	1	1	1	1
IPI00315535	103	RFDAAGTSVHGGVPQNGSICAHIPMIKESVE	1	67.8637	131.33	67.864	_FDAAGTSVHGGVPQN(de)GSICAHIPM(ox)IK_	FDAAGTSVHGGVPQN(1)GSICAHIPMIK	FDAAGTSVHGGVPQN(67.86)GSICAH	15	3	894	1	1	1	1
IPI00115599	162	YVVMSTAAIPMIKQSNGSIAVISSIAGKMTQ	1	55.8644	176.21	55.864	_QSN(de)GSIAVISSIAGK_	QSN(1)GSIAVISSIAGK	QSN(55.86)GSIAVISSIAGK	3	2	717	1	1	1	1
IPI00121627	295	YFNDYWNIQKDYYPINESIASIPIRVSFCPI	1	95.5698	211.57	95.57	_DYYPIN(de)ESIASIPIR_	DYYPIN(1)ESIASIPIR	DYYPIN(95.57)ESIASIPIR	6	2	876	1	1	1	1
IPI00123196	257	ISKIGISFNSITVMENGSIANVPHIREIHID	1	54.15	172.57	54.15	_IGISFN(de)SITVM(ox)EN(de)GSIAN(de)VPHIR_	IGISFN(1)SITVMEN(1)GSIAN(1)VP	IGISFN(54.15)SITVMEN(54.15)G	13	3	830	1	1	1	1
IPI00116913	2211	GINASSAAWARIHRINASIADIQSKIRSPPG	1	85.5541	128.95	85.554	_IN(de)ASIADIQSK_	IN(1)ASIADIQSK	IN(85.55)ASIADIQSK	2	2	581	1	1	1	1
IPI00850413	558	SSTDISIKIHVAQPENDSHVAIIKMYTSSDC	1	49.3332	210.14	49.333	_IHVAQPEN(de)DSHVAIIK_	IHVAQPEN(1)DSHVAIIK	IHVAQPEN(49.33)DSHVAIIK	8	4	444	1	1	1	1
IPI00467180	88	SGMINVKWDRIAPASNVSHTVVIRPIKAGYF	1	133.862	239.58	133.86	_IAPASN(de)VSHTVVIRPIK_	IAPASN(1)VSHTVVIRPIK	IAPASN(133.86)VSHTVVIRPIK	6	2	902	1	1	1	1
IPI00133365	212	RGHAHIAAIVNHDSYNFSHRIDHISFGEIVP	1	76.3411	140.22	76.341	_GHAHIAAIVN(de)HDSYN(de)FSHR_	GHAHIAAIVN(1)HDSYN(1)FSHR	GHAHIAAIVN(76.34)HDSYN(76.34	15	3	717	1	1	1	1
IPI00318595	403	YFFGKCFNAMEVDAINSSHPVSTPVENPAQI	1	67.4923	218.44	218.44	_CFNAM(ox)EVDAIN(de)SSHPVSTPVENPAQIR_	CFNAMEVDAIN(1)SSHPVSTPVENPAQI	CFN(-90.15)AMEVDAIN(67.49)SS	11	3	1001	1	1	1	1
IPI00331032	113	QSTIDCVDPISSIAANRSHIPISPCEHGWVY	1	131.329	142.23	131.33	_YEVDWN(de)QSTIDCVDPISSIAAN(de)R_	YEVDWN(1)QSTIDCVDPISSIAAN(1)R	YEVDWN(131.33)QSTIDCVDPISSIA	22	2	1328	1	1	1	1
IPI00108811	289	HQRDFISRDIGPAIANSSHDVKIIMIDDQRI	1	79.6139	198.16	79.614	_DIGPAIAN(de)SSHDVK_	DIGPAIAN(1)SSHDVK	DIGPAIAN(79.61)SSHDVK	8	2	713	1	1	1	1
IPI00223446	574	DGAKNEIQGKISNISNISHDIVQEATDHAYN	0.734619	4.42192	125	125	_ISNISN(de)ISHDIVQEATDHAYNIQQEADEISR_	ISN(0.265)ISN(0.735)ISHDIVQEA	ISN(-4.42)ISN(4.42)ISHDIVQEA	6	3	1171	1	1	1	1
IPI00128260	143	SMNPDGYEVAAAQGPNMSGYIVGRNNANGVD	1	80.9745	238.7	238.7	_IHIIPSM(ox)NPDGYEVAAAQGPN(de)M(ox)SGYIVGR_	IHIIPSMNPDGYEVAAAQGPN(1)MSGYI	IHIIPSMN(-80.97)PDGYEVAAAQGP	21	3	1031	1	1	1	1
IPI00408495	160	SVQEVNSKTQITCSINSSGVDIVGHRWMRGG	1	91.4746	234.38	91.475	_TQ(de)ITCSIN(de)SSGVDIVGHR_	TQITCSIN(1)SSGVDIVGHR	TQITCSIN(91.47)SSGVDIVGHR	8	2	973	1	1	1	1
IPI00331550	325	GQRIMGIRAIAGIVYNSSGTEPCYDIYRIYQ	1	93.4953	264.23	93.495	_AIAGIVYN(de)SSGTEPCYDIYR_	AIAGIVYN(1)SSGTEPCYDIYR	AIAGIVYN(93.5)SSGTEPCYDIYR	8	3	751	1	1	1	1
IPI00222429	210	NSNANAAGPIIVAGYNVSGSVRSDGEPMKGV	1	83.8668	145.61	117.21	_VTNSNAN(de)AAGPIIVAGYN(de)VSGSVR_	VTN(0.137)SN(0.006)AN(0.858)A	VTN(-7.98)SN(-21.89)AN(7.98)	18	2	1167	1	1	1	1
IPI00757771	283	WIWRKKENGVFEEISNSSGRFFITNKENYTE	1	97.9042	123.76	97.904	_KKEN(de)GVFEEISN(de)SSGR_	KKEN(1)GVFEEISN(1)SSGR	KKEN(97.9)GVFEEISN(97.9)SSGR	12	2	892	1	1	1	1
IPI00121550	158	RGERKVCRFKIDWIGNCSGINDDSYGYREGK	1	151.626	221.79	151.63	_IDWIGN(de)CSGIN(de)DDSYGYR_	IDWIGN(1)CSGIN(1)DDSYGYR	IDWIGN(151.63)CSGIN(151.63)D	6	2	1054	1	1	1	1
IPI00119063	3954	SNRHRRQIDRGVTHINISGIKMPRGIAIDWV	1	125.97	203.61	125.97	_GVTHIN(de)ISGIKM(ox)PR_	GVTHIN(1)ISGIKMPR	GVTHIN(125.97)ISGIKMPR	6	3	514	1	1	1	1
IPI00127560	118	GISPFHEFADVVFTANDSGHRHYTIAAIISP	1	127.586	234.38	127.59	_TIGISPFHEFADVVFTAN(de)DSGHR_	TIGISPFHEFADVVFTAN(1)DSGHR	TIGISPFHEFADVVFTAN(127.59)DS	18	4	630	1	1	1	1
IPI00226430	342	FISVQKAIDIDPSKTNVSGGAIAIGHPIGGS	1	130.267	130.27	130.27	_AIDIDPSKTN(de)VSGGAIAIGHPIGGSGSR_	AIDIDPSKTN(1)VSGGAIAIGHPIGGSG	AIDIDPSKTN(130.27)VSGGAIAIGH	10	3	883	1	1	1	1
IPI00129485	102	IRSQVQASYTFKATVNDSGEYRCQMEQTRIS	1	108.372	174.42	108.37	_ATVN(de)DSGEYR_	ATVN(1)DSGEYR	ATVN(108.37)DSGEYR	4	2	557	1	1	1	1
IPI00134691	297	CIQKKPISQEFEAYVNASGEHGIVVFSIGSM	1	54.3085	94.15	54.308	_KPISQEFEAYVN(de)ASGEHGIVVFSIGSM(ox)VSEIPEKK_	KPISQEFEAYVN(1)ASGEHGIVVFSIGS	KPISQEFEAYVN(54.31)ASGEHGIVV	12	4	957	1	1	1	1
IPI00403938	1394	IEIIDSNRIIQTAEHNISGAERTAHISGIPP	1	107.825	220.85	107.82	_IIQTAEHN(de)ISGAER_	IIQTAEHN(1)ISGAER	IIQTAEHN(107.82)ISGAER	8	2	770	1	1	1	1
IPI00467600	2391	KSISGRDIEHHITNVNVSFYDDIVNGTVIKT	1	71.1476	181.54	71.148	_DIEHHITN(de)VN(de)VSFYDDIVN(de)GTVIK_	DIEHHITN(1)VN(1)VSFYDDIVN(1)G	DIEHHITN(71.15)VN(71.15)VSFY	10	3	916	1	1	1	1
IPI00121362	185	ISMITADAKKTRAFMNSSFTIDPKSGDIIFD	1	68.3555	162.49	68.355	_AFM(ox)N(de)SSFTIDPK_	AFMN(1)SSFTIDPK	AFMN(68.36)SSFTIDPK	4	3	459	1	1	1	1
IPI00137831	47	VVRPDSEIGERPPEDNQSFQYDHEAFIGKED	1	88.7904	88.79	88.79	_VVRPDSEIGERPPEDN(de)QSFQYDHEAFIGK_	VVRPDSEIGERPPEDN(1)QSFQYDHEAF	VVRPDSEIGERPPEDN(88.79)QSFQY	16	4	841	1	1	1	1
IPI00624663	568	DAQNFEIEKCFANKVNISFPSAQSIPASDTH	1	68.5672	220.82	68.567	_VN(de)ISFPSAQSIPASDTHIK_	VN(1)ISFPSAQSIPASDTHIK	VN(68.57)ISFPSAQSIPASDTHIK	2	2	1007	1	1	1	1
IPI00129265	319	QAFDWGSSEKNYFHYNQSFPPSYNIKNMRIP	1	80.0543	170.78	170.78	_NYFHYN(de)QSFPPSYNIK_	NYFHYN(1)QSFPPSYNIK	N(-80.05)YFHYN(80.05)QSFPPSY	6	2	1010	1	1	1	1
IPI00381178	317	KDIITKKNVNISYTVNDSFFPQRPQKIIANK	1	71.7807	283.31	71.781	_N(de)VN(de)ISYTVN(de)DSFFPQRPQK_	N(1)VN(1)ISYTVN(1)DSFFPQRPQK	N(71.78)VN(71.78)ISYTVN(71.7	9	3	753	1	1	1	1
IPI00122557	317	KDIIKQKNVNISYIVNDSFFPQRPEKIIADQ	1	104.026	192.6	104.03	_QKN(de)VN(de)ISYIVN(de)DSFFPQRPEK_	QKN(1)VN(1)ISYIVN(1)DSFFPQRPE	QKN(104.03)VN(104.03)ISYIVN(	11	3	843	1	1	1	1
IPI00108844	58	SKNEVAIIERIRPIFNKSFESTVGQGSDTYS	1	132.645	132.65	132.65	_IRPIFN(de)KSFESTVGQGSDTYSYIFR_	IRPIFN(1)KSFESTVGQGSDTYSYIFR	IRPIFN(132.65)KSFESTVGQGSDTY	6	3	972	1	1	1	1
IPI00381303	489	VIVSNAIARISHYKQNFSFCREINISICPVS	1	95.4173	95.417	95.417	_ISHYKQN(de)FSFCR_	ISHYKQN(1)FSFCR	ISHYKQN(95.42)FSFCR	7	2	794	1	1	1	1
IPI00121312	184	QGDYTIKIDITDFEKNSSFAQYQSFKVGDKK	1	89.8587	231.83	89.859	_IDITDFEKN(de)SSFAQYQSFK_	IDITDFEKN(1)SSFAQYQSFK	IDITDFEKN(89.86)SSFAQYQSFK	9	2	1135	1	1	1	1
IPI00322463	105	IENGIVRYTSFEYPKNISFACNPGFFINGTS	1	80.8302	211.64	80.83	_YTSFEYPKN(de)ISFACN(de)PGFFIN(de)GTSSSK_	YTSFEYPKN(1)ISFACN(1)PGFFIN(1)	YTSFEYPKN(80.83)ISFACN(80.83	9	3	1023	1	1	1	1
IPI00114044	1129	VPSSISIMHSPPDAQNMSEVSISPMEISTFR	1	49.1988	98.76	49.199	_FAVESIVPSSISIM(ox)HSPPDAQN(de)M(ox)SEVSISPM(ox)EISTFR_	FAVESIVPSSISIMHSPPDAQN(1)MSEV	FAVESIVPSSISIMHSPPDAQN(49.2)	22	4	1018	1	1	1	1
IPI00136012	627	EQDIAYIQEIKEKVENGSETGPIPPEIQPII	1	60.9906	121.38	60.991	_EKVEN(de)GSETGPIPPEIQPIIEGEVK_	EKVEN(1)GSETGPIPPEIQPIIEGEVK	EKVEN(60.99)GSETGPIPPEIQPIIE	5	3	897	1	1	1	1
IPI00409148	148	ISDQWIITTAKNIFINHSETASAKDITPTIT	1	80.1077	190.27	166.86	_NIFIN(de)HSETASAK_	NIFIN(1)HSETASAK	N(-80.11)IFIN(80.11)HSETASAK	5	2	717	1	1	1	1
IPI00831484	390	EETASIIIRRFHSDINISESIIPAVIEKYIR	1	89.358	89.358	89.358	_RFHSDIN(de)ISESIIPAVIEK_	RFHSDIN(1)ISESIIPAVIEK	RFHSDIN(89.36)ISESIIPAVIEK	7	3	724	1	1	1	1
IPI00123428	100	NISTCFSSGDIFAAHNISERSQIGASEFQEF	1	84.3274	185.71	84.327	_EGPRN(de)ISTCFSSGDIFAAHN(de)ISER_	EGPRN(1)ISTCFSSGDIFAAHN(1)ISE	EGPRN(84.33)ISTCFSSGDIFAAHN(	20	3	890	1	1	1	1
IPI00128358	242	GCTAEGICCHKECIGNCSEPDDPTKCVACRN	1	109.477	109.48	109.48	_ECIGN(de)CSEPDDPTK_	ECIGN(1)CSEPDDPTK	ECIGN(109.48)CSEPDDPTK	5	2	812	1	1	1	1
IPI00115867	388	QKTAASIIWQAYPIINISEKIIPAAIEKYIG	1	132.791	170.16	132.79	_TAASIIWQAYPIIN(de)ISEK_	TAASIIWQAYPIIN(1)ISEK	TAASIIWQAYPIIN(132.79)ISEK	14	2	1010	1	1	1	1
IPI00987265	2290	CGNNRVGVVSIGVRKNISEIWDAYCYRVQDV	1	64.6504	77.912	64.65	_KN(de)ISEIWDAYCYR_	KN(1)ISEIWDAYCYR	KN(64.65)ISEIWDAYCYR	2	2	860	1	1	1	1

IPI00416285	449	GIETPKKIRVYGGIVNQSEINEGTAFFRVQE	1	76.7324	211.09	159.4	_VYGGIVN(de)QSEINEGTAFFR_	VYGGIVN(1)QSEINEGTAFFR	VYGGIVN(76.73)QSEIN(-76.73)E	7	3	701	1	1	1	1
IPI00132474	520	DEVNSEDMDAYCRKENSSEICSNNGECVCGQ	0.999965	44.4959	238.68	238.68	_KEN(de)SSEICSNN(de)GECVCGQCVCR_	KEN(1)SSEICSN(0.019)N(0.981)GI	KEN(44.5)SSEICSN(-17.17)N(17	3	3	884	1	1	1	1
IPI00855103	391	KIASHPAEQFTSKIGNFSEIATHNQTFIKKI	1	69.9345	199.37	69.935	_IGN(de)FSEIATHN(de)QTFIKK_	IGN(1)FSEIATHN(1)QTFIKK	IGN(69.93)FSEIATHN(69.93)QTF	3	2	976	1	1	1	1
IPI00115626	171	RPHFISVCDPDFSQINCSEGYIQNYRCRGED	1	91.7527	161.02	161.02	_IRPHFISVCDPDFSQIN(de)CSEGYIQNYR_	IRPHFISVCDPDFSQIN(1)CSEGYIQNYI	IRPHFISVCDPDFSQIN(91.75)CSEG	17	3	1106	1	1	1	1
IPI00127352	233	PIITGTIKKEDSCQINYSEGPCIGMQERYYY	1	118.719	195.96	118.72	_KEDSCQIN(de)YSEGPCIGM(ox)QER_	KEDSCQIN(1)YSEGPCIGMQER	KEDSCQIN(118.72)YSEGPCIGMQER	8	3	807	1	1	1	1
IPI00458003	594	IQNTPGIEEQANQRINISEGEVAATVKANIP	1	137.456	137.46	137.46	_IN(de)ISEGEVAATVK_	IN(1)ISEGEVAATVK	IN(137.46)ISEGEVAATVK	2	2	666	1	1	1	1
IPI00114236	71	MDQNSDGWQDGVGYINSSEGAVGRSIQPIYR	0.999998	56.7574	143.31	143.31	_YM(ox)DQNSDGWQDGVGYIN(de)SSEGAVGR_	YMDQNSDGWQDGVGYIN(1)SSEGAVGR	YMDQN(-56.76)SDGWQDGVGYIN(56	17	3	908	1	1	1	1
IPI00459432	133	SSAIVFTRIIEFDSTNASEGAQPPGKPYPPY	1	55.2471	162.83	55.247	_IIEFDSTN(de)ASEGAQPPGKPYPPYSIAK_	IIEFDSTN(1)ASEGAQPPGKPYPPYSIAI	IIEFDSTN(55.25)ASEGAQPPGKPYP	8	4	720	1	1	1	1
IPI00987265	607	SKGRVITMANQVITVNISEEGRIIIGPEGIP	1	94.5693	159.69	94.569	_VITM(ox)AN(de)QVITVN(de)ISEEGR_	VITMAN(1)QVITVN(1)ISEEGR	VITMAN(94.57)QVITVN(94.57)IS	12	3	665	1	1	1	1
IPI00135560	64	ITIPDVYGAKGHFYYNISDVRVTQIHIISSE	1	109.599	109.6	109.6	_GHFYYN(de)ISDVR_	GHFYYN(1)ISDVR	GHFYYN(109.6)ISDVR	6	2	686	1	1	1	1
IPI00310049	72	TAEFIEKKKEVHFFVNASDVDSVKAHINVSR	1	77.2224	138.91	77.222	_EVHFFVN(de)ASDVDSVK_	EVHFFVN(1)ASDVDSVK	EVHFFVN(77.22)ASDVDSVK	7	2	847	1	1	1	1
IPI00469218	116	KNTTRYSVQHMYFTYNISDTEHFPNAISKEI	1	74.9528	170.14	74.953	_YSVQHM(ox)YFTYN(de)ISDTEHFPN(de)AISK_	YSVQHMYFTYN(1)ISDTEHFPN(1)AISI	YSVQHMYFTYN(74.95)ISDTEHFPN(	11	3	971	1	1	1	1
IPI00222967	115	KEASHYSIHDIVISYNTSDSTVFPGAVAKGV	1	39.2149	222.48	39.215	_EASHYSIHDIVISYN(de)TSDSTVFPGAVAK_	EASHYSIHDIVISYN(1)TSDSTVFPGAV/	EASHYSIHDIVISYN(39.21)TSDSTV	15	3	1004	1	1	1	1
IPI00312711	244	QDTVINGDFIVRYDVNRSDSGGSIQIEEGYF	1	124.278	162.47	162.47	_YDVN(de)RSDSGGSIQIEEGYFVHHFAPENIPTM(ox)SK_	YDVN(1)RSDSGGSIQIEEGYFVHHFAPEI	YDVN(124.28)RSDSGGSIQIEEGYFV	4	4	936	1	1	1	1
IPI00719927	144	SENSPIGHSVIQVKANDSDQGANAEIDYTFH	1	109.497	109.5	109.5	_AN(de)DSDQGAN(de)AEIDYTFHQAPEVVRR_	AN(1)DSDQGAN(1)AEIDYTFHQAPEVVI	AN(109.5)DSDQGAN(109.5)AEIDY	2	3	936	1	1	1	1
IPI00136925	70	VERNIRIVVPINNRENISDPTSPIRRNFVYH	1	86.7722	196.71	86.772	_EN(de)ISDPTSPIRR_	EN(1)ISDPTSPIRR	EN(86.77)ISDPTSPIRR	2	2	693	1	1	1	1
IPI00116105	320	IQDVIADVGIKDIFTNQSDFADTTKDTPITI	1	105.627	188.39	105.63	_FSM(ox)SDTYDIQDVIADVGIKDIFTN(de)QSDFADTTK_	FSMSDTYDIQDVIADVGIKDIFTN(1)QSI	FSMSDTYDIQDVIADVGIKDIFTN(105	24	4	930	1	1	1	1
IPI01026704	341	EIKSIASEPTEKHFFNVSDEIAIVTIVKAIG	1	146.296	179.74	146.3	_HFFN(de)VSDEIAIVTIVK_	HFFN(1)VSDEIAIVTIVK	HFFN(146.3)VSDEIAIVTIVK	4	2	917	1	1	1	1
IPI00309249	490	SINVSSAASKQPIYINCSCVTGGSASAKTGS	1	183.598	183.6	183.6	_QPIYIN(de)CSCVTGGSASAK_	QPIYIN(1)CSCVTGGSASAK	QPIYIN(183.6)CSCVTGGSASAK	6	2	957	1	1	1	1
IPI00119063	2503	GGCQDICIITHQGHVNCSCRGGRIIQEDFTC	1	88.9277	106.49	104.06	_INN(de)GGCQDICIITHQGHVN(de)CSCR_	IN(0.115)N(0.885)GGCQDICIITHQ(	IN(-8.85)N(8.85)GGCQDICIITHQ	19	3	906	1	1	1	1
IPI00122592	79	PVRIKEQVVGIIAQNNCSCESKGGSIPIPFI	1	88.9922	195.46	88.992	_IKEQVVGIIAQN(de)N(de)CSCESK_	IKEQVVGIIAQN(1)N(1)CSCESK	IKEQVVGIIAQN(88.99)N(88.99)C	13	3	727	1	1	1	1
IPI00308990	180	KPGIKVISIAQAHSINFSCEQVRVFPAISTI	1	119.557	119.56	119.56	_VISIAQAHSIN(de)FSCEQVR_	VISIAQAHSIN(1)FSCEQVR	VISIAQAHSIN(119.56)FSCEQVR	11	3	687	1	1	1	1
IPI00310059	170	KRENAPSKKSICKKTNQSCEIVIDSTEKVNP	1	128.738	162.43	128.74	_TN(de)QSCEIVIDSTEK_	TN(1)QSCEIVIDSTEK	TN(128.74)QSCEIVIDSTEK	2	2	813	1	1	1	1
IPI00135560	143	GIQISQDSSGRIKVSNVSCEASVSKMNMAFG	1	69.6016	101.2	69.602	_VSN(de)VSCEASVSK_	VSN(1)VSCEASVSK	VSN(69.6)VSCEASVSK	3	2	634	1	1	1	1
IPI00123196	298	IAQHKYIQVVYIHNNNISAVGQNDFCRAGHP	1	54.2531	94.45	54.253	_YIQVVYIHN(de)N(de)N(de)ISAVGQN(de)DFCR_	YIQVVYIHN(1)N(1)N(1)ISAVGQN(1)	YIQVVYIHN(54.25)N(54.25)N(54	11	3	877	1	1	1	1
IPI00130420	357	FGVFAQSKPDSAEPVNRSAVCAFPIKYVNDF	1	84.9425	146.76	84.942	_QIGASPSDDIIFGVFAQSKPDSAEPVN(de)R_	QIGASPSDDIIFGVFAQSKPDSAEPVN(1)	QIGASPSDDIIFGVFAQSKPDSAEPVN(	27	3	983	1	1	1	1
IPI00469426	390	VFKTNSTQVSDVRAMNISASSMTITWKSNYD	1	149.298	149.3	149.3	_AM(ox)N(de)ISASSM(ox)TITWK_	AMN(1)ISASSMTITWK	AMN(149.3)ISASSMTITWK	3	2	787	1	1	1	1
IPI00894970	244	KGNVANWKISEEFIKNISASAREPPSENGTS	1	65.4716	108.58	65.472	_ISEEFIKN(de)ISASAR_	ISEEFIKN(1)ISASAR	ISEEFIKN(65.47)ISASAR	8	2	783	1	1	1	1
IPI00120761	660	TYTQKISVPDGIKVSNSSARGWVIHPIGIYR	1	146.959	162.37	146.96	_ISVPDGIKVSN(de)SSAR_	ISVPDGIKVSN(1)SSAR	ISVPDGIKVSN(146.96)SSAR	11	2	766	1	1	1	1
IPI00848693	951	ERIDCYPDEHGASEANCSARGCIWEASNTTR	1	66.9649	147.17	66.965	_IDCYPDEHGASEAN(de)CSAR_	IDCYPDEHGASEAN(1)CSAR	IDCYPDEHGASEAN(66.96)CSAR	14	3	685	1	1	1	1
IPI00114958	82	GSPTFYSFKYIIKEGNCSAQSGIAWQDCDFK	1	83.8336	112.82	83.834	_EGN(de)CSAQSGIAWQDCDFKDAEEAATGECTATVGKR_	EGN(1)CSAQSGIAWQDCDFKDAEEAATGI	EGN(83.83)CSAQSGIAWQDCDFKDAE	3	4	956	1	1	1	1
IPI00126769	345	ETEDDYGYQGHVQTCNFSAQMAKVYINDSVE	1	114.068	139.04	139.04	_NIGGIETEDDYGYQGHVQTCN(de)FSAQM(ox)AK_	NIGGIETEDDYGYQGHVQTCN(1)FSAQM/	N(-114.07)IGGIETEDDYGYQGHVQT	21	3	1051	1	1	1	1
IPI00330594	255	DISSQTQGFIQHSMDNISAQIQTVRDGMERA	1	86.5994	175.59	175.59	_GSIQSANDISSQTQGFIQHSM(ox)DN(de)ISAQIQTVR_	GSIQSANDISSQTQGFIQHSMDN(1)ISA(	GSIQSAN(-86.6)DISSQTQGFIQHSM	23	3	1160	1	1	1	1
IPI00137491	118	NIPWYVIAGNHDHIGNVSAQIAYSKISKRWN	0.999999	61.8707	173.18	74.364	_NIPWYVIAGN(de)HDHIGN(de)VSAQIAYSK_	NIPWYVIAGN(1)HDHIGN(1)VSAQIAY!	N(-43.39)IPWYVIAGN(43.39)HDH	16	3	924	1	1	1	1
IPI00553503	326	QRRIFNIYKMDFIIFNYSAPSYIRIQ_____	1	60.5499	162.29	60.55	_M(ox)DFIIFN(de)YSAPSYIR_	MDFIIFN(1)YSAPSYIR	MDFIIFN(60.55)YSAPSYIR	7	3	619	1	1	1	1
IPI00115482	5	____________MEAHNVSAPFNFSIPPGFGH	1	73.9176	192.16	73.918	_M(ox)EAHN(de)VSAPFN(de)FSIPPGFGHR_	MEAHN(1)VSAPFN(1)FSIPPGFGHR	MEAHN(73.92)VSAPFN(73.92)FSI	5	3	777	1	1	1	1
IPI00405437	430	TKERTEVIIQGTVSPNASAPDAVWEDYEFKC	1	57.1445	106.11	57.145	_TEVIIQGTVSPN(de)ASAPDAVWEDYEFK_	TEVIIQGTVSPN(1)ASAPDAVWEDYEFK	TEVIIQGTVSPN(57.14)ASAPDAVWE	12	3	956	1	1	1	1
IPI00330632	1385	AKVVVDCKEVGQKAINASANITSDGVEVIGR	1	103.17	207.76	103.17	_AIN(de)ASAN(de)ITSDGVEVIGR_	AIN(1)ASAN(1)ITSDGVEVIGR	AIN(103.17)ASAN(103.17)ITSDG	3	2	895	1	1	1	1
IPI00230718	276	GAGEVSPAEHSSKPTNISAKFKFSYFMGKNF	1	75.376	194.27	75.376	_GAGEVSPAEHSSKPTN(de)ISAK_	GAGEVSPAEHSSKPTN(1)ISAK	GAGEVSPAEHSSKPTN(75.38)ISAK	16	3	657	1	1	1	1
IPI00877197	518	NKRDAITAVRKMRAINGSAIYTGSSIDFVRN	1	76.3411	198.85	76.341	_AIN(de)GSAIYTGSSIDFVR_	AIN(1)GSAIYTGSSIDFVR	AIN(76.34)GSAIYTGSSIDFVR	3	3	591	1	1	1	1
IPI00453977	437	RVQGGSSVWGSVITHNSSAITYQSWGRSIKY	1	55.8436	126.86	55.844	_VQGGSSVWGSVITHN(de)SSAITYQSWGR_	VQGGSSVWGSVITHN(1)SSAITYQSWGR	VQGGSSVWGSVITHN(55.84)SSAITY	15	4	692	1	1	1	1
IPI00131830	270	DEEISCSVIEIKYTGNASAIIIIPDQGRMQQ	1	83.6916	185.27	83.692	_YTGN(de)ASAIIIIPDQ(de)GR_	YTGN(1)ASAIIIIPDQGR	YTGN(83.69)ASAIIIIPDQGR	4	3	564	1	1	1	1
IPI00135635	269	DEEISCSVIEIKYTGNASAIFIIPDQGRMQQ	1	81.3173	133.48	81.317	_YTGN(de)ASAIFIIPDQGR_	YTGN(1)ASAIFIIPDQGR	YTGN(81.32)ASAIFIIPDQGR	4	2	862	1	1	1	1
IPI00116913	2570	VVQRGIAAGARQIIANSSAIEETIIGHQGRI	1	62.8132	205.98	62.813	_QIIAN(de)SSAIEETIIGHQGR_	QIIAN(1)SSAIEETIIGHQGR	QIIAN(62.81)SSAIEETIIGHQGR	5	3	680	1	1	1	1
IPI00421237	134	QEIKTISDVQKFAEINGSAICSYNIKPSEYT	0.999949	42.9647	70.094	70.094	_FAEIN(de)GSAICSYNIKPSEYTITSK_	FAEIN(1)GSAICSYNIKPSEYTITSK	FAEIN(42.96)GSAICSYN(-42.96)	5	4	674	1	1	1	1
IPI00119522	111	IPRIQDIEITGSPVSNISAHIFSNISSIEKI	1	129.082	164.79	129.08	_IQ(de)DIEITGSPVSN(de)ISAHIFSN(de)ISSIEK_	IQDIEITGSPVSN(1)ISAHIFSN(1)IS!	IQDIEITGSPVSN(129.08)ISAHIFS	13	3	968	1	1	1	1
IPI00109996	110	EQWFRVNIRTIIGYYNQSAGGTHTIQWMYGC	1	45.9408	194.97	45.941	_TIIGYYN(de)Q(de)SAGGTHTIQ(de)WM(ox)YGCDVGSDGR_	TIIGYYN(1)QSAGGTHTIQWMYGCDVGSI	TIIGYYN(45.94)QSAGGTHTIQWMYG	7	3	1076	1	1	1	1
IPI00985828	124	EQSFRVDIRTAIRYYNQSAGGSHTIQWMAGC	1	38.9328	214.14	38.933	_YYN(de)QSAGGSHTIQWM(ox)AGCDVESDGR_	YYN(1)QSAGGSHTIQWMAGCDVESDGR	YYN(38.93)QSAGGSHTIQWMAGCDVE	3	4	702	1	1	1	1
IPI00123342	931	PRPKDKNGTRAEPPINASAGDQEEKVIPPAG	1	112.549	112.55	112.55	_DKN(de)GTRAEPPIN(de)ASAGDQEEK_	DKN(1)GTRAEPPIN(1)ASAGDQEEK	DKN(112.55)GTRAEPPIN(112.55)	12	2	1115	1	1	1	1
IPI00115458	160	MIKAINRGNYGWQAGNHSAFWGMTIDEGIRY	1	81.8069	185.61	81.807	_GN(de)YGWQAGN(de)HSAFWGM(ox)TIDEGIR_	GN(1)YGWQAGN(1)HSAFWGMTIDEGIR	GN(81.81)YGWQAGN(81.81)HSAFW	9	3	862	1	1	1	1
IPI00331214	320	QNPDNHCFCTEKVISNNCTSYGVIDIGKCKE	1	112.299	161.82	112.3	_VISN(de)N(de)CTSYGVIDIGK_	VISN(1)N(1)CTSYGVIDIGK	VISN(112.3)N(112.3)CTSYGVIDI	4	2	871	1	1	1	1
IPI00515360	904	GETCRCKNNVVGRICNECSDGSFHISKQNPD	1	93.4937	93.494	93.494	_ICN(de)ECSDGSFHISK_	ICN(1)ECSDGSFHISK	ICN(93.49)ECSDGSFHISK	3	2	828	1	1	1	1
IPI00119063	1768	PESKIYWISSGNHTINRCNIDGSEIEVIDTM	1	101.431	101.43	101.43	_IYWISSGN(de)HTIN(de)R_	IYWISSGN(1)HTIN(1)R	IYWISSGN(101.43)HTIN(101.43)	12	2	782	1	1	1	1
IPI00330833	733	RVARVTIGPICIRAFNECCTIANKIRKESPH	1	122.913	228.12	228.12	_AFN(de)ECCTIANK_	AFN(1)ECCTIANK	AFN(122.91)ECCTIAN(-122.91)K	3	2	665	1	1	1	1
IPI00108003	478	KVIIQSQIPIGTIKHNMTYFCKTHNSVGNSS	1	61.1666	76.713	61.167	_VIIQSQIPIGTIKHN(de)M(ox)TYFCK_	VIIQSQIPIGTIKHN(1)MTYFCK	VIIQSQIPIGTIKHN(61.17)MTYFCK	15	3	837		1	1	1
IPI00127238	411	AGQISQKETIVTIFDNRTWVRQGIVEDIEIP	1	113.629	113.63	113.63	_ETIVTIFDN(de)R_	ETIVTIFDN(1)R	ETIVTIFDN(113.63)R	9	2	605		1	1	1
IPI00762609	112	ERTRVKIRVYGQNINNETWSRIAFTEHERRR	0.5	0	183.89	183.89	_VYGQNIN(de)NETWSR_	VYGQNIN(0.5)N(0.5)ETWSR	VYGQN(-60.62)IN(0)N(0)ETWSR	8	2	791		1	1	1
IPI00653675	307	RYHGDPISCAKKITANSTWQPDKAKYVFKDV	1	129.772	129.77	129.77	_ITAN(de)STWQ(de)PDKAK_	ITAN(1)STWQPDKAK	ITAN(129.77)STWQPDKAK	4	2	731		1	1	1
IPI00625262	83	TIIIKFDGEPCDQSINITWFIKSADCYNEIY	1	159.345	159.34	159.34	_FDGEPCDQSIN(de)ITWFIK_	FDGEPCDQSIN(1)ITWFIK	FDGEPCDQSIN(159.34)ITWFIK	11	3	691		1	1	1
IPI00742385	66	REKIEEHGSMRAFVENITVIENSIVFKFHII	1	87.1601	339.58	87.16	_AFVEN(de)ITVIEN(de)SIVFK_	AFVEN(1)ITVIEN(1)SIVFK	AFVEN(87.16)ITVIEN(87.16)SIV	5	2	913		1	1	1
IPI00111385	169	RFGNKSAPSETIREVNITVIDRKICNDEKHY	1	133.069	133.07	133.07	_EVN(de)ITVIDRK_	EVN(1)ITVIDRK	EVN(133.07)ITVIDRK	3	2	594		1	1	1
IPI00323624	939	GVKKTIKVVPEGMRINKTVAIHTIDPEKIGQ	1	61.7674	110.55	61.767	_IN(de)KTVAIHTIDPEKIGQGGVQK_	IN(1)KTVAIHTIDPEKIGQGGVQK	IN(61.77)KTVAIHTIDPEKIGQGGVQ	2	3	783		1	1	1
IPI00461861	285	SIEMNKVMEIEKEIANATTKPEDRNDPMIIY	1	118.547	118.55	118.55	_VM(ox)EIEKEIAN(de)ATTKPEDR_	VMEIEKEIAN(1)ATTKPEDR	VMEIEKEIAN(118.55)ATTKPEDR	10	3	698		1	1	1
IPI00403586	270	YFKGNYDFVEAMIVNNHTSIDVERAAAIRAR	1	85.1951	125.88	125.88	_GNYDFVEAM(ox)IVN(de)N(de)HTSIDVER_	GNYDFVEAMIVN(1)N(1)HTSIDVER	GN(-74.97)YDFVEAMIVN(74.97)N	13	3	814		1	1	1
IPI00121776	51	GVSSISKDYCMYYNNNWTRIPSSIENATSIS	0.992587	18.2576	162.93	79.633	_DYCM(ox)YYN(de)NN(de)WTR_	DYCMYYN(0.504)N(0.504)N(0.993)	DYCMYYN(0)N(0)N(18.26)WTR	9	2	859		1	1	1
IPI00115892	119	DNMIIGPVKNVTKYGNMTQDHVMHIITRSGP	1	74.8534	89.624	74.853	_YGN(de)M(ox)TQDHVM(ox)HIITR_	YGN(1)MTQDHVMHIITR	YGN(74.85)MTQDHVMHIITR	3	3	617		1	1	1
IPI00459432	157	GKPYPPYSIAKFSWNNITNSIDIANISADFQ	0.561979	0	47.044	47.044	_FSWN(de)NITN(de)SIDIANISADFQGRPVDDPTGAFAN(de)GSITFK_	FSWN(0.562)N(0.562)ITN(0.45)SI	FSWN(0)N(0)ITN(0)SIDIAN(0)IS	5	4	1052		1	1	1
IPI00125497	600	VIITAEKPVGDISSPNETKYIISIDQDSVVK	1	124.633	124.63	124.63	_YSVIITAEKPVGDISSPN(de)ETK_	YSVIITAEKPVGDISSPN(1)ETK	YSVIITAEKPVGDISSPN(124.63)ET	18	3	750		1	1	1
IPI00405742	1053	AIERVTVEGMEYVFYNDTKVVFISPAVPEEP	1	83.3699	136.48	83.37	_VTVEGM(ox)EYVFYN(de)DTK_	VTVEGMEYVFYN(1)DTK	VTVEGMEYVFYN(83.37)DTK	12	2	906		1	1	1
IPI00272381	113	IENIIIKDMSDGFISNITIQRQYFPNDEDQV	1	82.202	198.74	82.202	_DM(ox)SDGFISN(de)ITIQR_	DMSDGFISN(1)ITIQR	DMSDGFISN(82.2)ITIQR	9	3	539		1	1	1
IPI00856221	590	PAFSIFPPDAPKIPENQTIPGEIPEHAGPAE	1	79.6897	79.69	79.69	_IPEN(de)QTIPGEIPEHAGPAEGVHDSR_	IPEN(1)QTIPGEIPEHAGPAEGVHDSR	IPEN(79.69)QTIPGEIPEHAGPAEGV	4	3	884		1	1	1
IPI00114842	38	YGYTNGIDDAHSIRCNITIKDPTPADPIWYE	1	79.6757	79.676	79.676	_CN(de)ITIKDPTPADPIWYEAK_	CN(1)ITIKDPTPADPIWYEAK	CN(79.68)ITIKDPTPADPIWYEAK	2	2	1117		1	1	1
IPI00138180	523	PVNPKFKFAIKNEDSNFTIINNHDNTANITV	0.990536	20.0928	122.53	122.53	_FAIKNEDSN(de)FTIIN(de)NHDN(de)TANITVK_	FAIKN(0.001)EDSN(0.991)FTIIN((	FAIKN(-29.61)EDSN(20.09)FTII	9	3	946		1	1	1
IPI00471081	527	PTVQDGIPPFNWNRFNETIHRGMPEVFDFNF	1	114.498	114.5	114.5	_FN(de)ETIHR_	FN(1)ETIHR	FN(114.5)ETIHR	2	2	459		1	1	1
IPI00223446	308	SVSSGAAAHRHVTDMNSTIHIIRTRISEREN	1	80.0144	138.63	80.014	_HVTDM(ox)N(de)STIHIIR_	HVTDMN(1)STIHIIR	HVTDMN(80.01)STIHIIR	6	3	519		1	1	1
IPI00387318	180	DRPIAPCGAIANSMFNDTIEIYIVANESDPK	0.999921	41.0497	104.9	104.9	_NEDRPIAPCGAIANSM(ox)FN(de)DTIEIYIVAN(de)ESDPKPIPIPIK_	NEDRPIAPCGAIANSMFN(1)DTIEIYIV/	N(-68.81)EDRPIAPCGAIAN(-41.0	18	4	1111		1	1	1
IPI00310059	147	EIPSDTHVYTKDIGRNVTIECPFKRENAPSK	1	123.792	123.79	123.79	_N(de)VTIECPFKR_	N(1)VTIECPFKR	N(123.79)VTIECPFKR	1	2	633		1	1	1
IPI00229198	182	DISFTFGEQNQARIGNFTIAYSAKPETADNQ	1	85.9025	126.25	126.25	_IGN(de)FTIAYSAKPETADNQR_	IGN(1)FTIAYSAKPETADNQR	IGN(85.9)FTIAYSAKPETADN(-85.	3	2	1049		1	1	1
IPI00742414	551	GQDHFHEIYKVTIIPNQTHYVVPKGEYISYF	1	97.4631	97.463	97.463	_VTIIPN(de)QTHYVVPK_	VTIIPN(1)QTHYVVPK	VTIIPN(97.46)QTHYVVPK	6	3	537		1	1	1
IPI00111286	188	PICIDCTDGFFSIQRNETHSICSACDESCKT	1	75.8677	75.868	75.868	_GPICIDCTDGFFSIQRN(de)ETHSICSACDESCK_	GPICIDCTDGFFSIQRN(1)ETHSICSACI	GPICIDCTDGFFSIQRN(75.87)ETHS	17	4	917		1	1	1
IPI00123652	97	QCFPAPKITCKDISGNETHFTGSEVGFIKPI	1	109.717	109.72	109.72	_DISGN(de)ETHFTGSEVGFIKPISCR_	DISGN(1)ETHFTGSEVGFIKPISCR	DISGN(109.72)ETHFTGSEVGFIKPI	5	3	851		1	1	1
IPI00475209	412	EIRYSCQQPYYKMIHNTTGVYTCSAHGTWTN	1	81.4614	180.8	180.8	_M(ox)IHN(de)TTGVYTCSAHGTWTNEVIKR_	MIHN(1)TTGVYTCSAHGTWTNEVIKR	MIHN(81.46)TTGVYTCSAHGTWTN(-	4	3	932		1	1	1
IPI00396840	209	RAKPIMEIIEKIGGWNITGPWAKDNFQDTIQ	1	62.0879	86.911	62.088	_IGGWN(de)ITGPWAK_	IGGWN(1)ITGPWAK	IGGWN(62.09)ITGPWAK	5	2	651		1	1	1
IPI00650038	160	SEDYGKNFKDITNIINNTFIRTEFGMAIGPE	0.978893	16.6635	120.84	120.84	_NFKDITNIIN(de)NTFIR_	NFKDITNIIN(0.979)N(0.021)TFIR	N(-61.65)FKDITN(-58.8)IIN(16	10	3	609		1	1	1
IPI00125813	90	IIINAEHGNSSIFIENSTFESFGYHSVSPDR	0.9951	22.469	95.108	95.108	_QENNIIIINAEHGN(de)SSIFIEN(de)STFESFGYHSVSPDR_	QEN(0.001)N(0.001)IIIIN(0.152)	QEN(-29.23)N(-29.23)IIIIN(-7	21	4	1018		1	1	1
IPI00464256	249	AAIKVNDSVPFHIGWNSTERSMRIQARYHDT	1	125.728	125.73	125.73	_AAAIKVN(de)DSVPFHIGWN(de)STER_	AAAIKVN(1)DSVPFHIGWN(1)STER	AAAIKVN(125.73)DSVPFHIGWN(12	17	3	772		1	1	1
IPI00109946	136	TIEVFDPVPKPSIEINKTEASTDSCHIRISC	1	58.9376	58.938	58.938	_ITIEVFDPVPKPSIEIN(de)KTEASTDSCHIR_	ITIEVFDPVPKPSIEIN(1)KTEASTDSCI	ITIEVFDPVPKPSIEIN(58.94)KTEA	17	4	825		1	1	1
IPI00378224	149	EPGVTEAEPVATEDANSTDSIKSPKVNCEER	1	64.7114	78.312	78.312	_ENIFGIQGAGGFQDREEEYYAEPGVTEAEPVATEDAN(de)STDSIKSPK_	ENIFGIQGAGGFQDREEEYYAEPGVTEAEI	EN(-64.71)IFGIQGAGGFQDREEEYY	37	4	1238		1	1	1
IPI00463492	1234	GKAEIQRGDPSIISVNGTDFTFRSAGAIIII	1	188.124	260.24	188.12	_GDPSIISVN(de)GTDFTFR_	GDPSIISVN(1)GTDFTFR	GDPSIISVN(188.12)GTDFTFR	9	3	576		1	1	1
IPI00323053	347	FIPERCQIDNIRAQTNYTCVAEIIYRGVKIV	1	66.2462	169.37	66.246	_AQTN(de)YTCVAEIIYR_	AQTN(1)YTCVAEIIYR	AQTN(66.25)YTCVAEIIYR	4	2	852		1	1	1
IPI00122584	54	PAPVCAQEGISCRVKNSTCIDDSWIHPKNIT	1	153.809	153.81	153.81	_VKN(de)STCIDDSWIHPK_	VKN(1)STCIDDSWIHPK	VKN(153.81)STCIDDSWIHPK	3	3	601		1	1	1
IPI00339885	801	VKENYAEIIDDGFIKNITAQICIDKKCPDYT	1	70.9174	150.94	70.917	_EN(de)YAEIIDDGFIKN(de)ITAQICIDKK_	EN(1)YAEIIDDGFIKN(1)ITAQICIDKI	EN(70.92)YAEIIDDGFIKN(70.92)	14	3	938		1	1	1

IPI00308971	1532	GHIFDISSISGRAGINASYSEKGIVFMSICE	1	90.827	90.827	90.827	_AGIN(de)ASYSEK_	AGIN(1)ASYSEK	AGIN(90.83)ASYSEK	4	2	521		1	1	1
IPI00224752	427	STGNVTNEIRVFHIHNESWVIITPKAKDQYA	1	84.4684	232.5	84.468	_VFHIHN(de)ESWVIITPK_	VFHIHN(1)ESWVIITPK	VFHIHN(84.47)ESWVIITPK	6	3	608		1	1	1
IPI00396840	538	TAVPDIYFENAMRFFNFSWRVTADQIRKAPN	1	130.272	130.27	130.27	_FFN(de)FSWR_	FFN(1)FSWR	FFN(130.27)FSWR	3	2	503		1	1	1
IPI00230084	56	WIQDIGIREDNEGVYNGSWGGRGEVITTYCP	0.996001	23.9634	121.02	121.02	_M(ox)STIIIHHPQYAWIQDIGIREDNEGVYN(de)GSWGGR_	MSTIIIHHPQYAWIQDIGIREDN(0.004)	MSTIIIHHPQYAWIQDIGIREDN(-23.	28	4	983		1	1	1
IPI00464256	239	IERYWISSRAAAIKVNDSVPFHIGWNSTERS	1	125.728	125.73	125.73	_AAAIKVN(de)DSVPFHIGWN(de)STER_	AAAIKVN(1)DSVPFHIGWN(1)STER	AAAIKVN(125.73)DSVPFHIGWN(12	7	3	772		1	1	1
IPI00338785	1384	ITKYFQMSIEAEKRVNASTTDPNSTVEQSAI	1	139.109	139.11	139.11	_VN(de)ASTTDPN(de)STVEQSAITR_	VN(1)ASTTDPN(1)STVEQSAITR	VN(139.11)ASTTDPN(139.11)STV	2	2	997		1	1	1
IPI00331032	97	EASFISQCMKYEVDWNQSTIDCVDPISSIAA	1	154.602	154.6	154.6	_YEVDWN(de)QSTIDCVDPISSIAAN(de)R_	YEVDWN(1)QSTIDCVDPISSIAAN(1)R	YEVDWN(154.6)QSTIDCVDPISSIAA	6	3	886		1	1	1
IPI00129168	4	____________MAINSSTEDGIKRIQDDCP	1	80.3118	82.543	80.312	_MAIN(de)SSTEDGIK_	MAIN(1)SSTEDGIK	MAIN(80.31)SSTEDGIK	4	2	634		1	1	1
IPI00469426	549	IPRGPDGTEGISSTVNGSTDPSAVTDIRVVN	1	246.773	246.77	246.77	_GPDGTEGISSTVN(de)GSTDPSAVTDIR_	GPDGTEGISSTVN(1)GSTDPSAVTDIR	GPDGTEGISSTVN(246.77)GSTDPSA	13	2	1218		1	1	1
IPI00469218	283	KVENKNRAIEIQFGMNASSSIFFIQGVRINM	1	71.5582	202.03	71.558	_AIEIQFGM(ox)N(de)ASSSIFFIQGVR_	AIEIQFGMN(1)ASSSIFFIQGVR	AIEIQFGMN(71.56)ASSSIFFIQGVR	9	3	778		1	1	1
IPI00122973	47	HPREAFIPQGGSVQVNCSSSCKEDISIGIET	1	162.09	162.09	162.09	_EAFIPQGGSVQVN(de)CSSSCK_	EAFIPQGGSVQVN(1)CSSSCK	EAFIPQGGSVQVN(162.09)CSSSCK	13	2	1028		1	1	1
IPI00223987	215	AIQDTRDIIIHSTGHNISRVTFMSAVSSQEK	1	88.0206	139.48	88.021	_DIIIHSTGHN(de)ISR_	DIIIHSTGHN(1)ISR	DIIIHSTGHN(88.02)ISR	10	3	489		1	1	1
IPI00135686	148	YGPGWVSMANAGKDTNGSQFFITTVKTSWID	1	133.66	133.66	133.66	_DTN(de)GSQFFITTVK_	DTN(1)GSQFFITTVK	DTN(133.66)GSQFFITTVK	3	2	730		1	1	1
IPI00134743	430	YGNGPGYKVVDGERENVSMVDYAHNNYQAQS	1	66.9694	110.06	110.06	_VVDGEREN(de)VSM(ox)VDYAHNNYQAQSAVPIR_	VVDGEREN(1)VSMVDYAHNNYQAQSAVP	VVDGEREN(66.97)VSMVDYAHN(-66	8	3	1060		1	1	1
IPI00128178	147	ISNSKADIIDTERFQNFSMATDQRINDIIIQ	1	106.492	106.49	106.49	_FQN(de)FSM(ox)ATDQR_	FQN(1)FSMATDQR	FQN(106.49)FSMATDQR	3	2	681		1	1	1
IPI00666034	2976	HIRVIQKITYESGFINYSKFEVESKVESQHV	1	154.357	154.36	154.36	_ITYESGFIN(de)YSK_	ITYESGFIN(1)YSK	ITYESGFIN(154.36)YSK	9	2	712		1	1	1
IPI00459432	185	DFQGRPVDDPTGAFANGSITFKVQAFSRSGR	0.999998	58.4203	119.54	119.54	_FSWN(de)NITNSIDIANISADFQGRPVDDPTGAFAN(de)GSITFK_	FSWN(0.495)N(0.495)ITN(0.009)S	FSWN(0)N(0)ITN(-17.45)SIDIAN	33	4	1052		1	1	1
IPI00221669	400	FSSAFQARISGNSIHNASIPEHIAYGSVITV	1	127.582	152.06	127.58	_ISGN(de)SIHN(de)ASIPEHIAYGSVITVK_	ISGN(1)SIHN(1)ASIPEHIAYGSVITV	ISGN(127.58)SIHN(127.58)ASIP	8	3	837		1	1	1
IPI00122411	498	YIDIESKVINEECKENESINIAARGDKMNVG	0.999991	50.6066	180.88	180.88	_VINEECKEN(de)ESINIAAR_	VINEECKEN(1)ESINIAAR	VIN(-86.67)EECKEN(50.61)ESIN	9	2	995		1	1	1
IPI00308990	317	RIDRNPSPDEIPQVGNISIKGNPFIDSESHS	1	74.7637	146.1	119.08	_IDRNPSPDEIPQVGN(de)ISIK_	IDRNPSPDEIPQVGN(1)ISIK	IDRN(-74.76)PSPDEIPQVGN(74.7	15	3	698		1	1	1
IPI00331318	198	QAQYWHDPIKESVYRNYSIFIADINQERCVN	1	170.22	259.68	259.68	_N(de)YSIFIADINQER_	N(1)YSIFIADINQER	N(170.22)YSIFIADIN(-170.22)Q	1	2	792		1	1	1
IPI00988518	1253	EGIYSCSAHSPIGKANTSIEIIIEGVRVKMN	1	108.716	160.48	108.72	_AN(de)TSIEIIIEGVR_	AN(1)TSIEIIIEGVR	AN(108.72)TSIEIIIEGVR	2	3	473		1	1	1
IPI00169617	174	AFNKVTDIEARVHGPNASHYTSIMTMITWER	1	97.0952	97.095	97.095	_VHGPN(de)ASHYTSIM(ox)TM(ox)ITWER_	VHGPN(1)ASHYTSIMTMITWER	VHGPN(97.1)ASHYTSIMTMITWER	5	3	789		1	1	1
IPI00947577	73	RNRFPESGDAARDNVNCSGVYEHEPIEIGKS	0.99998	47.0386	151.41	145.55	_DNVN(de)CSGVYEHEPIEIGK_	DNVN(1)CSGVYEHEPIEIGK	DN(-47.04)VN(47.04)CSGVYEHEP	4	3	688		1	1	1
IPI00760106	58	PGEMKKEIRIAGGENNCSGRVEIKIHDKWGT	1	82.5498	89.624	82.55	_IAGGEN(de)N(de)CSGRVEIK_	IAGGEN(1)N(1)CSGRVEIK	IAGGEN(82.55)N(82.55)CSGRVEI	7	2	803		1	1	1
IPI00153317	250	TEACGQKITFFNSTINTSGIVAQGEAIPIPG	0.850582	7.55314	80.067	80.067	_ITFFNSTIN(de)TSGIVAQGEAIPIPGAHRPGIVTK_	ITFFN(0.149)STIN(0.851)TSGIVA	ITFFN(-7.55)STIN(7.55)TSGIVA	9	4	853		1	1	1
IPI00116913	3261	SRIIIGGIPVSGTFHNFSGCISNVFVQRIRG	0.998694	28.8364	95.48	95.48	_IIIGGIPVSGTFHN(de)FSGCISNVFVQR_	IIIGGIPVSGTFHN(0.999)FSGCISN(	IIIGGIPVSGTFHN(28.84)FSGCISN	14	3	941		1	1	1
IPI00222489	276	DIISSSSEMVHRIVANISGCAAVNSETIMCC	1	111.645	204.23	111.65	_IVAN(de)ISGCAAVN(de)SETIM(ox)CCIR_	IVAN(1)ISGCAAVN(1)SETIMCCIR	IVAN(111.65)ISGCAAVN(111.65)	4	3	786		1	1	1
IPI00223231	133	IAGFPTVRFFQAFTKNGSGATIPGAGANVQT	1	92.6894	137.23	137.23	_FFQAFTKN(de)GSGATIPGAGANVQTIR_	FFQAFTKN(1)GSGATIPGAGANVQTIR	FFQAFTKN(92.69)GSGATIPGAGAN(	8	3	852		1	1	1
IPI00515360	3011	PEHEASFKITVPSSQNSSFRIRSPVISIEPP	1	80.6901	80.69	80.69	_ITVPSSQN(de)SSFR_	ITVPSSQN(1)SSFR	ITVPSSQN(80.69)SSFR	8	2	662		1	1	1
IPI00387318	190	ANSMFNDTIEIYIVANESDPKPIPIPIKKKG	0.999995	53.4536	104.9	104.9	_NEDRPIAPCGAIANSM(ox)FN(de)DTIEIYIVAN(de)ESDPKPIPIPIK_	NEDRPIAPCGAIANSMFN(1)DTIEIYIV	N(-68.81)EDRPIAPCGAIAN(-41.0	28	4	1111		1	1	1
IPI00128269	41	AVMNRHVCPVENWSYNESCSPDPAEQGGPKS	0.952679	13.0389	163.95	163.95	_HVCPVENWSYN(de)ESCSPDPAEQGGPK_	HVCPVEN(0.047)WSYN(0.953)ESCS	HVCPVEN(-13.04)WSYN(13.04)ES	11	3	949		1	1	1
IPI00118291	239	FGTVIFVSKVIEEPKNVSCETRDFKTIDCSW	1	116.511	116.51	116.51	_VIEEPKN(de)VSCETR_	VIEEPKN(1)VSCETR	VIEEPKN(116.51)VSCETR	7	2	781		1	1	1
IPI00118987	99	SFAEIKHKFIWSEPQNCSATKSNYIRGTGPY	1	133.148	133.15	133.15	_FIWSEPQN(de)CSATK_	FIWSEPQN(1)CSATK	FIWSEPQN(133.15)CSATK	8	2	785		1	1	1
IPI00153632	471	DTWKTIISEVRFVHVNTSAIIKISGAHIPDW	1	102.4	102.4	102.4	_FVHVN(de)TSAIIK_	FVHVN(1)TSAIIK	FVHVN(102.4)TSAIIK	5	2	615		1	1	1
IPI00894972	270	KVIVIITDGEASDKGNISAAHDITRYIIGIG	1	101.637	101.64	101.64	_VIVIITDGEASDKGN(de)ISAAHDITR_	VIVIITDGEASDKGN(1)ISAAHDITR	VIVIITDGEASDKGN(101.64)ISAAH	15	3	833		1	1	1
IPI00153143	96	ISKDDIEYAFEKWVGNWTYEIKKIPFWTSYS	1	74.9442	98.898	74.944	_WVGN(de)WTYEIK_	WVGN(1)WTYEIK	WVGN(74.94)WTYEIK	4	2	649	1		1	1
IPI00121378	361	IGDTIPVSCTISASRNATVVWMKDNIRIRSS	1	103.563	103.56	103.56	_N(de)ATVVWM(ox)K_	N(1)ATVVWMK	N(103.56)ATVVWMK	1	2	483	1		1	1
IPI00119095	151	CHCPFKEGTYSIPTSNFTVPDIEIPSWISTG	1	96.6486	133.58	133.58	_EGTYSIPTSN(de)FTVPDIEIPSWISTGNYR_	EGTYSIPTSN(1)FTVPDIEIPSWISTGN	EGTYSIPTSN(96.65)FTVPDIEIPSW	10	2	1573	1		1	1
IPI00311405	139	CEFATFPTGNRESQINITVMAKPTNWIEGTR	1	106.492	106.49	106.49	_ESQIN(de)ITVM(ox)AK_	ESQIN(1)ITVMAK	ESQIN(106.49)ITVMAK	5	2	626	1		1	1
IPI00338785	1391	SIEAEKRVNASTTDPNSTVEQSAITRDRVED	1	139.109	139.11	139.11	_VN(de)ASTTDPN(de)STVEQSAITR_	VN(1)ASTTDPN(1)STVEQSAITR	VN(139.11)ASTTDPN(139.11)STV	9	2	997	1		1	1
IPI00128984	58	IEKQRQQCIEEAQIENETTGCSKMWDNITCW	1	158.576	158.58	158.58	_QQCIEEAQIEN(de)ETTGCSK_	QQCIEEAQIEN(1)ETTGCSK	QQCIEEAQIEN(158.58)ETTGCSK	11	2	1063	1		1	1
IPI00138342	79	RFAPPQPAEPWSFVKNATSYPPMCSQDAGWA	1	52.3715	52.372	52.372	_N(de)ATSYPPM(ox)CSQDAGWAK_	N(1)ATSYPPMCSQDAGWAK	N(52.37)ATSYPPMCSQDAGWAK	1	2	951	1		1	1
IPI00315576	346	HPEFRVNITIRWSFSNGTSWQKERVQVWPGP	1	103.433	103.43	103.43	_WSFSN(de)GTSWQK_	WSFSN(1)GTSWQK	WSFSN(103.43)GTSWQK	5	2	665	1		1	1
IPI00114350	154	AEQTRKITDVETQVINQTSRIEIQIIENSIS	1	91.6567	91.657	91.657	_KITDVETQVIN(de)QTSR_	KITDVETQVIN(1)QTSR	KITDVETQVIN(91.66)QTSR	11	2	867	1		1	1
IPI00116913	926	YAHMMAIQPRIVARINVTSPDIFRIVFRYVN	1	83.3966	83.397	83.397	_IN(de)VTSPDIFR_	IN(1)VTSPDIFR	IN(83.4)VTSPDIFR	2	2	582	1		1	1
IPI00466999	239	TICIVTGTRKMISAFNATSGKVIWTINPNHI	1	96.6404	96.64	96.64	_M(ox)ISAFN(de)ATSGK_	MISAFN(1)ATSGK	MISAFN(96.64)ATSGK	6	2	572	1		1	1
IPI00120848	258	FNSISSITDDTFCKANDTRYIRERIEEIRIE	1	100.966	128.38	100.97	_VIHIQFN(de)SISSITDDTFCKAN(de)DTR_	VIHIQFN(1)SISSITDDTFCKAN(1)DT	VIHIQFN(100.97)SISSITDDTFCKA	21	4	697	1		1	1
IPI00122973	469	EMNGTYVCHAFSSHGNVTRNVYITVIYHSQN	1	71.735	73.138	71.735	_SVKQEM(ox)N(de)GTYVCHAFSSHGN(de)VTR_	SVKQEMN(1)GTYVCHAFSSHGN(1)VTR	SVKQEMN(71.73)GTYVCHAFSSHGN(	20	3	876	1		1	1
IPI00120187	166	FSKIRHIERIYIDHNNITRMPGPIPRSIREI	1	60.5976	141.88	60.598	_IYIDHN(de)N(de)ITR_	IYIDHN(1)N(1)ITR	IYIDHN(60.6)N(60.6)ITR	7	2	631	1		1	1
IPI00114457	82	EVPPGPIPEAVIAIYNSTRDRVAGESADPEP	1	67.8798	67.88	67.88	_IASPPSQGEVPPGPIPEAVIAIYN(de)STR_	IASPPSQGEVPPGPIPEAVIAIYN(1)ST	IASPPSQGEVPPGPIPEAVIAIYN(67.	24	5	553	1		1	1
IPI00856861	232	FIRPVDDIWSIIKKFNSTQIAAMAPEHEEPR	1	57.5196	162.59	57.52	_KFN(de)STQIAAM(ox)APEHEEPR_	KFN(1)STQIAAMAPEHEEPR	KFN(57.52)STQIAAMAPEHEEPR	3	3	692	1		1	1
IPI00117312	164	FKFSRDVFIPKPSWGNHTPIFRDAGMQIQGY	1	90.3665	90.367	90.367	_DVFIPKPSWGN(de)HTPIFR_	DVFIPKPSWGN(1)HTPIFR	DVFIPKPSWGN(90.37)HTPIFR	11	3	671	1		1	1
IPI00128178	253	AHVEQEVKQEVRVINNITNDIRIKDWEHSQT	0.999733	35.7403	275.84	275.84	_VINN(de)ITNDIR_	VINN(1)ITNDIR	VIN(-35.74)N(35.74)ITN(-77.5	4	2	587	1		1	1
IPI00626793	824	PAVVGIKIDVNSWIENFTKTSIRDPCAGPVC	1	81.6489	190.37	190.37	_IDVNSWIEN(de)FTK_	IDVNSWIEN(1)FTK	IDVN(-81.65)SWIEN(81.65)FTK	9	2	734	1		1	1
IPI00114206	553	RIRITDNMFCAGFKVNDTKRGDACEGDSGGP	1	82.4546	116.35	116.35	_ITDNM(ox)FCAGFKVN(de)DTK_	ITDNMFCAGFKVN(1)DTK	ITDN(-82.45)MFCAGFKVN(82.45)	13	2	939	1		1	1
IPI00876541	270	HTYKMDYNSFQGTPSNETKFTVIPEFIFEGD	1	104.63	172.91	172.91	_M(ox)DYNSFQGTPSN(de)ETK_	MDYNSFQGTPSN(1)ETK	MDYN(-104.63)SFQGTPSN(104.63	12	2	868	1		1	1
IPI00130764	289	VRCPMAGISHTTWHSNRTIYDTCIEPWIS__	1	87.5193	87.519	87.519	_CPM(ox)AGISHTTWHSN(de)R_	CPMAGISHTTWHSN(1)R	CPMAGISHTTWHSN(87.52)R	14	2	886	1		1	1
IPI00346062	237	IGHPNSTTSYKDAHFNSTITEFGETINNFVE	0.999984	49.2477	83.723	83.723	_DAHFN(de)STITEFGETINNFVER_	DAHFN(1)STITEFGETINNFVER	DAHFN(49.25)STITEFGETIN(-49.	5	3	815	1		1	1
IPI00751912	676	TKIDGATVDTHHIPVNVTIRRIAHGADAVAA	1	59.3449	73.296	59.345	_IDGATVDTHHIPVN(de)VTIR_	IDGATVDTHHIPVN(1)VTIR	IDGATVDTHHIPVN(59.34)VTIR	14	3	654	1		1	1
IPI01026704	1102	IIWKPTFIKAHFSSINITIRGEIQSENSSIT	1	87.0009	87.001	87.001	_AHFSSIN(de)ITIR_	AHFSSIN(1)ITIR	AHFSSIN(87)ITIR	7	2	630	1		1	1
IPI00109996	200	EGECVEWIHRYIKNGNATIIRTDSPKAHVTH	1	147.623	170.89	147.62	_YIKN(de)GN(de)ATIIR_	YIKN(1)GN(1)ATIIR	YIKN(147.62)GN(147.62)ATIIR	6	2	633	1		1	1
IPI00985828	214	EGECVEWIRRYIKNGNATIIRTDPPKAHVTH	1	147.623	170.89	147.62	_YIKN(de)GN(de)ATIIR_	YIKN(1)GN(1)ATIIR	YIKN(147.62)GN(147.62)ATIIR	6	2	633	1		1	1
IPI00138209	392	TVQDKDIVNSPNWRANYTIIKGNENGNFKIV	1	114.498	114.5	114.5	_AN(de)YTIIK_	AN(1)YTIIK	AN(114.5)YTIIK	2	2	412	1		1	1
IPI00123814	194	VVSIENGYKYMAIAINRTGRSIVYSCEWPIY	1	110.633	110.63	110.63	_YM(ox)AIAIN(de)R_	YMAIAIN(1)R	YMAIAIN(110.63)R	7	2	485	1		1	1
IPI00111793	415	ECRDYATGFCCRCVANYTGNGRQCVAEGSPQ	1	91.5493	108.7	91.549	_CVAN(de)YTGN(de)GR_	CVAN(1)YTGN(1)GR	CVAN(91.55)YTGN(91.55)GR	4	2	557	1		1	1
IPI00221669	515	APITRKHYQVTGYGINGTGDSNDFWRIEVVN	1	92.3071	98.443	92.307	_KHYQVTGYGIN(de)GTGDSN(de)DFWR_	KHYQVTGYGIN(1)GTGDSN(1)DFWR	KHYQVTGYGIN(92.31)GTGDSN(92.	11	3	806	1		1	1
IPI00113539	528	QIRDQCIVDDITYNVNDTFHKRHEEGHMINC	0.999112	30.5108	199.41	186.45	_DQCIVDDITYNVN(de)DTFHK_	DQCIVDDITYN(0.001)VN(0.999)DT	DQCIVDDITYN(-30.51)VN(30.51)	13	2	1099	1		1	1
IPI00848693	1572	NHNTIGTRRQDPVSWNKTFEDISRSVIETRY	1	89.9108	89.911	89.911	_RQDPVSWN(de)KTFEDISR_	RQDPVSWN(1)KTFEDISR	RQDPVSWN(89.91)KTFEDISR	8	3	660	1		1	1
IPI00136012	605	YTVSSSPSCYMYVYVNTTEVAIEQDIAYIQE	1	215.081	215.08	215.08	_VYTVSSSPSCYM(ox)YVYVN(de)TTEVAIEQDIAYIQEIK_	VYTVSSSPSCYMYVYVN(1)TTEVAIEQD	VYTVSSSPSCYMYVYVN(215.08)TTE	17	4	996	1		1	1
IPI00338209	204	IRPHISASTIRQIFFNGTETIRSQDPFPWPA	1	56.2251	98.898	56.225	_QIFFN(de)GTETIR_	QIFFN(1)GTETIR	QIFFN(56.23)GTETIR	5	2	664	1		1	1
IPI00114206	413	VITAAHCIIYPPWDKNFTENDIIVRIGKHSR	1	79.9422	127.58	79.942	_WVITAAHCIIYPPWDKN(de)FTEN(de)DIIVR_	WVITAAHCIIYPPWDKN(1)FTEN(1)DI	WVITAAHCIIYPPWDKN(79.94)FTEN	17	3	1059	1		1	1
IPI00677395	68	VNDKSGPIVFRKTMFNSTEIKFSVKSFSCSG	1	85.9581	85.958	85.958	_TM(ox)FN(de)STEIK_	TMFN(1)STEIK	TMFN(85.96)STEIK	4	2	544	1		1	1
IPI00113539	1006	QQTTKIDAPTNIQFVNETDRTVIVTWTPPRA	1	148.409	209.66	148.41	_IDAPTN(de)IQFVN(de)ETDR_	IDAPTN(1)IQFVN(1)ETDR	IDAPTN(148.41)IQFVN(148.41)E	11	2	868	1		1	1
IPI00113539	542	VNDTFHKRHEEGHMINCTCFGQGRGRWKCDP	1	65.2255	125.5	65.225	_RHEEGHM(ox)IN(de)CTCFGQGR_	RHEEGHMIN(1)CTCFGQGR	RHEEGHMIN(65.23)CTCFGQGR	9	2	1053	1		1	1
IPI00120953	396	RDCESTIPYICKRDINHTAQGIIEKDSWKYH	1	106.377	106.38	106.38	_DIN(de)HTAQGIIEKDSWK_	DIN(1)HTAQGIIEKDSWK	DIN(106.38)HTAQGIIEKDSWK	3	2	928	1		1	1
IPI00308971	430	CSSGFQRMSVINFECNKTAGKDGRGEPVFTG	0.999605	34.0317	158.52	94.465	_M(ox)SVINFECN(de)K_	MSVINFECN(1)K	MSVIN(-34.03)FECN(34.03)K	9	2	630	1		1	1
IPI00894972	668	RIKPITPQFQGRIIANISYTIQIDGHRMRSR	1	104.41	104.41	104.41	_IIAN(de)ISYTIQIDGHR_	IIAN(1)ISYTIQIDGHR	IIAN(104.41)ISYTIQIDGHR	4	3	572	1		1	1
IPI00320065	38	QKNYTFRCIQMSSFANRSWSRTDSVVWIGDI	1	77.379	150.04	77.379	_CIQM(ox)SSFAN(de)R_	CIQMSSFAN(1)R	CIQMSSFAN(77.38)R	9	2	616	1		1	1
IPI00221418	169	QGEAIIQKFQQIIIRNISVVVATHSPTIAKT	1	64.7792	64.779	64.779	_QGEAIIQKFQQIIIRN(de)ISVVVATHSPTIAK_	QGEAIIQKFQQIIIRN(1)ISVVVATHSP	QGEAIIQKFQQIIIRN(64.78)ISVVV	16	4	827	1		1	1
IPI00408850	283	AAGHQVPYKVIMKQVNGSVSGSQWNKDQQAP	0.999981	47.2073	79.089	79.089	_QVN(de)GSVSGSQWNK_	QVN(1)GSVSGSQWNK	QVN(47.21)GSVSGSQWN(-47.21)K	3	2	696	1		1	1
IPI00173158	178	IDFHIEFHGDRIIFANVSVRDVKPHSIDGIR	1	64.0402	121.73	64.04	_IIFAN(de)VSVR_	IIFAN(1)VSVR	IIFAN(64.04)VSVR	5	2	510	1		1	1
IPI00109946	186	KKSPGYVIDIIVTPQNKSTFYTCQVSNPVSS	1	118.522	118.52	118.52	_KSPGYVIDIIVTPQN(de)K_	KSPGYVIDIIVTPQN(1)K	KSPGYVIDIIVTPQN(118.52)K	15	3	592	1		1	1
IPI00119065	1349	IRHAHSQSTEAERRANASTFAVPSPVSNSAD	1	68.8496	94.845	94.845	_RAN(de)ASTFAVPSPVSNSADTR_	RAN(1)ASTFAVPSPVSNSADTR	RAN(68.85)ASTFAVPSPVSN(-68.8	3	3	684	1		1	1
IPI00319040	335	IVVKVIAQHQNIIFANSSSSMRYVIISNDNA	0.999975	45.9533	115.38	115.38	_VIAQHQNIIFAN(de)SSSSM(ox)R_	VIAQHQNIIFAN(1)SSSSMR	VIAQHQN(-45.95)IIFAN(45.95)S	12	3	674	1		1	1
IPI00322867	589	EIIAKRMKTEGEERANISSQVIKMSIDYHFV	1	108.431	108.43	108.43	_AN(de)ISSQVIK_	AN(1)ISSQVIK	AN(108.43)ISSQVIK	2	2	481	1		1	1
IPI00119063	2906	EAPKNPHCTSPEHKCNASSQFICSSGRCVAE	1	126.485	126.48	126.48	_CN(de)ASSQFICSSGR_	CN(1)ASSQFICSSGR	CN(126.48)ASSQFICSSGR	2	2	738	1		1	1
IPI00127406	551	IFDNPVDWREQYIHENYSRAIDGEGIVEQPC	1	79.492	104.24	79.492	_EQYIHEN(de)YSR_	EQYIHEN(1)YSR	EQYIHEN(79.49)YSR	7	2	670	1		1	1
IPI00129159	520	SVFYTKEGVDRERVENTSQPGEMQVTIQNIM	1	65.8549	105.32	105.32	_ERVEN(de)TSQPGEM(ox)QVTIQNIM(ox)PATVYIFK_	ERVEN(1)TSQPGEMQVTIQNIMPATVYI	ERVEN(65.85)TSQPGEMQVTIQN(-6	5	3	1086	1		1	1
IPI00469426	319	EEVPDANGTKRIPVTNISQIHKNSIVSVDPP	1	80.6901	80.69	80.69	_RIPVTN(de)ISQIHK_	RIPVTN(1)ISQIHK	RIPVTN(80.69)ISQIHK	6	3	470	1		1	1
IPI00124666	386	RPGSCITDSIRSRGYNSSQDIPSIVIDFVKI	1	81.6559	195.77	81.656	_SRGYN(de)SSQDIPSIVIDFVK_	SRGYN(1)SSQDIPSIVIDFVK	SRGYN(81.66)SSQDIPSIVIDFVK	5	3	709	1		1	1
IPI00411145	1415	YYTDVFIDVIRRADINGSNMETVIGHGIKTT	0.858358	7.82478	111.33	111.33	_RADIN(de)GSNM(ox)ETVIGHGIK_	RADIN(0.858)GSN(0.142)METVIGH	RADIN(7.82)GSN(-7.82)METVIGH	5	3	644	1		1	1

IPI00626537	105	FQVIMNSEKKCEVICNQSNKPITITVEQSRI	0.985768	18.4049	121.82	121.82 _KCEVICN(de)QSNKPITITVEQSR_	KCEVICN(0.986)QSN(0.014)KPITI	KCEVICN(18.4)QSN(-18.4)KPITI	7	3	836	1	1	1
IPI00119822	111	GQISERNIMTIVHFYNKSMDDSHTIQWIQDC	1	87.0429	94.767	94.767 _NIM(ox)TIVHFYN(de)K_	NIMTIVHFYN(1)K	N(-87.04)IMTIVHFYN(87.04)K	10	2	699	1	1	1
IPI00480532	52	EGPRVIETIPANYSINSSKKIKTVIPSNPIS	1	103.387	103.39	103.39 _VIETIPAN(de)YSIN(de)SSK_	VIETIPAN(1)YSIN(1)SSK	VIETIPAN(103.39)YSIN(103.39)	12	2	819	1	1	1
IPI00555055	73	AIIEYITAEVIEIAGNASKDIKVKRITPRHI	1	121.505	121.5	121.5 _VGATAAVYSAAIIEYITAEVIEIAGN(de)ASK_	VGATAAVYSAAIIEYITAEVIEIAGN(1)	VGATAAVYSAAIIEYITAEVIEIAGN(1	26	3	966	1	1	1
IPI00356462	328	SQQERKISIIQKKVDNASIVADDMRNAYISI	1	92.7725	92.773	92.773 _KVDN(de)ASIVADDM(ox)R_	KVDN(1)ASIVADDMR	KVDN(92.77)ASIVADDMR	4	2	726	1	1	1
IPI00314779	570	EGETAPISRAGVVVFNCSIRQIRSPSGFQDQ	1	130.098	130.1	130.1 _AGVVVFN(de)CSIR_	AGVVVFN(1)CSIR	AGVVVFN(130.1)CSIR	7	2	612	1	1	1
IPI00311405	286	ADANPPATEYHWTTINGSIPKGVEAQNRTIF	1	146.396	184.56	184.56 _ADANPPATEYHWTTIN(de)GSIPK_	ADANPPATEYHWTTIN(1)GSIPK	ADAN(-146.4)PPATEYHWTTIN(146	16	2	1143	1	1	1
IPI00320675	116	IKFSHNGTCAAEGKFNVSIIYGRTKTEGIVQ	1	59.4259	94.297	59.426 _FN(de)VSIIYGR_	FN(1)VSIIYGR	FN(59.43)VSIIYGR	2	2	535	1	1	1
IPI00162870	62	QGTFSPEDNSIKWFHNESIIPHQDANYVIQS	0.999999	61.6262	152.01	152.01 _WFHN(de)ESIIPHQDANYVIQSAR_	WFHN(1)ESIIPHQDANYVIQSAR	WFHN(61.63)ESIIPHQDAN(-61.63	4	3	843	1	1	1
IPI00223352	246	INKIQGYRWVDGASINFSHWNSGEPNDSRGH	1	69.7632	157.47	69.763 _WVDGASIN(de)FSHWN(de)SGEPN(de)DSR_	WVDGASIN(1)FSHWN(1)SGEPN(1)DS	WVDGASIN(69.76)FSHWN(69.76)S	8	2	1190	1	1	1
IPI00785452	276	GNEIRQFTRFMTERINVSHAGAPIGEEYIIV	1	111.769	111.77	111.77 _IN(de)VSHAGAPIGEEYIIVFSR_	IN(1)VSHAGAPIGEEYIIVFSR	IN(111.77)VSHAGAPIGEEYIIVFSR	2	3	725	1	1	1
IPI00118674	434	QAIPPSSIQRFIRARNISGVVIADHSGSFHN	1	85.2983	123.59	113.33 _N(de)ISGVVIADHSGSFHNR_	N(1)ISGVVIADHSGSFHNR	N(85.3)ISGVVIADHSGSFHN(-85.3	1	2	906	1	1	1
IPI00162870	164	YFHENSEIIIPKATHNDSGSYFCRGIIGHNN	1	80.4686	80.469	80.469 _ATHN(de)DSGSYFCR_	ATHN(1)DSGSYFCR	ATHN(80.47)DSGSYFCR	4	2	708	1	1	1
IPI00311159	189	QFEVICPAPRPGAASNISFQAPFRISSSAID	1	84.9747	84.975	84.975 _YQFEVICPAPRPGAASN(de)ISFQAPFR_	YQFEVICPAPRPGAASN(1)ISFQAPFR	YQFEVICPAPRPGAASN(84.97)ISFQ	17	3	942	1	1	1
IPI00114044	78	AENNEIISNIRDSVINISESVEDGPRGSPGN	1	139.858	139.86	139.86 _DSVIN(de)ISESVEDGPR_	DSVIN(1)ISESVEDGPR	DSVIN(139.86)ISESVEDGPR	5	3	540	1	1	1
IPI00130654	153	PFPTIDPEEKCQAYKNNSESFIHIYMYEVAR	0.818761	6.54905	142.94	142.94 _CQAYKN(de)NSESFIHIYM(ox)YEVAR_	CQAYKN(0.819)N(0.181)SESFIHIY	CQAYKN(6.55)N(-6.55)SESFIHIY	6	3	881	1	1	1
IPI00380296	175	HVIVAGAATWSIKIHNGSEEAIAQYKMNITS	1	102.4	102.4	102.4 _IHN(de)GSEEAIAQYK_	IHN(1)GSEEAIAQYK	IHN(102.4)GSEEAIAQYK	3	3	488	1	1	1
IPI00930882	302	MNAPIYIAEWQNITKNISEDRIIIAGTESSD	1	74.0167	107.72	107.72 _IM(ox)NAPIYIAEWQN(de)ITKN(de)ISEDR_	IMNAPIYIAEWQN(1)ITKN(1)ISEDR	IMN(-67.82)APIYIAEWQN(67.82)	17	3	880	1	1	1
IPI00153202	690	IKPRVSFYFFVTSPQNVSDVIPRSEVEDAIR	1	81.1566	81.157	81.157 _VSFYFFVTSPQN(de)VSDVIPR_	VSFYFFVTSPQN(1)VSDVIPR	VSFYFFVTSPQN(81.16)VSDVIPR	12	3	735	1	1	1
IPI00111163	329	QPIVAVADEGWYIIQNKSDDFIIGNHGYDNA	1	199.148	199.15	199.15 _VQPIVAVADEGWYIIQN(de)K_	VQPIVAVADEGWYIIQN(1)K	VQPIVAVADEGWYIIQN(199.15)K	17	3	682	1	1	1
IPI00129526	107	SIYKNKEIFIREIISNASDAIDKIRIISITD;	1	78.9316	105.99	78.932 _EIISN(de)ASDAIDKIR_	EIISN(1)ASDAIDKIR	EIISN(78.93)ASDAIDKIR	5	2	773	1	1	1
IPI00987265	1398	VCDCDHGICQEGIRGNGSCVCHAGWQGIRCD	1	120.663	120.66	120.66 _GN(de)GSCVCHAGWQGIR_	GN(1)GSCVCHAGWQGIR	GN(120.66)GSCVCHAGWQGIR	2	2	830	1	1	1
IPI00464256	510	PFFSIAEVRVGYQSQNISCFFRIVDRDSVWG	1	74.7687	150.12	74.769 _VGYQSQN(de)ISCFFR_	VGYQSQN(1)ISCFFR	VGYQSQN(74.77)ISCFFR	7	2	804	1	1	1
IPI00132600	159	EIEYYVGQSFANAMYNACRDVEAPSSNEKAI	0.999975	46.0067	136.66	136.66 _EIEYYVGQSFANAM(ox)YN(de)ACR_	EIEYYVGQSFANAMYN(1)ACR	EIEYYVGQSFAN(-46.01)AMYN(46.	16	2	1152	1	1	1
IPI00221859	286	QGNYSAGIPRGTYRVNITYNYPVRAFGGHKI	1	70.3298	176.95	176.95 _VN(de)ITYNYPVR_	VN(1)ITYNYPVR	VN(70.33)ITYN(-70.33)YPVR	2	2	620		1	1
IPI00120953	1157	NVSDTSAIPSTIEYGNRTYKIIRGNMTWYAA	1	124.399	124.4	124.4 _M(ox)QDTIEHHVN(de)VSDTSAIPSTIEYGN(de)R_	MQDTIEHHVN(1)VSDTSAIPSTIEYGN(	MQDTIEHHVN(124.4)VSDTSAIPSTI	25	3	978		1	1
IPI00399958	131	FDMNQDGIISWDEYRNVTYGTYIDDPDPDDG	1	176.552	243.97	243.97 _N(de)VTYGTYIDDPDPDDGFNYK_	N(1)VTYGTYIDDPDPDDGFNYK	N(176.55)VTYGTYIDDPDPDDGFN(-	1	2	1155		1	1
IPI00221798	140	YDTDRDGRVGWEEIRNATYGHYEPGEEFHDV	1	147.348	147.35	147.35 _N(de)ATYGHYEPGEEFHDVEDAETYKK_	N(1)ATYGHYEPGEEFHDVEDAETYKK	N(147.35)ATYGHYEPGEEFHDVEDAE	1	3	944		1	1
IPI00130271	202	NFTVIIKNNIHFPGHNYTTRNIIPTMNGSCT	1	103.829	136.27	136.27 _NNIHFPGHN(de)YTTR_	NNIHFPGHN(1)YTTR	N(-103.83)N(-103.83)IHFPGHN(	9	2	786		1	1
IPI00128862	107	RNCHNSSSRVHITVCNITSRRRIPYTQCRYQ	1	103.439	103.44	103.44 _VHITVCN(de)ITSR_	VHITVCN(1)ITSR	VHITVCN(103.44)ITSR	7	2	651		1	1
IPI00320605	626	PMCEDCPSCGSHCRDNHTSCAECIKFDKGPF	1	93.8392	93.839	93.839 _DN(de)HTSCAECIK_	DN(1)HTSCAECIK	DN(93.84)HTSCAECIK	2	2	668		1	1
IPI00674255	901	TIFHGENKQFNCSFENITRNQDITTIICKIK	1	122.326	122.33	122.33 _QFN(de)CSFEN(de)ITR_	QFN(1)CSFEN(1)ITR	QFN(122.33)CSFEN(122.33)ITR	8	2	709		1	1
IPI00129968	221	DAHSNEKYIINGSHANETRIKIKHIIEEDGG	1	89.4303	89.43	89.43 _YIIN(de)GSHAN(de)ETR_	YIIN(1)GSHAN(1)ETR	YIIN(89.43)GSHAN(89.43)ETR	9	2	689		1	1
IPI00134013	58	TTQYIWHRFSTEIGYNGTRHRENCGNQSADP	1	173.721	173.72	173.72 _FSTEIGYN(de)GTR_	FSTEIGYN(1)GTR	FSTEIGYN(173.72)GTR	8	2	623		1	1
IPI00653847	57	AEEIVRSSIAWESRANDTREEAGIPAAGEDE	1	80.1418	80.142	80.142 _AN(de)DTREEAGIPAAGEDETSWTER_	AN(1)DTREEAGIPAAGEDETSWTER	AN(80.14)DTREEAGIPAAGEDETSWT	2	3	836		1	1
IPI00221426	379	IDIGPTIIDIAGYDINKTQMDGMSIIPIIKG	0.995704	23.6511	54.003	54.003 _M(ox)IVSNIDIGPTIIDIAGYDIN(de)KTQM(ox)DGM(ox)SIIPIIK_	MIVSN(0.004)IDIGPTIIDIAGYDIN(	MIVSN(-23.65)IDIGPTIIDIAGYDI	21	4	964		1	1
IPI00453524	174	SGCIGGSPICAIRWINETQIKIVADSQYPFK	1	149.816	149.82	149.82 _WIN(de)ETQIK_	WIN(1)ETQIK	WIN(149.82)ETQIK	3	2	517		1	1
IPI00461861	211	GKKVIINFFVGTDDKNSTQHIIHFDQPRIGI	0.999998	57.9204	106.42	106.42 _VIINFFVGTDDKN(de)STQHIIHFDQPR_	VIINFFVGTDDKN(1)STQHIIHFDQPR	VIIN(-57.92)FFVGTDDKN(57.92)	13	3	981		1	1
IPI00468196	68	GMPHKCYHGKTGRVYNVTQHAMGIIVNKQVK	0.999999	62.0247	108.18	108.18 _TGRVYN(de)VTQHAM(ox)GIIVNKQVK_	TGRVYN(1)VTQHAMGIIVNKQVK	TGRVYN(62.02)VTQHAMGIIVN(-62	6	3	792		1	1
IPI00380801	224	PTIWIKHIVIKDSKINNTNVRNSEKVHSCDQ	0.842321	7.28652	167.12	167.12 _DSKIN(de)NTNVR_	DSKIN(0.842)N(0.157)TNVR	DSKIN(7.29)N(-7.29)TN(-33.89	5	2	581		1	1
IPI00225715	307	HTISGSKVQKNDFHRNITMFISRDIRKNINY	1	129.382	129.38	129.38 _N(de)ITM(ox)FISR_	N(1)ITMFISR	N(129.38)ITMFISR	1	2	500		1	1
IPI00131021	108	AYDICAKNRYHVIHINTTKNNPVMSIQDQVR	1	93.0956	125.74	93.096 _YHVIHIN(de)TTK_	YHVIHIN(1)TTK	YHVIHIN(93.1)TTK	7	3	410		1	1
IPI00356462	602	FKFQIKDTEENIHVINQTISEVIFPMDIKVD	0.929834	11.2228	64.26	64.26 _DTEENIHVIN(de)QTISEVIFPM(ox)DIK_	DTEEN(0.07)IHVIN(0.93)QTISEVI	DTEEN(-11.22)IHVIN(11.22)QTI	10	3	901		1	1
IPI00323134	651	PFAFDIPISPVTIKRNWTINRINGDFAQINI	0.999409	31.844	140.74	140.74 _RN(de)WTINRIN(de)GDFAQINIK_	RN(0.999)WTIN(0.096)RIN(0.904)	RN(31.84)WTIN(-9.76)RIN(9.76	2	3	726		1	1
IPI00130420	398	RCIQHFYGPNHEHCFNRTIIRNSSGCEARSD	0.999999	62.7371	171.73	171.73 _(ca)CIQHFYGPNHEHCFN(de)R_	CIQHFYGPNHEHCFN(1)R	CIQHFYGPN(-62.74)HEHCFN(62.7	15	3	701		1	1
IPI00137311	75	EVYVGAVNRIYKISGNITIIRAHVTGPVEDN	1	115.574	115.57	115.57 _ISGN(de)ITIIR_	ISGN(1)ITIIR	ISGN(115.57)ITIIR	4	2	494		1	1
IPI00338094	55	YQQDIGIGESRISHENGTIICSKGSTCYGIW	1	112.43	112.43	112.43 _ISHEN(de)GTIICSK_	ISHEN(1)GTIICSK	ISHEN(112.43)GTIICSK	5	2	680		1	1
IPI00221426	103	ASIITGKYPHNHHVVNNTIEGNCSSKAWQKI	0.89319	8.19668	131.56	131.56 _ASIITGKYPHNHHVVN(de)NTIEGN(de)CSSK_	ASIITGKYPHN(0.007)HHVVN(0.893)	ASIITGKYPHN(-21.71)HHVVN(8.2	16	3	960		1	1
IPI00342158	44	NIPKVIIPFTRATRVNFTIEASEGCYRWSST	1	162.722	162.72	162.72 _VN(de)FTIEASEGCYR_	VN(1)FTIEASEGCYR	VN(162.72)FTIEASEGCYR	2	2	774		1	1
IPI00118994	107	PRTQAWIQSSRDHIINKTHSICPRIICKDRT	1	105.002	105	105 _DHIIN(de)KTHSICPR_	DHIIN(1)KTHSICPR	DHIIN(105)KTHSICPR	5	2	796		1	1
IPI00127856	94	MEIREYHTIDDHCVYNSTHIGIQRENGTISK	1	80.6384	80.638	80.638 _EYHTIDDHCVYN(de)STHIGIQR_	EYHTIDDHCVYN(1)STHIGIQR	EYHTIDDHCVYN(80.64)STHIGIQR	12	3	820		1	1
IPI00124666	126	NQTECFNHVRFIQRINATHFYACGTHAFQPI	1	62.9579	62.958	62.958 _IN(de)ATHFYACGTHAFQPICAAIDAETFIIPTSFEEGKEK_	IN(1)ATHFYACGTHAFQPICAAIDAETF	IN(62.96)ATHFYACGTHAFQPICAAI	2	4	1072		1	1
IPI00467068	285	DSHKVTITFATDESGNHTGWKIHYTSTARPC	1	173.031	173.03	173.03 _VTITFATDESGN(de)HTGWK_	VTITFATDESGN(1)HTGWK	VTITFATDESGN(173.03)HTGWK	12	2	933		1	1
IPI00222305	500	KKDCSETIATFVKWQNDTGPSDKSDISQKQP	1	224.592	224.59	224.59 _WQN(de)DTGPSDKSDISQK_	WQN(1)DTGPSDKSDISQK	WQN(224.59)DTGPSDKSDISQK	3	2	904		1	1
IPI00395208	191	QIGDSVRKKHEPKVNNVTGNFTFVIRDIIPK	0.996168	24.1493	116.19	116.19 _VNN(de)VTGN(de)FTFVIR_	VN(0.004)N(0.996)VTGN(1)FTFVI	VN(-24.15)N(24.15)VTGN(65.97	3	2	742		1	1
IPI00849146	269	GPAAPADVRYSIRVINSTGHDVARCMADPGD	1	144.103	144.1	144.1 _VIN(de)STGHDVAR_	VIN(1)STGHDVAR	VIN(144.1)STGHDVAR	3	2	585		1	1
IPI00929820	202	REQGPSIVTPPKDIWNVTGAKVFISCEVIGI	1	113.856	113.86	113.86 _EQGPSIVTPPKDIWN(de)VTGAK_	EQGPSIVTPPKDIWN(1)VTGAK	EQGPSIVTPPKDIWN(113.86)VTGAK	15	2	1070		1	1
IPI00395208	195	SVRKKHEPKVNNVTGNFTFVIRDIIPKTNYC	1	65.9659	126.63	116.19 _VNN(de)VTGN(de)FTFVIR_	VN(0.004)N(0.996)VTGN(1)FTFVI	VN(-24.15)N(24.15)VTGN(65.97	7	2	742		1	1
IPI00177214	280	GEPIETKIKIMESHPNGTFSAKGVASVCVED	1	81.4315	223.22	81.431 _IM(ox)ESHPN(de)GTFSAK_	IMESHPN(1)GTFSAK	IMESHPN(81.43)GTFSAK	7	2	718		1	1
IPI00798466	1640	GWTTFFDYDSEGRITNVTFPTGVVTNIHGDM	0.999996	54.111	96.234	96.234 _ITN(de)VTFPTGVVTNIHGDM(ox)DK_	ITN(1)VTFPTGVVTNIHGDMDK	ITN(54.11)VTFPTGVVTN(-54.11)	3	2	1089		1	1
IPI00468674	937	DTSNITNVTVKARVWNSTFIEDYKDFDRVRV	1	162.461	162.46	162.46 _VWN(de)STFIEDYKDFDR_	VWN(1)STFIEDYKDFDR	VWN(162.46)STFIEDYKDFDR	3	2	968		1	1
IPI00321375	136	PKIVRWNPCIEPFRFNDTEVIQRISCMAIKG	1	108.47	108.47	108.47 _FN(de)DTEVIQR_	FN(1)DTEVIQR	FN(108.47)DTEVIQR	2	2	562		1	1
IPI00125138	107	IIDETMRFKDNTPNANATERIQEISNNINSC	0.818857	6.55363	98.592	98.592 _FKDNTPNAN(de)ATER_	FKDNTPN(0.181)AN(0.819)ATER	FKDN(-40.49)TPN(-6.55)AN(6.5	9	2	740		1	1
IPI00271262	294	VNSKIDNNGCSTQEVNITEIQSKKRNYEVQI	1	79.6897	79.69	79.69 _EVN(de)SKIDN(de)N(de)GCSTQEVN(de)ITEIQSK_	EVN(1)SKIDN(1)N(1)GCSTQEVN(1)	EVN(79.69)SKIDN(79.69)N(79.6	17	2	1356		1	1
IPI00109109	63	QIHHSKHHAAYVNNINATEEKYHEAIAKGDV	0.901018	12.6021	83.404	83.404 _HHAAYVNNIN(de)ATEEKYHEAIAK_	HHAAYVN(0.049)N(0.049)IN(0.90	HHAAYVN(-12.6)N(-12.6)IN(12.	10	3	842		1	1
IPI00134743	230	IDVIMGGGRKYMYPKNRTDVEYEIDEKARGT	1	124.417	124.42	124.42 _N(de)RTDVEYEIDEK_	N(1)RTDVEYEIDEK	N(124.42)RTDVEYEIDEK	1	2	756		1	1
IPI00113539	430	SNGAICHFPFIYNNRNYTDCTSEGRRDNMKW	1	110.838	110.84	110.84 _N(de)YTDCTSEGR_	N(1)YTDCTSEGR	N(110.84)YTDCTSEGR	1	2	602		1	1
IPI00119063	4280	GPKCTAQVCAGYCSNNSTCTVNQGNQPQCRC	0.780871	5.84877	72.316	72.316 _CTAQVCAGYCSNN(de)STCTVNQGNQPQCR_	CTAQVCAGYCSN(0.203)N(0.781)ST	CTAQVCAGYCSN(-5.85)N(5.85)ST	13	3	1046		1	1
IPI00119063	929	ICDGDNDCGNSEDESNATCSARTCPPNQFSC	0.999996	50.8972	111.53	111.53 _WICDGDN(de)DCGNSEDESN(de)ATCSAR_	WICDGDN(0.524)DCGN(0.476)SEDES	WICDGDN(0.42)DCGN(-0.42)SEDE	17	2	1318		1	1
IPI00138061	170	GIVHVHTGIQFGSRINYTCNQGYRIIGSSSA	1	115.574	205.53	115.57 _IN(de)YTCN(de)QGYR_	IN(1)YTCN(1)QGYR	IN(115.57)YTCN(115.57)QGYR	2	2	646		1	1
IPI01026927	309	FQKWASVVVPIGKEQNYTCHVYHEGIPEPIT	1	89.9791	224.4	89.979 _WASVVVPIGKEQN(de)YTCHVYHEGIPEPITIR_	WASVVVPIGKEQN(1)YTCHVYHEGIPEP	WASVVVPIGKEQN(89.98)YTCHVYHE	13	3	1165		1	1
IPI00969894	29	AEMEEIPVPHNIKINNITCDSFKISWDM___	1	46.785	46.785	46.785 _AQAGN(de)KSQDSGIAEM(ox)EEIPVPHN(de)IKIN(de)N(de)ITCDSFK_	AQAGN(1)KSQDSGIAEMEEIPVPHN(1)	AQAGN(46.79)KSQDSGIAEMEEIPVP	28	4	970		1	1
IPI00132454	277	FGEDYPGIVNPIDHTNVTAPQASMMFQYFVK	1	33.2886	143.19	33.289 _HISFGEDYPGIVN(de)PIDHTN(de)VTAPQASM(ox)M(ox)FQYFVK_	HISFGEDYPGIVN(1)PIDHTN(1)VTAP	HISFGEDYPGIVN(33.29)PIDHTN(3	19	4	973		1	1
IPI00464135	56	SEERIIRKFEAENISNYTAIIISQDGKTIYV	0.999464	32.7035	195.03	195.03 _KFEAENISN(de)YTAIIISQDGK_	KFEAEN(0.001)ISN(0.999)YTAIII	KFEAEN(-32.7)ISN(32.7)YTAIII	9	2	1122		1	1
IPI00112614	478	IMAFIAKNPEDVQSPNGSVYTWREAFNETNQ	1	86.5108	158.29	158.29 _NPEDVQSPN(de)GSVYTWR_	NPEDVQSPN(1)GSVYTWR	N(-86.51)PEDVQSPN(86.51)GSVY	9	2	925		1	1
IPI00551169	89	MTTEGGKWTVFQKRFNGSVSFFRGWSDYKIG	1	159.829	159.83	159.83 _FN(de)GSVSFFR_	FN(1)GSVSFFR	FN(159.83)GSVSFFR	2	2	531		1	1
IPI00461438	2	_______________MNMSVITIQEYEFEKQF	1	172.913	172.91	172.91 _M(ox)N(de)M(ox)SVITIQEYEFEK_	MN(1)MSVITIQEYEFEK	MN(172.91)MSVITIQEYEFEK	2	2	948		1	1
IPI00623114	3446	DVSDVNDNAPIFSRDNYSVIIQENKPVGFSV	0.999129	30.5981	85.362	85.362 _DN(de)YSVIIQENKPVGFSVIK_	DN(0.999)YSVIIQEN(0.001)KPVGF	DN(30.6)YSVIIQEN(-30.6)KPVGF	2	2	1076		1	1
IPI00410796	317	RDGHFKEDPYWENMINHSVHRRIIHRIGAVR	0.997579	26.1502	140.22	92.977 _DGHFKEDPYWENM(ox)IN(de)HSVHR_	DGHFKEDPYWEN(0.002)MIN(0.998)	DGHFKEDPYWEN(-26.15)MIN(26.1	15	3	843		1	1
IPI00322304	211	RGGERTNYYVEFSMRNCSTQHFPRSPIVFGF	1	98.044	98.044	98.044 _N(de)CSTQHFPR_	N(1)CSTQHFPR	N(98.04)CSTQHFPR	1	2	574		1	1
IPI00131091	224	GTWKISARFSDGIESNRSTHFEVKKYVIPNF	1	132.01	132.01	132.01 _FSDGIESN(de)R_	FSDGIESN(1)R	FSDGIESN(132.01)R	8	2	513		1	1
IPI00114671	109	RVGDEYQEIQIDGFDNESSNKTWMKNTGKTV	0.847027	7.43284	111.49	111.49 _VGDEYQEIQIDGFDN(de)ESSNKTWM(ox)K_	VGDEYQEIQIDGFDN(0.847)ESSN(0.	VGDEYQEIQIDGFDN(7.43)ESSN(-7	15	3	951		1	1
IPI00221426	109	KYPHNHHVVNNTIEGNCSSKAWQKIQEPYTF	0.991955	20.442	131.56	69.979 _YPHNHHVVN(de)NTIEGN(de)CSSK_	YPHN(0.055)HHVVN(0.811)N(0.14	YPHN(-11.73)HHVVN(7.78)N(-7.	15	3	737		1	1
IPI00270376	201	TPWIAEMTSPVISVKNASSEYSGTYSCTVQN	1	215.196	258.14	258.14 _N(de)ASSEYSGTYSCTVQNR_	N(1)ASSEYSGTYSCTVQNR	N(215.2)ASSEYSGTYSCTVQN(-215	1	2	963		1	1
IPI00136642	168	HFFFAKINCRIYRKANKSSDIVSANRIFGDK	1	96.4009	113.99	113.99 _AN(de)KSSDIVSANR_	AN(1)KSSDIVSANR	AN(96.4)KSSDIVSAN(-96.4)R	2	2	632		1	1
IPI00267963	88	IMVKMTKIRIIQYYGNFSRTANQQRIRIIEE	1	135.987	135.99	135.99 _IIQYYGN(de)FSR_	IIQYYGN(1)FSR	IIQYYGN(135.99)FSR	7	2	631		1	1
IPI00224237	546	NHINVYINIANIIRANESRIEEADQIYRQAI	1	111.831	162.94	111.83 _AN(de)ESRIEEADQIYR_	AN(1)ESRIEEADQIYR	AN(111.83)ESRIEEADQIYR	2	2	848		1	1
IPI00848693	681	KDQDPASFGNNSIIINSSRHYINIRYTIIPY	0.998006	26.4628	138.82	138.82 _NHN(de)GQGYKDQDPASFGNN(de)SIIIN(de)SSR_	N(0.129)HN(0.871)GQGYKDQDPASF	N(-8.34)HN(8.34)GQGYKDQDPASF	23	3	946		1	1
IPI00136210	6	__________MDTSMNFSRGIKMDIMQPYDF	1	128.035	218.6	128.03 _(ac)M(ox)DTSM(ox)N(de)FSR_	MDTSMN(1)FSR	MDTSMN(128.03)FSR	6	2	582		1	1
IPI00969894	6	__________MAQAGNKSQDSGIAEMEEIPV	1	46.785	46.785	46.785 _AQAGN(de)KSQDSGIAEM(ox)EEIPVPHN(de)IKIN(de)N(de)ITCDSFK_	AQAGN(1)KSQDSGIAEMEEIPVPHN(1)	AQAGN(46.79)KSQDSGIAEMEEIPVP	5	4	970		1	1
IPI00420867	135	TFIGSTAGMRIISQKNSSQARDIIAAVSQTI	1	117.091	117.09	117.09 _IISQKN(de)SSQAR_	IISQKN(1)SSQAR	IISQKN(117.09)SSQAR	6	2	617		1	1
IPI00469537	463	APTFTQTSYTMFVRENNSPAIHIGTISATDS	1	50.285	67.217	50.285 _(gl)EN(de)N(de)SPAIHIGTISATDSDSGSN(de)AHITYSIR_	EN(1)N(1)SPAIHIGTISATDSDSGSN(	EN(50.29)N(50.29)SPAIHIGTISA	2	3	1038		1	1

IPI00226229	182	PIRMRRIIRPFFIIQNSSMMKKTIKCIRWSI	1	91.3133	101.39	91.313	_PFFIIQ(de)N(de)SSM(ox)M(ox)K_	PFFIIQN(1)SSMMK	PFFIIQN(91.31)SSMMK	7	2	739			1	1
IPI00850057	124	EQWFRVSIRTAQRYYNQSKGGSHTFQRMFGC	1	98.9421	145	98.942	_YYN(de)QSKGGSHTFQR_	YYN(1)QSKGGSHTFQR	YYN(98.94)QSKGGSHTFQR	3	2	837			1	1
IPI00674255	877	DTEIEVKIQKENDNFNISKDDIDITIFHGEN	0.773939	8.35512	120.33	120.33	_IQKENDNFN(de)ISKDDIDITIFHGENK_	IQKEN(0.113)DN(0.113)FN(0.774)	IQKEN(-8.36)DN(-8.36)FN(8.36	9	3	983			1	1
IPI00118011	736	RIHHWSSPKPICSIVNSSIVVKAGEAVVIFQ	1	113.474	113.47	113.47	_IHHWSSPKPICSIVN(de)SSIVVK_	IHHWSSPKPICSIVN(1)SSIVVK	IHHWSSPKPICSIVN(113.47)SSIVV	15	3	797			1	1
IPI00624345	480	FYINPIRWIQQEFHSNASIPTHIVTFNVIEK	0.999999	61.074	141.31	141.31	_WIQQEFHSN(de)ASIPTHIVTFNVIEK_	WIQQEFHSN(1)ASIPTHIVTFNVIEK	WIQQEFHSN(61.07)ASIPTHIVTFN(	9	3	947			1	1
IPI00403938	1018	FFWTTPIAKFDRYRINYSIPTGQSMEVQIPK	1	199.148	199.15	199.15	_IN(de)YSIPTGQSM(ox)EVQIPK_	IN(1)YSIPTGQSMEVQIPK	IN(199.15)YSIPTGQSMEVQIPK	2	2	961			1	1
IPI00124697	75	PDEVIQKFSEIATVHNHSIPKEQIQEFVQSH	1	112.423	112.42	112.42	_FSEIATVHN(de)HSIPK_	FSEIATVHN(1)HSIPK	FSEIATVHN(112.42)HSIPK	9	2	791			1	1
IPI00453524	96	AWAPRYPAEGQRPIPNVSIPIRFDWRDKHVV	1	150.336	150.34	150.34	_YPAEGQRPIPN(de)VSIPIR_	YPAEGQRPIPN(1)VSIPIR	YPAEGQRPIPN(150.34)VSIPIR	11	3	637			1	1
IPI00129041	64	RCIVPHTVNISSAWRNHSIPIETKDGRQVPQ	1	101.381	101.38	101.38	_N(de)HSIPIETK_	N(1)HSIPIETK	N(101.38)HSIPIETK	1	2	520			1	1
IPI00131168	370	GTAFIVYGAPGFSKDNDSIITRKEFQEGINM	1	119.224	145.25	119.22	_DN(de)DSIITRK_	DN(1)DSIITRK	DN(119.22)DSIITRK	2	2	532			1	1
IPI00515360	3069	KIRDQEIEDNVHISPNGSIITIVGTRPSNHG	0.999272	31.374	89.723	89.723	_IRDQEIEDNVHISPN(de)GSIITIVGTRPSN(de)HGAYR_	IRDQEIEDN(0.001)VHISPN(0.999)(	IRDQEIEDN(-31.37)VHISPN(31.3	15	4	916			1	1
IPI01016174	1430	TIVKAKDQQAAEANKNASIIIKEIDIEKSRE	1	94.0899	103.76	94.09	_DQ(de)Q(de)AAEAN(de)KN(de)ASIIIK_	DQQAAEAN(1)KN(1)ASIIIK	DQQAAEAN(94.09)KN(94.09)ASII	10	3	573			1	1
IPI00122493	406	CNETSKIGDFIRYHYNCSIIDGTRIFSSHDY	1	83.2035	83.204	83.204	_YHYN(de)CSIIDGTR_	YHYN(1)CSIIDGTR	YHYN(83.2)CSIIDGTR	4	2	750			1	1
IPI00750217	99	VSCVDYDIIFANKMVNHSIHPTEPVKVTIPD	1	136.299	136.3	136.3	_M(ox)VN(de)HSIHPTEPVK_	MVN(1)HSIHPTEPVK	MVN(136.3)HSIHPTEPVK	3	2	753			1	1
IPI00133292	72	DEADEKTYNDVIFRYNGSIGIWRRCITIPKN	1	96.1429	96.143	96.143	_YN(de)GSIGIWR_	YN(1)GSIGIWR	YN(96.14)GSIGIWR	2	2	534			1	1
IPI00118011	35	GPSYSISGSWRVSNGNGSIEIPATVPGYVHS	1	49.8878	69.578	49.888	_VSN(de)GN(de)GSIEIPATVPGYVHSAIHQHGIIQDPYYR_	VSN(1)GN(1)GSIEIPATVPGYVHSAIH(	VSN(49.89)GN(49.89)GSIEIPATV	5	3	1231			1	1
IPI00343568	451	DAQSPFQNIHIIKVINISHSIIDISSEQIFD	0.996666	27.6359	54.732	54.732	_VIN(de)ISHSIIDISSEQIFDGIPAIQHINIQGNHFPK_	VIN(0.997)ISHSIIDISSEQIFDGIPA	VIN(27.64)ISHSIIDISSEQIFDGIP	3	4	975			1	1
IPI00129968	216	AQVPIDAHSNEKYIINGSHANETRIKIKHII	1	89.4303	89.43	89.43	_YIIN(de)GSHAN(de)ETR_	YIIN(1)GSHAN(1)ETR	YIIN(89.43)GSHAN(89.43)ETR	4	2	689			1	1
IPI00226714	180	RKDGAFHHIHSTPFGNYSFISVDATQRPGPK	1	67.8987	114.39	67.899	_KDGAFHHIHSTPFGN(de)YSFISVDATQRPGPK_	KDGAFHHIHSTPFGN(1)YSFISVDATQR	KDGAFHHIHSTPFGN(67.9)YSFISVD	15	3	1105			1	1
IPI00674255	896	DDIDITIFHGENKQFNCSFENITRNQDITTI	1	122.326	122.33	122.33	_QFN(de)CSFEN(de)ITR_	QFN(1)CSFEN(1)ITR	QFN(122.33)CSFEN(122.33)ITR	3	2	709			1	1
IPI00115976	596	NGAREDCREMKIYIRNESEFRDKISPIHIAI	1	110.662	110.66	110.66	_IYIRN(de)ESEFRDK_	IYIRN(1)ESEFRDK	IYIRN(110.66)ESEFRDK	5	3	524			1	1
IPI00113042	666	RNHAVSFDPIFSAVKNFSEAASDFHRRITQV	1	82.4042	124.08	124.08	_NHAVSFDPIFSAVKN(de)FSEAASDFHR_	NHAVSFDPIFSAVKN(1)FSEAASDFHR	N(-82.4)HAVSFDPIFSAVKN(82.4)	15	3	932			1	1
IPI00120953	1142	GFVCEKMQDTIEHHVNVSDTSAIPSTIEYGN	1	124.399	124.4	124.4	_M(ox)QDTIEHHVN(de)VSDTSAIPSTIEYGN(de)R_	MQDTIEHHVN(1)VSDTSAIPSTIEYGN(	MQDTIEHHVN(124.4)VSDTSAIPSTI	10	3	978			1	1
IPI00127134	60	VNQTAMADCRAVCGINTSDRCDFVRRNPDCR	1	110.923	110.92	110.92	_AVCGIN(de)TSDRCDFVR_	AVCGIN(1)TSDRCDFVR	AVCGIN(110.92)TSDRCDFVR	6	2	886			1	1
IPI00222809	162	FSVPPFAYADIARNINSSCRPHPGAWIRVVF	0.999742	35.8755	117.86	86.772	_NIN(de)SSCRPHPGAWIR_	NIN(1)SSCRPHPGAWIR	N(-35.88)IN(35.88)SSCRPHPGAW	3	3	589			1	1
IPI00377642	51	GCSEYTNRSCEECIRNVSCIWCNENKACMDY	0.999999	62.7446	136.38	136.38	_N(de)VSCIWCNENK_	N(1)VSCIWCNENK	N(62.74)VSCIWCN(-62.74)EN(-9	1	2	713			1	1
IPI00330843	415	PAPEEITVVIGQDRHNQSCEWCQTIAVRSYR	1	115.801	115.8	115.8	_HN(de)QSCEWCQTIAVR_	HN(1)QSCEWCQTIAVR	HN(115.8)QSCEWCQTIAVR	2	2	895			1	1
IPI00320065	60	DSVVWIGDIQTHRWSNDSATISFTKPWSQGK	1	161.908	161.91	161.91	_WSN(de)DSATISFTKPWSQGK_	WSN(1)DSATISFTKPWSQGK	WSN(161.91)DSATISFTKPWSQGK	3	2	1021			1	1
IPI00230319	532	FYDCSCVSGSGFQKGNHSARIGECPRDKCKT	1	80.3695	85.737	85.737	_IM(ox)NIEFYDCSCVSGSGFQKGN(de)HSAR_	IMNIEFYDCSCVSGSGFQKGN(1)HSAR	IMN(-80.37)IEFYDCSCVSGSGFQKG	21	3	961			1	1
IPI00761772	184	SIHAFQHIRGVQERVNISAPIIPKEDPIFTY	1	87.0877	87.088	87.088	_VN(de)ISAPIIPKEDPIFTYISK_	VN(1)ISAPIIPKEDPIFTYISK	VN(87.09)ISAPIIPKEDPIFTYISK	2	3	749			1	1
IPI00314429	10	______MNPDIDTGHNTSAPAHWGEIKDANF	0.99996	44.0046	89.261	89.261	_(ac)M(ox)NPDIDTGHN(de)TSAPAHWGEIK_	MNPDIDTGHN(1)TSAPAHWGEIK	MN(-44)PDIDTGHN(44)TSAPAHWGE	10	3	784			1	1
IPI00354300	1826	VTGGARMKEDSSRAVNVSAIPANIQKQIRSK	1	89.4845	89.484	89.484	_(gl)EDSSRAVN(de)VSAIPAN(de)IQ(de)K_	EDSSRAVN(1)VSAIPAN(1)IQK	EDSSRAVN(89.48)VSAIPAN(89.48	8	2	942			1	1
IPI00760106	57	APGEMKKEIRIAGGENNCSGRVEIKIHDKWG	1	112.022	112.02	112.02	_IAGGEN(de)N(de)CSGRVEIK_	IAGGEN(1)N(1)CSGRVEIK	IAGGEN(112.02)N(112.02)CSGRV	6	2	803			1	1
IPI00111960	926	ATAPTQVISNGIPVSNFTYSPDNKSIAIPVS	0.998871	29.4661	71.882	71.882	_EVTVIGVATAPTQVISN(de)GIPVSN(de)FTYSPDNK_	EVTVIGVATAPTQVISN(0.999)GIPVS	EVTVIGVATAPTQVISN(32.85)GIPV	23	3	1074	1	1		1
IPI00666034	3860	FGEITARAGMASPIYNVTWSAGWKTKADHVE	1	95.4284	145.69	95.428	_AGM(ox)ASPIYN(de)VTWSAGWK_	AGMASPIYN(1)VTWSAGWK	AGMASPIYN(95.43)VTWSAGWK	9	3	619	1	1		1
IPI00308691	45	TGVINAPQKVIEEFYNQTWNHRYGEPIPSTT	1	68.6757	124.45	68.676	_VIEEFYN(de)QTWN(de)HR_	VIEEFYN(1)QTWN(1)HR	VIEEFYN(68.68)QTWN(68.68)HR	7	3	580	1	1		1
IPI00221547	690	VQGKNEDQHIDVKIKNATVTQYQIKGIEPET	1	143.238	150.46	143.24	_IKN(de)ATVTQYQIK_	IKN(1)ATVTQYQIK	IKN(143.24)ATVTQYQIK	3	3	470	1	1		1
IPI00310049	107	VIMNNVEDIIEQQTFNDTVSPRASASYYEQY	0.999676	38.331	103.92	100.94	_IPFNVIM(ox)NNVEDIIEQQTFN(de)DTVSPR_	IPFNVIMNNVEDIIEQQTFN(1)DTVSPR	IPFN(-45.21)VIMN(-38.33)N(-3	20	4	763	1	1		1
IPI00119063	1559	NGGRGPCSHICIINYNRTVSCACPHIMKIHK	0.999373	32.0229	223.31	223.31	_GPCSHICIINYN(de)R_	GPCSHICIIN(0.001)YN(0.999)R	GPCSHICIIN(-32.02)YN(32.02)R	12	2	803	1	1		1
IPI00323857	135	AYDSSIVIFQGQPRVNITVIPSITSRKQIID	1	130.565	130.56	130.56	_VN(de)ITVIPSITSR_	VN(1)ITVIPSITSR	VN(130.56)ITVIPSITSR	2	2	651	1	1		1
IPI00124326	397	GDEITIMEVTERDAGNYTVIITNPISMEKQS	0.999854	38.355	151.95	100.41	_DAGN(de)YTVIITNPISM(ox)EK_	DAGN(1)YTVIITNPISMEK	DAGN(38.35)YTVIITN(-38.35)PI	4	3	628	1	1		1
IPI00674255	386	IIHSDITSVYGTVVMNRTVIFIGTGDGQIIK	1	74.579	116.43	74.579	_VQPIASSTIIHSDITSVYGTVVM(ox)N(de)R_	VQPIASSTIIHSDITSVYGTVVMN(1)R	VQPIASSTIIHSDITSVYGTVVMN(74.	24	3	902	1	1		1
IPI00138209	546	RAIDREAETVRNGIYNITVIAIDADGRSCTG	1	120.289	160.25	120.29	_N(de)GIYN(de)ITVIAIDADGR_	N(1)GIYN(1)ITVIAIDADGR	N(120.29)GIYN(120.29)ITVIAID	5	2	854	1	1		1
IPI00652358	292	AVGNVTKAIKSHGIWNNTVFIFSTDNGGQTR	1	70.5381	70.538	70.538	_AIKSHGIWN(de)N(de)TVFIFSTDN(de)GGQTR_	AIKSHGIWN(1)N(1)TVFIFSTDN(1)G(	AIKSHGIWN(70.54)N(70.54)TVFI	9	3	889	1	1		1
IPI00130117	613	TPVIRWKKGQRISQKNQTVFERNCQSEDCAA	1	110.306	110.31	110.31	_ISQKN(de)QTVFER_	ISQKN(1)QTVFER	ISQKN(110.31)QTVFER	5	2	676	1	1		1
IPI00123746	530	SIYKDPAGWISINPINGTVDTTAVIDRESPF	0.998908	29.6123	122.84	122.84	_YSIYKDPAGWISINPIN(de)GTVDTTAVIDR_	YSIYKDPAGWISIN(0.001)PIN(0.99	YSIYKDPAGWISIN(-29.61)PIN(29	17	3	1028	1	1		1
IPI00848693	1334	NNGSIVWGKVWPDYPNITVDPSIGWDHQVEQ	1	82.3596	144.27	82.36	_VWPDYPN(de)ITVDPSIGWDHQVEQYR_	VWPDYPN(1)ITVDPSIGWDHQVEQYR	VWPDYPN(82.36)ITVDPSIGWDHQVE	7	3	972	1	1		1
IPI00109727	94	VTISNQPYIKVITIANFTTKDEGDYFCEIQV	1	119.274	119.27	119.27	_VITIAN(de)FTTK_	VITIAN(1)FTTK	VITIAN(119.27)FTTK	6	2	555	1	1		1
IPI00458413	3790	IDTNYTATVRGIRGPNFTSPASITFTTGIKP	1	100.282	100.28	100.28	_GPN(de)FTSPASITFTTGIKPPQDIEAK_	GPN(1)FTSPASITFTTGIKPPQDIEAK	GPN(100.28)FTSPASITFTTGIKPPQ	3	3	873	1	1		1
IPI00674255	407	IGTGDGQIIKVVIGENITSNCPEVIYEIKEE	0.999809	37.1823	149.73	149.73	_VVIGEN(de)ITSNCPEVIYEIKEETPVFYK_	VVIGEN(1)ITSNCPEVIYEIKEETPVFY	VVIGEN(37.18)ITSN(-37.18)CPE	6	3	1058	1	1		1
IPI00127672	196	IHMAKQIGYKIIPAFNTTSGIPYPRINIKFG	1	143.939	143.94	143.94	_IIPAFN(de)TTSGIPYPR_	IIPAFN(1)TTSGIPYPR	IIPAFN(143.94)TTSGIPYPR	6	3	550	1	1		1
IPI00121421	3016	SVRHDYIIIPEGAITNTTRIRWWQPFVISNG	1	118.47	118.47	118.47	_HDYIIIPEGAITN(de)TTR_	HDYIIIPEGAITN(1)TTR	HDYIIIPEGAITN(118.47)TTR	13	2	908	1	1		1
IPI00990932	1766	VFAIDQSSGITERRFNETRDTITSIVSDINV	1	98.0363	131.4	131.4	_FN(de)ETRDTITSIVSDINVR_	FN(1)ETRDTITSIVSDINVR	FN(98.04)ETRDTITSIVSDIN(-98.	2	3	694	1	1		1
IPI00990932	1055	YGSRYQEIIEIESSINKTQWKSQVHSVAQSK	1	64.275	162.77	64.275	_YQEIIEIESSIN(de)KTQWK_	YQEIIEIESSIN(1)KTQWK	YQEIIEIESSIN(64.27)KTQWK	12	2	1056	1	1		1
IPI00990932	573	RAEQITIHAIGIGEANKTQIRQIAGKDERVN	1	122.138	122.14	122.14	_AEQITIHAIGIGEAN(de)KTQIR_	AEQITIHAIGIGEAN(1)KTQIR	AEQITIHAIGIGEAN(122.14)KTQIR	15	3	722	1	1		1
IPI00321190	80	ICKTVVTEAGNIIKDNATQEEIIHYIEKTCE	1	117.478	248.46	117.48	_TVVTEAGN(de)IIKDN(de)ATQEEIIHYIEK_	TVVTEAGN(1)IIKDN(1)ATQEEIIHYII	TVVTEAGN(117.48)IIKDN(117.48	13	3	944	1	1		1
IPI00624896	465	IGDNDSAKKVYEEVINVTPNDGFAKVHYGFI	0.999933	41.7174	80.545	77.746	_KVYEEVIN(de)VTPNDGFAK_	KVYEEVIN(1)VTPNDGFAK	KVYEEVIN(41.72)VTPN(-41.72)D	8	3	642	1	1		1
IPI00320420	102	TRDSEMKIKAFPEVCNETMMAIWEECKPCIK	1	78.5008	114.96	78.501	_AFPEVCN(de)ETM(ox)M(ox)AIWEECKPCIK_	AFPEVCN(1)ETMMAIWEECKPCIK	AFPEVCN(78.5)ETMMAIWEECKPCIK	7	4	695	1	1		1
IPI00222967	361	RAFQINTFNIKVQPFNVTKGQYSTAEECAAD	1	73.6646	94.922	73.665	_VQPFN(de)VTK_	VQPFN(1)VTK	VQPFN(73.66)VTK	5	2	467	1	1		1
IPI00321190	459	VCKKIVIYIEHNIEKNSTKEEIIAAIEKGCS	1	109.66	236.95	109.66	_IVIYIEHN(de)IEKN(de)STKEEIIAAIEK_	IVIYIEHN(1)IEKN(1)STKEEIIAAIE	IVIYIEHN(109.66)IEKN(109.66)	12	3	934	1	1		1
IPI00120674	734	PDCVDDSVSPIIIRINYTIVGEPIRSFGNIR	1	118.367	118.37	118.37	_IN(de)YTIVGEPIR_	IN(1)YTIVGEPIR	IN(118.37)YTIVGEPIR	2	2	638	1	1		1
IPI00623114	334	KAVGAVDWDSHPYGYNITIQAKDKGTPPQFS	1	117.666	117.67	117.67	_AVGAVDWDSHPYGYN(de)ITIQAK_	AVGAVDWDSHPYGYN(1)ITIQAK	AVGAVDWDSHPYGYN(117.67)ITIQA	15	3	769	1	1		1
IPI00403938	38	GVIKKIIRHKRESGINMTIPEENQPVVFNHI	0.999955	43.4581	162.42	162.42	_ESGIN(de)M(ox)TIPEENQPVVFNHIYNIK_	ESGIN(1)MTIPEENQPVVFNHIYNIK	ESGIN(43.46)MTIPEEN(-43.46)Q	5	3	935	1	1		1
IPI00126186	344	NIECVQKIGYICKKGNNTINPFIIPSASDVP	0.686606	6.17937	74.13	74.13	_KGN(de)NTINPFIIPSASDVPTGCPNQWWPYAGHCYR_	KGN(0.687)N(0.148)TIN(0.165)P	KGN(6.18)N(-6.67)TIN(-6.18)P	3	4	980	1	1		1
IPI00473190	63	SIGKIITHTIEGPSQNVTIIQIQPWQDPESW	1	126.319	126.32	126.32	_IITHTIEGPSQN(de)VTIIQIQPWQDPESWER_	IITHTIEGPSQN(1)VTIIQIQPWQDPES	IITHTIEGPSQN(126.32)VTIIQIQP	12	3	1140	1	1		1
IPI00129304	437	EEMKIVDSKHGQIIKNFTIIQGPPGPRGPKG	1	147.12	147.12	147.12	_HGQIIKN(de)FTIIQGPPGPR_	HGQIIKN(1)FTIIQGPPGPR	HGQIIKN(147.12)FTIIQGPPGPR	7	3	659	1	1		1
IPI00990932	826	DHSGSIGPREQESMMNITIHIVKKADIGRDR	1	52.0715	89.959	52.071	_EQESM(ox)M(ox)N(de)ITIHIVK_	EQESMMN(1)ITIHIVK	EQESMMN(52.07)ITIHIVK	7	3	569	1	1		1
IPI00309999	1696	IVQDAEAINEKAVKINETIGNQDKTAERNIE	1	74.8534	215.48	74.853	_AVKIN(de)ETIGN(de)QDKTAER_	AVKIN(1)ETIGN(1)QDKTAER	AVKIN(74.85)ETIGN(74.85)QDKT	5	3	630	1	1		1
IPI01023131	268	PFRYPKEFQKIYPIENITIAPDPHVPDSIPP	0.999756	36.1292	50.505	50.505	_IYPIEN(de)ITIAPDPHVPDSIPPVAYNPWM(ox)DIR_	IYPIEN(1)ITIAPDPHVPDSIPPVAYNP	IYPIEN(36.13)ITIAPDPHVPDSIPP	6	4	891	1	1		1
IPI00130624	385	PSMRSFIISIAAIHDNHTHSDIQVKIFVVPT	1	64.4542	128.69	64.454	_SFIISIAAIHDN(de)HTHSDIQ(de)VK_	SFIISIAAIHDN(1)HTHSDIQVK	SFIISIAAIHDN(64.45)HTHSDIQVK	12	3	783	1	1		1
IPI00321222	70	SVACRDGIRAQAECRNTTHIIQRQITRTQDS	1	64.1035	104.24	64.104	_AQAECRN(de)TTHIIQR_	AQAECRN(1)TTHIIQR	AQAECRN(64.1)TTHIIQR	7	2	850	1	1		1
IPI00338209	378	QQIDGRGDHHQISHYNITGVRGNRMGTSHVS	1	63.8937	112.96	63.894	_GDHHQISHYN(de)ITGVR_	GDHHQISHYN(1)ITGVR	GDHHQISHYN(63.89)ITGVR	10	3	579	1	1		1
IPI00128260	353	VHQGIKGMVIDENSNNITGAVISVTGINHDV	0.495811	1.48923	97.877	97.877	_GM(ox)VIDENSNN(de)ITGAVISVTGINHDVTSGEHGDYFR_	GMVIDEN(0.007)SN(0.352)N(0.49	GMVIDEN(-18.47)SN(-1.49)N(1.	10	3	1246	1	1		1
IPI00119299	421	TGRNPIGQAQSAVVINVTERVAPHDPTSIKV	1	128.707	166.76	128.71	_N(de)PIGQAQSAVVIN(de)VTER_	N(1)PIGQAQSAVVIN(1)VTER	N(128.71)PIGQAQSAVVIN(128.71	13	3	600	1	1		1
IPI00130271	74	SSVHTKVKGIAEVTENVTEGGVTKIGHSIFD	1	237.461	237.46	237.46	_GIAEVTEN(de)VTEGGVTK_	GIAEVTEN(1)VTEGGVTK	GIAEVTEN(237.46)VTEGGVTK	8	2	803	1	1		1
IPI00321190	334	TCQFVMNKFSEIIVNNATEEIIVKGISNACA	1	84.8736	197.15	84.874	_FSEIIVN(de)N(de)ATEEIIVK_	FSEIIVN(1)N(1)ATEEIIVK	FSEIIVN(84.87)N(84.87)ATEEII	8	2	911	1	1		1
IPI00623114	1993	PIKFTQDAYSAVVKENSTEARTIAVITAIGN	1	159.953	159.95	159.95	_FTQDAYSAVVKEN(de)STEAR_	FTQDAYSAVVKEN(1)STEAR	FTQDAYSAVVKEN(159.95)STEAR	13	2	1009	1	1		1
IPI00408495	270	FWFKTSDTGEEEAITNSTEANGKYVVVSTPE	1	125.775	312.86	125.78	_TSDTGEEEAITN(de)STEAN(de)GKYVVVSTPEK_	TSDTGEEEAITN(1)STEAN(1)GKYVVV	TSDTGEEEAITN(125.78)STEAN(12	12	3	987	1	1		1
IPI00308245	52	IMGVAIVGRNKNPQMNFTEANEACKMIGITI	0.999999	59.3584	198.23	198.23	_NKNPQM(ox)N(de)FTEANEACK_	NKNPQMN(1)FTEANEACK	N(-95.01)KN(-59.36)PQMN(59.3	7	3	638	1	1		1
IPI00119063	730	YWVDAFYDRIETIIINGTDRKIVYEGPEINH	1	67.385	160.52	67.385	_IETIIIN(de)GTDRK_	IETIIIN(1)GTDRK	IETIIIN(67.38)GTDRK	7	3	459	1	1		1
IPI00930882	205	GIVIGPIHKNQKDEINETDIKQINPTIGSQE	1	65.7628	109.15	109.15	_NQKDEIN(de)ETDIK_	NQKDEIN(1)ETDIK	N(-65.76)QKDEIN(65.76)ETDIK	7	2	724	1	1		1
IPI00620800	2347	TVEYTNEKHTIEIAPNSTARRQIAQIIEGRP	1	59.8981	87.719	59.898	_HTIEIAPN(de)STAR_	HTIEIAPN(1)STAR	HTIEIAPN(59.9)STAR	8	2	656	1	1		1
IPI00626793	324	QANVRNKKVIKDAVNNITAKGITDYKKGFSF	1	105.652	105.65	105.65	_VIKDAVN(de)N(de)ITAK_	VIKDAVN(1)N(1)ITAK	VIKDAVN(105.65)N(105.65)ITAK	8	2	644	1	1		1
IPI00122491	334	IPAVIPAARIPVAVINATAKAIAVTIQSH__	1	77.6404	77.64	77.64	_IPVAVIN(de)ATAK_	IPVAVIN(1)ATAK	IPVAVIN(77.64)ATAK	7	2	549	1	1		1
IPI00316469	638	WNNSHIAIVGKAMSSNETAAYKIMRSIDVDY	1	128.283	191.79	128.28	_AM(ox)SSN(de)ETAAYK_	AMSSN(1)ETAAYK	AMSSN(128.28)ETAAYK	5	2	595	1	1		1
IPI00115516	562	MDAQEETAAEIIIRINITAAQISQIEGIIQA	1	157.114	157.11	157.11	_IN(de)ITAAQISQIEGIIQAR_	IN(1)ITAAQISQIEGIIQAR	IN(157.11)ITAAQISQIEGIIQAR	2	3	647	1	1		1
IPI00128484	186	FATRTQKERSWSTVGNCTAAIRWIERYYCFQ	1	78.763	173.44	78.763	_SWSTVGN(de)CTAAIR_	SWSTVGN(1)CTAAIR	SWSTVGN(78.76)CTAAIR	7	2	712	1	1		1
IPI00400016	1378	DIINNIKDFDRRVNDNKTAAEEAIRRIPAIN	1	74.5809	183.96	166.62	_RVNDN(de)KTAAEEAIR_	RVNDN(1)KTAAEEAIR	RVN(-74.58)DN(74.58)KTAAEEAI	5	2	794	1	1		1
IPI00226360	204	IARVAQPGINYAIGTNTSYPNNIIRNIAREE	0.999999	61.5877	182.15	182.15	_VAQPGINYAIGTN(de)TSYPNNIIR_	VAQPGINYAIGTN(1)TSYPNNIIR	VAQPGIN(-78.25)YAIGTN(61.59)	13	2	1189	1	1		1
IPI00116105	232	NETSTVKVPMMVQSGNISYFRDSAIPCQMVQ	1	61.1103	111.79	61.11	_VPM(ox)M(ox)VQSGN(de)ISYFR_	VPMMVQSGN(1)ISYFR	VPMMVQSGN(61.11)ISYFR	9	3	555	1	1		1
IPI00224752	382	MVIAYDITSREWIPINHSVNSVVVRYGHSIA	1	75.3164	140.27	75.316	_EWIPIN(de)HSVN(de)SVVVR_	EWIPIN(1)HSVN(1)SVVVR	EWIPIN(75.32)HSVN(75.32)SVVV	6	2	876	1	1		1
IPI00225072	262	SKNRIIDIDSCIDRSNYSVIDITPVAAIIPK	1	214.807	214.81	214.81	_SN(de)YSVIDITPVAAIIPK_	SN(1)YSVIDITPVAAIIPK	SN(214.81)YSVIDITPVAAIIPK	2	2	902	1	1		1

IPI00135010	949	VYTMISSHDQFTKYINFSTSEKEKTSVVEHR	1	97.2141	97.214	97.214	_YIN(de)FSTSEKEK_	YIN(1)FSTSEKEK	YIN(97.21)FSTSEKEK	3	2	674	1	1	1
IPI00135975	505	SSSHSPEEQRKMVIWNDSTIRHSRTIQIDPN	1	57.3483	94.767	57.348	_M(ox)VIWN(de)DSTIR_	MVIWN(1)DSTIR	MVIWN(57.35)DSTIR	5	2	626	1	1	1
IPI00117986	243	NASSVPHRHGHFTGFNGSTIRDNIGAGYAED	1	59.1551	68.809	59.155	_HGHFTGFN(de)GSTIR_	HGHFTGFN(1)GSTIR	HGHFTGFN(59.16)GSTIR	8	3	478	1	1	1
IPI00406603	820	TIIRNIIGIDENFVINSSTEHRSSSYHENDM	0.999993	51.8091	223.11	62.055	_NIIGIDEN(de)FVIN(de)SSTEHR_	N(0.002)IIGIDEN(0.998)FVIN(1)S	N(-28.18)IIGIDEN(28.18)FVIN(	12	3	687	1	1	1
IPI00229992	543	QPEIGCIRVVAVSPANISREERREVFISVPG	1	75.7391	103.34	75.739	_VVAVSPAN(de)ISREER_	VVAVSPAN(1)ISREER	VVAVSPAN(75.74)ISREER	8	3	510	1	1	1
IPI00338209	347	QRWRIWDPSSHPGIQNISRDIIREDIAQRIR	1	49.1875	127.36	49.188	_IWDPSSHPGIQN(de)ISR_	IWDPSSHPGIQN(1)ISR	IWDPSSHPGIQN(49.19)ISR	12	2	854	1	1	1
IPI00130015	275	FASMGMIEARIRIITNNSQTPIISPQEVVSC	1	70.7009	136.86	70.701	_IITN(de)N(de)SQTPIISPQEVVSCSPYAQGCDGGFPYIIAGK_	IITN(1)N(1)SQTPIISPQEVVSCSPYA(	IITN(70.7)N(70.7)SQTPIISPQEV	4	4	997	1	1	1
IPI00130573	1067	HTNAHGKDIDTDFTSNASQPETKAIIENIIQ	1	204.13	204.13	204.13	_DIDTDFTSN(de)ASQPETK_	DIDTDFTSN(1)ASQPETK	DIDTDFTSN(204.13)ASQPETK	9	2	885	1	1	1
IPI00165807	335	IVIIQKIEPEHIQIQNISQEQIAQVATIATK	1	209.239	209.24	209.24	_IEPEHIQIQN(de)ISQEQIAQVATIATK_	IEPEHIQIQN(1)ISQEQIAQVATIATK	IEPEHIQIQN(209.24)ISQEQIAQVA	10	3	935	1	1	1
IPI00113158	62	HDQCSPWKKNACCSVNTSQEIHKADSRIYFN	1	85.7073	103.21	103.21	_NACCSVN(de)TSQEIHK_	NACCSVN(1)TSQEIHK	N(-85.71)ACCSVN(85.71)TSQEIH	7	2	825	1	1	1
IPI00400016	1239	AIEIEEINRKYEQAKNISQDIEKQAARVHEE	1	131.958	193.96	131.96	_YEQAKN(de)ISQDIEK_	YEQAKN(1)ISQDIEK	YEQAKN(131.96)ISQDIEK	6	2	784	1	1	1
IPI00221890	27	GPDHWHEIYPIAKGDNQSPIEIHTKDIKHDP	1	115.119	115.12	115.12	_GDN(de)QSPIEIHTK_	GDN(1)QSPIEIHTK	GDN(115.12)QSPIEIHTK	3	2	670	1	1	1
IPI00126050	351	QGGIGASQYYEIHKANISKYSIVMEADSGTF	1	75.0639	75.064	75.064	_IVIWTAEEQGGIGASQYYEIHKAN(de)ISK_	IVIWTAEEQGGIGASQYYEIHKAN(1)ISI	IVIWTAEEQGGIGASQYYEIHKAN(75.	24	4	752	1	1	1
IPI00119809	543	SFEGSKAPIPTAIDTNSSKTPSIFPCASGAF	1	52.9273	93.21	52.927	_APIPTAIDTN(de)SSKTPSIFPCASGAFSSFR_	APIPTAIDTN(1)SSKTPSIFPCASGAFS	APIPTAIDTN(52.93)SSKTPSIFPCA	10	4	758	1	1	1
IPI00124666	596	PCDQPSNIARAIWIINGSKSISDGQDGYRVG	1	94.6917	120.12	94.692	_AIWIIN(de)GSK_	AIWIIN(1)GSK	AIWIIN(94.69)GSK	6	2	502	1	1	1
IPI00985989	534	KNAVAYIGIHSPIRGNSSIYPVVSPSIQQIV	1	149.943	245.66	149.94	_GN(de)SSIYPVVSPSIQQIVIEK_	GN(1)SSIYPVVSPSIQQIVIEK	GN(149.94)SSIYPVVSPSIQQIVIEK	2	2	1080	1	1	1
IPI00323857	307	IPDTPQGIIAEARKINASIVTSFVEIPIVSN	1	87.352	127.17	87.352	_KIN(de)ASIVTSFVEIPIVSN(de)VSIR_	KIN(1)ASIVTSFVEIPIVSN(1)VSIR	KIN(87.35)ASIVTSFVEIPIVSN(87	3	3	797	1	1	1
IPI00112614	98	RYPTPGEAPGVVGNFNKSIVSRIFSDAQRII	0.984724	18.0931	141.85	141.85	_YPTPGEAPGVVGNFN(de)K_	YPTPGEAPGVVGN(0.015)FN(0.985)I	YPTPGEAPGVVGN(-18.09)FN(18.0	15	2	824	1	1	1
IPI00323857	322	NASIVTSFVEIPIVSNVSIRASSCGGVFQTT	1	87.352	127.17	87.352	_KIN(de)ASIVTSFVEIPIVSN(de)VSIR_	KIN(1)ASIVTSFVEIPIVSN(1)VSIR	KIN(87.35)ASIVTSFVEIPIVSN(87	18	3	797	1	1	1
IPI00649186	417	PIIRKTCNPETFPIRNESIQCPTARCQFAFY	0.999699	35.2151	94.882	89.468	_TCNPETFPIRN(de)ESIQCPTAR_	TCNPETFPIRN(1)ESIQCPTAR	TCN(-35.22)PETFPIRN(35.22)ES	11	3	798	1	1	1
IPI00119324	114	DDDYSFCRAIKGETVNTSIPFSFEGIIFPKG	1	89.3739	89.374	89.374	_AIKGETVN(de)TSIPFSFEGIIFPK_	AIKGETVN(1)TSIPFSFEGIIFPK	AIKGETVN(89.37)TSIPFSFEGIIFP	8	3	799	1	1	1
IPI00122257	313	DKGNVITSYGNPIIINSSIPEDATIKADINQ	0.975138	15.9462	151.19	151.19	_VEFDDKGNVITSYGNPIIIN(de)SSIPEDATIK_	VEFDDKGNVITSYGN(0.025)PIIIN(0.	VEFDDKGN(-41.97)VITSYGN(-15.	20	3	1084	1	1	1
IPI00378698	137	APPYIWPAHKNEIMINSSIMRITIRNIEEVE	1	74.2998	140.14	140.14	_NEIM(ox)IN(de)SSIM(ox)R_	NEIMIN(1)SSIMR	N(-74.3)EIMIN(74.3)SSIMR	6	2	671	1	1	1
IPI00310519	118	DRDRIIISPSAFHDGNFSIIIRAVDRGDEGV	1	50.8268	116.43	50.827	_IIISPSAFHDGN(de)FSIIIR_	IIISPSAFHDGN(1)FSIIIR	IIISPSAFHDGN(50.83)FSIIIR	12	3	668	1	1	1
IPI00113057	396	PDCTTKINARIVGGTNASIGEWPWQVSIQVK	1	53.4532	167.51	53.453	_IVGGTN(de)ASIGEWPWQ(de)VSIQ(de)VK_	IVGGTN(1)ASIGEWPWQVSIQVK	IVGGTN(53.45)ASIGEWPWQVSIQVK	6	3	758	1	1	1
IPI00138061	295	CTPPPYVENAVMISENRSIFSIRDIVEFRCH	1	105.137	212.39	105.14	_CTPPPYVEN(de)AVM(ox)ISEN(de)R_	CTPPPYVEN(1)AVMISEN(1)R	CTPPPYVEN(105.14)AVMISEN(105	16	3	666	1	1	1
IPI00987265	1171	AQIEAAKAYTIFVPTNHSIETQGNNSVIGID	1	68.4451	144.63	68.445	_AYTIFVPTN(de)HSIETQ(de)GN(de)N(de)SVIGIDTVR_	AYTIFVPTN(1)HSIETQGN(1)N(1)SV]	AYTIFVPTN(68.45)HSIETQGN(68.	9	3	984	1	1	1
IPI00471081	412	GYWASYNIPFHKTIYNWSGYPIIVHKIGIDY	1	138.012	143.46	138.01	_TIYN(de)WSGYPIIVHK_	TIYN(1)WSGYPIIVHK	TIYN(138.01)WSGYPIIVHK	4	2	846	1	1	1
IPI00229820	70	IHSAAASRDIGTASHNFSGAIPRVEHPSRGH	1	105.029	138.19	105.03	_DIGTASHN(de)FSGAIPR_	DIGTASHN(1)FSGAIPR	DIGTASHN(105.03)FSGAIPR	8	3	515	1	1	1
IPI00123223	1142	ISCIESSWKTIEQERNASFVYTKAIMAYAFA	1	113.987	210.41	113.99	_TIEQERN(de)ASFVYTK_	TIEQERN(1)ASFVYTK	TIEQERN(113.99)ASFVYTK	7	3	563	1	1	1
IPI00321190	214	DCMKIVSDVQTAVKTNSSFIQGFVDHVKEDC	1	55.9857	223.29	55.986	_TN(de)SSFIQGFVDHVKEDCDRIGPGVSDICK_	TN(1)SSFIQGFVDHVKEDCDRIGPGVSD]	TN(55.99)SSFIQGFVDHVKEDCDRIG	2	4	821	1	1	1
IPI00459134	152	CDCPIEDSAAWIREMNCSETISQIQKDIAHF	1	131.172	143.93	131.17	_EM(ox)N(de)CSETISQIQK_	EMN(1)CSETISQIQK	EMN(131.17)CSETISQIQK	3	3	529	1	1	1
IPI00666034	2126	RAAISRIPQQIHHYINASDWERQVAGAKEKI	1	75.3164	102.64	75.316	_IPQQIHHYIN(de)ASDWER_	IPQQIHHYIN(1)ASDWER	IPQQIHHYIN(75.32)ASDWER	10	3	670	1	1	1
IPI00130010	1048	TGEQVTFRCQSPYQMNGSDTVTCVNSRWIGQ	1	186.836	223.29	223.29	_CQSPYQM(ox)N(de)GSDTVTCVNSR_	CQSPYQMN(1)GSDTVTCVNSR	CQSPYQMN(186.84)GSDTVTCVN(-1	8	3	741	1	1	1
IPI00128076	269	DEEISCTVVEIKYKGNASAIFIIPDQGRMQQ	1	131.184	131.18	131.18	_YKGN(de)ASAIFIIPDQGR_	YKGN(1)ASAIFIIPDQGR	YKGN(131.18)ASAIFIIPDQGR	4	2	876	1	1	1
IPI00307966	101	CQEIISTFKGAFVSKNPCNITREDYAPIVKI	0.880942	8.69187	136.38	116.54	_GAFVSKN(de)PCNITR_	GAFVSKN(0.881)PCN(0.119)ITR	GAFVSKN(8.69)PCN(-8.69)ITR	7	3	489	1	1	1
IPI00652728	262	TFFPSKIFTPVSSVPNITWSEISAIEIIKSI	1	85.2483	85.248	85.248	_IFTPVSSVPN(de)ITWSEISAIEIIK_	IFTPVSSVPN(1)ITWSEISAIEIIK	IFTPVSSVPN(85.25)ITWSEISAIEI	10	3	849		1	1
IPI00848693	723	GDTVARPIIHEFYDDNNTWGIDRQFIWGPGI	0.809064	6.27095	134.32	134.32	_GDTVARPIIHEFYDDN(de)NTWGIDR_	GDTVARPIIHEFYDDN(0.809)N(0.19]	GDTVARPIIHEFYDDN(6.27)N(-6.2	16	3	902		1	1
IPI00311405	202	TRIKGEAEYQEIRNPNGTVTVISRYRIVPSR	0.998201	27.4423	115.63	108.45	_IKGEAEYQEIRNPN(de)GTVTVISR_	IKGEAEYQEIRN(0.002)PN(0.998)G]	IKGEAEYQEIRN(-27.44)PN(27.44	14	3	826		1	1
IPI00119063	1826	GTCNKADGSGSVVIRNSTTIVMHMKVYDESI	1	87.1843	87.184	87.184	_N(de)STTIVM(ox)HM(ox)K_	N(1)STTIVMHMK	N(87.18)STTIVMHMK	1	2	598		1	1
IPI00923031	58	NVTWRVTNIMTGQTANATSIIGTMTDAFPKI	0.993221	21.6589	92.8	52.09	_VTNIM(ox)TGQTAN(de)ATSIIGTM(ox)TDAFPK_	VTN(0.007)IMTGQTAN(0.993)ATSI]	VTN(-21.66)IMTGQTAN(21.66)AT	11	4	655		1	1
IPI00405742	798	WCRGQNRCVYEAICSNVTSECPPPVITRIQP	1	100.187	100.19	100.19	_CVYEAICSN(de)VTSECPPPVITR_	CVYEAICSN(1)VTSECPPPVITR	CVYEAICSN(100.19)VTSECPPPVIT	9	2	1227		1	1
IPI00515360	89	VGSGDFQMVYFRAIVNFTRSIEYSPQIEDAS	1	100.188	100.19	100.19	_AIVN(de)FTR_	AIVN(1)FTR	AIVN(100.19)FTR	4	2	411		1	1
IPI00674656	107	SIPFIHEHFKGRIIINWTQGQTSGVIRIINI	1	120.627	120.63	120.63	_IIIN(de)WTQGQTSGVIR_	IIIN(1)WTQGQTSGVIR	IIIN(120.63)WTQGQTSGVIR	4	2	844		1	1
IPI00134585	316	VYVQPNQKETAEYAANITQAVFDYFEDYFAM	1	63.3721	63.372	63.372	_ETAEYAAN(de)ITQAVFDYFEDYFAM(ox)EYAIPK_	ETAEYAAN(1)ITQAVFDYFEDYFAMEYA]	ETAEYAAN(63.37)ITQAVFDYFEDYF	8	3	1143		1	1
IPI00126834	225	ERETVKEIQVYISPRNTTISVHPSTRIQEGG	1	118.757	118.76	118.76	_N(de)TTISVHPSTR_	N(1)TTISVHPSTR	N(118.76)TTISVHPSTR	1	2	607		1	1
IPI00225715	501	YVVYGSQQGRISSSPNVTISCKDTYCNIGWT	1	85.5541	85.554	85.554	_ISSSPN(de)VTISCK_	ISSSPN(1)VTISCK	ISSSPN(85.55)VTISCK	6	2	647		1	1
IPI00118987	228	RFNPQKAVAFVKNVVNITINDEAAMVEAVAI	0.762554	6.2661	91.603	91.603	_NVVN(de)ITINDEAAM(ox)VEAVAIYNPVSFAFEVTEDFIM(ox)YK_	N(0.18)VVN(0.763)ITIN(0.057)DI	N(-6.27)VVN(6.27)ITIN(-11.24	4	4	1051		1	1
IPI00674255	717	TSTCERDVIRVSHVINDTHMKFSIPSSRKEM	1	60.4896	91.961	60.49	_VSHVIN(de)DTHM(ox)K_	VSHVIN(1)DTHMK	VSHVIN(60.49)DTHMK	6	2	649		1	1
IPI00123040	139	IIVRVNKGPIANPIWNVTGFTGRDWIRAEIA	0.999995	53.3626	114.63	114.63	_GPIANPIWN(de)VTGFTGR_	GPIANPIWN(1)VTGFTGR	GPIAN(-53.36)PIWN(53.36)VTGF	9	2	851		1	1
IPI00120222	415	ITTSTVQVQGIEPYANYTFTVKSQNRVSGID	1	108.496	108.5	108.5	_GVHFSPAASGITTSTVQVQGIEPYAN(de)YTFTVK_	GVHFSPAASGITTSTVQVQGIEPYAN(1)Y	GVHFSPAASGITTSTVQVQGIEPYAN(1	26	3	1125		1	1
IPI00124640	539	KVGNVECGEGHFCHDNQTCCKDSAGVWACCP	1	103.272	130.11	130.11	_VGNVECGEGHFCHDN(de)QTCCK_	VGNVECGEGHFCHDN(1)QTCCK	VGN(-103.27)VECGEGHFCHDN(103	15	3	804		1	1
IPI00132600	899	IHSGPPVYFVIEEGYNYSSRKGQNMVCGGMG	1	81.4796	81.48	81.48	_SIAQYIHSGPPVYFVIEEGYN(de)YSSR_	SIAQYIHSGPPVYFVIEEGYN(1)YSSR	SIAQYIHSGPPVYFVIEEGYN(81.48)	21	3	960		1	1
IPI00223714	77	AIKKAIAAAGYDVEKNNSRIKIGIKSIVSKG	0.967104	14.6833	117.2	117.2	_AIAAAGYDVEKN(de)NSR_	AIAAAGYDVEKN(0.967)N(0.033)SR	AIAAAGYDVEKN(14.68)N(-14.68)	12	2	790		1	1
IPI01026704	1083	ATITCHIIPSDVSQVNVSIIIWKPTFIKAHF	1	55.0512	55.051	55.051	_IATITCHIIPSDVSQVN(de)VSIIIWKPTFIK_	IATITCHIIPSDVSQVN(1)VSIIIWKPTI	IATITCHIIPSDVSQVN(55.05)VSII	17	4	824		1	1
IPI00400016	1105	VKDVDQNIMDRIQRVNSSIHSQISRIQNIRN	1	90.9131	96.604	90.913	_VN(de)SSIHSQISR_	VN(1)SSIHSQISR	VN(90.91)SSIHSQISR	2	2	615		1	1
IPI00125813	83	YKQENNIIIINAEHGNSSIFIENSTFESFGY	0.987838	18.2165	95.108	73.919	_QENNIIIINAEHGN(de)SSIFIEN(de)STFESFGYHSVSPDR_	QENNIIIIN(0.223)AEHGN(0.988)SS	QEN(-34.77)N(-34.77)IIIIN(-5	14	4	1018		1	1
IPI00170146	130	EVIIHNIPDAIGVIFNDSFSYQIKVIRMYGN	1	73.753	119.8	73.753	_IIGVPSIEDIDEVIIHN(de)IPDAIGVIFN(de)DSFSYQ(de)IK_	IIGVPSIEDIDEVIIHN(1)IPDAIGVIFN	IIGVPSIEDIDEVIIHN(73.75)IPDA	27	3	1296		1	1
IPI00876233	64	FYCRFTECCHEERPINTSAIKIDIEEKIFGQ	1	66.0679	78.505	66.068	_FTECCHEERPIN(de)TSAIK_	FTECCHEERPIN(1)TSAIK	FTECCHEERPIN(66.07)TSAIK	12	2	1047		1	1
IPI00119063	2476	IPQQPMGIIAVANDTNSCEISPCRINNGGCQ	0.896978	9.39851	75.3	75.3	_VDIPQQPM(ox)GIIAVANDTN(de)SCEISPCR_	VDIPQQPMGIIAVAN(0.103)DTN(0.8	VDIPQQPMGIIAVAN(-9.4)DTN(9.4	18	4	726		1	1
IPI00420867	376	FHFINITSRQSINTVNDTVWKFCQKPWKIVE	0.999963	44.3208	135.02	135.02	_QSINTVN(de)DTVWK_	QSINTVN(1)DTVWK	QSIN(-44.32)TVN(44.32)DTVWK	7	2	703	1		1
IPI00108098	197	EDNQIIVKKGFCEADNSTVSENNPEDWPVNT	0.987097	19.0605	123.44	123.44	_KGFCEADN(de)STVSENNPEDWPVNTEGCM(ox)EK_	KGFCEADN(0.987)STVSEN(0.012)N	KGFCEADN(19.06)STVSEN(-19.06	8	3	1121	1		1
IPI00132460	91	KKYVIYIERVQREKANGTTVHVGIHPSKVVI	1	80.3118	80.312	80.312	_AN(de)GTTVHVGIHPSK_	AN(1)GTTVHVGIHPSK	AN(80.31)GTTVHVGIHPSK	2	3	474	1		1
IPI00876306	80	IDICVSENETIKHISNDTTTPESTMTVGQAR	1	122.44	122.44	122.44	_HISN(de)DTTTPESTM(ox)TVGQAR_	HISN(1)DTTTPESTMTVGQAR	HISN(122.44)DTTTPESTMTVGQAR	4	2	1032	1		1
IPI00400016	1437	NKAHEAERIASAVQKNATSTKADAERTFGEV	1	102.277	102.28	102.28	_IASAVQKN(de)ATSTK_	IASAVQKN(1)ATSTK	IASAVQKN(102.28)ATSTK	8	2	660	1		1
IPI00396840	186	SEAERKAQVYYRACMNETRIEEIRAKPIMEI	1	82.3618	118.87	82.362	_ACM(ox)N(de)ETRIEEIR_	ACMN(1)ETRIEEIR	ACMN(82.36)ETRIEEIR	4	2	770	1		1
IPI00131168	484	YEIEFVFGIPIGRRVNYTRAEEIFSRSIMKT	1	79.4662	79.466	79.466	_VN(de)YTRAEEIFSR_	VN(1)YTRAEEIFSR	VN(79.47)YTRAEEIFSR	2	2	743	1		1
IPI00968978	454	GDQYSCMVGHEAIPMNFTQKTIDRISGKPTN	1	111.769	149.52	111.77	_QGDQYSCM(ox)VGHEAIPM(ox)N(de)FTQK_	QGDQYSCMVGHEAIPMN(1)FTQK	QGDQYSCMVGHEAIPMN(111.77)FTQ	17	3	825	1		1
IPI00153756	118	GIIGAAFKPEYNRIINETIYENAKIISDNTD	1	88.0283	177.83	177.83	_IIN(de)ETIYENAK_	IIN(1)ETIYENAK	IIN(88.03)ETIYEN(-88.03)AK	3	2	655	1		1
IPI00467600	1283	GIEKVIEIKKNRCDNNDTIIVRGKCGKCSQQ	0.951793	12.9543	147.26	147.26	_CDNN(de)DTIIVR_	CDN(0.048)N(0.952)DTIIVR	CDN(-12.95)N(12.95)DTIIVR	4	2	611	1		1
IPI00987951	499	FDEFFSQGCAPGYEKNSTICDICIGPIKCAP	1	171.255	175.28	171.26	_N(de)STICDICIGPIK_	N(1)STICDICIGPIK	N(171.26)STICDICIGPIK	1	2	746	1		1
IPI00130325	181	GKRIPKMTQIIGITPNATHIHRPPRDCQEIF	1	62.8132	70.96	62.813	_M(ox)TQIIGITPN(de)ATHIHRPPR_	MTQIIGITPN(1)ATHIHRPPR	MTQIIGITPN(62.81)ATHIHRPPR	10	4	543	1		1
IPI00153187	139	MDAYEVSNADFEKFVNSTGYITEAEKFGDSF	1	73.4663	73.466	73.466	_FVN(de)STGYITEAEK_	FVN(1)STGYITEAEK	FVN(73.47)STGYITEAEK	3	3	487	1		1
IPI00320605	51	DCIQSGPGCSWCQKINFTGPGEPDSIRCDTR	1	102.727	102.73	102.73	_IN(de)FTGPGEPDSIR_	IN(1)FTGPGEPDSIR	IN(102.73)FTGPGEPDSIR	2	2	702	1		1
IPI00272690	116	VWGKKAKEIYESIWQNFTDSKIRRIIGSIRT	1	118.201	118.2	118.2	_EIYESIWQN(de)FTDSK_	EIYESIWQN(1)FTDSK	EIYESIWQN(118.2)FTDSK	9	2	881	1		1
IPI00119299	782	KTRSIEPHHSDIKIKNITDISQKTIRIADIQ	1	103.083	152.07	103.08	_IKN(de)ITDISQK_	IKN(1)ITDISQK	IKN(103.08)ITDISQK	3	2	581	1		1
IPI00129304	323	EQSIKDIQDIHKDTENRTAVKFSQIEERFQV	1	98.4072	98.407	98.407	_DIQDIHKDTEN(de)R_	DIQDIHKDTEN(1)R	DIQDIHKDTEN(98.41)R	11	2	743	1		1
IPI00130420	201	SEKDRFINFFVGNTINSSYPPGYSIHSISVR	0.999019	30.0803	128.38	128.38	_FINFFVGNTIN(de)SSYPPGYSIHSISVR_	FINFFVGN(0.001)TIN(0.999)SSYPI	FIN(-69.96)FFVGN(-30.08)TIN(	11	3	973	1		1
IPI00121334	86	KRWIIVGAPTASWISNASVVNPGAIYRCGIR	0.999941	42.2761	89.668	89.668	_WIIVGAPTASWISN(de)ASVVNPGAIYR_	WIIVGAPTASWISN(1)ASVVNPGAIYR	WIIVGAPTASWISN(42.28)ASVVN(-	14	3	882	1		1
IPI00968978	469	NFTQKTIDRISGKPTNVSVSVIMSEGDGICY	1	162.422	162.42	162.42	_ISGKPTN(de)VSVSVIM(ox)SEGDGICY_	ISGKPTN(1)VSVSVIMSEGDGICY	ISGKPTN(162.42)VSVSVIMSEGDGI	7	2	1166	1		1
IPI00128358	910	FAIERGCRIRGISPGNYSVRVRATSIAGNGS	1	73.8866	97.813	73.887	_GISPGN(de)YSVR_	GISPGN(1)YSVR	GISPGN(73.89)YSVR	6	2	526	1		1
IPI00128206	115	RRTTSTIQVKNEEVKNMSVEINSKIESIIEE	0.999997	55.3209	147.25	128.71	_NEEVKN(de)M(ox)SVEINSK_	NEEVKN(1)MSVEINSK	N(-73.05)EEVKN(55.32)MSVEIN(	6	2	819	1		1
IPI00122175	61	ICPPYKENPGHIYNKNISQKDCDCIHVVEPM	0.999868	38.7975	164.88	164.88	_ENPGHIYNKN(de)ISQK_	ENPGHIYNKN(1)ISQK	EN(-107.78)PGHIYN(-38.8)KN(3	10	2	822	1		1
IPI00314132	173	DINDIIVVYTIQEVTPNASKFFSIEGVNYPAI	1	64.2814	119.62	119.62	_ATDADINDIIVYTIQEVTPN(de)ASK_	ATDADINDIIVYTIQEVTPN(1)ASK	ATDADIN(-64.28)DIIVYTIQEVTPN	20	3	831	1		1
IPI00453571	127	YYTPTGEPVFGGIPQNASIVTHIAHTFQDIK	1	82.7264	82.726	82.726	_EEIGTYPYYTPTGEPVFGGIPQN(de)ASIVTHIAHTFQDIK_	EEIGTYPYYTPTGEPVFGGIPQN(1)ASI	EEIGTYPYYTPTGEPVFGGIPQN(82.7	23	4	1049	1		1
IPI00128024	60	AIPVIPAHTRQIIIANNSIRSVPPGAFDHIP	0.98721	18.8753	110.53	110.53	_QIIIAN(de)NSIR_	QIIIAN(0.987)N(0.013)SIR	QIIIAN(18.88)N(-18.88)SIR	6	2	572	1		1
IPI00317487	83	QAQSDAAGIPGAESRNDSIPGAGSEADGIEG	1	133.611	133.61	133.61	_GQAQSDAAGIPGAESRN(de)DSIPGAGSEADGIEGK_	GQAQSDAAGIPGAESRN(1)DSIPGAGSE	GQAQSDAAGIPGAESRN(133.61)DSI	17	3	1038	1		1
IPI00221706	139	QQVAIKMDNISRNIGNTSEGPRCGGSIITAD	1	70.7677	152.54	152.54	_NIGN(de)TSEGPR_	NIGN(1)TSEGPR	N(-70.77)IGN(70.77)TSEGPR	4	2	523	1		1
IPI00124515	240	FRTAVSSRIRDNAIGNVSCTIHNEAIGQEKT	0.815674	6.45931	162.32	162.32	_IRDNAIGN(de)VSCTIHNEAIGQEK_	IRDN(0.184)AIGN(0.816)VSCTIHNI	IRDN(-6.46)AIGN(6.46)VSCTIHN	8	3	814	1		1
IPI00989576	121	QEASEAYIVGIFEDTNICAIHAKRVTIMPKD	1	81.3462	143.27	81.346	_FQSAAIGAIQEASEAYIVGIFEDTN(de)ICAIHAK_	FQSAAIGAIQEASEAYIVGIFEDTN(1)I(	FQSAAIGAIQEASEAYIVGIFEDTN(81	25	3	1147	1		1
IPI00405543	56	YIISTPIVFRVGAPENVTVQAHGHTEAFDTT	1	58.0933	58.093	58.093	_VGAPEN(de)VTVQAHGHTEAFDTTVSVK_	VGAPEN(1)VTVQAHGHTEAFDTTVSVK	VGAPEN(58.09)VTVQAHGHTEAFDTT	6	3	866			1
IPI00761633	588	CDPISETQQWIFEHINMTVIEKNSHYAIS__	1	47.3795	47.38	47.38	_(ca)CDPISETQQWIFEHIN(de)MTVIEKN(de)SHYAIS_	CDPISETQQWIFEHIN(1)MTVIEKN(1)S	CDPISETQQWIFEHIN(47.38)MTVIE	16	3	1159			1

IPI00131904	106	PPNYHNETSTETRVGNNTVHVHQEVHKITNN	0.5	0	89.46	89.46	_VGN(de)NTVHVHQEVHK_	VGN(0.5)N(0.5)TVHVHQEVHK	VGN(0)N(0)TVHVHQEVHK	3	2	800				1
IPI00222589	397	TCHASNIAGQRRQDVNITVATVPTWIRKPQD	1	138.388	138.39	138.39	_RQDVN(de)ITVATVPTWIR_	RQDVN(1)ITVATVPTWIR	RQDVN(138.39)ITVATVPTWIR	5	2	936				1
IPI00122399	161	EISSDCNHIIWNYKINITTDPKFESVAREVC	1	113.706	113.71	113.71	_IN(de)ITTDPK_	IN(1)ITTDPK	IN(113.71)ITTDPK	2	2	452				1
IPI00113480	711	TYPRDFVSCNTIPKINITSWKET________	1	128.811	128.81	128.81	_IN(de)ITSWKET_	IN(1)ITSWKET	IN(128.81)ITSWKET	2	2	547				1
IPI00798466	1202	YVGDFNYVRRIFPSGNVTSVIEISSNPAHRY	0.999999	60.7761	92.897	92.897	_RIFPSGN(de)VTSVIEISSNPAHR_	RIFPSGN(1)VTSVIEISSNPAHR	RIFPSGN(60.78)VTSVIEISSN(-60	7	3	761				1
IPI00314054	190	TIRRNESHWVSISTANFTSPVIEFWERNVIS	1	171.064	171.06	171.06	_RN(de)ESHWVSISTAN(de)FTSPVIEFWER_	RN(1)ESHWVSISTAN(1)FTSPVIEFWEI	RN(171.06)ESHWVSISTAN(171.06	13	3	965				1
IPI00400016	648	WRPAISPFEFQKIINNITSIKIRGTYSERTA	0.986769	18.7262	152.97	152.97	_IINN(de)ITSIK_	IIN(0.013)N(0.987)ITSIK	IIN(-18.73)N(18.73)ITSIK	4	2	509				1
IPI00122418	417	DTNPESKESDTKNEVNGTSEDIKSEVQRKYA	1	109.073	109.07	109.07	_N(de)EVN(de)GTSEDIKSEVQRK_	N(1)EVN(1)GTSEDIKSEVQRK	N(109.07)EVN(109.07)GTSEDIKS	4	2	968				1
IPI00119299	199	IINTMISGKDTVQHWNWTSDIPIQCATHSVS	1	113.735	113.73	113.73	_DTVQHWN(de)WTSDIPIQCATHSVSIR_	DTVQHWN(1)WTSDIPIQCATHSVSIR	DTVQHWN(113.73)WTSDIPIQCATHS	7	3	951				1
IPI00320618	71	VINITHNQIRRIPPTNFTRYSQIAIIDAGFN	1	133.134	133.13	133.13	_RIPPTN(de)FTR_	RIPPTN(1)FTR	RIPPTN(133.13)FTR	6	2	552				1
IPI00120674	391	GGAFIYTSKDKVTFINTTRVDSDMNDAYIGY	1	131.433	131.43	131.43	_DKVTFIN(de)TTR_	DKVTFIN(1)TTR	DKVTFIN(131.43)TTR	7	2	598				1
IPI00323726	802	SMEIIQKFIGITPFINYTRTIRFDEDKGFWC	1	93.2576	93.258	93.258	_FIGITPFIN(de)YTR_	FIGITPFIN(1)YTR	FIGITPFIN(93.26)YTR	9	2	722				1
IPI00453707	373	YWENIITDYSKFISYNVTRRKDYYQIVPRRI	1	113.176	113.18	113.18	_FISYN(de)VTR_	FISYN(1)VTR	FISYN(113.18)VTR	5	2	501				1
IPI00129526	502	EFGTNIKIGVIEDHSNRTRIAKIIRFQSSHH	1	141.879	141.88	141.88	_IGVIEDHSN(de)R_	IGVIEDHSN(1)R	IGVIEDHSN(141.88)R	9	2	571				1
IPI00608063	832	VSTTGAVPGKPRIVINHTQMNTAIIQWHPPV	0.92416	10.8585	52.019	52.019	_IVIN(de)HTQM(ox)NTAIIQWHPPVDTFGPIQGYR_	IVIN(0.924)HTQMN(0.076)TAIIQWI	IVIN(10.86)HTQMN(-10.86)TAII	4	4	842				1
IPI00165730	236	IIQISGDIEDIEPAINKTNTKPSIGSGSCSA	1	167.614	167.61	167.61	_FTIKPIGFIIIQ(de)ISGDIEDIEPAIN(de)K_	FTIKPIGFIIIQISGDIEDIEPAIN(1)K	FTIKPIGFIIIQISGDIEDIEPAIN(16	25	3	963				1
IPI00470184	215	PSEEANTTKAVISMINETMNEAPEETKIVIK	1	156.728	311.92	156.73	_AVISM(ox)IN(de)ETM(ox)N(de)EAPEETK_	AVISMIN(1)ETMN(1)EAPEETK	AVISMIN(156.73)ETMN(156.73)E	7	2	1021				1
IPI00115892	113	MRPMSMDNMIIGPVKNVTKYGNMTQDHVMHI	0.999992	51.0268	94.259	94.259	_M(ox)ATPIIM(ox)RPM(ox)SM(ox)DNM(ox)IIGPVKN(de)VTK_	MATPIIMRPMSMDNMIIGPVKN(1)VTK	MATPIIMRPMSMDN(-51.03)MIIGPV	22	3	957				1
IPI00127447	45	QKAVDQTIEKNMVIQNGTKVFNSWEKPPIPV	1	80.3649	135.43	135.43	_NM(ox)VIQN(de)GTK_	NMVIQN(1)GTK	N(-80.36)MVIQN(80.36)GTK	6	2	511				1
IPI00406603	1811	RDIHVVHFHGQTIIDNRTKQHQIGVWPIIPG	1	110.216	110.22	110.22	_DIHVVHFHGQTIIDN(de)R_	DIHVVHFHGQTIIDN(1)R	DIHVVHFHGQTIIDN(110.22)R	15	2	951				1
IPI00113797	128	FHHRFNPKASSSFRPNGTKFAIQYGTGRISG	1	96.8199	96.82	96.82	_ASSSFRPN(de)GTK_	ASSSFRPN(1)GTK	ASSSFRPN(96.82)GTK	8	2	577				1
IPI00470184	205	TVTKKVIVAPPSEEANTTKAVISMINETMNE	1	94.0069	94.007	94.007	_KVIVAPPSEEAN(de)TTK_	KVIVAPPSEEAN(1)TTK	KVIVAPPSEEAN(94.01)TTK	12	2	793				1
IPI00132576	6	__________MDIRPNHTIYINNMNDKIKKE	0.999964	42.927	76.82	76.82	_(ac)DIRPN(de)HTIYIN(de)N(de)MNDKIK_	DIRPN(1)HTIYIN(0.848)N(0.848)	DIRPN(42.93)HTIYIN(6.62)N(6.	5	3	749				1
IPI00468539	125	NDGAWHSVRIRRQFRNTTIYIDRAEAKWVEV	1	105.951	105.95	105.95	_N(de)TTIYIDRAEAK_	N(1)TTIYIDRAEAK	N(105.95)TTIYIDRAEAK	1	2	698				1
IPI00308971	1239	DPRHGNIYDIKPIGINDTIVSVGEYTYYIRV	1	103.034	103.03	103.03	_HGN(de)IYDIKPIGIN(de)DTIVSVGEYTYYIR_	HGN(1)IYDIKPIGIN(1)DTIVSVGEYT	HGN(103.03)IYDIKPIGIN(103.03	13	3	1039				1
IPI00126424	8	________MKTISSSNNTITGFIIIGFPCPR	0.5	0	66.893	66.893	_(ac)KTISSSN(de)NTITGFIIIGFPCPR_	KTISSSN(0.5)N(0.5)TITGFIIIGFP	KTISSSN(0)N(0)TITGFIIIGFPCPR	7	3	823				1
IPI00130010	1243	YYKARDSPPFRTKCINGTINYPTCV______	0.999681	34.9545	160.67	160.67	_(ca)CIN(de)GTINYPTCV_	CIN(1)GTINYPTCV	CIN(34.95)GTIN(-34.95)YPTCV	3	2	698				1
IPI00319509	128	CNQTTDVIIIHSKKINYTIKGNHRVVIRTID	1	183.258	183.26	183.26	_KIN(de)YTIK_	KIN(1)YTIK	KIN(183.26)YTIK	3	2	441				1
IPI00129867	168	NKVSRIPQGTFSNIENITIIDIQHNKIIDNA	0.5	0	114.99	114.99	_IPQGTFSN(de)IENITIIDIQHNK_	IPQGTFSN(0.5)IEN(0.5)ITIIDIQH	IPQGTFSN(0)IEN(0)ITIIDIQHN(-	11	3	799				1
IPI00134191	43	TGVINAPETIIKDFINYTIEERIEDIPSEGI	1	152.696	152.7	152.7	_DFIN(de)YTIEER_	DFIN(1)YTIEER	DFIN(152.7)YTIEER	4	2	651				1
IPI00458077	663	VEVIFVGANPKNSAENQTHQTFITVEKYEDS	0.999866	38.7216	183.7	183.7	_NSAEN(de)QTHQTFITVEK_	NSAEN(1)QTHQTFITVEK	N(-38.72)SAEN(38.72)QTHQTFIT	5	2	924				1
IPI00760106	834	SEFMSIRITNEAHKENCTGRIEVFYNGTWGS	1	75.5554	107.99	107.99	_ITNEAHKEN(de)CTGR_	ITNEAHKEN(1)CTGR	ITN(-75.56)EAHKEN(75.56)CTGR	9	2	766				1
IPI00309999	1593	IIGDIARIEQMTMNINITGPIPAPYKIIYGI	0.953508	13.1194	92.633	92.633	_IEQM(ox)TM(ox)NIN(de)ITGPIPAPYK_	IEQMTMN(0.046)IN(0.954)ITGPIP	IEQMTMN(-13.12)IN(13.12)ITGP	9	2	1083				1
IPI00971286	241	TTVKTAIKNNSIIKKNATFSIANPVYVGNAA	0.999853	35.6363	56.017	56.017	_N(de)ATFSIAN(de)PVYVGNAAWAHIIAAR_	N(1)ATFSIAN(0.539)PVYVGN(0.46	N(35.64)ATFSIAN(0.68)PVYVGN(	1	3	844				1
IPI00321074	118	ISNIDITANKIHEISNETFRGIRRIERIYIG	1	136.957	136.96	136.96	_IHEISN(de)ETFR_	IHEISN(1)ETFR	IHEISN(136.96)ETFR	6	2	624				1
IPI00331550	60	YFEQYMDHFNFESFGNKTFGQRFIVSDKFWK	0.965269	15.896	59.628	59.628	_VIDPDFHENYFEQYM(ox)DHFNFESFGN(de)KTFGQR_	VIDPDFHEN(0.01)YFEQYMDHFN(0.0	VIDPDFHEN(-19.89)YFEQYMDHFN(	25	4	970				1
IPI00117424	158	ERITISIIRGRETIKNQTFGGAETVPQEATA	1	75.8677	75.868	75.868	_ETIKN(de)QTFGGAETVPQEATATFN(de)STAIKK_	ETIKN(1)QTFGGAETVPQEATATFN(1)	ETIKN(75.87)QTFGGAETVPQEATAT	5	3	1029				1
IPI00126343	115	CPAPIGEFEKVCSNDNKTFDSSCHFFATKCT	0.5	0	100.04	100.04	_VCSN(de)DNKTFDSSCHFFATK_	VCSN(0.5)DN(0.5)KTFDSSCHFFATK	VCSN(0)DN(0)KTFDSSCHFFATK	6	3	756				1
IPI00121430	2530	IDGYTSPGFKMIEAYNITEKNFASVQGVSIE	1	120.96	120.96	120.96	_M(ox)IEAYN(de)ITEK_	MIEAYN(1)ITEK	MIEAYN(120.96)ITEK	6	2	615				1
IPI00123342	596	ISSIFGGGTSSDAKENGTDAVQEEEESPAEG	1	104.837	104.84	104.84	_EN(de)GTDAVQEEEESPAEGSK_	EN(1)GTDAVQEEEESPAEGSK	EN(104.84)GTDAVQEEEESPAEGSK	2	2	1004				1
IPI00944683	131	SARVFFKNEKPRPAVNVTCARIFDKVKRQEK	1	81.5533	101.32	101.32	_NEKPRPAVN(de)VTCAR_	NEKPRPAVN(1)VTCAR	N(-81.55)EKPRPAVN(81.55)VTCA	9	3	538				1
IPI00125370	5	___________MEAENHTAIVHFIIIGISED	1	56.0365	56.036	56.036	_(ac)M(ox)EAEN(de)HTAIVHFIIIGISEDPK_	MEAEN(1)HTAIVHFIIIGISEDPK	MEAEN(56.04)HTAIVHFIIIGISEDP	5	3	842				1
IPI00117424	176	FGGAETVPQEATATFNSTAIKKDGINFSCQA	1	75.8677	75.868	75.868	_ETIKN(de)QTFGGAETVPQEATATFN(de)STAIKK_	ETIKN(1)QTFGGAETVPQEATATFN(1)	ETIKN(75.87)QTFGGAETVPQEATAT	23	3	1029				1
IPI00130589	87	HGGPADEERHVGDIGNVTAGKDGVANVSIED	0.988907	19.5011	108.45	108.45	_HVGDIGN(de)VTAGKDGVANVSIEDR_	HVGDIGN(0.989)VTAGKDGVAN(0.01	HVGDIGN(19.5)VTAGKDGVAN(-19.	7	3	775				1
IPI00119063	4279	TGPKCTAQVCAGYCSNNSTCTVNQGNQPQCR	0.499121	0	73.11	73.11	_CTAQVCAGYCSN(de)NSTCTVNQGNQPQCR_	CTAQVCAGYCSN(0.499)N(0.499)ST	CTAQVCAGYCSN(0)N(0)STCTVN(-2	12	3	1046				1
IPI00117914	60	YDVRDHGDIAFVDVPNDSSFQIVKNPRSVGK	0.995095	23.0721	149.47	149.47	_DHGDIAFVDVPN(de)DSSFQIVKNPR_	DHGDIAFVDVPN(0.995)DSSFQIVKN(	DHGDIAFVDVPN(23.07)DSSFQIVKN	12	3	858				1
IPI00111856	249	IHFSFYVGSKIIEYRNTSSEYHIARAEREDA	1	95.6312	95.631	95.631	_IIEYRN(de)TSSEYHIAR_	IIEYRN(1)TSSEYHIAR	IIEYRN(95.63)TSSEYHIAR	6	3	618				1
IPI00988381	14	__MTYFVCTQIKVVRNNSQVPKIIYITTTNE	1	73.2082	73.208	73.208	_(ac)MTYFVCTQ(de)IKVVRN(de)N(de)SQ(de)VPK_	MTYFVCTQIKVVRN(1)N(1)SQVPK	MTYFVCTQIKVVRN(73.21)N(73.21	14	3	820				1
IPI00222967	280	NFTGSCQPQSAQIRINNSQIKYIDFIFAVKN	0.995334	23.2903	137.18	137.18	_IN(de)NSQIK_	IN(0.995)N(0.005)SQIK	IN(23.29)N(-23.29)SQIK	2	2	409				1
IPI00321718	149	PSIVNEVIKSVVAKFNASQIITQRAQVSIII	0.950195	12.8054	174.01	174.01	_VIPSIVNEVIKSVVAKFN(de)ASQIITQR_	VIPSIVN(0.05)EVIKSVVAKFN(0.95)	VIPSIVN(-12.81)EVIKSVVAKFN(1	18	3	952				1
IPI00222915	220	CSEEEAEIREISRKINCSQEQVPGSSQCDRE	1	81.723	81.723	81.723	_KIN(de)CSQEQVPGSSQCDREPEPR_	KIN(1)CSQEQVPGSSQCDREPEPR	KIN(81.72)CSQEQVPGSSQCDREPEP	3	3	868				1
IPI00223446	571	AEIDGAKNEIQGKISNISNISHDIVQEATDH	0.80224	6.08165	163.32	163.32	_ISN(de)ISNISHDIVQEATDHAYNIQQEADEISR_	ISN(0.802)ISN(0.198)ISHDIVQEA	ISN(6.08)ISN(-6.08)ISHDIVQEA	3	3	1171				1
IPI00323235	118	KEQPRTHYYAVAVVKNSSNFHINQIQGIRSC	0.604272	1.85148	139.48	139.48	_N(de)SSNFHINQIQGIR_	N(0.604)SSN(0.395)FHIN(0.001)(	N(1.85)SSN(-1.85)FHIN(-27.04	1	2	815				1
IPI00135560	91	ISSEIHFQPDQDIIINISNASIGIHFRRQII	0.833366	6.99072	99.536	99.536	_VTQIHIISSEIHFQPDQDIIIN(de)ISNASIGIHFR_	VTQIHIISSEIHFQPDQDIIIN(0.833)	VTQIHIISSEIHFQPDQDIIIN(6.99)	22	4	940				1
IPI00120212	171	SKEAGVERFIHVSHINASMKSSSKSIRSKAV	1	85.845	85.845	85.845	_FIHVSHIN(de)ASM(ox)K_	FIHVSHIN(1)ASMK	FIHVSHIN(85.84)ASMK	8	2	701				1
IPI00471090	38	AEPRNIVTNKWPKAVNQSMIRDTVPRTDNTF	1	118.334	118.5	118.33	_AVN(de)QSM(ox)IR_	AVN(1)QSMIR	AVN(118.33)QSMIR	3	2	468				1
IPI00118291	507	NISIDIQPYKIRITANNSMGASPESIMVISN	0.500011	0	69.981	69.981	_ITAN(de)NSM(ox)GASPESIM(ox)VISN(de)DSGHEEVKEK_	ITAN(0.5)N(0.5)SMGASPESIMVISN	ITAN(0)N(0)SMGASPESIMVISN(43	4	3	1037				1
IPI00112614	521	NINKIEPIPTEVRIINKSMEIIDERKFWAGI	1	114.239	114.24	114.24	_IIN(de)KSM(ox)EIIDER_	IIN(1)KSMEIIDER	IIN(114.24)KSMEIIDER	3	2	739				1
IPI00463492	636	IGIITKPGTIIPTDRNSSMCTVMRDGVFGNY	0.99999	49.8995	84.68	84.68	_NIIYHNIGIITKPGTIIPTDRN(de)SSM(ox)CTVM(ox)R_	NIIYHNIGIITKPGTIIPTDRN(1)SSMC	N(-66.53)IIYHN(-49.9)IGIITKP	22	4	863				1
IPI00126060	300	AFNIASRVYHQAGYINFSITDDMIPHDSGIR	1	87.352	135.49	87.352	_VYHQ(de)AGYIN(de)FSITDDM(ox)IPHDSGIR_	VYHQAGYIN(1)FSITDDMIPHDSGIR	VYHQAGYIN(87.35)FSITDDMIPHDS	9	3	923				1
IPI00515432	70	AFFIKEARPITPAAINGSIQRSEPFVVFQTK	1	107.688	107.69	107.69	_EARPITPAAIN(de)GSIQR_	EARPITPAAIN(1)GSIQR	EARPITPAAIN(107.69)GSIQR	11	2	848				1
IPI00798466	419	WRSQIFIDQPQFIKFNISIQKDAIIGVYGRK	1	114.498	114.5	114.5	_FN(de)ISIQK_	FN(1)ISIQK	FN(114.5)ISIQK	2	2	426				1
IPI00115539	2	_______________MNRSIQIISTPWIGAYI	1	89.358	89.358	89.358	_(ac)M(ox)N(de)RSIQ(de)IISTPWIGAYIK_	MN(1)RSIQIISTPWIGAYIK	MN(89.36)RSIQIISTPWIGAYIK	2	2	1076				1
IPI00848693	675	HNGQGYKDQDPASFGNNSIIINSSRHYINIR	0.787183	5.51186	114.92	114.92	_N(de)HNGQGYKDQDPASFGN(de)NSIIIN(de)SSR_	N(0.5)HN(0.5)GQGYKDQDPASFGN(0.	N(0)HN(0)GQGYKDQDPASFGN(5.51	17	3	946				1
IPI00314054	179	HSWNTPQCMEDTIRRNESHWVSISTANFTSP	1	171.064	171.06	171.06	_RN(de)ESHWVSISTAN(de)FTSPVIEFWER_	RN(1)ESHWVSISTAN(1)FTSPVIEFWEI	RN(171.06)ESHWVSISTAN(171.06	2	3	965				1
IPI00471293	232	TITEEEFSRAPYTIENSSHRRVIITEIERVR	1	109.721	109.72	109.72	_APYTIEN(de)SSHR_	APYTIEN(1)SSHR	APYTIEN(109.72)SSHR	7	2	638				1
IPI00119961	88	PENACEPIVPPPIKDNSSGTFIVIIRRIDCN	1	126.832	126.83	126.83	_DN(de)SSGTFIVIIR_	DN(1)SSGTFIVIIR	DN(126.83)SSGTFIVIIR	2	2	662				1
IPI00118291	522	NNSMGASPESIMVISNDSGHEEVKEKTIKGI	0.999979	43.7285	69.981	69.981	_ITAN(de)NSM(ox)GASPESIM(ox)VISN(de)DSGHEEVKEK_	ITAN(0.5)N(0.5)SMGASPESIMVISN	ITAN(0)N(0)SMGASPESIMVISN(43	19	3	1037				1
IPI00170145	965	DPSYNGAIIVSGNEKNHSFSFACNTKRINCF	0.744846	1.38004	107.17	107.17	_DGTDDPSYNGAIIVSGN(de)EKN(de)HSFSFACNTK_	DGTDDPSYN(0.162)GAIIVSGN(0.59	DGTDDPSYN(-3.42)GAIIVSGN(1.3	20	3	1083				1
IPI00118011	89	SIDNWTYSTEFKIPFNISEWQKVKIIFDGVD	1	106.291	106.29	106.29	_IPFN(de)ISEWQK_	IPFN(1)ISEWQK	IPFN(106.29)ISEWQK	4	2	632				1
IPI00876558	633	DNIIRAEAIESWKHMNSSEQAHTATMIIDTI	0.981762	17.3103	57.347	57.347	_HM(ox)N(de)SSEQAHTATM(ox)IIDTIEEGAFVIADNIIEPTR_	HMN(0.982)SSEQAHTATMIIDTIEEGAI	HMN(17.31)SSEQAHTATMIIDTIEEG	3	4	948				1
IPI00108849	109	TYRWWIRIQRERKPNNISDTVKEIFRIVPGN	0.980934	17.1137	172.13	123.51	_KPNN(de)ISDTVK_	KPN(0.019)N(0.981)ISDTVK	KPN(-17.11)N(17.11)ISDTVK	4	2	559				1
IPI00849192	89	NIIYSYTGEHIPRITNISDATKRNNIDFSIR	1	162.26	162.26	162.26	_ITN(de)ISDATKR_	ITN(1)ISDATKR	ITN(162.26)ISDATKR	3	2	560				1
IPI00170216	351	VSGPNGPCPPGFERVNGSCVDVDECATGGRC	1	100.77	100.77	100.77	_VN(de)GSCVDVDECATGGR_	VN(1)GSCVDVDECATGGR	VN(100.77)GSCVDVDECATGGR	2	2	849				1
IPI00944049	413	RSGNTQGDFYIRQINNVSAMIVIARPVTGPR	1	130.563	130.56	130.56	_Q(de)IN(de)N(de)VSAMIVIAR_	QIN(1)N(1)VSAMIVIAR	QIN(130.56)N(130.56)VSAMIVIA	4	2	716				1
IPI00130010	1069	TCVNSRWIGQPVCKDNSCVDPPHVPNATIVT	1	114.549	114.55	114.55	_DN(de)SCVDPPHVPN(de)ATIVTR_	DN(1)SCVDPPHVPN(1)ATIVTR	DN(114.55)SCVDPPHVPN(114.55)	2	2	997				1
IPI00132474	205	KTVMPYISTTPAKIRNPCTSEQNCTSPFSYK	1	103.5	103.5	103.5	_IRN(de)PCTSEQN(de)CTSPFSYK_	IRN(1)PCTSEQN(1)CTSPFSYK	IRN(103.5)PCTSEQN(103.5)CTSP	3	3	731				1
IPI00944716	174	EHGIIRENIIDISNANRCIQARE________	0.999704	35.2865	163.12	163.12	_ENIIDISNAN(de)R_	ENIIDISNAN(1)R	EN(-130.12)IIDISN(-35.29)AN(	10	2	630				1
IPI00119063	919	ENNRCIPNRWICDGDNDCGNSEDESNATCSA	0.52425	0.421558	111.53	111.53	_WICDGDN(de)DCGNSEDESN(de)ATCSAR_	WICDGDN(0.524)DCGN(0.476)SEDE	WICDGDN(0.42)DCGN(-0.42)SEDE	7	2	1318				1
IPI00453488	260	TSISHDGSVICTFTANSTYTKSEKPVSIQPS	1	113.515	113.51	113.51	_AVTSISHDGSVICTFTAN(de)STYTK_	AVTSISHDGSVICTFTAN(1)STYTK	AVTSISHDGSVICTFTAN(113.51)ST	18	3	821	1	1	1	
IPI00848693	757	PVIDQGAEKVKAYVPNATWYDYETGEEIGWR	1	84.6534	146.1	84.653	_AYVPN(de)ATWYDYETGEEIGWR_	AYVPN(1)ATWYDYETGEEIGWR	AYVPN(84.65)ATWYDYETGEEIGWR	5	3	808	1	1	1	
IPI00109108	592	GYQITAMANRTIIVDNNTWNNTHISRVGQAM	0.999568	32.7862	259.96	147.1	_TIIVDN(de)N(de)TWNN(de)THISR_	TIIVDN(1)N(0.991)TWN(0.18)N(0.	TIIVDN(32.79)N(19.53)TWN(-6.	6	3	634	1	1	1	
IPI00115458	77	AIPYIGATCYCDIFCNRTVSDCCPDFWDFCI	1	122.939	122.94	122.94	_GRADECAIPYIGATCYCDIFCN(de)R_	GRADECAIPYIGATCYCDIFCN(1)R	GRADECAIPYIGATCYCDIFCN(122.9	22	3	928	1	1	1	
IPI00471080	664	DIIFSDDTECISNIQNKTTYKTYIGPQYITI	1	197.151	309.15	197.15	_DIIFSDDTECISN(de)IQN(de)K_	DIIFSDDTECISN(1)IQN(1)K	DIIFSDDTECISN(197.15)IQN(197	16	2	1007	1	1	1	
IPI00111183	423	DMDPETGAFRVVINFNGTSQEIMAVSEHRIY	0.999984	47.9766	142.26	142.26	_VVINFN(de)GTSQEIM(ox)AVSEHR_	VVINFN(1)GTSQEIMAVSEHR	VVIN(-47.98)FN(47.98)GTSQEIM	6	2	1075	1	1	1	
IPI00308971	1750	INFESSTHCIADRYMNYTSIITFHCKRGVSM	1	105.649	127.56	105.65	_YM(ox)N(de)YTSIITFHCK_	YMN(1)YTSIITFHCK	YMN(105.65)YTSIITFHCK	3	3	566	1	1	1	
IPI00652358	189	EDYYTHEACAPIESINGTRCAIDIRDGEEPA	1	141.359	141.36	141.36	_GFDTYFGYIIGSEDYYTHEACAPIESIN(de)GTR_	GFDTYFGYIIGSEDYYTHEACAPIESIN(	GFDTYFGYIIGSEDYYTHEACAPIESIN	28	3	1183	1	1	1	
IPI00467068	641	DSCRGDSGGAIVFIDNETQRWFVGGIVSWGS	1	186.717	186.72	186.72	_GDSGGAIVFIDN(de)ETQR_	GDSGGAIVFIDN(1)ETQR	GDSGGAIVFIDN(186.72)ETQR	12	2	840	1	1	1	
IPI00120245	74	STSSRMFIIVGAPKANTTQPGIVEGGQVIKC	1	153.725	153.72	153.72	_AN(de)TTQPGIVEGGQVIK_	AN(1)TTQPGIVEGGQVIK	AN(153.72)TTQPGIVEGGQVIK	2	2	807	1	1	1	
IPI00119063	3489	CDRDNDCVDGSDEPANCTQMTCGVDEFRCKD	1	70.2507	180.26	180.26	_VWVCDRDNDCVDGSDEPAN(de)CTQM(ox)TCGVDEFR_	VWVCDRDNDCVDGSDEPAN(1)CTQMTCG	VWVCDRDN(-70.25)DCVDGSDEPAN(	19	3	1242	1	1	1	
IPI00312063	273	REHDCKDMSDEIGCVNVTQCDGPNKFKCHSG	1	158.037	248.46	248.46	_DM(ox)SDEIGCVN(de)VTQCDGPNK_	DMSDEIGCVN(1)VTQCDGPNK	DMSDEIGCVN(158.04)VTQCDGPN(-	10	2	1078	1	1	1	
IPI00119063	2473	RVDIPQQPMGIIAVANDTNSCEISPCRINNG	0.961061	13.9236	186.44	186.44	_IIRVDIPQQPM(ox)GIIAVAN(de)DTNSCEISPCR_	IIRVDIPQQPMGIIAVAN(0.961)DTN((	IIRVDIPQQPMGIIAVAN(13.92)DTN	18	3	1096	1	1	1	

IPI00875419	216	PSEEANTTKAVISMINETMNEAPEETKIVIK	1	62.2028	278.4	62.203	_AVISM(ox)IN(de)ETM(ox)N(de)EAPEETK_	AVISMIN(1)ETMN(1)EAPEETK	AVISMIN(62.2)ETMN(62.2)EAPEE	7	3	681	1	1	1
IPI00117094	59	RCDPIRIAMCQNIGYNVTKMPNIVGHEIQTD	0.999999	58.7639	178.15	178.15	_IAM(ox)CQNIGYN(de)VTK_	IAMCQNIGYN(1)VTK	IAMCQN(-58.76)IGYN(58.76)VTK	10	2	765	1	1	1
IPI00109727	42	ACIVNQNIRIDCRHENNTKDNSIQHEFSITR	0.985871	18.4394	205.18	205.18	_HEN(de)NTKDNSIQHEFSITR_	HEN(0.986)N(0.014)TKDNSIQHEFS	HEN(18.44)N(-18.44)TKDN(-51.	3	3	724	1	1	1
IPI00875419	206	TVTKKVIVAPPSEEANTTKAVISMINETMNE	1	102.903	102.9	102.9	_VIVAPPSEEAN(de)TTK_	VIVAPPSEEAN(1)TTK	VIVAPPSEEAN(102.9)TTK	11	2	729	1	1	1
IPI00226714	223	QMEEIVEFSKKSSQSNQTIWFGHFTTSTIMS	1	121.246	149.32	121.25	_SSQSN(de)QTIWFGHFTTSTIM(ox)SPSPGIR_	SSQSN(1)QTIWFGHFTTSTIMSPSPGIR	SSQSN(121.25)QTIWFGHFTTSTIMS	5	3	962	1	1	1
IPI00356462	560	IQMISDIHVQESKISNITIIIEMEKESARGE	1	124.434	124.43	124.43	_ISN(de)ITIIIEM(ox)EKESAR_	ISN(1)ITIIIEMEKESAR	ISN(124.43)ITIIIEMEKESAR	3	2	932	1	1	1
IPI00122493	181	CPRMVQNSDFVRYHYNGTIIDGTAFDNSYSR	1	79.7113	139.11	108.18	_YHYN(de)GTIIDGTAFDNSYSR_	YHYN(1)GTIIDGTAFDNSYSR	YHYN(79.71)GTIIDGTAFDN(-79.7	4	3	732	1	1	1
IPI00621027	800	ENIYSIACDKPQQVRNMTIFSDIVAEKFIDD	1	102.505	102.5	102.5	_N(de)M(ox)TIFSDIVAEK_	N(1)MTIFSDIVAEK	N(102.5)MTIFSDIVAEK	1	2	693	1	1	1
IPI00133500	408	IIPMNETDHINMVFSNKTIEHINAIIIGAYR	0.839534	6.32225	136.68	136.68	_QSQPVHIIPM(ox)N(de)ETDHINM(ox)VFSN(de)K_	QSQPVHIIPMN(0.848)ETDHIN(0.31	QSQPVHIIPMN(6.57)ETDHIN(-6.3	22	3	905	1	1	1
IPI00109108	596	TAMANRTIIVDNNTWNNTHISRVGQAMASTE	0.999982	44.4403	265.77	171.76	_TIIVDN(de)NTWN(de)N(de)THISR_	TIIVDN(0.5)N(0.5)TWN(1)N(1)TH	TIIVDN(0)N(0)TWN(44.44)N(59.	10	3	634	1	1	1
IPI00125530	330	DNTEYEKYDYREMIHNATFCIVPRGRRIGSF	1	83.087	83.087	83.087	_EM(ox)IHN(de)ATFCIVPR_	EMIHN(1)ATFCIVPR	EMIHN(83.09)ATFCIVPR	5	2	803	1	1	1
IPI00320204	476	EVPFVFGSFFWGMKINITEEEKIINRRMMKY	1	81.3376	88.087	81.338	_IN(de)ITEEEK_	IN(1)ITEEEK	IN(81.34)ITEEEK	2	2	489	1	1	1
IPI00416285	297	HCRHSVPVFCHPSFYNDTDFIGEEIDIVDVK	1	64.954	64.954	64.954	_HSVPVFCHPSFYN(de)DTDFIGEEIDIVDVK_	HSVPVFCHPSFYN(1)DTDFIGEEIDIVD	HSVPVFCHPSFYN(64.95)DTDFIGEE	13	4	821	1	1	1
IPI00623114	1904	SIIIPTYRGVNVITVNATDADSKAFSQVMYS	1	95.903	185.26	185.26	_GVNVITVN(de)ATDADSK_	GVNVITVN(1)ATDADSK	GVN(-95.9)VITVN(95.9)ATDADSK	8	2	753	1	1	1
IPI00314443	558	NCQFETAQKKCQEAINATCKGVSYCTGNSSE	1	84.6583	84.658	84.658	_KCQEAIN(de)ATCK_	KCQEAIN(1)ATCK	KCQEAIN(84.66)ATCK	7	3	442	1	1	1
IPI00119299	647	VGIGNRIFITWRHDPNMTCDYVIKWCNSSRS	1	102.4	102.4	102.4	_HDPN(de)M(ox)TCDYVIK_	HDPN(1)MTCDYVIK	HDPN(102.4)MTCDYVIK	4	2	755	1	1	1
IPI00830749	868	ITSQEDRQIINAIQINNTAVGHAIVIPARRD	0.933806	11.4945	213.99	213.99	_QIINAIQIN(de)NTAVGHAIVIPAR_	QIINAIQIN(0.934)N(0.066)TAVGH	QIIN(-58.96)AIQIN(11.49)N(-1	9	3	776	1	1	1
IPI00128989	255	QTQVASPAIHPPVSYNDTAPRIIFWAQNFSV	1	190.67	190.67	190.67	_QIIQTQVASPAIHPPVSYN(de)DTAPR_	QIIQTQVASPAIHPPVSYN(1)DTAPR	QIIQTQVASPAIHPPVSYN(190.67)D	19	3	869	1	1	1
IPI00466733	236	RFFDHCEKFITDVERNETAIYHVEAFKTGPE	1	73.4642	195.23	73.464	_FITDVERN(de)ETAIYHVEAFK_	FITDVERN(1)ETAIYHVEAFK	FITDVERN(73.46)ETAIYHVEAFK	8	3	762	1	1	1
IPI00115530	63	IIYISAEDFSIDHSPNSTAGPSCSIIQEAFR	1	39.3881	77.907	39.388	_IIYISAEDFSIDHSPN(de)STAGPSCSIIQEAFRR_	IIYISAEDFSIDHSPN(1)STAGPSCSII	IIYISAEDFSIDHSPN(39.39)STAGP	16	4	896	1	1	1
IPI00170006	306	HRHFNTSIVFPREVMNFTAENIYKWASENQE	0.999993	51.5542	91.62	91.62	_EVM(ox)N(de)FTAENIYK_	EVMN(1)FTAENIYK	EVMN(51.55)FTAEN(-51.55)IYK	4	2	738	1	1	1
IPI00120245	941	KSIIWTETFMNKENQNHSYSIKSSASFNIIE	0.999982	47.5163	97.095	74.944	_ENQN(de)HSYSIK_	ENQN(1)HSYSIK	EN(-47.52)QN(47.52)HSYSIK	4	2	611	1	1	1
IPI00323624	1617	YIMWGISSDIWGEKPNTSYIIGKDTWVEHWP	1	88.5651	88.565	88.565	_YIM(ox)WGISSDIWGEKPN(de)TSYIIGK_	YIMWGISSDIWGEKPN(1)TSYIIGK	YIMWGISSDIWGEKPN(88.57)TSYII	16	3	892	1	1	1
IPI00881745	174	IAGFDPSQDSIGPKNNDSTINNSATTIIIGI	0.750612	7.69283	103.71	103.71	_N(de)N(de)DSTINNSATTIIIGIQDCR_	N(0.751)N(0.751)DSTIN(0.249)N	N(7.69)N(7.69)DSTIN(-7.69)N(	2	3	775	1	1	1
IPI00406302	64	GDFAISIYREIVHQSNTSNIFFSPVSIATAF	1	156.702	156.7	156.7	_EIVHQSN(de)TSN(de)IFFSPVSIATAFAM(ox)ISIGSK_	EIVHQSN(1)TSN(1)IFFSPVSIATAFA	EIVHQSN(156.7)TSN(156.7)IFFS	7	3	1072	1	1	1
IPI00387289	489	FGSPFIKDGASEEETNISKMVMKFWANFARN	1	110.923	110.92	110.92	_DGASEEETN(de)ISK_	DGASEEETN(1)ISK	DGASEEETN(110.92)ISK	9	2	641	1	1	1
IPI00320420	327	CSTNNPAQANIRQEINDSIQVAERITEQYKE	1	84.1734	156.08	84.173	_QEIN(de)DSIQVAER_	QEIN(1)DSIQVAER	QEIN(84.17)DSIQVAER	4	2	702	1	1	1
IPI00471081	72	IKTVIDKNGDAYGYYNDSIKTTGWGIIEIRA	1	71.5582	196.64	71.558	_TVIDKN(de)GDAYGYYN(de)DSIK_	TVIDKN(1)GDAYGYYN(1)DSIK	TVIDKN(71.56)GDAYGYYN(71.56)	14	2	1019	1	1	1
IPI00284816	585	VSIGVKISEVRDGQQNISIIYTEPGASQTQT	1	116.347	116.35	116.35	_DGQQN(de)ISIIYTEPGASQTQTGASFR_	DGQQN(1)ISIIYTEPGASQTQTGASFR	DGQQN(116.35)ISIIYTEPGASQTQT	5	3	891	1	1	1
IPI00115116	88	VVGISASQANISKQINISIIVKETVMQQIIT	1	87.258	119.53	87.258	_QIN(de)ISIIVK_	QIN(1)ISIIVK	QIN(87.26)ISIIVK	3	2	515	1	1	1
IPI00340140	126	TASPRCHIRATSQFQNGSIITAFIPGISQCT	1	103.534	103.53	103.53	_ATSQFQN(de)GSIITAFIPGISQCTVYSAK_	ATSQFQN(1)GSIITAFIPGISQCTVYSA	ATSQFQN(103.53)GSIITAFIPGISQ	7	3	964	1	1	1
IPI00381303	497	RISHYKQNFSFCREINISICPVSQTSERFQV	1	183.358	183.36	183.36	_EIN(de)ISICPVSQTSER_	EIN(1)ISICPVSQTSER	EIN(183.36)ISICPVSQTSER	3	2	867	1	1	1
IPI00316469	624	AGMANRTTIVDNNTWNNSHIAIVGKAMSSNE	0.999672	37.9308	361.2	202.34	_TTIVDNNTWN(de)N(de)SHIAIVGK_	TTIVDNNTWN(1)N(1)SHIAIVGK	TTIVDN(-37.93)N(-37.93)TWN(3	10	3	700	1	1	1
IPI00420835	284	VSKDDITFVSGAPRANHSGAVVIIKRDMKSA	1	122.326	122.33	122.33	_AN(de)HSGAVVIIK_	AN(1)HSGAVVIIK	AN(122.33)HSGAVVIIK	2	2	555	1	1	1
IPI00321348	325	GERRIGPGEPIEIICNVSGAIPPPGRHAAYS	1	92.3103	149.73	92.31	_IGPGEPIEIICN(de)VSGAIPPPGR_	IGPGEPIEIICN(1)VSGAIPPPGR	IGPGEPIEIICN(92.31)VSGAIPPPG	12	3	749	1	1	1
IPI00405742	761	SFGKNIDSKIQVTIYNCSFGRSDCSICIAAD	1	95.4173	122.39	95.417	_IQVTIYN(de)CSFGR_	IQVTIYN(1)CSFGR	IQVTIYN(95.42)CSFGR	7	2	730	1	1	1
IPI00626950	320	VIKDPCFNPGYEKVVNVSEIYGTPCTKRFEK	1	88.5072	240.2	88.507	_VVN(de)VSEIYGTPCTK_	VVN(1)VSEIYGTPCTK	VVN(88.51)VSEIYGTPCTK	3	2	784	1	1	1
IPI00230718	61	QHYPIPIDCRMSPWSNWSECDPCIKQRFRSR	1	115.064	115.06	115.06	_M(ox)SPWSN(de)WSECDPCIK_	MSPWSN(1)WSECDPCIK	MSPWSN(115.06)WSECDPCIK	6	3	639	1	1	1
IPI00122973	204	RTEIDIRPQGIAIFSNVSEARSIRTFDIPAT	1	58.8484	169.25	58.848	_TEIDIRPQGIAIFSN(de)VSEAR_	TEIDIRPQGIAIFSN(1)VSEAR	TEIDIRPQGIAIFSN(58.85)VSEAR	15	2	1109	1	1	1
IPI00828688	350	AGEYICKVSNYIGQANQSAWITVIPKQQAPV	1	65.7918	159.8	159.8	_VSNYIGQAN(de)QSAWITVIPK_	VSNYIGQAN(1)QSAWITVIPK	VSN(-65.79)YIGQAN(65.79)QSAW	9	2	1046	1	1	1
IPI00138180	535	EDSNFTIINNHDNTANITVKYGQFNREHAKF	0.793969	6.15565	137.72	137.72	_FAIKNEDSN(de)FTIINNHDNTAN(de)ITVK_	FAIKN(0.002)EDSN(0.89)FTIIN(0.	FAIKN(-26.34)EDSN(10.13)FTII	21	3	946		1	1
IPI00125266	341	TIFIDDRRTPAKKCINHTTQKNISFATIYDV	1	135.83	135.83	135.83	_CIN(de)HTTQK_	CIN(1)HTTQK	CIN(135.83)HTTQK	3	2	502		1	1
IPI00223987	447	NWGIITFREETIIYDNATSSVADRKIVTKII	1	126.705	126.71	126.71	_EETIIYDN(de)ATSSVADRK_	EETIIYDN(1)ATSSVADRK	EETIIYDN(126.71)ATSSVADRK	8	3	638		1	1
IPI00137958	82	GSRATHAQPAIVTAPNETSPKMPFRAPPANS	1	75.0178	75.018	75.018	_ATHAQPAIVTAPN(de)ETSPK_	ATHAQPAIVTAPN(1)ETSPK	ATHAQPAIVTAPN(75.02)ETSPK	13	3	612		1	1
IPI00471273	133	IPQDVPCPGGSNAWDNVTSFKDKQICQGQRD	1	88.5406	88.541	88.541	_GFTQIQTIIIPQDVPCPGGSN(de)AWDN(de)VTSFKDK_	GFTQIQTIIIPQDVPCPGGSN(1)AWDN(	GFTQIQTIIIPQDVPCPGGSN(88.54)	25	3	1179		1	1
IPI00466733	475	IQSCQTSKECSPPKANITSDEI__________	1	182.886	182.89	182.89	_ECSPPKAN(de)ITSDEI_	ECSPPKAN(1)ITSDEI	ECSPPKAN(182.89)ITSDEI	8	2	781		1	1
IPI00473880	459	GDQYSCMVGHEAIPMNFTQKTIDRISGKPTN	1	80.8588	111.4	80.859	_QGDQYSCM(ox)VGHEAIPM(ox)N(de)FTQK_	QGDQYSCMVGHEAIPMN(1)FTQK	QGDQYSCMVGHEAIPMN(80.86)FTQK	17	3	825		1	1
IPI00137792	518	PGSSHVVIDHETYIINITQANAAGGTPSWKR	0.942615	12.1753	68.046	68.046	_VYQIDGNYPGSSHVVIDHETYIIN(de)ITQANAAGGTPSWK_	VYQIDGNYPGSSHVVIDHETYIIN(0.94	VYQIDGN(-35.55)YPGSSHVVIDHET	24	3	1373		1	1
IPI00308885	103	DKYKNIGAKIVQDVANNTNEEAGDGTTTATV	0.333333	0	73.11	73.11	_IVQDVAN(de)NTNEEAGDGTTTATVIAR_	IVQDVAN(0.333)N(0.333)TN(0.33	IVQDVAN(0)N(0)TN(0)EEAGDGTTT	7	3	854		1	1
IPI00850698	584	KESKVPTREENIQVYNVTKAEKPGKIPKDSK	0.99977	36.3894	99.451	99.451	_VPTREENIQVYN(de)VTK_	VPTREENIQVYN(1)VTK	VPTREEN(-36.39)IQVYN(36.39)V	12	2	896		1	1
IPI00139788	513	FDEFFSQGCAPGYEKNSTICDICIGPIKCAP	1	152.876	208.72	152.88	_N(de)STICDICIGPIK_	N(1)STICDICIGPIK	N(152.88)STICDICIGPIK	1	2	746		1	1
IPI00118674	54	IYIPINKTAPCVRIINATHQIGCQSSISGDT	1	69.6388	109.68	69.639	_IIN(de)ATHQIGCQSSISGDTGVIHVVEKEEDIK_	IIN(1)ATHQIGCQSSISGDTGVIHVVEK	IIN(69.64)ATHQIGCQSSISGDTGVI	3	4	845		1	1
IPI00129243	162	NIITSTDTKSKKIPINFTEGARKSRMFKHFP	1	91.9612	91.961	91.961	_KIPIN(de)FTEGAR_	KIPIN(1)FTEGAR	KIPIN(91.96)FTEGAR	5	2	624		1	1
IPI00131091	1387	GTIKIIRTYNVIDMKNTTCQDIQIEVKVTGA	1	76.3779	180.64	135.09	_TYNVIDM(ox)KN(de)TTCQDIQIEVK_	TYNVIDMKN(1)TTCQDIQIEVK	TYN(-76.38)VIDMKN(76.38)TTCQ	9	3	811		1	1
IPI00128269	37	VYAMAVMNRHVCPVENWSYNESCSPDPAEQG	0.5	0	189.82	189.82	_HVCPVEN(de)WSYNESCSPDPAEQGGPK_	HVCPVEN(0.5)WSYN(0.5)ESCSPDPA	HVCPVEN(0)WSYN(0)ESCSPDPAEQG	7	3	949		1	1
IPI00473880	474	NFTQKTIDRISGKPTNVSVSVIMSEGDGICY	1	223.177	223.18	223.18	_ISGKPTN(de)VSVSVIM(ox)SEGDGICY_	ISGKPTN(1)VSVSVIMSEGDGICY	ISGKPTN(223.18)VSVSVIMSEGDGI	7	2	1166		1	1
IPI00221833	314	PVYRGSPSVRDWMPNNHSVIIIDDFESPQKI	0.5	0	142.26	142.26	_DWM(ox)PN(de)NHSVIIIDDFESPQK_	DWMPN(0.5)N(0.5)HSVIIIDDFESPQ	DWMPN(0)N(0)HSVIIIDDFESPQK	6	3	801		1	1
IPI00169870	376	EIDCQIVEAVDGKAMNTSQVEAMGIQMIPGY	1	50.9863	116.06	50.986	_AM(ox)N(de)TSQVEAM(ox)GIQM(ox)IPGYRDPYHGRPITK_	AMN(1)TSQVEAMGIQMIPGYRDPYHGRP	AMN(50.99)TSQVEAMGIQMIPGYRDP	3	4	828		1	1
IPI00881836	220	CSEEEAEIREISRKINCSQEVPGSSQCDREP	1	75.239	75.239	75.239	_KIN(de)CSQEVPGSSQCDREPEPR_	KIN(1)CSQEVPGSSQCDREPEPR	KIN(75.24)CSQEVPGSSQCDREPEPR	3	3	825		1	1
IPI00111908	135	YMESDGIKVAGIIVINYSNDYNHWIATKSIG	0.981166	19.6323	88.992	84.14	_VAGIIVIN(de)YSNDYNHWIATK_	VAGIIVIN(0.981)YSN(0.011)DYN(	VAGIIVIN(19.63)YSN(-19.63)DY	8	3	765		1	1
IPI00116509	656	GNEKCSQHHQGKVTINESEICAGAEKIGSGP	1	104.455	104.45	104.45	_VTIN(de)ESEICAGAEK_	VTIN(1)ESEICAGAEK	VTIN(104.45)ESEICAGAEK	4	2	761		1	1
IPI00114065	325	SKITDTICGVGNMSANASDQERTPWQVTFKP	1	70.8373	70.837	70.837	_ITDTICGVGN(de)M(ox)SAN(de)ASDQER_	ITDTICGVGN(1)MSAN(1)ASDQER	ITDTICGVGN(70.84)MSAN(70.84)	14	3	720		1	1
IPI00119299	567	KPIPINEANGKIISYNVSCSINEETQSVIEI	0.998359	27.843	109.65	109.65	_IISYN(de)VSCSINEETQSVIEIFDPQHR_	IISYN(0.998)VSCSIN(0.002)EETQ	IISYN(27.84)VSCSIN(-27.84)EE	5	3	1027		1	1
IPI00130624	234	WRSITQVKEIGVVMYNCSCIARDITKIFEAY	1	198.867	198.87	198.87	_EIGVVM(ox)YN(de)CSCIAR_	EIGVVMYN(1)CSCIAR	EIGVVMYN(198.87)CSCIAR	8	2	845		1	1
IPI00881745	179	PSQDSIGPKNNDSTINNSATTIIIGIQDCRR	0.826355	4.81185	130.83	130.83	_N(de)NDSTIN(de)NSATTIIIGIQDCR_	N(0.568)N(0.568)DSTIN(0.826)N	N(0)N(0)DSTIN(4.81)N(-13.45)	7	2	1162		1	1
IPI00114065	321	MIDVSKITDTICGVGNMSANASDQERTPWQV	1	70.8373	70.837	70.837	_ITDTICGVGN(de)M(ox)SAN(de)ASDQER_	ITDTICGVGN(1)MSAN(1)ASDQER	ITDTICGVGN(70.84)MSAN(70.84)	10	3	720		1	1
IPI00459432	166	AKFSWNNITNSIDIANISADFQGRPVDDPTG	0.973536	17.6725	63.008	63.008	_FSWNNITNSIDIAN(de)ISADFQGRPVDDPTGAFAN(de)GSITFK_	FSWN(0.005)N(0.005)ITN(0.017)	FSWN(-22.68)N(-23.3)ITN(-17.	14	3	1402		1	1
IPI00129010	124	VSAVSINKGPKCFMANGTWGYPFHDGDYIKD	1	82.5896	164.53	82.59	_CFM(ox)AN(de)GTWGYPFHDGDYIK_	CFMAN(1)GTWGYPFHDGDYIK	CFMAN(82.59)GTWGYPFHDGDYIK	5	3	766	1		1
IPI00406603	959	EDTDVDKITNSPQNQNITVPRGESTSHTNTT	0.500918	0	94.08	94.08	_GSYEIIAAN(de)GEDTDVDKITNSPQN(de)QNITVPR_	GSYEIIAAN(0.937)GEDTDVDKITN(0.	GSYEIIAAN(12.19)GEDTDVDKITN(	26	3	1121	1		1
IPI00131223	1069	PSRAYGYSGVSIKVVNSTTGTGEHIRNAIWH	1	109.845	109.84	109.84	_VVN(de)STTGTGEHIR_	VVN(1)STTGTGEHIR	VVN(109.84)STTGTGEHIR	3	3	458	1		1
IPI00115089	129	STPIYIGATAGMRIINITSPEATAKVIEAVT	1	116.238	116.24	116.24	_IIN(de)ITSPEATAK_	IIN(1)ITSPEATAK	IIN(116.24)ITSPEATAK	3	2	630	1		1
IPI00468814	437	SIYNRGFSIIIMKNINVTSIGFRSIKEISAG	0.999807	37.1416	134.3	134.3	_NIN(de)VTSIGFR_	NIN(1)VTSIGFR	N(-37.14)IN(37.14)VTSIGFR	3	2	561	1		1
IPI00111960	390	RWGYSSTAIVRQVVENMTRTHFPIDVQWNDI	1	66.2987	97.431	66.299	_QVVEN(de)M(ox)TR_	QVVEN(1)MTR	QVVEN(66.3)MTR	5	2	497	1		1
IPI00134585	669	FAIARAQIINYKIAINITMYIKSEEDFIPWE	1	72.2004	96.113	72.2	_IAIN(de)ITM(ox)YIK_	IAIN(1)ITMYIK	IAIN(72.2)ITMYIK	4	2	599	1		1
IPI00109946	70	TWIHTKNQKIIEYNYNSTKTIFESEFKGRVY	0.99572	23.6666	136.5	136.5	_IIEYNYN(de)STK_	IIEYN(0.004)YN(0.996)STK	IIEYN(-23.67)YN(23.67)STK	7	2	623	1		1
IPI00133103	216	DYFGGPKVVTPEEYFNVTIQ___________	1	108.564	162.32	108.56	_VVTPEEYFN(de)VTIQ_	VVTPEEYFN(1)VTIQ	VVTPEEYFN(108.56)VTIQ	9	3	514	1		1
IPI00409393	339	IRYGQEQGTAPFQVSNHTGRIKVVFTPSICK	1	59.5022	117.3	59.502	_YGQEQGTAPFQVSN(de)HTGR_	YGQEQGTAPFQVSN(1)HTGR	YGQEQGTAPFQVSN(59.5)HTGR	14	3	660	1		1
IPI00310059	90	SKEYSGRANIINFPENNTFVINIEQITQDDT	0.527651	0	50.869	50.869	_AN(de)IIN(de)FPENNTFVIN(de)IEQITQDDTGSYK_	AN(0.609)IIN(0.463)FPEN(0.528)	AN(0)IIN(0)FPEN(0)N(0)TFVIN(	9	3	1068	1		1
IPI00123040	210	PHFIRIGDVEVNAGQNATFQCIATGRDAVHN	1	70.536	201.27	201.27	_IGDVEVNAGQN(de)ATFQCIATGR_	IGDVEVNAGQN(1)ATFQCIATGR	IGDVEVN(-70.54)AGQN(70.54)AT	11	2	1112	1		1
IPI00134390	332	DVSKYPDIIAEIIRRNWTETEVRGIIADNII	1	101.974	101.97	101.97	_RN(de)WTETEVR_	RN(1)WTETEVR	RN(101.97)WTETEVR	2	2	596	1		1
IPI00165807	80	HNSIHQRIIIKAPSHNTTEPDPHSISPEIQA	1	41.9364	48.607	41.936	_IIIKAPSHN(de)TTEPDPHSISPEIQAIISEVAQHDVQN(de)GR_	IIIKAPSHN(1)TTEPDPHSISPEIQAII	IIIKAPSHN(41.94)TTEPDPHSISPE	9	4	1037	1		1
IPI00119809	572	AFSSFRVVIRPFYITNSTDMV__________	1	147.561	209.79	147.56	_VVIRPFYITN(de)STDM(ox)V_	VVIRPFYITN(1)STDMV	VVIRPFYITN(147.56)STDMV	10	2	886	1		1
IPI00469426	257	TSVGVTQARITWSNANGTASYRMIIEEITTH	1	87.4985	88.075	87.498	_ITWSN(de)AN(de)GTASYR_	ITWSN(1)AN(1)GTASYR	ITWSN(87.5)AN(87.5)GTASYR	7	2	722	1		1
IPI00265291	323	DVEIDGIIPDIYKVYNGSVPFEERIIAVIEW	1	95.0939	95.094	95.094	_VYN(de)GSVPFEER_	VYN(1)GSVPFEER	VYN(95.09)GSVPFEER	3	2	649	1		1
IPI00121421	306	NTADWIQIEKIRAPSNVSTVIHIIYIPEEAK	1	95.6224	95.622	95.622	_APSN(de)VSTVIHIIYIPEEAK_	APSN(1)VSTVIHIIYIPEEAK	APSN(95.62)VSTVIHIIYIPEEAK	4	2	1042	1		1
IPI01026847	220	PIKFDKIIMNEGGHYNASSGKFVCSVPGIYY	0.999992	51.2191	85.006	85.006	_IIM(ox)NEGGHYN(de)ASSGK_	IIMNEGGHYN(1)ASSGK	IIMN(-51.22)EGGHYN(51.22)ASS	10	2	798	1		1
IPI00263041	249	DAFIKAHFSPIWKNFNDSIHSISQAIRNISI	0.985623	18.3605	93.823	93.823	_NFN(de)DSIHSISQAIR_	N(0.014)FN(0.986)DSIHSISQAIR	N(-18.36)FN(18.36)DSIHSISQAI	3	2	802	1		1
IPI00788443	734	TGEQVTVRCQSPYQMNGSDTVTCINGRWIGK	1	132.735	137.44	132.74	_CQSPYQM(ox)N(de)GSDTVTCIN(de)GR_	CQSPYQMN(1)GSDTVTCIN(1)GR	CQSPYQMN(132.74)GSDTVTCIN(13	8	2	1103	1		1
IPI00115793	467	IKIVKIPDFESRYVQNGTYTAKVVAISKEHP	1	99.2826	99.283	99.283	_YVQN(de)GTYTAK_	YVQN(1)GTYTAK	YVQN(99.28)GTYTAK	4	2	573			1
IPI00316359	543	ATFYINVIQQRQMAINATYIFNGITVSIPGM	0.8004	6.03147	59.585	59.585	_QM(ox)AIN(de)ATYIFNGITVSIPGM(ox)EK_	QMAIN(0.8)ATYIFN(0.2)GITVSIPG	QMAIN(6.03)ATYIFN(-6.03)GITV	5	3	811			1
IPI00121120	1398	TQMTFIRIISKEASQNITYICRNTVGYMDDQ	1	107.055	107.06	107.06	_EASQN(de)ITYICR_	EASQN(1)ITYICR	EASQN(107.06)ITYICR	5	2	678			1
IPI00469426	425	SIVYKIHVAGGTHSVNQTVNKTEAIIIGISS	1	95.5698	95.57	95.57	_IHVAGGTHSVN(de)QTVN(de)K_	IHVAGGTHSVN(1)QTVN(1)K	IHVAGGTHSVN(95.57)QTVN(95.57	11	2	832			1

IPI00930882	438	WNESSIFHIPRPVSINMTVKGQNEDPGSIIT	1	64.7228	64.723	64.723 _APIM(ox)PWN(de)ESSIFHIPRPVSIN(de)M(ox)TVK_	APIMPWN(1)ESSIFHIPRPVSIN(1)MT	APIMPWN(64.72)ESSIFHIPRPVSIN	21	3	967			1
IPI00111370	362	SDEHFQPRPEAVQFFNVTTIQKEIYDFAQEH	1	78.4285	78.428	78.428 _DKWGISDEHFQPRPEAVQFFN(de)VTTIQK_	DKWGISDEHFQPRPEAVQFFN(1)VTTIQK	DKWGISDEHFQPRPEAVQFFN(78.43)	21	3	1074			1
IPI00121430	2681	IDCYEIIEKDIKEAGNITTDGYEIIGKIIKG	1	103.66	103.66	103.66 _DIKEAGN(de)ITTDGYEIIGK_	DIKEAGN(1)ITTDGYEIIGK	DIKEAGN(103.66)ITTDGYEIIGK	7	2	969			1
IPI00131216	80	RFAPPQPAEPWSSVKNATSYPPMCFQDPVTG	0.973835	17.2751	61.616	61.616 _N(de)ATSYPPM(ox)CFQDPVTGQIVNDIITNRK_	N(0.974)ATSYPPMCFQDPVTGQIVN(0.	N(17.28)ATSYPPMCFQDPVTGQIVN(	1	3	1033			1
IPI01027808	1437	NKAHEAERIASAVQKNATSTKADAERTFGEV	1	136.604	136.6	136.6 _IASAVQKN(de)ATSTK_	IASAVQKN(1)ATSTK	IASAVQKN(136.6)ATSTK	8	2	660			1
IPI00849185	9	_______MGTPSHEINTTSSGAEVIQKTIEE	1	120.106	120.11	120.11 _(ac)GTPSHEIN(de)TTSSGAEVIQK_	GTPSHEIN(1)TTSSGAEVIQK	GTPSHEIN(120.11)TTSSGAEVIQK	8	2	1000			1
IPI00555130	508	HCRIIYPGYKIMGINNVTSQSWQPQTYQICI	0.795124	5.88944	43.895	43.895 _IMGINN(de)VTSQ(de)SWQ(de)PQ(de)TYQ(de)ICIVDPVSASVK_	IMGIN(0.205)N(0.795)VTSQSWQPQ	IMGIN(-5.89)N(5.89)VTSQSWQPQ	6	3	1124			1
IPI00115516	767	ISRHVAGIWAAVRESNSTSITQAAIIEKIIG	1	167.71	167.71	167.71 _ESN(de)STSITQAAIIEK_	ESN(1)STSITQAAIIEK	ESN(167.71)STSITQAAIIEK	3	2	797			1
IPI01027808	648	WRPAISPFEFQKIINNITSIKIRGTYSERSA	0.866178	8.11079	121.73	121.73 _IINN(de)ITSIK_	IIN(0.134)N(0.866)ITSIK	IIN(-8.11)N(8.11)ITSIK	4	2	509			1
IPI00229935	480	RTVPIAVIQIRVKHANETSIGITWRAPIGEW	1	90.6136	90.614	90.614 _HAN(de)ETSIGITWR_	HAN(1)ETSIGITWR	HAN(90.61)ETSIGITWR	3	2	693			1
IPI00129158	312	NDTPKNITKNTDGTYNYTSIFIVNSSAHRED	0.999998	56.9735	79.514	79.514 _NTDGTYN(de)YTSIFIVN(de)SSAHR_	NTDGTYN(1)YTSIFIVN(1)SSAHR	N(-56.97)TDGTYN(56.97)YTSIFI	7	3	755			1
IPI00461384	1364	RWRKAISVKHGKHISNATSATHEHIEGPATN	0.989273	19.6482	56.327	56.327 _HISN(de)ATSATHEHIEGPATNDIK_	HISN(0.989)ATSATHEHIEGPATN(0.	HISN(19.65)ATSATHEHIEGPATN(-	4	3	782			1
IPI00553703	1573	KFIVTQVPVHGHIIFNNTRSVMVFTKQDINE	0.848772	7.49157	70.529	70.529 _FIVTQVPVHGHIIFN(de)NTR_	FIVTQVPVHGHIIFN(0.849)N(0.151)	FIVTQVPVHGHIIFN(7.49)N(-7.49	15	3	698			1
IPI00675799	1096	YGSRYQEIIEIESSINKTQWKSQVHSVAQSK	1	209.254	209.25	209.25 _YQEIIEIESSIN(de)KTQWK_	YQEIIEIESSIN(1)KTQWK	YQEIIEIESSIN(209.25)KTQWK	12	2	1056			1
IPI00457533	1034	VSSGTGIPDMTGSVYNKTQTFDKQGFHAGTP	1	64.275	64.275	64.275 _GVSVSSGTGIPDMTGSVYN(de)K_	GVSVSSGTGIPDMTGSVYN(1)K	GVSVSSGTGIPDMTGSVYN(64.27)K	19	2	979			1
IPI00675799	614	RAEQITIHAIGIGEANKTQIRQIAGKDERVN	1	135.492	135.49	135.49 _IRAEQITIHAIGIGEAN(de)KTQIR_	IRAEQITIHAIGIGEAN(1)KTQIR	IRAEQITIHAIGIGEAN(135.49)KTQ	17	3	812			1
IPI00928320	77	ICKTVVTEAGNIIKDNATQEEIIHYIEKTCE	1	167.688	167.69	167.69 _DN(de)ATQ(de)EEIIHYIEK_	DN(1)ATQEEIIHYIEK	DN(167.69)ATQEEIIHYIEK	2	2	853			1
IPI00121833	58	GRRTPIGRASRGGFKNTTPDEIISAVITAVI	1	166.516	166.52	166.52 _N(de)TTPDEIISAVITAVIQDVR_	N(1)TTPDEIISAVITAVIQDVR	N(166.52)TTPDEIISAVITAVIQDVR	1	3	719			1
IPI00345112	922	VPVVQIHRQSPARIINCTNIDCIQISCAVGR	0.794867	5.8826	122.49	122.49 _IIN(de)CTNIDCIQISCAVGR_	IIN(0.795)CTN(0.205)IDCIQISCA	IIN(5.88)CTN(-5.88)IDCIQISCA	3	2	1055			1
IPI00129968	118	TWKKDDEPIETTGDFNTTKMGNTITSQYRFI	1	111.064	111.06	111.06 _KDDEPIETTGDFN(de)TTK_	KDDEPIETTGDFN(1)TTK	KDDEPIETTGDFN(111.06)TTK	13	2	906			1
IPI00461281	144	KENPGFDFSGAEISGNYTKGGPDFSNIEK__	1	69.9019	82.265	82.265 _ENPGFDFSGAEISGN(de)YTK_	ENPGFDFSGAEISGN(1)YTK	EN(-69.9)PGFDFSGAEISGN(69.9)	15	2	967			1
IPI00128628	19	IYSEYINPEKVIEHYNYTKETIDMQETTSRK	1	140.894	140.89	140.89 _VIEHYN(de)YTKETIDM(ox)QETTSR_	VIEHYN(1)YTKETIDMQETTSR	VIEHYN(140.89)YTKETIDMQETTSR	6	3	826			1
IPI00928320	453	VCKKIVIYIEHNIEKNSTKEEIIAAIEKGCS	1	171.255	264.92	171.26 _N(de)STKEEIIAAIEK_	N(1)STKEEIIAAIEK	N(171.26)STKEEIIAAIEK	1	2	724			1
IPI00330381	368	PKYIIDFSIFAYPIPNVTKEACHQ_______	1	118.47	118.47	118.47 _YIIDFSIFAYPIPN(de)VTK_	YIIDFSIFAYPIPN(1)VTK	YIIDFSIFAYPIPN(118.47)VTK	14	2	1002			1
IPI00228716	1828	IVRADDGIQSSDMRINITISDVNDHTPRFSR	0.999996	53.6643	95.981	95.981 _IN(de)ITISDVNDHTPR_	IN(1)ITISDVNDHTPR	IN(53.66)ITISDVN(-53.66)DHTP	2	2	798			1
IPI00556788	327	VEVHTAQTQTHREDYNSTIRVVSAIPIQHQD	1	121.092	121.09	121.09 _EDYN(de)STIR_	EDYN(1)STIR	EDYN(121.09)STIR	4	2	500			1
IPI00135231	96	KRVEEFKIKQMWKSPNGTIRNIIGGTVFREA	1	71.2626	71.263	71.263 _(gl)Q(de)M(ox)WKSPN(de)GTIRN(de)IIGGTVFR_	QMWKSPN(1)GTIRN(1)IIGGTVFR	QMWKSPN(71.26)GTIRN(71.26)II	7	3	760			1
IPI00129465	15	_MATTHAQGHQPVIGNDTIREHYDYVGKIAG	1	84.6534	84.653	84.653 _(ac)ATTHAQGHQPVIGN(de)DTIR_	ATTHAQGHQPVIGN(1)DTIR	ATTHAQGHQPVIGN(84.65)DTIR	14	2	980			1
IPI00153317	246	PGAWTEACGQKITFFNSTINTSGIVAQGEAI	0.5	0	72.231	72.231 _ITFFN(de)STINTSGIVAQGEAIPIPGAHRPGIVTK_	ITFFN(0.5)STIN(0.5)TSGIVAQGEA	ITFFN(0)STIN(0)TSGIVAQGEAIPI	5	4	853			1
IPI00757554	77	GQVTIVIIQEAYKQYNATINRFSVNFQKAAK	0.758236	4.96412	76.817	76.817 _Q(de)Q(de)GGQ(de)VTIVIIQ(de)EAYKQ(de)YN(de)ATINR_	QQGGQVTIVIIQEAYKQYN(0.758)ATI	QQGGQVTIVIIQEAYKQYN(4.96)ATI	19	2	1371			1
IPI00154056	267	KARIQGGVIIAQIIKNITIMATTSQFPKIIV	1	131.325	131.33	131.33 _ARIQGGVIIAQIIKN(de)ITIM(ox)ATTSQFPK_	ARIQGGVIIAQIIKN(1)ITIMATTSQFP	ARIQGGVIIAQIIKN(131.33)ITIMA	15	3	977			1
IPI00136642	129	ISISTAFAMTKIGACNDTIKQIMEVFKFDTI	1	205.827	205.83	205.83 _IGACN(de)DTIK_	IGACN(1)DTIK	IGACN(205.83)DTIK	5	2	497			1
IPI00118291	358	ATKANMTWKVHSHGNNYTIICQVKIQYGGEV	0.846229	7.40612	118.52	118.52 _VHSHGNN(de)YTIICQVK_	VHSHGN(0.154)N(0.846)YTIICQVK	VHSHGN(-7.41)N(7.41)YTIICQVK	7	2	886			1
IPI00675799	867	DHSGSIGPREQESMMNITIHIVKKADVGRDR	1	89.2306	89.231	89.231 _EQESM(ox)M(ox)N(de)ITIHIVK_	EQESMMN(1)ITIHIVK	EQESMMN(89.23)ITIHIVK	7	2	853			1
IPI00109109	97	VAIQPAIKFNGGGHINHTIFWTNISPKGGGE	0.840008	7.20291	118.55	118.55 _FNGGGHIN(de)HTIFWTNISPK_	FN(0.16)GGGHIN(0.84)HTIFWTNIS	FN(-7.2)GGGHIN(7.2)HTIFWTN(-	8	3	714			1
IPI00128484	246	PGRGHGRPRNGTAHGNSTHPMHSRCSPDPGI	1	71.3538	71.354	71.354 _N(de)GTAHGN(de)STHPM(ox)HSR_	N(1)GTAHGN(1)STHPMHSR	N(71.35)GTAHGN(71.35)STHPMHS	7	2	811			1
IPI00420412	6	__________MDEFCNSTFWNISIIKSPEAD	1	214.224	214.22	214.22 _(ac)M(ox)DEFCN(de)STFWN(de)ISIIK_	MDEFCN(1)STFWN(1)ISIIK	MDEFCN(214.22)STFWN(214.22)I	6	2	1033			1
IPI00120015	332	KPNFEEFFEIITPKINQTFSGIMTMINMQFV	0.999996	54.3415	66.809	66.809 _IN(de)QTFSGIM(ox)TM(ox)INM(ox)QFVIR_	IN(1)QTFSGIMTMINMQFVIR	IN(54.34)QTFSGIMTMIN(-54.34)	2	2	1147			1
IPI00460063	323	YDIVIVAAPINRKMSNITFRNFDPPIEEFND	1	143.703	143.7	143.7 _KM(ox)SN(de)ITFR_	KMSN(1)ITFR	KMSN(143.7)ITFR	4	2	507			1
IPI00120856	107	FYDTDDATFKWYDHSNMTFDKWADQDGEDIV	1	210.224	210.22	210.22 _WYDHSN(de)M(ox)TFDKWADQDGEDIVDTCGFIYTK_	WYDHSN(1)MTFDKWADQDGEDIVDTCGF	WYDHSN(210.22)MTFDKWADQDGEDI	6	3	1226			1
IPI00127461	6	__________MARENNVTEIIITGIFQDPNV	1	61.9621	61.962	61.962 _EN(de)N(de)VTEIIITGIFQ(de)DPN(de)VQ(de)K_	EN(1)N(1)VTEIIITGIFQDPN(1)VQK	EN(61.96)N(61.96)VTEIIITGIFQ	3	3	760			1
IPI00112322	327	GDHGVVVFSIGSMVSNMTEEKANAIAWAIAQ	1	46.6296	46.63	46.63 _DM(ox)EEFVQSSGDHGVVVFSIGSM(ox)VSN(de)M(ox)TEEK_	DMEEFVQSSGDHGVVVFSIGSMVSN(1)M	DMEEFVQSSGDHGVVVFSIGSMVSN(46	25	3	1109			1
IPI00928320	328	TCQFVMNKFSEIIVNNATEEIIVKGISNACA	0.841119	7.23785	118.21	118.21 _FSEIIVNN(de)ATEEIIVK_	FSEIIVN(0.159)N(0.841)ATEEIIV	FSEIIVN(-7.24)N(7.24)ATEEIIV	8	2	910			1
IPI00137311	1041	ININRAQISNPEVKYNYTEDPTIIRIDPEWS	1	130.565	130.56	130.56 _YN(de)YTEDPTIIR_	YN(1)YTEDPTIIR	YN(130.56)YTEDPTIIR	2	2	693			1
IPI00469426	429	KIHVAGGTHSVNQTVNKTEAIIIGISSSTIY	1	95.5698	95.57	95.57 _IHVAGGTHSVN(de)QTVN(de)K_	IHVAGGTHSVN(1)QTVN(1)K	IHVAGGTHSVN(95.57)QTVN(95.57	15	2	832			1
IPI00848693	1808	EVFANYNSTTKIISVNMTDKKISIHTFNSIT	1	106.4	106.4	106.4 _IISVN(de)M(ox)TDKK_	IISVN(1)MTDKK	IISVN(106.4)MTDKK	5	2	583			1
IPI00416285	490	ESGYDIAIIKIESAMNYTDFQRPICIPSKGD	1	140.277	140.28	140.28 _IESAM(ox)N(de)YTDFQRPICIPSKGDR_	IESAMN(1)YTDFQRPICIPSKGDR	IESAMN(140.28)YTDFQRPICIPSKG	6	3	872			1
IPI00138061	98	GHCPAPSQIPSAKPINITDESMFPIGTYIIY	1	124.656	124.66	124.66 _PIN(de)ITDESM(ox)FPIGTYIIYECIPGYIKR_	PIN(1)ITDESMFPIGTYIIYECIPGYIK	PIN(124.66)ITDESMFPIGTYIIYEC	3	3	1074			1
IPI00230194	4	____________MASNNTASIAQARKIVEQI	1	34.3856	34.386	34.386 _(ac)MASN(de)N(de)TASIAQ(de)AR_	MASN(1)N(1)TASIAQAR	MASN(34.39)N(34.39)TASIAQAR	4	2	690			1
IPI00128484	240	DYFVSCPGRGHGRPRNGTAHGNSTHPMHSRC	1	71.3538	71.354	71.354 _N(de)GTAHGN(de)STHPM(ox)HSR_	N(1)GTAHGN(1)STHPMHSR	N(71.35)GTAHGN(71.35)STHPMHS	1	2	811			1
IPI01027808	1378	DIINNIKDFDRRVNDNKTAAEEAIRRIPAIN	0.999975	46.0964	158.52	158.52 _RVNDN(de)KTAAEEAIR_	RVNDN(1)KTAAEEAIR	RVN(-46.1)DN(46.1)KTAAEEAIR	5	2	794			1
IPI00775779	205	QEIHPIETTVNPSGKNVSYNISSTVRVVINS	0.997237	25.5713	89.272	89.272 _DGQEIHPIETTVNPSGKN(de)VSYN(de)ISSTVR_	DGQEIHPIETTVN(0.004)PSGKN(0.9	DGQEIHPIETTVN(-25.57)PSGKN(2	18	3	1015			1
IPI00623114	3718	SGGTHVSTKQIIHKINSSVTDVEEIIGVRII	1	135.137	135.14	135.14 _IN(de)SSVTDVEEIIGVR_	IN(1)SSVTDVEEIIGVR	IN(135.14)SSVTDVEEIIGVR	2	2	816			1
IPI00309741	840	IEDKIRQFVDICSMSNVSVFIISHRCFGYYI	1	61.6164	61.616	61.616 _FIEDKIRQ(de)FVDICSMSN(de)VSVFIISHR_	FIEDKIRQFVDICSMSN(1)VSVFIISHR	FIEDKIRQFVDICSMSN(61.62)VSVF	17	3	1048			1
IPI01008188	754	ENRKEFVNIYSDYIINKSVEKQFKAFRRGFH	1	79.492	79.492	79.492 _(gl)EFVN(de)IYSDYIIN(de)K_	EFVN(1)IYSDYIIN(1)K	EFVN(79.49)IYSDYIIN(79.49)K	12	2	801			1
IPI00126685	5	___________MENGNISTVTVFVFTGFPQI	1	36.2533	36.253	36.253 _(ac)MEN(de)GN(de)ISTVTVFVFTGFPQ(de)IK_	MEN(1)GN(1)ISTVTVFVFTGFPQIK	MEN(36.25)GN(36.25)ISTVTVFVF	5	3	792			1
IPI00329843	737	SGCIQTIIHRAVDENNESTACFIIRSGCDVN	0.797539	4.68232	67.866	67.866 _AVDEN(de)N(de)ESTACFIIRSGCDVNSPR_	AVDEN(0.798)N(0.798)ESTACFIIR	AVDEN(4.68)N(4.68)ESTACFIIRS	6	3	905			1
IPI00775779	209	PIETTVNPSGKNVSYNISSTVRVVINSMDVN	0.999209	31.0004	89.272	89.272 _DGQEIHPIETTVNPSGKN(de)VSYN(de)ISSTVR_	DGQEIHPIETTVN(0.004)PSGKN(0.9	DGQEIHPIETTVN(-25.57)PSGKN(2	22	3	1015			1
IPI00466999	398	SDGVEIIQIVKAPDSNSSSIIITTRQGIVII	1	128.063	128.06	128.06 _APDSN(de)SSSIIITTR_	APDSN(1)SSSIIITTR	APDSN(128.06)SSSIIITTR	5	2	732			1
IPI00930882	424	GAIPGQPAKAPIMPWNESSIFHIPRPVSINM	1	64.7228	64.723	64.723 _APIM(ox)PWN(de)ESSIFHIPRPVSIN(de)M(ox)TVK_	APIMPWN(1)ESSIFHIPRPVSIN(1)MT	APIMPWN(64.72)ESSIFHIPRPVSIN	7	3	967			1
IPI00221547	464	HAPNVIDTGHNFAIINSSEPYFGDGPIKSK	0.818544	8.24009	66.378	66.378 _VIPEPIHAPNVIDTGHNFAIIN(de)SSEPYFGDGPIK_	VIPEPIHAPN(0.059)VIDTGHN(0.12	VIPEPIHAPN(-11.44)VIDTGHN(-8	22	3	1258			1
IPI00223352	256	DGASINFSHWNSGEPNDSRGHEDCIMMIHSG	1	69.7632	69.763	69.763 _WVDGASIN(de)FSHWN(de)SGEPN(de)DSR_	WVDGASIN(1)FSHWN(1)SGEPN(1)DS	WVDGASIN(69.76)FSHWN(69.76)S	18	2	1190			1
IPI00944760	188	KAEWSTKNYSCEAKNNISREISEIKKFPIVV	1	113.687	113.69	113.69 _N(de)N(de)ISREISEIK_	N(1)N(1)ISREISEIK	N(113.69)N(113.69)ISREISEIK	2	2	653			1
IPI01027808	1239	AIEIEEINRKYEQAKNISQDIEKQAARVHEE	1	159.178	196.95	159.18 _KYEQAKN(de)ISQDIEK_	KYEQAKN(1)ISQDIEK	KYEQAKN(159.18)ISQDIEK	7	2	848			1
IPI00464135	53	PIGSEERIIRKFEAENISNYTAIIISQDGKT	0.5	0	105.39	105.39 _KFEAEN(de)ISNYTAIIISQDGK_	KFEAEN(0.5)ISN(0.5)YTAIIISQDG	KFEAEN(0)ISN(0)YTAIIISQDGK	6	3	748			1
IPI00128287	220	GKNFPRKSEEMINIVNNSKDFVENVTSGNAV	0.316479	0	107.45	107.45 _KSEEM(ox)IN(de)IVNNSKDFVENVTSGNAVDFFPVIR_	KSEEMIN(0.316)IVN(0.316)N(0.3	KSEEMIN(0)IVN(0)N(0)SKDFVEN(	10	3	1211			1
IPI00173156	57	YEETKKETIEQISEFNDSIKKIMSGNMTIVD	1	102.621	102.62	102.62 _ETIEQISEFN(de)DSIK_	ETIEQISEFN(1)DSIK	ETIEQISEFN(102.62)DSIK	10	2	827			1
IPI00420412	11	_____MDEFCNSTFWNISIIKSPEADIPICF	1	214.224	214.22	214.22 _(ac)M(ox)DEFCN(de)STFWN(de)ISIIK_	MDEFCN(1)STFWN(1)ISIIK	MDEFCN(214.22)STFWN(214.22)I	11	2	1033			1
IPI01027808	1105	VKDVDQNIMDRIQRVNSSIHSQISRIQNIRN	1	83.1372	215.72	83.137 _VN(de)SSIHSQISR_	VN(1)SSIHSQISR	VN(83.14)SSIHSQISR	2	2	615			1
IPI00123942	437	DRHTDMDRIFNIDSGNGSIFTSKIIDRETII	1	88.5523	88.552	88.552 _IFN(de)IDSGN(de)GSIFTSK_	IFN(1)IDSGN(1)GSIFTSK	IFN(88.55)IDSGN(88.55)GSIFTS	8	2	801			1
IPI00330670	203	DAISAYKDNKIQIQQNISIEFIKIHQFIHNK	1	79.8312	83.087	83.087 _DNKIQ(de)IQ(de)Q(de)N(de)ISIEFIK_	DNKIQIQQN(1)ISIEFIK	DN(-79.83)KIQIQQN(79.83)ISIE	9	2	968			1
IPI00458077	74	QPPPITQTPNFPSFRNFSGYYIGVGRADCTG	1	109.236	109.24	109.24 _N(de)FSGYYIGVGR_	N(1)FSGYYIGVGR	N(109.24)FSGYYIGVGR	1	2	617			1
IPI00987265	1088	QQVATISATTRWQIHNISGKVWVQNATVDVP	1	114.401	114.4	114.4 _WQIHN(de)ISGK_	WQIHN(1)ISGK	WQIHN(114.4)ISGK	5	2	542			1
IPI00463399	77	SHSKKTVKKVVVEQNGSFQVKIPKNFICEH	1	98.1049	98.105	98.105 _KVVVVEQ(de)N(de)GSFQ(de)VK_	KVVVVEQN(1)GSFQVK	KVVVVEQN(98.1)GSFQVK	8	2	782			1
IPI00405437	396	SVPVVINIISSRQIMNTSFNPIRIVNTYGAF	1	65.8551	65.855	65.855 _QIM(ox)N(de)TSFN(de)PIRIVN(de)TYGAFGSVTK_	QIMN(1)TSFN(1)PIRIVN(1)TYGAFG	QIMN(65.86)TSFN(65.86)PIRIVN	4	3	893			1
IPI00115607	308	IKPAFIKPYGTVTAANSSFFITDGASAMIIMS	1	46.1504	47.175	46.15 _IKPAFIKPYGTVTAAN(de)SSFFITDGASAM(ox)IIM(ox)SEDR_	IKPAFIKPYGTVTAAN(1)SSFFITDGASA	IKPAFIKPYGTVTAAN(46.15)SSFIT	16	4	910			1
IPI00928320	211	DCMKIVSDVQTAVKTNSSFIQGFVDHVKEDC	1	73.6546	73.655	73.655 _IVSDVQTAVKTN(de)SSFIQGFVDHVKEDCDR_	IVSDVQTAVKTN(1)SSFIQGFVDHVKED	IVSDVQTAVKTN(73.65)SSFIQGFVD	12	3	1099			1
IPI00469218	79	ASAEVIKNGSSCGKENVSDPSITITFGRGYI	1	214.472	214.47	214.47 _EN(de)VSDPSITITFGR_	EN(1)VSDPSITITFGR	EN(214.47)VSDPSITITFGR	2	2	769			1
IPI00228879	133	ISIGDVAKKIGEMWNNISDNEKQPYVTKAAK	1	82.6628	82.663	82.663 _IGEM(ox)WN(de)N(de)ISDN(de)EKQ(de)PYVTK_	IGEMWN(1)N(1)ISDN(1)EKQPYVTK	IGEMWN(82.66)N(82.66)ISDN(82	7	3	763			1
IPI00121475	482	IDIIIENMGRISFGSNHSDFKGIIEAPIIGQ	1	88.3377	88.338	88.338 _ISFGSN(de)HSDFK_	ISFGSN(1)HSDFK	ISFGSN(88.34)HSDFK	6	2	620			1
IPI00377642	42	ASAQEPPRVGCSEYTNRSCEECIRNVSCIWC	1	125.969	125.97	125.97 _VGCSEYTN(de)R_	VGCSEYTN(1)R	VGCSEYTN(125.97)R	8	2	544			1
IPI00129159	84	EPVDTISVRGSSVIINCSAYSEPSPNIEWKK	1	90.6728	175.65	175.65 _GSSVIIN(de)CSAYSEPSPNIEWK_	GSSVIIN(1)CSAYSEPSPNIEWK	GSSVIIN(90.67)CSAYSEPSPN(-90	7	2	1170			1
IPI00123881	521	VSGHYDRSKSIKQNINISAPIMSRFDIFFII	1	97.5513	97.551	97.551 _SIKQN(de)IN(de)ISAPIM(ox)SR_	SIKQN(1)IN(1)ISAPIMSR	SIKQN(97.55)IN(97.55)ISAPIMS	7	3	564			1
IPI00129158	320	KNTDGTYNYTSIFIVNSSAHREDVVFTCQVK	0.999999	60.5915	79.514	79.514 _NTDGTYN(de)YTSIFIVN(de)SSAHR_	NTDGTYN(1)YTSIFIVN(1)SSAHR	N(-56.97)TDGTYN(56.97)YTSIFI	15	3	755			1
IPI01008326	173	EHGIIRENIIDISNANRCIQARE________:	0.999504	33.0466	160.22	160.22 _ENIIDISNAN(de)R_	ENIIDISNAN(1)R	EN(-155.88)IIDISN(-33.05)AN(	10	2	630			1
IPI00118674	561	DDRPIQHYIAVSSPTNTTYVVQYAIANITGK	1	125.587	125.59	125.59 _SYIDDRPIQHYIAVSSPTN(de)TTYVVQYAIAN(de)ITGK_	SYIDDRPIQHYIAVSSPTN(1)TTYVVQY	SYIDDRPIQHYIAVSSPTN(125.59)T	19	3	1268	1	1	
IPI00116476	118	RYDVSIIIWKIQFDDNGTYTCQVKNPPDVDG	1	115.816	115.82	115.82 _IQFDDN(de)GTYTCQVK_	IQFDDN(1)GTYTCQVK	IQFDDN(115.82)GTYTCQVK	6	2	845	1	1	
IPI00129159	501	IKITWRTPASDPHGDNITYSVFYTKEGVDRE	1	54.7643	124.18	54.764 _TPASDPHGDN(de)ITYSVFYTK_	TPASDPHGDN(1)ITYSVFYTK	TPASDPHGDN(54.76)ITYSVFYTK	10	3	705	1	1	
IPI00308484	462	TCPPAKIIDQACGTDNQTYASSCHIFATKCR	1	108.446	108.45	108.45 _IIDQACGTDN(de)QTYASSCHIFATK_	IIDQACGTDN(1)QTYASSCHIFATK	IIDQACGTDN(108.45)QTYASSCHIF	10	3	868	1	1	
IPI00115530	306	QKTKTQVFGPVDPTVNTTYAFFNTFFKEISS	1	143.911	195.03	143.91 _TQVFGPVDPTVN(de)TTYAFFN(de)TFFK_	TQVFGPVDPTVN(1)TTYAFFN(1)TFFK	TQVFGPVDPTVN(143.91)TTYAFFN(	12	3	882	1	1	
IPI00120953	1166	STIEYGNRTYKIIRGNMTWYAAGKSCRMHRA	1	79.2014	127.42	79.201 _GN(de)M(ox)TWYAAGK_	GN(1)MTWYAAGK	GN(79.2)MTWYAAGK	2	2	558	1	1	
IPI00124428	273	EQQRSAVSTSWIIPYNHTWSHEKVFVYTPTT	1	58.7119	172.91	58.712 _SAVSTSWIIPYN(de)HTWSHEK_	SAVSTSWIIPYN(1)HTWSHEK	SAVSTSWIIPYN(58.71)HTWSHEK	12	2	1123	1	1	

IPI00316469	620	GYQIAGMANRTTIVDNNTWNNSHIAIVGKAM	0.997687	25.8924	336.04	291.18	_TTIVDN(de)N(de)TWNN(de)SHIAIVGK_	TTIVDN(0.998)N(0.973)TWN(0.10	TTIVDN(25.89)N(15.27)TWN(-10	6	2	1051	1	1
IPI00172039	145	NTIICFVDNIFPPVINITWIRNSKSVTDGVY	0.998718	30.0426	56.172	56.172	_SPVIIGQPNTIICFVDNIFPPVIN(de)ITWIR_	SPVIIGQPNTIICFVDN(0.001)IFPPVI	SPVIIGQPN(-35.32)TIICFVDN(-3	24	4	835	1	1
IPI00123639	344	NFIITNDEAYAEEFGNETWGVTKAAEKQMKD	0.999987	49.0701	185.84	185.84	_SGTIFDNFIITNDEAYAEEFGN(de)ETWGVTK_	SGTIFDNFIITNDEAYAEEFGN(1)ETWG	SGTIFDN(-69.29)FIITN(-49.07)	22	3	1090	1	1
IPI00310797	172	VNMNNIENYVDNKVANITVVVNSIDGKCSKC	1	69.8995	131.32	131.32	_VAN(de)ITVVVNSIDGK_	VAN(1)ITVVVNSIDGK	VAN(69.9)ITVVVN(-69.9)SIDGK	3	3	477	1	1
IPI00321222	97	TQDSIIQAETQANSCNITVVTIQESIEKKVS	0.998403	27.961	182.67	182.67	_TQDSIIQAETQANSCN(de)ITVVTIQESIEKK_	TQDSIIQAETQAN(0.002)SCN(0.998)	TQDSIIQAETQAN(-27.96)SCN(27.	16	3	1084	1	1
IPI00307890	244	IVTAAHCFDNIRYWGNITVVMGEHDFSEKDG	1	60.6023	60.602	60.602	_YWGN(de)ITVVM(ox)GEHDFSEKDGDEQVR_	YWGN(1)ITVVMGEHDFSEKDGDEQVR	YWGN(60.6)ITVVMGEHDFSEKDGDEQ	4	3	943	1	1
IPI00126188	32	AVAIVIGSCRAIEVANGTVTSIPGATVTIIC	1	106.386	106.39	106.39	_AIEVAN(de)GTVTSIPGATVTIICPGK_	AIEVAN(1)GTVTSIPGATVTIICPGK	AIEVAN(106.39)GTVTSIPGATVTII	6	3	791	1	1
IPI00849192	51	PEKSVSVRAGGSATINCTVTSIIPVGPMRWF	1	139.044	139.04	139.04	_AGGSATIN(de)CTVTSIIPVGPM(ox)R_	AGGSATIN(1)CTVTSIIPVGPMR	AGGSATIN(139.04)CTVTSIIPVGPM	8	3	707	1	1
IPI00330714	54	YADDVNSEIVNIYTFNHTVTRNRTEGVRVSV	1	78.002	284.65	284.65	_TYADDVN(de)SEIVNIYTFN(de)HTVTR_	TYADDVN(1)SEIVNIYTFN(1)HTVTR	TYADDVN(55.79)SEIVN(-55.79)I	17	3	859	1	1
IPI00471238	371	VNGEAIYETHTWRSQNDTVTPDVWYTSKPEK	1	119.233	119.23	119.23	_SQN(de)DTVTPDVWYTSKPEKK_	SQN(1)DTVTPDVWYTSKPEKK	SQN(119.23)DTVTPDVWYTSKPEKK	3	3	742	1	1
IPI00331026	111	IARAQITVTVTQTEHNITVSQIPAPQTWRVF	1	60.9906	60.991	60.991	_AQITVTVTQTEHN(de)ITVSQIPAPQTWR_	AQITVTVTQTEHN(1)ITVSQIPAPQTWR	AQITVTVTQTEHN(60.99)ITVSQIPA	13	3	974	1	1
IPI00133500	296	HVWPEDHVFISTPNFNYTVQDFERFFTDIHF	0.76457	5.11555	56.211	56.211	_ITTTSPWM(ox)IPAPHVWPEDHVFISTPNFN(de)YTVQDFER_	ITTTSPWMIPAPHVWPEDHVFISTPN(0.2	ITTTSPWMIPAPHVWPEDHVFISTPN(-	28	5	859	1	1
IPI00331032	71	AEISQRCGWSPAEEINYTVPGIGSAGEASFI	1	125.54	125.54	125.54	_CGWSPAEEIN(de)YTVPGIGSAGEASFISQCM(ox)K_	CGWSPAEEIN(1)YTVPGIGSAGEASFIS(	CGWSPAEEIN(125.54)YTVPGIGSAG	10	3	1088	1	1
IPI00417180	71	VVVIPEVSWRIGKSINCTVKTYSVSHTIEDI	1	65.0378	109.11	65.038	_SIN(de)CTVK_	SIN(1)CTVK	SIN(65.04)CTVK	3	2	412	1	1
IPI00330481	184	TTNVSIIAPRSEHIVNFTVKAEVGGPSSYVY	1	81.7707	81.771	81.771	_SEHIVN(de)FTVK_	SEHIVN(1)FTVK	SEHIVN(81.77)FTVK	6	3	392	1	1
IPI00877260	44	IPGPPEQTSRIWRGPNVTVITGITRGNSRIF	1	62.3783	82.908	62.378	_IWRGPN(de)VTVITGITR_	IWRGPN(1)VTVITGITR	IWRGPN(62.38)VTVITGITR	6	3	562	1	1
IPI00330833	427	HDTDGVAVFVINIPSNVTVIKFEIRTDDPEI	1	53.5846	176.1	53.585	_SITHDTDGVAVFVIN(de)IPSN(de)VTVIK_	SITHDTDGVAVFVIN(1)IPSN(1)VTVIK	SITHDTDGVAVFVIN(53.58)IPSN(5	19	3	848	1	1
IPI00130271	187	EEKEAPRPAIIRSAENFTVIIKNNIHFPGHN	1	76.6551	95.477	76.655	_SAEN(de)FTVIIK_	SAEN(1)FTVIIK	SAEN(76.66)FTVIIK	4	2	562	1	1
IPI00119063	1617	PYYNYIISFTVPDIDNVTVIDYDAREQRVYW	1	45.2691	137.24	45.269	_GVDIDAPYYN(de)YIISFTVPDIDN(de)VTVIDYDAR_	GVDIDAPYYN(1)YIISFTVPDIDN(1)V	GVDIDAPYYN(45.27)YIISFTVPDID	22	3	1180	1	1
IPI00339885	893	QGQQQPGRAAIQFIQNYTVIASSVDSMDFIN	0.999999	61.3423	115.77	115.77	_AAIQFIQN(de)YTVIASSVDSM(ox)DFINDATDVNDAISYVTR_	AAIQFIQN(1)YTVIASSVDSMDFINDATI	AAIQFIQN(61.34)YTVIASSVDSMDF	8	4	1022	1	1
IPI00115536	417	VAGEIFQIERGIYHINITVGGIPFKESEIVQ	1	72.4959	72.496	72.496	_GIYHIN(de)ITVGGIPFK_	GIYHIN(1)ITVGGIPFK	GIYHIN(72.5)ITVGGIPFK	6	3	544	1	1
IPI00623114	661	ITATDGENFATPIYINITVAASRKPVNIQCE	1	85.6403	176.1	176.1	_ITATDGENFATPIYIN(de)ITVAASR_	ITATDGENFATPIYIN(1)ITVAASR	ITATDGEN(-85.64)FATPIYIN(85.	16	3	814	1	1
IPI00124830	73	WKINKSYIFIYDGNKNSTTTDQNFTSAKISV	1	83.2132	183.07	83.213	_SYIFIYDGN(de)KN(de)STTTDQN(de)FTSAK_	SYIFIYDGN(1)KN(1)STTTDQN(1)FTS	SYIFIYDGN(83.21)KN(83.21)STT	11	3	873	1	1
IPI00471089	208	IWYPKFNFSKRNIIPNITTSYIKSCIYNART	1	69.2738	93.345	93.345	_NIIPN(de)ITTSYIK_	NIIPN(1)ITTSYIK	N(-69.27)IIPN(69.27)ITTSYIK	5	2	689	1	1
IPI00314726	547	SDVFEAWRIIITAAPNITTSPAFRYDIIDVT	1	103.851	103.85	103.85	_IIITAAPN(de)ITTSPAFRYDIIDVTR_	IIITAAPN(1)ITTSPAFRYDIIDVTR	IIITAAPN(103.85)ITTSPAFRYDII	8	3	888	1	1
IPI00666034	2773	IQSPIFIIDANANIQNVTTSGNKAEIVASVT	0.969988	16.0769	125.45	125.45	_IQSPIFIIDANANIQN(de)VTTSGNK_	IQSPIFIIDAN(0.006)AN(0.024)IQN	IQSPIFIIDAN(-22.05)AN(-16.08	16	3	820	1	1
IPI00132189	134	TRHEIERSFDCCGIFNITTIRIQDDTSCSAV	1	108.893	179.65	108.89	_SFDCCGIFN(de)ITTIR_	SFDCCGIFN(1)ITTIR	SFDCCGIFN(108.89)ITTIR	9	2	853	1	1
IPI00130573	952	SEFIRGIVMNYPHITNITTIGQSVEYRHIWS	0.999966	44.6586	188.96	188.96	_GIVM(ox)NYPHITN(de)ITTIGQSVEYR_	GIVMNYPHITN(1)ITTIGQSVEYR	GIVMN(-44.66)YPHITN(44.66)IT	11	3	842	1	1
IPI00128905	109	NVNKIHQDEKAVIVNNITTGEKIIRDIQDQI	0.970592	15.1858	120.84	120.84	_AVIVNN(de)ITTGEK_	AVIVN(0.029)N(0.971)ITTGEK	AVIVN(-15.19)N(15.19)ITTGEK	6	2	630	1	1
IPI00122312	77	DNDIRTIEDIIFRAENRTTEAKEIIKAIQVY	1	99.5681	99.568	99.568	_TIEDIIFRAEN(de)R_	TIEDIIFRAEN(1)R	TIEDIIFRAEN(99.57)R	11	3	493	1	1
IPI00125266	258	KDAQWVGFITRSVIENTTSYEEAKNTITKTK	1	162.513	366.58	366.58	_SVIEN(de)TTSYEEAKNTITK_	SVIEN(1)TTSYEEAKNTITK	SVIEN(162.51)TTSYEEAKN(-162.	5	2	1015	1	1
IPI00129215	316	IIFAQIKSHPIHSPVNWTSYASSVESTPTKT	1	90.7289	90.729	90.729	_SHPIHSPVN(de)WTSYASSVESTPTK_	SHPIHSPVN(1)WTSYASSVESTPTK	SHPIHSPVN(90.73)WTSYASSVESTP	9	3	838	1	1
IPI00221547	595	SIQTTSDQQNIKVPGNITSVIISNIVPREQY	0.999994	52.5343	111.84	95.347	_VPGN(de)ITSVIISNIVPR_	VPGN(1)ITSVIISNIVPR	VPGN(52.53)ITSVIISN(-52.53)I	4	2	840	1	1
IPI00118291	578	SQDQRCDIQWKNIGPNTTSTTITSDDFKPGV	0.999899	39.9479	93.226	93.226	_NIGPN(de)TTSTTITSDDFKPGVR_	NIGPN(1)TTSTTITSDDFKPGVR	N(-39.95)IGPN(39.95)TTSTTITS	5	3	741	1	1
IPI00114252	78	YMCKTVVAPSTEGGINITSTFIRKNQCETKI	1	107.977	141.83	107.98	_TVVAPSTEGGIN(de)ITSTFIR_	TVVAPSTEGGIN(1)ITSTFIR	TVVAPSTEGGIN(107.98)ITSTFIR	12	3	655	1	1
IPI00153258	278	DKYRAIKVPMMYREGNFTSTFDKKFRCHIIK	1	61.8154	158.01	61.815	_EGN(de)FTSTFDKK_	EGN(1)FTSTFDKK	EGN(61.82)FTSTFDKK	3	3	426	1	1
IPI00990932	251	VGTTQNIRDIQNFIENVTSSVDVKDNCMRIG	1	80.6384	148.36	80.638	_DIQN(de)FIEN(de)VTSSVDVK_	DIQN(1)FIEN(1)VTSSVDVK	DIQN(80.64)FIEN(80.64)VTSSVD	8	2	905	1	1
IPI00109254	191	GKRSVTTSRTEGMIINVTSSIRVNATANDVF	1	115.064	115.06	115.06	_TEGM(ox)IIN(de)VTSSIR_	TEGMIIN(1)VTSSIR	TEGMIIN(115.06)VTSSIR	7	2	719	1	1
IPI00515360	3773	SITQGSIIVGNIAPVNGTSQGKFQGIDINEE	1	83.9035	289.35	126.25	_SITQGSIIVGNIAPVN(de)GTSQGK_	SITQGSIIVGNIAPVN(1)GTSQGK	SITQGSIIVGN(-83.9)IAPVN(83.9	16	3	715	1	1
IPI00121430	1765	TTQAPKSGPRNIQVYNATSNSITVKWDPASG	1	81.2866	140.96	140.96	_NIQVYN(de)ATSN(de)SITVK_	NIQVYN(1)ATSN(1)SITVK	N(-81.29)IQVYN(81.29)ATSN(11	6	2	827	1	1
IPI00469426	684	SYAYSVIIIKTGDGSNVTSNFTKDPSIIIPE	1	87.4889	87.489	87.489	_TGDGSN(de)VTSN(de)FTKDPSIIIPEIIPGVSYTVK_	TGDGSN(1)VTSN(1)FTKDPSIIIPEII	TGDGSN(87.49)VTSN(87.49)FTKD	6	3	1085	1	1
IPI00894972	86	RTSSEFCQPVSIHGSNHTSKYIGMTIATDAA	1	69.1882	119.08	69.188	_TSSEFCQPVSIHGSN(de)HTSK_	TSSEFCQPVSIHGSN(1)HTSK	TSSEFCQPVSIHGSN(69.19)HTSK	15	3	702	1	1
IPI00130010	819	YERSICINGKWDPEPNCTSKTSCPPPPQIPN	1	62.4075	106.93	62.408	_WDPEPN(de)CTSK_	WDPEPN(1)CTSK	WDPEPN(62.41)CTSK	6	2	618	1	1
IPI00221426	354	DIKVPIIVRGPGIKPNQTSKMIVSNIDIGPT	1	143.238	179.19	143.24	_GPGIKPN(de)QTSK_	GPGIKPN(1)QTSK	GPGIKPN(143.24)QTSK	7	2	564	1	1
IPI00230426	93	AHCAGRSITVIIGAHNKTSKEDTWQKIEVEK	1	161.622	161.62	161.62	_SITVIIGAHN(de)K_	SITVIIGAHN(1)K	SITVIIGAHN(161.62)K	10	2	577	1	1
IPI00380460	110	PPYGIFIINQKTGEINITSIVDREVTPFFII	1	106.578	106.58	106.58	_TGEIN(de)ITSIVDR_	TGEIN(1)ITSIVDR	TGEIN(106.58)ITSIVDR	5	2	660	1	1
IPI00985989	373	PGYPSHDGSFRKSRTNITSIIVQPISPSIVK	1	114.275	114.27	114.27	_TN(de)ITSIIVQPISPSIVK_	TN(1)ITSIIVQPISPSIVK	TN(114.27)ITSIIVQPISPSIVK	2	3	604	1	1
IPI00282289	140	RAIAHSEIDVRTAGANGTSGFFCVDEGGIPI	1	117.745	166.29	117.74	_TAGAN(de)GTSGFFCVDEGGIPIAQR_	TAGAN(1)GTSGFFCVDEGGIPIAQR	TAGAN(117.74)GTSGFFCVDEGGIPI	5	4	582	1	1
IPI00323053	427	ICYKNNSEKCKSIPNNVTSFEVESIKPYKYY	1	144.678	211.06	144.68	_SIPN(de)N(de)VTSFEVESIKPYK_	SIPN(1)N(1)VTSFEVESIKPYK	SIPN(144.68)N(144.68)VTSFEVE	5	2	1028	1	1
IPI00124830	80	IFIYDGNKNSTTTDQNFTSAKISVSDIINGI	1	83.2132	183.07	83.213	_SYIFIYDGN(de)KN(de)STTTDQN(de)FTSAK_	SYIFIYDGN(1)KN(1)STTTDQN(1)FTS	SYIFIYDGN(83.21)KN(83.21)STT	18	3	873	1	1
IPI00400016	1203	HKQEADDIVRVAKTANETSAEAYNIIIRTIA	1	160.152	266.45	266.45	_TAN(de)ETSAEAYNIIIR_	TAN(1)ETSAEAYNIIIR	TAN(160.15)ETSAEAYN(-160.15)	3	2	834	1	1
IPI00473190	176	PSKAANFTIKQINAYNRTRYEIQEFIQDTCV	0.999334	31.7603	114.02	114.02	_QINAYN(de)R_	QIN(0.001)AYN(0.999)R	QIN(-31.76)AYN(31.76)R	6	2	440	1	1
IPI00855103	274	DIKENCMRVGIVTYSNETRVISSISTGNNKT	1	56.2251	129.84	56.225	_VGIVTYSN(de)ETR_	VGIVTYSN(1)ETR	VGIVTYSN(56.23)ETR	8	2	620	1	1
IPI00323235	494	VDRTAGWNIPMGIIANQTRSCKFNEFFSQSC	1	103.744	197.75	197.75	_TAGWNIPM(ox)GIIAN(de)QTR_	TAGWNIPMGIIAN(1)QTR	TAGWN(-103.74)IPMGIIAN(103.7	13	2	880	1	1
IPI00121254	202	EVQGIPYFIKQPESVNVTRNTAFNITCQAVG	1	53.4444	91.729	53.444	_EIVSDPIYVEVQGIPYFIKQPESVN(de)VTR_	EIVSDPIYVEVQGIPYFIKQPESVN(1)V	EIVSDPIYVEVQGIPYFIKQPESVN(53	25	4	806	1	1
IPI00856861	273	PYYGRTGVNSGVMIMNMTRMRRKYFKNDMTT	1	78.5055	110.98	78.505	_TGVN(de)SGVM(ox)IM(ox)N(de)M(ox)TR_	TGVN(1)SGVMIMN(1)MTR	TGVN(78.51)SGVMIMN(78.51)MTR	11	2	781	1	1
IPI00115976	185	DPQNDPVGTCYISTENFTRIIEYAPCRSDFG	1	76.0654	219.87	171.47	_TEKDPQNDPVGTCYISTEN(de)FTR_	TEKDPQNDPVGTCYISTEN(1)FTR	TEKDPQN(-76.07)DPVGTCYISTEN(	19	3	858	1	1
IPI00114671	444	NDMRSDFSQTMIFQANTTRIFQGTRIVKTGV	1	121.239	181.51	121.24	_SDFSQTM(ox)IFQAN(de)TTR_	SDFSQTMIFQAN(1)TTR	SDFSQTMIFQAN(121.24)TTR	12	3	589	1	1
IPI00136513	143	EVRQTRFMFFVQFPHNATQTMNIRVIVIRPY	0.989434	19.7148	76.151	73.279	_FM(ox)FFVQFPHN(de)ATQTM(ox)NIR_	FMFFVQFPHN(0.989)ATQTMN(0.011)	FMFFVQFPHN(19.71)ATQTMN(-19.	10	3	755	1	1
IPI00330632	373	TEIITSEVTARSFMVNWTQSPGKVEKYRVVY	1	91.207	162.81	91.207	_SFM(ox)VN(de)WTQSPGKVEKYR_	SFMVN(1)WTQSPGKVEKYR	SFMVN(91.21)WTQSPGKVEKYR	5	3	692	1	1
IPI00623114	1942	EKFSMDHKTGTIAIQNTTQIRSRYEITVRAS	1	143.939	143.94	143.94	_TGTIAIQN(de)TTQIR_	TGTIAIQN(1)TTQIR	TGTIAIQN(143.94)TTQIR	8	2	709	1	1
IPI00990932	368	RIEGVTMFAMGIEGANNTQIEDIVSYPSRQS	0.78097	5.52132	43.895	43.895	_IEGVTM(ox)FAM(ox)GIEGAN(de)NTQIEDIVSYPSR_	IEGVTMFAMGIEGAN(0.781)N(0.219)	IEGVTMFAMGIEGAN(5.52)N(-5.52	15	3	1026	1	1
IPI00134743	140	EGTVGVSAATERTRCNTTQGNEVTSIIRWAK	0.999998	57.0578	136.39	136.39	_CN(de)TTQGNEVTSIIR_	CN(1)TTQGNEVTSIIR	CN(57.06)TTQGN(-57.06)EVTSII	2	2	797	1	1
IPI00830749	389	PGDEGGPGERGPPGVNGTQGFQGCPGQRGVK	1	162.461	162.46	162.46	_GPPGVN(de)GTQGFQGCPGQR_	GPPGVN(1)GTQGFQGCPGQR	GPPGVN(162.46)GTQGFQGCPGQR	6	3	606	1	1
IPI00309999	1610	TGPIPAPYKIIYGIENTTQEIKHIISPQRAP	1	122.966	122.97	122.97	_IIYGIEN(de)TTQEIK_	IIYGIEN(1)TTQEIK	IIYGIEN(122.97)TTQEIK	7	2	762	1	1
IPI00120166	294	NPCFPDPCKSSVEIINSTQAPMRDAGPCWVI	1	59.2475	133.48	59.248	_SSVEIIN(de)STQAPM(ox)R_	SSVEIIN(1)STQAPMR	SSVEIIN(59.25)STQAPMR	7	3	517	1	1
IPI00229935	398	NIVSYNEIWMKSFTVNWTPPAGDWEHYRIVI	1	102.776	136.26	102.78	_SFTVN(de)WTPPAGDWEHYR_	SFTVN(1)WTPPAGDWEHYR	SFTVN(102.78)WTPPAGDWEHYR	5	2	1032	1	1
IPI00127856	69	PDYRQEIQKTQMVFFNITPNIINDTMEIREY	0.999999	61.5145	192.25	192.25	_TQM(ox)VFFN(de)ITPNIIN(de)DTM(ox)EIR_	TQMVFFN(1)ITPN(0.208)IIN(0.79	TQMVFFN(61.51)ITPN(-5.8)IIN(	7	2	1216	1	1
IPI00221444	117	VPVEDREDPYSCRIINITNPVINQEIEAFSF	0.991275	20.5762	171.75	171.75	_IIN(de)ITNPVINQEIEAFSFPEDASSSR_	IIN(0.991)ITN(0.009)PVINQEIEAI	IIN(20.58)ITN(-20.58)PVIN(-4	3	3	965	1	1
IPI00270376	106	VHFTSNDVKSGDASINVTNIQISDIGTYQCK	1	126.913	276.26	126.91	_SGDASIN(de)VTN(de)IQISDIGTYQCK_	SGDASIN(1)VTN(1)IQISDIGTYQCK	SGDASIN(126.91)VTN(126.91)IQ	7	3	796	1	1
IPI00408495	306	ISNIDVNVDPGTYVCNATNAQGTTRETISIR	1	74.8658	185.7	185.7	_SQITISNIDVNVDPGTYVCN(de)ATN(de)AQGTTR_	SQITISNIDVNVDPGTYVCN(1)ATN(1)A	SQITISN(-88.91)IDVN(-74.87)V	20	3	1038	1	1
IPI00406603	841	RSSSYHENDMENPQSNITMVYIIPIGPKGSG	0.99991	40.2021	130.3	130.3	_SSSYHEN(de)DM(ox)ENPQSN(de)ITM(ox)VYIIPIGPK_	SSSYHEN(0.94)DMEN(0.06)PQSN(1)	SSSYHEN(11.94)DMEN(-11.94)PQ	15	3	1033	1	1
IPI00406603	238	RSQSPSIMYTINGFVNKTMPDITVCAHDHVS	1	134.807	134.81	134.81	_SQSPSIM(ox)YTIN(de)GFVN(de)K_	SQSPSIMYTIN(1)GFVN(1)K	SQSPSIMYTIN(134.81)GFVN(134.	15	2	902	1	1
IPI00123925	92	VFYNIETRESYIIISNSTMKSVNATDYGISP	1	102.97	102.97	102.97	_ESYIIISN(de)STM(ox)K_	ESYIIISN(1)STMK	ESYIIISN(102.97)STMK	8	2	702	1	1
IPI00411145	1077	VSIDIPYFADVVVPINMTMKNTIAIGVDPIE	1	91.7247	91.725	91.725	_M(ox)VSIDIPYFADVVVPIN(de)M(ox)TM(ox)K_	MVSIDIPYFADVVVPIN(1)MTMK	MVSIDIPYFADVVVPIN(91.72)MTMK	17	2	1217	1	1
IPI00153258	299	DKKFRCHIIKIPYQGNATMIVVIMEKTGDYI	1	112.5	112.5	112.5	_IPYQGN(de)ATM(ox)IVVIM(ox)EK_	IPYQGN(1)ATMIVVIMEK	IPYQGN(112.5)ATMIVVIMEK	6	3	614	1	1
IPI00308971	107	GDSIIRSSARSIIEFNTTMGCQPSDSQHRIQ	1	79.5108	177.44	79.511	_SIIEFN(de)TTM(ox)GCQPSDSQHR_	SIIEFN(1)TTMGCQPSDSQHR	SIIEFN(79.51)TTMGCQPSDSQHR	6	2	1113	1	1
IPI00127856	76	QKTQMVFFNITPNIINDTMEIREYHTIDDHC	0.957517	13.5823	192.25	61.87	_TQMVFFNITPNIIN(de)DTM(ox)EIR_	TQMVFFN(0.001)ITPN(0.042)IIN((	TQMVFFN(-32.78)ITPN(-13.58)I	14	3	805	1	1
IPI00119522	74	TAFTTVRTRAFSGSPNITKVVFINTQVRHIE	1	79.2014	101.95	79.201	_AFSGSPN(de)ITK_	AFSGSPN(1)ITK	AFSGSPN(79.2)ITK	7	2	512	1	1
IPI00129646	120	GRIKVSEDGYSIYMSNITKSDSGSYHAQINQ	1	103.387	103.39	103.39	_VSEDGYSIYM(ox)SN(de)ITK_	VSEDGYSIYMSN(1)ITK	VSEDGYSIYMSN(103.39)ITK	12	2	862	1	1
IPI00125522	157	FSQIVWKSAEGTFFINKTKIKDFPRFPHRGV	1	103.751	157.56	103.75	_SAEGTFFIN(de)KTK_	SAEGTFFIN(1)KTK	SAEGTFFIN(103.75)KTK	9	2	672	1	1
IPI00475154	106	AQSSQVISGCEISVSNETKEIIIAAVSEDSP	0.999999	59.6466	114.8	114.8	_SNIDPSNVDSIFYAAQSSQVISGCEISVSN(de)ETK_	SNIDPSNVDSIFYAAQSSQVISGCEISVS	SN(-65.32)IDPSN(-59.65)VDSIF	30	4	888	1	1
IPI00469426	688	SVIIIKTGDGSNVTSNFTKDPSIIIPEIIPG	1	87.4889	87.489	87.489	_TGDGSN(de)VTSN(de)FTKDPSIIIPEIIPGVSYTVK_	TGDGSN(1)VTSN(1)FTKDPSIIIPEII	TGDGSN(87.49)VTSN(87.49)FTKD	10	3	1085	1	1
IPI00323053	322	CNSDNISYVIHCEPENNTKCIRRNTFIPERC	0.95874	13.7051	93.381	93.381	_KCNSDN(de)ISYVIHCEPEN(de)NTK_	KCN(0.202)SDN(0.798)ISYVIHCEPI	KCN(-5.97)SDN(5.97)ISYVIHCEP	17	3	809	1	1
IPI00894972	927	IPVIYPVNIITKEQENSTIYISFTPKGPKTQ	1	90.5608	90.561	90.561	_EQEN(de)STIYISFTPK_	EQEN(1)STIYISFTPK	EQEN(90.56)STIYISFTPK	4	2	829	1	1
IPI00121190	603	GPHCVKTCPAGIMGENNTIVWKYADANNVCH	1	188.437	254.1	188.44	_TCPAGIM(ox)GEN(de)N(de)TIVWK_	TCPAGIMGEN(1)N(1)TIVWK	TCPAGIMGEN(188.44)N(188.44)T	10	2	905	1	1
IPI00762812	809	IRMSQHPIDIQEDFNNATIVNASSIIPKEAG	1	82.5933	82.593	82.593	_M(ox)SQHPIDIQEDFN(de)N(de)ATIVN(de)ASSIIPK_	MSQHPIDIQEDFN(1)N(1)ATIVN(1)A	MSQHPIDIQEDFN(82.59)N(82.59)	14	3	968	1	1
IPI00453829	42	AIPAEESRVQPMTASNWTIVMEGEWMIKFYA	1	65.1047	127.97	65.105	_VQPM(ox)TASN(de)WTIVM(ox)EGEWM(ox)IK_	VQPMTASN(1)WTIVMEGEWMIK	VQPMTASN(65.1)WTIVMEGEWMIK	8	3	801	1	1
IPI00118830	66	NSTIVITFEVTFRSKNITIVEIPDEVIVPRG	1	61.7318	182.34	61.732	_SKN(de)ITIVEIPDEVIVPR_	SKN(1)ITIVEIPDEVIVPR	SKN(61.73)ITIVEIPDEVIVPR	3	3	642	1	1
IPI00129253	529	CYKIYEKEAPFGTNCNITITSRFEQEFINYM	0.998773	29.1055	101.42	101.42	_EAPFGTNCN(de)ITITSR_	EAPFGTN(0.001)CN(0.999)ITITSR	EAPFGTN(-29.11)CN(29.11)ITIT	9	2	841	1	1
IPI00876028	323	TIVIPIRYYVRWRIRNATITQAITNKDSPFI	1	69.2432	90.793	90.793	_IRN(de)ATITQAITNK_	IRN(1)ATITQAITNK	IRN(69.24)ATITQAITN(-69.24)K	3	3	482	1	1
IPI00126834	561	WSRQINNGEIQPISENTTITFMSTKRDDSGI	1	125.032	150.69	125.03	_QIN(de)N(de)GEIQPISEN(de)TTITFM(ox)STK_	QIN(1)N(1)GEIQPISEN(1)TTITFMS	QIN(125.03)N(125.03)GEIQPISE	13	3	829	1	1

IPI00121378	265	TIQVIPPKNAIKEGDNITIQCIGNGNPPPEE	0.999799	36.3174	88.708	88.708	_EGDN(de)ITIQCIGNGN(de)PPPEEFM(ox)FYIPGQPEGIR_	EGDN(1)ITIQCIGN(0.138)GN(0.86	EGDN(36.32)ITIQCIGN(-7.98)GN	4	4	903	1	1
IPI00348266	316	EQRRNVIRTIPHHQYNATINKYSHDIAIIEI	1	56.5691	145.81	56.569	_TIPHHQYN(de)ATIN(de)K_	TIPHHQYN(1)ATIN(1)K	TIPHHQYN(56.57)ATIN(56.57)K	8	2	770	1	1
IPI00285117	330	FGGNFARQRYEDIVINETINKNRIIGQKTGI	1	64.7495	120.87	120.87	_QRYEDIVIN(de)ETINK_	QRYEDIVIN(1)ETINK	QRYEDIVIN(64.75)ETIN(-64.75)	9	3	579	1	1
IPI00126827	172	APPSCSIQGVPYVGTNVTINCKSPRSKPTAQ	0.986528	18.6467	109.85	88.627	_VIVPPAPPSCSIQGVPYVGTN(de)VTINCK_	VIVPPAPPSCSIQGVPYVGTN(0.987)V	VIVPPAPPSCSIQGVPYVGTN(18.65)	21	3	957	1	1
IPI00338018	601	IIKTTQGSKIYIKGVNETIIVNEIKSKESDI	1	68.7543	128.95	128.95	_GVN(de)ETIIVNEIK_	GVN(1)ETIIVNEIK	GVN(68.75)ETIIVN(-68.75)EIK	3	2	665	1	1
IPI00468236	803	DFKNTDKENPYIGIGNYTICRYRDFRNPPGH	0.999999	60.643	113.01	113.01	_NTDKENPYIGIGN(de)YTICR_	NTDKENPYIGIGN(1)YTICR	N(-62.19)TDKEN(-60.64)PYIGIG	13	3	710	1	1
IPI00119063	3663	CGTGVRTCPIDEFQCNNTICKPIAWKCDGED	1	70.9421	172.93	70.942	_TCPIDEFQ(de)CN(de)N(de)TICKPIAWK_	TCPIDEFQCN(1)N(1)TICKPIAWK	TCPIDEFQCN(70.94)N(70.94)TIC	10	3	833	1	1
IPI00331175	312	KTAFAPPDIPVCIIGNRTIANRNFDTCAKMQ	1	143.249	143.25	143.25	_TAFAPPDIPVCIIGN(de)R_	TAFAPPDIPVCIIGN(1)R	TAFAPPDIPVCIIGN(143.25)R	15	3	581	1	1
IPI00123223	1426	EVSNNNVIIYIDQVTNQTIAFSFIIQQDIPV	0.999958	48.4934	173.59	103.12	_TEVSNNNVIIYIDQVTN(de)QTIAFSFIIQQDIPVR_	TEVSNNNVIIYIDQVTN(1)QTIAFSFII	TEVSN(-48.49)N(-48.49)N(-48.	17	4	949	1	1
IPI00626555	241	HVHVHDIQSFGIDNINMTHYIKHISFGEDYP	0.959757	13.7747	192.57	139.7	_SFQQSHVHVHDIQSFGIDNIN(de)M(ox)THYIK_	SFQQSHVHVHDIQSFGIDN(0.04)IN(0.	SFQQSHVHVHDIQSFGIDN(-13.77)I	21	4	804	1	1
IPI00222967	265	EEKVPFIFNINPATTNFTGSCQPQSAQIRIN	0.997677	26.5955	94.389	92.034	_VPFIFNINPATTN(de)FTGSCQPQSAQIR_	VPFIFNIN(0.002)PATTN(0.998)FT	VPFIFN(-38.59)IN(-26.6)PATTN	13	4	728	1	1
IPI00405742	1005	AFEPIRSFVSGGRSINVTGQGFSIIQKFAMV	1	55.196	233.02	55.196	_SIN(de)VTGQGFSIIQK_	SIN(1)VTGQGFSIIQK	SIN(55.2)VTGQGFSIIQK	3	3	498	1	1
IPI00113797	331	PTIPPVSFHIGGVWFNITGQDYVIKIIQSDV	1	65.2071	65.207	65.207	_TPTIPPVSFHIGGVWFN(de)ITGQDYVIK_	TPTIPPVSFHIGGVWFN(1)ITGQDYVIK	TPTIPPVSFHIGGVWFN(65.21)ITGQ	17	3	963	1	1
IPI00118674	572	SSPTNTTYVVQYAIANITGKATNITREQCQD	1	125.587	125.59	125.59	_SYIDDRPIQHYIAVSSPTN(de)TTYVVQYAIAN(de)ITGK_	SYIDDRPIQHYIAVSSPTN(1)TTYVVQY	SYIDDRPIQHYIAVSSPTN(125.59)T	30	3	1268	1	1
IPI00400202	168	PDIEPVIKHAIEYFNNNTGHSHIFAIREVKS	0.85176	7.60631	89.866	89.866	_HAIEYFNN(de)NTGHSHIFAIR_	HAIEYFN(0.148)N(0.852)NTGHSHI	HAIEYFN(-7.61)N(7.61)N(-32.9	8	3	748	1	1
IPI00132474	406	DGVTINYKSYCKNGVNGTGENGRKCSNISIG	1	76.8199	265.12	76.82	_SYCKN(de)GVN(de)GTGEN(de)GRK_	SYCKN(1)GVN(1)GTGEN(1)GRK	SYCKN(76.82)GVN(76.82)GTGEN(	8	2	872	1	1
IPI00322575	917	PGQQPQPPITNIIIVNKTGAKIDDFIHSIEQ	0.96407	14.2865	61.499	38.691	_QSDYCWEIAPHM(ox)YFITPGQQPQPPITNIIIVN(de)K_	QSDYCWEIAPHMYFITPGQQPQPPITN(0.	QSDYCWEIAPHMYFITPGQQPQPPITN(	32	5	784	1	1
IPI00468055	281	GEPIETKIKIMESHPNGTFSAKGVASVCVED	1	65.8954	97.463	65.895	_IM(ox)ESHPN(de)GTFSAK_	IMESHPN(1)GTFSAK	IMESHPN(65.9)GTFSAK	7	2	718	1	1
IPI00123652	198	IIDYYGTRITRISITNETFRKTQIYP_____	1	131.819	131.82	131.82	_ISITN(de)ETFRK_	ISITN(1)ETFRK	ISITN(131.82)ETFRK	5	2	605	1	1
IPI00132474	50	CGECIQAGPNCGWCTNTTFIQEGMPTSARCD	0.99798	26.9388	104.38	104.38	_SCGECIQAGPNCGWCTN(de)TTFIQEGM(ox)PTSAR_	SCGECIQAGPN(0.002)CGWCTN(0.99	SCGECIQAGPN(-26.94)CGWCTN(26	17	4	853	1	1
IPI00136938	318	KMTIPTVDVIIGRSINGTFFGGWKSVDSVPN	1	101.929	101.93	101.93	_SIN(de)GTFFGGWK_	SIN(1)GTFFGGWK	SIN(101.93)GTFFGGWK	3	2	608	1	1
IPI00117957	275	IQRIGIGNNRITDIENGTFANIPRVREIHIE	1	85.1641	136.3	136.3	_ITDIEN(de)GTFANIPR_	ITDIEN(1)GTFANIPR	ITDIEN(85.16)GTFAN(-85.16)IP	6	3	521	1	1
IPI00468055	46	RDFIPSTISFTWNYQNNTEVIQGIRTFPTIR	0.995533	23.4759	84.946	69.361	_DFIPSTISFTWNYQN(de)N(de)TEVIQGIR_	DFIPSTISFTWN(0.006)YQN(0.996)	DFIPSTISFTWN(-23.48)YQN(23.4	15	3	949	1	1
IPI00468674	606	SIDAYPVINQAQAMENHTEVHFQKECGPDNK	0.999993	51.6499	122.49	122.49	_SIDAYPVINQAQAM(ox)EN(de)HTEVHFQK_	SIDAYPVINQAQAMEN(1)HTEVHFQK	SIDAYPVIN(-51.65)QAQAMEN(51.	16	4	698	1	1
IPI00170146	911	PGQIPQGIRTSIIVINNTESNIEDFIQSIKH	1	93.4997	276.68	93.5	_TSIIVIN(de)N(de)TESN(de)IEDFIQSIK_	TSIIVIN(1)N(1)TESN(1)IEDFIQSI	TSIIVIN(93.5)N(93.5)TESN(93.	7	3	794	1	1
IPI00278602	459	EPIIYMPPPPCQPIINTTESIRINHEIRGWV	1	84.6803	84.68	84.68	_AIM(ox)VISEEPIIYM(ox)PPPPCQPIIN(de)TTESIR_	AIMVISEEPIIYMPPPPCQPIIN(1)TTE	AIMVISEEPIIYMPPPPCQPIIN(84.6	23	4	836	1	1
IPI00118674	611	KDIYEYSWVQGPWNSNRTERIPQCVRSTVRI	0.977265	16.3331	121.72	121.72	_DIYEYSWVQGPWNSN(de)R_	DIYEYSWVQGPWN(0.023)SN(0.977)	DIYEYSWVQGPWN(-16.33)SN(16.3	15	2	1008	1	1
IPI00129220	436	GIVTSRSFRTASVSINQTEPPKVRIEDRSTT	1	174.592	174.59	174.59	_TASVSIN(de)QTEPPKVR_	TASVSIN(1)QTEPPKVR	TASVSIN(174.59)QTEPPKVR	7	2	814	1	1
IPI00113057	494	EGNYDIAIIKIQTPINYTEFQKPICIPSKAD	1	80.6384	137.61	80.638	_IQTPIN(de)YTEFQKPICIPSK_	IQTPIN(1)YTEFQKPICIPSK	IQTPIN(80.64)YTEFQKPICIPSK	6	3	760	1	1
IPI00311405	332	EATNPIGTRSGQVEVNITEFPYTPTPEHGRR	1	69.7632	69.763	69.763	_SGQVEVN(de)ITEFPYTPTPEHGR_	SGQVEVN(1)ITEFPYTPTPEHGR	SGQVEVN(69.76)ITEFPYTPTPEHGR	7	3	787	1	1
IPI00890869	106	APAEKKIECTQKGKSNQTECFNFIRFIQPYN	0.998158	27.3384	127.71	127.71	_GKSN(de)QTECFNFIR_	GKSN(0.998)QTECFN(0.002)FIR	GKSN(27.34)QTECFN(-27.34)FIR	4	2	801	1	1
IPI00129243	100	IFRSINGVIIPGGGANITDSGYSRVAKIFFS	1	99.3661	128.98	99.366	_SIN(de)GVIIPGGGAN(de)ITDSGYSR_	SIN(1)GVIIPGGGAN(1)ITDSGYSR	SIN(99.37)GVIIPGGGAN(99.37)I	13	2	1026	1	1
IPI00396840	382	VVYDKEYIRQVSTIINNTDKCIINNYMMWNI	1	67.4565	123.95	67.456	_QVSTIIN(de)N(de)TDK_	QVSTIIN(1)N(1)TDK	QVSTIIN(67.46)N(67.46)TDK	7	2	618	1	1
IPI00123746	671	NIPIMVTDSGKPPMTNITDIRVQVCSCKNSK	0.999825	37.6457	78.456	78.456	_ANYNIPIM(ox)VTDSGKPPM(ox)TN(de)ITDIR_	ANYNIPIMVTDSGKPPMTN(1)ITDIR	AN(-55.32)YN(-37.65)IPIMVTDS	19	3	899	1	1
IPI00127770	103	KTYCANEPISNCSQVNRTDICSVIPPTTPVP	1	92.6845	165.58	165.58	_TYCANEPISN(de)CSQVN(de)R_	TYCANEPISN(1)CSQVN(1)R	TYCAN(-42.56)EPISN(42.56)CSQ	15	2	958	1	1
IPI00131693	60	PCGTASWDAAVTWRVNGTDIAPDIINGSQII	1	137.731	137.73	137.73	_VN(de)GTDIAPDIIN(de)GSQIIIR_	VN(1)GTDIAPDIIN(1)GSQIIIR	VN(137.73)GTDIAPDIIN(137.73)	2	2	1006	1	1
IPI00119063	4365	QGACVVNKQTGDVTCNCTDGRVAPSCITCID	1	76.262	76.262	76.262	_QTGDVTCN(de)CTDGR_	QTGDVTCN(1)CTDGR	QTGDVTCN(76.26)CTDGR	8	2	743	1	1
IPI00138199	1523	SEDQCYTCPRETFIINTTCVKECPEGYHTDK	1	129.343	129.34	129.34	_ETFIIN(de)TTCVK_	ETFIIN(1)TTCVK	ETFIIN(129.34)TTCVK	6	2	664	1	1
IPI01026704	882	SGIEEIQKDSCESNQNITCRVGYPFIRTGDV	0.999491	32.9331	189.3	189.3	_DSCESNQN(de)ITCR_	DSCESN(0.001)QN(0.999)ITCR	DSCESN(-32.93)QN(32.93)ITCR	8	2	743	1	1
IPI00608044	277	FQKWASVVVPIGKEQNYTCHVYHEGIPEPIT	1	93.4953	221.47	93.495	_EQN(de)YTCHVYHEGIPEPITIR_	EQN(1)YTCHVYHEGIPEPITIR	EQN(93.5)YTCHVYHEGIPEPITIR	3	3	820	1	1
IPI00312980	277	FQKWASVVVPIGKEQNYTCHVNHEGIPEPIT	0.987172	18.8624	126.84	126.84	_EQN(de)YTCHVNHEGIPEPITIR_	EQN(0.987)YTCHVN(0.013)HEGIPE	EQN(18.86)YTCHVN(-18.86)HEGI	3	3	803	1	1
IPI00323053	489	KVNGMKTSRPTDNSINVTCGPPYETNGPKTF	0.830043	6.88762	126.26	126.26	_TSRPTDNSIN(de)VTCGPPYETN(de)GPK_	TSRPTDN(0.17)SIN(0.83)VTCGPPY	TSRPTDN(-6.89)SIN(6.89)VTCGP	10	3	836	1	1
IPI00122494	193	PVATEQIISTFITVGNNTCFYGKCYYCRETE	0.950566	12.8395	121.39	121.39	_TEVKPVATEQIISTFITVGN(de)NTCFYGK_	TEVKPVATEQIISTFITVGN(0.951)N(	TEVKPVATEQIISTFITVGN(12.84)N	20	3	1007	1	1
IPI00624663	881	KTVSWAVTPKSIGEVNFTATAEAIQSPEICG	1	95.823	241.44	95.823	_SIGEVN(de)FTATAEAIQSPEICGN(de)KITEVPAIVHK_	SIGEVN(1)FTATAEAIQSPEICGN(1)K	SIGEVN(95.82)FTATAEAIQSPEICG	6	4	882	1	1
IPI00308971	2078	ETVIVTYSKGYPCGGNKTASSVIEITCAKTV	1	87.1601	87.16	87.16	_GYPCGGN(de)KTASSVIEITCAK_	GYPCGGN(1)KTASSVIEITCAK	GYPCGGN(87.16)KTASSVIEITCAK	7	3	705	1	1
IPI00119063	3334	PTNFYIGGDGRTCVSNCTASQFVCKNDKCIP	1	63.8937	216.27	63.894	_TCVSN(de)CTASQFVCKN(de)DK_	TCVSN(1)CTASQFVCKN(1)DK	TCVSN(63.89)CTASQFVCKN(63.89	5	3	674	1	1
IPI00408895	110	IGDSRRQPEKTDAEINETARPISPVNPKIEK	0.999998	57.0046	111.77	111.77	_TDAEIN(de)ETARPISPVNPK_	TDAEIN(1)ETARPISPVNPK	TDAEIN(57)ETARPISPVN(-57)PK	6	2	977	1	1
IPI00123342	222	TATAISYGVFRRKDINSTAQNVMFYDMGSGS	0.998752	29.0339	78.128	78.128	_DIN(de)STAQNVM(ox)FYDM(ox)GSGSTVCTIVTYQTVK_	DIN(0.999)STAQN(0.001)VMFYDMG	DIN(29.03)STAQN(-29.03)VMFYD	3	3	1122	1	1
IPI00400073	44	CRRIQEGHEYDTFDINDTAQCFTKCGQSEHS	1	141.072	141.07	141.07	_RIQEGHEYDTFDIN(de)DTAQCFTK_	RIQEGHEYDTFDIN(1)DTAQCFTK	RIQEGHEYDTFDIN(141.07)DTAQCF	14	3	897	1	1
IPI00626555	266	FGEDYPGIVNPIDHTNVTAPQASMMFQYFVK	0.999305	31.5762	110.55	110.55	_HISFGEDYPGIVNPIDHTN(de)VTAPQASM(ox)M(ox)FQYFVK_	HISFGEDYPGIVN(0.001)PIDHTN(0.	HISFGEDYPGIVN(-31.58)PIDHTN(	19	4	972	1	1
IPI00400016	1221	SAEAYNIIIRTIAGENQTAIEIEEINRKYEQ	1	111.448	239.58	214.46	_TIAGEN(de)QTAIEIEEINR_	TIAGEN(1)QTAIEIEEINR	TIAGEN(111.45)QTAIEIEEIN(-11	6	2	951	1	1
IPI00129903	681	SIKTNIEGQVPFIPANFTAHITPYKEFYHYR	1	114.057	184.6	184.6	_TNIEGQVPFIPAN(de)FTAHITPYK_	TNIEGQVPFIPAN(1)FTAHITPYK	TN(-114.06)IEGQVPFIPAN(114.0	13	3	820	1	1
IPI00118333	288	TKGVMRASFSSREADNHTAFIRIKTNASDST	1	103.083	103.08	103.08	_EADN(de)HTAFIR_	EADN(1)HTAFIR	EADN(103.08)HTAFIR	4	2	588	1	1
IPI00226563	144	VAGVQDRVWDTAAAINRTAEPNIQSIERQIA	1	90.827	90.827	90.827	_VWDTAAAIN(de)R_	VWDTAAAIN(1)R	VWDTAAAIN(90.83)R	9	2	559	1	1
IPI00130117	515	RFHGKNVPGEIGINYNITADVAQKEKGQIPR	0.988969	19.5439	142.94	142.94	_NVPGEIGINYN(de)ITADVAQK_	NVPGEIGIN(0.011)YN(0.989)ITAD	N(-43.28)VPGEIGIN(-19.54)YN(	11	3	673	1	1
IPI00153389	149	RTEQYKKEIKSWVQGNITACGRSIFIFDEMD	1	80.3118	80.312	80.312	_SWVQGN(de)ITACGR_	SWVQGN(1)ITACGR	SWVQGN(80.31)ITACGR	6	2	675	1	1
IPI00330714	165	RVDNFVIRTGEIFTFNTTAAQPQYFKYEFPD	1	52.8241	182.49	52.824	_TGEIFTFN(de)TTAAQPQYFKYEFPDGVDSVIVK_	TGEIFTFN(1)TTAAQPQYFKYEFPDGVD	TGEIFTFN(52.82)TTAAQPQYFKYEF	8	3	1172	1	1
IPI00323053	311	EWKTKNIDFRKCNSDNISYVIHCEPENNTKC	0.947235	12.5679	122.44	122.44	_KCNSDN(de)ISYVIHCEPEN(de)NTK_	KCN(0.053)SDN(0.947)ISYVIHCEP	KCN(-12.57)SDN(12.57)ISYVIHC	6	3	809	1	1
IPI00136642	188	IVSANRIFGDKSITFNESYQDVSEVVYGAKI	1	90.7179	317.58	90.718	_SITFN(de)ESYQ(de)DVSEVVYGAK_	SITFN(1)ESYQDVSEVVYGAK	SITFN(90.72)ESYQDVSEVVYGAK	5	2	1069	1	1
IPI00165730	520	NAENAISARSDINPANGSYPFQAIHQRAHGG	1	63.6823	200.77	63.682	_SDIN(de)PAN(de)GSYPFQAIHQR_	SDIN(1)PAN(1)GSYPFQAIHQR	SDIN(63.68)PAN(63.68)GSYPFQA	7	3	673	1	1
IPI00154056	133	IAGIFPPNEVQHFNPNISWQPIPVHTVPITE	0.881192	8.07688	99.357	71.359	_TIM(ox)SAEANIAGIFPPNEVQHFN(de)PN(de)ISWQPIPVHTVPITEDR_	TIMSAEANIAGIFPPN(0.237)EVQHFN	TIMSAEAN(-39.32)IAGIFPPN(-8.	24	4	1150	1	1
IPI00129304	416	MQQDMMRSKIDTEVANISVVMEEMKIVDSKH	1	59.3449	107.5	59.345	_SKIDTEVAN(de)ISVVM(ox)EEM(ox)K_	SKIDTEVAN(1)ISVVMEEMK	SKIDTEVAN(59.34)ISVVMEEMK	9	3	686	1	1
IPI00123223	871	HTSSWIVTPKSIGNVNFSVSAEAQQSSEPCG	1	66.3652	203.79	66.365	_SIGN(de)VN(de)FSVSAEAQQSSEPCGSEVATVPETGRK_	SIGN(1)VN(1)FSVSAEAQQSSEPCGSE	SIGN(66.37)VN(66.37)FSVSAEAQ	6	3	1142	1	1
IPI00405742	127	CGSIFKGICAIRAMSNISVRIFYEDGSGEKS	1	179.372	179.37	179.37	_AM(ox)SN(de)ISVR_	AMSN(1)ISVR	AMSN(179.37)ISVR	4	2	448	1	1
IPI00321348	461	AGGTVYRGETASIICNISVRGGPPGIRIAAS	1	181.707	181.71	181.71	_GETASIICN(de)ISVR_	GETASIICN(1)ISVR	GETASIICN(181.71)ISVR	9	2	711	1	1
IPI00117842	294	TPHVIWPWGCDKQFFNASVQFDNMDPIIDYI	0.999774	36.4596	178.72	178.72	_QFFN(de)ASVQFDNM(ox)DPIIDYINQR_	QFFN(1)ASVQFDNMDPIIDYINQR	QFFN(36.46)ASVQFDN(-36.46)MD	4	3	898	1	1
IPI00396840	650	EAFKQQTECMVQQYSNYSVNGEPVNGRHTIG	1	64.4223	86.625	64.422	_QQTECM(ox)VQQYSN(de)YSVN(de)GEPVN(de)GR_	QQTECMVQQYSN(1)YSVN(1)GEPVN(1)	QQTECMVQQYSN(64.42)YSVN(64.4	12	3	903	1	1
IPI00130015	53	TWVFQVGPRSSRSDINCSVMEATEEKVVVHI	1	138.573	152.94	138.57	_SDIN(de)CSVM(ox)EATEEK_	SDIN(1)CSVMEATEEK	SDIN(138.57)CSVMEATEEK	4	2	815	1	1
IPI00118674	386	TSIDIWMHTDPMSQKNESVKNQVEDIIATIE	1	87.1385	101.05	87.138	_TSIDIWM(ox)HTDPM(ox)SQKN(de)ESVK_	TSIDIWMHTDPMSQKN(1)ESVK	TSIDIWMHTDPMSQKN(87.14)ESVK	16	3	794	1	1
IPI00123996	261	VFYTDSAIAKEGFSANYSVIQSSISEDFKCM	1	97.2709	165.19	97.271	_EGFSAN(de)YSVIQSSISEDFK_	EGFSAN(1)YSVIQSSISEDFK	EGFSAN(97.27)YSVIQSSISEDFK	6	2	1055	1	1
IPI00127447	412	ETGDIRTMVFPVMYINESVIIDKETANQIKS	1	53.4544	224.86	53.454	_TM(ox)VFPVM(ox)YIN(de)ESVIIDKETAN(de)QIK_	TMVFPVMYIN(1)ESVIIDKETAN(1)QI	TMVFPVMYIN(53.45)ESVIIDKETAN	10	3	940	1	1
IPI00116056	192	GHSWNSPNCTDPKIINASVIGDHTKYSKYKF	1	72.7049	72.705	72.705	_IIN(de)ASVIGDHTK_	IIN(1)ASVIGDHTK	IIN(72.7)ASVIGDHTK	3	3	424	1	1
IPI00122122	158	GRKISVKIYVRSIPVNDSVIDVFERRVREAN	1	59.6073	154.46	59.607	_SIPVN(de)DSVIDVFERR_	SIPVN(1)DSVIDVFERR	SIPVN(59.61)DSVIDVFERR	5	3	583	1	1
IPI00129867	299	NIQHIHIDHNKIKNVNMSVICPTTIRAEQDA	0.99969	35.09	174.95	174.95	_NVN(de)M(ox)SVICPTTIR_	NVN(1)MSVICPTTIR	N(-35.09)VN(35.09)MSVICPTTIR	3	2	761	1	1
IPI00132600	415	FGPFFRTEQIIIQAPNTSVHIYEPYPAGADV	1	65.2765	166.22	166.22	_TEQIIIQAPN(de)TSVHIYEPYPAGADVPFGPPINK_	TEQIIIQAPN(1)TSVHIYEPYPAGADVP	TEQIIIQAPN(65.28)TSVHIYEPYPA	10	3	1193	1	1
IPI00224752	913	PSTRGIKAATCINPINGSVCERPANHSAKQC	0.996638	24.7189	120.11	116.13	_AATCINPIN(de)GSVCERPAN(de)HSAK_	AATCIN(0.003)PIN(0.997)GSVCER	AATCIN(-24.72)PIN(24.72)GSVC	9	3	790	1	1
IPI00133520	253	SGSPKGDIINIRSAGNMSTYNCQIIKACSKK	1	104.167	154.69	104.17	_SAGN(de)M(ox)STYN(de)CQIIK_	SAGN(1)MSTYN(1)CQIIK	SAGN(104.17)MSTYN(104.17)CQI	4	2	803	1	1
IPI00467600	345	MTDICESKNPCHKNANCSTVSPGQTQCTCQK	0.542682	0.743274	67.416	67.416	_NAN(de)CSTVSPGQTQCTCQK_	N(0.457)AN(0.543)CSTVSPGQTQCT	N(-0.74)AN(0.74)CSTVSPGQTQCT	3	3	681	1	1
IPI00229935	199	ITAVSGEKRSFPVYINGSTVPSPVKDIGISP	1	131.556	131.56	131.56	_SFPVYIN(de)GSTVPSPVK_	SFPVYIN(1)GSTVPSPVK	SFPVYIN(131.56)GSTVPSPVK	7	2	847	1	1
IPI00111228	600	VGMEFFNGRITPNCCNTSTVADAYRFINHTV	0.998051	27.0926	106.93	106.93	_ITPNCCN(de)TSTVADAYR_	ITPN(0.002)CCN(0.998)TSTVADAY	ITPN(-27.09)CCN(27.09)TSTVAD	7	2	922	1	1
IPI00129250	134	NAIRSIPPGIFSTSANISTIVIRENQIREVS	1	77.5052	157.14	77.505	_SIPPGIFSTSAN(de)ISTIVIR_	SIPPGIFSTSAN(1)ISTIVIR	SIPPGIFSTSAN(77.51)ISTIVIR	12	3	659	1	1
IPI00758066	335	CTTPPSAPRSVVHHINGSTIRIEWSAPIESG	1	92.1898	111.12	92.19	_SVVHHIN(de)GSTIR_	SVVHHIN(1)GSTIR	SVVHHIN(92.19)GSTIR	7	2	661	1	1
IPI00118291	130	AIVDDTKSIPQSSWGNWSSWKEVNAKVSVEP	1	102.637	102.64	102.64	_SIPQSSWGN(de)WSSWK_	SIPQSSWGN(1)WSSWK	SIPQSSWGN(102.64)WSSWK	9	2	826	1	1
IPI00122117	546	IRNGDIVSSAARTYENGSSVEYRCFDNHFIQ	1	76.6551	86.911	76.655	_TYEN(de)GSSVEYR_	TYEN(1)GSSVEYR	TYEN(76.66)GSSVEYR	4	2	653	1	1
IPI00930841	1271	GGSCFRECGGRAIITNVSSVAIGSRRFGGII	1	95.0671	95.067	95.067	_AIITN(de)VSSVAIGSR_	AIITN(1)VSSVAIGSR	AIITN(95.07)VSSVAIGSR	5	2	695	1	1
IPI00153807	124	GFSSEKDFEDYIRYDNHSSSVIAAVVFEHSF	1	61.6123	61.612	61.612	_YDN(de)HSSSVIAAVVFEHSFN(de)HSQDPIPIAVK_	YDN(1)HSSSVIAAVVFEHSFN(1)HSQD	YDN(61.61)HSSSVIAAVVFEHSFN(6	3	4	828	1	1
IPI00112614	292	SWSDMRQEVMFITNVNSSSSSTQIYQAVSRI	0.817983	6.52633	185.71	185.71	_QEVM(ox)FITNVN(de)SSSSSTQIYQAVSR_	QEVMFITN(0.182)VN(0.818)SSSSS	QEVMFITN(-6.53)VN(6.53)SSSSS	10	3	898	1	1
IPI00128358	635	DATNPSVPIDPISVSNSSSQIIIKWKPPSDP	0.973864	15.7125	55.691	55.691	_SDIIYVQTDATNPSVPIDPISVSN(de)SSSQIIIK_	SDIIYVQTDATN(0.026)PSVPIDPISV	SDIIYVQTDATN(-15.71)PSVPIDPI	24	4	851	1	1
IPI00467600	626	STIHIRSMANQIITFNISSKGQIIANNVAVD	1	77.8901	188.4	188.4	_SM(ox)ANQIITFN(de)ISSK_	SMANQIITFN(1)ISSK	SMAN(-77.89)QIITFN(77.89)ISS	10	3	524	1	1
IPI00128484	419	AICIDKSIGPNTCSSNGSSIYFIHGPNIYCY	0.970029	16.4176	53.522	53.522	_SIGPN(de)TCSSN(de)GSSIYFIHGPNIYCYSSIDKINAAK_	SIGPN(0.978)TCSSN(0.97)GSSIYF	SIGPN(17.99)TCSSN(16.42)GSSI	10	4	960	1	1
IPI00153416	49	AGCCEEMREIKAQVANISSIIGEISRKQESD	1	85.4573	85.457	85.457	_AQVAN(de)ISSIIGEISR_	AQVAN(1)ISSIIGEISR	AQVAN(85.46)ISSIIGEISR	5	3	520	1	1
IPI00133500	108	PIGVDCWIDNTRIVYNHSSGRVSNAPGVQIR	1	64.7115	132.88	64.711	_IVYN(de)HSSGR_	IVYN(1)HSSGR	IVYN(64.71)HSSGR	4	2	517	1	1

IPI00464256	397	AGFRVTIWVHPFVNYNSSSFGEGVEREIFVR	0.964476	14.3377	116.73	116.73	_VTIWVHPFVNYN(de)SSSFGEGVER_	VTIWVHPFVN(0.036)YN(0.964)SSS	VTIWVHPFVN(-14.34)YN(14.34)S	12	3	842	1	1
IPI00162870	179	NDSGSYFCRGIIGHNNKSSASFRISIGDPGS	1	91.3133	119.21	91.313	_GIIGHN(de)N(de)KSSASFR_	GIIGHN(1)N(1)KSSASFR	GIIGHN(91.31)N(91.31)KSSASFR	7	3	497	1	1
IPI01008227	288	EFFSPEACRSMKITYNESRVFEGIPTYRFTA	1	75.7382	111.74	75.738	_ITYN(de)ESR_	ITYN(1)ESR	ITYN(75.74)ESR	4	2	442	1	1
IPI00120245	615	DATGIQPIINQFTPANVSRQAHIIIDCGEDN	1	203.063	235.92	203.06	_TAADATGIQPIIN(de)QFTPAN(de)VSR_	TAADATGIQPIIN(1)QFTPAN(1)VSR	TAADATGIQPIIN(203.06)QFTPAN(	19	2	1144	1	1
IPI00129158	293	RFYPEDIQIIWIENGNVSRNDTPKNITKNTD	1	49.0765	119.79	49.076	_FYPEDIQIIWIEN(de)GN(de)VSRN(de)DTPKN(de)ITK_	FYPEDIQIIWIEN(1)GN(1)VSRN(1)D	FYPEDIQIIWIEN(49.08)GN(49.08	15	3	1070	1	1
IPI00225715	604	VANRSIIIIGSPTWKNVSRMARSSHKKNQEE	1	100.223	155.43	100.22	_SIIIIGSPTWKN(de)VSR_	SIIIIGSPTWKN(1)VSR	SIIIIGSPTWKN(100.22)VSR	12	3	558	1	1
IPI00135805	497	VTRAIADVIQDIADDNSSRADYTQDPSVIPC	1	129.006	129.01	129.01	_AIADVIQDIADDN(de)SSR_	AIADVIQDIADDN(1)SSR	AIADVIQDIADDN(129.01)SSR	13	3	569	1	1
IPI00127770	98	CQEANKTYCANEPISNCSQVNRTDICSVIPP	0.999945	42.5595	165.58	165.58	_TYCANEPISN(de)CSQVN(de)R_	TYCANEPISN(1)CSQVN(1)R	TYCAN(-42.56)EPISN(42.56)CSQ	10	2	958	1	1
IPI00116509	569	HDVHERGEEKRKQIINISQIVYGPEGSDIVI	1	94.8561	113.63	94.856	_QIIN(de)ISQIVYGPEGSDIVIIK_	QIIN(1)ISQIVYGPEGSDIVIIK	QIIN(94.86)ISQIVYGPEGSDIVIIK	4	3	767	1	1
IPI00131693	70	VTWRVNGTDIAPDIINGSQIIIRSIEIGHSG	1	137.731	137.73	137.73	_VN(de)GTDIAPDIIN(de)GSQIIIR_	VN(1)GTDIAPDIIN(1)GSQIIIR	VN(137.73)GTDIAPDIIN(137.73)	12	2	1006	1	1
IPI00554989	111	TGPGIISMANAGPNTNGSQFFICTAKTEWID	0.987883	19.1132	196.02	196.02	_HTGPGIISM(ox)ANAGPNTN(de)GSQFFICTAK_	HTGPGIISMANAGPN(0.012)TN(0.98	HTGPGIISMAN(-70.67)AGPN(-19.	17	3	937	1	1
IPI00315155	75	KHGSVSIVVGAPRAINASQEETGAVFICPWK	1	83.9663	83.966	83.966	_AIN(de)ASQEETGAVFICPWK_	AIN(1)ASQEETGAVFICPWK	AIN(83.97)ASQEETGAVFICPWK	3	3	675	1	1
IPI00314673	310	YYEQITIKSMAGSGHNVSQEAIAIKRMIEMG	1	56.8168	158.98	56.817	_SM(ox)AGSGHN(de)VSQEAIAIKR_	SMAGSGHN(1)VSQEAIAIKR	SMAGSGHN(56.82)VSQEAIAIKR	8	2	937	1	1
IPI00153807	140	HSSSVIAAVVFEHSFNHSQDPIPIAVKYHIR	1	61.6123	61.612	61.612	_YDN(de)HSSSVIAAVVFEHSFN(de)HSQDPIPIAVK_	YDN(1)HSSSVIAAVVFEHSFN(1)HSQD	YDN(61.61)HSSSVIAAVVFEHSFN(6	19	4	828	1	1
IPI00229820	388	INPIYCDTVKQIYPYNSSNRIIGIIDMAVFD	0.999523	33.2091	112.36	112.36	_QIYPYN(de)SSNR_	QIYPYN(1)SSNR	QIYPYN(33.21)SSN(-33.21)R	6	2	622	1	1
IPI00222967	317	KEVNVYMYIANGSAFNISNKNISFWDAPIGS	0.99999	50.0484	197.14	197.14	_EVNVYM(ox)YIAN(de)GSAFN(de)ISNK_	EVNVYMYIAN(1)GSAFN(1)ISNK	EVN(-62.06)VYMYIAN(62.06)GSA	15	3	718	1	1
IPI00471022	502	VFIGTATGRIIKISINESMQVVSRRVITVAY	1	75.1019	75.102	75.102	_ISIN(de)ESM(ox)QVVSR_	ISIN(1)ESMQVVSR	ISIN(75.1)ESMQVVSR	4	2	690	1	1
IPI00129070	128	AKGWKENPVEFDSIFNESKYTWSWGSPDIIP	1	88.5121	173.37	173.37	_GWKENPVEFDSIFN(de)ESK_	GWKENPVEFDSIFN(1)ESK	GWKEN(-88.51)PVEFDSIFN(88.51	14	2	1014	1	1
IPI00330632	138	RKDPTKPKVKVVDKGNGSKPTSPEEVKFFCE	1	89.9108	114.63	89.911	_VVDKGN(de)GSKPTSPEEVK_	VVDKGN(1)GSKPTSPEEVK	VVDKGN(89.91)GSKPTSPEEVK	6	3	591	1	1
IPI00224752	1197	VATPDEQNRDIDMFINASKNFNINITWATSF	1	108.348	132.32	108.35	_DIDM(ox)FIN(de)ASK_	DIDMFIN(1)ASK	DIDMFIN(108.35)ASK	7	2	586	1	1
IPI00153258	184	FAFIHKDFDIKETYFNISKKYFDIEYVSINF	1	78.1489	147.95	78.149	_ETYFN(de)ISKK_	ETYFN(1)ISKK	ETYFN(78.15)ISKK	5	3	378	1	1
IPI00469218	178	VVIRDATIQAYISSGNFSKEETHCTQDGPSP	1	71.3439	165.02	71.344	_DATIQAYISSGN(de)FSK_	DATIQAYISSGN(1)FSK	DATIQAYISSGN(71.34)FSK	12	3	535	1	1
IPI00129613	245	IRITVIDANDNAPAFNQSIYRARVREDAPPG	1	64.5579	192.09	124.29	_ITVIDANDNAPAFN(de)QSIYR_	ITVIDANDNAPAFN(1)QSIYR	ITVIDAN(-78.04)DN(-64.56)APA	14	3	708	1	1
IPI00136259	235	KTDPANQFEWIENTINSSIWNKEKVYIIAHV	0.98505	19.1872	82.765	82.765	_TDPANQFEWIENTIN(de)SSIWNK_	TDPAN(0.003)QFEWIEN(0.012)TIN	TDPAN(-25.4)QFEWIEN(-19.19)T	15	3	837	1	1
IPI00132600	917	SRKGQNMVCGGMGCDNDSIVQQIFNAAEIDT	0.966455	14.5955	184.03	184.03	_KGQNM(ox)VCGGM(ox)GCDN(de)DSIVQQIFNAAEIDTYTR_	KGQN(0.034)MVCGGMGCDN(0.966)D	KGQN(-14.6)MVCGGMGCDN(14.6)D	14	3	1209	1	1
IPI00321091	54	FTCWWNPGSDGGIPTNYSITYSKEGEKNTYE	0.999937	42.0207	89.039	89.039	_SPDKETFTCWWNPGSDGGIPTN(de)YSITYSK_	SPDKETFTCWWNPGSDGGIPTN(1)YSIT	SPDKETFTCWWN(-42.02)PGSDGGIP	22	3	1104	1	1
IPI01026704	839	TEKNIIIVRSQNDKFNVSITVKNKGDSAYNT	1	92.9251	225.32	92.925	_SQN(de)DKFN(de)VSITVK_	SQN(1)DKFN(1)VSITVK	SQN(92.93)DKFN(92.93)VSITVK	7	3	495	1	1
IPI00988390	213	GVKFGHKQINPPHTANSSITSKVVTTMKPVT	1	73.5891	106.32	106.32	_QINPPHTAN(de)SSITSK_	QINPPHTAN(1)SSITSK	QIN(-73.59)PPHTAN(73.59)SSIT	9	3	533	1	1
IPI00320618	197	NKIIAIRSEEIEFIGNSSIRKIDISSNPIKE	1	158.519	158.52	158.52	_SEEIEFIGN(de)SSIR_	SEEIEFIGN(1)SSIR	SEEIEFIGN(158.52)SSIR	9	2	741	1	1
IPI00124078	104	YSNRTAIFPDIIVQGNASIRIQRVRVTDEGS	1	112.154	112.15	112.15	_TAIFPDIIVQGN(de)ASIR_	TAIFPDIIVQGN(1)ASIR	TAIFPDIIVQGN(112.15)ASIR	12	3	573	1	1
IPI00154056	177	YEQIQNETRQTPEYQNRSIQNAQFINMVANE	1	97.635	97.635	97.635	_QTPEYQN(de)R_	QTPEYQN(1)R	QTPEYQN(97.64)R	7	2	519	1	1
IPI00123704	118	WGQHVQKINKFIEPYNDSIQAQKNDVCRPGR	1	121.041	121.04	121.04	_FIEPYN(de)DSIQAQK_	FIEPYN(1)DSIQAQK	FIEPYN(121.04)DSIQAQK	6	2	777	1	1
IPI00311305	271	IIAQTWQKKKQEIPSNCSIPYHVYNIKSIGV	1	104.251	121.82	121.82	_KKQEIPSN(de)CSIPYHVYNIK_	KKQEIPSN(1)CSIPYHVYNIK	KKQEIPSN(104.25)CSIPYHVYN(-1	8	3	774	1	1
IPI00471187	125	GFSVQGDYQGRVIFKNYSINDATITIHNIGF	0.994895	22.8983	133.74	86.625	_VIFKN(de)YSINDATITIHNIGFSDSGK_	VIFKN(0.995)YSIN(0.005)DATITI	VIFKN(22.9)YSIN(-22.9)DATITI	5	3	919	1	1
IPI00468055	442	TERTVDKSTGKPTIYNVSIIMSDTGGTCY__	1	56.7664	250.86	56.766	_TVDKSTGKPTIYN(de)VSIIM(ox)SDTGGTCY_	TVDKSTGKPTIYN(1)VSIIMSDTGGTCY	TVDKSTGKPTIYN(56.77)VSIIMSDT	13	3	942	1	1
IPI00123223	1180	RDEIIKSIDEEAIKENNSIHWKRPQKSRKSE	1	68.2833	209.67	68.283	_SIDEEAIKEN(de)N(de)SIHWK_	SIDEEAIKEN(1)N(1)SIHWK	SIDEEAIKEN(68.28)N(68.28)SIH	10	2	958	1	1
IPI00515432	91	SEPFVVFQTKEIPVINVSIGPFSTSQVVARE	1	131.655	131.65	131.65	_EIPVIN(de)VSIGPFSTSQVVAR_	EIPVIN(1)VSIGPFSTSQVVAR	EIPVIN(131.65)VSIGPFSTSQVVAR	6	2	1058	1	1
IPI00129159	670	VAVRTISDVPSAAPQNISIEVRNSKSIVIHW	1	186.971	186.97	186.97	_TISDVPSAAPQN(de)ISIEVR_	TISDVPSAAPQN(1)ISIEVR	TISDVPSAAPQN(186.97)ISIEVR	12	2	949	1	1
IPI00126827	216	FGPAIDAVRGSIKITNISIAMSGVYVCKAQN	1	127.355	127.36	127.36	_ITN(de)ISIAM(ox)SGVYVCK_	ITN(1)ISIAMSGVYVCK	ITN(127.36)ISIAMSGVYVCK	3	3	558	1	1
IPI00130654	109	IQDMICDMEGIPQKHNFSHCCGKAGFPRRIC	1	111.866	150.14	150.14	_TANDAIQDMICDMEGIPQKHN(de)FSHCCGK_	TANDAIQDMICDMEGIPQKHN(1)FSHCC	TAN(-111.87)DAIQDMICDMEGIPQK	21	4	820	1	1
IPI00221556	81	EKTGHRPPNPAFWWVNGSGTEVKWTFEEIGK	1	79.313	150.69	150.69	_TGHRPPNPAFWWVN(de)GSGTEVK_	TGHRPPNPAFWWVN(1)GSGTEVK	TGHRPPN(-79.31)PAFWWVN(79.31	14	3	780	1	1
IPI00130661	442	QFIKSSSHIPPSSYFNASGRAYPDVAAISDG	1	55.7548	238.02	55.755	_SSSHIPPSSYFN(de)ASGR_	SSSHIPPSSYFN(1)ASGR	SSSHIPPSSYFN(55.75)ASGR	12	3	566	1	1
IPI00626793	781	RSIDNDNYVFTAPYFNKSGPGAYESGIMVSK	1	104.232	168.69	168.69	_SIDNDNYVFTAPYFN(de)K_	SIDNDNYVFTAPYFN(1)K	SIDN(-129.35)DN(-104.23)YVFT	15	2	955	1	1
IPI00222921	145	ISCTQEDRAFSTIIVNVSGKFFEYTIKGEIS	1	97.8599	97.86	97.86	_AFSTIIVN(de)VSGK_	AFSTIIVN(1)VSGK	AFSTIIVN(97.86)VSGK	8	2	619	1	1
IPI00314726	501	PDAVAAWKIIIRSVYNCSGEACSGHNRSPIV	1	103.649	183.96	183.96	_SVYN(de)CSGEACSGHNR_	SVYN(1)CSGEACSGHNR	SVYN(103.65)CSGEACSGHN(-103.	4	3	567	1	1
IPI00134585	601	TVYNRIDKGGITINANISGDAFIKINPDHIG	1	84.4097	101.96	84.41	_IDKGGITIN(de)AN(de)ISGDAFIK_	IDKGGITIN(1)AN(1)ISGDAFIK	IDKGGITIN(84.41)AN(84.41)ISG	11	3	650	1	1
IPI00154043	275	DIISDTSEVVYKTVANISGCEATDSEAIIHC	1	80.5855	251.35	80.585	_TVAN(de)ISGCEATDSEAIIHCIR_	TVAN(1)ISGCEATDSEAIIHCIR	TVAN(80.59)ISGCEATDSEAIIHCIR	4	3	773	1	1
IPI00468814	250	CSGPQDTDCFACRHFNDSGACVPRCPAPIVY	1	73.4351	76.655	73.435	_HFN(de)DSGACVPR_	HFN(1)DSGACVPR	HFN(73.44)DSGACVPR	3	2	631	1	1
IPI00126186	1204	CVYMDVDGYWRTSYCNESFYFICKKSDEIPA	1	93.7657	171.73	93.766	_TSYCN(de)ESFYFICKK_	TSYCN(1)ESFYFICKK	TSYCN(93.77)ESFYFICKK	5	3	617	1	1
IPI00848693	431	NNGQKIVIIIDPAISNNSFSSNPYGPYDRGS	0.999781	36.6015	112.88	88.071	_IVIIIDPAISN(de)N(de)SFSSNPYGPYDR_	IVIIIDPAISN(1)N(1)SFSSNPYGPYD	IVIIIDPAISN(36.6)N(36.6)SFSS	11	3	885	1	1
IPI00221859	171	GIPIAPCGAIANSIFNDSFSIWHQRQPSDPF	1	58.9522	84.602	58.952	_SDGIPIAPCGAIAN(de)SIFN(de)DSFSIWHQR_	SDGIPIAPCGAIAN(1)SIFN(1)DSFSI	SDGIPIAPCGAIAN(58.95)SIFN(58	18	3	992	1	1
IPI00119063	137	QHHCVPTPSGPTCYCNSSFQIQADGKTCKDF	1	81.3153	81.315	81.315	_M(ox)GCQHHCVPTPSGPTCYCN(de)SSFQIQADGK_	MGCQHHCVPTPSGPTCYCN(1)SSFQIQA	MGCQHHCVPTPSGPTCYCN(81.32)SS	19	3	1114	1	1
IPI00876541	86	FENVSFIESIRAHINNISFPIRGTEADIINI	0.999286	31.4584	113.63	113.63	_AHINN(de)ISFPIR_	AHIN(0.001)N(0.999)ISFPIR	AHIN(-31.46)N(31.46)ISFPIR	5	2	642	1	1
IPI00122581	174	PEVQWYKNCKPIIIDNVSFFGVKDKIIVRNV	0.999999	60.2763	170.11	170.11	_NCKPIIIDN(de)VSFFGVK_	NCKPIIIDN(1)VSFFGVK	N(-60.28)CKPIIIDN(60.28)VSFF	9	3	618	1	1
IPI00828222	1583	IGKAWGTPCEICPSVNTSEYKIICPGGEGFR	1	119.402	119.4	119.4	_AWGTPCEICPSVN(de)TSEYK_	AWGTPCEICPSVN(1)TSEYK	AWGTPCEICPSVN(119.4)TSEYK	13	2	1050	1	1
IPI00608044	107	EQSFHVSIRTIIGYYNQSESGSHTIQWMYGC	1	68.9516	82.586	68.952	_TIIGYYN(de)QSESGSHTIQWM(ox)YGCK_	TIIGYYN(1)QSESGSHTIQWMYGCK	TIIGYYN(68.95)QSESGSHTIQWMYG	7	3	914	1	1
IPI00129677	146	SDVRSISCQMAAFRGNGSERTCCPINWVEYE	1	74.6784	91.355	74.678	_SISCQM(ox)AAFRGN(de)GSER_	SISCQMAAFRGN(1)GSER	SISCQMAAFRGN(74.68)GSER	12	3	597	1	1
IPI00130573	975	VEYRHIWSIEISNKPNISEPEEPKIRFVAGI	0.877285	8.54242	82.007	82.007	_HIWSIEISNKPN(de)ISEPEEPK_	HIWSIEISN(0.123)KPN(0.877)ISE	HIWSIEISN(-8.54)KPN(8.54)ISE	12	3	783	1	1
IPI00229935	1041	RYDIIIINENGIIISNVSEPATARQHKFEDI	0.92347	7.80561	96.379	96.379	_YDIIIIN(de)ENGIIISN(de)VSEPATAR_	YDIIIIN(0.538)EN(0.538)GIIISN	YDIIIIN(0)EN(0)GIIISN(7.81)V	15	3	840	1	1
IPI00467600	2287	KCANIVGIVDYGTRTNKSEMWDVFCYRMKDV	1	71.548	100.38	71.548	_TN(de)KSEM(ox)WDVFCYR_	TN(1)KSEMWDVFCYR	TN(71.55)KSEMWDVFCYR	2	2	877	1	1
IPI00129041	91	QVPQKCRRYRIATIANFSEIGIEPGRDVDIE	1	89.5409	89.541	89.541	_IATIAN(de)FSEIGIEPGR_	IATIAN(1)FSEIGIEPGR	IATIAN(89.54)FSEIGIEPGR	6	3	564	1	1
IPI00990932	285	FSDRAQTISSIRSSANQSEFQQQIQKISIQT	1	142.83	235.22	142.83	_SSAN(de)QSEFQQQIQK_	SSAN(1)QSEFQQQIQK	SSAN(142.83)QSEFQQQIQK	4	3	542	1	1
IPI00123996	522	ENKVFMRKFKIAYSNNGSDWKTIMDDSKRKA	0.977054	16.2922	108.9	108.9	_IAYSNN(de)GSDWK_	IAYSN(0.023)N(0.977)GSDWK	IAYSN(-16.29)N(16.29)GSDWK	6	2	628	1	1
IPI00463492	1299	RSIVIISTRGEIKQINISDSIAVIGIAKPAH	1	86.7997	178.23	86.8	_QIN(de)ISDSIAVIGIAKPAHIYSK_	QIN(1)ISDSIAVIGIAKPAHIYSK	QIN(86.8)ISDSIAVIGIAKPAHIYSK	3	3	780	1	1
IPI00111807	1378	SSISDSSVIKSTTQTNSSDSHIVKTSAFPPI	1	90.4441	90.444	90.444	_STTQTN(de)SSDSHIVK_	STTQTN(1)SSDSHIVK	STTQTN(90.44)SSDSHIVK	6	2	753	1	1
IPI00857780	322	PIKMTQESAIIFPFINKSDSGTYGCTATSNM	1	69.716	125.28	69.716	_M(ox)TQESAIIFPFIN(de)K_	MTQESAIIFPFIN(1)K	MTQESAIIFPFIN(69.72)K	13	3	553	1	1
IPI00114065	634	QDFENGEFWPRSPFYNISDQISFQCYDGYVI	1	67.6437	113.47	67.644	_SPFYN(de)ISDQISFQCYDGYVIR_	SPFYN(1)ISDQISFQCYDGYVIR	SPFYN(67.64)ISDQISFQCYDGYVIR	5	3	858	1	1
IPI00623114	1553	DQDVPVKRNFARIVVNVSDKNDHAPWFTSPS	1	64.3772	72.145	64.377	_IVVN(de)VSDKN(de)DHAPWFTSPSYDGR_	IVVN(1)VSDKN(1)DHAPWFTSPSYDGR	IVVN(64.38)VSDKN(64.38)DHAPW	4	3	869	1	1
IPI00114457	136	YEKTKDISHSIYMFFNTSDIREAVPEPPIIS	1	86.3775	101.62	86.378	_TKDISHSIYM(ox)FFN(de)TSDIR_	TKDISHSIYMFFN(1)TSDIR	TKDISHSIYMFFN(86.38)TSDIR	13	3	731	1	1
IPI00848693	453	PYGPYDRGSAMKIWVNSSDGISPVIGKVWPG	1	116.838	132.84	116.84	_IWVN(de)SSDGISPVIGK_	IWVN(1)SSDGISPVIGK	IWVN(116.84)SSDGISPVIGK	4	3	525	1	1
IPI00625835	483	GCEKSVGTGTNMVFQNCSCIGSSGNSSAVIG	1	73.8051	130.78	73.805	_SVGTGTN(de)M(ox)VFQN(de)CSCIGSSGN(de)SSAVIGICK_	SVGTGTN(1)MVFQN(1)CSCIGSSGN(1)	SVGTGTN(73.81)MVFQN(73.81)CS	12	3	1038	1	1
IPI00381303	693	QISIVKTAIVQEVHQNFSAWCSQVIRIYKGQ	1	99.4091	133.78	99.409	_TAIVQEVHQN(de)FSAWCSQVIR_	TAIVQEVHQN(1)FSAWCSQVIR	TAIVQEVHQN(99.41)FSAWCSQVIR	10	2	1188	1	1
IPI00400073	142	DGVRIPKSIFGAIPGNRSAVRIAITVIDIGA	1	73.0667	123.63	73.067	_SIFGAIPGN(de)R_	SIFGAIPGN(1)R	SIFGAIPGN(73.07)R	9	2	517	1	1
IPI00625835	492	TNMVFQNCSCIGSSGNSSAVIGICKKGPECD	1	73.8051	130.78	73.805	_SVGTGTN(de)M(ox)VFQN(de)CSCIGSSGN(de)SSAVIGICK_	SVGTGTN(1)MVFQN(1)CSCIGSSGN(1)	SVGTGTN(73.81)MVFQN(73.81)CS	21	3	1038	1	1
IPI00654185	593	VSFIPRVVPEPNIVINFSATAIRNSAVKYFV	0.999952	43.1856	130.27	130.27	_VVPEPNIVIN(de)FSATAIR_	VVPEPNIVIN(1)FSATAIR	VVPEPN(-43.19)IVIN(43.19)FSA	10	3	614	1	1
IPI00464135	615	SNIATRIWVHNGAPVNASASCRVIPTGDIII	1	76.3575	121.13	76.358	_IWVHN(de)GAPVN(de)ASASCR_	IWVHN(1)GAPVN(1)ASASCR	IWVHN(76.36)GAPVN(76.36)ASAS	10	3	581	1	1
IPI00113854	364	IIPFDNIHEDPCIITNRSARIPCFIAGDTRS	0.99999	49.798	95.662	95.662	_AIIPFDNIHEDPCIITN(de)R_	AIIPFDNIHEDPCIITN(1)R	AIIPFDN(-49.8)IHEDPCIITN(49.	17	3	714	1	1
IPI00122973	311	IADQIIETQRTITVYNFSAPVITISQIEVSE	1	45.4885	136.34	45.489	_TITVYN(de)FSAPVITISQIEVSEGSQVTVK_	TITVYN(1)FSAPVITISQIEVSEGSQVT	TITVYN(45.49)FSAPVITISQIEVSE	6	3	1005	1	1
IPI00224752	922	TCINPINGSVCERPANHSAKQCRTPCAIRTA	0.999999	61.8803	120.11	116.13	_AATCINPIN(de)GSVCERPAN(de)HSAK_	AATCIN(0.003)PIN(0.997)GSVCER	AATCIN(-24.72)PIN(24.72)GSVC	18	3	790	1	1
IPI00319509	606	IKSGQEDHYWIDVEKNQSAKFQTSSNEWIII	1	69.979	243.86	69.979	_SGQEDHYWIDVEKN(de)QSAK_	SGQEDHYWIDVEKN(1)QSAK	SGQEDHYWIDVEKN(69.98)QSAK	14	2	1068	1	1
IPI00124326	521	EGKNKTVSTIVIQAANVSAIYKCEAINKAGR	1	90.7538	181.51	90.754	_TVSTIVIQAAN(de)VSAIYK_	TVSTIVIQAAN(1)VSAIYK	TVSTIVIQAAN(90.75)VSAIYK	11	2	890	1	1
IPI00760106	230	AGSGPIWIDDIACNGNESAIWDCKHRGWGKH	1	61.6123	61.612	61.612	_IGAGSGPIWIDDIACN(de)GN(de)ESAIWDCK_	IGAGSGPIWIDDIACN(1)GN(1)ESAIW	IGAGSGPIWIDDIACN(61.61)GN(61	18	3	941	1	1
IPI00121378	499	TCTAENQIERTVNSINVSAISIPEHDEADDI	0.999911	40.508	159.98	159.98	_TVNSIN(de)VSAISIPEHDEADDISDENREK_	TVNSIN(1)VSAISIPEHDEADDISDENR	TVN(-40.51)SIN(40.51)VSAISIP	6	3	1033	1	1
IPI00169870	91	RERTAIWVATDHNTDNTSAIIREWIVAVKGI	0.99648	24.5196	192.6	192.6	_TAIWVATDHNTDN(de)TSAIIR_	TAIWVATDHN(0.004)TDN(0.996)TS	TAIWVATDHN(-24.52)TDN(24.52)	13	2	1051	1	1
IPI00129304	271	DWIKEKVQSIQTIAANNSAIAKANNDTIEDM	1	63.1281	190.46	63.128	_VQSIQTIAAN(de)N(de)SAIAK_	VQSIQTIAAN(1)N(1)SAIAK	VQSIQTIAAN(63.13)N(63.13)SAI	10	3	544	1	1
IPI00754853	916	EDAPRGFPQIIGAAGNVSAGSVIIRWIPPVP	1	143.284	143.28	143.28	_GFPQIIGAAGN(de)VSAGSVIIR_	GFPQIIGAAGN(1)VSAGSVIIR	GFPQIIGAAGN(143.28)VSAGSVIIR	11	3	643	1	1
IPI00474494	107	EQIFRVNIRTIISYYNQSAGGSHTIQVISGC	1	151.422	151.42	151.42	_TIISYYN(de)QSAGGSHTIQVISGCEVGSDGR_	TIISYYN(1)QSAGGSHTIQVISGCEVGS	TIISYYN(151.42)QSAGGSHTIQVIS	7	3	1020	1	1
IPI00222967	312	KRFYIKEVNVYMYIANGSAFNISNKNISFWD	1	66.137	182.49	161.8	_EVNVYM(ox)YIAN(de)GSAFN(de)ISNK_	EVNVYMYIAN(1)GSAFN(0.939)ISN(	EVN(-106.47)VYMYIAN(66.14)GS	10	2	1076	1	1
IPI00221706	97	PDIEPECREIIMDFANSSAEITACMVRSARP	1	105.774	130.4	105.77	_EIIM(ox)DFAN(de)SSAEITACM(ox)VR_	EIIMDFAN(1)SSAEITACMVR	EIIMDFAN(105.77)SSAEITACMVR	8	3	731	1	1
IPI00330594	209	AMTSQTRGIIKSSTENTSAEIHVIGRGIEEA	1	108.595	155.22	108.59	_SSTEN(de)TSAEIHVIGR_	SSTEN(1)TSAEIHVIGR	SSTEN(108.59)TSAEIHVIGR	5	3	535	1	1
IPI00331440	166	GYDGQDHIEFCPKTINWSAAEPGAWATKVEW	1	121.436	183.23	121.44	_TIN(de)WSAAEPGAWATKVEWDEHK_	TIN(1)WSAAEPGAWATKVEWDEHK	TIN(121.44)WSAAEPGAWATKVEWDE	3	3	843	1	1
IPI00119063	3478	ITKRCIPRVWVCDRDNDCVDGSDEPANCTQM	0.835859	7.06918	74.287	74.287	_VWVCDRDN(de)DCVDGSDEPANCTQM(ox)TCGVDEFR_	VWVCDRDN(0.836)DCVDGSDEPAN(0.	VWVCDRDN(7.07)DCVDGSDEPAN(-7	8	4	932	1	1

IPI00223352	93	KMTISSIKDDIGACRNCCSVTKAQIQTTIAE	1	79.3106	79.311	79.311	_M(ox)TISSIKDDIGACRN(de)CCSVTK_	MTISSIKDDIGACRN(1)CCSVTK	MTISSIKDDIGACRN(79.31)CCSVTK	15	3	812	1	1
IPI00377953	479	GCSTGIEPNSARCSGNGTYTCGICECDPGYI	1	76.1184	76.118	76.118	_CSGN(de)GTYTCGICECDPGYIGTR_	CSGN(1)GTYTCGICECDPGYIGTR	CSGN(76.12)GTYTCGICECDPGYIGT	4	3	834		1
IPI00134131	194	EHFAKIGWKNHKHSVNNTYSQFQDEYSIEEV	0.5	0	71.592	71.592	_HSVN(de)NTYSQFQDEYSIEEVM(ox)K_	HSVN(0.5)N(0.5)TYSQFQDEYSIEEV	HSVN(0)N(0)TYSQFQDEYSIEEVMK	4	3	856		1
IPI00124497	197	GQRITWDSRRGFIIANATYKEIGIINCEATV	1	112.129	112.13	112.13	_GFIIAN(de)ATYK_	GFIIAN(1)ATYK	GFIIAN(112.13)ATYK	6	2	550		1
IPI00128432	522	PMQWDNSSNAGFTEANHTWIPTNSDYHTVNV	0.594007	0	44.021	44.021	_SPM(ox)QWDN(de)SSN(de)AGFTEANHTWIPTNSDYHTVNVDVQK_	SPMQWDN(0.636)SSN(0.636)AGFTE	SPMQWDN(0)SSN(0)AGFTEAN(0)HT	17	4	1028		1
IPI00947585	124	VSAVSINKGPKCFMANGTWGYPFHDGDYIKD	1	45.6112	163.8	45.611	_CFM(ox)AN(de)GTWGYPFHDGDYIKDQAIWSECKEPR_	CFMAN(1)GTWGYPFHDGDYIKDQAIWSE	CFMAN(45.61)GTWGYPFHDGDYIKDQ	5	4	950		1
IPI00109946	203	STFYTCQVSNPVSSKNDTVYFTIPCDIARSS	0.637427	2.45036	171.5	171.5	_STFYTCQVSNPVSSKN(de)DTVYFTIPCDIAR_	STFYTCQVSN(0.363)PVSSKN(0.637)	STFYTCQVSN(-2.45)PVSSKN(2.45	16	3	1125		1
IPI00122302	172	RIGTNRPSPSVIQEINVTVVTNMCRRRVNVC	0.836296	7.08418	91.583	91.583	_IGTNRPSPSVIQEIN(de)VTVVTNM(ox)CR_	IGTN(0.164)RPSPSVIQEIN(0.836)	IGTN(-7.08)RPSPSVIQEIN(7.08)	15	3	901		1
IPI00169870	179	NIITNPDTISIIIAENKTVVAPMIDSRAAYS	0.999999	61.3932	105.03	105.03	_DM(ox)WADYIIFM(ox)DIDNIITNPDTISIIIAEN(de)K_	DMWADYIIFMDIDNIITNPDTISIIIAEN	DMWADYIIFMDIDN(-66.46)IITN(-	29	4	883		1
IPI00123223	55	YTETPEKICIHIYQINETVTVTASIVSQSGR	1	69.7786	69.779	69.779	_ICIHIYQIN(de)ETVTVTASIVSQSGR_	ICIHIYQIN(1)ETVTVTASIVSQSGR	ICIHIYQIN(69.78)ETVTVTASIVSQ	9	3	897		1
IPI00110852	191	NYKDINGNVFQDAVFNQTVTVIEREDGIDGE	0.999705	32.2905	77.899	77.899	_DIN(de)GNVFQDAVFN(de)QTVTVIER_	DIN(0.5)GN(0.5)VFQDAVFN(1)QTV	DIN(0)GN(0)VFQDAVFN(32.29)QT	13	3	794		1
IPI00988518	346	FMAEVKMNPAGPVIENETVTIICSTPKEAPQ	0.999921	41.0487	68.369	68.369	_M(ox)NPAGPVIEN(de)ETVTIICSTPK_	MNPAGPVIEN(1)ETVTIICSTPK	MN(-41.05)PAGPVIEN(41.05)ETV	10	2	1145		1
IPI00652675	40	TGIPVRSVDFNRGTDNITVRQGDTAIIRCVV	1	111.607	111.61	111.61	_GTDN(de)ITVR_	GTDN(1)ITVR	GTDN(111.61)ITVR	4	2	439		1
IPI00153202	660	SSVAYAMRKYFSIIKNQTVPFIEEDVRVSDI	1	105.389	105.39	105.39	_KYFSIIKN(de)QTVPFIEEDVR_	KYFSIIKN(1)QTVPFIEEDVR	KYFSIIKN(105.39)QTVPFIEEDVR	8	3	776		1
IPI00221818	354	IIYFGYSIIGAHPIINQTVNRMQRAASGWRD	0.834804	7.03586	73.928	73.928	_IIYFGYSIIGAHPIIN(de)QTVNR_	IIYFGYSIIGAHPIIN(0.835)QTVN(0.	IIYFGYSIIGAHPIIN(7.04)QTVN(-	16	3	797		1
IPI00381303	310	YFIKIASSQKGFYRTNHTVMTMGSDFHYENA	0.993623	24.9361	59.585	59.585	_TN(de)HTVM(ox)TM(ox)GSDFHYENANM(ox)WFK_	TN(0.994)HTVMTMGSDFHYEN(0.003)	TN(24.94)HTVMTMGSDFHYEN(-24.	2	3	904		1
IPI00377953	705	CVMMFSYTEIPNGRSNITVIREPECGSAPNA	1	105.568	105.57	105.57	_SN(de)ITVIR_	SN(1)ITVIR	SN(105.57)ITVIR	2	2	402		1
IPI00314132	143	SFEIKTFNVSEDTKVNTTVIPETQIKATDAD	1	96.3913	96.391	96.391	_TFN(de)VSEDTKVN(de)TTVIPETQIK_	TFN(1)VSEDTKVN(1)TTVIPETQIK	TFN(96.39)VSEDTKVN(96.39)TTV	11	3	789		1
IPI00131021	127	NNPVMSIQDQVRFVKNITTWNEMKPGFYHGH	0.764443	5.11248	67.194	67.194	_FVKN(de)ITTWNEM(ox)KPGFYHGHISYIDFAK_	FVKN(0.764)ITTWN(0.236)EMKPGF	FVKN(5.11)ITTWN(-5.11)EMKPGF	4	4	816		1
IPI00808027	251	KPHVWKPRDWHRKQINITTVVAPHEAISRAE	1	83.54	83.54	83.54	_KQIN(de)ITTVVAPHEAISR_	KQIN(1)ITTVVAPHEAISR	KQIN(83.54)ITTVVAPHEAISR	4	3	627		1
IPI00120155	83	KTNHAAVPREQVTVINRTTSSVTFTDVVIPS	1	109.29	109.29	109.29	_EQVTVIN(de)R_	EQVTVIN(1)R	EQVTVIN(109.29)R	7	2	480		1
IPI00760106	444	TGATNTWIFPGSCNGNETTFWQCKNWQWGGI	0.999994	52.3571	76.049	76.049	_TGATNTWIFPGSCN(de)GN(de)ETTFWQCK_	TGATNTWIFPGSCN(1)GN(1)ETTFWQC	TGATN(-47.71)TWIFPGSCN(47.71	16	3	927		1
IPI00349830	389	FDSQCMGAMHWQVMHNVTTETKFIIKIIKEG	1	52.7904	52.79	52.79	_TPIHYFDSQCM(ox)GAM(ox)HWQVM(ox)HN(de)VTTETK_	TPIHYFDSQCMGAMHWQVMHN(1)VTTET	TPIHYFDSQCMGAMHWQVMHN(52.79)	21	4	825		1
IPI00118674	128	TTSRIAGIAVTIAKPNSTSSFSPSVQCPNDG	0.980365	18.2249	48.65	48.65	_IAGIAVTIAKPN(de)STSSFSPSVQCPNDGFGIYSNSYGPEFAHCK_	IAGIAVTIAKPN(0.98)STSSFSPSVQC	IAGIAVTIAKPN(18.22)STSSFSPSV	12	5	916		1
IPI00406603	1459	PDITHIPPPSPSPTINNTSISRKFNPIVVVG	1	65.4382	88.09	65.438	_TIPHPDITHIPPPSPSPTIN(de)N(de)TSISR_	TIPHPDITHIPPPSPSPTIN(1)N(1)TS	TIPHPDITHIPPPSPSPTIN(65.44)N	20	3	931		1
IPI00750091	254	YTKIFIYSGEPIYIGNETSIFGPKGNKTIAI	1	126.073	126.07	126.07	_IFIYSGEPIYIGN(de)ETSIFGPK_	IFIYSGEPIYIGN(1)ETSIFGPK	IFIYSGEPIYIGN(126.07)ETSIFGP	13	3	783		1
IPI00121274	252	SDFERIVIEKKTHMFNFTSGFKVDGVMDIGI	1	93.0108	93.011	93.011	_KTHM(ox)FN(de)FTSGFK_	KTHMFN(1)FTSGFK	KTHMFN(93.01)FTSGFK	6	3	488		1
IPI00404551	134	VHHKYNSDKSSTYVKNGTSFDIHYGSGSISG	1	77.9389	77.939	77.939	_N(de)GTSFDIHYGSGSISGYISQDTVSVPCK_	N(1)GTSFDIHYGSGSISGYISQDTVSVP	N(77.94)GTSFDIHYGSGSISGYISQD	1	3	993		1
IPI00111137	93	VAPCTDFFSFACEKANGTSDSFQAITEENKS	0.999974	45.8553	51.052	51.052	_SVAPCTDFFSFACEKAN(de)GTSDSFQAITEENK_	SVAPCTDFFSFACEKAN(1)GTSDSFQAI	SVAPCTDFFSFACEKAN(45.86)GTSD	17	4	866		1
IPI00461493	220	KSINVQKASTERITSNATSDINIKVDGRAIV	0.998201	27.4415	99.531	99.531	_ITSN(de)ATSDINIK_	ITSN(0.998)ATSDIN(0.002)IK	ITSN(27.44)ATSDIN(-27.44)IK	4	2	639		1
IPI00225715	271	FWSTNIYRITSFMIENGTSDCNIPENPIFIS	0.999097	32.6376	80.067	80.067	_ITSFM(ox)IEN(de)GTSDCNIPENPIFISCDGRN(de)HTISGSK_	ITSFMIEN(0.999)GTSDCN(0.006)I	ITSFMIEN(32.64)GTSDCN(-21.72	8	4	983		1
IPI00330542	305	INCFRGKGEDYRGTTNTTSAGVPCQRWDAQS	1	88.5614	88.561	88.561	_GTTN(de)TTSAGVPCQR_	GTTN(1)TTSAGVPCQR	GTTN(88.56)TTSAGVPCQR	4	2	726		1
IPI00876558	817	MMGYWSTQGCKIVDTNKTRTTCACSHITNFA	1	156.347	156.35	156.35	_IVDTN(de)KTR_	IVDTN(1)KTR	IVDTN(156.35)KTR	5	2	474		1
IPI00311305	77	IDSTRQTWNKSIYIINSTRSAIGRTIQHIYD	1	98.9426	98.943	98.943	_SIYIIN(de)STR_	SIYIIN(1)STR	SIYIIN(98.94)STR	6	2	534		1
IPI00848693	963	SEANCSARGCIWEASNTTRGPPCYFAHEIYS	1	205.562	205.56	205.56	_GCIWEASN(de)TTR_	GCIWEASN(1)TTR	GCIWEASN(205.56)TTR	8	2	648		1
IPI00134585	546	TSQMGYPVVTVSGRQNITQKRFIIDSKADPS	1	124.834	124.83	124.83	_QN(de)ITQKR_	QN(1)ITQKR	QN(124.83)ITQKR	2	2	445		1
IPI00172354	108	GFTGTMPATNVSIFINNTQISDTGTYQCIVN	0.815443	6.45028	98.94	98.94	_VGFTGTM(ox)PATN(de)VSIFIN(de)NTQISDTGTYQCIVNNIPDR_	VGFTGTMPATN(0.999)VSIFIN(0.81	VGFTGTMPATN(32.04)VSIFIN(6.4	17	4	1020		1
IPI00131830	39	DGTKEMDIVFHEHQDNGTQDDSITIASVNTD	0.990421	20.1451	54.311	54.311	_EM(ox)DIVFHEHQDN(de)GTQDDSITIASVNTDFAFSIYK_	EMDIVFHEHQDN(0.99)GTQDDSITIAS	EMDIVFHEHQDN(20.15)GTQDDSITI	12	4	977		1
IPI00420791	247	AIQGTKGICPIFCHTNGTPIGIGARATNPNI	1	93.7544	93.754	93.754	_GICPIFCHTN(de)GTPIGIGAR_	GICPIFCHTN(1)GTPIGIGAR	GICPIFCHTN(93.75)GTPIGIGAR	10	3	681		1
IPI00314132	84	STPYAFRIEGKDIFINVTPDYEENSIIQADV	0.790784	5.77462	84.762	84.762	_DIFIN(de)VTPDYEENSIIQADVECK_	DIFIN(0.791)VTPDYEEN(0.209)SI	DIFIN(5.77)VTPDYEEN(-5.77)SI	5	3	905		1
IPI00126188	150	TPSPTTKAVIFAKKINTTNGKSDFQVPCQYS	1	116.605	116.6	116.6	_KIN(de)TTN(de)GKSDFQVPCQYSQQIK_	KIN(1)TTN(1)GKSDFQVPCQYSQQIK	KIN(116.6)TTN(116.6)GKSDFQVP	3	3	863		1
IPI00224752	415	KDKIYMYGGKIDSTGNVTNEIRVFHIHNESW	0.645418	2.60125	81.565	81.565	_IDSTGN(de)VTNEIR_	IDSTGN(0.645)VTN(0.355)EIR	IDSTGN(2.6)VTN(-2.6)EIR	6	2	660		1
IPI00127447	206	FKPDVSPNFGIFYERNGTNDGEYVFITGEDN	0.5	0	155.86	155.86	_N(de)GTNDGEYVFITGEDNYIN(de)FSK_	N(0.5)GTN(0.5)DGEYVFITGEDN(0.	N(0)GTN(0)DGEYVFITGEDN(-12.8	1	3	834		1
IPI01026704	698	QKKNCRVEGKETVCINATMCFHVKIKSKEDS	1	64.2439	113.72	64.244	_ETVCIN(de)ATM(ox)CFHVK_	ETVCIN(1)ATMCFHVK	ETVCIN(64.24)ATMCFHVK	6	2	864		1
IPI00355482	73	YIIVKIEDPKVIQVVNVTKTSIAVTDFTVNI	1	79.1482	109.66	79.148	_VIQVVN(de)VTK_	VIQVVN(1)VTK	VIQVVN(79.15)VTK	6	2	501		1
IPI00828222	2699	NYYADNQTCDGEIIFNMTKKMCCCSYNIGRA	1	95.2371	102.78	102.78	_NYYADN(de)QTCDGEIIFN(de)M(ox)TKK_	NYYADN(1)QTCDGEIIFN(1)MTKK	N(-75.45)YYADN(75.45)QTCDGEI	16	3	815		1
IPI00131143	213	QKREAYRYIQKAAGMNHTKAIERVSYAIIFG	1	105.521	105.52	105.52	_AAGM(ox)N(de)HTK_	AAGMN(1)HTK	AAGMN(105.52)HTK	5	2	424		1
IPI00120351	101	QIIPVSEAIPHESFDNETIVNDIMIIKIERK	0.962158	14.0528	103.25	103.25	_NTQIIPVSEAIPHESFDN(de)ETIVNDIM(ox)IIK_	NTQIIPVSEAIPHESFDN(0.962)ETIV	N(-71.12)TQIIPVSEAIPHESFDN(1	18	3	1100		1
IPI00110896	114	RCTICDEEHGIEVETNCTITQNTKCKCKPDF	0.999952	43.1921	168.64	168.64	_RCTICDEEHGIEVETN(de)CTITQNTK_	RCTICDEEHGIEVETN(1)CTITQNTK	RCTICDEEHGIEVETN(43.19)CTITQ	16	3	970		1
IPI00126834	273	WGRKIDNEVIQIISGNATITIIAMRMEDSGV	1	67.1558	166.57	166.57	_KIDNEVIQIISGN(de)ATITIIAM(ox)R_	KIDNEVIQIISGN(1)ATITIIAMR	KIDN(-67.16)EVIQIISGN(67.16)	13	3	811		1
IPI00875870	913	GIKGVVTDEQGIPIANATISVSGINHGVKTA	0.868409	8.19497	102.66	102.66	_GVVTDEQGIPIAN(de)ATISVSGINHGVK_	GVVTDEQGIPIAN(0.868)ATISVSGIN	GVVTDEQGIPIAN(8.19)ATISVSGIN	13	3	860		1
IPI00895378	199	PSSQPPISCSEEGVGNATISPVMGEECVRVW	1	69.2844	69.284	69.284	_VIPSSQPPISCSEEGVGN(de)ATISPVM(ox)GEECVR_	VIPSSQPPISCSEEGVGN(1)ATISPVMG	VIPSSQPPISCSEEGVGN(69.28)ATI	18	4	827		1
IPI00356462	491	IRQEHSQIVIYHQSINETISKMQEAHIQIIS	1	86.213	86.213	86.213	_QEHSQIVIYHQSIN(de)ETISK_	QEHSQIVIYHQSIN(1)ETISK	QEHSQIVIYHQSIN(86.21)ETISK	14	3	752		1
IPI00225072	389	YPMMCHIIGIKPHPNNGTISHTKCIIVDQWC	0.499728	0	67.641	67.641	_QSTINTVDIYPMMCHIIGIKPHPN(de)NGTISHTK_	QSTIN(0.001)TVDIYPMMCHIIGIKPH	QSTIN(-29.63)TVDIYPMMCHIIGIK	25	4	905		1
IPI00225715	291	CNIPENPIFISCDGRNHTISGSKVQKNDFHR	0.805917	6.31361	80.067	80.067	_ITSFM(ox)IEN(de)GTSDCNIPENPIFISCDGRN(de)HTISGSK_	ITSFMIEN(0.999)GTSDCN(0.006)I	ITSFMIEN(32.64)GTSDCN(-21.72	28	4	983		1
IPI00277141	509	VKSQIIQHKKEADDYNITISEVKVNEMQFPP	1	156.243	156.24	156.24	_EADDYN(de)ITISEVK_	EADDYN(1)ITISEVK	EADDYN(156.24)ITISEVK	6	2	749		1
IPI00120351	151	KPGQVCTVAGWGKIANCTISDTIQEVNIEVQ	0.999999	59.6344	133.78	133.78	_IAN(de)CTISDTIQEVNIEVQK_	IAN(1)CTISDTIQEVNIEVQK	IAN(59.63)CTISDTIQEVN(-59.63	3	3	726		1
IPI00122285	81	SAAPIVPAVPRPEVDNITIRYRSIVYQINFD	1	93.7302	93.73	93.73	_RVSN(de)ESAAPIVPAVPRPEVDN(de)ITIR_	RVSN(1)ESAAPIVPAVPRPEVDN(1)IT	RVSN(93.73)ESAAPIVPAVPRPEVDN	21	3	901		1
IPI00113042	487	ERSIAYINSDSAIEGNYTIRVDCTPIINQIV	1	92.3804	92.38	92.38	_SIAYIN(de)SDSAIEGN(de)YTIR_	SIAYIN(1)SDSAIEGN(1)YTIR	SIAYIN(92.38)SDSAIEGN(92.38)	14	3	664		1
IPI00355482	96	VTDFTVNIKTFPGETNVTIQIWESEGRQTTI	1	119.377	127.29	119.38	_TFPGETN(de)VTIQIWESEGR_	TFPGETN(1)VTIQIWESEGR	TFPGETN(119.38)VTIQIWESEGR	7	2	1033		1
IPI00828867	1114	SVQQMSDGTHTGKAINFTIPFFDSSRGGRPR	1	97.5513	97.551	97.551	_AIN(de)FTIPFFDSSR_	AIN(1)FTIPFFDSSR	AIN(97.55)FTIPFFDSSR	3	2	758		1
IPI00109996	200	EGECVEWIHRYIKNGNATIIRTDSPKAHVTH;	1	86.3126	151.79	86.313	_YIKN(de)GN(de)ATIIR_	YIKN(1)GN(1)ATIIR	YIKN(86.31)GN(86.31)ATIIR	6	2	633		1
IPI00626950	255	IDIGGASTQITFVPQNSTIESPENSIQFRIY	0.917167	10.4424	51.617	51.617	_QETFGAIDIGGASTQITFVPQN(de)STIESPENSIQFR_	QETFGAIDIGGASTQITFVPQN(0.917)	QETFGAIDIGGASTQITFVPQN(10.44	22	4	947		1
IPI00173181	338	FIISVKSVAFQDCQVNITIEGRIDTQGFIRT	1	233.371	233.37	233.37	_SVAFQDCQVN(de)ITIEGR_	SVAFQDCQVN(1)ITIEGR	SVAFQDCQVN(233.37)ITIEGR	10	2	919		1
IPI00467600	1108	TSIQGSFIRIDKADGNITIEGASFVDGDNAA	0.999567	34.0066	59.282	59.282	_IDKADGN(de)ITIEGASFVDGDNAATNGVVHIIN(de)K_	IDKADGN(1)ITIEGASFVDGDN(0.076)	IDKADGN(34.01)ITIEGASFVDGDN(	7	4	818		1
IPI00118291	271	PGVDTTITWRKQRFQNYTICESFSKRCEVSN	1	125.033	125.03	125.03	_FQN(de)YTICESFSK_	FQN(1)YTICESFSK	FQN(125.03)YTICESFSK	3	2	763		1
IPI00112047	27	RKPGHDEGAHNPDYENITIAFRNKDQIKISQ	0.855732	7.73169	69.935	69.935	_PGHDEGAHNPDYEN(de)ITIAFR_	PGHDEGAHN(0.144)PDYEN(0.856)I	PGHDEGAHN(-7.73)PDYEN(7.73)I	14	2	1128		1
IPI00118291	323	TAENQIRKRSVNINFNITHRVHPKAPQDVTI	0.970819	15.2209	114.72	114.72	_SVNINFN(de)ITHR_	SVNIN(0.029)FN(0.971)ITHR	SVN(-54.47)IN(-15.22)FN(15.2	7	2	658		1
IPI00318748	200	TGAVKVTPGAIIGISNITHISIKYNNITKVP	1	69.3307	69.331	69.331	_VTPGAIIGISN(de)ITHISIK_	VTPGAIIGISN(1)ITHISIK	VTPGAIIGISN(69.33)ITHISIK	11	3	612		1
IPI00316045	229	QEANIRSCIISRIENNCTHGEDTWMECEDPF	0.5	0	178.71	178.71	_IEN(de)NCTHGEDTWM(ox)ECEDPFEIK_	IEN(0.5)N(0.5)CTHGEDTWMECEDPF	IEN(0)N(0)CTHGEDTWMECEDPFEIK	4	3	924		1
IPI00112614	1499	IPPPQRKQKTADIIQNITGRNISDYIVKTYV	1	107.088	107.09	107.09	_TADIIQN(de)ITGR_	TADIIQN(1)ITGR	TADIIQN(107.09)ITGR	7	2	602		1
IPI00123223	776	PDTITEWKAGAICISNDTGIGISSVVPIQAF	1	61.7245	112.32	61.725	_AGAICISN(de)DTGIGISSVVPIQ(de)AFKPFFVEVSIPYSVVR_	AGAICISN(1)DTGIGISSVVPIQAFKPF	AGAICISN(61.72)DTGIGISSVVPIQ	8	3	1347		1
IPI01027336	676	TVPPSIRKQPSPSPGNTTGGPIPGSAASPAH	0.999937	42.0056	67.367	67.367	_KQPSPSPGN(de)TTGGPIPGSAASPAHQASQPIYINHP_	KQPSPSPGN(1)TTGGPIPGSAASPAHQA	KQPSPSPGN(42.01)TTGGPIPGSAAS	9	4	866		1
IPI00221701	426	KICKPESGECINCIHNTTGFWCEKCIEGYVR	0.999889	39.5403	121.21	121.21	_ICKPESGECINCIHN(de)TTGFWCEK_	ICKPESGECINCIHN(1)TTGFWCEK	ICKPESGECIN(-39.54)CIHN(39.5	15	3	948		1
IPI00467908	46	GQEPCVNEGTCVTYHNGTGFCRCPEGFIGEY	0.999948	42.872	93.269	93.269	_GGQEPCVNEGTCVTYHN(de)GTGFCR_	GGQEPCVNEGTCVTYHN(1)GTGFCR	GGQEPCVN(-42.87)EGTCVTYHN(42	17	3	868		1
IPI00848693	1666	FPRARWYDYYKGVDINATGEWKTIEAPIEYI	1	93.0108	93.011	93.011	_GVDIN(de)ATGEWK_	GVDIN(1)ATGEWK	GVDIN(93.01)ATGEWK	5	2	596		1
IPI00138199	1711	KCHESCMECKGPGSKNCTGCSAGIIIDMDDN	1	111.86	118.52	118.52	_GPGSKN(de)CTGCSAGIIIDM(ox)DDNR_	GPGSKN(1)CTGCSAGIIIDMDDNR	GPGSKN(111.86)CTGCSAGIIIDMDD	6	3	786		1
IPI00114065	797	NIYIVIDGSDSIGSSNFTGAKRCITNIIEKV	0.999995	52.6839	80.355	80.355	_IVIDPSGSM(ox)NIYIVIDGSDSIGSSN(de)FTGAK_	IVIDPSGSMNIYIVIDGSDSIGSSN(1)F	IVIDPSGSMN(-52.68)IYIVIDGSDS	25	3	1026		1
IPI00123925	49	PEGNTKRAITIKDIINGTFSYKTYFPNWISE	1	186.772	186.77	186.77	_AITIKDIIN(de)GTFSYK_	AITIKDIIN(1)GTFSYK	AITIKDIIN(186.77)GTFSYK	9	2	843		1
IPI00123783	1656	GWTTFFDYDSEGRITNVTFPTGVVTNIHGDM	1	70.0935	117.3	117.3	_ITN(de)VTFPTGVVTNIHGDM(ox)DK_	ITN(1)VTFPTGVVTNIHGDMDK	ITN(70.09)VTFPTGVVTN(-70.09)	3	3	726		1
IPI00975193	528	QIRDQCIVDDITYNVNDTFHKRHEEGHMINC	1	62.8609	223.11	62.861	_DQCIVDDITYN(de)VN(de)DTFHK_	DQCIVDDITYN(1)VN(1)DTFHK	DQCIVDDITYN(62.86)VN(62.86)D	13	3	734		1
IPI00118475	183	CGSIPGCPGTAGFHSNQTFHFIKCCNYTHCN	1	74.3246	74.325	74.325	_GCGSIPGCPGTAGFHSN(de)QTFHFIK_	GCGSIPGCPGTAGFHSN(1)QTFHFIK	GCGSIPGCPGTAGFHSN(74.32)QTFH	17	3	860		1
IPI00987265	287	IPRDPCITNFGGCPSNSTFCIYRGPGKATCM	1	96.1768	198.24	198.24	_VCIPRDPCITNFGGCPSN(de)STFCIYR_	VCIPRDPCITNFGGCPSN(1)STFCIYR	VCIPRDPCITN(-96.18)FGGCPSN(9	18	3	998		1
IPI00761772	634	WIIINEAGIYWRAVGNSTFAIACIQRAINIA	1	106.525	106.52	106.52	_AVGN(de)STFAIACIQR_	AVGN(1)STFAIACIQR	AVGN(106.52)STFAIACIQR	4	2	755		1
IPI00674255	149	CEVRPIGNINRSSIRNGTEVVSCHPQGSTAG	1	109.37	109.37	109.37	_SSIRN(de)GTEVVSCHPQGSTAGVVYR_	SSIRN(1)GTEVVSCHPQGSTAGVVYR	SSIRN(109.37)GTEVVSCHPQGSTAG	5	3	855		1
IPI00970438	267	HVSFKPTVAQQRKCPNCTETAVNGEIVVMYD	0.999248	30.995	90.464	90.464	_KCPN(de)CTETAVN(de)GEIVVM(ox)YDVNREEK_	KCPN(0.999)CTETAVN(0.947)GEIV	KCPN(31)CTETAVN(12.5)GEIVVMY	4	3	992		1
IPI00128206	232	SSKSRAPRTTPPIQINETENTEQDDIPADCS	0.917183	10.4434	80.082	80.082	_TTPPIQIN(de)ETENTEQDDIPADCSAVYNR_	TTPPIQIN(0.917)ETEN(0.083)TEQ	TTPPIQIN(10.44)ETEN(-10.44)T	8	4	799		1
IPI00776098	58	KSIKDIKQFAPSPNCNKTEIIATIKNGDQTC	0.5	0	110.97	110.97	_QFAPSPN(de)CNKTEIIATIK_	QFAPSPN(0.5)CN(0.5)KTEIIATIK	QFAPSPN(0)CN(0)KTEIIATIK	9	3	678		1
IPI00128567	296	IIARIVGCPPGGAGGNDTEIIACIRTRPAQD	1	64.2981	64.298	64.298	_IVGCPPGGAGGN(de)DTEIIACIR_	IVGCPPGGAGGN(1)DTEIIACIR	IVGCPPGGAGGN(64.3)DTEIIACIR	12	3	710		1
IPI00848693	1229	PYWSIGFQICRYGYENDTEIANIYDEMVAKQ	0.999994	52.511	137.45	137.45	_YGYEN(de)DTEIANIYDEM(ox)VAK_	YGYEN(1)DTEIANIYDEMVAK	YGYEN(52.51)DTEIAN(-52.51)IY	5	3	752		1
IPI00112322	327	GDHGVVVFSIGSMVSNMTEEKANAIAWAIAQ;	1	206.328	206.33	206.33	_DM(ox)EEFVQSSGDHGVVVFSIGSM(ox)VSN(de)M(ox)TEEK_	DMEEFVQSSGDHGVVVFSIGSMVSN(1)M	DMEEFVQSSGDHGVVVFSIGSMVSN(20	25	3	1109		1
IPI00129253	1226	DCDDNQPGAICYYPGNETEEEVRAIDTAKCP	0.999986	48.4646	53.998	53.998	_TADCDDNQPGAICYYPGN(de)ETEEEVR_	TADCDDNQPGAICYYPGN(1)ETEEEVR	TADCDDN(-48.46)QPGAICYYPGN(4	18	3	969		1

IPI00308971	1358	SYMFEWRTQYACPPFNVTECSVQDAAGNSID	0.99995	43.0479	63.893	63.893 _TQYACPPFN(de)VTECSVQDAAGNSIDISSISR_	TQYACPPFN(1)VTECSVQDAAGNSIDIS	TQYACPPFN(43.05)VTECSVQDAAGN	9	3	1097	1
IPI00342691	93	EFDIHHVICIRKKITNITEAISDIPRYTTHI	1	128.313	128.31	128.31 _KITN(de)ITEAISDIPR_	KITN(1)ITEAISDIPR	KITN(128.31)ITEAISDIPR	4	3	525	1
IPI00975193	1006	QQTTKIDAPTNIQFVNETDRTVIVTWTPPRA	1	82.9247	181.51	82.925 _IDAPTN(de)IQFVN(de)ETDR_	IDAPTN(1)IQFVN(1)ETDR	IDAPTN(82.92)IQFVN(82.92)ETD	11	3	579	1
IPI00378224	262	PNIIIFQGAKPMARFNHTDRTIETIKIFIFN	1	89.1712	120.57	89.171 _FN(de)HTDRTIETIK_	FN(1)HTDRTIETIK	FN(89.17)HTDRTIETIK	2	3	493	1
IPI00273133	683	KYISVQAIEAPRITQNITDIIVNVSDSIEMR	1	85.6359	85.636	85.636 _ITQN(de)ITDIIVN(de)VSDSIEM(ox)R_	ITQN(1)ITDIIVN(1)VSDSIEMR	ITQN(85.64)ITDIIVN(85.64)VSD	4	3	727	1
IPI00127447	249	KTSIDWWTTDTCNMINGTDGDSFHPIISKDE	0.999906	40.2882	197.92	197.92 _TSIDWWTTDTCNM(ox)IN(de)GTDGDSFHPIISK_	TSIDWWTTDTCNMIN(1)GTDGDSFHPII	TSIDWWTTDTCN(-40.29)MIN(40.2	15	3	1077	1
IPI00134808	74	GPPPAIPNAIPASDVNRTDFESHTTIKYECI	0.992266	21.0823	62.064	50.827 _CGPPPAIPNAIPASDVN(de)RTDFESHTTIK_	CGPPPAIPN(0.008)AIPASDVN(0.99	CGPPPAIPN(-21.08)AIPASDVN(21	17	3	1003	1
IPI00626662	389	RWGNKGFFVQPTVFSNVTDEMRIAKEEIFGP	1	135.235	135.23	135.23 _GFFVQPTVFSN(de)VTDEM(ox)R_	GFFVQPTVFSN(1)VTDEMR	GFFVQPTVFSN(135.23)VTDEMR	11	3	664	1
IPI00131173	342	PNRRIKSVGIPVICINATDDVFSPSHAIPIE	1	117.208	117.21	117.21 _SVGIPVICIN(de)ATDDVFSPSHAIPIETAK_	SVGIPVICIN(1)ATDDVFSPSHAIPIET	SVGIPVICIN(117.21)ATDDVFSPSH	10	3	985	1
IPI00975193	430	SNGAICHFPFIYNNRNYTDCTSEGRRDNMKW	0.509023	0	110.9	110.9 _GGNSN(de)GAICHFPFIYN(de)N(de)RNYTDCTSEGR_	GGN(0.026)SN(0.974)GAICHFPFIY	GGN(-15.76)SN(15.76)GAICHFPF	19	3	1075	1
IPI00341285	310	NFYYMEGAKYAIICGNRTDAGQCPEGYVCVK	1	123.633	123.63	123.63 _YAIICGN(de)RTDAGQCPEGYVCVK_	YAIICGN(1)RTDAGQCPEGYVCVK	YAIICGN(123.63)RTDAGQCPEGYVC	7	3	845	1
IPI00453489	259	GYQCECPRGIVISEDNHTCQVPVICKSSAIE	1	68.9194	68.919	68.919 _GIVISEDN(de)HTCQVPVICK_	GIVISEDN(1)HTCQVPVICK	GIVISEDN(68.92)HTCQVPVICK	8	3	691	1
IPI00880302	462	KIPNNIKYDPKAIIANITCKKPDQHFKPYMK	1	107.788	107.79	107.79 _AIIAN(de)ITCK_	AIIAN(1)ITCK	AIIAN(107.79)ITCK	5	2	503	1
IPI00975193	542	VNDTFHKRHEEGHMINCTCFGQGRGRWKCDP	1	62.8132	88.561	62.813 _HEEGHM(ox)IN(de)CTCFGQGR_	HEEGHMIN(1)CTCFGQGR	HEEGHMIN(62.81)CTCFGQGR	8	2	975	1
IPI00828222	2689	MDMRRSICYRNYYADNQTCDGEIIFNMTKKM	1	75.4519	102.78	102.78 _NYYADN(de)QTCDGEIIFN(de)M(ox)TKK_	NYYADN(1)QTCDGEIIFN(1)MTKK	N(-75.45)YYADN(75.45)QTCDGEI	6	3	815	1
IPI00226346	56	TAAIPNHRCWAPIIDNDTASDNGSRIISQDD	1	94.5445	94.544	94.544 _CWAPIIDN(de)DTASDN(de)GSR_	CWAPIIDN(1)DTASDN(1)GSR	CWAPIIDN(94.54)DTASDN(94.54)	8	2	947	1
IPI00129631	106	KVCDAGKAIVAVDPGNHTAPRRCACTAGYHW	1	77.9117	77.912	77.912 _AIVAVDPGN(de)HTAPR_	AIVAVDPGN(1)HTAPR	AIVAVDPGN(77.91)HTAPR	9	2	710	1
IPI00115793	519	IVDSVRSVCEDEPYVNVTAEDIDGAQNSAPF	0.990136	20.0163	51.975	51.975 _SVCEDEPYVN(de)VTAEDIDGAQNSAPFSFSIIDQPPGTAQK_	SVCEDEPYVN(0.99)VTAEDIDGAQN(0.	SVCEDEPYVN(20.02)VTAEDIDGAQN	10	4	1050	1
IPI00128904	89	MIIDKIEEDINSSMTNSTAASRPPVTIRIVV	0.773722	5.33943	68.97	68.97 _AFAM(ox)IIDKIEEDINSSM(ox)TN(de)STAASRPPVTIR_	AFAMIIDKIEEDIN(0.226)SSMTN(0.	AFAMIIDKIEEDIN(-5.34)SSMTN(5	19	4	854	1
IPI00116913	248	GEIVVSIVNGRPGAINFSYSPIIRDFTKATN	1	54.2998	54.3	54.3 _IVPIEN(de)GEIVVSIVN(de)GRPGAIN(de)FSYSPIIR_	IVPIEN(1)GEIVVSIVN(1)GRPGAIN(	IVPIEN(54.3)GEIVVSIVN(54.3)G	22	3	1076	1
IPI00331318	213	NYSIFIADINQERCVNESYKKNIMAIKKFVM	1	128.383	128.38	128.38 _CVN(de)ESYKK_	CVN(1)ESYKK	CVN(128.38)ESYKK	3	2	515	1
IPI00626793	895	PSMMRHIVNISIYAFNKSYDYQSVCDPGAAP	1	107.088	107.09	107.09 _HIVN(de)ISIYAFN(de)K_	HIVN(1)ISIYAFN(1)K	HIVN(107.09)ISIYAFN(107.09)K	11	2	711	1
IPI00284816	947	PGVPGIPGEPGRFGINGSYAPGPAGIPGVPG	1	117.258	117.26	117.26 _FGIN(de)GSYAPGPAGIPGVPGK_	FGIN(1)GSYAPGPAGIPGVPGK	FGIN(117.26)GSYAPGPAGIPGVPGK	4	2	929	1
IPI00975193	1243	QSSCTFENINPGIEYNVSVYTVKDDKESAPI	0.984566	19.7955	68.916	45.38 _ITTTPTN(de)GQQGTSIEEVVHADQSSCTFENINPGIEYN(de)VSVYTVK_	ITTTPTN(0.991)GQQGTSIEEVVHADQ	ITTTPTN(22.38)GQQGTSIEEVVHAD	37	4	1208	1
IPI00857130	11	_____RFTAPDTIFANGSVYPPNEGFCPCRE	0.999999	62.1844	176.65	158.58 _FTAPDTIFAN(de)GSVYPPNEGFCPCR_	FTAPDTIFAN(1)GSVYPPNEGFCPCR	FTAPDTIFAN(62.18)GSVYPPN(-62	10	4	680	1
IPI00798576	2793	IPSRTEPMRVPIHCTNGSVVYHEVINAMQCR	0.953643	13.1327	67.644	67.644 _VPIHCTN(de)GSVVYHEVINAMQCR_	VPIHCTN(0.954)GSVVYHEVIN(0.04	VPIHCTN(13.13)GSVVYHEVIN(-13	7	3	862	1
IPI00320605	644	SCAECIKFDKGPFEKNCSVQCAGMTIQTIPI	1	84.3274	84.327	84.327 _FDKGPFEKN(de)CSVQCAGM(ox)TIQTIPIK_	FDKGPFEKN(1)CSVQCAGMTIQTIPIK	FDKGPFEKN(84.33)CSVQCAGMTIQT	9	3	963	1
IPI00322497	129	TYTIQGIIGCEIASDNSSVPTAVFAINGEEF	1	71.1175	71.117	71.117 _GTYTIQGIIGCEIASDN(de)SSVPTAVFAIN(de)GEEFM(ox)K_	GTYTIQGIIGCEIASDN(1)SSVPTAVFA	GTYTIQGIIGCEIASDN(71.12)SSVP	17	4	910	1
IPI00410796	144	GFSTPCAFVVAVSGANGSVIWERPVAQDVAI	1	88.2948	147.25	88.295 _SCADEGFSTPCAFVVAVSGAN(de)GSVIWERPVAQDVAIVK_	SCADEGFSTPCAFVVAVSGAN(1)GSVIW	SCADEGFSTPCAFVVAVSGAN(88.29)	21	4	999	1
IPI00310567	323	NIISEKPSVTTVYINNGSVIQGSSVATIYDR	0.787584	5.69944	55.587	55.587 _IQNIISEKPSVTTVYINN(de)GSVIQGSSVATIYDRK_	IQNIISEKPSVTTVYIN(0.212)N(0.7	IQN(-32.86)IISEKPSVTTVYIN(-5	18	4	925	1
IPI00626485	636	TKCDAGKICRNFQCVNASVINYDCDIQGKCH	0.999981	47.3042	265.22	265.22 _NFQCVN(de)ASVINYDCDIQGK_	NFQCVN(1)ASVINYDCDIQGK	N(-97.14)FQCVN(47.3)ASVIN(-4	6	2	1123	1
IPI00396759	461	GVVIKVIAKTSPFSINDSVIIEEIEAYNPAK	0.999999	61.52	98.443	98.443 _TSPFSIN(de)DSVIIEEIEAYNPAK_	TSPFSIN(1)DSVIIEEIEAYNPAK	TSPFSIN(61.52)DSVIIEEIEAYN(-	7	3	813	1
IPI00336218	465	FFRSGNIEHPEDKIFNTSVEVIPFDNPQSEK	0.862331	8.00009	71.085	71.085 _SGNIEHPEDKIFN(de)TSVEVIPFDNPQSEKEAIQEGR_	SGN(0.137)IEHPEDKIFN(0.862)TS	SGN(-8)IEHPEDKIFN(8)TSVEVIPF	13	4	989	1
IPI00133608	171	EAIPRYEPIPFSASMNESTPTGITDHIAQGP	1	73.8085	116.34	116.34 _YEPIPFSASM(ox)N(de)ESTPTGITDHIAQGPEPNIEESPK_	YEPIPFSASMN(1)ESTPTGITDHIAQGP	YEPIPFSASMN(73.81)ESTPTGITDH	11	4	951	1
IPI00776189	339	SYSRFYESGNSIRAENQSTNIPGPGRNIPNS	0.885315	6.5757	66.045	66.045 _AEN(de)QSTN(de)IPGPGRNIPN(de)SQM(ox)VNIR_	AEN(0.885)QSTN(0.885)IPGPGRN(	AEN(6.58)QSTN(6.58)IPGPGRN(-	3	3	876	1
IPI00785371	776	TWREQTVSDPFIVVSNTSTFVPYEIKVQAVN	1	60.2719	60.272	60.272 _EQTVSDPFIVVSN(de)TSTFVPYEIK_	EQTVSDPFIVVSN(1)TSTFVPYEIK	EQTVSDPFIVVSN(60.27)TSTFVPYE	13	3	868	1
IPI00377953	586	CGECKCHAGYIGDNCNCSTDVSTCRAKDGQI	0.802727	6.09501	101.5	101.5 _CHAGYIGDNCN(de)CSTDVSTCR_	CHAGYIGDN(0.197)CN(0.803)CSTD	CHAGYIGDN(-6.1)CN(6.1)CSTDVS	11	3	783	1
IPI00116429	179	DTDVNCVPCKPGHFQNTSSPRARCQPHTRCE	0.98976	19.8523	79.286	79.286 _IVICQPGTEAEVTDEIM(ox)DTDVNCVPCKPGHFQN(de)TSSPR_	IVICQPGTEAEVTDEIMDTDVN(0.01)C	IVICQPGTEAEVTDEIMDTDVN(-19.8	33	4	1080	1
IPI00320420	144	RSGSGIVGQQIEEFINQSSPFYFWMNGDRID	1	80.1022	80.102	80.102 _SGSGIVGQQIEEFIN(de)QSSPFYFWM(ox)N(de)GDR_	SGSGIVGQQIEEFIN(1)QSSPFYFWMN(	SGSGIVGQQIEEFIN(80.1)QSSPFYF	15	4	804	1
IPI00648288	729	SMMCIDRKCIQIQAINMSSCPIDSRGKVCSG	1	99.0555	134.25	99.055 _KCIQIQAIN(de)M(ox)SSCPIDSR_	KCIQIQAIN(1)MSSCPIDSR	KCIQIQAIN(99.06)MSSCPIDSR	9	3	713	1
IPI00315155	710	NIEGFERIVCTQKKENESRVAICEIGNPMKK	1	96.0589	107.06	96.059 _IVCTQKKEN(de)ESR_	IVCTQKKEN(1)ESR	IVCTQKKEN(96.06)ESR	9	2	747	1
IPI00228567	278	VRFTPDGKYIIVAQINISRISVIIAPPSGSI	1	113.472	113.47	113.47 _YIIVAQIN(de)ISR_	YIIVAQIN(1)ISR	YIIVAQIN(113.47)ISR	8	2	646	1
IPI00226346	62	HRCWAPIIDNDTASDNGSRIISQDDIIRISI	1	94.5445	94.544	94.544 _CWAPIIDN(de)DTASDN(de)GSR_	CWAPIIDN(1)DTASDN(1)GSR	CWAPIIDN(94.54)DTASDN(94.54)	14	2	947	1
IPI00626793	998	FKNDTKSFSGIIDCGNCSRIFHVEKIMNTNI	1	152.163	152.16	152.16 _SFSGIIDCGN(de)CSR_	SFSGIIDCGN(1)CSR	SFSGIIDCGN(152.16)CSR	10	2	737	1
IPI00124954	887	ERVKAIESAIKEAKENASRDRKRYQQEVDRI	1	118.495	118.5	118.5 _EAKEN(de)ASR_	EAKEN(1)ASR	EAKEN(118.5)ASR	5	2	453	1
IPI00118333	1057	YGFKVVNCQEFAISANASRDIVIIFTPDFTA	1	88.6585	104.44	104.44 _VVNCQEFAISAN(de)ASR_	VVNCQEFAISAN(1)ASR	VVN(-88.66)CQEFAISAN(88.66)A	12	2	834	1
IPI00113057	215	GCPMDIFQHSAFADINVSQVITPDAFVCRTI	1	44.021	50.322	44.021 _SCAISEIGCPMDIFQHSAFADIN(de)VSQVITPDAFVCR_	SCAISEIGCPMDIFQHSAFADIN(1)VSQ	SCAISEIGCPMDIFQHSAFADIN(44.0	23	3	1353	1
IPI00987265	2107	ADICQKGHGGCSKHANCSQVGTVVTCTCIPD	1	86.0796	86.08	86.08 _HAN(de)CSQVGTVVTCTCIPDYEGDGWSCR_	HAN(1)CSQVGTVVTCTCIPDYEGDGWSC	HAN(86.08)CSQVGTVVTCTCIPDYEG	3	3	1044	1
IPI00115976	87	PGRDGVSVIVGAPKANTSQPGVIQGGAVYVC	1	83.9388	83.939	83.939 _AN(de)TSQPGVIQGGAVYVCPWGTSPIQCTTIQFDSK_	AN(1)TSQPGVIQGGAVYVCPWGTSPIQC	AN(83.94)TSQPGVIQGGAVYVCPWGT	2	4	918	1
IPI00128432	331	KFIIEAKDIRNEIQVNTSQIPDTVTHYSEIY	0.992845	21.4227	66.378	66.378 _NEIQVN(de)TSQIPDTVTHYSEIYHDFTTTQVGM(ox)HDIVR_	N(0.007)EIQVN(0.993)TSQIPDTVT	N(-21.42)EIQVN(21.42)TSQIPDT	6	4	1052	1
IPI00114236	88	SEGAVGRSIQPIYRKNSSQIAFIIYNDQPPK	0.999999	61.6739	118.2	118.2 _KN(de)SSQIAFIIYNDQPPK_	KN(1)SSQIAFIIYNDQPPK	KN(61.67)SSQIAFIIYN(-61.67)D	2	3	655	1
IPI00116228	149	VGPGVISMANAGPNTNGSQFFICTIKTDWID	0.999092	30.4172	124.81	124.81 _HVGPGVISM(ox)ANAGPNTN(de)GSQFFICTIK_	HVGPGVISMANAGPN(0.001)TN(0.99	HVGPGVISMAN(-86.06)AGPN(-30.	17	3	945	1
IPI00266426	159	SAVDKIQQEFHCCGSNNSQDWQDSEWIRSGE	0.5	0	76.015	76.015 _IQQEFHCCGSN(de)NSQDWQDSEWIR_	IQQEFHCCGSN(0.5)N(0.5)SQDWQDS	IQQEFHCCGSN(0)N(0)SQDWQDSEWI	11	3	976	1
IPI00420835	731	IRAFPEKQISCVANQNGSQADCEIGNPFKRN	0.866439	8.1673	114.55	114.55 _QISCVANQN(de)GSQADCEIGNPFKR_	QISCVAN(0.132)QN(0.866)GSQADC	QISCVAN(-8.17)QN(8.17)GSQADC	9	3	865	1
IPI00132474	363	IKNIIPKSAVGTISGNSSNVIQIIIDAYNSI	0.5	0	109.27	109.27 _SAVGTISGN(de)SSNVIQIIIDAYNSISSEVIIENSKIPDGVTINYK_	SAVGTISGN(0.5)SSN(0.5)VIQIIID	SAVGTISGN(0)SSN(0)VIQIIIDAYN	9	5	926	1
IPI00884541	272	IHFPDIAVFPRIIYINVSNNIIQIPAGIPRG	0.890539	9.24107	140.96	140.96 _IIYIN(de)VSNNIIQIPAGIPR_	IIYIN(0.891)VSN(0.106)N(0.003)	IIYIN(9.24)VSN(-9.24)N(-24.1	5	3	704	1
IPI00122293	285	NYNKISDRGIPKNSFNISNIIVIHISHNKIS	0.866505	8.24592	78.505	78.505 _NSFN(de)ISNIIVIHISHNK_	N(0.004)SFN(0.867)ISN(0.13)II	N(-23.69)SFN(8.25)ISN(-8.25)	4	3	651	1
IPI00128432	512	YDSTTIVSKSPMQWDNSSNAGFTEANHTWIP	0.636289	0	44.021	44.021 _SPM(ox)QWDN(de)SSN(de)AGFTEANHTWIPTNSDYHTVNVDVQK_	SPMQWDN(0.636)SSN(0.636)AGFTE	SPMQWDN(0)SSN(0)AGFTEAN(0)HT	7	4	1028	1
IPI00454124	746	FNIRMGFNTINSIISNNSKQTSHAITIQKTM	0.666667	0	115.06	115.06 _MGFN(de)TIN(de)SIISN(de)NSK_	MGFN(1)TIN(0.667)SIISN(0.667)	MGFN(78.87)TIN(0)SIISN(0)N(0	12	2	822	1
IPI00308971	1423	AGTEPCPPEAAVCIINGSKPVNIGKVRDGPQ	0.993716	21.9904	119.34	119.34 _SISPHAGTEPCPPEAAVCIIN(de)GSKPVNIGK_	SISPHAGTEPCPPEAAVCIIN(0.994)G	SISPHAGTEPCPPEAAVCIIN(21.99)	21	3	1035	1
IPI00127447	224	NDGEYVFITGEDNYINFSKIVEWNGKTSIDW	0.95039	12.8234	155.86	155.86 _N(de)GTNDGEYVFITGEDNYIN(de)FSK_	N(0.5)GTN(0.5)DGEYVFITGEDN(0.	N(0)GTN(0)DGEYVFITGEDN(-12.8	19	3	834	1
IPI00119809	362	RFPMMIPQEIFEIQFNISIYQDHQAIFQRKT	1	82.4065	85.35	82.406 _FPM(ox)M(ox)IPQEIFEIQFN(de)ISIYQDHQAIFQR_	FPMMIPQEIFEIQFN(1)ISIYQDHQAIF	FPMMIPQEIFEIQFN(82.41)ISIYQD	15	4	880	1
IPI00626793	888	RFFGEIDPSMMRHIVNISIYAFNKSYDYQSV	1	107.088	107.09	107.09 _HIVN(de)ISIYAFN(de)K_	HIVN(1)ISIYAFN(1)K	HIVN(107.09)ISIYAFN(107.09)K	4	2	711	1
IPI00850930	341	DHVIAKEIETKMNIQNISIVISPTVQEFIIN	1	60.7469	72.771	60.747 _MN(de)IQN(de)ISIVISPTVQEFIIN(de)CIHR_	MN(1)IQN(1)ISIVISPTVQEFIIN(1)	MN(60.75)IQN(60.75)ISIVISPTV	5	3	948	1
IPI00989096	251	IREIQAPIGQSFCCGNASIVISPAVHIDIIS	1	89.9791	184.76	89.979 _EIQAPIGQSFCCGN(de)ASIVISPAVHIDIISIR_	EIQAPIGQSFCCGN(1)ASIVISPAVHID	EIQAPIGQSFCCGN(89.98)ASIVISP	14	4	842	1
IPI00129613	424	IKTSAAIDRETMPEYNISITARDSGIPSISA	1	80.3118	80.312	80.312 _ETM(ox)PEYN(de)ISITAR_	ETMPEYN(1)ISITAR	ETMPEYN(80.31)ISITAR	7	2	771	1
IPI00109727	118	YFCEIQVSGANPMSSNKSISVYRDKIVKCGG	0.999304	31.5687	97.94	97.94 _VITIAN(de)FTTKDEGDYFCEIQVSGANPM(ox)SSN(de)K_	VITIAN(1)FTTKDEGDYFCEIQVSGAN(	VITIAN(63.57)FTTKDEGDYFCEIQV	30	4	864	1
IPI00406603	1460	DITHIPPPSPSPTINNTSISRKFNPIVVVGI	1	65.4382	88.09	65.438 _TIPHPDITHIPPPSPSPTIN(de)N(de)TSISR_	TIPHPDITHIPPPSPSPTIN(1)N(1)TS	TIPHPDITHIPPPSPSPTIN(65.44)N	21	3	931	1
IPI00467600	2375	ISGTIFVPQNSGIPKNKSISGRDIEHHITNV	0.681874	3.31105	100.39	100.39 _HITDISISGTIFVPQNSGIPKN(de)K_	HITDISISGTIFVPQN(0.318)SGIPKN	HITDISISGTIFVPQN(-3.31)SGIPK	22	3	823	1
IPI00406901	141	TIDKKEVTEGGVVTVNCSIQEEKPPIFFKIE	1	93.8044	139.81	93.804 _EVTEGGVVTVN(de)CSIQEEKPPIFFKIEK_	EVTEGGVVTVN(1)CSIQEEKPPIFFKIE	EVTEGGVVTVN(93.8)CSIQEEKPPIF	11	3	1027	1
IPI00762273	411	EKIWKRGTTFIEKHINSSIPRPQPAIIIITA	1	83.3102	148.95	83.31 _HIN(de)SSIPRPQPAIIIITAAQDAAEVIK_	HIN(1)SSIPRPQPAIIIITAAQDAAEVI	HIN(83.31)SSIPRPQPAIIIITAAQD	3	4	718	1
IPI00471273	73	CCINQKGTIIGIDIQNCSIKDPGPNFIQAYT	1	179.789	179.79	179.79 _GTIIGIDIQN(de)CSIK_	GTIIGIDIQN(1)CSIK	GTIIGIDIQN(179.79)CSIK	10	2	767	1
IPI00337976	544	FRIPDNRIGRKFSIMNQSIISIPGSPFISRH	1	69.4849	69.485	69.485 _FSIM(ox)N(de)Q(de)SIISIPGSPFISR_	FSIMN(1)QSIISIPGSPFISR	FSIMN(69.48)QSIISIPGSPFISR	5	3	705	1
IPI00311893	170	HCRRCIIVGNGGVIANKSIGSRIDDYDIVIR	1	125.97	125.97	125.97 _CIIVGN(de)GGVIAN(de)K_	CIIVGN(1)GGVIAN(1)K	CIIVGN(125.97)GGVIAN(125.97)	12	2	659	1
IPI00172354	102	RFHGRVGFTGTMPATNVSIFINNTQISDTGT	0.99949	32.0386	98.94	98.94 _VGFTGTM(ox)PATN(de)VSIFIN(de)NTQISDTGTYQCIVNNIPDR_	VGFTGTMPATN(0.999)VSIFIN(0.81	VGFTGTMPATN(32.04)VSIFIN(6.4	11	4	1020	1
IPI00129240	168	EICSGKPFDAFTDIKNGSIFAFRGQYCYEID	1	151.441	151.44	151.44 _N(de)GSIFAFR_	N(1)GSIFAFR	N(151.44)GSIFAFR	1	2	457	1
IPI00453798	261	GDTGKIVAIAVIDEKNTSIEHTRIKSIIQEV	1	136.395	136.39	136.39 _IVAIAVIDEKN(de)TSIEHTR_	IVAIAVIDEKN(1)TSIEHTR	IVAIAVIDEKN(136.39)TSIEHTR	11	3	671	1
IPI00129867	223	MPPRIPANTMQIFIDNNSIEGIPENYFNVIP	0.480537	0	57.803	57.803 _NMPPRIPANTM(ox)QIFIDN(de)NSIEGIPENYFNVIPK_	N(0.002)MPPRIPAN(0.024)TMQIFI	N(-26.03)MPPRIPAN(-13.06)TMQ	17	4	952	1
IPI00127447	325	GFCIPEGNCMDSGVINISICKNGAPIIMSFP	1	53.0914	75.261	53.091 _YKVPAEIIAN(de)TSEN(de)AGFCIPEGN(de)CMDSGVIN(de)ISICK_	YKVPAEIIAN(1)TSEN(1)AGFCIPEGN	YKVPAEIIAN(53.09)TSEN(53.09)	31	4	995	1
IPI00123783	869	IGSDSTHVIPGESPFNKSIASVIRGQVITAD	1	67.6454	67.645	67.645 _ISFIIGSDSTHVIPGESPFN(de)K_	ISFIIGSDSTHVIPGESPFN(1)K	ISFIIGSDSTHVIPGESPFN(67.65)K	20	3	749	1
IPI00125549	190	FRDTANIHGKITCFNNFSIAAPESSPHPAHS	0.5	0	92.41	92.41 _ITCFN(de)NFSIAAPESSPHPAHSQVVSTGYSR_	ITCFN(0.5)N(0.5)FSIAAPESSPHPA	ITCFN(0)N(0)FSIAAPESSPHPAHSQ	6	3	1088	1
IPI00122705	191	ANGHPPGRISWPSNVNGSHREMKEPGSQPGT	0.890024	9.08103	84.169	84.169 _ISWPSNVN(de)GSHR_	ISWPSN(0.11)VN(0.89)GSHR	ISWPSN(-9.08)VN(9.08)GSHR	8	2	678	1
IPI00128334	233	CVPVIRCNTAAPMWINGSHPSSSEGIVSRTA	0.999845	38.0966	59.339	59.339 _CNTAAPM(ox)WIN(de)GSHPSSSEGIVSR_	CNTAAPMWIN(1)GSHPSSSEGIVSR	CN(-38.1)TAAPMWIN(38.1)GSHPS	10	3	826	1
IPI00471187	331	IASDNTIHFVHPITVNYSGVYVCKVSNSIGQ	0.969001	14.9498	49.525	49.525 _IDGQWPDGIIASDNTIHFVHPITVN(de)YSGVYVCK_	IDGQWPDGIIASDN(0.031)TIHFVHPI	IDGQWPDGIIASDN(-14.95)TIHFVH	25	4	930	1
IPI00226430	249	KIPSVFKKDGTVTAGNASGVSDGAGAVIIAS	1	72.3025	72.302	72.302 _DGTVTAGN(de)ASGVSDGAGAVIIASEDAVK_	DGTVTAGN(1)ASGVSDGAGAVIIASEDA	DGTVTAGN(72.3)ASGVSDGAGAVIIA	8	3	845	1
IPI00848991	100	MKIFQISIRNIRQYYNSSGTQSQRADGFRQQ	1	79.492	79.492	79.492 _QYYN(de)SSGTQSQR_	QYYN(1)SSGTQSQR	QYYN(79.49)SSGTQSQR	4	2	710	1
IPI00322026	393	GERAGDMDFTMIRIVNGSGPHQGRVEVFHDR	1	81.5247	81.525	81.525 _IVN(de)GSGPHQGR_	IVN(1)GSGPHQGR	IVN(81.52)GSGPHQGR	3	2	562	1
IPI00153416	244	FAIGNEINSYRIFIGNYSGNVGKDAIIYHNN	0.995178	23.1472	93.011	93.011 _IFIGN(de)YSGNVGK_	IFIGN(0.995)YSGN(0.005)VGK	IFIGN(23.15)YSGN(-23.15)VGK	5	2	635	1
IPI00988164	452	VKVGAFFGIFNVAVMNISGNCIRSIPEHVFQ	0.988708	19.6738	152.32	152.32 _VGAFFGIFNVAVM(ox)N(de)ISGNCIR_	VGAFFGIFN(0.011)VAVMN(0.989)I	VGAFFGIFN(-19.67)VAVMN(19.67	14	3	768	1
IPI00417180	292	CIQGKPISKEFEAYVNASGEHGIVVFSIGSM	1	82.6653	82.665	82.665 _EFEAYVN(de)ASGEHGIVVFSIGSM(ox)VSEIPEKK_	EFEAYVN(1)ASGEHGIVVFSIGSMVSEI	EFEAYVN(82.67)ASGEHGIVVFSIGS	7	4	818	1
IPI00653675	180	PPEYFIHDDMRNCGVNCSGDVFTAIIGEISS	0.5	0	85.595	85.595 _N(de)CGVNCSGDVFTAIIGEISSPNYPNPYPENSR_	N(0.5)CGVN(0.5)CSGDVFTAIIGEIS	N(0)CGVN(0)CSGDVFTAIIGEISSPN	5	4	883	1
IPI00356462	415	ISEDIESTRQAVIQVNQSFVSSTAQKDFAFM	1	50.305	75.018	50.305 _(gl)Q(de)AVIQ(de)VN(de)Q(de)SFVSSTAQ(de)K_	QAVIQVN(1)QSFVSSTAQK	QAVIQVN(50.3)QSFVSSTAQK	7	3	608	1

IPI00330833	1119	ICNSIIWIVEKCQIENGSFKENSQYIPIKIQ	0.978984	16.6822	79.81	79.81	_CQIEN(de)GSFKENSQYIPIK_	CQIEN(0.979)GSFKEN(0.021)SQYI	CQIEN(16.68)GSFKEN(-16.68)SQ	5	3	719		1
IPI00118333	166	FHASFFQNRKIIPGGNTSFDVVFIARVVGNV	1	102.277	102.28	102.28	_IIPGGN(de)TSFDVVFIAR_	IIPGGN(1)TSFDVVFIAR	IIPGGN(102.28)TSFDVVFIAR	6	3	570		1
IPI00128358	700	RTWSPPFESDDSQKHNQSEYDDSASECCSCP	1	71.8823	71.882	71.882	_TWSPPFESDDSQKHN(de)QSEYDDSASECCSCPK_	TWSPPFESDDSQKHN(1)QSEYDDSASEC	TWSPPFESDDSQKHN(71.88)QSEYDD	15	4	921		1
IPI00469387	55	PIPQRPISPIHPIGCNDSEVIAVAGFAIQNI	0.999901	41.2812	79.917	79.917	_SPPAPPIPQRPISPIHPIGCN(de)DSEVIAVAGFAIQNINR_	SPPAPPIPQRPISPIHPIGCN(1)DSEVI	SPPAPPIPQRPISPIHPIGCN(41.28)	21	4	1012		1
IPI01008676	437	YYIIKGNKTITCRNGNWSEPPMCIHACVIPE	0.998535	31.282	53.998	53.998	_N(de)GN(de)WSEPPMCIHACVIPENIMEAHNIIIK_	N(0.999)GN(0.999)WSEPPMCIHACV	N(31.28)GN(31.28)WSEPPMCIHAC	3	4	848		1
IPI00132279	185	MQYSRDSSGVINVMINGSEPKGAYPTRGFFA	0.964168	14.2988	69.485	69.485	_DSSGVINVM(ox)IN(de)GSEPK_	DSSGVIN(0.036)VMIN(0.964)GSEP	DSSGVIN(-14.3)VMIN(14.3)GSEP	11	3	555		1
IPI00115892	253	PNGTEVPHTKSRGRHNCSEPIDMEDISSGIG	1	158.437	158.44	158.44	_GRHN(de)CSEPIDM(ox)EDISSGIGVTR_	GRHN(1)CSEPIDMEDISSGIGVTR	GRHN(158.44)CSEPIDMEDISSGIGV	4	3	816		1
IPI00223446	333	ISERENQYTIRKIQINNSENTIRSIIPDVEG	0.848718	7.63136	96.82	96.82	_KIQIN(de)NSENTIR_	KIQIN(0.849)N(0.146)SEN(0.005)	KIQIN(7.63)N(-7.63)SEN(-22.4	5	2	716		1
IPI00127447	304	GIPAFRYKVPAEIIANTSENAGFCIPEGNCM	1	53.0914	152.13	53.091	_YKVPAEIIAN(de)TSEN(de)AGFCIPEGN(de)CMDSGVIN(de)ISICK_	YKVPAEIIAN(1)TSEN(1)AGFCIPEGN	YKVPAEIIAN(53.09)TSEN(53.09)	10	4	995		1
IPI00652605	32	IFCIFGPKKNPEAHMNVSEIIKHWDYPSEEY	0.999914	40.6444	86.803	86.803	_KNPEAHM(ox)N(de)VSEIIK_	KNPEAHMN(1)VSEIIK	KN(-40.64)PEAHMN(40.64)VSEII	8	3	543		1
IPI00466999	374	SIQIEEPEWEKHRSVNISEIIDVYSDGVEII	1	198.487	198.49	198.49	_SVN(de)ISEIIDVYSDGVEIIQIVK_	SVN(1)ISEIIDVYSDGVEIIQIVK	SVN(198.49)ISEIIDVYSDGVEIIQI	3	3	812		1
IPI00125747	128	PCPERIPSMKGPIDINMSEIAMDDIHEIFSR	1	49.8712	61.345	49.871	_GPIDIN(de)M(ox)SEIAM(ox)DDIHEIFSRDPAIK_	GPIDIN(1)MSEIAMDDIHEIFSRDPAIK	GPIDIN(49.87)MSEIAMDDIHEIFSR	6	4	741		1
IPI00129240	241	YWRFEDGVIDPGYPRNISEGFSGIPDNVDAA	1	70.297	107.41	107.41	_N(de)ISEGFSGIPDNVDAAFAIPAHR_	N(1)ISEGFSGIPDNVDAAFAIPAHR	N(70.3)ISEGFSGIPDN(-70.3)VDA	1	3	800		1
IPI00314132	135	INDNAPKFSFEIKTFNVSEDTKVNTTVIPET	1	96.3913	96.391	96.391	_TFN(de)VSEDTKVN(de)TTVIPETQIK_	TFN(1)VSEDTKVN(1)TTVIPETQIK	TFN(96.39)VSEDTKVN(96.39)TTV	3	3	789		1
IPI00153644	455	IQDIENQGINTGRSPNDSEAWHQKKPHTQSP	1	69.7206	81.625	69.721	_SPN(de)DSEAWHQK_	SPN(1)DSEAWHQK	SPN(69.72)DSEAWHQK	3	3	434		1
IPI00117841	246	QRAEVIAPPTITVECNGSEAHARWVARNRFH	1	120.106	120.11	120.11	_AEVIAPPTITVECN(de)GSEAHAR_	AEVIAPPTITVECN(1)GSEAHAR	AEVIAPPTITVECN(120.11)GSEAHA	14	3	742		1
IPI00273133	690	IEAPRITQNITDIIVNVSDSIEMRCPVAGAH	1	85.6359	85.636	85.636	_ITQN(de)ITDIIVN(de)VSDSIEM(ox)R_	ITQN(1)ITDIIVN(1)VSDSIEMR	ITQN(85.64)ITDIIVN(85.64)VSD	11	3	727		1
IPI00134390	121	HRMCQIYPETFMCVTNSSDIIQAFRRGKVAS	1	73.21	73.21	73.21	_M(ox)CQIYPETFM(ox)CVTN(de)SSDIIQAFR_	MCQIYPETFMCVTN(1)SSDIIQAFR	MCQIYPETFMCVTN(73.21)SSDIIQA	14	3	949		1
IPI00453492	889	IIFRAIEISTVTCQINCSDHGHCDSFTKRCV	1	59.8566	78.794	59.857	_AIEISTVTCQIN(de)CSDHGHCDSFTKR_	AIEISTVTCQIN(1)CSDHGHCDSFTKR	AIEISTVTCQIN(59.86)CSDHGHCDS	12	4	735		1
IPI00987265	746	TQCPGGFSNPCYGKGNCSDGVRGNGACICFP	0.790588	5.76948	96.303	96.303	_GFFGPDCTQCPGGFSNPCYGKGN(de)CSDGVR_	GFFGPDCTQCPGGFSN(0.209)PCYGKG	GFFGPDCTQCPGGFSN(-5.77)PCYGK	23	3	1067		1
IPI00856723	70	VTVIEGEVATISCQVNKSDDSVIQIINPNRQ	1	225.677	225.68	225.68	_DVTVIEGEVATISCQVN(de)K_	DVTVIEGEVATISCQVN(1)K	DVTVIEGEVATISCQVN(225.68)K	17	2	982		1
IPI00850337	223	GVNHEKYDNSIKIVSNASCTTNCIAPIAKVI	0.998449	28.086	146.81	146.81	_IVSN(de)ASCTTNCIAPIAK_	IVSN(0.998)ASCTTN(0.002)CIAPI	IVSN(28.09)ASCTTN(-28.09)CIA	4	2	911		1
IPI00130420	606	KNNKFDIRKTKVIIGNESCTITISESTTNTI	1	118.796	128.99	128.99	_VIIGN(de)ESCTITISESTTNTIK_	VIIGN(1)ESCTITISESTTNTIK	VIIGN(118.8)ESCTITISESTTN(-1	5	3	761		1
IPI00453489	291	SVPREIVGGIEIFITNTSCRGVSNGTHVNIV	1	127.834	127.83	127.83	_EIVGGIEIFITN(de)TSCR_	EIVGGIEIFITN(1)TSCR	EIVGGIEIFITN(127.83)TSCR	12	2	905		1
IPI00117424	186	ATATFNSTAIKKDGINFSCQAEIDIRPHGGY	1	88.5651	88.565	88.565	_DGIN(de)FSCQAEIDIRPHGGYIIR_	DGIN(1)FSCQAEIDIRPHGGYIIR	DGIN(88.57)FSCQAEIDIRPHGGYII	4	3	845		1
IPI00224654	138	IIQEEAEEKKIRTIINTSCDNMIMAIKSIKI	0.999986	48.6059	118.33	118.33	_TIIN(de)TSCDNM(ox)IM(ox)AIK_	TIIN(1)TSCDNMIMAIK	TIIN(48.61)TSCDN(-48.61)MIMA	4	2	879		1
IPI00345112	176	NPAKDPVGTCYVAIQNFSAYAEHSPCRNSNA	0.999846	38.1246	84.529	84.529	_TIKPNPAKDPVGTCYVAIQN(de)FSAYAEHSPCR_	TIKPNPAKDPVGTCYVAIQN(1)FSAYAE	TIKPN(-38.12)PAKDPVGTCYVAIQN	20	4	874		1
IPI00119063	2639	RCNQFVDCEDASDEMNCSATDCSSYFRIGVK	0.999999	59.8024	122.41	122.41	_CNQFVDCEDASDEM(ox)N(de)CSATDCSSYFR_	CNQFVDCEDASDEMN(1)CSATDCSSYFR	CN(-59.8)QFVDCEDASDEMN(59.8)	15	3	1062		1
IPI00929801	365	IMPFDSIHDDPCIITNRSARIPCFIAGDMRS	1	78.9316	153.86	78.932	_AIM(ox)PFDSIHDDPCIITN(de)R_	AIMPFDSIHDDPCIITN(1)R	AIMPFDSIHDDPCIITN(78.93)R	17	3	711		1
IPI00122285	64	GAGHIAVSVGIRRVSNESAAPIVPAVPRPEV	1	93.7302	93.73	93.73	_RVSN(de)ESAAPIVPAVPRPEVDN(de)ITIR_	RVSN(1)ESAAPIVPAVPRPEVDN(1)IT	RVSN(93.73)ESAAPIVPAVPRPEVDN	4	3	901		1
IPI00316045	228	GQEANIRSCIISRIENNCTHGEDTWMECEDP	0.5	0	178.71	178.71	_IEN(de)NCTHGEDTWM(ox)ECEDPFEIK_	IEN(0.5)N(0.5)CTHGEDTWMECEDPF	IEN(0)N(0)CTHGEDTWMECEDPFEIK	3	3	924		1
IPI00128484	414	EKVDGAICIDKSIGPNTCSSNGSSIYFIHGP	0.977896	17.9853	90.384	53.522	_SIGPN(de)TCSSN(de)GSSIYFIHGPNIYCYSSIDKINAAK_	SIGPN(0.978)TCSSN(0.97)GSSIYF	SIGPN(17.99)TCSSN(16.42)GSSI	5	4	960		1
IPI00312407	268	YCSHCRGIVTVKPCYNYCSNIMRGCIANQGD	0.999894	39.7407	108.71	108.71	_GIVTVKPCYN(de)YCSNIM(ox)R_	GIVTVKPCYN(1)YCSNIMR	GIVTVKPCYN(39.74)YCSN(-39.74	10	3	698		1
IPI00112549	296	TGNPKGAMITHQNIINDCSGFIKATESAFIA	0.999998	57.25	121.82	121.82	_GAM(ox)ITHQNIIN(de)DCSGFIK_	GAMITHQNIIN(1)DCSGFIK	GAMITHQN(-57.25)IIN(57.25)DC	11	3	679		1
IPI00122592	78	IPVRIKEQVVGIIAQNNCSCESKGGSIPIPF	1	88.9922	195.46	88.992	_IKEQVVGIIAQN(de)N(de)CSCESK_	IKEQVVGIIAQN(1)N(1)CSCESK	IKEQVVGIIAQN(88.99)N(88.99)C	12	3	727		1
IPI00111794	255	TRDERVGQPEIKPMRNECNSEESSCPFRVVP	0.617266	2.07575	66.325	66.325	_VGQPEIKPM(ox)RN(de)ECNSEESSCPFR_	VGQPEIKPMRN(0.617)ECN(0.383)S	VGQPEIKPMRN(2.08)ECN(-2.08)S	11	3	923		1
IPI00139788	494	TAGWNIPMGMIYNRINHCKFDEFFSQGCAPG	1	45.125	141.36	45.125	_IN(de)HCKFDEFFSQGCAPGYEKN(de)STICDICIGPIK_	IN(1)HCKFDEFFSQGCAPGYEKN(1)ST	IN(45.13)HCKFDEFFSQGCAPGYEKN	2	4	978		1
IPI00310916	155	ANNAKAVVKTFHETINCCGSNAITTITTTII	0.732976	4.38539	109.85	109.85	_TFHETIN(de)CCGSNAITTITTTIIR_	TFHETIN(0.733)CCGSN(0.267)AIT	TFHETIN(4.39)CCGSN(-4.39)AIT	7	3	876		1
IPI00223367	93	IAKIRGIRNIETFAMNICEHFISSFNHVTRA	0.713893	3.9711	82.765	82.765	_NIETFAM(ox)N(de)ICEHFISSFNHVTR_	NIETFAMN(0.714)ICEHFISSFN(0.2	N(-53.49)IETFAMN(3.97)ICEHFI	8	3	895		1
IPI00221859	145	DAQISGIASAIRHPANECAPYQFRSDGIPIA	1	81.709	81.709	81.709	_HPAN(de)ECAPYQFR_	HPAN(1)ECAPYQFR	HPAN(81.71)ECAPYQFR	4	2	746		1
IPI00282848	154	QEASEAYIVGIFEDTNICAIHAKRVTIMPKD	1	192.848	192.85	192.85	_FQSSAVM(ox)AIQEASEAYIVGIFEDTN(de)ICAIHAK_	FQSSAVMAIQEASEAYIVGIFEDTN(1)I	FQSSAVMAIQEASEAYIVGIFEDTN(19	25	3	1178		1
IPI00122973	456	AIIPIGVVKSVKQEMNGTYVCHAFSSHGNVT	1	71.735	73.138	71.735	_SVKQEM(ox)N(de)GTYVCHAFSSHGN(de)VTR_	SVKQEMN(1)GTYVCHAFSSHGN(1)VTR	SVKQEMN(71.73)GTYVCHAFSSHGN(	7	3	876	1	
IPI00118674	463	HNRYYQSIYDTAENINVTYPEWQSPEEDINF	0.51613	0.729909	46.415	46.415	_YYQSIYDTAENIN(de)VTYPEWQSPEEDINFVTDTAK_	YYQSIYDTAEN(0.436)IN(0.516)VT	YYQSIYDTAEN(-0.73)IN(0.73)VT	13	4	1012	1	
IPI00134378	103	EMKKQYDSFTFTASRNGTYKFCFSNEFSTFT	1	83.3699	153.22	83.37	_QYDSFTFTASRN(de)GTYK_	QYDSFTFTASRN(1)GTYK	QYDSFTFTASRN(83.37)GTYK	12	2	944	1	
IPI00758066	203	ISIHIFYKKCSWIITNITYFPETVPREIVVP	1	53.5645	82.529	53.565	_CSWIITN(de)ITYFPETVPR_	CSWIITN(1)ITYFPETVPR	CSWIITN(53.56)ITYFPETVPR	7	2	1050	1	
IPI00276430	74	ISVAISATKTEQIPVNKTYAACPQNWIGVEN	1	100.017	100.02	100.02	_TEQIPVN(de)K_	TEQIPVN(1)K	TEQIPVN(100.02)K	7	2	465	1	
IPI00221426	397	QMDGMSIIPIIKGDRNITWRSDVIVEYQGEG	1	99.8021	126.07	99.802	_GDRN(de)ITWR_	GDRN(1)ITWR	GDRN(99.8)ITWR	4	2	510	1	
IPI00762242	57	IGVINAPQKVIEQSYNATWIGRQGPGGPDSI	1	81.9037	81.904	81.904	_VIEQSYN(de)ATWIGR_	VIEQSYN(1)ATWIGR	VIEQSYN(81.9)ATWIGR	7	2	769	1	
IPI00124326	145	SVSDQHGIVYITENKNKTVVIPCRGSISNIN	0.5	0	65.423	65.423	_SPFIASVSDQHGIVYITEN(de)KNK_	SPFIASVSDQHGIVYITEN(0.5)KN(0.	SPFIASVSDQHGIVYITEN(0)KN(0)K	21	3	817	1	
IPI00222967	175	VVQKYWGIHIQAFVQNGTVSKNEQVCEEDQT	1	75.3164	75.316	75.316	_YWGIHIQAFVQN(de)GTVSK_	YWGIHIQAFVQN(1)GTVSK	YWGIHIQAFVQN(75.32)GTVSK	12	3	650	1	
IPI01027336	627	TSRPKMSIVMPAMAPNETVSGRGPPGDYEEM	1	65.231	97.417	65.231	_M(ox)SIVM(ox)PAM(ox)APN(de)ETVSGR_	MSIVMPAMAPN(1)ETVSGR	MSIVMPAMAPN(65.23)ETVSGR	11	2	920	1	
IPI00130573	854	YKITASARGYNPVTKNVTVRSEGAVQVNFTI	1	65.9773	130.1	130.1	_GYNPVTKN(de)VTVR_	GYNPVTKN(1)VTVR	GYN(-65.98)PVTKN(65.98)VTVR	8	2	675	1	
IPI00623114	999	VRIVQQIDFEKKQIYNITVRAKDKGKPVSIS	1	141.782	141.78	141.78	_QIYN(de)ITVR_	QIYN(1)ITVR	QIYN(141.78)ITVR	4	1	1008	1	
IPI00624663	55	YSGVPEKACVSINHVNETVMISITIEYAMQQ	1	76.807	81.48	76.807	_ACVSIN(de)HVN(de)ETVM(ox)ISITIEYAM(ox)QQTK_	ACVSIN(1)HVN(1)ETVMISITIEYAMQ	ACVSIN(76.81)HVN(76.81)ETVMI	9	3	1005	1	
IPI00760106	872	PTTVGVVCRQIGCADNGTVKPIPSDKTPSRP	1	130.215	130.21	130.21	_QIGCADN(de)GTVKPIPSDK_	QIGCADN(1)GTVKPIPSDK	QIGCADN(130.21)GTVKPIPSDK	7	2	901	1	
IPI00229516	705	CVMMFSYTEIPNGRSNITVIREPECGSAPNA	1	105.568	105.57	105.57	_SN(de)ITVIR_	SN(1)ITVIR	SN(105.57)ITVIR	2	2	402	1	
IPI00121776	135	NSVIIPSSRNKSTFQNVTVIIAVITQKDFKD	1	139.582	238.07	139.58	_STFQN(de)VTVIIAVITQK_	STFQN(1)VTVIIAVITQK	STFQN(139.58)VTVIIAVITQK	5	3	588	1	
IPI00762897	269	EYKAKDDTQVKGTEVNATVIGESDPIDEVQG	1	259.6	259.6	259.6	_GTEVN(de)ATVIGESDPIDEVQGFIFGK_	GTEVN(1)ATVIGESDPIDEVQGFIFGK	GTEVN(259.6)ATVIGESDPIDEVQGF	5	3	875	1	
IPI00120832	66	REKIEEHGSMRAFVENITVIENSIVFKFHII	1	151.484	315.66	151.48	_AFVEN(de)ITVIEN(de)SIVFK_	AFVEN(1)ITVIEN(1)SIVFK	AFVEN(151.48)ITVIEN(151.48)S	5	2	913	1	
IPI00111228	612	NCCNTSTVADAYRFINHTVGNKTKVEEGYYY	1	89.8046	89.805	89.805	_FIN(de)HTVGN(de)KTK_	FIN(1)HTVGN(1)KTK	FIN(89.8)HTVGN(89.8)KTK	3	2	631	1	
IPI00133074	263	EDFGGGRFVFMEEGSNKTVEPRAGRVSFFTS	1	92.2652	92.265	92.265	_FVFM(ox)EEGSN(de)KTVEPR_	FVFMEEGSN(1)KTVEPR	FVFMEEGSN(92.27)KTVEPR	9	2	894	1	
IPI00230412	357	YFDEFSFTKITGSAGNYTVCQKDICCHITYK	1	66.3926	93.494	66.393	_ITGSAGN(de)YTVCQK_	ITGSAGN(1)YTVCQK	ITGSAGN(66.39)YTVCQK	7	3	467	1	
IPI00115231	333	SRDQFDICAKTVVVDNETVATRIWTFFCHSP	1	99.9412	99.941	99.941	_TVVVDN(de)ETVATR_	TVVVDN(1)ETVATR	TVVVDN(99.94)ETVATR	6	2	653	1	
IPI00471264	54	YAPKGRNVITNQTINNDTVASSFIKSGKNRI	0.791476	5.80014	119.08	119.08	_NVITN(de)QTINN(de)DTVASSFIK_	N(0.005)VITN(0.995)QTIN(0.209)	N(-23.12)VITN(23.12)QTIN(-5.	10	3	694	1	
IPI00120225	757	DVMQVANTTMSSRSRNTTVADTYNITDPEEF	1	81.9736	81.974	81.974	_SRN(de)TTVADTYN(de)ITDPEEFETEYPFFESR_	SRN(1)TTVADTYN(1)ITDPEEFETEYP	SRN(81.97)TTVADTYN(81.97)ITD	3	3	1121	1	
IPI00119181	314	VMVVITDGDIFGDPINITTVINSPKMQGVVR	0.973414	15.6365	62.589	62.589	_VM(ox)VVITDGDIFGDPIN(de)ITTVINSPK_	VMVVITDGDIFGDPIN(0.973)ITTVIN	VMVVITDGDIFGDPIN(15.64)ITTVI	16	3	892	1	
IPI00356462	810	EKINQINFQNIYQIFNETTSQVNKCQQNMSH	0.999328	32.1591	124.63	124.63	_INQINFQNIYQIFN(de)ETTSQVNK_	INQINFQN(0.001)IYQIFN(0.999)E	IN(-49.1)QIN(-48.33)FQN(-32.	14	3	886	1	
IPI00224294	1095	RKGFDCGIEAKNIGFNCTTSQGKIAIGSIFH	0.999992	50.924	84.892	84.892	_NIGFN(de)CTTSQGK_	NIGFN(1)CTTSQGK	N(-50.92)IGFN(50.92)CTTSQGK	5	2	664	1	
IPI00132602	151	AAQTRKITDVEAQVINQTTRIEIQIIQHSIS	1	124.417	124.42	124.42	_KITDVEAQVIN(de)QTTR_	KITDVEAQVIN(1)QTTR	KITDVEAQVIN(124.42)QTTR	11	3	573	1	
IPI00123976	72	TAIAERCAWSPEEEWNITTPEIHVPAERRGQ	1	126.4	126.4	126.4	_CAWSPEEEWN(de)ITTPEIHVPAER_	CAWSPEEEWN(1)ITTPEIHVPAER	CAWSPEEEWN(126.4)ITTPEIHVPAE	10	3	885	1	
IPI00380441	206	FIFVQATSSPVSSGANATTIRKITIIFKNMQ	0.999858	38.4881	125.54	125.54	_VQGAWPIIDNDFIFVQATSSPVSSGAN(de)ATTIR_	VQGAWPIIDNDFIFVQATSSPVSSGAN(1)	VQGAWPIIDN(-38.49)DFIFVQATSS	27	4	842	1	
IPI00356462	1000	QIKIQKKPNTIKKTVNMTTIIIGRTQRNTDT	1	84.4793	84.479	84.479	_TVN(de)M(ox)TTIIIGR_	TVN(1)MTTIIIGR	TVN(84.48)MTTIIIGR	3	2	618	1	
IPI00111456	178	IGQGVGRIECIHVPANRTTGPPIEVSPINFP	1	79.9739	79.974	79.974	_IECIHVPAN(de)R_	IECIHVPAN(1)R	IECIHVPAN(79.97)R	9	2	605	1	
IPI00855103	2033	DFIPNTQRSPVRTEFNITSYSSKRIMKRHVD	1	64.5504	87.447	64.55	_TEFN(de)ITSYSSKR_	TEFN(1)ITSYSSKR	TEFN(64.55)ITSYSSKR	4	2	717	1	
IPI00229992	1251	ERRIQHGQFKYTSDPNVTSVGPSKSFFSGGR	1	106.424	106.42	106.42	_YTSDPN(de)VTSVGPSK_	YTSDPN(1)VTSVGPSK	YTSDPN(106.42)VTSVGPSK	6	2	727	1	
IPI00469221	2503	NDFIYSDVCRVETGENCTSPAPKEEIIPAEE	1	78.8139	78.814	78.814	_VETGEN(de)CTSPAPK_	VETGEN(1)CTSPAPK	VETGEN(78.81)CTSPAPK	6	2	696	1	
IPI00263041	839	AGSSEGATAIQMVKFNTTSINVGSSYFPEHG	0.989461	19.7262	98.227	98.227	_FN(de)TTSINVGSSYFPEHGYFR_	FN(0.989)TTSIN(0.011)VGSSYFPE	FN(19.73)TTSIN(-19.73)VGSSYF	2	3	775	1	
IPI00128905	144	RSYSSIQQDIFQFQKNQTSIEKKFSYDINQC	1	115.34	132.64	115.34	_SYSSIQQDIFQFQKN(de)QTSIEKK_	SYSSIQQDIFQFQKN(1)QTSIEKK	SYSSIQQDIFQFQKN(115.34)QTSIE	15	3	883	1	
IPI00380296	187	KIHNGSEEAIAQYKMNITSIAPIIEKIAKTS	1	82.0687	82.069	82.069	_M(ox)N(de)ITSIAPIIEK_	MN(1)ITSIAPIIEK	MN(82.07)ITSIAPIIEK	2	2	674	1	
IPI00170355	96	HTIRIIQDFSSDPASNITSHSIEKIPSAAEP	1	56.3664	56.366	56.366	_IIQDFSSDPASN(de)ITSHSIEKIPSAAEPVDHAPR_	IIQDFSSDPASN(1)ITSHSIEKIPSAAE	IIQDFSSDPASN(56.37)ITSHSIEKI	12	4	883	1	
IPI00756279	381	EDEDFQPREEAMIYHNQTSEIREIIDFTHMY	1	53.7538	124.09	53.754	_EEAM(ox)IYHN(de)QTSEIR_	EEAMIYHN(1)QTSEIR	EEAMIYHN(53.75)QTSEIR	8	3	580	1	
IPI00404189	667	HKGIDWYMEFFPIPSNTTSDFYFEKSANYFD	1	56.304	56.304	56.304	_GIDWYM(ox)EFFPIPSN(de)TTSDFYFEK_	GIDWYMEFFPIPSN(1)TTSDFYFEK	GIDWYMEFFPIPSN(56.3)TTSDFYFE	14	3	951	1	
IPI00750388	236	GAVKNVWQPERINAINITSAIDRGGRVPIPI	0.99993	41.5226	86.803	86.803	_INAIN(de)ITSAIDR_	INAIN(1)ITSAIDR	IN(-41.52)AIN(41.52)ITSAIDR	5	2	651	1	
IPI00116945	256	RVSSYRMWIENITNGNMTS_____________	1	86.489	170.66	170.66	_M(ox)WIENITN(de)GN(de)M(ox)TS_	MWIENITN(1)GN(1)MTS	MWIEN(-43.1)ITN(43.1)GN(86.4	10	2	773	1	
IPI00848991	203	ECIIWIQRYITMGGKNFTRTDPPKTTVTHQF	1	92.8661	92.866	92.866	_YITM(ox)GGKN(de)FTR_	YITMGGKN(1)FTR	YITMGGKN(92.87)FTR	8	2	653	1	
IPI00268030	65	ITDDQDVEIGSMQVMNKTRRIMEQGGAHFIN	0.999689	36.5049	54.225	54.225	_PNIIIVITDDQDVEIGSM(ox)QVMN(de)K_	PNIIIVITDDQDVEIGSMQVMN(1)K	PN(-36.5)IIIVITDDQDVEIGSMQVM	22	3	864	1	
IPI00990932	166	HFNIRTARDIGMFAPNMTRIIKDVIQYREGT	1	65.3952	75.695	65.395	_DIGM(ox)FAPN(de)M(ox)TR_	DIGMFAPN(1)MTR	DIGMFAPN(65.4)MTR	8	2	643	1	
IPI00136513	111	EYEIISFADTDISSINQTRIEFHFSGRMASG	1	131.037	131.04	131.04	_ETIIEHDQRQEEYEIISFADTDISSIN(de)QTR_	ETIIEHDQRQEEYEIISFADTDISSIN(1)	ETIIEHDQRQEEYEIISFADTDISSIN(	27	3	1195	1	
IPI00308328	106	KGEEFSGRGAYPVFFNFTRGNGIAFSDGERW	1	80.7633	80.763	80.763	_GAYPVFFN(de)FTR_	GAYPVFFN(1)FTR	GAYPVFFN(80.76)FTR	8	2	660	1	
IPI00310797	256	GGGWTVIQARIDGSTNFTREWKDYKAGFGNI	1	98.0483	98.048	98.048	_IDGSTN(de)FTR_	IDGSTN(1)FTR	IDGSTN(98.05)FTR	6	2	506	1	
IPI00116913	2395	ESGIMDIREAINQAVNTTREAEEINSRNQER	0.999818	37.3992	134.56	134.56	_EAINQAVN(de)TTR_	EAINQAVN(1)TTR	EAIN(-37.4)QAVN(37.4)TTR	8	2	609	1	
IPI00230718	430	VNADGCIKTDNGKTVNITRDNIIDDVISFIR	1	138.388	138.39	138.39	_TVN(de)ITRDN(de)IIDDVISFIR_	TVN(1)ITRDN(1)IIDDVISFIR	TVN(138.39)ITRDN(138.39)IIDD	3	3	703	1	

IPI00648288	661	PCSKHDVFCGFIICTNITRAPRIGQIQGEII	1	101.532	101.53	101.53	_HDVFCGFIICTN(de)ITR_	HDVFCGFIICTN(1)ITR	HDVFCGFIICTN(101.53)ITR	12	3	619	1
IPI00153840	67	SIVVIQNPARQTIFFNGTRAIKDERFQIEEF	1	72.3207	98.04	72.321	_QTIFFN(de)GTR_	QTIFFN(1)GTR	QTIFFN(72.32)GTR	6	2	543	1
IPI00136280	320	ERTFNIKFIRPFMQYNSTRAGGVEVDEDTIR	1	116.546	116.55	116.55	_FIRPFM(ox)QYN(de)STR_	FIRPFMQYN(1)STR	FIRPFMQYN(116.55)STR	9	3	526	1
IPI00649911	294	RQNDIKIKGMSETISNITQRISIIESHVVAI	1	104.41	111.79	104.41	_IKGM(ox)SETISN(de)ITQR_	IKGMSETISN(1)ITQR	IKGMSETISN(104.41)ITQR	10	3	532	1
IPI00458413	3465	RGPFDSFVVQYQDTENETQAIIVDGDQDKVI	1	44.9549	71.623	44.955	_GPFDSFVVQYQDTEN(de)ETQAIIVDGDQDKVIISGIEPK_	GPFDSFVVQYQDTEN(1)ETQAIIVDGDQ	GPFDSFVVQYQDTEN(44.95)ETQAII	15	4	1025	1
IPI00458393	520	SGAAYVIEQASSHIGNSTQAAVRDAVVGRPP	1	57.7348	57.735	57.735	_EIGGAIDSGAAYVIEQASSHIGN(de)STQAAVR_	EIGGAIDSGAAYVIEQASSHIGN(1)STQ	EIGGAIDSGAAYVIEQASSHIGN(57.7	23	3	992	1
IPI00129158	297	EDIQIIWIENGNVSRNDTPKNITKNTDGTYN	1	49.0765	119.79	49.076	_FYPEDIQIIWIEN(de)GN(de)VSRN(de)DTPKN(de)ITK_	FYPEDIQIIWIEN(1)GN(1)VSRN(1)D	FYPEDIQIIWIEN(49.08)GN(49.08	19	3	1070	1
IPI00110808	103	IAVDKANIDVMKERSNNTPDANVAPEVTVIS	0.333333	0	89.374	89.374	_ERSN(de)NTPDANVAPEVTVISR_	ERSN(0.333)N(0.333)TPDAN(0.33	ERSN(0)N(0)TPDAN(0)VAPEVTVIS	4	3	724	1
IPI00468674	657	RIQYSRDTKKIFISINVTNSPSSQRAGEDAH	1	84.8919	84.892	84.892	_IFISIN(de)VTN(de)SPSSQ(de)R_	IFISIN(1)VTN(1)SPSSQR	IFISIN(84.89)VTN(84.89)SPSSQ	6	2	833	1
IPI00111908	324	YKMSMANRGQNQPVINITNRQAFITAQNHGY	0.999967	44.7697	132.79	132.79	_GQNQPVIN(de)ITNR_	GQNQPVIN(1)ITNR	GQN(-67.5)QPVIN(44.77)ITN(-4	8	2	678	1
IPI00116945	251	PGVYTRVSSYRMWIENITNGNMTS_______	0.953196	13.0131	88.101	88.101	_M(ox)WIEN(de)ITN(de)GNM(ox)TS_	MWIEN(0.953)ITN(0.983)GN(0.06	MWIEN(13.01)ITN(17.54)GN(-13	5	2	773	1
IPI00131116	203	YETKRCKTMTIIEAGNNTMKVDCTSGFKEHN	0.973166	15.595	78.486	78.486	_TM(ox)TIIEAGN(de)NTM(ox)K_	TMTIIEAGN(0.973)N(0.027)TMK	TMTIIEAGN(15.6)N(-15.6)TMK	9	2	729	1
IPI00222819	648	VMYPRGYPYNGTNYVNVTMHIRSPITRAAYI	0.9982	26.6113	99.044	99.044	_GYPYNGTN(de)YVN(de)VTM(ox)HIR_	GYPYN(0.175)GTN(0.827)YVN(0.9	GYPYN(-6.78)GTN(6.78)YVN(26.	11	2	1009	1
IPI00111228	617	STVADAYRFINHTVGNKTKVEEGYYYINNFD	1	89.8046	89.805	89.805	_FIN(de)HTVGN(de)KTK_	FIN(1)HTVGN(1)KTK	FIN(89.8)HTVGN(89.8)KTK	8	2	631	1
IPI00622572	249	KVTSSRRFIASFNVVNTTKRDAGKYRCMICT	0.99917	30.807	136.93	79.466	_FIASFNVVN(de)TTKR_	FIASFN(0.001)VVN(0.999)TTKR	FIASFN(-30.81)VVN(30.81)TTKR	9	3	500	1
IPI00129158	302	IWIENGNVSRNDTPKNITKNTDGTYNYTSIF	1	49.0765	119.79	49.076	_FYPEDIQIIWIEN(de)GN(de)VSRN(de)DTPKN(de)ITK_	FYPEDIQIIWIEN(1)GN(1)VSRN(1)D	FYPEDIQIIWIEN(49.08)GN(49.08	24	3	1070	1
IPI00468814	353	SRYQTVDSSNIDGFVNCTKIIGNIDFIITGI	0.999999	60.1623	161.35	74.428	_YQTVDSSNIDGFVN(de)CTK_	YQTVDSSNIDGFVN(1)CTK	YQTVDSSN(-60.16)IDGFVN(60.16	14	3	650	1
IPI00338785	1535	ADEAKQNAQDVIIKTNATKEKVDKSNEDIRN	1	68.4106	112.17	111.79	_QNAQDVIIKTN(de)ATK_	QNAQDVIIKTN(1)ATK	QN(-68.41)AQDVIIKTN(68.41)AT	11	3	516	1
IPI00127407	539	VFSNPEDWKEKYIHENYTKAIAGKIVETPCP	1	89.3546	104.2	89.355	_YIHEN(de)YTK_	YIHEN(1)YTK	YIHEN(89.35)YTK	5	2	535	1
IPI00123658	134	RNITWEESKQYCTEQNATIVKTASQSTIDYI	1	115.342	115.34	115.34	_QYCTEQN(de)ATIVK_	QYCTEQN(1)ATIVK	QYCTEQN(115.34)ATIVK	7	2	728	1
IPI00124725	91	EVSFDVEIPKTAFITNFTITIDGVTYPGNVK	1	104.276	132.01	132.01	_TAFITN(de)FTITIDGVTYPGNVK_	TAFITN(1)FTITIDGVTYPGNVK	TAFITN(104.28)FTITIDGVTYPGN(	6	2	1137	1
IPI00115762	478	EGTTVIQDERFFPYANGTISIRDIQANDTGR	1	84.1687	84.169	84.169	_FFPYAN(de)GTISIR_	FFPYAN(1)GTISIR	FFPYAN(84.17)GTISIR	6	2	694	1
IPI00378635	122	KAPSFERGVYEITHNNKTISIKTINAMQQMS	1	133.807	133.81	133.81	_GVYEITHN(de)N(de)K_	GVYEITHN(1)N(1)K	GVYEITHN(133.81)N(133.81)K	9	2	589	1
IPI00125262	301	FAKWAKKHRIVFTHPNWTIS___________	1	91.5493	91.549	91.549	_IVFTHPN(de)WTIS_	IVFTHPN(1)WTIS	IVFTHPN(91.55)WTIS	7	2	658	1
IPI00322542	196	QGPCVEWIHRYIRIGNETIQRSDPPKAHVTH	1	111.455	111.46	111.46	_IGN(de)ETIQR_	IGN(1)ETIQR	IGN(111.46)ETIQR	3	2	466	1
IPI00322867	750	ISSPTSDFQIEVTPQNITINPSSSGSMFSWR	0.975524	16.005	118.57	118.57	_IGISSPTSDFQIEVTPQN(de)ITINPSSSGSM(ox)FSWR_	IGISSPTSDFQIEVTPQN(0.976)ITIN	IGISSPTSDFQIEVTPQN(16)ITIN(-	18	4	901	1
IPI00471264	49	DMQWNYAPKGRNVITNQTINNDTVASSFIKS	0.994765	23.1219	119.08	119.08	_NVITN(de)QTINN(de)DTVASSFIK_	N(0.005)VITN(0.995)QTIN(0.209)	N(-23.12)VITN(23.12)QTIN(-5.	5	3	694	1
IPI00272690	700	IEGSKIIIEKSTEVSNHTIKYGTRAKTFDVS	1	107.026	107.03	107.03	_STEVSN(de)HTIK_	STEVSN(1)HTIK	STEVSN(107.03)HTIK	6	2	559	1
IPI00229935	28	TISVVQTGVAEQVKCNFTIIESRVSSISASI	1	90.9131	90.913	90.913	_CN(de)FTIIESR_	CN(1)FTIIESR	CN(90.91)FTIIESR	2	2	571	1
IPI00400016	1173	ANVSITQPESTGEPNNMTIIAEEARRIAERH	0.501354	0	49.793	49.793	_M(ox)AAAN(de)VSITQPESTGEPN(de)NM(ox)TIIAEEAR_	MAAAN(0.997)VSITQPESTGEPN(0.5	MAAAN(22.65)VSITQPESTGEPN(0)	19	3	994	1
IPI00471089	131	CPEIPDKTSICDSDANCTIGSSDTHSSGIGT	1	73.9806	73.981	73.981	_TSICDSDAN(de)CTIGSSDTHSSGIGTGR_	TSICDSDAN(1)CTIGSSDTHSSGIGTGR	TSICDSDAN(73.98)CTIGSSDTHSSG	9	3	886	1
IPI00124497	714	NHKIQQEPGIIIGPGNSTIFIERVTEEDEGV	1	91.4006	91.401	91.401	_IQQEPGIIIGPGN(de)STIFIER_	IQQEPGIIIGPGN(1)STIFIER	IQQEPGIIIGPGN(91.4)STIFIER	13	3	728	1
IPI00757771	228	GEYHCVYHFVSAPKANATIEVKAAPDITGHK	1	108.433	110.87	108.43	_AN(de)ATIEVK_	AN(1)ATIEVK	AN(108.43)ATIEVK	2	2	424	1
IPI00279010	314	QQGTQEEQINVNIKGNITIERVHRNQSGIYG	1	120.457	120.46	120.46	_GN(de)ITIER_	GN(1)ITIER	GN(120.46)ITIER	2	2	402	1
IPI00808296	454	NYIIESNITSDAHKGNSTIAVPKRCDADAPK	1	162.358	162.36	162.36	_GN(de)STIAVPK_	GN(1)STIAVPK	GN(162.36)STIAVPK	2	2	444	1
IPI00466999	256	TSGKVIWTINPNHISNGTIAAPVVVIPDIDE	0.48249	0	78.978	78.978	_VIWTINPN(de)HISNGTIAAPVVVIPDIDEDGVR_	VIWTIN(0.035)PN(0.482)HISN(0.	VIWTIN(-11.39)PN(0)HISN(0)GT	12	3	1109	1
IPI00118250	388	DPISISPGTPPPIINNSTHRQMMSSPSTVKI	0.986506	18.5799	52.03	52.03	_SNASDDPISISPGTPPPIIN(de)N(de)STHR_	SN(0.027)ASDDPISISPGTPPPIIN(0.	SN(-18.58)ASDDPISISPGTPPPIIN	21	3	864	1
IPI00127556	445	ASIHWRREKIIIPAKNTTHIKTHSVGRKMII	1	86.8981	86.898	86.898	_IIIPAKN(de)TTHIK_	IIIPAKN(1)TTHIK	IIIPAKN(86.9)TTHIK	7	3	451	1
IPI00268030	132	SWQAQHESRTFAVYINSTGYRTAFFGKYINE	1	73.8478	73.848	73.848	_TFAVYIN(de)STGYR_	TFAVYIN(1)STGYR	TFAVYIN(73.85)STGYR	7	2	697	1
IPI00119035	333	CGGNIIGVRTVYIHANQTGYPDPSSRYDAIC	1	70.9421	70.942	70.942	_TVYIHAN(de)QTGYPDPSSR_	TVYIHAN(1)QTGYPDPSSR	TVYIHAN(70.94)QTGYPDPSSR	7	2	954	1
IPI00273133	104	ARPYCKVIIIAQTHANNTGSYHCYYKYIKAR	0.946259	12.4571	66.022	66.022	_VIIIAQTHAN(de)NTGSYHCYYK_	VIIIAQTHAN(0.946)N(0.054)TGSY	VIIIAQTHAN(12.46)N(-12.46)TG	10	3	785	1
IPI00405543	196	FIDPEGSQVDTIEGNNITGIASFPDFEIPSN	0.876322	8.57533	128.22	128.22	_ETVITFIDPEGSQVDTIEGNN(de)ITGIASFPDFEIPSNPK_	ETVITFIDPEGSQVDTIEGN(0.122)N(	ETVITFIDPEGSQVDTIEGN(-8.58)N	21	4	1024	1
IPI00308971	1305	IISQKITFENGIIKMNYTGGDTCHKVYQRST	1	80.5221	80.522	80.522	_M(ox)N(de)YTGGDTCHK_	MN(1)YTGGDTCHK	MN(80.52)YTGGDTCHK	2	2	651	1
IPI00223446	1361	KKFYFGGSPISPQYANFTGCISNAYFTRIDR	0.999792	36.8127	69.27	69.27	_FYFGGSPISPQYAN(de)FTGCISNAYFTR_	FYFGGSPISPQYAN(1)FTGCISNAYFTR	FYFGGSPISPQYAN(36.81)FTGCISN	14	3	989	1
IPI00122584	265	KQIFAPRQEEFHQRANVTFTISKFHWCCHHH	1	110.387	110.39	110.39	_AN(de)VTFTISK_	AN(1)VTFTISK	AN(110.39)VTFTISK	2	2	491	1
IPI00420835	958	GIDSKASIVIRSRIWNSTFIEEYSKINYIDI	1	110.306	110.31	110.31	_IWN(de)STFIEEYSK_	IWN(1)STFIEEYSK	IWN(110.31)STFIEEYSK	3	2	759	1
IPI00122493	69	CPREVQMGDFVRYHYNGTFEDGKKFDSSYDR	1	102.4	102.4	102.4	_YHYN(de)GTFEDGKK_	YHYN(1)GTFEDGKK	YHYN(102.4)GTFEDGKK	4	2	730	1
IPI00223446	780	SAYNTAVDSARDAVRNITEVVPQIIDQIRTV	1	112.667	112.67	112.67	_DAVRN(de)ITEVVPQIIDQIR_	DAVRN(1)ITEVVPQIIDQIR	DAVRN(112.67)ITEVVPQIIDQIR	5	3	694	1
IPI00114844	83	NINKTMTSGDPGETANATEVKKCITQPIKNI	1	78.95	115.73	78.95	_TM(ox)TSGDPGETAN(de)ATEVKK_	TMTSGDPGETAN(1)ATEVKK	TMTSGDPGETAN(78.95)ATEVKK	12	2	927	1
IPI00120793	196	ITIKQHTVTTTTKGENFTETDVKMMERVVEQ	1	73.4483	73.448	73.448	_QHTVTTTTKGEN(de)FTETDVK_	QHTVTTTTKGEN(1)FTETDVK	QHTVTTTTKGEN(73.45)FTETDVK	12	2	1070	1
IPI00223987	682	EYRSVSIIDKKSDVINITEQVQWVKVNSNMT	1	159.128	159.13	159.13	_SDVIN(de)ITEQVQWVK_	SDVIN(1)ITEQVQWVK	SDVIN(159.13)ITEQVQWVK	5	2	830	1
IPI00756745	2644	GIFTVQIDEDRRHMQNITEEQPIEVKKIFVG	1	123.758	123.76	123.76	_HM(ox)QN(de)ITEEQPIEVK_	HMQN(1)ITEEQPIEVK	HMQN(123.76)ITEEQPIEVK	4	2	857	1
IPI00808422	167	CSGYVQIEVCRGSPANVTEARISFYSGHSSF	1	94.4653	94.465	94.465	_GSPAN(de)VTEAR_	GSPAN(1)VTEAR	GSPAN(94.47)VTEAR	5	2	502	1
IPI00153202	546	QAAKYNGSIHKCDISNSTEAGQKIIKMISIG	1	83.2035	83.204	83.204	_CDISN(de)STEAGQK_	CDISN(1)STEAGQK	CDISN(83.2)STEAGQK	5	2	656	1
IPI00132927	53	EIKATGTAHFFNFIINTTDYRIIIKDEDHDR	0.997283	25.6476	119.4	119.4	_ATGTAHFFNFIIN(de)TTDYR_	ATGTAHFFN(0.003)FIIN(0.997)TT	ATGTAHFFN(-25.65)FIIN(25.65)	13	3	697	1
IPI00130627	93	NSEENPTPGVVINRPNGTDVYKGVIKDYTGE	0.930819	11.437	59.426	59.426	_N(de)GIPDEQIIVM(ox)M(ox)YDDIANSEENPTPGVVINRPN(de)GTDVYK_	N(0.993)GIPDEQIIVMMYDDIAN(0.0	N(25.04)GIPDEQIIVMMYDDIAN(-2	33	4	1089	1
IPI00312058	481	HIKDAQIFIQKKAVKNFTDVHPDYGARIQAI	1	57.1063	79.492	57.106	_N(de)FTDVHPDYGAR_	N(1)FTDVHPDYGAR	N(57.11)FTDVHPDYGAR	1	2	697	1
IPI00319200	39	RQPTFVVFPKDIKTSNITDTQIAEAYIYRYG	1	177.555	177.56	177.56	_TSN(de)ITDTQIAEAYIYR_	TSN(1)ITDTQIAEAYIYR	TSN(177.56)ITDTQIAEAYIYR	3	2	930	1
IPI00120225	765	TMSSRSRNTTVADTYNITDPEEFETEYPFFE	1	81.9736	81.974	81.974	_SRN(de)TTVADTYN(de)ITDPEEFETEYPFFESR_	SRN(1)TTVADTYN(1)ITDPEEFETEYP	SRN(81.97)TTVADTYN(81.97)ITD	11	3	1121	1
IPI00315576	372	VWPGPSGYSSITAIENSTDGKKQPPQIFVIY	1	148.286	148.29	148.29	_VQVWPGPSGYSSITAIEN(de)STDGK_	VQVWPGPSGYSSITAIEN(1)STDGK	VQVWPGPSGYSSITAIEN(148.29)ST	18	2	1198	1
IPI00221669	653	WHWPINYQGIRFSGANDTDFRVYIIGNPVVW	1	91.9612	132.32	91.961	_FSGAN(de)DTDFR_	FSGAN(1)DTDFR	FSGAN(91.96)DTDFR	5	2	566	1
IPI00128336	259	KHIRAVGDEFRSQHINSTDAADKMAPEEDWT	1	66.0043	95.651	66.004	_SQHIN(de)STDAADK_	SQHIN(1)STDAADK	SQHIN(66)STDAADK	5	2	644	1
IPI00222321	136	TCVDQDIKPQRNFVINMTCRFCWQIPETDYE	1	72.9817	92.538	77.677	_NFVIN(de)M(ox)TCR_	NFVIN(1)MTCR	N(-72.98)FVIN(72.98)MTCR	5	2	586	1
IPI00121254	210	IKQPESVNVTRNTAFNITCQAVGPPEPVNIF	0.957167	13.5226	137.52	137.52	_NTAFN(de)ITCQAVGPPEPVNIFWVQN(de)SSR_	N(0.043)TAFN(0.957)ITCQAVGPPE	N(-13.52)TAFN(13.52)ITCQAVGP	5	3	1017	1
IPI00129158	168	EVSGPADRGIPDQKVNFTCKSHGFSPRNITI	1	88.0206	93.011	88.021	_GIPDQKVN(de)FTCK_	GIPDQKVN(1)FTCK	GIPDQKVN(88.02)FTCK	8	2	704	1
IPI00126316	242	WTQDRMWRKNRSRNQNSTCIGTDINRNFDVS	0.995299	23.926	69.716	69.716	_NQN(de)STCIGTDINR_	N(0.004)QN(0.995)STCIGTDIN(0.	N(-23.93)QN(23.93)STCIGTDIN(	3	2	747	1
IPI00123814	141	VHSKGIKIGIYADVGNKTCAGFPGSFGSYDI	1	81.865	81.865	81.865	_IGIYADVGN(de)K_	IGIYADVGN(1)K	IGIYADVGN(81.87)K	9	2	526	1
IPI00848693	1362	VEQYRAYVAFPDFFRNSTATWWKKEIKEIHS	1	88.7296	88.73	88.73	_AYVAFPDFFRN(de)STATWWKK_	AYVAFPDFFRN(1)STATWWKK	AYVAFPDFFRN(88.73)STATWWKK	11	3	779	1
IPI00944095	533	AKTEDITEGNKSISENATATTEPKMTESTRV	1	227.625	227.63	227.63	_SISEN(de)ATATTEPK_	SISEN(1)ATATTEPK	SISEN(227.63)ATATTEPK	5	2	675	1
IPI00114733	119	EEVHTGIGEIIRSISNSTARNVTWKIGSRIY	1	69.8246	118.33	69.825	_SISN(de)STAR_	SISN(1)STAR	SISN(69.82)STAR	4	2	419	1
IPI00453524	53	ESIHRHRYINSFPHENSTAFYGVNQFSYIFP	0.999188	30.9024	163.74	163.74	_YINSFPHEN(de)STAFYGVNQFSYIFPEEFK_	YIN(0.001)SFPHEN(0.999)STAFYG	YIN(-30.9)SFPHEN(30.9)STAFYG	9	3	1126	1
IPI00314900	339	ITYRIIAREIGPIYTNITADIGTDPRGPRSP	1	65.0849	76.378	65.085	_EIGPIYTN(de)ITADIGTDPR_	EIGPIYTN(1)ITADIGTDPR	EIGPIYTN(65.08)ITADIGTDPR	8	2	974	1
IPI00458708	497	AKVHYNIGKNIADQGNQTAAIKYYREAVRIN	1	105.077	130.2	130.2	_NIADQGN(de)QTAAIK_	NIADQGN(1)QTAAIK	N(-105.08)IADQGN(105.08)QTAA	7	2	673	1
IPI00406603	1663	DESPEWFQEDDAVQPNSSYTYVWHATKRSGP	1	105.026	170.65	105.03	_TYEDESPEWFQEDDAVQPN(de)SSYTYVWHATKR_	TYEDESPEWFQEDDAVQPN(1)SSYTYVW	TYEDESPEWFQEDDAVQPN(105.03)S	19	4	946	1
IPI00118475	254	PMNQCIVATGIDVIGNRSYTVRGCATASWCQ	1	71.2069	121.82	121.82	_GPM(ox)NQCIVATGIDVIGN(de)R_	GPMNQCIVATGIDVIGN(1)R	GPMN(-71.21)QCIVATGIDVIGN(71	17	3	645	1
IPI00453632	115	GIGKRCEDDKVKIQNNISYQMADIHHIKEQI	0.5	0	66.246	66.246	_IQN(de)NISYQM(ox)ADIHHIK_	IQN(0.5)N(0.5)ISYQMADIHHIK	IQN(0)N(0)ISYQMADIHHIK	4	3	648	1
IPI00461962	143	HVSFSSDFSIQKKGFNASYIRVAVSIRNQKV	1	95.0987	95.099	95.099	_GFN(de)ASYIR_	GFN(1)ASYIR	GFN(95.1)ASYIR	3	2	465	1
IPI00121254	165	SIIAIFSIASVQRSDNGSYFCKMKVNNREIV	1	105.863	105.86	105.86	_SDN(de)GSYFCK_	SDN(1)GSYFCK	SDN(105.86)GSYFCK	3	2	540	1
IPI00228404	191	HPARASIPAPTIQSINISWNRFRAVPDIRDI	1	99.7746	172.57	172.57	_ASIPAPTIQSIN(de)ISWNR_	ASIPAPTIQSIN(1)ISWNR	ASIPAPTIQSIN(99.77)ISWN(-99.	12	3	624	1
IPI00113539	1243	QSSCTFENINPGIEYNVSVYTVKDDKESAPI	0.922966	10.9244	54.09	34.149	_ITTTPTN(de)GQQGTSIEEVVHADQSSCTFENINPGIEYN(de)VSVYTVK_	ITTTPTN(0.47)GQQGTSIEEVVHADQS	ITTTPTN(0)GQQGTSIEEVVHADQSSC	37	4	1208	1
IPI00876541	648	CPKDIAVFCHFTNAANSSVRSPSMKIKIIPR	0.87287	8.36703	139.6	139.6	_DIAVFCHFTNAAN(de)SSVR_	DIAVFCHFTN(0.127)AAN(0.873)SS	DIAVFCHFTN(-8.37)AAN(8.37)SS	13	3	637	1
IPI00463492	362	AWAIVGVIDGGSSSCNESVRNYENHSTGGKA	1	96.143	96.143	96.143	_QAWAIVGVIDGGSSSCN(de)ESVR_	QAWAIVGVIDGGSSSCN(1)ESVR	QAWAIVGVIDGGSSSCN(96.14)ESVR	17	3	732	1
IPI00719927	457	IDCNDNDPKFMISGYNFSVMENMPAISPVGM	0.991051	20.672	51.871	51.871	_FM(ox)ISGYN(de)FSVM(ox)ENM(ox)PAISPVGM(ox)VTVIDGDKGENAR_	FMISGYN(0.991)FSVMEN(0.008)MP	FMISGYN(20.67)FSVMEN(-20.67)	7	4	961	1
IPI00626350	103	PENFSVIITIRAQPANQSVIISIYDEKGVRQ	1	87.1483	92.773	87.148	_AQPAN(de)QSVIISIYDEK_	AQPAN(1)QSVIISIYDEK	AQPAN(87.15)QSVIISIYDEK	5	3	593	1
IPI00131000	254	KTVRAVGPRSGSEKWNFSVGHFEIRYIPDME	1	83.729	83.729	83.729	_SGSEKWN(de)FSVGHFEIR_	SGSEKWN(1)FSVGHFEIR	SGSEKWN(83.73)FSVGHFEIR	7	3	628	1
IPI00377727	942	IAGRYIGVNCVWRYYNFSVFQIDAPSFAYIK	1	123.023	123.02	123.02	_YYN(de)FSVFQIDAPSFAYIK_	YYN(1)FSVFQIDAPSFAYIK	YYN(123.02)FSVFQIDAPSFAYIK	3	3	725	1
IPI00113480	457	GPAAMKKYIPQYRSYNDSVDPRIANVFTNAF	1	106.199	106.2	106.2	_SYN(de)DSVDPR_	SYN(1)DSVDPR	SYN(106.2)DSVDPR	3	2	527	1
IPI00120674	1022	PHSNFRDQIERTPVINCSVAVCKRIQCDIPS	1	75.0877	75.088	75.088	_TPVIN(de)CSVAVCK_	TPVIN(1)CSVAVCK	TPVIN(75.09)CSVAVCK	5	2	675	1
IPI00227797	163	IVKIIEEVVRIRVAANFSTPVISTSDSSNPG	1	98.247	112.44	112.44	_VAAN(de)FSTPVISTSDSSNPGQER_	VAAN(1)FSTPVISTSDSSNPGQER	VAAN(98.25)FSTPVISTSDSSN(-98	4	3	756	1
IPI00471090	91	INSTRVTAEVTTHGTNTSTPTTREGTTDRVT	1	93.4953	93.495	93.495	_VTAEVTTHGTN(de)TSTPTTR_	VTAEVTTHGTN(1)TSTPTTR	VTAEVTTHGTN(93.5)TSTPTTR	11	2	938	1
IPI00331032	97	EASFISQCMKYEVDWNQSTIDCVDPISSIAA;	1	131.329	131.33	131.33	_YEVDWN(de)QSTIDCVDPISSIAAN(de)R_	YEVDWN(1)QSTIDCVDPISSIAAN(1)R	YEVDWN(131.33)QSTIDCVDPISSIA	6	2	1328	1
IPI00118250	387	DDPISISPGTPPPIINNSTHRQMMSSPSTVK	0.986506	18.5799	52.03	52.03	_SNASDDPISISPGTPPPIIN(de)N(de)STHR_	SN(0.027)ASDDPISISPGTPPPIIN(0.	SN(-18.58)ASDDPISISPGTPPPIIN	20	3	864	1

IPI00380778	88	VQQATIAASRQASSPNSSTAQQQTATTQASM	1	46.4146	51.321	46.415 _QASSPN(de)SSTAQQQTATTQASMN(de)IATTSAAQIISR_	QASSPN(1)SSTAQQQTATTQASMN(1)I	QASSPN(46.41)SSTAQQQTATTQASM	6	3	1152	1
IPI00336324	186	IGVTADDVKNVIIWGNHSSTQYPDVNHAKVK	0.999602	33.9999	98.507	98.507 _NVIIWGN(de)HSSTQYPDVNHAK_	NVIIWGN(1)HSSTQYPDVNHAK	N(-64.31)VIIWGN(34)HSSTQYPDV	7	3	761	1
IPI00131292	57	IAGTPEHRCIVPDTVNISSSWRNHSIPIETK	1	96.5915	96.591	96.591 _CIVPDTVN(de)ISSSWR_	CIVPDTVN(1)ISSSWR	CIVPDTVN(96.59)ISSSWR	8	2	818	1
IPI00896674	38	HHQEGMEAFIKSVAQNYSSITHIHSIGKSVR	1	131.448	131.45	131.45 _SVAQN(de)YSSITHIHSIGK_	SVAQN(1)YSSITHIHSIGK	SVAQN(131.45)YSSITHIHSIGK	5	2	922	1
IPI00112616	1557	CVIKSPANGSIGPMINISSGESRIIAARFFD	1	98.4429	98.443	98.443 _SPAN(de)GSIGPM(ox)IN(de)ISSGESR_	SPAN(1)GSIGPMIN(1)ISSGESR	SPAN(98.44)GSIGPMIN(98.44)IS	12	2	946	1
IPI00420867	327	TGDPGNCVVAIRSIFNFSSCKGQKDCAFNGI	1	82.9999	83	83 _SIFN(de)FSSCKGQK_	SIFN(1)FSSCKGQK	SIFN(83)FSSCKGQK	4	3	469	1
IPI00263505	834	TFTMEHIPKTISPTGNISSAPKDFAVYGIET	1	107.529	107.53	107.53 _TISPTGN(de)ISSAPK_	TISPTGN(1)ISSAPK	TISPTGN(107.53)ISSAPK	7	2	637	1
IPI00116913	2198	AGAIIPAIREQIQGINASSAAWARIHRINAS	1	112.299	112.3	112.3 _EQIQGIN(de)ASSAAWAR_	EQIQGIN(1)ASSAAWAR	EQIQGIN(112.3)ASSAAWAR	7	2	802	1
IPI00121254	229	QAVGPPEPVNIFWVQNSSRVNEKPERSPSVI	0.997424	26.4105	137.52	137.52 _NTAFN(de)ITCQAVGPPEPVNIFWVQN(de)SSR_	N(0.043)TAFN(0.957)ITCQAVGPPE	N(-13.52)TAFN(13.52)ITCQAVGP	24	3	1017	1
IPI00136565	192	EYPIVNVPTHKGINCNISRTRHHDEPGNSNM	0.883764	8.80994	122.52	122.52 _GINCN(de)ISR_	GIN(0.116)CN(0.884)ISR	GIN(-8.81)CN(8.81)ISR	5	2	468	1
IPI00120674	907	FGNKIIIKAIVASENNMSRTHKTKFQIEIPV	1	100.931	100.93	100.93 _AIVASEN(de)N(de)M(ox)SR_	AIVASEN(1)N(1)MSR	AIVASEN(100.93)N(100.93)MSR	8	2	605	1
IPI00162831	202	PIAPIVPISIVGSASNTSRRIDQTPEHQREQ	1	115.361	115.36	115.36 _GQQQVIPIPIAPIVPISIVGSASN(de)TSR_	GQQQVIPIPIAPIVPISIVGSASN(1)TS	GQQQVIPIPIAPIVPISIVGSASN(115	24	3	915	1
IPI00406603	1519	VTYNDPYRTDTRTDVNSSRNPDTIAAWYIRG	0.959045	13.6953	109.07	97.235 _TDVN(de)SSRNPDTIAAWYIR_	TDVN(0.959)SSRN(0.041)PDTIAAW	TDVN(13.7)SSRN(-13.7)PDTIAAW	4	3	694	1
IPI00331597	77	AIKKAIAAAGYDVEKNNSRIKIGIKSIVSKG	0.808086	6.24352	149.3	149.3 _AIAAAGYDVEKN(de)NSR_	AIAAAGYDVEKN(0.808)N(0.192)SR	AIAAAGYDVEKN(6.24)N(-6.24)SR	12	2	790	1
IPI00132279	88	KDPCSVIPSDYDIFINISRHPIPRDKSIFWE	1	104.837	104.84	104.84 _GVIDKDPCSVIPSDYDIFIN(de)ISR_	GVIDKDPCSVIPSDYDIFIN(1)ISR	GVIDKDPCSVIPSDYDIFIN(104.84)	20	3	875	1
IPI00129526	445	INFVKGVVDSDDIPINVSRETIQQHKIIKVI	1	186.051	186.05	186.05 _GVVDSDDIPIN(de)VSR_	GVVDSDDIPIN(1)VSR	GVVDSDDIPIN(186.05)VSR	11	2	744	1
IPI00119063	2621	CGVGEFRCRDGSCIGNSSRCNQFVDCEDASD	1	104.824	104.82	104.82 _DGSCIGN(de)SSR_	DGSCIGN(1)SSR	DGSCIGN(104.82)SSR	7	2	527	1
IPI00129646	231	PDIPYTCKAWNPVSQNSSQPVRIWQFCTGAS	1	69.0065	210.38	210.38 _AWNPVSQN(de)SSQPVR_	AWNPVSQN(1)SSQPVR	AWN(-69.01)PVSQN(69.01)SSQPV	8	2	786	1
IPI00330594	92	PPSIIIRGSNNSGHDNHSQFVRETEMQVAIQ	0.999999	58.4515	144.29	144.29 _GSN(de)NSGHDN(de)HSQFVR_	GSN(0.981)N(0.019)SGHDN(1)HSQ	GSN(17.11)N(-17.11)SGHDN(58.	9	2	829	1
IPI00112271	499	YNKEQFIMWKQSVGQNYSNVIAHIRWHQDWQ	0.999999	58.4404	152.99	152.99 _QSVGQN(de)YSNVIAHIR_	QSVGQN(1)YSNVIAHIR	QSVGQN(58.44)YSN(-58.44)VIAH	6	3	563	1
IPI00621024	50	GQKVTMSCKSSQSIINSSNQKNYIAWYQQKP	0.998667	28.7468	95.981	95.981 _SSQSIIN(de)SSNQK_	SSQSIIN(0.999)SSN(0.001)QK	SSQSIIN(28.75)SSN(-28.75)QK	7	2	647	1
IPI00406603	381	IPANMDKIYRSQHIDNFSNQIGKHYKKVIYR	0.707508	3.83618	105.46	105.46 _SQHIDN(de)FSNQIGK_	SQHIDN(0.708)FSN(0.292)QIGK	SQHIDN(3.84)FSN(-3.84)QIGK	6	2	745	1
IPI00381160	15	_MIPFIIATIGTAAINSSNPKDYCYSARIRS	0.987552	18.9945	62.203	62.203 _M(ox)IPFIIATIGTAAIN(de)SSNPK_	MIPFIIATIGTAAIN(0.988)SSN(0.0	MIPFIIATIGTAAIN(18.99)SSN(-1	15	3	693	1
IPI00322542	106	GRNFRVNIRTIIGYYNQSNDESHTIQWMYGC	0.982335	17.4516	153.17	153.17 _TIIGYYN(de)QSNDESHTIQWM(ox)YGCDVGPDGR_	TIIGYYN(0.982)QSN(0.018)DESHT	TIIGYYN(17.45)QSN(-17.45)DES	7	3	1132	1
IPI00122633	282	TGNPKGATISHHNIVNNSMIIGQRIKMPTKT	0.86098	8.23081	93.237	93.237 _GATISHHNIVN(de)NSM(ox)IIGQR_	GATISHHN(0.01)IVN(0.861)N(0.1	GATISHHN(-19.52)IVN(8.23)N(-	11	3	694	1
IPI00857855	171	CEEHGIIRENIIDISNASKSGFSYFSHPEYI	1	68.1034	96.464	96.464 _ENIIDISN(de)ASK_	ENIIDISN(1)ASK	EN(-68.1)IIDISN(68.1)ASK	8	2	603	1
IPI00283900	298	RSGPWDIIKATYIVINNSKPEITSKCWICID	0.823043	6.67556	73.91	73.91 _ATYIVIN(de)NSKPEITSK_	ATYIVIN(0.823)N(0.177)SKPEITS	ATYIVIN(6.68)N(-6.68)SKPEITS	7	3	594	1
IPI00138342	478	FGAPIIKEGASEEETNISKMVMKFWANFARN	1	145.294	145.29	145.29 _EGASEEETN(de)ISK_	EGASEEETN(1)ISK	EGASEEETN(145.29)ISK	9	2	648	1
IPI00114810	421	GEKYCGERSQFVVSSNSSKITVHFHSDHSYT	1	85.5541	85.554	85.554 _SQFVVSSN(de)SSK_	SQFVVSSN(1)SSK	SQFVVSSN(85.55)SSK	8	2	586	1
IPI00112614	196	KVFIQGYQIHIASICNGSKIEEIIQIGDAEV	1	93.5098	95.662	93.51 _VFIQGYQIHIASICN(de)GSK_	VFIQGYQIHIASICN(1)GSK	VFIQGYQIHIASICN(93.51)GSK	15	3	679	1
IPI00312980	107	EQSFRGSIRTAQSYYNQSKGGSHTIQWMYGC	1	71.5551	202.65	71.555 _TAQSYYN(de)QSK_	TAQSYYN(1)QSK	TAQSYYN(71.56)QSK	7	2	596	1
IPI00757830	478	GIIDSSKICDYENHFNTSKGGEIPDRPAGVG	0.962014	14.0356	87.667	87.667 _ICDYENHFN(de)TSK_	ICDYEN(0.038)HFN(0.962)TSK	ICDYEN(-14.04)HFN(14.04)TSK	9	3	510	1
IPI00626137	492	SMGTSNFRTVIQFVANISKEFEISDTDTRVG	1	112.43	112.43	112.43 _TVIQFVAN(de)ISK_	TVIQFVAN(1)ISK	TVIQFVAN(112.43)ISK	8	2	611	1
IPI00130758	170	AYIDINIDKCYVIPINTSIVMPPKNIIEIII	1	82.0687	82.069	82.069 _CYVIPIN(de)TSIVM(ox)PPK_	CYVIPIN(1)TSIVMPPK	CYVIPIN(82.07)TSIVMPPK	7	3	584	1
IPI00400016	1159	EIASREIEKAKMAAANVSITQPESTGEPNNM	0.997291	22.65	49.793	49.793 _M(ox)AAAN(de)VSITQPESTGEPN(de)NM(ox)TIIAEEAR_	MAAAN(0.997)VSITQPESTGEPN(0.5	MAAAN(22.65)VSITQPESTGEPN(0)	5	3	994	1
IPI00120155	551	AWDQIPVDDQNGFIRNYSISYRTSVGKEMVV	0.998512	28.2612	68.689	68.689 _VGKNEAVIAWDQIPVDDQN(de)GFIRN(de)YSISYR_	VGKN(0.003)EAVIAWDQIPVDDQN(0.	VGKN(-28.26)EAVIAWDQIPVDDQN(	24	4	868	1
IPI00130627	274	IVKSHTNTSHVMQYGNKSISTMKVMQFQGMK	1	73.1564	173.19	73.156 _SHTN(de)TSHVM(ox)Q(de)YGN(de)K_	SHTN(1)TSHVMQYGN(1)K	SHTN(73.16)TSHVMQYGN(73.16)K	13	2	812	1
IPI00113949	94	FNGTAYIPCPFTKAQNISISEIVVFWQDQQK	1	93.7676	93.768	93.768 _AQN(de)ISISEIVVFWQDQQK_	AQN(1)ISISEIVVFWQDQQK	AQN(93.77)ISISEIVVFWQDQQK	3	3	712	1
IPI00221426	271	TNKHWIIRQAKTPMTNSSIRFIDDAFRRRWQ	1	119.528	119.53	119.53 _TPM(ox)TN(de)SSIR_	TPMTN(1)SSIR	TPMTN(119.53)SSIR	5	2	512	1
IPI00649090	136	VSRQFQEGTRIWEATNSSIQQQIREKISQIG	1	125.975	158.04	125.97 _IWEATN(de)SSIQQQIR_	IWEATN(1)SSIQQQIR	IWEATN(125.97)SSIQQQIR	6	2	838	1
IPI00322304	497	TIPEANFPSFSIPNCNRSIQPEIQPFPQTAS	0.426413	0	76.049	76.049 _IPPINIGEVITIPEANFPSFSIPN(de)CNR_	IPPINIGEVITIPEAN(0.147)FPSFSI	IPPIN(-47.71)IGEVITIPEAN(-4.	26	4	753	1
IPI00117841	283	TIQVNQSSRSEPQEYNVSIPHFWVPNAGAIS	0.999644	34.4868	73.675	73.675 _SEPQEYN(de)VSIPHFWVPNAGAISFR_	SEPQEYN(1)VSIPHFWVPNAGAISFR	SEPQEYN(34.49)VSIPHFWVPN(-34	7	3	916	1
IPI00121240	25	CIIISPVTAFPWKTQNGSIPAVTRTEPTSNV	1	93.6229	93.623	93.623 _TQN(de)GSIPAVTR_	TQN(1)GSIPAVTR	TQN(93.62)GSIPAVTR	3	2	573	1
IPI00762091	68	CIEAQGVQVRVTPVCNASIPAQRWKWVSRNR	1	96.7446	96.745	96.745 _VTPVCN(de)ASIPAQR_	VTPVCN(1)ASIPAQR	VTPVCN(96.74)ASIPAQR	6	3	472	1
IPI00272690	719	KYGTRAKTFDVSNFQNSSIKRIIKKIQNIDR	0.9232	10.7993	73.156	73.156 _TFDVSNFQN(de)SSIKR_	TFDVSN(0.077)FQN(0.923)SSIKR	TFDVSN(-10.8)FQN(10.8)SSIKR	9	2	822	1
IPI00321717	295	KKVIQQIAMGDSAAQNKSIIKIWCKSITNVT	1	94.7168	94.717	94.717 _VIQQIAM(ox)GDSAAQN(de)K_	VIQQIAMGDSAAQN(1)K	VIQQIAMGDSAAQN(94.72)K	14	3	531	1
IPI00314798	146	RHPNCNSKIYIETIPNTSIIIPFHNEGWSSI	0.5	0	87.326	87.326 _IYIETIPN(de)TSIIIPFHNEGWSSIIR_	IYIETIPN(0.5)TSIIIPFHN(0.5)EG	IYIETIPN(0)TSIIIPFHN(0)EGWSS	8	3	972	1
IPI00225715	591	DFSWFGYSIHGVTVANRSIIIIGSPTWKNVS	1	79.4666	79.467	79.467 _VN(de)GEEDFSWFGYSIHGVTVAN(de)R_	VN(1)GEEDFSWFGYSIHGVTVAN(1)R	VN(79.47)GEEDFSWFGYSIHGVTVAN	21	3	829	1
IPI00463774	127	IPDRFKDFIIYIRCRNYSIIIDQPKKCAKKP	1	88.1865	88.187	88.187 _CRN(de)YSIIIDQPK_	CRN(1)YSIIIDQPK	CRN(88.19)YSIIIDQPK	3	3	503	1
IPI00110389	102	FIIQKIKSSRSYSFINSSIIGRSTYKEKYVY	1	96.8199	129.37	96.82 _SYSFIN(de)SSIIGR_	SYSFIN(1)SSIIGR	SYSFIN(96.82)SSIIGR	6	2	673	1
IPI00230145	112	GINAMECAIHIEKSVNQSIIEIHKIATDKND	1	107.088	107.09	107.09 _SVN(de)QSIIEIHK_	SVN(1)QSIIEIHK	SVN(107.09)QSIIEIHK	3	3	424	1
IPI00112616	1549	IPSGVGATCVIKSPANGSIGPMINISSGESR	1	98.4429	98.443	98.443 _SPAN(de)GSIGPM(ox)IN(de)ISSGESR_	SPAN(1)GSIGPMIN(1)ISSGESR	SPAN(98.44)GSIGPMIN(98.44)IS	4	2	946	1
IPI00345112	736	RMVVCDIGNPMVTGTNFSIGIRFAVPRIEKT	0.999999	60.6869	89.859	89.859 _M(ox)VVCDIGNPM(ox)VTGTN(de)FSIGIR_	MVVCDIGNPMVTGTN(1)FSIGIR	MVVCDIGN(-60.69)PMVTGTN(60.6	15	3	772	1
IPI00130627	265	KETIHKQYHIVKSHTNTSHVMQYGNKSISTM	1	73.1564	173.19	73.156 _SHTN(de)TSHVM(ox)Q(de)YGN(de)K_	SHTN(1)TSHVMQYGN(1)K	SHTN(73.16)TSHVMQYGN(73.16)K	4	2	812	1
IPI00123129	168	EEQAKASKKGMWSEGNGSHTIRDIKYTIENP	1	104.954	104.95	104.95 _KGM(ox)WSEGN(de)GSHTIR_	KGMWSEGN(1)GSHTIR	KGMWSEGN(104.95)GSHTIR	8	3	526	1
IPI00129485	183	VRYHHYSSNFSIPKANHSHSGDYYCKGSIGR	1	60.2551	164.9	60.255 _AN(de)HSHSGDYYCK_	AN(1)HSHSGDYYCK	AN(60.26)HSHSGDYYCK	2	3	481	1
IPI00310519	305	VTEPAFEPPARASPGNGSGHSSAPSPDPTIT	1	61.0959	61.096	61.096 _ASPGN(de)GSGHSSAPSPDPTITR_	ASPGN(1)GSGHSSAPSPDPTITR	ASPGN(61.1)GSGHSSAPSPDPTITR	5	3	665	1
IPI00330594	86	PEWNKEPPSIIIRGSNNSGHDNHSQFVRETE	0.980933	17.1135	144.29	144.29 _GSN(de)NSGHDN(de)HSQFVR_	GSN(0.981)N(0.019)SGHDN(1)HSQ	GSN(17.11)N(-17.11)SGHDN(58.	3	2	829	1
IPI00122977	356	DHTIKAKVGQIQISHNISFVIVVPVFPKHQI	1	68.2426	68.243	68.243 _VGQIQISHN(de)ISFVIVVPVFPK_	VGQIQISHN(1)ISFVIVVPVFPK	VGQIQISHN(68.24)ISFVIVVPVFPK	9	3	775	1
IPI00222589	108	CVARDNVTGEEVRSTNASFNIKWIEAGPVVI	0.996402	24.4243	117.52	117.52 _STN(de)ASFNIK_	STN(0.996)ASFN(0.004)IK	STN(24.42)ASFN(-24.42)IK	3	2	492	1
IPI00222369	508	DVVEHATKTIFPNYFNGSEIVIAGKMVDKKF	0.997397	25.8331	136.39	136.39 _TIFPNYFN(de)GSEIVIAGK_	TIFPN(0.003)YFN(0.997)GSEIVIA	TIFPN(-25.83)YFN(25.83)GSEIV	8	2	936	1
IPI00314132	408	PGFPDINSAVTYRVTNSSEFMMNKDIMITAV	0.999999	61.9101	91.914	91.914 _VTN(de)SSEFM(ox)M(ox)NK_	VTN(1)SSEFMMNK	VTN(61.91)SSEFMMN(-61.91)K	3	2	661	1
IPI00153810	172	CGGISYRDWSQNMYFNCSEDNPSRERCSVPY	0.99999	50.4024	191.21	191.21 _DWSQNM(ox)YFN(de)CSEDNPSR_	DWSQNMYFN(1)CSEDNPSR	DWSQN(-61.34)MYFN(50.4)CSEDN	9	2	1084	1
IPI00114634	166	NGSWEVVPGHNRTWANYSECIKFMTNETRER	1	105.46	105.46	105.46 _TWAN(de)YSECIK_	TWAN(1)YSECIK	TWAN(105.46)YSECIK	4	2	637	1
IPI00129253	135	QYRIAIKDGYAVANTNTSDVWKKGGSEENIC	0.923778	10.8348	128.96	128.96 _DGYAVANTN(de)TSDVWK_	DGYAVAN(0.076)TN(0.924)TSDVWK	DGYAVAN(-10.83)TN(10.83)TSDV	9	2	821	1
IPI00128641	347	FKDNTCIKNAIQAFGNGSDVTMWQPAPPVQT	0.5	0	59.567	59.567 _N(de)AIQAFGNGSDVTM(ox)WQPAPPVQTTTATTTAFR_	N(0.5)AIQAFGN(0.5)GSDVTMWQPAP	N(0)AIQAFGN(0)GSDVTMWQPAPPVQ	8	4	875	1
IPI00122973	267	IYIEIGGQMPTQESTNSSDSVSATAIVEVTE	1	97.3515	97.352	97.352 _IYIEIGGQM(ox)PTQESTN(de)SSDSVSATAIVEVTEEFDR_	IYIEIGGQMPTQESTN(1)SSDSVSATAI	IYIEIGGQMPTQESTN(97.35)SSDSV	16	4	956	1
IPI00133292	42	IGTDFWYEYRSPIQENSSDSNKIAWEDFIGD	0.757373	5.01893	55.884	55.884 _SPIQEN(de)SSDSNKIAWEDFIGDEADEKTYNDVIFR_	SPIQEN(0.757)SSDSN(0.238)KIAW	SPIQEN(5.02)SSDSN(-5.02)KIAW	6	4	984	1
IPI00471090	172	ATVAPHTATIAAGTVNTSDPHTRTPSPAKST	1	124.399	124.4	124.4 _TAM(ox)AATVAPHTATIAAGTVN(de)TSDPHTR_	TAMAATVAPHTATIAAGTVN(1)TSDPHT	TAMAATVAPHTATIAAGTVN(124.4)T	20	3	894	1
IPI00311808	249	DQIPVFVTMSQKNDRNISDEIFIRDIPIVFD	0.897067	9.40269	79.492	79.492 _NDRN(de)ISDEIFIR_	N(0.103)DRN(0.897)ISDEIFIR	N(-9.4)DRN(9.4)ISDEIFIR	4	2	747	1
IPI00130271	213	FPGHNYTTRNIIPTMNGSCTFHKTWDPQCSI	0.999981	47.1539	72.089	72.089 _NIIPTM(ox)N(de)GSCTFHK_	NIIPTMN(1)GSCTFHK	N(-47.15)IIPTMN(47.15)GSCTFH	7	3	546	1
IPI00122973	185	EITFTVIASRGDHGANFSCRTEIDIRPQGIA	1	54.4709	136.96	54.471 _GDHGAN(de)FSCR_	GDHGAN(1)FSCR	GDHGAN(54.47)FSCR	6	2	561	1
IPI00113726	2027	MREKGRKAREIAAAANESAVKTIEDVIAISI	1	106.174	106.17	106.17 _EIAAAAN(de)ESAVK_	EIAAAAN(1)ESAVK	EIAAAAN(106.17)ESAVK	7	2	588	1
IPI00114950	492	ANMVFQNCSCIRSSGNSSAVIGICKKGPDCA	1	91.6203	91.62	91.62 _SSGN(de)SSAVIGICK_	SSGN(1)SSAVIGICK	SSGN(91.62)SSAVIGICK	4	2	641	1
IPI00113480	365	IMPFDSIHDDPCIITNRSARIPCFIAGDMRS	1	60.9184	138.57	60.918 _AIM(ox)PFDSIHDDPCIITN(de)R_	AIMPFDSIHDDPCIITN(1)R	AIMPFDSIHDDPCIITN(60.92)R	17	3	711	1
IPI00122592	274	PRIYPPSSIPQGAEYNISAIVTIATKTFIRY	1	52.0897	52.09	52.09 _IYPPSSIPQGAEYN(de)ISAIVTIATK_	IYPPSSIPQGAEYN(1)ISAIVTIATK	IYPPSSIPQGAEYN(52.09)ISAIVTI	14	4	634	1
IPI00125182	96	IRQQTARRPSIKAAGNSSAIGGQGTSGQPQR	1	139.444	139.44	139.44 _AAGN(de)SSAIGGQGTSGQPQR_	AAGN(1)SSAIGGQGTSGQPQR	AAGN(139.44)SSAIGGQGTSGQPQR	4	2	873	1
IPI00882216	8	________MNCFQGTNASAIEKDIGPEQFPI	1	93.5545	108.72	108.72 _MNCFQGTN(de)ASAIEK_	MNCFQGTN(1)ASAIEK	MN(-93.55)CFQGTN(93.55)ASAIE	8	3	525	1
IPI00461861	145	IVAVQKAKTIYRSCINESAIDSRGGQPIIKI	1	88.0206	122.33	88.021 _SCIN(de)ESAIDSR_	SCIN(1)ESAIDSR	SCIN(88.02)ESAIDSR	4	2	627	1
IPI00453501	159	IGRYAYVRGGGGPWANGSAIAICQRYYHRGH	1	73.4977	73.498	73.498 _GGGGPWAN(de)GSAIAICQR_	GGGGPWAN(1)GSAIAICQR	GGGGPWAN(73.5)GSAIAICQR	8	2	837	1
IPI00410951	176	VKTIYETEVFSTDFSNVSAAQHKINSYVEKQ	1	86.4533	86.453	86.453 _TIYETEVFSTDFSN(de)VSAAQHK_	TIYETEVFSTDFSN(1)VSAAQHK	TIYETEVFSTDFSN(86.45)VSAAQHK	14	3	792	1
IPI00153749	400	VSISNRIAIYNSRFINICSEMDGRVRPIVYT	0.993471	21.823	99.044	99.044 _IAIYNSRFIN(de)ICSEM(ox)DGRVR_	IAIYN(0.007)SRFIN(0.993)ICSEM	IAIYN(-21.82)SRFIN(21.82)ICS	10	3	811	1
IPI00230108	90	KGIVPIAKVDCTANTNTCNKYGVSGYPTIKI	0.801805	6.88831	73.877	73.877 _VDCTANTN(de)TCNK_	VDCTAN(0.164)TN(0.802)TCN(0.0	VDCTAN(-6.89)TN(6.89)TCN(-13	8	2	700	1
IPI00458105	112	RGIRTFKTVFAEHISNECKRRFYKNWHKSKK	1	78.9027	78.903	78.903 _TVFAEHISN(de)ECKR_	TVFAEHISN(1)ECKR	TVFAEHISN(78.9)ECKR	9	3	531	1
IPI00119063	2943	QDDCGDGSDERGCHVNECISRKISGCSQDCE	1	106.291	106.29	106.29 _GCHVN(de)ECISR_	GCHVN(1)ECISR	GCHVN(106.29)ECISR	5	2	617	1
IPI00121190	56	IGTFEDHFISIQRMYNNCEVVIGNIEITYVQ	0.499522	0	60.489	60.489 _M(ox)YN(de)NCEVVIGNIEITYVQR_	MYN(0.5)N(0.5)CEVVIGN(0.001)I	MYN(0)N(0)CEVVIGN(-27.18)IEI	3	3	778	1

Table S5. Identified deglycosylated glycopeptides

PNP Strategy 1

De-glycopeptides Sequence	Mass	Proteins	Leading r	Unique (G	Unique (P	Charges	PEP	Score
YYQSIYDTAENIN(deVTYPEWQSPEEDINFVTDTAK	4042.8273	IPI00118674	IPI00118674	yes	yes	4	0.0059081	46.415
YYN(deYTISINGK	1334.6507	IPI00221426	IPI00221426	yes	yes	2	3.5626E-34	199.82
YYN(deQSAGGSHTIQWMAGCDVESDGR	2788.1606	IPI00985828	IPI00985828	yes	no	2,3,4	8.9548E-80	214.14
YYN(deFSVFQIDAPSFAYIK	2172.0568	IPI00377727	IPI00377727	yes	no	2,3	2.9196E-06	123.02
YYHGEISYIN(deVTRK	1741.8788	IPI00404551	IPI00404551	yes	no	2,3,4	1.8766E-32	194.59
YYHGEISYIN(deVTR	1613.7838	IPI00404551	IPI00404551	yes	no	2,3	3.7582E-43	203.7
YWGN(deITVVMGEHDFSEKDGDEQVR	2810.2606	IPI00307890	IPI00307890	yes	yes	3	0.014194	60.602
YWGIHIQAFVQN(deGTVSK	1947.0003	IPI00222967	IPI00222967	yes	no	3	0.0035835	75.316
YVYVADIIAHEIHVIEKQPNMN(deITQIK	3178.6849	IPI00310567	IPI00310567	yes	yes	3,4	7.9393E-22	124.69
YVVVSTPEKSQITISNIDVN(deVDPGTYVCNATNAQGTTR	4111.0168	IPI00408495	IPI00408495	yes	no	4	1.5572E-14	89.99
YVMIPVADQDKCVVHYEN(deSTVPEKK	2948.4412	IPI00409148	IPI00409148	yes	yes	3,4	3.5201E-24	146.15
YTSFEYPKNISFACNPGFFIN(deGTSSSK	3062.412	IPI00322463	IPI00322463	yes	no	2,3,4	1.587E-88	211.64
YTSFEYPKN(deISFACNPGFFINGTSSSK	3062.412	IPI00322463	IPI00322463	yes	no	2,3,4	1.587E-88	211.64
YTSDPN(deVTSVGPSK	1450.694	IPI00229992	IPI00229992	yes	yes	2	0.0018208	106.42
YTGN(deASAIIIIPDQGR	1687.8893	IPI00131830	IPI00131830	yes	no	2,3	1.6755E-59	185.27
YTGN(deASAIFIIPDQGR	1721.8737	IPI00135635	IPI00135635	yes	no	2,3	5.6048E-05	133.48
YSVQHMYFTYN(deISDTEHFPNAISK	2891.3225	IPI00469218	IPI00469218	yes	yes	2,3,4	2.4947E-34	170.14
YSQAAN(deSTKEIDDCEQANK	2170.9437	IPI00187353	IPI00187353	yes	yes	2	5.2847E-32	184.83
YSNESQDISVNGYN(deCTTSSVSSAIR	2738.209	IPI00322304	IPI00322304	yes	yes	3	2.8527E-14	120.33
YSN(deESQDISVNGYNCTTSSVSSAIR	2738.209	IPI00322304	IPI00322304	yes	yes	3	2.8527E-14	120.33
YSIYKDPAGWISINPIN(deGTVDTTAVIDR	3078.5662	IPI00123746	IPI00123746	yes	yes	3	1.0281E-25	133.15
YRAEFAVAN(deDTGFVDIPQQEK	2397.1601	IPI00128154	IPI00128154	yes	no	2,3	2.9192E-15	140.28
YQTVDSSNIDGFVN(deCTK	1946.868	IPI00468814	IPI00468814	yes	yes	2,3	1.7955E-14	161.35
YQTINC(deSVNVR	1352.6507	IPI00420835	IPI00420835	yes	no	2	0.019282	69.815
YQMDN(deVSSIVQIIGSHIEDVNADIIQTK	3129.5652	IPI00330594	IPI00330594	yes	yes	3,4	7.4347E-49	176.9
YQHVGQVIIFQAPEAGGRWN(deQTQK	2754.399	IPI00894972	IPI00894972	yes	no	4	7.8116E-07	93.684
YQFNTNVVFSDN(deGTIVDRYR	2407.1557	IPI00321375	IPI00321375	yes	no	3	0.026005	63.337
YQFNTNVVFSDN(deGTIVDR	2087.9912	IPI00321375	IPI00321375	yes	no	3	1.6886E-11	135.09
YQFEVICPAPRPGAASN(deISFQAPFR	2822.3963	IPI00311159	IPI00311159	yes	yes	3	1.9596E-05	84.975
YQEIIEIESSIN(deKTQWK	2108.079	IPI00990932	IPI00990932	yes	no	2	4.1899E-14	162.77
YQEGDITIYVINIHN(deVTK	2119.095	IPI00170098	IPI00170098	yes	no	2,3	5.5086E-11	153.36
YPTPGEAPGVVGNFN(deK	1645.81	IPI00112614	IPI00112614	yes	yes	3	0.012355	117.07
YPQDYQFYIQN(deFTAIPINTVVPPQR	3011.5181	IPI00110852	IPI00110852	yes	no	2,3,4,5	1.5762E-25	149.44
YPNN(deGSIVWGK	1233.6142	IPI00848693	IPI00848693	yes	yes	2	0.0062013	83.081
YPHKPEIN(deSTTHPGADIKENFCR	2710.2922	IPI00114206	IPI00114206	yes	yes	3	6.5881E-09	114.55
YPHKPEIN(deSTTHPGADIK	2004.0065	IPI00114206	IPI00114206	yes	yes	2,3	1.3155E-14	163.8
YN(deHTGQVVIYK	1320.6826	IPI00466371	IPI01026704	no	no	2,3	2.6863E-11	167.2
YMN(deYTSIITFHCK	1676.7691	IPI00308971	IPI00308971	yes	yes	3	0.0025961	127.56
YMGIPIPEDNIDHYRN(deSTVMSR	2607.221	IPI00125813	IPI00125813	yes	no	2,3	3.4764E-08	106.16
YMDQNSDGWQDGVGYIN(deSSEGAVGR	2704.146	IPI00114236	IPI00114236	yes	no	3	8.2446E-08	97.509
YMAIAIN(deR	950.50077	IPI00123814	IPI00123814	yes	yes	2	0.010808	110.63
YKYETTISPTSNISSNSFICIEN(deR	2823.3385	IPI00625835	IPI00625835	yes	no	3,4	8.5795E-80	184.68
YKYETTISPTSN(deISSNSFICIENR	2823.3385	IPI00625835	IPI00625835	yes	no	3,4	8.5795E-80	184.68
YKGTAGNAIMDGASQIVGEN(deR	2151.0379	IPI00279079	IPI00279079	yes	yes	2,3	9.2831E-59	207.43
YKGN(deASAIFIIPDQGR	1748.921	IPI00128076	IPI00128076	yes	yes	2,3	0.0001963	131.18
YKGIN(deITEDTYKPR	1696.8784	IPI00119809	IPI00119809	yes	yes	2,3,4	0.014851	100.38
YIYIASN(deHSNK	1308.6463	IPI00118173	IPI00118173	yes	no	2,3	0.0038245	91.313
YITMGGKN(deFTR	1286.6441	IPI00848991	IPI00848991	yes	no	2	0.0093052	92.866
YIQVVYIHNNN(deISAVGQNDFCR	2623.2602	IPI00123196	IPI00123196	yes	yes	3	2.2358E-25	163.79
YIQPIIAVQFTN(deITVDTEIRVECK	2849.4997	IPI00121550	IPI00121550	yes	yes	3	0.0024792	70.452
YIQPIIAVQFTN(deITVDTEIR	2333.2631	IPI00121550	IPI00121550	yes	yes	2,3	4.4772E-204	288.25
YINSFPHEN(deSTAFYGVGNQFSYIFPEEFK	3374.556	IPI00453524	IPI00453524	yes	yes	3	1.0847E-42	163.74
YIN(deFSTSEKEK	1344.6561	IPI00135010	IPI00135010	yes	no	2	0.0070005	97.214
YIN(deETQQITQKIK	1605.8726	IPI00123223	IPI00123223	yes	yes	2	0.0078744	110.51
YIN(deETQQITQK	1364.6936	IPI00123223	IPI00123223	yes	yes	2,3	6.3784E-11	162.68
YIMWGISSDIWGEKPN(deTSYIIGK	2657.32	IPI00323624	IPI00323624	yes	no	3	0.0001424	88.565
YIKNGN(deATIIR	1261.7143	IPI00985828	IPI00109996	no	no	2,3	8.699E-16	170.89
YIKNGN(deATIIR	1261.7143	IPI00985828	IPI00109996	no	no	2,3	8.699E-16	170.89
YIHEN(deYTK	1066.5084	IPI00127407	IPI00127407	yes	no	2	0.0127	104.2
YIEIGN(deETIIR	1319.7085	IPI00850057	IPI00850057	yes	no	2	2.3485E-13	160.22
YHYN(deGTIIDGTIFDSSYSR	2208.0124	IPI00130486	IPI00130486	yes	yes	2,3	5.3619E-08	123.19
YHYN(deGTIIDGTAFDNSYSR	2192.9763	IPI00122493	IPI00122493	yes	no	2,3	7.1162E-11	139.11
YHYN(deGTFIDGTIFDSSHNR	2243.0032	IPI00130486	IPI00130486	yes	yes	2,3	5.4453E-115	248.97
YHYN(deGTFEDGKK	1457.6575	IPI00122493	IPI00122493	yes	no	2	0.0044575	102.4
YHYN(deGTFEDGK	1329.5626	IPI00122493	IPI00122493	yes	no	2	0.040739	60.255
YHYN(deASIIDGTIIDSTWNIGK	2380.1699	IPI00130486	IPI00130486	yes	yes	2,3	0.0097125	61.276
YHHYSSN(deFSIPK	1478.6943	IPI00129485	IPI00129485	yes	no	2,3	9.3441E-24	187.85
YHGFIN(deTSYHR	1393.6527	IPI00154056	IPI00154056	yes	no	3	0.0027716	96.113
YHDYYITSTSN(deGSIEGIENR	2318.0451	IPI00130754	IPI00130754	yes	yes	3	0.0013354	81.157
YGTAIVHIYVNETIAN(deR	1933.0058	IPI00719927	IPI00719927	yes	no	2,3	0.0001309	112.8
YGTAIVHIYVN(deETIANR	1933.0058	IPI00719927	IPI00719927	yes	no	2,3	0.0001309	112.8
YGQEQGTAPFQVSN(deHTGR	1975.9137	IPI00409393	IPI00409393	yes	yes	2,3	2.4576E-05	117.3
YGMQNSGNEAAWN(deYTIEQYQK	2494.0859	IPI00134585	IPI00134585	yes	yes	2,3	1.5762E-204	202.16
YGKN(deDSITITQIK	1479.7933	IPI00123428	IPI00123428	yes	no	2,3	1.4066E-42	201.48
YGIVVIDECPGVGIVIPQSFGN(deESIR	2817.4371	IPI00309230	IPI00309230	yes	no	2,3	5.3732E-26	149.41
YGHPNGTQGN(deSTMWPVFTSTEQK	2566.1547	IPI00131168	IPI00131168	yes	yes	3	1.2955E-08	108.18
YGHPN(deGTQGNSTMWPVFTSTEQK	2566.1547	IPI00131168	IPI00131168	yes	yes	3	1.2955E-08	108.18
YFTNRI(deDVSQNVSSDTDQSCESTK	2780.2195	IPI00120769	IPI00120769	yes	no	3	0.0064041	65.855
YFFIQSVDSDGRN(deFTSSPPGQTQFK	2852.3406	IPI00344686	IPI00344686	yes	yes	3	3.5902E-33	167.51
YEVDWNQSTIDCVDPISSIAAN(deR	2652.2126	IPI00119459	IPI00331032	no	no	2,3	1.5848E-18	142.23
YEVDWN(deQSTIDCVDPISSIAANR	2652.2126	IPI00119459	IPI00331032	no	no	2,3	1.5848E-18	142.23
YEQIQN(deETR	1179.552	IPI00154056	IPI00154056	yes	no	2	0.0065213	106.29
YEQAKN(deISQDIEK	1564.7733	IPI00400016	IPI00400016	yes	no	2	0.0070182	131.96

YDVN(deRSDSGGSIQIEEGYFVHHFAPENIPTMSK	3723.7264	IPI00312711	IPI00312711	yes	no	3,4		8.653E-54	162.47
YDNHSSSVIAAVVFEHSFN(deHSQDPIPIAVK	3307.6262	IPI00153807	IPI00153807	yes	no		4	0.0001822	61.612
YDN(deHSSSVIAAVVFEHSFNHSQDPIPIAVK	3307.6262	IPI00153807	IPI00153807	yes	no		4	0.0001822	61.612
YDKSEDVGAAAMIN(deYTHIIMEAVPGHPAIYR	3431.6642	IPI00874858	IPI00874858	yes	no	4,5		1.3635E-17	110.94
YDIPASINYIIN(deK	1522.8031	IPI00129265	IPI00129265	yes	yes	2,3		2.2592E-23	186.46
YDIIIINENGIIISN(deVSEPATAR	2514.333	IPI00229935	IPI00229935	yes	yes		3	3.098E-06	96.379
YAVYWN(deSSNPR	1355.6258	IPI00114364	IPI00114364	yes	no		2	0.0021036	99.215
YATENDISSIHNSTITCIVN(deQTTSITGTSPEIMEK	3854.819	IPI00230319	IPI00230319	yes	no	3,4,5		2.7171E-26	115.47
YATENDISSIHN(deSTITCIVNQTTSITGTSPEIMEK	3854.819	IPI00230319	IPI00230319	yes	no	3,4,5		2.7171E-26	115.47
YAFQEIIGDISFIIPTIN(deFSKYIR	2847.5211	IPI00122557	IPI00122557	yes	no		3	0.0026787	100.28
YAFQEIIGDISFIIPTIN(deFSK	2415.2726	IPI00122557	IPI00122557	yes	no	2,3		1.0722E-58	202.16
WVITAAHCIIYPPWDKN(deFTENDIIVR	3170.6012	IPI00114206	IPI00114206	yes	yes	3,4		2.0936E-05	81.665
WVGN(deWTYEIK	1294.6346	IPI00153143	IPI00153143	yes	yes		2	0.0042009	98.898
WVDGASIN(deFSHWNSGEPNDSR	2374.0363	IPI00223352	IPI00223352	yes	yes		3	0.0006262	82.865
WTGHN(deVTVVQR	1295.6735	IPI00119063	IPI00119063	yes	yes	2,3		0.0038245	91.313
WTDNTEYN(deNTIPIRGEER	2207.0243	IPI00276430	IPI00276430	yes	yes	2,3		0.0001353	112.82
WSFSN(deGTSWQK	1326.5993	IPI00315576	IPI00315576	yes	yes		2	0.0014542	103.43
WKPPSDPNGN(deITHYIVYWER	2471.2022	IPI00128358	IPI00128358	yes	no		3	1.7778E-05	107.72
WIIVGAPTASWISN(deASVVNPGAIYR	2641.4017	IPI00121334	IPI00121334	yes	no		3	0.0010075	69.259
WGHN(deVTEFQQR	1400.6585	IPI00754386	IPI00754386	yes	yes	2,3		0.0046836	88.338
WGEVDIIGN(deCSQFYPDRYYEK	2638.1798	IPI00320675	IPI00320675	yes	yes		3	0.02272	62.203
WGEVDIIGN(deCSQFYPDR	2054.9156	IPI00320675	IPI00320675	yes	yes		2	1.2822E-10	154.49
WFHN(deESIIPHQDANYVIQSAR	2524.2248	IPI00162870	IPI00162870	yes	yes		3	0.0023516	72.421
WECKN(deDTIFGIK	1509.7286	IPI00126186	IPI00126186	yes	yes		2	0.0031429	124.12
WDPEPN(deCTSK	1232.5132	IPI00130010	IPI00130010	yes	no		2	0.045548	62.408
WASVVVPIGKEQN(deYTCR	2006.0044	IPI00109996	IPI00109996	yes	no	2,3		6.2904E-08	145.1
WAAVVVPIGKEQN(deYTCHVHHK	2472.2485	IPI00850057	IPI00850057	yes	no		3	0.0020874	81.428
VYVYAVN(deQTR	1211.6299	IPI00466371	IPI01026704	no	no		2	0.0061629	94.692
VYTYADTPNDFQISN(deFSIPEEDTKIKIPIIHQAIK	4048.0833	IPI00108811	IPI00108811	yes	yes		4	2.9129E-07	71.927
VYTYADTPNDFQISN(deFSIPEEDTKIK	3034.4448	IPI00108811	IPI00108811	yes	yes	3,4		1.7971E-46	180.63
VYTYADTPNDFQISN(deFSIPEEDTK	2793.2657	IPI00108811	IPI00108811	yes	yes	2,3		4.8428E-29	157.01
VYTVSSSPSCYMYVYVN(deTTEVAIEQDIAYIQEIK	3962.8846	IPI00136012	IPI00136012	yes	yes		4	1.3754E-113	215.08
VYSIPGREN(deYSSVDANGIQSQMISR	2770.3344	IPI00127100	IPI00127100	yes	yes		3	8.1416E-25	149.85
VYN(deGSVPFEER	1295.6146	IPI00265291	IPI00265291	yes	no		2	0.0029912	95.094
VYMKN(deVTVVIR	1320.7588	IPI00469218	IPI00469218	yes	yes	2,3		5.2957E-05	147.91
VYIN(deDSVEISRNENK	1778.8799	IPI00126769	IPI00126769	yes	yes		2	0.000132	139.6
VYIN(deDSVEISR	1293.6565	IPI00126769	IPI00126769	yes	yes		2	0.022243	68.355
VYIHPFHIIYHN(deK	1679.8936	IPI00654069	IPI00654069	yes	yes	2,3		0.0027206	112.13
VYGGIVN(deQSEINEGTAFFR	2100.0276	IPI00416285	IPI00416285	yes	yes	2,3		4.0052E-65	211.09
VYEEVINV(deTPNDGFAK	1793.8836	IPI00624896	IPI00624896	yes	no		2	0.0029413	80.545
VWVCDRDNDCVDGSDEPAN(deCTQMTCGVDEFR	3706.4487	IPI00119063	IPI00119063	yes	yes	3,4		7.4716E-30	127.53
VWVCDRDN(deDCVDGSDEPANCTQMTCGVDEFR	3706.4487	IPI00119063	IPI00119063	yes	yes	3,4		7.4716E-30	127.53
VWPDYPN(deITVDPSIGWDHQVEQYR	2913.3722	IPI00848693	IPI00848693	yes	yes	3,4		1.3107E-24	144.27
VWPDGVIPFVIGGN(deFTGSQR	2145.1007	IPI00125182	IPI00125182	yes	no	2,3		6.6588E-13	143.83
VWN(deQTEQEPAAYHIISICFVR	2560.2533	IPI00129079	IPI00129079	yes	no		3	4.9123E-23	160.55
VWDTAAAIN(deR	1115.5724	IPI00226563	IPI00226563	yes	no		2	0.0079796	90.827
VVTPEEYFN(deVTIQ	1537.7664	IPI00133103	IPI00133103	yes	no	2,3		3.2488E-10	162.32
VVSVDISFRPIN(deETFPVVYIETPKR	2904.5749	IPI00624663	IPI00624663	yes	yes	3,4,5		8.5805E-182	187.59
VVSVDISFRPIN(deETFPVVYIETPK	2748.4738	IPI00624663	IPI00624663	yes	yes	2,3,4		2.2613E-169	264.92
VVRPDSEIGERPPEDN(deQSFQYDHEAFIGK	3358.5854	IPI00137831	IPI00137831	yes	yes	3,4		2.2061E-06	88.79
VVPEPNIVIN(deFSATAIR	1839.0254	IPI00654185	IPI00654185	yes	no		3	1.76E-06	130.27
VVN(deVSEIYGTPCTK	1565.7759	IPI00626950	IPI00626950	yes	no	2,3		4.6365E-104	240.2
VVN(deSTTGTGEHIR	1369.695	IPI00131223	IPI00131223	yes	no		3	0.0029448	109.84
VVMDIPYEIWN(deETSAEVADIKK	2549.2723	IPI00119039	IPI00119039	yes	no	2,3,4		1.1415E-12	130.89
VVMDIPYEIWN(deETSAEVADIK	2421.1774	IPI00119039	IPI00119039	yes	no	2,3		9.1308E-15	135.38
VVIRPFYITN(deSTDMV	1753.9073	IPI00119809	IPI00119809	yes	no		2	6.184E-46	209.79
VVINFN(deGTSQEIMAVSEHR	2130.0528	IPI00111183	IPI00111183	yes	yes		3	3.9836E-06	114.59
VVIISGVEPRPPTPQVQFTIN(deASSEDHKR	3200.6942	IPI00122973	IPI00122973	yes	no	4,5		0.0008354	70.994
VVIHPN(deHSVVDIGIIK	1739.0094	IPI00409148	IPI00409148	yes	yes	2,3,4		1.0873E-146	259.96
VVIGEN(deITSNCPEVIYEIKEETPVFYK	3169.5893	IPI00674255	IPI00674255	yes	yes		3	9.675E-22	123.53
VVIAGSN(deMTICCMSPTK	1867.8664	IPI00119299	IPI00119299	yes	no	2,3		4.2273E-06	124.78
VVFISPAVPEEPEAYN(deITVIIR	2455.3363	IPI00405742	IPI00405742	yes	no	2,3,4		4.4966E-31	171.98
VVDKGN(deGSKPTSPEEVK	1769.9159	IPI00330632	IPI00330632	yes	no	2,3		0.0006528	114.63
VVAVSPAN(deISREER	1525.8213	IPI00229992	IPI00229992	yes	yes		3	0.017981	75.739
VVAVSPAN(deISR	1111.635	IPI00229992	IPI00229992	yes	yes		2	0.001462	103.34
VTTCHIPQQN(deATIYK	1772.888	IPI00410951	IPI00410951	yes	no	2,3		0.0005311	108.63
VTPVCN(deASIPAQR	1411.7242	IPI00762091	IPI00762091	yes	no	2,3		0.0035524	96.745
VTNSNANAAGPIIVAGYN(deVSGSVR	2330.1979	IPI00222429	IPI00222429	yes	yes	2,3		1.6259E-40	145.61
VTN(deSSEFMMNK	1286.5635	IPI00314132	IPI00314132	yes	no		2	0.0024139	91.914
VTIWVHPFVNYN(deSSSFGEGVER	2523.2183	IPI00464256	IPI00464256	yes	yes		3	1.9257E-09	116.73
VTINNWVAN(deKTEGR	1600.8322	IPI00136642	IPI00136642	yes	yes	2,3		4.6385E-32	190.41
VTINNWVAN(deK	1157.6193	IPI00136642	IPI00136642	yes	yes		2	0.000416	123.51
VTIDFN(deITNPENGPVIDDAIPNSVHGHIPFAK	3440.7365	IPI00466371	IPI00466371	yes	yes		3	1.0381E-10	93.166
VTIDFN(deITDPENGPVIDDAIPNSVHGHIPFAK	3441.7205	IPI01026704	IPI01026704	yes	yes	3,4,5		3.1894E-54	167.72
VTAEVTTHGTN(deTSTPTTR	1872.9177	IPI00471090	IPI00471090	yes	yes		2	0.0005141	93.495
VSTVTIVSATSTTAN(deMTMSPEGR	2340.1301	IPI00406603	IPI00406603	yes	yes		3	0.0001531	80.752
VSTIYANN(deGSVIQGSTVASVYHKR	2550.319	IPI00121114	IPI00121114	yes	yes	3,4		1.4458E-05	87.156
VSTIYANN(deGSVIQGSTVASVYHK	2394.2179	IPI00121114	IPI00121114	yes	yes	2,3,4		7.5507E-237	295.69
VSQVIHEGGHN(deVTKIIYESANIPDFRK	3050.5938	IPI00463764	IPI00463764	yes	no		4	0.0433	53.747
VSQVIHEGGHN(deVTK	1503.7794	IPI00463764	IPI00463764	yes	no	2,3,4		5.2942E-70	220.59
VSNYIGQAN(deQSAWITVIPK	2088.1004	IPI00828688	IPI00828688	yes	no		3	8.2066E-09	129.88
VSN(deVSCEASVSK	1265.5922	IPI00135560	IPI00135560	yes	no		2	0.01518	69.602
VSITN(deVSISDEGR	1375.6943	IPI00856723	IPI00856723	yes	no		2	0.002182	133.47
VSIQEIPGSEHIEMIAN(deATTIAYIKR	2883.5164	IPI00124428	IPI00124428	yes	yes	3,4		0.0009465	70.917
VSIQEIPGSEHIEMIAN(deATTIAYIK	2727.4153	IPI00124428	IPI00124428	yes	yes	2,3,4		2.2808E-33	165.1
VSGQMHMQN(deITFQTEASVAQQEKEFK	2995.4168	IPI00312711	IPI00312711	yes	no		3	5.6808E-06	86.557

VSGQMHMQN(deITFQTEASVAQQEK	2591.2108	IPI00312711	IPI00312711	yes	no		3	3.1995E-49	198.35
VSFYFFVTSPQN(deVSDVIPR	2201.1157	IPI00153202	IPI00153202	yes	no	2,3		0.0019104	81.157
VSEDGYSIYMSN(deITK	1705.7869	IPI00129646	IPI00129646	yes	no		2	0.000818	103.39
VRIDPPCTN(deTTAPSNYINNPYVR	2661.2969	IPI00658539	IPI00658539	yes	no		3	8.0371E-15	132.74
VREAN(deITEDQIIFFPK	1919.0153	IPI00122122	IPI00122122	yes	no	2,3		1.3776E-26	190.11
VQVWPGPSGYSSITAIEN(deSTDGK	2392.1547	IPI00315576	IPI00315576	yes	yes	2,3		4.602E-19	148.29
VQSIQTIAAN(deNSAIAK	1627.8893	IPI00129304	IPI00129304	yes	yes	2,3		3.3667E-34	190.46
VQPMTASN(deWTIVMEGEWMIK	2350.116	IPI00453829	IPI00453829	yes	no		3	4.8516E-09	127.97
VQPIVAVADEGWYIIQN(deK	2042.0837	IPI00111163	IPI00111163	yes	no		3	2.7171E-38	199.15
VQPIASSTIIHSDITSVYGTVVMN(deR	2687.3953	IPI00674255	IPI00674255	yes	yes	3,4		3.3904E-14	116.43
VQPFN(deVTK	931.51272	IPI00222967	IPI00222967	yes	no		2	0.042736	73.665
VQGGSSVWGSVITHN(deSSAITYQSWGR	2763.3365	IPI00453977	IPI00453977	yes	no	3,4		9.1881E-17	126.86
VQGAWPIIDNDFIFVQATSSPVSSGAN(deATTIR	3361.6943	IPI00380441	IPI00380441	yes	yes		4	2.6515E-30	125.54
VPNNAIEGIEN(deITAIYIHHNEIQEVGSSMR	3347.6568	IPI00120187	IPI00120187	yes	yes		4	2.9474E-05	71.002
VPMMVQSGN(deISYFR	1627.7851	IPI00116105	IPI00116105	yes	yes	2,3		0.0008948	111.79
VPGN(deITSVIISNIVPR	1677.9778	IPI00221547	IPI00221547	yes	no		2	0.0013253	95.347
VPFIFNINPATTN(deFTGSCQPQSAQIR	2907.4338	IPI00222967	IPI00222967	yes	no	3,4		5.1566E-08	94.389
VNYEGGTWDWIAEAISSN(deHTR	2405.1036	IPI00134585	IPI00134585	yes	yes	2,3		4.7672E-21	153.65
VNRFN(deSTEYQVVTR	1711.8642	IPI00119063	IPI00119063	yes	no	2,3		5.7574E-23	182.02
VNNREIVSDPIYVEVQGIPYFIKQP(deESVNVTR	3701.9417	IPI00121254	IPI00121254	yes	yes		4	0.0332	57.902
VNGTDIAPDIIN(deGSQIIIR	2008.0953	IPI00131693	IPI00131693	yes	no		2	1.9519E-25	137.73
VNGEEDFSWFGYSIHGVTVAN(deR	2483.1506	IPI00225715	IPI00225715	yes	yes		3	0.0007462	79.467
VNDNK(deTAAEEAIR	1429.7161	IPI00400016	IPI00400016	yes	no		2	1.0409E-22	183.96
VNCEERNVTGIEN(deFTIK	2021.984	IPI00378224	IPI00378224	yes	yes	2,3		0.011244	73.833
VNCEERN(deVTGIENFTIK	2021.984	IPI00378224	IPI00378224	yes	yes	2,3		0.011244	73.833
VNASTTDPN(deSTVEQSAITR	1989.9603	IPI00338785	IPI00338785	yes	no	2,3		0.0036002	85.61
VN(deYTRAEEIFSR	1483.7419	IPI00131168	IPI00131168	yes	yes		2	0.015255	79.466
VN(deKTEEDYAR	1223.5782	IPI00133103	IPI00133103	yes	no		2	6.5518E-09	154.15
VN(deITVIPSITSR	1298.7558	IPI00323857	IPI00323857	yes	no		2	0.0012571	130.56
VN(deISFPSAQSIPASDTHIK	2011.0375	IPI00624663	IPI00624663	yes	yes	2,3		1.693E-79	220.82
VN(deGTKEPIEFK	1260.6714	IPI00345112	IPI00345112	yes	no		2	0.014709	86.114
VN(deGTITQVIIVGAPTHDDVSK	2163.1535	IPI00225715	IPI00225715	yes	yes	2,3		3.4539E-59	207.43
VN(deGTDIAPDIINGSQIIIR	2008.0953	IPI00131693	IPI00131693	yes	no		2	1.9519E-25	137.73
VMVVITDGDIFGDPIN(deITTVINSPK	2657.3986	IPI00119181	IPI00119181	yes	no		3	0.0024846	62.589
VMSWWDYGYQITAMAN(deR	2090.9343	IPI00109108	IPI00109108	yes	yes	2,3		6.2231E-89	212.39
VMSWWDYGYQIAGMAN(deR	2046.908	IPI00316469	IPI00316469	yes	yes	2,3		5.2644E-75	220.21
VKPTPPYN(deISVTNSEEISSIIK	2415.2897	IPI00120155	IPI00120155	yes	yes		3	1.3884E-30	171.47
VIYIPAYN(deCTIRPVSKR	2049.1194	IPI00469387	IPI00469387	yes	no		4	0.0076302	93.348
VIYIPAYN(deCTIRPVSK	1893.0182	IPI00469387	IPI00469387	yes	no	2,3		0.0051433	89.624
VIWTINPNHISN(deGTIAAPVVVIPDIDEDGVR	3323.7514	IPI00466999	IPI00466999	yes	no	3,4,5		4.6595E-06	78.978
VIVPPAPPSCSIQGVPYVGTN(deVTINCK	2866.4721	IPI00126827	IPI00126827	yes	no	3,4		2.6001E-06	83.871
VIVAPPSEEAN(deTTK	1454.7617	IPI00875419	IPI00875419	yes	no		2	0.0021524	102.9
VITNQESPYQN(deHTGR	1742.8336	IPI00125310	IPI00125310	yes	no	2,3		9.181E-12	164.15
VITMANQVITVN(deISEEGR	1973.0252	IPI00987265	IPI00987265	yes	yes		3	1.4184E-25	159.69
VITIAN(deFTTK	1106.6336	IPI00109727	IPI00109727	yes	yes		2	0.0007097	119.27
VISNN(deCTSYGVIDIGK	1738.856	IPI00331214	IPI00331214	yes	yes	2,3		4.094E-12	161.99
VISN(deNCTSYGVIDIGK	1738.856	IPI00331214	IPI00331214	yes	yes	2,3		4.094E-12	161.99
VISIAQAHSIN(deFSCEQVR	2058.0317	IPI00308990	IPI00308990	yes	yes		3	1.2259E-05	119.56
VIQQIAMGDSAAQN(deK	1572.793	IPI00321717	IPI00321717	yes	no		3	0.0014251	94.717
VIPFN(deVTDYCQIVR	1722.8763	IPI00828222	IPI00828222	yes	no	2,3		7.8371E-56	210.64
VINN(deITNDIR	1170.6357	IPI00128178	IPI00128178	yes	no		2	0.0012718	113.69
VINDTWAWKN(deATIAEQAK	2058.0534	IPI00123342	IPI00123342	yes	yes	2,3		9.1884E-198	283.49
VINADQGTSATVQMIIN(deDTCPIFVR	2762.3731	IPI00320065	IPI00320065	yes	yes	3,4		6.2482E-16	121.25
VIN(deITDNTYFK	1326.682	IPI00130117	IPI00130117	yes	yes		2	0.0013003	106.01
VIN(deDTWAWKNATIAEQAK	2058.0534	IPI00123342	IPI00123342	yes	yes	2,3		9.1884E-198	283.49
VIN(deDTWAWK	1131.5713	IPI00123342	IPI00123342	yes	yes		2	0.031398	73.985
VIKDAVNN(deITAK	1284.7402	IPI00626793	IPI00626793	yes	no		2	0.0057513	105.65
VIIRPYITPNNQGIYIFQGN(deSTVR	2762.4868	IPI00319509	IPI00319509	yes	yes	2,3,4		7.3498E-257	222.91
VIIN(deITTVAANHGYTK	1713.9414	IPI00467600	IPI00467600	yes	no	2,3		1.9363E-58	218.28
VIIIIDPAISGN(deETEPYPAFTR	2415.2686	IPI00848693	IPI00848693	yes	yes	2,3		1.7564E-31	177.08
VIIIAQTHAN(deNTGSYHCYYK	2352.1321	IPI00273133	IPI00273133	yes	yes		3	0.0070479	66.022
VIHIQFNSISSITDDTFCKAN(deDTR	2781.3392	IPI00120848	IPI00120848	yes	yes	3,4		8.5825E-16	128.38
VIFYKDDAMVYN(deVTSR	1919.9451	IPI00406901	IPI00406901	yes	no	2,3		0.012009	74.769
VIFKN(deYSINDATITIHNIGFSDSGK	2753.4024	IPI00471187	IPI00471187	yes	no		3	2.3845E-19	133.74
VIETIPANYSIN(deSSK	1634.8516	IPI00480532	IPI00480532	yes	no	2,3		1.8781E-09	160.48
VIETIPAN(deYSINSSK	1634.8516	IPI00480532	IPI00480532	yes	no	2,3		1.8781E-09	160.48
VIEQSYN(deATWIGR	1535.7732	IPI00762242	IPI00762242	yes	yes		2	0.0035622	81.904
VIENEKFDTHEYHN(deESR	2145.9716	IPI00463492	IPI00463492	yes	yes	2,3		9.273E-08	142.39
VIEEFYN(deQTWNHR	1734.8114	IPI00308691	IPI00308691	yes	no	2,3		0.0025965	124.45
VIDIWDIAQSAN(deFTEKEIESFREEIK	3109.5608	IPI00469307	IPI00469307	yes	no		4	0.019489	60.735
VIDIWDIAQSAN(deFTEK	1848.9258	IPI00469307	IPI00469307	yes	no	2,3		6.8534E-06	141.85
VIAQHQNIIFAN(deSSSSMR	2002.0054	IPI00319040	IPI00319040	yes	no		3	3.3977E-05	115.38
VHSGN(deFSTIPQYFK	1623.8045	IPI01023131	IPI01023131	yes	no	2,3		0.012559	69.345
VGYQSQN(deISCFFR	1604.7406	IPI00464256	IPI00464256	yes	yes		2	2.9766E-05	150.12
VGVNKN(deQTVTATFGYPFR	1998.0323	IPI00114256	IPI00114256	yes	no	2,3,4		2.7249E-93	216.72
VGQIQISHN(deISFVIVVPVFPK	2320.3307	IPI00122977	IPI00122977	yes	yes		3	0.0046102	68.243
VGKNEAVIAWDQIPVDDQNGFIRN(deYSISYR	3466.727	IPI00120155	IPI00120155	yes	yes		4	0.0003539	68.689
VGIVTYSN(deETR	1237.6303	IPI00855103	IPI00855103	yes	no		2	0.0003375	129.84
VGATAAVYSAAIIEYITAEVIEIAGN(deASK	2894.5277	IPI00555055	IPI00555055	yes	no	2,3		3.1265E-48	171.08
VFVYTPTTN(deYTIR	1573.814	IPI00124428	IPI00124428	yes	yes	2,3		3.4051E-07	111.94
VFPYISVMVNN(deGSISYDHSKDGR	2584.238	IPI00321634	IPI00321634	yes	yes	3,4,5		2.0787E-09	116.06
VFPYISVMVNN(deGSISYDHSK	2256.0885	IPI00321634	IPI00321634	yes	yes	2,3,4		3.5593E-96	232.15
VFPYISAMVNN(deGSISYDHER	2298.0739	IPI00830356	IPI00830356	yes	no	2,3,4		8.389E-11	136.2
VFNGKDN(deISK	1120.5877	IPI00331214	IPI00331214	yes	yes		2	0.012231	98.898
VFKTN(deSTQVSDVR	1479.7682	IPI00469426	IPI00469426	yes	no	2,3		8.3039E-05	150.11
VFKPQSGADAIN(deDSQDFPFPETPAK	2705.2973	IPI00462465	IPI00462465	yes	no	3,4		1.856E-55	192.27

VFIVPVGN(deHSNIPFSR	1781.9577	IPI00221418	IPI00221418	yes	no	2,3	4.1748E-12	161.82
VFIQGYQIHIASICN(deGSK	2034.0357	IPI00112614	IPI00112614	yes	yes	3	0.00043	95.662
VFGSQN(deITTVKIK	1433.8242	IPI00123342	IPI00123342	yes	yes	3	0.0098362	81.92
VFGSQN(deITTVK	1192.6452	IPI00123342	IPI00123342	yes	yes	2	0.0022776	98.407
VFDIHQNMGSVN(deVSVGCTPAQIIETSR	2958.4328	IPI00116913	IPI00116913	yes	no	3	1.6689E-24	132.36
VETGVIKPGMVVTFAPVN(deVTTEVK	2514.3767	IPI00307837	IPI00307837	yes	yes	3,4	7.5682E-29	158.52
VETGEN(deCTSPAPK	1388.6242	IPI00469221	IPI00469221	yes	no	2	0.0055572	78.814
VEFDDKGNVITSYGNPIIIN(deSSIPEDATIK	3248.6453	IPI00122257	IPI00122257	yes	no	3	2.5447E-46	151.19
VDVIVAN(deITVTDKDQPHTPAWNAAYR	2893.4723	IPI00323134	IPI00323134	yes	no	3	1.1748E-07	95.624
VDNA(deSIVADDMRNAYISIEGK	2280.1056	IPI00356462	IPI00356462	yes	no	2	0.018058	65.022
VDIPQQPMGIIAVAN(deDTNSCEISPCR	2884.3517	IPI00119063	IPI00119063	yes	yes	3	0.0011941	66.3
VDIEDFEN(deNTAYAK	1627.7366	IPI00551169	IPI00551169	yes	no	2	2.6064E-28	134.88
VDFIWHPEVN(deGSMK	1657.7923	IPI00380296	IPI00380296	yes	yes	3	0.011626	69.716
VDDEMPQHAVISGPNIFINNINKTDN(deGTYR	3371.6205	IPI00856723	IPI00856723	yes	no	3,4	1.0161E-10	95.666
VDDEMPQHAVISGPNIFINNIN(deKTDNGTYR	3371.6205	IPI00856723	IPI00856723	yes	no	3,4	1.0161E-10	95.666
VDDEMPQHAVISGPNIFINNIN(deK	2564.2693	IPI00856723	IPI00856723	yes	no	3	1.2097E-22	159.75
VDCTANTN(deTCNK	1396.5711	IPI00230108	IPI00230108	yes	no	2	0.009647	73.877
VAQPGINYAIGTN(deTSYPNNIIR	2375.2234	IPI00226360	IPI00226360	yes	no	2,3	3.3918E-31	174.04
VAN(deITVVVNSIDGK	1427.7984	IPI00310797	IPI00310797	yes	yes	3	0.0012499	131.32
VAEVENGTKPDSDVPEHCIDTWSFDAATMDHN(deGTMIFFK	4410.9508	IPI00128484	IPI00128484	yes	yes	4	1.3431E-08	63.848
VAEVEN(deGTKPDSDVPEHCIDTWSFDAATMDHNGTMIFFK	4410.9508	IPI00128484	IPI00128484	yes	yes	4	1.3431E-08	63.848
VAAN(deFSTPVISTSDSSNPGQER	2263.0717	IPI00227797	IPI00227797	yes	no	3	3.7485E-09	112.44
TYEN(deGSSVEYR	1303.5681	IPI00122117	IPI00122117	yes	yes	2	0.0050957	86.911
TYEDESPEWFQEDDAVQPN(deSSYTYVWHATKR	3777.6496	IPI00406603	IPI00406603	yes	yes	4	2.6071E-16	105.03
TYEDESPEWFQEDDAVQPN(deSSYTYVWHATK	3621.5485	IPI00406603	IPI00406603	yes	yes	3	2.265E-59	170.65
TYCANEPISNCSQVN(deR	1911.8203	IPI00127770	IPI00127770	yes	yes	2	2.4908E-12	165.58
TYCANEPISN(deCSQVNR	1911.8203	IPI00127770	IPI00127770	yes	yes	2	2.4908E-12	165.58
TYAVYDIFDTAMINN(deSR	1992.9251	IPI00226790	IPI00226790	yes	no	2,3	2.2577E-28	188.41
TYADDVNSEIVNIYTFN(deHTVTR	2571.2241	IPI00330714	IPI00330714	yes	no	2,3	1.2774E-39	189.34
TWAN(deYSECIK	1270.5652	IPI00114634	IPI00114634	yes	yes	2	0.002365	105.46
TVYIHAN(deQTGYPDPSSR	1904.9017	IPI00119035	IPI00119035	yes	yes	2	0.0052529	70.942
TVVVDN(deETVATR	1302.6779	IPI00115231	IPI00115231	yes	no	2	0.0023943	99.941
TVVTEAGNIIKDN(deATQEEIIHYIEK	2827.4604	IPI00321190	IPI00321190	yes	no	2,3,4	8.1428E-142	248.46
TVVQYSPN(deIIFSGIEEIQKDSCESNQNITCR	3628.7138	IPI00466371	IPI01026704	no	no	3,4	3.9405E-11	98.491
TVVAPSTEGGIN(deITSTFIR	1962.0422	IPI00114252	IPI00114252	yes	no	2,3	1.8242E-12	141.83
TVSTIVIQAAN(deVSAIYK	1776.9986	IPI00124326	IPI00124326	yes	no	2,3	1.1251E-27	181.51
TVNSIN(deVSAISIPEHDEADDISDENREK	3096.4483	IPI00121378	IPI00121378	yes	no	3	2.3785E-38	159.98
TVNSIN(deVSAISIPEHDEADDISDENR	2839.3108	IPI00121378	IPI00121378	yes	no	3	3.4917E-20	132.26
TVN(deMTTIIIGR	1217.6802	IPI00356462	IPI00356462	yes	no	2	0.0056273	84.479
TVN(deITRDNIIDDVISFIR	2103.1324	IPI00230718	IPI00230718	yes	no	3	5.3642E-25	138.39
TVIQFVAN(deISK	1218.6972	IPI00626137	IPI00626137	yes	no	2	0.0007247	112.43
TVIDKNGDAYGYYN(deDSIK	2034.9535	IPI00471081	IPI00471081	yes	yes	2,3	2.9343E-37	196.64
TVFAEHISN(deECKR	1589.762	IPI00458105	IPI00458105	yes	yes	3	0.010836	78.903
TVDKSTGKPTIYN(deVSIIMSDTGGTCY	2807.3357	IPI00468055	IPI00468055	yes	yes	3	8.0758E-29	155.86
TVAN(deISGCEATDSEAIIHCIR	2316.0838	IPI00154043	IPI00154043	yes	yes	2,3,4	9.6617E-140	251.35
TTVKNITVIEPVTQPFIQVTN(deTTVK	2770.548	IPI00108535	IPI00108535	yes	no	2,3,4	1.9549E-55	192.27
TTVKN(deITVIEPVTQPFIQVTNTTVK	2770.548	IPI00108535	IPI00108535	yes	no	2,3,4	1.9549E-55	192.27
TTIVDNNTWN(deNSHIAIVGK	2096.0651	IPI00316469	IPI00316469	yes	yes	2,3	0	361.2
TTIVDN(deNTWNNSHIAIVGK	2096.0651	IPI00316469	IPI00316469	yes	yes	2,3	0	361.2
TSYCN(deESFYFICKK	1845.8066	IPI00126186	IPI00126186	yes	yes	3	7.4982E-16	171.32
TSYCN(deESFYFICK	1717.7116	IPI00126186	IPI00126186	yes	yes	2,3	4.5876E-21	171.73
TSSEFCQPVSIHGSN(deHTSK	2101.9487	IPI00894972	IPI00894972	yes	no	2,3	1.3488E-06	119.08
TSRPTDNSIN(deVTCGPPYETNGPK	2504.1602	IPI00323053	IPI00323053	yes	no	3	9.7455E-26	167.63
TSN(deITDTQIAEAYIYR	1857.9109	IPI00319200	IPI00319200	yes	no	2	2.9966E-18	177.56
TSIIVIN(deNTESNIEDFIQSIK	2377.2377	IPI00170146	IPI00170146	yes	yes	2,3	0	276.68
TSIDIWMHTDPMSQKN(deESVK	2346.0984	IPI00118674	IPI00118674	yes	yes	3	2.6627E-05	101.05
TSICDSDAN(deCTIGSSDTHSSGIGTGR	2655.1137	IPI00471089	IPI00471089	yes	no	3	0.0002731	73.981
TSDTGEEEAITN(deSTEANGKYVVVSTPEK	2955.3833	IPI00408495	IPI00408495	yes	no	3,4	3.9988E-179	265.13
TSDTGEEEAITN(deSTEANGK	1952.8447	IPI00408495	IPI00408495	yes	no	2,3	1.0474E-287	312.86
TR(deCNTTQGNEVTSIIR	1848.9112	IPI00134743	IPI00134743	yes	no	2	0.0073518	81.594
TQVFGPVDPTVN(deTTYAFFNTFFK	2640.29	IPI00115530	IPI00115530	yes	yes	3	4.9518E-38	143.91
TQN(deGSIPAVTR	1142.6044	IPI00121240	IPI00121240	yes	yes	2	0.003308	93.623
TQMVFFNITPNIIN(deDTMEIR	2396.1868	IPI00127856	IPI00127856	yes	yes	2,3	7.6642E-33	183.22
TQMVFFN(deITPNIINDTMEIR	2396.1868	IPI00127856	IPI00127856	yes	yes	2,3	7.6642E-33	183.22
TQITCSIN(deSSGVDIVGHR	1942.9531	IPI00408495	IPI00408495	yes	no	2,3	1.3352E-91	234.38
TQDSIIQAETQANSCN(deITVVTIQESIEKK	3247.6242	IPI00321222	IPI00321222	yes	yes	3,4	7.8761E-32	145.54
TPVIN(deCSVAVCK	1346.6686	IPI00120674	IPI00120674	yes	no	2	0.0087538	75.088
TPTIPPVSFHIGGVWFN(deITGQDYVIK	2885.5116	IPI00113797	IPI00113797	yes	yes	3	0.0014044	65.207
TPMTN(deSSIR	1005.4913	IPI00221426	IPI00221426	yes	yes	2	0.0023838	119.53
TPASDPHGDN(deITYSVFYTK	2111.98	IPI00129159	IPI00129159	yes	no	2,3	4.6929E-08	124.18
TNSTQ(deVSDVR	1105.5364	IPI00469426	IPI00469426	yes	no	2	0.022512	72.2
TNIEGQVPFIPAN(deFTAHITPYKEFYHYR	3352.6669	IPI00129903	IPI00129903	yes	yes	4	0.01089	55.469
TNIEGQVPFIPAN(deFTAHITPYK	2457.2692	IPI00129903	IPI00129903	yes	yes	3,4	8.1967E-39	184.6
TN(deSSFIQGFVDHVKEDCDRIGPGVSDICK	3279.5289	IPI00321190	IPI00321190	yes	no	4	0.015193	55.986
TN(deSSFIQGFVDHVKEDCDR	2253.012	IPI00321190	IPI00321190	yes	no	2,3,4	3.3717E-79	223.29
TN(deSSFIQGFVDHVK	1577.7838	IPI00321190	IPI00321190	yes	no	2,3	1.0912E-43	205.62
TN(deQSCEIVIDSTEK	1622.7458	IPI00310059	IPI00310059	yes	no	2	0.0013067	128.74
TN(deKSEMWDVFCYR	1734.7494	IPI00467600	IPI00467600	yes	no	2,3	0.0095022	100.38
TN(deITSIIVQPISPSIVK	1809.0612	IPI00985989	IPI00985989	yes	no	3	0.000104	114.27
TMVFPVMYIN(deESVIIDKETANQIK	2782.4285	IPI00127447	IPI00127447	yes	yes	2,3,4	6.2499E-94	224.86
TMVFPVMYIN(deESVIIDK	1998.0206	IPI00127447	IPI00127447	yes	yes	3	1.885E-272	223.7
TMTSGDPGETAN(deATEVKK	1835.8571	IPI00114844	IPI00114844	yes	yes	2,3	0.0050806	78.95
TMTSGDPGETAN(deATEVK	1707.7621	IPI00114844	IPI00114844	yes	yes	2	8.1563E-05	115.73
TMTIIEAGN(deNTMK	1422.6847	IPI00131116	IPI00131116	yes	yes	2	0.0037967	78.486
TMFN(deSTEIK	1069.5114	IPI00677395	IPI00677395	yes	no	2	0.012344	85.958
TKTQVFGPVDPT(deVNTTYAFFNTFFK	2869.4327	IPI00115530	IPI00115530	yes	yes	3	1.1996E-55	195.03

TKDISHSIYMFFN(deTSDIR	2174.0466	IPI00114457	IPI00114457	yes	yes	3	0.0038218	86.378
TIYN(deWSGYPIIVHK	1689.8879	IPI00471081	IPI00471081	yes	yes	2,3	0.0001389	143.46
TIYETEVFSTDFSN(deVSAAQHK	2373.1125	IPI00410951	IPI00410951	yes	yes	3	0.0003788	86.453
TITVYN(deFSAPVITISQIEVSEGSQVTVK	3009.591	IPI00122973	IPI00122973	yes	no	2,3,4	2.1894E-26	136.34
TITIINVTRN(deDTGPYVCETR	2322.1638	IPI00108535	IPI00108535	yes	no	2,3	1.9423E-96	238.19
TITIIN(deVTRNDTGPYVCETR	2322.1638	IPI00108535	IPI00108535	yes	no	2,3	1.9423E-96	238.19
TITIIN(deVTR	1029.6182	IPI00108535	IPI00108535	yes	no	2	5.1421E-09	148.56
TISPTGN(deISSAPK	1271.6721	IPI00263505	IPI00263505	yes	no	2	0.0032713	107.53
TISDVPSAAPQN(deISIEVR	1895.9953	IPI00129159	IPI00129159	yes	no	2	1.5191E-28	186.97
TIPHHQYN(deATINK	1535.7845	IPI00348266	IPI00348266	yes	yes	2,3	0.0002115	145.81
TIN(deWSAAEPGAWATKVEWDEHK	2525.1975	IPI00331440	IPI00331440	yes	no	3	1.0934E-10	121.44
TIN(deWSAAEPGAWATK	1601.7838	IPI00331440	IPI00331440	yes	no	2,3	1.021E-24	183.23
TIMSAEANIAGIFPPNEVQHFNPN(deISWQPIPVHTVPITEDR	4579.2958	IPI00154056	IPI00154056	yes	no	4,5	4.8568E-19	99.357
TIIVDNNTWN(deNTHISR	1896.9442	IPI00109108	IPI00109108	yes	yes	2,3	0	277
TIIVDN(deNTWNNTHISR	1896.9442	IPI00109108	IPI00109108	yes	yes	2,3	0	277
TIISYYN(deQSAGGSHTIQVISGCEVGSDGR	3055.4305	IPI00474494	IPI00474494	yes	no	3	5.619E-38	151.42
TIIPAAAQDVYYRDEIGN(deVSTSHIIIIDDSVEMEIRPR	4299.1845	IPI00309035	IPI00309035	yes	yes	4,5	2.9611E-11	77.233
TIIGYYN(deQSNDESHTIQWMYGCDVGPDGR	3375.4561	IPI00322542	IPI00322542	yes	yes	3,4	4.416E-38	153.17
TIIGYYN(deQSESGSHTIQWMYGCK	2722.2156	IPI00608044	IPI00608044	yes	no	3,4	0.000143	82.586
TIIGYYN(deQSAGGTHTIQWMYGCDVGSDGR	3206.4186	IPI00109996	IPI00109996	yes	yes	3,4	7.6254E-66	194.97
TIGISPFHEFADVVFTAN(deDSGHR	2516.2084	IPI00127560	IPI00127560	yes	yes	2,3,4	5.3422E-103	234.38
TIFPNYFN(deGSEIVIAGK	1868.9672	IPI00222369	IPI00222369	yes	yes	2	5.2949E-07	136.39
TIEQERN(deASFVYTKAIMAYAFAIAGNQNK	3248.6288	IPI00123223	IPI00123223	yes	yes	4	0.019157	53.747
TIEQERN(deASFVYTK	1684.842	IPI00123223	IPI00123223	yes	no	2,3	2.2197E-55	210.41
TIEDIIFRAEN(deR	1475.7732	IPI00122312	IPI00122312	yes	no	3	0.0066466	99.568
TIAGEN(deQTAIEIEEINR	1899.9538	IPI00400016	IPI00400016	yes	no	2,3	8.0274E-109	239.58
TGVNSGVMIMN(deMTR	1509.7102	IPI00856861	IPI00856861	yes	no	2,3	0.0006772	110.98
TGVHDGDFEYN(deITTTIAAINK	2279.107	IPI00108849	IPI00108849	yes	no	3	3.8166E-15	138.66
TGTIAIQN(deTTQIR	1415.7732	IPI00623114	IPI00623114	yes	no	2	0.0002907	143.94
TGHTNAHGK(deDIDTDFTSNASQPETK	2671.211	IPI00130573	IPI00130573	yes	yes	3	2.9597E-24	141.78
TGHRPPNPAFWWVN(deGSGTEVK	2336.145	IPI00221556	IPI00221556	yes	no	3	0.014996	66.874
TGEIN(deITSIVDR	1316.6936	IPI00380460	IPI00380460	yes	no	2	0.001894	106.58
TGEIFTFN(deTTAAQPQYFKYEFPDGVDSVIVK	3511.7188	IPI00330714	IPI00330714	yes	no	3,4	9.2496E-47	155.8
TGEIFTFN(deTTAAQPQYFK	2063	IPI00330714	IPI00330714	yes	no	2,3	3.6208E-28	182.49
TGEAN(deITQIYTQEAIDFIQTQHAR	2747.3515	IPI00310090	IPI00310090	yes	no	2,3,4	2.3565E-148	254.4
TGDGSNVTSN(deFTKDPSIIPEIIPGVSYTVK	3248.6816	IPI00469426	IPI00469426	yes	no	3	2.6368E-07	87.489
TGDGSN(deVTSNFTKDPSIIPEIIPGVSYTVK	3248.6816	IPI00469426	IPI00469426	yes	no	3	2.6368E-07	87.489
TFDVSNFQN(deSSIKR	1641.8111	IPI00272690	IPI00272690	yes	no	2	0.015625	73.156
TFAVYIN(deSTGYR	1390.6881	IPI00268030	IPI00268030	yes	yes	2	0.0096685	73.848
TEVSNNNVIIYIDQVTN(deQTIAFSFIIQQDIPVR	3791.9734	IPI00123223	IPI00123223	yes	yes	3,4	3.2828E-06	73.389
TEVKPVATEQIISTFITVGN(deNTCFYGK	3016.5216	IPI00122494	IPI00122494	yes	yes	3	0.0018759	67.979
TEVIIQGTVSPN(deASAPDAVWEDYEFK	2865.3709	IPI00405437	IPI00405437	yes	no	3	0.0059569	57.145
TEQIPVN(deK	927.50255	IPI00276430	IPI00276430	yes	yes	2	0.013937	100.02
TEQIIIQAPN(deTSVHIYEPYPAGADVPFGPPINK	3575.83	IPI00132600	IPI00132600	yes	no	3,4	1.7174E-54	166.22
TEKDPQNDPVGTCYISTEN(deFTR	2571.1547	IPI00115976	IPI00115976	yes	yes	2,3	1.6923E-85	219.87
TEIDIRPQGIAIFSN(deVSEAR	2215.1597	IPI00122973	IPI00122973	yes	no	2,3,4	1.1053E-19	169.25
TEGMIIN(deVTSSIR	1419.7392	IPI00109254	IPI00109254	yes	yes	2	0.0025755	115.06
TEFN(deITSYSSKR	1431.6994	IPI00855103	IPI00855103	yes	yes	2	0.047838	64.55
TEAGVFEYVADPTFEN(deFTGGVKK	2505.2064	IPI00405742	IPI00405742	yes	no	2,3,4	1.498E-103	238.8
TEAGVFEYVADPTFEN(deFTGGVK	2377.1114	IPI00405742	IPI00405742	yes	no	2,3	5.4875E-39	186.45
TDVN(deSSRNPDTIAAWYIR	2078.0181	IPI00406603	IPI00406603	yes	yes	3	0.0002509	109.07
TDPANQFEWIENTIN(deSSIWNKEK	2763.314	IPI00136259	IPI00136259	yes	yes	3,4	0.0003519	83.849
TDDEVVQREEEAIQIDGIN(deASQIR	2727.3311	IPI00129526	IPI00129526	yes	yes	3,4	2.2355E-66	204.47
TDAEIN(deETARPISPVNPK	1951.0011	IPI00408895	IPI00408895	yes	yes	2	0.0001511	111.77
TCVSN(deCTASQFVCKNDK	2017.8656	IPI00119063	IPI00119063	yes	yes	2,3	9.9377E-05	119.23
TCVSN(deCTASQFVCK	1660.7007	IPI00119063	IPI00119063	yes	yes	2,3	3.0186E-56	216.27
TCPIDEFQCN(deNTICKPIAWK	2494.1443	IPI00119063	IPI00119063	yes	yes	3	4.4242E-06	115.73
TCPAGIMGEN(deNTIVWK	1789.8491	IPI00121190	IPI00121190	yes	no	2,3	1.3625E-125	254.1
TASVSIN(deQTEPPKVR	1625.8737	IPI00129220	IPI00129220	yes	yes	2,3	5.1316E-17	174.59
TASVSIN(deQTEPPK	1370.7042	IPI00129220	IPI00129220	yes	yes	2	0.0025413	119.96
TAQSYYN(deQSK	1188.5411	IPI00312980	IPI00312980	yes	no	2	3.818E-45	202.65
TANDAIQDMICDMEGIPQKHN(deFSHCCGK	3276.3879	IPI00130654	IPI00130654	yes	no	4	0.0056402	57.238
TAN(deETSAEAYNIIIR	1664.837	IPI00400016	IPI00400016	yes	no	2,3	9.5447E-147	266.45
TAMAATVAPHTATIAAGTVN(deTSDPHTR	2662.3133	IPI00471090	IPI00471090	yes	yes	3	3.007E-22	124.4
TAIWVATDHNTDN(deTSAIIR	2098.0443	IPI00169870	IPI00169870	yes	yes	2,3	1.6911E-41	192.6
TAIVQEVHQN(deFSAWCSQVIR	2372.1695	IPI00381303	IPI00381303	yes	yes	2,3	1.3058E-10	133.78
TAIFPDIIVQGN(deASIR	1713.9414	IPI00124078	IPI00124078	yes	yes	3	0.0002429	112.15
TAGWNIPMGIIAN(deQTR	1741.8934	IPI00323235	IPI00323235	yes	yes	2,3	7.8962E-35	197.75
TAGAN(deGTSGFFCVDEGGIPIAQR	2324.0855	IPI00282289	IPI00282289	yes	no	3,4	4.9156E-26	166.29
TAFITN(deFTITIDGVTYPGNVK	2271.1787	IPI00124725	IPI00124725	yes	no	2	1.5149E-14	132.01
TAFAPPDIPVCIIGN(deR	1739.9029	IPI00331175	IPI00331175	yes	no	3	5.7847E-06	143.25
TAASIIWQAYPIIN(deISEK	2017.0884	IPI00115867	IPI00115867	yes	yes	2,3	2.2835E-16	170.16
TAADATGIQPIINQFTPAN(deVSR	2284.1812	IPI00120245	IPI00120245	yes	no	2,3	5.2667E-86	221.25
SYSSIQQDIFQFQKN(deQTSIEKK	2646.3289	IPI00128905	IPI00128905	yes	yes	3	1.6569E-08	115.34
SYSSIQQDIFQFQKN(deQTSIEK	2518.234	IPI00128905	IPI00128905	yes	yes	3	3.5562E-14	137.61
SYSFIN(deSSIIGR	1342.6881	IPI00110389	IPI00110389	yes	yes	2	0.001235	129.37
SYN(deDSVDPR	1051.4571	IPI00113480	IPI00113480	yes	no	2	0.0065494	106.2
SYIFIYDGNKNSTTTDQN(deFTSAK	2614.2187	IPI00124830	IPI00124830	yes	no	2,3	1.0647E-38	183.07
SYIFIYDGNKN(deSTTTDQNFTSAK	2614.2187	IPI00124830	IPI00124830	yes	no	2,3	1.0647E-38	183.07
SYIDDRPIQHYIAVSSPTNTTYVVQYAIAN(deITGK	3797.9265	IPI00118674	IPI00118674	yes	yes	3	1.2937E-34	125.59
SYIDDRPIQHYIAVSSPTN(deTTYVVQYAIANITGK	3797.9265	IPI00118674	IPI00118674	yes	yes	3	1.2937E-34	125.59
SYCKNGVN(deGTGENGRK	1739.8009	IPI00132474	IPI00132474	yes	yes	2,3	1.3333E-145	265.12
SYCKNGVN(deGTGENGR	1611.706	IPI00132474	IPI00132474	yes	yes	2,3	0.0051622	116.63
SWVQGN(deITACGR	1347.6354	IPI00153389	IPI00153389	yes	no	2	0.0048995	80.312
SWSTVGN(deCTAAIRWIERYYCFQGNK	3066.4229	IPI00128484	IPI00128484	yes	yes	3	2.3222E-05	88.396
SWSTVGN(deCTAAIR	1421.6721	IPI00128484	IPI00128484	yes	yes	2	6.8038E-16	173.44

SVYN(deCSGEACSGHNR	1696.6682	IPI00314726	IPI00314726	yes	yes	2,3	9.1431E-25	183.96
SVVHHIN(deGSTIR	1318.7106	IPI00758066	IPI00758066	yes	no	2,3	0.0014947	111.12
SVN(deQSIIEIHK	1266.6932	IPI00230145	IPI00230145	yes	yes	2,3	0.0011409	113.24
SVKQEMNGTYVCHAFSSHGN(deVTR	2608.1911	IPI00122973	IPI00122973	yes	no	3	0.0071596	71.735
SVKQEMN(deGTYVCHAFSSHGNVTR	2608.1911	IPI00122973	IPI00122973	yes	no	3	0.0071596	71.735
SVIEN(deTTSYEEAKNTITK	2027.0059	IPI00125266	IPI00125266	yes	yes	2,3	0	366.58
SVIEN(deTTSYEEAK	1469.6886	IPI00125266	IPI00125266	yes	yes	2	0.0021804	133.48
SVGTGTNMVFQNCSCIGSSGN(deSSAVIGICK	3091.3831	IPI00625835	IPI00625835	yes	no	3	7.524E-31	130.78
SVGTGTNMVFQN(deCSCIGSSGNSSAVIGICK	3091.3831	IPI00625835	IPI00625835	yes	no	3	7.524E-31	130.78
SVAQN(deYSSITHIHSIGK	1840.9432	IPI00896674	IPI00896674	yes	yes	2,3	1.0677E-06	131.45
STTQTN(deSSDSHIVK	1503.7165	IPI00111807	IPI00111807	yes	no	2	0.0023451	90.444
STN(deASFNIK	980.49271	IPI00222589	IPI00222589	yes	no	2	0.0030728	117.52
STGKPTIYNVSII(deMSDTGGTCY	2364.0977	IPI00468055	IPI00468055	yes	yes	2,3	1.6384E-139	250.86
STFQN(deVTVIIAVITQK	1761.0036	IPI00121776	IPI00121776	yes	yes	2,3	2.3556E-189	238.07
STEVSN(deHTIK	1114.5619	IPI00272690	IPI00272690	yes	no	2	0.0021113	107.03
SSVEIIN(deSTQAPMR	1531.7664	IPI00120166	IPI00120166	yes	yes	2,3	0.0009804	133.48
SSTEN(deTSAEIHVIGR	1599.7853	IPI00330594	IPI00330594	yes	yes	2,3	7.2133E-08	155.22
SSSYHENDMENPQSN(deITMVYIIPIGPK	3063.4318	IPI00406603	IPI00406603	yes	yes	3	4.2247E-26	132.36
SSSHIPPSSYFN(deASGR	1692.7856	IPI00130661	IPI00130661	yes	yes	2,3	5.361E-90	238.02
SSQSN(deQTIWFGHFTTSTIMSPSPGIR	2866.3708	IPI00226714	IPI00226714	yes	no	3	5.583E-26	149.32
SSQSIIN(deSSNQK	1291.6368	IPI00621024	IPI00621024	yes	yes	2	0.0025229	95.981
SSGN(deSSAVIGICK	1278.6238	IPI00114950	IPI00114950	yes	yes	2	0.0029934	91.62
SSAN(deQSEFQQQIQK	1621.7696	IPI00990932	IPI00990932	yes	no	2,3	1.621E-86	235.22
SRYPHKPE(deINSTTHPGADIK	2247.1396	IPI00114206	IPI00114206	yes	yes	2,3	5.6225E-10	136.26
SRNTTVADTYN(deITDPEEFETEYPFFESR	3357.495	IPI00120225	IPI00120225	yes	no	3	6.3445E-06	81.974
SRN(deTTVADTYNITDPEEFETEYPFFESR	3357.495	IPI00120225	IPI00120225	yes	no	3	6.3445E-06	81.974
SRGYN(deSSQDIPSIVIDFVK	2124.0851	IPI00124666	IPI00124666	yes	yes	2,3	0.0023292	87.49
SQSPSIMYTINGFVN(deK	1784.8767	IPI00406603	IPI00406603	yes	yes	2,3	1.9572E-17	134.81
SQNDKFN(deVSITVK	1478.7729	IPI00466371	IPI01026704	no	no	2,3	8.6335E-33	199.31
SQN(deDTVTPDVWYTSKPEKK	2222.0855	IPI00471238	IPI00471238	yes	yes	3	8.2478E-06	119.23
SQIVN(deETHWQYYGTSDTR	2183.9872	IPI00749655	IPI00749655	yes	no	2,3	8.2137E-21	174.02
SQITISNIDVNVDPGTYVCN(deATNAQGTTR	3108.4782	IPI00408495	IPI00408495	yes	no	2,3	1.7218E-56	185.7
SQHIN(deSTDAADK	1285.5899	IPI00128336	IPI00128336	yes	yes	2	0.0025337	95.651
SQHIDN(deFSNQIGK	1486.7165	IPI00406603	IPI00406603	yes	yes	2	1.6915E-05	105.46
SQFVVSSN(deSSK	1168.5724	IPI00114810	IPI00114810	yes	yes	2	0.0054874	85.554
SPVR(deTEFNITSYSSK	1714.8526	IPI00855103	IPI00855103	yes	yes	3	0.0061199	87.447
SPVIIGQPNTIICFVDNIFPPVIN(deITWIR	3335.8104	IPI00172039	IPI00172039	yes	no	3,4	0.0017668	57.738
SPIQEN(deSSDSNKIAWEDFIGDEADEKTYNDVIFR	3931.8024	IPI00133292	IPI00133292	yes	no	4	0.0014338	55.884
SPGAQDN(deVSVSQGMR	1531.7049	IPI00348586	IPI00348586	yes	no	2,3	0.0004022	114.7
SPFYN(deISDQISFQCYDGYVIR	2571.174	IPI00114065	IPI00114065	yes	no	3	3.0523E-05	113.47
SPFIASVSDQHGIVYITENKN(deK	2446.2492	IPI00124326	IPI00124326	yes	no	3	0.013379	65.423
SPDKETFTCWWNPGSDGGIPTN(deYSITYSK	3307.4768	IPI00321091	IPI00321091	yes	no	3	0.0001497	76.161
SPANGSIGPMIN(deISSGESR	1872.9	IPI00112616	IPI00112616	yes	no	2	0.0001765	98.443
SPAN(deGSIGPMINISSGESR	1872.9	IPI00112616	IPI00112616	yes	no	2	0.0001765	98.443
SNKYSQ(deAANSTKEIDDCEQANK	2500.1136	IPI00187353	IPI00187353	yes	yes	4	2.3628E-08	112.82
SNKYSQ(deAANSTK	1297.6262	IPI00187353	IPI00187353	yes	yes	2	0.0030521	122.34
SNIDPSNVDSIFYAAQSSQVISGCEISVSN(deETK	3545.6468	IPI00475154	IPI00475154	yes	yes	3,4	3.1141E-06	73.389
SNF(deTPATNEAPQATVFPK	1918.9425	IPI00172039	IPI00172039	yes	no	2	6.991E-11	151.41
SNASDDPISISPGTPPPIINN(deSTHR	2586.2674	IPI00118250	IPI00118250	yes	yes	3	0.014152	52.03
SNASDDPISISPGTPPPIIN(deNSTHR	2586.2674	IPI00118250	IPI00118250	yes	yes	3	0.014152	52.03
SN(deYSVIDITPVAAIIPK	1800.0033	IPI00225072	IPI00225072	yes	no	2,3	1.0696E-60	214.81
SN(deVTRPSEFNYIWIAPIPFIK	2491.3264	IPI00319509	IPI00319509	yes	no	2,3,4	1.8671E-14	137.14
SN(deITVIR	801.47085	IPI00229516	IPI00229516	yes	no	2	0.0078227	105.57
SN(deISIIR	801.47085	IPI00323134	IPI00323134	yes	no	2	0.0031313	123.98
SMANQIITFN(deISSK	1552.7919	IPI00467600	IPI00467600	yes	yes	3	4.4739E-24	188.4
SMAGSGHN(deVSQEAIAIKR	1854.937	IPI00314673	IPI00314673	yes	yes	2,3,4	6.196E-06	122.01
SMAGSGHN(deVSQEAIAIK	1698.8359	IPI00314673	IPI00314673	yes	yes	2,3	3.3394E-11	158.98
SKN(deITIVEIPDEVIVPR	1921.0884	IPI00118830	IPI00118830	yes	yes	2,3	2.8088E-27	182.34
SKIDTEVAN(deISVVMEEMK	2022.0013	IPI00129304	IPI00129304	yes	yes	3	0.0002079	107.5
SITVIIGAHN(deK	1151.6663	IPI00230426	IPI00230426	yes	yes	2	7.2473E-11	161.62
SITQGSIIVGNIAPVN(deGTSQGK	2140.1488	IPI00515360	IPI00515360	yes	no	2,3	6.6654E-236	289.35
SITHDTDGVAVFVINIPSN(deVTVIK	2538.3694	IPI00330833	IPI00330833	yes	yes	3	4.6809E-39	176.1
SITFN(deESYQDVSEVVYGAK	2135.0059	IPI00136642	IPI00136642	yes	yes	2,3	0	317.58
SISN(deSTAR	834.41954	IPI00114733	IPI00114733	yes	yes	2	0.0048622	118.33
SISEN(deATATTEPK	1347.6518	IPI00944095	IPI00944095	yes	no	2	2.9266E-70	227.63
SISCQMAAFRGN(deGSER	1769.7937	IPI00129677	IPI00129677	yes	no	2,3	0.0045411	91.355
SIQT(deTSDQQNIKVPGNITSVIISNIVPR	3021.6459	IPI00221547	IPI00221547	yes	no	3	4.8959E-18	111.84
SIPVN(deDSVIDVFERR	1744.9108	IPI00122122	IPI00122122	yes	no	2,3	2.5135E-07	151.07
SIPVN(deDSVIDVFER	1588.8097	IPI00122122	IPI00122122	yes	no	2,3	7.8791E-07	154.46
SIPQSSWGN(deWSSWK	1648.7634	IPI00118291	IPI00118291	yes	no	2	0.0021776	102.64
SIPPGIFSTSAN(deISTIVIR	1972.0993	IPI00129250	IPI00129250	yes	yes	2,3	2.3376E-15	157.14
SIPNN(deVTSFEVESIKPYK	2051.0575	IPI00323053	IPI00323053	yes	no	2,3	1.8748E-60	211.06
SINGVIIPGGGAN(deITDSGYSR	2047.0334	IPI00129243	IPI00129243	yes	no	2	1.5001E-11	128.98
SIN(deVTGQGFSIIQK	1490.8093	IPI00405742	IPI00405742	yes	no	2,3	2.4494E-86	233.02
SIN(deGTFFGGWK	1212.5928	IPI00136938	IPI00136938	yes	yes	2	0.0015831	101.93
SIN(deCTVK	820.41129	IPI00417180	IPI00417180	yes	no	2	0.007312	109.11
SIIW(deTETFMNKENQNHSYSIK	2569.2271	IPI00120245	IPI00120245	yes	no	3	1.0941E-05	99.891
SIIIIGSPTWKN(deVSR	1669.9515	IPI00225715	IPI00225715	yes	yes	2,3	1.9125E-07	155.43
SIIEFN(deTTMGCQPSDSQHR	2206.9736	IPI00308971	IPI00308971	yes	yes	2,3	3.2904E-25	177.44
SIIDHIHVGVGRDN(deVSQPK	2070.097	IPI00123428	IPI00123428	yes	no	2,3,4	1.7489E-18	161.35
SIGPNTCSSN(deGSSIYFIHGPNIYCYSSIDKINAAK	3834.7982	IPI00128484	IPI00128484	yes	yes	4	2.3996E-13	94.152
SIGNVN(deFSVSAEAQQSSEPCGSEVATVPETGRK	3421.6056	IPI00123223	IPI00123223	yes	yes	3,4,5	4.3881E-88	203.79
SIGEVN(deFTATAEAIQSPEICGNKITEVPAIVHK	3522.8028	IPI00624663	IPI00624663	yes	yes	4	1.7905E-11	95.823
SIGEVN(deFTATAEAIQSPEICGNK	2435.1639	IPI00624663	IPI00624663	yes	yes	2,3,4	9.2767E-121	241.44
SIFN(deFSSCKGQK	1401.6711	IPI00420867	IPI00420867	yes	no	3	0.010252	83
SIFN(deFSSCK	1088.4961	IPI00420867	IPI00420867	yes	no	2	0.022692	80.763

SIFGAIPGN(deR	1030.556	IPI00400073	IPI00400073	yes	yes	2	0.00041	123.63
SIDNDNYVFTAPYFN(deK	1906.8737	IPI00626793	IPI00626793	yes	no	2	8.6627E-13	168.69
SIDEEAIKEN(deNSIHWK	1911.9327	IPI00123223	IPI00123223	yes	no	2,3,4	3.9068E-46	209.67
SIDAYPVINQAQAMEN(deHTEVHFQK	2769.3181	IPI00468674	IPI00468674	yes	no	3,4	9.2315E-16	122.49
SICING(deKWDPEPNCTSK	2004.9033	IPI00130010	IPI00130010	yes	no	2,3	0.0008982	106.93
SHTNTSHVMQYGN(deK	1602.7209	IPI00130627	IPI00130627	yes	yes	2,3	8.2521E-16	173.19
SHTN(deTSHVMQYGNK	1602.7209	IPI00130627	IPI00130627	yes	yes	2,3	8.2521E-16	173.19
SHPIHSPVN(deWTSYASSVESTPTK	2511.203	IPI00129215	IPI00129215	yes	yes	3	1.901E-05	90.729
SHGIWN(deNTVFIFSTDNGGQTR	2350.1091	IPI00652358	IPI00652358	yes	no	3	0.0012912	78.78
SGTIFDNFIITNDEAYAEEFGN(deETWGVTK	3267.4884	IPI00123639	IPI00123639	yes	yes	3,4	1.5829E-56	185.84
SGSEKWN(deFSVGHFEIR	1878.9013	IPI00131000	IPI00131000	yes	no	3	0.0067256	83.729
SGQVEVN(deITEFPYTPTPEHGR	2357.1288	IPI00311405	IPI00311405	yes	yes	3	0.0036998	69.763
SGQEDHYWIDVEKN(deQSAK	2132.9763	IPI00319509	IPI00319509	yes	no	2,3,4	4.5354E-109	243.86
SGDASIN(deVTNIQISDIGTYQCK	2383.1326	IPI00270376	IPI00270376	yes	no	2,3	1.9832E-102	232.82
SFWYHDMDDPKGHIIIAQVA(deTNPQGITGTGNTTSEMDPSHR	4524.0863	IPI00321375	IPI00321375	yes	no	4	0.057035	33.165
SFTVN(deWTPPAGDWEHYR	2061.9333	IPI00229935	IPI00229935	yes	no	2,3	5.4401E-07	136.26
SFQQSHVHVHDIQSFGIDNIN(deMTHYIK	3194.5356	IPI00626555	IPI00626555	yes	no	3,4,5	4.1264E-65	192.12
SFPVYIN(deGSTVPSPVK	1690.893	IPI00229935	IPI00229935	yes	no	2,3	7.8259E-12	131.56
SFMVN(deWTQSPGKVEKYR	2056.02	IPI00330632	IPI00330632	yes	no	3	0.0085666	91.207
SFMVN(deWTQSPGKVEK	1736.8556	IPI00330632	IPI00330632	yes	no	2,3	2.9799E-11	162.81
SFMVN(deWTQSPGK	1380.6496	IPI00330632	IPI00330632	yes	no	2	7.9557E-07	155.66
SFIISIAAIHDN(deHTHSDIQVK	2345.2128	IPI00130624	IPI00130624	yes	yes	3,4	1.6185E-11	128.69
SFDCCGIFN(deITTIR	1702.7807	IPI00132189	IPI00132189	yes	yes	2,3	2.9364E-17	179.65
SEYQK(deWECKNDTIFGIK	2145.0201	IPI00126186	IPI00126186	yes	yes	2,3	3.7393E-30	178.51
SEPQEYN(deVSIPHFWVPNAGAISFR	2744.3347	IPI00117841	IPI00117841	yes	no	3	0.0003832	73.675
SEHIVN(deFTVK	1172.619	IPI00330481	IPI00330481	yes	yes	3	0.012372	81.771
SEEIEFIGN(deSSIR	1479.7205	IPI00320618	IPI00320618	yes	yes	2	6.1128E-07	158.52
SDVIN(deITEQVQWVK	1657.8675	IPI00223987	IPI00223987	yes	yes	2	1.9084E-07	159.13
SDN(deGSYFCK	1076.4233	IPI00121254	IPI00121254	yes	yes	2	0.0066525	105.86
SDINPAN(deGSYPFQAIHQR	2013.9657	IPI00165730	IPI00165730	yes	yes	2,3	3.0478E-48	200.77
SDIN(deCSVMEATEEK	1611.6756	IPI00130015	IPI00130015	yes	no	2	9.535E-07	152.94
SDIIYVQTDATNPSVPIDPISVSN(deSSSQIIIK	3400.7613	IPI00128358	IPI00128358	yes	no	4	0.0009652	52.249
SDGIPIAPCGAIANSIFN(deDSFSIWHQR	2972.4239	IPI00221859	IPI00221859	yes	yes	3	2.3536E-06	84.602
SDFSQTMIFQAN(deTTR	1745.8043	IPI00114671	IPI00114671	yes	no	2,3	1.2361E-24	181.51
SCVAITDAFPQN(deMSRR	1851.872	IPI00405742	IPI00405742	yes	no	3	0.039387	61.167
SCVAITDAFPQN(deMSR	1695.7709	IPI00405742	IPI00405742	yes	no	2,3	1.1897E-07	151.44
SCIN(deESAIDSR	1250.5561	IPI00461861	IPI00461861	yes	no	2	0.0003032	122.33
SCHTAVGTSEGWNVPMGIIYN(deQTGSCK	2953.3157	IPI00471080	IPI00471080	yes	no	3,4	2.1805E-31	142.32
SCGECIQAGPNCGWCTN(deTTFIQEGMPTSAR	3389.3992	IPI00132474	IPI00132474	yes	yes	4	1.9896E-16	104.38
SAVSTSWIIPYN(deHTWSHEK	2242.0807	IPI00124428	IPI00124428	yes	yes	2,3	8.6578E-25	172.91
SAVFHRIEGITN(deETYR	1891.9541	IPI00119299	IPI00119299	yes	no	2,3	0.000326	126.48
SAGN(deMSTYNCQIIK	1585.7229	IPI00133520	IPI00133520	yes	yes	2	7.3571E-07	154.69
SAEN(deFTVIIK	1120.6128	IPI00130271	IPI00130271	yes	no	2	0.0057967	95.477
SAEGTFFIN(deKTK	1341.6929	IPI00125522	IPI00125522	yes	no	2,3	1.3984E-06	157.56
SAEGTFFIN(deK	1112.5502	IPI00125522	IPI00125522	yes	no	2	0.0024682	104.82
RYIKNG(deNATIIR	1417.8154	IPI00985828	IPI00985828	yes	no	2	0.01873	91.313
RYIK(deNGNATIIR	1417.8154	IPI00985828	IPI00985828	yes	no	2	0.01873	91.313
RYIEIG(deNETIIR	1475.8096	IPI00850057	IPI00850057	yes	no	2,3	1.4237E-103	244
RVNDN(deKTAAEEAIR	1585.8172	IPI00400016	IPI00400016	yes	no	2,3	4.3807E-10	166.62
RVNASTTDP(deNSTVEQSAITR	2146.0614	IPI00338785	IPI00338785	yes	no	3	0.048901	54.964
RSPGAQD(deNVSVSQGMR	1687.806	IPI00348586	IPI00348586	yes	no	3	0.0035851	89.506
RSN(deFTPATNEAPQATVFPK	2075.0436	IPI00172039	IPI00172039	yes	no	2,3	1.1131E-12	148.01
RQDPVSWN(deKTFEDISR	1976.9704	IPI00848693	IPI00848693	yes	yes	3	0.012082	89.911
RQDPVSWN(deK	1128.5676	IPI00848693	IPI00848693	yes	yes	2	0.032685	89.047
RPYIVPIIWIN(deETGTIGDEKAEMFK	2919.5205	IPI00331214	IPI00331214	yes	yes	4	0.0020976	73.783
RPYIVPIIWIN(deETGTIGDEK	2313.2369	IPI00331214	IPI00331214	yes	yes	3	8.8006E-06	109.54
RPFGVVYEMEVDTIETTCHAIDPTPIAN(deCSVR	3676.7324	IPI00128249	IPI00128249	yes	yes	3,4	1.6309E-05	73.213
RNPSANTFIHI(deNASSFR	1930.9762	IPI00315535	IPI00315535	yes	yes	2,3	3.7885E-10	151.44
RN(deWTETEVR	1189.584	IPI00134390	IPI00134390	yes	yes	2	0.01547	101.97
RN(deFTAADWGHSR	1416.6647	IPI00339885	IPI00339885	yes	no	2,3	0.0038038	106.17
RMHI(deNGSNVQVIHR	1659.874	IPI00119063	IPI00119063	yes	yes	2,3,4	2.6363E-32	192.14
RMEISVGAIQAN(deR	1443.7616	IPI00108811	IPI00108811	yes	yes	2,3	0.0050259	131.17
RIQEGHEYDTFDIN(deDTAQCFTK	2687.1922	IPI00400073	IPI00400073	yes	yes	3	1.0112E-17	141.07
RIPVTN(deISQIHK	1404.8201	IPI00469426	IPI00469426	yes	no	3	0.0091088	80.69
RIGAIN(deNSIIIIEDR	1695.9632	IPI00115516	IPI00115516	yes	no	3	0.0032465	93.478
RIAVDWESIGYN(deITR	1791.9268	IPI00123040	IPI00123040	yes	no	3	2.2703E-07	151.99
RHEEGHMIN(deCTCFGQGR	2087.8836	IPI00113539	IPI00113539	yes	no	2,3	1.114E-05	125.5
RGVFITN(deETGQPIIGK	1728.9523	IPI00111960	IPI00111960	yes	no	2,3	8.4858E-35	198.85
RGTFTDCAIAN(deMTQQIR	1981.9462	IPI00621027	IPI00621027	yes	no	2,3	2.1624E-12	128.74
RGPECSQN(deYTAPTGVIK	1876.9101	IPI00123996	IPI00123996	yes	yes	2,3	2.6825E-14	163.56
RGDDIYTN(deVTVSIVEAIVGFEMDITHIDGHK	3443.7031	IPI00320241	IPI00320241	yes	yes	4	0.057351	38.925
RGCKDN(deATDSVPIR	1587.7787	IPI00119063	IPI00119063	yes	yes	2,3	0.015211	90.793
RFHSDIN(deISESIIPAVIEK	2167.1637	IPI00831484	IPI00831484	yes	yes	3	0.0010418	89.358
RFANEYP(deNITR	1379.6946	IPI00130573	IPI00130573	yes	yes	2	0.062362	63.727
RDQGN(deVTDMASMK	1451.6497	IPI00471081	IPI00471081	yes	yes	2,3	9.6825E-16	172.76
RDDYRPTWTI(deNQTEPVAGNYYPVNTR	3125.4955	IPI00381303	IPI00381303	yes	yes	3	1.2313E-05	88.627
RDDIHPTIPAGQYFIN(deITYNYPVHSFDGR	3405.6531	IPI00387318	IPI00387318	yes	no	3,4	4.9427E-19	118.24
RAN(deASTFAVPSPVSNSADTR	2047.0083	IPI00119065	IPI00119065	yes	yes	2,3	0.0016381	85.203
RADIN(deGSNMETVIGHGIK	1910.9632	IPI00411145	IPI00411145	yes	no	3	0.0001119	111.22
QYDSFTFTASRN(deGTYK	1884.8642	IPI00134378	IPI00134378	yes	no	2,3	9.0794E-08	153.22
QYCTEQN(deATIVK	1453.6871	IPI00123658	IPI00123658	yes	no	2	0.0013169	115.34
QVVEN(deMTR	975.48076	IPI00111960	IPI00111960	yes	no	2	0.014527	97.431
QVTPIFFYFQN(deR	1558.7932	IPI00319509	IPI00319509	yes	no	2,3	0.0010868	127.37
QVSTIIN(deNTDK	1231.6408	IPI00396840	IPI00396840	yes	no	2	0.0003106	123.95
QVN(deGSVSGSQWNK	1389.6637	IPI00408850	IPI00408850	yes	yes	2	0.0036703	97.463
QVEIIEYPYHEQIAVVAPEPIITGHN(deYTIK	3463.8028	IPI00223987	IPI00223987	yes	yes	3,4	3.057E-54	169.28

QVEEIIVN(deHTGIR	1506.8154	IPI00263041	IPI00263041	yes	yes	2,3	0.0032485	103.76
QVAIQTFGN(deQTSIIPAGGAGYK	2220.1539	IPI00228719	IPI00228719	yes	yes	2,3,4	0	356.04
QTTAMDFSYAN(deETVCWVHVGDSAAQTQIK	3257.4758	IPI00119063	IPI00119063	yes	no	3,4	0.0081183	57.432
QTQVGIVQYGAN(deVTHEFNINK	2359.1921	IPI00466371	IPI01026704	no	no	2,3	1.0814E-47	192.09
QTPEYQN(deR	1034.4781	IPI00154056	IPI00154056	yes	no	2	0.014885	97.635
QTIFFN(deGTR	1082.5509	IPI00153840	IPI00153840	yes	yes	2	0.010382	98.04
QTGDVTCN(deCTDGR	1482.5827	IPI00119063	IPI00119063	yes	yes	2	0.0073059	76.262
QSVGQN(deYSNVIAHIR	1684.8645	IPI00112271	IPI00112271	yes	yes	3	1.0184E-07	152.99
QSQPVHIIPMNETDHINMVFSN(deK	2678.2945	IPI00133500	IPI00133500	yes	yes	3,4	9.2848E-16	136.68
QSQPVHIIPMN(deETDHINMVFSNK	2678.2945	IPI00133500	IPI00133500	yes	yes	3,4	9.2848E-16	136.68
QSN(deGSIAVISSIAGK	1430.7729	IPI00115599	IPI00115599	yes	no	2,3	9.0879E-60	176.21
QSINTVN(deDTVWK	1403.7045	IPI00420867	IPI00420867	yes	yes	2	0.0008388	135.02
QSDYCWEIAPHMYFITPGQQPQPPITNIIIVN(deKTGAK	4255.1235	IPI00322575	IPI00322575	yes	no	4	1.3473E-06	63.776
QSDYCWEIAPHMYFITPGQQPQPPITNIIIVN(deK	3897.9222	IPI00322575	IPI00322575	yes	no	4	0.0003507	56.156
QRYN(deITAK	992.54033	IPI00130661	IPI00130661	yes	yes	2	2.654E-05	138.4
QRYEDIVIN(deETINK	1733.8948	IPI00285117	IPI00285117	yes	no	2,3	0.0038168	120.87
QQTECMVQQYSN(deYSVNGEPVNGR	2687.1704	IPI00396840	IPI00396840	yes	no	3	7.8348E-05	86.625
QQCIEEAQIEN(deETTGCSK	2123.9099	IPI00128984	IPI00128984	yes	yes	2,3	1.5521E-11	158.58
QPIYIN(deCSCVTGGSASAK	1911.8819	IPI00309249	IPI00309249	yes	yes	2	3.1003E-28	183.6
QNPMTIGNVVI(deFDKVITNQESPYQNHTGR	3299.6357	IPI00125310	IPI00125310	yes	no	3,4	1.2336E-13	103.03
QNFSN(deITVSTEDQVK	1708.8268	IPI00129677	IPI00129677	yes	no	2,3	1.2525E-57	216.71
QNAQDVIIKTN(deATK	1542.8366	IPI00338785	IPI00338785	yes	no	2,3	0.0037636	112.17
QN(deFSNITVSTEDQVK	1708.8268	IPI00129677	IPI00129677	yes	no	2,3	1.2525E-57	216.71
QKNVNISYIVN(deDSFFPQRPEK	2522.2918	IPI00122557	IPI00122557	yes	no	3,4	1.102E-37	183.74
QKNVN(deISYIVNDSFFPQRPEK	2522.2918	IPI00122557	IPI00122557	yes	no	3,4	1.102E-37	183.74
QKNITAFN(deETIFR	1580.8311	IPI00124700	IPI00124700	yes	yes	3	0.022834	75.479
QIYPYN(deSSNR	1240.5836	IPI00229820	IPI00229820	yes	yes	2	0.015124	79.148
QIYN(deITVR	1005.5607	IPI00623114	IPI00623114	yes	no	1,2	9.6091E-06	141.78
QIWDWSKHN(deITITQGK	1954.0061	IPI00128358	IPI00128358	yes	no	2	0.0002196	130.29
QINPPHTAN(deSSITSK	1593.8111	IPI00988390	IPI00988390	yes	yes	2,3	0.0006684	106.32
QINNGEIQPISEN(deTTITFMSTK	2465.2108	IPI00126834	IPI00126834	yes	no	3	2.4761E-48	150.69
QINAYN(deR	877.44061	IPI00473190	IPI00473190	yes	no	2	0.0060872	114.02
QIN(deITTEDDDIYHMTVPYGRPR	2633.2544	IPI00265291	IPI00265291	yes	yes	3	2.1149E-11	129.12
QIN(deISIIVK	1026.6437	IPI00115116	IPI00115116	yes	yes	2	0.002456	119.53
QIN(deISDSIAVIGIAKPAHIYSK	2337.3056	IPI00463492	IPI00463492	yes	yes	3,4	2.3333E-06	100.92
QIN(deISDSIAVIGIAK	1540.8825	IPI00463492	IPI00463492	yes	yes	2	6.8199E-18	178.23
QIITEEKIPN(deNTQWITWSPEGHKIAYVWK	3508.8143	IPI00125813	IPI00125813	yes	no	4	3.1974E-18	115.5
QIITEEKIPN(deNTQWITWSPEGHK	2748.3871	IPI00125813	IPI00125813	yes	no	2,3,4	3.3148E-172	257.73
QIIQTQVASPAIHPPVSYN(deDTAPR	2602.3503	IPI00128989	IPI00128989	yes	no	3	1.3696E-55	190.67
QIINAIQIN(deNTAVGHAIVIPARR	2481.4292	IPI00830749	IPI00830749	yes	yes	3,4	0.0079252	73.928
QIINAIQIN(deNTAVGHAIVIPAR	2325.3281	IPI00830749	IPI00830749	yes	yes	3	8.2155E-112	161.99
QIIN(deISQIVYGPEGSDIVIIK	2298.2835	IPI00116509	IPI00116509	yes	no	2,3	5.441E-09	113.63
QIIIAN(deNSIR	1140.6615	IPI00128024	IPI00128024	yes	yes	2	0.0005311	121.36
QIIAN(deSSAIEETIIGHQGR	2036.0651	IPI00116913	IPI00116913	yes	no	2,3	9.244E-53	205.98
QIGCADN(deGTVKPIPSDK	1798.8883	IPI00760106	IPI00760106	yes	no	2,3	4.9872E-06	130.21
QIGASPSDDIIFGVFAQSKPDSAEPVN(deR	2944.4567	IPI00130420	IPI00130420	yes	no	3,4	1.8754E-31	146.76
QIFFN(deGTETIR	1324.6776	IPI00338209	IPI00338209	yes	no	2	0.0021719	98.898
QHTVTTTTKGEN(deFTETDVK	2136.0335	IPI00120793	IPI00120793	yes	yes	2	0.008845	73.448
QHGQFSIAVVGIN(deITSIGIR	2109.1695	IPI00121190	IPI00121190	yes	no	3	6.7358E-06	107.03
QGPQAGGTTITIN(deGTHIDTGSKEDVR	2652.3103	IPI00405742	IPI00405742	yes	no	3,4	3.8207E-33	161.02
QGPQAGGTTITIN(deGTHIDTGSK	2153.0713	IPI00405742	IPI00405742	yes	no	2,3	2.3854E-161	260.06
QGEAIIQKFQQIIIRN(deISVVVATHSPTIAK	3301.8874	IPI00221418	IPI00221418	yes	no	4	0.0005651	64.779
QGDQYSCMVGHEAIPMN(deFTQK	2440.061	IPI00968978	IPI00968978	yes	no	2,3,4	7.2493E-19	149.52
QFWIFDVQNPDDVAKN(deSSK	2237.0753	IPI00331214	IPI00331214	yes	yes	2,3	1.4267E-14	152.94
QFFN(deASVQFDNMDPIIDYINQR	2674.2486	IPI00117842	IPI00117842	yes	yes	3	8.5003E-32	178.72
QEVMFITNVN(deSSSSSTQIYQAVSR	2675.2861	IPI00112614	IPI00112614	yes	yes	3	1.4201E-46	185.71
QEMNGTYVCHAFSSHGN(de)VTR	2293.9957	IPI00122973	IPI00122973	yes	no	3	0.0074829	73.138
QEMNGTY(deVCHAFSSHGNVTR	2293.9957	IPI00122973	IPI00122973	yes	no	3	0.0074829	73.138
QEIN(deDSIQVAER	1400.6896	IPI00320420	IPI00320420	yes	yes	2	7.4999E-07	156.08
QDPVSWNK(deTFEDISR	1820.8693	IPI00848693	IPI00848693	yes	yes	3	0.033913	68.034
QDIAISGN(deISSIYAMTQDK	2053.999	IPI00405742	IPI00405742	yes	no	2,3	9.4218E-203	282.72
QAWAIVGVIDGGSSSCN(deESVR	2191.0328	IPI00463492	IPI00463492	yes	yes	3	1.3848E-05	96.143
QASSPN(deSSTAQQQTATTQASMNIATTSAAQIISR	3450.6645	IPI00380778	IPI00380778	yes	no	3	0.011426	51.321
QAIQTMQSEFFYITTNIIN(deDTIEIR	2988.4903	IPI00118130	IPI00118130	yes	yes	2,3	4.7812E-67	204.31
PNIIIVITDDQDVEIGSMQVMN(deK	2571.2924	IPI00268030	IPI00268030	yes	yes	3	0.016014	54.225
NYTANATSSREEAWDY(deVQAQVK	2530.1724	IPI00108098	IPI00108098	yes	no	2,3	7.2953E-08	103.66
NYTANATSSREE(deAWDYVQAQVK	2530.1724	IPI00108098	IPI00108098	yes	no	2,3	7.2953E-08	103.66
NYN(deFTIACNTKR	1500.7143	IPI00322575	IPI00322575	yes	no	2	0.0049593	100.19
NYN(deFTIACNTK	1344.6132	IPI00322575	IPI00322575	yes	no	2,3	1.402E-23	182.1
NYKNPN(deITISFTAER	1766.8951	IPI00132600	IPI00132600	yes	no	2,3	1.2767E-06	113.53
NYFHYN(deQSFPPSYNIK	2017.9323	IPI00129265	IPI00129265	yes	yes	2,3	1.027E-17	170.78
NYEVQIFHVN(deATVTEEGTGIEFSR	2739.314	IPI00123223	IPI00123223	yes	yes	2,3,4	0	318.66
NVTVV(deIR	799.49159	IPI00469218	IPI00469218	yes	yes	2	0.007709	106.36
NVPGEIGINYN(deITADVAQK	2015.0324	IPI00130117	IPI00130117	yes	yes	3	1.5974E-12	142.94
NVNISYTVN(deDSFFPQRPQK	2253.1178	IPI00381178	IPI00381178	yes	yes	2,3,4	2.5524E-202	283.31
NVNISYIVNDS(deFFPQRPEK	2266.1382	IPI00122557	IPI00122557	yes	no	2,3,4	8.7255E-60	192.6
NVNIS(deYIVNDSFFPQRPEK	2266.1382	IPI00122557	IPI00122557	yes	no	2,3,4	8.7255E-60	192.6
NVN(deMSVICPTTIR	1503.7538	IPI00129867	IPI00129867	yes	no	2	0.0025999	127.71
NVN(deISYTVNDSFFPQRPQK	2253.1178	IPI00381178	IPI00381178	yes	yes	2,3,4	2.5524E-202	283.31
NVITNQTINN(deDTVASSFIK	2078.0644	IPI00471264	IPI00471264	yes	no	3	1.4219E-06	119.08
NVITN(deQTINNDTVASSFIK	2078.0644	IPI00471264	IPI00471264	yes	no	3	1.4219E-06	119.08
NVIIWGN(deHSSTQYPDVNHAK	2279.1083	IPI00336324	IPI00336324	yes	yes	3	3.7215E-15	157.11
NVAIVAGDTGN(deATGIGEQGPTR	2097.0451	IPI00122272	IPI00122272	yes	no	2,3	2.1224E-161	261.06
NTQADVINASWSVISN(deSTRHEIER	2726.3372	IPI00132189	IPI00132189	yes	yes	3,4	5.3865E-24	141.6
NTQADVINASWSVISN(deSTR	2062.0079	IPI00132189	IPI00132189	yes	yes	2,3	7.4791E-155	263.55
NTQADVIN(deASWSVISNSTRHEIER	2726.3372	IPI00132189	IPI00132189	yes	yes	3,4	5.3865E-24	141.6

NTQADVIN(deASWSVISNSTR	2062.0079	IPI00132189	IPI00132189	yes	yes	2,3		7.4791E-155	263.55
NTGEIN(deITSIIDREETPYFIITGYAIDSR	3300.6514	IPI00115793	IPI00115793	yes	no		3	8.73E-10	92.275
NTDKENPYIGIGN(deYTICR	2127.0055	IPI00468236	IPI00468236	yes	no	2,3		0.0001324	113.01
NTAFNITCQAVGPPEPVNIFWVQN(deSSR	3045.4767	IPI00121254	IPI00121254	yes	yes		3	2.6795E-26	137.52
NTAFN(deITCQAVGPPEPVNIFWVQNSSR	3045.4767	IPI00121254	IPI00121254	yes	yes		3	2.6795E-26	137.52
NSQFDMN(deSTDIAIK	1582.7297	IPI00346062	IPI00346062	yes	yes		2	0.0013415	119.54
NSNSN(deVIQVDQSGIGIPSRDYYINKTENEK	3381.6437	IPI00396840	IPI00396840	yes	no	3,4		9.736E-05	75.71
NSKYYNYTI(deSINGK	1663.8206	IPI00221426	IPI00221426	yes	yes		3	0.034934	89.43
NSK(deYYNYTISINGK	1663.8206	IPI00221426	IPI00221426	yes	yes		3	0.034934	89.43
NQSVGDP(deNVDIIR	1425.7212	IPI00127447	IPI00127447	yes	yes		2	0.0035693	96.591
NQN(deSTCIGTDINR	1491.6736	IPI00126316	IPI00126316	yes	yes		2	0.014091	69.716
NQKDEIN(deETDIK	1445.6998	IPI00930882	IPI00930882	yes	no		2	0.014423	79.875
NQIVEI(deEKVVIHPNHSVVDIGIIK	2692.5276	IPI00409148	IPI00409148	yes	yes		3	2.7178E-15	122.19
NPSANTFIHIN(deASSFR	1774.8751	IPI00315535	IPI00315535	yes	yes	2,3		0.0001994	117.67
NPNN(deNTIHPNIR	1402.7066	IPI00319509	IPI00319509	yes	no	2,3		7.9076E-09	136.38
NPIGQAQSAVVIN(deVTER	1794.9588	IPI00119299	IPI00119299	yes	no	2,3		1.7933E-59	166.76
NPCTSEQN(deCTSPFSYK	1918.7826	IPI00132474	IPI00132474	yes	yes	2,3		3.6897E-08	152.32
NNQTECFNHV(deR	1417.6157	IPI00124666	IPI00124666	yes	no		2	0.0061722	83.182
NNQT(deECFNHVR	1417.6157	IPI00124666	IPI00124666	yes	no		2	0.0061722	83.182
NNGAGYF(deIEHIAFK	1579.7783	IPI00111885	IPI00111885	yes	no		2	0.055287	52.527
NMICQWDPGRETYIETN(deYTIK	2631.2098	IPI00120155	IPI00120155	yes	yes		3	9.8061E-09	116.13
NKNPQMN(deFTEANEACK	1894.8302	IPI00308245	IPI00308245	yes	yes		3	0.0007427	110.77
NKANIQFGEN(deGTTISAVTNK	2106.0705	IPI00127447	IPI00127447	yes	yes	2,3		9.709E-155	263.55
NITVIEPVTQPFIQVTN(de)TTVK	2341.2893	IPI00108535	IPI00108535	yes	no	2,3		1.3826E-14	132.79
NITVI(deEPVTQPFIQVTNTTVK	2341.2893	IPI00108535	IPI00108535	yes	no	2,3		1.3826E-14	132.79
NITAFNET(deIFR	1324.6776	IPI00124700	IPI00124700	yes	yes		2	0.0001353	138.45
NIQVYN(deATSNSITVK	1650.8577	IPI00121430	IPI00121430	yes	no		2	2.3853E-05	140.96
NIQAVNEIIATISQCN(deDTSSAAMVQCIR	3106.4846	IPI00138342	IPI00138342	yes	no	3,4		2.3937E-26	135.76
NIPWYVIAGNHDHIGN(deVSAQIAYSK	2766.3878	IPI00137491	IPI00137491	yes	yes	3,4		0.003749	60.991
NIN(deVTSIGFR	1119.6037	IPI00468814	IPI00468814	yes	yes		2	0.009691	87.298
NIMTIVHFYN(deK	1378.7067	IPI00119822	IPI00119822	yes	no		2	0.0029716	94.767
NIMIDIQKDTAV(deEGEEIEVNCTAMASKPATTIR	3647.7845	IPI00856723	IPI00856723	yes	no		4	0.0013731	56.903
NIKEE(deKANVVASAIAQIPQK	2150.2059	IPI00153143	IPI00153143	yes	yes		3	2.3302E-12	147.09
NIIPTMN(deGSCTFHK	1618.7596	IPI00130271	IPI00130271	yes	no		3	0.0082609	72.089
NIIPN(deITTSYIK	1375.7711	IPI00471089	IPI00471089	yes	no		2	0.0026085	93.345
NIINDYVSN(deQTQGMIKEIISEIDER	2921.4441	IPI00131830	IPI00131830	yes	no	3,4		2.1287E-19	134.32
NIINDYVSN(deQTQGMIK	1836.904	IPI00131830	IPI00131830	yes	no	2,3		6.5092E-125	250.52
NIIGIDENFVIN(deSSTEHR	2057.0178	IPI00406603	IPI00406603	yes	yes	2,3		2.0166E-75	223.11
NIGPN(deTTSTTITSDDFKPGVR	2220.1022	IPI00118291	IPI00118291	yes	no		3	5.9244E-05	93.226
NIGN(deTSEGPR	1043.4996	IPI00221706	IPI00221706	yes	yes		2	0.0028397	102.53
NIGGIETEDDYGYQGHVQTCN(deFSAQMAK	3132.3553	IPI00126769	IPI00126769	yes	yes	3,4		8.6247E-27	139.04
NIGFN(deCTTSQGK	1325.6034	IPI00224294	IPI00224294	yes	yes		2	0.0034809	84.892
NIFIN(deHSETASAK	1430.7154	IPI00409148	IPI00409148	yes	yes	2,3		4.2504E-32	190.27
NIADQGN(deQTAAIK	1342.6841	IPI00458708	IPI00458708	yes	no		2	0.0027411	130.2
NI(deTSPVGVQPIINEHTFCAGITK	2495.2842	IPI00409148	IPI00409148	yes	yes		3	5.1478E-06	92.563
NHFTVAQAEAFN(deK	1475.7157	IPI00626112	IPI00626112	yes	yes		2	0.0051102	79.466
NGNATI(deIR	857.47191	IPI00985828	IPI00109996	no	no		2	1.6082E-17	131.5
NGNATI(deIR	857.47191	IPI00985828	IPI00109996	no	no		2	1.6082E-17	131.5
NGNA(deTIIR	857.47191	IPI00985828	IPI00109996	no	no		2	1.6082E-17	131.5
NGNA(deTIIR	857.47191	IPI00985828	IPI00109996	no	no		2	1.6082E-17	131.5
NGIYN(deITVIAIDADGR	1703.8842	IPI00138209	IPI00138209	yes	no	2,3		1.7403E-09	160.25
NGIPDEQIIVMMYDDIANSEENPTPGVVINRPN(deGTDVYK	4317.057	IPI00130627	IPI00130627	yes	yes		4	1.358E-07	65.883
NGIASGSGN(deCSTGPNGDGSFHAWSIIEVK	2918.3253	IPI00322497	IPI00322497	yes	yes		3	1.1877E-42	166.65
NGHITN(deYTVVYR	1435.7208	IPI00110264	IPI00110264	yes	yes		2	0.010489	73.466
NGDAYGYYN(de)DSIK	1478.6314	IPI00471081	IPI00471081	yes	yes		2	0.0027768	115.91
NGDAYG(deYYNDSIK	1478.6314	IPI00471081	IPI00471081	yes	yes		2	0.0027768	115.91
NFVIN(deMTCR	1153.5372	IPI00222321	IPI00222321	yes	no		2	0.013802	92.538
NFN(deDSIHSISQAIR	1600.7958	IPI00263041	IPI00263041	yes	yes		2	0.0022437	93.823
NF(deTAADWGHSR	1260.5636	IPI00339885	IPI00339885	yes	no		2	0.042949	59.57
NF(deNVEKINGEWHTIIIASDKR	2483.2921	IPI00968968	IPI00944716	no	no		3	0.0090137	77.969
NEIMIN(deSSIMR	1306.6373	IPI00378698	IPI00378698	yes	no		2	0.02908	59.155
NEEVKN(deMSVEINSK	1619.7825	IPI00128206	IPI00128206	yes	yes		2	0.0155	77.068
NDTGPYVCET(deR	1310.5561	IPI00108535	IPI00108535	yes	no		2	0.0003038	122.46
NDRN(deISDEIFIR	1490.7478	IPI00311808	IPI00311808	yes	no		2	0.015203	79.492
NCKPIIIDN(deVSFFGVK	1849.976	IPI00122581	IPI00122581	yes	no		3	0.000326	126.48
NAVN(deCTYKNEDDCVVR	1955.8466	IPI00266264	IPI00266264	yes	yes		2	0.0030276	97.836
NATIAEQAKI(dePATEKPVIISK	2221.2682	IPI00123342	IPI00123342	yes	yes		3	3.6458E-21	159.81
NAN(deCSTVSPGQTQCTCQK	2039.8459	IPI00467600	IPI00467600	yes	yes	2,3		0.0090432	66.436
NAIQAFGN(deGTDVNMSPK	1762.8308	IPI00116355	IPI00116355	yes	no	2,3		8.1226E-28	183.74
NAIQAFGN(deGSDVTMWQPAPPVQTTTATTTTAFR	3479.678	IPI00128641	IPI00128641	yes	no		4	0.0001565	59.567
NAIN(deITFHAQNIGEGGAYEAEIR	2487.2142	IPI00115976	IPI00115976	yes	yes		3	0.0004892	90.22
NAIKEGDNITIQ(deCIGNGNPPPEEFMFYIPGQPEGIR	4014.9244	IPI00121378	IPI00121378	yes	no		4	0.025875	47.66
NAIK(deEGDNITIQCIGNGNPPPEEFMFYIPGQPEGIR	4014.9244	IPI00121378	IPI00121378	yes	no		4	0.025875	47.66
NACCSVN(deTSQEIHK	1646.7141	IPI00113158	IPI00113158	yes	yes		2	0.0021232	103.21
N(deTTSYPPMCSQDAVGGQVISEIFTNRK	2999.4117	IPI00387289	IPI00387289	yes	yes		3	1.3267E-10	100.76
N(deTTSYPPMCSQDAVGGQVISEIFTNR	2871.3167	IPI00387289	IPI00387289	yes	yes		3	1.074E-39	175.28
N(deTTSAAMVHCIR	1359.6387	IPI00131216	IPI00131216	yes	yes		2	0.0011773	117.93
N(deSTIQAANIAGIK	1299.7147	IPI00222937	IPI00222937	yes	no		2	2.0439E-32	195.1
N(deSTICDICIGPIK	1489.7269	IPI00987951	IPI00987951	yes	no		2	8.9575E-16	171.26
N(deMTIFSDIVAEK	1366.6803	IPI00621027	IPI00621027	yes	no		2	0.0021857	102.5
N(deFTDVHPDYGAR	1390.6266	IPI00312058	IPI00312058	yes	no		2	0.0055044	79.492
N(deATVVWMK	947.48987	IPI00121378	IPI00121378	yes	no		2	0.012477	103.56
N(deATTYPPMCSQDAAR	1681.7188	IPI00128399	IPI00128399	yes	yes		2	0.0003761	124.16
N(deATSYPPMCSQDAGWAK	1882.7978	IPI00138342	IPI00138342	yes	yes		2	0.044642	52.372
N(deATIVNEADKIR	1342.7205	IPI00319509	IPI00319509	yes	no		2	0.0036373	115.34
N(de)ATIAEQAK	944.49271	IPI00123342	IPI00123342	yes	yes		2	0.033121	72.643

MYVTN(deDTEVAENNYEAIKDFFR	2668.2115	IPI00658539	IPI00658539	yes	no	2,3	1.1535E-08	117.67
MYSEGSDIVPQSN(deETAIHYFKK	2543.2002	IPI00131143	IPI00131143	yes	no	2,3	3.5245E-15	139.08
MYSEGSDIVPQSN(deETAIHYFK	2415.1053	IPI00131143	IPI00131143	yes	no	2,3	1.3892E-14	132.51
MYN(deNCEVVIGNIEITYVQR	2314.1086	IPI00121190	IPI00121190	yes	no	3	0.014509	60.489
MWIENITNGN(deMTS	1509.6592	IPI00116945	IPI00116945	yes	no	2	5.8975E-16	170.66
MWIEN(deITNGNMTS	1509.6592	IPI00116945	IPI00116945	yes	no	2	5.8975E-16	170.66
MVVCDIGNPMVTGTN(deFSIGIR	2280.1065	IPI00345112	IPI00345112	yes	yes	3	9.4509E-05	89.859
MVSIDIPYFADVVVPIN(deMTMK	2382.2037	IPI00411145	IPI00411145	yes	no	2,3	1.2347E-05	91.725
MVN(deTTFICTATNAVGTGR	1912.9135	IPI00109375	IPI00109375	yes	no	2,3	1.7831E-130	259.18
MVIWN(deDSTIR	1233.6176	IPI00135975	IPI00135975	yes	yes	2	0.0059477	94.767
MTQIIGITPN(deATHIHRPPR	2152.1688	IPI00130325	IPI00130325	yes	yes	3,4	0.011225	70.96
MTQESAIIFPFIN(deK	1637.8487	IPI00857780	IPI00857780	yes	no	2,3	0.0013035	125.28
MTISSIKDDIGACRN(deCCSVTK	2415.1015	IPI00223352	IPI00223352	yes	yes	3	0.0072295	79.311
MTISQN(deNSIIR	1275.6605	IPI00108535	IPI00108535	no	no	2	5.8239E-09	160.52
MSVINFECN(deKTAGK	1597.7592	IPI00308971	IPI00308971	yes	yes	2	0.0060192	94.09
MSVINFECN(deK	1240.558	IPI00308971	IPI00308971	yes	yes	2	0.015191	78.653
MSQHPIDIQEDFNNATIVN(deASSIIPK	2881.428	IPI00762812	IPI00762812	yes	yes	3	5.1972E-06	82.593
MSQHPIDIQEDFNN(deATIVNASSIIPK	2881.428	IPI00762812	IPI00762812	yes	yes	3	5.1972E-06	82.593
MSPWSN(deWSECDPCIK	1895.7641	IPI00230718	IPI00230718	yes	no	3	0.0004064	115.06
MSIVMPAMAPN(deETVSGR	1789.8525	IPI01027336	IPI01027336	yes	no	2,3	0.0004062	97.417
MRSPGAQ(deDNVSVSQGMR	1818.8465	IPI00348586	IPI00348586	yes	no	3	1.3048E-08	149
MQVVSN(deGTVTTAIWR	1661.8559	IPI00331175	IPI00331175	yes	no	2,3	1.2155E-11	161.84
MPSQASAGNVYPQPIIN(deSSMCIEDSR	2851.2939	IPI00224752	IPI00224752	yes	yes	3	2.1701E-20	132.72
MPFPIDQDFYVSPTFQDIIN(deR	2542.2202	IPI00318175	IPI00318175	yes	yes	3	1.0464E-05	97.32
MNCFQGTN(deASAIEK	1569.6916	IPI00882216	IPI00882216	yes	yes	3	0.0016048	108.72
MN(deYTGGDTCHK	1282.5071	IPI00308971	IPI00308971	yes	yes	2	0.0074039	80.522
MN(deITSIAPIIEK	1328.7374	IPI00380296	IPI00380296	yes	no	2	0.0037129	82.069
MISAFN(deATSGK	1125.5488	IPI00466999	IPI00466999	yes	no	2	0.00258	96.64
MIPFIIATIGTAAIN(deSSNPK	2058.1183	IPI00381160	IPI00381160	yes	no	3	0.0094418	62.203
MIEN(deGSISFIPTIR	1576.8283	IPI00123194	IPI00123194	yes	yes	2,3	1.1657E-10	163.51
MHIN(deGSNVQVIHR	1503.7729	IPI00119063	IPI00119063	yes	yes	2,3,4	0.0005939	140.08
MGDREAIGN(deASQIFDSWIK	2137.0262	IPI00134585	IPI00134585	yes	yes	2,3	3.0604E-15	158.92
MGCQHHCVPTPSGPTCYCN(deSSFQIQADGK	3323.3675	IPI00119063	IPI00119063	yes	no	3,4	4.7971E-06	81.315
MFSQN(deDTR	997.42873	IPI00129250	IPI00129250	yes	yes	2	9.3267E-06	138.4
MEISVGAIQAN(de)R	1287.6605	IPI00108811	IPI00108811	yes	yes	2	0.000912	134.08
MEAHNVSAPFN(deFSIPPGFGHR	2311.0957	IPI00115482	IPI00115482	yes	no	2,3,4	2.9312E-47	192.16
MEAHN(deVSAPFNFSIPPGFGHR	2311.0957	IPI00115482	IPI00115482	yes	no	2,3,4	2.9312E-47	192.16
MDYNSFQGTPSN(deETK	1717.7254	IPI00876541	IPI00876541	yes	no	2	2.3296E-17	172.91
MDFIIFN(deYSAPSYIR	1835.8916	IPI00553503	IPI00553503	yes	yes	2,3	1.152E-11	162.29
MAIIQYGSQNQQQVAFPITYN(deVTTIHEAIER	3562.7879	IPI00621027	IPI00621027	yes	yes	3,4	3.7698E-67	180.7
MAAANVSITQPESTGEPNN(deMTIIAEEAR	2944.3906	IPI00400016	IPI00400016	yes	no	3	0.0061268	49.793
MAAAN(deVSITQPESTGEPNNMTIIAEEAR	2944.3906	IPI00400016	IPI00400016	yes	no	3	0.0061268	49.793
MAAAINATGRPIAFSCSWPAYEGGIPPKVN(deYTEVSR	3880.9029	IPI00315593	IPI00315593	yes	no	4	2.0001E-05	62.393
MAAAIN(deATGRPIAFSCSWPAYEGGIPPKVNYTEVSR	3880.9029	IPI00315593	IPI00315593	yes	no	4	2.0001E-05	62.393
MAAAIN(deATGR	974.49675	IPI00315593	IPI00315593	yes	no	2	1.7586E-07	125.74
KYQTIN(deCSVNVR	1480.7456	IPI00420835	IPI00420835	yes	no	2,3	0.0022435	124.12
KYHDYYITSTS(deNGSIEGIENR	2446.1401	IPI00130754	IPI00130754	yes	yes	3	0.0004596	88.565
KYFDQVDIS(deNGIDWSIDHK	2279.0859	IPI00133456	IPI00133456	yes	yes	3	1.9155E-05	109.42
KYEQAK(deNISQDIEK	1692.8683	IPI00400016	IPI00400016	yes	no	2,3	6.999E-32	193.96
KWPERISAI(deDNIINHSSIFIK	2480.354	IPI00123342	IPI00123342	yes	yes	3,4	6.789E-05	97.502
KWGH(deNVTEFQQR	1528.7535	IPI00754386	IPI00754386	yes	yes	2,3	0.0086339	81.017
KVYEEVIN(deVTPNDGFAK	1921.9785	IPI00624896	IPI00624896	yes	no	3	0.0061653	77.746
KVPSN(deSTETVIESDQFQPGVR	2317.155	IPI00119299	IPI00119299	yes	no	2,3	6.0624E-31	176.94
KVIIN(deNSIDEPR	1396.7674	IPI00411145	IPI00411145	yes	no	2,3	0.0051425	93.345
KVEVEPIN(deSTAVHVSWK	1922.0262	IPI00110264	IPI00110264	yes	yes	2,3	1.5529E-14	166.48
KVEVEAVN(deATAVK	1356.7613	IPI00608063	IPI00608063	yes	no	2,3	0.005095	118.08
KVEAEAIN(deATAIR	1384.7674	IPI00754853	IPI00754853	yes	no	2	0.010249	79.337
KVDN(deASIVADDMR	1432.698	IPI00356462	IPI00356462	yes	no	2	0.0059832	92.773
KTYAVYDIFDTAMIN(deNSR	2121.0201	IPI00226790	IPI00226790	yes	no	2,3	1.4231E-15	170.11
KTTIEN(deFTCPEYK	1629.7709	IPI00420810	IPI00420810	yes	yes	3	0.0059887	90.731
KTGVHDGDFEY(deNITTTIAAINK	2407.202	IPI00108849	IPI00108849	yes	no	3	4.7629E-11	125.18
KTGEA(deNITQIYTQEAIDFIQTQHAR	2875.4464	IPI00310090	IPI00310090	yes	no	3,4	1.2541E-38	170.59
KTCNPETFPIRN(deESIQCPTAR	2518.2057	IPI00649186	IPI00649186	yes	no	3	0.0001386	93.381
KT(deNQSCEIVIDSTEKVNPSYIGR	2637.3068	IPI00310059	IPI00310059	yes	no	3	6.213E-25	162.43
KT(deNQSCEIVIDSTEK	1750.8407	IPI00310059	IPI00310059	yes	no	2,3	4.057E-05	141.85
KSPGYVIDIIVTPQN(deK	1770.988	IPI00109946	IPI00109946	yes	yes	3	0.0003798	118.52
KSDTQNIIYN(deVSTGR	1694.8588	IPI01023214	IPI01023214	yes	no	2,3	1.9023E-07	153.39
KSCHTAVGTSEGWNVPMGIIY(deNQTGSCK	3081.4107	IPI00471080	IPI00471080	yes	no	4	0.023696	48.489
KQYDSFTFTASR(deNGTYK	2012.9592	IPI00134378	IPI00134378	yes	no	2	0.010868	88.385
KQVTPIFFYFQ(deNR	1686.8882	IPI00319509	IPI00319509	yes	no	2,3	4.4541E-06	152.85
KQEIPSN(deCSIPYHVYNIK	2189.0939	IPI00311305	IPI00311305	yes	yes	3	0.015554	67.646
KPISQEFEAYVN(deASGEHGIVVFSIGSMVSEIPEKK	3805.9237	IPI00134691	IPI00134691	yes	no	4,5,6	5.399E-17	94.15
KPIIGHYKPDTIAVVIEN(deGTSIDR	2635.4334	IPI00410796	IPI00410796	yes	no	3,4	3.3613E-05	88.396
KNVNISYTV(deNDSFFPQRPQK	2381.2128	IPI00381178	IPI00381178	yes	yes	2,3,4,5	3.568E-154	260.97
KNV(deNISYTVNDSFFPQRPQK	2381.2128	IPI00381178	IPI00381178	yes	yes	2,3,4,5	3.568E-154	260.97
KNIQAVNEIIATISQC(deNDTSSAAMVQCIR	3234.5795	IPI00138342	IPI00138342	yes	no	3,4	0.0004322	68.309
KNACCSV(deNTSQEIHK	1774.8091	IPI00113158	IPI00113158	yes	yes	3	0.0041651	88.282
KN(deITSPVGVQPIINEHTFCAGITK	2623.3792	IPI00409148	IPI00409148	yes	yes	3	1.6827E-15	123.36
KN(deITDIVEGAKK	1314.7507	IPI00649186	IPI00649186	yes	no	3	0.01575	100.04
KN(deITDIVEGAK	1186.6558	IPI00649186	IPI00649186	yes	no	2	0.0007586	119.39
KN(deISEIWDAYCYR	1716.793	IPI00987265	IPI00987265	yes	no	2,3	0.012174	77.912
KMVIW(deNDSTIR	1361.7126	IPI00135975	IPI00135975	yes	yes	3	0.026268	73.499
KKENGVFEEISN(deSSGR	1779.8751	IPI00757771	IPI00757771	yes	no	2,3	2.8525E-05	121.31
KIVIYIEHNIEK(deNSTK	1928.0731	IPI00321190	IPI00321190	yes	no	2	0.048139	70.235
KITDVETQVIN(deQTSR	1730.9163	IPI00114350	IPI00114350	yes	yes	2	0.0033521	91.657
KITDVEAQVIN(deQTTR	1714.9214	IPI00132602	IPI00132602	yes	yes	3	0.0007909	124.42

KISTSPFAIN(deITMIPK	1759.9906	IPI00666034	IPI00666034	yes	no		3	1.386E-05	141.34
KISTN(deITIVCKPGDIESAPVIR	2410.3254	IPI00308971	IPI00308971	yes	yes		3	9.1276E-21	157.22
KINIDGS(deNYTIIK	1477.814	IPI00119063	IPI00119063	yes	yes	2,3		4.6494E-05	150.26
KINASIVTSFVEIPIVSN(deVSIR	2385.3631	IPI00323857	IPI00323857	yes	no		3	3.3065E-11	127.17
KIN(deASIVTSFVEIPIVSNVSIR	2385.3631	IPI00323857	IPI00323857	yes	no		3	3.3065E-11	127.17
KIIASPNEEN(deMTEIISMR	2075.0391	IPI00322575	IPI00322575	yes	no	2,3,4		2.249E-15	168.05
KIDFIVIN(deETR	1346.7558	IPI00125813	IPI00125813	yes	no	2,3		0.0006611	129.37
KHYQVTGYGIN(deGTGDSNDFWR	2414.104	IPI00221669	IPI00221669	yes	no		3	0.0034022	92.307
KGQNMVCGGMGCDN(deDSIVQQIFNAAEIDTYTR	3591.5851	IPI00132600	IPI00132600	yes	no	3,4,5		1.6478E-67	185.06
KGN(deNTINPFIIPSASDVPTGCPNQWWPYAGHCYR	3916.8202	IPI00126186	IPI00126186	yes	yes		4	9.3157E-08	74.13
KGMWSEGN(deGSHTIR	1558.7311	IPI00123129	IPI00123129	yes	no		3	0.0037489	104.95
KGFCEADN(deSTVSENNPEDWPVNTEGCMEK	3343.3704	IPI00108098	IPI00108098	yes	yes	3,4		2.847E-10	97.124
KGCADYCN(deQTITK	1557.6916	IPI00987265	IPI00987265	yes	no	2,3		0.0069709	105.65
KFPVPFQKEN(deVTATIVEIGR	2272.258	IPI00134691	IPI00134691	yes	yes	2,3,4		1.5844E-89	171.29
KFN(deSTQIAAMAPEHEEPR	2054.9844	IPI00856861	IPI00856861	yes	no		3	1.0772E-14	162.59
KEQETCIAPEIEHGN(deYSTTQR	2490.1445	IPI00122117	IPI00122117	yes	yes		3	1.0059E-17	145.02
KENGVFEEISNS(deSGR	1651.7802	IPI00757771	IPI00757771	yes	no	2,3		5.7041E-13	123.76
KENG(deVFEEISNSSGR	1651.7802	IPI00757771	IPI00757771	yes	no	2,3		5.7041E-13	123.76
KEN(deSSYQVINWR	1522.7528	IPI00153316	IPI00153316	yes	yes	2,3		0.0023017	125.82
KEN(deSSEICSNNGECVCGQCVCR	2646.0349	IPI00132474	IPI00132474	yes	no		3	5.1846E-21	154.49
KEDSCQIN(deYSEGPCIGMQER	2400.0144	IPI00127352	IPI00127352	yes	yes	2,3		8.3747E-42	195.96
KDTCAQECSHFN(deITKVESR	2309.0529	IPI00132474	IPI00132474	yes	no		3	0.012911	80.859
KDTCAQECSHFN(deITK	1837.8087	IPI00132474	IPI00132474	yes	no	2,3		0.0007763	119.62
KDGSQDFN(deETWENYEK	1988.8388	IPI00128206	IPI00128206	yes	yes		2	0.0052475	81.594
KCQEAIN(deATCK	1321.6119	IPI00314443	IPI00314443	yes	no		3	0.011328	84.658
KCNSDNISYVIHCEPEN(deNTK	2421.0689	IPI00323053	IPI00323053	yes	no		3	0.0001521	93.381
KCNSDN(deISYVIHCEPENNTK	2421.0689	IPI00323053	IPI00323053	yes	no		3	0.0001521	93.381
KCEVICN(deQSNKPITITVEQSR	2503.2523	IPI00626537	IPI00626537	yes	yes		3	4.2044E-10	121.82
KCEAN(deITISSPAR	1445.7297	IPI00894972	IPI00894972	yes	no	2,3		0.0073163	104.41
KAWGISVINP(deNKTK	1554.8882	IPI00989096	IPI00989096	yes	yes	2,3,4		9.5788E-05	150.05
KAFITN(deFSMIIDGVTYPGVVKEK	2556.3662	IPI00312711	IPI00312711	yes	no	3,4		7.7626E-21	152.13
KAFITN(deFSMIIDGVTYPGVVK	2299.2286	IPI00312711	IPI00312711	yes	no	2,3,4		6.877E-18	146.72
IYWTDGDNISMANMDGSN(deHTIIFSGQK	3014.3539	IPI00119063	IPI00119063	yes	yes	3,4		5.4095E-38	156.74
IYWTDGDNIS(deMANMDGSNHTIIFSGQK	3014.3539	IPI00119063	IPI00119063	yes	yes	3,4		5.4095E-38	156.74
IYWISSGNHTIN(deR	1559.7845	IPI00119063	IPI00119063	yes	yes	2,3		2.2933E-123	254.18
IYWISSGN(deHTINR	1559.7845	IPI00119063	IPI00119063	yes	yes	2,3		2.2933E-123	254.18
IYVIDGTQN(deDTAFVFPR	1954.9789	IPI00308658	IPI00308658	yes	no	2,3		5.3282E-07	136.36
IYSDQCHHNISIIPPPTEIVCN(deR	2762.3269	IPI00123442	IPI00123442	yes	yes		3	1.2492E-06	99.409
IYSDQCHHN(deISIIPPPTEIVCNR	2762.3269	IPI00123442	IPI00123442	yes	yes		3	1.2492E-06	99.409
IYPPSSIPQGAEYN(deISAIVTIATK	2532.3476	IPI00122592	IPI00122592	yes	yes	3,4		0.016346	52.09
IYPIEN(deITIAPDPHVPDSIPPVAYNPWMDIR	3542.7908	IPI01023131	IPI01023131	yes	no	3,4		0.0029976	50.505
IYNVTHQFCN(deASVMDPTCVR	2411.0821	IPI00132600	IPI00132600	yes	no	2,3		4.865E-96	235.95
IYN(deVTHQFCNASVMDPTCVR	2411.0821	IPI00132600	IPI00132600	yes	no	2,3		4.865E-96	235.95
IYIETIPN(deTSIIIPFHNEGWSSIIR	2912.5436	IPI00314798	IPI00314798	yes	yes		3	4.6425E-06	87.326
IYIEIGGQMPTQESTN(deSSDSVSATAIVEVTEEFDR	3802.7731	IPI00122973	IPI00122973	yes	no		4	4.729E-14	97.352
IYIDHNN(deITR	1257.6466	IPI00120187	IPI00120187	yes	yes	2,3		3.9168E-07	141.88
IYAGMVSIMDEAVGN(deVTK	1896.9325	IPI00652358	IPI00652358	yes	no	2,3		4.6489E-09	145.91
IWVN(deSSDGISPVIGK	1570.8355	IPI00848693	IPI00848693	yes	yes	2,3		0.0001794	132.84
IWVHNGAPVN(deASASCR	1737.8369	IPI00464135	IPI00464135	yes	yes	2,3		0.0001814	121.13
IWRGPN(deVTVITGITR	1681.9628	IPI00877260	IPI00877260	yes	no		3	0.007667	82.908
IWN(deSTFIEEYSK	1515.7246	IPI00420835	IPI00420835	yes	no		2	0.0015664	110.31
IWIPVN(deITWADIEDKDGR	2140.0953	IPI00126253	IPI00126253	yes	no	2,3		2.3632E-15	169.28
IWIPVN(deITWADIEDK	1811.9458	IPI00126253	IPI00126253	yes	no	2,3		0.0003541	126.09
IWEATN(deSSIQQQIR	1672.8533	IPI00649090	IPI00649090	yes	no	2,3		3.2969E-07	158.04
IWDPSSHPGIQN(deISR	1705.8536	IPI00338209	IPI00338209	yes	no	2,3		0.0003182	127.36
IVYN(deHSSGR	1031.5148	IPI00133500	IPI00133500	yes	yes		2	3.5773E-05	132.88
IVVN(deVSDKNDHAPWFTSPSYDGR	2603.2405	IPI00623114	IPI00623114	yes	no		3	0.0009128	79.461
IVTQTIPCN(deKTIFWSK	1935.0288	IPI00307966	IPI00307966	yes	yes	2,3		0.0001896	131.56
IVTQTIPCN(deK	1172.6223	IPI00307966	IPI00307966	yes	yes		2	1.3483E-07	147.75
IVPCEFGVIYPIAEN(deFSR	2110.0557	IPI00121908	IPI00121908	yes	no	2,3		7.7403E-09	145.46
IVKIPSGTIVISN(deATEGDGGIYR	2359.2747	IPI00129159	IPI00129159	yes	no		3	2.4796E-111	158.92
IVIYIEHNIEKN(deSTKEEIIAAIEK	2796.5273	IPI00321190	IPI00321190	yes	no	2,3,4		5.7562E-133	236.95
IVIYIEHNIEKN(deSTK	1799.9781	IPI00321190	IPI00321190	yes	no	2,4		0.001121	124.25
IVIWTAEEQGGIGASQYYEIHKAN(deISK	3004.5294	IPI00126050	IPI00126050	yes	no		4	0.0005103	75.064
IVIIIDPAISN(deNSFSSNPYGPYDR	2651.3231	IPI00848693	IPI00848693	yes	yes	2,3,4		1.0257E-13	112.88
IVGSPFIGCTVVN(deK	1489.7963	IPI00134808	IPI00134808	yes	no	2,3		1.44E-32	191.79
IVGGTN(deASIGEWPWQVSIQVK	2268.1903	IPI00113057	IPI00113057	yes	yes	2,3		1.669E-25	167.51
IVFTHPN(deWTIS	1313.6768	IPI00125262	IPI00125262	yes	yes		2	0.0037563	91.549
IVEDIESFIKPYSVEEQKN(deITSCPDGAPFIQHGPDYR	4278.0579	IPI00124221	IPI00124221	yes	yes	4,5		9.1521E-14	97.312
IVDVN(deITSEGKVK	1400.7875	IPI00113528	IPI00113528	yes	no	2,3		0.0072112	131.66
IVDVN(deITSEGK	1173.6241	IPI00113528	IPI00113528	yes	no		2	3.8899E-07	154.36
IV(deVPINNRENISDPTSPIRR	2289.2553	IPI00136925	IPI00136925	yes	no	2,3		0.0004885	93.754
IV(deVPINNRENISDPTSPIR	2133.1542	IPI00136925	IPI00136925	yes	no		2	2.1047E-41	196.71
ITYN(deESR	881.4243	IPI01008227	IPI01008227	yes	no		2	0.0068777	111.74
ITWSNAN(deGTASYR	1439.6793	IPI00469426	IPI00469426	yes	no		2	0.0031956	88.075
ITVVYAEN(deGTVIQGTTVASVYK	2312.2264	IPI00317356	IPI00317356	yes	yes	2,3		7.8373E-223	217.86
ITVIDANDNAPAFN(deQSIYR	2121.0491	IPI00129613	IPI00129613	yes	yes	2,3		1.807E-41	192.09
ITTTSPWMIPAPHVWPEDHVFISTPNFN(deYTVQDFER	4272.0415	IPI00133500	IPI00133500	yes	yes	4,5,6		0.0001122	56.977
ITTTPTNGQQGTSIEEVVHADQSSCTFENINPGIEYN(deVSVYTVK	4827.2821	IPI00113539	IPI00113539	yes	no	4,5		8.4839E-06	54.09
ITSCATN(deASMCGDEAR	1742.7022	IPI00119063	IPI00119063	yes	yes	2,3		3.5413E-59	219.52
ITPNCCN(deTSTVADAYR	1841.8036	IPI00111228	IPI00111228	yes	yes		2	0.0004779	106.93
ITNSPQNQN(de)ITVPR	1580.8271	IPI00406603	IPI00406603	yes	yes		2	0.00953	71.279
ITN(deQTIGFSFAVEQDIPVK	2106.0997	IPI00624663	IPI00624663	yes	yes	2,3,4		2.5294E-96	237.14
ITN(deISIAMSGVYYVCK	1654.8422	IPI00126827	IPI00126827	yes	no		3	0.0003089	127.36
ITIAIN(deNTITPHTIPPGTIVYK	2376.3417	IPI00309230	IPI00309230	yes	no	2,3,4		2.8939E-209	281.07
ITHIPDDIPSNITVIN(deITHNQIR	2623.4082	IPI00320618	IPI00320618	yes	yes	3,4		1.6111E-06	98.816

ITHIPDDIPSN(deITVINITHNQIR	2623.4082	IPI00320618	IPI00320618	yes	yes	3,4	1.6111E-06	98.816
ITGSAGN(deYTVCQK	1397.6609	IPI00230412	IPI00230412	yes	yes	2,3	0.0032104	93.494
ITEFTHN(deSTMDYK	1585.7083	IPI00130010	IPI00130010	yes	no	2,3	0.0024667	119.96
ITDNMFCAGFKVN(deDTK	1859.8546	IPI00114206	IPI00114206	yes	yes	2	0.0060355	85.619
ITDIEN(deGTFANIPR	1559.7944	IPI00117957	IPI00117957	yes	yes	3	0.0006973	136.3
ITATDGENFATPIYIN(deITVAASR	2437.2489	IPI00623114	IPI00623114	yes	no	3	8.0026E-32	176.1
ISVPDGIKVSN(deSSAR	1528.8209	IPI00120761	IPI00120761	yes	no	2,3	3.2471E-11	162.37
ISTSPFAINI(deTMIPK	1631.8957	IPI00666034	IPI00666034	yes	no	2,3	2.6739E-07	132.86
ISTNI(deTIVCKPGDIESAPVIR	2282.2304	IPI00308971	IPI00308971	yes	yes	2	3.3111E-47	191.22
ISQN(deASIGPHVRPIPIQYEDKEVEPGTICDVAGWGVVTHAGR	4552.2921	IPI00116945	IPI00116945	yes	no	4	1.1192E-07	65.272
ISQN(deASIGPHVRPIPIQYEDK	2361.2441	IPI00116945	IPI00116945	yes	no	3	4.3532E-05	95.409
ISQKN(deQTVFER	1348.7099	IPI00130117	IPI00130117	yes	no	2	0.0023895	110.31
ISQAIGN(deITVVQK	1369.7929	IPI00112032	IPI00112032	yes	no	2,3	5.3195E-16	174.95
ISPIHIAIN(deFSIDPK	1663.9297	IPI00115976	IPI00115976	yes	yes	3	6.0202E-13	124.51
ISPGGAEMFQVQDMVVSQEKGN(deCSIQR	2994.3998	IPI00130117	IPI00130117	yes	no	3,4	0.0002863	76.049
ISNISN(deISHDIVQEATDHAYNIQQEADEISR	3509.6659	IPI00223446	IPI00223446	yes	yes	4	2.4191E-06	80.355
ISN(deVTPEDAGTYYCVK	1815.8349	IPI00129158	IPI00129158	yes	no	2	0.0001099	127.29
ISN(deITIIIEMEKESAR	1845.987	IPI00356462	IPI00356462	yes	no	2	0.000372	124.43
ISITN(deETFRK	1207.6561	IPI00123652	IPI00123652	yes	no	2	2.5783E-05	131.82
ISITN(deETFR	1079.5611	IPI00123652	IPI00123652	yes	no	2	0.0027068	118.71
ISISEN(deYTISIANAK	1622.8516	IPI00121378	IPI00121378	yes	no	2,3	6.0908E-34	194.3
ISIN(deESMQVVSR	1361.6973	IPI00471022	IPI00471022	yes	yes	2	0.0084863	75.102
ISHYN(deDTYPISPPQR	1786.8638	IPI00113039	IPI00113039	yes	no	2,3	7.4518E-12	165.34
ISHYKQN(deFSFCR	1585.746	IPI00381303	IPI00381303	yes	yes	2	0.0070188	95.417
ISHDGN(deETIPIHIYVK	1834.9577	IPI00405742	IPI00405742	yes	no	2,3,4	5.3492E-106	241.8
ISGKPTN(deVSVSVIMSEGDGICY	2312.1028	IPI00968978	IPI00968978	yes	no	2	7.214E-25	162.42
ISEN(deGSSVAGIISSPNMEK	1918.9306	IPI00399798	IPI00399798	yes	no	2,3	3.729E-19	164.94
ISEGNRTITI(deINVTRNDTGPYVCETR	2978.488	IPI00108535	IPI00108535	yes	no	3,4	1.9337E-06	92.034
ISEGNR(deTITIINVTRNDTGPYVCETR	2978.488	IPI00108535	IPI00108535	yes	no	3,4	1.9337E-06	92.034
ISEGNR(deTITIINVTR	1685.9424	IPI00108535	IPI00108535	yes	no	2	0.043764	66.059
ISEGN(deRTITIINVTRNDTGPYVCETR	2978.488	IPI00108535	IPI00108535	yes	no	3,4	1.9337E-06	92.034
ISEGN(deRTITIINVTR	1685.9424	IPI00108535	IPI00108535	yes	no	2	0.043764	66.059
ISEEFIKN(deISASAR	1563.8257	IPI00894970	IPI00894970	yes	no	2	0.0044681	108.58
ISDTN(deITAIPQGIPTSITEVHIDGNKITKVDAPSIK	3786.0415	IPI00123196	IPI00123196	yes	yes	4	0.0039614	51.595
ISDTN(deITAIPQGIPTSITEVHIDGNKITK	3075.6452	IPI00123196	IPI00123196	yes	yes	3,4,5	1.2527E-18	115.88
ISDTN(deITAIPQGIPTSITEVHIDGNK	2733.4185	IPI00123196	IPI00123196	yes	yes	2,3	2.5524E-29	151.01
ISCAFKTEN(deQTR	1453.6984	IPI00120245	IPI00120245	yes	no	2,3	0.0024515	134.56
ISASGAEIEAIEAQVIN(deISIK	2155.1736	IPI00312711	IPI00312711	yes	no	2,3	3.8225E-102	231.83
ISAIDNIIN(deHSSIFIKGAR	2068.1429	IPI00123342	IPI00123342	yes	yes	3	0.011918	70.802
ISAIDNIIN(deHSSIFIK	1783.9832	IPI00123342	IPI00123342	yes	yes	2,3	1.5968E-72	227
IRPIFN(deKSFESTVGQGSDTYSYIFR	2911.4505	IPI00108844	IPI00108844	yes	yes	3	1.6683E-32	132.65
IRPHFISVCDPDFSQIN(deCSEGYIQNYR	3314.5237	IPI00115626	IPI00115626	yes	yes	3,4	6.5978E-42	161.02
IRNPCTSE(deQNCTSPFSYK	2187.9677	IPI00132474	IPI00132474	yes	yes	2,3	4.727E-15	168.08
IRN(deSSIGGVINKYDVVIR	2002.1324	IPI00314673	IPI00314673	yes	yes	3,4	0.0002798	114.01
IRN(deSSIGGVINK	1256.7201	IPI00314673	IPI00314673	yes	yes	2	3.0062E-10	165.58
IRN(deATITQAITNK	1442.8205	IPI00876028	IPI00876028	yes	yes	3	0.012514	80.312
IRIN(deTTSDEKDPTNPFR	2003.0072	IPI00131881	IPI00131881	yes	no	3	0.004695	98.943
IRDYEEN(deSSSCHKEVQIIK	2334.1274	IPI00330594	IPI00330594	yes	yes	3	0.0038867	88.992
IRDYEEN(deSSSCHK	1623.6947	IPI00330594	IPI00330594	yes	yes	2,3	0.0089541	94.122
IRDNAIGN(deVSCTIHNEAIGQEK	2438.1972	IPI00124515	IPI00124515	yes	no	3	5.4941E-08	105.91
IR(deVNGTKEPIEFK	1529.8566	IPI00345112	IPI00345112	yes	no	2,3	0.023546	96.64
IQVTIYN(deCSFGR	1456.7133	IPI00405742	IPI00405742	yes	no	2	0.001106	122.39
IQTPIN(deYTEFQKPICIPSK	2276.1875	IPI00113057	IPI00113057	yes	yes	2,3,4	3.5689E-10	137.61
IQSPIFIIDANANIQN(deVTTSGNK	2457.2864	IPI00666034	IPI00666034	yes	no	3	8.0864E-12	125.45
IQQEPGIIIGPGN(deSTIFIER	2181.1794	IPI00124497	IPI00124497	yes	yes	3	0.000107	91.401
IQNN(deISYQMADIHHIK	1923.9625	IPI00453632	IPI00453632	yes	no	3	0.012026	66.246
IQIEFRPIDIN(deSTAAGIPR	2110.1535	IPI00115510	IPI00115510	yes	yes	2,3	4.3056E-08	128.79
IQFDDN(deGTYTCQVK	1687.7512	IPI00116476	IPI00116476	yes	no	2	0.0013728	115.82
IQDIEITGSPVSNISAHIFSN(deISSIEK	2898.4975	IPI00119522	IPI00119522	yes	yes	3,4	1.4373E-42	164.79
IQDIEITGSPVSN(deISAHIFSNISSIEK	2898.4975	IPI00119522	IPI00119522	yes	yes	3,4	1.4373E-42	164.79
IQDFIVDNETFSGFIQHN(deISIPR	2689.35	IPI00112614	IPI00112614	yes	yes	3,4	1.7807E-19	149.85
IQDFIVDN(deETFSGFIQHNISIPR	2689.35	IPI00112614	IPI00112614	yes	yes	3,4	1.7807E-19	149.85
IQCVDGN(deWTTIPVCIEEER	2318.0671	IPI00130010	IPI00130010	yes	no	2	7.5696E-53	206.7
IPYQGN(deATMIVVIMEK	1805.942	IPI00153258	IPI00153258	yes	yes	3	0.0001577	112.5
IPVAVIN(deATAK	1095.6652	IPI00122491	IPI00122491	yes	yes	2	0.010131	77.64
IPSSIENATSISIMN(deITGTAICHISDIPPDGIR	3492.7593	IPI00121776	IPI00121776	yes	yes	3,4	0.0001311	61.499
IPSSIEN(deATSISIMNITGTAICHISDIPPDGIR	3492.7593	IPI00121776	IPI00121776	yes	yes	3,4	0.0001311	61.499
IPQQIHHYIN(deASDWER	2005.9759	IPI00666034	IPI00666034	yes	no	2,3	0.0007021	102.64
IPPINIGEVITIPEANFPSFSIPNCN(deR	3008.543	IPI00322304	IPI00322304	yes	yes	4	0.0001579	76.049
IPNNTQWITW(deSPEGHK	1906.9326	IPI00125813	IPI00125813	yes	no	2,3	4.2404E-18	176.4
IPN(deISIDVIQPSFPEIIIESHMVMIR	2990.5973	IPI00221669	IPI00221669	yes	no	3,4	0.0008994	65.425
IPFNVIMNNVEDIIEQQTFN(deDTVSPR	3032.4913	IPI00310049	IPI00310049	yes	yes	3,4	0.0036181	59.814
INTT(deSDEKDPTNPFRFPNIGVEK	2618.2976	IPI00131881	IPI00131881	yes	no	3	0.017971	67.251
INTT(deSDEKDPTNPFR	1733.822	IPI00131881	IPI00131881	yes	no	2	0.0039818	95.067
INSAPVEGYSEHVGN(deKTTIR	2171.0971	IPI00349830	IPI00349830	yes	no	3,4	5.0158E-06	114.99
INSAPVEGYSEHVGN(deK	1699.8166	IPI00349830	IPI00349830	yes	no	2,3	2.2673E-45	205.68
INQINFQNIYQIFN(deETTSQVNK	2655.3293	IPI00356462	IPI00356462	yes	no	3	2.6398E-11	124.63
INNGGCQDICIITHQGHVN(deCSCR	2712.1737	IPI00119063	IPI00119063	yes	yes	3	0.0037558	65.149
INN(deITNIGPIDMK	1441.7599	IPI00330680	IPI00330680	yes	no	2,3	0.0024919	128.76
INMTIPDAIVPTFSISN(deHSIK	2297.209	IPI00469218	IPI00469218	yes	yes	2,3	6.4027E-05	100.39
INIDGSN(deYTIIK	1349.7191	IPI00119063	IPI00119063	yes	yes	2	0.0025462	95.264
INETHIFN(deGSNWIMIIYK	2192.1088	IPI00108844	IPI00108844	yes	yes	2,3	1.7488E-60	211.57
INAIN(deITSAIDR	1299.7147	IPI00750388	IPI00750388	yes	no	2	0.0032478	86.803
IN(deYTIVGEPIR	1273.703	IPI00120674	IPI00120674	yes	no	2	0.0004346	118.37
IN(deVTSPDIFR	1160.619	IPI00116913	IPI00116913	yes	yes	2	0.011583	83.397
IN(deVTEVYDKIK	1320.7289	IPI00225072	IPI00225072	yes	no	2,3	0.0009413	130.56

IN(deVSYTGERPSSNMVIVDVK	2207.1256	IPI00624663	IPI00624663	yes	yes	2,3	2.3472E-12	141.64
IN(deVSHAGAPIGEEYIIVFSR	2171.1375	IPI00785452	IPI00785452	yes	no	3	2.7453E-06	111.77
IN(deMTIPDAIVPTFSISNHSIK	2297.209	IPI00469218	IPI00469218	yes	yes	2,3	6.4027E-05	100.39
IN(deITEEEKIINRR	1626.9053	IPI00320204	IPI00320204	yes	yes	3	0.046346	76.655
IN(deITEEEKIINR	1470.8042	IPI00320204	IPI00320204	yes	yes	2	0.0085379	88.087
IN(deITEEEK	974.49204	IPI00320204	IPI00320204	yes	yes	2	0.030775	81.338
IN(deITAAQISQIEGIIQAR	1938.0898	IPI00115516	IPI00115516	yes	no	3	2.6623E-11	157.11
IN(deISEGEVAATVK	1329.714	IPI00458003	IPI00458003	yes	yes	2	0.0012353	137.46
IN(deGTDPIVAADSKR	1455.7682	IPI00119063	IPI00119063	yes	yes	2,3	0.0033548	107.69
IN(deFTGPGEPDSIR	1401.6888	IPI00320605	IPI00320605	yes	no	2	0.0039484	102.73
IN(deETHIFNGSNWIMIIYK	2192.1088	IPI00108844	IPI00108844	yes	yes	2,3	1.7488E-60	211.57
IN(deASIADIQSK	1158.6245	IPI00116913	IPI00116913	yes	no	2	0.0054874	85.554
IMQDPIQQAEGIYCN(deR	1934.8979	IPI00314549	IPI00314549	yes	yes	2,3	1.7203E-25	186.56
IMNAPIYIAEWQNITKN(deISEDR	2618.3163	IPI00930882	IPI00930882	yes	no	3	1.1041E-07	107.72
IMNAPIYIAEWQN(deITKNISEDR	2618.3163	IPI00930882	IPI00930882	yes	no	3	1.1041E-07	107.72
IMNAPIYIAEWQN(deITK	1903.9866	IPI00930882	IPI00930882	yes	no	3	2.7311E-12	164.65
IMESHPN(deGTFSAK	1417.666	IPI00468055	IPI00468055	yes	no	2,3	0.003403	97.463
IKV(deSNVSCEASVSK	1506.7712	IPI00135560	IPI00135560	yes	no	2	0.0063229	101.2
IKN(deTTNQIAIIK	1355.8136	IPI00985989	IPI00985989	yes	no	2,3	0.0030078	121.02
IKN(deITDISQK	1158.6608	IPI00119299	IPI00119299	yes	no	2,3	8.6587E-09	152.07
IKN(deATVTQYQIK	1405.7929	IPI00221547	IPI00221547	yes	no	2,3	2.1963E-05	150.46
IKIMESH(dePNGTFSAK	1658.845	IPI00468055	IPI00468055	yes	no	2,3	0.029411	68.972
IKIDN(deYSTQEIGR	1535.7944	IPI00122257	IPI00122257	yes	no	2,3	1.0476E-31	190.85
IKGMSETISN(deITQR	1576.8243	IPI00649911	IPI00649911	yes	no	3	0.0053899	104.41
IKEQVVGIIAQNN(deCSCESK	2176.0616	IPI00122592	IPI00122592	yes	yes	3	4.7933E-32	185.33
IK(deFIEAGIYEVPIIITDSGNPPKSNISIIR	3296.8384	IPI00323134	IPI00323134	yes	no	4	6.9908E-07	88.168
IIYISAEDFSIDHSPN(deSTAGPSCSIIQEAFRR	3580.7256	IPI00115530	IPI00115530	yes	yes	4	0.053249	39.388
IIYISAEDFSIDHSPN(deSTAGPSCSIIQEAFR	3424.6245	IPI00115530	IPI00115530	yes	yes	3,4	6.4023E-06	77.907
IIYGIEN(deTTQEIK	1520.8086	IPI00309999	IPI00309999	no	no	2	0.0025592	122.97
IIY(deEGGDIPDFRKENSSYQVINWR	2898.4301	IPI00153316	IPI00153316	yes	yes	3	7.0137E-13	112.56
IITN(deNSQTPIISPQEVVSCSPYAQGCDGGFPYIIAGK	3980.9288	IPI00130015	IPI00130015	yes	yes	4	8.5526E-45	136.86
IITHTIEGPSQN(deVTIIQIQPWQDPESWER	3414.7208	IPI00473190	IPI00473190	yes	no	3,4	6.3318E-23	126.32
IISQAPSTPSPNMFTINN(deETGDIITVAAGIDREK	3599.8141	IPI00323134	IPI00323134	yes	no	3,4	9.9372E-13	93.411
IISQAPSTPSPNMFTINN(deETGDIITVAAGIDR	3342.6766	IPI00323134	IPI00323134	yes	no	3,4,5	0.0002313	59.971
IIQVVYIHSNN(deITK	1640.925	IPI00123194	IPI00123194	yes	yes	2,3	4.7463E-104	240.46
IIQTAEHN(deISGAER	1537.7849	IPI00403938	IPI00403938	yes	no	2,3	5.1406E-70	220.85
IIQQQSN(deQSSQFIHSVER	2128.0661	IPI00876541	IPI00876541	yes	no	3	5.7025E-05	111.34
IIQDFSSDPASN(deITSHSIEKIPSAAEPVDHAPR	3528.7485	IPI00170355	IPI00170355	yes	no	4	0.0009637	56.366
IIPHIEKPIQN(deFTICFR	2125.1507	IPI00309214	IPI00309214	yes	yes	2,4	0.0019688	98.943
IIPAFN(deTTSGIPYPR	1645.8828	IPI00127672	IPI00127672	yes	no	3	1.5784E-05	143.94
IINKFN(deSSSSSIEEK	1681.8523	IPI00338561	IPI00338561	yes	yes	2,3	0.0009866	125.97
IINDYVSN(deQTQGMIK	1722.8611	IPI00135635	IPI00135635	yes	no	2	0.0008114	103.5
IIN(deTTDVYIIPSINPDGFER	2276.1689	IPI00130573	IPI00130573	yes	yes	2	2.7042E-15	152.06
IIN(deQTIRENIKK	1468.8726	IPI00460063	IPI00460063	yes	no	2	0.01266	106.26
IIN(deQTIRENIK	1340.7776	IPI00460063	IPI00460063	yes	no	2	4.7659E-05	148.28
IIN(deQTIR	856.51305	IPI00460063	IPI00460063	yes	no	2	2.1617E-13	150.84
IIN(deQTADMIQIASK	1544.8232	IPI00126864	IPI00126864	yes	no	2,3	0.0021449	102.29
IIN(deITSPEATAK	1256.6976	IPI00115089	IPI00115089	yes	yes	2	0.001281	116.24
IIN(deITNPVINQEIEAFSFPEDASSSR	2890.4349	IPI00221444	IPI00221444	yes	no	3,4	3.6619E-12	110.68
IIN(deITFIDITR	1317.7656	IPI00343568	IPI00343568	yes	no	2	2.3666E-05	147.33
IIN(deHSIIIHK	1186.7186	IPI00406603	IPI00406603	yes	yes	2	9.4357E-10	158.05
IIN(deETIYENAK	1306.6769	IPI00153756	IPI00153756	yes	yes	2	0.0026659	96.604
IIN(deASVIGDHTK	1266.6932	IPI00116056	IPI00116056	yes	yes	3	0.010512	72.705
IIMNEGGHYN(deASSGK	1576.7304	IPI01026847	IPI01026847	yes	no	2	0.002443	85.006
IIITAAPN(deITTSPAFRYDIIDVTR	2660.4538	IPI00314726	IPI00314726	yes	yes	3	6.1046E-10	103.85
IIITAAPN(deITTSPAFR	1684.9512	IPI00314726	IPI00314726	yes	yes	3	0.0058962	71.344
IIISPSAFHDGN(deFSIIIR	1999.0891	IPI00310519	IPI00310519	yes	no	3	2.9303E-05	116.43
IIISPEEN(deVTITCTAENQIER	2429.2108	IPI00121378	IPI00121378	yes	no	2,3	3.1837E-48	197.72
IIIPAKN(deTTHIK	1347.8238	IPI00127556	IPI00127556	yes	no	3	0.0089383	86.898
IIIN(deFTSMDIYR	1484.7697	IPI00125182	IPI00125182	yes	no	2	0.0022383	101.89
IIIKAPSHN(deTTEPDPHSISPEIQAIISEVAQHDVQNGR	4141.1192	IPI00165807	IPI00165807	yes	no	4	0.017627	41.936
IIGGGDEDAIRPQMQQIIDFETAIAN(deITIPQEKR	3779.9516	IPI00396840	IPI00396840	yes	no	4,5	0.000705	58.924
IIGGGDEDAIRPQMQQIIDFETAIAN(deITIPQEK	3623.8505	IPI00396840	IPI00396840	yes	no	3,4,5	5.5328E-35	138.36
IIFAN(deVSVR	1017.5971	IPI00173158	IPI00173158	yes	no	1,2	0.0018437	121.73
IIEYNYN(deSTK	1243.6085	IPI00109946	IPI00109946	yes	no	2	0.0062687	94.465
IIEFDSTN(deASEGAQPPGKPYPPYSIAK	2876.4232	IPI00459432	IPI00459432	yes	yes	3,4	5.3342E-42	162.83
IIDQACGTDN(deQTYASSCHIFATK	2600.1635	IPI00308484	IPI00308484	yes	yes	3	4.4593E-09	108.45
IIDIIPDGYPQISCIPKEEN(deATIATYPEFGVIDIK	3932.0169	IPI00124221	IPI00124221	yes	no	4	2.8241E-06	66.083
IIASPNEENM(deTEIISMR	1946.9441	IPI00322575	IPI00322575	yes	no	2,3	6.9055E-190	203.2
IIASN(deITETMR	1247.6544	IPI00132600	IPI00132600	yes	no	2	0.013213	73.632
IIAN(deISYTIQIDGHR	1712.921	IPI00894972	IPI00894972	yes	no	3	0.000782	104.41
IHYIYIQNNFITEIPIESFQN(deATGIR	3093.5924	IPI00122293	IPI00122293	yes	yes	3,4	4.3725E-111	233.56
IHVAQPEN(deDSHVAIIK	1769.9424	IPI00850413	IPI00850413	yes	yes	2,3,4	1.2325E-57	210.14
IHN(deGSEEAIAQYK	1458.7103	IPI00380296	IPI00380296	yes	no	3	0.0039946	102.4
IHKDN(deTTCYEFKK	1682.8086	IPI00119063	IPI00119063	yes	yes	2,3	0.038814	78.324
IHKDN(deTTCYEFK	1554.7137	IPI00119063	IPI00119063	yes	yes	2	0.0067778	98.105
IHIIPSMNPDGYEVAAAQGPN(deMSGYIVGR	3056.4848	IPI00128260	IPI00128260	yes	yes	3	1.3962E-56	184.34
IHIGNYN(deGTAGDAIR	1570.7852	IPI00310797	IPI00310797	yes	yes	2	0.004237	78.505
IH(deRINASIADIQSK	1564.8685	IPI00116913	IPI00116913	yes	no	2,3	0.0036029	128.95
IGYDPYANPPNYGNPDPIVIN(deNTHR	2810.3412	IPI00316430	IPI00316430	yes	no	3,4	5.14E-20	136.64
IGVTN(deASIVIFRPGSVR	1785.0261	IPI00652675	IPI00652675	yes	yes	2,3	5.1076E-48	207.92
IGVQMHPGQEIHN(deFTITGR	2134.0742	IPI00119299	IPI00119299	yes	no	2,3,4	4.9125E-08	123.63
IGPGEPIEIICN(deVSGAIPPPGR	2242.178	IPI00321348	IPI00321348	yes	no	3,4	5.4522E-19	149.73
IGNFSEIATHN(deQTFIKK	1947.0214	IPI00855103	IPI00855103	yes	no	2,4	0.013278	69.935
IGNFSEIATHN(deQTFIK	1818.9264	IPI00855103	IPI00855103	yes	no	2,3	2.5982E-35	199.37
IGN(deWTGEWPETEIVANIWMKQPDAAR	3011.46	IPI00322497	IPI00322497	yes	yes	4	0.019127	55.734

IGN(deWTGEWPETEIVANIWMK	2373.1464	IPI00322497	IPI00322497	yes	yes	2,3,4	1.5404E-53	208.49
IGN(deVTTWISSSNPPVTFAAIYWEEPDVSGHK	3401.6568	IPI00225072	IPI00225072	yes	no	3,4	0.001292	52.707
IGN(deFSEIATHNQTFIKK	1947.0214	IPI00855103	IPI00855103	yes	no	2,4	0.013278	69.935
IGN(deFSEIATHNQTFIK	1818.9264	IPI00855103	IPI00855103	yes	no	2,3	2.5982E-35	199.37
IGN(deETIQR	929.49304	IPI00322542	IPI00322542	yes	yes	2	0.010838	111.46
IGIYADVGN(deK	1048.5553	IPI00123814	IPI00123814	yes	no	2	0.012326	81.865
IGISSPTSDFQIEVTPQN(deITINPSSSGSMFSWR	3582.7301	IPI00322867	IPI00322867	yes	no	4	2.4019E-24	118.57
IGISFNSITVMEN(deGSIANVPHIR	2468.2846	IPI00123196	IPI00123196	yes	yes	3	7.2163E-169	172.57
IGHTN(deASIMIFEVK	1558.8177	IPI00858152	IPI00858152	yes	yes	3	0.0012601	129.54
IGDVEVNAGQN(deATFQCIATGR	2220.0593	IPI00123040	IPI00123040	yes	no	3	0.0022295	73.11
IGAINSTISN(deESKEAFIDWAR	2321.1652	IPI00187353	IPI00187353	yes	yes	2,3	1.3351E-15	140.63
IGAINSTISN(deESK	1332.6885	IPI00187353	IPI00187353	yes	yes	2	0.0027233	130.2
IGAINN(deSIIIIEDR	1539.8621	IPI00115516	IPI00115516	yes	no	3	0.0013456	125.03
IGAIN(deSTISNESKEAFIDWAR	2321.1652	IPI00187353	IPI00187353	yes	yes	2,3	1.3351E-15	140.63
IGAIN(deSTISNESK	1332.6885	IPI00187353	IPI00187353	yes	yes	2	0.0027233	130.2
IGAGSGPIWIDDIACNGN(deESAIWDCK	2818.2691	IPI00760106	IPI00760106	yes	no	3	0.0023465	61.612
IFISIN(deVTNSPSSQR	1661.8737	IPI00468674	IPI00468674	yes	no	2	0.010702	84.892
IFIFN(deQTGIEAKK	1507.8399	IPI00378224	IPI00378224	yes	yes	2,3	0.0061644	89.231
IFIFN(deQTGIEAK	1379.7449	IPI00378224	IPI00378224	yes	yes	2	1.5143E-16	178.15
IFFVNP(deAPPYIWPAHKNEIMINSSIMR	3184.6354	IPI00378698	IPI00378698	yes	no	3,4	2.1664E-06	88.036
IETIIIN(deGTDRK	1371.7722	IPI00119063	IPI00119063	yes	no	3	0.037787	67.385
IETIIIN(deGTDR	1243.6772	IPI00119063	IPI00119063	yes	no	2	6.0002E-09	160.52
IEPEHIQIQN(deISQEQIAQVATIATK	2801.4923	IPI00165807	IPI00165807	yes	no	3	6.1204E-68	209.24
IEN(deITTGTYTIHAQK	1688.8733	IPI00222429	IPI00222429	yes	yes	2,3	2.9921E-17	171.08
IEN(deISSTESGYTATITR	1841.9007	IPI00111960	IPI00111960	yes	no	2,3	8.2013E-173	271.64
IEKIEGIIAN(deVSR	1440.83	IPI00115516	IPI00115516	yes	no	2,3	0.0042565	136.17
IEIVPIIYDN(deDSIFVQTDK	2221.1518	IPI00405543	IPI00405543	yes	yes	2,3	8.232E-25	173.28
IEGVTMFAMGIEGAN(deNTQIEDIVSYPSR	3041.4474	IPI00990932	IPI00990932	yes	no	3,4	0.013839	43.895
IEGITN(de)ETYR	1194.5881	IPI00119299	IPI00119299	yes	no	2	0.0011019	114.86
IEGIIAN(de)VSR	1070.6084	IPI00115516	IPI00115516	yes	no	2	0.010971	84.658
IEDRFN(deSTIGPSEEQEKNWPGGPGR	2799.3212	IPI00115516	IPI00115516	yes	no	3,4	2.7725E-55	195.18
IEDRFN(deSTIGPSEEQEK	1977.928	IPI00115516	IPI00115516	yes	no	2,3	4.68E-11	159.81
IEDGFHPDAVAWAN(deITNAIR	2209.0916	IPI00165730	IPI00165730	yes	no	2,3	2.8542E-20	169.61
IECIHVPAN(deR	1207.6132	IPI00111456	IPI00111456	yes	no	2	0.014246	79.974
IEAYFSIEGFPAEQN(deASNR	2142.0018	IPI00114319	IPI00114319	yes	yes	2,3	1.1227E-113	240.68
IEAVIPAEFFEVISSSQN(deGSYHHIR	2829.4086	IPI00342158	IPI00342158	yes	no	3,4,5	2.0906E-29	157.98
IDWIGN(deCSGINDDSYGYR	2103.8956	IPI00121550	IPI00121550	yes	yes	2,3	2.2198E-48	203.26
IDVSQN(deVSSDTDQSCESTK	2098.8961	IPI00120769	IPI00120769	yes	no	2	7.1658E-07	120.11
IDVNSWIEN(deFTK	1464.7249	IPI00626793	IPI00626793	yes	no	2	0.002481	97.273
IDSFN(deDSTFMIVYTPITPTTQR	2546.2363	IPI00322575	IPI00322575	yes	no	2	3.8385E-21	150.91
IDPPCTNTT(deAPSNYINNPYVR	2406.1274	IPI00658539	IPI00658539	yes	no	2,3	1.9398E-09	118.54
IDNYS(deTQEIGR	1294.6153	IPI00122257	IPI00122257	yes	no	2	0.014418	72.705
IDKGGITINAN(deISGDAFIK	1946.0473	IPI00134585	IPI00134585	yes	no	2,3	6.6278E-05	101.96
IDITDFEKN(deSSFAQYQSFK	2267.0746	IPI00121312	IPI00121312	yes	yes	2,3	2.2491E-95	231.83
IDIPVNTSIPN(deVTEIK	1751.9669	IPI00466371	IPI01026704	no	no	2,3	1.0538E-12	168.41
IDIPVN(deTSIPNVTEIK	1751.9669	IPI00466371	IPI01026704	no	no	2,3	1.0538E-12	168.41
IDGSTN(deFTR	1009.4829	IPI00310797	IPI00310797	yes	yes	2	0.010376	98.048
IDGATVDTHHIPVN(deVTIR	1957.0381	IPI00751912	IPI00751912	yes	yes	3	0.0036335	73.296
IDFIVINE(deTR	1218.6608	IPI00125813	IPI00125813	yes	no	2	0.0013893	111.63
IDETDCIGN(deWTWQEGSQQTIK	2508.1227	IPI00122973	IPI00122973	yes	no	2,3	4.3589E-31	175.52
IDCYPDEHGASEAN(deCSAR	2050.8109	IPI00848693	IPI00848693	yes	yes	2,3	5.2648E-09	147.17
IDAYNEAAVSIIN(deSSTR	1822.9061	IPI00380296	IPI00380296	yes	yes	2,3	1.4937E-08	147.06
IDAPTNIQFVN(deETDRTVIVTWTPPR	2882.4927	IPI00113539	IPI00113539	yes	no	3,4	1.9523E-13	111.49
IDAPTNIQFVN(deETDR	1731.8428	IPI00113539	IPI00113539	yes	no	2,3	4.3825E-46	209.66
ICQN(deISKK	989.5328	IPI00126184	IPI00126184	yes	yes	2	7.4537E-05	131.06
ICQN(deISK	861.43784	IPI00126184	IPI00126184	yes	yes	2	0.028066	85.265
ICN(deECSDGSFHISK	1652.6923	IPI00515360	IPI00515360	yes	no	2	0.0022416	93.494
ICHTHGWN(deETSEIMPPGAVFSCIYR	2961.336	IPI00122399	IPI00122399	yes	no	3	0.0090373	55.734
ICEGDRFCIIDVMSTGSSSVGN(deATR	2731.2364	IPI00749655	IPI00749655	yes	no	3	2.4183E-06	90.912
ICDYENHFN(deTSK	1526.646	IPI00757830	IPI00757830	yes	no	2,3	0.0031424	87.667
IAYSNN(deGSDWK	1253.5677	IPI00123996	IPI00123996	yes	yes	2	0.01075	76.927
IAVTN(deTTMTGTVIK	1448.7909	IPI00108041	IPI00108041	yes	yes	2	1.0277E-23	186.05
IAVDWESIGYNI(deTR	1635.8257	IPI00123040	IPI00123040	yes	no	2,3	4.0835E-16	170.66
IATIAN(deFSEIGIEPGR	1686.8941	IPI00129041	IPI00129041	yes	no	3	0.0018703	89.541
IASPPSQGEVPPGPIPEAVIAIYN(deSTR	2759.4494	IPI00114457	IPI00114457	yes	yes	3,5	0.0006896	67.88
IASAVQKN(deATSTK	1317.7252	IPI00400016	IPI00400016	yes	no	2	0.011081	102.28
IAQEGGAAAIIIAN(deNSVIIPSSR	2264.2488	IPI00121776	IPI00121776	yes	yes	2,3,4	8.9385E-12	124.86
IAPASN(deVSHTVVIRPIK	1801.0574	IPI00467180	IPI00467180	yes	yes	2,3,4	2.2171E-108	239.58
IAPASN(deVSHTVVIR	1462.8256	IPI00467180	IPI00467180	yes	yes	3	0.0013634	112.08
IAN(deFSQSCTIYSGDDIVEAIPKPCPGCPR	3251.505	IPI00114958	IPI00114958	yes	no	3,4	7.0966E-38	156.41
IAN(deETGGHGSGPR	1251.5956	IPI00309230	IPI00309230	yes	no	2,3	3.7686E-23	183.96
IAMCQNIGYN(deVTK	1510.7272	IPI00117094	IPI00117094	yes	yes	2	2.436E-06	151.99
IAIYNSRFIN(deICSEMDGRVR	2413.1995	IPI00153749	IPI00153749	yes	yes	3	0.0066687	99.044
IAITPAHIIFIADN(deHTEPAAHFR	2554.3445	IPI00230324	IPI00230324	yes	no	3,4	4.343E-09	108.56
IAISFPN(deITSDGYPVIYPTGWSSSDNVNCRPR	3582.7202	IPI00466371	IPI01026704	no	no	3,4,5	3.4647E-16	101.5
IAIN(deITMYIK	1178.6733	IPI00134585	IPI00134585	yes	yes	2	0.0053382	96.113
IACIN(deGTVIR	1115.6121	IPI00134808	IPI00134808	yes	no	2	7.4627E-20	180.07
I(deNHCKFDEFFSQGCAPGYEKNSTICDICIGPIK	3904.7681	IPI00987951	IPI00987951	yes	no	4	5.9604E-11	93.176
I(deAVISGCKNTTSAAMVHCIR	2188.0915	IPI00131216	IPI00131216	yes	yes	2,3,4	1.4623E-05	108.45
HYQVTGYGING(deTGDSNDFWR	2286.009	IPI00221669	IPI00221669	yes	no	2	3.4602E-05	98.443
HYAN(deITNGMYR	1338.6139	IPI00224073	IPI00224073	yes	no	2	0.0015015	116.03
HVVIN(deGSSFGADGFPYVQVIK	2233.1532	IPI00742377	IPI00742377	yes	no	2,3	1.0415E-09	119.8
HVEDKFN(deETTQR	1502.7114	IPI00130654	IPI00130654	yes	no	2,3	7.9247E-16	176.18
HTIEIAPN(deSTAR	1308.6786	IPI00620800	IPI00620800	yes	no	2	0.003136	87.719
HTGPGIISMANAGPNTN(deGSQFFICTAK	2790.3218	IPI00554989	IPI00554989	yes	yes	3	2.6531E-10	98.766
HTDDITSIN(deNTIVNIR	1824.933	IPI00129304	IPI00129304	yes	yes	2,3	2.073E-25	185.95

HSVPVFCHPSFYN(deDTDFIGEEIDIVDVK	3278.523	IPI00416285	IPI00416285	yes	yes	4	0.0006676	64.954
HSVIWNAMIHPIQN(deMTIK	2132.1023	IPI00111794	IPI00111794	yes	no	2,3	0.0001138	103.6
HSQQPIITYEKYN(deVTDTPK	2261.1328	IPI00322418	IPI00322418	yes	yes	3,4	6.1321E-19	167.28
HSIEHFNN(deNTDHSHIFTIR	2318.0941	IPI00114958	IPI00114958	yes	no	3,4	1.1835E-12	144.97
HPGNFSSISCDYFAVN(deQSR	2184.9647	IPI00115976	IPI00115976	yes	yes	2,3	3.588E-08	126.07
HPGN(deFSSISCDYFAVNQSR	2184.9647	IPI00115976	IPI00115976	yes	yes	2,3	3.588E-08	126.07
HNN(deDTQHIWESDSNEFSVIADPR	2710.2008	IPI00129526	IPI00129526	yes	no	2,3,4	2.0174E-130	200.99
HN(de)ITITQGK	1010.5509	IPI00128358	IPI00128358	yes	no	2	0.0045495	112.71
HMQN(deITEEQPIEVK	1694.8298	IPI00756745	IPI00756745	yes	no	2	0.001319	123.76
HIWSIEISNKPN(deISEPEEPK	2346.1856	IPI00130573	IPI00130573	yes	yes	3	0.0033261	82.007
HIVFWN(deSSNPK	1327.6673	IPI00130752	IPI00130752	yes	no	2	0.0033679	93.345
HISN(deDTTTPESTMTVGQAR	2044.9484	IPI00876306	IPI00876306	yes	yes	2,3	5.6991E-08	122.44
HISFGEDYPGIVNPIDHTN(deVTAPQASMMFQYFVK	3852.828	IPI00626555	IPI00626555	yes	no	3,4,5	3.2629E-20	110.55
HIN(deITTISVAGGFR	1484.81	IPI00850413	IPI00850413	yes	yes	2	3.8317E-16	171.26
HIN(deASNPSEPATIIFTAAR	2009.033	IPI00421157	IPI00421157	yes	yes	2,3	1.1941E-113	240.13
HIIEN(deATASVSEAERK	1753.8959	IPI00396840	IPI00396840	yes	no	2,3	1.3579E-105	239.61
HIIEN(deATASVSEAER	1625.8009	IPI00396840	IPI00396840	yes	no	2,3	2.3191E-72	228.38
HGQIIKN(deFTIIQGPPGPR	1972.1007	IPI00129304	IPI00129304	yes	yes	3	1.4738E-08	147.12
HGHFTGFN(deGSTIR	1429.6851	IPI00117986	IPI00117986	yes	yes	2,3	0.015692	68.809
HGFYCYIIGSTISTFTDAN(deHTCTNEK	3036.3382	IPI00126186	IPI00126186	yes	yes	3	2.8291E-67	200.29
HFN(deDSGACVPR	1258.5513	IPI00468814	IPI00468814	yes	yes	2	0.010987	76.655
HFKGQTQN(deYSTIIIEEASER	2350.1553	IPI00124666	IPI00124666	yes	no	2,3	3.1601E-133	252.05
HFFN(deVSDEIAIVTIVK	1830.988	IPI00466371	IPI01026704	no	no	2,3	6.5042E-19	179.74
HEN(deNTKDNSIQHEFSITR	2169.0199	IPI00109727	IPI00109727	yes	yes	2,3	6.5714E-07	135.23
HEEGHMINC(deTCFGQGR	1931.7825	IPI00113539	IPI00113539	yes	no	2,3	0.0013427	94.717
HDYIIIPEGAITN(deTTR	1812.937	IPI00121421	IPI00121421	yes	no	2	0.0001931	118.47
HDVFCGFIICTN(deITR	1851.876	IPI00648288	IPI00648288	yes	no	3	0.0009529	101.53
HDPN(deMTCDYVIK	1491.6486	IPI00119299	IPI00119299	yes	no	2	0.0021946	102.4
HAN(deWTITPIK	1179.64	IPI00317356	IPI00317356	yes	yes	2,3	9.6956E-11	160.36
HAIEYFNN(deNTGHSHIFAIR	2240.0875	IPI00400202	IPI00400202	yes	no	3	0.0004429	89.866
HAEISFVFITDGVTGN(deDSIEESVHSMR	2976.3924	IPI00621027	IPI00621027	yes	yes	4	0.0001605	75.773
GYPYNGTNYVN(deVTMHIR	1997.9418	IPI00222819	IPI00222819	yes	yes	2,3	0.0019276	99.044
GYPCGGN(deKTASSVIEITCAK	2111.998	IPI00308971	IPI00308971	yes	yes	3	0.0016636	87.16
GYNSS(deQDIPSIVIDFVK	1880.952	IPI00124666	IPI00124666	yes	yes	2,3	3.4773E-37	195.77
GYNPVTKN(deVTVR	1346.7306	IPI00130573	IPI00130573	yes	yes	2	0.0034482	130.1
GYITSFEMFN(deSTFK	1670.765	IPI00262024	IPI00262024	yes	no	2,3	7.5602E-145	264.29
GYIITINFTKN(deTTR	1640.8886	IPI00469218	IPI00469218	yes	yes	2,3	4.0089E-218	296.22
GYIITIN(deFTKNTTR	1640.8886	IPI00469218	IPI00469218	yes	yes	2,3	4.0089E-218	296.22
GYIITIN(deFTK	1168.6492	IPI00469218	IPI00469218	yes	yes	2	2.8507E-88	231.42
GWKENPVEFDSIFN(deESK	2024.948	IPI00129070	IPI00129070	yes	no	2,3	6.8917E-20	173.37
GWIN(deGSIVGFYK	1339.6925	IPI00134585	IPI00134585	yes	no	2	1.4929E-59	194.24
GVYEITHNN(deK	1173.5778	IPI00378635	IPI00378635	yes	no	2,3	6.6253E-12	133.81
GVVDSDDIPIN(deVSR	1484.7471	IPI00129526	IPI00129526	yes	no	2	1.0588E-23	186.05
GVTSVSQIFHSPDIAIRDTYVN(deASQSIYGSSPR	3551.7645	IPI00122977	IPI00122977	yes	no	4	0.0009367	56.533
GVTNDIISVQGNTGPSWIN(deKTEK	2457.25	IPI00344686	IPI00344686	yes	yes	2,3,4	8.8846E-129	186.06
GVTHIN(deISGIKMPR	1521.845	IPI00119063	IPI00119063	yes	yes	3	0.0035809	125.97
GVTHIN(deISGIK	1137.6506	IPI00119063	IPI00119063	yes	yes	2,3	6.2281E-160	203.61
GVSSSEN(deFTSPVMEFWERR	2244.027	IPI00466652	IPI00466652	yes	no	3	1.5129E-10	138.91
GVSSSEN(deFTSPVMEFWER	2087.9259	IPI00466652	IPI00466652	yes	no	2,3	9.8578E-29	188.04
GVQIIVFPEDGIHGFN(deFTR	2145.1007	IPI00321375	IPI00321375	yes	no	2,3,4	1.27E-16	160.43
GVNVITVN(deATDADSK	1502.7577	IPI00623114	IPI00623114	yes	no	2	7.2415E-25	185.26
GVN(deVTMPSQPGVPPISSTQIQIDPAIQEFQIVDISRR	4017.0994	IPI00515360	IPI00515360	yes	no	3,4	2.0776E-08	73.227
GVN(deVTMPSQPGVPPISSTQIQIDPAIQEFQIVDISR	3860.9982	IPI00515360	IPI00515360	yes	no	3,4,5	1.4546E-19	106.44
GVN(deETIIVNEIK	1327.7347	IPI00338018	IPI00338018	yes	no	2	0.0012273	128.95
GVIPGEDWTVFQSN(deHSTYEPVIIAK	2786.3915	IPI00404189	IPI00404189	yes	no	2,3,4	0.0001739	77.631
GVIMVGN(deETTYEDGHGARKNITDIVEGAK	3073.5139	IPI00649186	IPI00649186	yes	no	3	0.042133	55.881
GVIMVGN(deETTYEDGHGARK	2032.9636	IPI00649186	IPI00649186	yes	no	3	0.05641	54.281
GVIMVGN(deETTYEDGHGAR	1904.8687	IPI00649186	IPI00649186	yes	no	2,3	1.2115E-109	245.19
GVIDKDPCSVIPSDYDIFIN(deISR	2622.3	IPI00132279	IPI00132279	yes	yes	3	2.27E-08	104.84
GVFITNE(deTGQPIIGK	1572.8512	IPI00111960	IPI00111960	yes	no	2,3	2.9172E-146	260.96
GVDSCHGDSGGAFAFQVPN(deVTVPK	2445.1383	IPI00653675	IPI00653675	yes	no	3	1.0257E-05	84.946
GVDIDAPYYNYIISFTVPDIDN(deVTVIDYDAR	3535.7035	IPI00119063	IPI00119063	yes	yes	3,4	1.3323E-34	137.24
GVAVTN(deTSQIGFR	1348.7099	IPI00471089	IPI00471089	yes	no	2	5.4347E-86	233.59
GV(deIMVGNETTYEDGHGARKNITDIVEGAK	3073.5139	IPI00649186	IPI00649186	yes	no	3	0.042133	55.881
GTFTDCAIANM(deTQQIR	1825.8451	IPI00621027	IPI00621027	yes	no	2,3	3.2039E-194	279.44
GTEVN(deATVIGESDPIDEVQGFIFGK	2621.2861	IPI00762897	IPI00762897	yes	yes	2,3	1.0968E-169	259.6
GTDPSISPSPAATPPAPGAEISYIN(deHTFR	2950.4461	IPI00762091	IPI00762091	yes	yes	3,4	9.4528E-23	124
GTCEQGPSIVTPPKDIWN(deVTGAK	2454.2213	IPI00652107	IPI00652107	yes	no	3	2.6893E-11	124.31
GTAGNAIMDGASQIVGEN(de)R	1859.8796	IPI00279079	IPI00279079	yes	yes	2,3	0	308.02
GSYEIIAANGEDTDVDKITNSPQNQN(deITVPR	3358.6277	IPI00406603	IPI00406603	yes	yes	3	8.1032E-34	133.27
GSTQYIENIGFN(deMSK	1687.7876	IPI00116105	IPI00116105	yes	yes	2,3	2.1164E-06	149.86
GSPGN(deASQGSIHIHSPQIAIQADPR	2537.2735	IPI00114044	IPI00114044	yes	yes	2,3,4	2.5083E-08	100.28
GSPAN(deVTEAR	1000.4938	IPI00808422	IPI00808422	yes	no	2	0.0097104	94.465
GSNNSGHDN(deHSQFVR	1654.7196	IPI00330594	IPI00330594	yes	yes	2,3	1.5793E-05	144.29
GSNFN(deISKTDNIEECQK	1982.9004	IPI00113057	IPI00113057	yes	yes	2,3	2.8548E-109	248.13
GSNFN(deISK	865.42938	IPI00113057	IPI00113057	yes	yes	2	0.0025563	120.99
GSN(deNSGHDNHSQFVR	1654.7196	IPI00330594	IPI00330594	yes	yes	2,3	1.5793E-05	144.29
GSN(deFTAICVIK	1208.6223	IPI00120155	IPI00120155	yes	yes	2	2.6444E-07	156.36
GSIQSANDISSQTQGFIQHSMDN(deISAQIQTVR	3460.6641	IPI00330594	IPI00330594	yes	yes	3,4	4.6466E-60	175.59
GSIN(deVSYNIGPGFTGSEYSR	2103.9861	IPI00113042	IPI00113042	yes	no	2,3	5.1911E-258	300.74
GSEVEDEDIEIFN(deTSVQIRPPSTAPGPETAAFIER	3800.8381	IPI00381357	IPI00381357	yes	no	3,4	6.9911E-73	178.95
GSAIPHPSN(deATWEIK	1606.8104	IPI00119299	IPI00119299	yes	no	2,3	2.0579E-05	141.85
GRADECAIPYIGATCYCDIFCN(deR	2781.1768	IPI00115458	IPI00115458	yes	no	3	3.2366E-11	122.94
GQTQNYST(deIIIEEASER	1937.933	IPI00124666	IPI00124666	yes	no	2,3	7.1316E-48	202.16
GQTGIGFIYASGIGVN(deSSQAK	2054.0433	IPI00131143	IPI00131143	yes	no	2,3	4.459E-48	196.78
GQQQVIPIPIAPIVPISIVGSASN(deTSR	2741.544	IPI00162831	IPI00162831	yes	yes	3,4	1.7298E-18	115.36

GQNQPVIN(deITNR	1352.7161	IPI00111908	IPI00111908	yes	no		2	0.0010771	139.48
GQAQSDAAGIPGAESRN(deDSIPGAGSEADGIEGK	3111.4341	IPI00317487	IPI00317487	yes	yes	3,4		1.6384E-34	133.61
GPPGVN(deGTQGFQGCPGQR	1812.8326	IPI00830749	IPI00830749	yes	yes	2,3		5.7206E-15	162.46
GPNKIQC(deVDGNWTTIPVCIEEER	2714.2792	IPI00130010	IPI00130010	yes	no		3	4.0254E-09	111.49
GPN(deFTSPASITFTTGIKPPQDIEAK	2616.3435	IPI00458413	IPI00458413	yes	no		3	6.9277E-08	100.28
GPMNQCIVATGIDVIGN(deR	1913.9452	IPI00118475	IPI00118475	yes	yes		3	3.2022E-06	121.82
GPGIKPN(deQTSK	1125.6142	IPI00221426	IPI00221426	yes	yes		2	1.0052E-16	179.19
GPFDSFVVQYQDTEN(deETQAIIVDGDQDKVIISGIEPK	4094.0008	IPI00458413	IPI00458413	yes	no	4,5		7.1994E-08	71.623
GPECSQNY(deTAPTGVIK	1720.809	IPI00123996	IPI00123996	yes	yes		2	9.4561E-26	188.27
GPDISN(deCTSHWVNQIAQK	2053.964	IPI00876558	IPI00876558	yes	no		3	3.8392E-28	182.02
GPCSHICIINYN(deR	1602.7395	IPI00119063	IPI00119063	yes	yes	2,3		0.0030016	109.44
GNYGWQAGN(deHSAFWGMTIDEGIR	2566.1448	IPI00115458	IPI00115458	yes	no	2,3		2.2862E-39	185.61
GN(deTTAIDKEIAR	1287.6783	IPI00108535	IPI00108535	no	no	2,3		2.3472E-06	155.48
GN(deTTAIDK	818.4134	IPI00108535	IPI00108535	no	no		2	0.049839	70.056
GN(deSTIAVPK	885.49198	IPI00808296	IPI00808296	yes	no		2	9.1054E-17	162.36
GN(deSSIYPVVSPSIQQIVIEK	2157.1681	IPI00985989	IPI00985989	yes	no	2,3		2.2593E-114	245.66
GN(deQSSQWIYEAMAK	1611.7351	IPI00137792	IPI00137792	yes	yes		2	5.1167E-05	148.03
GN(deMTWYAAGK	1097.4964	IPI00120953	IPI00120953	yes	yes		2	0.0002008	127.42
GN(deITIER	801.43447	IPI00279010	IPI00279010	yes	no		2	0.0045245	120.46
GN(deITEYQCHQYITK	1753.8094	IPI00122399	IPI00122399	yes	no	2,3		6.3374E-56	212.39
GN(deISTEKFVEEIK	1492.7773	IPI00466371	IPI01026704	no	no		2	0.008835	88.021
GN(deHSIFGIEVATIGQGPDCPSVNER	2653.2555	IPI00459432	IPI00459432	yes	yes		3	6.8895E-111	236.38
GN(deGSCVCHAGWQGIR	1657.7202	IPI00987265	IPI00987265	yes	no		2	0.000487	120.66
GMVIDENSNN(deITGAVISVTGINHDVTSGEHGDYFR	3717.7329	IPI00128260	IPI00128260	yes	yes		4	4.1096E-19	102.57
GMSETISNIT(deQR	1335.6453	IPI00649911	IPI00649911	yes	no		2	0.0014164	111.79
GMNYN(deSSVVK	1097.5175	IPI00416285	IPI00416285	yes	yes		2	4.1388E-07	141.08
GKSN(deQTECFNFIR	1599.7464	IPI00890869	IPI00890869	yes	yes		2	0.0096446	83.087
GKNN(deQTECFNHVR	1602.7321	IPI00124666	IPI00124666	yes	no	2,3		0.0041205	136.7
GKIYWTDGDNISMANMDG(deSNHTIIFSGQK	3199.4703	IPI00119063	IPI00119063	yes	yes		4	8.8661E-07	87.511
GKIYWTDGDN(deISMANMDGSNHTIIFSGQK	3199.4703	IPI00119063	IPI00119063	yes	yes		4	8.8661E-07	87.511
GIYHIN(deITVGGIPFK	1627.9086	IPI00115536	IPI00115536	yes	yes		3	0.0067009	72.496
GIWKIPFSPENTREEDFYVN(deETSTVK	3085.5033	IPI00116105	IPI00116105	yes	yes	3,4		3.7633E-10	101.6
GIVSGGVYNSHVGCIPYTIPPCEHHVN(deGSRPPCTGEGDTPR	4430.0379	IPI00113517	IPI00113517	yes	yes	4,5		7.4643E-05	56.288
GIVMNYPHITN(deITTIGQSVEYR	2505.2686	IPI00130573	IPI00130573	yes	yes		3	1.7704E-39	188.96
GISYQIIGN(deHSK	1315.6884	IPI00623114	IPI00623114	yes	no	2,3		0.0011066	128.54
GISPGN(deYSVR	1048.5302	IPI00128358	IPI00128358	yes	no		2	0.0047069	97.813
GIPDQKVN(deFTCK	1405.7024	IPI00129158	IPI00129158	yes	no		2	0.0072347	93.011
GINIT(deEDTYKPR	1405.7201	IPI00119809	IPI00119809	yes	yes	2,3		2.8061E-69	223.31
GINCN(deISR	932.4498	IPI00136565	IPI00136565	yes	no		2	0.0020797	122.52
GIMVGN(deGSVIGVVQAVDAETGK	2100.0885	IPI00342158	IPI00342158	yes	no	2,3		6.5019E-140	250.05
GIMIIIN(deDTQHFSNNVKGEIGQFYR	2893.4545	IPI00312711	IPI00312711	yes	no		3	1.0531E-14	120.71
GIMIIIN(deDTQHFSNNVK	1942.9935	IPI00312711	IPI00312711	yes	no	2,3		2.1892E-27	184.02
GIIN(deATISVAEINHPVTTYK	2140.1528	IPI00130573	IPI00130573	yes	yes	2,3		3.887E-114	242.98
GIIGISN(deATIYWHIPDTAYPGIYR	2690.3857	IPI00458077	IPI00458077	yes	yes		3	5.7732E-08	99.86
GIIGHNN(deKSSASFR	1486.7641	IPI00162870	IPI00162870	yes	yes	2,3		0.006242	96.464
GIIGHNN(deK	851.46135	IPI00162870	IPI00162870	yes	yes		2	0.0043223	119.21
GIFPDGSHEISGN(deTSITPDK	2070.9858	IPI00894972	IPI00894972	yes	no		3	4.1683E-06	109.87
GIDWYMEFFPIPSN(deTTSDFYFEK	2833.2622	IPI00404189	IPI00404189	yes	yes		3	0.012975	56.304
GIAN(deISNFIR	1103.6087	IPI00129158	IPI00129158	yes	no		2	1.0169E-25	183.03
GIAEVTEN(deVTEGGVTK	1602.8101	IPI00130271	IPI00130271	yes	no		2	1.0126E-88	237.46
GHVDPAN(deDTFDIDPR	1667.754	IPI00453501	IPI00453501	yes	no		2	0.0014288	95.236
GHTDRCDEAQAIQVWN(deDTHPEVISQKPFDK	3520.643	IPI00108003	IPI00108003	yes	no		4	0.0006927	62.605
GHIIIAQVATNPQGITGTGN(deTTSEMDPSHRK	3230.6102	IPI00321375	IPI00321375	yes	no		4	0.012604	47.225
GHIIIAQVATNPQGITGTGN(deTTSEMDPSHR	3102.5153	IPI00321375	IPI00321375	yes	no	3,4		4.472E-67	182.19
GHICN(deQTQNIQSSK	1613.758	IPI00987886	IPI00987886	yes	yes	2,3		0.001371	115.12
GHFYYN(deISDVR	1369.6415	IPI00135560	IPI00135560	yes	no	2,3		0.0009257	109.6
GHAIGISCQSEN(deGTAPITYHIMK	2484.189	IPI00406901	IPI00406901	yes	no	3,4		1.8335E-49	199.41
GHAHIAAIVNHDSYN(deFSHR	2145.0253	IPI00133365	IPI00133365	yes	no	3,4		3.1444E-10	132.84
GGTVEYTN(deEKHTIEIAPNSTAR	2387.1717	IPI00620800	IPI00620800	yes	no		3	0.016083	63.447
GGITINANISG(deDAFIK	1589.8413	IPI00134585	IPI00134585	yes	no		2	0.0094219	88.133
GGIN(deITAVTVTAENDHTVAFIGTSDGR	2715.3464	IPI00405742	IPI00405742	yes	no		3	3.5819E-22	123.36
GGGGPWAN(deGSAIAICQR	1670.7947	IPI00453501	IPI00453501	yes	no		2	0.003925	73.498
GFQWVTGDN(deHTSYSR	1753.7808	IPI00129423	IPI00129423	yes	yes		2	8.3888E-25	184.48
GFPQIIGAAGN(deVSAGSVIIR	1926.0687	IPI00754853	IPI00754853	yes	no		3	7.1797E-13	143.28
GFN(deATYHVR	1063.5199	IPI00411025	IPI00411025	yes	yes	2,3		0.0030181	117.7
GFN(deASYIR	926.46102	IPI00461962	IPI00461962	yes	yes		2	0.015895	95.099
GFGVAFVGN(deYTGSIPNEAAINTVR	2453.2339	IPI00165807	IPI00165807	yes	no		3	8.8995E-06	99.409
GFDTYFGYIIGSEDYYTHEACAPIESIN(deGTR	3545.5722	IPI00652358	IPI00652358	yes	no		3	1.1125E-39	141.36
GETASIICN(deISVR	1418.7188	IPI00321348	IPI00321348	yes	no		2	5.128E-23	181.71
GENPSQYGITAFNHPIN(deITK	2200.0913	IPI00112614	IPI00112614	yes	yes		3	3.1387E-08	121.26
GEIQSEN(deSSITISSSNR	1807.8548	IPI00466371	IPI01026704	no	no	2,3		2.7028E-91	235.75
GEIN(deSTIFSSRPK	1434.7467	IPI00460063	IPI00460063	yes	no	2,3		5.7899E-32	194.98
GEIN(deSTIFSSR	1209.599	IPI00460063	IPI00460063	yes	no		2	0.01639	71.241
GECYYTN(deGTQR	1347.5514	IPI00110910	IPI00110910	yes	no		2	4.0506E-33	197.07
GDTHTQIIEGIQFN(deITQTSEADIHK	2795.3726	IPI00406302	IPI00406302	no	no	3,4		7.3685E-124	193.42
GDSGGAIVFIDN(deETQR	1677.7958	IPI00467068	IPI00467068	yes	no		2	1.6982E-25	186.72
GDRN(deITWR	1016.5152	IPI00221426	IPI00221426	yes	yes	2,3		0.0033912	126.07
GDN(deQSPIEIHTK	1337.6575	IPI00221890	IPI00221890	yes	yes	2,3		0.0013258	136.93
GDHHQISHYN(deITGVR	1732.8394	IPI00338209	IPI00338209	yes	no	2,3		0.0003931	112.96
GDHGAN(deFSCR	1119.4516	IPI00122973	IPI00122973	yes	no		2	5.9184E-06	136.96
GDGPFTVFVPHADIISN(deMSQDEIAR	2715.2963	IPI00987265	IPI00987265	yes	no		3	1.6193E-29	158.52
GDEKEN(deITAEAIDISIK	1844.9367	IPI00124725	IPI00124725	yes	no	2,3		0	200.67
GDDIYTNV(deTVSIVEAIVGFEMDITHIDGHK	3287.602	IPI00320241	IPI00320241	yes	yes		4	1.3212E-53	165.13
GCKDNA(deTDSVPIR	1431.6776	IPI00119063	IPI00119063	yes	yes	2,3		1.2718E-42	202.29
GCHVN(deECISR	1230.5234	IPI00119063	IPI00119063	yes	yes		2	0.0022305	106.29
GCADYCNQ(deTITKR	1585.6977	IPI00987265	IPI00987265	yes	no		2	0.018287	73.386

GAYPVFFN(deFTR	1317.6506	IPI00308328	IPI00308328	yes	yes		2	0.0074186	88.441
GATISHHNIVN(deNSMIIGQR	2061.0538	IPI00122633	IPI00122633	yes	yes	2,3		5.4705E-06	117.26
GAIQIPTVSFSHEESN(deTTAIAEFGEYIRK	3194.5884	IPI00224073	IPI00224073	yes	yes	3,4		0.0006118	66.017
GAIQIPTVSFSHEESN(deTTAIAEFGEYIR	3066.4934	IPI00224073	IPI00224073	yes	yes		3	5.3721E-23	129.23
GAIDIMIQVN(deMTPGHSSAPPK	2163.0816	IPI00224073	IPI00224073	yes	yes	2,3,4		2.8404E-25	162.77
GAGEVSPAEHSSKPTN(deISAK	1965.9756	IPI00230718	IPI00230718	yes	no	2,3		1.7055E-41	194.27
GAFVSKNPCN(deITREDYAPIVK	2378.2053	IPI00307966	IPI00307966	yes	yes		3	1.5229E-37	180.78
GAFVSKNPCN(deITR	1462.7351	IPI00307966	IPI00307966	yes	yes	2,3		0.0041199	136.38
GAFISN(deFTMTVNGMTFTSSIK	2253.081	IPI00970438	IPI00970438	yes	no	2,3		3.3539E-26	169.51
GAFFPIKEDN(deWSIPNR	1889.9424	IPI00135560	IPI00135560	yes	no	2,3		0.0002452	129.38
GAAAPSAPHWN(deETAEK	1635.7641	IPI00648288	IPI00648288	yes	no		3	0.0044743	74.789
FYPEDIQIIWIENGNVSRNDTPKN(deITK	3203.6251	IPI00129158	IPI00129158	yes	no	3,4		2.2042E-16	119.79
FYPEDIQIIWIENGNVSRN(deDTPKNITK	3203.6251	IPI00129158	IPI00129158	yes	no	3,4		2.2042E-16	119.79
FYPEDIQIIWIENGN(deVSRNDTPKNITK	3203.6251	IPI00129158	IPI00129158	yes	no	3,4		2.2042E-16	119.79
FYPEDIQIIWIENGN(deVSR	2192.0902	IPI00129158	IPI00129158	yes	no	2,3		0.0029295	78.234
FYFGGSPISPQYAN(deFTGCISNAYFTR	2964.3541	IPI00223446	IPI00223446	yes	yes		3	0.0007571	69.27
FVPNSNMN(deFTGQAYSGR	1888.8526	IPI00108535	IPI00108535	no	no	2,3		0	235.71
FVN(deSTGYITEAEK	1457.7038	IPI00153187	IPI00153187	yes	yes		3	0.0092215	73.466
FVFMEEGSN(deKTVEPR	1768.8454	IPI00133074	IPI00133074	yes	yes	2,3		0.0044346	92.265
FVFMEEGSN(deK	1186.5329	IPI00133074	IPI00133074	yes	yes		2	0.016505	77.379
FTQDAYSAVVKEN(deSTEAR	2014.9596	IPI00623114	IPI00623114	yes	no		2	1.3984E-11	159.95
FTFTSHTPGEHQICIHSN(deSTK	2428.123	IPI00473680	IPI00473680	yes	no	2,3,4		9.2099E-259	222.3
FTFTSHTPGDHQICIHSN(deSTR	2442.1135	IPI00153468	IPI00153468	yes	no	2,3,4		3.2512E-48	197.67
FTCN(deQTTDVIIIHSK	1775.8876	IPI00319509	IPI00319509	yes	yes	2,3		1.2483E-87	231.06
FTAPDTIFAN(deGSVYPPNEGFCPCR	2716.205	IPI01008227	IPI01008227	yes	no	2,3,4		6.5147E-33	161.66
FSVN(deQTTIITHEK	1516.7886	IPI00848693	IPI00848693	yes	yes	2,3		8.0167E-07	157.86
FSPPVVN(deVTWIR	1413.7769	IPI00110808	IPI00110808	yes	no		3	0.0005311	137.89
FSMSDTYDIQDVIADVGIKDIFTN(deQSDFADTTK	3699.7138	IPI00116105	IPI00116105	yes	no	3,4		3.6283E-60	176.7
FSHN(deGTCAAEGK	1277.5459	IPI00320675	IPI00320675	yes	yes		2	0.00156	110.38
FSGAN(deDTDFR	1128.4836	IPI00221669	IPI00221669	yes	no		2	1.2376E-05	132.32
FSEIIVNN(deATEEIIVK	1817.9775	IPI00321190	IPI00321190	yes	no		2	1.0236E-34	197.15
FSATEVPEKGAGEVSP(deAEHSSKPTNISAK	2954.4621	IPI00230718	IPI00230718	yes	no	3,4		7.7413E-56	186.96
FS(deIAIIGHYNRGNISTEK	2019.0538	IPI00466371	IPI01026704	no	no		3	0.02928	65.374
FQSAAIGAIQEASEAYIVGIFEDTN(deICAIHAK	3436.6973	IPI00989576	IPI00989576	yes	no		3	5.3679E-40	143.27
FQISPQIQFSPEEVIGMVIN(deYSR	2681.3523	IPI00123342	IPI00123342	yes	no		3	0	244.91
FQIIN(deFSSSEIK	1411.7347	IPI00856723	IPI00856723	yes	no		2	0.0007018	136.3
FPVPFQKENV(deTATIVEIGR	2144.163	IPI00134691	IPI00134691	yes	yes		3	2.2666E-08	129.88
FNSTE(deYQVVTR	1342.6517	IPI00119063	IPI00119063	yes	no	2,3		7.9994E-17	177.81
FN(deVSIIYGR	1067.5764	IPI00320675	IPI00320675	yes	yes		2	0.012994	94.297
FN(deTTSINVGSSYFPEHGYFR	2322.0705	IPI00263041	IPI00263041	yes	no		3	3.6248E-05	98.227
FN(deITETPEADIHQGFGNIIQSISQPEDQDQINIGNAMFIEK	4601.202	IPI00131830	IPI00131830	yes	no	4,5,6		4.8552E-08	63.625
FN(deETRDTITSIVSDINVR	2079.0596	IPI00990932	IPI00990932	yes	yes		3	1.0761E-06	131.4
FMISGYN(deFSVMENMPAISPVGMVTVIDGDKGENAR	3775.7718	IPI00719927	IPI00719927	yes	no		4	0.0005572	51.871
FMGIPTKDDNIEHYKN(deSTVMAR	2566.2308	IPI00123925	IPI00123925	yes	no		3	0.0013455	84.493
FMFFVQFPHN(deATQTMNIR	2228.0659	IPI00136513	IPI00136513	yes	yes		3	0.0020616	76.151
FITDVERN(deETAIYHVEAFK	2281.1379	IPI00466733	IPI00466733	yes	no	2,3		3.0276E-41	195.23
FIRPFMQYN(deSTR	1558.7715	IPI00136280	IPI00136280	yes	yes		3	0.0034008	116.55
FIQSAEFFN(deYTVR	1620.7936	IPI00127406	IPI00127406	yes	yes	2,3		5.8655E-16	174.39
FINYN(deQTVSR	1240.62	IPI00226932	IPI00226932	yes	no		2	0.0004849	122.69
FINHTVGN(deKTK	1257.683	IPI00111228	IPI00111228	yes	yes		2	0.012404	89.805
FINHTVGN(deK	1028.5403	IPI00111228	IPI00111228	yes	yes		2	0.026397	78.934
FINFFVGNTIN(deSSYPPGYSIHSISVR	2915.4606	IPI00130420	IPI00130420	yes	no		3	5.837E-17	128.38
FIN(deISSTGIR	1106.6084	IPI00131898	IPI00131898	yes	yes		2	0.0069252	93.058
FIN(deHTVGNKTK	1257.683	IPI00111228	IPI00111228	yes	yes		2	0.012404	89.805
FIN(deHTVGNK	1028.5403	IPI00111228	IPI00111228	yes	yes		2	0.026397	78.934
FIIKGPN(deKIQCVDGNWTTIPVCIEEER	3215.6107	IPI00130010	IPI00130010	yes	no		3	0.0068213	64.589
FIFKN(deQTIEIHVIYGPR	2074.1364	IPI00122973	IPI00122973	yes	no	2,3,4		1.528E-47	203.9
FIEYVTSECHFYN(deGTQHVR	2386.0801	IPI00474580	IPI00474580	yes	no	2,3,4		3.3585E-06	115.8
FIEPYN(deDSIQAQK	1551.7569	IPI00123704	IPI00123704	yes	yes		2	0.0025167	121.04
FIASFNVVN(deTTKR	1495.8147	IPI00622572	IPI00622572	yes	no	2,3		0.0026803	136.93
FI(deEAGIYEVPIIITDSGNPPKSNISIIR	3055.6594	IPI00323134	IPI00323134	yes	no	3,4		1.2868E-42	167.59
FHVN(deYTQPIVAVK	1514.8245	IPI00123704	IPI00123704	yes	no	2,3		0.0025808	118.24
FHVHQPVTQPFIQVTN(deTTVK	2320.2328	IPI00658311	IPI00658311	yes	no	2,3,4		1.5484E-85	199.01
FGYIIHTDN(deR	1234.6095	IPI00475209	IPI00475209	yes	no		2	0.000687	119.53
FGTCSQICN(deNTK	1428.6126	IPI00119063	IPI00119063	yes	yes	2,3		1.6069E-23	186.16
FGN(deETFIIHIDNGR	1631.8056	IPI00170355	IPI00170355	yes	yes	2,3		9.7405E-07	153.39
FGHSPIIN(deITGGISHASDVVIYHQHK	2826.4565	IPI00119063	IPI00119063	yes	yes	3,4,5		3.7752E-20	131.56
FGEFGN(deYSIIVQHASSGANK	2125.0229	IPI00317488	IPI00317488	yes	no	2,3		3.3571E-66	218.25
FFPYAN(deGTISIR	1384.7139	IPI00115762	IPI00115762	yes	no		2	0.0035691	84.169
FFN(deDSIVDPVDSEWFGFYR	2339.0535	IPI00331318	IPI00331318	yes	no	2,3		1.8978E-25	178.72
FDEVYDAIAGAHPN(deITVYK	2122.0371	IPI00111163	IPI00111163	yes	no		2	4.3244E-15	154.19
FDAAGTSVHGGVPQN(deGSICAHIPMIK	2663.2948	IPI00315535	IPI00315535	yes	yes	3,4		3.7546E-20	131.33
FCNIVPTEHCFIGN(deGTEYR	2313.0307	IPI00227857	IPI00227857	yes	yes		3	2.2706E-10	135.09
FAVESIVPSSISIMHSPPDAQN(deMSEVSISPMEISTFR	4019.9319	IPI00114044	IPI00114044	yes	yes	3,4,5		4.3897E-15	98.76
FAQPQWHIIHINGTFSN(deETEPDTEPCVDGWVYDR	4057.833	IPI00226346	IPI00226346	yes	no		4	7.5106E-05	60.029
FAQPQWHIIHIN(deGTFSNETEPDTEPCVDGWVYDR	4057.833	IPI00226346	IPI00226346	yes	no		4	7.5106E-05	60.029
FAPPEAPEPWSG(deVRDGTSQPAICPQNVTMNMEGIK	3810.7804	IPI00320204	IPI00320204	yes	yes		4	0.0003845	54.732
FANEYPN(deITR	1223.5935	IPI00130573	IPI00130573	yes	yes		2	0.0006997	119.39
FAIIKDYR(dePSAGNNSIYQDTVVFK	2745.4126	IPI00322463	IPI00322463	yes	no		3	8.6085E-05	83.063
FAEIN(deGSAICSYNIKPSEYTITSK	2692.3054	IPI00421237	IPI00421237	yes	no		3	9.6131E-06	89.483
F(deDEFFSQGCAPGYEKNSTICDICIGPIK	3252.4566	IPI00987951	IPI00987951	yes	no	3,4		3.1145E-48	175.28
F(deAPPQPAEPWSSVKNATTYPPMCSQDAAR	3203.4805	IPI00128399	IPI00128399	yes	yes		3	7.9084E-10	92.886
F(deAPPQPAEPWSFVKNATSYPPMCSQDAGWAK	3464.5958	IPI00138342	IPI00138342	yes	yes		4	0.0072712	50.857
EYEGAVYPDN(deTTDFQR	1903.8224	IPI00605187	IPI00605187	yes	no	2,3		1.2439E-27	190.19
EWN(deGTYHCIFR	1481.651	IPI00876541	IPI00876541	yes	no	2,3		0.0032615	93.839
EWIPIN(deHSVNSVVVR	1747.937	IPI00224752	IPI00224752	yes	yes	2,3		3.4783E-05	140.27

EVTVIGVATAPTQVISNGIPVSN(deFTYSPDNK	3217.6507	IPI00111960	IPI00111960	yes	yes	3,4,5	0.0005205	58.812
EVNVYMYIANGSAFN(deISNK	2133.0201	IPI00222967	IPI00222967	yes	no	2,3	7.5049E-32	182.49
EVNVYMYIAN(deGSAFNISNK	2133.0201	IPI00222967	IPI00222967	yes	no	2,3	7.5049E-32	182.49
EVNSQIDNNGCSTQEVN(deITEIQSKK	2834.3352	IPI00123223	IPI00123223	yes	yes	3	0.037878	62.099
EVNSQIDNNGCSTQEVN(deITEIQSK	2706.2403	IPI00123223	IPI00123223	yes	yes	2,3,4	7.5524E-56	195.15
EVN(deKTCEAIIFK	1450.749	IPI00115116	IPI00115116	yes	yes	2,3	0.0003315	145.17
EVMN(deFTAENIYK	1457.6861	IPI00170006	IPI00170006	yes	no	2	0.0025862	91.62
EVHFFVN(deASDVDSVK	1691.8155	IPI00310049	IPI00310049	yes	yes	2,3	6.1402E-05	138.91
ETYIETN(de)YTIK	1373.6715	IPI00120155	IPI00120155	yes	yes	2	0.0052592	86.344
ETYFN(deISKK	1128.5815	IPI00153258	IPI00153258	yes	no	2,3	1.295E-08	147.95
ETVITFIDPEGSQVDTIEGNN(deITGIASFPDFEIPSNPK	4090.9899	IPI00405543	IPI00405543	yes	yes	4	4.4703E-40	128.22
ETIIEHDQRQEEYEIISFADTDISSIN(deQTR	3579.6965	IPI00136513	IPI00136513	yes	yes	3	1.3981E-33	131.04
ETFIIN(deTTCVK	1324.6697	IPI00138199	IPI00138199	yes	yes	2	0.0003352	129.34
ESYIISN(deSTMK	1384.6908	IPI00123925	IPI00123925	yes	no	2	0.002146	102.97
ESSEHPWK(deWTDNTEYNNTIPIRGEER	3187.4595	IPI00276430	IPI00276430	yes	yes	3,4	2.2656E-10	105.46
ESQTIGDQCVYN(deSTHIGFQR	2339.0601	IPI00118130	IPI00118130	yes	yes	2,3	5.48E-156	268.25
ESQIN(deITVMAKPTNWIEGTR	2287.1631	IPI00311405	IPI00311405	yes	yes	2,3	2.6818E-05	103.4
ESQIN(deITVMAK	1232.6435	IPI00311405	IPI00311405	yes	yes	2	0.001157	106.49
ESNPITAQQTT(deKIDAPTNIQFVNETDR	3030.4894	IPI00113539	IPI00113539	yes	no	3	5.1228E-06	86.08
ESIIPVTIQCN(deITSSSHTIMYSYWTR	3086.4841	IPI00757771	IPI00757771	yes	no	3	0.0016789	63.165
ESGIQN(deVSTCR	1249.5721	IPI01008227	IPI01008227	yes	no	2	0.0010656	107.97
ESGIN(deMTIPEENQPVVFNHIYNIK	2785.3745	IPI00403938	IPI00403938	yes	no	3	4.8026E-33	163.79
ERVEN(deTSQPGEMQVTIQNIMPATVYIFK	3222.6053	IPI00129159	IPI00129159	yes	no	3	2.3178E-37	151.18
ERTEVIIQGTVS(dePNASAPDAVWEDYEFK	3150.5146	IPI00405437	IPI00405437	yes	no	3	1.7629E-13	106.11
ERSN(deNTPDANVAPEVTVISR	2168.0822	IPI00110808	IPI00110808	yes	no	3	0.0009494	89.374
EREDVQAIN(deISVPYGPIPEDFQR	2671.3242	IPI01023131	IPI01023131	yes	no	3	4.0418E-09	111.45
EQYIHEN(deYSR	1337.6	IPI00127406	IPI00127406	yes	no	2,3	0.0025621	104.24
EQSFQGSN(deMTIFYR	1706.7723	IPI00453571	IPI00453571	yes	no	2	3.2884E-41	149.22
EQN(deYTCHVYHEGIPEPITIR	2455.159	IPI00608044	IPI00608044	yes	no	2,3,4	7.3712E-80	221.47
EQN(deYTCHVNHEGIPEPITIR	2406.1386	IPI00312980	IPI00312980	yes	no	3	0.0046615	69.422
EQN(de)YTCR	969.39743	IPI00109996	IPI00109996	yes	no	2	0.0075334	107.57
EQN(de)YTCHVHHK	1451.6364	IPI00850057	IPI00850057	yes	no	2,3	4.1449E-07	153.95
EQIQGIN(deASSAAWAR	1600.7958	IPI00116913	IPI00116913	yes	no	2	0.000385	112.3
EQIN(deISIDHR	1223.6258	IPI00127407	IPI00127407	yes	no	2,3	0.0059755	95.094
EQETCIAPEIEHGNY(deSTTQR	2362.0496	IPI00122117	IPI00122117	yes	yes	3	6.3342E-13	144.17
EQESMMN(deITIHIVK	1671.8324	IPI00990932	IPI00990932	yes	no	3	0.0015725	89.959
EQEN(deSTIYISFTPK	1655.8043	IPI00894972	IPI00894972	yes	no	2	0.0023331	90.561
ENS(deSYQVINWR	1394.6579	IPI00153316	IPI00153316	yes	yes	2	2.2523E-24	188.91
ENQN(deHSYSIK	1218.5629	IPI00120245	IPI00120245	yes	no	2	0.015982	78.342
ENPGHIYNKN(deISQK	1640.8271	IPI00122175	IPI00122175	yes	no	2	0.0039985	110.38
ENIIDISN(deASK	1202.6143	IPI00857855	IPI00857855	yes	no	2	0.0026961	96.464
ENGVD(deAVHPGYGFISER	1845.8646	IPI00943457	IPI00943457	yes	no	2,3	0.0017706	87.287
EN(deMTFGSTIVTNPK	1537.7446	IPI00466371	IPI01026704	no	no	2	1.6173E-10	160.8
EN(deISDPTSPIRR	1383.7106	IPI00136925	IPI00136925	yes	no	2	0.0064102	86.772
EN(deISDPTSPIR	1227.6095	IPI00136925	IPI00136925	yes	no	2	0.0052755	86.288
EN(deGTFSKYEGGVETFAHIIVIR	2466.2543	IPI00118130	IPI00118130	yes	yes	3,4	8.3002E-86	224.86
EN(de)NSIHWK	1026.4883	IPI00123223	IPI00123223	yes	no	2	0.040779	74.92
EMN(deCSETISQIQK	1566.7018	IPI00459134	IPI00459134	yes	no	2,3	0.0002827	143.93
EMIHN(deATFCIVPR	1586.7698	IPI00125530	IPI00125530	yes	no	2	0.0033893	83.087
EMGNTVMDIIRNYTAN(deATSSR	2343.0947	IPI00108098	IPI00108098	yes	no	3	2.2524E-05	96.391
EMGNTVMDIIRN(deYTANATSSR	2343.0947	IPI00108098	IPI00108098	yes	no	3	2.2524E-05	96.391
EKVQSIQTIA(deANNSAIAK	1885.0269	IPI00129304	IPI00129304	yes	yes	3	3.4288E-20	170.78
EKVEN(deGSETGPIPPEIQPIIEGEVK	2688.3858	IPI00136012	IPI00136012	yes	yes	2,3,4	1.7541E-15	121.38
EKSCVAITDAFP(deQNMSR	1952.9084	IPI00405742	IPI00405742	yes	no	2,3	8.2829E-06	128.71
EKEDRPFHQAV(deVNDTQAFWHNK	2695.2891	IPI00406901	IPI00406901	yes	no	3,4	0.0036028	80.752
EIYESIWQN(deFTDSK	1758.8101	IPI00272690	IPI00272690	yes	yes	2	0.0013788	118.2
EIVSDPIYVEVQGIPYFIKQPESVN(deVTR	3218.6863	IPI00121254	IPI00121254	yes	yes	3,4	1.8178E-09	91.729
EIVHQSN(deTSNIFFSPVSIATAFAMISIGSK	3195.6274	IPI00406302	IPI00406302	no	no	3,4	4.1469E-276	218.93
EITGIRN(deISSQEGSTDVVAR	2131.0869	IPI00677395	IPI00677395	yes	no	2,3	7.6945E-80	226.57
EIQIAGFDPSQDSIGPKNN(deDSTINNSATTIIIGIQDCR	4101.9913	IPI00881745	IPI00881745	yes	no	4	0.0116	44.167
EIPVIN(deVSIGPFSTSQVVAR	2112.1579	IPI00515432	IPI00515432	yes	no	2,3	1.6935E-10	131.65
EIN(deISICPVSQTSER	1731.8461	IPI00381303	IPI00381303	yes	yes	2,3	1.0026E-24	183.36
EIKGDGPFTVFVPHADI(deISNMSQDEIAR	3085.5179	IPI00987265	IPI00987265	yes	no	3	3.5116E-37	150.94
EIIYSN(deGSIIFQMITMK	1987.0159	IPI00108535	IPI00108535	no	no	2,3	6.9607E-173	269.49
EIIVHPN(deYTR	1240.6564	IPI00113750	IPI00113750	yes	yes	2,3	1.3797E-18	175.15
EIIVDEIISN(deSSTIASYK	1981.0256	IPI00173181	IPI00173181	yes	no	2,3	4.1602E-28	181.34
EIISN(deASDAIDKIR	1543.8206	IPI00554929	IPI00129526	no	no	2	0.013148	105.99
EIIMDFAN(deSSAEITACMVR	2156.9904	IPI00221706	IPI00221706	yes	yes	3	3.0686E-09	130.4
EIHHIQEQN(deVSNVFIDKEEFFIGSK	2986.4825	IPI00605187	IPI00605187	yes	no	3,4	6.8947E-20	138.82
EIGPIYTN(deITADIGTDPR	1944.9793	IPI00314900	IPI00314900	yes	yes	2,3	0.0031003	76.378
EIGGAIDSGAAYVIEQASSHIGN(deSTQAAVR	2971.4635	IPI00458393	IPI00458393	yes	no	3	0.0008265	57.735
EIGAIIYN(deCSNIAQDIEK	2050.0041	IPI00221418	IPI00221418	yes	no	2,3	5.5237E-129	252.83
EIEYYVGQSFANAMYN(deACR	2284.9881	IPI00132600	IPI00132600	yes	yes	3	6.7886E-06	110.84
EIAAAAN(deESAVK	1172.6037	IPI00113726	IPI00113726	yes	no	2	0.0019295	106.17
EHSIFIDIHPVTGIPMN(deCSVK	2393.1872	IPI01008227	IPI01008227	yes	no	2,3	1.6757E-15	139.88
EGTYSIPTSN(deFTVPDIEIPSWISTGNYR	3143.5088	IPI00119095	IPI00119095	yes	yes	2,4	3.5167E-26	133.58
EGSRTDDEVVQREEEAIQI(deDGINASQIR	3156.5283	IPI00129526	IPI00129526	yes	yes	3,4	1.1324E-42	169.41
EGPRNISTCFSSGDIFAAHN(deISER	2664.2351	IPI00123428	IPI00123428	yes	no	3	4.2599E-46	185.71
EGPRN(deISTCFSSGDIFAAHNISER	2664.2351	IPI00123428	IPI00123428	yes	no	3	4.2599E-46	185.71
EGN(deFTSTFDKK	1272.5986	IPI00153258	IPI00153258	yes	yes	2,3	3.1942E-07	158.01
EGN(deCSAQSGIAWQDCDFKDAEEAATGECTATVGKR	3818.6207	IPI00114958	IPI00114958	yes	no	4	2.795E-09	83.834
EGN(deCSAQSGIAWQDCDFKDAEEAATGECTATVGK	3662.5196	IPI00114958	IPI00114958	yes	no	4	4.3005E-06	65.244
EGN(deCSAQSGIAWQDCDFK	2071.8364	IPI00114958	IPI00114958	yes	no	2,3	4.9004E-05	112.82
EGKFDEVYDAIAGA(deHPNITVYKK	2564.2911	IPI00111163	IPI00111163	yes	no	3,4	1.552E-14	134.29
EGKFDEVYDAIAGA(deHPNITVYK	2436.1961	IPI00111163	IPI00111163	yes	no	3,4	1.371E-58	203.89
EGFSAN(deYSVIQSSISEDFK	2106.9746	IPI00123996	IPI00123996	yes	yes	2,3	3.6719E-19	165.19

EGDN(deITIQCIGNGNPPPEEFMFYIPGQPEGIR	3588.6654	IPI00121378	IPI00121378	yes	no		4	0.0025185	52.721
EGASEEETN(deISK	1292.5732	IPI00138342	IPI00138342	yes	yes		2	0.0001173	145.29
EFVENSECIQCHPECIPQAMN(deITCTGR	3279.3876	IPI00121190	IPI00121190	yes	no	3,4		1.5201E-48	175.86
EFQHN(deSTMYYK	1446.6238	IPI00788443	IPI00788443	yes	no	2,3		0.0022739	98.105
EEQFN(deSTFR	1156.5149	IPI00475246	IPI00475246	yes	no	1,2		5.4597E-14	159.83
EEN(de)ATIATYPEFGVIDIK	2008.9993	IPI00124221	IPI00124221	yes	no	2,3		1.6157E-109	243.95
EEIGTYPYYTPTGEPVFGGIPQN(deASIVTHIAHTFQDIK	4190.0637	IPI00453571	IPI00453571	yes	no		4	3.1487E-14	82.726
EEAMIYHN(deQTSEIR	1719.7886	IPI00756279	IPI00756279	yes	no	2,3		0.0013156	124.09
EDTVTITCEGTHNPGN(deSSTQWFHNGR	2944.2794	IPI00129485	IPI00129485	yes	no		3	4.8151E-80	211.43
EDSCQINY(deSEGPCIGMQER	2271.9195	IPI00127352	IPI00127352	yes	yes	2,3		1.3685E-41	193.85
EDRPFHQAVVN(deDTQAFWHNK	2438.1516	IPI00406901	IPI00406901	yes	no		3	1.7703E-41	194.05
EDIIA(deIRQNFSNITVSTEDQVK	2519.2867	IPI00129677	IPI00129677	yes	no		3	1.6472E-25	168.79
ED(deIIAIRQNFSNITVSTEDQVK	2519.2867	IPI00129677	IPI00129677	yes	no		3	1.6472E-25	168.79
ECIGN(deCSEPDDPTK	1620.6396	IPI00128358	IPI00128358	yes	no		2	0.0015331	109.48
ECHTIQN(deYTIWR	1619.7515	IPI00125220	IPI00125220	yes	no	2,3		2.0293E-104	247.88
EAYKN(deISIIYSQMQK	1814.9237	IPI00221706	IPI00221706	yes	no	2,3		3.1085E-11	162.48
EATIVGEN(deETYPR	1477.7049	IPI00131143	IPI00131143	yes	no		2	5.1192E-16	175.15
EASNHSSGAGIVQIN(deKSNDKETVVGR	2696.3478	IPI00108844	IPI00108844	yes	no		3	3.5425E-07	94.463
EASNHSSGAGIVQIN(deK	1610.8012	IPI00108844	IPI00108844	yes	no	2,3		3.9166E-25	182.15
EASN(deHSSGAGIVQINKSNDKETVVGR	2696.3478	IPI00108844	IPI00108844	yes	no		3	3.5425E-07	94.463
EASN(deHSSGAGIVQINK	1610.8012	IPI00108844	IPI00108844	yes	no	2,3		3.9166E-25	182.15
EASHYSIHDIVISYN(deTSDSTVFPGAVAK	3007.4563	IPI00222967	IPI00222967	yes	no		3	1.0352E-103	222.48
EAQYN(deSTFR	1114.5043	IPI00807983	IPI00807983	yes	no		2	1.8341E-06	140.49
EAPFGTNCN(deITITSR	1679.7937	IPI00129253	IPI00129253	yes	no		2	0.0009597	101.42
EANIT(deEDQIIFFPK	1663.8457	IPI00122122	IPI00122122	yes	no	2,3		1.7264E-16	176.25
EAINQAVN(deTTR	1215.6208	IPI00116913	IPI00116913	yes	no		2	0.000244	134.56
EAIGNASQI(deFDSWIK	1677.8362	IPI00134585	IPI00134585	yes	yes	2,3		1.1878E-34	198.95
EAHNVSAPFNF(deSIPPGFGHR	2180.0552	IPI00115482	IPI00115482	yes	no		3	0.0016508	79.837
EAHNV(deSAPFNFSIPPGFGHR	2180.0552	IPI00115482	IPI00115482	yes	no		3	0.0016508	79.837
EAFN(deETNQAIQTISR	1720.838	IPI00112614	IPI00112614	yes	yes	2,3		5.2857E-125	252.82
EADN(deHTAFIR	1172.5574	IPI00118333	IPI00118333	yes	yes		2	0.0027502	103.08
EACIQHYYVN(deASYIVWK	2143.0197	IPI00120155	IPI00120155	yes	yes		2	5.3429E-07	136.35
DYYPIN(deESIASIPIR	1749.8938	IPI00121627	IPI00121627	yes	yes	2,3		3.1626E-57	211.57
DYYIN(deKTENEK	1415.6569	IPI00396840	IPI00396840	yes	no	2,3		1.8784E-11	169.65
DYRPSAGN(deNSIYQDTVVFK	2173.044	IPI00322463	IPI00322463	yes	no	2,3		5.7926E-42	199.15
DWSQNMYFN(deCSEDNPSR	2148.8266	IPI00153810	IPI00153810	yes	no		2	6.9531E-37	191.21
DWIWNQMHIDEEKN(deESIPHYVGK	2867.3337	IPI00138180	IPI00138180	yes	yes		3	0.0002893	85.087
DWCN(deWTIISR	1349.6187	IPI00124933	IPI00124933	yes	no		2	0.00078	118.48
DVIWFRPTIINDTGN(deYTCMIR	2584.2566	IPI00929886	IPI00929886	yes	no		3	0.0001785	92.39
DVIWFRPTIIN(deDTGNYTCMIR	2584.2566	IPI00929886	IPI00929886	yes	no		3	0.0001785	92.39
DVGSGTTN(deNSQACAQFIEQYFHNSDITEFMR	3566.5467	IPI00130661	IPI00130661	yes	yes	3,4		2.1336E-87	205.13
DVFIPKPSWGN(deHTPIFR	2010.0476	IPI00117312	IPI00117312	yes	yes		3	0.0039561	90.367
DTYVN(de)ASQSIYGSSPR	1743.8064	IPI00122977	IPI00122977	yes	no	2,3		4.1261E-45	202.18
DTTGSHTFQGMFGCEITNN(deR	2271.9637	IPI00137987	IPI00137987	yes	yes		3	1.8104E-25	176.94
DTISIN(deATNIK	1188.635	IPI00121190	IPI00121190	yes	no		2	3.1922E-07	155.48
DTCAQECSHFNI(deTK	1709.7138	IPI00132474	IPI00132474	yes	no	2,3		1.3619E-56	218.21
DTAVEGEEIEVN(deCTAMASKPATTIR	2692.2684	IPI00856723	IPI00856723	yes	no	2,3		9.1555E-25	147.9
DSYPDGN(deITWYRNGK	1784.8118	IPI00121378	IPI00121378	yes	no	2,3		2.6731E-11	164.15
DSYPDGN(deITWYR	1485.6525	IPI00121378	IPI00121378	yes	no		2	0.0011379	124.12
DSVIN(deISESVEDGPR	1615.7689	IPI00114044	IPI00114044	yes	yes		3	4.2878E-05	139.86
DSGIWINGFDYTGMSHVTPHIPEIN(deDTIR	3284.5561	IPI00407222	IPI00407222	yes	yes	3,4		1.3836E-13	102.21
DSCESNQN(deITCR	1482.5827	IPI00466371	IPI01026704	no	no		2	3.6295E-24	189.3
DQGNV(deTDMASMK	1295.5486	IPI00471081	IPI00471081	yes	yes		2	0.0007711	126.09
DQCIVDDITYNVN(deDTFHKR	2352.0805	IPI00113539	IPI00113539	yes	no	2,3,4		0.0001412	97.904
DQCIVDDITYNVN(deDTFHK	2195.9793	IPI00113539	IPI00113539	yes	no	2,3		4.2894E-21	177.04
DNTTC(deYEFKK	1304.5707	IPI00119063	IPI00119063	yes	yes		2	0.0042924	106.62
DNTTC(deYEFK	1176.4757	IPI00119063	IPI00119063	yes	yes		2	0.018641	85.958
DNSCVDPPHVPN(deATIVTR	1990.9531	IPI00130010	IPI00130010	yes	no	2,3		2.4017E-07	135.18
DNATQEEIIHYIE(deK	1701.821	IPI00321190	IPI00321190	yes	no	2,3		1.8016E-08	160.48
DMSDEIGCVN(deVTQCDGPNKFK	2413.0348	IPI00312063	IPI00312063	yes	no		3	0.0005824	111.33
DKNGTRAEPPIN(deASAGDQEEK	2226.0513	IPI00123342	IPI00123342	yes	yes	2,3		4.3154E-08	112.55
DKN(deGTRAEPPINASAGDQEEK	2226.0513	IPI00123342	IPI00123342	yes	yes	2,3		4.3154E-08	112.55
DKISPIHIA(deINFSIDPK	1907.0516	IPI00115976	IPI00115976	yes	yes		3	0.0093404	76.391
DIYEYSWVQGPWNSN(deRTER	2399.0931	IPI00118674	IPI00118674	yes	yes	2,3		0.0001049	100.45
DIYEYSWVQGPWNSN(deR	2012.9017	IPI00118674	IPI00118674	yes	yes		2	0.0001662	121.72
DIVV(deEIYSQWMKNPNNNTIHPNIR	2894.4497	IPI00319509	IPI00319509	yes	no	3,4		5.3856E-05	88.755
DIVQNVFMSN(deMSMDIQSHPSSCPK	2751.2125	IPI00121190	IPI00121190	yes	no	3,4		3.7008E-16	124.2
DIVGN(deITEIESEDIQIEAIIMR	2500.2731	IPI00122399	IPI00122399	yes	no	2,3		0	262.12
DISVFAPN(deMTEIIKDVTQYR	2339.1831	IPI00855103	IPI00855103	yes	no	2,3		6.6104E-05	99.747
DISVFAPN(deMTEIIK	1576.8171	IPI00855103	IPI00855103	yes	no		2	0.0021943	96.591
DISHSIYMFFNTS(deDIR	1944.904	IPI00114457	IPI00114457	yes	yes		3	0.0007328	101.62
DIQNFIEN(deVTSSVDVK	1806.9	IPI00990932	IPI00990932	yes	no	2,3		1.8749E-06	148.36
DIQDIHKDTEN(deR	1482.7063	IPI00129304	IPI00129304	yes	yes		2	0.0067507	98.407
DINSQIEIQNVTN(deDTHITIIGIKPDTTYDIK	3511.8046	IPI00110264	IPI00110264	yes	yes	3,4		1.0456E-22	112.26
DINSQIEIQN(deVTNDTHITIIGIKPDTTYDIK	3511.8046	IPI00110264	IPI00110264	yes	yes	3,4		1.0456E-22	112.26
DIN(deSTAQNVMFYDMGSGSTVCTIVTYQTVK	3329.5254	IPI00123342	IPI00123342	yes	no	3,4		5.5776E-06	78.128
DIN(deHTAQGIIEKDSWK	1853.9272	IPI00120953	IPI00120953	yes	no		2	0.0013996	106.38
DIIFSDDTECISNIQN(deK	2010.9204	IPI00471080	IPI00471080	yes	no		2	2.9877E-198	287.01
DIGTASHN(deFSGAIPR	1541.7587	IPI00229820	IPI00229820	yes	yes	2,3		7.5387E-05	138.19
DIGPAIAN(deSSHDVK	1422.7103	IPI00108811	IPI00108811	yes	yes	2,3		3.3207E-33	198.16
DIGMFAPN(deMTR	1251.574	IPI00990932	IPI00990932	yes	no		2	0.0077619	75.695
DIFTNQSDFADTTKDTPITITVIH(deK	2820.4182	IPI00116105	IPI00116105	yes	yes	3,4		1.4483E-38	172.3
DIFTN(de)QSDFADTTK	1601.7209	IPI00116105	IPI00116105	yes	no		2	4.6231E-24	188.39
DIEHHITNVNVSFYDDIVN(deGTVIK	2741.3661	IPI00467600	IPI00467600	yes	yes		3	2.9756E-46	181.54
DIEHHITNVN(deVSFYDDIVNGTVIK	2741.3661	IPI00467600	IPI00467600	yes	yes		3	2.9756E-46	181.54
DIDTDFTSN(deASQPETK	1767.7799	IPI00130573	IPI00130573	yes	yes		2	3.0912E-45	204.13

DIDMFIN(deASK	1152.5485	IPI00224752	IPI00224752	yes	yes		2	1.2012E-05	132.32
DIAVGDDYTN(deR	1237.5575	IPI00468674	IPI00468674	yes	no		2	0.0011462	107.03
DIAVFCHFTNAAN(deSSVR	1907.8948	IPI00876541	IPI00876541	yes	no	2,3		1.7966E-07	139.6
DHVNVSMIEATDFPFN(deTTEWEGYIPK	3039.396	IPI00136942	IPI00136942	yes	yes		3	3.9012E-29	155.25
DHVN(deVSMIEATDFPFNTTEWEGYIPK	3039.396	IPI00136942	IPI00136942	yes	yes		3	3.9012E-29	155.25
DGYAVANTN(deTSDVWK	1639.7478	IPI00129253	IPI00129253	yes	no		2	0.0002729	128.96
DGTSQPAICPQN(deVTMNMEGIK	2290.0392	IPI00320204	IPI00320204	yes	yes	2,3		4.863E-18	142.43
DGSDSAAMVYN(deSSQEWGIR	2071.8905	IPI00119522	IPI00119522	yes	yes	2,3		6.355E-230	296.73
DGSCIGN(deSSR	1051.4353	IPI00119063	IPI00119063	yes	yes		2	0.0024682	104.82
DGQQN(deISIIYTEPGASQTQTGASFR	2668.2729	IPI00284816	IPI00284816	yes	no		3	3.5514E-14	116.35
DGIAIN(deITIR	1084.6241	IPI00119065	IPI00119065	yes	yes		2	9.6144E-06	134.3
DGASEEETN(deISK	1278.5576	IPI00387289	IPI00387289	yes	yes		2	0.0015123	110.92
DFIPSTISFTWNYQN(deNTEVIQGIR	2842.3926	IPI00468055	IPI00468055	yes	no		3	2.1318E-09	117.37
DEIGNVSTSHIIIIDDSV(deEMEIRPR	2837.4229	IPI00309035	IPI00309035	yes	yes		3	3.4398E-07	95.5
DDYRPTWTIN(deQTEPVAGNYYPVNTR	2969.3944	IPI00381303	IPI00381303	yes	yes	2,3,4		3.7946E-33	167.34
DDIHPTIPAGQYFINI(deTYNYPVHSFDGRK	3377.6469	IPI00387318	IPI00387318	yes	no		4	0.041943	58.904
DDIHPTIPAGQYFINI(deTYNYPVHSFDGR	3249.552	IPI00387318	IPI00387318	yes	no	3,4		5.412E-66	198.49
DDAMVYN(de)VTSR	1269.566	IPI00406901	IPI00406901	yes	no		2	0.023236	67.519
DCVSCQN(deVSR	1223.5023	IPI00121190	IPI00121190	yes	no		2	0.0038027	99.752
DCDFIEDGEERN(deCTGK	1943.7626	IPI00127280	IPI00127280	yes	yes		3	0.011863	75.462
DAVRN(deITEVVPQIIDQIR	2078.1484	IPI00223446	IPI00223446	yes	yes		3	0.0001376	112.67
DATIQAYISSGN(deFSK	1600.7733	IPI00469218	IPI00469218	yes	yes	2,3		7.9179E-12	165.02
DAMVGN(deYTCEVTEISR	1843.808	IPI00124830	IPI00124830	yes	no	2,3		9.9354E-35	197.15
DAHFN(deSTITEFGETINNFVER	2440.1295	IPI00346062	IPI00346062	yes	yes		3	0.001381	77.899
DAGVGFPEYQEEDN(deSTYNFR	2336.9822	IPI00308971	IPI00308971	yes	yes	2,3		3.4625E-13	147.17
DAGN(deYTVIITNPISMEK	1864.9241	IPI00124326	IPI00124326	yes	no	2,3		1.7618E-10	151.95
DAAQCSGGSVAHIAEIGIPTN(deITHIIIFR	3060.5815	IPI00331407	IPI00331407	yes	no		3	8.7511E-05	72.676
CYVIPIN(deTSIVMPPK	1730.9099	IPI00130758	IPI00130758	yes	yes		3	0.0027948	82.069
CVVHYEN(de)STVPEKK	1688.8192	IPI00409148	IPI00409148	yes	yes	2,3,4		2.8226E-86	235.73
CVVHYEN(de)STVPEK	1560.7242	IPI00409148	IPI00409148	yes	yes	2,3		0.0015101	136.3
CVAN(deYTGNGR	1110.4876	IPI00111793	IPI00111793	yes	yes		2	0.0019402	108.7
CTPPPYVENAVMISEN(deR	1975.9132	IPI00138061	IPI00138061	yes	no		3	1.5399E-60	212.39
CSWIITN(deITYFPETVPR	2096.0401	IPI00758066	IPI00758066	yes	no	2,3		0.0022933	82.529
CSIIHSN(deISVK	1256.6547	IPI00133222	IPI00133222	yes	no	2,3		0.0003033	127.87
CRN(deYSIIIDQPK	1505.766	IPI00463774	IPI00463774	yes	no		3	0.0085043	88.187
CQSPYQMN(deGSDTVTCVNSR	2202.9092	IPI00130010	IPI00130010	no	no	2,3		1.1849E-79	223.29
CQSPYQMN(deGSDTVTCINGR	2186.9143	IPI00788443	IPI00788443	yes	no	2,3		3.718E-10	137.44
CQAYKN(deNSESFIHIYMYEVAR	2622.1995	IPI00130654	IPI00130654	yes	no		3	1.8639E-17	142.94
CQAIDFFPQN(deITMR	1739.8123	IPI00331440	IPI00331440	yes	no		3	0.0008508	134.68
CPMAGISHTTWHSN(deR	1753.7777	IPI00130764	IPI00130764	yes	no		2	0.002142	87.519
CPIITPFNDTNVVHTVNTAIAAFNTQNN(deGTYFK	3681.7886	IPI00128249	IPI00128249	yes	yes	3,4		2.5843E-40	141.48
CPIITPFN(deDTNVVHTVNTAIAAFNTQNNGTYFK	3681.7886	IPI00128249	IPI00128249	yes	yes	3,4		2.5843E-40	141.48
CNSVITYN(deITPVVQK	1734.8975	IPI00222967	IPI00222967	yes	no	2,3		1.3425E-24	181.51
CN(deTTQGNEVTSIIR	1591.7624	IPI00134743	IPI00134743	yes	no		2	0.0006866	136.39
CN(deFTIIESR	1138.5441	IPI00229935	IPI00229935	yes	no		2	0.015334	90.913
CN(deASSQFICSSGR	1472.6136	IPI00119063	IPI00119063	yes	yes		2	0.0026477	126.48
CIVPHTVN(deISSAWR	1638.83	IPI00129041	IPI00129041	yes	no	2,3		0.0012958	129.77
CIVPDTVN(deISSSWR	1632.793	IPI00131292	IPI00131292	yes	yes		2	0.0022608	96.591
CISPNVTSCACTIN(deFTIK	2084.9693	IPI00387318	IPI00387318	yes	yes	2,3		1.4769E-75	224.86
CISPN(deVTSCACTINFTIK	2084.9693	IPI00387318	IPI00387318	yes	yes	2,3		1.4769E-75	224.86
CISKN(deYTTDIITKIPGYPVK	2310.2294	IPI00124640	IPI00124640	yes	yes		3	0.003175	88.19
CISKN(deYTTDIITK	1555.7916	IPI00124640	IPI00124640	yes	yes		3	0.009486	95.428
CIQMSSFAN(deR	1212.538	IPI00320065	IPI00320065	yes	no		2	3.3746E-08	150.04
CIPHFAMIGNDTVMCTEQGN(deWTRIPECIEVK	3705.687	IPI00322463	IPI00322463	yes	no		4	3.4946E-23	116.35
CIPHFAMIGNDTVMCTEQGN(deWTR	2737.1869	IPI00322463	IPI00322463	yes	no		3	9.2848E-16	136.68
CIPHFAMIGN(deDTVMCTEQGNWTRIPECIEVK	3705.687	IPI00322463	IPI00322463	yes	no		4	3.4946E-23	116.35
CIPHFAMIGN(deDTVMCTEQGNWTR	2737.1869	IPI00322463	IPI00322463	yes	no		3	9.2848E-16	136.68
CHEGN(deGTFECGACR	1653.6082	IPI00132474	IPI00132474	yes	yes	2,3		0.0009478	134.04
CGWSPAEEIN(deYTVPGIGSAGEASFISQCMK	3245.4468	IPI00331032	IPI00331032	yes	yes		3	5.9813E-30	125.54
CGNCN(deITSIEDEDFCK	1960.7601	IPI00137599	IPI00137599	yes	yes	2,3		2.4647E-35	199.37
CFNAMEVDAIN(deSSHPVSTPVENPAQIR	2982.3964	IPI00318595	IPI00318595	yes	no	3,4		2.9971E-48	171.5
CFMAN(deGTWGYPFHDGDYIK	2277.9612	IPI00129010	IPI00129010	yes	no	2,3		4.006E-19	164.53
CF(deANKVNISFPSAQSIPASDTHIK	2631.3115	IPI00624663	IPI00624663	yes	yes	2,3,4		2.3268E-39	179.58
CEVICNQ(deSNKPITITVEQSR	2375.1573	IPI00626537	IPI00626537	yes	yes		3	4.7283E-08	125.73
CEQEASEDIKPAITGN(deK	1888.8837	IPI00134808	IPI00134808	yes	no	2,3		1.3031E-14	168.08
CDNN(deDTIIVR	1218.5663	IPI00467600	IPI00467600	yes	yes		2	1.5648E-07	147.26
CDISN(deSTEAGQK	1308.5616	IPI00153202	IPI00153202	yes	no		2	0.0036869	83.204
CDGDFDCEDRTDEAN(deCSVK	2291.8365	IPI00515360	IPI00515360	yes	no		3	7.7456E-10	133.71
CCGWVSHYN(deWTENEEIMGFTK	2647.093	IPI00108098	IPI00108098	yes	no		3	3.5657E-11	125.73
CAWSPEEEWN(deITTPEIHVPAER	2650.2122	IPI00123976	IPI00123976	yes	yes		3	1.9106E-11	126.4
CAMDIGVNYIGTVN(deVTHTGIQCQIWR	3005.431	IPI00114206	IPI00114206	yes	yes		3	1.0716E-16	126.02
CAISHAITIEFHQDGNGPENVGIYN(deISR	3111.4832	IPI00222429	IPI00222429	yes	yes	3,4		3.6663E-34	134.47
AYVPN(deATWYDYETGEEIGWR	2419.0757	IPI00848693	IPI00848693	yes	yes	2,3		4.486E-13	146.1
AYVFERN(deQSVGDPNVDIIR	2191.1022	IPI00127447	IPI00127447	yes	yes	2,3		7.7119E-32	182.35
AYVAFPDFFRN(deSTATWWKK	2334.1586	IPI00848693	IPI00848693	yes	yes		3	0.0041783	88.73
AYTIFVPTNHSIETQGN(deNSVIGIDTVR	2945.4883	IPI00987265	IPI00987265	yes	no	3,4		3.0427E-65	194.68
AYTIFVPTN(deHSIETQGNNSVIGIDTVR	2945.4883	IPI00987265	IPI00987265	yes	no	3,4		3.0427E-65	194.68
AWNPVSQN(deSSQPVR	1568.7696	IPI00129646	IPI00129646	yes	no		2	8.0611E-56	210.38
AWN(deYTVDTPHGMFVSGTPFEQSVWITDVGSGPYGHTVKK	4295.0422	IPI00228567	IPI00228567	yes	yes		5	0.0001191	55.314
AWN(deYTVDTPHGMFVSGTPFEQSVWITDVGSGPYGHTVK	4166.9473	IPI00228567	IPI00228567	yes	yes		4	5.9206E-40	127.2
AWGTPCEICPSVN(deTSEYK	2097.9136	IPI00828222	IPI00828222	yes	no		2	1.3107E-05	119.4
AWGISVINPN(deKTK	1426.7932	IPI00989096	IPI00989096	yes	no	2,3		8.7953E-10	162.48
AVYEAIRN(deCSIEDDSVR	1995.932	IPI00856861	IPI00856861	yes	no		2	2.1775E-05	123.08
AVVGSYTTTN(deVSIIAPR	1747.9468	IPI00330481	IPI00330481	yes	yes	2,3		4.3176E-21	179.14
AVTSISHDGSVICTFTAN(deSTYTK	2459.1639	IPI00453488	IPI00453488	yes	yes		3	1.1552E-09	113.51
AVN(deQTGAIYQCDYSTSR	1932.8636	IPI00120674	IPI00120674	yes	no	2,3		4.1625E-37	194.92

AVKIN(deETIGNQDKTAER	1885.9858	IPI00309999	IPI00309999	yes	yes	3	9.307E-20	176.4
AVIVNN(deITTGEK	1257.6929	IPI00128905	IPI00128905	yes	yes	2	0.0010964	120.84
AVISMIN(deETMNEAPEETK	2005.9336	IPI00875419	IPI00875419	yes	no	2,3	1.5451E-174	278.4
AVIGDHGDE(deIFSVFGSPFIKDGASEEETNISK	3394.6205	IPI00387289	IPI00387289	yes	yes	4	9.5388E-07	83.934
AVGAVDWDSHPYGYN(deITIQAK	2304.1175	IPI00623114	IPI00623114	yes	no	3	2.5641E-09	117.67
AVAYGEKN(deITFQGPIPK	1831.9832	IPI00111794	IPI00111794	yes	no	2,3,4	1.9382E-109	248.54
ATYIVIN(deNSKPEITSK	1776.9622	IPI00283900	IPI00283900	yes	yes	3	0.013005	73.91
ATVN(deDSGEYR	1110.4942	IPI00129485	IPI00129485	yes	no	2	1.5741E-18	174.42
ATSQFQN(deGSIITAFIPGISQCTVYSAK	2888.4378	IPI00340140	IPI00340140	yes	yes	3	1.4966E-13	103.53
ATPNEGFFNQN(deITTFYYDRIGIYPR	2996.4457	IPI00315535	IPI00315535	yes	yes	3	0.018024	58.366
ATPNEGFFNQN(deITTFYYDR	2297.0389	IPI00315535	IPI00315535	yes	yes	2,3	1.577E-12	143.04
ATHN(deDSGSYFCR	1413.5732	IPI00162870	IPI00162870	yes	yes	2	0.0047838	80.469
ATGTAHFFNFIIN(deTTDYR	2088.0065	IPI00132927	IPI00132927	yes	no	3	1.3107E-05	119.4
ATDADINDIIVYTIQEVTPN(deASK	2490.249	IPI00314132	IPI00314132	yes	no	3	0.0059633	61.222
ASVVN(deVTQHCTMESWESMNEVAR	2664.1731	IPI00121240	IPI00121240	yes	yes	2,3,4	9.4202E-19	143.04
ASSIMVN(deITEIFPKPK	1773.9699	IPI00406901	IPI00406901	yes	no	3	5.6526E-88	235.22
ASQQISN(deETSSFGFNIIRK	2126.0756	IPI00153258	IPI00153258	yes	no	3	0.014853	66.826
ASQQISN(deETSSFGFNIIR	1997.9807	IPI00153258	IPI00153258	yes	no	2,3	1.8463E-173	274.43
ASPGN(deGSGHSSAPSPDPTITR	1991.9297	IPI00310519	IPI00310519	yes	yes	3	0.010059	61.096
ASIPAPTIQSIN(deISWNR	1866.9952	IPI00228404	IPI00228404	yes	yes	3	2.7812E-20	172.57
ASGN(deFSQVDWFIIHKER	2033.0119	IPI00469123	IPI00469123	yes	no	3	0.011152	72.898
ASGN(deFSQVDWFIIHK	1747.8682	IPI00469123	IPI00469123	yes	no	2,3	7.1266E-18	178.14
ARN(deISGVVIADHSGSFHNR	2036.03	IPI00118674	IPI00118674	yes	yes	2,3	0.0078109	75.682
AQVAN(deISSIIGEISR	1556.8522	IPI00153416	IPI00153416	yes	yes	3	0.0024613	85.457
AQPAN(deQSVIISIYDEK	1774.9101	IPI00626350	IPI00626350	yes	yes	2,3	0.0015623	92.773
AQN(deISISEIVVFWQDQQK	2132.0902	IPI00113949	IPI00113949	yes	no	3	0.0005035	93.768
AQITVTVTQTEHN(deITVSQIPAPQTWR	2918.525	IPI00331026	IPI00331026	yes	yes	3	0.0027451	60.991
AQAECRN(deTTHIIQR	1696.8427	IPI00321222	IPI00321222	yes	yes	2	0.0055961	104.24
AQAAIDKAN(deASR	1214.6367	IPI00119065	IPI00119065	yes	yes	2	0.0071439	94.023
APSN(deVSTVIHIIYIPEEAK	2080.1205	IPI00121421	IPI00121421	yes	no	2,3	0.0001018	95.622
APSHNTTEP(deDPHSISPEIQAIISEVAQHDVQNGR	3673.7721	IPI00165807	IPI00165807	yes	no	4,5	0.0039759	48.607
APSHN(de)TTEPDPHSISPEIQAIISEVAQHDVQNGR	3673.7721	IPI00165807	IPI00165807	yes	no	4,5	0.0039759	48.607
APQHVVNHIPPYTN(deVSIK	2013.0796	IPI00123040	IPI00123040	yes	no	2,3	0.0012165	87.676
APIPTAIDTN(deSSKTPSIFPCASGAFSSFR	3026.4808	IPI00119809	IPI00119809	yes	no	3,4	7.8394E-10	93.21
APIPTAIDTN(deSSK	1313.6827	IPI00119809	IPI00119809	yes	no	2	0.039868	58.32
ANYNIPIMVTDSGKPPMTN(deITDIR	2660.3302	IPI00123746	IPI00123746	yes	yes	3	0.0002977	78.456
ANVVA(deSAIAQIPQK	1408.8038	IPI00153143	IPI00153143	yes	yes	2,3	1.1145E-06	151.9
ANQIVIPHN(deTTFQTEPTK	2038.0484	IPI00466371	IPI01026704	no	no	2,3	8.1981E-76	227
ANIQFGENGT(deTISAVTNK	1863.9327	IPI00127447	IPI00127447	yes	yes	2,3	3.1549E-48	200.45
ANIINFPEN(deNTFVINIEQITQDDTGSYK	3197.5517	IPI00310059	IPI00310059	yes	no	2,3,4	1.1112E-22	127.3
ANEQVIQNIN(deHTYK	1670.8376	IPI00876541	IPI00876541	yes	no	2,3	7.3341E-07	154.88
ANA(deSTFAVPSPVSNSADTR	1890.9072	IPI00119065	IPI00119065	yes	yes	2,3	0.0004441	89.859
AN(deYTIIKGNENGNFK	1681.8424	IPI00138209	IPI00138209	yes	no	3	0.0055489	89.959
AN(deYTIIK	821.4647	IPI00138209	IPI00138209	yes	no	2	0.006133	114.5
AN(deVTFTISK	979.53385	IPI00122584	IPI00122584	yes	yes	2	0.0052631	110.39
AN(deTTQPGIVEGGQVIK	1610.8628	IPI00120245	IPI00120245	yes	no	2,3	3.0642E-08	153.72
AN(deISSQVIK	958.54475	IPI00322867	IPI00322867	yes	no	2	0.0058637	108.43
AN(deHSHSGDYYCK	1437.5732	IPI00129485	IPI00129485	yes	no	2,3	2.3941E-59	164.9
AN(deHSGAVVIIK	1107.64	IPI00420835	IPI00420835	yes	no	2	0.0003032	122.33
AN(deGTTVHVGIHPSK	1416.7474	IPI00132460	IPI00132460	yes	no	3	0.0040037	80.312
AN(deDSDQGANAEIDYTFHQAPEVVR	2646.1946	IPI00719927	IPI00719927	yes	no	3	1.2337E-19	134.86
AN(deATIEVK	844.46543	IPI00757771	IPI00757771	yes	no	1,2	0.01167	110.87
AMSSN(deETAAYK	1171.5179	IPI00316469	IPI00316469	yes	yes	2	1.0856E-32	191.79
AMSN(deISVR	876.44874	IPI00405742	IPI00405742	yes	no	2	2.0779E-28	179.37
AMN(deISASSMTITWK	1539.7425	IPI00469426	IPI00469426	yes	no	2,3	1.8871E-05	149.3
AMIINQHVPMESSHIIQFVN(deWSSIIPERYKK	3694.9116	IPI00387282	IPI00387282	yes	yes	3,4,5	8.89E-07	82.302
AMIINQHVPMESSHIIQFVN(deWSSIIPERYK	3566.8167	IPI00387282	IPI00387282	yes	yes	3,4,5	9.1411E-23	112.74
AMIINQHVPMESSHIIQFVN(deWSSIIPER	3275.6584	IPI00387282	IPI00387282	yes	yes	3,4,5	6.5593E-38	153.24
AMGVNVIAANTHN(deTSMHMTGSGIYSPEAVR	3115.4638	IPI00230412	IPI00230412	yes	yes	3,4	4.6587E-30	122.51
AMFN(deITIIYPNNIIAISNMIPK	2490.3379	IPI00319509	IPI00319509	yes	yes	2,3,4	3.8552E-63	190.96
AIWIIN(deGSK	1000.5706	IPI00124666	IPI00124666	yes	yes	2	0.0022746	120.12
AIVASENN(deMSR	1190.5714	IPI00120674	IPI00120674	yes	no	2	0.0047427	100.93
AISPN(deSTISSAPK	1271.6721	IPI00460291	IPI00460291	yes	no	2	0.0032545	87.083
AISCIES(deSWKTIEQERNASFVYTK	2846.3909	IPI00123223	IPI00123223	yes	yes	3	7.4421E-05	84.249
AINASAN(deITSDGVEVIGR	1785.9221	IPI00330632	IPI00330632	yes	no	2,3	7.6387E-49	207.76
AIN(deVTISSMGRNGIK	1559.8454	IPI00131091	IPI00131091	yes	no	2	0.0034407	118.08
AIN(deVTISSMGR	1147.6019	IPI00131091	IPI00131091	yes	no	2	0.0003158	127.17
AIN(deGSAIYTGSSIDFVR	1769.8948	IPI00877197	IPI00877197	yes	no	2,3	9.9232E-38	198.85
AIN(deDSIHYINR	1314.668	IPI00808345	IPI00808345	yes	no	2,3	6.3784E-11	162.68
AIN(deASQEETGAVFICPWK	2019.9724	IPI00315155	IPI00315155	yes	yes	3	0.0018141	83.966
AIN(deASANITSDGVEVIGR	1785.9221	IPI00330632	IPI00330632	yes	no	2,3	7.6387E-49	207.76
AIMVISEEPIIYMPPPPCQPIIN(deTTESIR	3308.6859	IPI00278602	IPI00278602	yes	yes	4	5.4459E-07	84.68
AIMPFDSIHDDPCIITN(deR	2113.9925	IPI00113480	IPI00113480	yes	no	3	1.0288E-07	138.57
AIMKEVMNIIQPIN(deVTK	1941.0791	IPI00126050	IPI00126050	yes	no	3	0.0001506	115.8
AIMDICDQIEKN(deQTK	1805.8652	IPI00658632	IPI00658632	yes	yes	2,3	0.0013096	109.35
AIKGETVN(deTSIPFSFEGIIFPK	2394.2835	IPI00119324	IPI00119324	yes	no	3	0.0002964	89.374
AIITN(deVSSVAIGSR	1386.7831	IPI00930841	IPI00930841	yes	yes	2	0.0022513	95.067
AIIQGIIPEQN(deYTVQIIAYYKDK	2680.4476	IPI00330632	IPI00330632	yes	no	3	0.0016008	76.155
AIIQGIIPEQN(deYTVQIIAYYK	2437.3257	IPI00330632	IPI00330632	yes	no	3	5.1197E-25	161.35
AIIPFDNIHEDPCIITN(deR	2137.0626	IPI00113854	IPI00113854	yes	no	3	0.00043	95.662
AIGYEN(deATQAIGR	1362.6892	IPI00119809	IPI00119809	yes	no	2	1.2531E-23	188.13
AIGITEMFEPSKAN(deFTK	1882.9499	IPI00115065	IPI00115065	yes	yes	2,3	8.0018E-05	119.68
AIEVAN(deGTVTSIPGATVTIICPGK	2368.2672	IPI00126188	IPI00126188	yes	yes	3	1.4584E-10	106.39
AIDIDPSKTN(deVSGGAIAIGHPIGGSGSR	2646.3725	IPI00226430	IPI00226430	yes	yes	3,4	4.6147E-48	178.11
AIAGIVYN(deSSGTEPCYDIYR	2248.047	IPI00331550	IPI00331550	yes	yes	2,3	3.0859E-155	264.23
AIADVIQDIADDN(deSSR	1701.817	IPI00135805	IPI00135805	yes	no	3	9.2544E-05	129.01

AIAAAGYDVEKN(deNSR	1577.7798	IPI00331597	IPI00331597	yes	no	2,3	5.0267E-17	178.67
AHINN(deISFPIR	1280.699	IPI00876541	IPI00876541	yes	yes	2	0.0006661	113.63
AHFSSIN(deITIR	1257.683	IPI00466371	IPI01026704	no	no	2	0.0050696	87.001
AGYFN(deFTSATITYIAQEDGPVVIGSTSAPGQGGIIAQR	3856.9272	IPI0046718C	IPI0046718C	yes	yes	4,5	1.0163E-50	138.88
AGVVVFN(deCSIR	1220.6336	IPI00314779	IPI00314779	yes	yes	2	0.0003387	130.1
AGPN(deGTIFVVDAYK	1450.7456	IPI00221998	IPI00221998	yes	yes	2,3	1.6659E-10	160.81
AGMASPIYN(deVTWSAGWK	1837.8821	IPI00666034	IPI00666034	yes	no	2,3	1.9804E-08	145.69
AGGSATIN(deCTVTSIIPVGPMR	2101.066	IPI00849192	IPI00849192	yes	no	3	3.0675E-15	139.04
AGEQINNHVKN(deK	1350.7004	IPI00116105	IPI00116105	yes	no	2,3	0.0037544	116.03
AFYN(deGTWYRR	1332.6364	IPI00858318	IPI00858318	yes	no	2	0.014748	91.867
AFYN(deGTWYR	1176.5352	IPI00858318	IPI00858318	yes	no	2	0.016446	89.247
AFVEN(deITVIENSIVFK	1821.9877	IPI00120832	IPI00120832	yes	no	2,3	0	315.66
AFSTIIVN(deVSGK	1234.6921	IPI00222921	IPI00222921	yes	no	2	0.0024619	97.86
AFSGSPN(deITK	1020.524	IPI00119522	IPI00119522	yes	yes	2	0.0029332	101.95
AFQIN(deTFNIKVQPFNVTK	2108.1419	IPI00222967	IPI00222967	yes	no	3	0.0012671	94.922
AFPEVCN(deETMMAIWEECKPCIK	2742.1984	IPI0032042C	IPI0032042C	yes	no	3,4	4.8525E-09	114.96
AFNISPN(deDTSSGSCGINIVTIKVENK	2764.3702	IPI00469218	IPI00469218	yes	yes	3,4	5.1757E-16	122.53
AFNISPN(deDTSSGSCGINIVTIK	2294.1213	IPI00469218	IPI00469218	yes	yes	2,3	9.8632E-77	184.35
AFN(deITWISTDFK	1441.7242	IPI00118069	IPI00118069	yes	no	2	0.002211	102.97
AFN(deECCTIANK	1326.5697	IPI00330833	IPI00330833	yes	yes	2	1.4674E-70	228.12
AFMN(deSSFTIDPK	1356.6384	IPI00121362	IPI00121362	yes	no	2,3	1.7243E-10	162.49
AFITNF(deSMIIDGVTYPGVVK	2171.1337	IPI00312711	IPI00312711	yes	no	2,3	5.8608E-09	129.2
AFIN(deGTGVETVVSADIPNAHGIAVDWVSR	2994.5199	IPI00119063	IPI00119063	yes	yes	2,3,4	3.8196E-86	181.35
AFFDETKN(deNTR	1341.6313	IPI00120674	IPI00120674	yes	no	2	0.0009073	127.87
AEQITIHAIGIGEAN(deKTQIR	2162.1808	IPI00990932	IPI00990932	yes	no	3	7.8977E-08	122.14
AEPPINASAGDQ(deEEK	1554.7162	IPI00123342	IPI00123342	yes	yes	2,3	0.0008162	103.83
AEISN(deHTRPVIIVPGCIGNRIEAK	2643.4279	IPI0013350C	IPI0013350C	yes	yes	3	1.4763E-08	101.38
AEISN(deHTRPVIIVPGCIGNR	2202.1692	IPI0013350C	IPI0013350C	yes	yes	2,3	3.7738E-10	133.45
AEDYGPVEVISHWHPN(deITINIVDDHTPWVK	3480.7103	IPI00121627	IPI00121627	yes	yes	4,5	0.0001006	63.115
ADSIYSQVVGISASQAN(deISK	2037.0378	IPI00115116	IPI00115116	yes	yes	2,3	1.2545E-96	237
ADN(deYTYEHIR	1280.5786	IPI00273133	IPI00273133	yes	yes	2	0.0011019	114.86
ADANPPATEYHWTTIN(deGSIPK	2282.0968	IPI00311405	IPI00311405	yes	yes	2,3	1.3863E-38	184.56
ACVSINHVN(deETVMISITIEYAMQQTK	2979.4504	IPI00624663	IPI00624663	yes	no	3	8.6449E-06	81.48
ACN(deATNWIEYMFNK	1760.7651	IPI0013260C	IPI0013260C	yes	no	2	0.0013249	112.72
ACMN(deETRIEEIR	1520.7075	IPI0039684C	IPI0039684C	yes	no	2,3	0.0031513	118.87
AATCINPINGSVCERPAN(deHSAK	2366.1219	IPI00224752	IPI00224752	yes	yes	3	3.8675E-05	92.993
AATCINPIN(deGSVCERPANHSAK	2366.1219	IPI00224752	IPI00224752	yes	yes	3	3.8675E-05	92.993
AAN(deCTQVIVWHTR	1554.7725	IPI00116744	IPI00116744	yes	yes	2,3	9.5045E-05	148.57
AAIQFIQN(deYTVIASSVDSMDFINDATDVNDAISYVTR	4066.947	IPI00339885	IPI00339885	yes	no	4	7.5388E-27	115.77
AAGVIEDVYN(deRTQGIIAGHGIIQIA	2578.3867	IPI00169617	IPI00169617	yes	yes	3	1.4806E-55	193.85
AAGVIEDVYN(deR	1205.6041	IPI00169617	IPI00169617	yes	yes	2	0.0008847	110.08
AAGN(deSSAIGGQGTSGQPQR	1742.8296	IPI00125182	IPI00125182	yes	no	2	5.8159E-11	139.44
AAEN(deFTIIVK	1104.6179	IPI00471089	IPI00471089	yes	no	2	1.0787E-09	157.68
AACAVRPQEVTMVN(deGTITNPVTGK	2513.273	IPI00308971	IPI00308971	yes	yes	2,3	2.1119E-05	86.539

N-terminal Ser/Thr	281
ratio	15.21%

PNP strategy 2

De-glycopeptides Sequence	Mass	Proteins	Leading ra	Unique (G	Unique (P	Charges	PEP	Score
AAAIKVN(de)DSVPFHIGWNSTER	2311.1709	IPI00464256	IPI00464256	yes	yes	3	9.2438E-08	125.73
AAAIKVNDSVPFHIGWN(de)STER	2311.1709	IPI00464256	IPI00464256	yes	yes	3	9.2438E-08	125.73
AACAVRPQEVTMVN(de)GTITNPVTGK	2513.273	IPI00308971	IPI00308971	yes	yes	2	3.2299E-10	116.35
AAEN(de)FTIIVK	1104.6179	IPI00471089	IPI00471089	yes	no	2	0.0002505	127.87
AAGMN(de)HTK	828.39122	IPI00131143	IPI00131143	yes	no	2	0.017199	105.52
AAGVIEDVYN(de)RTQGIIAGHGIIQIA	2578.3867	IPI00169617	IPI00169617	yes	yes	2,3	9.1159E-16	139.02
AAIQFIQN(de)YTVIASSVDSMDFINDATDVNDAISYVTR	4066.947	IPI00339885	IPI00339885	yes	no	4	0.000111	61.099
AAN(de)CTQVIVWHTR	1554.7725	IPI00116744	IPI00116744	yes	yes	2,3	1.2005E-06	153.94
AATCINPIN(de)GSVCERPANHSAK	2366.1219	IPI00224752	IPI00224752	yes	yes	3	2.3399E-07	120.11
AATCINPINGSVCERPAN(de)HSAK	2366.1219	IPI00224752	IPI00224752	yes	yes	3	2.3399E-07	120.11
ACN(de)ATNWIEYMFNK	1760.7651	IPI0013260C	IPI0013260C	yes	no	2	0.011373	79.771
ADN(de)YTYEHIR	1280.5786	IPI00273133	IPI00273133	yes	yes	2,3	0.0017498	111.63
ADSIYSQVVGISASQAN(de)ISK	2037.0378	IPI00115116	IPI00115116	yes	yes	3	6.4933E-20	160.47
AEDYGPVEVISHWHPN(de)ITINIVDDHTPWVK	3480.7103	IPI00121627	IPI00121627	yes	yes	3,4,5	5.4646E-96	161.04
AEISN(de)HTRPVIIVPGCIGNR	2202.1692	IPI0013350C	IPI0013350C	yes	yes	3	1.3414E-13	151.62
AEISN(de)HTRPVIIVPGCIGNRIEAK	2643.4279	IPI0013350C	IPI0013350C	yes	yes	3,4	9.8587E-09	115.42
AEN(de)QSTNIPGPGRNIPNSQMVNIR	2606.2983	IPI00776189	IPI00776189	yes	no	3	0.011781	66.045
AEPPINASAGDQ(de)EEK	1554.7162	IPI00123342	IPI00123342	yes	yes	2,3	1.2037E-33	195.6
AEQITIHAIGIGEANKT(de)QIR	2162.1808	IPI00990932	IPI00990932	yes	no	3	3.4702E-06	128.59
AEVIAPPTITVECN(de)GSEAHAR	2221.0797	IPI00117841	IPI00117841	yes	no	3	7.9562E-08	120.11
AFAMIIDKIEEDINSSMTN(de)STAASRPPVTIR	3377.6959	IPI00128904	IPI00128904	yes	yes	4	0.0001479	68.97
AFFDETKN(de)NTR	1341.6313	IPI00120674	IPI00120674	yes	no	2,3	0.0018118	113.61
AFIN(de)GTGVETVVSADIPNAHGIAVDWVSR	2994.5199	IPI00119063	IPI00119063	yes	yes	3,4	3.9069E-90	211.5
AFITNF(de)SMIIDGVTYPGVVK	2171.1337	IPI00312711	IPI00312711	yes	no	2,3	4.0463E-94	233.31
AFITNF(de)SMIIDGVTYPGVVKEK	2428.2712	IPI00312711	IPI00312711	yes	no	3,4	2.9148E-22	160.55
AFMN(de)SSFTIDPK	1356.6384	IPI00121362	IPI00121362	yes	no	2,3	6.8729E-29	149.69
AFN(de)ECCTIANK	1326.5697	IPI00330833	IPI00330833	yes	yes	2	1.1412E-30	188.91
AFN(de)ITWISTDFK	1441.7242	IPI00118069	IPI00118069	yes	no	2	7.9466E-31	190.85
AFNISPN(de)DTSSGSCGINIVTIK	2294.1213	IPI00469218	IPI00469218	yes	yes	2,3	1.7355E-174	273.51
AFNISPN(de)DTSSGSCGINIVTIKVENK	2764.3702	IPI00469218	IPI00469218	yes	yes	3	2.9511E-08	109.75
AFPEVCN(de)ETMMAIWEECKPCIK	2742.1984	IPI0032042C	IPI0032042C	yes	no	3,4	2.9622E-39	184.22
AFQIN(de)TFNIKVQPFNVTK	2108.1419	IPI00222967	IPI00222967	yes	no	3	0.0002991	117
AFSGSPN(de)ITK	1020.524	IPI00119522	IPI00119522	yes	yes	2	0.007393	101.39
AFSTIIVN(de)VSGK	1234.6921	IPI00222921	IPI00222921	yes	no	2	0.0008965	126.95
AFVEN(de)ITVIENSIVFK	1821.9877	IPI00742385	IPI00742385	yes	no	2,3	0	339.58

AFYN(de)GTWYR	1176.5352	IPI0085831ε	IPI0085831ε	yes	no		2	0.014021	101.11
AGAICISN(de)DTGIGISSVVPIQAFK	2417.2624	IPI00123223	IPI00123223	yes	yes		3	1.25E-08	112.32
AGAICISN(de)DTGIGISSVVPIQAFKPFFVEVSIPYSVVR	4037.1336	IPI00123223	IPI00123223	yes	yes		3	0.0003199	61.725
AGEQINNHVKN(de)K	1350.7004	IPI00116105	IPI00116105	yes	no		2	0.034181	89.403
AGEQINNHVKN(de)KTQGK	1764.9231	IPI00116105	IPI00116105	yes	no	2,3		0.0087803	86.548
AGGSATIN(de)CTVTSIIPVGPMR	2101.066	IPI00849192	IPI00849192	yes	no		3	1.7312E-07	107.15
AGIN(de)ASYSEK	1038.4982	IPI00308971	IPI00308971	yes	yes		2	0.016896	90.827
AGMASPIYN(de)VTWSAGWK	1837.8821	IPI00666034	IPI00666034	yes	no	2,3		1.2221E-05	132.51
AGPN(de)GTIFVVDAYK	1450.7456	IPI00221998	IPI00221998	yes	yes	2,3		5.4015E-32	190.87
AGYFN(de)FTSATITYIAQEDGPVVIGSTSAPGQGGIIAQR	3856.9272	IPI0046718C	IPI0046718C	yes	yes	4,5		2.6679E-35	143.79
AHINN(de)ISFPIR	1280.699	IPI00876541	IPI00876541	yes	yes		2	0.004183	97.734
AIAAAGYDVEKN(de)NSR	1577.7798	IPI00223714	IPI00223714	no	no		2	0.0008158	117.2
AIADVIQDIADDN(de)SSR	1701.817	IPI00135805	IPI00135805	yes	no		3	0.0011943	95.854
AIAGIVYN(de)SSGTEPCYDIYR	2248.047	IPI0033155C	IPI0033155C	yes	yes	2,3		1.0037E-11C	248.35
AIDIDPSKTN(de)VSGGAIAIGHPIGGSGSR	2646.3725	IPI0022643C	IPI0022643C	yes	yes	3,4		6.0429E-15ε	171.05
AIEIQFGMN(de)ASSSIFFIQGVR	2314.178	IPI0046921ε	IPI0046921ε	yes	yes	2,3		2.6699E-59	202.03
AIEISTVTCQIN(de)CSDHGHCDSFTK	2779.2	IPI00453492	IPI00453492	yes	no		3	0.0009742	78.794
AIEISTVTCQIN(de)CSDHGHCDSFTKR	2935.3011	IPI00453492	IPI00453492	yes	no		4	0.018329	59.857
AIEVAN(de)GTVTSIPGATVTIICPGK	2368.2672	IPI0012618ε	IPI0012618ε	yes	yes		3	5.6119E-12	117.48
AIGITEMFEPSKAN(de)FTK	1882.9499	IPI00115065	IPI00115065	yes	yes	2,3		0.0012722	105.39
AIGYEN(de)ATQAIGR	1362.6892	IPI00119809	IPI00119809	yes	no		2	5.4381E-54	210.91
AIIAN(de)ITCK	1002.5532	IPI00880302	IPI00880302	yes	no		2	0.0065507	107.79
AIIPFDNIHEDPCIITN(de)R	2137.0626	IPI00113854	IPI00113854	yes	no		3	0.0030227	83.106
AIIQGIIPEQN(de)YTVQIIAYYK	2437.3257	IPI00330632	IPI00330632	yes	no		3	3.1138E-08	124.63
AIIQGIIPEQN(de)YTVQIIAYYKDK	2680.4476	IPI00330632	IPI00330632	yes	no		3	0.0042432	77.39
AIITN(de)VSSVAIGSR	1386.7831	IPI00930841	IPI00930841	yes	yes		2	0.0001603	119.54
AIKGETVN(de)TSIPFSFEGIIFPK	2394.2835	IPI00119324	IPI00119324	yes	no		3	1E-07	122.29
AIMDICDQIEKN(de)QTK	1805.8652	IPI00658632	IPI00658632	yes	yes	2,3		9.1E-06	142.54
AIMKEVMNII(de)QPINVTK	1941.0791	IPI0012605C	IPI0012605C	yes	no		3	0.001159	107.69
AIMPFDSIHDDPCIITN(de)R	2113.9925	IPI00929801	IPI00929801	yes	no	2,3		3.0197E-12	153.86
AIMVISEEPIIYMPPPPCQPIIN(de)TTESIR	3308.6859	IPI00278602	IPI00278602	yes	yes		4	2.8885E-06	95.71
AIN(de)ASANITSDGVEVIGR	1785.9221	IPI00330632	IPI00330632	yes	no	2,3		6.172E-105	240.42
AIN(de)ASQEETGAVFICPWK	2019.9724	IPI00315155	IPI00315155	yes	yes	2,3		1.0666E-08	141.6
AIN(de)DSIHYINR	1314.668	IPI00808345	IPI00808345	yes	no		3	0.0009218	110.38
AIN(de)FTIPFFDSSR	1513.7565	IPI00828867	IPI00828867	yes	yes		2	0.0033374	97.551
AIN(de)GSAIYTGSSIDFVR	1769.8948	IPI00877197	IPI00877197	yes	no	2,3		2.1996E-141	262.25
AIN(de)VTISSMGR	1147.6019	IPI00131091	IPI00131091	yes	no		2	0.0004778	131.17
AIN(de)VTISSMGRNGIK	1559.8454	IPI00131091	IPI00131091	yes	no		2	4.8433E-17	175.66
AINASAN(de)ITSDGVEVIGR	1785.9221	IPI00330632	IPI00330632	yes	no	2,3		6.172E-105	240.42
AINS(de)STEDGIKR	1289.6575	IPI0012916ε	IPI0012916ε	yes	yes		2	0.025942	82.543
AISCIES(de)SWKTIEQERNASFVYTK	2846.3909	IPI00123223	IPI00123223	yes	yes		3	0.017489	64.2
AISPN(de)STISSAPK	1271.6721	IPI00460291	IPI00460291	yes	no		2	0.0001823	140.82
AITIKDIIN(de)GTFSYK	1682.9243	IPI00123925	IPI00123925	yes	no		2	1.4779E-24	186.77
AIVAVDPGN(de)HTAPR	1416.7474	IPI00129631	IPI00129631	yes	yes		2	0.013854	77.912
AIVN(de)FTR	819.46029	IPI0051536C	IPI0051536C	yes	no		2	0.0090831	100.19
AIWIIN(de)GSK	1000.5706	IPI00124666	IPI00124666	yes	yes		2	0.0031096	119.68
AKGVAV(de)TNTSQIGFR	1547.842	IPI00471089	IPI00471089	yes	no	2,3		1.0612E-16	169.04
AMFN(de)ITIIYPNNIIAISNMIPK	2490.3379	IPI00319509	IPI00319509	yes	yes	2,3,4		2.5972E-12	143.57
AMGVNVIAANTHN(de)TSMHMTGSGIYSPEAVR	3115.4638	IPI00230412	IPI00230412	yes	yes	3,4		7.5349E-07	96.793
AMIINQHVPMESSHIIQFVN(de)WSSIIPER	3275.6584	IPI00387282	IPI00387282	yes	yes	3,4,5		5.7022E-28	156.88
AMIINQHVPMESSHIIQFVN(de)WSSIIPERYK	3566.8167	IPI00387282	IPI00387282	yes	yes	3,4,5		0.0001201	77.689
AMIINQHVPMESSHIIQFVN(de)WSSIIPERYKK	3694.9116	IPI00387282	IPI00387282	yes	yes	3,4,5		8.3699E-11	106.93
AMN(de)ISASSMTITWK	1539.7425	IPI00469426	IPI00469426	yes	no	2,3		1.4031E-05	143.05
AMN(de)TSQVEAMGIQMIPGYR	2095.9853	IPI0016987C	IPI0016987C	yes	yes		3	2.4267E-05	116.06
AMN(de)TSQVEAMGIQMIPGYRDPYHGRPITK	3260.5893	IPI0016987C	IPI0016987C	yes	yes		4	0.024014	50.986
AMSN(de)ISVR	876.44874	IPI00405742	IPI00405742	yes	no		2	5.6341E-33	179.37
AMSSN(de)ETAAYK	1171.5179	IPI00316469	IPI00316469	yes	yes		2	6.2524E-10	162.49
AN(de)DSDQGANAEIDYTFHQAPEVVR	2646.1946	IPI00719927	IPI00719927	yes	no		3	0.0090161	61.616
AN(de)DSDQGANAEIDYTFHQAPEVVRR	2802.2957	IPI00719927	IPI00719927	yes	no		3	1.7145E-07	109.5
AN(de)HSGAVVIIK	1107.64	IPI00420835	IPI00420835	yes	no		2	0.006283	93.345
AN(de)HSGAVVIIKR	1263.7412	IPI00420835	IPI00420835	yes	no		2	0.016044	87.719
AN(de)TSIEIIIEGVR	1413.7827	IPI0098851ε	IPI0098851ε	yes	no	2,3		6.0419E-10	160.48
AN(de)TSQPGVIQGGAVYVCPWGTSPIQCTTIQFDSK	3666.7447	IPI00115976	IPI00115976	yes	yes		4	4.1036E-07	83.939
AN(de)TTQPGIVEGGQVIK	1610.8628	IPI00120245	IPI00120245	yes	no	2,3		6.991E-26	185.02
ANEQVIQNIN(de)HTYK	1670.8376	IPI00876541	IPI00876541	yes	no	2,3		3.7705E-07	149
ANIQFGENGT(de)TISAVTNK	1863.9327	IPI00127447	IPI00127447	yes	yes	2,3		1.4013E-48	205.65
ANIQKGENPSQYGITAFNHPIN(de)ITK	2754.4089	IPI00112614	IPI00112614	yes	yes		3	9.6755E-09	114.92
ANQIVIPHN(de)TTFQTEPTK	2038.0484	IPI00466371	IPI01026704	no	no	2,3		3.8786E-48	199.58
ANYNIPIMVTDSGKPPMTN(de)ITDIR	2660.3302	IPI00123746	IPI00123746	yes	yes		3	0.0016395	83.063
APIPTAIDTN(de)SSK	1313.6827	IPI00119809	IPI00119809	yes	no		2	0.014419	79.466
APIPTAIDTN(de)SSKTPSIFPCASGAFSSFR	3026.4808	IPI00119809	IPI00119809	yes	no	3,4		9.9927E-09	107.03
APQHVVNHIPPYTN(de)VSIK	2013.0796	IPI0012304C	IPI0012304C	yes	no		3	0.0055614	78.326
AQAAIDKAN(de)ASR	1214.6367	IPI00119065	IPI00119065	yes	yes	2,3		1.1369E-81	229.75
AQAECRN(de)TTHIIQR	1696.8427	IPI00321222	IPI00321222	yes	yes		2	0.014268	88.087
AQITVTVTQTEHN(de)ITVSQIPAPQTWR	2918.525	IPI00331026	IPI00331026	yes	yes		3	1.2829E-08	108.08
AQTN(de)YTCVAEIIYR	1700.8192	IPI00323053	IPI00323053	yes	no	2,3		4.8174E-16	169.37
AQVAN(de)ISSIIGEISR	1556.8522	IPI00153416	IPI00153416	yes	yes	2,3		2.3039E-24	180.81
ASGN(de)FSQVDWFIIHK	1747.8682	IPI00469123	IPI00469123	yes	no	2,3		2.2038E-33	192.96
ASQQISN(de)ETSSFGFNIIR	1997.9807	IPI00153258	IPI00153258	yes	no	2,3		1.912E-123	178.72
ASQQISN(de)ETSSFGFNIIRK	2126.0756	IPI00153258	IPI00153258	yes	no	2,3		2.7722E-07	116.6
ASSIMVN(de)ITEIFPKPK	1773.9699	IPI00406901	IPI00406901	yes	no		3	3.6475E-102	249.3
ASVVN(de)VTQHCTMESWESMNEVAR	2664.1731	IPI0012124C	IPI0012124C	yes	yes	3,4		7.3464E-60	199.97
ASVVN(de)VTQHCTMESWESMNEVARR	2820.2742	IPI0012124C	IPI0012124C	yes	yes		3	0.050846	43.476
ATHAQPAIVTAPN(de)ETSPK	1831.9428	IPI0013795ε	IPI0013795ε	yes	yes		3	0.0074292	75.018
ATPNEGFFNQN(de)ITTFYYDR	2297.0389	IPI00315535	IPI00315535	yes	yes	2,3		5.7987E-20	164.94
ATSQFQN(de)GSIITAFIPGISQCTVYSAK	2888.4378	IPI0034014C	IPI0034014C	yes	yes		3	6.8393E-10	115.55

ATVN(de)DSGEYR	1110.4942	IPI00129485	IPI00129485	yes	no		2	0.0002738	125.72
AVAYGEKN(de)ITFQGPIPK	1831.9832	IPI00111794	IPI00111794	yes	no	2,3,4		8.3309E-74	225.32
AVGAVDWDSHPYGYN(de)ITIQAK	2304.1175	IPI00623114	IPI00623114	yes	no		3	1.2793E-11	138.08
AVGN(de)STFAIACIQR	1506.7613	IPI00761772	IPI00761772	yes	no		2	0.0014262	106.52
AVIGDHGDEIFSVFGSPFIKDGASEEETN(de)ISK	3394.6205	IPI00387289	IPI00387289	yes	yes	3,4		1.2656E-07	98.299
AVISMIN(de)ETMNEAPEETK	2005.9336	IPI00875419	IPI00875419	yes	no	2,3		1.671E-75	228.3
AVIVNN(de)ITTGEK	1257.6929	IPI00128905	IPI00128905	yes	yes		2	0.01673	79.633
AVKIN(de)ETIGNQDK	1428.7573	IPI00309999	IPI00309999	yes	yes		2	0.050978	76.728
AVKIN(de)ETIGNQDKTAER	1885.9858	IPI00309999	IPI00309999	yes	yes		3	7.8277E-60	215.48
AVN(de)QTGAIYQCDYSTSR	1932.8636	IPI00120674	IPI00120674	yes	no		2	1.1319E-20	171.21
AVTSISHDGSVICTFTAN(de)STYTK	2459.1639	IPI00453488	IPI00453488	yes	yes		3	1.058E-12	144.27
AVVGSYTTTN(de)VSIIAPR	1747.9468	IPI00330481	IPI00330481	yes	yes	2,3		4.8337E-15	158.64
AVYEAIRN(de)CSIEDDSVR	1995.932	IPI00856861	IPI00856861	yes	no		2	0.0019527	97.235
AWEKEIHHI(de)QEQNVSNVFIDKEEFFIGSK	3500.7365	IPI00605187	IPI00605187	yes	no	3,4		1.736E-26	149.18
AWGISVINPNK(de)TK	1426.7932	IPI00989096	IPI00989096	yes	no		3	0.0029178	110.8
AWGTPCEICPSVN(de)TSEYK	2097.9136	IPI00828222	IPI00828222	yes	no	2,3		7.7535E-12	148.36
AWN(de)YTVDTPHGMFVSGTPFEQSVWITDVGSGPYGHTVK	4166.9473	IPI00228567	IPI00228567	yes	yes	4,5		5.1652E-35	140.27
AWN(de)YTVDTPHGMFVSGTPFEQSVWITDVGSGPYGHTVKK	4295.0422	IPI00228567	IPI00228567	yes	yes	4,5		4.7438E-06	65.134
AY(de)AISGPTVQDGIPPFNWNRFNETIHR	3099.5315	IPI00471081	IPI00471081	yes	yes	3,4		0.0001619	91.166
AYTIFVPTN(de)HSIETQGNNSVIGIDTVR	2945.4883	IPI00987265	IPI00987265	yes	no	3,4		2.0107E-19	144.63
AYTIFVPTNHSIETQGN(de)NSVIGIDTVR	2945.4883	IPI00987265	IPI00987265	yes	no	3,4		2.0107E-19	144.63
AYVFERN(de)QSVGDPNVDIIR	2191.1022	IPI00127447	IPI00127447	yes	yes	2,3		1.2216E-41	195.38
AYVPN(de)ATWYDYETGEEIGWR	2419.0757	IPI00848693	IPI00848693	yes	yes	2,3		7.3646E-26	171.72
CAISHAITIEFHQDGNGPENVGIYN(de)ISR	3111.4832	IPI00222429	IPI00222429	yes	yes	3,4,5		4.5498E-120	239.57
CAMDIGVNYIGTVN(de)VTHTGIQCQIWR	3005.431	IPI00114206	IPI00114206	yes	yes	3,4		1.4296E-22	157.28
CCGWVSHYN(de)WTENEEIMGFTK	2647.093	IPI00108098	IPI00108098	yes	no		3	2.9471E-13	147.06
CDGDFDCEDRTDEAN(de)CSVK	2291.8365	IPI00515360	IPI00515360	yes	no		3	0.034315	69.485
CDGDFDCEDRTDEAN(de)CSVKQPGEVCGPTHFQCVSTNR	4346.7416	IPI00515360	IPI00515360	yes	no		4	0.0069943	49.944
CEQEASEDIKPAITGN(de)K	1888.8837	IPI00134808	IPI00134808	yes	no	2,3		1.5348E-07	138.05
CEQEASEDIKPAITGN(de)KTMQYVPNSHDVK	3288.5391	IPI00134808	IPI00134808	yes	no		4	0.033764	51.586
CF(de)ANKVNISFPSAQSIPASDTHIK	2631.3115	IPI00624663	IPI00624663	yes	yes	2,3,4		1.8159E-67	204.12
CFMAN(de)GTWGYPFHDGDYIK	2277.9612	IPI00947585	IPI00947585	yes	no		3	7.7606E-20	163.8
CFMAN(de)GTWGYPFHDGDYIKDQAIWSECKEPR	3777.6439	IPI00947585	IPI00947585	yes	yes		4	0.000207	67.655
CFNAMEVDAIN(de)SSHPVSTPVENPAQIR	2982.3964	IPI00318595	IPI00318595	yes	no	3,4		2.6106E-84	218.44
CGNCN(de)ITSIEDEDFCK	1960.7601	IPI00137599	IPI00137599	yes	yes	2,3		6.1255E-46	207.92
CGPPPAIPNAIPASDVN(de)RTDFESHTTIK	3004.4713	IPI00134808	IPI00134808	yes	no	3,4		0.0069127	62.064
CGWSPAEEIN(de)YTVPGIGSAGEASFISQCMK	3245.4468	IPI00331032	IPI00331032	yes	yes		3	6.8524E-07	100.7
CHAGYIGDNCN(de)CSTDVSTCR	2345.8882	IPI00377953	IPI00377953	yes	yes		3	4.6209E-05	101.5
CHEGN(de)GTFECGACR	1653.6082	IPI00132474	IPI00132474	yes	yes	2,3		7.5876E-05	131.69
CIIVGNGGVIAN(de)K	1313.7126	IPI00311893	IPI00311893	yes	no		2	0.0010788	125.97
CIN(de)HTTQK	1000.476	IPI00125266	IPI00125266	yes	yes		2	0.0021491	135.83
CIPHFAMIGN(de)DTVMCTEQGNWTR	2737.1869	IPI00322463	IPI00322463	yes	no		3	3.8324E-49	192.18
CIPHFAMIGN(de)DTVMCTEQGNWTRIPECIEVK	3705.687	IPI00322463	IPI00322463	yes	no	3,4		2.2201E-40	158.91
CIPHFAMIGNDTVMCTEQGN(de)WTR	2737.1869	IPI00322463	IPI00322463	yes	no		3	3.8324E-49	192.18
CIPHFAMIGNDTVMCTEQGN(de)WTRIPECIEVK	3705.687	IPI00322463	IPI00322463	yes	no	3,4		2.2201E-40	158.91
CIQIQAINM(de)SSCPIDSR	1991.9227	IPI00648288	IPI00648288	yes	no		3	7.4915E-06	134.25
CISKN(de)YTTDIITK	1555.7916	IPI00124640	IPI00124640	yes	yes	2,3		0.0005664	140.31
CISPN(de)VTSCACTINFTIK	2084.9693	IPI00387318	IPI00387318	yes	yes	2,3		6.5941E-123	254.82
CISPNVTSCACTIN(de)FTIK	2084.9693	IPI00387318	IPI00387318	yes	yes	2,3		6.5941E-123	254.82
CIVPHTVN(de)ISSAWR	1638.83	IPI00129041	IPI00129041	yes	no	2,3		0.0019418	103.76
CN(de)ITIKDPTPADPIWYEAK	2231.0933	IPI00114842	IPI00114842	yes	no		2	0.011223	79.676
CN(de)TTQGNEVTSIIR	1591.7624	IPI00134743	IPI00134743	yes	no		2	7.4834E-11	160.47
CNQFVDCEDASDEMN(de)CSATDCSSYFR	3167.1307	IPI00119063	IPI00119063	yes	yes		3	9.3405E-13	122.41
CNSVITYN(de)ITPVVQK	1734.8975	IPI00222967	IPI00222967	yes	no	2,3		7.6133E-159	277.94
CNTAAPMWIN(de)GSHPSSSEGIVSR	2457.1165	IPI00128334	IPI00128334	yes	yes		3	0.015743	59.339
CPIITPFN(de)DTNVVHTVNTAIAAFNTQNNGTYFK	3681.7886	IPI00128249	IPI00128249	yes	yes	3,4		4.4818E-06	79.67
CPIITPFNDTNVVHTVNTAIAAFNTQNN(de)GTYFK	3681.7886	IPI00128249	IPI00128249	yes	yes	3,4		4.4818E-06	79.67
CQAIDFFPQN(de)ITMR	1739.8123	IPI00331440	IPI00331440	yes	no		3	0.000123	124.07
CQAIDFFPQN(de)ITMRWIKDNQPIDAK	3048.495	IPI00331440	IPI00331440	yes	no		3	0.054847	50.752
CQEI(de)ISVDCSTNNPAQANIRQEINDSIQVAER	3671.7268	IPI00320420	IPI00320420	yes	yes		3	3.0073E-07	93.667
CQIEN(de)GSFKENSQYIPIK	2154.0416	IPI00330833	IPI00330833	yes	yes		3	0.013751	79.81
CQSPYQMN(de)GSDTVTCVNSR	2202.9092	IPI00130010	IPI00130010	yes	no		2	7.6963E-14	153.04
CSGN(de)GTYTCGICECDPGYIGTR	2496.9767	IPI00377953	IPI00377953	yes	no		3	0.0021601	76.118
CSIIHSN(de)ISVK	1256.6547	IPI00133222	IPI00133222	yes	no	2,3		2.0537E-41	199.82
CTPPPYVENAVMISEN(de)R	1975.9132	IPI00138061	IPI00138061	yes	no	2,3		5.0866E-15	157.8
CV(de)VHYENSTVPEKKNITSPVGVQPIINEHTFCAGITK	4166.0929	IPI00409148	IPI00409148	yes	yes	4,5		1.0327E-11	98.189
CVN(de)ESYKK	1026.4804	IPI00331318	IPI00331318	yes	no		2	0.015465	128.38
CVVHYEN(de)STVPEK	1560.7242	IPI00409148	IPI00409148	yes	yes		2	0.0005456	127.02
CVVHYEN(de)STVPEKK	1688.8192	IPI00409148	IPI00409148	yes	yes	2,3,4		7.7923E-69	225.97
CVVHYENSTVPEKKNITS(de)PVGVQPIINEHTFCAGITK	4166.0929	IPI00409148	IPI00409148	yes	yes	4,5		1.0327E-11	98.189
CVYEAICSN(de)VTSECPPPVITR	2451.1233	IPI00405742	IPI00405742	yes	no		2	1.5922E-05	100.19
CWAPIIDN(de)DTASDNGSR	1890.8166	IPI00226346	IPI00226346	yes	no		2	0.0014888	94.544
CWAPIIDNDTASDN(de)GSR	1890.8166	IPI00226346	IPI00226346	yes	no		2	0.0014888	94.544
DAAQCSGGSVAHIAEIGIPTN(de)ITHIIIFR	3060.5815	IPI00331407	IPI00331407	yes	no	3,4		1.5689E-33	162.82
DAGN(de)YTVIITNPISMEK	1864.9241	IPI00124326	IPI00124326	yes	no	2,3		5.2113E-06	135.09
DAGVGFPEYQEEDN(de)STYNFR	2336.9822	IPI00308971	IPI00308971	yes	yes		2	1.9878E-79	221.51
DAMVGN(de)YTCEVTEISR	1843.808	IPI00124830	IPI00124830	yes	no	2,3		2.3233E-119	252.82
DATIQAYISSGN(de)FSK	1600.7733	IPI00469218	IPI00469218	yes	yes		2	0.000967	108.14
DCDFIEDGEERNCTG(de)K	1943.7626	IPI00127280	IPI00127280	yes	yes	2,3		0.0020444	107.74
DCVSCQN(de)VSR	1223.5023	IPI00121190	IPI00121190	yes	no		2	6.2617E-23	186.74
DDIHPTIPAGQYFIN(de)ITYNYPVHSFDGR	3249.552	IPI00387318	IPI00387318	yes	no	3,4		1.102E-62	190.67
DDYRPTWTIN(de)QTEPVAGNYYPVNTR	2969.3944	IPI00381303	IPI00381303	yes	yes	3,4		3.7171E-16	143.91
DEIGNVSTSHIIIIDDSV(de)EMEIRPR	2837.4229	IPI00309035	IPI00309035	yes	yes		3	1.281E-12	126.25
DFIPSTISFTWNYQN(de)NTEVIQGIR	2842.3926	IPI00468055	IPI00468055	yes	no		3	0.0004114	84.946
DGIAIN(de)ITIR	1084.6241	IPI00119065	IPI00119065	yes	yes		2	2.4585E-05	145.28
DGIN(de)FSCQAEIDIRPHGGYIIR	2530.2387	IPI00117424	IPI00117424	yes	no		3	0.0011801	88.565

DGQQN(de)ISIIYTEPGASQTQTGASFR	2668.2729	IPI00284816	IPI00284816	yes	no		3	1.93E-13	130.37
DGSDSAAMVYN(de)SSQEWGIR	2071.8905	IPI00119522	IPI00119522	yes	yes	2,3		2.3929E-215	292.6
DGTSQPAICPQN(de)VTMNMEGIK	2290.0392	IPI00320204	IPI00320204	yes	yes	2,3,4		7.1154E-08	119.37
DGTVTAGN(de)ASGVSDGAGAVIIASEDAVK	2531.2351	IPI00226430	IPI00226430	yes	yes		3	0.0001385	89.297
DHVN(de)VSMIEATDFPFNTTEWEGYIPK	3039.396	IPI00136942	IPI00136942	yes	yes		3	1.1611E-16	140.59
DHVNVSMIEATDFPFN(de)TTEWEGYIPK	3039.396	IPI00136942	IPI00136942	yes	yes		3	1.1611E-16	140.59
DIAVGDDYTN(de)R	1237.5575	IPI00468674	IPI00468674	yes	no		2	0.0045714	96.604
DIDMFIN(de)ASK	1152.5485	IPI00224752	IPI00224752	yes	yes		2	0.0006274	115.33
DIDTDFTSN(de)ASQPETK	1767.7799	IPI00130573	IPI00130573	yes	yes		2	2.1932E-18	170.11
DIEHHITNVN(de)VSFYDDIVNGTVIK	2741.3661	IPI00467600	IPI00467600	yes	yes		3	2.2553E-80	215.65
DIEHHITNVNVSFYDDIVN(de)GTVIK	2741.3661	IPI00467600	IPI00467600	yes	yes		3	2.2553E-80	215.65
DIFIN(de)VTPDYEENSIIQADVECK	2711.2636	IPI00314132	IPI00314132	yes	no		3	0.0006605	84.762
DIFTNQSDFADTTKDTPITITVIH(de)K	2820.4182	IPI00116105	IPI00116105	yes	yes		3	6.2969E-08	106.86
DIGPAIAN(de)SSHDVK	1422.7103	IPI00108811	IPI00108811	yes	yes	2,3		2.4162E-11	165.49
DIGTASHN(de)FSGAIPR	1541.7587	IPI00229820	IPI00229820	yes	yes	2,3		1.6477E-19	178.14
DIIFSDDTECISNIQN(de)K	2010.9204	IPI00471080	IPI00471080	yes	no		2	1.226E-236	309.15
DIIFSDDTECISNIQN(de)KTTYK	2504.1741	IPI00471080	IPI00471080	yes	no		3	0.0048576	80.638
DIIIHSTGHN(de)ISR	1461.7688	IPI00223987	IPI00223987	yes	yes	2,3		0.0002196	139.48
DIN(de)STAQNVMFYDMGSGSTVCTIVTYQTVK	3329.5254	IPI00123342	IPI00123342	yes	no	3,4		1.6219E-27	149.94
DINGNVFQDAVFN(de)QTVTVIER	2378.1866	IPI00110852	IPI00110852	yes	no		3	0.0038339	77.899
DINSQIEIQN(de)VTNDTHITIIGIKPDTTYDIK	3511.8046	IPI00110264	IPI00110264	yes	yes	3,4		5.7202E-34	149.07
DINSQIEIQNVTN(de)DTHITIIGIKPDTTYDIK	3511.8046	IPI00110264	IPI00110264	yes	yes	3,4		5.7202E-34	149.07
DIQNFIEN(de)VTSSVDVK	1806.9	IPI00990932	IPI00990932	yes	no		2	6.0961E-157	176.94
DISGN(de)ETHFTGSEVGFIKPISCR	2550.2173	IPI00123652	IPI00123652	yes	no		3	1.6588E-07	109.72
DISHSIYMFFN(de)TSDIR	1944.904	IPI00114457	IPI00114457	yes	yes	2,3		3.026E-05	130.24
DISVFAPN(de)MTEIIK	1576.8171	IPI00855103	IPI00855103	yes	no		2	0.0001199	124.51
DISVFAPN(de)MTEIIKDVTQYR	2339.1831	IPI00855103	IPI00855103	yes	no		3	0.0018408	88.427
DITNIINNTF(de)IR	1432.7674	IPI00650038	IPI00650038	yes	no		2	0.0026054	101.53
DIVGN(de)ITEIESEDIQIEAIIMR	2500.2731	IPI00122399	IPI00122399	yes	no	2,3		2.7559E-207	197.46
DIVQNVFMSN(de)MSMDIQSHPSSCPK	2751.2125	IPI00121190	IPI00121190	yes	no	3,4		3.7107E-12	116.73
DIVV(de)EIYSQWMKNPNNNTIHPNIR	2894.4497	IPI00319509	IPI00319509	yes	no	3,4		5.3323E-12	124.08
DIWN(de)VTGAK	1002.5134	IPI00652107	IPI00652107	yes	no		2	0.064725	71.555
DIYEYSWVQGPWNSN(de)RTER	2399.0931	IPI00118674	IPI00118674	yes	yes		3	0.0003995	96.673
DKISPIHIA(de)INFSIDPK	1907.0516	IPI00115976	IPI00115976	yes	yes		3	0.0042392	90.707
DKN(de)GTRAEPPINASAGDQEEK	2226.0513	IPI00123342	IPI00123342	yes	yes		2	0.0027333	86.727
DKNGTRAEPPIN(de)ASAGDQEEK	2226.0513	IPI00123342	IPI00123342	yes	yes		2	0.0027333	86.727
DMEEFVQSSGDHGVVVFSIGSMVSN(de)MTEEK	3274.4468	IPI00128137	IPI00112322	no	no		3	3.3225E-77	206.33
DMSDEIGCVNVTQCDGPNK(de)FK	2413.0348	IPI00312063	IPI00312063	yes	no		3	4.1519E-07	111.78
DMSDGFISN(de)ITIQR	1595.7614	IPI00272381	IPI00272381	yes	no	2,3		2.9077E-34	198.74
DMWADYIIFMDIDNIITNPDTISIIIAEN(de)K	3496.7146	IPI00169870	IPI00169870	yes	yes	3,4		1.1452E-07	105.03
DNATDS(de)VPIR	1086.5306	IPI00119063	IPI00119063	yes	yes		2	0.0016828	111.86
DNATQEEIIHYIE(de)K	1701.821	IPI00321190	IPI00321190	yes	no	2,3		3.0937E-23	181.51
DNSCVDPPHVPN(de)ATIVTR	1990.9531	IPI00130010	IPI00130010	yes	no	2,3		1.4013E-48	205.65
DNTTC(de)YEFK	1176.4757	IPI00119063	IPI00119063	yes	yes		2	0.036794	85.958
DNTTC(de)YEFKK	1304.5707	IPI00119063	IPI00119063	yes	yes		2	0.0032196	112.13
DNVN(de)CSGVYEHEPIEIGK	2058.9317	IPI00947577	IPI00947577	yes	yes	2,3		5.1879E-12	151.41
DQCIVDDITYNVN(de)DTFHK	2195.9793	IPI00975193	IPI00975193	yes	no	2,3		9.7815E-75	223.11
DQCIVDDITYNVN(de)DTFHKR	2352.0805	IPI00975193	IPI00975193	yes	no	2,3		7.2694E-05	117.3
DQDCDKF(de)NQCGTCTEFKECHTIQNYTIWR	3753.5705	IPI00125220	IPI00125220	yes	no		4	0.025662	52.97
DQGNV(de)TDMASMK	1295.5486	IPI00471081	IPI00471081	yes	yes	2,3		0.0001473	142.85
DSGIWINGFDYTGMSHVTPHIPEIN(de)DTIR	3284.5561	IPI00407222	IPI00407222	yes	yes	3,4		6.9718E-14	117.43
DSSGVINVMIN(de)GSEPK	1645.7981	IPI00132279	IPI00132279	yes	no		3	0.017829	69.485
DSYPDGN(de)ITWYR	1485.6525	IPI00121378	IPI00121378	yes	no		2	0.0006367	116.01
DSYPDGN(de)ITWYRNGK	1784.8118	IPI00121378	IPI00121378	yes	no	2,3		0.0011039	119.25
DTAVEGEEIEVNCTAMASKP(de)ATTIR	2692.2684	IPI00856723	IPI00856723	yes	no	2,3		5.8224E-18	119.38
DTCAQECSHFNI(de)TK	1709.7138	IPI00132474	IPI00132474	yes	no	2,3		4.4974E-07	147.35
DTISIN(de)ATNIK	1188.635	IPI00121190	IPI00121190	yes	no		2	6.3113E-68	228.12
DTN(de)GSQFFITTVK	1456.7198	IPI00135686	IPI00135686	yes	yes		2	0.00041	133.66
DTTGSHTFQGMFGCEITNN(de)R	2271.9637	IPI00137987	IPI00137987	yes	yes		3	4.3222E-20	162.77
DTYVN(de)ASQSIYGSSPR	1743.8064	IPI00122977	IPI00122977	yes	no		2	2.7296E-09	150.91
DVGSGTTN(de)NSQACAQFIEQYFHNSDITEFMR	3566.5467	IPI00130661	IPI00130661	yes	yes	3,4		1.0298E-41	167.28
DVIWFRPTIIN(de)DTGNYTCMIR	2584.2566	IPI00929886	IPI00929886	yes	no		3	3.8589E-16	153.86
DVIWFRPTIINDTGN(de)YTCMIR	2584.2566	IPI00929886	IPI00929886	yes	no		3	3.8589E-16	153.86
DVTVIEGEVATISCQVN(de)K	1960.9776	IPI00856723	IPI00856723	yes	no		2	5.9561E-75	225.68
DWCN(de)WTIISR	1349.6187	IPI00124933	IPI00124933	yes	no		2	0.010358	97.911
DWIWNQMHIDEEKN(de)ESIPHYVGK	2867.3337	IPI00138180	IPI00138180	yes	yes	3,4		8.512E-42	143.71
DWMPNN(de)HSVIIIDDFESPQK	2384.1107	IPI00221833	IPI00221833	yes	yes		3	1.1545E-09	142.26
DYCMYYNNN(de)WTR	1698.6555	IPI00121776	IPI00121776	yes	yes		2	0.0001926	142.38
DYRPSAGN(de)NSIYQDTVVFK	2173.044	IPI00322463	IPI00322463	yes	no	2,3		4.6531E-19	159.69
DYYIN(de)KTENEK	1415.6569	IPI00396840	IPI00396840	yes	no		2	0.014501	95.477
DYYPIN(de)ESIASIPIR	1749.8938	IPI00121627	IPI00121627	yes	yes	2,3		1.0491E-24	184.99
E(de)AETVRNGIYNITVIAIDADGR	2389.2238	IPI00138209	IPI00138209	yes	no		3	0.0056922	81.807
EACIQHYYVN(de)ASYIVWK	2143.0197	IPI00120155	IPI00120155	yes	yes		2	2.7598E-05	127.22
EADDYN(de)ITISEVK	1495.7042	IPI00277141	IPI00277141	yes	yes		2	4.4184E-07	156.24
EADN(de)HTAFIR	1172.5574	IPI00118333	IPI00118333	yes	yes		2	0.000294	122.69
EAETV(de)RNGIYNITVIAIDADGR	2389.2238	IPI00138209	IPI00138209	yes	no		3	0.0056922	81.807
EAFIPQGGSVQVN(de)CSSSCK	2053.9197	IPI00122973	IPI00122973	yes	no	2,3		1.1424E-19	162.09
EAFN(de)ETNQAIQTISR	1720.838	IPI00112614	IPI00112614	yes	yes	2,3		5.9618E-119	259.29
EAIGNASQI(de)FDSWIK	1677.8362	IPI00134585	IPI00134585	yes	yes	2,3		1.5764E-24	183.23
EAKEN(de)ASR	903.44101	IPI00124954	IPI00124954	yes	yes		2	1.1496E-05	118.5
EANIT(de)EDQIIFFPK	1663.8457	IPI00122122	IPI00122122	yes	no	2,3		6.4732E-55	210.37
EAPFGTNCN(de)ITITSR	1679.7937	IPI00129253	IPI00129253	yes	no		2	1.7435E-05	134.25
EAQYN(de)STFR	1114.5043	IPI00807983	IPI00807983	yes	no		2	0.0016233	133.23
EASHYSIHDIVISYN(de)TSDSTVFPGAVAK	3007.4563	IPI00222967	IPI00222967	yes	no	2,3,4		2.6934E-51	183.56
EASN(de)HSSGAGIVQINK	1610.8012	IPI00108844	IPI00108844	yes	no	2,3		5.4976E-68	173.28
EASN(de)HSSGAGIVQINKSNDKETVVGR	2696.3478	IPI00108844	IPI00108844	yes	no		3	0.0045543	73.672

EASNHSSGAGIVQIN(de)K	1610.8012	IPI00108844	IPI00108844	yes	no	2,3	5.4976E-68	173.28
EASNHSSGAGIVQIN(de)KSNDKETVVGR	2696.3478	IPI00108844	IPI00108844	yes	no	3	0.0045543	73.672
EATIVGEN(de)ETYPR	1477.7049	IPI00131143	IPI00131143	yes	no	2	3.1756E-10	163.99
EAYKN(de)ISIIYSQMQK	1814.9237	IPI00221706	IPI00221706	yes	no	2,3	9.9117E-17	169.64
ECHTIQN(de)YTIWR	1619.7515	IPI0012522C	IPI0012522C	yes	no	2,3	3.7762E-68	227.93
ECIGN(de)CSEPDDPTK	1620.6396	IPI00128358	IPI00128358	yes	no	2	0.0030274	98.281
ECSPPKAN(de)ITSDEI	1559.7137	IPI00466733	IPI00466733	yes	yes	2	7.1486E-23	182.89
ED(de)IIAIRQNFSNITVSTEDQVK	2519.2867	IPI00129677	IPI00129677	yes	no	2,3	1.6963E-71	214.59
EDIIA(de)IRQNFSNITVSTEDQVK	2519.2867	IPI00129677	IPI00129677	yes	no	2,3	1.6963E-71	214.59
EDRPFHQAVVN(de)DTQAFWHNK	2438.1516	IPI00406901	IPI00406901	yes	no	3	1.087E-25	174.87
EDSCQINY(de)SEGPCIGMQER	2271.9195	IPI00127352	IPI00127352	yes	yes	2,3	1.0214E-93	232.51
EDTVTITCEGTHNPGN(de)SSTQWFHNGR	2944.2794	IPI00129485	IPI00129485	yes	no	3	6.118E-68	199.99
EEEAIQIDGIN(de)ASQIR	1784.8905	IPI00129526	IPI00129526	yes	yes	2	3.9148E-67	159.81
EEN(de)ATIATYPEFGVIDIK	2008.9993	IPI00124221	IPI00124221	yes	no	2	6.6747E-61	214.46
EEQFN(de)STFR	1156.5149	IPI00475246	IPI00475246	yes	no	2	1.9361E-06	148.56
EETIIYDN(de)ATSSVADRK	1910.9222	IPI00223987	IPI00223987	yes	yes	2,3	5.8349E-05	126.71
EFEAYVN(de)ASGEHGIVVFSIGSMVSEIPEKK	3252.6013	IPI0041718C	IPI0041718C	yes	no	4	6.3616E-05	82.665
EFQHN(de)STMYYK	1446.6238	IPI00788443	IPI00788443	yes	no	2,3	0.0019871	103.91
EFVENSECIQCHPECIPQAMN(de)ITCTGR	3279.3876	IPI0012119C	IPI0012119C	yes	no	3,4	2.8804E-59	189.19
EGDN(de)ITIQCIGNGNPPPEEFMFYIPGQPEGIR	3588.6654	IPI00121378	IPI00121378	yes	no	3,4	2.7251E-07	88.708
EGFSAN(de)YSVIQSSISEDFK	2106.9746	IPI00123996	IPI00123996	yes	yes	2	1.1611E-41	189.48
EGKFDEVYDAIAGAHPN(de)ITVYK	2436.1961	IPI00111163	IPI00111163	yes	no	3,4	2.5432E-39	184.83
EGKFDEVYDAIAGAHPN(de)ITVYKK	2564.2911	IPI00111163	IPI00111163	yes	no	3,4	5.6377E-09	129.46
EGN(de)CSAQSGIAWQDCDFKDAEEAATGECTATVGK	3662.5196	IPI00114958	IPI00114958	yes	no	3,4	6.1134E-147	243.48
EGN(de)CSAQSGIAWQDCDFKDAEEAATGECTATVGKR	3818.6207	IPI00114958	IPI00114958	yes	no	3,4	1.7371E-26	135.84
EGN(de)FTSTFDK	1144.5037	IPI00153258	IPI00153258	yes	yes	2	0.060137	67.153
EGN(de)FTSTFDKK	1272.5986	IPI00153258	IPI00153258	yes	yes	2,3	0.000211	144.1
EGPRN(de)ISTCFSSGDIFAAHNISER	2664.2351	IPI00123428	IPI00123428	yes	no	3	4.1224E-20	151.89
EGPRNISTCFSSGDIFAAHN(de)ISER	2664.2351	IPI00123428	IPI00123428	yes	no	3	4.1224E-20	151.89
EGSRTDDEVVQREEEAIQI(de)DGINASQIR	3156.5283	IPI00129526	IPI00129526	yes	yes	3,4	1.4282E-21	137.38
EHSIFIDIHPVTGIPMN(de)CSVK	2393.1872	IPI01008227	IPI01008227	no	no	2,3,4	9.3061E-12	140.35
EI(de)QIAGFDPSQDSIGPKNNDSTINNSATTIIIGIQDCR	4101.9913	IPI00881745	IPI00881745	yes	no	4	2.1075E-10	88.814
EIAN(de)ATTKPEDRNDPMIIYNK	2432.2006	IPI00461861	IPI00461861	yes	yes	3	0.0026249	84.375
EIDDC(de)EQANKIGAINSTISNESK	2535.1759	IPI00187353	IPI00187353	yes	yes	3	4.7563E-08	121.44
EIDDCEQANK(de)IGAINSTISNESK	2535.1759	IPI00187353	IPI00187353	yes	yes	3	4.7563E-08	121.44
EIGAIIYN(de)CSNIAQDIEK	2050.0041	IPI00221418	IPI00221418	yes	no	2,3	2.7498E-48	202.34
EIGVVMYN(de)CSCIAR	1670.7579	IPI00130624	IPI00130624	yes	yes	2	1.0196E-42	198.87
EIHHIQEQN(de)VSNVFIDKEEFFIGSK	2986.4825	IPI00605187	IPI00605187	yes	no	3,4	3.881E-56	193.59
EIIMDFAN(de)SSAEITACMVR	2156.9904	IPI00221706	IPI00221706	yes	yes	3	1.1968E-79	227
EIIVDEIISN(de)SSTIASYK	1981.0256	IPI00173181	IPI00173181	yes	no	3	0.007128	75.376
EIIVHPN(de)YTR	1240.6564	IPI0011375C	IPI0011375C	yes	yes	2	2.9201E-08	157.38
EIIYSN(de)GSIIFQMITMK	1987.0159	IPI00108535	IPI00108535	no	no	2,3	7.6869E-21C	295.79
EIKGDGPFTVFVPHADI(de)ISNMSQDEIAR	3085.5179	IPI00987265	IPI00987265	yes	no	3	9.4499E-22	139.47
EIN(de)ISICPVSQTSER	1731.8461	IPI00381303	IPI00381303	yes	yes	2,3	1.8516E-08	154.51
EIPVIN(de)VSIGPFSTSQVVAR	2112.1579	IPI00515432	IPI00515432	yes	no	2,3	1.1965E-09	142.26
EIQAPIGQSFCCGN(de)ASIVISPAVHIDIISIR	3364.7272	IPI00989096	IPI00989096	yes	no	3,4	2.799E-59	184.76
EIQIAGFDPSQDSIGPKNNDSTIN(de)NSATTIIIGIQDCR	4101.9913	IPI00881745	IPI00881745	yes	no	4	2.1075E-10	88.814
EITD(de)TGKNAVNCTYKNEDDCVVR	2700.2119	IPI00266264	IPI00266264	yes	yes	3	5.9546E-08	120.9
EITGIRN(de)ISSQEGSTDVVAR	2131.0869	IPI00677395	IPI00677395	yes	no	2	6.968E-09	137.13
EIVGGIEIFITN(de)TSCR	1807.9138	IPI00453489	IPI00453489	yes	no	2	4.2326E-05	127.83
EIVHQSN(de)TSNIFFSPVSIATAFAMISIGSK	3195.6274	IPI00406302	IPI00406302	no	no	3	9.6821E-13	107.75
EIVSDPIYVEVQGIPYFIKQPESVN(de)VTR	3218.6863	IPI00121254	IPI00121254	yes	yes	3,4	0.0001434	89.946
EKEDRPFHQAV(de)VNDTQAFWHNK	2695.2891	IPI00406901	IPI00406901	yes	no	3	1.0526E-22	165.66
EKSCVAITDAFP(de)QNMSR	1952.9084	IPI00405742	IPI00405742	yes	no	2	0.0025712	94.384
EKV(de)ENGSETGPIPPEIQPIIEGEVK	2688.3858	IPI00136012	IPI00136012	yes	yes	2,3,4	4.3605E-80	213.05
EKVQSIQTIA(de)ANNSAIAK	1885.0269	IPI00129304	IPI00129304	yes	yes	3	2.1509E-11	148.7
EMDIVFHEHQDN(de)GTQDDSITIASVNTDFAFSIYK	3886.7632	IPI0013183C	IPI0013183C	yes	yes	4	0.0008918	54.311
EMGNTVMDIIRN(de)YTANATSSR	2343.0947	IPI00108098	IPI00108098	yes	no	3	0.0011175	87.24
EMGNTVMDIIRN(de)YTANATSSREEAWDYVQAQVK	3789.7727	IPI00108098	IPI00108098	yes	no	4	0.011749	50.857
EMGNTVMDIIRNYTAN(de)ATSSR	2343.0947	IPI00108098	IPI00108098	yes	no	3	0.0011175	87.24
EMGNTVMDIIRNYTAN(de)ATSSREEAWDYVQAQVK	3789.7727	IPI00108098	IPI00108098	yes	no	4	0.011749	50.857
EMIHN(de)ATFCIVPR	1586.7698	IPI0012553C	IPI0012553C	yes	no	2,3	0.0016233	105.03
EMN(de)CSETISQIQK	1566.7018	IPI00459134	IPI00459134	yes	no	2	0.0012276	107.99
EN(de)GTFSKYEGGVETFAHIIVIR	2466.2543	IPI0011813C	IPI0011813C	yes	yes	3,4	2.8913E-10C	231.77
EN(de)MTFGSTIVTNPK	1537.7446	IPI00466371	IPI01026704	no	no	2,3	1.6066E-07	153.79
ENGTFSKYEGGVETFAHIIVIR(de)K	2594.3493	IPI0011813C	IPI0011813C	yes	yes	3,4	0.0063038	74.776
ENIFGIQGAGGFQDREEEYYAEPGVTEAEPVATEDAN(de)STDSII	4633.0892	IPI00378224	IPI00378224	yes	yes	4	0.0086666	43.566
ENQN(de)HSYSIK	1218.5629	IPI00120245	IPI00120245	yes	no	2	0.022278	85.29
ENYAEIIDDGFIKN(de)ITAQICIDKK	2810.416	IPI00339885	IPI00339885	yes	no	3,4	3.3468E-20	150.94
EQEN(de)STIYISFTPK	1655.8043	IPI00894972	IPI00894972	yes	no	2	2.3279E-23	183.23
EQESMMN(de)ITIHIVK	1671.8324	IPI00990932	IPI00990932	yes	no	2,3	9.2935E-05	125.89
EQESMMN(de)ITIHIVKK	1799.9274	IPI00990932	IPI00990932	yes	no	3	0.033008	71.558
EQETCIAPEIEHGNY(de)STTQR	2362.0496	IPI00122117	IPI00122117	yes	yes	3	3.5868E-20	163.79
EQIN(de)ISIDHR	1223.6258	IPI00127407	IPI00127407	yes	no	2,3	0.0026899	108.47
EQN(de)YTCHVHHK	1451.6364	IPI00850057	IPI00850057	yes	no	2	0.0025784	102.4
EQN(de)YTCHVNHEGIPEPITIR	2406.1386	IPI0031298C	IPI0031298C	yes	no	3	1.455E-06	126.84
EQN(de)YTCHVYHEGIPEPITIR	2455.159	IPI00608044	IPI00608044	yes	no	2,3	1.9447E-20	165.66
EQN(de)YTCR	969.39743	IPI00109996	IPI00109996	yes	no	2	0.049796	88.948
EQSFQGSN(de)MTIFYR	1706.7723	IPI00453571	IPI00453571	yes	no	2	7.2206E-11	160.47
EQTVSDPFIVVSN(de)TSTFVPYEIK	2599.3058	IPI00785371	IPI00785371	yes	no	3	0.014645	60.272
EQVTVIN(de)R	957.52434	IPI00120155	IPI00120155	yes	yes	2	0.0092827	109.29
EREDVQAIN(de)ISVPYGPIPEDFQR	2671.3242	IPI01023131	IPI01023131	yes	no	3	0.0036868	79.143
ERTEVIIQGTVS(de)PNASAPDAVWEDYEFK	3150.5146	IPI00405437	IPI00405437	yes	no	3	1.375E-08	108.32
ESGIN(de)MTIPEENQPVVFNHIYNIK	2785.3745	IPI00403938	IPI00403938	yes	no	3	4.04E-27	162.42
ESGIQN(de)VSTCR	1249.5721	IPI01008227	IPI01008227	no	no	2	1.2131E-41	203.61
ESIIPVTIQCN(de)ITSSSHTIMYSYWTR	3086.4841	IPI00757771	IPI00757771	yes	no	3	0.0016528	68.369

ESNPITAQQTT(de)KIDAPTNIQFVNETDR	3030.4894	IPI00975193	IPI00975193	yes	no		3	3.288E-75	147.87
ESQTIGDQCVYN(de)STHIGFQR	2339.0601	IPI00118130	IPI00118130	yes	yes	2,3		2.407E-191	288.78
ESQTIGDQCVYN(de)STHIGFQRENGTFSK	3102.4101	IPI00118130	IPI00118130	yes	yes		3	4.7249E-13	121.33
ESQTIGDQCVYNSTHIGFQREN(de)GTFSK	3102.4101	IPI00118130	IPI00118130	yes	yes		3	4.7249E-13	121.33
ESSEHPWK(de)WTDNTEYNNTIPIRGEER	3187.4595	IPI00276430	IPI00276430	yes	yes	3,4		5.0836E-09	115.39
ESYIIISN(de)STMK	1384.6908	IPI00123925	IPI00123925	yes	no		2	0.0021704	102.87
ETAEYAAN(de)ITQAVFDYFEDYFAMEYAIPK	3409.5377	IPI00134585	IPI00134585	yes	no		3	0.0011728	63.372
ETFIIN(de)TTCVK	1324.6697	IPI00138199	IPI00138199	yes	yes		2	0.0004095	116.03
ETIVTIFDN(de)R	1206.6245	IPI00127238	IPI00127238	yes	no		2	0.0011551	113.63
ETMPEYN(de)ISITAR	1523.729	IPI00129613	IPI00129613	yes	yes		2	0.012937	80.312
ETVCIN(de)ATMCFHVK	1708.7735	IPI00466371	IPI01026704	no	no		2	0.0005004	113.72
ETYFN(de)ISK	1000.4866	IPI00153258	IPI00153258	yes	no		2	0.0072582	103.55
ETYFN(de)ISKK	1128.5815	IPI00153258	IPI00153258	yes	no		2	0.006552	127.76
ETYIETN(de)YTIK	1373.6715	IPI00120155	IPI00120155	yes	yes		2	0.0096114	87.667
EVHFFVNAS(de)DVDSVK	1691.8155	IPI00310049	IPI00310049	yes	yes	2,3		2.8034E-08	152.94
EVMN(de)FTAENIYK	1457.6861	IPI00170006	IPI00170006	yes	no		2	0.0009556	111.39
EVMNIIQPIN(de)VTK	1497.8225	IPI00126050	IPI00126050	yes	no		2	8.6857E-42	198.85
EVN(de)ITVIDRK	1185.6717	IPI00111385	IPI00111385	yes	no		2	0.0003539	133.07
EVNSQIDNNGCSTQEVN(de)ITEIQSK	2706.2403	IPI00123223	IPI00123223	yes	yes	2,3,4		1.5246E-142	251.12
EVNSQIDNNGCSTQEVN(de)ITEIQSKK	2834.3352	IPI00123223	IPI00123223	yes	yes		3	5.3449E-08	109.37
EVNVYMYIAN(de)GSAFNISNK	2133.0201	IPI00222967	IPI00222967	yes	no	2,3		1.8917E-42	197.14
EVNVYMYIAN(de)GSAFNISNKNISFWDAPIGSSYMCNK	4103.8856	IPI00222967	IPI00222967	yes	no		4	0.031493	47.159
EVNVYMYIANGSAFN(de)ISNK	2133.0201	IPI00222967	IPI00222967	yes	no	2,3		1.8917E-42	197.14
EVNVYMYIANGSAFN(de)ISNKNISFWDAPIGSSYMCNK	4103.8856	IPI00222967	IPI00222967	yes	no		4	0.031493	47.159
EVTEGGVVTVN(de)CSIQEEKPPIFFK	2706.3575	IPI00406901	IPI00406901	yes	no		3	1.2602E-15	139.81
EVTEGGVVTVN(de)CSIQEEKPPIFFKIEK	3076.5791	IPI00406901	IPI00406901	yes	no	3,4		9.3292E-05	93.804
EVTVIGVATAPTQVISNGIPVSN(de)FTYSPDNK	3217.6507	IPI00111960	IPI00111960	yes	yes	3,4		5.4441E-05	71.882
EWIPIN(de)HSVNSVVVR	1747.937	IPI00224752	IPI00224752	yes	yes	2,3		5.8567E-06	138.73
EWN(de)GTYHCIFR	1481.651	IPI00876541	IPI00876541	yes	no	2,3		0.003391	100.04
EYEGAVYPDN(de)TTDFQR	1903.8224	IPI00605187	IPI00605187	yes	no		2	2.028E-34	190.82
F(de)APPQPAEPWSFVKNTTSYPPMCSQDAVGGQVISEIFTNR	4453.1147	IPI00387289	IPI00387289	yes	yes		5	0.0094011	44.023
F(de)APPQPAEPWSFVKNTTSYPPMCSQDAVGGQVISEIFTNRK	4581.2097	IPI00387289	IPI00387289	yes	yes		7	0.019202	42.669
F(de)APPQPAEPWSSVKNATTYPPMCSQDAAR	3203.4805	IPI00128399	IPI00128399	yes	yes		3	0.0001289	86.791
F(de)DEFFSQGCAPGYEKNSTICDICIGPIK	3252.4566	IPI00139788	IPI00139788	yes	no	2,3,4,5		7.842E-77	208.72
F(de)EDGVIDPGYPRNISEGFSGIPDNVDAAFAIPAHR	3742.8016	IPI00129240	IPI00129240	yes	yes		4	0.013852	45.836
F(de)IRNGIASGSGNCSTGPNGDGSFHAWSIIEVK	3334.5789	IPI00322497	IPI00322497	yes	yes		4	0.0002821	67.496
FAEIN(de)GSAICSYNIKPSEYTITSK	2692.3054	IPI00421237	IPI00421237	yes	no	3,4		0.0078553	70.094
FAIIKDYR(de)PSAGNNSIYQDTVVFK	2745.4126	IPI00322463	IPI00322463	yes	no	3,4		3.9584E-13	134.32
FAIKNEDSN(de)FTIINNHDNTANITVK	2832.4042	IPI00138180	IPI00138180	yes	yes		3	1.833E-26	160.2
FAIKNEDSNFTIINNHDNTAN(de)ITVK	2832.4042	IPI00138180	IPI00138180	yes	yes		3	1.833E-26	160.2
FANEYPNI(de)TR	1223.5935	IPI00130573	IPI00130573	yes	yes		2	0.015815	91.961
FAPPEAPEPWSG(de)VRDGTSQPAICPQNVTMNMEGIK	3810.7804	IPI00320204	IPI00320204	yes	yes		4	0.0018248	54.732
FAQPQWHIIHIN(de)GTFSNETEPDTEPCVDGWVYDR	4057.833	IPI00226346	IPI00226346	yes	no		4	2.4984E-05	70.57
FAQPQWHIIHINGTFSN(de)ETEPDTEPCVDGWVYDR	4057.833	IPI00226346	IPI00226346	yes	no		4	2.4984E-05	70.57
FAVESIVPSSISIMHSPPDAQN(de)MSEVSISPMEISTFR	4019.9319	IPI00114044	IPI00114044	yes	yes	3,4,5		1.3771E-12	96.707
FCIIDVMSTGSSSVGN(de)ATR	2000.9296	IPI00749655	IPI00749655	yes	no	2,3		3.1806E-05	118.55
FCNIVPTEHCFIGN(de)GTEYR	2313.0307	IPI00227857	IPI00227857	yes	yes	2,3		5.8304E-14	154.26
FDAAGTSVHGGVPQN(de)GSICAHIPMIK	2663.2948	IPI00315535	IPI00315535	yes	yes	3,4		6.5515E-37	177.87
FDGEPCDQSIN(de)ITWFIK	2068.9564	IPI00625262	IPI00625262	yes	no	2,3		4.4653E-15	159.34
FDKGPFEKN(de)CSVQCAGMTIQTIPIK	2868.3972	IPI00320605	IPI00320605	yes	no		3	0.0014427	84.327
FFN(de)DSIVDPVDSEWFGFYR	2339.0535	IPI00331318	IPI00331318	yes	no		2	4.7296E-65	211.33
FFN(de)FSWR	1002.4712	IPI00396840	IPI00396840	yes	no	1,2		0.0010163	130.27
FFQAFTKN(de)GSGATIPGAGANVQTIR	2552.3136	IPI00223231	IPI00223231	yes	no		3	1.1085E-15	137.23
FGEFGN(de)YSIIVQHASSGANK	2125.0229	IPI00317488	IPI00317488	yes	no	2,3		2.62E-128	257.73
FGHSPIIN(de)ITGGISHASDVVIYHQHK	2826.4565	IPI00119063	IPI00119063	yes	yes	3,4,5		8.6317E-29	167.53
FGIN(de)GSYAPGPAGIPGVPGK	1854.9628	IPI00284816	IPI00284816	yes	no		2	1.4146E-10	117.26
FGN(de)ETFIIHIDNGR	1631.8056	IPI00170355	IPI00170355	yes	yes	2,3		6.4633E-07	148.68
FGTCSQICN(de)NTK	1428.6126	IPI00119063	IPI00119063	yes	yes	2,3		6.1482E-31	192.65
FGYIIHTDN(de)R	1234.6095	IPI00475209	IPI00475209	yes	no		2	0.0030545	107.24
FHSDINI(de)SESIIPAVIEK	2011.0626	IPI00831484	IPI00831484	yes	yes		3	9.7146E-07	136.01
FHVHQPVTQPFIQVTN(de)TTVK	2320.2328	IPI00658311	IPI00658311	yes	no	2,3,4		1.6093E-80	229.02
FHVNY(de)TQPIVAVK	1514.8245	IPI00123704	IPI00123704	yes	no	2,3		0.0028993	99.531
FI(de)EAGIYEVPIIITDSGNPPKSNISIIR	3055.6594	IPI00323134	IPI00323134	yes	no	3,4		5.532E-64	198.64
FIEPYN(de)DSIQAQK	1551.7569	IPI00123704	IPI00123704	yes	yes		2	0.0006051	128.31
FIEYVTSECHFYN(de)GTQHVR	2386.0801	IPI00474580	IPI00474580	yes	no	2,3		2.4014E-11	147.24
FIFKN(de)QTIEIHVIYGPR	2074.1364	IPI00122973	IPI00122973	yes	no	2,3,4		3.8252E-104	242.42
FIIKGPN(de)KIQCVDGNWTTIPVCIEEER	3215.6107	IPI00130010	IPI00130010	yes	no	3,4		1.2672E-29	161.53
FIN(de)ISSTGIR	1106.6084	IPI00131898	IPI00131898	yes	yes		2	0.0057342	103.34
FINYN(de)QTVSR	1240.62	IPI00226932	IPI00226932	yes	no		2	0.0026223	108.7
FIQ(de)KHINASNPSEPATIIFTAAR	2525.3391	IPI00421157	IPI00421157	yes	yes		3	0.0029843	81.356
FIQSAEFFNY(de)TVR	1620.7936	IPI00127406	IPI00127406	yes	yes	2,3		8.0802E-68	220.97
FIRNGIASG(de)SGNCSTGPNGDGSFHAWSIIEVK	3334.5789	IPI00322497	IPI00322497	yes	yes		4	0.0002821	67.496
FITDVERN(de)ETAIYHVEAFK	2281.1379	IPI00466733	IPI00466733	yes	no		3	1.963E-41	193.32
FIWSEPQN(de)CSATK	1566.7137	IPI00118987	IPI00118987	yes	no		2	0.0004286	133.15
FMFFVQFPHN(de)ATQTMNIR	2228.0659	IPI00136513	IPI00136513	yes	yes		3	0.0020118	84.14
FMGIPTKDDNIEHYKN(de)STVMAR	2566.2308	IPI00123925	IPI00123925	yes	no		3	0.0088704	73.688
FN(de)ETIHR	915.45626	IPI00471081	IPI00471081	yes	yes		2	0.012023	114.5
FN(de)ETRDTITSIVSDINVR	2079.0596	IPI00990932	IPI00990932	yes	yes		3	1.0561E-11	153.22
FN(de)HTDRTIETIK	1473.7576	IPI00378224	IPI00378224	yes	yes	2,3		0.0021052	120.57
FN(de)ITETPEADIHQGFGNIIQSISQPEDQDQINIGNAMFIEK	4601.202	IPI00131830	IPI00131830	yes	no		4	3.3988E-05	57.325
FNSTE(de)YQVVTR	1342.6517	IPI00119063	IPI00119063	yes	no		2	1.6869E-10	166.48
FPMMIPQEIFEIQFN(de)ISIYQDHQAIFQR	3482.7155	IPI00119809	IPI00119809	yes	yes		4	6.9572E-05	85.35
FQIIN(de)FSSSEIK	1411.7347	IPI00856723	IPI00856723	yes	no		2	0.0006073	132.97
FQISPQIQFSPEEVIGMVIN(de)YSR	2681.3523	IPI00123342	IPI00123342	yes	no	2,3		1.9578E-116	219.22
FQN(de)FSMATDQR	1343.5928	IPI00128178	IPI00128178	yes	no		2	0.00123	106.49
FQN(de)YTICESFSK	1522.6762	IPI00118291	IPI00118291	yes	no		2	0.000861	125.03

FQSSAVMAIQEASEAYIVGIFEDTN(de)ICAIHAK	3512.6956	IPI00282848	IPI00282848	yes	no		3	1.003E-70	192.85
FSATEVPEKGAGEVSP(de)AEHSSKPTNISAK	2954.4621	IPI00230718	IPI00230718	yes	no		3	1.2109E-75	201.29
FSEIIVNN(de)ATEEIIVK	1817.9775	IPI00321190	IPI00321190	yes	no		2	2.4203E-57	209.31
FSHN(de)GTCAAEGK	1277.5459	IPI00320675	IPI00320675	yes	yes	2,3		1.6756E-06	154.1
FSIMN(de)QSIISIPGSPFISR	2093.0979	IPI00337976	IPI00337976	yes	no		3	0.010013	69.485
FSMSDTYDIQDVIADVGIKDIFTN(de)QSDFADTTK	3699.7138	IPI00116105	IPI00116105	yes	no	3,4		8.7393E-59	179.86
FSPPVVN(de)VTWIR	1413.7769	IPI00110808	IPI00110808	yes	no		3	0.0008523	127.87
FSVN(de)QTTIITHEK	1516.7886	IPI00848693	IPI00848693	yes	yes	2,3		9.0018E-42	198.85
FSWNN(de)ITNSIDIANISADFQGRPVDDPTGAFANGSITFK	4200.0189	IPI00459432	IPI00459432	yes	yes		4	0.012822	47.044
FSWNNITNSIDIAN(de)ISADFQGRPVDDPTGAFANGSITFK	4200.0189	IPI00459432	IPI00459432	yes	yes		4	0.012822	47.044
FSWNNITNSIDIANISADFQGRPVDDPTGAFAN(de)GSITFK	4200.0189	IPI00459432	IPI00459432	yes	yes		4	0.012822	47.044
FTAPDTIFAN(de)GSVYPPNEGFCPCR	2716.205	IPI01008227	IPI01008227	no	no	2,3,4		1.0425E-35	176.65
FTAPDTIFAN(de)GSVYPPNEGFCPCR	2716.205	IPI01008227	IPI01008227	no	no	2,3,4		1.0425E-35	176.65
FTCN(de)QTTDVIIIHSK	1775.8876	IPI00319509	IPI00319509	yes	yes	2,3		6.1226E-44	201.3
FTCN(de)QTTDVIIIHSKK	1903.9826	IPI00319509	IPI00319509	yes	yes		3	0.0050139	87.519
FTECCHEERPIN(de)TSAIK	2090.9514	IPI00876233	IPI00876233	yes	no	2,3		0.017429	78.505
FTFTSHTPGDHQICIHSN(de)STR	2442.1135	IPI00153468	IPI00153468	yes	no	2,3,4		5.4064E-31	175.56
FTFTSHTPGEHQICIHSN(de)STK	2428.123	IPI00473680	IPI00473680	yes	no	2,3,4		4.4812E-203	222.7
FTQDAYSAVVKEN(de)STEAR	2014.9596	IPI00623114	IPI00623114	yes	no	2,3		8.8565E-13	157.22
FVHVN(de)TSAIIK	1227.6976	IPI00153632	IPI00153632	yes	yes	2,3		0.0025784	102.4
FVKN(de)ITTWNEMKPGFYHGHISYIDFAK	3242.6012	IPI00131021	IPI00131021	yes	yes		4	0.0035649	67.194
FVPNSNMN(de)FTGQAYSGR	1888.8526	IPI00108535	IPI00108535	no	no	2,3		4.4516E-37	194.23
FYPEDIQIIWIENGN(de)VSR	2192.0902	IPI00129158	IPI00129158	no	no		3	0.0002007	112.8
GAAAPSAPHWN(de)ETAEK	1635.7641	IPI00648288	IPI00648288	yes	no		2	0.0008044	104.94
GAFFPIKEDN(de)WSIPNR	1889.9424	IPI00135560	IPI00135560	yes	no	2,3		0.0001168	128.22
GAFISN(de)FTMTVNGMTFTSSIK	2253.081	IPI00970438	IPI00970438	yes	no	2,3		2.4141E-23	166.61
GAFVSKNPCN(de)ITR	1462.7351	IPI00307966	IPI00307966	yes	yes	2,3		0.0006328	139.48
GAFVSKNPCN(de)ITREDYAPIVK	2378.2053	IPI00307966	IPI00307966	yes	yes	2,3,4		2.2617E-30	174.87
GAGEVSPAEHSSKPTN(de)ISAK	1965.9756	IPI00230718	IPI00230718	yes	no	2,3,4		1.1409E-32	181.64
GAIDIMIQVN(de)MTPGHSSAPPK	2163.0816	IPI00224073	IPI00224073	yes	yes	2,3,4		1.4529E-49	198.14
GAIQIPTVSFSHEESN(de)TTAIAEFGEYIR	3066.4934	IPI00224073	IPI00224073	yes	yes	3,4		2.7165E-41	171.84
GAIQIPTVSFSHEESN(de)TTAIAEFGEYIRK	3194.5884	IPI00224073	IPI00224073	yes	yes	3,4		4.8253E-07	105.32
GAMITHQNIIN(de)DCSGFIK	2017.9714	IPI00112549	IPI00112549	yes	no	2,3		4.4783E-37	190.19
GCADYCNQ(de)TITKR	1585.6977	IPI00987265	IPI00987265	yes	no		3	0.0063486	98.353
GCGSIPGCPGTAGFHSN(de)QTFHFIK	2576.1689	IPI00118475	IPI00118475	yes	yes		3	0.0014632	74.325
GCIWEASN(de)TTR	1293.5772	IPI00848693	IPI00848693	yes	yes		2	7.8055E-42	205.56
GCKDNA(de)TDSVPIR	1431.6776	IPI00119063	IPI00119063	yes	yes	2,3		3.3051E-06	154.1
GDDIYTNV(de)TVSIVEAIVGFEMDITHIDGHK	3287.602	IPI00320241	IPI00320241	yes	yes		4	8.8051E-34	160.84
GDEKENITA(de)EAIDISIK	1844.9367	IPI00124725	IPI00124725	yes	no	2,3		3.6579E-15	165.45
GDGPFTVFVPHADIISN(de)MSQDEIAR	2715.2963	IPI00987265	IPI00987265	yes	no		3	4.0363E-35	168.81
GDHHQISHYN(de)ITGVR	1732.8394	IPI00338209	IPI00338209	yes	no		2	0.0004449	114.63
GDN(de)QSPIEIHTK	1337.6575	IPI00221890	IPI00221890	yes	yes	2,3		1.5965E-41	199.31
GDPSIISVN(de)GTDFTFR	1724.837	IPI00463492	IPI00463492	yes	yes	2,3		1.0599E-120	260.24
GDSGGAIVFIDN(de)ETQR	1677.7958	IPI00467068	IPI00467068	yes	no		2	3.6758E-13	162.59
GDTHTQIIEGIQFN(de)ITQTSEADIHK	2795.3726	IPI00406302	IPI00406302	no	no	3,4		1.9372E-125	244.31
GDTVARPIIHEFYDDN(de)NTWGIDR	2703.2677	IPI00848693	IPI00848693	yes	yes		3	1.3028E-27	134.32
GECYYTN(de)GTQR	1347.5514	IPI00110910	IPI00110910	yes	no		2	0.01085	85.554
GEIN(de)STIFSSR	1209.599	IPI00460063	IPI00460063	yes	no		2	0.0005828	125.03
GEIN(de)STIFSSRPK	1434.7467	IPI00460063	IPI00460063	yes	no	2,3		4.4346E-82	230.96
GEIQSEN(de)SSITISSSNR	1807.8548	IPI00466371	IPI01026704	no	no	2,3		3.8437E-90	239.75
GENPSQYGITAFNHPIN(de)ITK	2200.0913	IPI00112614	IPI00112614	yes	yes		3	1.6605E-05	116.98
GETASIICN(de)ISVR	1418.7188	IPI00321348	IPI00321348	yes	no		2	0.0001093	143.46
GFDTYFGYIIGSEDYYTHEACAPIESIN(de)GTR	3545.5722	IPI00652358	IPI00652358	yes	no		3	4.3338E-06	80.359
GFFGPDCTQCPGGFSNPCYGKGN(de)CSDGVR	3197.2848	IPI00987265	IPI00987265	yes	no		3	8.7463E-06	96.303
GFFVQPTVFSN(de)VTDEMR	1972.9353	IPI00336362	IPI00626662	no	no	2,3		7.0085E-48	207.4
GFGVAFVGN(de)YTGSIPNEAAINTVR	2453.2339	IPI00165807	IPI00165807	yes	no	2,3		1.3161E-56	195.46
GFIIAN(de)ATYK	1096.5917	IPI00124497	IPI00124497	yes	no		2	0.0016013	112.13
GFN(de)ATYHVR	1063.5199	IPI00411025	IPI00411025	yes	yes		2	0.0033778	123.63
GFPQIIGAAGN(de)VSAGSVIIR	1926.0687	IPI00754853	IPI00754853	yes	no	2,3		4.8535E-55	156.11
GFQWVTGDN(de)HTSYSR	1753.7808	IPI00129423	IPI00129423	yes	yes		2	4.4324E-25	187
GFTQIQTIIIPQDVPCPGGSNAWDN(de)VTSFK	3289.6078	IPI00471273	IPI00471273	yes	no		4	0.0077594	51.586
GFTQIQTIIIPQDVPCPGGSNAWDN(de)VTSFKDK	3532.7297	IPI00471273	IPI00471273	yes	no		3	3.8636E-06	83.788
GGIN(de)ITAVTVTAENDHTVAFIGTSDGR	2715.3464	IPI00405742	IPI00405742	yes	no		3	5.4781E-06	98.121
GGITINANISG(de)DAFIK	1589.8413	IPI00134585	IPI00134585	yes	no		2	0.0007812	114.27
GGNSNGAICHFPFIYNNRN(de)YTDCTSEGR	3220.3839	IPI00975193	IPI00975193	yes	no		3	1.7793E-08	110.9
GGQEPCVNEGTCVTYHN(de)GTGFCR	2599.0638	IPI00467908	IPI00467908	yes	no		3	0.0001632	93.269
GHAHIAAIVNHDSYN(de)FSHR	2145.0253	IPI00133365	IPI00133365	yes	no	3,4		3.7821E-09	140.22
GHAIGISCQSEN(de)GTAPITYHIMK	2484.189	IPI00406901	IPI00406901	yes	no	3,4		1.3181E-31	175.56
GHFYYN(de)ISDVR	1369.6415	IPI00135560	IPI00135560	yes	no		2	0.0004575	119.76
GHICN(de)QTQNIQSSK	1613.758	IPI00987886	IPI00987886	yes	yes	2,3		0.0023395	101.75
GHIIIAQVATNPQGITGTGN(de)TTSEMDPSHR	3102.5153	IPI00321375	IPI00321375	yes	no	3,4		2.092E-52	186.96
GHIIIAQVATNPQGITGTGN(de)TTSEMDPSHRK	3230.6102	IPI00321375	IPI00321375	yes	no	3,4		9.1124E-16	120.33
GHTDRCDEAQAIQVWN(de)DTHPEVISQKPFDK	3520.643	IPI00108003	IPI00108003	yes	no		4	0.0041575	61.109
GHVDPAN(de)DTFDIDPR	1667.754	IPI00453501	IPI00453501	yes	no	2,3		0.0019923	99.011
GIAEVTEN(de)VTEGGVTK	1602.8101	IPI00130271	IPI00130271	yes	no		2	1.8824E-139	269.92
GIAN(de)ISNFIR	1103.6087	IPI00129158	IPI00129158	no	no	1,2		6.9245E-07	152.7
GICPIFCHTN(de)GTPIGIGAR	2040.0033	IPI00420791	IPI00420791	yes	no		3	0.0003773	93.754
GIFPDGSHEISGN(de)TSITPDK	2070.9858	IPI00894972	IPI00894972	yes	no		3	6.9997E-06	123.14
GIIGHNN(de)KSSASFR	1486.7641	IPI00162870	IPI00162870	yes	yes		2	0.0001498	139.48
GIIGISN(de)ATIYWHIPDTAYPGIYR	2690.3857	IPI00458077	IPI00458077	yes	yes		3	2.9295E-08	106.86
GIIN(de)ATISVAEINHPVTTYK	2140.1528	IPI00130573	IPI00130573	yes	yes	2,3		1.05E-215	293.01
GIMIIIN(de)DTQHFSNNVK	1942.9935	IPI00312711	IPI00312711	yes	no	2,3		4.7974E-74	221.85
GIMIIIN(de)DTQHFSNNVKGEIGQFYR	2893.4545	IPI00312711	IPI00312711	yes	no		3	3.7843E-16	143.57
GIMVGN(de)GSVIGVVQAVDAETGK	2100.0885	IPI00342158	IPI00342158	yes	no	2,3		7.8924E-101	233.28
GINIT(de)EDTYKPR	1405.7201	IPI00119809	IPI00119809	yes	yes	2,3		7.458E-42	207.47
GIRGPN(de)FTSPASITFTTGIKPPQDIEAK	2942.5502	IPI00458413	IPI00458413	yes	no	3,4		0.0011037	73.546

GISYQIIGN(de)HSK	1315.6884	IPI00623114	IPI00623114	yes	no	3	0.0065839	90.653
GIVIGPIHKNQKDEIN(de)ETDIK	2360.27	IPI00930882	IPI00930882	yes	no	3	0.0004866	93.495
GIVISEDN(de)HTCQVPVICK	2068.0081	IPI00453489	IPI00453489	yes	no	3	0.014718	68.919
GIVMNYPHITN(de)ITTIGQSVEYR	2505.2686	IPI00130573	IPI00130573	yes	yes	3	1.4261E-48	190.23
GIVSGGVYNSHVGCIPYTIPPCEHHVNG(de)SRPPCTGEGDTPR	4430.0379	IPI00113517	IPI00113517	yes	yes	4,5	5.7082E-05	60.645
GIVTVKPCYN(de)YCSNIMR	2073.9798	IPI00312407	IPI00312407	yes	yes	3	0.00112	108.71
GIWKIPFSPENTREEDFYVN(de)ETSTVK	3085.5033	IPI00116105	IPI00116105	yes	yes	3,4	3.9498E-13	128.67
GIYHIN(de)ITVGGIPFK	1627.9086	IPI00115536	IPI00115536	yes	yes	3	3.3234E-05	130.1
GIYHIN(de)ITVGGIPFKESEIVQPINPR	2890.5705	IPI00115536	IPI00115536	yes	yes	3	0.0013248	78.975
GKNN(de)QTECFNHVR	1602.7321	IPI00124666	IPI00124666	yes	no	2,3	9.7367E-06	147.33
GKSN(de)QTECFNFIR	1599.7464	IPI00890869	IPI00890869	yes	yes	2	0.0018062	127.71
GMNYN(de)SSVVK	1097.5175	IPI00416285	IPI00416285	yes	yes	2	0.0030098	107.03
GMVIDENSNN(de)ITGAVISVTGINHDVTSGEHGDYFR	3717.7329	IPI00128260	IPI00128260	yes	yes	3,4	3.3096E-12	97.877
GN(de)HSIFGIEVATIGQGPDCPSVNER	2653.2555	IPI00459432	IPI00459432	yes	yes	3,4	2.8076E-16	139.95
GN(de)ISTEKFVEEIK	1492.7773	IPI00466371	IPI01026704	no	no	2	0.0015656	119.25
GN(de)ITEYQCHQYITK	1753.8094	IPI00122399	IPI00122399	yes	no	2,3	7.1933E-136	267.47
GN(de)MTWYAAGK	1097.4964	IPI00120953	IPI00120953	yes	yes	2	0.0002581	126.67
GN(de)QSSQWIYEAMAK	1611.7351	IPI00137792	IPI00137792	yes	yes	2	0.0005308	113.47
GN(de)SSIYPVVSPSIQQIVIEK	2157.1681	IPI00985989	IPI00985989	yes	no	2,3	6.1068E-07	132.74
GN(de)TTAIDKEIAR	1287.6783	IPI00108535	IPI00108535	no	no	2,3	1.3881E-21	181.67
GNYDFVEAMIVNN(de)HTSIDVER	2422.1223	IPI00403586	IPI00403586	yes	yes	3	1.2175E-48	194.27
GNYGWQAGN(de)HSAFWGMTIDEGIR	2566.1448	IPI00115458	IPI00115458	yes	no	2,3	3.5759E-31	171.07
GPCSHICIINYN(de)R	1602.7395	IPI00119063	IPI00119063	yes	yes	2,3	0.0005225	117.98
GPDGTEGISSTVN(de)GSTDPSAVTDIR	2432.1303	IPI00469426	IPI00469426	yes	no	2,3	1.137E-125	246.77
GPDISN(de)CTSHWVNQIAQK	2053.964	IPI00876558	IPI00876558	yes	no	3	7.2136E-27	139.49
GPECSQNY(de)TAPTGVIK	1720.809	IPI00123996	IPI00123996	yes	yes	2	0.025307	65.477
GPGIKPN(de)QTSK	1125.6142	IPI00221426	IPI00221426	yes	yes	2	5.6723E-06	152.7
GPGSKN(de)CTGCSAGIIIDMDDNR	2337.0148	IPI00138199	IPI00138199	yes	yes	3	1.6572E-07	118.52
GPIANPIWN(de)VTGFTGR	1698.8842	IPI00123040	IPI00123040	yes	no	3	0.0005276	114.31
GPICIDCTDGFFSIQRN(de)ETHSICSACDESCK	3663.5157	IPI00111286	IPI00111286	yes	yes	3,4	2.1993E-05	75.868
GPIDIN(de)MSEIAMDDIHEIFSRDPAIK	2926.4205	IPI00125747	IPI00125747	yes	yes	4	0.015148	61.345
GPN(de)VTVITGITR	1226.6983	IPI00877260	IPI00877260	yes	no	2	0.013534	82.069
GPNKIQC(de)VDGNWTTIPVCIEEER	2714.2792	IPI00130010	IPI00130010	yes	no	3	1.3917E-07	116.73
GPVKMPSQASAGNVYPQ(de)PIINSSMCIEDSR	3232.5315	IPI00224752	IPI00224752	yes	yes	4	0.0032187	60.621
GQTGIGFIYASGIGVN(de)SSQAK	2054.0433	IPI00131143	IPI00131143	yes	no	2,3	1.1048E-100	234.39
GQTQNYST(de)IIIEEASER	1937.933	IPI00124666	IPI00124666	yes	no	2,3	2.3424E-60	215.51
GRADECAIPYIGATCYCDIFCN(de)R	2781.1768	IPI00115458	IPI00115458	yes	no	3	5.7787E-175	277.6
GRHN(de)CSEPIDMEDISSGIGVTR	2429.1064	IPI00115892	IPI00115892	yes	yes	3	3.754E-22	158.44
GSAIPHPSN(de)ATWEIK	1606.8104	IPI00119299	IPI00119299	yes	no	2,3	3.0774E-24	178.23
GSEVEDEDIEIFN(de)TSVQIRPPSTAPGPETAAFIER	3800.8381	IPI00381357	IPI00381357	yes	no	3,4	4.5698E-68	182.54
GSEVEDEDIEIFN(de)TSVQIRPPSTAPGPETAAFIERIEMEQAQK	4758.2971	IPI00381357	IPI00381357	yes	no	5	4.8446E-05	59.401
GSIN(de)VSYNIGPGFTGSEYSR	2103.9861	IPI00113042	IPI00113042	yes	no	2,3	4.1971E-110	242.22
GSIQSANDISSQTQGFIQHSMDN(de)ISAQIQTVR	3460.6641	IPI00330594	IPI00330594	yes	yes	3,4	4.6516E-59	179.81
GSN(de)FTAICVIK	1208.6223	IPI00120155	IPI00120155	yes	yes	2	5.7312E-15	171.32
GSNFN(de)ISK	865.42938	IPI00113057	IPI00113057	yes	yes	2	0.0043509	132.88
GSNFN(de)ISKTDNIEECQK	1982.9004	IPI00113057	IPI00113057	yes	yes	3	0.011785	82.102
GSPGN(de)ASQGSIHIHSPQIAIQADPR	2537.2735	IPI00114044	IPI00114044	yes	yes	2,3	1.1593E-09	115.88
GSTQYIENIGFN(de)MSK	1687.7876	IPI00116105	IPI00116105	yes	yes	2,3	6.4796E-44	200.55
GTAGNAIMDGASQIVGEN(de)R	1859.8796	IPI00279079	IPI00279079	yes	yes	2,3	1.4407E-167	271.92
GTCEQGPSIVTPPKDIWN(de)VTGAK	2454.2213	IPI00652107	IPI00652107	yes	no	3	6.3602E-08	119.38
GTDN(de)ITVR	874.45084	IPI00652675	IPI00652675	yes	no	2	0.0089756	111.61
GTDPSISPSPAATPPAPGAEISYIN(de)HTFR	2950.4461	IPI00762091	IPI00762091	yes	yes	3	1.6374E-19	130.3
GTFTDCAIANM(de)TQQIR	1825.8451	IPI00621027	IPI00621027	yes	no	2,3	1.8952E-138	260.85
GTIIGIDIQN(de)CSIK	1530.8076	IPI00471273	IPI00471273	yes	no	2	3.8549E-23	179.79
GTTN(de)TTSAGVPCQR	1448.6678	IPI00330542	IPI00330542	yes	no	2	0.0048641	88.561
GTTVITSISSVIHDS(de)KEFPNPEMFDPGHFINGNGNFK	4032.9316	IPI00134503	IPI00134503	yes	no	4	7.0467E-05	64.213
GTTVITSISSVIHDSKE(de)FPNPEMFDPGHFINGNGNFK	4032.9316	IPI00134503	IPI00134503	yes	no	4	7.0467E-05	64.213
GTYTIQGIIGCEIASDN(de)SSVPTAVFAINGEEFMK	3618.7222	IPI00322497	IPI00322497	yes	yes	4	1.2524E-05	71.117
GV(de)IMVGNETTYEDGHGARKNITDIVEGAK	3073.5139	IPI00649186	IPI00649186	yes	no	3	6.4525E-19	128.38
GVAVTN(de)TSQIGFR	1348.7099	IPI00471089	IPI00471089	yes	no	2	2.8113E-31	194.12
GVDIDAPYYNYIISFTVPDIDN(de)VTVIDYDAR	3535.7035	IPI00119063	IPI00119063	yes	yes	4	0.0027928	53.679
GVDIN(de)ATGEWK	1188.5775	IPI00848693	IPI00848693	yes	yes	2	0.0064791	93.011
GVDSCHGDSGGAFAFQVPN(de)VTVPK	2445.1383	IPI00653675	IPI00653675	yes	no	3	3.9893E-13	128.05
GVENDVFIRYPNN(de)GSIVWGK	2263.1386	IPI00848693	IPI00848693	yes	yes	2,3	3.249E-11	122.44
GVFITNE(de)TGQPIIGK	1572.8512	IPI00111960	IPI00111960	yes	no	2,3	2.8998E-137	260.96
GVHFSPAASGITTSTVQVQGIEPYAN(de)YTFTVK	3369.6881	IPI00120222	IPI00120222	yes	yes	3	1.9119E-11	108.5
GVIMVGN(de)ETTYEDGHGAR	1904.8687	IPI00649186	IPI00649186	yes	no	2,3	1.4515E-37	192.16
GVIMVGN(de)ETTYEDGHGARKNITDIVEGAK	3073.5139	IPI00649186	IPI00649186	yes	no	3	6.4525E-19	128.38
GVIPGEDWTVFQSN(de)HSTYEPVIIAK	2786.3915	IPI00404189	IPI00404189	yes	no	3,4	1.505E-05	98.269
GVN(de)ETIIVNEIK	1327.7347	IPI00338018	IPI00338018	yes	no	2	2.5294E-05	146.77
GVN(de)VTMPSQPGVPPISSTQIQIDPAIQEFQIVDISR	3860.9982	IPI00515360	IPI00515360	yes	no	3,4	9.3196E-13	103.14
GVN(de)VTMPSQPGVPPISSTQIQIDPAIQEFQIVDISRR	4017.0994	IPI00515360	IPI00515360	yes	no	4,5	1.3436E-08	82.126
GVNVITVN(de)ATDADSK	1502.7577	IPI00623114	IPI00623114	yes	no	2	1.5254E-17	174.39
GVQIIVFPEDGIHGFN(de)FTR	2145.1007	IPI00321375	IPI00321375	yes	no	2,3,4	1.4626E-19	160.47
GVSSSEN(de)FTSPVMEFWER	2087.9259	IPI00466652	IPI00466652	yes	no	2,3	1.6978E-89	235.24
GVSSSEN(de)FTSPVMEFWERR	2244.027	IPI00466652	IPI00466652	yes	no	3	0.0020701	87.647
GVTHIN(de)ISGIK	1137.6506	IPI00119063	IPI00119063	yes	yes	2,3	1.1819E-09	158.04
GVTHIN(de)ISGIKMPR	1521.845	IPI00119063	IPI00119063	yes	yes	3	0.012462	90.614
GVTNDIISVQGNTGPSWIN(de)KTEK	2457.25	IPI00344686	IPI00344686	yes	yes	3	2.6561E-06	105.77
GVTSVSQIFHSPDIAIRDTYVN(de)ASQSIYGSSPR	3551.7645	IPI00122977	IPI00122977	yes	no	3,4	7.6098E-59	181.24
GVVTDEQGIPIAN(de)ATISVSGINHGVK	2575.3606	IPI00875870	IPI00875870	yes	no	3	4.4446E-06	102.66
GWIN(de)GSIVGFYK	1339.6925	IPI00134585	IPI00134585	yes	no	2	1.5091E-54	218.06
GWKENPVEFDSIFN(de)ESK	2024.948	IPI00129070	IPI00129070	yes	no	2,3	2.0153E-20	173.87
GYIITIN(de)FTK	1168.6492	IPI00469218	IPI00469218	yes	yes	2	1.0374E-97	240.1
GYIITIN(de)FTKNTTR	1640.8886	IPI00469218	IPI00469218	yes	yes	2,3	4.4704E-83	235.22
GYIITINFTKN(de)TTR	1640.8886	IPI00469218	IPI00469218	yes	yes	2,3	4.4704E-83	235.22

GYITSFEMFN(de)STFK	1670.765	IPI00262024	IPI00262024	yes	no	2,3	3.972E-136	268.93
GYPCGGN(de)KTASSVIEITCAK	2111.998	IPI00308971	IPI00308971	yes	yes	3	0.0012309	91.401
GYPGDEGGPGERGPPGVN(de)GTQGFQGCPGQR	2984.322	IPI00830749	IPI00830749	yes	yes	3	0.0006392	68.97
HAEISFVFITDGVTGN(de)DSIEESVHSMR	2976.3924	IPI00621027	IPI00621027	yes	yes	3	1.1399E-60	151.01
HAIEYFNN(de)NTGHSHIFAIR	2240.0875	IPI00400202	IPI00400202	yes	no	3	0.0010065	88.19
HAN(de)CSQVGTVVTCTCIPDYEGDGWSCR	3128.2845	IPI00987265	IPI00987265	yes	no	3	0.0001103	86.08
HAN(de)WTITPIK	1179.64	IPI00317356	IPI00317356	yes	yes	2,3	7.3331E-11	166.14
HDPN(de)MTCDYVIK	1491.6486	IPI00119299	IPI00119299	yes	no	2	0.0005942	132.79
HDYIIIPEGAITN(de)TTR	1812.937	IPI00121421	IPI00121421	yes	no	2	0.0005777	112.5
HEEGHMIN(de)CTCFGQGR	1931.7825	IPI00975193	IPI00975193	yes	no	2,3	0.00225	88.561
HEN(de)NTKDNSIQHEFSITR	2169.0199	IPI00109727	IPI00109727	yes	yes	2,3	2.3317E-08	142.97
HF(de)IIDSKSNVTRPSEFNYIWIAPIPFIK	3331.7758	IPI00319509	IPI00319509	yes	no	3	0.060377	49.638
HFFNVSDEIAIVT(de)IVK	1830.988	IPI00466371	IPI01026704	no	no	2,3	4.3044E-45	200.94
HFKGQTQN(de)YSTIIIEEASER	2350.1553	IPI00124666	IPI00124666	yes	no	2,3,4	1.663E-32	178.51
HFN(de)DSGACVPR	1258.5513	IPI00468814	IPI00468814	yes	yes	2	0.005258	95.094
HGFYCYIIGSTISTFTDAN(de)HTCTNEK	3036.3382	IPI00126186	IPI00126186	yes	yes	3	2.3854E-36	177.09
HGHFTGFN(de)GSTIR	1429.6851	IPI00117986	IPI00117986	yes	yes	2	0.017862	76.588
HGQIIKN(de)FTIIQGPPGPR	1972.1007	IPI00129304	IPI00129304	yes	yes	3,4	1.1875E-05	132.81
HIIEN(de)ATASVSEAER	1625.8009	IPI00396840	IPI00396840	yes	no	2,3	1.0413E-11	157.74
HIIEN(de)ATASVSEAERK	1753.8959	IPI00396840	IPI00396840	yes	no	2,3	2.6277E-25	182.02
HIN(de)ASNPSEPATIIFTAAR	2009.033	IPI00421157	IPI00421157	yes	yes	2,3	1.2113E-13	150.69
HIN(de)ITTISVAGGFR	1484.81	IPI00850413	IPI00850413	yes	yes	2,3	0.0082814	82.925
HIN(de)SSIPRPQPAIIIITAAQDAAEVIK	2865.6076	IPI00762273	IPI00762273	yes	no	3,4	2.9675E-23	148.95
HISFGEDYPGIVNPIDHTN(de)VTAPQASMMFQYFVK	3852.828	IPI00626555	IPI00626555	yes	no	3,4	2.6243E-12	112.26
HITDISISGTIFVPQNSGIPKN(de)K	2465.3278	IPI00467600	IPI00467600	yes	yes	3	4.5173E-05	100.39
HIVFWN(de)SSNPK	1327.6673	IPI00130752	IPI00130752	yes	no	2	0.0004371	118.9
HIVN(de)ISIYAFNK	1417.7718	IPI00626793	IPI00626793	yes	no	2	0.0021797	107.09
HIVNISIYAFN(de)K	1417.7718	IPI00626793	IPI00626793	yes	no	2	0.0021797	107.09
HIWSIEISNKPN(de)ISEPEEPK	2346.1856	IPI00130573	IPI00130573	yes	yes	3	0.000141	101.05
HN(de)ITITQGK	1010.5509	IPI00128358	IPI00128358	yes	no	2	0.04533	80.706
HNN(de)DTQHIWESDSNEFSVIADPR	2710.2008	IPI00129526	IPI00129526	yes	no	2,3,4	1.5899E-81	207.22
HPAN(de)ECAPYQFR	1488.6568	IPI00221859	IPI00221859	yes	yes	2	0.014006	81.709
HPGN(de)FSSISCDYFAVNQSR	2184.9647	IPI00115976	IPI00115976	yes	yes	2,3	4.3662E-06	128.79
HPGNFSSISCDYFAVN(de)QSR	2184.9647	IPI00115976	IPI00115976	yes	yes	2,3	4.3662E-06	128.79
HSIEHFNN(de)NTDHSHIFTIR	2318.0941	IPI00114958	IPI00114958	yes	no	2,3,4	2.0326E-42	197.15
HSQQPIITYEKYN(de)VTDTPK	2261.1328	IPI00322418	IPI00322418	yes	yes	3,4	0.0037555	85.563
HSVIWNAMIHPIQN(de)MTIK	2132.1023	IPI00111794	IPI00111794	yes	no	2,3	9.8228E-05	113.01
HSVN(de)NTYSQFQDEYSIEEVMK	2547.1224	IPI00134131	IPI00134131	yes	no	3	3.6179E-30	170.53
HSVPVFCHPSFYN(de)DTDFIGEEIDIVDVK	3278.523	IPI00416285	IPI00416285	yes	yes	3,4	0.0001413	80.683
HTDDITSIN(de)NTIVNIR	1824.933	IPI00129304	IPI00129304	yes	yes	2	0.0007478	106.36
HTGPGIISMANAGPNTN(de)GSQFFICTAK	2790.3218	IPI00554989	IPI00554989	yes	yes	3	8.2967E-60	196.02
HTIEIAPN(de)STAR	1308.6786	IPI00620800	IPI00620800	yes	no	2	0.0057994	92.295
HVCPVEN(de)WSYNESCSPDPAEQGGPK	2843.1915	IPI00128269	IPI00128269	yes	yes	3	2.3243E-56	189.82
HVCPVENWSYN(de)ESCSPDPAEQGGPK	2843.1915	IPI00128269	IPI00128269	yes	yes	3	2.3243E-56	189.82
HVEDKFN(de)ETTQR	1502.7114	IPI00130654	IPI00130654	yes	no	2,3	1.4981E-14	171.32
HVGPGVISMANAGPNTN(de)GSQFFICTIK	2816.3738	IPI00116228	IPI00116228	yes	yes	3	3.3798E-14	124.81
HVTDMN(de)STIHIIR	1535.7878	IPI00223446	IPI00223446	yes	yes	3	0.0002346	138.63
HVVIN(de)GSSFGADGFPYVQVIK	2233.1532	IPI00742377	IPI00742377	yes	no	2,3	1.3843E-05	100.97
HYAN(de)ITNGMYR	1338.6139	IPI00224073	IPI00224073	yes	no	2	0.0033924	99.215
I(de)AVISGCKNTTSAAMVHCIR	2188.0915	IPI00131216	IPI00131216	yes	yes	2,3,4	3.2131E-25	168.05
I(de)NHCKFDEFFSQGCAPGYEKNSTICDICIGPIK	3904.7681	IPI00139788	IPI00139788	yes	no	4	2.0546E-29	141.36
IACIN(de)GTVIR	1115.6121	IPI00134808	IPI00134808	yes	no	2	0.0024075	109.42
IAGGENN(de)CSGRVEIK	1602.7784	IPI00760106	IPI00760106	yes	no	2	0.0097614	89.624
IAGIAVTIAKPN(de)STSSFSPSVQCPNDGFGIYSNSYGPEFAHCK	4575.1475	IPI00118674	IPI00118674	yes	yes	5	0.0026354	48.65
IAISFPN(de)ITSDGYPVIYPTGWSSSDNVNCRPR	3582.7202	IPI00466371	IPI01026704	no	no	3,4,5	7.7968E-30	144.48
IAITPAHIIFIADN(de)HTEPAAHFR	2554.3445	IPI00230324	IPI00230324	yes	no	4	6.1147E-06	103.88
IAMCQNIGYN(de)VTK	1510.7272	IPI00117094	IPI00117094	yes	yes	2	0.0006415	114.56
IAN(de)CTISDTIQEVNIEVQK	2174.0889	IPI00120351	IPI00120351	yes	yes	3	1.0358E-06	133.78
IAN(de)ETGGHGSGPR	1251.5956	IPI00309230	IPI00309230	yes	no	2,3	0.0004417	132.79
IAN(de)FSQSCTIYSGDDIVEAIPKPCPGCPR	3251.505	IPI00114958	IPI00114958	yes	no	3,4	7.515E-172	261.54
IAPASN(de)VSHTVVIR	1462.8256	IPI00467180	IPI00467180	yes	yes	2,3	0.0021906	102.5
IAPASN(de)VSHTVVIRPIK	1801.0574	IPI00467180	IPI00467180	yes	yes	2,3,4	4.365E-88	230.82
IAQEGGAAAIIIAN(de)NSVIIPSSR	2264.2488	IPI00121776	IPI00121776	yes	yes	2,3,4	1.6278E-09	129.78
IATIAN(de)FSEIGIEPGR	1686.8941	IPI00129041	IPI00129041	yes	no	2	0.001092	103.87
IATITCHIIPSDVSQVN(de)VSIIIWKPTFIK	3292.8257	IPI00466371	IPI01026704	no	no	4	0.01234	55.051
IAVDWESIGYNI(de)TR	1635.8257	IPI00123040	IPI00123040	yes	no	3	9.8699E-11	158.11
IAVTN(de)TTMTGTVIK	1448.7909	IPI00108041	IPI00108041	yes	yes	2	2.966E-05	139.66
IAYSNN(de)GSDWK	1253.5677	IPI00123996	IPI00123996	yes	yes	2	0.001056	108.9
ICEGDRFCIIDVMSTGSSSVGN(de)ATR	2731.2364	IPI00749655	IPI00749655	yes	no	3	1.0277E-34	169.9
ICHTHGWN(de)ETSEIMPPGAVFSCIYR	2961.336	IPI00122399	IPI00122399	yes	no	3	3.9633E-12	116.45
ICIHIYQIN(de)ETVTVTASIVSQSGR	2688.3905	IPI00123223	IPI00123223	yes	yes	3	0.0028205	69.779
ICKPESGECINCIHN(de)TTGFWCEK	2839.2186	IPI00221701	IPI00221701	yes	yes	3	4.9362E-08	121.21
ICN(de)ECSDGSFHISK	1652.6923	IPI00515360	IPI00515360	yes	no	2,3	7.8087E-05	131.35
ICQN(de)ISKK	989.5328	IPI00126184	IPI00126184	yes	yes	2	0.0008068	144.12
ID(de)IPVNTSIPNVTEIKENMTFGSTIVTNPK	3271.701	IPI00466371	IPI01026704	no	no	3,4	1.8626E-35	118.52
IDAPTNIQFVN(de)ETDR	1731.8428	IPI00975193	IPI00975193	yes	no	2,3	2.0949E-24	181.51
IDAPTNIQFVN(de)ETDRTVIVTWTPPR	2882.4927	IPI00975193	IPI00975193	yes	no	3	1.0193E-15	138.04
IDAYNEAAVSIIN(de)SSTR	1822.9061	IPI00380296	IPI00380296	yes	yes	2,3	0.0005193	103.1
IDCYPDEHGASEAN(de)CSAR	2050.8109	IPI00848693	IPI00848693	yes	yes	2,3	1.8457E-08	137.45
IDETDCIGN(de)WTWQEGSQQTIK	2508.1227	IPI00122973	IPI00122973	yes	no	3	1.7027E-40	188.04
IDFIVINE(de)TR	1218.6608	IPI00125813	IPI00125813	yes	no	2	0.0003007	121.68
IDGQWPDGIIASDNTIHFVHPITVN(de)YSGVYVCK	3714.8141	IPI00471187	IPI00471187	yes	no	4	0.0054918	49.525
IDIPVN(de)TSIPNVTEIK	1751.9669	IPI00466371	IPI01026704	no	no	2,3	2.6378E-13	165.58
IDIPVN(de)TSIPNVTEIKENMTFGSTIVTNPK	3271.701	IPI00466371	IPI01026704	no	no	3,4	1.8626E-35	118.52
IDIPVNTSIPN(de)VTEIK	1751.9669	IPI00466371	IPI01026704	no	no	2,3	2.6378E-13	165.58
IDIPVNTSIPN(de)VTEIKENMTFGSTIVTNPK	3271.701	IPI00466371	IPI01026704	no	no	3,4	1.8626E-35	118.52

IDITDFEKN(de)SSFAQYQSFK	2267.0746	IPI00121312	IPI00121312	yes	yes	2	9.9954E-79	223.36
IDKADGN(de)ITIEGASFVDGDNAATNGVVHIINK	3267.6371	IPI00467600	IPI00467600	yes	no	4	0.0035537	59.282
IDKADGNITIEGASFVDGDNAATNGVVHIIN(de)K	3267.6371	IPI00467600	IPI00467600	yes	no	4	0.0035537	59.282
IDKGGITINAN(de)ISGDAFIK	1946.0473	IPI00134585	IPI00134585	yes	no	2,3	8.2447E-05	119.46
IDPPCTN(de)TTAPSNYINNPYVR	2406.1274	IPI00658539	IPI00658539	yes	no	2,3	1.5748E-22	161.08
IDRNPSPDEIPQVGN(de)ISIK	2091.096	IPI00308990	IPI00308990	yes	yes	3	7.8999E-05	119.8
IDSFN(de)DSTFMIVYTPITPTTQR	2546.2363	IPI00322575	IPI00322575	yes	no	3	7.0987E-06	103.41
IDSTGN(de)VTNEIR	1317.6525	IPI00224752	IPI00224752	yes	yes	2	0.014195	81.565
IDWIGN(de)CSGINDDSYGYR	2103.8956	IPI00121550	IPI00121550	yes	yes	2	3.5476E-48	200.39
IEAVIPAEFFEVISSSQN(de)GSYHHIR	2829.4086	IPI00342158	IPI00342158	yes	no	3,4	1.2746E-35	175.33
IEAYFSIEGFPAEQN(de)ASNR	2142.0018	IPI00114319	IPI00114319	yes	yes	2,3	1.3699E-52	201.37
IEDGFHPDAVAWAN(de)ITNAIR	2209.0916	IPI00165730	IPI00165730	yes	no	2,3	2.3073E-14	155.79
IEDRFN(de)STIGPSEEQEK	1977.928	IPI00115516	IPI00115516	yes	no	2,3	4.2494E-08	144.73
IEDRFN(de)STIGPSEEQEKNWPGGPGR	2799.3212	IPI00115516	IPI00115516	yes	no	3,4	1.1645E-11	118.05
IEGITN(de)ETYR	1194.5881	IPI00119299	IPI00119299	yes	no	2	0.0012296	113.38
IEGVTMFAMGIEGAN(de)NTQIEDIVSYPSR	3041.4474	IPI00990932	IPI00990932	yes	no	3,4	0.0001632	75.71
IEIVPIIYDN(de)DSIFVQTDK	2221.1518	IPI00405543	IPI00405543	yes	yes	2,3	1.5095E-52	200.58
IEKIEGIIAN(de)VSR	1440.83	IPI00115516	IPI00115516	yes	no	2,3	0.0010377	135.02
IEN(de)ISSTESGYTATITR	1841.9007	IPI00111960	IPI00111960	yes	no	2,3	1.2653E-21(	300.97
IEN(de)ITTGTYTIHAQK	1688.8733	IPI00222429	IPI00222429	yes	yes	2,3	1.5764E-24	183.23
IEN(de)NCTHGEDTWMECEDPFEIK	2753.1044	IPI00316045	IPI00316045	yes	yes	3	2.2777E-39	178.71
IENN(de)CTHGEDTWMECEDPFEIK	2753.1044	IPI00316045	IPI00316045	yes	yes	3	2.2777E-39	178.71
IEPEHIQIQN(de)ISQEQIAQVATIATK	2801.4923	IPI00165807	IPI00165807	yes	no	3	1.2731E-56	193.87
IETIIIN(de)GTDRK	1371.7722	IPI00119063	IPI00119063	yes	no	2	0.0015902	113.99
IFFVNP(de)APPYIWPAHKNEIMINSSIMR	3184.6354	IPI00378698	IPI00378698	yes	no	4	0.0001918	88.755
IFIFN(de)QTGIEAK	1379.7449	IPI00378224	IPI00378224	yes	yes	2	3.7853E-15	173.19
IFIFN(de)QTGIEAKK	1507.8399	IPI00378224	IPI00378224	yes	yes	3	0.001135	120.57
IFIGN(de)YSGNVGK	1267.6561	IPI00153416	IPI00153416	yes	yes	2	0.0054575	93.011
IFIYSGEPIYIGN(de)ETSIFGPK	2344.1991	IPI00750091	IPI00750091	yes	yes	3	1.5728E-08	126.07
IFNKFAVESIVPSSISIMHSPP(de)DAQNMSEVSISPMEISTFR	4522.2222	IPI00114044	IPI00114044	yes	yes	5	0.025116	39.144
IFTPVSSVPN(de)ITWSEISAIEIIK	2543.3887	IPI00652728	IPI00652728	yes	no	3	0.0006069	85.248
IGAGSGPIWIDDIACNGN(de)ESAIWDCK	2818.2691	IPI00760106	IPI00760106	yes	no	3	3.2576E-35	169.19
IGAIN(de)STISNESK	1332.6885	IPI00187353	IPI00187353	yes	yes	2	0.032503	74.789
IGAIN(de)STISNESKEAFIDWAR	2321.1652	IPI00187353	IPI00187353	yes	yes	3	5.269E-07	117.2
IGAINN(de)SIIIIEDR	1539.8621	IPI00115516	IPI00115516	yes	no	2,3	4.3453E-07	147.7
IGAINN(de)SIIIIEDRIQQISIK	2350.3584	IPI00115516	IPI00115516	yes	no	3	0.053594	62.203
IGAINSTISN(de)ESK	1332.6885	IPI00187353	IPI00187353	yes	yes	2	0.032503	74.789
IGAINSTISN(de)ESKEAFIDWAR	2321.1652	IPI00187353	IPI00187353	yes	yes	3	5.269E-07	117.2
IGGWN(de)ITGPWAK	1298.6772	IPI00396840	IPI00396840	yes	no	2	0.0083718	86.911
IGHTN(de)ASIMIFEVK	1558.8177	IPI00858152	IPI00858152	yes	yes	3	0.0001558	119.39
IGISFNSITVMEN(de)GSIANVPHIR	2468.2846	IPI00123196	IPI00123196	yes	yes	2,3,4	1.4677E-72	214.55
IGN(de)FSEIATHNQTFIK	1818.9264	IPI00855103	IPI00855103	yes	no	2	2.9067E-18	171.71
IGN(de)FTIAYSAKPETADNQR	2095.0334	IPI00229198	IPI00229198	yes	no	2,3	1.3913E-05	126.25
IGN(de)VTTWISSSNPPVTFAAIYWEEPDVSGHK	3401.6568	IPI00225072	IPI00225072	yes	no	3,4	6.4678E-16	117.94
IGN(de)WTGEWPETEIVANIWMK	2373.1464	IPI00322497	IPI00322497	yes	yes	2,3,4	1.3618E-94	237.73
IGNFSEIATHN(de)QTFIK	1818.9264	IPI00855103	IPI00855103	yes	no	2	2.9067E-18	171.71
IGPGEPIEIICN(de)VSGAIPPPGR	2242.178	IPI00321348	IPI00321348	yes	no	2,3,4	1.3882E-22	158.17
IGTNRPSPSVIQEIN(de)VTVVTNMCR	2684.3738	IPI00122302	IPI00122302	yes	yes	3	0.0005373	91.583
IGVQMHPGQEIHN(de)FTITGR	2134.0742	IPI00119299	IPI00119299	yes	no	2,3,4	3.5981E-09	140.32
IGVTN(de)ASIVIFRPGSVR	1785.0261	IPI00652675	IPI00652675	yes	yes	2,3	0.0005286	120.77
IGYDPYANPPNYGNPDPIVIN(de)NTHR	2810.3412	IPI00316430	IPI00316430	yes	no	3,4	1.6676E-22	137.72
IHIGNYN(de)GTAGDAIR	1570.7852	IPI00310797	IPI00310797	yes	yes	2	0.0012694	104.95
IHIIPSMNPDGYEVAAAQGPN(de)MSGYIVGR	3056.4848	IPI00128260	IPI00128260	yes	yes	3	4.409E-120	238.7
IHKDN(de)TTCYEFK	1554.7137	IPI00119063	IPI00119063	yes	yes	2,3	0.015264	93.345
IHRIN(de)ASIADIQSK	1564.8685	IPI00116913	IPI00116913	yes	no	2	0.0036691	107.74
IHVAQPEN(de)DSHVAIIK	1769.9424	IPI00850413	IPI00850413	yes	yes	2,3	2.1767E-34	190.24
IHYIYIQNNFITEIPIESFQN(de)ATGIR	3093.5924	IPI00122293	IPI00122293	yes	yes	3,4	9.7614E-81	214.35
II(de)DIDSCIDRSNYSVIDITPVAAIIPK	3000.5842	IPI00225072	IPI00225072	yes	no	3,4	4.4637E-06	103.84
IIASN(de)ITETMR	1247.6544	IPI00132600	IPI00132600	yes	no	2	0.00032	136.96
IIASPNEENM(de)TEIISMR	1946.9441	IPI00322575	IPI00322575	yes	no	2,3	1.2005E-47	200.97
IIDIIPDGYPQISCIPKEEN(de)ATIATYPEFGVIDIK	3932.0169	IPI00124221	IPI00124221	yes	no	3,4,5	2.0261E-05	70.15
IIDQACGTDN(de)QTYASSCHIFATK	2600.1635	IPI00308484	IPI00308484	yes	yes	3	4.8988E-31	168.78
IIEFDSTN(de)ASEGAQPPGKPYPPYSIAK	2876.4232	IPI00459432	IPI00459432	yes	yes	3,4	1.3243E-38	177.32
IIGGGDEDAIRPQMQQIIDFETAIAN(de)ITIPQEK	3623.8505	IPI00396840	IPI00396840	yes	no	3,4	2.1103E-70	192.18
IIGGGDEDAIRPQMQQIIDFETAIAN(de)ITIPQEKR	3779.9516	IPI00396840	IPI00396840	yes	no	4	0.017608	47.647
IIGVPSIEDIDEVIIHNIPDAIGVIFN(de)DSFSYQIK	3883.0295	IPI00170146	IPI00170146	yes	no	3,4	7.0292E-20	119.8
IIIGGIPVSGTFHN(de)FSGCISNVFVQR	2818.4589	IPI00116913	IPI00116913	yes	no	3,4	2.6233E-10	116.21
IIIN(de)FTSMDIYR	1484.7697	IPI00125182	IPI00125182	yes	no	2	0.0007915	123.64
IIIN(de)WTQGQTSGVIR	1684.9261	IPI00674656	IPI00674656	yes	no	2	0.0001976	120.63
IIISPEEN(de)VTITCTAENQIER	2429.2108	IPI00121378	IPI00121378	yes	no	2,3	2.9277E-134	252.83
IIISPSAFHDGN(de)FSIIIR	1999.0891	IPI00310519	IPI00310519	yes	no	3	6.9549E-06	129.93
IIITAAPN(de)ITTSPAFR	1684.9512	IPI00314726	IPI00314726	yes	yes	3	0.0052257	81.311
IIITAAPN(de)ITTSPAFRYDIIDVTR	2660.4538	IPI00314726	IPI00314726	yes	yes	3	0.0016297	85.146
IIN(de)ASVIGDHTK	1266.6932	IPI00116056	IPI00116056	yes	yes	3	0.017444	79.089
IIN(de)ATHQIGCQSSISGDTGVIHVVEKEEDIK	3376.6933	IPI00118674	IPI00118674	yes	yes	4,5	6.3374E-11	109.68
IIN(de)HSIIIHK	1186.7186	IPI00406603	IPI00406603	yes	yes	2,3	3.2283E-11	167.12
IIN(de)ITFIDITR	1317.7656	IPI00343568	IPI00343568	yes	no	2	0.0004106	117.79
IIN(de)ITNPVINQEIEAFSFPEDASSSR	2890.4349	IPI00221444	IPI00221444	yes	no	3	1.4088E-35	171.75
IIN(de)QTADMIQIASK	1544.8232	IPI00126864	IPI00126864	yes	no	3	0.010327	80.746
IIN(de)QTIR	856.51305	IPI00460063	IPI00460063	yes	no	2	0.017481	101.64
IIN(de)QTIRENIK	1340.7776	IPI00460063	IPI00460063	yes	no	2	1.6485E-09	163.12
IIN(de)TTDVYIIPSINPDGFER	2276.1689	IPI00130573	IPI00130573	yes	yes	2,3	1.0228E-09	142.99
IINDYVSN(de)QTQGMIK	1722.8611	IPI00135635	IPI00135635	yes	no	2	0.0017385	100.58
IINKFN(de)SSSSSIEEK	1681.8523	IPI00338561	IPI00338561	yes	yes	2	0.016228	87.566
IIPAFN(de)TTSGIPYPR	1645.8828	IPI00127672	IPI00127672	yes	no	3	0.017906	72.681
IIPGGN(de)TSFDVVFIAR	1704.9199	IPI00118333	IPI00118333	yes	yes	3	0.0009187	102.28

IIPHIEKPIQN(de)FTICFR	2125.1507	IPI00309214	IPI00309214	yes	yes	2,3,4	0.0008784	115.87
IIQQQSN(de)QSSQFIHSVER	2128.0661	IPI00876541	IPI00876541	yes	no	2	1.979E-48	204.23
IIQTAEHN(de)ISGAER	1537.7849	IPI00403938	IPI00403938	yes	no	2,3	1.7886E-135	262.42
IIQVVYIHSNN(de)ITK	1640.925	IPI00123194	IPI00123194	yes	yes	2,3	3.4828E-16	171.81
IIRVDIPQQPMGIIAVAN(de)DTNSCEISPCR	3266.621	IPI00119063	IPI00119063	yes	yes	3	1.9387E-51	186.44
IIRVDIPQQPMGIIAVAN(de)DTNSCEISPCR	3266.621	IPI00119063	IPI00119063	yes	yes	3	1.9387E-51	186.44
IISQAPSTPSPNMFTINN(de)ETGDIITVAAGIDR	3342.6766	IPI00323134	IPI00323134	yes	no	3,4	2.1395E-07	90.616
IISQAPSTPSPNMFTINN(de)ETGDIITVAAGIDREK	3599.8141	IPI00323134	IPI00323134	yes	no	3,4,5	9.5466E-23	131.62
IISYN(de)VSCSINEETQSVIEIFDPQHR	3077.4764	IPI00119299	IPI00119299	yes	no	3	1.0828E-13	129.43
IITHTIEGPSQN(de)VTIIQIQPWQDPESWER	3414.7208	IPI00473190	IPI00473190	yes	no	3,4	2.9488E-09	108.44
IITN(de)NSQTPIISPQEVVSCSPYAQGCDGGFPYIIAGK	3980.9288	IPI00130015	IPI00130015	yes	yes	4	1.3871E-26	131.68
IIY(de)EGGDIPDFRKENSSYQVINWR	2898.4301	IPI00153316	IPI00153316	yes	yes	3,4	0.010946	87.654
IIYFGYSIIGAHPIIN(de)QTVNR	2388.2954	IPI00221818	IPI00221818	yes	yes	3	0.0040205	73.928
IIYGIEN(de)TTQEIK	1520.8086	IPI00309999	IPI00309999	no	no	2	0.0004353	132.97
IIYIN(de)VSNNIIQIPAGIPR	2107.2154	IPI00884541	IPI00884541	yes	yes	3	3.3853E-09	140.96
IIYISAEDFSIDHSPN(de)STAGPSCSIIQEAFR	3424.6245	IPI00115530	IPI00115530	yes	yes	3,4	6.014E-49	170.59
IIYISAEDFSIDHSPN(de)STAGPSCSIIQEAFRR	3580.7256	IPI00115530	IPI00115530	yes	yes	3,4	6.6853E-06	80.132
IK(de)FIEAGIYEVPIIITDSGNPPKSNISIIR	3296.8384	IPI00323134	IPI00323134	yes	no	3,4,5	1.271E-22	143.91
IKEQVVGIIAQN(de)NCSCESK	2176.0616	IPI00122592	IPI00122592	yes	yes	3	1.9753E-41	195.46
IKEQVVGIIAQNN(de)CSCESK	2176.0616	IPI00122592	IPI00122592	yes	yes	3	1.9753E-41	195.46
IKGEAEYQEIRNPN(de)GTVTVISR	2473.2925	IPI00311405	IPI00311405	yes	yes	3,4	0.0009807	90.718
IKIDN(de)YSTQEIGR	1535.7944	IPI00122257	IPI00122257	yes	no	2,3	9.7217E-22	179.67
IKN(de)ATVTQYQIK	1405.7929	IPI00221547	IPI00221547	yes	no	2,3	0.0009637	138.99
IKN(de)TTNQIAIIK	1355.8136	IPI00985989	IPI00985989	yes	no	2,3	0.0002399	145.31
IKVSEDGYSIYMSN(de)ITK	1946.9659	IPI00129646	IPI00129646	yes	no	3	2.1584E-05	134.25
IKVSN(de)VSCEASVSK	1506.7712	IPI00135560	IPI00135560	yes	no	3	0.0048711	105.03
IMESHPN(de)GTFSAK	1417.666	IPI00468055	IPI00468055	yes	no	2,3	0.0001135	143.31
IMNAPIYIAEWQN(de)ITK	1903.9866	IPI00930882	IPI00930882	yes	no	3	4.214E-07	139.58
IMQDPIQQAEGIYCN(de)R	1934.8979	IPI00314549	IPI00314549	yes	yes	2,3	2.9722E-09	150.05
IN(de)ETHIFNGSNWIMIIYK	2192.1088	IPI00108844	IPI00108844	yes	yes	2,3	1.2068E-21	174.59
IN(de)GTDPIVAADSK	1299.667	IPI00119063	IPI00119063	yes	yes	2	0.0070781	85.909
IN(de)GTDPIVAADSKR	1455.7682	IPI00119063	IPI00119063	yes	yes	2,3	0.0084744	92.039
IN(de)HCKFDEFFSQGCAPGYEKNSTICDICIGPIK	3904.7681	IPI00139788	IPI00139788	yes	no	4	2.0546E-29	141.36
IN(de)ISEGEVAATVK	1329.714	IPI00458003	IPI00458003	yes	yes	2	3.885E-05	146
IN(de)ITAAQISQIEGIIQAR	1938.0898	IPI00115516	IPI00115516	yes	no	2,3	6.704E-107	165.3
IN(de)ITEEEKIINR	1470.8042	IPI00320204	IPI00320204	yes	yes	2	3.5706E-08	157.56
IN(de)ITEEEKIINRR	1626.9053	IPI00320204	IPI00320204	yes	yes	2	0.002835	112.51
IN(de)KTVAIHTIDPEK	1577.8777	IPI00323624	IPI00323624	yes	no	2	0.0026475	110.55
IN(de)KTVAIHTIDPEKIGQGGVQK	2345.3067	IPI00323624	IPI00323624	yes	no	3	0.052232	61.767
IN(de)MTIPDAIVPTFSISNHSIK	2297.209	IPI00469218	IPI00469218	yes	yes	2,3,4	4.2671E-48	189.55
IN(de)TTSDEKDPTNPFR	1733.822	IPI00131881	IPI00131881	yes	no	2,3	0.002569	109.07
IN(de)TTSDEKDPTNPFRFPNIGVEK	2618.2976	IPI00131881	IPI00131881	yes	no	3	0.0015938	87.352
IN(de)VSYTGERPSSNMVIVDVK	2207.1256	IPI00624663	IPI00624663	yes	yes	2,3	0.0040801	83.758
IN(de)VTEVYDKIK	1320.7289	IPI00225072	IPI00225072	yes	no	3	0.0015193	122.7
IN(de)YTIVGEPIR	1273.703	IPI00120674	IPI00120674	yes	no	2	0.0004494	132.79
INETHIFN(de)GSNWIMIIYK	2192.1088	IPI00108844	IPI00108844	yes	yes	2,3	1.2068E-21	174.59
INIDGSN(de)YTIIK	1349.7191	IPI00119063	IPI00119063	yes	yes	2	0.011616	83.53
INMTIPDAIVPTFSISN(de)HSIK	2297.209	IPI00469218	IPI00469218	yes	yes	2,3,4	4.2671E-48	189.55
INN(de)ITNIGPIDMK	1441.7599	IPI00330680	IPI00330680	yes	no	2,3	0.0005987	114.74
INNGGCQDICIITHQGHVN(de)CSCR	2712.1737	IPI00119063	IPI00119063	yes	yes	3	1.1702E-07	106.49
INSAPVEGYSEHVGN(de)K	1699.8166	IPI00349830	IPI00349830	yes	no	2,3	3.1075E-13	163.41
IPEN(de)QTIPGEIPEHAGPAEGVHDSR	2649.2783	IPI00856221	IPI00856221	yes	yes	3	0.0006721	79.69
IPFNVIMNNVEDIIEQQTFN(de)DTVSPR	3032.4913	IPI00310049	IPI00310049	yes	yes	3,4	0.00117	71.052
IPN(de)ISIDVIQPSFPEIIIESHMVMIR	2990.5973	IPI00221669	IPI00221669	yes	no	3	0.0002123	85.248
IPNNTQWITW(de)SPEGHK	1906.9326	IPI00125813	IPI00125813	yes	no	2,3	4.7183E-05	126.77
IPQQIHHYIN(de)ASDWER	2005.9759	IPI00666034	IPI00666034	yes	no	2	0.0007162	107.5
IPSSIEN(de)ATSISIMNITGTAICHISDIPPDGIR	3492.7593	IPI00121776	IPI00121776	yes	yes	3	9.1324E-40	129.35
IPSSIENATSISIMN(de)ITGTAICHISDIPPDGIR	3492.7593	IPI00121776	IPI00121776	yes	yes	3	9.1324E-40	129.35
IPVAVIN(de)ATAK	1095.6652	IPI00122491	IPI00122491	yes	yes	2	0.009307	88.187
IPYQGN(de)ATMIVVIMEK	1805.942	IPI00153258	IPI00153258	yes	yes	2,3	5.3412E-10	155.98
IQCVDGN(de)WTTIPVCIEEER	2318.0671	IPI00130010	IPI00130010	yes	no	2	2.8156E-65	214.59
IQCVDGN(de)WTTIPVCIEEERTCGDIPEIEHGSAK	3812.7444	IPI00130010	IPI00130010	yes	no	4	7.7114E-05	69.952
IQDFIVDN(de)ETFSGFIQHNISIPR	2689.35	IPI00112614	IPI00112614	yes	yes	3,4	6.1992E-24	167.24
IQDFIVDNETFSGFIQHN(de)ISIPR	2689.35	IPI00112614	IPI00112614	yes	yes	3,4	6.1992E-24	167.24
IQDIEITGSPVSN(de)ISAHIFSNISSIEK	2898.4975	IPI00119522	IPI00119522	yes	yes	2,3,4	4.8906E-30	159.76
IQDIEITGSPVSNISAHIFSN(de)ISSIEK	2898.4975	IPI00119522	IPI00119522	yes	yes	2,3,4	4.8906E-30	159.76
IQFDDN(de)GTYTCQVK	1687.7512	IPI00116476	IPI00116476	yes	no	2	0.0010697	109.35
IQIEFRPIDIN(de)STAAGIPR	2110.1535	IPI00115510	IPI00115510	yes	yes	2,3	8.7668E-09	141.6
IQNIISEKPSVTTVYINN(de)GSVIQGSSVATIYDRK	3693.9578	IPI00310567	IPI00310567	yes	yes	4	0.0022124	55.587
IQQEFHCCGSN(de)NSQDWQDSEWIR	2923.2038	IPI00266426	IPI00266426	yes	yes	3	0.0016243	76.015
IQSPIFIIDANANIQN(de)VTTSGNK	2457.2864	IPI00666034	IPI00666034	yes	no	3	2.6959E-17	154.84
IQSPIFIIDANANIQN(de)VTTSGNKAEIVASVTAK	3426.8358	IPI00666034	IPI00666034	yes	no	4	7.558E-16	119.37
IQTPIN(de)YTEFQKPICIPSK	2276.1875	IPI00113057	IPI00113057	yes	yes	2,3,4	1.9449E-13	153.74
IQVTIYN(de)CSFGR	1456.7133	IPI00405742	IPI00405742	yes	no	2	0.0001583	143.42
IRAEQITIHAIGIGEAN(de)KTQIR	2431.3659	IPI00990932	IPI00990932	yes	no	3	5.3143E-05	100.21
IRDYEEN(de)SSSCHKEVQIIK	2334.1274	IPI00330594	IPI00330594	yes	yes	3	0.0001094	118.21
IRN(de)ATITQAITNK	1442.8205	IPI00876028	IPI00876028	yes	yes	3	0.01562	90.793
IRN(de)SSIGGVINK	1256.7201	IPI00314673	IPI00314673	yes	yes	2,3	0.000456	143.42
IRN(de)SSIGGVINKYDVVIR	2002.1324	IPI00314673	IPI00314673	yes	yes	3	5.0377E-05	126.16
IRNPCTSE(de)QNCTSPFSYK	2187.9677	IPI00132474	IPI00132474	yes	yes	2,3	3.2634E-37	193.8
IRPHFISVCDPDFSQIN(de)CSEGYIQNYR	3314.5237	IPI00115626	IPI00115626	yes	yes	3,4	8.3463E-112	234.36
IRPIFN(de)K	886.53887	IPI00108844	IPI00108844	yes	yes	3	0.060546	100.07
IRPIFN(de)KSFESTVGQGSDTYSYIFR	2911.4505	IPI00108844	IPI00108844	yes	yes	3,4	5.0341E-21	156.05
IRVN(de)GTKEPIEFK	1529.8566	IPI00345112	IPI00345112	yes	no	2,3	0.0046252	106.67
IS(de)HYKQNFSFCR	1585.746	IPI00381303	IPI00381303	yes	yes	2	0.0029559	110.92
ISAIDNIIN(de)HSSIFIK	1783.9832	IPI00123342	IPI00123342	yes	yes	2,3	2.515E-119	252.47

ISASGAEIEAIEAQVIN(de)ISIK	2155.1736	IPI00312711	IPI00312711	yes	no	2,3	1.2564E-100	233.63
ISCAFKTEN(de)QTR	1453.6984	IPI00120245	IPI00120245	yes	no	2	5.3225E-15	175.74
ISDTN(de)ITAIPQGIPTSITEVHIDGNK	2733.4185	IPI00123196	IPI00123196	yes	yes	3	1.3928E-22	157.3
ISDTN(de)ITAIPQGIPTSITEVHIDGNKITK	3075.6452	IPI00123196	IPI00123196	yes	yes	3,4	1.8351E-05	95.123
ISDTN(de)ITAIPQGIPTSITEVHIDGNKITKVDAPSIK	3786.0415	IPI00123196	IPI00123196	yes	yes	4	0.0002326	63.203
ISEEFIKN(de)ISASAR	1563.8257	IPI00894970	IPI00894970	yes	no	2	0.0003654	124.16
ISEGN(de)RTITIINVTR	1685.9424	IPI00108535	IPI00108535	yes	no	2	0.0006223	107.35
ISEGN(de)RTITIINVTRNDTGPYVCETR	2978.488	IPI00108535	IPI00108535	yes	no	3,4	0.0034858	76.049
ISEGNR(de)TITIINVTR	1685.9424	IPI00108535	IPI00108535	yes	no	2	0.0006223	107.35
ISEGNR(de)TITIINVTRNDTGPYVCETR	2978.488	IPI00108535	IPI00108535	yes	no	3,4	0.0034858	76.049
ISEGNRTITI(de)INVTRNDTGPYVCETR	2978.488	IPI00108535	IPI00108535	yes	no	3,4	0.0034858	76.049
ISEN(de)GSSVAGIISSPNMEK	1918.9306	IPI00399798	IPI00399798	yes	no	2,3	1.0578E-19	162.32
ISFIIGSDSTHVIPGESPFN(de)K	2244.1426	IPI00123783	IPI00123783	yes	no	3	0.0093786	67.645
ISGKPTN(de)VSVSVIMSEGDGICY	2312.1028	IPI00473880	IPI00473880	yes	no	2	1.2385E-85	223.18
ISGNSIHN(de)ASIPEHIAYGSVITVK	2506.318	IPI00221669	IPI00221669	yes	no	3	8.3948E-21	152.06
ISHDGN(de)ETIPIHIYVK	1834.9577	IPI00405742	IPI00405742	yes	no	2,3,4	4.9188E-46	208.15
ISHYN(de)DTYPISPPQR	1786.8638	IPI00113039	IPI00113039	yes	no	2,3	9.8476E-12	158.29
ISIN(de)ESMQVVSR	1361.6973	IPI00471022	IPI00471022	yes	yes	2	0.0096606	84.753
ISISEN(de)YTISIANAK	1622.8516	IPI00121378	IPI00121378	yes	no	2,3	1.2107E-56	216
ISITN(de)ETFRK	1207.6561	IPI00123652	IPI00123652	yes	no	2	0.0011586	115.57
ISN(de)ITIIIEMEKESAR	1845.987	IPI00356462	IPI00356462	yes	no	2	0.0005434	123.08
ISN(de)VTPEDAGTYYCVK	1815.8349	IPI00129158	IPI00129158	yes	no	2	8.54E-06	135.23
ISNISN(de)ISHDIVQEATDHAYNIQQEADEISR	3509.6659	IPI00223446	IPI00223446	yes	yes	3	6.7833E-59	179.47
ISPGGAEMFQVQDMVVSQEKGN(de)CSIQR	2994.3998	IPI00130117	IPI00130117	yes	no	4	9.5119E-06	100.19
ISPIHIAIN(de)FSIDPK	1663.9297	IPI00115976	IPI00115976	yes	yes	2,3	5.3454E-08	148.72
ISQAIGN(de)ITVVQK	1369.7929	IPI00112032	IPI00112032	yes	no	2,3	4.8043E-15	167.89
ISQKN(de)QTVFER	1348.7099	IPI00130117	IPI00130117	yes	no	2,3	0.0043971	105.46
ISQN(de)ASIGPHVRPIPIQYEDK	2361.2441	IPI00116945	IPI00116945	yes	no	3	0.040348	63.998
ISQN(de)ASIGPHVRPIPIQYEDKEVEPGTICDVAGWGVVTHAGR	4552.2921	IPI00116945	IPI00116945	yes	no	4,5	6.9592E-16	95.36
ISSSPN(de)VTISCK	1291.6442	IPI00225715	IPI00225715	yes	no	2	0.0090198	85.554
ISTNI(de)TIVCKPGDIESAPVIR	2282.2304	IPI00308971	IPI00308971	yes	yes	2	7.7591E-59	202.22
ISTSPFAINI(de)TMIPK	1631.8957	IPI00666034	IPI00666034	yes	no	2,3	1.3904E-33	194.98
ISVPDGIKVSN(de)SSAR	1528.8209	IPI00120761	IPI00120761	yes	no	2	0.0002387	125.22
ISWPSNVN(de)GSHR	1352.6585	IPI00122705	IPI00122705	yes	no	2	0.010779	84.169
ITAN(de)STWQPDKAK	1458.7467	IPI00653675	IPI00653675	yes	no	2	0.0023345	129.77
ITATDGENFATPIYIN(de)ITVAASR	2437.2489	IPI00623114	IPI00623114	yes	no	3	1.0804E-13	146.97
ITCFNN(de)FSIAAPESSPHPAHSQVVSTGYSR	3260.5309	IPI00125549	IPI00125549	yes	no	3,4	8.2463E-06	92.41
ITDIEN(de)GTFANIPR	1559.7944	IPI00117957	IPI00117957	yes	yes	2	7.071E-43	202.25
ITDTICGVGN(de)MSANASDQER	2137.9368	IPI00114065	IPI00114065	yes	no	3	0.0078466	70.837
ITDTICGVGNMSAN(de)ASDQER	2137.9368	IPI00114065	IPI00114065	yes	no	3	0.0078466	70.837
ITEFTHN(de)STMDYK	1585.7083	IPI00130010	IPI00130010	yes	no	2,3	0.0005799	122.78
ITFFNSTIN(de)TSGIVAQGEAIPIPGAHRPGIVTK	3405.8409	IPI00153317	IPI00153317	yes	yes	3,4	3.0402E-16	125.52
ITHIPDDIPSN(de)ITVINITHNQIR	2623.4082	IPI00320618	IPI00320618	yes	yes	3,4	7.0493E-17	153.25
ITHIPDDIPSNITVIN(de)ITHNQIR	2623.4082	IPI00320618	IPI00320618	yes	yes	3,4	7.0493E-17	153.25
ITIAIN(de)NTITPHTIPPGTIVYK	2376.3417	IPI00309230	IPI00309230	yes	no	2,3	1.4753E-134	254.26
ITIEVFDPVPKPSIEIN(de)KTEASTDSCHIR	3295.6758	IPI00109946	IPI00109946	yes	yes	4	0.0094503	58.938
ITN(de)ISIAMSGVYVCK	1654.8422	IPI00126827	IPI00126827	yes	no	2	1.8169E-17	173.51
ITN(de)QTIGFSFAVEQDIPVK	2106.0997	IPI00624663	IPI00624663	yes	yes	2,3	8.87E-111	249.52
ITN(de)QTIGFSFAVEQDIPVKNIKPAPIK	2967.6433	IPI00624663	IPI00624663	yes	yes	4	0.037238	45.878
ITN(de)VTFPTGVVTNIHGDMDK	2158.0729	IPI00123783	IPI00123783	yes	no	3	7.7876E-07	117.3
ITPNCCN(de)TSTVADAYR	1841.8036	IPI00111228	IPI00111228	yes	yes	2	0.0010308	99.663
ITQN(de)ITDIIVNVSDSIEMR	2160.1096	IPI00273133	IPI00273133	yes	yes	3	0.0011961	85.636
ITQNITDIIVN(de)VSDSIEMR	2160.1096	IPI00273133	IPI00273133	yes	yes	3	0.0011961	85.636
ITSCATN(de)ASMCGDEAR	1742.7022	IPI00119063	IPI00119063	yes	yes	2,3	4.4654E-45	200.33
ITSFMIEN(de)GTSDCNIPENPIFISCDGR	3086.3784	IPI00225715	IPI00225715	yes	yes	3	0.0002149	78.976
ITSFMIEN(de)GTSDCNIPENPIFISCDGRNHTISGSK	3910.7924	IPI00225715	IPI00225715	yes	yes	4	4.9772E-08	80.067
ITSFMIENGTSDCNIPENPIFISCDGRN(de)HTISGSK	3910.7924	IPI00225715	IPI00225715	yes	yes	4	4.9772E-08	80.067
ITSN(de)ATSDINIK	1275.667	IPI00461493	IPI00461493	yes	no	2	0.0031372	99.531
ITTTPTNGQQGTSIEEVVHADQSSCTFENINPGIEYN(de)VSVYTV	4827.2821	IPI00975193	IPI00975193	yes	no	4,5	4.0624E-08	68.916
ITTTSPWMIPAPHVWPEDHVFISTPNFN(de)YTVQDFER	4272.0415	IPI00133500	IPI00133500	yes	yes	4,5	0.0001177	61.725
ITVIDANDNAPAFN(de)QSIYR	2121.0491	IPI00129613	IPI00129613	yes	yes	2,3	1.9518E-128	259.64
ITVPSSQN(de)SSFR	1321.6626	IPI00515360	IPI00515360	yes	no	2	0.015343	80.69
ITVVYAEN(de)GTVIQGTTVASVYK	2312.2264	IPI00317356	IPI00317356	yes	yes	2,3	4.3586E-250	311.65
ITYESGFIN(de)YSK	1420.6874	IPI00666034	IPI00666034	yes	no	2	1.553E-06	154.36
ITYN(de)ESR	881.4243	IPI01008227	IPI01008227	yes	no	2	0.023094	99.139
IVAIAVIDEKN(de)TSIEHTR	2008.0953	IPI00453798	IPI00453798	yes	yes	3	1.6498E-18	136.39
IVAN(de)ISGCAAVNSETIMCCIR	2338.0902	IPI00222489	IPI00222489	yes	yes	3	2.7468E-76	204.23
IVCTQKKEN(de)ESR	1490.7511	IPI00315155	IPI00315155	yes	no	2,3	0.0043827	107.06
IVDTN(de)KTR	945.52434	IPI00876558	IPI00876558	yes	no	2	2.7257E-06	156.35
IVDVN(de)ITSEGK	1173.6241	IPI00113528	IPI00113528	yes	no	2	0.0019443	104.24
IVDVN(de)ITSEGKVK	1400.7875	IPI00113528	IPI00113528	yes	no	2	0.029883	82.85
IVEDIESFIKPYSVEEQKN(de)ITSCPDGAPFIQHGPDYR	4278.0579	IPI00124221	IPI00124221	yes	yes	4,5	7.6544E-15	107.95
IVGCPPGGAGGN(de)DTEIIACIR	2126.0249	IPI00128567	IPI00128567	yes	yes	3	0.013236	64.298
IVGGTN(de)ASIGEWPWQVSIQVK	2268.1903	IPI00113057	IPI00113057	yes	yes	2,3	6.6671E-49	196.4
IVGSPFIGCTVVN(de)K	1489.7963	IPI00134808	IPI00134808	yes	no	2,3	6.4695E-55	210.38
IVICQPGTEAEVTDEIMDTDVNCVPCKPGHFQN(de)TSSPR	4300.9497	IPI00116429	IPI00116429	yes	yes	4	2.1909E-08	79.286
IVIDPSGSMNIYIVIDGSDSIGSSN(de)FTGAK	3056.5012	IPI00114065	IPI00114065	yes	no	3	3.991E-05	80.355
IVIDPSGSMNIYIVIDGSDSIGSSN(de)FTGAKR	3212.6023	IPI00114065	IPI00114065	yes	no	4	0.0001237	67.778
IVIIIDPAISN(de)NSFSSNPYGPYDR	2651.3231	IPI00848693	IPI00848693	yes	yes	3,4	1.4815E-08	111.57
IVIQNG(de)AHEVFNHK	1604.8423	IPI00134704	IPI00134704	yes	yes	3	0.019958	73.881
IVIWTAEEQGGIGASQYYEIHKAN(de)ISK	3004.5294	IPI00126050	IPI00126050	yes	no	3,4	6.7981E-09	113.51
IVIYIEHNIEKN(de)STK	1799.9781	IPI00321190	IPI00321190	yes	no	2,3,4	2.1917E-11	160.47
IVIYIEHNIEKN(de)STKEEIIAAIEK	2796.5273	IPI00321190	IPI00321190	yes	no	2,3,4	2.0219E-108	230.71
IVKIPSGTIVISN(de)ATEGDGGIYR	2359.2747	IPI00129159	IPI00129159	yes	no	3	8.2875E-31	172.66
IVN(de)GSGPHQGR	1120.5738	IPI00322026	IPI00322026	yes	yes	2	0.016738	81.525
IVPCEFGVIYPIAEN(de)FSR	2110.0557	IPI00121908	IPI00121908	yes	no	2,3	0.0001263	111.33

IVPIENGEIVVSIVNGRPGAIN(de)FSYSPIIR	3222.7765	IPI00116913	IPI00116913	yes	yes	3	0.018203	54.3
IVQAQYWHDPIKESVYRN(de)YSIFIADINQER	3694.8532	IPI00331318	IPI00331318	yes	no	3,4	8.7472E-05	84.404
IVQDVAN(de)NTNEEAGDGTTTATVIAR	2559.2413	IPI00308885	IPI00308885	yes	no	3	0.0013663	74.251
IVSN(de)ASCTTNCIAPIAK	1818.8968	IPI00850337	IPI00850337	yes	no	2	2.7833E-09	146.81
IVTQTIPCN(de)K	1172.6223	IPI00307966	IPI00307966	yes	yes	2	1.0316E-31	196.27
IVTQTIPCN(de)KTIFWSK	1935.0288	IPI00307966	IPI00307966	yes	yes	2	5.0554E-25	179.81
IVVN(de)VSDKNDHAPWFTSPSYDGR	2603.2405	IPI00623114	IPI00623114	yes	no	3	0.0089119	72.145
IVVPINNREN(de)ISDPTSPIR	2133.1542	IPI00136925	IPI00136925	yes	no	2	1.6279E-08	140.89
IVVPINNREN(de)ISDPTSPIRR	2289.2553	IPI00136925	IPI00136925	yes	no	3	2.7087E-05	124.46
IVYN(de)HSSGR	1031.5148	IPI00133500	IPI00133500	yes	yes	2	0.013288	101.65
IWDPSSHPGIQN(de)ISR	1705.8536	IPI00338209	IPI00338209	yes	no	2,3	8.9377E-12	159.18
IWIPVN(de)ITWADIEDK	1811.9458	IPI00126253	IPI00126253	yes	no	3	6.0864E-05	126.1
IWIPVN(de)ITWADIEDKDGR	2140.0953	IPI00126253	IPI00126253	yes	no	2,3	9.5323E-21	169.09
IWVHNGAPVN(de)ASASCR	1737.8369	IPI00464135	IPI00464135	yes	yes	2,3	0.0009048	113.59
IWVN(de)SSDGISPVIGK	1570.8355	IPI00848693	IPI00848693	yes	yes	2,3	6.0881E-34	197.17
IYAGMVSIMDEAVGNV(de)TK	1896.9325	IPI00652358	IPI00652358	yes	no	2,3	1.5425E-21	172.97
IYN(de)VTHQFCNASVMDPTCVR	2411.0821	IPI00132600	IPI00132600	yes	no	2,3	1.5365E-42	195.77
IYNVTHQFCN(de)ASVMDPTCVR	2411.0821	IPI00132600	IPI00132600	yes	no	2,3	1.5365E-42	195.77
IYPIEN(de)ITIAPDPHVPDSIPPVAYNPWMDIR	3542.7908	IPI01023131	IPI01023131	yes	no	3,4	0.0005101	61.911
IYSDQCHHN(de)ISIIPPPTEIVCNR	2762.3269	IPI00123442	IPI00123442	yes	yes	3	0.0009842	85.35
IYSDQCHHNISIIPPPTEIVCN(de)R	2762.3269	IPI00123442	IPI00123442	yes	yes	3	0.0009842	85.35
IYVIDGTQN(de)DTAFVFPR	1954.9789	IPI00308658	IPI00308658	yes	no	3	2.7878E-08	142.45
IYWISSGN(de)HTINR	1559.7845	IPI00119063	IPI00119063	yes	yes	2,3	5.9678E-42	202.29
IYWISSGNHTIN(de)R	1559.7845	IPI00119063	IPI00119063	yes	yes	2,3	5.9678E-42	202.29
IYWTDGDN(de)ISMANMDGSNHTIIFSGQK	3014.3539	IPI00119063	IPI00119063	yes	yes	3	4.5794E-20	146.06
IYWTDGDNISMANMDGSN(de)HTIIFSGQK	3014.3539	IPI00119063	IPI00119063	yes	yes	3	4.5794E-20	146.06
KAFITN(de)FSMIIDGVTYPGVVK	2299.2286	IPI00312711	IPI00312711	yes	no	2,3,4	1.9277E-16	155.03
KAFITN(de)FSMIIDGVTYPGVVKEK	2556.3662	IPI00312711	IPI00312711	yes	no	3,4,5	9.0579E-40	185.61
KAWGISVINPN(de)KTK	1554.8882	IPI00989096	IPI00989096	yes	yes	2,3	0.0001236	138.01
KCEAN(de)ITISSPAR	1445.7297	IPI00894972	IPI00894972	yes	no	2	0.012604	88.282
KCIQIQAIN(de)MSSCPIDSR	2120.0177	IPI00648288	IPI00648288	yes	no	3	0.000503	99.055
KCNSDN(de)ISYVIHCEPENNTK	2421.0689	IPI00323053	IPI00323053	yes	no	3	2.685E-05	122.44
KCNSDNISYVIHCEPEN(de)NTK	2421.0689	IPI00323053	IPI00323053	yes	no	3	2.685E-05	122.44
KCPN(de)CTETAVNGEIVVMYDVNREEK	2954.3572	IPI00970438	IPI00970438	yes	no	3,4	0.0006553	90.464
KCQEAIN(de)ATCK	1321.6119	IPI00314443	IPI00314443	yes	no	2,3	0.012042	93.839
KDGSQDFN(de)ETWENYEK	1988.8388	IPI00128206	IPI00128206	yes	yes	2	7.4495E-09	148.52
KDTCAQECSHFN(de)ITK	1837.8087	IPI00132474	IPI00132474	yes	no	2,3	2.0031E-11	158.17
KEDSCQIN(de)YSEGPCIGMQER	2400.0144	IPI00127352	IPI00127352	yes	yes	2,3	5.5189E-33	183.56
KEN(de)SSEICSNNGECVCGQCVCR	2646.0349	IPI00132474	IPI00132474	yes	no	3,4	8.1454E-59	198.96
KEN(de)SSYQVINWR	1522.7528	IPI00153316	IPI00153316	yes	yes	3	0.0005041	141.1
KENG(de)VFEEISNSSGR	1651.7802	IPI00757771	IPI00757771	yes	no	3	0.01607	84.566
KENGVFEEISNS(de)SGR	1651.7802	IPI00757771	IPI00757771	yes	no	3	0.01607	84.566
KEQETCIAPEIEHGN(de)YSTTQR	2490.1445	IPI00122117	IPI00122117	yes	yes	3	2.9769E-30	171
KEVHFFVNA(de)SDVDSVK	1819.9105	IPI00310049	IPI00310049	yes	yes	3	9.4799E-05	126.77
KFHVN(de)YTQPIVAVK	1642.9195	IPI00123704	IPI00123704	yes	no	2	6.0188E-05	139.86
KFPVPFQKEN(de)VTATIVEIGR	2272.258	IPI00134691	IPI00134691	yes	yes	2,3,4	1.5033E-61	176.94
KGCADYCN(de)QTITK	1557.6916	IPI00987265	IPI00987265	yes	no	2,3	5.2235E-06	149.69
KGIVSGGVYNSHVGCIPYTIPPCEHHVN(de)GSRPPCTGEGDTPF	4558.1329	IPI00113517	IPI00113517	yes	yes	5	0.0018523	52.931
KGN(de)NTINPFIIPSASDVPTGCPNQWWPYAGHCYR	3916.8202	IPI00126186	IPI00126186	yes	yes	4	3.9711E-05	68.942
KGQNMVCGGMGCDN(de)DSIVQQIFNAAEIDTYTR	3591.5851	IPI00132600	IPI00132600	yes	no	3,4,5	4.0397E-59	184.03
KGYW(de)ASYNIPFHKTIYNWSGYPIIVHK	3281.6815	IPI00471081	IPI00471081	yes	yes	5	0.023579	55.734
KIDFIVIN(de)ETR	1346.7558	IPI00125813	IPI00125813	yes	no	2,3	0.000401	121.04
KIDNEVIQIISGN(de)ATITIIAMR	2412.341	IPI00126834	IPI00126834	yes	no	3	3.638E-23	166.57
KIIASPNEEN(de)MTEIISMR	2075.0391	IPI00322575	IPI00322575	yes	no	2,3	2.5698E-37	191.93
KIN(de)ASIVTSFVEIPIVSNVSIR	2385.3631	IPI00323857	IPI00323857	yes	no	2,3,4	1.9439E-39	186.06
KIN(de)CSQEVPGSSQCDREPEPR	2472.1122	IPI00881836	IPI00881836	yes	yes	3	0.0097365	75.239
KIN(de)TTNGKSDFQVPCQYSQQIK	2583.2751	IPI00126188	IPI00126188	yes	yes	3	3.9604E-07	116.6
KINASIVTSFVEIPIVSN(de)VSIR	2385.3631	IPI00323857	IPI00323857	yes	no	2,3,4	1.9439E-39	186.06
KINIDGS(de)NYTIIK	1477.814	IPI00119063	IPI00119063	yes	yes	2,3	0.0007241	135.02
KIPIN(de)FTEGAR	1244.6877	IPI00129243	IPI00129243	yes	no	2	0.014254	91.961
KIQIN(de)NSENTIR	1428.7685	IPI00223446	IPI00223446	yes	yes	2	0.0077503	96.82
KISTN(de)ITIVCKPGDIESAPVIR	2410.3254	IPI00308971	IPI00308971	yes	yes	3,4	0.0004195	93.92
KISTSPFAIN(de)ITMIPK	1759.9906	IPI00666034	IPI00666034	yes	no	3	4.6424E-05	131.86
KITN(de)ITEAISDIPR	1569.8726	IPI00342691	IPI00342691	yes	yes	3	0.0001967	128.31
KIVIDPSGSMNIYIVIDGSDSIGSS(de)NFTGAK	3184.5962	IPI00114065	IPI00114065	yes	no	4	0.01217	50.986
KIVIYIEHNIEK(de)NSTK	1928.0731	IPI00321190	IPI00321190	yes	no	3	0.064161	68.972
KKENGVFEEISN(de)SSGR	1779.8751	IPI00757771	IPI00757771	yes	no	2,3	3.312E-12	159.18
KKEVHFFVN(de)ASDVDSVK	1948.0054	IPI00310049	IPI00310049	yes	yes	3	0.006928	86.548
KKQEIPSN(de)CSIPYHVYNIK	2317.1889	IPI00311305	IPI00311305	yes	yes	3	6.7519E-05	121.82
KMVIWN(de)DSTIR	1361.7126	IPI00135975	IPI00135975	yes	yes	3	0.0070768	99.283
KN(de)ISEIWDAYCYR	1716.793	IPI00987265	IPI00987265	yes	no	3	0.0002954	142.1
KN(de)ITDIVEGAKK	1314.7507	IPI00649186	IPI00649186	yes	no	3	0.064325	77.597
KN(de)ITSPVGVQPIINEHTFCAGITK	2623.3792	IPI00409148	IPI00409148	yes	yes	3	9.0301E-13	126.91
KN(de)SSQIAFIIYNDQPPK	1962.0211	IPI00114236	IPI00114236	yes	no	3	0.0005031	118.2
KNIQAVNEIIATISQC(de)NDTSSAAMVQCIR	3234.5795	IPI00138342	IPI00138342	yes	no	3,4,5	5.9424E-07	98.684
KNPEAHMN(de)VSEIIK	1608.8294	IPI00652605	IPI00652605	yes	no	3	0.010063	86.803
KNV(de)NISYTVNDSFFPQRPQK	2381.2128	IPI00381178	IPI00381178	yes	yes	2,3,4,5	3.4769E-111	250.08
KNVNISYTV(de)NDSFFPQRPQK	2381.2128	IPI00381178	IPI00381178	yes	yes	2,3,4,5	3.4769E-111	250.08
KPIIGHYKPDTIAVVIEN(de)GTSIDR	2635.4334	IPI00410796	IPI00410796	yes	no	3,4	6.6646E-08	109.85
KPISQEFEAYVN(de)ASGEHGIVVFSIGSMVSEIPEKK	3805.9237	IPI00134691	IPI00134691	yes	no	4,5	7.3655E-12	100.58
KQIIN(de)ISQIVYGPEGSDIVIIK	2426.3785	IPI00116509	IPI00116509	yes	no	3	1.7501E-07	113.86
KQIN(de)ITTVVAPHEAISR	1876.053	IPI00808027	IPI00808027	yes	no	3	0.0066438	83.54
KQPSPSPGN(de)TTGGPIPGSAASPAHQASQPIYINHP	3460.7124	IPI01027336	IPI01027336	yes	no	4	5.8239E-07	67.367
KQVTPIFFYFQ(de)NR	1686.8882	IPI00319509	IPI00319509	yes	no	2,3	0.0010961	119.39
KSCHTAVGTSEGWNVPMGIIY(de)NQTGSCK	3081.4107	IPI00471080	IPI00471080	yes	no	3,4	0.0001751	84.947
KSDTQNIIYN(de)VSTGR	1694.8588	IPI01023214	IPI01023214	yes	no	3	0.0001284	125.97

KTCNPETFPIR(de)NESIQCPTAR	2518.2057	IPI00649186	IPI00649186	yes	no	3	0.0002474	94.882
KTGEA(de)NITQIYTQEAIDFIQTQHAR	2875.4464	IPI00310090	IPI00310090	yes	no	3,4	3.7571E-46	188.23
KTGVHDGDFEY(de)NITTTIAAINK	2407.202	IPI00108849	IPI00108849	yes	no	3,4	7.4368E-12	142.97
KTHMFN(de)FTSGFK	1443.6969	IPI00121274	IPI00121274	yes	yes	3	0.01058	93.011
KTN(de)QSCEIVIDSTEK	1750.8407	IPI00310059	IPI00310059	yes	no	2,3	1.1294E-11	162.41
KTN(de)QSCEIVIDSTEKVNPSYIGR	2637.3068	IPI00310059	IPI00310059	yes	no	3	1.7125E-30	168.27
KTTIEN(de)FTCPEYK	1629.7709	IPI00420810	IPI00420810	yes	yes	2,3	0.008624	94.122
KTYAVYDIFDTAMIN(de)NSR	2121.0201	IPI00226790	IPI00226790	yes	no	2,3	2.8563E-08	139.08
KVC(de)NGIGIGEFKDTISINATNIK	2491.3105	IPI00121190	IPI00121190	yes	no	3,4	2.5947E-05	104.38
KVEAEAIN(de)ATAIR	1384.7674	IPI00754853	IPI00754853	yes	no	2	0.014575	85.457
KVEVEAVN(de)ATAVK	1356.7613	IPI00608063	IPI00608063	yes	no	2,3	1.4212E-09	159.22
KVEVEPIN(de)STAVHVSWK	1922.0262	IPI00110264	IPI00110264	yes	yes	2,3	2.098E-08	145.48
KVIIN(de)NSIDEPR	1396.7674	IPI00411145	IPI00411145	yes	no	3	0.0061851	99.752
KVIVAPPSEEA(de)NTTK	1582.8566	IPI00875419	IPI00875419	yes	no	2	0.0055355	93.51
KVPS(de)NSTETVIESDQFQPGVR	2317.155	IPI00119299	IPI00119299	yes	no	2,3	1.0087E-11	143.67
KVYEEVIN(de)VTPNDGFAK	1921.9785	IPI00624896	IPI00624896	yes	no	2,3	0.0004885	118.48
KWGH(de)NVTEFQQR	1528.7535	IPI00754386	IPI00754386	yes	yes	2,3	0.0016919	128.08
KWPERISAI(de)DNIINHSSIFIK	2480.354	IPI00123342	IPI00123342	yes	yes	3,4	3.6478E-07	117.26
KYEQAK(de)NISQDIEK	1692.8683	IPI00400016	IPI00400016	yes	no	2	1.1623E-10	164.41
KYFDQVDIS(de)NGIDWSIDHK	2279.0859	IPI00133456	IPI00133456	yes	yes	3	2.9063E-06	132.51
KYFSIIKN(de)QTVPFIEEDVR	2325.2369	IPI00153202	IPI00153202	yes	no	3	0.0001609	105.39
KYHDYYITSTSN(de)GSIEGIENR	2446.1401	IPI00130754	IPI00130754	yes	yes	3	1.3519E-08	129.46
KYQTIN(de)CSVNVR	1480.7456	IPI00420835	IPI00420835	yes	no	3	0.0012635	123.67
MAAAIN(de)ATGRPIAFSCSWPAYEGGIPPKVNYTEVSR	3880.9029	IPI00315593	IPI00315593	yes	no	4	3.7463E-05	68.942
MAAAINATGRPIAFSCSWPAYEGGIPPKVN(de)YTEVSR	3880.9029	IPI00315593	IPI00315593	yes	no	4	3.7463E-05	68.942
MAIIQYGSQNQQQVAFPITYN(de)VTTIHEAIER	3562.7879	IPI00621027	IPI00621027	yes	yes	3,4,5	7.2267E-59	178.53
MAIN(de)SSTEDGIK	1264.5969	IPI00129168	IPI00129168	yes	yes	2	0.015839	80.312
MCQIYPETFMCVTN(de)SSDIIQAFR	2810.2536	IPI00134390	IPI00134390	yes	yes	3	0.0021234	73.21
MDFIIFN(de)YSAPSYIR	1835.8916	IPI00553503	IPI00553503	yes	yes	2	4.1919E-06	140.96
MDGHCAPIRTEAGVFE(de)YVADPTFENFTGGVK	3414.5649	IPI00405742	IPI00405742	yes	no	4	4.7442E-06	83.934
MDGHCAPIRTEAGVFE(de)YVADPTFENFTGGVKK	3542.6599	IPI00405742	IPI00405742	yes	no	3,4,5	2.0316E-07	94.73
MEAHN(de)VSAPFNFSIPPGFGHR	2311.0957	IPI00115482	IPI00115482	yes	no	2,3,4	6.4322E-17	155.79
MEAHNVSAPFN(de)FSIPPGFGHR	2311.0957	IPI00115482	IPI00115482	yes	no	2,3,4	6.4322E-17	155.79
MEISVGAIQAN(de)R	1287.6605	IPI00108811	IPI00108811	yes	yes	2	0.0009151	111.94
MFSQN(de)DTR	997.42873	IPI00129250	IPI00129250	yes	yes	2	0.0074272	128.38
MGCQHHCVPTPSGPTCYCN(de)SSFQIQADGK	3323.3675	IPI00119063	IPI00119063	yes	no	3,4	2.1131E-10	115.79
MGDREAIGN(de)ASQIFDSWIK	2137.0262	IPI00134585	IPI00134585	yes	yes	2,3	6.0726E-20	166.77
MGFNTINSIISN(de)NSK	1638.8036	IPI00454124	IPI00454124	yes	no	2	4.0659E-06	141.37
MHING(de)SNVQVIHR	1503.7729	IPI00119063	IPI00119063	yes	yes	2,3,4	2.8261E-15	172.13
MIEN(de)GSISFIPTIR	1576.8283	IPI00123194	IPI00123194	yes	yes	2,3	3.4988E-32	193.47
MIHN(de)TTGVYTCSAHGTWTNEVIKR	2775.3221	IPI00475209	IPI00475209	yes	no	3	3.5007E-05	99.092
MITNEKIDAYNEA(de)AVSIINSSTR	2539.2588	IPI00380296	IPI00380296	yes	yes	3	0.019556	64.522
MNIQN(de)ISIVISPTVQEFIINCIHR	2838.4884	IPI00850930	IPI00850930	yes	yes	3	0.0084218	72.771
MNPAGPVIEN(de)ETVTIICSTPK	2270.1287	IPI00985818	IPI00985818	yes	no	2,3	0.0084538	68.369
MPFPIDQDFYVSPTFQDIIN(de)R	2542.2202	IPI00318175	IPI00318175	yes	yes	3	7.9562E-08	120.11
MPSQASAGNVYPQPIIN(de)SSMCIEDSR	2851.2939	IPI00224752	IPI00224752	yes	yes	3,4	1.5709E-17	146.25
MQVVSN(de)GTVTTAIWR	1661.8559	IPI00331175	IPI00331175	yes	no	2,3	4.6763E-06	140.22
MRSPGAQ(de)DNVSVSQGMR	1818.8465	IPI00348586	IPI00348586	yes	no	3	3.9912E-11	152.94
MSPWSN(de)WSECDPCIK	1895.7641	IPI00230718	IPI00230718	yes	no	2,3	0.0002919	116.43
MSQHPIDIQEDFNN(de)ATIVNASSIIPK	2881.428	IPI00762812	IPI00762812	yes	yes	3	0.0002723	86.946
MSQHPIDIQEDFNNATIVN(de)ASSIIPK	2881.428	IPI00762812	IPI00762812	yes	yes	3	0.0002723	86.946
MTISQN(de)NSIIR	1275.6605	IPI00108535	IPI00108535	no	no	2	8.5268E-10	160.52
MTISSIKDDIGACRN(de)CCSVTK	2415.1015	IPI00223352	IPI00223352	yes	yes	3	0.0013683	88.385
MTQESAIIFPFIN(de)K	1637.8487	IPI00857780	IPI00857780	yes	no	3	0.0117	79.466
MVN(de)TTFICTATNAVGTGR	1912.9135	IPI00109375	IPI00109375	yes	no	2,3	5.8739E-123	251.41
MVSIDIPYFADVVVPIN(de)MTMK	2382.2037	IPI00411145	IPI00411145	yes	no	3	0.0050164	69.081
MYSEGSDIVPQSN(de)ETAIHYFK	2415.1053	IPI00131143	IPI00131143	yes	no	2,3	3.1457E-23	166.47
MYSEGSDIVPQSN(de)ETAIHYFKK	2543.2002	IPI00131143	IPI00131143	yes	no	2,3	4.7068E-39	178.43
MYVTN(de)DTEVAENNYEAIK	2102.9467	IPI00658539	IPI00658539	yes	no	2	2.7866E-24	131.31
MYVTN(de)DTEVAENNYEAIKDFFR	2668.2115	IPI00658539	IPI00658539	yes	no	2,3	1.2839E-07	120.24
N(de)ATIVNEADKIR	1342.7205	IPI00319509	IPI00319509	yes	no	2	0.0018635	117.7
N(de)ATTYPPMCSQDAAR	1681.7188	IPI00128399	IPI00128399	yes	yes	2,3	4.9387E-05	126.48
N(de)GSIFAFR	910.4661	IPI00129240	IPI00129240	yes	no	2	1.6447E-10	151.44
N(de)GTNDGEYVFITGEDNYINFSK	2496.1081	IPI00127447	IPI00127447	yes	yes	3	6.8312E-17	155.86
N(de)GTSFDIHYGSGSISGYISQDTVSVPCK	2975.3607	IPI00404551	IPI00404551	yes	no	3	0.000174	77.939
N(de)ISEGFSGIPDNVDAAFAIPAHR	2397.1713	IPI00129240	IPI00129240	yes	yes	3	6.7066E-08	107.41
N(de)MTIFSDIVAEK	1366.6803	IPI00621027	IPI00621027	yes	no	2	0.000636	118.13
N(de)STICDICIGPIK	1489.7269	IPI00139788	IPI00139788	yes	no	2	1.551E-06	152.88
N(de)STIQAANIAGIK	1299.7147	IPI00222937	IPI00222937	yes	no	2	0.0004592	132.31
N(de)STTIVMHMK	1160.5682	IPI00119063	IPI00119063	yes	yes	2	0.016568	87.184
N(de)TTISVHPSTR	1211.6258	IPI00126834	IPI00126834	yes	no	2	0.0004336	118.76
N(de)TTSAAMVHCIR	1359.6387	IPI00131216	IPI00131216	yes	yes	2	0.016321	79.492
N(de)TTSYPPMCSQDAVGGQVISEIFTNR	2871.3167	IPI00387289	IPI00387289	yes	yes	3	2.3522E-45	181.82
N(de)TTSYPPMCSQDAVGGQVISEIFTNRK	2999.4117	IPI00387289	IPI00387289	yes	yes	3,4	5.4547E-05	93.804
N(de)VTIECPFKR	1262.6441	IPI00310059	IPI00310059	yes	no	2	0.0003408	123.79
NACCSVN(de)TSQEIHK	1646.7141	IPI00113158	IPI00113158	yes	yes	2	0.015582	76.358
NAIN(de)ITFHAQNIGEGGAYEAEIR	2487.2142	IPI00115976	IPI00115976	yes	yes	2,3,4	1.6817E-17	155.87
NAIQAFGN(de)GTDVNMSPK	1762.8308	IPI00116355	IPI00116355	yes	no	2	2.8381E-15	162.26
NAKGDEKEN(de)ITAEAIDISIK	2158.1117	IPI00124725	IPI00124725	yes	no	2,3	5.8352E-05	115.57
NAN(de)CSTVSPGQTQCTCQK	2039.8459	IPI00467600	IPI00467600	yes	yes	3	0.016514	67.416
NATIAEQAKI(de)PATEKPVIISK	2221.2682	IPI00123342	IPI00123342	yes	yes	3	3.9779E-07	116.29
NAVN(de)CTYKNEDDCVVR	1955.8466	IPI00266264	IPI00266264	yes	yes	2	0.0065233	88.992
NCGVN(de)CSGDVFTAIIGEISSPNYPNPYPENSR	3527.5722	IPI00653675	IPI00653675	yes	no	4	4.1484E-07	85.595
NCKPIIIDN(de)VSFFGVK	1849.976	IPI00122581	IPI00122581	yes	no	3	6.3176E-18	170.11
NCKPIIIDN(de)VSFFGVKDK	2093.0979	IPI00122581	IPI00122581	yes	no	3	0.0005136	105.39
NEDRPIAPCGAIANSMFN(de)DTIEIYIVANESDPKPIPIPIK	4422.224	IPI00387318	IPI00387318	yes	no	4,5	9.2358E-15	91.942

NEDRPIAPCGAIANSMFNDTIEIYIVAN(de)ESDPKPIPIPIK	4422.224	IPI00387318	IPI00387318	yes	no	4,5	9.2358E-15	91.942
NEIMIN(de)SSIMR	1306.6373	IPI00378698	IPI00378698	yes	no	2	0.028723	71.176
NEIQVN(de)TSQIPDTVTHYSEIYHDFTTTQVGMHDIVR	4187.9858	IPI00128432	IPI00128432	yes	yes	4	9.2989E-06	66.378
NF(de)TAADWGHSR	1260.5636	IPI00339885	IPI00339885	yes	no	2	0.0009286	110.31
NFKDITNIIN(de)NTFIR	1821.9737	IPI00650038	IPI00650038	yes	no	3	0.0005541	120.84
NFQCVN(de)ASVINYDCDIQGK	2243.994	IPI00626485	IPI00626485	yes	no	2,3	9.8581E-147	265.22
NGDAYG(de)YYNDSIK	1478.6314	IPI00471081	IPI00471081	yes	yes	2	0.0002542	141.85
NGDAYGYYN(de)DSIK	1478.6314	IPI00471081	IPI00471081	yes	yes	2	0.0002542	141.85
NGIASGSGN(de)CSTGPNGDGSFHAWSIIEVK	2918.3253	IPI00322497	IPI00322497	yes	yes	3,4	2.9539E-41	172.37
NGIYN(de)ITVIAIDADGR	1703.8842	IPI00138209	IPI00138209	yes	no	2,3	7.1124E-15	167.82
NGN(de)WSEPPMCIHACVIPENIMEAHNIIIK	3386.6032	IPI01008676	IPI01008676	yes	no	4	0.0093343	53.998
NGNA(de)TIIR	857.47191	IPI00985828	IPI00109996	no	no	2	0.014243	120.99
NGNATI(de)IR	857.47191	IPI00985828	IPI00109996	no	no	2	0.014243	120.99
NGTNDGEYVFITGEDNYIN(de)FSK	2496.1081	IPI00127447	IPI00127447	yes	yes	3	6.8312E-17	155.86
NI(de)KEEKANVVASAIAQIPQK	2150.2059	IPI00153143	IPI00153143	yes	yes	3	1.7291E-13	150.91
NI(de)TSPVGVQPIINEHTFCAGITK	2495.2842	IPI00409148	IPI00409148	yes	yes	3	0.0001632	93.269
NIETFAMN(de)ICEHFISSFNHVTR	2666.237	IPI00223367	IPI00223367	yes	no	3,4	9.5126E-08	121.26
NIFIN(de)HSETASAK	1430.7154	IPI00409148	IPI00409148	yes	yes	2,3	1.7616E-217	216.88
NIGGIETEDDYGYQGHVQTCN(de)FSAQMAK	3132.3553	IPI00126769	IPI00126769	yes	yes	3,4	2.2353E-06	101.41
NIGPN(de)TTSTTITSDDFKPGVR	2220.1022	IPI00118291	IPI00118291	yes	no	3	0.0056888	79.393
NIIGIDENFVIN(de)SSTEHR	2057.0178	IPI00406603	IPI00406603	yes	yes	2,3	1.9432E-21	172.84
NIINDYVSN(de)QTQGMIK	1836.904	IPI00131830	IPI00131830	yes	no	2,3	1.4163E-96	232.53
NIINDYVSN(de)QTQGMIKEIISEIDER	2921.4441	IPI00131830	IPI00131830	yes	no	3,4	1.7511E-44	180.04
NIIPN(de)ITTSYIK	1375.7711	IPI00471089	IPI00471089	yes	no	2	0.0013829	106.26
NIKRPYIVPII(de)WINETGTIGDEK	2668.4588	IPI00331214	IPI00331214	yes	yes	3	5.0096E-12	142.98
NIMIDIQKDTAVEGEEIEVN(de)CTAMASKPATTIR	3647.7845	IPI00856723	IPI00856723	yes	no	3,4	3.386E-11	108.49
NIPWYVIAGNHDHIGN(de)VSAQIAYSK	2766.3878	IPI00137491	IPI00137491	yes	yes	2,3,4	1.1546E-16	144.27
NIQAVNEIIATISQCN(de)DTSSAAMVQCIR	3106.4846	IPI00138342	IPI00138342	yes	no	2,3,4	2.7369E-51	183.07
NIQVYN(de)ATSNSITVK	1650.8577	IPI00121430	IPI00121430	yes	no	2	3.9948E-70	222.45
NITAFN(de)ETIFR	1324.6776	IPI00124700	IPI00124700	yes	yes	2	3.3668E-06	149.23
NITVI(de)EPVTQPFIQVTNTTVK	2341.2893	IPI00108535	IPI00108535	yes	no	3	4.6767E-50	195.46
NITVIEPVTQPFIQVTN(de)TTVK	2341.2893	IPI00108535	IPI00108535	yes	no	3	4.6767E-50	195.46
NKANIQFGEN(de)GTTISAVTNK	2106.0705	IPI00127447	IPI00127447	yes	yes	2,3	1.116E-41	191.78
NKNPQMN(de)FTEANEACK	1894.8302	IPI00308245	IPI00308245	yes	yes	3	3.6323E-25	182.02
NMICQWDPGRETYIETN(de)YTIK	2631.2098	IPI00120155	IPI00120155	yes	yes	3	3.7084E-11	137.69
NMPPRIPANTMQIFIDN(de)NSIEGIPENYFNVIPK	3785.8909	IPI00129867	IPI00129867	yes	no	4	0.0019952	57.803
NN(de)DSTINNSATTIIIGIQDCR	2319.1125	IPI00881745	IPI00881745	yes	no	3	1.0793E-05	103.71
NNDSTIN(de)NSATTIIIGIQDCR	2319.1125	IPI00881745	IPI00881745	yes	no	3	1.0793E-05	103.71
NNQT(de)ECFNHVR	1417.6157	IPI00124666	IPI00124666	yes	no	2	0.0092184	88.338
NPCNITREDY(de)APIVK	1788.8829	IPI00307966	IPI00307966	yes	yes	2,3	6.7285E-06	143.93
NPCTSEQN(de)CTSPFSYK	1918.7826	IPI00132474	IPI00132474	yes	yes	2	4.1489E-13	161.91
NPIGQAQSAVVIN(de)VTER	1794.9588	IPI00119299	IPI00119299	yes	no	2,3	1.4749E-08	144.73
NPNN(de)NTIHPNIR	1402.7066	IPI00319509	IPI00319509	yes	no	2,3	0.0007839	122.39
NPSANTFIHINA(de)SSFR	1774.8751	IPI00315535	IPI00315535	yes	yes	2	3.5056E-05	129.43
NQIVEI(de)EKVVIHPNHSVVDIGIIK	2692.5276	IPI00409148	IPI00409148	yes	yes	3,4	5.9534E-27	165.19
NSFN(de)ISNIIVIHISHNK	1949.0483	IPI00122293	IPI00122293	yes	yes	3	0.0060494	78.505
NSQFDMN(de)STDIAIK	1582.7297	IPI00346062	IPI00346062	yes	yes	2	0.0001276	123.42
NTDKENPYIGIGN(de)YTICR	2127.0055	IPI00468236	IPI00468236	yes	no	3	0.000388	108.77
NTETNVF(de)PQDKVVIAGSNMTICCMSPTK	3141.4603	IPI00119299	IPI00119299	yes	no	3,4	1.6964E-51	187.16
NTGEIN(de)ITSIIDREETPYFIITGYAIDSR	3300.6514	IPI00115793	IPI00115793	yes	no	3	2.1473E-19	133.95
NTQADVIN(de)ASWSVISNSTR	2062.0079	IPI00132189	IPI00132189	yes	yes	2,3	1.6139E-52	199.99
NTQADVIN(de)ASWSVISNSTRHEIER	2726.3372	IPI00132189	IPI00132189	yes	yes	3	5.649E-05	101.49
NTQADVINASWSVISN(de)STR	2062.0079	IPI00132189	IPI00132189	yes	yes	2,3	1.6139E-52	199.99
NTQADVINASWSVISN(de)STRHEIER	2726.3372	IPI00132189	IPI00132189	yes	yes	3	5.649E-05	101.49
NTQIIPVSEAIPHESFDN(de)ETIVNDIMIIK	3279.6697	IPI00120351	IPI00120351	yes	yes	3	8.502E-07	103.25
NVAIVAGDTGN(de)ATGIGEQGPTR	2097.0451	IPI00122272	IPI00122272	yes	no	2,3	0	381.1
NVN(de)ISYIVNDSFFPQRPEK	2266.1382	IPI00122557	IPI00122557	yes	no	2,3,4	1.2004E-109	248.13
NVN(de)ISYTVNDSFFPQR	1899.9115	IPI00381178	IPI00381178	yes	yes	2	0.0016383	92.977
NVN(de)ISYTVNDSFFPQRPQK	2253.1178	IPI00381178	IPI00381178	yes	yes	2,3,4	4.1645E-299	326.53
NVN(de)ISYTVNDSFFPQRPQKIIANK	2792.461	IPI00381178	IPI00381178	yes	yes	3	0.0008894	90.972
NVN(de)MSVICPTTIR	1503.7538	IPI00129867	IPI00129867	yes	no	2	1.4562E-15	174.95
NVNISYIVNDS(de)FFPQRPEK	2266.1382	IPI00122557	IPI00122557	yes	no	2,3,4	1.2004E-109	248.13
NVNISYTVN(de)DSFFPQR	1899.9115	IPI00381178	IPI00381178	yes	yes	2	0.0016383	92.977
NVNISYTVN(de)DSFFPQRPQK	2253.1178	IPI00381178	IPI00381178	yes	yes	2,3,4	4.1645E-299	326.53
NVNISYTVN(de)DSFFPQRPQKIIANK	2792.461	IPI00381178	IPI00381178	yes	yes	3	0.0008894	90.972
NVPGEIGINYN(de)ITADVAQK	2015.0324	IPI00130117	IPI00130117	yes	yes	3	4.1244E-05	106.57
NVTVV(de)IR	799.49159	IPI00469218	IPI00469218	yes	yes	2	0.055841	86.794
NVVN(de)ITINDEAAMVEAVAIYNPVSFAFEVTEDFIMYK	4166.0268	IPI00118987	IPI00118987	yes	yes	3,4	0.0042385	44.021
NYEVQIFHVN(de)ATVTEEGTGIEFSR	2739.314	IPI00123223	IPI00123223	yes	yes	2,3	1.2773E-206	284.98
NYFHYN(de)QSFPPSYNIK	2017.9323	IPI00129265	IPI00129265	yes	yes	2,3	2.8854E-32	142.1
NYKNPN(de)ITISFTAER	1766.8951	IPI00132600	IPI00132600	yes	no	2,3	0.0004251	122.63
NYN(de)FTIACNTK	1344.6132	IPI00322575	IPI00322575	yes	no	2,3	0.0002402	142.83
NYN(de)FTIACNTKR	1500.7143	IPI00322575	IPI00322575	yes	no	2	0.017394	86.313
NYYADN(de)QTCDGEIIFNMTK	2295.9776	IPI00828222	IPI00828222	yes	no	3	0.0002346	94.845
NYYADN(de)QTCDGEIIFNMTKK	2424.0726	IPI00828222	IPI00828222	yes	no	3	7.1416E-05	102.78
NYYADNQTCDGEIIFN(de)MTK	2295.9776	IPI00828222	IPI00828222	yes	no	3	0.0002346	94.845
NYYADNQTCDGEIIFN(de)MTKK	2424.0726	IPI00828222	IPI00828222	yes	no	3	7.1416E-05	102.78
PGHDEGAHNPDYEN(de)ITIAFR	2252.0247	IPI00112047	IPI00112047	yes	yes	2	0.0093657	69.935
QAIQTMQSEFFYITTNIIN(de)DTIEIR	2988.4903	IPI00118130	IPI00118130	yes	yes	3	1.4606E-183	191.65
QAVIQVN(de)QSFVSSTAQK	1833.9585	IPI00356462	IPI00356462	yes	no	3	0.008099	75.018
QCDREHDCKDMSDEIGCVN(de)VTQCDGPNK	3366.3428	IPI00312063	IPI00312063	yes	no	4	0.00232	68.445
QCQEQIKEVN(de)KTCEAIIFK	2365.177	IPI00115116	IPI00115116	yes	yes	3	4.0028E-06	133.25
QDIAISGN(de)ISSIYAMTQDK	2053.999	IPI00405742	IPI00405742	yes	no	2,3	9.5196E-128	255.82
QEHSQIVIYHQSIN(de)ETISK	2253.139	IPI00356462	IPI00356462	yes	no	3	0.00123	86.213
QEIN(de)DSIQVAER	1400.6896	IPI00320420	IPI00320420	yes	yes	2	8.4348E-31	190.37
QENNIIIINAEHGN(de)SSIFIENSTFESFGYHSVSPDR	4064.9141	IPI00125813	IPI00125813	yes	no	4	2.0208E-11	95.108

QENNIIIINAEHGNSSIFIEN(de)STFESFGYHSVSPDR	4064.9141	IPI00125813	IPI00125813	yes	no		4	2.0208E-11	95.108
QETFGAIDIGGASTQITFVPQN(de)STIESPENSIQFR	3781.8435	IPI00626950	IPI00626950	yes	no		4	0.0019344	51.617
QEVMFITNVN(de)SSSSSTQIYQAVSR	2675.2861	IPI00112614	IPI00112614	yes	yes		3	0.017627	55.228
QFAPSPNCN(de)KTEIIATIK	2031.0459	IPI00776098	IPI00776098	yes	no		3	0.0003672	110.97
QFFN(de)ASVQFDNMDPIIDYINQR	2674.2486	IPI00117842	IPI00117842	yes	yes		3	3.237E-12	143.52
QFWIFDVQNPDDVAKN(de)SSK	2237.0753	IPI00331214	IPI00331214	yes	yes	2,3		1.9129E-65	218.25
QGDQYSCMVGHEAIPMN(de)FTQK	2440.061	IPI00473880	IPI00473880	yes	no	2,3,4		1.2346E-07	111.4
QGPQAGGTTITIN(de)GTHIDTGSK	2153.0713	IPI00405742	IPI00405742	yes	no		3	9.8711E-08	111.66
QGPQAGGTTITIN(de)GTHIDTGSKEDVR	2652.3103	IPI00405742	IPI00405742	yes	no	3,4		1.3465E-08	113.36
QHGQFSIAVVGINIT(de)SIGIR	2109.1695	IPI00121190	IPI00121190	yes	no		3	0.0012629	84.375
QIGASPSDDIIFGVFAQSKPDSAEPVN(de)R	2944.4567	IPI00130420	IPI00130420	yes	no	3,4		0.0012317	72.316
QIIAN(de)SSAIEETIIGHQGR	2036.0651	IPI00116913	IPI00116913	yes	no	2,3		1.8129E-191	290.57
QIINAIQIN(de)NTAVGHAIVIPAR	2325.3281	IPI00830749	IPI00830749	yes	yes		3	4.0075E-72	213.99
QIINAIQIN(de)NTAVGHAIVIPARR	2481.4292	IPI00830749	IPI00830749	yes	yes		4	5.5646E-08	117.3
QIIQTQVASPAIHPPVSYN(de)DTAPR	2602.3503	IPI00128989	IPI00128989	yes	no		3	1.4548E-20	148.01
QIITEEKIPN(de)NTQWITWSPEGHK	2748.3871	IPI00125813	IPI00125813	yes	no	2,3,4,5		2.9935E-277	318.69
QIITEEKIPN(de)NTQWITWSPEGHKIAYVWK	3508.8143	IPI00125813	IPI00125813	yes	no		4	5.4588E-06	100.79
QIN(de)ISDSIAVIGIAK	1540.8825	IPI00463492	IPI00463492	yes	yes		2	4.1867E-12	163.8
QIN(de)ISDSIAVIGIAKPAHIYSK	2337.3056	IPI00463492	IPI00463492	yes	yes	3,4		1.5388E-08	127.32
QIN(de)ISIIVK	1026.6437	IPI00115116	IPI00115116	yes	yes		2	0.0029289	128.03
QIN(de)ITTEDDDIYHMTVPYGRPR	2633.2544	IPI00265291	IPI00265291	yes	yes		3	6.6294E-05	97.32
QINAYN(de)R	877.44061	IPI00473190	IPI00473190	yes	no		2	0.011951	104.11
QINNGEIQPISEN(de)TTITFMSTK	2465.2108	IPI00126834	IPI00126834	yes	no		3	5.1899E-49	195.53
QINPPHTAN(de)SSITSK	1593.8111	IPI00988390	IPI00988390	yes	yes		3	0.0035626	88.552
QISCVANQN(de)GSQADCEIGNPFKR	2592.1809	IPI00420835	IPI00420835	yes	no		3	7.5725E-08	114.55
QIWDWSKHN(de)ITITQGK	1954.0061	IPI00128358	IPI00128358	yes	no		2	3.724E-07	144.97
QIYPYN(de)SSNR	1240.5836	IPI00229820	IPI00229820	yes	yes		2	0.0015335	112.36
QKN(de)VNISYIVNDSFFPQRPEK	2522.2918	IPI00122557	IPI00122557	yes	no	3,4,5		2.4168E-85	223.11
QKNVNISYIVN(de)DSFFPQRPEK	2522.2918	IPI00122557	IPI00122557	yes	no	3,4,5		2.4168E-85	223.11
QN(de)FSFCR	957.41269	IPI00381303	IPI00381303	yes	yes		2	0.04925	89.142
QN(de)FSNITVSTEDQVK	1708.8268	IPI00129677	IPI00129677	yes	no		2	3.1963E-158	272.95
QN(de)ITQKR	886.49846	IPI00134585	IPI00134585	yes	no		2	0.016975	124.83
QNFSN(de)ITVSTEDQVK	1708.8268	IPI00129677	IPI00129677	yes	no		2	3.1963E-158	272.95
QNPMTIGNVVI(de)FDKVITNQESPYQNHTGR	3299.6357	IPI00125310	IPI00125310	yes	no	3,4		8.8437E-06	95.834
QPEKTD(de)AEINETARPISPVNPK	2433.25	IPI00408895	IPI00408895	yes	yes		3	0.010645	73.809
QPIYIN(de)CSCVTGGSASAK	1911.8819	IPI00309249	IPI00309249	yes	yes		2	2.4954E-48	202.96
QPKDCDFIEDGEERN(de)CTGK	2296.9689	IPI00127280	IPI00127280	yes	yes		3	1.6063E-05	126.25
QPNMN(de)ITQIK	1185.6176	IPI00310567	IPI00310567	yes	yes		2	0.020428	86.344
QQTECMVQQYSN(de)YSVNGEPVNGR	2687.1704	IPI00396840	IPI00396840	yes	no		3	9.8077E-08	115.42
QRYEDIVIN(de)ETINK	1733.8948	IPI00285117	IPI00285117	yes	no	2,3		0.0002491	130.07
QRYHGF(de)INTSYHR	1677.8124	IPI00154056	IPI00154056	yes	no		3	0.031567	82.362
QRYN(de)ITAK	992.54033	IPI00130661	IPI00130661	yes	yes		2	2.4655E-05	134.26
QSDYCWEIAPHMYFITPGQQPQPPITNIIIVN(de)K	3897.9222	IPI00322575	IPI00322575	yes	no	4,5		0.0003557	61.499
QSDYCWEIAPHMYFITPGQQPQPPITNIIIVN(de)KTGAK	4255.1235	IPI00322575	IPI00322575	yes	no	4,5		0.000407	59.792
QSN(de)GSIAVISSIAGK	1430.7729	IPI00115599	IPI00115599	yes	no	2,3		2.4411E-44	205.98
QSQPVHIIPMN(de)ETDHINMVFSNK	2678.2945	IPI00133500	IPI00133500	yes	yes	3,4		2.9836E-08	114.88
QSQPVHIIPMNETDHINMVFSN(de)K	2678.2945	IPI00133500	IPI00133500	yes	yes	3,4		2.9836E-08	114.88
QSTINTVDIYPMMCHIIGIKPHPNN(de)GTISHTK	3616.7953	IPI00225072	IPI00225072	yes	no		4	0.0001458	67.641
QTGDVTCN(de)CTDGR	1482.5827	IPI00119063	IPI00119063	yes	yes		2	0.0027074	100.4
QTPEYQN(de)R	1034.4781	IPI00154056	IPI00154056	yes	no		2	0.0087677	113.18
QTQVGIVQYGAN(de)VTHEFNINK	2359.1921	IPI00466371	IPI01026704	no	no	2,3		8.7219E-101	237.31
QTQVGIVQYGAN(de)VTHEFNINKYSSTEEVIVAANK	3750.8853	IPI00466371	IPI01026704	no	no		4	0.015319	47.919
QTTAMDFSYAN(de)ETVCWVHVGDSAAQTQIK	3257.4758	IPI00119063	IPI00119063	yes	no	3,4		8.4953E-10	114.01
QVAIQTFGN(de)QTSIIPAGGAGYK	2220.1539	IPI00228719	IPI00228719	yes	yes	2,3,4		1.2593E-30	168.3
QVEEIIVN(de)HTGIR	1506.8154	IPI00263041	IPI00263041	yes	yes		2	0.0009175	111.83
QVEIIEYPYHEQIAVVAPEPIITGHN(de)YTIK	3463.8028	IPI00223987	IPI00223987	yes	yes	3,4		7.6963E-42	171.3
QVSTIIN(de)NTDK	1231.6408	IPI00396840	IPI00396840	yes	no		2	6.9594E-07	155.86
QVTPIFFYFQN(de)R	1558.7932	IPI00319509	IPI00319509	yes	no	2,3		0.0005216	134.75
QYYN(de)SSGTQSQR	1417.6222	IPI00848991	IPI00848991	yes	no		2	0.016914	79.492
RAEDYGPVEVISHWHP(de)NITINIVDDHTPWVK	3636.8114	IPI00121627	IPI00121627	yes	yes		4	3.1519E-07	79.382
RAEDYGPVEVISHWHPNITI(de)NIVDDHTPWVK	3636.8114	IPI00121627	IPI00121627	yes	yes		4	3.1519E-07	79.382
RCTICDEEHGIEVETN(de)CTITQNTK	2907.2797	IPI00110896	IPI00110896	yes	yes		3	1.3095E-34	168.64
RDDIHPTIPAGQYFI(de)NITYNYPVHSFDGR	3405.6531	IPI00387318	IPI00387318	yes	no	3,4		2.1619E-22	143.32
RDDYRPTWTI(de)NQTEPVAGNYYPVNTR	3125.4955	IPI00381303	IPI00381303	yes	yes	3,4		1.5425E-13	133.74
RDQGN(de)VTDMASMK	1451.6497	IPI00471081	IPI00471081	yes	yes		3	9.3003E-07	155.86
RFANEYPN(de)ITR	1379.6946	IPI00130573	IPI00130573	yes	yes		2	0.0007781	134.99
RFHSDIN(de)ISESIIPAVIEK	2167.1637	IPI00831484	IPI00831484	yes	yes		3	0.000817	93.495
RFIQSAEFFN(de)YTVR	1776.8948	IPI00127406	IPI00127406	yes	yes		2	0.0079856	93.348
RFTAPDTIFA(de)NGSVYPPNEGFCPCR	2872.3061	IPI00857130	IPI00857130	yes	yes		3	0.0010092	83.251
RGCKDN(de)ATDSVPIR	1587.7787	IPI00119063	IPI00119063	yes	yes	2,3		0.0080521	101.3
RGDDIYTN(de)VTVSIVEAIVGFEMDITHIDGHK	3443.7031	IPI00320241	IPI00320241	yes	yes	3,4,5,6		1.91E-83	201.66
RGPECSQN(de)YTAPTGVIK	1876.9101	IPI00123996	IPI00123996	yes	yes	2,3		0.0007125	112.24
RGTFTDCAIAN(de)MTQQIR	1981.9462	IPI00621027	IPI00621027	yes	no	2,3		3.7007E-11	152.37
RGVFITN(de)ETGQPIIGK	1728.9523	IPI00111960	IPI00111960	yes	no	2,3		8.235E-09	147.56
RIAVDWESIGYN(de)ITR	1791.9268	IPI00123040	IPI00123040	yes	no	2,3		0.0018713	108.58
RIGAIN(de)NSIIIIEDR	1695.9632	IPI00115516	IPI00115516	yes	no	2,3		1.3033E-05	137.8
RIQEGHEYDTFDIN(de)DTAQCFTK	2687.1922	IPI00400073	IPI00400073	yes	yes		3	4.1291E-59	200.6
RIYAGMVSIMDEAVGN(de)VTK	2053.0336	IPI00652358	IPI00652358	yes	no		3	8.1266E-05	110.77
RMEISVGAIQAN(de)R	1443.7616	IPI00108811	IPI00108811	yes	yes		3	0.0002383	142.97
RMHIN(de)GSNVQVIHR	1659.874	IPI00119063	IPI00119063	yes	yes	2,3		0.0049967	100.55
RN(de)FTAADWGHSR	1416.6647	IPI00339885	IPI00339885	yes	no		3	0.0027912	106.17
RNPSANTFIHIN(de)ASSFR	1930.9762	IPI00315535	IPI00315535	yes	yes	2,3		6.7549E-11	148.41
RPFGVVYEMEVDTIETTCHAIDPTPIAN(de)CSVR	3676.7324	IPI00128249	IPI00128249	yes	yes	3,4,5		2.8557E-35	156.73
RPYIVPIIWIN(de)ETGTIGDEK	2313.2369	IPI00331214	IPI00331214	yes	yes		3	2.8651E-05	118.48
RPYIVPIIWIN(de)ETGTIGDEKAEMFK	2919.5205	IPI00331214	IPI00331214	yes	yes	3,4		7.4821E-12	121.21
RSN(de)FTPATNEAPQATVFPK	2075.0436	IPI00172039	IPI00172039	yes	no	2,3		2.2583E-32	179.87

RSPGAQD(de)NVSVSQGMR	1687.806	IPI00348586	IPI00348586	yes	no	3	1.2208E-12	158.81
RVIYIPAY(de)NCTIRPVSK	2049.1194	IPI00469387	IPI00469387	yes	no	2,3	0.001318	109.1
RVM(de)EVNFISYVVMSTAAIPMIKQSNGSIAVISSIAGK	3911.0723	IPI00115599	IPI00115599	yes	no	5	0.012365	47.989
RVN(de)ASTTDPNSTVEQSAITR	2146.0614	IPI00338785	IPI00338785	yes	no	3	0.0012316	89.049
RVNDN(de)KTAAEEAIR	1585.8172	IPI00400016	IPI00400016	yes	no	2,3	0.0079686	101.43
RVSN(de)ESAAPIVPAVPRPEVDNITIR	2699.4719	IPI00122285	IPI00122285	yes	yes	3	0.0005193	93.73
RVSNESAAPIVPAVPRPEVDN(de)ITIR	2699.4719	IPI00122285	IPI00122285	yes	yes	3	0.0005193	93.73
RYIEIG(de)NETIIR	1475.8096	IPI00850057	IPI00850057	yes	no	2,3	2.9849E-41	199.82
SAEGTFFIN(de)K	1112.5502	IPI00125522	IPI00125522	yes	no	2	0.0019854	110.84
SAEGTFFIN(de)KTK	1341.6929	IPI00125522	IPI00125522	yes	no	2,3	0.0017741	132.79
SAEN(de)FTVIIK	1120.6128	IPI00130271	IPI00130271	yes	no	2	7.8137E-07	152.07
SAGN(de)MSTYNCQIIK	1585.7229	IPI00133520	IPI00133520	yes	yes	2,3	5.2245E-05	135.02
SAVFHRIEGITN(de)ETYR	1891.9541	IPI00119299	IPI00119299	yes	no	2	0.0030919	98.676
SAVGTISGN(de)SSNVIQIIIDAYNSISSEVIIENSKIPDGVTINYK	4622.3967	IPI00132474	IPI00132474	yes	yes	5	5.6149E-21	109.27
SAVSTSWIIPYN(de)HTWSHEK	2242.0807	IPI00124428	IPI00124428	yes	yes	2,3	9.7406E-26	174.39
SCADEGFSTPCAFVVAVSGAN(de)GSVIWERPVAQDVAIVK	3992.9401	IPI00410796	IPI00410796	yes	yes	3,4	8.0318E-37	147.25
SCAISEIGCPMDIFQHSAFADIN(de)VSQVITPDAFVCR	4054.8686	IPI00113057	IPI00113057	yes	yes	3,4	0.0021856	50.322
SCGECIQAGPNCGWCTN(de)TTFIQEGMPTSAR	3389.3992	IPI00132474	IPI00132474	yes	yes	3,4	3.6636E-20	133.29
SCHTAVGTSEGWNVPMGIIYN(de)QTGSCK	2953.3157	IPI00471080	IPI00471080	yes	no	3,4	1.3462E-05	95.138
SCVAITDAFPQN(de)MSR	1695.7709	IPI00405742	IPI00405742	yes	no	2,3	3.8061E-08	151.04
SDFSQTMIFQAN(de)TTR	1745.8043	IPI00114671	IPI00114671	yes	no	2	2.3809E-44	205.95
SDGIPIAPCGAIANSIFN(de)DSFSIWHQR	2972.4239	IPI00221859	IPI00221859	yes	yes	3,4	7.8104E-14	122.47
SDIIYVQTDATNPSVPIDPISVSN(de)SSSQIIIK	3400.7613	IPI00128358	IPI00128358	yes	no	4	0.0011822	55.691
SDIN(de)CSVMEATEEK	1611.6756	IPI00130015	IPI00130015	yes	no	2	9.8235E-11	157.8
SDINPAN(de)GSYPFQAIHQR	2013.9657	IPI00165730	IPI00165730	yes	yes	2,3	3.1938E-89	231.37
SEEIEFIGN(de)SSIR	1479.7205	IPI00320618	IPI00320618	yes	yes	2	9.5989E-05	143.94
SEHIVN(de)FTVK	1172.619	IPI00330481	IPI00330481	yes	yes	2,3	0.000315	119.53
SEYQK(de)WECKNDTIFGIK	2145.0201	IPI00126186	IPI00126186	yes	yes	2,3	6.9887E-48	207.92
SFDCCGIFN(de)ITTIR	1702.7807	IPI00132189	IPI00132189	yes	yes	2,3	8.4402E-08	155.66
SFIISIAAIHDN(de)HTHSDIQVK	2345.2128	IPI00130624	IPI00130624	yes	yes	2,3,4	3.0374E-23	166.57
SFMVN(de)WTQSPGKVEK	1736.8556	IPI00330632	IPI00330632	yes	no	2,3	1.8181E-43	200.77
SFMVN(de)WTQSPGKVEKYR	2056.02	IPI00330632	IPI00330632	yes	no	3	0.0017328	102.28
SFPVYIN(de)GSTVPSPVK	1690.893	IPI00229935	IPI00229935	yes	no	2	6.8053E-116	170.11
SFQQSHVHVHDIQSFGIDNIN(de)MTHYIK	3194.5356	IPI00626555	IPI00626555	yes	no	3,4	1.8656E-59	192.57
SFSGIIDCGN(de)CSR	1471.6184	IPI00626793	IPI00626793	yes	no	2	1.7861E-06	152.16
SFTVN(de)WTPPAGDWEHYR	2061.9333	IPI00229935	IPI00229935	yes	no	3	0.0030745	83.966
SFWYHDMDDPKGHIIIAQVA(de)TNPQGITGTGNTTSEMDPSHR	4524.0863	IPI00321375	IPI00321375	yes	no	4	2.3845E-07	67.064
SGDASIN(de)VTNIQISDIGTYQCK	2383.1326	IPI00270376	IPI00270376	yes	no	2,3	6.7388E-175	276.26
SGNIEHPEDKIFN(de)TSVEVIPFDNPQSEKEAIQEGR	3952.9079	IPI00336218	IPI00336218	yes	yes	4	1.9007E-05	71.085
SGQEDHYWIDVEKN(de)QSAK	2132.9763	IPI00319509	IPI00319509	yes	no	2,3,4	2.3765E-105	248.1
SGQVEVN(de)ITEFPYTPTPEHGR	2357.1288	IPI00311405	IPI00311405	yes	yes	3	7.8516E-12	141.64
SGSGIVGGQIEEFIN(de)QSSPFYFWMNGDR	3192.4611	IPI00320420	IPI00320420	yes	no	4	0.0001368	80.102
SGTIFDNFIITNDEAYAEEFGN(de)ETWGVTK	3267.4884	IPI00123639	IPI00123639	yes	yes	3,4	5.4872E-07	104.33
SHGIWN(de)NTVFIFSTDNGGQTR	2350.1091	IPI00652358	IPI00652358	yes	no	3	0.0014195	84.208
SHPIHSPVN(de)WTSYASSVESTPTK	2511.203	IPI00129215	IPI00129215	yes	yes	3	2.7474E-08	117.38
SIAQYIHSGPPVYFVIEEGYN(de)YSSR	2875.3817	IPI00132600	IPI00132600	yes	no	3	0.0037427	81.48
SIASEPTEKHFFN(de)VSDEIAIVTIVK	2773.4538	IPI00466371	IPI01026704	no	no	3	1.2096E-34	168.78
SIAYINSDSAIEGN(de)YTIR	1985.9694	IPI00113042	IPI00113042	yes	no	3	0.0013072	92.38
SICINGKWDPEPN(de)CTSK	2004.9033	IPI00130010	IPI00130010	yes	no	2,3	0.0002034	126.16
SIDAYPVINQAQAMEN(de)HTEVHFQK	2769.3181	IPI00468674	IPI00468674	yes	no	3,4	0.0001904	93.371
SIDEEAIKEN(de)NSIHWK	1911.9327	IPI00123223	IPI00123223	yes	no	2,3,4	1.0018E-97	218.6
SIDNDNYVFTAPYFN(de)K	1906.8737	IPI00626793	IPI00626793	yes	no	2	5.0708E-45	199.49
SIDNDNYVFTAPYFN(de)KSGPGAYESGIMVSK	3270.5179	IPI00626793	IPI00626793	yes	no	4	0.039619	47.616
SIFGAIPGN(de)R	1030.556	IPI00400073	IPI00400073	yes	yes	2	0.0001784	132.91
SIGEVN(de)FTATAEAIQSPEICGNK	2435.1639	IPI00624663	IPI00624663	yes	yes	2,3,4	2.2055E-208	265.41
SIGEVN(de)FTATAEAIQSPEICGNKITEVPAIVHK	3522.8028	IPI00624663	IPI00624663	yes	yes	3,4,5	2.7303E-98	216.25
SIGEVN(de)FTATAEAIQSPEICGNKITEVPAIVHKDTVVK	4065.1092	IPI00624663	IPI00624663	yes	yes	3,4,5	7.2953E-11	92.449
SIGNVN(de)FSVSAEAQQSSEPCGSEVATVPETGRK	3421.6056	IPI00123223	IPI00123223	yes	yes	3,4,5	9.4747E-189	215.36
SIGPN(de)TCSSNGSSIYFIHGPNIYCYSSIDKINAAK	3834.7982	IPI00128484	IPI00128484	yes	yes	4	2.7311E-10	90.384
SIGPNTCSSN(de)GSSIYFIHGPNIYCYSSIDKINAAK	3834.7982	IPI00128484	IPI00128484	yes	yes	4	2.7311E-10	90.384
SIIDHIHVGVGRDN(de)VSQPK	2070.097	IPI00123428	IPI00123428	yes	no	2,3	3.8946E-25	172.93
SIIEFN(de)TTMGCQPSDSQHR	2206.9736	IPI00308971	IPI00308971	yes	yes	2,3	6.7329E-42	193.19
SIIIIGSPTWKN(de)VSR	1669.9515	IPI00225715	IPI00225715	yes	yes	3	0.0002146	107.53
SIIW(de)TETFMNKENQNHSYSIK	2569.2271	IPI00120245	IPI00120245	yes	no	3	6.7197E-05	97.095
SIN(de)CTVK	820.41129	IPI00417180	IPI00417180	yes	no	2	1.5624E-17	143.61
SIN(de)GTFFGGWK	1212.5928	IPI00136938	IPI00136938	yes	yes	2	0.009307	88.187
SIN(de)VTGQGFSIIQK	1490.8093	IPI00405742	IPI00405742	yes	no	2,3	6.4801E-32	189.3
SINGVIIPGGGAN(de)ITDSGYSR	2047.0334	IPI00129243	IPI00129243	yes	no	2	4.8003E-09	133.74
SIPNN(de)VTSFEVESIKPYK	2051.0575	IPI00323053	IPI00323053	yes	no	2,3	5.0467E-37	191.07
SIPPGIFSTSAN(de)ISTIVIR	1972.0993	IPI00129250	IPI00129250	yes	yes	2,3	3.2573E-33	185.99
SIPQSSWGN(de)WSSWK	1648.7634	IPI00118291	IPI00118291	yes	no	2	0.0031919	97.45
SIPVN(de)DSVIDVFER	1588.8097	IPI00122122	IPI00122122	yes	no	2,3	4.2187E-11	163.71
SIPVN(de)DSVIDVFERR	1744.9108	IPI00122122	IPI00122122	yes	no	2,3	4.6922E-06	143.93
SIQTTSDQQNIKVPGN(de)ITSVIISNIVPR	3021.6459	IPI00221547	IPI00221547	yes	no	3	0.0005254	77.241
SISCQMAAFRGN(de)GSER	1769.7937	IPI00129677	IPI00129677	yes	no	2,3	0.0006923	121.99
SISPHAGTEPCPPEAAVCIIN(de)GSKPVNIGK	3099.5481	IPI00308971	IPI00308971	yes	no	3,4	7.3214E-14	119.34
SITFN(de)ESYQDVSEVVYGAK	2135.0059	IPI00136642	IPI00136642	yes	yes	2,3	0	340.42
SITHDTDGVAVFVINIPSN(de)VTVIK	2538.3694	IPI00330833	IPI00330833	yes	yes	2,3,4	8.0545E-36	177
SITQGSIIVGNIAPVN(de)GTSQGK	2140.1488	IPI00515360	IPI00515360	yes	no	2,3	8.3264E-198	283.55
SITVIIGAHN(de)K	1151.6663	IPI00230426	IPI00230426	yes	yes	2	3.8972E-06	147.91
SIYIIN(de)STR	1065.5819	IPI00311305	IPI00311305	yes	yes	2	0.016951	98.943
SKIDTEVAN(de)ISVVMEEMK	2022.0013	IPI00129304	IPI00129304	yes	yes	3	3.0143E-60	209.89
SKN(de)ITIVEIPDEVIVPR	1921.0884	IPI00118830	IPI00118830	yes	yes	3	0.0005168	120.92
SMAGSGHN(de)VSQEAIAIK	1698.8359	IPI00314673	IPI00314673	yes	yes	2,3	2.7246E-08	142.39
SMAGSGHN(de)VSQEAIAIKR	1854.937	IPI00314673	IPI00314673	yes	yes	2,3	0.0002614	108.71
SMANQIITFN(de)ISSK	1552.7919	IPI00467600	IPI00467600	yes	yes	2,3	4.1277E-23	178.84

SMKITYN(de)ESR	1227.5918	IPI01008227	IPI01008227	yes	no	2	0.0034182	113.38
SN(de)ISIIR	801.47085	IPI00323134	IPI00323134	yes	no	2	1.9125E-05	111.82
SN(de)ITVIR	801.47085	IPI00377953	IPI00377953	yes	no	2	0.0086862	105.57
SN(de)VTRPSEFNYIWIAPIPFIK	2491.3264	IPI00319509	IPI00319509	yes	no	2,3,4	5.6682E-30	168.64
SN(de)YSVIDITPVAAIIPK	1800.0033	IPI00225072	IPI00225072	yes	no	2,3	2.0189E-122	254.05
SNIDPSNVDSIFYAAQSSQVISGCEISVSN(de)ETK	3545.6468	IPI00475154	IPI00475154	yes	yes	3,4	2.5125E-12	114.8
SNKYSQ(de)AANSTKEIDDCEQANK	2500.1136	IPI00187353	IPI00187353	yes	yes	4	2.7224E-11	137.73
SPDKETFTCWWNPGSDGGIPTN(de)YSITYSK	3307.4768	IPI00321091	IPI00321091	yes	no	3	0.0001025	89.039
SPFYN(de)ISDQISFQCYDGYVIR	2571.174	IPI00114065	IPI00114065	yes	no	3	0.0050065	72.772
SPGAQDN(de)VSVSQGMR	1531.7049	IPI00348586	IPI00348586	yes	no	2,3	3.9585E-44	203.87
SPMQWDN(de)SSNAGFTEANHTWIPTNSDYHTVNVDVQK	4089.8188	IPI00128432	IPI00128432	yes	yes	4	0.0054541	44.021
SPMQWDNSSNAGFTEAN(de)HTWIPTNSDYHTVNVDVQK	4089.8188	IPI00128432	IPI00128432	yes	yes	4	0.0054541	44.021
SPN(de)DSEAWHQK	1297.5687	IPI00153644	IPI00153644	yes	yes	2,3	0.01659	81.625
SPPAPPIPQRPISPIHPIGCN(de)DSEVIAVAGFAIQNINR	4042.1211	IPI00469387	IPI00469387	yes	yes	4	2.0414E-08	79.917
SPVIIGQPNTIICFVDNIFPPVIN(de)ITWIR	3335.8104	IPI00172039	IPI00172039	yes	no	4	0.0038696	56.172
SPVNIGE(de)PNIIICFIDKFSPPVVNVTWIR	3323.774	IPI00110808	IPI00110808	yes	no	4	0.010116	57.145
SQITISNIDVNVDPGTYVCN(de)ATNAQGTTR	3108.4782	IPI00408495	IPI00408495	yes	no	2,3	7.3906E-202	193.53
SQIVNETHWQY(de)YGTSDTR	2183.9872	IPI00749655	IPI00749655	yes	no	2,3	1.8089E-21	173.21
SQN(de)DTVTPDVWYTSKPEKK	2222.0855	IPI00471238	IPI00471238	yes	yes	3	1.0355E-05	127.3
SQNDKFN(de)VSITVK	1478.7729	IPI00466371	IPI01026704	no	no	2,3	7.5471E-275	225.32
SQSPSIMYTINGFVN(de)K	1784.8767	IPI00406603	IPI00406603	yes	yes	2,3	1.303E-25	181.51
SR(de)TNITSIIVQPISPSIVK	2052.1943	IPI00985989	IPI00985989	yes	no	3	4.5958E-09	144.3
SRYPHKPE(de)INSTTHPGADIK	2247.1396	IPI00114206	IPI00114206	yes	yes	2,3	3.9643E-20	166.52
SSAN(de)QSEFQQQIQK	1621.7696	IPI00990932	IPI00990932	yes	no	2	4.2063E-16	170.49
SSIRN(de)GTEVVSCHPQGSTAGVVYR	2560.2452	IPI00674255	IPI00674255	yes	yes	3	6.2182E-08	109.37
SSQSN(de)QTIWFGHFTTSTIMSPSPGIR	2866.3708	IPI00226714	IPI00226714	yes	no	3	0.010277	64.589
SSSHIPPSSYFN(de)ASGR	1692.7856	IPI00130661	IPI00130661	yes	yes	2,3	3.1373E-102	247.93
SSSYHENDMENPQSN(de)ITMVYIIPIGPK	3063.4318	IPI00406603	IPI00406603	yes	yes	3	1.9242E-16	130.3
SSTEN(de)TSAEIHVIGR	1599.7853	IPI00330594	IPI00330594	yes	yes	2,3	1.5187E-70	226.93
SSVEIIN(de)STQAPMR	1531.7664	IPI00120166	IPI00120166	yes	yes	2	2.7964E-05	140.1
STFYTCQVSNPVSSKN(de)DTVYFTIPCDIAR	3369.5646	IPI00109946	IPI00109946	yes	yes	3	5.1449E-41	171.5
STGKPTIYNVSII(de)MSDTGGTCY	2364.0977	IPI00468055	IPI00468055	yes	yes	2	1.0652E-222	299.71
STIIIHHPQYAWIQDIGIREDNEGVYN(de)GSWGGR	3780.8397	IPI00230084	IPI00230084	yes	no	4	4.3379E-06	66.925
STTQTN(de)SSDSHIVK	1503.7165	IPI00111807	IPI00111807	yes	no	2,3	0.0010583	109.44
SVAFQDCQVN(de)ITIEGR	1835.8836	IPI00173181	IPI00173181	yes	no	2	5.9126E-86	233.37
SVAPCTDFFSFACEKAN(de)GTSDSFQAITEENK	3457.5079	IPI00111137	IPI00111137	yes	yes	4	0.01784	51.052
SVCEDEPYVN(de)VTAEDIDGAQNSAPFSFSIIDQPPGTAQK	4195.9168	IPI00115793	IPI00115793	yes	yes	4	0.0008917	51.975
SVGIPVICIN(de)ATDDVFSPSHAIPIETAK	2950.511	IPI00131173	IPI00131173	yes	yes	3	1.2338E-13	117.21
SVGTGTNMVFQN(de)CSCIGSSGNSSAVIGICK	3091.3831	IPI00625835	IPI00625835	yes	no	3,4	8.2061E-36	160.88
SVGTGTNMVFQNCSCIGSSGN(de)SSAVIGICK	3091.3831	IPI00625835	IPI00625835	yes	no	3,4	8.2061E-36	160.88
SVIEN(de)TTSYEEAK	1469.6886	IPI00125266	IPI00125266	yes	yes	2	0.0004997	125.97
SVIEN(de)TTSYEEAKNTITK	2027.0059	IPI00125266	IPI00125266	yes	yes	2,3	3.3605E-123	258.9
SVN(de)ISEIIDVYSDGVEIIQIVK	2432.305	IPI00466999	IPI00466999	yes	no	3	4.8646E-208	198.49
SVNINFN(de)ITHR	1313.684	IPI00118291	IPI00118291	yes	no	2	0.0005279	114.72
SVVHHIN(de)GSTIR	1318.7106	IPI00758066	IPI00758066	yes	no	2,3	0.010059	84.718
SVYN(de)CSGEACSGHNR	1696.6682	IPI00314726	IPI00314726	yes	yes	2,3	4.697E-44	203.11
SWSTVGN(de)CTAAIR	1421.6721	IPI00128484	IPI00128484	yes	yes	2	6.3883E-05	145.1
SWVQGN(de)ITACGR	1347.6354	IPI00153389	IPI00153389	yes	no	2	0.00125	107.99
SYCKNGVN(de)GTGENGR	1611.706	IPI00132474	IPI00132474	yes	yes	2,3	6.3484E-08	155.66
SYCKNGVN(de)GTGENGRK	1739.8009	IPI00132474	IPI00132474	yes	yes	2,3	1.9791E-101	247.93
SYIDDRPIQHYIAVSSPTN(de)TTYVVQYAIANITGK	3797.9265	IPI00118674	IPI00118674	yes	yes	3,4	8.672E-06	76.002
SYIDDRPIQHYIAVSSPTNTTYVVQYAIAN(de)ITGK	3797.9265	IPI00118674	IPI00118674	yes	yes	3,4	8.672E-06	76.002
SYIFIYDGNKN(de)STTTDQNFTSAK	2614.2187	IPI00124830	IPI00124830	yes	no	2,3	5.0742E-30	154.84
SYIFIYDGNKNSTTTDQN(de)FTSAK	2614.2187	IPI00124830	IPI00124830	yes	no	2,3	5.0742E-30	154.84
TAADATGIQPIINQFTPAN(de)VSR	2284.1812	IPI00120245	IPI00120245	yes	no	2,3	4.9552E-101	235.92
TAASIIWQAYPIIN(de)ISEK	2017.0884	IPI00115867	IPI00115867	yes	yes	2,3	2.2573E-28	178.51
TADCDDNQPGAICYYPGN(de)ETEEEVR	2902.1658	IPI00129253	IPI00129253	yes	yes	3	0.017511	53.998
TADIIQN(de)ITGR	1200.6463	IPI00112614	IPI00112614	yes	yes	2	0.0012207	107.09
TAFAPPDIPVCIIGN(de)R	1739.9029	IPI00331175	IPI00331175	yes	no	3	1.3234E-05	134.21
TAGAN(de)GTSGFFCVDEGGIPIAQR	2324.0855	IPI00282289	IPI00282289	yes	no	2,4	3.3019E-12	137.89
TAGWNIPMGIIAN(de)QTR	1741.8934	IPI00323235	IPI00323235	yes	yes	2,3	1.7705E-07	143.97
TAIFPDIIVQGN(de)ASIR	1713.9414	IPI00124078	IPI00124078	yes	yes	2,3	4.1489E-13	161.91
TAIVQEVHQN(de)FSAWCSQVIR	2372.1695	IPI00381303	IPI00381303	yes	yes	2,3	5.4094E-80	227.46
TAIWVATDHNTDN(de)TSAIIR	2098.0443	IPI00169870	IPI00169870	yes	yes	2,3	5.9652E-65	209.23
TAN(de)ETSAEAYNIIIR	1664.837	IPI00400016	IPI00400016	yes	no	3	3.1278E-05	130.61
TANDAIQDMICDMEGIPQKHN(de)FSHCCGK	3276.3879	IPI00130654	IPI00130654	yes	no	4	2.1389E-26	150.14
TASVSIN(de)QTEPPK	1370.7042	IPI00129220	IPI00129220	yes	yes	2	0.0035498	96.591
TASVSIN(de)QTEPPKVR	1625.8737	IPI00129220	IPI00129220	yes	yes	2,3	1.9348E-43	200.55
TCNPETFPIRN(de)ESIQCPTAR	2390.1107	IPI00649186	IPI00649186	yes	no	3	0.0016708	89.468
TCPAGIMGEN(de)NTIVWK	1789.8491	IPI00121190	IPI00121190	yes	no	2,3	2.6169E-119	251.84
TCPIDEFQCN(de)NTICKPIAWK	2494.1443	IPI00119063	IPI00119063	yes	yes	2,3	1.7263E-25	172.93
TCVSN(de)CTASQFVCK	1660.7007	IPI00119063	IPI00119063	yes	yes	2,3	3.7389E-55	214.18
TCVSN(de)CTASQFVCKNDK	2017.8656	IPI00119063	IPI00119063	yes	yes	2	0.0060977	86.367
TDAEIN(de)ETARPISPVNPK	1951.0011	IPI00408895	IPI00408895	yes	yes	2	0.0008414	97.081
TDDEVVQREEEAIQIDGIN(de)ASQIR	2727.3311	IPI00129526	IPI00129526	yes	yes	2,3,4	7.5768E-109	235.98
TDPANQFEWIENTIN(de)SSIWNK	2506.1765	IPI00136259	IPI00136259	yes	yes	3	0.0011933	82.765
TDPANQFEWIENTIN(de)SSIWNKEK	2763.314	IPI00136259	IPI00136259	yes	yes	4	0.017218	65.784
TEAGVFEYVADPTFEN(de)FTGGVK	2377.1114	IPI00405742	IPI00405742	yes	no	2,3	3.1995E-60	207.76
TEAGVFEYVADPTFEN(de)FTGGVKK	2505.2064	IPI00405742	IPI00405742	yes	no	2,3,4	1.7508E-86	226.57
TEGMIIN(de)VTSSIR	1419.7392	IPI00109254	IPI00109254	yes	yes	2	0.0005589	121.45
TEIDIRPQGIAIFSN(de)VSEAR	2215.1597	IPI00122973	IPI00122973	yes	no	2,3	4.3876E-33	185.85
TEKDPQNDPVGTCYISTEN(de)FTR	2571.1547	IPI00115976	IPI00115976	yes	yes	2,3	3.4388E-30	168.81
TEQIIIQAPN(de)TSVHIYEPYPAGADVPFGPPINK	3575.83	IPI00132600	IPI00132600	yes	no	3,4,5	1.5745E-99	218.86
TEVIIQGTVSPN(de)ASAPDAVWEDYEFK	2865.3709	IPI00405437	IPI00405437	yes	no	4	0.0053671	61.437
TEVKPVATEQIISTFITVGN(de)NTCFYGK	3016.5216	IPI00122494	IPI00122494	yes	yes	3,4	2.8267E-13	121.39
TEVSNNNVIIYIDQVTN(de)QTIAFSFIIQQDIPVR	3791.9734	IPI00123223	IPI00123223	yes	yes	3,4	1.7618E-49	173.59

TFHETIN(de)CCGSNAITTITTTIIR	2623.2734	IPI00310916	IPI00310916	yes	no		3	5.7041E-08	109.85
TFN(de)VSEDTKVNTTVIPETQIK	2363.222	IPI00314132	IPI00314132	yes	no		3	0.000124	96.391
TFNVSEDTKVN(de)TTVIPETQIK	2363.222	IPI00314132	IPI00314132	yes	no		3	0.000124	96.391
TFPGETN(de)VTIQIWESEGR	2062.996	IPI00355482	IPI00355482	yes	yes	2,3		5.4825E-06	127.29
TGATNTWIFPGSCNGN(de)ETTFWQCK	2776.201	IPI00760106	IPI00760106	yes	no		3	0.0019014	76.049
TGDGSN(de)VTSNFTKDPSIIIPEIIPGVSYTVK	3248.6816	IPI00469426	IPI00469426	yes	no		3	4.2442E-08	103.14
TGDGSNVTSN(de)FTKDPSIIIPEIIPGVSYTVK	3248.6816	IPI00469426	IPI00469426	yes	no		3	4.2442E-08	103.14
TGEAN(de)ITQIYTQEAIDFIQTQHAR	2747.3515	IPI00310090	IPI00310090	yes	no	3,4		2.5582E-143	257.96
TGEIFTFN(de)TTAAQPQYFK	2063	IPI00330714	IPI00330714	yes	no		2	8.5579E-237	302.96
TGEIFTFN(de)TTAAQPQYFKYEFPDGVDSVIVK	3511.7188	IPI00330714	IPI00330714	yes	no	3,4		3.0056E-40	157.9
TGEIN(de)ITSIVDR	1316.6936	IPI00380460	IPI00380460	yes	no		2	3.5456E-06	150.22
TGHRPPNPAFWWVN(de)GSGTEVK	2336.145	IPI00221556	IPI00221556	yes	no		3	7.7407E-16	150.69
TGHTNAHGK(de)DIDTDFTSNASQPETK	2671.211	IPI00130573	IPI00130573	yes	yes		3	5.4924E-08	108.08
TGTIAIQN(de)TTQIR	1415.7732	IPI00623114	IPI00623114	yes	no		2	2.388E-06	150.34
TGVHDGDFEYN(de)ITTTIAAINK	2279.107	IPI00108849	IPI00108849	yes	no		3	5.8974E-09	130.39
TGVNSGVMIMN(de)MTR	1509.7102	IPI00856861	IPI00856861	yes	no		2	0.0022293	95.236
TIAGEN(de)QTAIEIEEINR	1899.9538	IPI00400016	IPI00400016	yes	no		3	1.3668E-104	242.19
TIAGEN(de)QTAIEIEEINRK	2028.0487	IPI00400016	IPI00400016	yes	no		3	0.0066237	82.59
TIEDIIFRAEN(de)R	1475.7732	IPI00122312	IPI00122312	yes	no		3	0.015607	93.096
TIEQERN(de)ASFVYTK	1684.842	IPI00123223	IPI00123223	yes	no	2,3		8.1568E-23	182.1
TIGISPFHEFADVVFTAN(de)DSGHR	2516.2084	IPI00127560	IPI00127560	yes	yes	2,3,4		1.5898E-136	245.78
TIIGYYN(de)QSAGGTHTIQWMYGCDVGSDGR	3206.4186	IPI00109996	IPI00109996	yes	yes		3	2.8558E-07	105.17
TIIGYYN(de)QSESGSHTIQWMYGCK	2722.2156	IPI00608044	IPI00608044	yes	no		3	1.4351E-31	175.33
TIIN(de)TSCDNMIMAIK	1723.8307	IPI00224654	IPI00224654	yes	yes		2	0.0002073	118.33
TIIPAAAQDVYYRDEIGN(de)VSTSHIIIDDSVEMEIRPR	4299.1845	IPI00309035	IPI00309035	yes	yes	4,5,6		1.6377E-05	65.33
TIISYYN(de)QSAGGSHTIQVISGCEVGSDGR	3055.4305	IPI00474494	IPI00474494	yes	no		3	2.4401E-33	160
TIIVDN(de)NTWNNTHISR	1896.9442	IPI00109108	IPI00109108	yes	yes	2,3		0	265.77
TIIVDNNTWN(de)NTHISR	1896.9442	IPI00109108	IPI00109108	yes	yes	2,3		0	265.77
TIKPNPAKDPVGTCYVAIQN(de)FSAYAEHSPCR	3490.6762	IPI00345112	IPI00345112	yes	no	4,5		4.2095E-06	84.529
TIMSAEANIAGIFPPNEVQHFNPN(de)ISWQPIPVHTVPITEDR	4579.2958	IPI00154056	IPI00154056	yes	no	4,5		1.2566E-25	121.1
TIN(de)WSAAEPGAWATK	1601.7838	IPI00331440	IPI00331440	yes	no	2,3		2.77E-06	143.28
TIPHHQYN(de)ATINK	1535.7845	IPI00348266	IPI00348266	yes	yes		2	0.00879	84.173
TIPHPDITHIPPPSPSPTIN(de)NTSISR	2788.4508	IPI00406603	IPI00406603	yes	yes		3	1.3952E-05	88.09
TIPHPDITHIPPPSPSPTINN(de)TSISR	2788.4508	IPI00406603	IPI00406603	yes	yes		3	1.3952E-05	88.09
TISDVPSAAPQN(de)ISIEVR	1895.9953	IPI00129159	IPI00129159	yes	no	2,3		2.2644E-48	203.53
TITIIN(de)VTR	1029.6182	IPI00108535	IPI00108535	yes	no		2	5.2923E-07	155.11
TITIIN(de)VTRNDTGPYVCETR	2322.1638	IPI00108535	IPI00108535	yes	no	2,3		8.2546E-65	212.72
TITIINVTRN(de)DTGPYVCETR	2322.1638	IPI00108535	IPI00108535	yes	no	2,3		8.2546E-65	212.72
TITVYN(de)FSAPVITISQIEVSEGSQVTVK	3009.591	IPI00122973	IPI00122973	yes	no	3,4		1.3737E-90	217.65
TIYN(de)WSGYPIIVHK	1689.8879	IPI00471081	IPI00471081	yes	yes	2,3		6.9089E-11	161.04
TKQHGQFSIAVVGIN(de)ITSIGIR	2338.3121	IPI00121190	IPI00121190	yes	no		3	1.3603E-07	120.4
TKTQVFGPVDPT(de)VNTTYAFFNTFFK	2869.4327	IPI00115530	IPI00115530	yes	yes	3,4		4.8699E-56	192.27
TMVFPVMYIN(de)ESVIIDK	1998.0206	IPI00127447	IPI00127447	yes	yes	2,3		7.1031E-12	155.2
TMVFPVMYIN(de)ESVIIDKETANQIK	2782.4285	IPI00127447	IPI00127447	yes	yes	3,4		2.5472E-16	145.43
TN(de)HTVMTMGSDFHYENANMWFK	2660.1246	IPI00381303	IPI00381303	yes	yes		3	0.012601	59.585
TN(de)ITSIIVQPISPSIVK	1809.0612	IPI00985989	IPI00985989	yes	no		3	2.5858E-05	127.83
TN(de)KSEMWDVFCYR	1734.7494	IPI00467600	IPI00467600	yes	no		3	0.010711	95.417
TN(de)SSFIQGFVDHVK	1577.7838	IPI00321190	IPI00321190	yes	no	2,3		2.0044E-23	183.96
TN(de)SSFIQGFVDHVKEDCDR	2253.012	IPI00321190	IPI00321190	yes	no	3,4		0.0001145	114.52
TN(de)SSFIQGFVDHVKEDCDRIGPGVSDICK	3279.5289	IPI00321190	IPI00321190	yes	no	3,4		3.418E-09	113.6
TNIEGQVPFIPAN(de)FTAHITPYK	2457.2692	IPI00129903	IPI00129903	yes	yes		3	7.8055E-23	162.42
TNIEGQVPFIPAN(de)FTAHITPYKEFYHYR	3352.6669	IPI00129903	IPI00129903	yes	yes	3,4		0.0001679	88.836
TNQ(de)SCEIVIDSTEKVNPSYIGR	2509.2119	IPI00310059	IPI00310059	yes	no		3	0.0001371	95.414
TPASDPHGDN(de)ITYSVFYTK	2111.98	IPI00129159	IPI00129159	yes	no	2,3		6.4114E-05	103.88
TPIHYFDSQCMGAMHWQVMHN(de)VTTETK	3248.43	IPI00349830	IPI00349830	yes	no		4	0.0079914	52.79
TPTIPPVSFHIGGVWFN(de)ITGQDYVIK	2885.5116	IPI00113797	IPI00113797	yes	yes	3,4		4.6568E-21	148.04
TQDSIIQAETQANSCN(de)ITVVTIQESIEK	3119.5292	IPI00321222	IPI00321222	yes	yes		3	6.1517E-05	88.413
TQDSIIQAETQANSCN(de)ITVVTIQESIEKK	3247.6242	IPI00321222	IPI00321222	yes	yes	3,4		3.9934E-51	182.67
TQITCSIN(de)SSGVDIVGHR	1942.9531	IPI00408495	IPI00408495	yes	no	2,3		1.0071E-210	298.93
TQMVFFN(de)ITPNIINDTMEIR	2396.1868	IPI00127856	IPI00127856	yes	yes	2,3		2.9686E-42	192.25
TQMVFFNITPNIIN(de)DTMEIR	2396.1868	IPI00127856	IPI00127856	yes	yes	2,3		2.9686E-42	192.25
TQVFGPVDPTVN(de)TTYAFFNTFFK	2640.29	IPI00115530	IPI00115530	yes	yes	2,3		4.831E-60	204.21
TQYACPPFN(de)VTECSVQDAAGNSIDISSISR	3286.4871	IPI00308971	IPI00308971	yes	yes		3	0.0005711	63.893
TSDTGEEEAITN(de)STEANGK	1952.8447	IPI00408495	IPI00408495	yes	no	2,3		8.8236E-300	328.92
TSDTGEEEAITN(de)STEANGKYVVVSTPEK	2955.3833	IPI00408495	IPI00408495	yes	no	3,4		8.7055E-105	229.1
TSIDIWMHTDPMSQKN(de)ESVK	2346.0984	IPI00118674	IPI00118674	yes	yes		3	0.0013559	89.484
TSIDWWTTDTCNMIN(de)GTDGDSFHPIISK	3211.4227	IPI00127447	IPI00127447	yes	yes		3	1.1316E-63	197.92
TSIDWWTTDTCNMIN(de)GTDGDSFHPIISKDEVIYIFPSDICR	4819.188	IPI00127447	IPI00127447	yes	yes		4	0.04434	38.376
TSIIVIN(de)NTESNIEDFIQSIK	2377.2377	IPI00170146	IPI00170146	yes	yes	2,3		6.0994E-175	260.24
TSPFSIN(de)DSVIIEEIEAYNPAK	2436.206	IPI00396759	IPI00396759	yes	no		3	2.0808E-05	98.443
TSRPTDNSIN(de)VTCGPPYETNGPK	2504.1602	IPI00323053	IPI00323053	yes	no		3	1.6456E-08	126.26
TSSEFCQPVSIHGSN(de)HTSK	2101.9487	IPI00894972	IPI00894972	yes	no		3	2.5778E-06	128.99
TSYCN(de)ESFYFICK	1717.7116	IPI00126186	IPI00126186	yes	yes	2,3		6.6804E-42	201.48
TSYCN(de)ESFYFICKK	1845.8066	IPI00126186	IPI00126186	yes	yes	2,3		0.0003616	116.84
TTIVDN(de)NTWNNSHIAIVGK	2096.0651	IPI00316469	IPI00316469	yes	yes	2,3		0	360.07
TTIVDNNTWN(de)NSHIAIVGK	2096.0651	IPI00316469	IPI00316469	yes	yes	2,3		0	360.07
TTPPIQIN(de)ETENTEQDDIPADCSAVYNR	3190.4361	IPI00128206	IPI00128206	yes	yes		4	0.0001484	80.082
TTVKN(de)ITVIEPVTQPFIQVTNTTVK	2770.548	IPI00108535	IPI00108535	yes	no	2,3,4		8.3591E-45	186.13
TTVKNITVIEPVTQPFIQVTN(de)TTVK	2770.548	IPI00108535	IPI00108535	yes	no	2,3,4		8.3591E-45	186.13
TVAN(de)ISGCEATDSEAIIHCIR	2316.0838	IPI00154043	IPI00154043	yes	yes	2,3,4		0	264.03
TVDKSTGKPTIYN(de)VSIIMSDTGGTCY	2807.3357	IPI00468055	IPI00468055	yes	yes		3	1.38E-56	195.39
TVIDKNGDAYGYYN(de)DSIK	2034.9535	IPI00471081	IPI00471081	yes	yes	2,3		5.1554E-28	183.7
TVNSIN(de)VSAISIPEHDEADDISDENREK	3096.4483	IPI00121378	IPI00121378	yes	no		3	2.455E-51	186.92
TVSTIVIQAAN(de)VSAIYK	1776.9986	IPI00124326	IPI00124326	yes	no	2,3		1.3695E-68	174.59
TVVAPSTEGGIN(de)ITSTFIR	1962.0422	IPI00114252	IPI00114252	yes	no	2,3		1.6176E-13	148.38
TVVQYSPNIIFSGIEEIQKDSCESNQN(de)ITCR	3628.7138	IPI00466371	IPI01026704	no	no	3,4		6.8054E-22	128.17

TVVTEAGNIIKDN(de)ATQEEIIHYIEK	2827.4604	IPI00321190	IPI00321190	yes	no	2,3,4	2.4056E-206	284.98
TWSPPFESDDSQKHN(de)QSEYDDSASECCSCPK	3677.4253	IPI00128358	IPI00128358	yes	no	4	9.4937E-05	71.882
TYADDVNSEIVNIYTFN(de)HTVTR	2571.2241	IPI00330714	IPI00330714	yes	no	2,3	1.1852E-197	284.65
TYAVYDIFDTAMINN(de)SR	1992.9251	IPI00226790	IPI00226790	yes	no	3	2.7404E-28	185.67
TYCANEPISN(de)CSQVNR	1911.8203	IPI00127770	IPI00127770	yes	yes	2	0.0015314	102.08
TYCANEPISNCSQVN(de)R	1911.8203	IPI00127770	IPI00127770	yes	yes	2	0.0015314	102.08
TYEN(de)GSSVEYR	1303.5681	IPI00122117	IPI00122117	yes	yes	2	0.0036736	99.215
TYNVIDMKN(de)TTCQDIQIEVK	2412.1665	IPI00131091	IPI00131091	yes	no	3	1.2623E-32	180.64
VAEVEN(de)GTKPDSDVPEHCIDTWSFDAATMDHNGTMIFFK	4410.9508	IPI00128484	IPI00128484	yes	yes	4	7.0367E-40	110.43
VAEVENGTKPDSDVPEHCIDTWSFDAATMDHN(de)GTMIFFK	4410.9508	IPI00128484	IPI00128484	yes	yes	4	7.0367E-40	110.43
VAGIIVIN(de)YSNDYNHWIATK	2290.1746	IPI00111908	IPI00111908	yes	no	2,3	1.1135E-25	173.98
VAN(de)ITVVVNSIDGK	1427.7984	IPI00310797	IPI00310797	yes	yes	2	1.8642E-11	166.04
VAQPGINYAIGTN(de)TSYPNNIIR	2375.2234	IPI00226360	IPI00226360	yes	no	2,3	1.4232E-48	193.8
VCIPRDPCITNFGGCPSN(de)STFCIYR	2990.3296	IPI00987265	IPI00987265	yes	no	3	4.0005E-57	198.24
VCNGIG(de)IGEFKDTISINATNIK	2363.2155	IPI00121190	IPI00121190	yes	no	3	3.7155E-22	162.42
VDDEMPQHAVISGPNIFINNIN(de)KTDNGTYR	3371.6205	IPI00856723	IPI00856723	yes	no	3,4	4.2789E-05	91.416
VDDEMPQHAVISGPNIFINNINKTDN(de)GTYR	3371.6205	IPI00856723	IPI00856723	yes	no	3,4	4.2789E-05	91.416
VDFIWHPEVN(de)GSMK	1657.7923	IPI00380296	IPI00380296	yes	yes	3	0.0039232	92.856
VDIEDFEN(de)NTAYAK	1627.7366	IPI00551169	IPI00551169	yes	no	2	9.8875E-136	266.2
VDIPQQPMGIIAVANDTN(de)SCEISPCR	2884.3517	IPI00119063	IPI00119063	yes	yes	3,4	7.6468E-13	123.32
VDIPQQPMGIIAVANDTN(de)SCEISPCR	2884.3517	IPI00119063	IPI00119063	yes	yes	3,4	7.6468E-13	123.32
VDVIVAN(de)ITVTDKDQPHTPAWNAAYR	2893.4723	IPI00323134	IPI00323134	yes	no	3,4	2.1293E-08	111.6
VEFDDKGNVITSYGNPIIIN(de)SSIPEDATIK	3248.6453	IPI00122257	IPI00122257	yes	no	3,4	9.6655E-14	116.99
VEN(de)GSETGPIPPEIQPIIEGEVK	2431.2482	IPI00136012	IPI00136012	yes	yes	3	3.6885E-08	114.75
VETGVIKPGMVVTFAPVN(de)VTTEVK	2514.3767	IPI00307837	IPI00307837	yes	yes	2,3,4	8.5352E-16	142.04
VFDIHQNMGSVN(de)VSVGCTPAQIIETSR	2958.4328	IPI00116913	IPI00116913	yes	no	3	1.4822E-38	175.42
VFGSQN(de)ITTVK	1192.6452	IPI00123342	IPI00123342	yes	yes	2	1.2347E-41	203.51
VFHIHN(de)ESWVIITPK	1818.9781	IPI00224752	IPI00224752	yes	yes	2,3	9.9846E-85	232.5
VFIVPVGN(de)HSNIPFSR	1781.9577	IPI00221418	IPI00221418	yes	no	2,3	2.35E-57	209.6
VFKPQSGADAIN(de)DSQDFPFPETPAK	2705.2973	IPI00462465	IPI00462465	yes	no	3,4	1.6003E-161	261.26
VFKTN(de)STQVSDVR	1479.7682	IPI00469426	IPI00469426	yes	no	2,3	0.001595	119.88
VFNGKDN(de)ISK	1120.5877	IPI00331214	IPI00331214	yes	yes	2	0.0013733	123.51
VFPYISAMVNN(de)GSISYDHER	2298.0739	IPI00830356	IPI00830356	yes	no	2,3,4	1.732E-05	111.57
VFPYISVMVNN(de)GSISYDHSK	2256.0885	IPI00321634	IPI00321634	yes	yes	2,3,4	1.8256E-33	185.33
VFPYISVMVNN(de)GSISYDHSKDGR	2584.238	IPI00321634	IPI00321634	yes	yes	2,3,4,5	1.7499E-07	107.72
VFVYTPTTN(de)YTIR	1573.814	IPI00124428	IPI00124428	yes	yes	2	0.0025397	108.56
VGAFFGIFNVAVMN(de)ISGNCIR	2285.1449	IPI00988164	IPI00988164	yes	no	3	2.401E-62	152.32
VGFTGTMPATN(de)VSIFINNTQISDTGTYQCIVNNIPDR	4057.9514	IPI00172354	IPI00172354	yes	no	4	1.5754E-12	98.94
VGFTGTMPATNVSIFIN(de)NTQISDTGTYQCIVNNIPDR	4057.9514	IPI00172354	IPI00172354	yes	no	4	1.5754E-12	98.94
VGIVTYSN(de)ETR	1237.6303	IPI00855103	IPI00855103	yes	no	2	0.009307	88.187
VGNVECGEGHFCHDN(de)QTCCK	2406.9198	IPI00124640	IPI00124640	yes	yes	3	9.867E-07	130.11
VGQPEIKPMRN(de)ECNSEESSCPFR	2750.2211	IPI00111794	IPI00111794	yes	no	3	0.018334	66.325
VGVNKN(de)QTVTATFGYPFR	1998.0323	IPI00114256	IPI00114256	yes	no	2,3,4	3.8778E-74	220.64
VHGPN(de)ASHYTSIMTMITWER	2330.0936	IPI00169617	IPI00169617	yes	yes	3	5.9334E-05	97.095
VHSGN(de)FSTIPQYFK	1623.8045	IPI01023131	IPI01023131	yes	no	2	0.0144	77.42
VIDIWDIAQSAN(de)FTEK	1848.9258	IPI00469307	IPI00469307	yes	no	2,3	1.5685E-09	153.75
VIEEFYN(de)QTWNHR	1734.8114	IPI00308691	IPI00308691	yes	no	3	0.003539	96.64
VIEEPKN(de)VSCETR	1559.7614	IPI00118291	IPI00118291	yes	no	2	0.0014367	116.51
VIENEKFDTHEYHN(de)ESR	2145.9716	IPI00463492	IPI00463492	yes	yes	3	0.0010962	110.35
VIETIPAN(de)YSINSSK	1634.8516	IPI00480532	IPI00480532	yes	no	2,3	3.4477E-05	129.77
VIFKN(de)YSINDATITIHNIGFSDSGK	2753.4024	IPI00471187	IPI00471187	yes	no	3	1.2959E-161	263.09
VIFYKDDAMVYN(de)VTSR	1919.9451	IPI00406901	IPI00406901	yes	no	2,3	3.9913E-09	154.07
VIIGN(de)ESCTITISESTTNTIK	2280.1519	IPI00130420	IPI00130420	yes	no	3	7.1494E-09	128.99
VIIIIDPAISGN(de)ETEPYPAFTR	2415.2686	IPI00848693	IPI00848693	yes	yes	2,3,4	2.7099E-101	237.76
VIIN(de)ITTVAANHGYTK	1713.9414	IPI00467600	IPI00467600	yes	no	2,3	5.0063E-05	130.47
VIIQSQIPIGTIKHN(de)MTYFCK	2490.3127	IPI00108003	IPI00108003	yes	no	3,4	0.0072142	76.713
VIIRPYITPNNQGIYIFQGN(de)STVR	2762.4868	IPI00319509	IPI00319509	yes	yes	2,3,4	1.4292E-79	211.17
VIKDAVNN(de)ITAK	1284.7402	IPI00626793	IPI00626793	yes	no	3	0.011952	101.89
VIN(de)DTWAWK	1131.5713	IPI00123342	IPI00123342	yes	yes	2	0.027883	91.584
VIN(de)DTWAWKNATIAEQAK	2058.0534	IPI00123342	IPI00123342	yes	yes	2	5.552E-123	258.9
VIN(de)ITDNTYFK	1326.682	IPI00130117	IPI00130117	yes	yes	2	0.0065639	92.866
VINADQGTSATVQMIIN(de)DTCPIFVR	2762.3731	IPI00320065	IPI00320065	yes	yes	2,3,4	1.9915E-45	185.71
VINDTWAWKN(de)ATIAEQAK	2058.0534	IPI00123342	IPI00123342	yes	yes	2	5.552E-123	258.9
VINEECKEN(de)ESINIAAR	1987.9633	IPI00122411	IPI00122411	yes	yes	2	2.2117E-27	180.88
VIPFN(de)VTDYCQIVR	1722.8763	IPI00828222	IPI00828222	yes	no	2,3	1.9393E-116	251.3
VIPSSQPPISCSEEGVGN(de)ATISPVMGEECVR	3285.5316	IPI00895378	IPI00895378	yes	no	4	5.063E-05	69.284
VIQVVN(de)VTK	998.61243	IPI00355482	IPI00355482	yes	yes	2	0.0057508	109.66
VISIAQAHSIN(de)FSCEQVR	2058.0317	IPI00308990	IPI00308990	yes	yes	2,3	4.7892E-06	132.13
VISN(de)NCTSYGVIDIGK	1738.856	IPI00331214	IPI00331214	yes	yes	2,3	8.6124E-26	184.22
VISNN(de)CTSYGVIDIGK	1738.856	IPI00331214	IPI00331214	yes	yes	2,3	8.6124E-26	184.22
VITIAN(de)FTTK	1106.6336	IPI00109727	IPI00109727	yes	yes	2	0.033601	77.64
VITIAN(de)FTTKDEGDYFCEIQVSGANPMSSNK	3435.5963	IPI00109727	IPI00109727	yes	yes	4	6.5437E-08	97.94
VITIANFTTKDEGDYFCEIQVSGANPMSSN(de)K	3435.5963	IPI00109727	IPI00109727	yes	yes	4	6.5437E-08	97.94
VITMANQVITVN(de)ISEEGR	1973.0252	IPI00987265	IPI00987265	yes	yes	3	1.5899E-16	166.48
VITNQESPYQN(de)HTGR	1742.8336	IPI00125310	IPI00125310	yes	no	2,3	4.5903E-18	177.04
VIVAPPSEEAN(de)TTK	1454.7617	IPI00875419	IPI00875419	yes	no	2	0.0091963	82.102
VIVIITDGEASDKGN(de)ISAAHDITR	2494.3027	IPI00894972	IPI00894972	yes	no	3	3.3119E-05	101.64
VIVPPAPPSCSIQGVPYVGTN(de)VTINCK	2866.4721	IPI00126827	IPI00126827	yes	no	3,4	5.3763E-09	109.85
VIYIPAYN(de)CTIRPVSK	1893.0182	IPI00469387	IPI00469387	yes	no	3	0.0029863	99.531
VKN(de)STCIDDSWIHPK	1798.8672	IPI00122584	IPI00122584	yes	yes	3	6.5593E-08	153.81
VKPTPPYN(de)ISVTNSEEISSIIK	2415.2897	IPI00120155	IPI00120155	yes	yes	3	3.2659E-22	159.95
VMSWWDYGYQIAGMAN(de)R	2046.908	IPI00316469	IPI00316469	yes	yes	2,3	4.8519E-28	182.57
VMSWWDYGYQITAMAN(de)R	2090.9343	IPI00109108	IPI00109108	yes	yes	2,3	4.4559E-22	177.84
VN(de)GTDIAPDIINGSQIIIR	2008.0953	IPI00131693	IPI00131693	yes	no	3	3.0583E-06	131.25
VN(de)GTITQVIIVGAPTHDDVSK	2163.1535	IPI00225715	IPI00225715	yes	yes	2,3	1.7238E-48	192.62
VN(de)ISFPSAQSIPASDTHIK	2011.0375	IPI00624663	IPI00624663	yes	yes	2,3	3.7963E-94	237.3

VN(de)ITVIPSITSR	1298.7558	IPI00323857	IPI00323857	yes	no	2	0.0007722	114.24
VN(de)KTEEDYAR	1223.5782	IPI00133103	IPI00133103	yes	no	2,3	0.0006336	130.01
VN(de)KTEEDYARDSIFVR	1940.9592	IPI00133103	IPI00133103	yes	no	2	0.0024844	106.3
VN(de)SSIHSQISR	1226.6367	IPI00400016	IPI00400016	yes	no	2	0.0045714	96.604
VNCEERN(de)VTGIENFTIK	2021.984	IPI00378224	IPI00378224	yes	yes	2,3	3.1557E-36	192.24
VNCEERNVTGIEN(de)FTIK	2021.984	IPI00378224	IPI00378224	yes	yes	2,3	3.1557E-36	192.24
VNDNK(de)TAAEEAIR	1429.7161	IPI00400016	IPI00400016	yes	no	2	0.0014269	131.32
VNGTDIAPDIIN(de)GSQIIIR	2008.0953	IPI00131693	IPI00131693	yes	no	3	3.0583E-06	131.25
VNRFN(de)STEYQVVTR	1711.8642	IPI00119063	IPI00119063	yes	no	2,3	0.0003123	126.71
VNYEGGTWDWIAEAISSN(de)HTR	2405.1036	IPI00134585	IPI00134585	yes	yes	3	1.4842E-22	161.47
VPFIFNINPATTN(de)FTGSCQPQSAQIR	2907.4338	IPI00222967	IPI00222967	yes	no	3,4	8.892E-47	188.24
VPIHCTN(de)GSVVYHEVINAMQCR	2583.2145	IPI00798576	IPI00798576	yes	yes	3	0.0072084	67.644
VPMMVQSGN(de)ISYFR	1627.7851	IPI00116105	IPI00116105	yes	yes	3	3.7284E-05	136.3
VPNNAIEGIEN(de)ITAIYIHHNEIQEVGSSMR	3347.6568	IPI00120187	IPI00120187	yes	yes	3,4,5	3.817E-15	125.54
VPSN(de)STETVIESDQFQPGVR	2189.06	IPI00119299	IPI00119299	yes	no	3	1.452E-09	141.18
VPTREENIQVYN(de)VTK	1788.937	IPI00850698	IPI00850698	yes	yes	2	0.0055858	99.451
VQGGSSVWGSVITHN(de)SSAITYQSWGR	2763.3365	IPI00453977	IPI00453977	yes	no	3,4	1.9265E-13	126.45
VQPFN(de)VTK	931.51272	IPI00222967	IPI00222967	yes	no	1,2	0.018024	101.43
VQPIASSTIIHSDITSVYGTVVMN(de)R	2687.3953	IPI00674255	IPI00674255	yes	yes	3,4	8.2671E-17	145.04
VQPMTASN(de)WTIVMEGEWMIK	2350.116	IPI00453829	IPI00453829	yes	no	3	3.2083E-14	150.91
VQSIQTIAAN(de)NSAIAK	1627.8893	IPI00129304	IPI00129304	yes	yes	2,3	1.3044E-09	154.39
VREAN(de)ITEDQIIFFPK	1919.0153	IPI00122122	IPI00122122	yes	no	2,3,4	3.0911E-05	134.76
VRIDPPC(de)TNTTAPSNYINNPYVR	2661.2969	IPI00658539	IPI00658539	yes	no	3	2.7013E-16	148.06
VSESEKSQIVN(de)ETHWQYYGTSDTR	2843.2998	IPI00749655	IPI00749655	yes	no	3	1.3024E-13	135.98
VSGQMHMQN(de)ITFQTEASVAQQEK	2591.2108	IPI00312711	IPI00312711	yes	no	3	4.7566E-08	109.5
VSGQMHMQN(de)ITFQTEASVAQQEKEFK	2995.4168	IPI00312711	IPI00312711	yes	no	3,4	0.005255	65.184
VSHVIN(de)DTHMK	1279.6343	IPI00674255	IPI00674255	yes	yes	2,3	0.0068452	91.961
VSIQEIPGSEHIEMIAN(de)ATTIAYIK	2727.4153	IPI00124428	IPI00124428	yes	yes	3,4	4.8931E-57	196.89
VSIQEIPGSEHIEMIAN(de)ATTIAYIKR	2883.5164	IPI00124428	IPI00124428	yes	yes	3	0.0025698	70.699
VSITN(de)VSISDEGR	1375.6943	IPI00856723	IPI00856723	yes	no	2	0.0004167	133.48
VSNYIGQAN(de)QSAWITVIPK	2088.1004	IPI00828688	IPI00828688	yes	no	3	3.2042E-05	118.48
VSQVIHEGGHN(de)VTK	1503.7794	IPI00463764	IPI00463764	yes	no	2,3,4	5.3145E-69	224.59
VSQVIHEGGHN(de)VTKIIYESANIPDFRK	3050.5938	IPI00463764	IPI00463764	yes	no	3,4	1.9579E-05	97.5
VSSESINF(de)DPVDKVKPTPPYNISVTNSEEISSIIK	3832.9622	IPI00120155	IPI00120155	yes	yes	4	0.016783	45.325
VSTIYANN(de)GSVIQGSTVASVYHK	2394.2179	IPI00121114	IPI00121114	yes	yes	2,3,4	7.571E-223	291.78
VSTIYANN(de)GSVIQGSTVASVYHKR	2550.319	IPI00121114	IPI00121114	yes	yes	3,4	7.553E-05	97.271
VSTVTIVSATSTTAN(de)MTMSPEGR	2340.1301	IPI00406603	IPI00406603	yes	yes	2,3,4	2.0485E-175	276.68
VTGITTSTTEITWDPPVIAERNGHITN(de)YTVVYR	3703.8846	IPI00110264	IPI00110264	yes	yes	3,4	8.4426E-06	81.297
VTIDFN(de)ITDPENGPVIDDAIPNSVHGHIPFAK	3441.7205	IPI01026704	IPI01026704	yes	yes	3,4	4.119E-30	144.7
VTIDFN(de)ITNPENGPVIDDAIPNSVHGHIPFAK	3440.7365	IPI00466371	IPI00466371	yes	yes	4,5	9.2159E-09	98.491
VTIIPN(de)QTHYVVPK	1607.9035	IPI00742414	IPI00742414	yes	no	3	0.0031893	97.463
VTIN(de)ESEICAGAEK	1519.7188	IPI00116509	IPI00116509	yes	no	2	0.0018044	104.45
VTINNWVAN(de)K	1157.6193	IPI00136642	IPI00136642	yes	yes	2	0.020004	87.568
VTINNWVAN(de)KTEGR	1600.8322	IPI00136642	IPI00136642	yes	yes	2,3	4.5438E-24	188.12
VTIWVHPFVNYN(de)SSSFGEGVER	2523.2183	IPI00464256	IPI00464256	yes	yes	3	1.4107E-05	100.92
VTNIMTGQTAN(de)ATSIIGTMTDAFPK	2582.272	IPI00923031	IPI00923031	yes	no	3,4	1.2668E-08	110.67
VTNSNANAAGPIIVAGYN(de)VSGSVR	2330.1979	IPI00222429	IPI00222429	yes	yes	3	3.9332E-22	140.73
VTPGAIIGISN(de)ITHISIK	1833.0724	IPI00318748	IPI00318748	yes	no	3	0.014226	69.331
VTTCHIPQQN(de)ATIYK	1772.888	IPI00410951	IPI00410951	yes	no	2,3	0.0007379	110.98
VTVEGMEYVFYN(de)DTK	1793.8182	IPI00405742	IPI00405742	yes	no	2,3	4.7927E-12	136.48
VVAVSPAN(de)ISR	1111.635	IPI00229992	IPI00229992	yes	yes	2	0.0097898	87.363
VVAVSPAN(de)ISREER	1525.8213	IPI00229992	IPI00229992	yes	yes	2,3	0.0002186	131.69
VVDGEREN(de)VSMVDYAHNNYQAQSAVPIR	3160.4996	IPI00134743	IPI00134743	yes	yes	3	1.0408E-08	110.06
VVDKGN(de)GSKPTSPEEVK	1769.9159	IPI00330632	IPI00330632	yes	no	3	0.0015576	104.94
VVFISPAVPEEPEAYN(de)ITVIIR	2455.3363	IPI00405742	IPI00405742	yes	no	2,3,4	2.896E-49	197.1
VVIAGSN(de)MTICCMSPTK	1867.8664	IPI00119299	IPI00119299	yes	no	2,3	2.7073E-12	156.67
VVIGEN(de)ITSNCPEVIYEIKEETPVFYK	3169.5893	IPI00674255	IPI00674255	yes	yes	3	2.7011E-23	149.73
VVIHPN(de)HSVVDIGIIK	1739.0094	IPI00409148	IPI00409148	yes	yes	2,3,4	8.6636E-139	266.45
VVIISGVEPRPPTPQVQFTIN(de)ASSEDHKR	3200.6942	IPI00122973	IPI00122973	yes	no	3,4,5	2.6823E-09	114.2
VVINFN(de)GTSQEIMAVSEHR	2130.0528	IPI00111183	IPI00111183	yes	yes	3	9.901E-33	180.62
VVMDIPYEIWN(de)ETSAEVADIK	2421.1774	IPI00119039	IPI00119039	yes	no	2,3	4.5552E-49	197.09
VVMDIPYEIWN(de)ETSAEVADIKK	2549.2723	IPI00119039	IPI00119039	yes	no	2,3,4	1.0683E-08	127.01
VVN(de)VSEIYGTPCTK	1565.7759	IPI00626950	IPI00626950	yes	no	3	0.0027177	99.844
VVN(de)VSEIYGTPCTKR	1721.8771	IPI00626950	IPI00626950	yes	no	3	0.026524	74.987
VVNCQEFAISAN(de)ASR	1664.7941	IPI00118333	IPI00118333	yes	yes	2	0.0013323	104.44
VVPEPNIVIN(de)FSATAIR	1839.0254	IPI00654185	IPI00654185	yes	no	3	0.0006208	99.344
VVRPDSEIGERPPEDN(de)QSFQYDHEAFIGK	3358.5854	IPI00137831	IPI00137831	yes	yes	4	1.4354E-13	121.38
VVSVDISFRPIN(de)ETFPVVYIETPK	2748.4738	IPI00624663	IPI00624663	yes	yes	2,3,4	1.1572E-124	242.18
VVSVDISFRPIN(de)ETFPVVYIETPKR	2904.5749	IPI00624663	IPI00624663	yes	yes	3,4,5	5.535E-243	219.61
VWDTAAAIN(de)R	1115.5724	IPI00226563	IPI00226563	yes	no	2	0.010745	97.456
VWN(de)QTEQEPAAYHIISICFVR	2560.2533	IPI00129079	IPI00129079	yes	no	3	0.010724	66.068
VWPDGVIPFVIGGN(de)FTGSQR	2145.1007	IPI00125182	IPI00125182	yes	no	2,3	8.2354E-07	131.25
VWPDYPN(de)ITVDPSIGWDHQVEQYR	2913.3722	IPI00848693	IPI00848693	yes	yes	3	0.0003648	102.07
VWVCDRDN(de)DCVDGSDEPANCTQMTCGVDEFR	3706.4487	IPI00119063	IPI00119063	yes	yes	3,4	1.72E-58	180.26
VWVCDRDNDCVDGSDEPAN(de)CTQMTCGVDEFR	3706.4487	IPI00119063	IPI00119063	yes	yes	3,4	1.72E-58	180.26
VYEEVINV(de)TPNDGFAK	1793.8836	IPI00624896	IPI00624896	yes	no	2	0.0017502	92.264
VYGGIVN(de)QSEINEGTAFFR	2100.0276	IPI00416285	IPI00416285	yes	yes	2,3	1.1702E-42	197.84
VYGQNINN(de)ETWSR	1579.7379	IPI00762609	IPI00762609	yes	no	2	0.0037803	95.631
VYIHPFHIIYHN(de)K	1679.8936	IPI00654069	IPI00654069	yes	yes	2,3,4	1.4134E-15	175.15
VYIN(de)DSVEISR	1293.6565	IPI00126769	IPI00126769	yes	yes	2	0.033214	72.089
VYIN(de)DSVEISRNENK	1778.8799	IPI00126769	IPI00126769	yes	yes	2	1.1128E-32	188.59
VYMKN(de)VTVVIR	1320.7588	IPI00469218	IPI00469218	yes	yes	2,3	1.1019E-05	147.74
VYQIDGNYPGSSHVVIDHETYIIN(de)ITQANAAGGTPSWK	4115.0025	IPI00137792	IPI00137792	yes	yes	4	3.344E-05	63.44
VYSIPGREN(de)YSSVDANGIQSQMISR	2770.3344	IPI00127100	IPI00127100	yes	yes	3	5.1565E-05	96.016
VYTYADTPNDFQISN(de)FSIPEEDTK	2793.2657	IPI00108811	IPI00108811	yes	yes	2,3,4	3.4655E-16	141.36
VYTYADTPNDFQISN(de)FSIPEEDTKIK	3034.4448	IPI00108811	IPI00108811	yes	yes	3,4	2.0866E-27	164.94

VYTYADTPNDFQISN(de)FSIPEEDTKIKIPIIHQAIK	4048.0833	IPI00108811	IPI00108811	yes	yes	4	7.2636E-15	111.81
VYVYAVN(de)QTR	1211.6299	IPI00466371	IPI01026704	no	no	2	0.0021195	110.39
WAAVVVPIGKEQN(de)YTCHVHHK	2472.2485	IPI00850057	IPI00850057	yes	no	3	0.0065659	78.078
WASVVVPIGKEQN(de)YTCR	2006.0044	IPI00109996	IPI00109996	yes	no	2,3	3.2409E-11	154.51
WECKN(de)DTIFGIK	1509.7286	IPI00126186	IPI00126186	yes	yes	2	0.002764	111.79
WGEVDIIGN(de)CSQFYPDR	2054.9156	IPI00320675	IPI00320675	yes	yes	2	2.5978E-08	142.78
WGHN(de)VTEFQQR	1400.6585	IPI00754386	IPI00754386	yes	yes	2	0.025162	75.819
WKPPSDPNGN(de)ITHYIVYWER	2471.2022	IPI00128358	IPI00128358	yes	no	3	0.0026183	89.846
WTDNTEYN(de)NTIPIRGEER	2207.0243	IPI00276430	IPI00276430	yes	yes	2,3	1.0575E-08	145.33
WTGHN(de)VTVVQR	1295.6735	IPI00119063	IPI00119063	yes	yes	2	0.0005031	121.68
YAFQEIIGDISFIIPTIN(de)FSK	2415.2726	IPI00122557	IPI00122557	yes	no	2,3	1.4196E-17	157.22
YAIICGN(de)RTDAGQCPEGYVCVK	2530.1403	IPI00341285	IPI00341285	yes	yes	3	7.4463E-08	123.63
YATENDISSIHN(de)STITCIVNQTTSITGTSPEIMEK	3854.819	IPI00230319	IPI00230319	yes	no	3,4	1.9522E-15	111.97
YATENDISSIHNSTITCIVN(de)QTTSITGTSPEIMEK	3854.819	IPI00230319	IPI00230319	yes	no	3,4	1.9522E-15	111.97
YAVYWN(de)SSNPR	1355.6258	IPI00114364	IPI00114364	yes	no	2	0.0045714	96.604
YDIIIINENGIIISN(de)VSEPATAR	2514.333	IPI00229935	IPI00229935	yes	yes	3	4.3323E-05	96.55
YDIPASINYIIN(de)K	1522.8031	IPI00129265	IPI00129265	yes	yes	2,3	0.0002331	138.99
YDKSEDVGAAAMIN(de)YTHIIMEAVPGHPAIYR	3431.6642	IPI00874858	IPI00874858	yes	no	3,4	2.0082E-08	103.25
YDN(de)HSSSVIAAVVFEHSFNHSQDPIPIAVK	3307.6262	IPI00153807	IPI00153807	yes	no	4	0.018136	49.618
YDNHSSSVIAAVVFEHSFN(de)HSQDPIPIAVK	3307.6262	IPI00153807	IPI00153807	yes	no	4	0.018136	49.618
YDVN(de)RSDSGGSIQIEEGYFVHHFAPENIPTMSK	3723.7264	IPI00312711	IPI00312711	yes	no	4	2.6831E-34	149.04
YEPIPFSASMN(de)ESTPTGITDHIAQGPEPNIEESPK	3782.7622	IPI00133608	IPI00133608	yes	yes	4	1.8049E-17	116.34
YEPIPFSASMN(de)ESTPTGITDHIAQGPEPNIEESPKR	3938.8633	IPI00133608	IPI00133608	yes	yes	4	1.3284E-05	68.93
YEQAKN(de)ISQDIEK	1564.7733	IPI00400016	IPI00400016	yes	no	2	0.033837	81.703
YEQIQN(de)ETR	1179.552	IPI00154056	IPI00154056	yes	no	2	0.0030228	118.4
YEVDWN(de)QSTIDCVDPISSIAANR	2652.2126	IPI00331032	IPI00331032	yes	no	3	4.8478E-17	154.6
YEVDWNQSTIDCVDPISSIAAN(de)R	2652.2126	IPI00331032	IPI00331032	yes	no	3	4.8478E-17	154.6
YFFIQSVDSDGRN(de)FTSSPPGQTQFK	2852.3406	IPI00344686	IPI00344686	yes	yes	3,4	1.035E-34	170.14
YFTNRIDVSQN(de)VSSDTDQSCESTK	2780.2195	IPI00120769	IPI00120769	yes	no	3	6.3971E-13	132.01
YGHPN(de)GTQGNSTMWPVFTSTEQK	2566.1547	IPI00131168	IPI00131168	yes	yes	3	3.1521E-08	115.42
YGHPNGTQGN(de)STMWPVFTSTEQK	2566.1547	IPI00131168	IPI00131168	yes	yes	3	3.1521E-08	115.42
YGIVVIDECPGVGIVIPQSFGN(de)ESIR	2817.4371	IPI00309230	IPI00309230	yes	no	2,3	1.9442E-27	159.98
YGKN(de)DSITITQIK	1479.7933	IPI00123428	IPI00123428	yes	no	2,3	6.6153E-31	194.98
YGMQNSGNEAAWN(de)YTIEQYQK	2494.0859	IPI00134585	IPI00134585	yes	yes	3	2.3476E-59	202.6
YGN(de)MTQDHVMHIITR	1814.8556	IPI00115892	IPI00115892	yes	no	2,3	0.0027557	89.624
YGTAIVHIYVN(de)ETIANR	1933.0058	IPI00719927	IPI00719927	yes	no	2,3	7.1617E-21	175.51
YGTAIVHIYVNETIAN(de)R	1933.0058	IPI00719927	IPI00719927	yes	no	2,3	7.1617E-21	175.51
YGYEN(de)DTEIANIYDEMVAK	2236.9834	IPI00848693	IPI00848693	yes	yes	3	5.0786E-09	137.45
YHGFIN(de)TSYHR	1393.6527	IPI00154056	IPI00154056	yes	no	2,3	4.1151E-06	147.37
YHHYSSN(de)FSIPK	1478.6943	IPI00129485	IPI00129485	yes	no	2,3	0.0007928	122.7
YHYN(de)ASIIDGTIIDSTWNIGK	2380.1699	IPI00130486	IPI00130486	yes	yes	3	1.7039E-09	135.09
YHYN(de)GTFIDGTIFDSSHNR	2243.0032	IPI00130486	IPI00130486	yes	yes	2,3	8.7438E-26	174.78
YHYN(de)GTIIDGTAFDNSYSR	2192.9763	IPI00122493	IPI00122493	yes	no	2,3	2.358E-05	120.9
YHYN(de)GTIIDGTIFDSSYSR	2208.0124	IPI00130486	IPI00130486	yes	yes	2,3	2.6059E-25	168.05
YIEIGN(de)ETIIR	1319.7085	IPI00850057	IPI00850057	yes	no	2	0.0005716	114.24
YIIVAQIN(de)ISR	1288.7503	IPI00228567	IPI00228567	yes	yes	2	0.0006413	113.47
YIKNGN(de)ATIIR	1261.7143	IPI00985828	IPI00109996	no	no	2,3	1.1114E-05	151.79
YIMWGISSDIWGEKPN(de)TSYIIGK	2657.32	IPI00323624	IPI00323624	yes	no	3	0.016583	65.808
YIN(de)ETQQITQK	1364.6936	IPI00123223	IPI00123223	yes	yes	2	1.2131E-41	203.61
YIN(de)ETQQITQKIK	1605.8726	IPI00123223	IPI00123223	yes	yes	2	0.0009547	135.81
YIN(de)FSTSEKEK	1344.6561	IPI00135010	IPI00135010	yes	no	2	0.0029553	109.24
YIQPIIAVQFTN(de)ITVDTEIR	2333.2631	IPI00121550	IPI00121550	yes	yes	2,3	4.4779E-110	241.68
YIQVVYIHNNN(de)ISAVGQNDFCR	2623.2602	IPI00123196	IPI00123196	yes	yes	2,3,4	5.4057E-86	221.79
YIYIASN(de)HSNK	1308.6463	IPI00118173	IPI00118173	yes	no	2	0.0040377	98.156
YKGIN(de)ITEDTYKPR	1696.8784	IPI00119809	IPI00119809	yes	yes	2,3	9.5074E-23	182.02
YKGN(de)ASAIFIIPDQGR	1748.921	IPI00128076	IPI00128076	yes	yes	2,3	3.7648E-05	134.24
YKGTAGNAIMDGASQIVGEN(de)R	2151.0379	IPI00279079	IPI00279079	yes	yes	2,3	4.3283E-48	193.22
YKVPAEIIAN(de)TSENAGFCIPEGNCMDSGVINISICK	3970.8573	IPI00127447	IPI00127447	yes	yes	4,5,6	1.5077E-43	152.13
YKVPAEIIANTSENAGFCIPEGNCMDSGVIN(de)ISICK	3970.8573	IPI00127447	IPI00127447	yes	yes	4,5,6	1.5077E-43	152.13
YKYETTISPTSN(de)ISSNSFICIENR	2823.3385	IPI00625835	IPI00625835	yes	no	2,3,4	4.7414E-94	227.4
YKYETTISPTSNISSNSFICIEN(de)R	2823.3385	IPI00625835	IPI00625835	yes	no	2,3,4	4.7414E-94	227.4
YMDQNSDGWQDGVGYIN(de)SSEGAVGR	2704.146	IPI00114236	IPI00114236	yes	no	3	1.4653E-16	143.31
YMGIPIPEDNIDHYRN(de)STVMSR	2607.221	IPI00125813	IPI00125813	yes	no	2,3	6.1394E-05	96.544
YMN(de)YTSIITFHCK	1676.7691	IPI00308971	IPI00308971	yes	yes	3	0.0012301	107.97
YN(de)HTGQVVIYK	1320.6826	IPI00466371	IPI01026704	no	no	2,3	9.2469E-10	160.18
YPHKPEIN(de)STTHPGADIK	2004.0065	IPI00114206	IPI00114206	yes	yes	2,3	2.0802E-37	195.6
YPHKPEIN(de)STTHPGADIKENFCR	2710.2922	IPI00114206	IPI00114206	yes	yes	3	8.9811E-05	96.234
YPQDYQFYIQN(de)FTAIPINTVVPPQR	3011.5181	IPI00110852	IPI00110852	yes	no	2,3,4	8.0962E-21	149.44
YPTPGEAPGVVGNFN(de)K	1645.81	IPI00112614	IPI00112614	yes	yes	2,3	0.0003888	118.37
YQEGDITIYVINIHN(de)VTK	2119.095	IPI00170098	IPI00170098	yes	no	3	0.0001347	108.77
YQEIIEIESSIN(de)KTQWK	2108.079	IPI00990932	IPI00990932	yes	no	2,3	2.3386E-74	228.46
YQFNTNVVFSDN(de)GTIVDR	2087.9912	IPI00321375	IPI00321375	yes	no	2,3	9.9369E-49	206.64
YQFNTNVVFSDN(de)GTIVDRYR	2407.1557	IPI00321375	IPI00321375	yes	no	3	0.0050712	82.877
YQHVGQVIIFQAPEAGGRWN(de)QTQK	2754.399	IPI00894972	IPI00894972	yes	no	3,4	3.4216E-64	148.85
YQMDN(de)VSSIVQIIGSHIEDVNADIIQTK	3129.5652	IPI00330594	IPI00330594	yes	yes	3,4	4.95E-33	157.9
YRAEFAVAN(de)DTGFVDIPQQEK	2397.1601	IPI00128154	IPI00128154	yes	no	2,3	2.3632E-30	174.2
YSIYKDPAGWISINPIN(de)GTVDTTAVIDR	3078.5662	IPI00123746	IPI00123746	yes	yes	3	1.4414E-13	122.84
YSN(de)ESQDISVNGYNCTTSSVSSAIR	2738.209	IPI00322304	IPI00322304	yes	yes	2,3	1.5218E-56	195.7
YSNESQDISVNGYN(de)CTTSSVSSAIR	2738.209	IPI00322304	IPI00322304	yes	yes	2,3	1.5218E-56	195.7
YSQAAN(de)STKEIDDCEQANK	2170.9437	IPI00187353	IPI00187353	yes	yes	2,3	4.8212E-13	148.06
YSVIITAEKPVGDISSPN(de)ETK	2247.1634	IPI00125497	IPI00125497	yes	yes	3	8.9712E-08	124.63
YSVQHMYFTYN(de)ISDTEHFPNAISK	2891.3225	IPI00469218	IPI00469218	yes	yes	2,3,4	6.2351E-35	168.3
YTGN(de)ASAIFIIPDQGR	1721.8737	IPI00135635	IPI00135635	yes	no	2,3	1.0993E-57	214.81
YTGN(de)ASAIIIIPDQGR	1687.8893	IPI00131830	IPI00131830	yes	no	2,3	2.0817E-42	186.98
YTSFEYPKN(de)ISFACNPGFFINGTSSSK	3062.412	IPI00322463	IPI00322463	yes	no	2,3,4	6.9947E-39	177.91
YTSFEYPKNISFACNPGFFIN(de)GTSSSK	3062.412	IPI00322463	IPI00322463	yes	no	2,3,4	6.9947E-39	177.91

YVMIPVADQDKCVVHYEN(de)STVPEKK	2948.4412	IPI00409148	IPI00409148	yes	yes	3,4	2.4143E-45	187.47
YVVVSTPEKSQITISNIDVN(de)VDPGTYVCNATNAQGTTR	4111.0168	IPI00408495	IPI00408495	yes	no	4	1.921E-15	112.57
YVYVADIIAHEIHVIEKQPNMN(de)ITQIK	3178.6849	IPI00310567	IPI00310567	yes	yes	3,4	6.4614E-19	142.23
YWGN(de)ITVVMGEHDFSEKDGDEQVR	2810.2606	IPI00307890	IPI00307890	yes	yes	3	0.002311	81.016
YYHGEISYIN(de)VTR	1613.7838	IPI00404551	IPI00404551	yes	no	2,3	3.9853E-23	187.44
YYHGEISYIN(de)VTRK	1741.8788	IPI00404551	IPI00404551	yes	no	2,4	8.7651E-32	192.39
YYN(de)QSAGGSHTIQWMAGCDVESDGR	2788.1606	IPI00985828	IPI00985828	yes	no	3,4	1.3188E-68	207.43
YYN(de)YTISINGK	1334.6507	IPI00221426	IPI00221426	yes	yes	2	0.0013515	111.52

N-terminal Ser/Thr 257
ratio 13.90%

Conventional HC strategy 1

De-glycopeptides Sequence	Mass	Proteins	Leading ra	Unique (G	Unique (P	Charges	PEP	Score
YYN(de)YTISINGK	1334.6507	IPI00221426	IPI00221426	yes	yes	2	6.9856E-15	184.03
YYN(de)QSKGGSHTFQR	1671.7754	IPI00850057	IPI00850057	yes	no	2	0.0029419	145
YYN(de)QSAGGSHTIQWMAGCDVESDGR	2788.1606	IPI00985828	IPI00985828	yes	no	2,3	7.9418E-56	207.43
YYHGEISYIN(de)VTRK	1741.8788	IPI00404551	IPI00404551	yes	no	2,3,4	0.0005892	148.41
YYHGEISYIN(de)VTR	1613.7838	IPI00404551	IPI00404551	yes	no	2,3	3.838E-09	160.84
YVYVADIIAHEIHVIEKQPNMN(de)ITQIK	3178.6849	IPI00310567	IPI00310567	yes	yes	3,4	3.3737E-147	245.66
YVQN(de)GTYTAK	1143.556	IPI00115793	IPI00115793	yes	yes	2	0.017716	99.283
YVMIPVADQDKCVVHYEN(de)STVPEKK	2948.4412	IPI00409148	IPI00409148	yes	yes	3	1.3197E-08	122.19
YTSFEYPKNISFA(de)CNPGFFINGTSSSK	3062.412	IPI00322463	IPI00322463	yes	no	3	1.92E-74	214.65
YTSFEYPKN(de)ISFACNPGFFINGTSSSK	3062.412	IPI00322463	IPI00322463	yes	no	3	1.92E-74	214.65
YTGN(de)ASAIIIIPDQGR	1687.8893	IPI00131830	IPI00131830	yes	no	2,3	1.4462E-110	241.68
YTGN(de)ASAIFIIPDQGR	1721.8737	IPI00135635	IPI00135635	yes	no	2,3	9.708E-48	209.89
YSVQHMYFTYN(de)ISDTEHFPNAISK	2891.3225	IPI00469218	IPI00469218	yes	yes	2,3,4	3.4254E-18	171.42
YSVIITAEKPVGDISSPN(de)ETK	2247.1634	IPI00125497	IPI00125497	yes	yes	2,3	9.5358E-21	181.54
YSQAAN(de)STKEIDDCEQANK	2170.9437	IPI00187353	IPI00187353	yes	yes	2,3	0.000327	140.89
YSNESQDISVNGYN(de)CTTSSVSSAIR	2738.209	IPI00322304	IPI00322304	yes	yes	2,3	1.7526E-68	213.05
YSN(de)ESQDISVNGYNCTTSSVSSAIR	2738.209	IPI00322304	IPI00322304	yes	yes	2,3	1.7526E-68	213.05
YRAEFAVAN(de)DTGFVDIPQQEK	2397.1601	IPI00128154	IPI00128154	yes	no	2,3	6.2011E-21	183.05
YQMDN(de)VSSIVQIIGSHIEDVNADIIQTK	3129.5652	IPI00330594	IPI00330594	yes	yes	3,4	9.6091E-40	192.94
YQHVGQVIIFQAPEAGGRWN(de)QTQK	2754.399	IPI00894972	IPI00894972	yes	no	3	1.7758E-08	132.14
YQFNTNVVFSDN(de)GTIVDR	2087.9912	IPI00321375	IPI00321375	yes	no	2,3	2.5388E-19	184.32
YQFEVICPAPRPGAASN(de)ISFQAPFR	2822.3963	IPI00311159	IPI00311159	yes	yes	3	1.2311E-08	120.25
YQEIIEIESSIN(de)KTQWK	2108.079	IPI00675799	IPI00675799	yes	no	2	2.2947E-47	209.25
YQEGDITIYVINIHN(de)VTK	2119.095	IPI00170098	IPI00170098	yes	no	2	0.0016833	101.49
YPQDYQFYIQN(de)FTAIPINTVVPPQR	3011.5181	IPI00110852	IPI00110852	yes	no	2,3,4	9.3384E-10	136.14
YPNN(de)GSIVWGK	1233.6142	IPI00848693	IPI00848693	yes	yes	2	0.0087193	102.4
YPHNHHVVNNTIEGN(de)CSSK	2205.9974	IPI00221426	IPI00221426	yes	yes	3	0.025361	73.168
YPHNHHVVN(de)NTIEGNCSSK	2205.9974	IPI00221426	IPI00221426	yes	yes	3	0.025361	73.168
YPHKPEIN(de)STTHPGADIKENFCR	2710.2922	IPI00114206	IPI00114206	yes	yes	3	0.013955	77.037
YPHKPEIN(de)STTHPGADIK	2004.0065	IPI00114206	IPI00114206	yes	yes	2,3	0.0090011	99.392
YPAEGQRPIPN(de)VSIPIR	1906.0425	IPI00453524	IPI00453524	yes	yes	2,3	0.0006845	150.34
YN(de)YTEDPTIIR	1383.667	IPI00137311	IPI00137311	yes	yes	2	0.0015286	130.56
YN(de)HTGQVVIYK	1320.6826	IPI00466371	IPI01026704	no	no	2,3	1.4714E-09	169.52
YN(de)GSIGIWR	1064.5403	IPI00133292	IPI00133292	yes	no	2	0.031481	96.143
YMN(de)YTSIITFHCK	1676.7691	IPI00308971	IPI00308971	yes	yes	2	0.024118	84.468
YMGIPIPEDNIDHYRN(de)STVMSR	2607.221	IPI00125813	IPI00125813	yes	no	3	0.01059	82.877
YMDQNSDGWQDGVGYIN(de)SSEGAVGR	2704.146	IPI00114236	IPI00114236	yes	no	3	2.8288E-05	95.996
YMAIAIN(de)R	950.50077	IPI00123814	IPI00123814	yes	yes	2	0.020618	129.4
YKYETTISPTSNISSNSFICIEN(de)R	2823.3385	IPI00625835	IPI00625835	yes	no	2,3	1.3246E-48	198.1
YKYETTISPTSN(de)ISSNSFICIENR	2823.3385	IPI00625835	IPI00625835	yes	no	2,3	1.3246E-48	198.1
YKGTAGNAIMDGASQIVGEN(de)R	2151.0379	IPI00279079	IPI00279079	yes	yes	2,3	3.5497E-06	153.36
YKGIN(de)ITEDTYKPR	1696.8784	IPI00119809	IPI00119809	yes	yes	2,3	7.3042E-36	200.19
YIYIASN(de)HSNK	1308.6463	IPI00118173	IPI00118173	yes	no	2	0.0069329	107.03
YIQVVYIHNNN(de)ISAVGQNDFCR	2623.2602	IPI00123196	IPI00123196	yes	yes	2,3	1.3508E-247	220.64
YIQPIIAVQFTN(de)ITVDTEIR	2333.2631	IPI00121550	IPI00121550	yes	yes	2,3	1.911E-20	180.08
YIN(de)ETQQITQK	1364.6936	IPI00123223	IPI00123223	yes	yes	2	1.5475E-147	191.79
YIMWGISSDIWGEKPN(de)TSYIIGK	2657.32	IPI00323624	IPI00323624	yes	no	3	0.003814	85.636
YIKNGN(de)ATIIR	1261.7143	IPI00985828	IPI00985828	no	no	2	7.5902E-06	162.26
YIKNGN(de)ATIIR	1261.7143	IPI00985828	IPI00985828	no	no	2	7.5902E-06	162.26
YIINGSHAN(de)ETR	1373.6688	IPI00129968	IPI00129968	yes	yes	2	0.021839	89.43
YIIN(de)GSHANETR	1373.6688	IPI00129968	IPI00129968	yes	yes	2	0.021839	89.43
YIIDFSIFAYPIPN(de)VTK	2000.0659	IPI00330381	IPI00330381	yes	no	2	0.0008131	118.47
YIEIGN(de)ETIIR	1319.7085	IPI00850057	IPI00850057	yes	no	2	2.2153E-39	203.51
YHYN(de)GTIIDGTIFDSSYSR	2208.0124	IPI00130486	IPI00130486	yes	yes	2,3	4.8516E-62	216.3
YHYN(de)GTIIDGTAFDNSYSR	2192.9763	IPI00122493	IPI00122493	yes	no	2,3	0.0002248	139.11
YHYN(de)GTFIDGTIFDSSHNR	2243.0032	IPI00130486	IPI00130486	yes	yes	2,3	3.5324E-19	181.54
YHYN(de)CSIIDGTR	1497.6671	IPI00122493	IPI00122493	yes	no	2	0.020968	83.204
YHYN(de)ASIIDGTIIDSTWNIGK	2380.1699	IPI00130486	IPI00130486	yes	yes	2,3	5.3951E-51	204.47
YHVIHIN(de)TTK	1224.6615	IPI00131021	IPI00131021	yes	yes	2,3	0.002399	125.74
YHHYSSN(de)FSIPK	1478.6943	IPI00129485	IPI00129485	yes	no	2,3	3.097E-05	151.26
YHGFIN(de)TSYHR	1393.6527	IPI00154056	IPI00154056	yes	no	2,3	0.0015949	124.42
YHDYYITSTSNG(de)SIEGIENR	2318.0451	IPI00130754	IPI00130754	yes	yes	2,3	2.0878E-05	119.62
YGTAIVHIYVNETIAN(de)R	1933.0058	IPI00719927	IPI00719927	yes	no	2	2.9382E-08	167.29
YGTAIVHIYVN(de)ETIANR	1933.0058	IPI00719927	IPI00719927	yes	no	2	2.9382E-08	167.29
YGQEQGTAPFQVSN(de)HTGR	1975.9137	IPI00409393	IPI00409393	yes	yes	3	0.000863	106.6
YGN(de)MTQDHVMHIITR	1814.8556	IPI00115892	IPI00115892	yes	no	2,3	0.0026925	101.6
YGMQNSGNEAAWN(de)YTIEQYQK	2494.0859	IPI00134585	IPI00134585	yes	yes	2,3	3.1749E-136	250.26
YGKN(de)DSITITQIK	1479.7933	IPI00123428	IPI00123428	yes	no	2,3	0.0081635	136.17
YGIVVIDECPGVGIVIPQSFGN(de)ESIR	2817.4371	IPI00309230	IPI00309230	yes	no	3	5.1452E-11	113.7
YGHPNGTQGN(de)STMWPVFTSTEQK	2566.1547	IPI00131168	IPI00131168	yes	yes	3	6.1351E-09	149.44
YGHPN(de)GTQGNSTMWPVFTSTEQK	2566.1547	IPI00131168	IPI00131168	yes	yes	3	6.1351E-09	149.44
YFTNRI(de)DVSQNVSSDTDQSCESTK	2780.2195	IPI00120769	IPI00120769	yes	no	3	1.7873E-08	144.57

YFFIQSVDSDGRN(de)FTSSPPGQTQFK	2852.3406	IPI00344686	IPI00344686	yes	yes		3	2.6508E-55	207.07
YEVDWNQSTIDCVDPISSIAAN(de)R	2652.2126	IPI00331032	IPI00331032	yes	no	2,3		5.1565E-18	172.75
YEVDWN(de)QSTIDCVDPISSIAANR	2652.2126	IPI00331032	IPI00331032	yes	no	2,3		5.1565E-18	172.75
YEQIQN(de)ETR	1179.552	IPI00154056	IPI00154056	yes	no		2	0.0030725	147.62
YE(de)TEKNNGAGYFIEHIAFK	2230.0695	IPI00111885	IPI00111885	yes	no		3	2.1393E-12	173.51
YDVN(de)RSDSGGSIQIEEGYFVHHFAPENIPTMSK	3723.7264	IPI00312711	IPI00312711	yes	no	3,4		6.4432E-20	118.42
YDKSEDVGAAAMIN(de)YTHIIMEAVPGHPAIYR	3431.6642	IPI00874858	IPI00874858	yes	no	3,4		4.0797E-19	139.22
YDIPASINYIIN(de)K	1522.8031	IPI00129265	IPI00129265	yes	yes		2	1.0606E-09	166.62
YAVYWN(de)SSNPR	1355.6258	IPI00114364	IPI00114364	yes	no		2	0.0022899	122.42
YATENDISSIHNSTITCIVN(de)QTTSITGTSPEIMEK	3854.819	IPI00230319	IPI00230319	yes	no	3,4		7.6954E-15	103.14
YATENDISSIHN(de)STITCIVNQTTSITGTSPEIMEK	3854.819	IPI00230319	IPI00230319	yes	no	3,4		7.6954E-15	103.14
YAFQEIIGDISFIIPTIN(de)FSK	2415.2726	IPI00122557	IPI00122557	yes	no		3	1.0561E-05	139.11
WYDHSN(de)MTFDKWADQDGEDIVDTCGFIYTK	3656.55	IPI00120856	IPI00120856	yes	no		3	8.7916E-69	210.22
WVITAAHCIIYPPWDKN(de)FTENDIIVR	3170.6012	IPI00114206	IPI00114206	yes	yes		3	2.7756E-13	127.58
WVGN(de)WTYEIK	1294.6346	IPI00153143	IPI00153143	yes	yes		2	1.4763E-07	169.65
WVDGASINFSHWNSGEPN(de)DSR	2374.0363	IPI00223352	IPI00223352	yes	yes	2,3		3.2013E-07	157.47
WVDGASIN(de)FSHWNSGEPNDSR	2374.0363	IPI00223352	IPI00223352	yes	yes	2,3		3.2013E-07	157.47
WTGHN(de)VTVVQR	1295.6735	IPI00119063	IPI00119063	yes	yes		2	1.7387E-06	160.52
WTDNTEYN(de)NTIPIRGEER	2207.0243	IPI00276430	IPI00276430	yes	yes	2,3		0.0006569	147.07
WTDNTEYN(de)NTIPIR	1735.8166	IPI00276430	IPI00276430	yes	yes		2	0.043072	67.102
WSN(de)DSATISFTKPWSQGK	2038.9749	IPI00320065	IPI00320065	yes	no		2	2.5799E-07	161.91
WSFSN(de)GTSWQK	1326.5993	IPI00315576	IPI00315576	yes	yes		2	4.8508E-10	174.77
WQN(de)DTGPSDKSDISQK	1804.8228	IPI00222305	IPI00222305	yes	yes		2	8.3226E-75	224.59
WQIHN(de)ISGK	1081.5669	IPI00987265	IPI00987265	yes	no		2	0.01069	114.4
WKPPSDPNGN(de)ITHYIVYWER	2471.2022	IPI00128358	IPI00128358	yes	no		3	0.014471	87.24
WIQQEFHSN(de)ASIPTHIVTFNVIEK	2837.4501	IPI00624345	IPI00624345	yes	yes		3	8.3393E-09	144.27
WIN(de)ETQIK	1030.5447	IPI00453524	IPI00453524	yes	yes		2	0.0002413	149.82
WICDGDNDCGNSEDESN(de)ATCSAR	2631.9497	IPI00119063	IPI00119063	yes	yes	2,3		3.9508E-09	140.73
WGHN(de)VTEFQQR	1400.6585	IPI00754386	IPI00754386	yes	yes	2,3		1.3503E-06	162.38
WGEVDIIGN(de)CSQFYPDRYYEK	2638.1798	IPI00320675	IPI00320675	yes	yes	2,3		1.9174E-05	133.86
WGEVDIIGN(de)CSQFYPDR	2054.9156	IPI00320675	IPI00320675	yes	yes		2	4.9825E-36	196.71
WFHN(de)ESIIPHQDANYVIQSAR	2524.2248	IPI00162870	IPI00162870	yes	yes		3	2.9066E-06	126.8
WECKN(de)DTIFGIK	1509.7286	IPI00126186	IPI00126186	yes	yes	2,3		0.0047107	118.87
WASVVVPIGKEQN(de)YTCR	2006.0044	IPI00109996	IPI00109996	yes	no	2,3		0.0038219	109.83
WASVVVPIGKEQN(de)YTCHVYHEGIPEPITIR	3491.766	IPI01026927	IPI01026927	yes	no	3,4		2.9883E-05	89.979
WAAVVVPIGKEQN(de)YTCHVHHK	2472.2485	IPI00850057	IPI00850057	yes	no	2,3		1.4991E-05	131.25
VYVYAVN(de)QTR	1211.6299	IPI00466371	IPI01026704	no	no		2	3.9724E-12	178.08
VYTYADTPNDFQISN(de)FSIPEEDTKIKIPIIHQAIK	4048.0833	IPI00108811	IPI00108811	yes	yes		3	0.0007131	77.322
VYTYADTPNDFQISN(de)FSIPEEDTKIK	3034.4448	IPI00108811	IPI00108811	yes	yes		3	1.5581E-29	179.58
VYTVSSSPSCYMYVYVN(de)TTEVAIEQDIAYIQEIK	3962.8846	IPI00136012	IPI00136012	yes	yes		4	0.0010248	64.726
VYSIPGREN(de)YSSVDANGIQSQMISR	2770.3344	IPI00127100	IPI00127100	yes	yes		3	6.4552E-18	168.84
VYQIDGNYPGSSHVVIDHETYIIN(de)ITQANAAGGTPSWKR	4271.1036	IPI00137792	IPI00137792	yes	yes		4	0.024165	38.366
VYQIDGNYPGSSHVVIDHETYIIN(de)ITQANAAGGTPSWK	4115.0025	IPI00137792	IPI00137792	yes	yes	3,4		0.0001974	72.163
VYN(de)GSVPFEER	1295.6146	IPI00265291	IPI00265291	yes	no		2	1.5991E-15	185.25
VYMKN(de)VTVVIR	1320.7588	IPI00469218	IPI00469218	yes	yes	2,3		6.1498E-06	162.36
VYIN(de)DSVEISRNENK	1778.8799	IPI00126769	IPI00126769	yes	yes		2	1.5983E-25	188.12
VYIN(de)DSVEISR	1293.6565	IPI00126769	IPI00126769	yes	yes		2	1.6706E-14	179.35
VYIHPFHIIYHN(de)K	1679.8936	IPI00654069	IPI00654069	yes	yes	2,3		2.4614E-06	157.78
VYGQNINN(de)ETWSR	1579.7379	IPI00762609	IPI00762609	yes	no		2	4.2547E-19	183.89
VYGGIVN(de)QSEINEGTAFFR	2100.0276	IPI00416285	IPI00416285	yes	yes	2,3		1.2704E-93	229.96
VWVCDRDNDCVDGSDEPAN(de)CTQMTCGVDEFR	3706.4487	IPI00119063	IPI00119063	yes	yes		3	4.945E-05	87.991
VWPDGVIPFVIGGN(de)FTGSQR	2145.1007	IPI00125182	IPI00125182	yes	no	2,3		0.0016488	91.401
VWN(de)STFIEDYKDFDR	1933.8846	IPI00468674	IPI00468674	yes	no		2	3.1316E-07	162.46
VWN(de)QTEQEPAAYHIISICFVR	2560.2533	IPI00129079	IPI00129079	yes	no		3	0.0002006	100.35
VVTPEEYFN(de)VTIQ	1537.7664	IPI00133103	IPI00133103	yes	no		2	0.015048	88.075
VVSVDISFRPIN(de)ETFPVVYIETPKR	2904.5749	IPI00624663	IPI00624663	yes	yes		3	2.0388E-101	232.49
VVSVDISFRPIN(de)ETFPVVYIETPK	2748.4738	IPI00624663	IPI00624663	yes	yes	2,3,4		1.6566E-24	181.54
VVRPDSEIGERPPEDN(de)QSFQYDHEAFIGK	3358.5854	IPI00137831	IPI00137831	yes	yes		3	0.0006865	82.517
VVN(de)VSEIYGTPCTK	1565.7759	IPI00626950	IPI00626950	yes	no		2	0.0003631	125.67
VVN(de)STTGTGEHIR	1369.695	IPI00131223	IPI00131223	yes	no		2	0.029648	76.728
VVMDIPYEIWN(de)ETSAEVADIKK	2549.2723	IPI00119039	IPI00119039	yes	no	2,3,4		3.7511E-06	135.86
VVMDIPYEIWN(de)ETSAEVADIK	2421.1774	IPI00119039	IPI00119039	yes	no		2	2.3277E-51	208.98
VVIRPFYITN(de)STDMV	1753.9073	IPI00119809	IPI00119809	yes	no		2	0.022734	94.532
VVINFN(de)GTSQEIMAVSEHR	2130.0528	IPI00111183	IPI00111183	yes	yes	2,3		0.0003849	142.26
VVIISGVEPRPPTPQVQFTIN(de)ASSEDHKR	3200.6942	IPI00122973	IPI00122973	yes	no		3	7.4901E-09	104.38
VVIHPN(de)HSVVDIGIIK	1739.0094	IPI00409148	IPI00409148	yes	yes	2,3,4		6.0887E-36	199.13
VVIAGSN(de)MTICCMSPTK	1867.8664	IPI00119299	IPI00119299	yes	no		2	1.8435E-18	178
VVFISPAVPEEPEAYN(de)ITVIIR	2455.3363	IPI00405742	IPI00405742	yes	no	2,3,4		3.2236E-06	113.49
VVDGEREN(de)VSMVDYAHNNYQAQSAVPIR	3160.4996	IPI00134743	IPI00134743	yes	yes		3	0.0004221	83.992
VTVEGMEYVFYN(de)DTK	1793.8182	IPI00405742	IPI00405742	yes	no		2	2.6113E-08	166.9
VTTCHIPQQN(de)ATIYK	1772.888	IPI00410951	IPI00410951	yes	no		2	0.0052644	93.237
VTNSNANAAGPIIVAGYN(de)VSGSVR	2330.1979	IPI00222429	IPI00222429	yes	yes	2,3		3.1985E-69	216.67
VTITFATDESGN(de)HTGWK	1862.8799	IPI00467068	IPI00467068	yes	no		2	1.8667E-12	173.03
VTINNWVAN(de)KTEGR	1600.8322	IPI00136642	IPI00136642	yes	yes	2,3		1.556E-18	183.81
VTINNWVAN(de)K	1157.6193	IPI00136642	IPI00136642	yes	yes		2	0.0012886	155.06
VTIN(de)ESEICAGAEK	1519.7188	IPI00116509	IPI00116509	yes	no		2	0.0012398	130.47
VTIIPN(de)QTHYVVPK	1607.9035	IPI00742414	IPI00742414	yes	no		2	0.011707	89.231
VTIDFN(de)ITNPENGPVIDDAIPNSVHGHIPFAK	3440.7365	IPI00466371	IPI00466371	yes	yes		3	1.08E-19	127.83
VTIDFN(de)ITDPENGPVIDDAIPNSVHGHIPFAK	3441.7205	IPI01026704	IPI01026704	yes	yes	3,4		1.2878E-37	180.9
VSTVTIVSATSTTAN(de)MTMSPEGR	2340.1301	IPI00406603	IPI00406603	yes	yes		2	5.697E-188	266.53
VSTIYANN(de)GSVIQGSTVASVYHKR	2550.319	IPI00121114	IPI00121114	yes	yes	2,3		1.3688E-05	101.33
VSTIYANN(de)GSVIQGSTVASVYHK	2394.2179	IPI00121114	IPI00121114	yes	yes	2,3		2.2612E-55	208.92
VSQVIHEGGHN(de)VTKIIYESANIPDFRK	3050.5938	IPI00463764	IPI00463764	yes	no	3,4		3.3907E-38	186.62
VSQVIHEGGHN(de)VTKIIYESANIPDFR	2922.4988	IPI00463764	IPI00463764	yes	no		3	1.2222E-17	163.91
VSQVIHEGGHN(de)VTK	1503.7794	IPI00463764	IPI00463764	yes	no	2,3		2.7794E-05	147.56
VSNYIGQAN(de)QSAWITVIPK	2088.1004	IPI00828688	IPI00828688	yes	no		2	1.6571E-06	159.8

VSNGN(de)GSIEIPATVPGYVHSAIHQHGIIQDPYYR	3688.8386	IPI00118011	IPI00118011	yes	no	3,4	0.0009971	69.578
VSITN(de)VSISDEGR	1375.6943	IPI00856723	IPI00856723	yes	no	2	4.2165E-122	249.33
VSIQEIPGSEHIEMIAN(de)ATTIAYIKR	2883.5164	IPI00124428	IPI00124428	yes	yes	3	0.01021	59.212
VSIQEIPGSEHIEMIAN(de)ATTIAYIK	2727.4153	IPI00124428	IPI00124428	yes	yes	2,3,4	9.282E-10	149.33
VSGQMHMQN(de)ITFQTEASVAQQEK	2591.2108	IPI00312711	IPI00312711	yes	no	3	8.0759E-06	103.4
VSFYFFVTSPQN(de)VSDVIPR	2201.1157	IPI00153202	IPI00153202	yes	no	3	0.012565	76.85
VSESEKSQIVN(de)ETHWQYYGTSDTR	2843.2998	IPI00749655	IPI00749655	yes	no	3	1.9561E-09	152.13
VRIDPPC(de)TNTTAPSNYINNPYVR	2661.2969	IPI00658539	IPI00658539	yes	no	3	1.5521E-07	113.49
VQPIVAVADEGWYIIQN(de)K	2042.0837	IPI00111163	IPI00111163	yes	no	2,3	4.9492E-62	218.12
VQGGSSVWGSVITHN(de)SSAITYQSWGR	2763.3365	IPI00453977	IPI00453977	yes	no	2,3,4	1.1284E-13	124.51
VPTREENIQVYN(de)VTK	1788.937	IPI00850698	IPI00850698	yes	yes	2	0.014341	100.41
VPNNAIEGIEN(de)ITAIYIHHNEIQEVGSSMR	3347.6568	IPI00120187	IPI00120187	yes	yes	3,4	0.0010378	69.981
VNYEGGTWDWIAEAISSN(de)HTR	2405.1036	IPI00134585	IPI00134585	yes	yes	3	0.0091428	74.793
VNRFN(de)STEYQVVTR	1711.8642	IPI00119063	IPI00119063	yes	no	2,3	9.2685E-47	203.11
VNNVTGN(de)FTFVIR	1479.7834	IPI00395208	IPI00395208	yes	no	2	0.0008865	126.63
VNN(de)VTGNFTFVIR	1479.7834	IPI00395208	IPI00395208	yes	no	2	0.0008865	126.63
VNDSVPFHIGWN(de)STER	1856.8806	IPI00464256	IPI00464256	yes	yes	2,3	0.0065508	94.09
VNCEERNVTGIEN(de)FTIK	2021.984	IPI00378224	IPI00378224	yes	yes	2,3	1.2896E-17	178.51
VNCEERN(de)VTGIENFTIK	2021.984	IPI00378224	IPI00378224	yes	yes	2,3	1.2896E-17	178.51
VNASTTDPN(de)STVEQSAITR	1989.9603	IPI00338785	IPI00338785	yes	no	2	0.0003327	139.11
VN(de)SSIHSQISR	1226.6367	IPI01027808	IPI01027808	yes	no	2	2.067E-51	215.72
VN(de)KTEEDYARDSIFVR	1940.9592	IPI00133103	IPI00133103	yes	no	2	0.0056513	117.08
VN(de)KTEEDYAR	1223.5782	IPI00133103	IPI00133103	yes	no	2	4.6696E-27	201.46
VN(de)ITYNYPVR	1237.6455	IPI00221859	IPI00221859	yes	yes	2	0.0016432	143.24
VN(de)ISFPSAQSIPASDTHIK	2011.0375	IPI00624663	IPI00624663	yes	yes	2,3	9.231E-06	157.47
VN(de)ISAPIIPKEDPIFTYISK	2244.2406	IPI00761772	IPI00761772	yes	no	3	0.0099072	87.088
VN(de)GTKEPIEFK	1260.6714	IPI00345112	IPI00345112	yes	no	2	7.5722E-39	204.17
VN(de)GTITQVIIVGAPTHDDVSK	2163.1535	IPI00225715	IPI00225715	yes	yes	2	5.0835E-06	109.85
VN(de)FTIEASEGCYR	1544.6929	IPI00342158	IPI00342158	yes	no	2	2.9345E-09	162.72
VN(de)DSVPFHIGWNSTER	1856.8806	IPI00464256	IPI00464256	yes	yes	2,3	0.0065508	94.09
VN(de)ASTTDPNSTVEQSAITR	1989.9603	IPI00338785	IPI00338785	yes	no	2	0.0003327	139.11
VMSWWDYGYQITAMAN(de)R	2090.9343	IPI00109108	IPI00109108	yes	yes	2,3	3.8646E-19	184.22
VMSWWDYGYQIAGMAN(de)R	2046.908	IPI00316469	IPI00316469	yes	yes	2,3	4.3635E-05	151.8
VMEIEKEIAN(de)ATTKPEDR	2073.0412	IPI00461861	IPI00461861	yes	yes	3	0.0027194	118.55
VME(de)VNFISYVVMSTAAIPMIKQSNGSIAVISSIAGK	3754.9712	IPI00115599	IPI00115599	yes	no	3,4	0.001093	64.152
VKPTPPYN(de)ISVTNSEEISSIIK	2415.2897	IPI00120155	IPI00120155	yes	yes	3	5.5961E-06	125.23
VKN(de)STCIDDSWIHPK	1798.8672	IPI00122584	IPI00122584	yes	yes	2	0.024205	91.076
VIYIPAYN(de)CTIRPVSK	1893.0182	IPI00469387	IPI00469387	yes	no	2	0.030221	87.148
VIVIITDGEASDKGN(de)ISAAHDITR	2494.3027	IPI00894972	IPI00894972	yes	no	3	0.024149	66.325
VITNQESPYQN(de)HTGR	1742.8336	IPI00125310	IPI00125310	yes	no	2,3	1.7109E-75	225.85
VITMANQVITVN(de)ISEEGR	1973.0252	IPI00987265	IPI00987265	yes	yes	2	2.2856E-93	231.86
VISNN(de)CTSYGVIDIGK	1738.856	IPI00331214	IPI00331214	yes	yes	2	0.0010264	139.29
VISN(de)NCTSYGVIDIGK	1738.856	IPI00331214	IPI00331214	yes	yes	2	0.0010264	139.29
VISIAQAHSIN(de)FSCEQVR	2058.0317	IPI00308990	IPI00308990	yes	yes	2,3	8.0248E-08	160.8
VIPFN(de)VTDYCQIVR	1722.8763	IPI00828222	IPI00828222	yes	no	2	0.0039424	105.46
VIPEPIHAPNVIDTGHNFAIIN(de)ISSEPYFGDGPIK	3770.9308	IPI00221547	IPI00221547	yes	no	3,4	0.0007636	66.378
VINN(de)ITNDIR	1170.6357	IPI00128178	IPI00128178	yes	no	2	7.6867E-94	237.84
VINEECKEN(de)ESINIAAR	1987.9633	IPI00122411	IPI00122411	yes	yes	2,3	0.008208	105.28
VINDTWAWKN(de)ATIAEQAK	2058.0534	IPI00123342	IPI00123342	yes	yes	2,3	1.3733E-26	192.6
VINADQGTSATVQMIIN(de)DTCPIFVR	2762.3731	IPI00320065	IPI00320065	yes	yes	2,3	3.2994E-09	121.62
VIN(de)STGHDVAR	1167.5996	IPI00849146	IPI00849146	yes	no	2	0.0005585	144.1
VIN(de)ITDNTYFK	1326.682	IPI00130117	IPI00130117	yes	yes	2	2.1882E-39	203.61
VIN(de)ISHSIIDISSEQIFDGIPAIQHINIQGNHFPK	3894.0428	IPI00343568	IPI00343568	yes	yes	4	0.0017001	54.732
VIN(de)DTWAWKNATIAEQAK	2058.0534	IPI00123342	IPI00123342	yes	yes	2,3	1.3733E-26	192.6
VIN(de)DTWAWK	1131.5713	IPI00123342	IPI00123342	yes	yes	2	0.014156	118.71
VIIRPYITPNNQGIYIFQGN(de)STVR	2762.4868	IPI00319509	IPI00319509	yes	yes	2,3,4	1.3985E-32	188.24
VIIQSQIPIGTIKHN(de)MTYFCK	2490.3127	IPI00108003	IPI00108003	yes	no	3	0.0002235	106.93
VIINFFVGTDDKN(de)STQHIIHFDQPR	2940.4882	IPI00461861	IPI00461861	yes	no	3	2.6536E-07	109.05
VIIN(de)ITTVAANHGYTK	1713.9414	IPI00467600	IPI00467600	yes	no	2	1.1759E-35	195.6
VIIIIDPAISGN(de)ETEPYPAFTR	2415.2686	IPI00848693	IPI00848693	yes	yes	2,3	2.8227E-65	217.17
VIHIQFNSISSITDDTFCKAN(de)DTR	2781.3392	IPI00120848	IPI00120848	yes	yes	3	1.5724E-08	128.41
VIFYKDDAMVYN(de)VTSR	1919.9451	IPI00406901	IPI00406901	yes	no	2,3	6.2346E-08	167.89
VIETIPANYSIN(de)SSKK	1762.9465	IPI00480532	IPI00480532	yes	no	2	0.017676	82.102
VIETIPAN(de)YSINSSKK	1762.9465	IPI00480532	IPI00480532	yes	no	2	0.017676	82.102
VIENEKFDTHEYHN(de)ESR	2145.9716	IPI00463492	IPI00463492	yes	yes	2,3	8.6859E-92	232.15
VIEHYN(de)YTKETIDMQETTSR	2457.1482	IPI00128628	IPI00128628	yes	yes	3	0.0001258	140.89
VIEEPKN(de)VSCETR	1559.7614	IPI00118291	IPI00118291	yes	no	2	0.010531	110.51
VIDIWDIAQSAN(de)FTEKEIESFREEIK	3109.5608	IPI00469307	IPI00469307	yes	no	3	0.0007302	84.946
VIDIWDIAQSAN(de)FTEK	1848.9258	IPI00469307	IPI00469307	yes	no	2,3	3.0135E-08	166.77
VIAQHQNIIFAN(de)SSSSMR	2002.0054	IPI00319040	IPI00319040	yes	no	2,3	0.0031435	99.044
VHSHGNN(de)YTIICQVK	1768.8679	IPI00118291	IPI00118291	yes	no	2	0.0012576	118.52
VHSGN(de)FSTIPQYFK	1623.8045	IPI01023131	IPI01023131	yes	no	2	0.0010139	119.96
VHITVCN(de)ITSR	1298.6765	IPI00128862	IPI00128862	yes	no	2	0.008318	103.44
VHGPN(de)ASHYTSIMTMITWER	2330.0936	IPI00169617	IPI00169617	yes	yes	3	4.8298E-05	107.57
VGYQSQN(de)ISCFFR	1604.7406	IPI00464256	IPI00464256	yes	yes	2	3.5326E-09	161.48
VGVNKN(de)QTVTATFGYPFR	1998.0323	IPI00114256	IPI00114256	yes	no	2,3	7.4296E-62	219.23
VGDEYQEIQIDGFDN(de)ESSNKTWMK	2832.2549	IPI00114671	IPI00114671	yes	no	3	1.5283E-07	111.49
VGCSEYTN(de)R	1084.4608	IPI00377642	IPI00377642	yes	yes	2	0.0084774	125.97
VGATAAVYSAAIIEYITAEVIEIAGN(de)ASK	2894.5277	IPI00555055	IPI00555055	yes	no	2,3	2.4986E-85	165.62
VFVYTPTTN(de)YTIR	1573.814	IPI00124428	IPI00124428	yes	yes	2	1.186E-85	228.78
VFPYISVMVNN(de)GSISYDHSKDGR	2584.238	IPI00321634	IPI00321634	yes	yes	2,3	4.6757E-08	142.88
VFPYISVMVNN(de)GSISYDHSK	2256.0885	IPI00321634	IPI00321634	yes	yes	2,3	9.2159E-10	166.47
VFPYISAMVNN(de)GSISYDHER	2298.0739	IPI00830356	IPI00830356	yes	no	2	1.1099E-05	132.59
VFNGKDN(de)ISK	1120.5877	IPI00331214	IPI00331214	yes	yes	2	0.000242	165.63
VFKTN(de)STQVSDVR	1479.7682	IPI00469426	IPI00469426	yes	no	2	0.018424	104.55
VFKPQSGADAIN(de)DSQDFPFPETPAK	2705.2973	IPI00462465	IPI00462465	yes	no	3	1.4202E-05	101.75

VFIVPVGN(de)HSNIPFSR	1781.9577	IPI00221418	IPI00221418	yes	no	2,3	0.0013747	115.82
VFHIHN(de)ESWVIITPK	1818.9781	IPI00224752	IPI00224752	yes	yes	2	1.1708E-91	229.97
VFGSQN(de)ITTVK	1192.6452	IPI00123342	IPI00123342	yes	yes	2	5.6639E-40	209.19
VFDIHQNMGSVN(de)VSVGCTPAQIIETSR	2958.4328	IPI00116913	IPI00116913	yes	no	3	0.0006604	79.36
VETGVIKPGMVVTFAPVN(de)VTTEVK	2514.3767	IPI00307837	IPI00307837	yes	yes	2,3	3.3233E-05	103.02
VDVIVAN(de)ITVTDKDQPHTPAWNAAYR	2893.4723	IPI00323134	IPI00323134	yes	no	3	1.2734E-23	176.67
VDIPQQPMGIIAVAN(de)DTNSCEISPCR	2884.3517	IPI00119063	IPI00119063	yes	yes	3	6.8677E-11	113.31
VDIEDFEN(de)NTAYAK	1627.7366	IPI00551169	IPI00551169	yes	no	2	4.3461E-253	296.22
VDFIWHPEVN(de)GSMK	1657.7923	IPI00380296	IPI00380296	yes	yes	2	0.0004504	125.61
VDDEMPQHAVISGPNIFINNINKTDN(de)GTYR	3371.6205	IPI00856723	IPI00856723	yes	no	3,4	3.9371E-19	148.89
VDDEMPQHAVISGPNIFINNIN(de)KTDNGTYR	3371.6205	IPI00856723	IPI00856723	yes	no	3,4	3.9371E-19	148.89
VAGIIVIN(de)YSNDYNHWIATK	2290.1746	IPI00111908	IPI00111908	yes	no	2,3	3.1173E-38	195.77
VAEVENGTKPDSDVPEHCIDTWSFDAATMDHN(de)GTMIFFK	4410.9508	IPI00128484	IPI00128484	yes	yes	4	0.0003625	69.7
VAEVEN(de)GTKPDSDVPEHCIDTWSFDAATMDHNGTMIFFK	4410.9508	IPI00128484	IPI00128484	yes	yes	4	0.0003625	69.7
TV(de)VTEAGNIIKDNATQEEIIHYIEK	2827.4604	IPI00928320	IPI00928320	yes	no	3	6.8221E-10	153.65
TTIVDNNTWN(de)NSHIAIVGK	2096.0651	IPI00316469	IPI00316469	yes	yes	2	4.2201E-08	160.45
TQITCSIN(de)SSGVDIVGHR	1942.9531	IPI00408495	IPI00408495	yes	no	2,3	0.0015096	102.57
TITIIN(de)VTR	1029.6182	IPI00108535	IPI00108535	yes	no	2	2.248E-11	177.78
TIIVDNNTWN(de)NTHISR	1896.9442	IPI00109108	IPI00109108	yes	yes	2	9.5323E-92	228.9
TIIVDN(de)NTWNNTHISR	1896.9442	IPI00109108	IPI00109108	yes	yes	2	9.5323E-92	228.9
TIIGYYN(de)QSAGGTHTIQWMYGCDVGSDGR	3206.4186	IPI00109996	IPI00109996	yes	yes	3	2.7361E-07	94.73
TIGISPFHEFADVVFTAN(de)DSGHR	2516.2084	IPI00127560	IPI00127560	yes	yes	3	0.0001185	93.269
TGRVYN(de)VTQHAMGIIVNKQVK	2355.2845	IPI00468196	IPI00468196	yes	no	3	0.0026446	97.095
TEIDIRPQGIAIFSN(de)VSEAR	2215.1597	IPI00122973	IPI00122973	yes	no	3	0.0009651	101.67
TAASIIWQAYPIIN(de)ISEK	2017.0884	IPI00115867	IPI00115867	yes	yes	3	0.0003959	147.07
STIIIHHPQYAWIQDIGIREDNEGVYNG(de)SWGGR	3780.8397	IPI00230084	IPI00230084	yes	no	4	0.0095609	65.275
SN(de)VTRPSEFNYIWIAPIPFIK	2491.3264	IPI00319509	IPI00319509	yes	no	3	0.030479	75.97
SIKQNIN(de)ISAPIMSR	1670.9138	IPI00123881	IPI00123881	yes	no	3	0.016471	97.551
SIIDHIHVGVGRDN(de)VSQPK	2070.097	IPI00123428	IPI00123428	yes	no	2	0.0003152	126
RYIKNG(de)NATIIR	1417.8154	IPI00985828	IPI00985828	yes	no	2,3	0.019075	111.52
RYIK(de)NGNATIIR	1417.8154	IPI00985828	IPI00985828	yes	no	2,3	0.019075	111.52
RYIEIG(de)NETIIR	1475.8096	IPI00850057	IPI00850057	yes	no	2,3	4.2192E-52	212.9
RVNDN(de)KTAAEEAIR	1585.8172	IPI01027808	IPI01027808	yes	no	2	1.6943E-05	158.52
RVM(de)EVNFISYVVMSTAAIPMIKQSNGSIAVISSIAGK	3911.0723	IPI00115599	IPI00115599	yes	no	4	0.0003825	63.412
RVIYIPAY(de)NCTIRPVSK	2049.1194	IPI00469387	IPI00469387	yes	no	2,3	2.4355E-07	164.53
RSPGAQDN(de)VSVSQGMR	1687.806	IPI00348586	IPI00348586	yes	no	3	0.0022957	117.08
RSN(de)FTPATNEAPQATVFPK	2075.0436	IPI00172039	IPI00172039	yes	no	3	0.0003489	119.46
RQDPVSWN(de)KTFEDISR	1976.9704	IPI00848693	IPI00848693	yes	yes	3	0.007841	110.04
RPFGVVYEMEVDTIETTCHAIDPTPIAN(de)CSVR	3676.7324	IPI00128249	IPI00128249	yes	yes	3,4	1.7446E-08	96.505
RNPSANTFIHIN(de)ASSFR	1930.9762	IPI00315535	IPI00315535	yes	yes	2,3	0.0003803	143.67
RN(de)WTINRINGDFAQINIK	2172.1552	IPI00323134	IPI00323134	yes	no	3	0.0034071	140.74
RN(de)WTETEVR	1189.584	IPI00134390	IPI00134390	yes	yes	2	0.015127	123.63
RN(de)FTAADWGHSR	1416.6647	IPI00339885	IPI00339885	yes	no	2,3	0.0070091	106.58
RMHIN(de)GSNVQVIHR	1659.874	IPI00119063	IPI00119063	yes	yes	2,3	4.0391E-47	203.87
RIYAGMVSIMDEAVGN(de)VTK	2053.0336	IPI00652358	IPI00652358	yes	no	2	0.044985	50.883
RIPVTN(de)ISQIHK	1404.8201	IPI00469426	IPI00469426	yes	no	3	0.013595	97.463
RIGAI(de)NNSIIIIEDR	1695.9632	IPI00115516	IPI00115516	yes	no	2,3	0.0010219	140.27
RIAVDWESIGYN(de)ITR	1791.9268	IPI00123040	IPI00123040	yes	no	3	0.0009567	130.2
RHEEGHMIN(de)CTCFGQGR	2087.8836	IPI00113539	IPI00113539	yes	no	2,3	0.0010876	121.13
RGVFITN(de)ETGQPIIGK	1728.9523	IPI00111960	IPI00111960	yes	no	2,3	5.9231E-60	212.11
RGTFTDCAIAN(de)MTQQIR	1981.9462	IPI00621027	IPI00621027	yes	no	3	0.0013412	140.96
RGPECSQN(de)YTAPTGVIK	1876.9101	IPI00123996	IPI00123996	yes	yes	2,3	6.4961E-05	153.94
RGDDIYTN(de)VTVSIVEAIVGFEMDITHIDGHK	3443.7031	IPI00320241	IPI00320241	yes	yes	4	3.1265E-19	118.85
RGCKDN(de)ATDSVPIR	1587.7787	IPI00119063	IPI00119063	yes	yes	2	0.0093796	135.02
RFHSDIN(de)ISESIIPAVIEK	2167.1637	IPI00831484	IPI00831484	yes	yes	2,3	0.0004037	116.96
RFANEYPN(de)ITR	1379.6946	IPI00130573	IPI00130573	yes	yes	2	0.020613	93.011
RDQGN(de)VTDMASMK	1451.6497	IPI00471081	IPI00471081	yes	yes	3	0.0015438	129.37
RDDYRPTWTI(de)NQTEPVAGNYYPVNTR	3125.4955	IPI00381303	IPI00381303	yes	yes	3	1.5893E-12	134.81
RDDIHPTIPAGQYFIN(de)ITYNYPVHSFDGR	3405.6531	IPI00387318	IPI00387318	yes	no	3,4	5.8108E-76	214.16
ratio	1. 04%							
RAN(de)ASTFAVPSPVSNSADTR	2047.0083	IPI00119065	IPI00119065	yes	yes	3	0.0031248	89.344
RADIN(de)GSNMETVIGHGIK	1910.9632	IPI00411145	IPI00411145	yes	no	3	0.02227	79.837
QVVEN(de)MTR	975.48076	IPI00111960	IPI00111960	yes	no	2	0.029357	121.09
QVTPIFFYFQN(de)R	1558.7932	IPI00319509	IPI00319509	yes	no	2,3	0.0070583	136.48
QVN(de)GSVSGSQWNK	1389.6637	IPI00408850	IPI00408850	yes	yes	2	0.026662	79.089
QVEIIEYPYHEQIAVVAPEPIITGHN(de)YTIK	3463.8028	IPI00223987	IPI00223987	yes	yes	3,4	1.6843E-19	121.58
QVEEIIVN(de)HTGIR	1506.8154	IPI00263041	IPI00263041	yes	yes	2	0.019808	84.468
QVAIQTFGN(de)QTSIIPAGGAGYK	2220.1539	IPI00228719	IPI00228719	yes	yes	2,3	0	312.9
QTTAMDFSYAN(de)ETVCWVHVGDSAAQTQIK	3257.4758	IPI00119063	IPI00119063	yes	no	3,4	0.0056716	56.298
QTQVGIVQYGAN(de)VTHEFNINK	2359.1921	IPI00466371	IPI01026704	no	no	2,3	7.5699E-10	162.09
QSQPVHIIPMNETDHINMVFSN(de)K	2678.2945	IPI00133500	IPI00133500	yes	yes	3	0.0062407	73.783
QSQPVHIIPMN(de)ETDHINMVFSNK	2678.2945	IPI00133500	IPI00133500	yes	yes	3	0.0062407	73.783
QSN(de)GSIAVISSIAGK	1430.7729	IPI00115599	IPI00115599	yes	no	2	7.1086E-56	207.79
QRYN(de)ITAK	992.54033	IPI00130661	IPI00130661	yes	yes	2	8.8701E-07	189.7
QQGGQVTIVIIQEAYKQYN(de)ATINR	2734.4402	IPI00757554	IPI00757554	yes	yes	2	0.0044561	76.817
QQCIEEAQIEN(de)ETTGCSK	2123.9099	IPI00128984	IPI00128984	yes	yes	2	1.11E-18	179.1
QPIYIN(de)CSCVTGGSASAK	1911.8819	IPI00309249	IPI00309249	yes	yes	2	0.000511	118.48
QNFSN(de)ITVSTEDQVK	1708.8268	IPI00129677	IPI00129677	yes	no	2	0	308
QN(de)FSNITVSTEDQVK	1708.8268	IPI00129677	IPI00129677	yes	no	2	0	308
QMWKSPN(de)GTIRNIIGGTVFR	2274.2055	IPI00135231	IPI00135231	yes	no	3	0.029852	71.263
QMAIN(de)ATYIFNGITVSIPGMEK	2397.2072	IPI00316359	IPI00316359	yes	no	3	0.015757	59.585
QKNVNISYI(de)VNDSFFPQRPEK	2522.2918	IPI00122557	IPI00122557	yes	no	2,3	1.0333E-05	158.58
QKN(de)VNISYIVNDSFFPQRPEK	2522.2918	IPI00122557	IPI00122557	yes	no	2,3	1.0333E-05	158.58
QIN(de)ITTEDDDIYHMTVPYGRPR	2633.2544	IPI00265291	IPI00265291	yes	yes	3	1.9708E-05	117.2
QIN(de)ISIIVK	1026.6437	IPI00115116	IPI00115116	yes	yes	2	0.019594	113.62
QIMN(de)TSFNPIRIVNTYGAFGSVTK	2657.3636	IPI00405437	IPI00405437	yes	no	3	0.017877	65.855

QIITEEKIPN(de)NTQWITWSPEGHK	2748.3871	IPI00125813	IPI00125813	yes	no	2,3	6.4227E-42	199.99
QIIQTQVASPAIHPPVSYN(de)DTAPR	2602.3503	IPI00128989	IPI00128989	yes	no	3	0.0082142	64.203
QIINAIQIN(de)NTAVGHAIVIPARR	2481.4292	IPI00830749	IPI00830749	yes	yes	3	8.0196E-08	111.33
QIINAIQIN(de)NTAVGHAIVIPAR	2325.3281	IPI00830749	IPI00830749	yes	yes	3	0.0001565	99.021
QIIAN(de)SSAIEETIIGHQGR	2036.0651	IPI00116913	IPI00116913	yes	no	2,3	0.0023993	147.16
QIGASPSDDIIFGVFAQSKPDSAEPVN(de)R	2944.4567	IPI00130420	IPI00130420	yes	no	3	0.0034386	75.054
QIFFN(de)GTETIR	1324.6776	IPI00338209	IPI00338209	yes	no	2	0.017728	99.752
QHGQFSIAVVGIN(de)ITSIGIR	2109.1695	IPI00121190	IPI00121190	yes	no	3	1.9284E-05	113.33
QGPQAGGTTITIN(de)GTHIDTGSKEDVR	2652.3103	IPI00405742	IPI00405742	yes	no	3	0.0005357	85.932
QGPQAGGTTITIN(de)GTHIDTGSK	2153.0713	IPI00405742	IPI00405742	yes	no	2	0.0052563	92.932
QGDQYSCMVGHEAIPMN(de)FTQK	2440.061	IPI00473880	IPI00473880	yes	no	2,3	6.0259E-06	120.75
QFWIFDVQNPDDVAKN(de)SSK	2237.0753	IPI00331214	IPI00331214	yes	yes	2,3	1.3492E-14	139.67
QFNCSFEN(de)ITR	1414.6299	IPI00674255	IPI00674255	yes	yes	2	0.0034392	122.33
QFN(de)CSFENITR	1414.6299	IPI00674255	IPI00674255	yes	yes	2	0.0034392	122.33
QENNIIIINAEHGNSSIFIEN(de)STFESFGYHSVSPDR	4064.9141	IPI00125813	IPI00125813	yes	no	3,4	0.0073715	49.504
QEMNGTYVCHAFSSHGN(de)VTR	2293.9957	IPI00122973	IPI00122973	yes	no	3	0.022373	58.712
QEIN(de)DSIQVAER	1400.6896	IPI00320420	IPI00320420	yes	yes	2	1.4657E-29	199.8
QDIAISGN(de)ISSIYAMTQDK	2053.999	IPI00405742	IPI00405742	yes	no	2	4.797E-113	244.52
QAIQTMQSEFFYITTNIIN(de)DTIEIR	2988.4903	IPI00118130	IPI00118130	yes	yes	3	9.6048E-24	127.8
PINETFPVVYIE(de)TPKR	1902.0251	IPI00624663	IPI00624663	yes	yes	2,3	1.4076E-35	196.95
PINETFPVVYIE(de)TPK	1745.924	IPI00624663	IPI00624663	yes	yes	2	0.015225	82.349
PIN(de)ITDESMFPIGTYIIYECIPGYIKR	3202.6083	IPI00138061	IPI00138061	yes	no	3	1.2969E-13	124.66
PGAASN(de)ISFQAPFR	1461.7365	IPI00311159	IPI00311159	yes	yes	2	0.0029933	108.71
PFFIIQN(de)SSMMK	1441.7098	IPI00226229	IPI00226229	yes	no	2	0.0040663	101.39
NYTAN(de)ATSSREEAWDYVQAQVK	2530.1724	IPI00108098	IPI00108098	yes	no	2,3	5.2182E-38	155.94
NYN(de)FTIACNTK	1344.6132	IPI00322575	IPI00322575	yes	no	2	4.5977E-10	174.9
NYKNPN(de)ITISFTAER	1766.8951	IPI00132600	IPI00132600	yes	no	2,3	0.0027776	122.63
NYFHYN(de)QSFPPSYNIK	2017.9323	IPI00129265	IPI00129265	yes	yes	2,3	1.1708E-12	175.26
NYEVQIFHVN(de)ATVTEEGTGIEFSR	2739.314	IPI00123223	IPI00123223	yes	yes	3	1.2783E-16	125.92
NVNISYTVN(de)DSFFPQRPQKIIANK	2792.461	IPI00381178	IPI00381178	yes	yes	3	9.7478E-09	127.17
NVNISYTVN(de)DSFFPQRPQK	2253.1178	IPI00381178	IPI00381178	yes	yes	2,3	0	331.34
NVNISYTVN(de)DSFFPQR	1899.9115	IPI00381178	IPI00381178	yes	yes	2	0.0054037	91.355
NVNISYIVN(de)DSFFPQRPEK	2266.1382	IPI00122557	IPI00122557	yes	no	2,3	3.4602E-93	231.86
NVNI(de)SYTVNDSFFPQRPQKIIANK	2792.461	IPI00381178	IPI00381178	yes	yes	3	9.7478E-09	127.17
NVNI(de)SYTVNDSFFPQRPQK	2253.1178	IPI00381178	IPI00381178	yes	yes	2,3	0	331.34
NVNI(de)SYTVNDSFFPQR	1899.9115	IPI00381178	IPI00381178	yes	yes	2	0.0054037	91.355
NVN(de)ISYIVNDSFFPQRPEK	2266.1382	IPI00122557	IPI00122557	yes	no	2,3	3.4602E-93	231.86
NVAIVAGDTGN(de)ATGIGEQGPTR	2097.0451	IPI00122272	IPI00122272	yes	no	2	0	320.86
NTQADVINASWSVISN(de)STRHEIER	2726.3372	IPI00132189	IPI00132189	yes	yes	3	1.3873E-08	130.11
NTQADVINASWSVISN(de)STR	2062.0079	IPI00132189	IPI00132189	yes	yes	2	1.3437E-260	289.76
NTQADVIN(de)ASWSVISNSTRHEIER	2726.3372	IPI00132189	IPI00132189	yes	yes	3	1.3873E-08	130.11
NTQADVIN(de)ASWSVISNSTR	2062.0079	IPI00132189	IPI00132189	yes	yes	2	1.3437E-260	289.76
NTGEIN(de)ITSIIDREETPYFIITGYAIDSR	3300.6514	IPI00115793	IPI00115793	yes	no	3	2.7147E-11	109.68
N-te)rminal Se)r/Thr	16							
NTDGTYNYTSIFIVN(de)SSAHR	2259.0556	IPI00129158	IPI00129158	no	no	3	0.011	79.514
NTDGTYN(de)YTSIFIVNSSAHR	2259.0556	IPI00129158	IPI00129158	no	no	3	0.011	79.514
NSS(de)IGGVINKYDVVIR	1732.9472	IPI00314673	IPI00314673	yes	yes	2	0.0001232	154.88
NSQFDMN(de)STDIAIK	1582.7297	IPI00346062	IPI00346062	yes	yes	2	1.41E-18	177.38
NSNSN(de)VIQVDQSGIGIPSRDYYINKTENEK	3381.6437	IPI00396840	IPI00396840	yes	no	3	2.3468E-07	94.955
NQIVEI(de)EKVVIHPNHSVVDIGIIK	2692.5276	IPI00409148	IPI00409148	yes	yes	3	9.2365E-51	148.01
NPSANTFIHINA(de)SSFR	1774.8751	IPI00315535	IPI00315535	yes	yes	2,3	4.0686E-12	170.09
NPNN(de)NTIHPNIR	1402.7066	IPI00319509	IPI00319509	yes	no	2	5.716E-11	173.72
NPEDVQSPN(de)GSVYTWR	1847.8438	IPI00112614	IPI00112614	yes	yes	2	1.233E-05	158.29
NPCTSEQN(de)CTSPFSYK	1918.7826	IPI00132474	IPI00132474	yes	yes	2	5.5871E-111	244.05
NPCNITREDY(de)APIVK	1788.8829	IPI00307966	IPI00307966	yes	yes	2,3	0.010105	107.46
NNIHFPGHN(de)YTTR	1569.7437	IPI00130271	IPI00130271	yes	no	2	0.0008836	136.27
NNDSTIN(de)NSATTIIIGIQDCR	2319.1125	IPI00881745	IPI00881745	yes	no	2	6.1938E-06	130.83
NN(de)QTECFNHVR	1417.6157	IPI00124666	IPI00124666	yes	no	2	0.0013743	129.34
NN(de)ISREISEIK	1301.6939	IPI00947760	IPI00947760	yes	no	2	0.027347	113.69
NN(de)DSTINNSATTIIIGIQDCR	2319.1125	IPI00881745	IPI00881745	yes	no	2	6.1938E-06	130.83
NMICQWDPGRETYIETN(de)YTIK	2631.2098	IPI00120155	IPI00120155	yes	yes	3	2.8746E-05	122.44
NKANIQFGEN(de)GTTISAVTNK	2106.0705	IPI00127447	IPI00127447	yes	yes	2,3	1.3227E-50	211.06
NITVIEPVTQPFIQVTN(de)TTVK	2341.2893	IPI00108535	IPI00108535	yes	no	2,3	1.1593E-06	153.36
NITAFN(de)ETIFR	1324.6776	IPI00124700	IPI00124700	yes	yes	2	2.6847E-97	237.53
NISTCFSSGDIFAAHN(de)ISER	2225.0171	IPI00123428	IPI00123428	yes	no	2	1.0825E-05	127.58
NISFACNPGFFIN(de)GTSSSK	2046.9469	IPI00322463	IPI00322463	yes	no	2	1.8528E-93	230.06
NISFACNPG(de)FFINGTSSSK	2046.9469	IPI00322463	IPI00322463	yes	no	2	1.8528E-93	230.06
NIQAVNEIIATISQCN(de)DTSSAAMVQCIR	3106.4846	IPI00138342	IPI00138342	yes	no	3	2.9238E-05	90.1
NIPWYVIAGNHDHIGN(de)VSAQIAYSK	2766.3878	IPI00137491	IPI00137491	yes	yes	2,3	8.4642E-19	173.18
NIN(de)VTSIGFR	1119.6037	IPI00468814	IPI00468814	yes	yes	2	0.0024221	134.3
NIN(de)SSCRPHPGAWIR	1763.8638	IPI00222809	IPI00222809	yes	no	2,3	0.004996	117.86
NIMTIVHFYN(de)K	1378.7067	IPI00119822	IPI00119822	yes	no	2	0.0061771	113.22
NIMIDIQKDTAV(de)EGEEIEVNCTAMASKPATTIR	3647.7845	IPI00856723	IPI00856723	yes	no	3,4	0.0025413	69.47
NIKRPYIVPIIWIN(de)ETGTIGDEK	2668.4588	IPI00331214	IPI00331214	yes	yes	3	4.0328E-08	147.53
NIINDYVSN(de)QTQGMIKEIISEIDER	2921.4441	IPI00131830	IPI00131830	yes	no	3,4	0.0015781	85.518
NIINDYVSN(de)QTQGMIK	1836.904	IPI00131830	IPI00131830	yes	no	2,3	0	263.99
NIGGIETEDDYGYQGHVQTCN(de)FSAQMAK	3132.3553	IPI00126769	IPI00126769	yes	yes	3	1.7914E-13	136.56
NIFIN(de)HSETASAK	1430.7154	IPI00409148	IPI00409148	yes	yes	2	5.9772E-244	289.38
NI(de)TSPVGVQPIINEHTFCAGITK	2495.2842	IPI00409148	IPI00409148	yes	yes	2	1.2345E-24	178.32
NHNGQGYKDQDPASFGNNSIIIN(de)SSR	2832.3175	IPI00848693	IPI00848693	yes	yes	3	1.0136E-11	138.82
NHAVSFDPIFSAVKN(de)FSEAASDFHR	2792.3307	IPI00113042	IPI00113042	yes	no	3	6.1592E-09	124.08
NGTAHGN(de)STHPMHSR	1602.707	IPI00128484	IPI00128484	yes	yes	2	0.030639	71.354
NGNATI(de)IR	857.47191	IPI00985828	IPI00985828	no	no	2	0.010982	137.14
NGNATI(de)IR	857.47191	IPI00985828	IPI00985828	no	no	2	0.010982	137.14
NGNA(de)TIIR	857.47191	IPI00985828	IPI00985828	no	no	2	0.010982	137.14
NGNA(de)TIIR	857.47191	IPI00985828	IPI00985828	no	no	2	0.010982	137.14

NGIASGSGN(de)CSTGPNGDGSFHAWSIIEVK	2918.3253	IPI00322497	IPI00322497	yes	yes		3	3.9877E-15	154.7
NGHITN(de)YTVVYR	1435.7208	IPI00110264	IPI00110264	yes	yes		2	1.3795E-74	195.77
NGDAYGYYN(de)DSIK	1478.6314	IPI00471081	IPI00471081	yes	yes		2	0.0073169	104.55
NFN(de)DSIHSISQAIR	1600.7958	IPI00263041	IPI00263041	yes	yes		2	0.0036613	106.42
NF(de)TAADWGHSR	1260.5636	IPI00339885	IPI00339885	yes	no		2	2.3463E-07	167.71
NF(de)NVEKINGEWHTIIIASDKR	2483.2921	IPI00968968	IPI01008326	no	no		3	7.5417E-51	209.23
NEDRPIAPCGAIANSMFNDTIEIYIVAN(de)ESDPKPIPIPIK	4422.224	IPI00387318	IPI00387318	yes	no		4	5.6416E-17	104.9
NEDRPIAPCGAIANSMFN(de)DTIEIYIVANESDPKPIPIPIK	4422.224	IPI00387318	IPI00387318	yes	no		4	5.6416E-17	104.9
NAVNCTYKNED(de)DCVVR	1955.8466	IPI00266264	IPI00266264	yes	yes		2	1.2295E-11	171.46
NATIAEQAKI(de)PATEKPVIISK	2221.2682	IPI00123342	IPI00123342	yes	yes		2	3.2792E-05	136.46
NAKGDEKEN(de)ITAEAIDISIK	2158.1117	IPI00124725	IPI00124725	yes	no	2,3		9.9907E-50	208.98
NAIQAFGN(de)GTDVNMSPK	1762.8308	IPI00116355	IPI00116355	yes	no		2	0.0008695	139.08
NAINITFHAQN(de)IGEGGAYEAEIR	2487.2142	IPI00115976	IPI00115976	yes	yes	2,3		7.1084E-164	260.53
N(de)YTDCTSEGR	1201.467	IPI00113539	IPI00113539	yes	no		2	0.012817	110.84
N(de)YTANATSSREEAWDYVQAQVK	2530.1724	IPI00108098	IPI00108098	yes	no	2,3		5.2182E-38	155.94
N(de)YSIFIADINQER	1581.7787	IPI00331318	IPI00331318	yes	no		2	3.5051E-143	259.68
N(de)VTYGTYIDDPDPDDGFNYK	2307.9808	IPI00399958	IPI00399958	yes	no		2	1.8101E-114	243.97
N(de)VTIECPFKR	1262.6441	IPI00310059	IPI00310059	yes	no		2	0.0025219	142.43
N(de)VSCIWCNENK	1422.602	IPI00377642	IPI00377642	yes	no		2	0.0056913	110.8
N(de)TTSYPPMCSQDAVGGQVISEIFTNRK	2999.4117	IPI00387289	IPI00387289	yes	yes		3	0.0075054	59.429
N(de)TTSYPPMCSQDAVGGQVISEIFTNR	2871.3167	IPI00387289	IPI00387289	yes	yes		3	0.0017403	70.316
N(de)TTSAAMVHCIR	1359.6387	IPI00131216	IPI00131216	yes	yes		2	0.022139	85.845
N(de)TTPDEIISAVITAVIQDVR	2154.1532	IPI00121833	IPI00121833	yes	no		3	1.9378E-47	166.52
N(de)TTCQDIQIEVK	1447.6977	IPI00131091	IPI00131091	yes	no		2	0.0014169	139.46
N(de)STKEEIIAAIEK	1444.7773	IPI00928320	IPI00928320	yes	no		2	9.0222E-13	171.26
N(de)STIQAANIAGIK	1299.7147	IPI00222937	IPI00222937	yes	no		2	4.8254E-45	210.38
N(de)RTDVEYEIDEK	1509.6947	IPI00134743	IPI00134743	yes	yes		2	0.0051648	124.42
N(de)QTIEIHVIYGPR	1538.8205	IPI00122973	IPI00122973	yes	no		2	1.4301E-44	208.27
N(de)QSVGDPNVDIIR	1425.7212	IPI00127447	IPI00127447	yes	yes		2	3.6498E-70	221.86
N(de)MTIFSDIVAEK	1366.6803	IPI00621027	IPI00621027	yes	no		1	0.017961	108.47
N(de)ITVIEPVTQPFIQVTNTTVK	2341.2893	IPI00108535	IPI00108535	yes	no	2,3		1.1593E-06	153.36
N(de)ITMFISR	980.51134	IPI00225715	IPI00225715	yes	yes		2	0.020632	129.38
N(de)ITFQGPIPK	1113.6182	IPI00111794	IPI00111794	yes	no		2	0.0019991	139.43
N(de)ISVVVATHSPTIAK	1535.8671	IPI00221418	IPI00221418	yes	no		2	0.0005383	127.57
N(de)ISTCFSSGDIFAAHNISER	2225.0171	IPI00123428	IPI00123428	yes	no		2	1.0825E-05	127.58
N(de)ISGVVIADHSGSFHNR	1808.8918	IPI00118674	IPI00118674	yes	yes	2,3		0.0009579	113.33
N(de)HSIPIETK	1037.5506	IPI00129041	IPI00129041	yes	no		2	0.025728	101.38
N(de)GTAHGNSTHPMHSR	1602.707	IPI00128484	IPI00128484	yes	yes		2	0.030639	71.354
N(de)FSGYYIGVGR	1231.5986	IPI00458077	IPI00458077	yes	yes		2	0.0060906	109.24
N(de)ETHSICSACDESCK	1796.6764	IPI00111286	IPI00111286	yes	yes		2	0.0042083	98.582
N(de)CSTQHFPR	1145.5036	IPI00322304	IPI00322304	yes	yes		2	0.029084	98.044
N(de)ATYGHYEPGEEFHDVEDAETYKK	2828.2202	IPI00221798	IPI00221798	yes	no		3	9.9445E-09	147.35
N(de)ATVVWMKDNIR	1445.7449	IPI00121378	IPI00121378	yes	no		2	0.0159	109.44
N(de)ATVVWMK	947.48987	IPI00121378	IPI00121378	yes	no		2	0.0056545	147.19
N(de)ATTYPPMCSQDAAR	1681.7188	IPI00128399	IPI00128399	yes	yes		2	0.0008967	141.34
N(de)ATSYPPMCSQDAGWAK	1882.7978	IPI00138342	IPI00138342	yes	yes		2	0.0003233	141.86
N(de)ATSYPPMCFQDPVTGQIVNDIITNRK	3078.4903	IPI00131216	IPI00131216	yes	yes		3	0.0066506	61.616
N(de)ATIVNEADKIR	1342.7205	IPI00319509	IPI00319509	yes	no		2	0.0050047	133.15
N(de)ASSEYSGTYSCTVQNR	1922.8065	IPI00270376	IPI00270376	yes	no		2	0.013173	80.545
MYVTN(de)DTEVAENNYEAIKDFFR	2668.2115	IPI00658539	IPI00658539	yes	no	2,3		1.7084E-09	160.67
MYVTN(de)DTEVAENNYEAIK	2102.9467	IPI00658539	IPI00658539	yes	no		2	0.002322	100.45
MYSEGSDIVPQSN(de)ETAIHYFKK	2543.2002	IPI00131143	IPI00131143	yes	no	2,3		4.4657E-10	163.41
MYSEGSDIVPQSN(de)ETAIHYFK	2415.1053	IPI00131143	IPI00131143	yes	no	2,3		4.9294E-06	149.95
MVN(de)TTFICTATNAVGTGR	1912.9135	IPI00109375	IPI00109375	yes	no	2,3		1.366E-204	278.07
MVN(de)HSIHPTEPVK	1487.7555	IPI00750217	IPI00750217	yes	no		2	0.0026341	136.3
MTISQN(de)NSIIR	1275.6605	IPI00108535	IPI00108535	no	no		2	1.9515E-199	227.37
MSVINFECN(de)K	1240.558	IPI00308971	IPI00308971	yes	yes		2	0.029398	91.937
MSTIIIHHPQYAWIQDIGIREDNEGVYN(de)GSWGGR	3911.8802	IPI00230084	IPI00230084	yes	no		4	1.4956E-25	121.02
MSQHPIDIQEDFNNATIVN(de)ASSIIPK	2881.428	IPI00762812	IPI00762812	yes	yes		3	0.0045299	61.437
MSPWSN(de)WSECDPCIK	1895.7641	IPI00230718	IPI00230718	yes	no		2	0.0011665	120.92
MRSPGAQD(de)NVSVSQGMR	1818.8465	IPI00348586	IPI00348586	yes	no		3	7.2362E-18	177.58
MQVVSN(de)GTVTTAIWR	1661.8559	IPI00331175	IPI00331175	yes	no		2	0.002987	109.07
MQDTIEHHVNVSDTSAIPSTIEYGN(de)R	2913.3563	IPI00120953	IPI00120953	yes	yes		3	1.177E-13	124.4
MQDTIEHHVN(de)VSDTSAIPSTIEYGNR	2913.3563	IPI00120953	IPI00120953	yes	yes		3	1.177E-13	124.4
MPSQASAGNVYPQPIIN(de)SSMCIEDSR	2851.2939	IPI00224752	IPI00224752	yes	yes		3	0.0003784	80.102
MPFPIDQDFYVSPTFQDIIN(de)R	2542.2202	IPI00318175	IPI00318175	yes	yes		3	0.0001637	102.69
MNPDIDTGHN(de)TSAPAHWGEIK	2290.0437	IPI00314429	IPI00314429	yes	no		3	0.0026798	79.97
MN(de)MSVITIQEYEFEK	1860.8638	IPI00461438	IPI00461438	yes	yes		2	1.461E-12	172.91
MIVSNIDIGPTIIDIAGYDIN(de)KTQMDGMSIIPIIK	3801.997	IPI00221426	IPI00221426	yes	yes		4	0.0039151	54.003
MIHN(de)TTGVYTCSAHGTWTNEVIKR	2775.3221	IPI00475209	IPI00475209	yes	no	3,4		0.0008498	87.062
MIHN(de)TTGVYTCSAHGTWTNEVIK	2619.221	IPI00475209	IPI00475209	yes	no		3	0.013091	64.705
MIEN(de)GSISFIPTIR	1576.8283	IPI00123194	IPI00123194	yes	yes	2,3		3.5123E-75	222.75
MHING(de)SNVQVIHR	1503.7729	IPI00119063	IPI00119063	yes	yes	2,3		1.1665E-07	160.18
MGDREAIGN(de)ASQIFDSWIK	2137.0262	IPI00134585	IPI00134585	yes	yes	2,3		1.2101E-07	163.53
MFSQN(de)DTR	997.42873	IPI00129250	IPI00129250	yes	yes		2	0.018992	131.06
MENGN(de)ISTVTVFVFTGFPQIK	2328.1824	IPI00126685	IPI00126685	yes	yes		3	0.031301	36.253
MEISVGAIQAN(de)R	1287.6605	IPI00108811	IPI00108811	yes	yes		2	0.0015118	117.86
MEAHNVSAPFN(de)FSIPPGFGHR	2311.0957	IPI00115482	IPI00115482	yes	no	2,3,4		1.1536E-39	197.86
MEAHN(de)VSAPFNFSIPPGFGHR	2311.0957	IPI00115482	IPI00115482	yes	no	2,3,4		1.1536E-39	197.86
MDYNSFQGTPSN(de)ETK	1717.7254	IPI00876541	IPI00876541	yes	no		2	0.0022789	113.65
MDTSMN(de)FSR	1087.4427	IPI00136210	IPI00136210	yes	yes		2	5.7379E-48	218.6
MDGHCAPIRTEAGVFEYVADPTFEN(de)FTGGVKK	3542.6599	IPI00405742	IPI00405742	yes	no	3,4		0.0018423	77.522
MDGHCAPIRTEAGVFEYVADPTFEN(de)FTGGVK	3414.5649	IPI00405742	IPI00405742	yes	no		3	4.2349E-15	113.64
MDFIIFN(de)YSAPSYIR	1835.8916	IPI00553503	IPI00553503	yes	yes		2	0.0006942	127.75
MDEFCNSTFWN(de)ISIIK	2003.9121	IPI00420412	IPI00420412	yes	yes		2	1.4902E-60	214.22
MDEFCN(de)STFWNISIIK	2003.9121	IPI00420412	IPI00420412	yes	yes		2	1.4902E-60	214.22

MASN(de)NTASIAQAR	1333.6408	IPI00230194	IPI00230194	yes	yes		2	0.031968	34.386
MAIIQYGSQNQQQVAFPITYN(de)VTTIHEAIER	3562.7879	IPI00621027	IPI00621027	yes	yes		4	2.2495E-15	109.68
MAAAINATGRPIAFSCSWPAYEGGIPPKVN(de)YTEVSR	3880.9029	IPI00315593	IPI00315593	yes	no		4	2.194E-06	88.986
MAAAIN(de)ATGRPIAFSCSWPAYEGGIPPKVNYTEVSR	3880.9029	IPI00315593	IPI00315593	yes	no		4	2.194E-06	88.986
KYQTIN(de)CSVNVR	1480.7456	IPI00420835	IPI00420835	yes	no		2	0.0053616	109
KYHDYYITSTSN(de)GSIEGIENR	2446.1401	IPI00130754	IPI00130754	yes	yes	2,3		1.2006E-14	173.21
KYFDQVDIS(de)NGIDWSIDHK	2279.0859	IPI00133456	IPI00133456	yes	yes	2,3		1.6131E-48	207.43
KYEQAKN(de)ISQDIEK	1692.8683	IPI01027808	IPI01027808	yes	no		2	1.5929E-35	196.95
KWPERISAI(de)DNIINHSSIFIK	2480.354	IPI00123342	IPI00123342	yes	yes		3	1.2837E-05	126.84
KWGH(de)NVTEFQQR	1528.7535	IPI00754386	IPI00754386	yes	yes		2	0.0013287	141.37
KVVVVEQN(de)GSFQVK	1559.8671	IPI00463399	IPI00463399	yes	no		2	0.030111	98.105
KVPSN(de)STETVIESDQFQPGVR	2317.155	IPI00119299	IPI00119299	yes	no	2,3		4.2828E-06	126.84
KVIVAPPSEEAN(de)TTK	1582.8566	IPI00875419	IPI00875419	no	no		2	0.0018504	131.72
KVIIN(de)NSIDEPR	1396.7674	IPI00411145	IPI00411145	yes	no		2	0.0018539	124.86
KVEVEPIN(de)STAVHVSWK	1922.0262	IPI00110264	IPI00110264	yes	yes	2,3		1.2249E-75	225.01
KVEVEAVN(de)ATAVK	1356.7613	IPI00608063	IPI00608063	yes	no		2	4.022E-25	187.16
KVEAEAIN(de)ATAIR	1384.7674	IPI00754853	IPI00754853	yes	no		2	0.0014818	120.92
KVDN(de)ASIVADDMR	1432.698	IPI00356462	IPI00356462	yes	no		2	0.010163	102.64
KVCNGIGIGEFKDTISIN(de)ATNIK	2491.3105	IPI00121190	IPI00121190	yes	no		3	2.8943E-17	171.47
KTYAVYDIFDTAMIN(de)NSR	2121.0201	IPI00226790	IPI00226790	yes	no	2,3		7.6763E-08	164.53
KTTIEN(de)FTCPEYK	1629.7709	IPI00420810	IPI00420810	yes	yes		2	0.0026961	114.7
KTN(de)QSCEIVIDSTEKVNPSYIGR	2637.3068	IPI00310059	IPI00310059	yes	no		3	1.1796E-32	190.31
KTN(de)QSCEIVIDSTEK	1750.8407	IPI00310059	IPI00310059	yes	no		2	1.3429E-35	196.8
KTMFN(de)STEIK	1197.6064	IPI00677395	IPI00677395	yes	no		2	0.028311	103.08
KTGVHDGDFEYN(de)ITTTIAAINK	2407.202	IPI00108849	IPI00108849	yes	no	2,3		3.048E-06	149.36
KTGEAN(de)ITQIYTQEAIDFIQTQHAR	2875.4464	IPI00310090	IPI00310090	yes	no		3	1.3073E-84	222.24
KSSQSN(de)QTIWFGHFTTSTIMSPSPGIR	2994.4658	IPI00226714	IPI00226714	yes	no		3	0.0017344	74.748
KSPGYVIDIIVTPQN(de)K	1770.988	IPI00109946	IPI00109946	yes	yes	2,3		0.0016573	137.44
KSEEMINIVN(de)NSKDFVENVTSGNAVDFFPVIR	3611.793	IPI00128287	IPI00128287	yes	yes		3	1.9892E-15	116.28
KSDTQNIIYN(de)VSTGR	1694.8588	IPI01023214	IPI01023214	yes	no		2	0.0005768	125.45
KSCHTAVGTSEGWNVPMGIIYN(de)QTGSCK	3081.4107	IPI00471080	IPI00471080	yes	no		3	0.0035791	64.845
KQVTPIFFYFQ(de)NR	1686.8882	IPI00319509	IPI00319509	yes	no	2,3		0.0012534	124.86
KPISQEFEAYVN(de)ASGEHGIVVFSIGSMVSEIPEKK	3805.9237	IPI00134691	IPI00134691	yes	no		4	1.1446E-09	98.151
KPIIGHYKPDTIAVVIEN(de)GTSIDR	2635.4334	IPI00410796	IPI00410796	yes	no		3	3.263E-08	146.89
KNVNISYTV(de)NDSFFPQRPQK	2381.2128	IPI00381178	IPI00381178	yes	yes	2,3		0	316.97
KNVN(de)ISYTVNDSFFPQRPQK	2381.2128	IPI00381178	IPI00381178	yes	yes	2,3		0	316.97
KNIQAVNEIIATISQC(de)NDTSSAAMVQCIR	3234.5795	IPI00138342	IPI00138342	yes	no		3	0.010221	55.063
KN(de)ITSPVGVQPIINEHTFCAGITK	2623.3792	IPI00409148	IPI00409148	yes	yes		3	0.0053493	72.916
KN(de)ISEIWDAYCYR	1716.793	IPI00987265	IPI00987265	yes	no		2	3.957E-13	170.56
KMSN(de)ITFR	995.52224	IPI00460063	IPI00460063	yes	no		2	0.0098274	143.7
KMISAFN(de)ATSGK	1253.6438	IPI00466999	IPI00466999	yes	no		2	0.016333	97.69
KKENGVFEEISN(de)SSGR	1779.8751	IPI00757771	IPI00757771	yes	no	2,3		3.7333E-116	214.81
KITDVETQVIN(de)QTSR	1730.9163	IPI00114350	IPI00114350	yes	yes		2	0.0013426	121.27
KISTN(de)ITIVCKPGDIESAPVIR	2410.3254	IPI00308971	IPI00308971	yes	yes		3	8.3826E-06	124.2
KIPIN(de)FTEGAR	1244.6877	IPI00129243	IPI00129243	yes	no		2	0.020314	93.345
KINIDGSN(de)YTIIK	1477.814	IPI00119063	IPI00119063	yes	yes		2	6.4418E-34	199.13
KIN(de)CSQEVPGSSQCDREPEPR	2472.1122	IPI00881836	IPI00881836	yes	yes		3	4.7935E-05	145.91
KIIASPNEEN(de)MTEIISMR	2075.0391	IPI00322575	IPI00322575	yes	no	2,3		4.2185E-08	164.93
KIDFIVIN(de)ETR	1346.7558	IPI00125813	IPI00125813	yes	no	2,3		2.9038E-14	178.15
KHYQVTGYGIN(de)GTGDSNDFWR	2414.104	IPI00221669	IPI00221669	yes	no		3	0.0006841	115.26
KGIVSGGVYNSHVGCIPYTIPPCEHHV(de)NGSRPPCTGEGDTPF	4558.1329	IPI00113517	IPI00113517	yes	yes		4	0.0058732	44.674
KGCADYCN(de)QTITK	1557.6916	IPI00987265	IPI00987265	yes	no		2	0.0012733	124.51
KFPVPFQKEN(de)VTATIVEIGR	2272.258	IPI00134691	IPI00134691	yes	yes	2,3		6.5926E-28	188.58
KFN(de)STQIAAMAPEHEEPR	2054.9844	IPI00856861	IPI00856861	yes	no		3	0.0003056	130.01
KFHVN(de)YTQPIVAVK	1642.9195	IPI00123704	IPI00123704	yes	no		2	0.0030233	134.21
KFEAENISN(de)YTAIIISQDGK	2240.1325	IPI00464135	IPI00464135	yes	no	2,3		6.8226E-11	168.68
KFEAEN(de)ISNYTAIIISQDGK	2240.1325	IPI00464135	IPI00464135	yes	no	2,3		6.8226E-11	168.68
KEQETCIAPEIEHGN(de)YSTTQR	2490.1445	IPI00122117	IPI00122117	yes	yes	2,3		6.3316E-06	138.28
KENGVFEEISNS(de)SGR	1651.7802	IPI00757771	IPI00757771	yes	no		2	5.1915E-47	207.55
KENG(de)VFEEISNSSGR	1651.7802	IPI00757771	IPI00757771	yes	no		2	5.1915E-47	207.55
KEN(de)SSYQVINWR	1522.7528	IPI00153316	IPI00153316	yes	yes	2,3		6.5952E-81	228.78
KEN(de)SSEICSNNGECVCGQCVCR	2646.0349	IPI00132474	IPI00132474	yes	no		3	4.2464E-06	137.13
KEDSCQIN(de)YSEGPCIGMQER	2400.0144	IPI00127352	IPI00127352	yes	yes	2,3		1.7983E-20	182.82
KDTCAQECSHFN(de)ITKVESR	2309.0529	IPI00132474	IPI00132474	yes	no		3	3.9812E-12	171.29
KDTCAQECSHFN(de)ITK	1837.8087	IPI00132474	IPI00132474	yes	no	2,3		4.2721E-110	243.04
KDGSQDFN(de)ETWENYEK	1988.8388	IPI00128206	IPI00128206	yes	yes		2	0.0003688	149.52
KDGAFHHIHSTPFGN(de)YSFISVDATQRPGPK	3310.6272	IPI00226714	IPI00226714	yes	no		3	0.0050269	67.899
KDDEPIETTGDFN(de)TTK	1809.8269	IPI00129968	IPI00129968	yes	yes		2	0.0025101	111.06
KCEVIC(de)NQSNKPITITVEQSR	2503.2523	IPI00626537	IPI00626537	yes	yes		3	2.0927E-05	124.08
KCEAN(de)ITISSPAR	1445.7297	IPI00894972	IPI00894972	yes	no		2	0.017489	90.444
KAWGISVINPN(de)KTK	1554.8882	IPI00989096	IPI00989096	yes	yes	2,3		0.0015686	125.73
KAFITN(de)FSMIIDGVTYPGVVKEK	2556.3662	IPI00312711	IPI00312711	yes	no		3	2.9459E-32	186.01
KAFITN(de)FSMIIDGVTYPGVVK	2299.2286	IPI00312711	IPI00312711	yes	no	2,3		7.9483E-06	145.04
K(de)IVIYIEHNIEKNSTK	1928.0731	IPI00928320	IPI00928320	yes	no	2,3		1.0029E-11	173.28
IYWTDGDNISMANMDGSN(de)HTIIFSGQKGPVGIAIDFPESK	4325.0409	IPI00119063	IPI00119063	yes	yes		4	0.0006539	51.264
IYWTDGDNISMANMDGSN(de)HTIIFSGQK	3014.3539	IPI00119063	IPI00119063	yes	yes	2,3		3.1478E-14	125.73
IYWTDGDN(de)ISMANMDGSNHTIIFSGQKGPVGIAIDFPESK	4325.0409	IPI00119063	IPI00119063	yes	yes		4	0.0006539	51.264
IYWTDGDN(de)ISMANMDGSNHTIIFSGQK	3014.3539	IPI00119063	IPI00119063	yes	yes	2,3		3.1478E-14	125.73
IYWISSGNHTIN(de)R	1559.7845	IPI00119063	IPI00119063	yes	yes	2,3		7.0216E-19	155.08
IYWISSGN(de)HTINR	1559.7845	IPI00119063	IPI00119063	yes	yes	2,3		7.0216E-19	155.08
IYVIDGTQN(de)DTAFVFPR	1954.9789	IPI00308658	IPI00308658	yes	no		2	0.0004842	131.72
IYSDQCHHNISIIPPPTEIVCN(de)R	2762.3269	IPI00123442	IPI00123442	yes	yes		3	0.0023875	81.878
IYSDQCHHN(de)ISIIPPPTEIVCNR	2762.3269	IPI00123442	IPI00123442	yes	yes		3	0.0023875	81.878
IYNVTHQFCN(de)ASVMDPTCVR	2411.0821	IPI00126000	IPI00126000	yes	no	2,3		1.1616E-28	189.9
IYN(de)VTHQFCNASVMDPTCVR	2411.0821	IPI00126000	IPI00126000	yes	no	2,3		1.1616E-28	189.9
IYIRN(de)ESEFRDK	1568.7947	IPI00115976	IPI00115976	yes	yes		3	0.013929	110.66

IYIDHNN(de)ITR	1257.6466	IPI00120187	IPI00120187	yes	yes		2	0.0023201	131.83
IYAGMVSIMDEAVGNV(de)TK	1896.9325	IPI00652358	IPI00652358	yes	no		2	2.3805E-20	149.95
IWIPVN(de)ITWADIEDKDGR	2140.0953	IPI00126253	IPI00126253	yes	no		2	2.5486E-07	161.99
IVTQTIPCN(de)KTIFWSK	1935.0288	IPI00307966	IPI00307966	yes	yes		2	0.032618	106.42
IVTQTIPCN(de)K	1172.6223	IPI00307966	IPI00307966	yes	yes		2	7.9334E-19	190.96
IVSDVQTAVKTN(de)SSFIQGFVDHVKEDCDR	3293.5987	IPI00928320	IPI00928320	yes	no		3	0.004246	73.655
IVQDVAN(de)NTNEEAGDGTTTATVIAR	2559.2413	IPI00308885	IPI00308885	yes	no	2,3		2.5918E-18	172.37
IVPCEFGVIYPIAEN(de)FSR	2110.0557	IPI00121908	IPI00121908	yes	no		3	0.016122	75.911
IVKIPSGTIVISN(de)ATEGDGGIYR	2359.2747	IPI00129159	IPI00129159	yes	no		3	6.2812E-08	140.63
IVGSPFIGCTVVN(de)K	1489.7963	IPI00134808	IPI00134808	yes	no		2	1.8161E-08	162.93
IVEDIESFIKPYSVEEQKN(de)ITSCPDGAPFIQHGPDYR	4278.0579	IPI00124221	IPI00124221	yes	yes		4	0.0003879	77.522
IVDVN(de)ITSEGKVK	1400.7875	IPI00113528	IPI00113528	yes	no		2	0.0025652	143.94
IVDVN(de)ITSEGK	1173.6241	IPI00113528	IPI00113528	yes	no		2	0.0017004	124.12
IVAN(de)ISGCAAVNSETIMCCIR	2338.0902	IPI00222489	IPI00222489	yes	yes	2,3		9.1833E-22	184.6
IV(de)VPINNRENISDPTSPIR	2133.1542	IPI00136925	IPI00136925	yes	no	2,3		0.0032271	105.39
ITYESGFIN(de)YSK	1420.6874	IPI00666034	IPI00666034	yes	no		2	0.0003681	143.98
ITWSNAN(de)GTASYR	1439.6793	IPI00469426	IPI00469426	yes	no		2	0.0011366	141.34
ITVVYAEN(de)GTVIQGTTVASVYK	2312.2264	IPI00317356	IPI00317356	yes	yes	2,3		2.197E-16	177.08
ITVPSSQN(de)SSFR	1321.6626	IPI00515360	IPI00515360	yes	no		2	0.011516	93.345
ITSCATN(de)ASMCGDEAR	1742.7022	IPI00119063	IPI00119063	yes	yes		2	0	309.88
ITN(de)VTFPTGVVTNIHGDMDK	2158.0729	IPI00798466	IPI00798466	yes	no	2,3		0.0011045	96.234
ITN(de)QTIGFSFAVEQDIPVK	2106.0997	IPI00624663	IPI00624663	yes	yes	2,3		0	321.68
ITIEVFDPVPKPSIEIN(de)KTEASTDSCHIR	3295.6758	IPI00109946	IPI00109946	yes	yes		3	2.7418E-11	110.83
ITIAIN(de)NTITPHTIPPGTIVYK	2376.3417	IPI00309230	IPI00309230	yes	no	2,3		4.5159E-22	184.01
ITHIPDDIPSNITVIN(de)ITHNQIR	2623.4082	IPI00320618	IPI00320618	yes	yes		3	0.0001812	92.993
ITHIPDDIPSN(de)ITVINITHNQIR	2623.4082	IPI00320618	IPI00320618	yes	yes		3	0.0001812	92.993
ITFFNSTIN(de)TSGIVAQGEAIPIPGAHRPGIVTK	3405.8409	IPI00153317	IPI00153317	yes	yes	3,4		3.6028E-16	115.55
ITFFN(de)STINTSGIVAQGEAIPIPGAHRPGIVTK	3405.8409	IPI00153317	IPI00153317	yes	yes	3,4		3.6028E-16	115.55
ITEFTHN(de)STMDYK	1585.7083	IPI00130010	IPI00130010	yes	no		2	0.001008	141.37
ITDTICGVGNMSAN(de)ASDQER	2137.9368	IPI00114065	IPI00114065	yes	no		2	7.2709E-06	127.49
ITDTICGVGN(de)MSANASDQER	2137.9368	IPI00114065	IPI00114065	yes	no		2	7.2709E-06	127.49
ITDNMFCAGFKVN(de)DTK	1859.8546	IPI00114206	IPI00114206	yes	yes		2	0.010723	104.45
ITAN(de)STWQPDKAK	1458.7467	IPI00653675	IPI00653675	yes	no		2	3.587E-32	199.13
ISVPDGIKVSN(de)SSAR	1528.8209	IPI00120761	IPI00120761	yes	no		2	0.0083016	110.84
ISTSPFAIN(de)ITMIPK	1631.8957	IPI00666034	IPI00666034	yes	no		2	6.8266E-08	163.8
ISTNI(de)TIVCKPGDIESAPVIR	2282.2304	IPI00308971	IPI00308971	yes	yes		2	9.4587E-29	187.43
ISQN(de)ASIGPHVRPIPIQYEDKEVEPGTICDVAGWGVVTHAGR	4552.2921	IPI00116945	IPI00116945	yes	no		4	1.3488E-25	115.9
ISQAIGN(de)ITVVQK	1369.7929	IPI00112032	IPI00112032	yes	no		2	0.0079124	96.866
ISPIHIAIN(de)FSIDPK	1663.9297	IPI00115976	IPI00115976	yes	yes		2	0.0031121	104.95
ISPGGAEMFQVQDMVVSQEKGN(de)CSIQR	2994.3998	IPI00130117	IPI00130117	yes	no		3	0.0002291	87.319
ISNISN(de)ISHDIVQEATDHAYNIQQEADEISR	3509.6659	IPI00223446	IPI00223446	yes	yes	3,4		3.6342E-20	125
ISN(de)VTPEDAGTYYCVK	1815.8349	IPI00129158	IPI00129158	yes	no		2	1.6703E-08	167.69
ISN(de)ITIIIEMEKESAR	1845.987	IPI00356462	IPI00356462	yes	no		2	0.016498	97.235
ISISEN(de)YTISIANAK	1622.8516	IPI00121378	IPI00121378	yes	no		2	0.0005461	146.59
ISHYN(de)DTYPISPPQR	1786.8638	IPI00113039	IPI00113039	yes	no	2,3		1.2203E-91	229.68
ISHYKQN(de)FSFCR	1585.746	IPI00381303	IPI00381303	yes	yes	2,3		0.026539	101.53
ISHEN(de)GTIICSK	1357.666	IPI00338094	IPI00338094	yes	yes		2	0.0026811	112.43
ISHDGN(de)ETIPIHIYVK	1834.9577	IPI00405742	IPI00405742	yes	no	2,3		0	312.78
ISGNSIHN(de)ASIPEHIAYGSVITVK	2506.318	IPI00221669	IPI00221669	yes	no	2,3		5.0277E-10	152.47
ISGN(de)ITIIR	985.59203	IPI00137311	IPI00137311	yes	yes		2	0.017366	115.57
ISGKPTN(de)VSVSVIMSEGDGICY	2312.1028	IPI00473880	IPI00473880	yes	no		2	2.483E-39	199.97
ISFGSN(de)HSDFK	1237.5728	IPI00121475	IPI00121475	yes	no		2	0.019647	88.338
ISEN(de)GSSVAGIISSPNMEK	1918.9306	IPI00399798	IPI00399798	yes	no		2	7.4072E-06	158.44
ISEGNRTITIINVTRN(de)DTGPYVCETR	2978.488	IPI00108535	IPI00108535	yes	no		4	9.0003E-07	102.48
ISEGNR(de)TITIINVTRNDTGPYVCETR	2978.488	IPI00108535	IPI00108535	yes	no		4	9.0003E-07	102.48
ISEGN(de)RTITIINVTRNDTGPYVCETR	2978.488	IPI00108535	IPI00108535	yes	no		4	9.0003E-07	102.48
ISEEFIKN(de)ISASAR	1563.8257	IPI00894970	IPI00894970	yes	no	2,3		9.2053E-08	160.8
ISDTN(de)ITAIPQGIPTSITEVHIDGNKITKVDAPSIK	3786.0415	IPI00123196	IPI00123196	yes	yes	3,4		0.001957	70.109
ISDTN(de)ITAIPQGIPTSITEVHIDGNKITK	3075.6452	IPI00123196	IPI00123196	yes	yes		3	1.205E-08	103.03
ISDTN(de)ITAIPQGIPTSITEVHIDGNK	2733.4185	IPI00123196	IPI00123196	yes	yes		2	0.0001915	83.826
ISCAFKTEN(de)QTR	1453.6984	IPI00120245	IPI00120245	yes	no		2	0.018894	105.95
ISASGAEIEAIEAQVIN(de)ISIK	2155.1736	IPI00312711	IPI00312711	yes	no	2,3		1.3926E-182	267.39
ISAIDNIIN(de)HSSIFIKGAR	2068.1429	IPI00123342	IPI00123342	yes	yes		3	0.0009926	115.5
ISAIDNIIN(de)HSSIFIK	1783.9832	IPI00123342	IPI00123342	yes	yes	2,3		1.1663E-153	262.23
IRPIFN(de)KSFESTVGQGSDTYSYIFR	2911.4505	IPI00108844	IPI00108844	yes	yes	3,4		4.332E-13	163.91
IRPHFISVCDPDFSQIN(de)CSEGYIQNYR	3314.5237	IPI00115626	IPI00115626	yes	yes	3,4		1.1064E-38	191.4
IRNPCTSE(de)QNCTSPFSYK	2187.9677	IPI00132474	IPI00132474	yes	yes	2,3		8.0652E-12	170.11
IRN(de)SSIGGVINKYDVVIR	2002.1324	IPI00314673	IPI00314673	yes	yes		3	0.0020814	111.01
IRDYEEN(de)SSSCHKEVQIIK	2334.1274	IPI00330594	IPI00330594	yes	yes		3	0.023303	86.488
IRDQEIEDNVHISPN(de)GSIITIVGTRPSNHGAYR	3657.8612	IPI00515360	IPI00515360	yes	yes	3,4		0.0021588	73.13
IRAEQITIHAIGIGEAN(de)KTQIR	2431.3659	IPI00675799	IPI00675799	yes	no		3	1.1421E-05	135.49
IQVTIYN(de)CSFGR	1456.7133	IPI00405742	IPI00405742	yes	no		2	5.6743E-06	153.39
IQKENDNFN(de)ISKDDIDITIFHGENK	2946.4359	IPI00674255	IPI00674255	yes	yes		3	8.1655E-09	123.6
IQIEFRPIDIN(de)STAAGIPR	2110.1535	IPI00115510	IPI00115510	yes	yes	2,3		0.0009243	145.86
IQDIEITGSPVSNISAHIFSN(de)ISSIEK	2898.4975	IPI00119522	IPI00119522	yes	yes	2,3,4		1.035E-10	109.68
IQDIEITGSPVSN(de)ISAHIFSNISSIEK	2898.4975	IPI00119522	IPI00119522	yes	yes	2,3,4		1.035E-10	109.68
IQDFIVDNETFSGFIQHN(de)ISIPR	2689.35	IPI00112614	IPI00112614	yes	yes	2,3		6.8688E-06	106.73
IQDFIVDN(de)ETFSGFIQHNISIPR	2689.35	IPI00112614	IPI00112614	yes	yes	2,3		6.8688E-06	106.73
IQCVDGN(de)WTTIPVCIEEER	2318.0671	IPI00130010	IPI00130010	yes	no		2	5.6683E-27	186.15
IPSSIENATSISIMN(de)ITGTAICHISDIPPDGIR	3492.7593	IPI00121776	IPI00121776	yes	yes		3	7.0869E-21	131.79
IPSSIEN(de)ATSISIMNITGTAICHISDIPPDGIR	3492.7593	IPI00121776	IPI00121776	yes	yes		3	7.0869E-21	131.79
IPNNTQWITW(de)SPEGHK	1906.9326	IPI00125813	IPI00125813	yes	no	2,3		3.1182E-12	171.78
IPN(de)ISIDVIQPSFPEIIIESHMVMIR	2990.5973	IPI00221669	IPI00221669	yes	no		3	3.7575E-14	126.02
IPFSPENTREEDFYVNETST(de)VK	2601.2235	IPI00116105	IPI00116105	yes	yes		3	0.017198	77.899
IPEN(de)QTIPGEIPEHAGPAEGVHDSR	2649.2783	IPI00856221	IPI00856221	yes	yes		3	0.0001158	90.464
INSAPVEGYSEHVGN(de)KTTIR	2171.0971	IPI00349830	IPI00349830	yes	no		2	7.7829E-05	119.62

INSAPVEGYSEHVGN(de)K	1699.8166	IPI00349830	IPI00349830	yes	no		2	0.0008457	142.54
INNGGCQDICIITHQGHVN(de)CSCR	2712.1737	IPI00119063	IPI00119063	yes	yes		3	0.0020384	82.609
INN(de)ITNIGPIDMK	1441.7599	IPI00330680	IPI00330680	yes	no		2	0.0008211	125.73
INMTIPDAIVPTFSISN(de)HSIK	2297.209	IPI00469218	IPI00469218	yes	yes	2,3		2.2738E-158	254.32
INIDGSNY(de)TIIK	1349.7191	IPI00119063	IPI00119063	yes	yes		2	5.716E-11	173.72
INETHIFN(de)GSNWIMIIYK	2192.1088	IPI00108844	IPI00108844	yes	yes	2,3		8.1919E-08	160.65
IN(de)YTCNQGYR	1287.5666	IPI00138061	IPI00138061	yes	no		2	6.6974E-37	205.53
IN(de)YSIPTGQSMEVQIPK	1903.9713	IPI00403938	IPI00403938	yes	no		2	3.6852E-36	199.15
IN(de)VTSPDIFR	1160.619	IPI00116913	IPI00116913	yes	yes		2	0.0019593	139.86
IN(de)VTEVYDKIKR	1476.83	IPI00225072	IPI00225072	yes	no		2	0.0073012	127.79
IN(de)VTEVYDKIK	1320.7289	IPI00225072	IPI00225072	yes	no		2	0.0001505	154.15
IN(de)VTEVYDK	1079.5499	IPI00225072	IPI00225072	yes	no		2	0.024878	120.06
IN(de)VSYTGERPSSNMVIVDVK	2207.1256	IPI00624663	IPI00624663	yes	yes	2,3		3.6877E-05	141.13
IN(de)VSYTGER	1037.5142	IPI00624663	IPI00624663	yes	yes		2	0.0045109	133.81
IN(de)VSHAGAPIGEEYIIVFSR	2171.1375	IPI00785452	IPI00785452	yes	no		3	0.0004278	99.663
IN(de)TTSDEKDPTNPFRFPNIGVEK	2618.2976	IPI00131881	IPI00131881	yes	no		3	0.03739	76.176
IN(de)TTSDEKDPTNPFR	1733.822	IPI00131881	IPI00131881	yes	no		2	0.0022526	123.76
IN(de)SSVTDVEEIIGVR	1629.8574	IPI00623114	IPI00623114	yes	no		2	0.0017414	135.14
IN(de)QTFSGIMTMINMQFVIR	2243.1265	IPI00120015	IPI00120015	yes	yes		2	0.014235	66.809
IN(de)MTIPDAIVPTFSISNHSIK	2297.209	IPI00469218	IPI00469218	yes	yes	2,3		2.2738E-158	254.32
IN(de)KTVAIHTIDPEKIGQGGVQK	2345.3067	IPI00323624	IPI00323624	yes	no	2,3		1.9046E-05	146.13
IN(de)KTVAIHTIDPEK	1577.8777	IPI00323624	IPI00323624	yes	no	2,3		7.3718E-26	186.42
IN(de)ITISDVNDHTPR	1593.8111	IPI00228716	IPI00228716	yes	no		2	0.0065099	95.981
IN(de)ITEEEKIINRR	1626.9053	IPI00320204	IPI00320204	yes	yes	2,3		0.019054	110.08
IN(de)ITEEEKIINR	1470.8042	IPI00320204	IPI00320204	yes	yes		2	1.6709E-15	183.79
IN(de)ISEGEVAATVK	1329.714	IPI00458003	IPI00458003	yes	yes		2	6.02E-103	241.42
IN(de)GTDPIVAADSKR	1455.7682	IPI00119063	IPI00119063	yes	yes		2	1.7722E-47	207.4
IN(de)GTDPIVAADSK	1299.667	IPI00119063	IPI00119063	yes	yes		2	5.6642E-71	227.47
IN(de)ETHIFNGSNWIMIIYK	2192.1088	IPI00108844	IPI00108844	yes	yes	2,3		8.1919E-08	160.65
IN(de)ATHFYACGTHAFQPICAAIDAETFIIPTSFEEGKEK	4284.0296	IPI00124666	IPI00124666	yes	no		4	0.0009884	62.958
IMQDPIQQAEGIYCN(de)R	1934.8979	IPI00314549	IPI00314549	yes	yes		2	5.041E-26	188.9
IMNIEFYDCSCVSGSGFQKGN(de)HSAR	2863.2476	IPI00230319	IPI00230319	yes	no		3	0.0015314	85.737
IMNAPIYIAEWQNITKN(de)ISEDR	2618.3163	IPI00930882	IPI00930882	yes	no		3	0.031113	72.359
IMNAPIYIAEWQN(de)ITKNISEDR	2618.3163	IPI00930882	IPI00930882	yes	no		3	0.031113	72.359
IMNAPIYIAEWQN(de)ITK	1903.9866	IPI00930882	IPI00930882	yes	no		2	4.1601E-47	205.98
IMGINN(de)VTSQSWQPQTYQICIVDPVSASVK	3362.6639	IPI00555130	IPI00555130	yes	yes		3	0.016621	43.895
IMESHPN(de)GTFSAK	1417.666	IPI00177214	IPI00177214	yes	no		2	3.5369E-70	223.22
IKVSN(de)VSCEASVSK	1506.7712	IPI00135560	IPI00135560	yes	no		2	0.005375	116.78
IKPAFIKPYGTVTAAN(de)SSFITDGASAMIIMSEDR	3601.816	IPI00115607	IPI00115607	yes	no	3,4		0.0023982	72.728
IKN(de)TTNQIAIIK	1355.8136	IPI00985989	IPI00985989	yes	no		3	0.023251	103.43
IKIMESH(de)PNGTFSAK	1658.845	IPI00177214	IPI00177214	yes	no		2	0.0021662	124.78
IKIDN(de)YSTQEIGR	1535.7944	IPI00122257	IPI00122257	yes	no	2,3		3.6179E-33	194.55
IKFIEAGIYEVPIIITDSGNPPK(de)SNISIIR	3296.8384	IPI00323134	IPI00323134	yes	no	3,4		9.451E-21	153.87
IKEQVVGIIAQNN(de)CSCESK	2176.0616	IPI00122592	IPI00122592	yes	yes		3	0.0009808	114.99
IIYISAEDFSIDHSPN(de)STAGPSCSIIQEAFRR	3580.7256	IPI00115530	IPI00115530	yes	yes		3	0.0020021	58.722
IIYISAEDFSIDHSPN(de)STAGPSCSIIQEAFR	3424.6245	IPI00115530	IPI00115530	yes	yes		3	0.0017638	59.893
IIY(de)EGGDIPDFRKENSSYQVINWR	2898.4301	IPI00153316	IPI00153316	yes	yes		3	0.027743	66.536
IISYN(de)VSCSINEETQSVIEIFDPQHR	3077.4764	IPI00119299	IPI00119299	yes	no		3	1.0337E-10	109.65
IISVN(de)MTDKK	1147.6271	IPI00848693	IPI00848693	yes	yes		2	0.026563	106.4
IISQKN(de)SSQAR	1230.668	IPI00420867	IPI00420867	yes	no		2	0.015234	117.09
IISQAPSTPSPNMFTINN(de)ETGDIITVAAGIDREK	3599.8141	IPI00323134	IPI00323134	yes	no	3,4		3.8762E-10	99.753
IIQYYGN(de)FSR	1259.6299	IPI00267963	IPI00267963	yes	yes		2	0.0023214	135.99
IIQVVYIHSNN(de)ITK	1640.925	IPI00123194	IPI00123194	yes	yes		2	3.681E-47	203.11
IIQTAEHN(de)ISGAER	1537.7849	IPI00403938	IPI00403938	yes	no		2	1.8419E-47	205.27
IIQQQSN(de)QSSQFIHSVER	2128.0661	IPI00876541	IPI00876541	yes	no	2,3		9.8687E-37	199.01
IIPHIEKPIQN(de)FTICFR	2125.1507	IPI00309214	IPI00309214	yes	yes	2,3		0.00237	135.08
IINN(de)ITSIK	1014.6073	IPI01027808	IPI01027808	yes	no		2	0.011792	121.73
IINKFN(de)SSSSSIEEK	1681.8523	IPI00338561	IPI00338561	yes	yes		2	5.4415E-18	182.57
IINDYVSN(de)QTQGMIK	1722.8611	IPI00135635	IPI00135635	yes	no		2	5.5395E-60	211.7
IIN(de)TTDVYIIPSINPDGFER	2276.1689	IPI00130573	IPI00130573	yes	yes		2	0.0095965	77.287
IIN(de)QTIRENIK	1340.7776	IPI00460063	IPI00460063	yes	no		2	3.4291E-14	182.28
IIN(de)QTIR	856.51305	IPI00460063	IPI00460063	yes	no		2	3.8944E-08	172.14
IIN(de)QTADMIQIASK	1544.8232	IPI00126864	IPI00126864	yes	no		2	0	225.09
IIN(de)ITSPEATAK	1256.6976	IPI00115089	IPI00115089	yes	yes		2	0.011286	93.766
IIN(de)ITFIDITR	1317.7656	IPI00343568	IPI00343568	yes	no		2	0.0001399	150.49
IIN(de)HSIIIHK	1186.7186	IPI00406603	IPI00406603	yes	yes		2	7.4601E-05	163.38
IIN(de)CTNIDCIQISCAVGR	2106.002	IPI00345112	IPI00345112	yes	yes		2	0.0003273	122.49
IIN(de)ATHQIGCQSSISGDTGVIHVVEKEEDIK	3376.6933	IPI00118674	IPI00118674	yes	yes	3,4		2.3557E-05	89.723
IIMNEGGHYN(de)ASSGK	1576.7304	IPI01026847	IPI01026847	yes	no		2	0.01215	87.519
IIISPEEN(de)VTITCTAENQIER	2429.2108	IPI00121378	IPI00121378	yes	no		2	5.5721E-06	129.51
IIIN(de)FTSMDIYR	1484.7697	IPI00125182	IPI00125182	yes	no		2	0.0015387	118.87
IIIKAPSHN(de)TTEPDPHSISPEIQAIISEVAQHDVQNGR	4141.1192	IPI00165807	IPI00165807	yes	no		4	0.0013749	62.57
IIIGGIPVSGTFHN(de)FSGCISNVFVQR	2818.4589	IPI00116913	IPI00116913	yes	no		3	2.2429E-06	95.48
IIGGDEDAIRPQMQQIIDFETAIAN(de)ITIPQEK	3623.8505	IPI00396840	IPI00396840	yes	no		3	4.2211E-20	145.33
IIFAN(de)VSVR	1017.5971	IPI00173158	IPI00173158	yes	no		2	0.0024698	144.77
IIEYNYN(de)STK	1243.6085	IPI00109946	IPI00109946	yes	no		2	0.0022734	136.5
IIEFDSTN(de)ASEGAQPPGKPYPPYSIAK	2876.4232	IPI00459432	IPI00459432	yes	yes	2,3		4.4098E-18	166.21
IIDIIPDGYPQISCIPKEEN(de)ATIATYPEFGVIDIK	3932.0169	IPI00124221	IPI00124221	yes	no	3,4		0.0018683	69.492
IIASPNEENM(de)TEIISMR	1946.9441	IPI00322575	IPI00322575	yes	no	2,3		6.8635E-06	158.98
IIASN(de)ITETMR	1247.6544	IPI00132600	IPI00132600	yes	no		2	2.1132E-117	253.76
IIAN(de)ISYTIQIDGHR	1712.921	IPI00894972	IPI00894972	yes	no		2	0.019801	78.285
IIAENNEIISNIRDSVIN(de)ISESVEDGPR	3095.5735	IPI00114044	IPI00114044	yes	yes	3,4		4.3077E-13	133.47
IHYIYIQNNFITEIPIESFQN(de)ATGIR	3093.5924	IPI00122293	IPI00122293	yes	yes		3	6.3771E-13	138.09
IHVAQPEN(de)DSHVAIIK	1769.9424	IPI00850413	IPI00850413	yes	yes	2,3		0.0004659	147.1
IHVAGGTHSVNQTVN(de)K	1660.8645	IPI00469426	IPI00469426	yes	no		2	0.0060005	95.57
IHVAGGTHSVN(de)QTVNK	1660.8645	IPI00469426	IPI00469426	yes	no		2	0.0060005	95.57

IHRIN(de)ASIADIQSK	1564.8685	IPI00116913	IPI00116913	yes	no	2,3	7.4079E-08	162.37
IHN(de)GSEEAIAQYK	1458.7103	IPI00380296	IPI00380296	yes	no	2	0.0015976	130.24
IHKDN(de)TTCYEFKK	1682.8086	IPI00119063	IPI00119063	yes	yes	2	0.0032224	143.42
IHIIPSMNPDGYEVAAAQGPN(de)MSGYIVGR	3056.4848	IPI00128260	IPI00128260	yes	yes	3	1.2597E-50	199.75
IHIGNYN(de)GTAGDAIR	1570.7852	IPI00310797	IPI00310797	yes	yes	2	0.0021258	111.64
IHHWSSPKPICSIVN(de)SSIVVK	2387.2784	IPI00118011	IPI00118011	yes	yes	2,3	2.9013E-05	113.47
IGYDPYANPPNYGNPDPIVIN(de)NTHR	2810.3412	IPI00316430	IPI00316430	yes	no	3	1.5131E-09	124.4
IGVTN(de)ASIVIFRPGSVR	1785.0261	IPI00652675	IPI00652675	yes	yes	3	0.024891	90.759
IGVQMHPGQEIHN(de)FTITGR	2134.0742	IPI00119299	IPI00119299	yes	no	2,3	0.0002031	122.49
IGPGEPIEIICN(de)VSGAIPPPGR	2242.178	IPI00321348	IPI00321348	yes	no	2,3	9.4523E-06	119.27
IGNFSEIATHN(de)QTFIK	1818.9264	IPI00855103	IPI00855103	yes	no	2	0.0023996	112.34
IGN(de)WTGEWPETEIVANIWMK	2373.1464	IPI00322497	IPI00322497	yes	yes	2,3	3.9359E-95	231.06
IGN(de)VTTWISSSNPPVTFAAIYWEEPDVSGHK	3401.6568	IPI00225072	IPI00225072	yes	no	3	1.9522E-12	105.63
IGN(de)FTIAYSAKPETADNQR	2095.0334	IPI00229198	IPI00229198	yes	no	2,3	0.0008746	109.07
IGN(de)FSEIATHNQTFIK	1818.9264	IPI00855103	IPI00855103	yes	no	2	0.0023996	112.34
IGISFNSITVMEN(de)GSIANVPHIREIHIDNNKIIR	3814.0312	IPI00123196	IPI00123196	yes	yes	4	0.044284	33.487
IGISFNSITVMEN(de)GSIANVPHIR	2468.2846	IPI00123196	IPI00123196	yes	yes	2,3	7.1552E-69	216.17
IGHWIPAAPGRIEKIEGIIAN(de)VSR	2596.4602	IPI00115516	IPI00115516	yes	no	3	0.014442	73.783
IGHTN(de)ASIMIFEVK	1558.8177	IPI00858152	IPI00858152	yes	yes	2	0.0010054	144.68
IGGWN(de)ITGPWAK	1298.6772	IPI00396840	IPI00396840	yes	no	2	0.0026628	134.56
IGEMWNN(de)ISDNEKQPYVTK	2265.0736	IPI00228879	IPI00228879	yes	no	3	0.020846	82.663
IGDVEVNAGQN(de)ATFQCIATGR	2220.0593	IPI00123040	IPI00123040	yes	no	2	7.472E-40	201.27
IGAINSTISN(de)ESKEAFIDWAR	2321.1652	IPI00187353	IPI00187353	yes	yes	2,3	3.5485E-05	117.26
IGAINSTISN(de)ESK	1332.6885	IPI00187353	IPI00187353	yes	yes	2	5.3424E-06	156.6
IGAIN(de)STISNESKEAFIDWAR	2321.1652	IPI00187353	IPI00187353	yes	yes	2,3	3.5485E-05	117.26
IGAIN(de)STISNESK	1332.6885	IPI00187353	IPI00187353	yes	yes	2	5.3424E-06	156.6
IGAIN(de)NSIIIIEDR	1539.8621	IPI00115516	IPI00115516	yes	no	2	1.3149E-26	189.09
IGACN(de)DTIK	990.48043	IPI00136642	IPI00136642	yes	yes	2	1.1512E-35	205.83
IFNIDSGN(de)GSIFTSK	1598.794	IPI00123942	IPI00123942	yes	yes	2	0.013275	88.552
IFIFN(de)QTGIEAKK	1507.8399	IPI00378224	IPI00378224	yes	yes	2	0.0016137	127.71
IFIFN(de)QTGIEAK	1379.7449	IPI00378224	IPI00378224	yes	yes	2	1.4929E-72	214
IESAMN(de)YTDFQRPICIPSKGDR	2597.2366	IPI00416285	IPI00416285	yes	yes	3	2.6256E-05	140.28
IEN(de)ITTGTYTIHAQK	1688.8733	IPI00222429	IPI00222429	yes	yes	2	0.031319	70.98
IEN(de)ISSTESGYTATITR	1841.9007	IPI00111960	IPI00111960	yes	no	2	2.4044E-202	276.4
IEKIEGIIAN(de)VSR	1440.83	IPI00115516	IPI00115516	yes	no	2	0.0090138	129.38
IEIVPIIYDN(de)DSIFVQTDK	2221.1518	IPI00405543	IPI00405543	yes	yes	2,3	4.7867E-62	216.35
IEGITN(de)ETYR	1194.5881	IPI00119299	IPI00119299	yes	no	2	5.8108E-27	196.11
IEGIIAN(de)VSR	1070.6084	IPI00115516	IPI00115516	yes	no	2	0.0064049	119.74
IEDRFN(de)STIGPSEEQEKNWPGGPGR	2799.3212	IPI00115516	IPI00115516	yes	no	3	8.2536E-09	123.55
IEDRFN(de)STIGPSEEQEK	1977.928	IPI00115516	IPI00115516	yes	no	2,3	0.0027942	140.89
IEDGFHPDAVAWAN(de)ITNAIR	2209.0916	IPI00165730	IPI00165730	yes	no	2,3	1.785E-28	187.47
IEAYFSIEGFPAEQN(de)ASNR	2142.0018	IPI00114319	IPI00114319	yes	yes	2,3	3.03E-19	182.15
IEAVIPAEFFEVISSSQN(de)GSYHHIR	2829.4086	IPI00342158	IPI00342158	yes	no	4	1.2439E-08	112.74
IDWIGN(de)CSGINDDSYGYR	2103.8956	IPI00121550	IPI00121550	yes	yes	2	5.0965E-19	182.76
IDVSQN(de)VSSDTDQSCESTK	2098.8961	IPI00120769	IPI00120769	yes	no	2	2.5852E-112	238.8
IDVNSWIEN(de)FTK	1464.7249	IPI00626793	IPI00626793	yes	no	2	2.7297E-22	190.37
IDSFN(de)DSTFMIVYTPITPTTQR	2546.2363	IPI00322575	IPI00322575	yes	no	2,3	1.5635E-06	127.49
IDRNPSPDEIPQVGN(de)ISIK	2091.096	IPI00308990	IPI00308990	yes	yes	2,3	0.0004277	149.44
IDPPCTN(de)TTAPSNYINNPYVR	2406.1274	IPI00658539	IPI00658539	yes	no	2,3	6.8836E-16	177
IDNYS(de)TQEIGR	1294.6153	IPI00122257	IPI00122257	yes	no	2	1.2812E-07	166.7
IDITDFEKN(de)SSFAQYQSFK	2267.0746	IPI00121312	IPI00121312	yes	yes	3	5.9505E-13	176.28
IDIPVNTSIPNVTEIKEN(de)MTFGSTIVTNPK	3271.701	IPI00466371	IPI01026704	no	no	3	2.8338E-13	111.3
IDIPVNTSIPN(de)VTEIKENMTFGSTIVTNPK	3271.701	IPI00466371	IPI01026704	no	no	3	2.8338E-13	111.3
IDIPVNTSIPN(de)VTEIK	1751.9669	IPI00466371	IPI01026704	no	no	2	5.7386E-26	186.77
IDIPVN(de)TSIPNVTEIKENMTFGSTIVTNPK	3271.701	IPI00466371	IPI01026704	no	no	3	2.8338E-13	111.3
IDIPVN(de)TSIPNVTEIK	1751.9669	IPI00466371	IPI01026704	no	no	2	5.7386E-26	186.77
IDGATVDTHHIPVN(de)VTIR	1957.0381	IPI00751912	IPI00751912	yes	yes	2,3	0.0062823	91.076
IDFIVINE(de)TR	1218.6608	IPI00125813	IPI00125813	yes	no	2	0.0020625	138.75
IDETDCIGN(de)WTWQEGSQQTIK	2508.1227	IPI00122973	IPI00122973	yes	no	2,3	1.0154E-115	239.75
IDCYPDEHGASEAN(de)CSAR	2050.8109	IPI00848693	IPI00848693	yes	yes	2,3	0.0002831	136.39
IDC(de)RHENNTKDNSIQHEFSITR	2713.2627	IPI00109727	IPI00109727	yes	yes	3	0.01632	79.676
IDAYNEAAVSIIN(de)SSTR	1822.9061	IPI00380296	IPI00380296	yes	yes	2	0.0006869	137.69
IDAPTNIQFVN(de)ETDRTVIVTWTPPR	2882.4927	IPI00113539	IPI00113539	yes	no	3,4	1.1569E-08	120.71
IDAPTNIQFVN(de)ETDR	1731.8428	IPI00113539	IPI00113539	yes	no	2	8.4463E-130	250.19
ICQN(de)ISKK	989.5328	IPI00126184	IPI00126184	yes	yes	2	0.042348	115.7
ICQN(de)ISK	861.43784	IPI00126184	IPI00126184	yes	yes	2	3.0671E-05	161.11
ICN(de)ECSDGSFHISK	1652.6923	IPI00515360	IPI00515360	yes	no	2	0.0005351	124.16
ICHTHGWN(de)ETSEIMPPGAVFSCIYR	2961.336	IPI00122399	IPI00122399	yes	no	3	0.0004494	85.062
ICEGDRFCIIDVMSTGSSSVGN(de)ATR	2731.2364	IPI00749655	IPI00749655	yes	no	3	1.0453E-24	190.88
IAVTN(de)TTMTGTVIK	1448.7909	IPI00108041	IPI00108041	yes	yes	2	1.2506E-08	165.49
IASPPSQGEVPPGPIPEAVIAIYN(de)STR	2759.4494	IPI00114457	IPI00114457	yes	yes	3	0.0004043	80.83
IASAVQKN(de)ATSTK	1317.7252	IPI01027808	IPI01027808	yes	no	2	0.007833	136.6
IAQEGGAAAIIIAN(de)NSVIIPSSR	2264.2488	IPI00121776	IPI00121776	yes	yes	3	0.012189	63.225
IAPASN(de)VSHTVVIRPIK	1801.0574	IPI00467180	IPI00467180	yes	yes	2	1.1171E-25	186.72
IAPASN(de)VSHTVVIR	1462.8256	IPI00467180	IPI00467180	yes	yes	2	0.0020734	113.4
IAN(de)FSQSCTIYSGDDIVEAIPKPCPGCPR	3251.505	IPI00114958	IPI00114958	yes	no	3,4	6.7938E-24	172.24
IAN(de)ETGGHGSGPR	1251.5956	IPI00309230	IPI00309230	yes	no	2	0.0012787	117.08
IAMCQNIGYN(de)VTK	1510.7272	IPI00117094	IPI00117094	yes	yes	2	2.0179E-18	178.15
IAITPAHIIFIADN(de)HTEPAAHFR	2554.3445	IPI00230324	IPI00230324	yes	no	4	0.02217	56.424
IAISFPN(de)ITSDGYPVIYPTGWSSSDNVNCRPR	3582.7202	IPI00466371	IPI01026704	no	no	3	2.7639E-19	120.42
IAIN(de)ITMYIK	1178.6733	IPI00134585	IPI00134585	yes	yes	2	0.028473	92.611
IAGGENN(de)CSGRVEIK	1602.7784	IPI00760106	IPI00760106	yes	no	2	0.0050861	132.31
IAGGEN(de)NCSGRVEIK	1602.7784	IPI00760106	IPI00760106	yes	no	2	0.0050861	132.31
IACIN(de)GTVIR	1115.6121	IPI00134808	IPI00134808	yes	no	2	8.8316E-05	162.38
I(de)VQAQYWHDPIKESVYRNYSIFIADINQER	3694.8532	IPI00331318	IPI00331318	yes	no	3,4	1.8468E-07	94.019
I(de)VIYIEHNIEKNSTKEEIIAAIEK	2796.5273	IPI00928320	IPI00928320	yes	no	2,3,4	2.876E-187	264.92

I(de)AVISGCKNTTSAAMVHCIR	2188.0915	IPI00131216	IPI00131216	yes	yes	2,3		1.9036E-13	171.71
HYGPGWVSMANAGKDTN(de)GSQFFITTVK	2912.3916	IPI00135686	IPI00135686	yes	yes		3	2.4669E-13	125.31
HYAN(de)ITNGMYR	1338.6139	IPI00224073	IPI00224073	yes	no		2	0.0046693	114.83
HVYIGDKNAIN(de)ITFHAQNIGEGGAYEAEIR	3299.6323	IPI00115976	IPI00115976	yes	yes		3	7.9991E-20	120.54
HVVIN(de)GSSFGADGFPYVQVIK	2233.1532	IPI00742377	IPI00742377	yes	no		3	1.0373E-05	118.24
HVTDMN(de)STIHIIR	1535.7878	IPI00223446	IPI00223446	yes	yes	2,3		0.0052052	106.26
HVEDKFN(de)ETTQR	1502.7114	IPI00130654	IPI00130654	yes	no		2	0.0031653	141.58
HVCPVENWSYN(de)ESCSPDPAEQGGPK	2843.1915	IPI00128269	IPI00128269	yes	yes		3	1.6244E-08	110.4
HVCPVEN(de)WSYNESCSPDPAEQGGPK	2843.1915	IPI00128269	IPI00128269	yes	yes		3	1.6244E-08	110.4
HTDDITSIN(de)NTIVNIR	1824.933	IPI00129304	IPI00129304	yes	yes	2,3		3.4505E-08	166.48
HSVPVFCHPSFYN(de)DTDFIGEEIDIVDVK	3278.523	IPI00416285	IPI00416285	yes	yes		3	0.003711	59.55
HSVIWNAMIHPIQN(de)MTIK	2132.1023	IPI00111794	IPI00111794	yes	no	2,3		0.0003847	115.31
HSQQPIITYEKYN(de)VTDTPK	2261.1328	IPI00322418	IPI00322418	yes	yes		2	1.2912E-07	161.95
HSIEHFNN(de)NTDHSHIFTIR	2318.0941	IPI00114958	IPI00114958	yes	no	2,3,4		1.055E-49	211.06
HPGNFSSISCDYFAVN(de)QSR	2184.9647	IPI00115976	IPI00115976	yes	yes	2,3		1.737E-12	169.49
HPGN(de)FSSISCDYFAVNQSR	2184.9647	IPI00115976	IPI00115976	yes	yes	2,3		1.737E-12	169.49
HNN(de)DTQHIWESDSNEFSVIADPR	2710.2008	IPI00129526	IPI00129526	yes	no	2,3,4		7.5675E-102	235.68
HN(de)QSCEWCQTIAVR	1787.7832	IPI00330843	IPI00330843	yes	yes		2	0.0016245	115.8
HN(de)ITITQGK	1010.5509	IPI00128358	IPI00128358	yes	no		2	0.0001105	148.56
HIVFWN(de)SSNPK	1327.6673	IPI00130752	IPI00130752	yes	no		2	0.0054799	111.63
HISN(de)ATSATHEHIEGPATNDIK	2342.1251	IPI00461384	IPI00461384	yes	yes		3	0.028466	56.327
HISFGEDYPGIVNPIDHTN(de)VTAPQASMMFQYFVK	3852.828	IPI00132454	IPI00132454	yes	no	3,4		2.8403E-26	120.15
HIN(de)ITTISVAGGFR	1484.81	IPI00850413	IPI00850413	yes	yes	2,3		0.0008445	137.8
HIN(de)ASNPSEPATIIFTAAR	2009.033	IPI00421157	IPI00421157	yes	yes	2,3		5.3415E-133	250.94
HIIEN(de)ATASVSEAERK	1753.8959	IPI00396840	IPI00396840	yes	no	2,3		1.3957E-60	218.12
HIIEN(de)ATASVSEAER	1625.8009	IPI00396840	IPI00396840	yes	no	2,3		8.1305E-36	197.49
HHAAYVNNIN(de)ATEEKYHEAIAK	2522.2302	IPI00109109	IPI00109109	yes	yes		3	1.72E-05	131.45
HGFYCYIIGSTISTFTDAN(de)HTCTNEK	3036.3382	IPI00126186	IPI00126186	yes	yes		3	1.0552E-13	133.74
HFKGQTQN(de)YSTIIIEEASER	2350.1553	IPI00124666	IPI00124666	yes	no		3	0.000107	144.68
HFFN(de)VSDEIAIVTIVK	1830.988	IPI00466371	IPI01026704	no	no		2	0.0086094	86.367
HF(de)IIDSKSNVTRPSEFNYIWIAPIPFIK	3331.7758	IPI00319509	IPI00319509	yes	no		3	0.0003995	84.181
HEN(de)NTKDNSIQHEFSITR	2169.0199	IPI00109727	IPI00109727	yes	yes	2,3		1.2884E-47	205.18
HEEGHMINC(de)TCFGQGR	1931.7825	IPI00113539	IPI00113539	yes	no	2,3		0.0011967	119.96
HDPN(de)MTCDYVIK	1491.6486	IPI00119299	IPI00119299	yes	no		2	0.028819	81.565
HAN(de)WTITPIK	1179.64	IPI00317356	IPI00317356	yes	yes		2	0.014806	106.62
HAN(de)ETSIGITWR	1383.6895	IPI00229935	IPI00229935	yes	yes		2	0.013619	90.614
HAEISFVFITDGVTGN(de)DSIEESVHSMR	2976.3924	IPI00621027	IPI00621027	yes	yes		3	2.0426E-13	139.69
GYN(de)SSQDIPSIVIDFVK	1880.952	IPI00124666	IPI00124666	yes	yes		2	1.3539E-47	206.62
GYITSFEMFN(de)STFK	1670.765	IPI00262024	IPI00262024	yes	no		2	2.5446E-197	271.56
GYIITINFTKN(de)TTR	1640.8886	IPI00469218	IPI00469218	yes	yes		2	7.9684E-109	239.61
GYIITIN(de)FTKNTTR	1640.8886	IPI00469218	IPI00469218	yes	yes		2	7.9684E-109	239.61
GYIITIN(de)FTK	1168.6492	IPI00469218	IPI00469218	yes	yes		2	9.2133E-28	201.84
GWIN(de)GSIVGFYK	1339.6925	IPI00134585	IPI00134585	yes	no		2	1.1877E-40	206.36
GVTSVSQIFHSPDIAIRDTYVN(de)ASQSIYGSSPR	3551.7645	IPI00122977	IPI00122977	yes	no		3	1.6256E-05	83.788
GVTNDIISVQGNTGPSWIN(de)KTEK	2457.25	IPI00344686	IPI00344686	yes	yes	2,3		2.0453E-10	159.77
GVTHIN(de)ISGIKMPR	1521.845	IPI00119063	IPI00119063	yes	yes		2	0.024878	100.11
GVTHIN(de)ISGIK	1137.6506	IPI00119063	IPI00119063	yes	yes	1,2,3		0.0012092	138.24
GVSVSSGTGIPDMTGSVYN(de)K	1954.9306	IPI00457533	IPI00457533	yes	no		2	0.027208	64.275
GVSSSEN(de)FTSPVMEFWERR	2244.027	IPI00466652	IPI00466652	yes	no		3	0.0048856	95.067
GVSSSEN(de)FTSPVMEFWER	2087.9259	IPI00466652	IPI00466652	yes	no	2,3		1.253E-36	198.85
GVQIIVFPEDGIHGFN(de)FTR	2145.1007	IPI00321375	IPI00321375	yes	no	2,3		2.0703E-21	185.85
GVNVITVN(de)ATDADSK	1502.7577	IPI00623114	IPI00623114	yes	no		2	2.4445E-26	190.82
GVN(de)VTMPSQPGVPPISSTQIQIDPAIQEFQIVDISR	3860.9982	IPI00515360	IPI00515360	yes	no		3	0.0003895	72.37
GVIPGEDWTVFQSN(de)HSTYEPVIIAK	2786.3915	IPI00404189	IPI00404189	yes	no		2	0.0002406	87.802
GVIMVGNETTYEDGHGARKN(de)ITDIVEGAK	3073.5139	IPI00649186	IPI00649186	yes	no		3	0.0014248	80.835
GVIMVGN(de)ETTYEDGHGARKNITDIVEGAK	3073.5139	IPI00649186	IPI00649186	yes	no		3	0.0014248	80.835
GVIMVGN(de)ETTYEDGHGAR	1904.8687	IPI00649186	IPI00649186	yes	no	2,3		0.0006603	117.18
GVFITNE(de)TGQPIIGK	1572.8512	IPI00111960	IPI00111960	yes	no		2	2.8014E-75	224.16
GVDSCHGDSGGAFAFQVPN(de)VTVPK	2445.1383	IPI00653675	IPI00653675	yes	no		3	0.0090145	63.225
GVAVTN(de)TSQIGFR	1348.7099	IPI00471089	IPI00471089	yes	no		2	1.0606E-09	166.62
GTPSHEIN(de)TTSSGAEVIQK	1954.9596	IPI00849185	IPI00849185	yes	no		2	0.0005739	120.11
GTFTDCAIANM(de)TQQIR	1825.8451	IPI00621027	IPI00621027	yes	no		2	1.7061E-106	199.58
GTDPSISPSPAATPPAPGAEISYIN(de)HTFR	2950.4461	IPI00762091	IPI00762091	yes	yes		3	2.4756E-09	102.21
GTCEQGPSIVTPPKDIWN(de)VTGAK	2454.2213	IPI00652107	IPI00652107	yes	no		3	0.0015375	89.557
GTAGNAIMDGASQIVGEN(de)R	1859.8796	IPI00279079	IPI00279079	yes	yes	2,3		5.6375E-232	284.5
GSYEIIAANGEDTDVDKITNSPQNQN(de)ITVPR	3358.6277	IPI00406603	IPI00406603	yes	yes		3	3.0726E-19	148.87
GSTQYIENIGFN(de)MSK	1687.7876	IPI00116105	IPI00116105	yes	yes		2	0.0013827	140.8
GSSVIIN(de)CSAYSEPSPNIEWK	2337.0947	IPI00129159	IPI00129159	yes	no		2	3.3639E-15	175.65
GSPGN(de)ASQGSIHIHSPQIAIQADPR	2537.2735	IPI00114044	IPI00114044	yes	yes	2,3		6.4424E-09	116.43
GSNFN(de)ISKTDNIEECQK	1982.9004	IPI00113057	IPI00113057	yes	yes	2,3		0.0001228	155.57
GSNFN(de)ISK	865.42938	IPI00113057	IPI00113057	yes	yes		2	0.0038848	150.27
GSN(de)FTAICVIK	1208.6223	IPI00120155	IPI00120155	yes	yes		2	0.0026581	102.97
GSIQSANDISSQTQGFIQHSMDN(de)ISAQIQTVR	3460.6641	IPI00330594	IPI00330594	yes	yes	3,4		7.1253E-20	135.34
GSIN(de)VSYNIGPGFTGSEYSR	2103.9861	IPI00113042	IPI00113042	yes	no		2	1.3979E-05	120.23
GSEVEDEDIEIFN(de)TSVQIRPPSTAPGPETAAFIER	3800.8381	IPI00381357	IPI00381357	yes	no	3,4		4.882E-27	132.78
GSAIPHPSN(de)ATWEIK	1606.8104	IPI00119299	IPI00119299	yes	no		2	6.7403E-08	162.79
GRADECAIPYIGATCYCDIFCN(de)R	2781.1768	IPI00115458	IPI00115458	yes	no		3	9.1688E-05	100.02
GQTQNYST(de)IIIEEASER	1937.933	IPI00124666	IPI00124666	yes	no	2,3		9.6957E-61	215.51
GQTGIGFIYASGIGVN(de)SSQAK	2054.0433	IPI00131143	IPI00131143	yes	no		2	4.3863E-51	205.78
GPDISN(de)CTSHWVNQIAQK	2053.964	IPI00876558	IPI00876558	yes	no		2	0.0004878	112.3
GPDGTEGISSTVN(de)GSTDPSAVTDIR	2432.1303	IPI00469426	IPI00469426	yes	no	2,3		3.834E-09	145.81
GNYGWQAGN(de)HSAFWGMTIDEGIR	2566.1448	IPI00115458	IPI00115458	yes	no	2,3		7.4794E-09	132.14
GNYDFVEAMIVNN(de)HTSIDVER	2422.1223	IPI00403586	IPI00403586	yes	yes	2,3		6.4354E-06	148.01
GN(de)TTAIDKEIAR	1287.6783	IPI00108535	IPI00108535	no	no		2	9.0551E-07	160.18
GN(de)QSSQWIYEAMAK	1611.7351	IPI00137792	IPI00137792	yes	yes		2	0.0026227	112.96
GN(de)ITEYQCHQYITK	1753.8094	IPI00122399	IPI00122399	yes	no		2	4.8122E-27	192.46

GN(de)ISTEKFVEEIK	1492.7773	IPI00466371	IPI01026704	no	no		2	7.0714E-13	172.57
GN(de)HSIFGIEVATIGQGPDCPSVNER	2653.2555	IPI00459432	IPI00459432	yes	yes		3	1.12E-09	125.31
GN(de)GSCVCHAGWQGIR	1657.7202	IPI00987265	IPI00987265	yes	no		2	0.0010029	130.24
GMNYN(de)SSVVK	1097.5175	IPI00416285	IPI00416285	yes	yes		2	0.015607	110.84
GKIYWTDGDNISMANMDG(de)SNHTIIFSGQK	3199.4703	IPI00119063	IPI00119063	yes	yes		3	8.31E-14	131.27
GKIYWTDG(de)DNISMANMDGSNHTIIFSGQK	3199.4703	IPI00119063	IPI00119063	yes	yes		3	8.31E-14	131.27
GIWKIPFSPENTREEDFYVN(de)ETSTVK	3085.5033	IPI00116105	IPI00116105	yes	yes		3	0.022183	60.464
GIVSGGVYNSHVGCIPYTIPPCEHHVN(de)GSRPPCTGEGDTPR	4430.0379	IPI00113517	IPI00113517	yes	yes		4	0.0025411	48.774
GISYQIIGN(de)HSK	1315.6884	IPI00623114	IPI00623114	yes	no		2	0.0001135	134.56
GINIT(de)EDTYKPR	1405.7201	IPI00119809	IPI00119809	yes	yes		2	8.178E-53	219.43
GIMVGN(de)GSVIGVVQAVDAETGK	2100.0885	IPI00342158	IPI00342158	yes	no	2,3		0	337.87
GIMIIIN(de)DTQHFSNNVKGEIGQFYR	2893.4545	IPI00312711	IPI00312711	yes	no		3	0.000161	92.993
GIMIIIN(de)DTQHFSNNVK	1942.9935	IPI00312711	IPI00312711	yes	no	2,3		1.0472E-131	252.82
GIIN(de)ATISVAEINHPVTTYK	2140.1528	IPI00130573	IPI00130573	yes	yes	2,3		1.2491E-28	189.55
GIIGISN(de)ATIYWHIPDTAYPGIYR	2690.3857	IPI00458077	IPI00458077	yes	yes		3	4.6181E-10	153.11
GIFPDGSHEISGN(de)TSITPDK	2070.9858	IPI00894972	IPI00894972	yes	no		2	0.0025636	87.49
GIAN(de)ISNFIR	1103.6087	IPI00129158	IPI00129158	no	no		2	0.0020419	146.11
GHVDPAN(de)DTFDIDPR	1667.754	IPI00453501	IPI00453501	yes	no	2,3		0.020655	77.527
GHTDRCDEAQAIQVWN(de)DTHPEVISQKPFDK	3520.643	IPI00108003	IPI00108003	yes	no		4	0.0034651	74.819
GHIIIAQVATNPQGITGTGN(de)TTSEMDPSHRK	3230.6102	IPI00321375	IPI00321375	yes	no		3	1.7183E-19	122.34
GHIIIAQVATNPQGITGTGN(de)TTSEMDPSHR	3102.5153	IPI00321375	IPI00321375	yes	no		3	3.69E-46	189.51
GHICN(de)QTQNIQSSK	1613.758	IPI00987886	IPI00987886	yes	yes		2	0.0003285	125.97
GHFYYN(de)ISDVR	1369.6415	IPI00135560	IPI00135560	yes	no		2	0.0050337	113.38
GHAIGISCQSEN(de)GTAPITYHIMK	2484.189	IPI00406901	IPI00406901	yes	no		3	1.4718E-08	146.78
GHAHIAAIVNHDSYN(de)FSHR	2145.0253	IPI00133365	IPI00133365	yes	no	2,3		2.3277E-08	164.2
GGIN(de)ITAVTVTAENDHTVAFIGTSDGR	2715.3464	IPI00405742	IPI00405742	yes	no		3	0.0012932	70.699
GFTQIQTIIIPQDVPCPGGSNAWDN(de)VTSFKDK	3532.7297	IPI00471273	IPI00471273	yes	no		3	3.0888E-05	88.541
GFQWVTGDN(de)HTSYSR	1753.7808	IPI00129423	IPI00129423	yes	yes		2	5.0925E-06	159.18
GFN(de)ATYHVR	1063.5199	IPI00411025	IPI00411025	yes	yes		2	0.014574	118.18
GFGVAFVGN(de)YTGSIPNEAAINTVR	2453.2339	IPI00165807	IPI00165807	yes	no		3	5.5728E-05	95.5
GFDTYFGYIIGSEDYYTHEACAPIESIN(de)GTR	3545.5722	IPI00652358	IPI00652358	yes	no		3	7.7033E-22	156.88
GENPSQYGITAFNHPIN(de)ITK	2200.0913	IPI00112614	IPI00112614	yes	yes		2	3.0599E-05	144.17
GEIQSEN(de)SSITISSSNR	1807.8548	IPI00466371	IPI01026704	no	no	2,3		3.279E-258	289.32
GEIN(de)STIFSSRPK	1434.7467	IPI00460063	IPI00460063	yes	no		2	0.0013386	147.73
GECYYTN(de)GTQR	1347.5514	IPI00110910	IPI00110910	yes	no		2	0.0005585	144.1
GDTHTQIIEGIQFN(de)ITQTSEADIHK	2795.3726	IPI00406302	IPI00406302	yes	no	3,4		1.0485E-84	221.14
GDSGGAIVFIDN(de)ETQR	1677.7958	IPI00467068	IPI00467068	yes	no		2	0.027657	71.08
GDPSIISVN(de)GTDFTFR	1724.837	IPI00463492	IPI00463492	yes	yes		2	4.4879E-05	152.94
GDGPFTVFVPHADIISN(de)MSQDEIAR	2715.2963	IPI00987265	IPI00987265	yes	no		3	5.9581E-09	117.38
GDEKENITA(de)EAIDISIK	1844.9367	IPI00124725	IPI00124725	yes	no	2,3		0.0009502	145.52
GCKDNA(de)TDSVPIR	1431.6776	IPI00119063	IPI00119063	yes	yes		2	3.0438E-13	175.29
GCADYCNQ(de)TITKR	1585.6977	IPI00987265	IPI00987265	yes	no		2	0.0010977	143.25
GAIQIPTVSFSHEESN(de)TTAIAEFGEYIRK	3194.5884	IPI00224073	IPI00224073	yes	yes		3	0.0004819	80.786
GAIQIPTVSFSHEESN(de)TTAIAEFGEYIR	3066.4934	IPI00224073	IPI00224073	yes	yes		3	4.4651E-14	128.29
GAIDIMIQVN(de)MTPGHSSAPPK	2163.0816	IPI00224073	IPI00224073	yes	yes	2,3		7.3449E-06	116.39
GAGEVSPAEHSSKPTN(de)ISAK	1965.9756	IPI00230718	IPI00230718	yes	no		3	0.023259	80.738
GAFVSKNPCN(de)ITREDYAPIVK	2378.2053	IPI00307966	IPI00307966	yes	yes	2,3		2.2232E-05	149.36
GAFVSKNPCN(de)ITR	1462.7351	IPI00307966	IPI00307966	yes	yes		2	0.027034	98.592
GAFISN(de)FTMTVNGMTFTSSIK	2253.081	IPI00970438	IPI00970438	yes	no		2	0.017047	71.756
GAFFPIKEDN(de)WSIPNR	1889.9424	IPI00135560	IPI00135560	yes	no		2	0.0051928	115.56
GAAAPSAPHWN(de)ETAEK	1635.7641	IPI00648288	IPI00648288	yes	no		2	0.0002776	126.16
FVPNSNMN(de)FTGQAYSGR	1888.8526	IPI00108535	IPI00108535	no	no	2,3		7.6966E-10	154.39
FVHVN(de)TSAIIK	1227.6976	IPI00153632	IPI00153632	yes	yes		2	0.0064223	108.35
FTFTSHTPGEHQICIHSN(de)STK	2428.123	IPI00473680	IPI00473680	yes	no	2,3		6.5865E-15	174.02
FTFTSHTPGDHQICIHSN(de)STR	2442.1135	IPI00153468	IPI00153468	yes	no	2,3		6.4857E-51	203.06
FTCN(de)QTTDVIIIHSK	1775.8876	IPI00319509	IPI00319509	yes	yes	2,3		5.3099E-151	259.96
FTAPDTIFAN(de)GSVYPPNEGFCPCR	2716.205	IPI01008227	IPI01008227	yes	no	2,3		7.2406E-09	139.28
FSWNNITNSIDIANISADFQGRPVDDPTGAFAN(de)GSITFK	4200.0189	IPI00459432	IPI00459432	yes	yes	3,4		0.0011547	65.898
FSWNNITNSIDIAN(de)ISADFQGRPVDDPTGAFANGSITFK	4200.0189	IPI00459432	IPI00459432	yes	yes	3,4		0.0011547	65.898
FSWNN(de)ITNSIDIANISADFQGRPVDDPTGAFANGSITFK	4200.0189	IPI00459432	IPI00459432	yes	yes	3,4		0.0011547	65.898
FSVN(de)QTTIITHEK	1516.7886	IPI00848693	IPI00848693	yes	yes		2	2.0117E-09	164.64
FSTEIGYN(de)GTR	1243.5833	IPI00134013	IPI00134013	yes	yes		2	6.8173E-10	173.72
FSPPVVN(de)VTWIR	1413.7769	IPI00110808	IPI00110808	yes	no		2	0.013382	90.861
FSMSDTYDIQDVIADVGIKDIFTN(de)QSDFADTTKDTPITITVIHK	4918.411	IPI00116105	IPI00116105	yes	yes		4	0.0022952	46.449
FSMSDTYDIQDVIADVGIKDIFTN(de)QSDFADTTK	3699.7138	IPI00116105	IPI00116105	yes	no		3	6.504E-38	180.21
FSHN(de)GTCAAEGK	1277.5459	IPI00320675	IPI00320675	yes	yes		2	1.1512E-07	162.31
FSEIIVNN(de)ATEEIIVK	1817.9775	IPI00928320	IPI00928320	yes	no		2	0.0011823	118.21
FSEIATVHN(de)HSIPK	1578.8154	IPI00124697	IPI00124697	yes	no		2	0.0022549	112.42
FSDGIESN(de)R	1023.4621	IPI00131091	IPI00131091	yes	no		2	0.0054052	132.01
FSATEVPEKGAGEVSP(de)AEHSSKPTNISAK	2954.4621	IPI00230718	IPI00230718	yes	no	3,4		1.1763E-13	135.62
FQN(de)FSMATDQR	1343.5928	IPI00128178	IPI00128178	yes	no		2	1.656E-05	158.42
FQISPQIQFSPEEVIGMVIN(de)YSR	2681.3523	IPI00123342	IPI00123342	yes	no	2,3		5.7007E-55	203.71
FQIIN(de)FSSSEIK	1411.7347	IPI00856723	IPI00856723	yes	no		2	4.2563E-16	183.89
FPVPFQKENV(de)TATIVEIGR	2144.163	IPI00134691	IPI00134691	yes	yes	2,3		3.318E-05	157.57
FNSTE(de)YQVVTR	1342.6517	IPI00119063	IPI00119063	yes	no	2,3		5.1371E-05	152.4
FNGGGHIN(de)HTIFWTNISPK	2139.065	IPI00109109	IPI00109109	yes	yes	2,3		0.0002592	118.55
FN(de)VSIIYGR	1067.5764	IPI00320675	IPI00320675	yes	yes		2	0.0024384	141.95
FN(de)ITETPEADIHQGFGNIIQSISQPEDQDQINIGNAMFIEK	4601.202	IPI00131830	IPI00131830	yes	no	3,5		6.8988E-05	56.231
FN(de)GSVSFFR	1059.5138	IPI00551169	IPI00551169	yes	no		2	0.0005422	159.83
FN(de)ETIHR	915.45626	IPI00471081	IPI00471081	yes	yes		2	0.027465	123.72
FN(de)DTEVIQR	1120.5513	IPI00321375	IPI00321375	yes	no		2	0.011233	108.47
FMGIPTKDDNIEHYKN(de)STVMAR	2566.2308	IPI00123925	IPI00123925	yes	no		3	0.0012483	94.057
FKDNTPNAN(de)ATER	1476.6957	IPI00125138	IPI00125138	yes	no		2	0.010645	110.38
FIWSEPQN(de)CSATK	1566.7137	IPI00118987	IPI00118987	yes	no		2	0.0011112	118.37
FIVTQVPVHGHIIFN(de)NTR	2091.1378	IPI00553703	IPI00553703	yes	yes		3	0.024387	70.529
FITDVERN(de)ETAIYHVEAFK	2281.1379	IPI00466733	IPI00466733	yes	no		3	0.0003293	130.29

FIQSAEFFN(de)YTVR	1620.7936	IPI00127406	IPI00127406	yes	yes		2	7.5949E-26	192.39
FIQ(de)KHINASNPSEPATIIFTAAR	2525.3391	IPI00421157	IPI00421157	yes	yes		3	0.021711	71.735
FINYN(de)QTVSR	1240.62	IPI00226932	IPI00226932	yes	no		2	4.7586E-62	224.58
FIN(de)ISSTGIR	1106.6084	IPI00131898	IPI00131898	yes	yes		2	0.008217	117.09
FIEYVTSECHFYN(de)GTQHVR	2386.0801	IPI00474580	IPI00474580	yes	no		3	6.5334E-05	150.76
FIEDKIRQFVDICSMSN(de)VSVFIISHR	3139.5947	IPI00309741	IPI00309741	yes	yes		3	0.011243	61.616
FIEAGIYEVPIIITDSGNPPKSN(de)ISIIR	3055.6594	IPI00323134	IPI00323134	yes	no	2,3,4		3.083E-11	115.96
FHVNY(de)TQPIVAVK	1514.8245	IPI00123704	IPI00123704	yes	no		2	0.01654	86.944
FHVHQPVTQPFIQVTN(de)TTVK	2320.2328	IPI00658311	IPI00658311	yes	no	2,3,4		3.9539E-114	242.1
FHSDINI(de)SESIIPAVIEK	2011.0626	IPI00831484	IPI00831484	yes	yes	2,3		0.0005078	145.48
FGYIIHTDN(de)R	1234.6095	IPI00475209	IPI00475209	yes	no		2	0.006447	119.68
FGTCSQICN(de)NTK	1428.6126	IPI00119063	IPI00119063	yes	yes		2	4.1508E-66	225.33
FGN(de)ETFIIHIDNGR	1631.8056	IPI00170355	IPI00170355	yes	yes		2	0.0018109	118.13
FGHSPIIN(de)ITGGISHASDVVIYHQHK	2826.4565	IPI00119063	IPI00119063	yes	yes	3,4,5		1.6582E-06	96.848
FGEFGN(de)YSIIVQHASSGANK	2125.0229	IPI00317488	IPI00317488	yes	no	2,3		9.29E-21	183.06
FFQAFTKN(de)GSGATIPGAGANVQTIR	2552.3136	IPI00223231	IPI00223231	yes	no		3	0.028732	60.635
FFN(de)FSWR	1002.4712	IPI00396840	IPI00396840	yes	no		2	0.028311	130.27
FFN(de)DSIVDPVDSEWFGFYR	2339.0535	IPI00331318	IPI00331318	yes	no		2	6.8032E-05	150.69
FDGEPCDQSIN(de)ITWFIK	2068.9564	IPI00625262	IPI00625262	yes	no		2	0.0048333	94.882
FDEFFSQGCAPGYEKN(de)STICDICIGPIK	3252.4566	IPI00139788	IPI00139788	yes	no	2,3,4		7.0446E-30	178.17
FDAAGTSVHGGVPQN(de)GSICAHIPMIK	2663.2948	IPI00315535	IPI00315535	yes	yes		3	8.0727E-15	156.29
FCNIVPTEHCFIGN(de)GTEYR	2313.0307	IPI00227857	IPI00227857	yes	yes	2,3		0.0008141	102.98
FCIIDVMSTGSSSVGN(de)ATR	2000.9296	IPI00749655	IPI00749655	yes	no		2	1.1302E-93	231.86
FAVESIVPSSISIMHSPPDAQN(de)MSEVSISPMEISTFR	4019.9319	IPI00114044	IPI00114044	yes	yes	3,4		0.0001549	62.833
FAQPQWHIIHINGTFSN(de)ETEPDTEPCVDGWVYDR	4057.833	IPI00226346	IPI00226346	yes	no		3	2.315E-21	115.66
FAQPQWHIIHIN(de)GTFSNETEPDTEPCVDGWVYDR	4057.833	IPI00226346	IPI00226346	yes	no		3	2.315E-21	115.66
FAPPEAPEPWSG(de)VRDGTSQPAICPQNVTMNMEGIK	3810.7804	IPI00320204	IPI00320204	yes	yes		4	0.0013373	58.47
FAIKNEDSNFTIINNHDNTAN(de)ITVK	2832.4042	IPI00138180	IPI00138180	yes	yes		3	1.976E-08	137.72
FAIKNEDSN(de)FTIINNHDNTANITVK	2832.4042	IPI00138180	IPI00138180	yes	yes		3	1.976E-08	137.72
FAIIKDYR(de)PSAGNNSIYQDTVVFK	2745.4126	IPI00322463	IPI00322463	yes	no		3	0.01262	74.776
FAEIN(de)GSAICSYNIKPSEYTITSK	2692.3054	IPI00421237	IPI00421237	yes	no		3	0.0004542	91.637
F(de)IFKNQTIEIHVIYGPR	2074.1364	IPI00122973	IPI00122973	yes	no	2,3,4		6.6779E-36	200.69
F(de)APPQPAEPWSFVKNTTSYPPMCSQDAVGGQVISEIFTNR	4453.1147	IPI00387289	IPI00387289	yes	yes		4	0.0067457	47.156
EYHTIDDHCVYN(de)STHIGIQR	2457.1132	IPI00127856	IPI00127856	yes	yes		3	0.0063366	80.638
EYEGAVYPDN(de)TTDFQR	1903.8224	IPI00605187	IPI00605187	yes	no		2	0.0003883	130.27
EWN(de)GTYHCIFR	1481.651	IPI00876541	IPI00876541	yes	no		2	0.001132	127.42
EVNSQIDNNGCSTQEVN(de)ITEIQSKK	2834.3352	IPI00123223	IPI00123223	yes	yes		3	3.9464E-08	109.22
EVNSQIDNNGCSTQEVN(de)ITEIQSK	2706.2403	IPI00123223	IPI00123223	yes	yes	2,3		9.3961E-102	234.36
EVNSKIDNNGCSTQEVN(de)ITEIQSK	2706.2767	IPI00271262	IPI00271262	yes	yes		2	0.0010561	79.69
EVN(de)KTCEAIIFK	1450.749	IPI00115116	IPI00115116	yes	yes		2	4.5989E-10	169.52
EVN(de)ITVIDRK	1185.6717	IPI00111385	IPI00111385	yes	no		2	0.022222	105.46
EVMN(de)FTAENIYK	1457.6861	IPI00170006	IPI00170006	yes	no		2	0.022516	85.522
EVHFFVN(de)ASDVDSVK	1691.8155	IPI00310049	IPI00310049	yes	yes		2	2.9037E-05	153.79
ETYIETN(de)YTIK	1373.6715	IPI00120155	IPI00120155	yes	yes		2	0.024778	84.507
ETIVTIFDN(de)R	1206.6245	IPI00127238	IPI00127238	yes	no		2	0.0003881	159.09
ETIEQISEFN(de)DSIK	1651.7941	IPI00173156	IPI00173156	yes	no		2	0.0047709	102.62
ESSEHPWK(de)WTDNTEYNNTIPIRGEER	3187.4595	IPI00276430	IPI00276430	yes	yes		3	1.0887E-29	182.15
ESQTIGDQCVYNSTHIGFQREN(de)GTFSK	3102.4101	IPI00118130	IPI00118130	yes	yes		3	2.7894E-13	125.45
ESQTIGDQCVYN(de)STHIGFQRENGTFSK	3102.4101	IPI00118130	IPI00118130	yes	yes		3	2.7894E-13	125.45
ESQTIGDQCVYN(de)STHIGFQR	2339.0601	IPI00118130	IPI00118130	yes	yes		2	2.9893E-156	254.82
ESQIN(de)ITVMAKPTNWIEGTR	2287.1631	IPI00311405	IPI00311405	yes	yes		3	0.0060263	93.226
ESNPITAQQTT(de)KIDAPTNIQFVNETDR	3030.4894	IPI00113539	IPI00113539	yes	no		3	3.7602E-13	123.64
ESN(de)STSITQAAIIEK	1590.8101	IPI00115516	IPI00115516	yes	no		2	1.2454E-08	167.71
ESIIPVTIQCN(de)ITSSSHTIMYSYWTR	3086.4841	IPI00757771	IPI00757771	yes	no		3	0.025235	50.752
ESGIQN(de)VSTCR	1249.5721	IPI01008227	IPI01008227	yes	no		2	8.2256E-07	164.9
ERVEN(de)TSQPGEMQVTIQNIMPATVYIFK	3222.6053	IPI00129159	IPI00129159	yes	no		3	1.9641E-08	105.32
ERTEVIIQGTVSPN(de)ASAPDAVWEDYEFK	3150.5146	IPI00405437	IPI00405437	yes	no		3	0.011368	69.248
EREDVQAIN(de)ISVPYGPIPEDFQR	2671.3242	IPI01023131	IPI01023131	yes	no		3	6.3545E-08	144.57
EQYIHEN(de)YSR	1337.6	IPI00127406	IPI00127406	yes	no		2	1.6602E-48	214.82
EQSFQGSN(de)MTIFYR	1706.7723	IPI00453571	IPI00453571	yes	no		2	0.0071377	98.582
EQNYTCHVYHEGI(de)PEPITIR	2455.159	IPI01026927	IPI01026927	yes	no	2,3		1.6939E-78	224.4
EQN(de)YTCHVHHK	1451.6364	IPI00850057	IPI00850057	yes	no		2	0.0014005	120.06
EQIN(de)ISIDHR	1223.6258	IPI00127407	IPI00127407	yes	no		2	8.3588E-77	230.41
EQGPSIVTPPKDIWN(de)VTGAK	2136.1215	IPI00929820	IPI00929820	yes	yes		2	7.4517E-05	113.86
EQESMMN(de)ITIHIVK	1671.8324	IPI00675799	IPI00675799	yes	no		2	0.0086373	89.231
ENYAEIIDDGFIKN(de)ITAQICIDKK	2810.416	IPI00339885	IPI00339885	yes	no		3	1.2051E-32	187.73
ENS(de)SYQVINWR	1394.6579	IPI00153316	IPI00153316	yes	yes		2	3.419E-07	167.2
ENQN(de)HSYSIK	1218.5629	IPI00120245	IPI00120245	yes	no		2	0.015335	104.82
ENPGFDFSGAEISGN(de)YTK	1931.8537	IPI00461281	IPI00461281	yes	no		2	0.010206	82.265
ENPEQSRVT(de)INNWVANKTEGR	2441.2047	IPI00136642	IPI00136642	yes	yes		3	0.009639	85.51
ENPEQ(de)SRVTINNWVANKTEGR	2441.2047	IPI00136642	IPI00136642	yes	yes		3	0.009639	85.51
ENN(de)VTEIIITGIFQDPNVQK	2271.1747	IPI00127461	IPI00127461	yes	yes		3	0.017685	61.962
ENIIDISNAN(de)R	1257.6313	IPI00968968	IPI01008326	no	no		2	2.2841E-06	160.22
ENIFGIQGAGGFQDREEEYYAEPGVTEAEPVATEDAN(de)STDSII	4945.269	IPI00378224	IPI00378224	yes	yes		4	1.075E-06	78.312
ENGVD(de)AVHPGYGFISER	1845.8646	IPI00943457	IPI00943457	yes	no		2	0.025728	64.507
ENGTFSKYEGGVETFAHIIVIR(de)K	2594.3493	IPI00118130	IPI00118130	yes	yes		3	0.014426	76.704
EN(de)VSDPSITITFGR	1534.7627	IPI00469218	IPI00469218	yes	yes		2	2.0183E-60	214.47
EN(de)NSPAIHIGTISATDSDSGSNAHITYSIR	3127.4806	IPI00469537	IPI00469537	yes	yes		3	0.0005953	67.217
EN(de)ISDPTSPIR	1227.6095	IPI00136925	IPI00136925	yes	no		2	0.0016699	124.21
EN(de)GTFSKYEGGVETFAHIIVIR	2466.2543	IPI00118130	IPI00118130	yes	yes	2,3		5.5999E-97	233.63
EMIHN(de)ATFCIVPR	1586.7698	IPI00125530	IPI00125530	yes	no		2	0.018275	113.99
EMGNT(de)VMDIIRNYTANATSSREEAWDYVQAQVK	3789.7727	IPI00108098	IPI00108098	yes	no	3,4		0.0076039	57.339
EKVEN(de)GSETGPIPPEIQPIIEGEVK	2688.3858	IPI00136012	IPI00136012	yes	yes	2,3		4.5406E-09	150.14
EKSCVAITDAFPQN(de)MSR	1952.9084	IPI00405742	IPI00405742	yes	no		3	0.028338	91.657
EKEDRPFHQAV(de)VNDTQAFWHNK	2695.2891	IPI00406901	IPI00406901	yes	no		3	1.5505E-05	120.11
EKDVIWFRPTIINDTGN(de)YTCMIR	2841.3942	IPI00929886	IPI00929886	yes	no		3	3.7608E-09	151.96

EKDVIWFRPTIIN(de)DTGNYTCMIR	2841.3942	IPI00929886	IPI00929886	yes	no		3	3.7608E-09	151.96
EIVHQSN(de)TSNIFFSPVSIATAFAMISIGSK	3195.6274	IPI00406302	IPI00406302	yes	no		3	1.2043E-21	156.7
EITGIRN(de)ISSQEGSTDVVAR	2131.0869	IPI00677395	IPI00677395	yes	no	2,3		2.9021E-63	218.14
EITDTGKNAVN(de)CTYKNEDDCVVR	2700.2119	IPI00266264	IPI00266264	yes	yes		3	7.2603E-08	140.35
EIN(de)ISICPVSQTSER	1731.8461	IPI00381303	IPI00381303	yes	yes		2	5.3663E-110	240.92
EIKGDGPFTVFVPHADI(de)ISNMSQDEIAR	3085.5179	IPI00987265	IPI00987265	yes	no		3	2.5863E-08	106.1
EIIYSN(de)GSIIFQMITMK	1987.0159	IPI00108535	IPI00108535	no	no	2,3		3.3275E-153	258.92
EIIVHPN(de)YTR	1240.6564	IPI00113750	IPI00113750	yes	yes		2	0.0018788	148.56
EIIVDEIISN(de)SSTIASYK	1981.0256	IPI00173181	IPI00173181	yes	no		2	0.0004525	119.76
EIISN(de)ASDAIDKIR	1543.8206	IPI00129526	IPI00129526	no	no		2	7.939E-173	267.93
EIHHIQEQN(de)VSNVFIDKEEFFIGSK	2986.4825	IPI00605187	IPI00605187	yes	no	3,4		5.0585E-32	181.61
EIGVVMYN(de)CSCIAR	1670.7579	IPI00130624	IPI00130624	yes	yes		2	0.021038	85.45
EIGAIIYN(de)CSNIAQDIEK	2050.0041	IPI00221418	IPI00221418	yes	no		2	2.3987E-261	295.79
EIEYYVGQSFANAMYN(de)ACR	2284.9881	IPI00132600	IPI00132600	yes	yes		3	0.0003031	112.67
EHSIFIDIHPVTGIPMN(de)CSVK	2393.1872	IPI01008227	IPI01008227	yes	no	2,3		4.3065E-06	128.79
EGTYSIPTSN(de)FTVPDIEIPSWISTGNYR	3143.5088	IPI00119095	IPI00119095	yes	yes	2,3		5.6981E-09	106.1
EGSRTDDEVVQREEEAIQIDGIN(de)ASQIR	3156.5283	IPI00129526	IPI00129526	yes	yes	3,4		7.2319E-30	179.17
EGPRNISTCFSSGDIF(de)AAHNISER	2664.2351	IPI00123428	IPI00123428	yes	no		3	4.0253E-140	247.92
EGN(de)CSAQSGIAWQDCDFKDAEEAATGECTATVGKR	3818.6207	IPI00114958	IPI00114958	yes	no		3	1.7229E-14	105.21
EGN(de)CSAQSGIAWQDCDFKDAEEAATGECTATVGK	3662.5196	IPI00114958	IPI00114958	yes	no		3	2.952E-38	173.11
EGN(de)CSAQSGIAWQDCDFK	2071.8364	IPI00114958	IPI00114958	yes	no		2	2.1761E-148	185.33
EGKFDEVYDAIAGAHPN(de)ITVYKK	2564.2911	IPI00111163	IPI00111163	yes	no		3	1.0059E-07	116.6
EGKFDEVYDAIAGAHPN(de)ITVYK	2436.1961	IPI00111163	IPI00111163	yes	no	2,3		1.0157E-06	154.42
EFVNIYSDYIIN(de)K	1616.8086	IPI01008188	IPI01008188	yes	no		2	0.01626	79.492
EFVENSECIQCHPECIPQAMN(de)ITCTGR	3279.3876	IPI00121190	IPI00121190	yes	no	3,4		2.3811E-13	120.25
EFQHN(de)STMYYK	1446.6238	IPI00788443	IPI00788443	yes	no		2	0.0008005	143
EETIIYDN(de)ATSSVADRK	1910.9222	IPI00223987	IPI00223987	yes	yes		2	0.022935	78.078
EEQFN(de)STFR	1156.5149	IPI00475246	IPI00475246	yes	no		2	0.0009509	145.25
EEN(de)ATIATYPEFGVIDIK	2008.9993	IPI00124221	IPI00124221	yes	no		2	1.2107E-48	209.25
EEEAIQIDGIN(de)ASQIR	1784.8905	IPI00129526	IPI00129526	yes	yes		2	0.0052099	91.657
EDYN(de)STIR	996.45124	IPI00556788	IPI00556788	yes	no		2	0.024111	121.09
EDVQAINIS(de)VPYGPIPEDFQR	2386.1805	IPI01023131	IPI01023131	yes	no		3	0.034685	47.088
EDTVTITCEGTHNPGN(de)SSTQWFHNGR	2944.2794	IPI00129485	IPI00129485	yes	no		3	3.2153E-13	130.89
EDSSRAVN(de)VSAIPANIQK	1897.9858	IPI00354300	IPI00354300	yes	no		2	0.018528	89.484
EDSCQINY(de)SEGPCIGMQER	2271.9195	IPI00127352	IPI00127352	yes	yes	2,3		2.9506E-62	218.14
EDRPFHQAVVN(de)DTQAFWHNK	2438.1516	IPI00406901	IPI00406901	yes	no		3	2.1459E-13	169.61
EDIIA(de)IRQNFSNITVSTEDQVK	2519.2867	IPI00129677	IPI00129677	yes	no		2	1.4359E-39	201.52
ED(de)IIAIRQNFSNITVSTEDQVK	2519.2867	IPI00129677	IPI00129677	yes	no		2	1.4359E-39	201.52
ECSPPKAN(de)ITSDEI	1559.7137	IPI00466733	IPI00466733	yes	yes		2	0.001625	125.03
ECIGN(de)CSEPDDPTK	1620.6396	IPI00128358	IPI00128358	yes	no		2	9.1144E-09	165.86
ECHTIQN(de)YTIWR	1619.7515	IPI00125220	IPI00125220	yes	no	2,3		8.1398E-210	280.21
EAYKN(de)ISIIYSQMQK	1814.9237	IPI00221706	IPI00221706	yes	no	2,3		0.0038737	129.38
EATIVGEN(de)ETYPR	1477.7049	IPI00131143	IPI00131143	yes	no		2	0.0012631	128.54
EASQN(de)ITYICR	1353.6347	IPI00121120	IPI00121120	yes	yes		2	0.0069216	107.06
EASNHSSGAGIVQIN(de)KSNDKETVVGR	2696.3478	IPI00108844	IPI00108844	yes	no		3	2.1082E-12	119.38
EASNHSSGAGIVQIN(de)K	1610.8012	IPI00108844	IPI00108844	yes	no		2	1.3378E-92	235.88
EASN(de)HSSGAGIVQINKSNDKETVVGR	2696.3478	IPI00108844	IPI00108844	yes	no		3	2.1082E-12	119.38
EASN(de)HSSGAGIVQINK	1610.8012	IPI00108844	IPI00108844	yes	no		2	1.3378E-92	235.88
EASHYSIHDIVISYN(de)TSDSTVFPGAVAK	3007.4563	IPI00222967	IPI00222967	yes	no	2,3,4		5.6769E-31	183.56
EAQYN(de)STFR	1114.5043	IPI00807983	IPI00807983	yes	no		2	0.018705	114.4
EAN(de)ITEDQIIFFPK	1663.8457	IPI00122122	IPI00122122	yes	no		2	5.0461E-173	263.56
EAIGNASQI(de)FDSWIK	1677.8362	IPI00134585	IPI00134585	yes	yes		2	0.0018262	114.01
EAFN(de)ETNQAIQTISR	1720.838	IPI00112614	IPI00112614	yes	yes		2	6.2011E-110	240.2
EAFIPQGGSVQVN(de)CSSSCK	2053.9197	IPI00122973	IPI00122973	yes	no		2	0.0001072	139.02
EACIQHYYVN(de)ASYIVWK	2143.0197	IPI00120155	IPI00120155	yes	yes		2	0.0009393	114.29
E(de)SVYRNYSIFIADINQER	2216.0862	IPI00331318	IPI00331318	yes	no		3	0.0006581	131.48
E(de)MGNTVMDIIRNYTANATSSREEAWDYVQAQVK	3789.7727	IPI00108098	IPI00108098	yes	no	3,4		0.0076039	57.339
DYYPIN(de)ESIASIPIR	1749.8938	IPI00121627	IPI00121627	yes	yes		3	0.0008729	143.25
DYYIN(de)KTENEK	1415.6569	IPI00396840	IPI00396840	yes	no		2	2.1765E-09	174.24
DYRPSAGN(de)NSIYQDTVVFK	2173.044	IPI00322463	IPI00322463	yes	no	2,3		0.0003008	149.95
DYCMYYNNN(de)WTR	1698.6555	IPI00121776	IPI00121776	yes	yes		2	1.2685E-07	162.93
DWMPNN(de)HSVIIIDDFESPQK	2384.1107	IPI00221833	IPI00221833	yes	yes		3	0.0025887	101.92
DWIWNQMHIDEEKN(de)ESIPHYVGK	2867.3337	IPI00138180	IPI00138180	yes	yes		3	1.3212E-07	115.57
DWCN(de)WTIISR	1349.6187	IPI00124933	IPI00124933	yes	no		2	3.4026E-12	179.19
DVGSGTTN(de)NSQACAQFIEQYFHNSDITEFMR	3566.5467	IPI00130661	IPI00130661	yes	yes	3,4		1.3035E-20	126.46
DVFIPKPSWGN(de)HTPIFR	2010.0476	IPI00117312	IPI00117312	yes	yes		3	0.022711	91.969
DTYVN(de)ASQSIYGSSPR	1743.8064	IPI00122977	IPI00122977	yes	no		2	3.6139E-176	265.77
DTISIN(de)ATNIK	1188.635	IPI00121190	IPI00121190	yes	no		2	1.09E-64	221.55
DTEENIHVIN(de)QTISEVIFPMDIK	2684.3367	IPI00356462	IPI00356462	yes	no		3	0.013617	64.26
DTCAQECSHFNI(de)TK	1709.7138	IPI00132474	IPI00132474	yes	no		2	2.0947E-26	185.95
DTAVEGEEIEVN(de)CTAMASKPATTIR	2692.2684	IPI00856723	IPI00856723	yes	no	2,3		5.7656E-08	112.58
DSYPDGN(de)ITWYRNGK	1784.8118	IPI00121378	IPI00121378	yes	no		2	0.0001376	154.72
DSYPDGN(de)ITWYR	1485.6525	IPI00121378	IPI00121378	yes	no		2	0.0041331	107.97
DSKIN(de)NTNVR	1159.5945	IPI00380801	IPI00380801	yes	no		2	8.7779E-05	167.12
DSGIWINGFDYTGMSHVTPHIPEIN(de)DTIR	3284.5561	IPI00407222	IPI00407222	yes	yes	3,4		1.9255E-14	146.76
DQQAAEANKN(de)ASIIIK	1712.9057	IPI01016174	IPI01016174	yes	no		3	0.0077398	103.76
DQGNV(de)TDMASMK	1295.5486	IPI00471081	IPI00471081	yes	yes		2	1.3922E-66	227.06
DQDCDKF(de)NQCGTCTEFKECHTIQNYTIWR	3753.5705	IPI00125220	IPI00125220	yes	no	3,4		0.0043114	73.341
DQCIVDDITYNVN(de)DTFHKR	2352.0805	IPI00113539	IPI00113539	yes	no	2,3		6.455E-37	199.41
DQCIVDDITYNVN(de)DTFHK	2195.9793	IPI00113539	IPI00113539	yes	no	2,3		8.8629E-37	199.41
DNVN(de)CSGVYEHEPIEIGK	2058.9317	IPI00947577	IPI00947577	yes	yes		2	0.014157	78.021
DNTTC(de)YEFKK	1304.5707	IPI00119063	IPI00119063	yes	yes		2	0.000132	162.26
DNTTC(de)YEFK	1176.4757	IPI00119063	IPI00119063	yes	yes		2	0.003897	135.43
DNSCVDPPHVPN(de)ATIVTR	1990.9531	IPI00130010	IPI00130010	yes	no	2,3		1.8122E-12	171.29
DNKIQIQQN(de)ISIEFIK	1930.0524	IPI00330670	IPI00330670	yes	yes		2	0.032577	83.087
DNATDS(de)VPIR	1086.5306	IPI00119063	IPI00119063	yes	yes		2	5.8108E-27	196.11

DN(de)YSVIIQENKPVGFSVIK	2149.1419	IPI00623114	IPI00623114	yes	no	2		0.0014074	107.57
DN(de)HTSCAECIK	1333.5391	IPI00320605	IPI00320605	yes	no	2		0.013545	93.839
DN(de)DSIITRK	1060.5513	IPI00131168	IPI00131168	yes	yes	2		0.0037714	145.25
DN(de)ATQEEIIHYIEK	1701.821	IPI00928320	IPI00928320	yes	no	2,3		7.6723E-08	167.69
DMSDGFISN(de)ITIQR	1595.7614	IPI00272381	IPI00272381	yes	no	2		3.8711E-75	222.16
DMSDEIGCVN(de)VTQCDGPNK	2137.8715	IPI00312063	IPI00312063	yes	no	2		1.1671E-132	248.46
DMEEFVQSSGDHGVVVFSIGSMVSN(de)MTEEK	3274.4468	IPI00112322	IPI00112322	yes	no	3		0.010037	46.63
DKWGISDEHFQPRPEAVQFFN(de)VTTIQK	3216.5992	IPI00111370	IPI00111370	yes	yes	3		0.0027693	78.428
DKNGTRAEPPIN(de)ASAGDQEEK	2226.0513	IPI00123342	IPI00123342	yes	yes	2,3		2.1349E-20	180.31
DKN(de)GTRAEPPINASAGDQEEK	2226.0513	IPI00123342	IPI00123342	yes	yes	2,3		2.1349E-20	180.31
DKISPIHIA(de)INFSIDPK	1907.0516	IPI00115976	IPI00115976	yes	yes	2,3		0.0010966	125.22
DIVV(de)EIYSQWMKNPNNNTIHPNIR	2894.4497	IPI00319509	IPI00319509	yes	no	3		3.4358E-08	136.68
DIVQNVFMSN(de)MSMDIQSHPSSCPK	2751.2125	IPI00121190	IPI00121190	yes	no	3		1.175E-08	114.55
DIVGN(de)ITEIESEDIQIEAIIMR	2500.2731	IPI00122399	IPI00122399	yes	no	3		1.1962E-40	202.39
DITNIIN(de)NTFIR	1432.7674	IPI00650038	IPI00650038	yes	no	2		1.1512E-07	162.31
DISVFAPN(de)MTEIIKDVTQYR	2339.1831	IPI00855103	IPI00855103	yes	no	2		0.010485	88.574
DISGN(de)ETHFTGSEVGFIKPISCR	2550.2173	IPI00123652	IPI00123652	yes	no	3		5.4059E-08	145.71
DINSQIEIQNVTN(de)DTHITIIGIKPDTTYDIK	3511.8046	IPI00110264	IPI00110264	yes	yes	3		1.2605E-24	162.71
DINSQIEIQN(de)VTNDTHITIIGIKPDTTYDIK	3511.8046	IPI00110264	IPI00110264	yes	yes	3		1.2605E-24	162.71
DIN(de)HTAQGIIEKDSWK	1853.9272	IPI00120953	IPI00120953	yes	no	3		0.0024048	122.93
DIMDYYKDTTGSHTFQGMFGCEITNN(de)R	3200.3638	IPI00137987	IPI00137987	yes	yes	3		2.3208E-13	131.33
DIKEAGN(de)ITTDGYEIIGK	1935.9789	IPI00121430	IPI00121430	yes	no	2		0.0049766	103.66
DIIIHSTGHN(de)ISR	1461.7688	IPI00223987	IPI00223987	yes	yes	2,3		4.521E-06	155.66
DIIFSDDTECISNIQN(de)KTTYK	2504.1741	IPI00471080	IPI00471080	yes	no	2		0.0038438	91.64
DIGPAIAN(de)SSHDVK	1422.7103	IPI00108811	IPI00108811	yes	yes	2		2.7275E-75	223.06
DIFTNQSDFADTTKDTPITITVIH(de)K	2820.4182	IPI00116105	IPI00116105	yes	yes	3		1.0709E-08	121.25
DIFTN(de)QSDFADTTK	1601.7209	IPI00116105	IPI00116105	yes	no	2		0	328.14
DIEHHITNVNVSFYDDIVN(de)GTVIK	2741.3661	IPI00467600	IPI00467600	yes	yes	3		1.9334E-33	191.22
DIEHHITNVN(de)VSFYDDIVNGTVIK	2741.3661	IPI00467600	IPI00467600	yes	yes	3		1.9334E-33	191.22
DIAVGDDYTN(de)R	1237.5575	IPI00468674	IPI00468674	yes	no	2		0.0021933	122.7
DHVNVSMIEATDFPFN(de)TTEWEGYIPK	3039.396	IPI00136942	IPI00136942	yes	yes	3		3.2431E-13	119.38
DHVN(de)VSMIEATDFPFNTTEWEGYIPK	3039.396	IPI00136942	IPI00136942	yes	yes	3		3.2431E-13	119.38
DHIIN(de)KTHSICPR	1589.8096	IPI00118994	IPI00118994	yes	yes	2		0.017765	105
DGTSQPAICPQN(de)VTMNMEGIK	2290.0392	IPI00320204	IPI00320204	yes	yes	2		3.2633E-75	180.86
DGSQDFNE(de)TWENYEK	1860.7439	IPI00128206	IPI00128206	yes	yes	2		0.023134	114.01
DGSDSAAMVYN(de)SSQEWGIR	2071.8905	IPI00119522	IPI00119522	yes	yes	2		3.6724E-179	269.49
DGQQN(de)ISIIYTEPGASQTQTGASFR	2668.2729	IPI00284816	IPI00284816	yes	no	2,3		1.0433E-43	194.97
DGQEIHPIETTVNPSGKNVSYN(de)ISSTVR	3041.5054	IPI00775779	IPI00775779	yes	no	3		0.0001841	89.272
DGQEIHPIETTVNPSGKN(de)VSYNISSTVR	3041.5054	IPI00775779	IPI00775779	yes	no	3		0.0001841	89.272
DGIAIN(de)ITIR	1084.6241	IPI00119065	IPI00119065	yes	yes	2		0.0018371	147.62
DGHFKEDPYWENMIN(de)HSVHR	2510.1186	IPI00410796	IPI00410796	yes	yes	3		0.0017968	99.891
DGAFHHIHSTPFGNY(de)SFISVDATQRPGPK	3182.5322	IPI00226714	IPI00226714	yes	no	3		2.5537E-13	114.39
DEIGN(de)VSTSHIIIIDDSVEMEIRPR	2837.4229	IPI00309035	IPI00309035	yes	yes	3		0.027095	63.225
DDYRPTWTIN(de)QTEPVAGNYYPVNTR	2969.3944	IPI00381303	IPI00381303	yes	yes	2,3		6.613E-25	181.83
DDIHPTIPAGQYFINI(de)TYNYPVHSFDGR	3249.552	IPI00387318	IPI00387318	yes	no	3,4		1.2507E-13	119.02
DCVSCQN(de)VSR	1223.5023	IPI00121190	IPI00121190	yes	no	2		0.0095447	115.29
DCDFIEDGEERN(de)CTGK	1943.7626	IPI00127280	IPI00127280	yes	yes	2		1.1618E-18	185.27
DAMVGN(de)YTCEVTEISR	1843.808	IPI00124830	IPI00124830	yes	no	2		4.9926E-177	270.51
DAHFN(de)STITEFGETINNFVER	2440.1295	IPI00346062	IPI00346062	yes	yes	3		0.026075	73.918
DAAQCSGGSVAHIAEIGIPTN(de)ITHIIIFR	3060.5815	IPI00331407	IPI00331407	yes	no	3		0.0007581	76.471
CVVHYEN(de)STVPEKK	1688.8192	IPI00409148	IPI00409148	yes	yes	2,3		3.0359E-36	201.48
CVVHYEN(de)STVPEK	1560.7242	IPI00409148	IPI00409148	yes	yes	2		0.0020616	132.59
CVAN(de)YTGNGR	1110.4876	IPI00111793	IPI00111793	yes	yes	2		0.025359	99.973
CTAQVCAGYCSNN(de)STCTVNQGNQPQCR	3134.2481	IPI00119063	IPI00119063	yes	yes	3		0.0011645	72.316
CSIIHSN(de)ISVKANVDQRIR	2209.175	IPI00133222	IPI00133222	yes	yes	3		0.039827	42.382
CSIIHSN(de)ISVK	1256.6547	IPI00133222	IPI00133222	yes	no	2		1.9627E-08	154.66
CQSPYQMN(de)GSDTVTCINGR	2186.9143	IPI00788443	IPI00788443	yes	no	2		0.0007113	99.392
CQEAIN(de)ATCK	1193.5169	IPI00314443	IPI00314443	yes	no	2		0.0053032	129.82
CQAYKN(de)NSESFIHIYMYEVAR	2622.1995	IPI00130654	IPI00130654	yes	no	3		0.0042993	93.753
CQAIDFFPQN(de)ITMR	1739.8123	IPI00331440	IPI00331440	yes	no	2		0.0004618	143.93
CPMAGISHTTWHSN(de)R	1753.7777	IPI00130764	IPI00130764	yes	no	2,3		0.0005353	141.54
CPIITPFNDTNVVHTVNTAIAAFNTQNN(de)GTYFK	3681.7886	IPI00128249	IPI00128249	yes	yes	3,4		2.6384E-06	88.708
CPIITPFN(de)DTNVVHTVNTAIAAFNTQNNGTYFK	3681.7886	IPI00128249	IPI00128249	yes	yes	3,4		2.6384E-06	88.708
CNSVITYN(de)ITPVVQK	1734.8975	IPI00222967	IPI00222967	yes	no	2		0.0008369	117
CN(de)ITIKDPTPADPIWYEAK	2231.0933	IPI00114842	IPI00114842	yes	no	2		2.5626E-18	182.31
CN(de)ASSQFICSSGR	1472.6136	IPI00119063	IPI00119063	yes	yes	2		0.0004262	146.96
CIVPHTVN(de)ISSAWR	1638.83	IPI00129041	IPI00129041	yes	no	2		0.003893	138.55
CISPNVTSCACTIN(de)FTIK	2084.9693	IPI00387318	IPI00387318	yes	yes	2		1.4196E-61	214.66
CISPN(de)VTSCACTINFTIK	2084.9693	IPI00387318	IPI00387318	yes	yes	2		1.4196E-61	214.66
CISKN(de)YTTDIITK	1555.7916	IPI00124640	IPI00124640	yes	yes	2		1.8845E-21	147.73
CIQMSSFAN(de)R	1212.538	IPI00320065	IPI00320065	yes	no	2		3.0931E-77	235.01
CIQHFYGPNHEHCFN(de)R	2114.8952	IPI00130420	IPI00130420	yes	no	2		0.0024423	119.54
CIPHFAMIGNDTVMCTEQGN(de)WTRIPECIEVK	3705.687	IPI00322463	IPI00322463	yes	no	3,4		8.7719E-05	85.573
CIPHFAMIGNDTVMCTEQGN(de)WTR	2737.1869	IPI00322463	IPI00322463	yes	no	3		1.0379E-09	148.2
CIPHFAMIGN(de)DTVMCTEQGNWTRIPECIEVK	3705.687	IPI00322463	IPI00322463	yes	no	3,4		8.7719E-05	85.573
CIPHFAMIGN(de)DTVMCTEQGNWTR	2737.1869	IPI00322463	IPI00322463	yes	no	3		1.0379E-09	148.2
CIN(de)HTTQK	1000.476	IPI00125266	IPI00125266	yes	yes	2		0.012795	144.12
CHEGN(de)GTFECGACR	1653.6082	IPI00132474	IPI00132474	yes	yes	2		0.0004315	150.34
CGNCN(de)ITSIEDEDFCK	1960.7601	IPI00137599	IPI00137599	yes	yes	2		3.3069E-230	287.91
CFNAMEVDAIN(de)SSHPVSTPVENPAQIR	2982.3964	IPI00318595	IPI00318595	yes	no	2,3,4		1.2606E-49	198.1
CFMAN(de)GTWGYPFHDGDYIK	2277.9612	IPI00129010	IPI00129010	yes	no	2,3		0.0015904	101.67
CF(de)ANKVNISFPSAQSIPASDTHIK	2631.3115	IPI00624663	IPI00624663	yes	yes	2,3		3.2122E-18	175.52
CEVICN(de)QSNKPITITVEQSR	2375.1573	IPI00626537	IPI00626537	yes	yes	2		0.0057112	90.718
CEQEASEDIKPAITGN(de)KTMQYVPNSHDVK	3288.5391	IPI00134808	IPI00134808	yes	no	3		0.0066035	58.924
CEQEASEDIKPAITGN(de)K	1888.8837	IPI00134808	IPI00134808	yes	no	2		7.4614E-92	235.26
CDGDFDCEDRTDEAN(de)CSVK	2291.8365	IPI00515360	IPI00515360	yes	no	2		0.0011016	124.29

CCGWVSHYN(de)WTENEEIMGFTK	2647.093	IPI00108098	IPI00108098	yes	no		3	9.8235E-06	113.86
CAMDIGVNYIGTVN(de)VTHTGIQCQIWR	3005.431	IPI00114206	IPI00114206	yes	yes		3	0.014261	55.302
CAISHAITIEFHQDGNGPENVGIYN(de)ISR	3111.4832	IPI00222429	IPI00222429	yes	yes	3,4		7.9279E-19	166.65
AYVPN(de)ATWYDYETGEEIGWR	2419.0757	IPI00848693	IPI00848693	yes	yes		2	1.8246E-05	147.49
AYTIFVPTNHSIETQGN(de)NSVIGIDTVR	2945.4883	IPI00987265	IPI00987265	yes	no		3	1.3981E-06	97.5
AY(de)AISGPTVQDGIPPFNWNRFNETIHR	3099.5315	IPI00471081	IPI00471081	yes	yes		3	5.9147E-08	104.52
AWN(de)YTVDTPHGMFVSGTPFEQSVWITDVGSGPYGHTVK	4166.9473	IPI00228567	IPI00228567	yes	yes		4	0.0004875	51.471
AWEKEIHHI(de)QEQNVSNVFIDKEEFFIGSK	3500.7365	IPI00605187	IPI00605187	yes	no	3,4		3.4384E-39	186.82
AVYEAIRN(de)CSIEDDSVR	1995.932	IPI00856861	IPI00856861	yes	no		2	0.0011079	127.83
AVVGSYTTTN(de)VSIIAPR	1747.9468	IPI00330481	IPI00330481	yes	yes		2	3.0362E-06	159.88
AVTSISHDGSVICTFTAN(de)STYTK	2459.1639	IPI00453488	IPI00453488	yes	yes		2	1.333E-08	137.72
AVN(de)QTGAIYQCDYSTSR	1932.8636	IPI00120674	IPI00120674	yes	no		2	2.0752E-47	204.05
AVISMIN(de)ETMNEAPEETK	2005.9336	IPI00875419	IPI00875419	no	no	2,3		9.4205E-292	300.54
AVIGDHGDEIFSVFGSPFIKDGASEEETN(de)ISK	3394.6205	IPI00387289	IPI00387289	yes	yes	3,4		2.2955E-21	158.29
AVDENN(de)ESTACFIIRSGCDVNSPR	2710.2075	IPI00329843	IPI00329843	yes	yes		3	0.031756	67.866
AVCGIN(de)TSDRCDFVR	1768.7985	IPI00127134	IPI00127134	yes	no		2	0.0082643	110.92
ATVN(de)DSGEYR	1110.4942	IPI00129485	IPI00129485	yes	no		2	0.0011941	155.48
ATTHAQGHQPVIGN(de)DTIR	1914.966	IPI00129465	IPI00129465	yes	yes		2	0.022163	84.653
ATSQFQN(de)GSIITAFIPGISQCTVYSAK	2888.4378	IPI00340140	IPI00340140	yes	yes		3	1.0268E-05	91.643
ATPNEGFFNQN(de)ITTFYYDR	2297.0389	IPI00315535	IPI00315535	yes	yes	2,3		1.4067E-19	184.15
ATIITFICDRDAGVGFPEYQEEDN(de)STYNFR	3527.594	IPI00308971	IPI00308971	yes	yes		3	0.0043742	67.31
ATHN(de)DSGSYFCR	1413.5732	IPI00162870	IPI00162870	yes	yes		2	0.022619	82.202
ATHAQPAIVTAPN(de)ETSPK	1831.9428	IPI00137958	IPI00137958	yes	yes		2	0.016444	75.564
ASVVN(de)VTQHCTMESWESMNEVAR	2664.1731	IPI00121240	IPI00121240	yes	yes		3	2.5965E-06	107.33
ASSIMVN(de)ITEIFPKPK	1773.9699	IPI00406901	IPI00406901	yes	no		2	1.1813E-35	201.3
ASQQISN(de)ETSSFGFNIIRK	2126.0756	IPI00153258	IPI00153258	yes	no		2	0.0003522	111.22
ASQQISN(de)ETSSFGFNIIR	1997.9807	IPI00153258	IPI00153258	yes	no		3	1.064E-26	186.72
ASGN(de)FSQVDWFIIHK	1747.8682	IPI00469123	IPI00469123	yes	no		3	0.0047222	95.981
ARIQGGVIIAQIIKN(de)ITIMATTSQFPK	2911.6681	IPI00154056	IPI00154056	yes	no		3	4.8704E-13	131.33
AQTN(de)YTCVAEIIYR	1700.8192	IPI00323053	IPI00323053	yes	no		2	4.035E-75	220.46
AQAGNKSQDSGIAEMEEIPVPHNIKINN(de)ITCDSFK	3854.8567	IPI00969894	IPI00969894	yes	yes		4	0.022351	46.785
AQAGN(de)KSQDSGIAEMEEIPVPHNIKINNITCDSFK	3854.8567	IPI00969894	IPI00969894	yes	yes		4	0.022351	46.785
AQAAIDKAN(de)ASR	1214.6367	IPI00119065	IPI00119065	yes	yes		2	0.0003854	149.69
APSN(de)VSTVIHIIYIPEEAK	2080.1205	IPI00121421	IPI00121421	yes	no	2,3		0.0002308	109.72
APQHVVNHIPPYTN(de)VSIK	2013.0796	IPI00123040	IPI00123040	yes	no		3	0.0043164	95.428
APIMPWNESSIFHIPRPVSIN(de)MTVK	2863.4877	IPI00930882	IPI00930882	yes	no		3	0.025202	64.723
APIMPWN(de)ESSIFHIPRPVSINMTVK	2863.4877	IPI00930882	IPI00930882	yes	no		3	0.025202	64.723
APDSN(de)SSSIIITTR	1460.7471	IPI00466999	IPI00466999	yes	no		2	0.0007116	128.06
ANQIVIPHN(de)TTFQTEPTK	2038.0484	IPI00466371	IPI01026704	no	no	2,3		7.8834E-05	151.44
ANIQFGENGT(de)TISAVTNK	1863.9327	IPI00127447	IPI00127447	yes	yes	2,3		9.9933E-155	258.8
ANIINFPEN(de)NTFVINIEQITQDDTGSYK	3197.5517	IPI00310059	IPI00310059	yes	no	2,3		4.4988E-14	150.03
ANEQVIQNIN(de)HTYK	1670.8376	IPI00876541	IPI00876541	yes	no		2	0.0013977	140.31
ANA(de)STFAVPSPVSNSADTR	1890.9072	IPI00119065	IPI00119065	yes	yes		2	0.015995	73.918
AN(de)YTIIK	821.4647	IPI00138209	IPI00138209	yes	no		2	1.3935E-12	182.68
AN(de)TTQPGIVEGGQVIK	1610.8628	IPI00120245	IPI00120245	yes	no		2	2.5563E-05	156.11
AN(de)TSIEIIIEGVR	1413.7827	IPI00988518	IPI00988518	yes	no		2	0.0013836	116.63
AN(de)KSSDIVSANR	1260.6422	IPI00136642	IPI00136642	yes	yes		2	0.0083967	113.99
AN(de)ISSQVIK	958.54475	IPI00322867	IPI00322867	yes	no		2	0.013398	119.68
AN(de)HSGAVVIIKR	1263.7412	IPI00420835	IPI00420835	yes	no		2	0.013595	97.463
AN(de)ESRIEEADQIYR	1692.8067	IPI00224237	IPI00224237	yes	no		2	6.7502E-08	162.94
AN(de)DTREEAGIPAAGEDETSWTER	2504.1052	IPI00653847	IPI00653847	yes	no		3	0.0080982	80.142
AN(de)DSDQGANAEIDYTFHQAPEVVRR	2802.2957	IPI00719927	IPI00719927	yes	no		3	7.7893E-09	143.04
AN(de)DSDQGANAEIDYTFHQAPEVVR	2646.1946	IPI00719927	IPI00719927	yes	no	2,3		8.3534E-09	143.91
AMN(de)TSQVEAMGIQMIPGYRDPYHGRPITK	3260.5893	IPI00169870	IPI00169870	yes	yes		3	7.5267E-07	92.147
AMN(de)ISASSMTITWK	1539.7425	IPI00469426	IPI00469426	yes	no		2	2.977E-75	220.46
AMIINQHVPMESSHIIQFVN(de)WSSIIPERYKK	3694.9116	IPI00387282	IPI00387282	yes	yes	3,4,5,6		8.476E-08	94.594
AMIINQHVPMESSHIIQFVN(de)WSSIIPERYK	3566.8167	IPI00387282	IPI00387282	yes	yes	3,4		4.1176E-15	113.31
AMIINQHVPMESSHIIQFVN(de)WSSIIPER	3275.6584	IPI00387282	IPI00387282	yes	yes	3,4,5		4.4463E-14	140.59
AMGVNVIAANTHN(de)TSMHMTGSGIYSPEAVR	3115.4638	IPI00230412	IPI00230412	yes	yes	3,4		9.4849E-16	112.48
AMFN(de)ITIIYPNNIIAISNMIPK	2490.3379	IPI00319509	IPI00319509	yes	yes	2,3		8.53E-07	147.35
AISPN(de)STISSAPK	1271.6721	IPI00460291	IPI00460291	yes	no		2	0.0014887	116.19
AINASAN(de)ITSDGVEVIGR	1785.9221	IPI00330632	IPI00330632	yes	no		2	6.1681E-204	273.78
AIN(de)VTISSMGRNGIK	1559.8454	IPI00131091	IPI00131091	yes	no		2	0.012135	109.84
AIN(de)VTISSMGR	1147.6019	IPI00131091	IPI00131091	yes	no		2	3.6127E-05	155.86
AIN(de)SSTEDGIK	1133.5564	IPI00129168	IPI00129168	yes	yes		2	0.010755	101.39
AIN(de)GSAIYTGSSIDFVR	1769.8948	IPI00877197	IPI00877197	yes	no	2,3		0	326.05
AIN(de)DSIHYINR	1314.668	IPI00808345	IPI00808345	yes	no		2	9.981E-05	151.1
AIN(de)ASANITSDGVEVIGR	1785.9221	IPI00330632	IPI00330632	yes	no		2	6.1681E-204	273.78
AIMKEVMNIIQPIN(de)VTK	1941.0791	IPI00126050	IPI00126050	yes	no	2,3		0.0019582	138.97
AIMDICDQIEKN(de)QTK	1805.8652	IPI00658632	IPI00658632	yes	yes		2	4.8222E-50	164.15
AIIQGIIPEQN(de)YTVQIIAYYKDK	2680.4476	IPI00330632	IPI00330632	yes	no		3	0.027557	69.148
AIIQGIIPEQN(de)YTVQIIAYYK	2437.3257	IPI00330632	IPI00330632	yes	no		3	0.0070421	77.506
AIGYEN(de)ATQAIGR	1362.6892	IPI00119809	IPI00119809	yes	no		2	3.4119E-44	203.87
AIGITEMFEPSKAN(de)FTK	1882.9499	IPI00115065	IPI00115065	yes	yes		2	0.0031496	138.05
AIEIQFGMN(de)ASSSIFFIQGVR	2314.178	IPI00469218	IPI00469218	yes	yes		3	0.0049735	81.51
AIDIDPSKTN(de)VSGGAIAIGHPIGGSGSR	2646.3725	IPI00226430	IPI00226430	yes	yes	3,4		2.3498E-38	189.84
AIAGIVYN(de)SSGTEPCYDIYR	2248.047	IPI00331550	IPI00331550	yes	yes	2,3		4.6269E-29	192.6
AHFSSIN(de)ITIR	1257.683	IPI00466371	IPI01026704	no	no		2	1.7749E-29	186.81
AGYFN(de)FTSATITYIAQEDGPVVIGSTSAPGQGGIIAQR	3856.9272	IPI00467180	IPI00467180	yes	yes	3,4		1.097E-33	122.7
AGVVVFN(de)CSIR	1220.6336	IPI00314779	IPI00314779	yes	yes		2	0.010265	99.215
AGPN(de)GTIFVVDAYK	1450.7456	IPI00221998	IPI00221998	yes	yes		2	5.1633E-27	192.32
AGIN(de)ASYSEK	1038.4982	IPI00308971	IPI00308971	yes	yes		2	0.001301	155
AGEQINNHVKN(de)KTQGK	1764.9231	IPI00116105	IPI00116105	yes	no	2,3		0.0001269	155.57
AGEQINNHVKN(de)K	1350.7004	IPI00116105	IPI00116105	yes	no		2	0.020861	136.53
AFYN(de)GTWYRR	1332.6364	IPI00858318	IPI00858318	yes	no		2	0.027548	98.033
AFYN(de)GTWYR	1176.5352	IPI00858318	IPI00858318	yes	no		2	0.012374	120.99

AFVEN(de)ITVIENSIVFK	1821.9877	IPI00742385	IPI00742385	yes	no	2,3	0	357.71
AFNISPN(de)DTSSGSCGINIVTIK	2294.1213	IPI00469218	IPI00469218	yes	yes	2,3	3.3828E-06	137.12
AFN(de)ITWISTDFK	1441.7242	IPI00118069	IPI00118069	yes	no	2	4.1559E-08	166.48
AFN(de)ECCTIANK	1326.5697	IPI00330833	IPI00330833	yes	yes	2	1.5991E-15	185.25
AFMN(de)SSFTIDPK	1356.6384	IPI00121362	IPI00121362	yes	no	2	4.5096E-06	155.86
AFITNF(de)SMIIDGVTYPGVVK	2171.1337	IPI00312711	IPI00312711	yes	no	2,3	3.3094E-50	208.49
AFIN(de)GTGVETVVSADIPNAHGIAVDWVSR	2994.5199	IPI00119063	IPI00119063	yes	yes	3,4	3.2171E-19	162.01
AFFDETKN(de)NTR	1341.6313	IPI00120674	IPI00120674	yes	no	2	0.0036529	138.99
AEQITIHAIGIGEANKT(de)QIR	2162.1808	IPI00675799	IPI00675799	yes	no	3	0.010911	85.937
AEPPINASAGDQ(de)EEK	1554.7162	IPI00123342	IPI00123342	yes	yes	2	0.0005839	146.2
AEISN(de)HTRPVIIVPGCIGNRIEAK	2643.4279	IPI00133500	IPI00133500	yes	yes	3,4	0.0005349	91.64
AEISN(de)HTRPVIIVPGCIGNR	2202.1692	IPI00133500	IPI00133500	yes	yes	2,3	2.5303E-05	136.34
AEDYGPVEVISHWHPN(de)ITINIVDDHTPWVK	3480.7103	IPI00121627	IPI00121627	yes	yes	3,4	7.5641E-06	90.1
ADSIYSQVVGISASQAN(de)ISK	2037.0378	IPI00115116	IPI00115116	yes	yes	2,3	3.4057E-233	280.62
ADN(de)YTYEHIR	1280.5786	IPI00273133	IPI00273133	yes	yes	2	0.028903	89.548
ADANPPATEYHWTTIN(de)GSIPK	2282.0968	IPI00311405	IPI00311405	yes	yes	2,3	4.2008E-06	130.89
ACN(de)ATNWIEYMFNK	1760.7651	IPI00132600	IPI00132600	yes	no	2	3.0161E-08	160.78
AAN(de)CTQVIVWHTR	1554.7725	IPI00116744	IPI00116744	yes	yes	2	0.030557	76.01
AAGVIEDVYN(de)RTQGIIAGHGIIQIA	2578.3867	IPI00169617	IPI00169617	yes	yes	2	4.801E-08	112.47
AAGVIEDVYN(de)R	1205.6041	IPI00169617	IPI00169617	yes	yes	2	7.2755E-08	138.76
AAEN(de)FTIIVK	1104.6179	IPI00471089	IPI00471089	yes	no	2	0.0022375	129.82
AACAVRPQEVTMVN(de)GTITNPVTGK	2513.273	IPI00308971	IPI00308971	yes	yes	2,3	4.3569E-08	118.37
A(de)YVFERNQSVGDPNVDIIR	2191.1022	IPI00127447	IPI00127447	yes	yes	2,3	1.8175E-48	209.66
A(de)VAYGEKNITFQGPIPK	1831.9832	IPI00111794	IPI00111794	yes	no	2,3	6.6698E-09	168.69
A(de)RNISGVVIADHSGSFHNR	2036.03	IPI00118674	IPI00118674	yes	yes	3	0.0005256	123.59

N-terminal Ser/Thr	15
ratio	1.08%

Conventional HC strategy 2

De-glycopeptides Sequence	Mass	Proteins	Leading r	Unique (G	Unique (P	Charges	PEP	Score
YYN(de)YTISINGK	1334.6507	IPI00221426	IPI00221426	yes	yes	2	0.0007149	155.48
YYN(de)QSKGGSHTFQR	1671.7754	IPI00850057	IPI00850057	no	no	2	0.0023752	132.97
YYN(de)QSAGGSHTIQWMAGCDVESDGR	2788.1606	IPI00985828	IPI00985828	yes	no	3	6.6265E-84	258.37
YYHGEISYIN(de)VTRK	1741.8788	IPI00404551	IPI00404551	yes	no	2,3	1.6875E-06	168.89
YYHGEISYIN(de)VTR	1613.7838	IPI00404551	IPI00404551	yes	no	2,3	8.0537E-14	184.98
YVYVADIIAHEIHVIEKQPNMN(de)ITQIK	3178.6849	IPI00310567	IPI00310567	yes	yes	3,4	5.8569E-69	249.2
YVMIPVADQDKCVVHYEN(de)STVPEKK	2948.4412	IPI00409148	IPI00409148	yes	yes	3	5.5065E-09	159.06
YTSFEYPKNISFA(de)CNPGFFINGTSSSK	3062.412	IPI00322463	IPI00322463	yes	no	3	1.9882E-24	207.86
YTSFEYPKN(de)ISFACNPGFFINGTSSSK	3062.412	IPI00322463	IPI00322463	yes	no	3	1.9882E-24	207.86
YTGN(de)ASAIIIIPDQGR	1687.8893	IPI00131830	IPI00131830	yes	no	2,3	1.9261E-77	260.24
YTGN(de)ASAIFIIPDQGR	1721.8737	IPI00135635	IPI00135635	yes	no	2,3	4.64E-13	195.89
YSVQHMYFTYN(de)ISDTEHFPNAISK	2891.3225	IPI00469218	IPI00469218	yes	yes	3,4	2.5493E-07	172.69
YSVIITAEKPVGDISSPN(de)ETK	2247.1634	IPI00125497	IPI00125497	yes	yes	2,3	3.4224E-08	184.56
YSQAAN(de)STKEIDDCEQANK	2170.9437	IPI00187353	IPI00187353	yes	yes	2	0.0008741	124.31
YSNESQDISVNGYN(de)CTTSSVSSAIR	2738.209	IPI00322304	IPI00322304	yes	yes	2,3	3.103E-16	160.84
YSN(de)ESQDISVNGYNCTTSSVSSAIR	2738.209	IPI00322304	IPI00322304	yes	yes	2,3	3.103E-16	160.84
YSIYKDPAGWISINPIN(de)GTVDTTAVIDR	3078.5662	IPI00123746	IPI00123746	yes	yes	3	1.817E-05	99.614
YRAEFAVAN(de)DTGFVDIPQQEK	2397.1601	IPI00128154	IPI00128154	yes	no	2,3	8.5064E-28	218.79
YQTIN(de)CSVNVR	1352.6507	IPI00420835	IPI00420835	yes	no	2	0.0028127	132.17
YQMDN(de)VSSIVQIIGSHIEDVNADIIQTK	3129.5652	IPI00330594	IPI00330594	yes	yes	3,4	2.1055E-11	140.45
YQHVGQVIIFQAPEAGGRWN(de)QTQK	2754.399	IPI00894972	IPI00894972	yes	no	3	4.2253E-07	180.31
YQFNTNVVFSDN(de)GTIVDR	2087.9912	IPI00321375	IPI00321375	yes	no	2,3	1.5995E-08	189.77
YQFEVICPAPRPGAASN(de)ISFQAPFR	2822.3963	IPI00311159	IPI00311159	yes	yes	3	1.2639E-08	128.67
YQEIIEIESSIN(de)KTQWK	2108.079	IPI00990932	IPI00990932	yes	no	2,3	1.3759E-133	294.05
YQEGDITIYVINIHN(de)VTK	2119.095	IPI00170098	IPI00170098	yes	no	2	2.3568E-05	176.11
YPTPGEAPGVVGNFN(de)K	1645.81	IPI00112614	IPI00112614	yes	yes	2	0.0004048	141.85
YPQDYQFYIQN(de)FTAIPINTVVPPQR	3011.5181	IPI00110852	IPI00110852	yes	no	2,3,4	9.3345E-10	180.8
YPNN(de)GSIVWGK	1233.6142	IPI00848693	IPI00848693	yes	yes	2	4.2663E-05	163.12
YPHNHHVVNNTIEGNC(de)SSK	2205.9974	IPI00221426	IPI00221426	yes	yes	3	0.036124	69.979
YPHNHHVVNNTIEGN(de)CSSK	2205.9974	IPI00221426	IPI00221426	yes	yes	3	0.036124	69.979
YPHKPEIN(de)STTHPGADIKENFCR	2710.2922	IPI00114206	IPI00114206	yes	yes	3	0.0014367	105.91
YPHKPEIN(de)STTHPGADIK	2004.0065	IPI00114206	IPI00114206	yes	yes	2,3	0.0005818	166.48
YPAEGQRPIPN(de)VSIPIR	1906.0425	IPI00453524	IPI00453524	yes	yes	2,3	0.0006405	142.45
YN(de)HTGQVVIYK	1320.6826	IPI00466371	IPI01026704	no	no	2,3	0.0002469	160.67
YN(de)GSIGIWR	1064.5403	IPI00133292	IPI00133292	yes	no	2	0.019871	117.7
YMGIPIPEDNIDHYRN(de)STVMSR	2607.221	IPI00125813	IPI00125813	yes	no	2,3	0.0020032	96.544
YMDQNSDGWQDGVGYIN(de)SSEGAVGR	2704.146	IPI00114236	IPI00114236	yes	no	3	3.0204E-07	122.34
YMAIAIN(de)R	950.50077	IPI00123814	IPI00123814	yes	yes	2	0.029971	113.7
YKYETTISPTSNISSNSFICIEN(de)R	2823.3385	IPI00625835	IPI00625835	yes	no	3	2.9837E-39	223.89
YKYETTISPTSN(de)ISSNSFICIENR	2823.3385	IPI00625835	IPI00625835	yes	no	3	2.9837E-39	223.89
YKGTAGNAIMDGASQIVGEN(de)R	2151.0379	IPI00279079	IPI00279079	yes	yes	2,3	7.8819E-05	171.84
YKGN(de)ASAIFIIPDQGR	1748.921	IPI00128076	IPI00128076	yes	yes	2	0.0070309	110.12
YKGIN(de)ITEDTYKPR	1696.8784	IPI00119809	IPI00119809	yes	yes	2	4.6929E-179	311.99
YIYIASN(de)HSNK	1308.6463	IPI00118173	IPI00118173	yes	no	2	0.0081211	108.7
YIQVVYIHNNN(de)ISAVGQNDFCR	2623.2602	IPI00123196	IPI00123196	yes	yes	3	1.1425E-64	248.18
YIQPIIAVQFTN(de)ITVDTEIR	2333.2631	IPI00121550	IPI00121550	yes	yes	2,3	2.2121E-08	188.26
YIN(de)FSTSEKEK	1344.6561	IPI00135010	IPI00135010	yes	no	2	0.0044621	134.3
YIN(de)ETQQITQKIKTK	1835.0153	IPI00123223	IPI00123223	yes	yes	2	0.010629	115.1
YIN(de)ETQQITQKIK	1605.8726	IPI00123223	IPI00123223	yes	yes	2	1.2402E-13	186.16
YIN(de)ETQQITQK	1364.6936	IPI00123223	IPI00123223	yes	yes	2	2.407E-16	187.85
YIKNGN(de)ATIIR	1261.7143	IPI00985828	IPI00985828	no	no	2,3	4.2568E-11	184.24
YIKN(de)GNATIIR	1261.7143	IPI00985828	IPI00985828	no	no	2,3	4.2568E-11	184.24
YIINGSHAN(de)ETR	1373.6688	IPI00129968	IPI00129968	yes	yes	2	0.0061022	107.65
YIIN(de)GSHANETR	1373.6688	IPI00129968	IPI00129968	yes	yes	2	0.0061022	107.65
YIEIGN(de)ETIIR	1319.7085	IPI00850057	IPI00850057	yes	no	2	3.4822E-24	206.36

YHYN(de)GTIIDGTIFDSSYSR	2208.0124	IPI00130486	IPI00130486	yes	yes	2,3		2.325E-27	215.98
YHYN(de)GTFIDGTIFDSSHNR	2243.0032	IPI00130486	IPI00130486	yes	yes		2	2.8036E-37	226.57
YHYN(de)CSIIDGTR	1497.6671	IPI00122493	IPI00122493	yes	no		2	0.020508	90.793
YHYN(de)ASIIDGTIIDSTWNIGK	2380.1699	IPI00130486	IPI00130486	yes	yes		3	0.0001119	124.68
YHVIHIN(de)TTK	1224.6615	IPI00131021	IPI00131021	yes	yes		2	0.018875	105.98
YHHYSSN(de)FSIPK	1478.6943	IPI00129485	IPI00129485	yes	no	2,3		6.0177E-19	196.24
YHGFIN(de)TSYHR	1393.6527	IPI00154056	IPI00154056	yes	no	2,3		0.0009137	152.7
YHDYYITSTSN(de)GSIEGIENR	2318.0451	IPI00130754	IPI00130754	yes	yes		3	0.0007777	108.75
YGTAIVHIYVNETIAN(de)R	1933.0058	IPI00719927	IPI00719927	yes	no		2	0.00437	118.31
YGTAIVHIYVN(de)ETIANR	1933.0058	IPI00719927	IPI00719927	yes	no		2	0.00437	118.31
YGN(de)MTQDHVMHIITR	1814.8556	IPI00115892	IPI00115892	yes	no	2,3		0.0024611	107.65
YGMQNSGNEAAWN(de)YTIEQYQK	2494.0859	IPI00134585	IPI00134585	yes	yes	2,3		3.8767E-95	268.25
YGKN(de)DSITITQIK	1479.7933	IPI00123428	IPI00123428	yes	no	2,3		8.3662E-42	232.37
YGIVVIDECPGVGIVIPQSFGN(de)ESIR	2817.4371	IPI00309230	IPI00309230	yes	no		3	4.1719E-09	145.44
YGHPNGTQGN(de)STMWPVFTSTEQK	2566.1547	IPI00131168	IPI00131168	yes	yes		3	2.5164E-05	114.46
YGHPN(de)GTQGNSTMWPVFTSTEQK	2566.1547	IPI00131168	IPI00131168	yes	yes		3	2.5164E-05	114.46
YFTNRI(de)DVSQNVSSDTDQSCESTK	2780.2195	IPI00120769	IPI00120769	yes	no		3	2.1599E-06	154.84
YFFIQSVDSDGRN(de)FTSSPPGQTQFK	2852.3406	IPI00344686	IPI00344686	yes	yes		3	9.9149E-24	203.71
YEVDWNQSTIDCVDPISSIAAN(de)R	2652.2126	IPI00331032	IPI00331032	yes	no	2,3		6.0207E-15	200.99
YEVDWN(de)QSTIDCVDPISSIAANR	2652.2126	IPI00331032	IPI00331032	yes	no	2,3		6.0207E-15	200.99
YEQIQN(de)ETR	1179.552	IPI00154056	IPI00154056	yes	no		2	0.0019903	164.65
YEQAKN(de)ISQDIEK	1564.7733	IPI00400016	IPI00400016	yes	no		2	0.0073126	152.88
YE(de)TEKNNGAGYFIEHIAFK	2230.0695	IPI00111885	IPI00111885	yes	no		3	0.0002611	171.56
YDVN(de)RSDSGGSIQIEEGYFVHHFAPENIPTMSK	3723.7264	IPI00312711	IPI00312711	yes	no	3,4		9.499E-15	122.8
YDKSEDVGAAAMIN(de)YTHIIMEAVPGHPAIYR	3431.6642	IPI00874858	IPI00874858	yes	no	3,4		4.9929E-15	149.62
YDIPASINYIIN(de)K	1522.8031	IPI00129265	IPI00129265	yes	yes		2	0.0002328	159.08
YAVYWN(de)SSNPR	1355.6258	IPI00114364	IPI00114364	yes	no		2	0.0048513	119.76
YATENDISSIHNSTITCIVN(de)QTTSITGTSPEIMEK	3854.819	IPI00230319	IPI00230319	yes	no	3,4		3.3432E-12	114.35
YATENDISSIHN(de)STITCIVNQTTSITGTSPEIMEK	3854.819	IPI00230319	IPI00230319	yes	no	3,4		3.3432E-12	114.35
YAFQEIIGDISFIIPTIN(de)FSK	2415.2726	IPI00122557	IPI00122557	yes	no	2,3		9.4661E-34	195.96
WVITAAHCIIYPPWDKN(de)FTENDIIVR	3170.6012	IPI00114206	IPI00114206	yes	yes	3,4		5.1621E-06	117.31
WVGN(de)WTYEIK	1294.6346	IPI00153143	IPI00153143	yes	yes		2	0.01621	108.7
WVDGASIN(de)FSHWNSGEPNDSR	2374.0363	IPI00223352	IPI00223352	yes	yes	2,3		0.0001248	121.46
WTGHN(de)VTVVQR	1295.6735	IPI00119063	IPI00119063	yes	yes	2,3		0.0021103	136.96
WTDNTEYN(de)NTIPIRGEER	2207.0243	IPI00276430	IPI00276430	yes	yes	2,3		0.0002945	147.56
WSN(de)DSATISFTKPWSQGK	2038.9749	IPI00320065	IPI00320065	yes	no	2,3		1.1068E-05	181.41
WSFSN(de)GTSWQK	1326.5993	IPI00315576	IPI00315576	yes	yes		2	2.2279E-12	192.5
WQN(de)DTGPSDKSDISQK	1804.8228	IPI00222305	IPI00222305	yes	yes		2	6.4596E-37	224.59
WKPPSDPNGN(de)ITHYIVYWER	2471.2022	IPI00128358	IPI00128358	yes	no		3	0.000177	146.81
WIQQEFHSN(de)ASIPTHIVTFNVIEK	2837.4501	IPI00624345	IPI00624345	yes	yes		3	3.0912E-07	141.31
WIN(de)ETQIK	1030.5447	IPI00453524	IPI00453524	yes	yes		2	0.01478	131.06
WIIVGAPTASWISN(de)ASVVNPGAIYR	2641.4017	IPI00121334	IPI00121334	yes	no		3	0.0004503	89.668
WICDGDNDCGNSEDESN(de)ATCSAR	2631.9497	IPI00119063	IPI00119063	yes	yes	2,3		8.1908E-06	111.53
WICDGDN(de)DCGNSEDESNATCSAR	2631.9497	IPI00119063	IPI00119063	yes	yes	2,3		8.1908E-06	111.53
WGHN(de)VTEFQQR	1400.6585	IPI00754386	IPI00754386	yes	yes	2,3		0.0011476	146.16
WGEVDIIGN(de)CSQFYPDR	2054.9156	IPI00320675	IPI00320675	yes	yes		2	6.6402E-14	202.96
WFHN(de)ESIIPHQDANYVIQSAR	2524.2248	IPI00162870	IPI00162870	yes	yes		3	0.0001001	152.01
WECKN(de)DTIFGIK	1509.7286	IPI00126186	IPI00126186	yes	yes	2,3		0.0036778	145.31
WASVVVPIGKEQN(de)YTCR	2006.0044	IPI00109996	IPI00109996	yes	no	2,3		0.0001556	154.51
WASVVVPIGKEQN(de)YTCHVVYHEGIPEPITIR	3491.766	IPI01026927	IPI01026927	yes	no	3,4		1.7982E-06	107.88
WAAVVVPIGKEQN(de)YTCHVHHK	2472.2485	IPI00850057	IPI00850057	yes	no	2,3,4		1.4075E-05	179.14
VYVYAVN(de)QTR	1211.6299	IPI00466371	IPI01026704	no	no		2	4.1391E-15	198.25
VYTYADTPNDFQISN(de)FSIPEEDTKIKIPIIHQAIK	4048.0833	IPI00108811	IPI00108811	yes	yes		4	3.2125E-07	85.185
VYTYADTPNDFQISN(de)FSIPEEDTKIK	3034.4448	IPI00108811	IPI00108811	yes	yes		3	1.327E-09	138.04
VYTYADTPNDFQISN(de)FSIPEEDTK	2793.2657	IPI00108811	IPI00108811	yes	yes		3	0.0016084	86.557
VYTVSSSPSCYMYVYVN(de)TTEVAIEQDIAYIQEIK	3962.8846	IPI00136012	IPI00136012	yes	yes		4	0.0005156	66.501
VYSIPGREN(de)YSSVDANGIQSQMISR	2770.3344	IPI00127100	IPI00127100	yes	yes		3	7.0241E-08	168.68
VYMKN(de)VTVVIR	1320.7588	IPI00469218	IPI00469218	yes	yes	2,3		7.7034E-05	164.33
VYIN(de)DSVEISRNENK	1778.8799	IPI00126769	IPI00126769	yes	yes		2	4.8912E-63	250.52
VYIN(de)DSVEISR	1293.6565	IPI00126769	IPI00126769	yes	yes		2	1.1687E-08	182.71
VYIHPFHIIYHN(de)K	1679.8936	IPI00654069	IPI00654069	yes	yes	2,3		1.6694E-09	176.48
VYHQAGYIN(de)FSITDDMIPHDSGIR	2748.2966	IPI00126060	IPI00126060	yes	no		3	1.1337E-06	135.49
VYGQNINN(de)ETWSR	1579.7379	IPI00762609	IPI00762609	yes	no		2	6.8972E-06	164.68
VYGGIVN(de)QSEINEGTAFFR	2100.0276	IPI00416285	IPI00416285	yes	yes		2	1.211E-62	250.47
VWPDYPN(de)ITVDPSIGWDHQVEQYR	2913.3722	IPI00848693	IPI00848693	yes	yes		3	0.0023479	81.016
VWPDGVIPFVIGGN(de)FTGSQR	2145.1007	IPI00125182	IPI00125182	yes	no	2,3		4.1423E-05	162.59
VWN(de)STFIEDYKDFDR	1933.8846	IPI00468674	IPI00468674	yes	no	2,3		2.5815E-14	194.77
VWN(de)QTEQEPAAYHIISICFVR	2560.2533	IPI00129079	IPI00129079	yes	no		3	0.0001347	120.9
VVSVDISFRPIN(de)ETFPVVYIETPKR	2904.5749	IPI00624663	IPI00624663	yes	yes	3,4		1.728E-23	205.78
VVSVDISFRPIN(de)ETFPVVYIETPK	2748.4738	IPI00624663	IPI00624663	yes	yes		3	2.018E-05	118.37
VVRPDSEIGERPPEDN(de)QSFQYDHEAFIGK	3358.5854	IPI00137831	IPI00137831	yes	yes		3	2.7618E-05	85.423
VVMDIPYEIWN(de)ETSAEVADIKK	2549.2723	IPI00119039	IPI00119039	yes	no	2,4		2.8268E-06	172.81
VVMDIPYEIWN(de)ETSAEVADIK	2421.1774	IPI00119039	IPI00119039	yes	no	2,3		5.5208E-28	218.72
VVIISGVEPRPPTPQVQFTIN(de)ASSEDHKR	3200.6942	IPI00122973	IPI00122973	yes	no		3	4.0449E-14	151.42
VVIHPN(de)HSVVDIGIIK	1739.0094	IPI00409148	IPI00409148	yes	yes	2,3,4		9.6707E-28	219.35
VVIGEN(de)ITSNCPEVIYEIKEETPVFYK	3169.5893	IPI00674255	IPI00674255	yes	yes		3	4.5901E-06	113.42
VVIAGSN(de)MTICCMSPTK	1867.8664	IPI00119299	IPI00119299	yes	no		2	7.2615E-20	210.39
VVFISPAVPEEPEAYN(de)ITVIIR	2455.3363	IPI00405742	IPI00405742	yes	no	2,3		6.2272E-05	162.65
VVDGEREN(de)VSMVDYAHNNYQAQSAVPIR	3160.4996	IPI00134743	IPI00134743	yes	yes		3	1.9844E-05	102.21
VVAVSPAN(de)ISREER	1525.8213	IPI00229992	IPI00229992	yes	yes		2	0.020367	97.69
VTVEGMEYVFYN(de)DTK	1793.8182	IPI00405742	IPI00405742	yes	no		2	4.2928E-07	171.81
VTTCHIPQQN(de)ATIYK	1772.888	IPI00410951	IPI00410951	yes	no		2	0.024074	85.909
VTQIHIISSEIHFQPDQDIIIN(de)ISNASIGIHFR	3754.9795	IPI00135560	IPI00135560	yes	no		4	1.2555E-09	99.536
VTNSNANAAGPIIVAGYN(de)VSGSVR	2330.1979	IPI00222429	IPI00222429	yes	yes		2	2.3746E-21	204.53
VTNIMTGQTAN(de)ATSIIGTMTDAFPK	2582.272	IPI00923031	IPI00923031	yes	no		3	0.016616	53.304
VTITFATDESGN(de)HTGWK	1862.8799	IPI00467068	IPI00467068	yes	no		2	3.7402E-06	181.51

VTINNWVAN(de)KTEGR	1600.8322	IPI00136642	IPI00136642	yes	yes	2,3		5.4168E-21	207.52
VTIIPN(de)QTHYVVPK	1607.9035	IPI00742414	IPI00742414	yes	no		2	0.0013149	133.16
VTIDFN(de)ITNPENGPVIDDAIPNSVHGHIPFAK	3440.7365	IPI00466371	IPI00466371	yes	yes		3	6.4407E-15	127.3
VTIDFN(de)ITDPENGPVIDDAIPNSVHGHIPFAK	3441.7205	IPI01026704	IPI01026704	yes	yes	3,4		1.3419E-28	194.33
VSTVTIVSATSTTAN(de)MTMSPEGR	2340.1301	IPI00406603	IPI00406603	yes	yes		2	1.6039E-98	273.42
VSTIYANN(de)GSVIQGSTVASVYHKR	2550.319	IPI00121114	IPI00121114	yes	yes		3	0.010031	72.644
VSTIYANN(de)GSVIQGSTVASVYHK	2394.2179	IPI00121114	IPI00121114	yes	yes	2,3		1.0421E-265	338.24
VSQVIHEGGHN(de)VTKIIYESANIPDFRK	3050.5938	IPI00463764	IPI00463764	yes	no	3,4		0.0002296	106.58
VSQVIHEGGHN(de)VTKIIYESANIPDFR	2922.4988	IPI00463764	IPI00463764	yes	no		3	9.2514E-09	128.12
VSQVIHEGGHN(de)VTK	1503.7794	IPI00463764	IPI00463764	yes	no	2,3		1.9059E-50	240.46
VSNGN(de)GSIEIPATVPGYVHSAIHQHGIIQDPYYR	3688.8386	IPI00118011	IPI00118011	yes	no		4	0.0038814	57.156
VSITN(de)VSISDEGR	1375.6943	IPI00856723	IPI00856723	yes	no		2	1.4006E-65	246.64
VSIQEIPGSEHIEMIAN(de)ATTIAYIKR	2883.5164	IPI00124428	IPI00124428	yes	yes		3	0.0036703	72.916
VSIQEIPGSEHIEMIAN(de)ATTIAYIK	2727.4153	IPI00124428	IPI00124428	yes	yes	2,3,4		1.2256E-07	160.88
VSHVIN(de)DTHMK	1279.6343	IPI00674255	IPI00674255	yes	yes		2	0.016273	100.55
VSGQMHMQN(de)ITFQTEASVAQQEKEFK	2995.4168	IPI00312711	IPI00312711	yes	no		3	0.0001721	98.002
VSGQMHMQN(de)ITFQTEASVAQQEK	2591.2108	IPI00312711	IPI00312711	yes	no		3	2.034E-06	155.86
VSFYFFVTSPQN(de)VSDVIPR	2201.1157	IPI00153202	IPI00153202	yes	no	2,3		0.0005698	120.66
VSESEKSQIVN(de)ETHWQYYGTSDTR	2843.2998	IPI00749655	IPI00749655	yes	no		3	1.4006E-06	173.43
VRIDPPC(de)TNTTAPSNYINNPYVR	2661.2969	IPI00658539	IPI00658539	yes	no		3	1.64E-05	159.95
VQPIVAVADEGWYIIQN(de)K	2042.0837	IPI00111163	IPI00111163	yes	no	2,3		2.8254E-08	187
VQPIASSTIIHSDITSVYGTVVMN(de)R	2687.3953	IPI00674255	IPI00674255	yes	yes		3	0.016384	61.616
VQPFN(de)VTK	931.51272	IPI00222967	IPI00222967	yes	no		2	0.021732	134.26
VQGGSSVWGSVITHN(de)SSAITYQSWGR	2763.3365	IPI00453977	IPI00453977	yes	no	3,4		1.7884E-06	119.34
VPNNAIEGIEN(de)ITAIYIHHNEIQEVGSSMR	3347.6568	IPI00120187	IPI00120187	yes	yes	3,4		8.8861E-13	132.24
VPMMVQSGN(de)ISYFR	1627.7851	IPI00116105	IPI00116105	yes	yes		2	0.0036745	101.75
VNYEGGTWDWIAEAISSN(de)HTR	2405.1036	IPI00134585	IPI00134585	yes	yes		3	0.0001152	122.01
VNRFN(de)STEYQVVTR	1711.8642	IPI00119063	IPI00119063	yes	no	2,3		3.6772E-39	232.53
VNNVTGN(de)FTFVIR	1479.7834	IPI00395208	IPI00395208	yes	no		2	0.0010959	146.96
VNN(de)VTGNFTFVIR	1479.7834	IPI00395208	IPI00395208	yes	no		2	0.0010959	146.96
VNDSVPFHIGWN(de)STER	1856.8806	IPI00464256	IPI00464256	yes	yes		2	0.0006438	140.22
VNDNK(de)TAAEEAIR	1429.7161	IPI00400016	IPI00400016	yes	no		2	0.0055566	116.63
VNCEERNVTGIEN(de)FTIK	2021.984	IPI00378224	IPI00378224	yes	yes		2	4.5809E-05	173.03
VNCEERN(de)VTGIENFTIK	2021.984	IPI00378224	IPI00378224	yes	yes		2	4.5809E-05	173.03
VNASTTDPN(de)STVEQSAITR	1989.9603	IPI00338785	IPI00338785	yes	no		2	7.536E-38	231.86
VN(de)YTRAEEIFSR	1483.7419	IPI00131168	IPI00131168	yes	yes		2	0.019809	98.902
VN(de)SSIHSQISR	1226.6367	IPI00400016	IPI00400016	yes	no		2	1.6816E-138	286.93
VN(de)KTEEDYARDSIFVR	1940.9592	IPI00133103	IPI00133103	yes	no		2	0.0004276	161.04
VN(de)ITYNYPVR	1237.6455	IPI00221859	IPI00221859	yes	yes		2	7.4157E-06	176.95
VN(de)ITVIPSITSR	1298.7558	IPI00323857	IPI00323857	yes	no		2	0.0021966	143.25
VN(de)ISFPSAQSIPASDTHIK	2011.0375	IPI00624663	IPI00624663	yes	yes	2,3		3.3229E-05	175.8
VN(de)ISAPIIPKEDPIFTYISKR	2400.3417	IPI00761772	IPI00761772	yes	no		3	0.0077098	99.092
VN(de)ISAPIIPKEDPIFTYISK	2244.2406	IPI00761772	IPI00761772	yes	no		3	0.0085202	93.226
VN(de)GTKEPIEFK	1260.6714	IPI00345112	IPI00345112	yes	no		2	7.6285E-17	196.88
VN(de)GTITQVIIVGAPTHDDVSK	2163.1535	IPI00225715	IPI00225715	yes	yes	2,3		0.0001076	129.32
VN(de)GSCVDVDECATGGR	1694.6988	IPI00170216	IPI00170216	yes	no		2	0.0077762	100.77
VN(de)FTIEASEGCYR	1544.6929	IPI00342158	IPI00342158	yes	no		2	0.0013313	134.21
VN(de)DSVPFHIGWNSTER	1856.8806	IPI00464256	IPI00464256	yes	yes		2	0.0006438	140.22
VN(de)ASTTDPNSTVEQSAITR	1989.9603	IPI00338785	IPI00338785	yes	no		2	7.536E-38	231.86
VMSWWDYGYQITAMAN(de)R	2090.9343	IPI00109108	IPI00109108	yes	yes	2,3		3.3136E-41	216.29
VMSWWDYGYQIAGMAN(de)R	2046.908	IPI00316469	IPI00316469	yes	yes	2,3		8.9049E-20	209.66
VMEIEKEIAN(de)ATTKPEDR	2073.0412	IPI00461861	IPI00461861	yes	yes		2	0.012632	107.72
VME(de)VNFISYVVMSTAAIPMIKQSNGSIAVISSIAGK	3754.9712	IPI00115599	IPI00115599	yes	no		4	0.016235	43.831
VKPTPPYN(de)ISVTNSEEISSIIK	2415.2897	IPI00120155	IPI00120155	yes	yes		3	2.6514E-05	121.63
VKN(de)STCIDDSWIHPK	1798.8672	IPI00122584	IPI00122584	yes	yes		2	0.0075164	110.38
VIYIPAYN(de)CTIRPVSK	1893.0182	IPI00469387	IPI00469387	yes	no		2	0.010975	103.61
VIVIITDGEASDKGN(de)ISAAHDITR	2494.3027	IPI00894972	IPI00894972	yes	no		3	0.0026135	87.772
VITNQESPYQN(de)HTGR	1742.8336	IPI00125310	IPI00125310	yes	no	2,3		6.8101E-79	263.56
VITMANQVITVN(de)ISEEGR	1973.0252	IPI00987265	IPI00987265	yes	yes	2,3		4.4106E-19	205.78
VITIAN(de)FTTK	1106.6336	IPI00109727	IPI00109727	yes	yes		2	0.0007338	164.65
VISNN(de)CTSYGVIDIGK	1738.856	IPI00331214	IPI00331214	yes	yes		2	0.0009735	137.81
VISN(de)NCTSYGVIDIGK	1738.856	IPI00331214	IPI00331214	yes	yes		2	0.0009735	137.81
VISIAQAHSIN(de)FSCEQVR	2058.0317	IPI00308990	IPI00308990	yes	yes	2,3		0.0001722	145.91
VIPSIVNEVIKSVVAKFN(de)ASQIITQR	2852.6488	IPI00321718	IPI00321718	yes	no		3	5.3314E-23	174.01
VIPFN(de)VTDYCQIVR	1722.8763	IPI00828222	IPI00828222	yes	no		2	0.0055272	107.61
VINN(de)ITNDIR	1170.6357	IPI00128178	IPI00128178	yes	no		2	3.5923E-121	275.84
VINEECKEN(de)ESINIAAR	1987.9633	IPI00122411	IPI00122411	yes	yes		3	0.0004525	144.29
VINDTWAWKN(de)ATIAEQAK	2058.0534	IPI00123342	IPI00123342	yes	yes	2,3		1.1347E-78	264.03
VINADQGTSATVQMIIN(de)DTCPIFVR	2762.3731	IPI00320065	IPI00320065	yes	yes		3	1.0193E-08	127.35
VIN(de)STGHDVAR	1167.5996	IPI00849146	IPI00849146	yes	no		2	0.022901	93.623
VIN(de)ITDNTYFK	1326.682	IPI00130117	IPI00130117	yes	yes		2	2.1129E-11	185.25
VIN(de)ISHSIIDISSEQIFDGIPAIQHINIQGNHFPK	3894.0428	IPI00343568	IPI00343568	yes	yes		4	0.0025517	57.745
VIN(de)DTWAWKNATIAEQAK	2058.0534	IPI00123342	IPI00123342	yes	yes	2,3		1.1347E-78	264.03
VIN(de)DTWAWK	1131.5713	IPI00123342	IPI00123342	yes	yes		2	0.0047021	151.22
VIKDAVNN(de)ITAK	1284.7402	IPI00626793	IPI00626793	yes	no		2	0.0039361	143.25
VIIRPYITPNNQGIYIFQGN(de)STVR	2762.4868	IPI00319509	IPI00319509	yes	yes	2,3,4		3.0347E-40	231.93
VIIQSQIPIGTIKHN(de)MTYFCK	2490.3127	IPI00108003	IPI00108003	yes	no		3	0.0018697	102.58
VIINFFVGTDDKN(de)STQHIIHFDQPR	2940.4882	IPI00461861	IPI00461861	yes	no		3	0.0003103	106.42
VIIN(de)ITTVAANHGYTK	1713.9414	IPI00467600	IPI00467600	yes	no		2	9.6934E-20	209.6
VIIIIDPAISGN(de)ETEPYPAFTR	2415.2686	IPI00848693	IPI00848693	yes	yes		2	2.3984E-14	197
VIHIQFNSISSITDDTFCKAN(de)DTR	2781.3392	IPI00120848	IPI00120848	yes	yes		3	0.000104	111.66
VIFYKDDAMVYN(de)VTSR	1919.9451	IPI00406901	IPI00406901	yes	no	2,3		0.0001565	157.86
VIETIPANYSIN(de)SSKK	1762.9465	IPI00480532	IPI00480532	yes	no		2	0.023899	93.478
VIETIPAN(de)YSINSSKK	1762.9465	IPI00480532	IPI00480532	yes	no		2	0.023899	93.478
VIENEKFDTHEYHN(de)ESR	2145.9716	IPI00463492	IPI00463492	yes	yes	2,3		5.0475E-19	207.92
VIEEPKN(de)VSCETR	1559.7614	IPI00118291	IPI00118291	yes	no		2	0.0091086	109.44

VIEEFYN(de)QTWNHR	1734.8114	IPI00308691	IPI00308691	yes	no		2	2.4065E-80	257.76
VIDPDFHENYFEQYMDHFNFESFGN(de)KTFGQR	3857.6845	IPI00331550	IPI00331550	yes	yes		4	0.0096288	59.628
VIDIWDIAQSAN(de)FTEKEIESFREEIK	3109.5608	IPI00469307	IPI00469307	yes	no		3	0.0011839	89.663
VIDIWDIAQSAN(de)FTEK	1848.9258	IPI00469307	IPI00469307	yes	no	2,3		0.0001178	163.99
VIAQHQNIIFAN(de)SSSSMR	2002.0054	IPI00319040	IPI00319040	yes	no	2,3		0.004768	101.42
VHSGN(de)FSTIPQYFK	1623.8045	IPI01023131	IPI01023131	yes	no		2	0.018613	90.444
VHITVCN(de)ITSR	1298.6765	IPI00128862	IPI00128862	yes	no		2	0.004449	122.7
VHGPN(de)ASHYTSIMTMITWER	2330.0936	IPI00169617	IPI00169617	yes	yes		3	4.5705E-05	147.09
VGYQSQN(de)ISCFFR	1604.7406	IPI00464256	IPI00464256	yes	yes		2	1.6243E-52	236.28
VGVNKN(de)QTVTATFGYPFR	1998.0323	IPI00114256	IPI00114256	yes	no	2,3		9.4205E-62	246.12
VGNVECGEGHFCHDN(de)QTCCK	2406.9198	IPI00124640	IPI00124640	yes	yes		3	0.026701	70.334
VGN(de)NTVHVHQEVHK	1596.8121	IPI00131904	IPI00131904	yes	yes		2	0.020113	89.46
VGDEYQEIQIDGFDN(de)ESSNKTWMK	2832.2549	IPI00114671	IPI00114671	yes	no		3	1.7707E-06	136.58
VGATAAVYSAAIIEYITAEVIEIAGN(de)ASK	2894.5277	IPI00555055	IPI00555055	yes	no		3	2.0608E-89	258.31
VGAPEN(de)VTVQAHGHTEAFDTTVSVK	2593.2772	IPI00405543	IPI00405543	yes	yes		3	0.02367	58.093
VFVYTPTTN(de)YTIR	1573.814	IPI00124428	IPI00124428	yes	yes		2	3.9692E-10	181.66
VFPYISVMVNN(de)GSISYDHSKDGR	2584.238	IPI00321634	IPI00321634	yes	yes	2,3		9.9123E-06	150.68
VFPYISVMVNN(de)GSISYDHSK	2256.0885	IPI00321634	IPI00321634	yes	yes	2,3		1.2614E-05	180.31
VFPYISAMVNN(de)GSISYDHER	2298.0739	IPI00830356	IPI00830356	yes	no		2	0.0029694	98.816
VFNGKDN(de)ISK	1120.5877	IPI00331214	IPI00331214	yes	yes		2	1.9321E-05	174.4
VFKTN(de)STQVSDVR	1479.7682	IPI00469426	IPI00469426	yes	no		2	0.0041444	119.96
VFKPQSGADAIN(de)DSQDFPFPETPAK	2705.2973	IPI00462465	IPI00462465	yes	no		3	0.0011846	83.31
VFIVPVGN(de)HSNIPFSR	1781.9577	IPI00221418	IPI00221418	yes	no	2,3		8.9441E-05	159.18
VFHIHN(de)ESWVIITPK	1818.9781	IPI00224752	IPI00224752	yes	yes		2	6.9009E-15	199.31
VFGSQN(de)ITTVK	1192.6452	IPI00123342	IPI00123342	yes	yes		2	8.371E-57	240.85
VFDIHQNMGSVN(de)VSVGCTPAQIIETSR	2958.4328	IPI00116913	IPI00116913	yes	no		3	1.7305E-06	117.22
VETGVIKPGMVVTFAPVN(de)VTTEVK	2514.3767	IPI00307837	IPI00307837	yes	yes		3	0.0087711	76.015
VEFDDKGNVITSYGNPIIIN(de)SSIPEDATIK	3248.6453	IPI00122257	IPI00122257	yes	no		3	3.5047E-14	140.26
VDVIVAN(de)ITVTDKDQPHTPAWNAAYR	2893.4723	IPI00323134	IPI00323134	yes	no		3	1.0189E-12	189.82
VDIPQQPMGIIAVANDTN(de)SCEISPCR	2884.3517	IPI00119063	IPI00119063	yes	yes		3	0.0002154	102.21
VDIEDFEN(de)NTAYAK	1627.7366	IPI00551169	IPI00551169	yes	no		2	9.3057E-39	228.03
VDFIWHPEVN(de)GSMK	1657.7923	IPI00380296	IPI00380296	yes	yes		2	0.0084592	102.73
VDDEMPQHAVISGPNIFINNINKTDN(de)GTYR	3371.6205	IPI00856723	IPI00856723	yes	no	3,4		3.1201E-12	161.22
VDDEMPQHAVISGPNIFINNIN(de)KTDNGTYR	3371.6205	IPI00856723	IPI00856723	yes	no	3,4		3.1201E-12	161.22
VCSNDN(de)KTFDSSCHFFATK	2263.9627	IPI00126343	IPI00126343	yes	no		3	0.0055482	100.04
VCNGIG(de)IGEFKDTISINATNIK	2363.2155	IPI00121190	IPI00121190	yes	no		3	0.0055462	92.492
VCNG(de)IGIGEFKDTISINATNIK	2363.2155	IPI00121190	IPI00121190	yes	no		3	0.0055462	92.492
VAQPGINYAIGTN(de)TSYPNNIIR	2375.2234	IPI00226360	IPI00226360	yes	no	2,3		2.8336E-09	177.44
VAEVENGTKPDSDVPEHCIDTWSFDAATMDHN(de)GTMIFFK	4410.9508	IPI00128484	IPI00128484	yes	yes	4,5		1.7298E-10	91.115
VAEVEN(de)GTKPDSDVPEHCIDTWSFDAATMDHNGTMIFFK	4410.9508	IPI00128484	IPI00128484	yes	yes	4,5		1.7298E-10	91.115
TV(de)VTEAGNIIKDNATQEEIIHYIEK	2827.4604	IPI00321190	IPI00321190	yes	no		3	7.6664E-06	117.38
TSDTGEEEAITN(de)STEANGK	1952.8447	IPI00408495	IPI00408495	yes	no		2	1.6651E-27	218.37
TQITCSIN(de)SSGVDIVGHR	1942.9531	IPI00408495	IPI00408495	yes	no	2,3		0.0003357	158.92
TN(de)SSFIQGFVDHVK	1577.7838	IPI00321190	IPI00321190	yes	no		2	0.037605	79.771
TIYN(de)WSGYPIIVHK	1689.8879	IPI00471081	IPI00471081	yes	yes		2	2.8331E-15	199.13
TITIIN(de)VTR	1029.6182	IPI00108535	IPI00108535	yes	no		2	0.0069093	140.49
TIIGYYN(de)QSAGGTHTIQWMYGCDVGSDGR	3206.4186	IPI00109996	IPI00109996	yes	yes		3	4.5278E-06	91.722
TIGISPFHEFADVVFTAN(de)DSGHR	2516.2084	IPI00127560	IPI00127560	yes	yes		3	4.5869E-06	127.59
TGRVYN(de)VTQHAMGIIVNKQVK	2355.2845	IPI00468196	IPI00468196	yes	no		3	0.0009363	108.18
TAASIIWQAYPIIN(de)ISEK	2017.0884	IPI00115867	IPI00115867	yes	yes	2,3		1.4387E-49	243.01
STIIIHHPQYAWIQDIGIREDNEGVYN(de)GSWGGR	3780.8397	IPI00230084	IPI00230084	yes	no		4	1.3996E-19	143.25
SN(de)VTRPSEFNYIWIAPIPFIK	2491.3264	IPI00319509	IPI00319509	yes	no		3	0.0025674	100.77
SIIDHIHVGVGRDN(de)VSQPK	2070.097	IPI00123428	IPI00123428	yes	no	2,3		0.0022178	110.56
SIAQYIHSGPPVYFVIEEGYN(de)YSSR	2875.3817	IPI00132600	IPI00132600	yes	no		3	0.0003017	90.884
SFIISIAAIHDN(de)HTHSDIQVK	2345.2128	IPI00130624	IPI00130624	yes	yes		3	9.708E-05	136.34
RYIKNG(de)NATIIR	1417.8154	IPI00985828	IPI00985828	yes	no	2,3		0.0059853	136.38
RYIK(de)NGNATIIR	1417.8154	IPI00985828	IPI00985828	yes	no	2,3		0.0059853	136.38
RYIEIG(de)NETIIR	1475.8096	IPI00850057	IPI00850057	yes	no	2,3		9.9537E-26	212.9
RVNDN(de)KTAAEEAIR	1585.8172	IPI00400016	IPI00400016	yes	no		2	6.2469E-15	201.06
RVM(de)EVNFISYVVMSTAAIPMIKQSNGSIAVISSIAGK	3911.0723	IPI00115599	IPI00115599	yes	no		4	0.0013988	57.076
RVIYIPAY(de)NCTIRPVSK	2049.1194	IPI00469387	IPI00469387	yes	no	2,3		0.0002355	168.1
RV(de)NYTRAEEIFSR	1639.8431	IPI00131168	IPI00131168	yes	yes		2	0.013461	115.34
RSN(de)FTPATNEAPQATVFPK	2075.0436	IPI00172039	IPI00172039	yes	no	2,3		0.0015493	115.87
RQDVN(de)ITVATVPTWIR	1868.0268	IPI00222589	IPI00222589	yes	yes		2	0.0015544	138.39
RQDPVSWN(de)KTFEDISR	1976.9704	IPI00848693	IPI00848693	yes	yes	2,3		0.0007861	145.86
RPYIVPIIWIN(de)ETGTIGDEKAEMFK	2919.5205	IPI00331214	IPI00331214	yes	yes		3	3.7248E-07	155.25
RPYIVPIIWIN(de)ETGTIGDEK	2313.2369	IPI00331214	IPI00331214	yes	yes		3	0.000765	115.87
RPFGVVYEMEVDTIETTCHAIDPTPIAN(de)CSVR	3676.7324	IPI00128249	IPI00128249	yes	yes	3,4		3.8486E-09	96.387
RNPSANTFIHIN(de)ASSFR	1930.9762	IPI00315535	IPI00315535	yes	yes	2,3		1.3609E-05	179.81
RNESHWVSISTAN(de)FTSPVIEFWER	2891.3991	IPI00314054	IPI00314054	yes	yes		3	8.0457E-06	171.06
RN(de)WTINRINGDFAQINIK	2172.1552	IPI00323134	IPI00323134	yes	no		3	0.0029545	138.57
RN(de)FTAADWGHSR	1416.6647	IPI00339885	IPI00339885	yes	no	2,3		0.0022909	129.54
RN(de)ESHWVSISTANFTSPVIEFWER	2891.3991	IPI00314054	IPI00314054	yes	yes		3	8.0457E-06	171.06
RMHIN(de)GSNVQVIHR	1659.874	IPI00119063	IPI00119063	yes	yes		3	7.3463E-21	206.18
RIYAGMVSIMDEAVGN(de)VTK	2053.0336	IPI00652358	IPI00652358	yes	no		2	0.043796	64.298
RIPVTN(de)ISQIHK	1404.8201	IPI00469426	IPI00469426	yes	no		3	0.0029409	137.84
RIPPTN(de)FTR	1100.6091	IPI00320618	IPI00320618	yes	yes		2	0.017449	133.13
RIGAI(de)NNSIIIIEDR	1695.9632	IPI00115516	IPI00115516	yes	no		3	0.0018791	126.63
RIFPSGN(de)VTSVIEISSNPAHR	2280.1975	IPI00798466	IPI00798466	yes	no		3	0.0069567	92.897
RHEEGHMIN(de)CTCFGQGR	2087.8836	IPI00113539	IPI00113539	yes	no	2,3		0.012767	101.01
RGVFITN(de)ETGQPIIGK	1728.9523	IPI00111960	IPI00111960	yes	no	2,3		0.0001634	166.3
RGTFTDCAIAN(de)MTQQIR	1981.9462	IPI00621027	IPI00621027	yes	no	2,3		0.0002696	156.57
RGPECSQN(de)YTAPTGVIK	1876.9101	IPI00123996	IPI00123996	yes	yes	2,3		0.0002738	157.58
RGDDIYTN(de)VTVSIVEAIVGFEMDITHIDGHK	3443.7031	IPI00320241	IPI00320241	yes	yes	3,4,5		4.7885E-14	173.36
RGCKDN(de)ATDSVPIR	1587.7787	IPI00119063	IPI00119063	yes	yes		2	0.023466	106.52
RFHSDIN(de)ISESIIPAVIEK	2167.1637	IPI00831484	IPI00831484	yes	yes		3	6.4392E-08	186.72

RDQGN(de)VTDMASMK	1451.6497	IPI00471081	IPI00471081	yes	yes	2,3		0.0010871	132.79
RDDYRPTWTI(de)NQTEPVAGNYYPVNTR	3125.4955	IPI00381303	IPI00381303	yes	yes		3	3.9104E-08	160.2
RDDIHPTIPAGQYFI(de)NITYNYPVHSFDGR	3405.6531	IPI00387318	IPI00387318	yes	no	3,4		8.4627E-126	275.54
RAN(de)ASTFAVPSPVSNSADTR	2047.0083	IPI00119065	IPI00119065	yes	yes	2,3		0.007435	94.845
RAEDYGPVEVISHWHPN(de)ITINIVDDHTPWVK	3636.8114	IPI00121627	IPI00121627	yes	yes		4	1.3908E-13	101.73
RADIN(de)GSNMETVIGHGIK	1910.9632	IPI00411145	IPI00411145	yes	no		3	0.0046507	111.33
QVN(de)GSVSGSQWNK	1389.6637	IPI00408850	IPI00408850	yes	yes		2	0.0031303	116.51
QVEIIEYPYHEQIAVVAPEPIITGHN(de)YTIK	3463.8028	IPI00223987	IPI00223987	yes	yes	3,4		4.8495E-15	164.82
QVEEIIVN(de)HTGIR	1506.8154	IPI00263041	IPI00263041	yes	yes		2	0.0012981	143.94
QVAIQTFGN(de)QTSIIPAGGAGYK	2220.1539	IPI00228719	IPI00228719	yes	yes	2,3		9.621E-159	299.69
QTTAMDFSYAN(de)ETVCWVHVGDSAAQTQIK	3257.4758	IPI00119063	IPI00119063	yes	no		3	0.0036047	65.213
QTQVGIVQYGAN(de)VTHEFNINK	2359.1921	IPI00466371	IPI01026704	no	no	2,3		4.9216E-13	193.85
QSQPVHIIPMN(de)ETDHINMVFSNK	2678.2945	IPI00133500	IPI00133500	yes	yes		3	0.025072	56.327
QSN(de)GSIAVISSIAGK	1430.7729	IPI00115599	IPI00115599	yes	no		2	1.6909E-38	229.68
QSINTVN(de)DTVWK	1403.7045	IPI00420867	IPI00420867	yes	yes		2	0.0064483	105.65
QRYN(de)ITAK	992.54033	IPI00130661	IPI00130661	yes	yes		2	5.3683E-30	219.51
QQCIEEAQIEN(de)ETTGCSK	2123.9099	IPI00128984	IPI00128984	yes	yes		2	5.1988E-62	246.21
QPIYIN(de)CSCVTGGSASAK	1911.8819	IPI00309249	IPI00309249	yes	yes		2	0.0001746	145.43
QNFSN(de)ITVSTEDQVK	1708.8268	IPI00129677	IPI00129677	yes	no		2	3.043E-260	338.8
QN(de)FSNITVSTEDQVK	1708.8268	IPI00129677	IPI00129677	yes	no		2	3.043E-260	338.8
QKNVNISYI(de)VNDSFFPQRPEK	2522.2918	IPI00122557	IPI00122557	yes	no	2,3		0.0002739	175.8
QKN(de)VNISYIVNDSFFPQRPEK	2522.2918	IPI00122557	IPI00122557	yes	no	2,3		0.0002739	175.8
QINN(de)VSAMIVIAR	1427.7919	IPI00944049	IPI00944049	yes	no		2	0.0009616	130.56
QIN(de)ITTEDDDIYHMTVPYGRPR	2633.2544	IPI00265291	IPI00265291	yes	yes		3	0.0077777	88.19
QIITEEKIPN(de)NTQWITWSPEGHK	2748.3871	IPI00125813	IPI00125813	yes	no	2,3		8.4295E-52	242.59
QIIIAN(de)NSIR	1140.6615	IPI00128024	IPI00128024	yes	yes		2	0.014411	110.53
QIIAN(de)SSAIEETIIGHQGR	2036.0651	IPI00116913	IPI00116913	yes	no	2,3		0.0015106	131.67
QIGASPSDDIIFGVFAQSKPDSAEPVN(de)R	2944.4567	IPI00130420	IPI00130420	yes	no		4	3.2916E-14	129.9
QIFFN(de)GTETIR	1324.6776	IPI00338209	IPI00338209	yes	no		2	0.014852	100.04
QHGQFSIAVVGIN(de)ITSIGIR	2109.1695	IPI00121190	IPI00121190	yes	no		3	0.000263	158.64
QGPQAGGTTITIN(de)GTHIDTGSKEDVR	2652.3103	IPI00405742	IPI00405742	yes	no	2,3		6.0153E-06	116.43
QGPQAGGTTITIN(de)GTHIDTGSK	2153.0713	IPI00405742	IPI00405742	yes	no	2,3		0.0001909	117.48
QGDQYSCMVGHEAIPMN(de)FTQK	2440.061	IPI00968978	IPI00968978	yes	no	2,3		4.8198E-05	125.73
QFWIFDVQNPDDVAKN(de)SSK	2237.0753	IPI00331214	IPI00331214	yes	yes	2,3		1.4403E-12	197.67
QFNCSFEN(de)ITR	1414.6299	IPI00674255	IPI00674255	yes	yes		2	0.0053084	117.79
QFN(de)CSFENITR	1414.6299	IPI00674255	IPI00674255	yes	yes		2	0.0053084	117.79
QENNIIIINAEHGNSSIFIEN(de)STFESFGYHSVSPDR	4064.9141	IPI00125813	IPI00125813	yes	no		4	0.018637	45.685
QENNIIIINAEHGN(de)SSIFIENSTFESFGYHSVSPDR	4064.9141	IPI00125813	IPI00125813	yes	no		4	0.018637	45.685
QEMNGTYVCHAFSSHGN(de)VTR	2293.9957	IPI00122973	IPI00122973	yes	no		3	0.014728	68.243
QDIAISGN(de)ISSIYAMTQDK	2053.999	IPI00405742	IPI00405742	yes	no	2,3		2.0348E-19	208.37
QAIQTMQSEFFYITTNIIN(de)DTIEIR	2988.4903	IPI00118130	IPI00118130	yes	yes		3	1.2407E-18	167.73
PSWGN(de)HTPIFR	1310.652	IPI00117312	IPI00117312	yes	yes		2	3.7829E-11	187.85
PINETFPVVYIE(de)TPKR	1902.0251	IPI00624663	IPI00624663	yes	yes	2,3		0.0001097	151.56
PINETFPVVYIE(de)TPK	1745.924	IPI00624663	IPI00624663	yes	yes		2	0.031176	106.82
PGAASN(de)ISFQAPFR	1461.7365	IPI00311159	IPI00311159	yes	yes		2	1.0425E-10	186.56
PFFIIQN(de)SSMMK	1441.7098	IPI00226229	IPI00226229	yes	no		2	0.018297	81.625
NYTAN(de)ATSSREEAWDYVQAQVK	2530.1724	IPI00108098	IPI00108098	yes	no		3	3.8605E-06	175.8
NYTAN(de)ATSSREEAWDYVQAQVK	2530.1724	IPI00108098	IPI00108098	yes	no		3	3.8605E-06	175.8
NYN(de)FTIACNTKR	1500.7143	IPI00322575	IPI00322575	yes	no		2	0.0026052	127.17
NYN(de)FTIACNTK	1344.6132	IPI00322575	IPI00322575	yes	no		2	0.000637	156.36
NYKNPN(de)ITISFTAER	1766.8951	IPI00132600	IPI00132600	yes	no	2,3		4.9746E-08	157.56
NYFHYN(de)QSFPPSYNIK	2017.9323	IPI00129265	IPI00129265	yes	yes		2	7.8109E-48	205.98
NYEVQIFHVN(de)ATVTEEGTGIEFSR	2739.314	IPI00123223	IPI00123223	yes	yes		3	2.3732E-06	124.86
NVVN(de)ITINDEAAMVEAVAIYNPVSFAFEVTEDFIMYK	4166.0268	IPI00118987	IPI00118987	yes	yes		4	1.1042E-10	91.603
NVNISYTVN(de)DSFFPQRPQKIIANK	2792.461	IPI00381178	IPI00381178	yes	yes		3	0.0002921	111.18
NVNISYTVN(de)DSFFPQRPQK	2253.1178	IPI00381178	IPI00381178	yes	yes	2,3		9.7631E-205	319.55
NVNISYIVN(de)DSFFPQRPEK	2266.1382	IPI00122557	IPI00122557	yes	no	2,3		3.2743E-94	269.49
NVNI(de)SYTVNDSFFPQRPQKIIANK	2792.461	IPI00381178	IPI00381178	yes	yes		3	0.0002921	111.18
NVNI(de)SYTVNDSFFPQRPQK	2253.1178	IPI00381178	IPI00381178	yes	yes	2,3		9.7631E-205	319.55
NVN(de)ISYIVNDSFFPQRPEK	2266.1382	IPI00122557	IPI00122557	yes	no	2,3		3.2743E-94	269.49
NVAIVAGDTGN(de)ATGIGEQGPTR	2097.0451	IPI00122272	IPI00122272	yes	no		2	6.1857E-98	273.33
NTQADVINASWSVISN(de)STRHEIER	2726.3372	IPI00132189	IPI00132189	yes	yes		3	2.4378E-06	132.74
NTQADVINASWSVISN(de)STR	2062.0079	IPI00132189	IPI00132189	yes	yes	2,3		0	370.36
NTQADVIN(de)ASWSVISNSTRHEIER	2726.3372	IPI00132189	IPI00132189	yes	yes		3	2.4378E-06	132.74
NTQADVIN(de)ASWSVISNSTR	2062.0079	IPI00132189	IPI00132189	yes	yes	2,3		0	370.36
NTGEIN(de)ITSIIDREETPYFIITGYAIDSR	3300.6514	IPI00115793	IPI00115793	yes	no		3	6.3269E-14	179.33
NSS(de)IGGVINKYDVVIR	1732.9472	IPI00314673	IPI00314673	yes	yes		2	0.0001782	149.97
NSQFDMN(de)STDIAIK	1582.7297	IPI00346062	IPI00346062	yes	yes		2	0.0001274	155.57
NSNSN(de)VIQVDQSGIGIPSRDYYINKTENEK	3381.6437	IPI00396840	IPI00396840	yes	no		3	1.5839E-07	115.11
NSAEN(de)QTHQTFITVEK	1845.8857	IPI00458077	IPI00458077	yes	yes		2	1.5651E-08	183.7
NRCDNN(de)DTIIVR	1488.7103	IPI00467600	IPI00467600	yes	yes		2	0.0019377	133.15
NQKDEIN(de)ETDIK	1445.6998	IPI00930882	IPI00930882	yes	no		2	0.0097888	109.15
NQIVEI(de)EKVVIHPNHSVVDIGIIK	2692.5276	IPI00409148	IPI00409148	yes	yes		3	8.9427E-10	186.15
NPSANTFIHINA(de)SSFR	1774.8751	IPI00315535	IPI00315535	yes	yes		2	1.6649E-62	250.52
NPNN(de)NTIHPNIR	1402.7066	IPI00319509	IPI00319509	yes	no		2	5.5191E-34	223.31
NPIGQAQSAVVIN(de)VTER	1794.9588	IPI00119299	IPI00119299	yes	no		2	0.0018319	129.43
NPEDVQSPN(de)GSVYTWR	1847.8438	IPI00112614	IPI00112614	yes	yes		2	4.6354E-05	170.09
NPCTSEQN(de)CTSPFSYK	1918.7826	IPI00132474	IPI00132474	yes	yes		2	7.9507E-155	296.13
NPCNITREDY(de)APIVK	1788.8829	IPI00307966	IPI00307966	yes	yes		2	0.0002748	148.52
NNQT(de)ECFNHVR	1417.6157	IPI00124666	IPI00124666	yes	no		2	0.0010284	149.49
NNIHFPGHN(de)YTTR	1569.7437	IPI00130271	IPI00130271	yes	no		2	0.0023777	119.69
NMVIQN(de)GTK	1003.5121	IPI00127447	IPI00127447	yes	yes		2	0.010106	135.43
NMICQWDPGRETYIETN(de)YTIK	2631.2098	IPI00120155	IPI00120155	yes	yes		3	1.0367E-05	180.62
NKNPQMN(de)FTEANEACK	1894.8302	IPI00308245	IPI00308245	yes	yes	2,3		6.5912E-13	198.23
NKANIQFGEN(de)GTTISAVTNK	2106.0705	IPI00127447	IPI00127447	yes	yes	2,3		5.8504E-63	252.05
NITVIEPVTQPFIQVTN(de)TTVK	2341.2893	IPI00108535	IPI00108535	yes	no		2	2.3893E-29	223.46

NITAFN(de)ETIFR	1324.6776	IPI00124700	IPI00124700	yes	yes		2	4.9182E-44	224.66
NISTCFSSGDIFAAHN(de)ISER	2225.0171	IPI00123428	IPI00123428	yes	no		2	3.1105E-19	204.47
NISFACNPGFFIN(de)GTSSSK	2046.9469	IPI00322463	IPI00322463	yes	no		2	2.485E-62	247
NISFACNPG(de)FFINGTSSSK	2046.9469	IPI00322463	IPI00322463	yes	no		2	2.485E-62	247
NIQAVNEIIATISQCN(de)DTSSAAMVQCIR	3106.4846	IPI00138342	IPI00138342	yes	no		4	0.0006064	72.935
NIPWYVIAGNHDHIGN(de)VSAQIAYSK	2766.3878	IPI00137491	IPI00137491	yes	yes		3	0.0015675	77.575
NIN(de)SSCRPHPGAWIR	1763.8638	IPI00222809	IPI00222809	yes	no	2,3		0.017012	100.22
NIMTIVHFYN(de)K	1378.7067	IPI00119822	IPI00119822	yes	no		2	0.0040741	127.87
NIMIDIQKDTAV(de)EGEEIEVNCTAMASKPATTIR	3647.7845	IPI00856723	IPI00856723	yes	no		4	1.3358E-11	118.9
NIKRPYIVPII(de)WINETGTIGDEK	2668.4588	IPI00331214	IPI00331214	yes	yes		3	1.4778E-09	157.85
NIIYHNIGIITKPGTIIPTDRN(de)SSMCTVMR	3414.7574	IPI00463492	IPI00463492	yes	yes		4	8.5522E-06	84.68
NIINDYVSN(de)QTQGMIKEIISEIDER	2921.4441	IPI00131830	IPI00131830	yes	no	3,4		2.4334E-09	180.13
NIINDYVSN(de)QTQGMIK	1836.904	IPI00131830	IPI00131830	yes	no	2,3		2.1565E-156	304.23
NIIGIDENFVIN(de)SSTEHR	2057.0178	IPI00406603	IPI00406603	yes	yes	2,3		0.000155	149.52
NIGN(de)TSEGPR	1043.4996	IPI00221706	IPI00221706	yes	yes		2	0.0018523	152.54
NIGGIETEDDYGYQGHVQTCN(de)FSAQMAK	3132.3553	IPI00126769	IPI00126769	yes	yes		3	1.8821E-11	142.79
NIFIN(de)HSETASAK	1430.7154	IPI00409148	IPI00409148	yes	yes	2,3		7.2418E-98	273.72
NHNGQGYKDQDPASFGNNSIIN(de)SSR	2832.3175	IPI00848693	IPI00848693	yes	yes		3	1.39E-05	114.92
NHNGQGYKDQDPASFGN(de)NSIINSSR	2832.3175	IPI00848693	IPI00848693	yes	yes		3	1.39E-05	114.92
NHAVSFDPIFSAVKN(de)FSEAASDFHR	2792.3307	IPI00113042	IPI00113042	yes	no	3,4		3.9947E-17	194.77
NGNATI(de)IR	857.47191	IPI00985828	IPI00985828	no	no		2	0.014287	144.12
NGNATI(de)IR	857.47191	IPI00985828	IPI00985828	no	no		2	0.014287	144.12
NGNA(de)TIIR	857.47191	IPI00985828	IPI00985828	no	no		2	0.014287	144.12
NGNA(de)TIIR	857.47191	IPI00985828	IPI00985828	no	no		2	0.014287	144.12
NGIASGSGN(de)CSTGPNGDGSFHAWSIIEVK	2918.3253	IPI00322497	IPI00322497	yes	yes		3	0.011282	60.86
NGHITN(de)YTVVYR	1435.7208	IPI00110264	IPI00110264	yes	yes		2	2.5942E-14	189.24
NF(de)TAADWGHSR	1260.5636	IPI00339885	IPI00339885	yes	no		2	0.0020817	137.16
NF(de)NVEKINGEWHTIIIASDKR	2483.2921	IPI00944716	IPI00944716	no	no		3	5.4531E-19	204.94
NEVN(de)GTSEDIKSEVQRK	1931.9548	IPI00122418	IPI00122418	yes	no		2	0.015437	109.07
NEKPRPAVN(de)VTCAR	1610.8311	IPI00944683	IPI00944683	yes	yes		3	0.033205	101.32
NEIMIN(de)SSIMR	1306.6373	IPI00378698	IPI00378698	yes	no		2	0.0007552	140.14
NEEVKN(de)MSVEINSKIESIIEEK	2561.2894	IPI00128206	IPI00128206	yes	yes		3	5.4392E-05	147.25
NEEVKN(de)MSVEINSK	1619.7825	IPI00128206	IPI00128206	yes	yes		2	0.0027995	128.71
NEDRPIAPCGAIANSMFNDTIEIYIVAN(de)ESDPKPIPIPIK	4422.224	IPI00387318	IPI00387318	yes	no		4	0.0017113	52.377
NEDRPIAPCGAIANSMFN(de)DTIEIYIVANESDPKPIPIPIK	4422.224	IPI00387318	IPI00387318	yes	no		4	0.0017113	52.377
NAVN(de)CTYKNEDDCVVR	1955.8466	IPI00266264	IPI00266264	yes	yes	2,3		0.0001247	168.1
NATIAEQAKI(de)PATEKPVIISK	2221.2682	IPI00123342	IPI00123342	yes	yes	2,3		0.0004111	168.05
NAKGDEKEN(de)ITAEAIDISIK	2158.1117	IPI00124725	IPI00124725	yes	no	2,3		8.2716E-37	224.59
NAIQAFGN(de)GTDVNMSPK	1762.8308	IPI00116355	IPI00116355	yes	no		2	6.3295E-05	171.78
NAIN(de)ITFHAQNIGEGGAYEAEIR	2487.2142	IPI00115976	IPI00115976	yes	yes		3	2.3728E-51	237.24
NACCSVN(de)TSQEIHK	1646.7141	IPI00113158	IPI00113158	yes	yes		2	0.012759	95.981
N(de)YTDCTSEGR	1201.467	IPI00113539	IPI00113539	yes	no		2	0.010676	118.28
N(de)YSIFIADINQER	1581.7787	IPI00331318	IPI00331318	yes	no		2	5.9377E-06	165.86
N(de)VTYGTYIDDPDPDDGFNYK	2307.9808	IPI00399958	IPI00399958	yes	no		2	3.0119E-157	304.09
N(de)VTIECPFKR	1262.6441	IPI00310059	IPI00310059	yes	no		2	7.9364E-42	227.88
N(de)VSCIWCNENK	1422.602	IPI00377642	IPI00377642	yes	no		2	0.0021936	136.38
N(de)TTSYPPMCSQDAVGGQVISEIFTNRK	2999.4117	IPI00387289	IPI00387289	yes	yes		3	0.0008156	78.272
N(de)TTSYPPMCSQDAVGGQVISEIFTNR	2871.3167	IPI00387289	IPI00387289	yes	yes		3	0.011992	63.883
N(de)TTSAAMVHCIR	1359.6387	IPI00131216	IPI00131216	yes	yes		2	0.0021394	122.33
N(de)TTIYIDRAEAK	1393.7201	IPI00468539	IPI00468539	yes	no		2	0.011404	105.95
N(de)TTISVHPSTR	1211.6258	IPI00126834	IPI00126834	yes	no		2	0.005201	117.2
N(de)TTHIIQR	981.53558	IPI00321222	IPI00321222	yes	yes		2	0.037975	104.2
N(de)STTIVMHMK	1160.5682	IPI00119063	IPI00119063	yes	yes		2	0.0016259	136.96
N(de)STKEEIIAAIEK	1444.7773	IPI00321190	IPI00321190	yes	no		2	1.3928E-13	185.27
N(de)STIQAANIAGIK	1299.7147	IPI00222937	IPI00222937	yes	no		2	0.0005135	154.1
N(de)SSNFHINQIQGIR	1626.8227	IPI00323235	IPI00323235	yes	yes		2	0.0009665	139.48
N(de)RTDVEYEIDEK	1509.6947	IPI00134743	IPI00134743	yes	yes		2	0.0029528	124.45
N(de)QTIEIHVIYGPR	1538.8205	IPI00122973	IPI00122973	yes	no		2	3.0456E-53	242.48
N(de)QSVGDPNVDIIR	1425.7212	IPI00127447	IPI00127447	yes	yes		2	5.031E-14	188.13
N(de)ITVIEPVTQPFIQVTNTTVK	2341.2893	IPI00108535	IPI00108535	yes	no		2	2.3893E-29	223.46
N(de)ITSPVGVQPIINEHTFCAGITK	2495.2842	IPI00409148	IPI00409148	yes	yes	2,3		1.3463E-06	175.86
N(de)ITMFISR	980.51134	IPI00225715	IPI00225715	yes	yes		2	0.031589	111.61
N(de)ITFQGPIPK	1113.6182	IPI00111794	IPI00111794	yes	no		2	0.0089509	123.51
N(de)ISVVVATHSPTIAK	1535.8671	IPI00221418	IPI00221418	yes	no		2	1.4782E-14	194.55
N(de)ISTCFSSGDIFAAHNISER	2225.0171	IPI00123428	IPI00123428	yes	no		2	3.1105E-19	204.47
N(de)ISGVVIADHSGSFHNR	1808.8918	IPI00118674	IPI00118674	yes	yes		2	7.0548E-05	153.75
N(de)HSIPIETK	1037.5506	IPI00129041	IPI00129041	yes	no		2	0.0037005	162.88
N(de)ETHSICSACDESCK	1796.6764	IPI00111286	IPI00111286	yes	yes		2	0.001086	128.71
N(de)CSTQHFPR	1145.5036	IPI00322304	IPI00322304	yes	yes		2	0.017048	122.26
N(de)ATYGHYEPGEEFHDVEDAETYKK	2828.2202	IPI00221798	IPI00221798	yes	no		3	1.6789E-06	148.75
N(de)ATVVWMKDNIR	1445.7449	IPI00121378	IPI00121378	yes	no		2	0.014264	103.88
N(de)ATVVWMK	947.48987	IPI00121378	IPI00121378	yes	no		2	0.012987	147.19
N(de)ATTYPPMCSQDAAR	1681.7188	IPI00128399	IPI00128399	yes	yes		2	4.1098E-08	181.26
N(de)ATSYPPMCSQDAGWAK	1882.7978	IPI00138342	IPI00138342	yes	yes		2	0.0001784	148.52
N(de)ATIVNEADKIR	1342.7205	IPI00319509	IPI00319509	yes	no		2	2.0685E-05	168.31
N(de)ATFSIANPVYVGNAAWAHIIAAR	2526.3132	IPI00971286	IPI00971286	yes	no		3	0.030765	67.227
N(de)ASSEYSGTYSCTVQNR	1922.8065	IPI00270376	IPI00270376	yes	no		2	5.3731E-77	258.14
MYVTN(de)DTEVAENNYEAIKDFFR	2668.2115	IPI00658539	IPI00658539	yes	no	2,3		3.2069E-09	183.56
MYSEGSDIVPQSN(de)ETAIHYFKK	2543.2002	IPI00131143	IPI00131143	yes	no	2,3		4.4945E-05	157.54
MYSEGSDIVPQSN(de)ETAIHYFK	2415.1053	IPI00131143	IPI00131143	yes	no	2,3		0.0001959	159.19
MVN(de)TTFICTATNAVGTGR	1912.9135	IPI00109375	IPI00109375	yes	no	2,3		0	330.54
MVN(de)HSIHPTEPVK	1487.7555	IPI00750217	IPI00750217	yes	no	2,3		0.0015286	134.68
MTYFVCTQIKVVRN(de)NSQVPK	2411.2454	IPI00988381	IPI00988381	yes	yes		3	0.033612	73.208
MTQIIGITPN(de)ATHIHRPPR	2152.1688	IPI00130325	IPI00130325	yes	yes		3	0.0098144	96.489
MTISQN(de)NSIIR	1275.6605	IPI00108535	IPI00108535	no	no		2	7.4578E-85	257.8
MSVINFECN(de)KTAGK	1597.7592	IPI00308971	IPI00308971	yes	yes		2	0.0001402	158.52

MSVINFECN(de)K	1240.558	IPI00308971	IPI00308971	yes	yes		2	0.036661	94.465
MSQHPIDIQEDFNNATIVN(de)ASSIIPK	2881.428	IPI00762812	IPI00762812	yes	yes		3	0.0055751	65.425
MRSPGAQDN(de)VSVSQGMR	1818.8465	IPI00348586	IPI00348586	yes	no		3	8.8992E-14	202.63
MQVVSN(de)GTVTTAIWR	1661.8559	IPI00331175	IPI00331175	yes	no		2	5.7464E-06	161.24
MQDTIEHHVNVSDTSAIPSTIEYGN(de)R	2913.3563	IPI00120953	IPI00120953	yes	yes		3	1.434E-07	122.3
MQDTIEHHVN(de)VSDTSAIPSTIEYGNR	2913.3563	IPI00120953	IPI00120953	yes	yes		3	1.434E-07	122.3
MPSQASAGNVYPQPIIN(de)SSMCIEDSR	2851.2939	IPI00224752	IPI00224752	yes	yes		3	3.3238E-10	138.44
MPFPIDQDFYVSPTFQDIIN(de)R	2542.2202	IPI00318175	IPI00318175	yes	yes		3	0.0004657	110.56
MNPDIDTGHN(de)TSAPAHWGEIK	2290.0437	IPI00314429	IPI00314429	yes	no		3	0.0026099	89.261
MN(de)RSIQIISTPWIGAYIK	2090.1347	IPI00115539	IPI00115539	yes	yes		2	0.01302	89.358
MN(de)MSVITIQEYEFEK	1860.8638	IPI00461438	IPI00461438	yes	yes		2	7.9681E-06	154.51
MIVSNIDIGPTIIDIAGYDIN(de)KTQMDGMSIIPIIK	3801.997	IPI00221426	IPI00221426	yes	yes		4	0.0031694	54.003
MITNEKIDAYNEAAVSIIN(de)SSTR	2539.2588	IPI00380296	IPI00380296	yes	yes		3	0.017437	74.048
MIHN(de)TTGVYTCSAHGTWTNEVIKR	2775.3221	IPI00475209	IPI00475209	yes	no		3	4.0631E-07	180.8
MIEN(de)GSISFIPTIR	1576.8283	IPI00123194	IPI00123194	yes	yes	2,3		1.1314E-15[illegible]	300.51
MIEAYN(de)ITEK	1210.5904	IPI00121430	IPI00121430	yes	no		2	0.011285	120.96
MHING(de)SNVQVIHR	1503.7729	IPI00119063	IPI00119063	yes	yes	2,3		3.9536E-06	168.31
MGDREAIGN(de)ASQIFDSWIKGSASIPVNIR	3131.5822	IPI00134585	IPI00134585	yes	yes		3	0.0048378	68.361
MGDREAIGN(de)ASQIFDSWIK	2137.0262	IPI00134585	IPI00134585	yes	yes	2,3		0.0002228	144.97
MFSQN(de)DTR	997.42873	IPI00129250	IPI00129250	yes	yes		2	0.027424	128.36
MEISVGAIQAN(de)R	1287.6605	IPI00108811	IPI00108811	yes	yes		2	0.0024106	144.57
MEAHNVSAPFN(de)FSIPPGFGHR	2311.0957	IPI00115482	IPI00115482	yes	no	2,3,4		1.0186E-39	233.59
MEAHN(de)VSAPFNFSIPPGFGHR	2311.0957	IPI00115482	IPI00115482	yes	no	2,3,4		1.0186E-39	233.59
MEAEN(de)HTAIVHFIIGISEDPK	2463.2468	IPI00125370	IPI00125370	yes	yes		3	0.028866	56.036
MDYNSFQGTPSN(de)ETK	1717.7254	IPI00876541	IPI00876541	yes	no		2	0.0045752	110.84
MDTSMN(de)FSR	1087.4427	IPI00136210	IPI00136210	yes	yes		2	2.7785E-42	232.41
MDIRP(de)NHTIYINNMNDKIK	2329.1671	IPI00132576	IPI00132576	yes	no	2,3		0.0020735	105.39
MDGHCAPIRTEAGVFEYVADPTFEN(de)FTGGVKK	3542.6599	IPI00405742	IPI00405742	yes	no		4	0.0002239	78.3
MDGHCAPIRTEAGVFEYVADPTFEN(de)FTGGVK	3414.5649	IPI00405742	IPI00405742	yes	no	3,4		1.8522E-06	100.23
MDFIIFN(de)YSAPSYIR	1835.8916	IPI00553503	IPI00553503	yes	yes		2	0.0014052	121.79
MATPIIMRPMSMDNMIIGPVKN(de)VTK	2787.4342	IPI00115892	IPI00115892	yes	no		3	0.0024096	94.259
MAIIQYGSQNQQQVAFPITYN(de)VTTIHEAIER	3562.7879	IPI00621027	IPI00621027	yes	yes		4	3.4617E-07	109.26
MAAAINATGRPIAFSCSWPAYEGGIPPKVN(de)YTEVSR	3880.9029	IPI00315593	IPI00315593	yes	no		4	3.871E-14	103.27
MAAAIN(de)ATGRPIAFSCSWPAYEGGIPPKVNYTEVSR	3880.9029	IPI00315593	IPI00315593	yes	no		4	3.871E-14	103.27
KYQTI(de)NCSVNVR	1480.7456	IPI00420835	IPI00420835	yes	no		2	0.0022909	129.54
KYHDYYITSTS(de)NGSIEGIENR	2446.1401	IPI00130754	IPI00130754	yes	yes		3	9.1285E-29	223.18
KYFDQVDIS(de)NGIDWSIDHK	2279.0859	IPI00133456	IPI00133456	yes	yes		3	1.1089E-39	233.79
KYEQAK(de)NISQDIEK	1692.8683	IPI00400016	IPI00400016	yes	no		2	9.4728E-29	220.27
KWPERISAI(de)DNIINHSSIFIK	2480.354	IPI00123342	IPI00123342	yes	yes		3	2.3853E-05	180.1
KWGH(de)NVTEFQQR	1528.7535	IPI00754386	IPI00754386	yes	yes		2	0.0018282	156.36
KVYEEVIN(de)VTPNDGFAK	1921.9785	IPI00624896	IPI00624896	yes	no		2	0.0025898	133.94
KVPSN(de)STETVIESDQFQPGVR	2317.155	IPI00119299	IPI00119299	yes	no	2,3		0.0001923	122.49
KVIVAPPSEEAN(de)TTK	1582.8566	IPI00470184	IPI00470184	yes	no		2	0.02631	94.007
KVIIN(de)NSIDEPR	1396.7674	IPI00411145	IPI00411145	yes	no		2	0.016799	100.55
KVEVEPIN(de)STAVHVSWK	1922.0262	IPI00110264	IPI00110264	yes	yes	2,3		5.0981E-27	217.35
KVEVEAVN(de)ATAVK	1356.7613	IPI00608063	IPI00608063	yes	no		2	9.0746E-14	188.12
KVEAEAIN(de)ATAIR	1384.7674	IPI00754853	IPI00754853	yes	no		2	0.0064349	114.64
KVDN(de)ASIVADDMR	1432.698	IPI00356462	IPI00356462	yes	no		2	0.0090035	111.64
KTYAVYDIFDTAMIN(de)NSR	2121.0201	IPI00226790	IPI00226790	yes	no	2,3		3.2572E-19	210.39
KTTIEN(de)FTCPEYK	1629.7709	IPI00420810	IPI00420810	yes	yes		2	0.011752	105.03
KTN(de)QSCEIVIDSTEKVNPSYIGR	2637.3068	IPI00310059	IPI00310059	yes	no		3	3.2693E-05	147.67
KTN(de)QSCEIVIDSTEK	1750.8407	IPI00310059	IPI00310059	yes	no		2	3.7254E-21	211.9
KTMFN(de)STEIK	1197.6064	IPI00677395	IPI00677395	yes	no		2	0.017917	124.12
KTISSSN(de)NTITGFIIGFPCPR	2422.2679	IPI00126424	IPI00126424	yes	yes		3	0.024697	66.893
KTGVHDGDFEYN(de)ITTTIAAINK	2407.202	IPI00108849	IPI00108849	yes	no		3	3.9869E-06	170.61
KTGEAN(de)ITQIYTQEAIDFIQTQHAR	2875.4464	IPI00310090	IPI00310090	yes	no	3,4		1.0187E-42	228.31
KTCNPETFPIRN(de)ESIQCPTAR	2518.2057	IPI00649186	IPI00649186	yes	no		3	0.011002	96.391
KSPGYVIDIIVTPQN(de)K	1770.988	IPI00109946	IPI00109946	yes	yes		2	0.0003011	147.17
KSDTQNIIYN(de)VSTGR	1694.8588	IPI01023214	IPI01023214	yes	no		2	0.0053253	113.88
KSCHTAVGTSEGWNVPMGIIYN(de)QTGSCK	3081.4107	IPI00471080	IPI00471080	yes	no		3	5.9904E-10	123.44
KQVTPIFFYFQN(de)R	1686.8882	IPI00319509	IPI00319509	yes	no	2,3		0.0067135	113.99
KPNN(de)ISDTVK	1114.5982	IPI00108849	IPI00108849	yes	yes		2	0.01612	123.51
KPISQEFEAYVN(de)ASGEHGIVVFSIGSMVSEIPEKK	3805.9237	IPI00134691	IPI00134691	yes	no		4	2.0233E-09	93.384
KPIIGHYKPDTIAVVIEN(de)GTSIDR	2635.4334	IPI00410796	IPI00410796	yes	no		3	1.2841E-06	179.19
KNVNISYTV(de)NDSFFPQRPQK	2381.2128	IPI00381178	IPI00381178	yes	yes	2,3,4		0	390.4
KNVN(de)ISYTVNDSFFPQRPQK	2381.2128	IPI00381178	IPI00381178	yes	yes	2,3,4		0	390.4
KN(de)ITDIVEGAKK	1314.7507	IPI00649186	IPI00649186	yes	no		2	0.019378	111.12
KN(de)ISEIWDAYCYR	1716.793	IPI00987265	IPI00987265	yes	no		2	7.0166E-18	196.33
KMVIWN(de)DSTIR	1361.7126	IPI00135975	IPI00135975	yes	yes		2	0.025851	101.89
KMISAFN(de)ATSGK	1253.6438	IPI00466999	IPI00466999	yes	no		2	0.0033897	124.51
KKENGVFEEISN(de)SSGR	1779.8751	IPI00757771	IPI00757771	yes	no	2,3		4.5825E-27	218.66
KITDVETQVIN(de)QTSR	1730.9163	IPI00114350	IPI00114350	yes	yes		2	2.4621E-06	164.93
KITAN(de)STWQPDKAK	1586.8417	IPI00653675	IPI00653675	yes	no		2	2.9596E-06	182.02
KISTN(de)ITIVCKPGDIESAPVIR	2410.3254	IPI00308971	IPI00308971	yes	yes		3	0.0001559	116.73
KINIDGSN(de)YTIIK	1477.814	IPI00119063	IPI00119063	yes	yes		2	1.1793E-31	216.88
KINASIVTSFVEIPIVSN(de)VSIR	2385.3631	IPI00323857	IPI00323857	yes	no	2,3		1.3972E-05	127.17
KIN(de)YTIK	878.52255	IPI00319509	IPI00319509	yes	yes		2	3.9411E-09	183.26
KIN(de)CSQEQVPGSSQCDREPEPR	2600.1707	IPI00222915	IPI00222915	yes	yes		3	0.021078	81.723
KIN(de)ASIVTSFVEIPIVSNVSIR	2385.3631	IPI00323857	IPI00323857	yes	no	2,3		1.3972E-05	127.17
KIIASPNEEN(de)MTEIISMR	2075.0391	IPI00322575	IPI00322575	yes	no	2,3		4.8038E-06	181.51
KIDFIVIN(de)ETR	1346.7558	IPI00125813	IPI00125813	yes	no	2,3		1.1427E-11	190.85
KHYQVTGYGIN(de)GTGDSNDFWR	2414.104	IPI00221669	IPI00221669	yes	no		3	4.5898E-06	181.94
KGN(de)NTINPFIIPSASDVPTGCPNQWWPYAGHCYR	3916.8202	IPI00126186	IPI00126186	yes	yes		4	0.0019093	61.526
KGIVSGGVYNSHVGCIPYTIPPCEHHV(de)NGSRPPCTGEGDTPF	4558.1329	IPI00113517	IPI00113517	yes	yes		4	8.808E-06	64.221
KGFCEADN(de)STVSENNPEDWPVNTEGCMEK	3343.3704	IPI00108098	IPI00108098	yes	yes		3	4.665E-11	123.44
KGCADYCN(de)QTITK	1557.6916	IPI00987265	IPI00987265	yes	no		2	1.3433E-05	163.99

KFPVPFQKEN(de)VTATIVEIGR	2272.258	IPI00134691	IPI00134691	yes	yes	2,3	1.0157E-18	205.78
KFN(de)STQIAAMAPEHEEPR	2054.9844	IPI00856861	IPI00856861	yes	no	3	0.0006009	157.58
KFHVN(de)YTQPIVAVK	1642.9195	IPI00123704	IPI00123704	yes	no	2	9.3621E-07	170.95
KFEAENISN(de)YTAIISQDGK	2240.1325	IPI00464135	IPI00464135	yes	no	2	1.5921E-12	195.03
KEVHFFVN(de)ASDVDSVK	1819.9105	IPI00310049	IPI00310049	yes	yes	2	0.017075	98.507
KEQETCIAPEIEHGN(de)YSTTQR	2490.1445	IPI00122117	IPI00122117	yes	yes	3	5.2746E-05	150.68
KENGVFEEISNS(de)SGR	1651.7802	IPI00757771	IPI00757771	yes	no	2	1.4624E-21	212.95
KENG(de)VFEEISNSSGR	1651.7802	IPI00757771	IPI00757771	yes	no	2	1.4624E-21	212.95
KEN(de)SSYQVINWR	1522.7528	IPI00153316	IPI00153316	yes	yes	2,3	4.9081E-19	200.21
KEN(de)SSEICSNNGECVCGQCVCR	2646.0349	IPI00132474	IPI00132474	yes	no	3	5.5172E-51	238.68
KEDSCQIN(de)YSEGPCIGMQER	2400.0144	IPI00127352	IPI00127352	yes	yes	2,3	6.8259E-09	192.62
KDTCAQECSHFN(de)ITKVESR	2309.0529	IPI00132474	IPI00132474	yes	no	3	7.3131E-49	241.68
KDTCAQECSHFN(de)ITK	1837.8087	IPI00132474	IPI00132474	yes	no	2,3	1.6236E-49	237.48
KDGSQDFN(de)ETWENYEK	1988.8388	IPI00128206	IPI00128206	yes	yes	2	4.7653E-05	171.78
KCEVICN(de)QSNKPITITVEQSR	2503.2523	IPI00626537	IPI00626537	yes	yes	3	0.0009792	114.46
KCEAN(de)ITISSPAR	1445.7297	IPI00894972	IPI00894972	yes	no	2	0.0024419	138.21
KAWGISVINP(de)NKTK	1554.8882	IPI00989096	IPI00989096	yes	yes	2,3	0.0028181	143.93
KAFITN(de)FSMIIDGVTYPGVVKEK	2556.3662	IPI00312711	IPI00312711	yes	no	3	0.0001715	163.68
KAFITN(de)FSMIIDGVTYPGVVK	2299.2286	IPI00312711	IPI00312711	yes	no	2,3	7.9784E-05	152.47
K(de)NITSPVGVQPIINEHTFCAGITK	2623.3792	IPI00409148	IPI00409148	yes	yes	3	0.002136	91.337
K(de)IVIYIEHNIEKNSTK	1928.0731	IPI00321190	IPI00321190	yes	no	2,3	1.9909E-12	195.23
IYWTDGDNISMANMDGSN(de)HTIIFSGQKGPVGIAIDFPESK	4325.0409	IPI00119063	IPI00119063	yes	yes	4	0.0006077	51.975
IYWTDGDNISMANMDGSN(de)HTIIFSGQK	3014.3539	IPI00119063	IPI00119063	yes	yes	3	1.093E-09	146.97
IYWTDGDN(de)ISMANMDGSNHTIIFSGQKGPVGIAIDFPESK	4325.0409	IPI00119063	IPI00119063	yes	yes	4	0.0006077	51.975
IYWTDGDN(de)ISMANMDGSNHTIIFSGQK	3014.3539	IPI00119063	IPI00119063	yes	yes	3	1.093E-09	146.97
IYWISSGNHTIN(de)R	1559.7845	IPI00119063	IPI00119063	yes	yes	2,3	5.0678E-14	188.09
IYWISSGN(de)HTINR	1559.7845	IPI00119063	IPI00119063	yes	yes	2,3	5.0678E-14	188.09
IYVIDGTQN(de)DTAFVFPR	1954.9789	IPI00308658	IPI00308658	yes	no	2	0.000138	168.41
IYSDQCHHNISIIPPPTEIVCN(de)R	2762.3269	IPI00123442	IPI00123442	yes	yes	3	0.0083815	74.698
IYSDQCHHN(de)ISIIPPPTEIVCNR	2762.3269	IPI00123442	IPI00123442	yes	yes	3	0.0083815	74.698
IYPIEN(de)ITIAPDPHVPDSIPPVAYNPWMDIR	3542.7908	IPI01023131	IPI01023131	yes	no	3	1.044E-06	98.967
IYNVTHQFCN(de)ASVMDPTCVR	2411.0821	IPI00132600	IPI00132600	yes	no	2,3	5.0365E-09	191.78
IYN(de)VTHQFCNASVMDPTCVR	2411.0821	IPI00132600	IPI00132600	yes	no	2,3	5.0365E-09	191.78
IYIRN(de)ESEFRDK	1568.7947	IPI00115976	IPI00115976	yes	yes	3	0.048504	97.734
IYIRN(de)ESEFR	1325.6728	IPI00115976	IPI00115976	yes	yes	2	0.022623	112.36
IYIDHNN(de)ITR	1257.6466	IPI00120187	IPI00120187	yes	yes	2	9.5174E-06	175.15
IYAGMVSIMDEAVGNV(de)TK	1896.9325	IPI00652358	IPI00652358	yes	no	2	6.6069E-06	178.88
IWIPVN(de)ITWADIEDKDGR	2140.0953	IPI00126253	IPI00126253	yes	no	2,3	3.1412E-27	219.23
IWDPSSHPGIQN(de)ISR	1705.8536	IPI00338209	IPI00338209	yes	no	2	0.0011159	127.57
IVTQTIPCN(de)KTIFWSK	1935.0288	IPI00307966	IPI00307966	yes	yes	2	0.0019637	136.63
IVTQTIPCN(de)K	1172.6223	IPI00307966	IPI00307966	yes	yes	2	2.3393E-31	218.6
IVPCEFGVIYPIAEN(de)FSR	2110.0557	IPI00121908	IPI00121908	yes	no	3	0.0017468	115.38
IVKIPSGTIVISN(de)ATEGDGGIYR	2359.2747	IPI00129159	IPI00129159	yes	no	3	0.0168	72.644
IVIWTAEEQGGIGASQYYEIHKAN(de)ISK	3004.5294	IPI00126050	IPI00126050	yes	no	3	0.043486	53.779
IVIN(de)HTQMNTAIIQWHPPVDTFGPIQGYR	3345.7081	IPI00608063	IPI00608063	yes	no	4	0.021483	52.019
IVGSPFIGCTVVN(de)K	1489.7963	IPI00134808	IPI00134808	yes	no	2	0.0055131	107.65
IVGGTN(de)ASIGEWPWQVSIQVK	2268.1903	IPI00113057	IPI00113057	yes	yes	2	3.1523E-78	257.72
IVEDIESFIKPYSVEEQKN(de)ITSCPDGAPFIQHGPDYR	4278.0579	IPI00124221	IPI00124221	yes	yes	4	1.6627E-16	119.42
IVDVN(de)ITSEGKVK	1400.7875	IPI00113528	IPI00113528	yes	no	2	0.0027885	126.1
IVDVN(de)ITSEGK	1173.6241	IPI00113528	IPI00113528	yes	no	2	0.004046	125.03
IVAN(de)ISGCAAVNSETIMCCIR	2338.0902	IPI00222489	IPI00222489	yes	yes	2	1.1414E-49	238.8
IV(de)VPINNRENISDPTSPIRR	2289.2553	IPI00136925	IPI00136925	yes	no	3	0.0033943	107.51
IV(de)VPINNRENISDPTSPIR	2133.1542	IPI00136925	IPI00136925	yes	no	2,3	0.0012002	131.18
IV(de)SDVQTAVKTNSSFIQGFVDHVKEDCDR	3293.5987	IPI00321190	IPI00321190	yes	no	3	0.0002704	80.786
ITYESGFIN(de)YSK	1420.6874	IPI00666034	IPI00666034	yes	no	2	1.7229E-66	247.88
ITVVYAEN(de)GTVIQGTTVASVYK	2312.2264	IPI00317356	IPI00317356	yes	yes	2,3	6.3063E-79	223.93
ITVPSSQN(de)SSFR	1321.6626	IPI00515360	IPI00515360	yes	no	2	0.02934	85.522
ITSCATN(de)ASMCGDEAR	1742.7022	IPI00119063	IPI00119063	yes	yes	2	2.2564E-180	314.51
ITNEAHKEN(de)CTGR	1528.7052	IPI00760106	IPI00760106	yes	no	2	0.0099566	107.99
ITN(de)VTFPTGVVTNIHGDMDK	2158.0729	IPI00798466	IPI00798466	yes	no	2,3	0.0003213	130.56
ITN(de)QTIGFSFAVEQDIPVK	2106.0997	IPI00624663	IPI00624663	yes	yes	2,3	3.1056E-205	321.68
ITN(de)ISDATKR	1117.6091	IPI00849192	IPI00849192	yes	yes	2	0.001706	162.26
ITIEVFDPVPKPSIEIN(de)KTEASTDSCHIR	3295.6758	IPI00109946	IPI00109946	yes	yes	3	9.4216E-12	151.06
ITIAIN(de)NTITPHTIPPGTIVYK	2376.3417	IPI00309230	IPI00309230	yes	no	2,3	7.5515E-80	255.98
ITHIPDDIPSNITVIN(de)ITHNQIR	2623.4082	IPI00320618	IPI00320618	yes	yes	3	3.5595E-05	114.55
ITHIPDDIPSN(de)ITVINITHNQIR	2623.4082	IPI00320618	IPI00320618	yes	yes	3	3.5595E-05	114.55
ITFFNSTIN(de)TSGIVAQGEAIPIPGAHRPGIVTK	3405.8409	IPI00153317	IPI00153317	yes	yes	3,4	1.3096E-06	85.002
ITEFTHN(de)STMDYK	1585.7083	IPI00130010	IPI00130010	yes	no	2	0.0017146	127.79
ITDNMFCAGFKVN(de)DTK	1859.8546	IPI00114206	IPI00114206	yes	yes	2,3	0.0048594	116.35
ITANS(de)TWQPDKAK	1458.7467	IPI00653675	IPI00653675	yes	no	2	9.8821E-30	216.88
ITANNSMGASPESIMVISN(de)DSGHEEVKEK	3073.4332	IPI00118291	IPI00118291	yes	yes	3	0.0012468	69.981
ITAN(de)NSMGASPESIMVISNDSGHEEVKEK	3073.4332	IPI00118291	IPI00118291	yes	yes	3	0.0012468	69.981
ISVPDGIKVSN(de)SSAR	1528.8209	IPI00120761	IPI00120761	yes	no	2,3	0.0020812	130.47
ISTSPFAIN(de)ITMIPK	1631.8957	IPI00666034	IPI00666034	yes	no	2	7.3826E-07	169.94
ISTNI(de)TIVCKPGDIESAPVIR	2282.2304	IPI00308971	IPI00308971	yes	yes	2	0.0001901	122.53
ISSSPN(de)VTISCK	1291.6442	IPI00225715	IPI00225715	yes	no	2	4.0114E-09	177.81
ISQN(de)ASIGPHVRPIPIQYEDKEVEPGTICDVAGWGVVTHAGR	4552.2921	IPI00116945	IPI00116945	yes	no	4,5	2.7632E-33	141.13
ISQKN(de)QTVFER	1348.7099	IPI00130117	IPI00130117	yes	no	2	0.017798	105.65
ISQAIGN(de)ITVVQK	1369.7929	IPI00112032	IPI00112032	yes	no	2	0.003407	115.34
ISPIHIAIN(de)FSIDPK	1663.9297	IPI00115976	IPI00115976	yes	yes	2	0.0003214	142.91
ISPGGAEMFQVQDMVVSQEKGN(de)CSIQR	2994.3998	IPI00130117	IPI00130117	yes	no	3	0.0005035	78.352
ISNISN(de)ISHDIVQEATDHAYNIQQEADEISR	3509.6659	IPI00223446	IPI00223446	yes	yes	3,4	1.1283E-12	163.32
ISN(de)VTPEDAGTYYCVK	1815.8349	IPI00129158	IPI00129158	yes	no	2	4.1389E-06	179.17
ISN(de)ISNISHDIVQEATDHAYNIQQEADEISR	3509.6659	IPI00223446	IPI00223446	yes	yes	3,4	1.1283E-12	163.32
ISISEN(de)YTISIANAK	1622.8516	IPI00121378	IPI00121378	yes	no	2	3.6172E-16	203.27
ISHYN(de)DTYPISPPQR	1786.8638	IPI00113039	IPI00113039	yes	no	2,3	2.4087E-50	242.19

ISHYKQN(de)FSFCR	1585.746	IPI00381303	IPI00381303	yes	yes		2	0.0032816	122.46
ISHEN(de)GTIICSK	1357.666	IPI00338094	IPI00338094	yes	yes		2	0.0011381	131.35
ISHDGN(de)ETIPIHIYVK	1834.9577	IPI00405742	IPI00405742	yes	no	2,3		3.3975E-113	283.1
ISGNSIHN(de)ASIPEHIAYGSVITVK	2506.318	IPI00221669	IPI00221669	yes	no		3	0.0001067	110.4
ISGN(de)ITIIR	985.59203	IPI00137311	IPI00137311	yes	yes		2	0.0077663	138.22
ISGKPTN(de)VSVSVIMSEGDGICY	2312.1028	IPI00968978	IPI00968978	yes	no		2	1.5651E-30	223.54
ISEN(de)GSSVAGIISSPNMEK	1918.9306	IPI00399798	IPI00399798	yes	no		2	0.0001336	145.52
ISEGNRTITIINVTRN(de)DTGPYVCETR	2978.488	IPI00108535	IPI00108535	yes	no	3,4		0.0018219	92.01
ISEGNR(de)TITIINVTRNDTGPYVCETR	2978.488	IPI00108535	IPI00108535	yes	no	3,4		0.0018219	92.01
ISEGNR(de)TITIINVTR	1685.9424	IPI00108535	IPI00108535	yes	no		2	0.0099454	107.74
ISEGN(de)RTITIINVTRNDTGPYVCETR	2978.488	IPI00108535	IPI00108535	yes	no	3,4		0.0018219	92.01
ISEGN(de)RTITIINVTR	1685.9424	IPI00108535	IPI00108535	yes	no		2	0.0099454	107.74
ISEEFIKN(de)ISASAR	1563.8257	IPI00894970	IPI00894970	yes	no	2,3		0.0066232	112.42
ISDTN(de)ITAIPQGIPTSITEVHIDGNKITKVDAPSIK	3786.0415	IPI00123196	IPI00123196	yes	yes		4	1.2776E-05	72.687
ISDTN(de)ITAIPQGIPTSITEVHIDGNKITK	3075.6452	IPI00123196	IPI00123196	yes	yes	3,4		3.6746E-11	124.31
ISDTN(de)ITAIPQGIPTSITEVHIDGNK	2733.4185	IPI00123196	IPI00123196	yes	yes	2,3		2.6579E-09	117.35
ISCAFKTEN(de)QTR	1453.6984	IPI00120245	IPI00120245	yes	no		2	0.022082	97.69
ISASGAEIEAIEAQVIN(de)ISIK	2155.1736	IPI00312711	IPI00312711	yes	no	2,3		1.5546E-206	320.92
ISAIDNIIN(de)HSSIFIK	1783.9832	IPI00123342	IPI00123342	yes	yes	2,3		1.3078E-178	305.29
IRPIFN(de)KSFESTVGQGSDTYSYIFR	2911.4505	IPI00108844	IPI00108844	yes	yes	3,4		2.0744E-08	143.47
IRPHFISVCDPDFSQIN(de)CSEGYIQNYR	3314.5237	IPI00115626	IPI00115626	yes	yes		3	1.6679E-09	175.86
IRNPCTSE(de)QNCTSPFSYK	2187.9677	IPI00132474	IPI00132474	yes	yes	2,3		6.8986E-19	205.78
IRN(de)SSIGGVINKYDVVIR	2002.1324	IPI00314673	IPI00314673	yes	yes		3	0.0040303	130.4
IRN(de)SSIGGVINK	1256.7201	IPI00314673	IPI00314673	yes	yes		2	0.019809	98.902
IRDYEEN(de)SSSCHKEVQIIK	2334.1274	IPI00330594	IPI00330594	yes	yes		3	0.0006659	149
IRDQEIEDNVHISPN(de)GSIITIVGTRPSNHGAYR	3657.8612	IPI00515360	IPI00515360	yes	yes		4	3.941E-07	89.723
IRDNAIGN(de)VSCTIHNEAIGQEK	2438.1972	IPI00124515	IPI00124515	yes	no		3	0.0001095	162.32
IRAEQITIHAIGIGEAN(de)KTQIR	2431.3659	IPI00990932	IPI00990932	yes	no		3	0.0001626	116.39
IQKIIQD(de)FFNGKEINK	1934.0625	IPI00323357	IPI00323357	yes	no		3	0.0038976	137.46
IQKENDNFN(de)ISKDDIDITIFHGENK	2946.4359	IPI00674255	IPI00674255	yes	yes		3	7.4696E-06	120.33
IQIEFRPIDIN(de)STAAGIPR	2110.1535	IPI00115510	IPI00115510	yes	yes	2,3		9.6781E-08	183.31
IQDIEITGSPVSNISAHIFSN(de)ISSIEK	2898.4975	IPI00119522	IPI00119522	yes	yes	3,4		3.5013E-11	183.01
IQDIEITGSPVSN(de)ISAHIFSNISSIEK	2898.4975	IPI00119522	IPI00119522	yes	yes	3,4		3.5013E-11	183.01
IQDFIVDNETFSGFIQHN(de)ISIPR	2689.35	IPI00112614	IPI00112614	yes	yes		3	1.5387E-05	164.99
IQDFIVDN(de)ETFSGFIQHNISIPR	2689.35	IPI00112614	IPI00112614	yes	yes		3	1.5387E-05	164.99
IQCVDGN(de)WTTIPVCIEEER	2318.0671	IPI00130010	IPI00130010	yes	no		2	1.0432E-06	183.06
IPVAVIN(de)ATAK	1095.6652	IPI00122491	IPI00122491	yes	yes		2	0.0045602	121.9
IPSSIENATSISIMN(de)ITGTAICHISDIPPDGIR	3492.7593	IPI00121776	IPI00121776	yes	yes	3,4		1.2977E-15	128.91
IPSSIEN(de)ATSISIMNITGTAICHISDIPPDGIR	3492.7593	IPI00121776	IPI00121776	yes	yes	3,4		1.2977E-15	128.91
IPQQIHHYIN(de)ASDWER	2005.9759	IPI00666034	IPI00666034	yes	no		3	0.021619	85.837
IPQGTFSNIEN(de)ITIIDIQHNK	2394.2543	IPI00129867	IPI00129867	yes	no		3	0.0002386	114.99
IPNNTQWITW(de)SPEGHK	1906.9326	IPI00125813	IPI00125813	yes	no	2,3		5.077E-06	178.23
IPN(de)ISIDVIQPSFPEIIIESHMVMIR	2990.5973	IPI00221669	IPI00221669	yes	no		3	3.5628E-05	108.5
IPFSPENTREEDFYVN(de)ETSTVK	2601.2235	IPI00116105	IPI00116105	yes	yes		3	4.1057E-06	175.33
IPFNVIMNNVEDIIEQQTFN(de)DTVSPR	3032.4913	IPI00310049	IPI00310049	yes	yes	3,4		0.0001835	103.92
IPFN(de)ISEWQK	1260.6503	IPI00118011	IPI00118011	yes	no		2	0.018566	106.29
IPEN(de)QTIPGEIPEHAGPAEGVHDSR	2649.2783	IPI00856221	IPI00856221	yes	yes		3	0.0005282	87.319
INSAPVEGYSEHVGN(de)KTTIR	2171.0971	IPI00349830	IPI00349830	yes	no	2,3		4.1682E-05	177
INSAPVEGYSEHVGN(de)K	1699.8166	IPI00349830	IPI00349830	yes	no		2	4.6182E-05	154.88
INNGGCQDICIITHQGHVN(de)CSCR	2712.1737	IPI00119063	IPI00119063	yes	yes		3	0.0019578	93.381
INN(de)ITNIGPIDMK	1441.7599	IPI00330680	IPI00330680	yes	no		2	8.1626E-14	185.95
INMTIPDAIVPTFSISN(de)HSIK	2297.209	IPI00469218	IPI00469218	yes	yes	2,3		1.1613E-156	299.43
INIDGSNY(de)TIIK	1349.7191	IPI00119063	IPI00119063	yes	yes		2	4.292E-19	198.32
INETHIFN(de)GSNWIMIIYK	2192.1088	IPI00108844	IPI00108844	yes	yes	2,3		0.0001307	162.46
IN(de)YTIVGEPIR	1273.703	IPI00120674	IPI00120674	yes	no		2	0.018082	97.463
IN(de)YTCNQGYR	1287.5666	IPI00138061	IPI00138061	yes	no		2	1.3251E-22	211.64
IN(de)YSIPTGQSMEVQIPK	1903.9713	IPI00403938	IPI00403938	yes	no		2	0.0020387	128.74
IN(de)VTSPDIFR	1160.619	IPI00116913	IPI00116913	yes	yes		2	0.0037173	136.48
IN(de)VTEVYDKIK	1320.7289	IPI00225072	IPI00225072	yes	no		2	6.4707E-24	206.1
IN(de)VTEVYDK	1079.5499	IPI00225072	IPI00225072	yes	no		2	0.040974	97.734
IN(de)VSYTGERPSSNMVIVDVK	2207.1256	IPI00624663	IPI00624663	yes	yes	2,3		5.6965E-05	147.09
IN(de)VSYTGER	1037.5142	IPI00624663	IPI00624663	yes	yes		2	0.0002412	172.27
IN(de)VSHAGAPIGEEYIIVFSR	2171.1375	IPI00785452	IPI00785452	yes	no		3	0.0077947	87.24
IN(de)TTSDEKDPTNPFRFPNIGVEK	2618.2976	IPI00131881	IPI00131881	yes	no		3	0.0015143	105.5
IN(de)TTSDEKDPTNPFR	1733.822	IPI00131881	IPI00131881	yes	no		2	3.0184E-07	173.71
IN(de)NSQIK	815.45012	IPI00222967	IPI00222967	yes	no		2	0.027071	137.18
IN(de)MTIPDAIVPTFSISNHSIK	2297.209	IPI00469218	IPI00469218	yes	yes	2,3		1.1613E-156	299.43
IN(de)KTVAIHTIDPEKIGQGGVQK	2345.3067	IPI00323624	IPI00323624	yes	no		3	9.4813E-05	169.67
IN(de)KTVAIHTIDPEK	1577.8777	IPI00323624	IPI00323624	yes	no		2	0.0001052	159.18
IN(de)ITTDPK	900.49165	IPI00122399	IPI00122399	yes	no		2	0.026159	113.71
IN(de)ITSWKET	1090.5659	IPI00113480	IPI00113480	yes	no		2	0.022495	128.81
IN(de)ITAAQISQIEGIIQAR	1938.0898	IPI00115516	IPI00115516	yes	no		3	3.8498E-19	205.78
IN(de)ISEGEVAATVK	1329.714	IPI00458003	IPI00458003	yes	yes		2	2.7386E-32	220.85
IN(de)GTDPIVAADSKR	1455.7682	IPI00119063	IPI00119063	yes	yes		2	7.82E-29	217.48
IN(de)GTDPIVAADSK	1299.667	IPI00119063	IPI00119063	yes	yes		2	6.6369E-66	250.52
IN(de)FTGPGEPDSIR	1401.6888	IPI00320605	IPI00320605	yes	no		2	1.3822E-05	162.97
IN(de)ETHIFNGSNWIMIIYK	2192.1088	IPI00108844	IPI00108844	yes	yes	2,3		0.0001307	162.46
IN(de)ATHFYACGTHAFQPICAAIDAETFIIPTSFEEGKEK	4284.0296	IPI00124666	IPI00124666	yes	no		4	0.0002488	63.776
IMQDPIQQAEGIYCN(de)R	1934.8979	IPI00314549	IPI00314549	yes	yes	2,3		1.514E-05	172.91
IMNIEFYDCSCVSGSGFQKGN(de)HSAR	2863.2476	IPI00230319	IPI00230319	yes	no		3	0.0004305	105.06
IMNAPIYIAEWQNITKN(de)ISEDR	2618.3163	IPI00930882	IPI00930882	yes	no		3	0.0075954	81.157
IMNAPIYIAEWQN(de)ITKNISEDR	2618.3163	IPI00930882	IPI00930882	yes	no		3	0.0075954	81.157
IMNAPIYIAEWQN(de)ITK	1903.9866	IPI00930882	IPI00930882	yes	no	2,3		8.7806E-06	175.66
IMESHPN(de)GTFSAK	1417.666	IPI00177214	IPI00177214	yes	no		2	0.0003652	157.56
IKVSN(de)VSCEASVSK	1506.7712	IPI00135560	IPI00135560	yes	no		2	0.0057274	114.59
IKN(de)TTNQIAIIK	1355.8136	IPI00985989	IPI00985989	yes	no		2	2.7502E-05	166.7

IKN(de)ITDISQK	1158.6608	IPI00119299	IPI00119299	yes	no		2	0.020783	115.29
IKN(de)ATVTQYQIK	1405.7929	IPI00221547	IPI00221547	yes	no		2	0.0075149	113.69
IKIMESH(de)PNGTFSAK	1658.845	IPI00177214	IPI00177214	yes	no		2	0.000503	145.46
IKIDN(de)YSTQEIGR	1535.7944	IPI00122257	IPI00122257	yes	no	2,3		0.0008945	154.36
IKGEAEYQEIRNPN(de)GTVTVISR	2473.2925	IPI00311405	IPI00311405	yes	yes		3	0.0001777	115.63
IKFIEAGIYEVPIIITDSGNPPKSN(de)ISIIR	3296.8384	IPI00323134	IPI00323134	yes	no	3,4		7.5863E-14	140.09
IKEQVVGIIAQNN(de)CSCESK	2176.0616	IPI00122592	IPI00122592	yes	yes		3	5.406E-05	176.49
IIY(de)EGGDIPDFRKENSSYQVINWR	2898.4301	IPI00153316	IPI00153316	yes	yes		3	3.9765E-05	118.96
IITN(de)NSQTPIISPQEVVSCSPYAQGCDGGFPYIIAGK	3980.9288	IPI00130015	IPI00130015	yes	yes		4	7.7495E-18	122.06
IITHTIEGPSQN(de)VTIIQIQPWQDPESWER	3414.7208	IPI00473190	IPI00473190	yes	no		3	1.9109E-15	112.26
IISQKN(de)SSQAR	1230.668	IPI00420867	IPI00420867	yes	no		2	0.0097875	115.29
IISQAPSTPSPNMFTINN(de)ETGDIITVAAGIDREK	3599.8141	IPI00323134	IPI00323134	yes	no	3,4		1.173E-09	104.83
IIQYYGN(de)FSR	1259.6299	IPI00267963	IPI00267963	yes	yes		2	0.0038739	135.99
IIQVVYIHSNN(de)ITK	1640.925	IPI00123194	IPI00123194	yes	yes	2,3		6.7758E-29	213.99
IIQTAEHN(de)ISGAER	1537.7849	IPI00403938	IPI00403938	yes	no		2	0.021041	152.07
IIQQQSN(de)QSSQFIHSVER	2128.0661	IPI00876541	IPI00876541	yes	no	2,3		9.7794E-06	180.31
IIPHIEKPIQN(de)FTICFR	2125.1507	IPI00309214	IPI00309214	yes	yes	2,3,4		0.0012761	140.46
IIPAFN(de)TTSGIPYPR	1645.8828	IPI00127672	IPI00127672	yes	no		2	0.0075789	102.65
IINN(de)ITSIK	1014.6073	IPI00400016	IPI00400016	yes	no		2	0.0044261	152.97
IINKFN(de)SSSSSIEEK	1681.8523	IPI00338561	IPI00338561	yes	yes		2	4.3161E-50	242.19
IINDYVSN(de)QTQGMIK	1722.8611	IPI00135635	IPI00135635	yes	no		2	2.8405E-10	187
IIN(de)TTDVYIIPSINPDGFER	2276.1689	IPI00130573	IPI00130573	yes	yes		2	0.0005762	111.83
IIN(de)QTIRENIKK	1468.8726	IPI00460063	IPI00460063	yes	no		2	0.0052273	128.85
IIN(de)QTIRENIK	1340.7776	IPI00460063	IPI00460063	yes	no		2	1.3695E-07	179.57
IIN(de)QTIR	856.51305	IPI00460063	IPI00460063	yes	no		2	1.3221E-10	187.34
IIN(de)QTADMIQIASK	1544.8232	IPI00126864	IPI00126864	yes	no		2	5.9389E-29	216.27
IIN(de)KSMEIIDER	1459.7705	IPI00112614	IPI00112614	yes	yes		2	0.0083079	114.24
IIN(de)ITFIDITR	1317.7656	IPI00343568	IPI00343568	yes	no		2	1.6591E-11	186.76
IIN(de)HSIIIHK	1186.7186	IPI00406603	IPI00406603	yes	yes	2,3		0.0004778	167.67
IIN(de)ETIYENAK	1306.6769	IPI00153756	IPI00153756	yes	yes		2	1.1236E-07	177.83
IIISPSAFHDGN(de)FSIIIR	1999.0891	IPI00310519	IPI00310519	yes	no		2	0.0010949	126.07
IIISPEEN(de)VTITCTAENQIER	2429.2108	IPI00121378	IPI00121378	yes	no		2	1.1997E-49	237.76
IIIN(de)WTQGQTSGVIR	1684.9261	IPI00674656	IPI00674656	yes	no		2	1.7952E-287	242.91
IIIN(de)FTSMDIYR	1484.7697	IPI00125182	IPI00125182	yes	no		2	4.9286E-09	177.42
IIIGGIPVSGTFHN(de)FSGCISNVFVQR	2818.4589	IPI00116913	IPI00116913	yes	no		3	0.0002019	99.603
IIGVPSIEDIDEVIIHNIPDAIGVIFN(de)DSFSYQIK	3883.0295	IPI00170146	IPI00170146	yes	no		4	0.0016993	59.536
IIGGGDEDAIRPQMQQIIDFETAIAN(de)ITIPQEK	3623.8505	IPI00396840	IPI00396840	yes	no		3	2.6413E-18	151.12
IIFAN(de)VSVR	1017.5971	IPI00173158	IPI00173158	yes	no		2	0.0070308	140.16
IIEYRN(de)TSSEYHIAR	1850.9275	IPI00111856	IPI00111856	yes	no		3	0.023783	95.631
IIEFDSTN(de)ASEGAQPPGKPYPPYSIAK	2876.4232	IPI00459432	IPI00459432	yes	yes		3	8.6079E-18	197.85
IIDIIPDGYPQISCIPKEEN(de)ATIATYPEFGVIDIK	3932.0169	IPI00124221	IPI00124221	yes	no		3	3.4746E-05	75.887
IIDIDSCIDRSN(de)YSVIDITPVAAIIPK	3000.5842	IPI00225072	IPI00225072	yes	no		3	0.0047965	69.98
IIASPNEENM(de)TEIISMR	1946.9441	IPI00322575	IPI00322575	yes	no	2,3		6.5789E-06	176.4
IIASN(de)ITETMR	1247.6544	IPI00132600	IPI00132600	yes	no		2	2.5187E-05	168.02
IIAN(de)ISYTIQIDGHR	1712.921	IPI00894972	IPI00894972	yes	no		2	0.0011695	130.98
IIAENNEIISNIRDSVIN(de)ISESVEDGPR	3095.5735	IPI00114044	IPI00114044	yes	yes		3	4.9195E-10	153.59
IHYIYIQNNFITEIPIESFQN(de)ATGIR	3093.5924	IPI00122293	IPI00122293	yes	yes	3,4		8.4932E-10	163.4
IHVAQPEN(de)DSHVAIIK	1769.9424	IPI00850413	IPI00850413	yes	yes	2,3		7.8705E-05	158.11
IHRIN(de)ASIADIQSK	1564.8685	IPI00116913	IPI00116913	yes	no	2,3		1.0505E-14	194.48
IHN(de)GSEEAIAQYK	1458.7103	IPI00380296	IPI00380296	yes	no		2	0.0034598	115.12
IHKDN(de)TTCYEFKK	1682.8086	IPI00119063	IPI00119063	yes	yes	2,3		2.7748E-05	164.53
IHKDN(de)TTCYEFK	1554.7137	IPI00119063	IPI00119063	yes	yes		2	0.0019741	133.32
IHIIPSMNPDGYEVAAAQGPN(de)MSGYIVGR	3056.4848	IPI00128260	IPI00128260	yes	yes		3	1.0882E-48	229.1
IHIGNYN(de)GTAGDAIR	1570.7852	IPI00310797	IPI00310797	yes	yes		2	0.026939	83.633
IHHWSSPKPICSIVN(de)SSIVVK	2387.2784	IPI00118011	IPI00118011	yes	yes		3	9.0701E-05	146.81
IHEISN(de)ETFR	1244.6149	IPI00321074	IPI00321074	yes	yes		2	0.0035632	136.96
IGYDPYANPPNYGNPDPIVIN(de)NTHR	2810.3412	IPI00316430	IPI00316430	yes	no		3	9.5287E-10	156.87
IGVTN(de)ASIVIFRPGSVR	1785.0261	IPI00652675	IPI00652675	yes	yes		3	0.0042967	116.19
IGVQMHPGQEIHN(de)FTITGR	2134.0742	IPI00119299	IPI00119299	yes	no	2,3,4		0.00054	125.28
IGVIEDHSN(de)R	1138.5731	IPI00129526	IPI00129526	yes	no		2	0.0019861	141.88
IGNFSEIATHN(de)QTFIK	1818.9264	IPI00855103	IPI00855103	yes	no		2	7.7732E-05	157.74
IGN(de)WTGEWPETEIVANIWMK	2373.1464	IPI00322497	IPI00322497	yes	yes	2,3		2.0428E-49	239.55
IGN(de)VTTWISSSNPPVTFAAIYWEEPDVSGHK	3401.6568	IPI00225072	IPI00225072	yes	no		3	2.7131E-14	143.08
IGN(de)FTIAYSAKPETADNQR	2095.0334	IPI00229198	IPI00229198	yes	no		2	0.0010421	128.38
IGN(de)FSEIATHNQTFIK	1818.9264	IPI00855103	IPI00855103	yes	no		2	7.7732E-05	157.74
IGISFNSITVMEN(de)GSIANVPHIR	2468.2846	IPI00123196	IPI00123196	yes	yes	2,3		5.1037E-159	300.97
IGHTN(de)ASIMIFEVK	1558.8177	IPI00858152	IPI00858152	yes	yes		2	0.0005723	147.35
IGGWN(de)ITGPWAK	1298.6772	IPI00396840	IPI00396840	yes	no		2	0.0017659	149.41
IGAINSTISN(de)ESKEAFIDWAR	2321.1652	IPI00187353	IPI00187353	yes	yes		3	0.0040275	98.391
IGAINSTISN(de)ESK	1332.6885	IPI00187353	IPI00187353	yes	yes		2	5.315E-06	166.86
IGAIN(de)STISNESKEAFIDWAR	2321.1652	IPI00187353	IPI00187353	yes	yes		3	0.0040275	98.391
IGAIN(de)STISNESK	1332.6885	IPI00187353	IPI00187353	yes	yes		2	5.315E-06	166.86
IGAIN(de)NSIIIIEDR	1539.8621	IPI00115516	IPI00115516	yes	no		2	3.9849E-21	205.3
IFTPVSSVPN(de)ITWSEISAIEIIK	2543.3887	IPI00652728	IPI00652728	yes	no		3	0.0023905	89.668
IFIFN(de)QTGIEAKK	1507.8399	IPI00378224	IPI00378224	yes	yes		2	0.0024792	141.58
IFIFN(de)QTGIEAK	1379.7449	IPI00378224	IPI00378224	yes	yes		2	3.7975E-82	263.44
IFFVNP(de)APPYIWPAHKNEIMINSSIMR	3184.6354	IPI00378698	IPI00378698	yes	no		4	0.015815	56.29
IETIIIN(de)GTDR	1243.6772	IPI00119063	IPI00119063	yes	no		2	0.0027555	132.5
IEQMTMNIN(de)ITGPIPAPYK	2130.0853	IPI00309999	IPI00309999	yes	no		2	0.0030162	92.633
IEPEHIQIQN(de)ISQEQIAQVATIATK	2801.4923	IPI00165807	IPI00165807	yes	no		3	2.6993E-05	101.75
IEN(de)ITTGTYTIHAQK	1688.8733	IPI00222429	IPI00222429	yes	yes		2	0.0011236	131.86
IEN(de)ISSTESGYTATITR	1841.9007	IPI00111960	IPI00111960	yes	no		2	8.3572E-77	254.82
IEIVPIIYDN(de)DSIFVQTDK	2221.1518	IPI00405543	IPI00405543	yes	yes		2	4.8985E-38	232.54
IEGITN(de)ETYR	1194.5881	IPI00119299	IPI00119299	yes	no		2	0.0044687	134.13
IEGIIAN(de)VSR	1070.6084	IPI00115516	IPI00115516	yes	no		2	0.002338	140.78
IEDRFN(de)STIGPSEEQEKNWPGGPGR	2799.3212	IPI00115516	IPI00115516	yes	no		3	1.1956E-08	131.56

IEDRFN(de)STIGPSEEQEK	1977.928	IPI00115516	IPI00115516	yes	no	2,3	3.848E-08	186.98
IEDGFHPDAVAWAN(de)ITNAIR	2209.0916	IPI00165730	IPI00165730	yes	no	2,3	1.9138E-20	213.03
IEAYFSIEGFPAEQN(de)ASNR	2142.0018	IPI00114319	IPI00114319	yes	yes	2,3	2.5088E-77	254.82
IEAVIPAEFFEVISSSQN(de)GSYHHIR	2829.4086	IPI00342158	IPI00342158	yes	no	4	3.3596E-06	118.52
IDWIGN(de)CSGINDDSYGYR	2103.8956	IPI00121550	IPI00121550	yes	yes	2	7.9108E-29	223.5
IDVSQN(de)VSSDTDQSCESTK	2098.8961	IPI00120769	IPI00120769	yes	no	2	6.5145E-95	272.48
IDVNSWIEN(de)FTK	1464.7249	IPI00626793	IPI00626793	yes	no	2	5.1651E-81	254.89
IDSFN(de)DSTFMIVYTPITPTTQR	2546.2363	IPI00322575	IPI00322575	yes	no	2,3	2.0747E-06	163.45
IDRNPSPDEIPQVGN(de)ISIK	2091.096	IPI00308990	IPI00308990	yes	yes	2,3	0.0002245	146.1
IDPPCTN(de)TTAPSNYINNPYVR	2406.1274	IPI00658539	IPI00658539	yes	no	2,3	1.7967E-13	199.97
IDNYS(de)TQEIGR	1294.6153	IPI00122257	IPI00122257	yes	no	2	0.005201	117.2
IDITDFEKN(de)SSFAQYQSFK	2267.0746	IPI00121312	IPI00121312	yes	yes	3	0.00097	164.01
IDIPVNTSIPNVTEIKEN(de)MTFGSTIVTNPK	3271.701	IPI00466371	IPI01026704	no	no	3	2.5903E-12	154.7
IDIPVNTSIPN(de)VTEIKENMTFGSTIVTNPK	3271.701	IPI00466371	IPI01026704	no	no	3	2.5903E-12	154.7
IDIPVNTSIPN(de)VTEIK	1751.9669	IPI00466371	IPI01026704	no	no	2	2.7513E-05	152.94
IDIPVN(de)TSIPNVTEIKENMTFGSTIVTNPK	3271.701	IPI00466371	IPI01026704	no	no	3	2.5903E-12	154.7
IDIPVN(de)TSIPNVTEIK	1751.9669	IPI00466371	IPI01026704	no	no	2	2.7513E-05	152.94
IDGATVDTHHIPVN(de)VTIR	1957.0381	IPI00751912	IPI00751912	yes	yes	3	0.002808	107.61
IDFIVINE(de)TR	1218.6608	IPI00125813	IPI00125813	yes	no	2	0.0048149	133.05
IDETDCIGN(de)WTWQEGSQQTIK	2508.1227	IPI00122973	IPI00122973	yes	no	2,3	2.2077E-78	259.64
IDCYPDEHGASEAN(de)CSAR	2050.8109	IPI00848693	IPI00848693	yes	yes	2	4.2578E-08	183.77
IDAYNEAAVSIIN(de)SSTR	1822.9061	IPI00380296	IPI00380296	yes	yes	2	0.0001931	146.88
IDAPTNIQFVN(de)ETDRTVIVTWTPPR	2882.4927	IPI00113539	IPI00113539	yes	no	3,4	2.4901E-09	171.35
IDAPTNIQFVN(de)ETDR	1731.8428	IPI00113539	IPI00113539	yes	no	2,3	4.5798E-08	180.7
ICQN(de)ISK	861.43784	IPI00126184	IPI00126184	yes	yes	2	0.018067	147.34
ICN(de)ECSDGSFHISK	1652.6923	IPI00515360	IPI00515360	yes	no	2	0.0001112	155.57
ICHTHGWN(de)ETSEIMPPGAVFSCIYR	2961.336	IPI00122399	IPI00122399	yes	no	3	0.0007094	84.573
ICEGDRFCIIDVMSTGSSSVGN(de)ATR	2731.2364	IPI00749655	IPI00749655	yes	no	3	1.9802E-09	171.5
IAVTN(de)TTMTGTVIK	1448.7909	IPI00108041	IPI00108041	yes	yes	2	1.019E-08	182.02
IAVISGCKN(de)TTSAAMVHCIR	2188.0915	IPI00131216	IPI00131216	yes	yes	2,3	6.0428E-05	161.86
IAVDWESIGYN(de)ITR	1635.8257	IPI00123040	IPI00123040	yes	no	2	5.8164E-15	194.55
IATITCHIIPSDVSQVN(de)VSIIWKPTFIK	3292.8257	IPI00466371	IPI01026704	no	no	3	0.0002273	79.382
IASPPSQGEVPPGPIPEAVIAIYN(de)STR	2759.4494	IPI00114457	IPI00114457	yes	yes	3	0.016582	57.694
IASAVQKN(de)ATSTK	1317.7252	IPI00400016	IPI00400016	yes	no	2	0.028542	92.65
IAQEGGAAAIIIAN(de)NSVIIPSSR	2264.2488	IPI00121776	IPI00121776	yes	yes	3	0.0055647	78.708
IAPASN(de)VSHTVVIRPIK	1801.0574	IPI00467180	IPI00467180	yes	yes	2	6.1446E-27	216.01
IAPASN(de)VSHTVVIR	1462.8256	IPI00467180	IPI00467180	yes	yes	2	0.0015501	121.99
IAN(de)FSQSCTIYSGDDIVEAIPKPCPGCPR	3251.505	IPI00114958	IPI00114958	yes	no	3	5.625E-74	246.39
IAN(de)ETGGHGSGPR	1251.5956	IPI00309230	IPI00309230	yes	no	2	0.0007931	150.26
IAITPAHIIFIADN(de)HTEPAAHFR	2554.3445	IPI00230324	IPI00230324	yes	no	4	0.028261	61.87
IAISFPN(de)ITSDGYPVIYPTGWSSSDNVNCRPR	3582.7202	IPI00466371	IPI01026704	no	no	3,4	5.2367E-18	164.31
IAGGENN(de)CSGRVEIK	1602.7784	IPI00760106	IPI00760106	yes	no	2	0.033501	90.707
IAGGEN(de)NCSGRVEIK	1602.7784	IPI00760106	IPI00760106	yes	no	2	0.033501	90.707
IACIN(de)GTVIR	1115.6121	IPI00134808	IPI00134808	yes	no	2	0.0045447	133.89
I(de)VQAQYWHDPIKESVYRNYSIFIADINQER	3694.8532	IPI00331318	IPI00331318	yes	no	3,4	8.3657E-06	102.21
I(de)VIYIEHNIEKNSTKEEIIAAIEK	2796.5273	IPI00321190	IPI00321190	yes	no	2,3	2.9093E-117	284.07
I(de)VIYIEHNIEKNSTK	1799.9781	IPI00321190	IPI00321190	yes	no	2	2.8042E-07	174.39
HYGPGWVSMANAGKDTN(de)GSQFFITTVK	2912.3916	IPI00135686	IPI00135686	yes	yes	3	0.0001557	104.67
HYAN(de)ITNGMYR	1338.6139	IPI00224073	IPI00224073	yes	no	2,3	0.0030532	124.12
HVVIN(de)GSSFGADGFPYVQVIK	2233.1532	IPI00742377	IPI00742377	yes	no	3	0.0008199	105.89
HVTDMN(de)STIHIIR	1535.7878	IPI00223446	IPI00223446	yes	yes	2,3	0.0010595	148.62
HVGDIGN(de)VTAGKDGVANVSIEDR	2322.1564	IPI00130589	IPI00130589	yes	yes	2,3	3.7589E-06	141.08
HVEDKFN(de)ETTQR	1502.7114	IPI00130654	IPI00130654	yes	no	2,3	0.0033461	122.33
HVCPVENWSYN(de)ESCSPDPAEQGGPK	2843.1915	IPI00128269	IPI00128269	yes	yes	3	1.0678E-07	163.95
HTIEIAPN(de)STAR	1308.6786	IPI00620800	IPI00620800	yes	no	2	0.0079292	101.89
HTDDITSIN(de)NTIVNIR	1824.933	IPI00129304	IPI00129304	yes	yes	2,3	3.538E-77	256.98
HSVIWNAMIHPIQN(de)MTIK	2132.1023	IPI00111794	IPI00111794	yes	no	2,3	0.0001577	159.22
HSQQPIITYEKYN(de)VTDTPK	2261.1328	IPI00322418	IPI00322418	yes	yes	2,3	1.3844E-19	211.89
HSIEHFNN(de)NTDHSHIFTIR	2318.0941	IPI00114958	IPI00114958	yes	no	2,3,4	5.4827E-113	278.4
HPGNFSSISCDYFAVN(de)QSR	2184.9647	IPI00115976	IPI00115976	yes	yes	2,3	0.0005761	160.47
HPGN(de)FSSISCDYFAVNQSR	2184.9647	IPI00115976	IPI00115976	yes	yes	2,3	0.0005761	160.47
HNN(de)DTQHIWESDSNEFSVIADPR	2710.2008	IPI00129526	IPI00129526	yes	no	2,3,4	1.2347E-64	243.99
HN(de)QSCEWCQTIAVR	1787.7832	IPI00330843	IPI00330843	yes	yes	2	0.0013099	133.25
HN(de)ITITQGK	1010.5509	IPI00128358	IPI00128358	yes	no	2	0.0082428	136.96
HMN(de)SSEQAHTATMIIDTIEEGAFVIADNIIEPTR	3753.7978	IPI00876558	IPI00876558	yes	no	4	0.0016551	57.347
HIVFWN(de)SSNPK	1327.6673	IPI00130752	IPI00130752	yes	no	2	0.011303	103.34
HISN(de)DTTTPESTMTVGQAR	2044.9484	IPI00876306	IPI00876306	yes	yes	3	0.0010949	114.29
HISFGEDYPGIVNPIDHTN(de)VTAPQASMMFQYFVK	3852.828	IPI00132454	IPI00132454	yes	no	3,4	3.8184E-20	143.19
HIN(de)ITTISVAGGFR	1484.81	IPI00850413	IPI00850413	yes	yes	2,3	4.9947E-29	216.54
HIN(de)ASNPSEPATIIFTAAR	2009.033	IPI00421157	IPI00421157	yes	yes	2,3	2.8416E-95	273.46
HIIEN(de)ATASVSEAERK	1753.8959	IPI00396840	IPI00396840	yes	no	2,3	8.2947E-20	211.7
HIIEN(de)ATASVSEAER	1625.8009	IPI00396840	IPI00396840	yes	no	2,3	4.2016E-39	232.78
HHAAYVNNIN(de)ATEEKYHEAIAK	2522.2302	IPI00109109	IPI00109109	yes	yes	3	1.7522E-13	193.8
HGQIIKN(de)FTIIQGPPGPR	1972.1007	IPI00129304	IPI00129304	yes	yes	3	0.0004057	142.54
HGNIYDIKPIGIN(de)DTIVSVGEYTYYIR	3112.587	IPI00308971	IPI00308971	yes	yes	3	3.4413E-06	103.03
HGHFTGFN(de)GSTIR	1429.6851	IPI00117986	IPI00117986	yes	yes	2,3	0.010956	98.337
HGFYCYIIGSTISTFTDAN(de)HTCTNEK	3036.3382	IPI00126186	IPI00126186	yes	yes	3	4.1945E-56	243.23
HFKGQTQN(de)YSTIIIEEASER	2350.1553	IPI00124666	IPI00124666	yes	no	3	3.0078E-19	208.49
HFFN(de)VSDEIAIVTIVK	1830.988	IPI00466371	IPI01026704	no	no	2	0.021426	86.005
HEEGHMINC(de)TCFGQGR	1931.7825	IPI00113539	IPI00113539	yes	no	2,3	0.001633	128.76
HDYIIIPEGAITN(de)TTR	1812.937	IPI00121421	IPI00121421	yes	no	2	0.0080354	100.43
HAN(de)WTITPIK	1179.64	IPI00317356	IPI00317356	yes	yes	2	0.0072407	127.42
HAEISFVFITDGVTGN(de)DSIEESVHSMR	2976.3924	IPI00621027	IPI00621027	yes	yes	3	8.2429E-24	174.19
GYN(de)SSQDIPSIVIDFVK	1880.952	IPI00124666	IPI00124666	yes	yes	2	7.6783E-134	294.05
GYITSFEMFN(de)STFK	1670.765	IPI00262024	IPI00262024	yes	no	2	2.7394E-78	259.1
GYIITINFTKN(de)TTR	1640.8886	IPI00469218	IPI00469218	yes	yes	2	5.1472E-114	283.82

GYIITIN(de)FTKNTTR	1640.8886	IPI00469218	IPI00469218	yes	yes		2	5.1472E-114	283.82
GYIITIN(de)FTK	1168.6492	IPI00469218	IPI00469218	yes	yes		2	2.3906E-31	218.49
GWIN(de)GSIVGFYK	1339.6925	IPI00134585	IPI00134585	yes	no		2	5.3258E-19	197.07
GVTSVSQIFHSPDIAIRDTYVN(de)ASQSIYGSSPR	3551.7645	IPI00122977	IPI00122977	yes	no	3,4		1.2937E-09	106.28
GVTNDIISVQGNTGPSWIN(de)KTEK	2457.25	IPI00344686	IPI00344686	yes	yes	2,3		8.2442E-07	142.23
GVTHIN(de)ISGIKMPR	1521.845	IPI00119063	IPI00119063	yes	yes		2	0.044569	119.62
GVTHIN(de)ISGIK	1137.6506	IPI00119063	IPI00119063	yes	yes	2,3		0.000838	153.74
GVSSSEN(de)FTSPVMEFWERR	2244.027	IPI00466652	IPI00466652	yes	no		3	0.0022583	112.17
GVSSSEN(de)FTSPVMEFWER	2087.9259	IPI00466652	IPI00466652	yes	no	2,3		9.7201E-09	191.47
GVQIIVFPEDGIHGFN(de)FTR	2145.1007	IPI00321375	IPI00321375	yes	no	2,3		7.9059E-38	231.77
GVN(de)VTMPSQPGVPPISSTQIQIDPAIQEFQIVDISRR	4017.0994	IPI00515360	IPI00515360	yes	no		4	0.017353	49.069
GVN(de)VTMPSQPGVPPISSTQIQIDPAIQEFQIVDISR	3860.9982	IPI00515360	IPI00515360	yes	no		3	4.5245E-26	139.85
GVIPGEDWTVFQSN(de)HSTYEPVIIAK	2786.3915	IPI00404189	IPI00404189	yes	no	2,3		0.0003726	92.01
GVIMVGN(de)ETTYEDGHGARKNITDIVEGAK	3073.5139	IPI00649186	IPI00649186	yes	no		3	1.0093E-07	118.37
GVIMVGN(de)ETTYEDGHGAR	1904.8687	IPI00649186	IPI00649186	yes	no	2,3		0.0009472	137.73
GVHFSPAASGITTSTVQVQGIEPYAN(de)YTFTVK	3369.6881	IPI00120222	IPI00120222	yes	yes		3	0.0016935	61.538
GVFITNE(de)TGQPIIGK	1572.8512	IPI00111960	IPI00111960	yes	no		2	2.8561E-11	192.65
GVENDVFIRYPNN(de)GSIVWGK	2263.1386	IPI00848693	IPI00848693	yes	yes	2,3		0.0004051	135.49
GVDSCHGDSGGAFAFQVPN(de)VTVPK	2445.1383	IPI00653675	IPI00653675	yes	no		3	0.0027611	79.942
GVAVTN(de)TSQIGFR	1348.7099	IPI00471089	IPI00471089	yes	no		2	2.0001E-44	204.85
GV(de)IMVGNETTYEDGHGARKNITDIVEGAK	3073.5139	IPI00649186	IPI00649186	yes	no		3	1.0093E-07	118.37
GTFTDCAIANM(de)TQQIR	1825.8451	IPI00621027	IPI00621027	yes	no		2	5.2499E-94	268.61
GTDPSISPSPAATPPAPGAEISYIN(de)HTFR	2950.4461	IPI00762091	IPI00762091	yes	yes		3	0.0001354	79.177
GTCEQGPSIVTPPKDIWN(de)VTGAK	2454.2213	IPI00652107	IPI00652107	yes	no		3	6.245E-05	114.4
GTAGNAIMDGASQIVGEN(de)R	1859.8796	IPI00279079	IPI00279079	yes	yes	2,3		1.2498E-94	269.61
GSTQYIENIGFN(de)MSK	1687.7876	IPI00116105	IPI00116105	yes	yes		2	1.6845E-05	156
GSPGN(de)ASQGSIHIHSPQIAIQADPR	2537.2735	IPI00114044	IPI00114044	yes	yes	2,3		0.0001846	106
GSNFN(de)ISKTDNIEECQK	1982.9004	IPI00113057	IPI00113057	yes	yes	2,3		5.6394E-08	184
GSNFN(de)ISK	865.42938	IPI00113057	IPI00113057	yes	yes		2	0.017251	140.45
GSN(de)FTAICVIK	1208.6223	IPI00120155	IPI00120155	yes	yes		2	0.001262	142.97
GSIQSANDISSQTQGFIQHSMDN(de)ISAQIQTVR	3460.6641	IPI00330594	IPI00330594	yes	yes	3,4		8.753E-36	205.12
GSIN(de)VSYNIGPGFTGSEYSR	2103.9861	IPI00113042	IPI00113042	yes	no		2	0.0004992	113.51
GSEVEDEDIEIFN(de)TSVQIRPPSTAPGPETAAFIER	3800.8381	IPI00381357	IPI00381357	yes	no	3,4		5.1388E-30	202.58
GSAIPHPSN(de)ATWEIK	1606.8104	IPI00119299	IPI00119299	yes	no		2	0.01801	90.725
GQTQNYST(de)IIIEEASER	1937.933	IPI00124666	IPI00124666	yes	no	2,3		8.9898E-49	239.75
GQTGIGFIYASGIGVN(de)SSQAK	2054.0433	IPI00131143	IPI00131143	yes	no	2,3		1.3552E-49	236.96
GQAQSDAAGIPGAESRN(de)DSIPGAGSEADGIEGK	3111.4341	IPI00317487	IPI00317487	yes	yes		3	1.0861E-09	107.31
GPNKIQC(de)VDGNWTTIPVCIEEER	2714.2792	IPI00130010	IPI00130010	yes	no		3	0.0019986	98.543
GPIANPIWN(de)VTGFTGR	1698.8842	IPI00123040	IPI00123040	yes	no		2	0.0027011	114.63
GPDISN(de)CTSHWVNQIAQK	2053.964	IPI00876558	IPI00876558	yes	no		2	0.0012405	119.8
GPDGTEGISSTVN(de)GSTDPSAVTDIR	2432.1303	IPI00469426	IPI00469426	yes	no		2	7.6588E-08	166.65
GPCSHICIINYN(de)R	1602.7395	IPI00119063	IPI00119063	yes	yes		2	3.7325E-33	223.31
GNYGWQAGN(de)HSAFWGMTIDEGIR	2566.1448	IPI00115458	IPI00115458	yes	no		3	1.4865E-06	160.67
GNYDFVEAMIVNN(de)HTSIDVER	2422.1223	IPI00403586	IPI00403586	yes	yes	2,3		0.000188	155.79
GN(de)TTAIDKEIAR	1287.6783	IPI00108535	IPI00108535	no	no	2,3		5.3103E-14	188.48
GN(de)SSIYPVVSPSIQQIVIEK	2157.1681	IPI00985989	IPI00985989	yes	no		2	0.0015408	101.53
GN(de)QSSQWIYEAMAK	1611.7351	IPI00137792	IPI00137792	yes	yes		2	0.0019953	121.13
GN(de)ITEYQCHQYITK	1753.8094	IPI00122399	IPI00122399	yes	no		2	3.5213E-08	178.23
GN(de)ISTEKFVEEIK	1492.7773	IPI00466371	IPI01026704	no	no		2	0.010161	107.65
GN(de)HSIFGIEVATIGQGPDCPSVNER	2653.2555	IPI00459432	IPI00459432	yes	yes		3	6.0263E-18	201.13
GN(de)GSCVCHAGWQGIR	1657.7202	IPI00987265	IPI00987265	yes	no		2	0.0083406	101.62
GMVIDENSNN(de)ITGAVISVTGINHDVTSGEHGDYFR	3717.7329	IPI00128260	IPI00128260	yes	yes		3	8.0817E-19	141.23
GMNYN(de)SSVVK	1097.5175	IPI00416285	IPI00416285	yes	yes		2	0.022949	104.79
GKNN(de)QTECFNHVR	1602.7321	IPI00124666	IPI00124666	yes	no		2	0.0024436	139.48
GKIYWTDGDNISMANMDG(de)SNHTIIFSGQK	3199.4703	IPI00119063	IPI00119063	yes	yes		3	1.0612E-12	146.5
GKIYWTDG(de)DNISMANMDGSNHTIIFSGQK	3199.4703	IPI00119063	IPI00119063	yes	yes		3	1.0612E-12	146.5
GIWKIPFSPENTREED(de)FYVNETSTVK	3085.5033	IPI00116105	IPI00116105	yes	yes		3	0.0045894	77.287
GIVSGGVYNSHVGCIPYTIPPCEHHVN(de)GSRPPCTGEGDTPR	4430.0379	IPI00113517	IPI00113517	yes	yes		4	0.0002026	54.596
GIVIGPI(de)HKNQKDEINETDIK	2360.27	IPI00930882	IPI00930882	yes	no		3	0.014684	93.371
GISYQIIGN(de)HSK	1315.6884	IPI00623114	IPI00623114	yes	no		2	0.0013357	128.68
GISPGN(de)YSVR	1048.5302	IPI00128358	IPI00128358	yes	no		2	0.012091	113.69
GIRGPN(de)FTSPASITFTTGIKPPQDIEAK	2942.5502	IPI00458413	IPI00458413	yes	no		3	0.011806	67.33
GINIT(de)EDTYKPR	1405.7201	IPI00119809	IPI00119809	yes	yes		2	1.086E-32	219.43
GIMVGN(de)GSVIGVVQAVDAETGK	2100.0885	IPI00342158	IPI00342158	yes	no	2,3		5.273E-297	353.39
GIMIIIN(de)DTQHFSNNVKGEIGQFYR	2893.4545	IPI00312711	IPI00312711	yes	no		3	0.000488	104.03
GIMIIIN(de)DTQHFSNNVK	1942.9935	IPI00312711	IPI00312711	yes	no	2,3		6.3743E-79	264.03
GIIN(de)ATISVAEINHPVTTYK	2140.1528	IPI00130573	IPI00130573	yes	yes	2,3		0.0005	113.49
GIIGISN(de)ATIYWHIPDTAYPGIYR	2690.3857	IPI00458077	IPI00458077	yes	yes		3	6.6605E-10	183.74
GIFPDGSHEISGN(de)TSITPDK	2070.9858	IPI00894972	IPI00894972	yes	no		2	0.0004922	113.73
GIAN(de)ISNFIR	1103.6087	IPI00129158	IPI00129158	yes	no		2	0.0003283	169.93
GIAEVTEN(de)VTEGGVTK	1602.8101	IPI00130271	IPI00130271	yes	no		2	4.7846E-49	239.58
GHVDPAN(de)DTFDIDPR	1667.754	IPI00453501	IPI00453501	yes	no	2,3		0.0026862	115.12
GHTDRCDEAQAIQVWN(de)DTHPEVISQKPFDK	3520.643	IPI00108003	IPI00108003	yes	no		4	0.0095992	64.845
GHIIIAQVATNPQGITGTGN(de)TTSEMDPSHRK	3230.6102	IPI00321375	IPI00321375	yes	no	3,4		1.1661E-13	143.47
GHIIIAQVATNPQGITGTGN(de)TTSEMDPSHR	3102.5153	IPI00321375	IPI00321375	yes	no	3,4		3.9209E-13	165.47
GHICN(de)QTQNIQSSK	1613.758	IPI00987886	IPI00987886	yes	yes		2	0.0010779	137.46
GHFYYN(de)ISDVR	1369.6415	IPI00135560	IPI00135560	yes	no		2	0.0073012	110.08
GHAIGISCQSEN(de)GTAPITYHIMK	2484.189	IPI00406901	IPI00406901	yes	no		3	2.4991E-06	135.81
GHAHIAAIVNHDSYN(de)FSHR	2145.0253	IPI00133365	IPI00133365	yes	no		3	4.0181E-21	213.51
GGIN(de)ITAVTVTAENDHTVAFIGTSDGR	2715.3464	IPI00405742	IPI00405742	yes	no		3	0.0001083	98.121
GFQWVTGDN(de)HTSYSR	1753.7808	IPI00129423	IPI00129423	yes	yes		2	1.2076E-06	166.04
GFN(de)ATYHVR	1063.5199	IPI00411025	IPI00411025	yes	yes		2	0.01597	123.86
GFGVAFVGN(de)YTGSIPNEAAINTVR	2453.2339	IPI00165807	IPI00165807	yes	no		3	2.0188E-06	127.49
GENPSQYGITAFNHPIN(de)ITK	2200.0913	IPI00112614	IPI00112614	yes	yes	2,3		0.0002383	125.79
GEIQSEN(de)SSITISSSNR	1807.8548	IPI00466371	IPI01026704	no	no	2,3		1.3699E-132	287.57
GEIN(de)STIFSSRPK	1434.7467	IPI00460063	IPI00460063	yes	no	2,3		5.9797E-18	197.36

GECYYTN(de)GTQR	1347.5514	IPI00110910	IPI00110910	yes	no		2	0.032818	88.338
GDTVARPIIHEFYDDN(de)NTWGIDR	2703.2677	IPI00848693	IPI00848693	yes	yes		3	0.0009967	98.443
GDTHTQIIEGIQFN(de)ITQTSEADIHK	2795.3726	IPI00123924	IPI00406302	no	no	3,4		2.8473E-120	281.29
GDPSIISVN(de)GTDFTFR	1724.837	IPI00463492	IPI00463492	yes	yes		2	7.9916E-09	188.44
GDN(de)QSPIEIHTK	1337.6575	IPI00221890	IPI00221890	yes	yes		2	4.1772E-14	190.85
GDHHQISHYN(de)ITGVR	1732.8394	IPI00338209	IPI00338209	yes	no	2,3		1.2864E-06	165.49
GDGPFTVFVPHADIISN(de)MSQDEIAR	2715.2963	IPI00987265	IPI00987265	yes	no		3	1.4932E-05	112.36
GDEKENITA(de)EAIDISIK	1844.9367	IPI00124725	IPI00124725	yes	no	2,3		6.0486E-05	172.66
GDDIYTNV(de)TVSIVEAIVGFEMDITHIDGHK	3287.602	IPI00320241	IPI00320241	yes	yes		4	5.7831E-12	129.43
GCKDNA(de)TDSVPIR	1431.6776	IPI00119063	IPI00119063	yes	yes	2,3		0.0027502	126.71
GCADYCNQ(de)TITKR	1585.6977	IPI00987265	IPI00987265	yes	no		2	1.6376E-13	183.89
GAIQIPTVSFSHEESN(de)TTAIAEFGEYIRK	3194.5884	IPI00224073	IPI00224073	yes	yes	3,4		6.1023E-06	102.21
GAIQIPTVSFSHEESN(de)TTAIAEFGEYIR	3066.4934	IPI00224073	IPI00224073	yes	yes		3	1.8588E-11	140.2
GAIDIMIQVN(de)MTPGHSSAPPK	2163.0816	IPI00224073	IPI00224073	yes	yes	2,3		3.517E-05	171.13
GAGEVSPAEHSSKPTN(de)ISAK	1965.9756	IPI00230718	IPI00230718	yes	no	2,3		0.0019479	104.03
GAFVSKNPCN(de)ITREDYAPIVK	2378.2053	IPI00307966	IPI00307966	yes	yes	2,3		0.0002096	147.09
GAFVSKNPCN(de)ITR	1462.7351	IPI00307966	IPI00307966	yes	yes		2	0.016163	100.55
GAFISN(de)FTMTVNGMTFTSSIK	2253.081	IPI00970438	IPI00970438	yes	no		2	6.3921E-05	153.36
GAFFPIKEDN(de)WSIPNR	1889.9424	IPI00135560	IPI00135560	yes	no		2	0.0063655	111.22
GAAAPSAPHWN(de)ETAEK	1635.7641	IPI00648288	IPI00648288	yes	no		2	0.0014947	130.27
FVPNSNMN(de)FTGQAYSGR	1888.8526	IPI00108535	IPI00108535	no	no	2,3		4.5244E-05	166.57
FVN(de)STGYITEAEK	1457.7038	IPI00153187	IPI00153187	yes	yes		2	6.363E-06	165.34
FVHVN(de)TSAIIK	1227.6976	IPI00153632	IPI00153632	yes	yes		2	0.020574	95.477
FTQDAYSAVVKEN(de)STEAR	2014.9596	IPI00623114	IPI00623114	yes	no	2,3		0.0025848	132.79
FTIKPIGFIIIQISGDIEDIEPAIN(de)K	2883.5997	IPI00165730	IPI00165730	yes	no		3	2.2674E-18	167.61
FTFTSHTPGEHQICIHSN(de)STK	2428.123	IPI00473680	IPI00473680	yes	no	2,3		6.7924E-14	202.16
FTFTSHTPGDHQICIHSN(de)STR	2442.1135	IPI00153468	IPI00153468	yes	no	2,3		7.1542E-28	216.3
FTECCHEERPIN(de)TSAIK	2090.9514	IPI00876233	IPI00876233	yes	no	2,3		0.0033617	130.01
FTCN(de)QTTDVIIIHSK	1775.8876	IPI00319509	IPI00319509	yes	yes	2,3		4.7202E-133	287.85
FTAPDTIFAN(de)GSVYPPNEGFCPCR	2716.205	IPI01008227	IPI01008227	yes	no	2,3		7.3806E-07	168.68
FSWNNITNSIDIANISADFQGRPVDDPTGAFAN(de)GSITFK	4200.0189	IPI00459432	IPI00459432	yes	yes		4	3.2872E-17	119.54
FSWNN(de)ITNSIDIANISADFQGRPVDDPTGAFANGSITFK	4200.0189	IPI00459432	IPI00459432	yes	yes		4	3.2872E-17	119.54
FSVN(de)QTTIITHEK	1516.7886	IPI00848693	IPI00848693	yes	yes		2	3.9486E-10	181.67
FSTEIGYN(de)GTR	1243.5833	IPI00134013	IPI00134013	yes	yes		2	6.2661E-08	180.24
FSPPVVN(de)VTWIR	1413.7769	IPI00110808	IPI00110808	yes	no		2	0.017381	92.856
FSMSDTYDIQDVIADVGIKDIFTN(de)QSDFADTTKDTPITITVIHK	4918.411	IPI00116105	IPI00116105	yes	yes	4,5		2.9972E-22	111.67
FSMSDTYDIQDVIADVGIKDIFTN(de)QSDFADTTK	3699.7138	IPI00116105	IPI00116105	yes	no		3	7.8521E-45	220.98
FSHN(de)GTCAAEGK	1277.5459	IPI00320675	IPI00320675	yes	yes		2	0.0032488	117.2
FSEIIVNN(de)ATEEIIVK	1817.9775	IPI00321190	IPI00321190	yes	no		2	0.0014197	128.64
FSEIATVHN(de)HSIPK	1578.8154	IPI00124697	IPI00124697	yes	no		2	1.9259E-06	163.99
FSDGIESN(de)RSTHFEVK	1851.8751	IPI00131091	IPI00131091	yes	no	2,3		0.0026671	120.87
FSDGIESN(de)R	1023.4621	IPI00131091	IPI00131091	yes	no		2	0.0056377	145.25
FSATEVPEKGAGEVSP(de)AEHSSKPTNISAK	2954.4621	IPI00230718	IPI00230718	yes	no	3,4		1.1893E-11	153.52
FQSAAIGAIQEASEAYIVGIFEDTN(de)ICAIHAK	3436.6973	IPI00989576	IPI00989576	yes	no		3	0.030568	50.593
FQN(de)FSMATDQR	1343.5928	IPI00128178	IPI00128178	yes	no		2	1.6115E-05	169.89
FQISPQIQFSPEEVIGMVIN(de)YSR	2681.3523	IPI00123342	IPI00123342	yes	no	2,3		2.6155E-65	252.1
FQIIN(de)FSSSEIK	1411.7347	IPI00856723	IPI00856723	yes	no		2	2.4302E-08	182.89
FPVPFQKENV(de)TATIVEIGR	2144.163	IPI00134691	IPI00134691	yes	yes	2,3		5.9878E-19	206.21
FN(de)VSIIYGR	1067.5764	IPI00320675	IPI00320675	yes	yes		2	0.0069093	140.49
FN(de)STEYQVVTR	1342.6517	IPI00119063	IPI00119063	yes	no		2	3.2107E-33	214
FN(de)ITETPEADIHQGFGNIIQSISQPEDQDQINIGNAMFIEK	4601.202	IPI00131830	IPI00131830	yes	no		4	2.8791E-12	84.787
FN(de)ISIQK	848.4756	IPI00798466	IPI00798466	yes	no		2	0.030988	114.5
FN(de)GSVSFFR	1059.5138	IPI00551169	IPI00551169	yes	no		2	0.0023326	147.74
FN(de)ETRDTITSIVSDINVR	2079.0596	IPI00990932	IPI00990932	yes	yes		3	0.0020767	127.29
FN(de)ETIHR	915.45626	IPI00471081	IPI00471081	yes	yes		2	0.030364	136.8
FN(de)DTEVIQR	1120.5513	IPI00321375	IPI00321375	yes	no		2	0.02577	110.39
FMGIPTKDDNIEHYKN(de)STVMAR	2566.2308	IPI00123925	IPI00123925	yes	no		3	0.0094112	81.854
FKDNTPNAN(de)ATER	1476.6957	IPI00125138	IPI00125138	yes	no		2	0.019233	98.592
FIWSEPQN(de)CSATK	1566.7137	IPI00118987	IPI00118987	yes	no		2	0.0057294	107.46
FISYN(de)VTR	998.51853	IPI00453707	IPI00453707	yes	yes		2	0.026205	113.18
FIQSAEFFN(de)YTVR	1620.7936	IPI00127406	IPI00127406	yes	yes	2,3		9.4335E-20	203.32
FINYN(de)QTVSR	1240.62	IPI00226932	IPI00226932	yes	no		2	2.4589E-22	209.97
FINFFVGNTIN(de)SSYPPGYSIHSISVR	2915.4606	IPI00130420	IPI00130420	yes	no		3	0.0002375	92.932
FIN(de)ISSTGIR	1106.6084	IPI00131898	IPI00131898	yes	yes		2	0.0093069	122.7
FIIKGPN(de)KIQCVDGNWTTIPVCIEEER	3215.6107	IPI00130010	IPI00130010	yes	no		3	5.8774E-05	89.201
FIHVSHIN(de)ASMK	1382.7129	IPI00120212	IPI00120212	yes	yes		2	0.0090991	122.39
FIGITPFIN(de)YTR	1440.7765	IPI00323726	IPI00323726	yes	yes		2	0.016961	93.258
FIEYVTSECHFYN(de)GTQHVR	2386.0801	IPI00474580	IPI00474580	yes	no		3	0.0003568	166.35
FIEAGIYEVPIIITDSGNPPKSNIS(de)IIR	3055.6594	IPI00323134	IPI00323134	yes	no	3,4		3.4331E-11	139.51
FHVNY(de)TQPIVAVK	1514.8245	IPI00123704	IPI00123704	yes	no		2	0.0050832	109.44
FHVHQPVTQPFIQVTN(de)TTVK	2320.2328	IPI00658311	IPI00658311	yes	no	2,3,4		3.6101E-40	233.79
FHSDINI(de)SESIIPAVIEK	2011.0626	IPI00831484	IPI00831484	yes	yes	2,3		1.4907E-05	178.75
FGYIIHTDN(de)R	1234.6095	IPI00475209	IPI00475209	yes	no		2	0.0016929	148.32
FGTCSQICN(de)NTK	1428.6126	IPI00119063	IPI00119063	yes	yes		2	2.4701E-14	189.3
FGN(de)ETFIIHIDNGR	1631.8056	IPI00170355	IPI00170355	yes	yes		2	0.0022241	119.31
FGHSPIIN(de)ITGGISHASDVVIYHQHKQPEVTNPCDR	4022.981	IPI00119063	IPI00119063	yes	yes		4	0.031955	45.748
FGHSPIIN(de)ITGGISHASDVVIYHQHK	2826.4565	IPI00119063	IPI00119063	yes	yes	3,4,5		4.8866E-08	153.36
FGEFGN(de)YSIIVQHASSGANK	2125.0229	IPI00317488	IPI00317488	yes	no	2,3		6.8982E-114	280.81
FFQAFTKN(de)GSGATIPGAGANVQTIR	2552.3136	IPI00223231	IPI00223231	yes	no		3	0.0012466	82.267
FFN(de)FSWR	1002.4712	IPI00396840	IPI00396840	yes	no		2	0.020216	144.38
FFN(de)DSIVDPVDSEWFGFYR	2339.0535	IPI00331318	IPI00331318	yes	no	2,3		1.8674E-77	257.2
FDGEPCDQSIN(de)ITWFIK	2068.9564	IPI00625262	IPI00625262	yes	no		2	0.0037056	109.36
FDEFFSQGCAPGYEKN(de)STICDICIGPIK	3252.4566	IPI00987951	IPI00987951	yes	no	3,4		1.9908E-14	190.85
FDAAGTSVHGGVPQN(de)GSICAHIPMIK	2663.2948	IPI00315535	IPI00315535	yes	yes		3	3.7265E-08	161.02
FCNIVPTEHCFIGN(de)GTEYR	2313.0307	IPI00227857	IPI00227857	yes	yes		3	0.0006659	119.08
FAVESIVPSSISIMHSPPDAQN(de)MSEVSISPMEISTFR	4019.9319	IPI00114044	IPI00114044	yes	yes	3,4		6.1784E-10	87.772

FAQPQWHIIHINGTFSN(de)ETEPDTEPCVDGWVYDR	4057.833	IPI00226346	IPI00226346	yes	no		4	0.0006869	63.427
FAQPQWHIIHIN(de)GTFSNETEPDTEPCVDGWVYDR	4057.833	IPI00226346	IPI00226346	yes	no		4	0.0006869	63.427
FANEYPN(de)ITR	1223.5935	IPI00130573	IPI00130573	yes	yes		2	0.0078763	125.97
FAIKNEDSN(de)FTIINNHDNTANITVK	2832.4042	IPI00138180	IPI00138180	yes	yes		3	2.0356E-07	161.47
FAIIKDYR(de)PSAGNNSIYQDTVVFK	2745.4126	IPI00322463	IPI00322463	yes	no		3	8.6341E-06	126.25
FAEIN(de)GSAICSYNIKPSEYTITSK	2692.3054	IPI00421237	IPI00421237	yes	no		3	0.0001175	111.4
F(de)IFKNQTIEIHVIYGPR	2074.1364	IPI00122973	IPI00122973	yes	no	2,3,4		4.2134E-27	218.43
F(de)APPQPAEPWSFVKNTTSYPPMCSQDAVGGQVISEIFTNR	4453.1147	IPI00387289	IPI00387289	yes	yes		4	5.7495E-05	65.375
EYHTIDDHCVYN(de)STHIGIQR	2457.1132	IPI00127856	IPI00127856	yes	yes		3	3.1594E-19	203.99
EYEGAVYPDN(de)TTDFQR	1903.8224	IPI00605187	IPI00605187	yes	no		2	3.823E-05	170.78
EWN(de)GTYHCIFR	1481.651	IPI00876541	IPI00876541	yes	no		2	1.6318E-11	186.87
EWIPIN(de)HSVNSVVVR	1747.937	IPI00224752	IPI00224752	yes	yes		2	6.9702E-06	160.18
EVTVIGVATAPTQVISNGIPVSN(de)FTYSPDNK	3217.6507	IPI00111960	IPI00111960	yes	yes		3	0.0001271	76.439
EVNSQIDNNGCSTQEVN(de)ITEIQSK	2706.2403	IPI00123223	IPI00123223	yes	yes	2,3		2.8379E-159	299.51
EVNSKIDNNGCSTQEVN(de)ITEIQSK	2706.2767	IPI00271262	IPI00271262	yes	yes		3	1.9456E-06	115.39
EVN(de)KTCEAIIFK	1450.749	IPI00115116	IPI00115116	yes	yes		2	9.333E-09	176.18
EVN(de)ITVIDRK	1185.6717	IPI00111385	IPI00111385	yes	no		2	0.018238	120.06
EVHFFVNA(de)SDVDSVK	1691.8155	IPI00310049	IPI00310049	yes	yes		2	0.0047681	109.07
ETYIETN(de)YTIK	1373.6715	IPI00120155	IPI00120155	yes	yes		2	0.0057433	113.22
ETIVTIFDN(de)R	1206.6245	IPI00127238	IPI00127238	yes	no		2	0.0018436	152.07
ETIKNQTFGGAETVPQEATATFN(de)STAIKK	3081.5619	IPI00117424	IPI00117424	yes	no		3	0.0010451	75.868
ETIKN(de)QTFGGAETVPQEATATFNSTAIKK	3081.5619	IPI00117424	IPI00117424	yes	no		3	0.0010451	75.868
ETAEYAAN(de)ITQAVFDYFEDYFAMEYAIPK	3409.5377	IPI00134585	IPI00134585	yes	no		3	0.02616	51.119
ESSEHPWK(de)WTDNTEYNNTIPIRGEER	3187.4595	IPI00276430	IPI00276430	yes	yes		3	0.0003342	108.13
ESQTIGDQCVYNSTHIGFQREN(de)GTFSK	3102.4101	IPI00118130	IPI00118130	yes	yes		3	0.0003818	85.224
ESQTIGDQCVYN(de)STHIGFQRENGTFSK	3102.4101	IPI00118130	IPI00118130	yes	yes		3	0.0003818	85.224
ESQTIGDQCVYN(de)STHIGFQR	2339.0601	IPI00118130	IPI00118130	yes	yes	2,3		1.3004E-37	228.08
ESQIN(de)ITVMAKPTNWIEGTR	2287.1631	IPI00311405	IPI00311405	yes	yes	2,3		0.0003424	137.69
ESNPITAQQTT(de)KIDAPTNIQFVNETDR	3030.4894	IPI00113539	IPI00113539	yes	no		3	6.4216E-10	139.04
ESIIPVTIQCN(de)ITSSSHTIMYSYWTR	3086.4841	IPI00757771	IPI00757771	yes	no		3	0.0048604	69.398
ESGIQN(de)VSTCR	1249.5721	IPI01008227	IPI01008227	yes	no		2	2.5519E-05	167.18
ESGIN(de)MTIPEENQPVVFNHIYNIK	2785.3745	IPI00403938	IPI00403938	yes	no		3	1.9239E-06	130.01
ERVEN(de)TSQPGEMQVTIQNIMPATVYIFK	3222.6053	IPI00129159	IPI00129159	yes	no		3	1.4411E-11	141.36
ERTEVIIQGTVSPN(de)ASAPDAVWEDYEFK	3150.5146	IPI00405437	IPI00405437	yes	no		3	0.0004897	77.641
ERSWSTVGN(de)CTAAIR	1706.8159	IPI00128484	IPI00128484	yes	yes		2	0.0073876	110.55
ERKP(de)NNISDTVK	1399.7419	IPI00108849	IPI00108849	yes	yes		2	9.8348E-06	172.13
EREDVQAIN(de)ISVPYGPIPEDFQR	2671.3242	IPI01023131	IPI01023131	yes	no		3	6.6238E-06	130.49
EQYIHEN(de)YSR	1337.6	IPI00127406	IPI00127406	yes	no		2	0.012354	112.83
EQSFQGSN(de)MTIFYR	1706.7723	IPI00453571	IPI00453571	yes	no		2	0.010931	99.927
EQNYTCHVYHEGI(de)PEPITIR	2455.159	IPI01026927	IPI01026927	yes	no	2,3		5.4305E-05	171.04
EQIN(de)ISIDHR	1223.6258	IPI00127407	IPI00127407	yes	no		2	0.01621	108.7
EQGPSIVTPPKDIWN(de)VTGAK	2136.1215	IPI00929820	IPI00929820	yes	yes		2	0.0056374	97.384
EQESMMN(de)ITIHIVK	1671.8324	IPI00990932	IPI00990932	yes	no		2	0.0009212	119.88
ENYAEIIDDGFIKN(de)ITAQICIDKK	2810.416	IPI00339885	IPI00339885	yes	no		3	5.3177E-51	235.24
ENS(de)SYQVINWR	1394.6579	IPI00153316	IPI00153316	yes	yes		2	4.1334E-17	197.07
ENPGHIYNKN(de)ISQK	1640.8271	IPI00122175	IPI00122175	yes	no		2	3.1278E-06	164.88
ENIIDISNAN(de)R	1257.6313	IPI00944716	IPI00944716	no	no		2	1.4333E-11	191.29
ENIFGIQGAGGFQDREEEYYAEPGVTEAEPVATEDAN(de)STDSII	4945.269	IPI00378224	IPI00378224	yes	yes	4,5		1.6143E-09	70.835
ENGTFSKYEGGVETFAHIIVIR(de)K	2594.3493	IPI00118130	IPI00118130	yes	yes		3	3.5278E-05	148.06
EN(de)NSPAIHIGTISATDSDSGSNAHITYSIR	3127.4806	IPI00469537	IPI00469537	yes	yes		3	1.3185E-06	89.297
EN(de)ISDPTSPIR	1227.6095	IPI00136925	IPI00136925	yes	no		2	0.0016547	140.14
EN(de)GTFSKYEGGVETFAHIIVIR	2466.2543	IPI00118130	IPI00118130	yes	yes	3,4		1.0924E-96	265.37
EN(de)GTDAVQEEEESPAEGSK	2004.8396	IPI00123342	IPI00123342	yes	yes		2	0.0017472	104.84
EMN(de)CSETISQIQK	1566.7018	IPI00459134	IPI00459134	yes	no		2	4.3624E-18	196.5
EMGNTVMDIIRN(de)YTANATSSREEAWDYVQAQVK	3789.7727	IPI00108098	IPI00108098	yes	no		4	0.045213	43.206
EMGNTVMDIIRN(de)YTANATSSR	2343.0947	IPI00108098	IPI00108098	yes	no		3	0.030656	66.777
EMGNT(de)VMDIIRNYTANATSSREEAWDYVQAQVK	3789.7727	IPI00108098	IPI00108098	yes	no		4	0.045213	43.206
EMGNT(de)VMDIIRNYTANATSSR	2343.0947	IPI00108098	IPI00108098	yes	no		3	0.030656	66.777
EKVEN(de)GSETGPIPPEIQPIIEGEVK	2688.3858	IPI00136012	IPI00136012	yes	yes	2,3		2.3648E-09	170.11
EKSCVAITDAFPQN(de)MSR	1952.9084	IPI00405742	IPI00405742	yes	no		2	0.0096227	104.88
EKEDRPFHQAVVN(de)DTQAFWHNK	2695.2891	IPI00406901	IPI00406901	yes	no		3	0.0001497	155.94
EKDVIWFRPTIINDTGN(de)YTCMIR	2841.3942	IPI00929886	IPI00929886	yes	no		3	4.2127E-06	143.57
EKDVIWFRPTIIN(de)DTGNYTCMIR	2841.3942	IPI00929886	IPI00929886	yes	no		3	4.2127E-06	143.57
EIYESIWQN(de)FTDSK	1758.8101	IPI00272690	IPI00272690	yes	yes		2	0.031467	82.102
EITGIRN(de)ISSQEGSTDVVAR	2131.0869	IPI00677395	IPI00677395	yes	no	2,3		2.2634E-08	190.46
EITD(de)TGKNAVNCTYKNEDDCVVR	2700.2119	IPI00266264	IPI00266264	yes	yes		3	0.030659	75.215
EIKGDGPFTVFVPHADI(de)ISNMSQDEIAR	3085.5179	IPI00987265	IPI00987265	yes	no		3	4.0281E-11	138.96
EIIYSN(de)GSIIFQMITMK	1987.0159	IPI00108535	IPI00108535	no	no	2,3		1.1584E-112	278.27
EIIVHPN(de)YTR	1240.6564	IPI00113750	IPI00113750	yes	yes		2	1.168E-06	182.28
EIIVDEIISN(de)SSTIASYK	1981.0256	IPI00173181	IPI00173181	yes	no		2	0.0001434	151.95
EIISN(de)ASDAIDKIR	1543.8206	IPI00129526	IPI00129526	no	no		2	4.8292E-79	263.56
EIHHIQEQN(de)VSNVFIDKEEFFIGSK	2986.4825	IPI00605187	IPI00605187	yes	no		3	2.0308E-68	247.64
EIGAIIYN(de)CSNIAQDIEK	2050.0041	IPI00221418	IPI00221418	yes	no		2	1.2735E-132	284.57
EIEYYVGQSFANAMYN(de)ACR	2284.9881	IPI00132600	IPI00132600	yes	yes		2	0.0005134	136.66
EHSIFIDIHPVTGIPMN(de)CSVK	2393.1872	IPI01008227	IPI01008227	yes	no	2,3		2.3912E-08	183.74
EGTYSIPTSN(de)FTVPDIEIPSWISTGNYR	3143.5088	IPI00119095	IPI00119095	yes	yes		2	4.1285E-07	92.463
EGSRTDDEVVQREEEAIQIDGIN(de)ASQIR	3156.5283	IPI00129526	IPI00129526	yes	yes	3,4		1.603E-18	194.75
EGPRNISTCFSSGDIF(de)AAHNISER	2664.2351	IPI00123428	IPI00123428	yes	no		3	1.924E-80	260.06
EGN(de)CSAQSGIAWQDCDFKDAEEAATGECTATVGK	3662.5196	IPI00114958	IPI00114958	yes	no		3	3.9937E-20	137.42
EGN(de)CSAQSGIAWQDCDFK	2071.8364	IPI00114958	IPI00114958	yes	no		2	3.6707E-85	195.96
EGKFDEVYDAIAGAHPN(de)ITVYK	2436.1961	IPI00111163	IPI00111163	yes	no		3	1.0456E-06	142.58
EFVENSECIQCHPECIPQAMN(de)ITCTGR	3279.3876	IPI00121190	IPI00121190	yes	no	3,4		2.4191E-09	129.2
EFQHN(de)STMYYK	1446.6238	IPI00788443	IPI00788443	yes	no		2	0.0032921	131.82
EFEQIQVFMSSVIDMFPIGPN(de)K	2555.244	IPI00990932	IPI00990932	yes	yes		3	0.013966	64.2
EEQFN(de)STFR	1156.5149	IPI00475246	IPI00475246	yes	no		2	0.0078667	137.95
EEN(de)ATIATYPEFGVIDIK	2008.9993	IPI00124221	IPI00124221	yes	no		2	3.4345E-19	206.62

EEIGTYPYYTPTGEPVFGGIPQN(de)ASIVTHIAHTFQDIK	4190.0637	IPI00453571	IPI00453571	yes	no		4	0.017711	41.935
EEEAIQIDGIN(de)ASQIR	1784.8905	IPI00129526	IPI00129526	yes	yes		2	3.5017E-13	197.75
EDTVTITCEGTHNPGN(de)SSTQWFHNGR	2944.2794	IPI00129485	IPI00129485	yes	no		3	7.3394E-10	139.11
EDSSRAVN(de)VSAIPANIQK	1897.9858	IPI00354300	IPI00354300	yes	no		2	0.0050296	98.508
EDSCQINY(de)SEGPCIGMQER	2271.9195	IPI00127352	IPI00127352	yes	yes		2	2.8743E-77	254.82
EDRPFHQAVVNDT(de)QAFWHNK	2438.1516	IPI00406901	IPI00406901	yes	no		3	1.1889E-12	197.14
EDIIA(de)IRQNFSNITVSTEDQVK	2519.2867	IPI00129677	IPI00129677	yes	no	2,3		9.8409E-06	172
ED(de)IIAIRQNFSNITVSTEDQVK	2519.2867	IPI00129677	IPI00129677	yes	no	2,3		9.8409E-06	172
ECIGN(de)CSEPDDPTK	1620.6396	IPI00128358	IPI00128358	yes	no		2	3.4599E-07	171.81
ECHTIQN(de)YTIWR	1619.7515	IPI00125220	IPI00125220	yes	no	2,3		7.7825E-67	251.24
EAYKN(de)ISIIYSQMQK	1814.9237	IPI00221706	IPI00221706	yes	no	2,3		3.8266E-10	188.59
EATIVGEN(de)ETYPR	1477.7049	IPI00131143	IPI00131143	yes	no		2	0.009308	100.22
EASNHSSGAGIVQIN(de)KSNDKETVVGR	2696.3478	IPI00108844	IPI00108844	yes	no		3	0.0055786	75.868
EASNHSSGAGIVQIN(de)K	1610.8012	IPI00108844	IPI00108844	yes	no	2,3		8.1987E-38	231.71
EASN(de)HSSGAGIVQINKSNDKETVVGR	2696.3478	IPI00108844	IPI00108844	yes	no		3	0.0055786	75.868
EASN(de)HSSGAGIVQINK	1610.8012	IPI00108844	IPI00108844	yes	no	2,3		8.1987E-38	231.71
EASHYSIHDIVISYN(de)TSDSTVFPGAVAK	3007.4563	IPI00222967	IPI00222967	yes	no	2,3,4		3.5426E-20	202.73
EARPITPAAIN(de)GSIQR	1692.9271	IPI00515432	IPI00515432	yes	no		2	0.0085052	107.69
EAQYN(de)STFR	1114.5043	IPI00807983	IPI00807983	yes	no		2	0.0056377	145.25
EAN(de)ITEDQIIFFPK	1663.8457	IPI00122122	IPI00122122	yes	no		3	0.0033294	113.99
EAFN(de)ETNQAIQTISR	1720.838	IPI00112614	IPI00112614	yes	yes		2	5.1753E-155	295.37
EAFIPQGGSVQVN(de)CSSSCK	2053.9197	IPI00122973	IPI00122973	yes	no		2	0.0005899	131.98
EACIQHYYVN(de)ASYIVWK	2143.0197	IPI00120155	IPI00120155	yes	yes		2	0.0002066	146.3
E(de)SVYRNYSIFIADINQER	2216.0862	IPI00331318	IPI00331318	yes	no		3	0.0008323	162.81
DYYPIN(de)ESIASIPIR	1749.8938	IPI00121627	IPI00121627	yes	yes	2,3		9.2645E-07	167.98
DYYIN(de)KTENEK	1415.6569	IPI00396840	IPI00396840	yes	no		2	4.2313E-17	199.82
DYRPSAGN(de)NSIYQDTVVFK	2173.044	IPI00322463	IPI00322463	yes	no	2,3		3.3336E-05	179.1
DYCMYYNNN(de)WTR	1698.6555	IPI00121776	IPI00121776	yes	yes		2	2.8152E-09	179.93
DWIWNQMHIDEEKN(de)ESIPHYVGK	2867.3337	IPI00138180	IPI00138180	yes	yes		3	0.0056894	86.727
DWCN(de)WTIISR	1349.6187	IPI00124933	IPI00124933	yes	no		2	1.2226E-09	189.13
DVIWFRPTIINDTGNYT(de)CMIR	2584.2566	IPI00929886	IPI00929886	yes	no		3	0.0002221	156.13
DVGSGTTN(de)NSQACAQFIEQYFHNSDITEFMR	3566.5467	IPI00130661	IPI00130661	yes	yes	3,4		1.6623E-15	164.31
DVFIP(de)KPSWGNHTPIFR	2010.0476	IPI00117312	IPI00117312	yes	yes	2,3		0.0092565	130.21
DTYVN(de)ASQSIYGSSPR	1743.8064	IPI00122977	IPI00122977	yes	no		2	1.1346E-178	306.53
DTVQHWN(de)WTSDIPIQCATHSVSIR	2850.3508	IPI00119299	IPI00119299	yes	no		3	2.0563E-05	113.73
DTTGSHTFQGMFGCEITNN(de)R	2271.9637	IPI00137987	IPI00137987	yes	yes		3	0.000392	118.48
DTISIN(de)ATNIK	1188.635	IPI00121190	IPI00121190	yes	no		2	7.127E-34	221.55
DTEENIHVIN(de)QTISEVIFPMDIK	2684.3367	IPI00356462	IPI00356462	yes	no		3	0.028418	63.69
DTCAQECSHFNI(de)TKVESR	2180.9579	IPI00132474	IPI00132474	yes	no		3	0.0023707	130.4
DTCAQECSHFNI(de)TK	1709.7138	IPI00132474	IPI00132474	yes	no		2	2.6329E-39	232.17
DTAVEGEEIEVN(de)CTAMASKPATTIR	2692.2684	IPI00856723	IPI00856723	yes	no		3	0.0006886	95.624
DSYPDGN(de)ITWYRNGK	1784.8118	IPI00121378	IPI00121378	yes	no		2	7.1503E-11	192.39
DSYPDGN(de)ITWYR	1485.6525	IPI00121378	IPI00121378	yes	no		2	0.0060213	107.17
DSKIN(de)NTNVR	1159.5945	IPI00380801	IPI00380801	yes	no		2	0.021667	113.69
DSGIWINGFDYTGMSHVTPHIPEIN(de)DTIR	3284.5561	IPI00407222	IPI00407222	yes	yes		3	1.1449E-14	144.56
DQQAAEANKN(de)ASIIIK	1712.9057	IPI01016174	IPI01016174	yes	no		3	0.026137	83.499
DQGNV(de)TDMASMK	1295.5486	IPI00471081	IPI00471081	yes	yes		2	1.6243E-81	257.76
DQDCDKF(de)NQCGTCTEFKECHTIQNYTIWR	3753.5705	IPI00125220	IPI00125220	yes	no		4	3.3565E-05	82.302
DQCIVDDITYNVN(de)DTFHKR	2352.0805	IPI00113539	IPI00113539	yes	no	2,3		7.5559E-09	192.6
DQCIVDDITYNVN(de)DTFHK	2195.9793	IPI00113539	IPI00113539	yes	no	2,3		4.3996E-94	269.59
DNVN(de)CSGVYEHEPIEIGK	2058.9317	IPI00947577	IPI00947577	yes	yes		2	2.3018E-05	176.28
DNSCVDPPHVPN(de)ATIVTR	1990.9531	IPI00130010	IPI00130010	yes	no	2,3		4.6604E-77	256.67
DNATDS(de)VPIR	1086.5306	IPI00119063	IPI00119063	yes	yes		2	6.5544E-07	157.68
DN(de)YSVIIQENKPVGFSVIK	2149.1419	IPI00623114	IPI00623114	yes	no		2	0.021004	85.362
DN(de)TTCYEFKK	1304.5707	IPI00119063	IPI00119063	yes	yes		2	0.0029896	155
DN(de)SSGTFIVIIR	1320.7038	IPI00119961	IPI00119961	yes	no		2	0.0019282	126.83
DN(de)SCVDPPHVPNATIVTR	1990.9531	IPI00130010	IPI00130010	yes	no	2,3		4.6604E-77	256.67
DN(de)HTSCAECIK	1333.5391	IPI00320605	IPI00320605	yes	no		2	0.0009939	150.46
DN(de)DSIITRK	1060.5513	IPI00131168	IPI00131168	yes	yes		2	0.029389	145.25
DN(de)DSIITR	932.45632	IPI00131168	IPI00131168	yes	yes		2	0.044658	91.658
DN(de)ATQEEIIHYIEK	1701.821	IPI00321190	IPI00321190	yes	no	2,3		4.5808E-07	171.26
DMSDGFISN(de)ITIQR	1595.7614	IPI00272381	IPI00272381	yes	no		2	3.1742E-114	283.82
DKVTFIN(de)TTR	1193.6404	IPI00120674	IPI00120674	yes	no		2	0.0098327	131.43
DKNGTRAEPPIN(de)ASAGDQEEK	2226.0513	IPI00123342	IPI00123342	yes	yes	2,3		1.1583E-78	263.55
DKN(de)GTRAEPPINASAGDQEEK	2226.0513	IPI00123342	IPI00123342	yes	yes	2,3		1.1583E-78	263.55
DKISPIHIA(de)INFSIDPK	1907.0516	IPI00115976	IPI00115976	yes	yes	2,3		5.3451E-05	172.82
DIWN(de)VTGAK	1002.5134	IPI00652107	IPI00652107	no	no	1,2		0.035656	113.62
DIWN(de)VTGAK	1002.5134	IPI00652107	IPI00652107	no	no	1,2		0.035656	113.62
DIVV(de)EIYSQWMKNPNNNTIHPNIR	2894.4497	IPI00319509	IPI00319509	yes	no		3	0.0007815	102.98
DIVQNVFMSN(de)MSMDIQSHPSSCPK	2751.2125	IPI00121190	IPI00121190	yes	no		3	3.6769E-05	110.75
DIVGN(de)ITEIESEDIQIEAIIMR	2500.2731	IPI00122399	IPI00122399	yes	no		3	2.03E-29	219.87
DITNIIN(de)NTFIR	1432.7674	IPI00650038	IPI00650038	yes	no		2	3.9383E-19	198.74
DISVFAPN(de)MTEIIKDVTQYR	2339.1831	IPI00855103	IPI00855103	yes	no		3	0.020153	83.418
DISGN(de)ETHFTGSEVGFIKPISCR	2550.2173	IPI00123652	IPI00123652	yes	no		3	7.6848E-10	190.67
DIRPN(de)HTIYINNMNDKIK	2198.1266	IPI00132576	IPI00132576	yes	no		3	0.018418	76.82
DIQDIHKDTEN(de)R	1482.7063	IPI00129304	IPI00129304	yes	yes		2	0.0019377	149.69
DINSQIEIQNVTN(de)DTHITIIGIKPDTTYDIK	3511.8046	IPI00110264	IPI00110264	yes	yes		3	4.8492E-15	168.25
DINSQIEIQN(de)VTNDTHITIIGIKPDTTYDIK	3511.8046	IPI00110264	IPI00110264	yes	yes		3	4.8492E-15	168.25
DIN(de)HTAQGIIEK	1337.6939	IPI00120953	IPI00120953	yes	no		2	0.0020489	141.37
DIIIHSTGHN(de)ISR	1461.7688	IPI00223987	IPI00223987	yes	yes		2	1.0968E-18	201.48
DIHVVHFHGQTIIDN(de)R	1899.9704	IPI00406603	IPI00406603	yes	yes		2	0.0038919	110.22
DIGTASHN(de)FSGAIPR	1541.7587	IPI00229820	IPI00229820	yes	yes		2	1.2151E-05	157.38
DIGPAIAN(de)SSHDVK	1422.7103	IPI00108811	IPI00108811	yes	yes		2	3.9849E-21	205.3
DIFTNQSDFADTTKDTPITITVIH(de)K	2820.4182	IPI00116105	IPI00116105	yes	yes		3	2.3453E-09	166.27
DIEHHITNVNVSFYDDIVN(de)GTVIK	2741.3661	IPI00467600	IPI00467600	yes	yes		3	4.0427E-30	220.63
DIEHHITNVN(de)VSFYDDIVNGTVIK	2741.3661	IPI00467600	IPI00467600	yes	yes		3	4.0427E-30	220.63

DIDTDFTSN(de)ASQPETK	1767.7799	IPI00130573	IPI00130573	yes	yes		2	7.9916E-09	188.44
DIAVGDDYTN(de)R	1237.5575	IPI00468674	IPI00468674	yes	no		2	0.0056909	113.61
DHVNVSMIEATDFPFN(de)TTEWEGYIPK	3039.396	IPI00136942	IPI00136942	yes	yes		3	3.9459E-11	183.07
DHVN(de)VSMIEATDFPFNTTEWEGYIPK	3039.396	IPI00136942	IPI00136942	yes	yes		3	3.9459E-11	183.07
DHIIN(de)KTHSICPR	1589.8096	IPI00118994	IPI00118994	yes	yes		2	0.0022794	144.27
DHGDIAFVDVPN(de)DSSFQIVKNPR	2569.2561	IPI00117914	IPI00117914	yes	yes		3	5.9361E-112	228.39
DGTSQPAICPQN(de)VTMNMEGIK	2290.0392	IPI00320204	IPI00320204	yes	yes		2	7.2736E-30	223.54
DGTDDPSYNGAIIVSGNEKN(de)HSFSFACNTK	3244.4367	IPI00170145	IPI00170145	yes	no		3	2.1316E-06	107.17
DGSQDFNE(de)TWENYEK	1860.7439	IPI00128206	IPI00128206	yes	yes		2	1.2866E-08	182.57
DGSDSAAMVYN(de)SSQEWGIR	2071.8905	IPI00119522	IPI00119522	yes	yes		2	1.0186E-204	315.94
DGIAIN(de)ITIR	1084.6241	IPI00119065	IPI00119065	yes	yes		2	0.0037173	136.48
DGHFKEDPYWENMIN(de)HSVHR	2510.1186	IPI00410796	IPI00410796	yes	yes		3	0.0002025	140.22
DGAFHHIHSTPFGN(de)YSFISVDATQRPGPK	3182.5322	IPI00226714	IPI00226714	yes	no		3	8.0232E-09	133.41
DFIN(de)YTIEER	1298.6143	IPI00134191	IPI00134191	yes	no		2	0.0018552	152.7
DEIGN(de)VSTSHIIIIDDSVEMEIRPR	2837.4229	IPI00309035	IPI00309035	yes	yes		3	0.0024912	79.467
DDYRPTWTIN(de)QTEPVAGNYYPVNTR	2969.3944	IPI00381303	IPI00381303	yes	yes	3,4		5.7996E-55	240.44
DDIHPTIPAGQYFIN(de)ITYNYPVHSFDGR	3249.552	IPI00387318	IPI00387318	yes	no	3,4		1.1557E-11	175.42
DCVSCQN(de)VSR	1223.5023	IPI00121190	IPI00121190	yes	no		2	6.8408E-06	177.44
DCDFIEDGEERN(de)CTGK	1943.7626	IPI00127280	IPI00127280	yes	yes		2	4.9776E-28	222.45
DAMVGN(de)YTCEVTEISR	1843.808	IPI00124830	IPI00124830	yes	no		2	5.5194E-49	239.55
DAHFN(de)STITEFGETINNFVER	2440.1295	IPI00346062	IPI00346062	yes	yes		3	0.0070384	83.723
DAGN(de)YTVIITNPISMEK	1864.9241	IPI00124326	IPI00124326	yes	no		2	0.0019444	134.93
DAAQCSGGSVAHIAEIGIPTN(de)ITHIIIFR	3060.5815	IPI00331407	IPI00331407	yes	no		3	1.1527E-12	146.75
CVYEAICSN(de)VTSECPPPVITR	2451.1233	IPI00405742	IPI00405742	yes	no		3	1.5613E-09	193.19
CVVHYEN(de)STVPEKK	1688.8192	IPI00409148	IPI00409148	yes	yes	2,3,4		7.3812E-08	181.71
CVVHYEN(de)STVPEK	1560.7242	IPI00409148	IPI00409148	yes	yes		2	0.0023324	119.88
CVAN(de)YTGNGR	1110.4876	IPI00111793	IPI00111793	yes	yes		2	0.0044883	134.57
CTPPPYVENAVMISEN(de)R	1975.9132	IPI00138061	IPI00138061	yes	no		2	4.1806E-05	155.1
CTAQVCAGYCSNN(de)STCTVNQGNQPQCR	3134.2481	IPI00119063	IPI00119063	yes	yes		3	0.0011242	73.11
CTAQVCAGYCSN(de)NSTCTVNQGNQPQCR	3134.2481	IPI00119063	IPI00119063	yes	yes		3	0.0011242	73.11
CSIIHSN(de)ISVK	1256.6547	IPI00133222	IPI00133222	yes	no		2	7.1499E-12	190.53
CQSPYQMN(de)GSDTVTCVNSR	2202.9092	IPI00130010	IPI00130010	yes	no	2,3		3.2954E-05	142.94
CQAYKN(de)NSESFIHIYMYEVAR	2622.1995	IPI00130654	IPI00130654	yes	no		3	8.4578E-05	135.18
CQAIDFFPQN(de)ITMR	1739.8123	IPI00331440	IPI00331440	yes	no		2	0.000577	127.71
CPMAGISHTTWHSN(de)R	1753.7777	IPI00130764	IPI00130764	yes	no	2,3		5.1494E-11	190.24
CPIITPFNDTNVVHTVNTAIAAFNTQNN(de)GTYFK	3681.7886	IPI00128249	IPI00128249	yes	yes	3,4		2.5203E-18	161.49
CPIITPFN(de)DTNVVHTVNTAIAAFNTQNNGTYFK	3681.7886	IPI00128249	IPI00128249	yes	yes	3,4		2.5203E-18	161.49
CNSVITYN(de)ITPVVQK	1734.8975	IPI00222967	IPI00222967	yes	no		2	7.6912E-29	219.52
CN(de)ITIKDPTPADPIWYEAK	2231.0933	IPI00114842	IPI00114842	yes	no		2	0.001305	150.91
CN(de)ASSQFICSSGR	1472.6136	IPI00119063	IPI00119063	yes	yes		2	0.0015131	127.32
CIVPHTVN(de)ISSAWRNHSIPIETK	2658.37	IPI00129041	IPI00129041	yes	no		3	0.031276	57.41
CIVPHTVN(de)ISSAWR	1638.83	IPI00129041	IPI00129041	yes	no		2	5.6014E-06	166.03
CISPNVTSCACTIN(de)FTIK	2084.9693	IPI00387318	IPI00387318	yes	yes		2	1.8804E-36	225.54
CISPN(de)VTSCACTINFTIK	2084.9693	IPI00387318	IPI00387318	yes	yes		2	1.8804E-36	225.54
CISKN(de)YTTDIITK	1555.7916	IPI00124640	IPI00124640	yes	yes		2	0.032145	91.307
CIQMSSFAN(de)R	1212.538	IPI00320065	IPI00320065	yes	no		2	7.2309E-68	248.88
CIQHFYGPNHEHCFN(de)R	2114.8952	IPI00130420	IPI00130420	yes	no		3	9.9973E-05	171.73
CIPHFAMIGNDTVMCTEQGN(de)WTRIPECIEVK	3705.687	IPI00322463	IPI00322463	yes	no		4	4.268E-09	120.63
CIPHFAMIGNDTVMCTEQGN(de)WTR	2737.1869	IPI00322463	IPI00322463	yes	no		3	1.8474E-06	149.44
CIPHFAMIGN(de)DTVMCTEQGNWTRIPECIEVK	3705.687	IPI00322463	IPI00322463	yes	no		4	4.268E-09	120.63
CIPHFAMIGN(de)DTVMCTEQGNWTR	2737.1869	IPI00322463	IPI00322463	yes	no		3	1.8474E-06	149.44
CIN(de)GTINYPTCV	1410.6272	IPI00130010	IPI00130010	yes	no		2	0.001397	160.67
CHEGN(de)GTFECGACR	1653.6082	IPI00132474	IPI00132474	yes	yes	2,3		3.0839E-06	169.4
CGNCN(de)ITSIEDEDFCK	1960.7601	IPI00137599	IPI00137599	yes	yes		2	1.002E-112	280.7
CFNAMEVDAIN(de)SSHPVSTPVENPAQIR	2982.3964	IPI00318595	IPI00318595	yes	no		3	5.0249E-45	233.68
CF(de)ANKVNISFPSAQSIPASDTHIK	2631.3115	IPI00624663	IPI00624663	yes	yes	2,3,4		1.2157E-39	229.75
CEVICNQ(de)SNKPITITVEQSR	2375.1573	IPI00626537	IPI00626537	yes	yes		3	0.0016497	106.57
CEQEASEDIKPAITGN(de)K	1888.8837	IPI00134808	IPI00134808	yes	no		2	1.4063E-36	231.06
CDPISETQQWIFEHIN(de)MTVIEKNSHYAIS	3489.6333	IPI00761633	IPI00761633	yes	yes		3	0.03058	47.38
CDGDFDCEDRTDEAN(de)CSVK	2291.8365	IPI00515360	IPI00515360	yes	no		2	7.2265E-08	191.35
CCGWVSHYN(de)WTENEEIMGFTK	2647.093	IPI00108098	IPI00108098	yes	no		3	0.029768	53.768
CAMDIGVNYIGTVN(de)VTHTGIQCQIWR	3005.431	IPI00114206	IPI00114206	yes	yes		3	0.0003138	80.236
CAISHAITIEFHQDGNGPENVGIYN(de)ISR	3111.4832	IPI00222429	IPI00222429	yes	yes	3,4		9.3476E-20	198.6
C(de)IVPHTVNISSAWRNHSIPIETK	2658.37	IPI00129041	IPI00129041	yes	no		3	0.031276	57.41
AYTIFVPTNHSIETQGN(de)NSVIGIDTVR	2945.4883	IPI00987265	IPI00987265	yes	no		3	0.0001824	87.802
AYTIFVPTN(de)HSIETQGNNSVIGIDTVR	2945.4883	IPI00987265	IPI00987265	yes	no		3	0.0001824	87.802
AY(de)AISGPTVQDGIPPFNWNRFNETIHR	3099.5315	IPI00471081	IPI00471081	yes	yes	3,4		0.0001812	100.76
AWN(de)YTVDTPHGMFVSGTPFEQSVWITDVGSGPYGHTVK	4166.9473	IPI00228567	IPI00228567	yes	yes		4	0.0001382	64.885
AWGISVINPN(de)KTK	1426.7932	IPI00989096	IPI00989096	yes	no		2	0.0005154	158.11
AWEKEIHHI(de)QEQNVSNVFIDKEEFFIGSK	3500.7365	IPI00605187	IPI00605187	yes	no		3	8.6266E-12	150.36
AVYEAIRN(de)CSIEDDSVR	1995.932	IPI00856861	IPI00856861	yes	no		2	0.0029646	120.63
AVVGSYTTTN(de)VSIIAPR	1747.9468	IPI00330481	IPI00330481	yes	yes		2	8.7727E-05	151.48
AVNAHVEIGYSTIVGVFHTEYGAIN(de)R	2816.4246	IPI00132444	IPI00132444	yes	yes	3,4		2.319E-09	171.29
AVN(de)QTGAIYQCDYSTSR	1932.8636	IPI00120674	IPI00120674	yes	no		2	6.5469E-10	193.19
AVN(de)QSMIR	917.47529	IPI00471090	IPI00471090	yes	yes		2	0.029604	118.5
AVKIN(de)ETIGNQDK	1428.7573	IPI00309999	IPI00309999	yes	yes		2	0.0036271	121.18
AVISMIN(de)ETMNEAPEETK	2005.9336	IPI00470184	IPI00470184	yes	no	2,3		1.3368E-179	311.92
AVGAVDWDSHPYGYN(de)ITIQAK	2304.1175	IPI00623114	IPI00623114	yes	no		2	0.030995	66.874
AVCGIN(de)TSDRCDFVR	1768.7985	IPI00127134	IPI00127134	yes	no		2	0.046389	84.468
AVCGIN(de)TSDR	1091.503	IPI00127134	IPI00127134	yes	no		2	0.02362	101.38
ATVN(de)DSGEYR	1110.4942	IPI00129485	IPI00129485	yes	no		2	0.0089509	123.51
ATPNEGFFNQN(de)ITTFYYDR	2297.0389	IPI00315535	IPI00315535	yes	yes	2,3		1.3579E-62	250.04
ATIITFICDRDAGVGFPEYQEEDN(de)STYNFR	3527.594	IPI00308971	IPI00308971	yes	yes		3	3.2051E-14	149.73
ATHN(de)DSGSYFCR	1413.5732	IPI00162870	IPI00162870	yes	yes		2	0.0045144	112.51
ATDADINDIIVYTIQEVTPN(de)ASK	2490.249	IPI00314132	IPI00314132	yes	no		3	1.666E-05	119.62
ASVVN(de)VTQHCTMESWESMNEVAR	2664.1731	IPI00121240	IPI00121240	yes	yes	2,3		2.5673E-06	124.2

ASSSFRPN(de)GTK	1150.5731	IPI00113797	IPI00113797	yes	yes		2	0.033848	96.82
ASSIMVN(de)ITEIFPKPK	1773.9699	IPI00406901	IPI00406901	yes	no		2	2.3102E-08	186.42
ASQQISN(de)ETSSFGFNIIR	1997.9807	IPI00153258	IPI00153258	yes	no	2,3		3.7023E-62	248.46
ASIITGKYPHNHHVVN(de)NTIEGNCSSK	2876.3988	IPI00221426	IPI00221426	yes	yes		3	3.7142E-09	131.56
ASIITGKYPHNHHVV(de)NNTIEGNCSSK	2876.3988	IPI00221426	IPI00221426	yes	yes		3	3.7142E-09	131.56
ASGN(de)FSQVDWFIIHK	1747.8682	IPI00469123	IPI00469123	yes	no	2,3		0.0011385	129.01
AQTN(de)YTCVAEIIYR	1700.8192	IPI00323053	IPI00323053	yes	no		2	1.6232E-06	165.49
AQAGNKSQDSGIAEMEEIPVPHNIKINN(de)ITCDSFK	3854.8567	IPI00969894	IPI00969894	yes	yes		4	0.026275	49.967
AQAGN(de)KSQDSGIAEMEEIPVPHNIKINNITCDSFK	3854.8567	IPI00969894	IPI00969894	yes	yes		4	0.026275	49.967
AQAAIDKAN(de)ASR	1214.6367	IPI00119065	IPI00119065	yes	yes		2	1.0608E-08	175.21
APYTIEN(de)SSHR	1273.6051	IPI00471293	IPI00471293	yes	no		2	0.0075123	109.72
APQHVVNHIPPYTN(de)VSIK	2013.0796	IPI00123040	IPI00123040	yes	no	2,3		0.0003396	159.04
APIPTAIDTN(de)SSKTPSIFPCASGAFSSFR	3026.4808	IPI00119809	IPI00119809	yes	no		4	0.032709	52.366
APIPTAIDTN(de)SSK	1313.6827	IPI00119809	IPI00119809	yes	no		2	0.0027209	118.24
ANQIVIPHN(de)TTFQTEPTK	2038.0484	IPI00466371	IPI01026704	no	no	2,3		2.2327E-05	176.49
ANISKYSIVMEADSGTFIPTGIQF(de)TGSDK	3076.5063	IPI00126050	IPI00126050	yes	no		3	0.01779	59.046
ANIQKGENPSQYGITAF(de)NHPINITK	2754.4089	IPI00112614	IPI00112614	yes	yes		3	0.047571	48.597
ANIQFGENGT(de)TISAVTNK	1863.9327	IPI00127447	IPI00127447	yes	yes	2,3		3.8652E-95	273.82
ANEQVIQNIN(de)HTYK	1670.8376	IPI00876541	IPI00876541	yes	no		2	8.5401E-07	169.3
AN(de)YTIIKGNENGNFK	1681.8424	IPI00138209	IPI00138209	yes	no		2	0.0004752	145.1
AN(de)YTIIK	821.4647	IPI00138209	IPI00138209	yes	no		2	0.0023591	161.68
AN(de)TSIEIIIEGVR	1413.7827	IPI00988518	IPI00988518	yes	no		2	0.0014353	129.54
AN(de)KSSDIVSANR	1260.6422	IPI00136642	IPI00136642	yes	yes		2	0.012091	104.59
AN(de)ISSQVIK	958.54475	IPI00322867	IPI00322867	yes	no		2	0.015968	123.86
AN(de)GTTVHVGIHPSK	1416.7474	IPI00132460	IPI00132460	yes	no		2	0.0016696	121.45
AN(de)ESRIEEADQIYR	1692.8067	IPI00224237	IPI00224237	yes	no		2	0.0024492	128.22
AN(de)DTREEAGIPAAGEDETSWTER	2504.1052	IPI00653847	IPI00653847	yes	no		3	0.0004474	108.25
AN(de)DSDQGANAEIDYTFHQAPEVVRR	2802.2957	IPI00719927	IPI00719927	yes	no		3	3.1657E-12	186.11
AN(de)DSDQGANAEIDYTFHQAPEVVR	2646.1946	IPI00719927	IPI00719927	yes	no		3	2.487E-07	156.51
AMSSN(de)ETAAYKIMR	1571.7436	IPI00316469	IPI00316469	yes	yes		2	0.013204	92.063
AMN(de)ISASSMTITWK	1539.7425	IPI00469426	IPI00469426	yes	no		2	4.2469E-39	228.03
AMIINQHVPMESSHIIQFVN(de)WSSIIPERYKK	3694.9116	IPI00387282	IPI00387282	yes	yes	3,4,5,6		1.2689E-14	170.59
AMIINQHVPMESSHIIQFVN(de)WSSIIPERYK	3566.8167	IPI00387282	IPI00387282	yes	yes	3,4,5		1.6081E-14	143.48
AMIINQHVPMESSHIIQFVN(de)WSSIIPER	3275.6584	IPI00387282	IPI00387282	yes	yes	3,4		5.4695E-12	181.82
AMGVNVIAANTHN(de)TSMHMTGSGIYSPEAVR	3115.4638	IPI00230412	IPI00230412	yes	yes	3,4		3.9842E-15	135.62
AMFN(de)ITIIYPNNIIAISNMIPK	2490.3379	IPI00319509	IPI00319509	yes	yes	2,3		4.1228E-07	141.83
AKEWGYAS(de)HNGPDHWHEIYPIAK	2705.2775	IPI00221890	IPI00221890	yes	yes		3	0.0017339	94.057
AIWIIN(de)GSK	1000.5706	IPI00124666	IPI00124666	yes	yes		2	0.019243	118.71
AIVN(de)FTR	819.46029	IPI00515360	IPI00515360	yes	no		2	0.03315	106.36
AISPN(de)STISSAPK	1271.6721	IPI00460291	IPI00460291	yes	no		2	0.0010868	146.59
AISCIESSWKTIEQERN(de)ASFVYTK	2846.3909	IPI00123223	IPI00123223	yes	yes		3	0.0058892	87.352
AINASAN(de)ITSDGVEVIGR	1785.9221	IPI00330632	IPI00330632	yes	no		2	0.0030716	106.57
AIN(de)VTISSMGRNGIK	1559.8454	IPI00131091	IPI00131091	yes	no		2	0.0077158	106.42
AIN(de)VTISSMGR	1147.6019	IPI00131091	IPI00131091	yes	no		2	0.0014458	142.97
AIN(de)SSTEDGIKR	1289.6575	IPI00129168	IPI00129168	yes	yes		2	0.009621	100.04
AIN(de)GSAIYTGSSIDFVR	1769.8948	IPI00877197	IPI00877197	yes	no		2	1.5744E-178	305.43
AIN(de)DSIHYINR	1314.668	IPI00808345	IPI00808345	yes	no		2	0.005026	118.48
AIN(de)ASANITSDGVEVIGR	1785.9221	IPI00330632	IPI00330632	yes	no		2	0.0030716	106.57
AIMKEVMNIIQPIN(de)VTK	1941.0791	IPI00126050	IPI00126050	yes	no	2,3		2.3891E-07	170.89
AIMDICDQIEKN(de)QTK	1805.8652	IPI00658632	IPI00658632	yes	yes		2	1.165E-256	237.46
AIKSHGIWN(de)NTVFIFSTDNGGQTR	2662.3252	IPI00652358	IPI00652358	yes	no		3	0.024008	70.538
AIKGETVN(de)TSIPFSFEGIIFPK	2394.2835	IPI00119324	IPI00119324	yes	no		3	0.016034	77.631
AIIQGIIPEQN(de)YTVQIIAYYKDK	2680.4476	IPI00330632	IPI00330632	yes	no		3	0.023121	70.334
AIIQGIIPEQN(de)YTVQIIAYYK	2437.3257	IPI00330632	IPI00330632	yes	no		3	0.0001059	170.11
AIGYEN(de)ATQAIGR	1362.6892	IPI00119809	IPI00119809	yes	no		2	2.5625E-24	208.82
AIGITEMFEPSKAN(de)FTK	1882.9499	IPI00115065	IPI00115065	yes	yes		2	0.002819	123.4
AIEIQFGMN(de)ASSSIFFIQGVR	2314.178	IPI00469218	IPI00469218	yes	yes		3	5.8925E-05	139.88
AIDREAETVRNGIYN(de)ITVIAIDADGR	2844.473	IPI00138209	IPI00138209	yes	no		3	0.020081	68.634
AIDIDPSKTN(de)VSGGAIAIGHPIGGSGSR	2646.3725	IPI00226430	IPI00226430	yes	yes		3	1.1834E-10	145.2
AIAGIVYN(de)SSGTEPCYDIYR	2248.047	IPI00331550	IPI00331550	yes	yes	2,3		3.9606E-78	260.31
AIAAAGYDVEKN(de)NSR	1577.7798	IPI00223714	IPI00223714	no	no		2	4.5498E-05	158.93
AHFSSIN(de)ITIR	1257.683	IPI00466371	IPI01026704	no	no		2	2.5598E-13	193.28
AGYFN(de)FTSATITYIAQEDGPVVIGSTSAPGQGGIIAQR	3856.9272	IPI00467180	IPI00467180	yes	yes		4	2.1724E-27	150.82
AGVVVFN(de)CSIR	1220.6336	IPI00314779	IPI00314779	yes	yes		2	0.020574	95.477
AGPN(de)GTIFVVDAYK	1450.7456	IPI00221998	IPI00221998	yes	yes		2	5.8164E-15	194.55
AGMASPIYN(de)VTWSAGWK	1837.8821	IPI00666034	IPI00666034	yes	no		2	0.026892	82.007
AGIN(de)ASYSEK	1038.4982	IPI00308971	IPI00308971	yes	yes		2	0.0010053	161.67
AGEQINNHVKN(de)KTQGK	1764.9231	IPI00116105	IPI00116105	yes	no	2,3		0.0079229	118.21
AGEQINNHVKN(de)K	1350.7004	IPI00116105	IPI00116105	yes	no		2	0.025003	96.604
AFYN(de)GTWYR	1176.5352	IPI00858318	IPI00858318	yes	no		2	0.0010322	168.83
AFVEN(de)ITVIENSIVFK	1821.9877	IPI00742385	IPI00742385	yes	no	2,3		0	370.3
AFQIN(de)TFNIKVQPFNVTK	2108.1419	IPI00222967	IPI00222967	yes	no		3	0.032864	82.349
AFPEVCN(de)ETMMAIWEECKPCIK	2742.1984	IPI00320420	IPI00320420	yes	no		3	0.0030842	88.283
AFNISPN(de)DTSSGSCGINIVTIK	2294.1213	IPI00469218	IPI00469218	yes	yes	2,3		7.3206E-116	277.25
AFN(de)ITWISTDFK	1441.7242	IPI00118069	IPI00118069	yes	no		2	0.000688	158.58
AFN(de)ECCTIANK	1326.5697	IPI00330833	IPI00330833	yes	yes		2	2.2026E-05	162.49
AFMN(de)SSFTIDPK	1356.6384	IPI00121362	IPI00121362	yes	no		2	3.5093E-19	199.8
AFITNF(de)SMIIDGVTYPGVVK	2171.1337	IPI00312711	IPI00312711	yes	no	2,3		7.0773E-28	220.62
AFIN(de)GTGVETVVSADIPNAHGIAVDWVSR	2994.5199	IPI00119063	IPI00119063	yes	yes	3,4		1.576E-14	178.11
AFFDETKN(de)NTR	1341.6313	IPI00120674	IPI00120674	yes	no		2	0.0027234	141.08
AEQITIHAIGIGEANKT(de)QIR	2162.1808	IPI00990932	IPI00990932	yes	no		3	0.028883	77.031
AEPPINASAGDQ(de)EEK	1554.7162	IPI00123342	IPI00123342	yes	yes	2,3		0.008687	101.2
AEISN(de)HTRPVIIVPGCIGNRIEAK	2643.4279	IPI00133500	IPI00133500	yes	yes	3,4		1.0586E-05	122.64
AEISN(de)HTRPVIIVPGCIGNR	2202.1692	IPI00133500	IPI00133500	yes	yes	2,3		0.0001981	168.41
AEDYGPVEVISHWHPNITINI(de)VDDHTPWVK	3480.7103	IPI00121627	IPI00121627	yes	yes		4	3.3306E-06	89.079
AEDYGPVEVISHWHPNI(de)TINIVDDHTPWVK	3480.7103	IPI00121627	IPI00121627	yes	yes		4	3.3306E-06	89.079

ADSIYSQVVGISASQAN(de)ISK	2037.0378	IPI0011511€	IPI0011511€	yes	yes		3	9.9095E-78	254.58
ADN(de)YTYEHIR	1280.5786	IPI0027313З	IPI0027313З	yes	yes		2	0.020031	104.79
ADANPPATEYHWTTIN(de)GSIPK	2282.0968	IPI00311405	IPI00311405	yes	yes	2,3		0.0005902	107.65
ACN(de)ATNWIEYMFNK	1760.7651	IPI0013260C	IPI0013260C	yes	no		2	4.0563E-05	160.78
ACMN(de)ETRIEEIR	1520.7075	IPI0039684C	IPI0039684C	yes	no		2	0.0023808	131.06
AAN(de)CTQVIVWHTR	1554.7725	IPI00116744	IPI00116744	yes	yes		2	0.0013798	142.1
AAGVIEDVYN(de)RTQGIIAGHGIIQIA	2578.3867	IPI00169617	IPI00169617	yes	yes		2	1.0745E-08	161.2
AAEN(de)FTIIVK	1104.6179	IPI0047108§	IPI0047108§	yes	no		2	0.016008	108.9
AACAVRPQEVTMVN(de)GTITNPVTGK	2513.273	IPI00308971	IPI00308971	yes	yes	2,3		1.198E-06	175.93
A(de)YVFERNQSVGDPNVDIIR	2191.1022	IPI00127447	IPI00127447	yes	yes	2,3		6.2046E-19	205.95
A(de)VAYGEKNITFQGPIPK	1831.9832	IPI00111794	IPI00111794	yes	no	2,3		2.9802E-27	219.97
A(de)RNISGVVIADHSGSFHNR	2036.03	IPI00118674	IPI00118674	yes	yes		3	0.014586	90.385
A(de)QAECRNTTHIIQR	1696.8427	IPI00321222	IPI00321222	yes	yes	2,3		0.025582	94.122

N-terminal Ser/Thr	15
ratio	1.04%
